# The importance of enhancer methylation for epigenetic regulation of tumorigenesis in squamous lung cancer

**DOI:** 10.1038/s12276-021-00718-4

**Published:** 2022-01-05

**Authors:** Jae-Won Cho, Hyo Sup Shim, Chang Young Lee, Seong Yong Park, Min Hee Hong, Insuk Lee, Hye Ryun Kim

**Affiliations:** 1 Department of Biotechnology, College of Life Science and Biotechnology, Yonsei University, Seoul, 03722 Republic of Korea; 2 Department of Pathology, Severance Hospital, Yonsei University College of Medicine, Seoul, 03722 Republic of Korea; 3 Department of Thoracic and Cardiovascular Surgery, Yonsei University College of Medicine, Seoul, 03722 Republic of Korea; 4 Division of Medical Oncology, Department of Internal Medicine, Yonsei Cancer Center, Yonsei University College of Medicine, Seoul, 03722 Republic of Korea

**Keywords:** Cancer genomics, Non-small-cell lung cancer

## Abstract

Lung squamous cell carcinoma (LUSC) is a subtype of non-small cell lung cancer (NSCLC). LUSC occurs at the bronchi, shows a squamous appearance, and often occurs in smokers. To determine the epigenetic regulatory mechanisms of tumorigenesis, we performed a genome-wide analysis of DNA methylation in tumor and adjacent normal tissues from LUSC patients. With the Infinium Methylation EPIC Array, > 850,000 CpG sites, including ~350,000 CpG sites for enhancer regions, were profiled, and the differentially methylated regions (DMRs) overlapping promoters (pDMRs) and enhancers (eDMRs) between tumor and normal tissues were identified. Dimension reduction based on DMR profiles revealed that eDMRs alone and not pDMRs alone can differentiate tumors from normal tissues with the equivalent performance of total DMRs. We observed a stronger negative correlation of LUSC-specific gene expression with methylation for enhancers than promoters. Target genes of eDMRs rather than pDMRs were found to be enriched for tumor-associated genes and pathways. Furthermore, DMR methylation associated with immune infiltration was more frequently observed among enhancers than promoters. Our results suggest that methylation of enhancer regions rather than promoters play more important roles in epigenetic regulation of tumorigenesis and immune infiltration in LUSC.

## Lung cancer: importance of enhancer methylation in tumor development

The aberrant methylation of specific regions of DNA may play a significant role in regulating tumorigenesis in lung squamous cell carcinoma (LUSC). LUSC development is linked to smoking, but epigenetic regulation mechanisms in LUSC are unclear. Hye Ryun Kim at Yonsei University College of Medicine and Insusk Lee at Yonsei University in Seoul, Korea, and co-workers compared DNA methylation patterns in samples of tumor and adjacent normal tissues from 37 LUSC patients. They paid particular attention to differentially methylated regions (DMRs) that overlapped short DNA sequences that regulate gene expression. DMRs overlapping enhancers—regions that regulate expression of a gene from a distance—rather than promoters were more associated with tumor formation from normal tissues, suggesting that methylation of enhancers rather than promoters could be a more important player in cancer development.

## Introduction

Lung squamous cell carcinoma (LUSC) is a subtype accounting for 20~30% of total cases of non-small-cell lung cancer (NSCLC). LUSC occurs at the bronchi, shows a squamous appearance, and often leads to keratinization^1^. LUSC often occurs in smokers; thus, several studies have demonstrated an association between smoking and LUSC^2,3^. The identification of oncogenic driver alterations and their matched targeted therapies are largely limited to the other major subtype of NSCLC, adenocarcinoma^4^. However, LUSC patients respond to immune checkpoint blockade, partly owing to their relatively high mutation burden. Therefore, many clinical trials on immune checkpoint blockade have been undertaken for LUSC patients^5^. Currently, the fibroblast growth factor receptor (FGFR), insulin-like growth factor (IGF), and PI3-AKT signaling pathways have been examined as new targeted therapies^4^.

For improved treatment strategies for LUSC, a better understanding of the molecular mechanisms associated with the regulation of tumorigenesis and tumor immunogenicity is necessary. Given the substantial impact of epigenetic regulation on tumor biology, genome-wide analysis of DNA methylation and the associated transcriptome have been utilized for several cancer types, including LUSC^6^. The human genome has much larger distal regulatory regions than proximal ones, and the former are called enhancers and the latter promoters. Despite occupying a much larger portion of genomic DNA than promoters and exerting a substantial effect on cancer progression and prognosis^7,8^, enhancers have been relatively unexplored for the study of DNA methylation in the majority of cancer types, including LUSC. This lack of research is partial because the major platform technology for DNA methylation profiling is the Infinium HumanMethylation450 BeadChip (450 K), which mostly covers the annotated promoter regions. Recently, the Infinium Methylation EPIC Array (850 K), which covers > 850,000 CpG sites, including ~350,000 CpG sites for enhancer regions, has become available^9^. Therefore, more comprehensive and cost-effective studies of enhancer DNA methylation in human tumor samples are now feasible.

In the present study, we generated methylation profiles for 37 patients with LUSC who were recruited from Korea using an Infinium Methylation EPIC Array (850 K) and identified differentially methylated regions (DMRs). These DMRs were then subdivided into two groups: those overlapping with annotated promoter regions (pDMRs) and those overlapping with annotated enhancer regions (eDMRs). Through dimension reduction analysis, we found that eDMRs alone and not pDMRs alone can classify tumors from normal tissues with equivalent performance to total DMRs. We observed a stronger negative correlation of LUSC-specific gene expression with methylation for enhancers rather than promoters. Gene set enrichment analysis revealed that target genes of eDMRs rather than pDMRs were enriched for tumorigenic genes and pathways. In addition, we found that single nucleotide polymorphisms (SNPs) related to the risk of LUSC were enriched within DMRs. Finally, methylation-associated immune infiltration was more frequently observed among enhancers than promoters. These results together suggest that methylation of enhancers rather than promoters play more important roles in epigenetic regulation of tumorigenesis and immune infiltration in LUSC.

## Materials and methods

### Patient cohorts

A study cohort of 37 LUSC patients was established by recruiting patients from Yonsei Cancer Center (YCC), Seoul, Korea (Supplementary Table 1). Tumor tissue and adjacent normal tissue biopsies were performed on each patient during surgery.

### DNA methylation profiling

Thirty-seven pairs of fresh tumor tissue and adjacent normal tissue specimens were selected from the archives of Severance Hospital. The DNA methylation profiles of the patients were obtained using the Infinium Methylation EPIC Array (850 K). The experimental procedure for DNA methylation profiling is described in the Supplementary Methods.

### DNA methylation analysis

Raw methylation data (IDATs) were processed by the RnBeads^10^ and Minfi^11^ packages. Prior to data processing, the getQC function of the Minfi package was used to evaluate sample quality, followed by functional normalization. Using RnBeads, we filtered out noninformative CpG sites by removing the sites with a detection *P* value > 0.01 using “remove.sites”. Thereafter, the rnb.execute.low.coverage.masking, rnb.execute.sex.removal, rnb.execute.context.removal, rnb.execute.cross.reactive.removal, rnb.execute.snp.removal, and rnb.execute.greedycut functions were applied. Given that the patients were from a Korean population, we additionally removed Korean single nucleotide polymorphisms (SNPs) with minor allele frequencies >0.01, as per the KOVA^12^ and KRGDB (KRGDB, http://152.99.75.168/KRGDB/menuPages/intro.jsp) databases. Consequently, 651,062 of 866,895 CpG sites remained. DMRs were identified by the RnBeads package, which evaluates the methylation level of a given region such as a promoter or enhancer and calculates the significance of differences between comparison groups. We used the predefined promoter region from the RnBeads package and assigned enhancers based on a publicly available enhancer-promoter interaction (EPI) map specific for lung cancer^13^. This EPI map was constructed by using four types of chromatin interaction data: chromatin interaction analysis by paired-end tag sequencing (ChIA-PET), correlations between proximal and distal DNase I hypersensitive sites (DHSs), the association of cap analysis gene expression (CAGE) tag correlation between enhancer RNAs (eRNAs) and mRNAs, and integrated methods for predicting enhancer targets (IM-PET) with stringent filtration for PET counts ≥ 5. For each differentially methylated enhancer, target genes were mapped using the same EPI map. We collected pDMRs from the promoter region and eDMRs from the enhancer region, in which the false discovery rate (FDR) < 0.01. Finally, target genes were filtered with the Consensus Coding gene sequence^14^ database. Among the DMRs and DEGs, we defined functional DMRs as those showing an opposite direction of change in the methylation and gene expression levels, which share the same target gene.

### Gene expression analysis

RNA sequencing was performed for the same 37 pairs of fresh tumor tissue and adjacent normal tissue specimens from previous patients. Each sample was subsequently applied for sequencing library preparation, which was conducted using TruSeq RNA Access Library Prep Guide Part # 15049525 Rev. B with the TruSeq RNA Access Library Prep Kit (Illumina). RNA sequencing was performed with a HiSeq 2500 system (Illumina), and the obtained sequencing data were processed according to the manufacturer’s protocol. Trimming was performed using Trimmomatic 0.32^15^. Thereafter, STAR-2.5.2a^16^ was applied to the reference genome (GENCODE, h19 (GRCh37.p13, release 19)) for read mapping^17^. FeatureCounts^18^ was used for transcript quantification. Differentially expressed genes were analyzed using DESeq2^19^. The threshold for DEGs was given as |logFC | >2 and a *q*-value < 0.01.

### The cancer genome atlas (TCGA) DNA methylation data analysis

The methylation data (450 K) of LUSC patients who had paired tumor and normal samples were obtained from TCGA (https://gdac.broadinstitute.org/). Each CpG site collapsed into a single gene that shared the same promoter region. pDMRs were obtained by the same process as YCC data.

### Dimension reduction and clustering of samples based on DMR profiles

Uniform manifold approximation and projection (UMAP) and t-stochastic neighbor embedding (tSNE) were performed with beta values of pDMRs alone, eDMRs alone, or total DMRs in the tumor and normal samples from the YCC cohort. tSNE was performed by using the Rtsne package in *R* with perplexity 10. UMAP was performed by using the Seurat package with the RunUMAP function (dims = 1:10). K-means clustering was performed by using the k-means function in *R* with the number of clusters = 2. The entropy was calculated for cluster evaluation.

### Correlation analysis between methylation and gene expression of LUSC-specific genes

We obtained TCGA data downloaded from the UCSC XENA browser. LUSC-specific genes were defined by comparing 32 other cancer types and normal LUSC samples with a fold change > 2 and *p*-value < 0.05 using the one-sided Wilcoxon signed-rank test. LUSC-specific genes compared to lung adenocarcinoma (LUAD) were also obtained via the same procedure above, except the comparison set was only LUAD. We analyzed overlapping genes only between TCGA data and our cohort. Genes with expression levels < 1 were removed from all the samples before correlation analysis. Correlation analysis between the methylation value and the expression value was obtained by Spearman’s correlation coefficient from the promoter and enhancer regions, respectively.

### Gene set enrichment analysis

We performed enrichment analysis using Fisher’s exact test. We also used the Benjamini-Hochberg (BH) correction to obtain the FDR for multiple gene set analysis. We compiled gene sets from various databases: cancer hallmark gene sets from CancerSEA^20^, cancer-associated genes from CancerMine^21^ using information for NSCLC only, and pathway gene sets from Reactome^22^. We compiled 125 cancer-testis (CT) genes from Cheng et al.^23^ by taking extremely highly expressed genes in tumors of the TCGA-LUSC cohort, which were annotated as the “high-confidence testis-specific coding genes” (C1) group.

### Super-enhancer (SE) analysis

The SEs of lung tissue were compiled from Hnisz et al.^24^. DMRs for SE regions (seDMRs) were defined by their overlap with eDMRs. seDMR target genes were obtained from the target genes of overlapping eDMRs.

### Risk-related SNP enrichment analysis

The risk-related SNPs for LUSC were obtained from the genome-wide association study (GWAS) catalog (https://www.ebi.ac.uk/gwas/home)^25^. SNPs related to the risk of LUSC were collected from GWASs that contained LUSC patients. We used hg19 coordinates to annotate these SNPs. We assigned a risk-related SNP on a DMR if the SNP was located within 5 kbp from the DMR. Thereafter, we tested the significance of enrichment of these SNPs among DMRs using the binomial distribution.

### Immune infiltration analysis

The proportion of immune infiltrates was evaluated using CIBERSORTx^26^ with the default signature matrix (LM22) from matched RNA-seq data. We only considered differentially abundant immune infiltrates between tumor and normal tissue obtained using the Wilcoxon signed-rank test with BH correction (*q*-value < 0.01). The correlation between the methylation level and the abundance of immune infiltrates was obtained by Spearman’s correlation analysis with *q*-value < 0.01 by BH correction. For the overlap analysis for genomic regions of interest with partially methylated domains (PMDs), we obtained PMD regions for EPIC array from Zhou et al. (https://zwdzwd.github.io/pmd)^27^ and filtered for the 850 K CpG sites.

## Results

### Overview of differentially methylated regions in LUSC

**Fig. 1:**
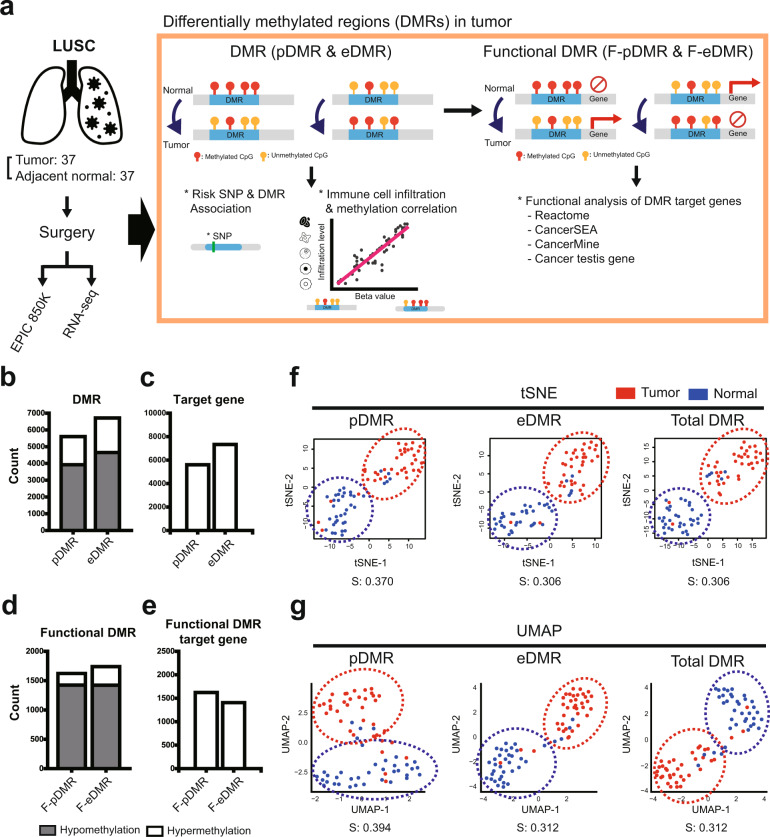
Overview of differentially methylated regions in lung squamous cell carcinoma (LUSC). **a** Overview of our genome-wide methylation analysis: (i) Study design, (ii) Analysis of differentially methylated regions (DMRs), risk-related single nucleotide polymorphisms (SNPs) and immune infiltration, and (iii) Gene set enrichment analysis of functional DMRs. **b**–**e** Stacked bar plots for the count of promoter DMRs (pDMRs) and enhancer DMRs (eDMRs) (**b**), the count of target genes of pDMRs and eDMRs (**c**), the count of functional pDMRs (F-pDMRs) and functional eDMRs (F-eDMRs) (**d**), and the count of target genes of F-pDMRs and F-eDMRs (**e**). **f**–**g** Dimension reduction of methylation profiles for tumor and normal samples using t-stochastic neighbor embedding (tSNE) (**f**) and uniform manifold approximation and projection (UMAP) (**g**).

The overall strategy of our study is summarized in Fig. 1a. Using the Infinium Methylation EPIC Array, we profiled the methylation status of > 850,000 CpG sites for tumors and adjacent normal tissues obtained from 37 LUSC patients. We also profiled the transcriptome of these samples using RNA sequencing. Thereafter, we obtained DMRs between tumors and normal tissues from promoter and enhancer regions using the RnBeads package^10^. To identify DMRs overlapping with promoter or enhancer DNA regions, we first selected annotated promoters and enhancer regions. Thereafter, we considered only 17,660 promoter regions and 27,283 enhancer regions that can be profiled by Infinium Methylation EPIC Array. RnBeads analysis identified 5603 pDMRs (3924 hypo- and 1679 hypermethylated) and 7332 eDMRs (4654 hypomethylated and 2061 hypermethylated) (Fig. 1b and Supplementary Table 2a-e). Hypomethylation was dominant in both the pDMRs and eDMRs, which is consistent with the previous observation that cancer cells tend to be hypomethylated compared to normal cells^28^. To validate the DMR analysis quality, we compared pDMRs from this study with those identified from the TCGA cohort (TCGA-LUSC). We found that the pDMRs from our study significantly overlapped with those from the TCGA cohort (*p* < 0.0001 by Fisher’s exact test, Supplementary Fig. 1a), retrieving > 66% of the pDMRs from TCGA-LUSC.

Functional interpretation of methylation dynamics at regulatory regions requires information on their target genes (Fig. 1c and Supplementary Table 2f-g). Genes located downstream of each promoter were assigned as promoter targets. Thus, the number of pDMR target genes was 5603, which was the same as the number of pDMRs. We compiled enhancer targets based on the enhancer-promoter interaction network in lung cancer^13^. Given that an enhancer can interact with multiple promoters, the number of eDMR target genes might differ from the number of eDMRs. We identified 7332 eDMR target genes. Functional DMRs (F-pDMRs and F-eDMRs) were defined using an inverse relationship between DMR methylation and the differential expression of target genes (Supplementary Table 3). We found that both F-pDMRs and F-eDMRs were biased toward hypomethylation (Fig. 1d). The number of target genes was similar between the F-pDMRs and F-eDMRs (Fig. 1e).

### eDMR profiles outperformed pDMR profiles in classifying tumors from normal tissues

Epigenetic profiles characterize cellular states. Thus, we can distinguish tumors from normal tissues based on the DMR profiles of samples. We compared epigenetic profiles based on pDMRs alone, eDMRs alone, and total DMRs to distinguish tumors from normal tissues using dimension reduction and clustering analysis. We utilized two commonly used nonlinear manifold approaches for dimension reduction methods: tSNE and UMAP. We observed well-separated samples based on DMR profiles with both tSNE and UMAP (Fig. 1f, g). K-means clustering identified two groups of samples, tumor, and normal tissues. We assessed the quality of clusters based on entropy measures, where lower entropy indicates better classification between tumors and normal tissues. Notably, profiles with eDMRs alone showed as good classification performance as profiles with total DMRs with both tSNE and UMAP. In contrast, profiles of pDMRs alone showed inferior classification performance to the profiles of total DMRs. These results suggest that enhancer methylation is the dominant epigenetic factor that characterizes tumors compared with normal tissues in LUSC.

### Methylation of enhancers has a stronger effect on LUSC-specific gene expression than that of promoters

**Fig. 2:**
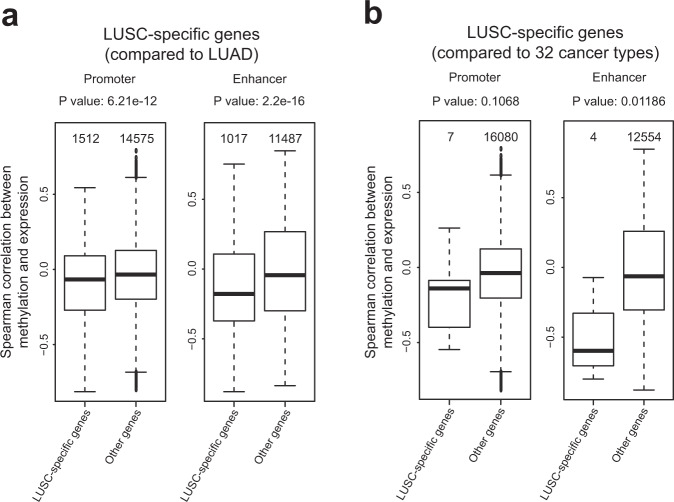
Correlation analysis of methylation with lung squamous cell carcinoma (LUSC)-specific gene expression. **a** The box plot shows the correlation of methylation with the expression of LUSC-specific genes (compared to TCGA-LUAD) vs. all other genes. **b** The box plot shows the correlation of methylation with the expression of LUSC-specific genes (compared to 32 TCGA cancer types and normal tissue of LUSC) vs. all other genes. Significance was assessed with a two-sided Wilcoxon signed-rank test. Box plots show the median, first and third quartiles, and outliers.

LUSC has distinct characteristics from LUAD, another major type of NSCLC, and other types of cancers with different tissue origins. The phenotypic and molecular properties specific to LUSC might be associated with LUSC-specific gene expression. To study the influence of DNA methylation of promoter and enhancer regions on LUSC-specific gene expression, we measured the correlation between the methylation level of the regulatory regions and their targets, which are LUSC-specific genes. A similar analytical scheme was used for the study of DNA methylation in advanced prostate cancer^29^. We first defined two different sets of LUSC-specific genes: (1) differentially expressed genes compared with LUAD and (2) differentially expressed genes compared with all other types of cancers, including LUAD and normal tissue of LUSC. We identified LUSC-specific genes using transcriptome data based on 33 types of cancers from the TCGA cohort (Supplementary Table 4). We found 1512 and 1017 LUSC-specific genes compared to LUAD among pDMR and eDMR targets, respectively, and found that DMR methylation was significantly more negatively correlated with their expression than that of other genes (Fig. 2a). Notably, the negative correlation between methylation and target gene expression was stronger for eDMRs. We also found seven and four LUSC-specific genes, compared to 32 other types of cancers from the TCGA cohort and normal tissue of LUSC, among pDMR and eDMR targets, respectively. Similarly, LUSC-specific genes showed a more negative correlation with pDMRs and eDMRs than nonspecific genes (Fig. 2b). However, only eDMR-targeted LUSC-specific genes showed a significantly stronger negative correlation with methylation than nonspecific genes. These results suggest that DNA methylation regulates the expression of genes involved in LUSC-specific properties, and eDMRs have a stronger regulatory effect than pDMRs on LUSC-specific gene expression.

### eDMR rather than pDMR target genes are associated with tumorigenesis in LUSC

**Fig. 3:**
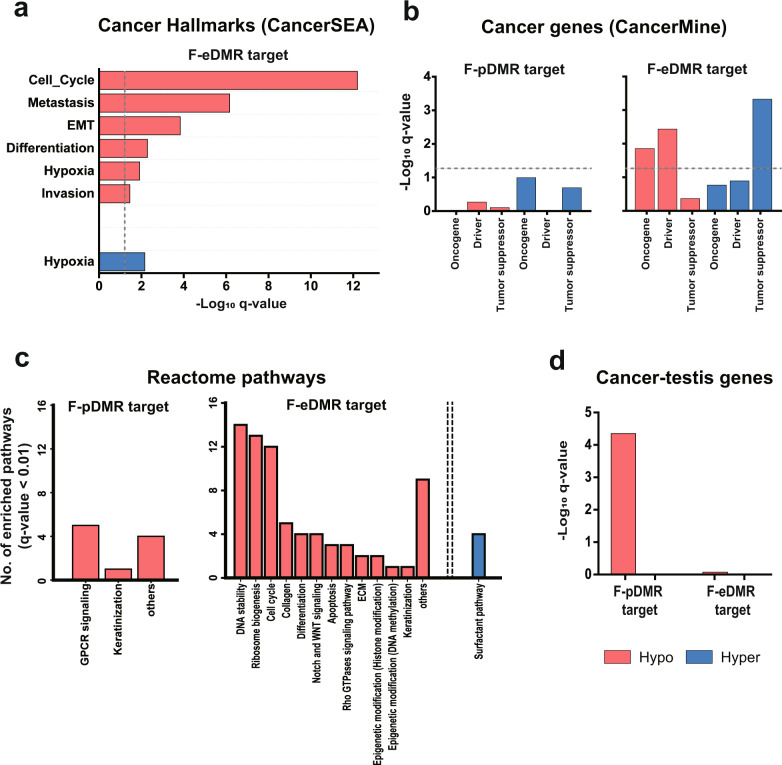
Gene set enrichment analysis for target genes of promoter differentially methylated regions (pDMRs) and enhancer differentially methylated regions (eDMRs). **a** Enrichment of cancer hallmark gene sets from the CancerSEA database for functional (F)-eDMR target genes. The dashed line indicates the significance threshold (*q*-value < 0.05). **b** Enrichment of each category of cancer-associated genes from the CancerMine database for F-pDMR and F-eDMR target genes. The dashed line indicates the significance threshold (*q*-value < 0.05). **c** Enrichment of Reactome pathway gene sets for F-pDMR and F-eDMR target genes. The bar graph indicates the number of significantly enriched pathways (*q*-value < 0.01) for each category. **d** Enrichment of cancer-testis genes for F-pDMR and F-eDMR target genes.

For functional interpretation of epigenetic regulation mediated by DNA methylation in LUSC, we performed gene set enrichment analysis for DMR targets using various databases for pathway and cancer-associated processes. First, we assessed the enrichment of functional DMR targets for cancer hallmark genes by CancerSEA^20^. Notably, we observed no significantly enriched cancer hallmarks among the F-pDMR targets (by *q*-value <0.05). In contrast, the F-eDMR targets were significantly enriched in “cell cycle”, “metastasis”, “EMT”, “differentiation”, “hypoxia”, and “invasion” with hypomethylation and enriched for “hypoxia” with hypermethylation (Fig. 3a and Supplementary Table 5a). This result indicates that methylation of enhancers rather than promoters is largely attributable to the epigenetic regulation of cancer hallmark pathways in LUSC.

Thereafter, we tested the enrichment of functional DMR targets for cancer-associated genes such as oncogenes, tumor suppressor genes, and driver genes annotated by the CancerMine^21^ database. Consistent with the enrichment analysis for cancer hallmark genes, we observed significant enrichment of cancer genes among the F-eDMR but not F-pDMR targets (by *p*-value < 0.05, Fig. 3b and Supplementary Table 5b-c). Notably, we found that oncogenes and driver genes were enriched for hypomethylated eDMR targets (i.e., potentially upregulated in tumors), while tumor suppressor genes were enriched for hypermethylated F-eDMR targets (i.e., potentially downregulated in tumors). This result suggests that methylation of enhancer rather than promoter regions primarily mediates epigenetic control of tumor progression in LUSC.

In addition to canonical pathways for tumorigenesis, other biological processes may be associated with LUSC characteristics. Thus, we performed gene set enrichment analysis of functional DMR targets for pathway genes annotated by the Reactome^22^ database. There were ten pathways enriched among the F-pDMR targets (*q*-value < 0.01), which could be categorized into “G-protein-coupled receptor (GPCR) signaling”, “keratinization”, or others (Fig. 3c and Supplementary Table 5d). GPCRs comprising a large family of cell-surface receptors are involved in tumorigenesis, and their signaling pathway is a major target for cancer drug development^30^. Keratinization is one of the most common features of LUSC and is associated with poor clinical outcomes^31^. These results indicate that methylation of promoter regions mediates epigenetic regulation of genes involved in tumor-associated GPCR signaling and keratinization in LUSC. These pathways were also found to be most significantly enriched among functional F-pDMR targets in the TCGA-LUSC cohort (Supplementary Fig. 1b, Supplementary Tables 5e, and 6), which validates our findings.

Similar to the results with other cancer-associated genes, we observed a much stronger association of F-eDMR targets with Reactome pathways. There were 73 and four enriched pathways among hypomethylated and hypermethylated F-eDMR targets, respectively (Fig. 3c and Supplementary Table 5f). The majority of enriched pathways were associated with cancer progression and were assigned to 14 categories. Several were involved in cellular proliferation and migration, such as “DNA stability”, “cell cycle”, “ribosome biogenesis”, “apoptosis”, “differentiation”, “Notch and WNT signaling”, “Rho GTPase signaling”, and “ECM”. Epigenetic modification pathways were also enriched for F-eDMR targets and were previously reported to be involved in the regulation of cancer progression^32^. “Collagen” is another enriched pathway known to accelerate fibrosis in lung cancer, leading to poor prognosis^33,34^. The surfactant-related pathway was enriched for target genes regulated by hypermethylated F-eDMR. Previously, the anticancer activity of surfactant proteins was reported in multiple studies^35–37^. Therefore, downregulated surfactant pathways in tumors could promote cancer progression in LUSC. Overall, pathway enrichment analysis using the Reactome database confirmed the association of DMR target genes with tumorigenesis and the dominant regulatory roles of enhancer methylation in LUSC.

### pDMRs play dominant roles in regulation of cancer-testis gene expression in LUSC

Our unbiased functional analysis of DMR targets revealed the dominant roles of enhancer methylation in the regulation of tumorigenesis and other pathways that characterize neoplasms in LUSC. While seeking cancer-associated gene families primarily regulated by methylation of promoter rather than enhancer regions, we found that CT genes were greatly enriched among the F-pDMR targets. Gene products of CT genes are CT antigens composed of a large family of tumor-associated antigens. CT antigens are expressed in human tumors and not in normal tissues, except in germ cells such as testis and placenta. These antigens can be abnormally expressed in cancer cells and thus are considered promising targets for cancer immunotherapy. However, the molecular functions of CT antigens in germ or tumor cells remain largely unknown, although evidence of their contribution to tumor cell physiology and neoplastic behaviors has accumulated^38^. We obtained 125 high-confidence CT genes using the extremely high expression in LUSC tumors^23^ and subsequently performed enrichment analysis for both F-pDMR and F-eDMR targets. Notably, CT genes were significantly enriched among F-pDMR targets only (Fig. 3d and Supplementary Table 5g). In addition, only hypomethylated DMR targets were enriched for CT genes, which is consistent with a previous observation of a negative correlation between the average promoter methylation levels of CT genes and the number of activated CT genes^23^. These results suggest that, contrary to other types of cancer-associated genes, epigenetic regulation of CT antigen expression is primarily controlled by promoter methylation in LUSC.

### Methylation of super-enhancers is associated with tumorigenesis in LUSC

**Fig. 4:**
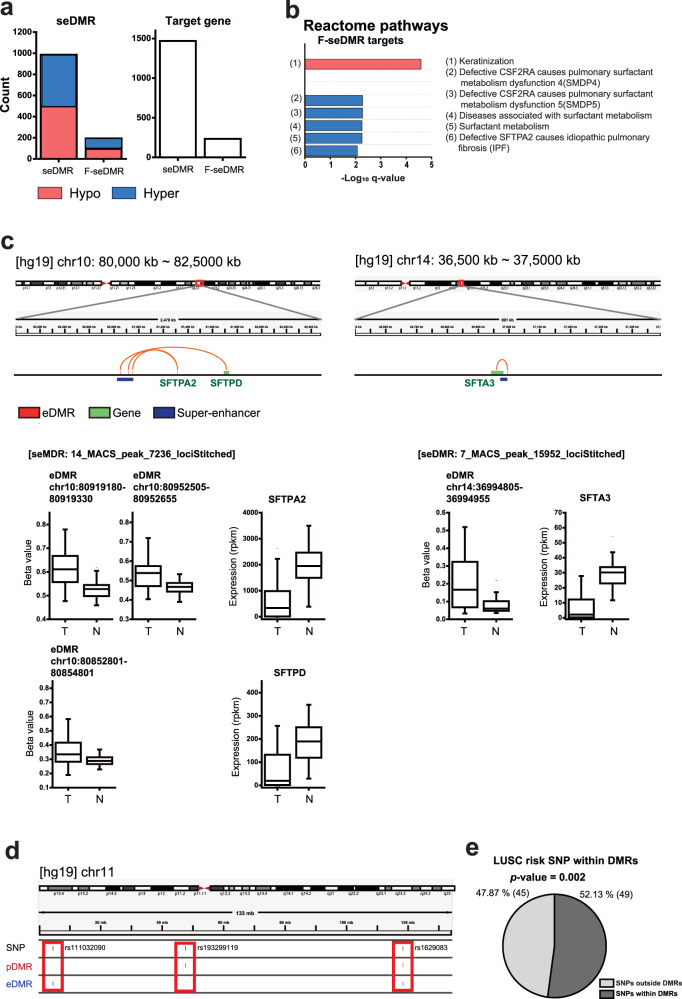
Functional analysis of super-enhancer differentially methylated region (seDMR) targets and enrichment of lung squamous cell carcinoma (LUSC) risk-related single nucleotide polymorphisms (SNPs) for DMRs. **a** Stacked bar plots of the number of seDMRs, functional (F)-seDMRs, and their target genes with hypo- or hypermethylation. **b** Enrichment of Reactome pathway gene sets for F-seDMR target genes. **c** An example of seDMR and target interactions visualized with Integrative Genomic View (IGV). Methylation levels of seDMR regions and expression levels of their target genes were compared between tumor (T) and normal tissue (N). **d** An example of LUSC risk-related SNPs residing within DMR regions. **e** Pie chart for the distribution of LUSC risk-related SNPs within DMRs and outside of DMRs. The significance of SNPs within DMRs was assessed using binomial distribution.

Previously, aberrant DNA methylation of the SE region in human cancer was demonstrated by whole-genome bisulfite sequencing^39^. SEs are known to play key roles in the control of cell identity and diseases^24^. Given the strong influence of enhancer methylation on tumorigenesis in LUSC, we hypothesized that SEs also play important roles in epigenetic regulation of cancer progression. Based on the overlap between annotated SEs and DMRs, we defined super-enhancer DMRs (seDMRs) (Supplementary Table 7a-b). We found ~1000 seDMRs composed of an approximately equal number of hypomethylated and hypermethylated regions and found ~1500 target genes (Fig. 4a and Supplementary Table 7c-f). When we filtered them by the negative correlation between methylation and target gene expression levels to identify functional seDMRs (F-seDMRs), we obtained < 200 F-seDMRs. We found that Reactome pathways for “keratinization” and “surfactant metabolism” were significantly enriched for target genes of hypomethylated and hypermethylated F-seDMRs, respectively (*q*-value < 0.01, Fig. 4b and Supplementary Table 7g). These results support the known protumor and antitumor activities of keratinization and surfactant, respectively^31,35–37^. Notably, all five enriched Reactome pathways among hypermethylated F-seDMR targets were associated with surfactant metabolism. Pulmonary surfactant is a lipoprotein complex involved in various pulmonary functions, including compliance. Surfactant proteins A and D are involved in innate immunity by opsonizing bacterial cells in the alveoli. We found that genes for surfactant protein A2 (*SFTPA2*), surfactant-associated protein A3 (*SFTA3*), and surfactant protein D (*SFTPD*) are target genes of two hypermethylated F-seDMRs (Fig. 4c). These results suggest that the downregulation of the expression of surfactant proteins by hypermethylation of SEs may be a major epigenetic regulatory mechanism for promoting tumor progression in lung tissue.

### LUSC risk-related SNPs are enriched for DMRs

Previously, we investigated the functional roles of methylation at promoters and enhancers using their regulatory target genes. Thereafter, we confirmed the regulatory importance of DMRs in LUSC tumorigenesis using the orthogonal approach. Over 90% of disease-associated SNPs are located in non-coding regions, and the majority are believed to be involved in the regulation of gene expression. Disease-associated SNPs or risk-related SNPs were shown to be associated with DNA methylation changes in diseases, including cancers^40–42^. Therefore, we hypothesized that SNPs that increase the risk of LUSC are associated with regulatory regions that change the methylation level in tumors. We compiled LUSC risk-related SNPs from eight categories of the GWAS catalog^25^ that contain LUSC patients (Supplementary Table 8a). Among 94 SNPs that could be profiled by the Infinium Methylation EPIC Array, 49 overlapped with DMRs (Supplementary Table 8b-e). For example, rs193299119 was found in the pDMR, rs111032090 was found in the eDMRs, and rs1629083 was found in both the pDMRs and eDMRs (Fig. 4d). We found that LUSC risk-related SNPs were significantly enriched for DMRs (Fig. 4e, *p*-value = 0.002, by binomial distribution). These results confirm the importance of DNA methylation in epigenetic regulation of LUSC tumorigenesis. Owing to the limited number of risk-related SNPs, we did not observe significant enrichment for either pDMRs or eDMRs alone.

### eDMRs are more important in the control of immune infiltration than pDMRs

**Fig. 5:**
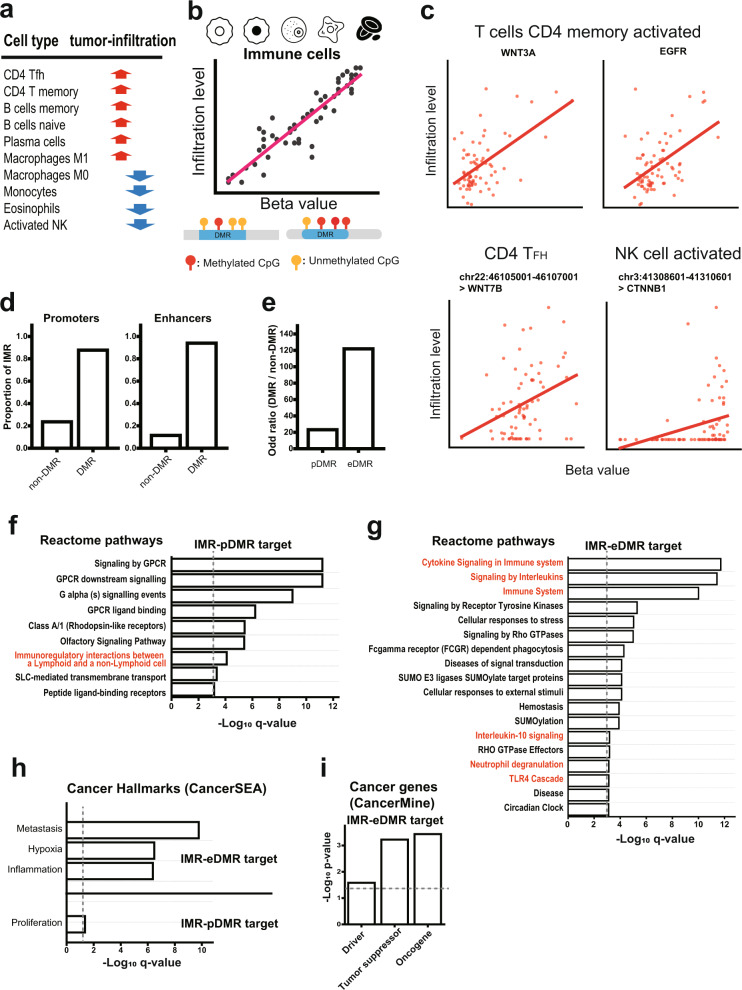
Regulation of immune infiltration by DNA methylation. **a** Ten immune cell types that showed abundance changes in tumors. **b** Schematic overview to define infiltration-associated methylation regions (IMRs) using correlation analysis between methylation and immune cell infiltration (*q*-value < 0.01, Spearman’s correlation). **c** Examples of promoters and enhancers whose methylation level correlates with immune cell infiltration. **d** Proportion of IMRs among differentially methylated regions (DMRs) vs. non-DMRs. **e** Odds ratio of DMRs to non-DMRs among IMRs. **f**, **g** Reactome pathway gene sets significantly enriched for genes regulated by IMR-pDMRs (**f**) or by IMR-eDMRs (**g**) outside partially methylated domains (PMDs) (only those with *q*-value < 0.001 are presented in the plot). The dashed line indicates the significance threshold (*q*-value < 0.001) for the presented bar plots. **h** Enrichment of cancer hallmark gene sets from the CancerSEA database for the genes regulated by IMR-eDMRs or IMR-pDMRs outside PMDs. The dashed line indicates the significance threshold (*q*-value < 0.05). **i** Enrichment of each category of cancer-associated genes from the CancerMine database for the genes regulated by IMR-eDMRs outside PMDs. The dashed line indicates the significance threshold (*p*-value < 0.05).

Aberrant methylation may affect cancer cells as well as the tumor microenvironment^43^. Although LUSC has a higher tumor mutation burden than LUAD, LUSC patients did not benefit from targetable driver mutation treatment. Immune checkpoint inhibitors are thus more effective in LUSC treatment, and the tumor microenvironment might be a critical factor for LUSC immunotherapy. Therefore, we investigated the relationship between the level of DMR methylation and immune cell infiltration. The relative abundance of infiltrated immune cells was estimated by deconvolution of bulk transcriptome data using CIBERSORTx^26^ with a pre-established signature matrix (LM22). We found ten distinct types of differentially abundant immune cells between tumor and normal tissues (by *q*-value < 0.01, Fig. 5a and Supplementary Table 9a). For these immune cell types, we identified promoters and enhancers whose methylation level showed a positive or negative correlation with their infiltration level (by Spearman correlation *q*-value < 0.01, Fig. 5b and Supplementary Table 9b-e). We defined these promoters and enhancers as infiltration-associated methylation regions (IMRs). Several pDMRs and eDMRs showed a strong correlation between methylation and tumor infiltration levels for a particular type of immune cell. For example, activity changes in oncogenic pathways often modulate the tumor microenvironment^44,45^, and we observed a strong correlation between promoter or enhancer methylation and target gene expression for the MAPK pathway (EGFR) and WNT-beta-catenin pathways (CTNNB1, WNT3A and WNT7B) (Fig. 5c). We found a substantially higher proportion of DMRs than non-DMRs among IMRs (87.7% vs. 23.6% for promoters and 94% vs. 11.4% for enhancers) (Fig. 5d and Supplementary Table 9f). Notably, the odds ratio of DMRs over non-DMRs was much higher for enhancer regions (Fig. 5e), which suggests that eDMRs are more important in immune infiltration control than pDMRs.

We performed functional interpretations for IMRs overlapping pDMRs (IMR-pDMRs) and eDMRs (IMR-eDMRs). Previous work demonstrated that CpG hypermethylation of immune genes located in lamina-associated, late-replicating regions termed PMDs^27^ is associated with immune evasion^46^. We found that only 727 of 4915 IMR-pDMRs (14.8%) and 136 of 6313 IMR-eDMRs (2.2%) overlapped with the PMDs (Supplementary Table 10a). Twelve Reactome^22^ pathway gene sets were significantly enriched for genes regulated by IMR-pDMRs overlapping with PMDs (by *q*-value < 0.01), including those for “GPCR signaling” and “keratinization” (Supplementary Table 10b), which were also enriched for F-pDMR targets (Fig. 3c). However, we found no Reactome pathway gene sets enriched for genes regulated by IMR-eDMRs overlapping with PMDs (Supplementary Table 10c) due to their small proportion of the genome. Therefore, we extended gene set enrichment analysis to the genes regulated by IMR-DMRs outside PMDs. We identified a similar number of Reactome pathway gene sets significantly enriched for genes regulated by IMR-pDMRs outside PMDs (*q*-value < 0.01) (Fig. 5f and Supplementary Table 10d**)**. In contrast, we identified 71 Reactome pathway gene sets significantly enriched for genes regulated by IMR-eDMRs outside PMDs. Notably, 12 of them were relevant to immunity, whereas only a single pathway was relevant to the IMR-pDMR targets (Fig. 5g and Supplementary Table 10e**)**. Furthermore, cancer hallmarks by CancerSEA^20^ and cancer-associated genes by the CancerMine^21^ database were more associated (by *q*-value < 0.05) with genes regulated by IMR-eDMRs than IMR-pDMRs outside PMDs (Fig. 5h-i and Supplementary Table 10d-e). These results suggest that some DMRs regulate immune infiltration of tumors and support that eDMRs are more important than pDMRs in epigenetic regulation of tumor immunity in LUSC.

## Discussion

The human genome has larger noncoding regulatory DNA regions for enhancers than promoters. However, epigenetic regulatory roles have been primarily investigated for promoters, owing to a lack of cost-effective profiling array platforms and comprehensive maps for enhancers until recently. Given the positioning at a higher hierarchy for epigenetic regulation, enhancers may exert more global and drastic control over cellular identity and disease progression in cancer^47^. A stronger influence of eDMRs on cancer-associated gene expression was suggested previously^8^. Enrichment of risk-related SNPs among eDMRs was also reported in breast cancer^42^. However, these studies were based on HumanMethylation450 BeadChip (450 K), which has extremely limited coverage for enhancer regions. Therefore, there is an increasing demand for the study of enhancer methylation and its regulatory roles in human cancer with a more comprehensive landscape for enhancers. To the best of our knowledge, this is the first study that carried out genome-wide methylation profiles for LUSC patients using a methylation array, including >350,000 CpG sites for enhancer regions. These methylation profiles enabled a comprehensive comparison of the regulatory impact of methylation between promoters and enhancers in LUSC.

Overall, the results from our functional enrichment analysis demonstrated that methylation of enhancers rather than promoters plays major roles in tumorigenesis and immune infiltration. Given a comparable number of target genes between pDMRs and eDMRs, the observation of substantially greater regulatory roles of eDMR may not be attributable to the size effect. In addition to functional analysis using the cancer knowledge base, we performed data-driven functional analysis based on the negative correlation of the methylation level with the expression of LUSC-specific genes. This orthogonal approach showed that methylation of enhancers has a stronger regulatory effect on LUSC-specific genes than that of promoters. This finding suggests that enhancer methylation plays a major role in canonical cancer pathways and neoplastic characteristics specific to LUSC compared with other types of cancer. For example, perturbation of keratinization and pulmonary surfactant pathways have been reported to affect clinical outcomes mainly for LUSC^31,35–37^, and these factors were found to be associated with eDMR targets in this study. Furthermore, we found that genes encoding surfactant proteins are regulated by the methylation of SEs. This finding implies that perturbation of these pathways might be mediated by aberrant methylation of enhancers in LUSC.

As we observed a greater regulatory contribution of enhancer methylation than promoter methylation, genome-wide methylation analysis might be necessary with similar enhancer coverage of several other types of cancers. Such a cancer-wide investigation will reveal whether epigenetic regulation of tumorigenesis and immune infiltration mediated by enhancer methylation is the core regulatory architecture of cancers. Although we profiled >350,000 annotated enhancers in this study, many more enhancers were identified. This limitation may be overcome using bisulfite sequencing or improving methylation array coverage. The improved breadth (profiling more cancer types) and depth (covering more enhancers) of DNA methylation studies for tumor samples will facilitate our understanding of complex networks of epigenetic regulation in cancer.

## Supplementary information

Supplementary Materials

## Data Availability

Methylome and transcriptome data generated in this study were deposited at Gene Expression Omnibus (GSE158420 for RNA-seq data, GSE158422 for DNA methylation data, and GSE158433 for all data). All supplementary tables can be downloaded from https://netbiolab.org/wiki/Cho_etal_2021_Table_S1-10.zip.
