# Supplementary material for: The importance of enhancer methylation for epigenetic regulation of tumorigenesis in squamous lung cancer
Source: Exp Mol Med. 2022 Jan 5;54(1):12–22. doi: 10.1038/s12276-021-00718-4 (PMC8813945; doi:10.1038/s12276-021-00718-4)
Supplement: Supplementary file 1 — Supplementary Materials [file 12276_2021_718_MOESM1_ESM.pdf]

Supplementary Materials

The importance of enhancer methylation for epigenetic regulation of tumorigenesis in squamous lung cancer

Contents

- **Supplementary Methods**

- **Supplementary Fig. 1. Comparison of differentially methylated promoter regions (pDMRs) and their associated pathways between two cohorts: Yonsei Cancer Center (YCC) and TCGA**

- **Supplementary Table 1-10**

Supplementary Methods

DNA methylation profiling

37 pairs of fresh tumor tissue and adjacent normal tissue specimens were selected from the archives of Severance Hospital. DNA methylation profiles of the patients were obtained by using the Infinium Methylation EPIC Array (850K). DNA samples were assessed for their quality using a NanoDrop® ND-2000 UV-Vis Spectrophotometer (Thermo Fisher Scientific, Waltham, MA, USA). The samples were separated in agarose gels. Those with intact genomic DNA, showing no smear in the gel, were selected for subsequent experiments. Intact genomic DNA was diluted to 50 ng/µL based on Quant-iT PicoGreen (Invitrogen, Carlsbad, CA, USA) quantitation. Concentrations were adjusted according to these results.

For bisulfite conversion, 600 ng of input gDNA was required. Bisulfite-modified gDNA was prepared using the EZ DNA Methylation kit (Zymo Research) according to the manufacturer's instructions. Conversion reagent was added, followed by subsequent incubation in a thermocycler to denature the samples. CT-converted DNA was washed and de-sulfonated with de-sulfonation buffer, after which the DNA was washed again and eluted with 12 µL elution buffer. The whole-genome amplification process required 250 ng of input bisulfite-converted DNA (MA1) and created a sufficient quantity of DNA (1,000X amplification) for use on a single BeadChip in the Infinium methylation assay (Illumina RPM and MSM). After amplification, the product was fragmented using a proprietary reagent (FMS), precipitated with 2-propanol (plus precipitating reagent; PM1), and re-suspended in formamide-containing hybridization buffer (RA1). The DNA samples were denatured for 20 min at 95°C and placed in a humidified container for a minimum of 16 h at 48°C, allowing CpG loci to hybridize with the 50-mer capture probes.

Following hybridization, the BeadChip/Te-Flow chamber assembly was placed on a

temperature-controlled Tecan flow-through chamber rack, and subsequent washing, extension, and staining were performed by adding reagents to the Te-Flow chamber. For the allele-specific single-base extension assay, primers were extended by polymerase and labeled nucleotide mix (TEM), and then stained by repeated application of STM (staining reagent) and ATM (anti-staining reagent). After staining, the slides were washed with low-salt wash buffer (PB1), immediately coated with XC4, and imaged using the iScan System (Illumina). The iScan System has a two-color (532 nm/658 nm) confocal fluorescent scanner with 0.54 μm pixel resolution. The scanner excited the fluorophores generated during signal amplification/staining of the allele-specific (one color) extension products on the BeadChips. Image intensities were extracted using Illumina's GenomeStudio Software.

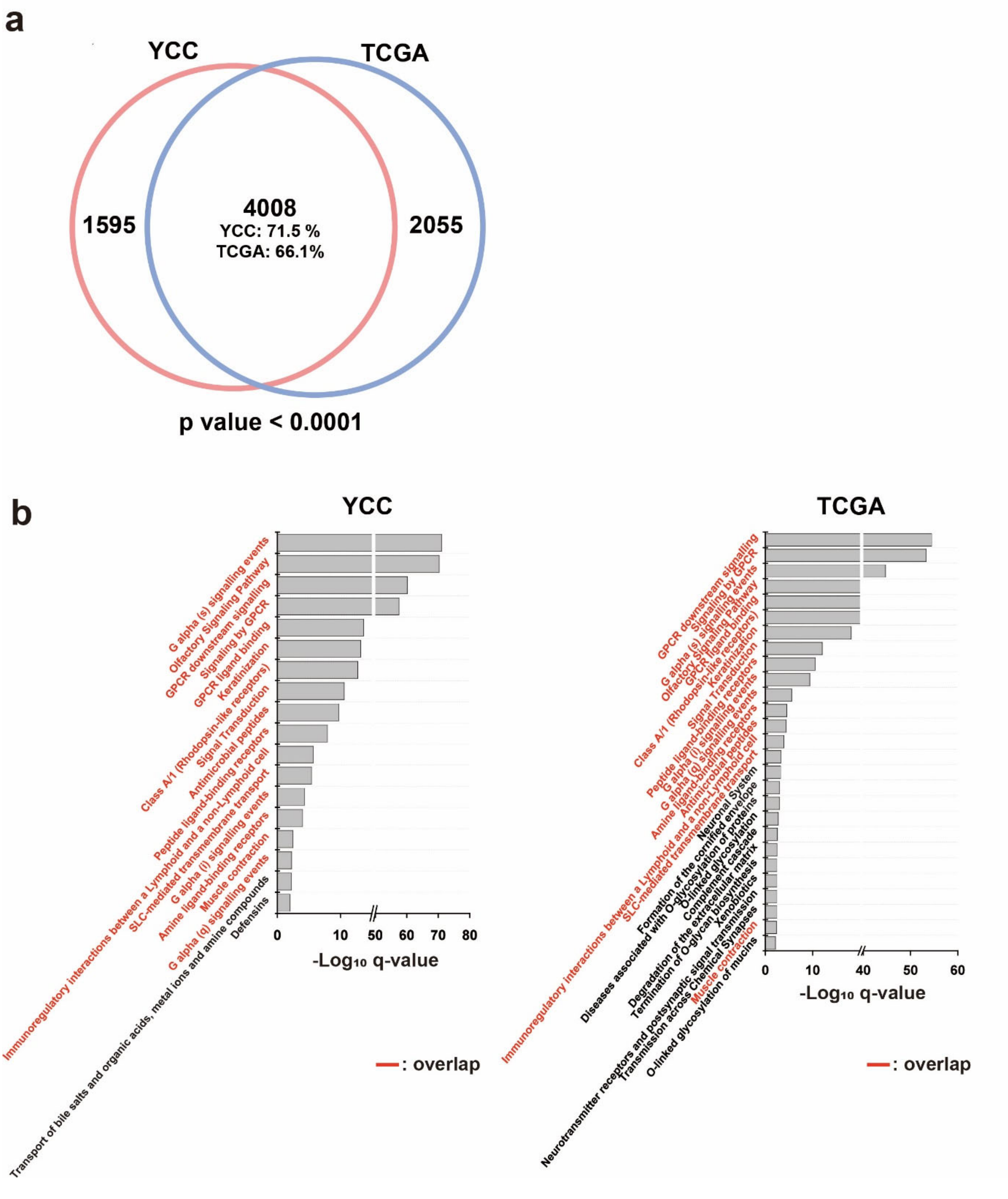

Supplementary Fig. 1. Comparison of differentially methylated promoter regions (pDMRs) and their associated pathways between two cohorts: Yonsei Cancer Center (YCC) and TCGA. (a) Venn diagram of pDMRs between YCC and TCGA cohorts. (b) Pathway enrichment analysis based on Reactome database. Pathways that overlap between the two cohorts are labeled by red color.

Supplementary Table 1. Summary of clinocopathology information of patients

Characteristics	Cancer type
	Squamous cell carcinoima
Number of patients	37
Age in years (median, range)	70 (55-80)
Sex	
Male	35 (95%)
Female	2 (5%)
Smoking History	
Never	3 (8.1%)
Former	10 (27%)
Current	26 (64.9%)
adjuvant CTx	
Yes(receive)	30 (81.1%)
No(not receive)	7 (18.9%)
Stage	
I	0 (0%)
II	26 (70.3%)
III	11 (29.7%)

Supplementary Table 2a. Statistics

	Detactable region	DMR	hypo	hyper	target gene
pDMR	17660	5603	3924	1679	5603
eDMR	27283	6713	4652	2061	7332

Supplementary Table 2b. Promoter results from RnBeads (DMR) analysis

gene	mean.mean.	mean.mean.g2	mean.mean.di	mean.mean.qu	comb.p.val	comb.p.adj.fdr	num.sites	mean.r	mean.num.na.g2
SYTL1	0.2671555	0.432841443	-0.16568597	-0.76933422	1.54E-16	1.38E-12	10	0	0
ZNF385A	0.3173793	0.447082321	-0.12970305	-0.94785034	8.62E-17	1.38E-12	11	0	0
PTPN6	0.3254868	0.496325503	-0.1708387	-0.69071572	4.24E-16	1.90E-12	12	0	0
TBX4	0.36327	0.099767942	0.263502053	2.001752064	3.84E-16	1.90E-12	6	0	0
MAP1LC3B2	0.3550477	0.565142995	-0.21009529	-0.71987495	1.20E-15	4.30E-12	10	0	0
KLRD1	0.7538305	0.85336907	-0.09953854	-0.17672483	2.89E-15	8.63E-12	1	0	0
TSTD1	0.3870941	0.54859151	-0.16149744	-0.81671016	1.11E-14	2.85E-11	13	0	0
RNASE3	0.3791339	0.637104846	-0.25797094	-0.73373278	1.69E-14	3.80E-11	1	0	0
CST7	0.3553437	0.575925609	-0.22058187	-0.80531327	2.31E-14	4.60E-11	2	0	0
HOXD12	0.3831914	0.189724389	0.193467033	1.090421391	4.54E-14	8.15E-11	12	0	0
HOXD4	0.4320586	0.213338012	0.218720591	1.050171904	5.19E-14	8.46E-11	17	0	0
HOXA5	0.5725208	0.356265726	0.216255095	0.678082228	6.41E-14	9.58E-11	35	0	0
LGALS9B	0.2249591	0.362168788	-0.13720971	-0.66354755	7.25E-14	1.00E-10	1	0	0
CDHR2	0.2930391	0.46757413	-0.174535	-0.60825245	1.05E-13	1.11E-10	10	0	0
SIM1	0.3664792	0.161860866	0.204618294	1.2288484	1.01E-13	1.11E-10	6	0	0
HOXB1	0.7704567	0.598909896	0.171546821	0.37511638	9.33E-14	1.11E-10	13	0	0
PARVG	0.438204	0.603502994	-0.16529898	-0.46686312	1.04E-13	1.11E-10	10	0	0
NUP210L	0.6010681	0.409366218	0.191701884	0.683162965	1.25E-13	1.25E-10	8	0	0
ATG16L1	0.6774855	0.854748972	-0.17726348	-0.33095213	1.78E-13	1.68E-10	1	0	0
PFN3	0.6815969	0.538663035	0.142933816	0.449153226	2.37E-13	2.13E-10	9	0	0
TCN1	0.5043939	0.720643515	-0.21624959	-0.58230476	2.68E-13	2.29E-10	5	0	0
PISD	0.2121195	0.347858079	-0.1357386	-0.76858983	2.91E-13	2.37E-10	6	0	0
OR6Q1	0.367029	0.589879742	-0.22285074	-0.6699978	3.25E-13	2.54E-10	1	0	0
CABLES1	0.3028302	0.465998632	-0.16316846	-0.6784503	4.71E-13	3.52E-10	8	0	0
SFTA3	0.2789536	0.125756465	0.15319718	1.177703095	5.42E-13	3.89E-10	15	0	0
ANTXRL	0.5050115	0.658594628	-0.15358316	-0.37652721	6.89E-13	4.46E-10	1	0	0
C17orf107	0.2020925	0.345201556	-0.14310905	-0.90996494	6.82E-13	4.46E-10	9	0	0
HOXB7	0.4630884	0.261898728	0.20118967	0.976591938	6.96E-13	4.46E-10	5	0	0
IL1A	0.4421877	0.588691081	-0.14650338	-0.44601705	8.13E-13	4.86E-10	6	0	0
EVPLL	0.3568325	0.521950556	-0.16511805	-0.53617068	7.90E-13	4.86E-10	1	0	0
SLCO2B1	0.0861606	0.188775829	-0.10261524	-1.04762475	8.43E-13	4.88E-10	1	0	0
AJAP1	0.2777195	0.093741198	0.1839783	1.540651424	1.30E-12	7.26E-10	4	0	0
IL1B	0.4315606	0.609701244	-0.17814059	-0.70110323	1.48E-12	8.07E-10	6	0	0
SCT	0.6452224	0.48865252	0.156569899	0.823851087	1.60E-12	8.47E-10	7	0	0
ZPBP	0.3948957	0.639122365	-0.24422667	-0.68094015	1.77E-12	9.07E-10	1	0	0
CD80	0.1373546	0.26664064	-0.12928602	-0.85173872	2.38E-12	1.19E-09	4	0	0
SPTA1	0.3078471	0.570359113	-0.26251206	-0.87411398	2.59E-12	1.20E-09	2	0	0
HOXD9	0.3965094	0.203996823	0.192512598	1.055429978	2.62E-12	1.20E-09	10	0	0
SERPING1	0.5721984	0.413405826	0.158792534	0.472247567	2.54E-12	1.20E-09	8	0	0
OR1S2	0.6436841	0.850416563	-0.20673242	-0.39644159	2.95E-12	1.32E-09	1	0	0
GRIK2	0.3323774	0.185816197	0.146561181	0.939193733	3.26E-12	1.39E-09	15	0	0
RNF135	0.4006495	0.690659824	-0.29001036	-0.77080676	3.22E-12	1.39E-09	1	0	0
HLA-DOB	0.6875483	0.8495973	-0.16204903	-0.30105058	3.40E-12	1.42E-09	2	0	0
CD5L	0.46191	0.550918505	-0.08900846	-0.24927931	4.11E-12	1.68E-09	1	0	0
SPATA12	0.3184265	0.490525597	-0.17209907	-0.6222379	5.48E-12	2.18E-09	3	0	0
REG1B	0.6747742	0.897074036	-0.2222998	-0.40559189	5.74E-12	2.24E-09	1	0	0
PLA2G6	0.5748673	0.448592041	0.126275235	0.350897954	6.07E-12	2.32E-09	1	0	0
GPBAR1	0.7619319	0.608792774	0.153139131	0.392340554	7.04E-12	2.58E-09	3	0	0
KIF2B	0.6702389	0.826737066	-0.15649814	-0.30667921	7.02E-12	2.58E-09	8	0	0
ART4	0.762676	0.654613743	0.108062282	0.231817878	7.81E-12	2.75E-09	13	0	0
ZNF358	0.6733654	0.504151056	0.169214362	0.460072571	7.82E-12	2.75E-09	4	0	0
OR51I1	0.6038741	0.771621766	-0.16774771	-0.35510578	9.42E-12	3.25E-09	2	0	0
OTX2	0.4319205	0.229406073	0.202514443	1.019839378	9.80E-12	3.32E-09	9	0	0
AGER	0.7342007	0.630235891	0.103964773	0.234450602	1.06E-11	3.52E-09	5	0	0
CIITA	0.3000933	0.456650973	-0.15655769	-0.64462492	1.13E-11	3.70E-09	10	0	0
IFNA2	0.4383932	0.628690954	-0.19029773	-0.55331635	1.24E-11	3.95E-09	2	0	0
FCN1	0.5844452	0.758434639	-0.17398947	-0.37038833	1.25E-11	3.95E-09	2	0	0
ADAM28	0.2165738	0.364614229	-0.14804043	-0.67511963	1.34E-11	4.12E-09	5	0	0
PRDM11	0.5144441	0.661948722	-0.14750461	-0.39614458	1.38E-11	4.12E-09	7	0	0
SCO1	0.1865768	0.319428372	-0.13285154	-0.53193381	1.37E-11	4.12E-09	3	0	0
GNA15	0.5257787	0.645649279	-0.11987059	-0.45175115	1.66E-11	4.88E-09	8	0	0

C1QTNF1	0.670038	0.542924131	0.127113827	0.34670957	1.70E-11	4.92E-09	10	0	0
FAM107A	0.5608188	0.416805137	0.144013699	0.421178157	1.88E-11	5.36E-09	4	0	0
GJC2	0.6756077	0.572683252	0.102924449	0.238009556	1.94E-11	5.43E-09	9	0	0
ZNF154	0.4217419	0.210547129	0.211194803	1.347678264	2.09E-11	5.76E-09	12	0	0
CCDC81	0.4792876	0.315343339	0.163944292	0.703263106	2.24E-11	6.08E-09	9	0	0
PAX3	0.2895078	0.139625724	0.149882112	1.126867387	2.34E-11	6.16E-09	15	0	0
DAPP1	0.3858309	0.568174643	-0.18234376	-0.58053943	2.31E-11	6.16E-09	7	0	0
GAPT	0.5415253	0.726966406	-0.18544109	-0.45218667	2.54E-11	6.61E-09	4	0	0
SPP1	0.4245541	0.570277583	-0.14572348	-0.58300793	2.72E-11	6.96E-09	7	0	0
RBM47	0.3956694	0.536346641	-0.14067724	-0.45703905	2.86E-11	7.18E-09	10	0	0
NEK8	0.2045464	0.290750944	-0.08620456	-0.65387901	2.88E-11	7.18E-09	4	0	0
ZKSCAN4	0.418589	0.25578412	0.162804843	1.001249557	2.97E-11	7.29E-09	9	0	0
SPOCK1	0.4016714	0.635996395	-0.23432502	-0.65003298	3.62E-11	8.77E-09	1	0	0
DPP6	0.3780699	0.203096837	0.174973054	0.981465054	3.82E-11	9.15E-09	13	0	0
OR10W1	0.5881209	0.774847941	-0.18672703	-0.39879133	4.19E-11	9.90E-09	3	0	0
SENP2	0.7950238	0.703382639	0.09164114	0.195951064	5.09E-11	1.19E-08	4	0	0
SLC7A11	0.3459786	0.452101675	-0.10612303	-0.47692521	5.43E-11	1.25E-08	7	0	0
OR5A1	0.5484208	0.764479013	-0.21605822	-0.47598715	5.56E-11	1.26E-08	2	0	0
OR10K2	0.606456	0.828435333	-0.2219793	-0.4485488	5.74E-11	1.27E-08	3	0	0
LYVE1	0.5702	0.756152647	-0.18595262	-0.40707953	5.70E-11	1.27E-08	3	0	0
RPH3A	0.4628064	0.640927013	-0.17812064	-0.46124629	6.21E-11	1.36E-08	1	0	0
S100A8	0.4313988	0.582953527	-0.15155473	-0.42351618	6.41E-11	1.37E-08	7	0	0
CD300E	0.4609069	0.624813208	-0.16390629	-0.4945245	6.37E-11	1.37E-08	7	0	0
DRD5	0.4754422	0.278694697	0.196747508	0.821725069	6.70E-11	1.40E-08	5	0	0
HEPACAM	0.6254818	0.775035093	-0.14955326	-0.31429806	6.65E-11	1.40E-08	7	0	0
HTR3D	0.4284219	0.56826989	-0.13984797	-0.4225385	7.04E-11	1.45E-08	3	0	0
DRD4	0.3965957	0.222070217	0.174525531	1.38045657	7.85E-11	1.60E-08	9	0	0
BFSP2	0.6864433	0.801749261	-0.11530596	-0.22565622	7.96E-11	1.60E-08	5	0	0
VSX1	0.3136723	0.166229352	0.147442964	1.071430997	8.21E-11	1.64E-08	14	0	0
TBX5	0.2931897	0.132514103	0.160675564	1.248131479	8.37E-11	1.65E-08	19	0	0
OR10J3	0.5262766	0.723127924	-0.19685129	-0.45108758	8.47E-11	1.65E-08	1	0	0
PCDHGA6	0.4157818	0.26663758	0.149144245	0.776374628	9.14E-11	1.76E-08	10	0	0
HYAL2	0.5653501	0.438712947	0.12663716	0.340712239	9.79E-11	1.85E-08	11	0	0
SIGLECL1	0.2790593	0.455912284	-0.176853	-0.68869297	9.81E-11	1.85E-08	1	0	0
SLC4A10	0.361888	0.227767816	0.134120201	0.849554176	1.00E-10	1.85E-08	8	0	0
RNASE6	0.6938078	0.786497858	-0.09269006	-0.23933581	9.93E-11	1.85E-08	4	0	0
P4HA2	0.33134	0.443308472	-0.11196847	-0.48149034	1.02E-10	1.86E-08	9	0	0
BAHD1	0.2261779	0.304661997	-0.07848412	-0.41589352	1.13E-10	2.05E-08	6	0	0
FPR1	0.3800507	0.545301558	-0.16525087	-0.53933311	1.19E-10	2.14E-08	2	0	0
LPAR5	0.4034263	0.505863094	-0.10243681	-0.36225757	1.26E-10	2.23E-08	13	0	0
INTS1	0.3554343	0.458271207	-0.1028369	-0.35088232	1.35E-10	2.34E-08	5	0	0
OR9I1	0.7121242	0.851600238	-0.13947602	-0.24986874	1.34E-10	2.34E-08	2	0	0
TAS2R16	0.643995	0.787920824	-0.14392584	-0.28864207	1.36E-10	2.35E-08	6	0	0
TMC6	0.4461009	0.558130547	-0.1120296	-0.4618142	1.38E-10	2.36E-08	13	0	0
MLIP	0.5816896	0.782021106	-0.20033151	-0.40262352	1.41E-10	2.38E-08	5	0	0
SLC34A1	0.7711931	0.767960593	0.003232476	0.003122672	1.43E-10	2.40E-08	2	0	0
LIMD2	0.342726	0.178064498	0.164661538	1.186449621	1.44E-10	2.40E-08	4	0	0
ECSCR	0.7709315	0.683343228	0.08758831	0.179041482	1.47E-10	2.42E-08	6	0	0
CNBD1	0.5895795	0.77118509	-0.18160558	-0.3752281	1.50E-10	2.44E-08	4	0	0
WDR72	0.6821444	0.861129299	-0.17898488	-0.33193867	1.58E-10	2.56E-08	2	0	0
LAIR1	0.3721186	0.528528785	-0.15641018	-0.50117026	1.68E-10	2.69E-08	7	0	0
ZNF578	0.3568341	0.243988823	0.11284526	0.857180345	1.74E-10	2.76E-08	10	0	0
OR14J1	0.6464889	0.804961583	-0.15847267	-0.32107736	1.78E-10	2.80E-08	7	0	0
S100A11	0.05051	0.090130817	-0.03962084	-0.51558135	1.83E-10	2.86E-08	5	0	0
SPATA18	0.2365981	0.308794985	-0.07219686	-0.44962955	1.92E-10	2.97E-08	12	0	0
CD68	0.2673334	0.343459671	-0.07612624	-0.49756194	2.04E-10	3.13E-08	7	0	0
ZNF835	0.3068283	0.137065425	0.169762914	1.322544154	2.15E-10	3.27E-08	8	0	0
HCAR3	0.3991642	0.599137462	-0.19997331	-0.57408808	2.21E-10	3.32E-08	1	0	0
EXD3	0.3570044	0.47107715	-0.11407276	-0.41049411	2.28E-10	3.41E-08	3	0	0
ETS2	0.1881371	0.281248848	-0.09311175	-0.61869149	2.37E-10	3.51E-08	7	0	0
KCNJ8	0.4676433	0.236389977	0.231253323	1.055028863	2.44E-10	3.58E-08	2	0	0
PUM2	0.3990036	0.515494368	-0.1164908	-0.46380766	2.48E-10	3.62E-08	5	0	0
ABCC9	0.2526462	0.108463505	0.144182712	1.351058792	2.54E-10	3.67E-08	15	0	0

OR10Z1	0.5526913	0.773041503	-0.22035016	-0.47911028	2.66E-10	3.78E-08	4	0	0
DENND1B	0.1687255	0.238457774	-0.06973231	-0.40282755	2.70E-10	3.78E-08	15	0	0
ZSCAN18	0.5333437	0.460374153	0.072969571	0.339738767	2.69E-10	3.78E-08	8	0	0
SMOX	0.2808301	0.411164093	-0.13033394	-0.53420561	2.68E-10	3.78E-08	1	0	0
CLIC3	0.2932636	0.397902023	-0.10463837	-0.52080054	2.86E-10	3.94E-08	11	0	0
KRTAP11-1	0.6937668	0.832054255	-0.13828748	-0.26703126	2.85E-10	3.94E-08	5	0	0
ZNF540	0.4936692	0.427892258	0.065776989	0.484598958	2.99E-10	4.09E-08	11	0	0
LYPD8	0.5924698	0.727878643	-0.13540884	-0.31998309	3.18E-10	4.25E-08	8	0	0
FOXI2	0.316937	0.156228459	0.160708509	1.193506531	3.14E-10	4.25E-08	12	0	0
ZBP1	0.4826946	0.583458247	-0.10076366	-0.40499312	3.17E-10	4.25E-08	7	0	0
GPR141	0.5333608	0.636359945	-0.10299916	-0.31744833	3.28E-10	4.33E-08	2	0	0
EPDR1	0.5333608	0.636359945	-0.10299916	-0.31744833	3.28E-10	4.33E-08	2	0	0
PARVB	0.8718186	0.812110924	0.059707655	0.105387465	3.43E-10	4.48E-08	8	0	0
OSGIN2	0.3127933	0.425136659	-0.1123434	-0.49566418	3.98E-10	5.18E-08	12	0	0
RAB17	0.7823576	0.874383652	-0.09202606	-0.15868215	4.10E-10	5.29E-08	2	0	0
CPB1	0.0714295	0.135542236	-0.06411271	-0.83781402	4.22E-10	5.40E-08	1	0	0
OR6P1	0.500816	0.67267901	-0.17186297	-0.46926257	4.33E-10	5.47E-08	6	0	0
ZSCAN1	0.3681315	0.16985409	0.19827737	1.141316741	4.36E-10	5.47E-08	7	0	0
PLXNB2	0.5933056	0.482407669	0.110897922	0.292440546	4.36E-10	5.47E-08	2	0	0
SOX17	0.3251378	0.176170009	0.148967823	1.266955458	4.55E-10	5.67E-08	10	0	0
LSP1	0.497966	0.637537923	-0.13957194	-0.39194419	4.66E-10	5.76E-08	5	0	0
C15orf62	0.3670762	0.512682187	-0.14560594	-0.50333243	4.73E-10	5.81E-08	11	0	0
SLC22A10	0.7173004	0.865374486	-0.1480741	-0.267349	4.84E-10	5.88E-08	1	0	0
OCSTAMP	0.5585982	0.68064748	-0.12204928	-0.30670665	4.85E-10	5.88E-08	8	0	0
HOXD3	0.3067247	0.101739822	0.204984886	1.578132984	4.97E-10	5.92E-08	7	0	0
CCDC140	0.3476861	0.183354518	0.164331564	0.972004044	4.96E-10	5.92E-08	9	0	0
ZNF132	0.3210924	0.192467447	0.128624903	1.413577222	4.98E-10	5.92E-08	9	0	0
HAS1	0.2459659	0.16354701	0.082418877	0.565902494	5.12E-10	6.00E-08	2	0	0
TNFSF10	0.2879687	0.355271381	-0.06730265	-0.66305418	5.47E-10	6.37E-08	4	0	0
GHSR	0.2911054	0.138688256	0.152417134	1.280208938	5.60E-10	6.48E-08	17	0	0
ARMC5	0.3270377	0.395577215	-0.06853952	-0.42591747	5.70E-10	6.56E-08	12	0	0
AFF3	0.4295786	0.595488568	-0.16590998	-0.4827898	5.74E-10	6.56E-08	4	0	0
OR5M3	0.6958371	0.868468609	-0.17263149	-0.31565544	5.81E-10	6.59E-08	1	0	0
OR3A2	0.6378912	0.79545618	-0.15756503	-0.32731694	5.90E-10	6.63E-08	5	0	0
OR7A5	0.6655312	0.648518008	0.017013168	0.003692382	5.91E-10	6.63E-08	3	0	0
ZNF529	0.2660654	0.080423033	0.185642383	1.783700183	6.15E-10	6.85E-08	13	0	0
HOXD8	0.2866308	0.111159097	0.175471751	1.418059519	6.34E-10	6.94E-08	16	0	0
GCSAM	0.4179731	0.545511808	-0.12753866	-0.63837011	6.29E-10	6.94E-08	5	0	0
ALG1L	0.5301503	0.808881795	-0.27873154	-0.60029443	6.35E-10	6.94E-08	1	0	0
SCRG1	0.7517042	0.840265655	-0.08856143	-0.15877361	6.39E-10	6.94E-08	2	0	0
KRTAP7-1	0.7007506	0.845018761	-0.14426821	-0.27352296	6.45E-10	6.97E-08	6	0	0
INSL4	0.7259856	0.896218591	-0.170233	-0.30018157	6.64E-10	7.13E-08	1	0	0
PAX7	0.2643863	0.101209747	0.163176558	1.29956372	6.81E-10	7.15E-08	6	0	0
ANGPTL1	0.6359666	0.446877541	0.189089095	0.508622301	6.84E-10	7.15E-08	4	0	0
ANGPT2	0.6583455	0.554797328	0.103548213	0.270511267	6.78E-10	7.15E-08	7	0	0
OR51B6	0.5156563	0.703414849	-0.18775854	-0.44062141	6.86E-10	7.15E-08	1	0	0
FUT2	0.5710653	0.686091498	-0.1150262	-0.29836478	6.76E-10	7.15E-08	7	0	0
MUSTN1	0.7200747	0.643871313	0.076203383	0.166459568	7.09E-10	7.31E-08	9	0	0
NOS3	0.7198689	0.794784022	-0.07491512	-0.15170825	7.06E-10	7.31E-08	4	0	0
UBQLNL	0.6220865	0.773697339	-0.15161082	-0.32270986	7.47E-10	7.65E-08	5	0	0
NREP	0.6688212	0.798141015	-0.1293198	-0.27873731	7.68E-10	7.83E-08	4	0	0
MPEG1	0.6128137	0.79059192	-0.17777823	-0.37567043	7.73E-10	7.83E-08	5	0	0
NKAPL	0.5121895	0.409975946	0.102213539	0.715623705	7.86E-10	7.88E-08	12	0	0
OR4C3	0.5069752	0.687022274	-0.18004706	-0.43110964	7.83E-10	7.88E-08	1	0	0
UNC5D	0.452659	0.369764651	0.082894387	0.571473303	8.02E-10	7.95E-08	12	0	0
A2ML1	0.6745467	0.866308104	-0.19176142	-0.36044884	8.01E-10	7.95E-08	4	0	0
ABLIM1	0.5517652	0.438997879	0.112767337	0.362967593	8.59E-10	8.47E-08	8	0	0
LILRA4	0.4585032	0.58101109	-0.1225079	-0.36986852	8.74E-10	8.56E-08	7	0	0
RASSF6	0.3293345	0.40104781	-0.07171331	-0.46748137	8.93E-10	8.71E-08	13	0	0
SH2B2	0.5235964	0.634922189	-0.11132574	-0.26009012	9.60E-10	9.30E-08	6	0	0
SPAM1	0.7237947	0.853797536	-0.13000286	-0.23994527	1.00E-09	9.66E-08	4	0	0
C2orf40	0.6472423	0.804294413	-0.15705215	-0.31588175	1.02E-09	9.80E-08	3	0	0
OR8B4	0.6538968	0.795537193	-0.1416404	-0.27937161	1.03E-09	9.80E-08	4	0	0

UBQLN3	0.6963405	0.829537823	-0.13319733	-0.25215938	1.11E-09	1.05E-07	3	0	0
PRRX1	0.2657157	0.128267962	0.137447697	1.169395925	1.15E-09	1.07E-07	7	0	0
SH3TC1	0.6125262	0.504697361	0.107828847	0.27441019	1.14E-09	1.07E-07	1	0	0
KLHL31	0.7021896	0.601140507	0.101049051	0.263703668	1.15E-09	1.07E-07	10	0	0
LAIR2	0.3350403	0.518928505	-0.18388819	-0.61824264	1.14E-09	1.07E-07	2	0	0
KRT81	0.7586284	0.699003877	0.059624562	0.138034032	1.17E-09	1.08E-07	5	0	0
EVX1	0.3424644	0.198980238	0.143484203	0.861568162	1.21E-09	1.11E-07	18	0	0
TAC1	0.3062867	0.121479975	0.184806693	1.241610375	1.22E-09	1.12E-07	8	0	0
PATE2	0.6271834	0.798145135	-0.17096177	-0.35350392	1.26E-09	1.15E-07	3	0	0
SERPINB13	0.6426441	0.811614182	-0.16897011	-0.33436723	1.28E-09	1.16E-07	8	0	0
FANCD2OS	0.8212719	0.724279214	0.096992714	0.188446448	1.37E-09	1.23E-07	11	0	0
SPINT4	0.687572	0.891984949	-0.20441292	-0.37076116	1.39E-09	1.24E-07	1	0	0
ABHD17C	0.4665326	0.433964108	0.032568485	0.032571333	1.40E-09	1.25E-07	3	0	0
SOX1	0.3007212	0.193345327	0.107375855	0.690250434	1.43E-09	1.27E-07	13	0	0
OR10J1	0.53291	0.738304359	-0.20539432	-0.45061755	1.45E-09	1.28E-07	5	0	0
IQCJ-SCHIP'	0.4720876	0.670750991	-0.1986634	-0.49677872	1.46E-09	1.28E-07	4	0	0
IQCJ	0.4720876	0.670750991	-0.1986634	-0.49677872	1.46E-09	1.28E-07	4	0	0
TRIM15	0.5311305	0.693010862	-0.16188032	-0.43253787	1.51E-09	1.31E-07	14	0	0
OR2B11	0.6043486	0.746717663	-0.14236901	-0.35107487	1.53E-09	1.33E-07	7	0	0
OR4D9	0.642671	0.793448094	-0.15077714	-0.30367461	1.55E-09	1.34E-07	3	0	0
AGXT2	0.6498012	0.806425692	-0.15662453	-0.31868923	1.58E-09	1.35E-07	8	0	0
GPR31	0.5951422	0.729223331	-0.13408112	-0.31829223	1.58E-09	1.35E-07	5	0	0
ATP10A	0.7240846	0.829882877	-0.10579829	-0.20145213	1.64E-09	1.39E-07	3	0	0
BPIFA1	0.5571989	0.734930982	-0.17773209	-0.3961317	1.65E-09	1.39E-07	6	0	0
UMOD	0.5846503	0.778897172	-0.19424688	-0.41165168	1.68E-09	1.41E-07	3	0	0
OR6Y1	0.748466	0.883823819	-0.13535777	-0.23690589	1.77E-09	1.47E-07	1	0	0
WDFY1	0.4613246	0.388987569	0.072337024	0.182368456	1.76E-09	1.47E-07	7	0	0
GALNT8	0.5327878	0.661637584	-0.12884982	-0.30255396	1.79E-09	1.49E-07	7	0	0
FCER1A	0.6258502	0.799031206	-0.17318105	-0.35130846	1.82E-09	1.51E-07	7	0	0
NETO1	0.2870452	0.141226099	0.145819075	0.997275121	1.87E-09	1.54E-07	5	0	0
GPR62	0.5458811	0.653767005	-0.10788595	-0.26096924	1.90E-09	1.55E-07	6	0	0
OR52E2	0.6040421	0.770336955	-0.16629485	-0.34540498	1.89E-09	1.55E-07	2	0	0
NPLOC4	0.6283916	0.502318253	0.126073389	0.366543433	1.92E-09	1.56E-07	4	0	0
OR5H6	0.6047005	0.775362076	-0.17066163	-0.36680703	1.97E-09	1.59E-07	2	0	0
OR2M5	0.7200608	0.883059109	-0.16299829	-0.29611837	2.01E-09	1.61E-07	5	0	0
SALL3	0.2303795	0.098332006	0.132047506	1.220672611	2.07E-09	1.65E-07	14	0	0
KRTAP20-2	0.6574093	0.815045603	-0.15763626	-0.31581589	2.07E-09	1.65E-07	5	0	0
TP53I13	0.1796379	0.273799364	-0.09416141	-0.55516096	2.10E-09	1.66E-07	3	0	0
SORCS3	0.1943703	0.063983872	0.130386448	1.525945999	2.13E-09	1.68E-07	10	0	0
ST7L	0.3438821	0.476459703	-0.13257765	-0.51469568	2.15E-09	1.68E-07	3	0	0
PATE1	0.5981384	0.745255074	-0.14711672	-0.32122053	2.15E-09	1.68E-07	6	0	0
RTN4	0.599214	0.439890473	0.159323481	0.434905136	2.17E-09	1.69E-07	2	0	0
GNG4	0.3691847	0.213823468	0.155361212	0.916615816	2.18E-09	1.69E-07	11	0	0
REG3A	0.5693085	0.739305591	-0.1699971	-0.37181635	2.20E-09	1.70E-07	5	0	0
OR9Q2	0.5787116	0.715854525	-0.1371429	-0.30632253	2.23E-09	1.71E-07	10	0	0
NRXN3	0.5703173	0.71047273	-0.14015542	-0.30981349	2.22E-09	1.71E-07	4	0	0
TMEM132D	0.3462108	0.210589465	0.135621331	0.919313498	2.26E-09	1.73E-07	6	0	0
PRDM14	0.2968004	0.137883028	0.158917349	1.194077568	2.30E-09	1.75E-07	8	0	0
TTYH1	0.2471148	0.112914608	0.13420017	1.822111157	2.35E-09	1.78E-07	10	0	0
SLC41A3	0.7054082	0.628827017	0.076581162	0.167535906	2.38E-09	1.79E-07	7	0	0
GRXCR1	0.5797326	0.750368337	-0.17063577	-0.38049247	2.40E-09	1.80E-07	6	0	0
TNK2	0.5078933	0.694375051	-0.1864817	-0.44707154	2.43E-09	1.81E-07	4	0	0
OR51B5	0.6173818	0.773337451	-0.15595567	-0.33317593	2.43E-09	1.81E-07	5	0	0
RP1	0.6512634	0.797360835	-0.14609741	-0.31039812	2.56E-09	1.90E-07	4	0	0
DBH	0.6844977	0.788980243	-0.10448252	-0.20763307	2.58E-09	1.90E-07	8	0	0
MSGN1	0.6305066	0.796922134	-0.16641551	-0.34548304	2.62E-09	1.91E-07	6	0	0
PRSS58	0.6728736	0.861258897	-0.18838532	-0.35148297	2.61E-09	1.91E-07	1	0	0
SCARF1	0.6480987	0.55128781	0.096810911	0.259095898	2.60E-09	1.91E-07	8	0	0
VAX1	0.2393623	0.111001802	0.128360536	1.227254547	2.72E-09	1.98E-07	11	0	0
KCNA2	0.3749439	0.489146156	-0.11420228	-0.41534619	2.75E-09	1.98E-07	5	0	0
PATE3	0.4186404	0.573615902	-0.15497548	-0.43542961	2.75E-09	1.98E-07	4	0	0
SIGLEC5	0.5294807	0.653306352	-0.12382561	-0.33497663	2.82E-09	2.01E-07	6	0	0
SIGLEC14	0.5294807	0.653306352	-0.12382561	-0.33497663	2.82E-09	2.01E-07	6	0	0

ANXA2R	0.4453376	0.368115774	0.077221815	0.084453696	2.93E-09	2.09E-07	7	0	0
RNF112	0.6323834	0.632997833	-0.00061443	0.062651213	2.94E-09	2.09E-07	8	0	0
OR6K2	0.722868	0.879534846	-0.15666681	-0.28487925	2.97E-09	2.09E-07	6	0	0
OR4D10	0.6700434	0.805410249	-0.13536681	-0.27751041	2.99E-09	2.10E-07	5	0	0
NELL2	0.5097708	0.691905117	-0.18213436	-0.43340054	3.08E-09	2.15E-07	1	0	0
VSTM2B	0.2283052	0.076016019	0.152289149	1.669837999	3.07E-09	2.15E-07	3	0	0
HMGB4	0.6256332	0.800544913	-0.17491168	-0.35694395	3.14E-09	2.18E-07	4	0	0
CMA1	0.7011519	0.837320969	-0.13616908	-0.25517861	3.17E-09	2.19E-07	3	0	0
ZNF716	0.6068878	0.770961359	-0.16407356	-0.34254419	3.20E-09	2.21E-07	3	0	0
CDHR5	0.5849236	0.449545181	0.13537837	0.634404936	3.22E-09	2.21E-07	11	0	0
OR5J2	0.5680082	0.745726408	-0.17771823	-0.38878405	3.21E-09	2.21E-07	2	0	0
TFAP2B	0.3292599	0.15465976	0.174600119	1.163142463	3.27E-09	2.22E-07	5	0	0
DENND2A	0.3920934	0.47208307	-0.07998965	-0.30577707	3.26E-09	2.22E-07	4	0	0
PIEZO2	0.2722973	0.143263147	0.129034181	0.993150323	3.27E-09	2.22E-07	12	0	0
SFN	0.4811188	0.611563297	-0.13044446	-0.34535658	3.31E-09	2.22E-07	10	0	0
HOXA11	0.3172315	0.194989877	0.122241579	0.727985897	3.30E-09	2.22E-07	30	0	0
MNDA	0.5323509	0.676948471	-0.14459754	-0.37403239	3.37E-09	2.25E-07	7	0	0
KRTAP8-1	0.6364319	0.800555012	-0.16412309	-0.3295992	3.49E-09	2.32E-07	5	0	0
APCS	0.4854798	0.682202986	-0.19672321	-0.48424017	3.53E-09	2.34E-07	3	0	0
MS4A4A	0.5528302	0.672185934	-0.11935573	-0.31944568	3.63E-09	2.40E-07	8	0	0
SLC5A4	0.6266487	0.756420375	-0.12977168	-0.27647577	3.65E-09	2.41E-07	6	0	0
OR10G9	0.6252832	0.800274143	-0.17499094	-0.352592	3.67E-09	2.41E-07	3	0	0
JRK	0.6144127	0.705816017	-0.09140333	-0.2049767	3.73E-09	2.44E-07	3	0	0
IL22RA2	0.7920238	0.909968708	-0.11794491	-0.19844023	3.81E-09	2.47E-07	3	0	0
CLDN15	0.7529048	0.670852666	0.082052173	0.168381929	3.80E-09	2.47E-07	10	0	0
C16orf78	0.6373387	0.781684115	-0.14434543	-0.30156034	3.82E-09	2.47E-07	7	0	0
BARHL2	0.316054	0.160697244	0.155356706	1.140742192	3.90E-09	2.51E-07	11	0	0
OR5B12	0.6847275	0.820881965	-0.13615446	-0.27026055	3.89E-09	2.51E-07	6	0	0
KLK2	0.5782705	0.719784609	-0.14151412	-0.31099107	3.95E-09	2.53E-07	1	0	0
CASP14	0.6430088	0.820689243	-0.17768041	-0.34700631	3.96E-09	2.53E-07	5	0	0
COL17A1	0.671141	0.804990529	-0.13384952	-0.28775703	4.09E-09	2.60E-07	5	0	0
ZNF709	0.5684051	0.477402927	0.091002177	0.379420109	4.10E-09	2.60E-07	9	0	0
EFCAB3	0.7089302	0.889817354	-0.18088714	-0.32378045	4.21E-09	2.66E-07	1	0	0
GREB1	0.5743614	0.725086972	-0.15072558	-0.3746389	4.34E-09	2.72E-07	7	0	0
SIRPB1	0.4496106	0.582099256	-0.13248865	-0.39966101	4.32E-09	2.72E-07	3	0	0
SLAMF6	0.3110568	0.43646888	-0.12541209	-0.53472049	4.37E-09	2.73E-07	3	0	0
ADM5	0.6806442	0.59176376	0.088880465	0.189637755	4.38E-09	2.73E-07	9	0	0
OR6N1	0.6591235	0.82008512	-0.16096159	-0.3198388	4.41E-09	2.73E-07	6	0	0
OR4D1	0.697024	0.840964527	-0.14394053	-0.27116434	4.45E-09	2.75E-07	8	0	0
ZIC1	0.3226808	0.167136042	0.15554472	0.923999276	4.48E-09	2.76E-07	12	0	0
SLC28A3	0.6742901	0.844171219	-0.16988116	-0.32818921	4.52E-09	2.77E-07	4	0	0
HBE1	0.7014276	0.835873543	-0.13444596	-0.25453416	4.60E-09	2.81E-07	4	0	0
CD1D	0.5566086	0.676945289	-0.12033671	-0.28548309	4.67E-09	2.84E-07	8	0	0
CD96	0.7502046	0.863937933	-0.1137333	-0.20002409	4.74E-09	2.87E-07	2	0	0
CLDN18	0.7133266	0.858875844	-0.1455492	-0.26905955	4.76E-09	2.87E-07	11	0	0
TMEM132C	0.2480924	0.107541962	0.140550396	1.289802962	4.76E-09	2.87E-07	5	0	0
VPS53	0.7155312	0.902940711	-0.18740954	-0.33148358	4.93E-09	2.96E-07	1	0	0
PARP8	0.8359725	0.74700308	0.088969373	0.171340889	5.09E-09	3.04E-07	2	0	0
OR4D11	0.6527562	0.839004221	-0.186248	-0.35914091	5.14E-09	3.06E-07	2	0	0
GLIPR1	0.4102407	0.505160574	-0.09491986	-0.56402532	5.19E-09	3.08E-07	8	0	0
MYL10	0.4495436	0.575225943	-0.12568234	-0.44250209	5.37E-09	3.18E-07	3	0	0
NFE2L3	0.2782277	0.346402162	-0.06817448	-0.37011394	5.44E-09	3.21E-07	5	0	0
FBP2	0.6504356	0.681800779	-0.03136514	-0.10437275	5.46E-09	3.21E-07	3	0	0
CTLA4	0.5568851	0.741798206	-0.18491313	-0.43987265	5.53E-09	3.24E-07	3	0	0
DDHD1	0.1226267	0.1753152	-0.05268845	-0.38811139	5.55E-09	3.24E-07	7	0	0
RNF148	0.6919763	0.780550778	-0.08857444	-0.18447471	5.68E-09	3.31E-07	4	0	0
RNF111	0.5803706	0.431761967	0.148608655	0.477673065	5.71E-09	3.31E-07	6	0	0
SEC23A	0.6958786	0.857888559	-0.16200996	-0.298425	5.79E-09	3.35E-07	2	0	0
FCRL1	0.4980582	0.657864917	-0.15980669	-0.46825175	5.85E-09	3.37E-07	7	0	0
CD300LF	0.4265567	0.538718795	-0.11216211	-0.36235227	5.97E-09	3.43E-07	5	0	0
NXPE2	0.5951282	0.740999681	-0.14587144	-0.34657569	6.00E-09	3.44E-07	7	0	0
LRRN3	0.706414	0.594884878	0.111529107	0.245699084	6.38E-09	3.64E-07	4	0	0
SIRPG	0.5799533	0.747822739	-0.16786947	-0.39376029	6.39E-09	3.64E-07	6	0	0

REEP1	0.3113437	0.375892376	-0.06454866	-0.31191309	6.63E-09	3.77E-07	10	0	0
GAPVD1	0.0818029	0.134067708	-0.05226481	-0.4151654	6.68E-09	3.78E-07	5	0	0
OR5M8	0.6521497	0.79916886	-0.1470192	-0.30126155	6.75E-09	3.81E-07	4	0	0
FSIP2	0.4298896	0.340947049	0.08894255	0.50139415	6.85E-09	3.85E-07	10	0	0
KRTAP19-8	0.7232318	0.856634002	-0.1334022	-0.25284783	6.88E-09	3.85E-07	4	0	0
OR52A5	0.6726287	0.79135224	-0.1187235	-0.24611689	6.97E-09	3.88E-07	3	0	0
ALDH1A3	0.6191101	0.767866439	-0.14875631	-0.30620988	6.97E-09	3.88E-07	1	0	0
DQX1	0.7290261	0.858959239	-0.12993317	-0.25570715	7.13E-09	3.96E-07	10	0	0
NXPE4	0.6735453	0.803950967	-0.13040564	-0.27931955	7.19E-09	3.98E-07	5	0	0
MEI1	0.4489377	0.313418456	0.135519214	1.000076019	7.23E-09	3.99E-07	9	0	0
IP6K2	0.5034716	0.363263302	0.140208321	0.487083247	7.27E-09	4.00E-07	2	0	0
OR5I1	0.6208188	0.792990303	-0.17217152	-0.35376008	7.36E-09	4.04E-07	5	0	0
FUT7	0.4247801	0.5233899	-0.09860984	-0.29571158	7.43E-09	4.06E-07	3	0	0
IL6R	0.212639	0.285713543	-0.07307452	-0.45485073	7.53E-09	4.10E-07	10	0	0
OR8B12	0.5716979	0.737862822	-0.16616493	-0.36148943	7.56E-09	4.11E-07	4	0	0
CABS1	0.760504	0.854839072	-0.09433511	-0.16749711	7.66E-09	4.15E-07	3	0	0
PAX9	0.3174784	0.155581787	0.161896649	1.131781647	7.68E-09	4.15E-07	10	0	0
LAMA4	0.629401	0.515450097	0.113950913	0.310316084	7.72E-09	4.16E-07	8	0	0
KRTAP20-3	0.7212258	0.842738779	-0.12151295	-0.22268231	7.91E-09	4.25E-07	5	0	0
MROH2B	0.6867401	0.842422937	-0.15568283	-0.30039559	7.99E-09	4.27E-07	6	0	0
HOXA2	0.3639349	0.222814845	0.14112007	0.866779605	8.00E-09	4.27E-07	20	0	0
ARL11	0.3489469	0.417545615	-0.06859869	-0.45092661	8.09E-09	4.30E-07	7	0	0
REG3G	0.6672017	0.815191033	-0.14798935	-0.28064057	8.28E-09	4.39E-07	6	0	0
ANKRD22	0.4302076	0.543616669	-0.11340908	-0.39021454	8.30E-09	4.39E-07	10	0	0
ACTR3C	0.2274036	0.294103205	-0.06669961	-0.55871021	8.41E-09	4.44E-07	8	0	0
GMNC	0.6202519	0.780385435	-0.16013357	-0.36911171	8.53E-09	4.49E-07	4	0	0
TBX15	0.4246891	0.281885728	0.142803418	0.639801086	8.58E-09	4.50E-07	12	0	0
OR10J5	0.6038124	0.756449526	-0.1526371	-0.33350747	8.95E-09	4.68E-07	4	0	0
TMEM88	0.781328	0.691457496	0.089870472	0.210120713	9.00E-09	4.69E-07	19	0	0
GP2	0.6419661	0.786027255	-0.14406112	-0.29606166	9.12E-09	4.74E-07	9	0	0
OR8B8	0.6666912	0.80111997	-0.13442875	-0.25857524	9.19E-09	4.76E-07	8	0	0
OR1N1	0.6328149	0.760170129	-0.12735522	-0.27986491	9.43E-09	4.86E-07	5	0	0
OR51M1	0.565163	0.713569002	-0.14840597	-0.32681209	9.42E-09	4.86E-07	2	0	0
NME8	0.7743201	0.880663403	-0.10634326	-0.1916415	9.60E-09	4.93E-07	5	0	0
SECISBP2	0.6283185	0.766038087	-0.13771963	-0.2818511	9.78E-09	5.01E-07	1	0	0
OR52E6	0.6356698	0.77093078	-0.13526096	-0.27627319	1.00E-08	5.11E-07	3	0	0
OR13G1	0.558212	0.747533983	-0.18932201	-0.42266095	1.01E-08	5.17E-07	3	0	0
CACNG7	0.1971669	0.055599011	0.141567922	1.67110081	1.02E-08	5.18E-07	5	0	0
KRT72	0.5571427	0.476143661	0.080999067	0.307841262	1.04E-08	5.27E-07	7	0	0
FRMD6	0.631067	0.802619076	-0.1715521	-0.34145541	1.04E-08	5.27E-07	6	0	0
CDX2	0.3936557	0.257511405	0.136144264	0.68047325	1.05E-08	5.30E-07	8	0	0
OR6N2	0.6534778	0.814752193	-0.16127438	-0.32189539	1.06E-08	5.33E-07	4	0	0
TAS2R60	0.7634013	0.891339596	-0.12793831	-0.225467	1.06E-08	5.33E-07	4	0	0
OR10T2	0.5463472	0.751180954	-0.2048338	-0.45228677	1.08E-08	5.38E-07	3	0	0
SGCA	0.4368508	0.520025958	-0.08317515	-0.38082359	1.08E-08	5.38E-07	5	0	0
CCDC168	0.7411281	0.897445034	-0.15631696	-0.27275133	1.09E-08	5.42E-07	1	0	0
FUT9	0.2643398	0.131584673	0.132755084	1.001804036	1.10E-08	5.43E-07	9	0	0
ZNF839	0.0779538	0.104618809	-0.02666499	-0.33466886	1.11E-08	5.47E-07	5	0	0
OR1L1	0.6173558	0.755443395	-0.1380876	-0.32257199	1.14E-08	5.61E-07	3	0	0
PCYOX1	0.3316853	0.446581896	-0.11489657	-0.45875641	1.19E-08	5.81E-07	4	0	0
LYPD1	0.4097266	0.52803919	-0.11831264	-0.55052834	1.19E-08	5.81E-07	8	0	0
IRX4	0.2744543	0.137606192	0.136848155	1.14790581	1.18E-08	5.81E-07	16	0	0
SLC23A1	0.6798873	0.811379387	-0.13149207	-0.25416679	1.19E-08	5.81E-07	2	0	0
OR10AG1	0.5544963	0.718399177	-0.16390289	-0.36855758	1.22E-08	5.94E-07	4	0	0
SULT1C4	0.4593176	0.333685115	0.125632479	0.612955231	1.23E-08	5.95E-07	9	0	0
LY6K	0.4266138	0.554605286	-0.12799149	-0.44379644	1.25E-08	6.03E-07	13	0	0
OR2G2	0.7411203	0.886010617	-0.14489028	-0.26143863	1.25E-08	6.03E-07	3	0	0
DNASE1L3	0.4999924	0.601671336	-0.10167895	-0.35247389	1.26E-08	6.07E-07	5	0	0
C9orf84	0.6837622	0.871154964	-0.18739275	-0.34652137	1.27E-08	6.09E-07	7	0	0
SLC12A7	0.2473332	0.331478876	-0.08414567	-0.20585144	1.29E-08	6.14E-07	11	0	0
TEX26	0.6123477	0.501990526	0.110357218	0.318874524	1.29E-08	6.14E-07	11	0	0
MYL1	0.5096113	0.657133044	-0.14752172	-0.38761161	1.32E-08	6.24E-07	4	0	0
ALOX5AP	0.5499109	0.667491415	-0.1175805	-0.29088417	1.34E-08	6.34E-07	7	0	0

OLFML1	0.6579442	0.56098592	0.096958278	0.247457272	1.38E-08	6.47E-07	6	0	0
LILRA1	0.6630489	0.814368164	-0.15131923	-0.29593042	1.37E-08	6.47E-07	2	0	0
OR9A2	0.6727583	0.81584692	-0.1430886	-0.27620675	1.39E-08	6.51E-07	5	0	0
EGFL8	0.8340258	0.776154343	0.057871501	0.110374497	1.40E-08	6.56E-07	4	0	0
KIF4B	0.516961	0.648739775	-0.13177877	-0.31611296	1.41E-08	6.57E-07	5	0	0
LCE1F	0.4328212	0.544168826	-0.11134764	-0.30687469	1.43E-08	6.68E-07	3	0	0
LGSN	0.7173601	0.870740661	-0.15338059	-0.28306103	1.45E-08	6.74E-07	4	0	0
POM121L12	0.717437	0.802957416	-0.08552039	-0.16034816	1.48E-08	6.84E-07	5	0	0
OR6F1	0.6923985	0.85284084	-0.16044231	-0.30470536	1.50E-08	6.95E-07	4	0	0
C1orf189	0.8079099	0.733232678	0.074677186	0.14194549	1.52E-08	7.03E-07	4	0	0
OR4D6	0.6272036	0.778020319	-0.15081669	-0.32490684	1.56E-08	7.16E-07	4	0	0
PTGFR	0.5882393	0.722716886	-0.13447756	-0.35746519	1.57E-08	7.19E-07	3	0	0
OR11L1	0.7053282	0.818725739	-0.11339755	-0.21802545	1.57E-08	7.19E-07	7	0	0
CHRNA1	0.7387098	0.844039708	-0.10532996	-0.19711486	1.58E-08	7.19E-07	7	0	0
SLC47A2	0.673863	0.803913196	-0.13005015	-0.25159623	1.63E-08	7.43E-07	4	0	0
ELAVL4	0.4219349	0.317941576	0.103993354	0.835279804	1.66E-08	7.55E-07	11	0	0
GATA2	0.3399755	0.20732859	0.13264691	1.055218276	1.67E-08	7.55E-07	8	0	0
MT1A	0.4836453	0.450747956	0.032897311	0.531997619	1.68E-08	7.57E-07	8	0	0
KRT36	0.3783947	0.330242941	0.048151778	0.426208834	1.68E-08	7.57E-07	4	0	0
EFNA4	0.1034252	0.150638199	-0.04721301	-0.37793472	1.71E-08	7.65E-07	16	0	0
EFNA3	0.1034252	0.150638199	-0.04721301	-0.37793472	1.71E-08	7.65E-07	16	0	0
TREML2	0.4559482	0.575057402	-0.11910918	-0.32552452	1.72E-08	7.68E-07	6	0	0
APLNR	0.5047573	0.583888197	-0.07913094	-0.17730365	1.72E-08	7.68E-07	4	0	0
WFDC6	0.5188831	0.674783567	-0.15590042	-0.34852059	1.77E-08	7.86E-07	7	0	0
OR5AC2	0.6673495	0.81569059	-0.1483411	-0.28524011	1.79E-08	7.91E-07	3	0	0
OR2M4	0.709619	0.862190162	-0.15257119	-0.28292314	1.82E-08	8.03E-07	3	0	0
TMEM184A	0.6634018	0.640254453	0.023147352	0.085050054	1.86E-08	8.19E-07	2	0	0
RTKN	0.4458209	0.56334768	-0.11752677	-0.38994443	1.88E-08	8.21E-07	8	0	0
SORL1	0.1961598	0.253160444	-0.05700069	-0.24587469	1.87E-08	8.21E-07	7	0	0
KRTAP4-6	0.4769602	0.641346836	-0.1643866	-0.419622	1.88E-08	8.21E-07	1	0	0
FAM228A	0.734163	0.691145084	0.043017894	0.231428086	1.89E-08	8.22E-07	7	0	0
ZNF677	0.2225837	0.10138638	0.12119731	1.169406931	1.89E-08	8.22E-07	8	0	0
DNHD1	0.7552463	0.672588635	0.082657698	0.182171867	1.93E-08	8.37E-07	10	0	0
KRTAP13-2	0.6259628	0.767760652	-0.14179781	-0.29151513	1.93E-08	8.38E-07	3	0	0
TET2	0.238735	0.32874461	-0.09000963	-0.41247732	1.95E-08	8.42E-07	7	0	0
FSHB	0.5028804	0.647048282	-0.14416786	-0.35545939	1.96E-08	8.44E-07	5	0	0
CCDC141	0.4717939	0.413332784	0.058461158	0.647647032	1.98E-08	8.50E-07	7	0	0
SP140	0.6309942	0.765107806	-0.1341136	-0.28948597	1.98E-08	8.50E-07	4	0	0
OR51T1	0.5760292	0.705257753	-0.12922858	-0.29007137	1.98E-08	8.50E-07	6	0	0
MDGA2	0.2557335	0.133170901	0.12256261	0.933329084	2.00E-08	8.53E-07	10	0	0
MTUS2	0.6510131	0.793195856	-0.14218278	-0.28697834	2.01E-08	8.56E-07	7	0	0
KRTAP6-3	0.7171054	0.806158337	-0.08905294	-0.17301718	2.03E-08	8.62E-07	8	0	0
OR2M7	0.6220685	0.843227817	-0.22115929	-0.43285002	2.12E-08	8.92E-07	1	0	0
PCDHGA5	0.390439	0.271736131	0.118702848	0.584891661	2.12E-08	8.92E-07	12	0	0
FSCB	0.6427857	0.78069398	-0.13790823	-0.27591432	2.11E-08	8.92E-07	4	0	0
KRTAP21-1	0.6529026	0.803894001	-0.15099137	-0.3077599	2.11E-08	8.92E-07	4	0	0
PAH	0.2143317	0.079904495	0.134427159	1.51441051	2.13E-08	8.94E-07	10	0	0
SIGLEC12	0.4460938	0.569570216	-0.12347642	-0.36843777	2.14E-08	8.96E-07	11	0	0
ELMO2	0.5835544	0.74565542	-0.16210106	-0.35049829	2.16E-08	9.02E-07	3	0	0
MAMSTR	0.5354152	0.606405283	-0.07099011	-0.30772768	2.19E-08	9.12E-07	9	0	0
SPARC	0.4476785	0.403873527	0.043805002	0.627121623	2.26E-08	9.41E-07	9	0	0
NMRAL1	0.7364112	0.853367061	-0.1169559	-0.21173617	2.27E-08	9.41E-07	3	0	0
SIX6	0.2457728	0.116080576	0.129692207	1.122399295	2.29E-08	9.51E-07	11	0	0
ZFP30	0.2586928	0.138058544	0.120634209	1.536037111	2.32E-08	9.56E-07	7	0	0
ZNF781	0.2586928	0.138058544	0.120634209	1.536037111	2.32E-08	9.56E-07	7	0	0
MYL4	0.5262281	0.712253924	-0.18602583	-0.42965932	2.33E-08	9.57E-07	1	0	0
REG1A	0.7109	0.886142334	-0.17524233	-0.31392875	2.36E-08	9.69E-07	1	0	0
CPA1	0.6771184	0.593132161	0.08398621	0.212878863	2.37E-08	9.72E-07	6	0	0
KCNU1	0.7267566	0.833890634	-0.10713407	-0.20701957	2.39E-08	9.73E-07	7	0	0
LZTS2	0.1961081	0.259784701	-0.06367659	-0.31408588	2.38E-08	9.73E-07	9	0	0
SLC16A10	0.2062945	0.209879497	-0.00358505	-0.23596057	2.41E-08	9.80E-07	9	0	0
COLEC11	0.7063301	0.63793771	0.06839243	0.205811817	2.45E-08	9.90E-07	15	0	0
TMEM212	0.793146	0.719106916	0.074039066	0.172865596	2.45E-08	9.90E-07	8	0	0

STAT6	0.5632191	0.481441452	0.081777657	0.247038825	2.45E-08	9.90E-07	2	0	0
UGT1A6	0.5596082	0.694203108	-0.13459494	-0.31868578	2.58E-08	1.04E-06	5	0	0
OR5B21	0.7618742	0.869113281	-0.10723908	-0.19594029	2.62E-08	1.05E-06	3	0	0
CYP11B2	0.6121146	0.758533912	-0.14641934	-0.31154787	2.63E-08	1.06E-06	6	0	0
BPIFB4	0.6204166	0.771334814	-0.15091818	-0.3096353	2.64E-08	1.06E-06	1	0	0
MAMDC2	0.5432676	0.436709722	0.106557905	0.423048727	2.69E-08	1.08E-06	7	0	0
TSPAN32	0.4017853	0.506351077	-0.1045658	-0.34873925	2.70E-08	1.08E-06	27	0	0
COX7A2L	0.771411	0.739225098	0.032185939	0.069807144	2.72E-08	1.08E-06	5	0	0
TMEM216	0.650552	0.783099397	-0.13254744	-0.35615531	2.75E-08	1.09E-06	9	0	0
APOBEC1	0.5954187	0.756866848	-0.16144815	-0.32985957	2.75E-08	1.09E-06	5	0	0
CGB8	0.6528854	0.820018735	-0.16713331	-0.32438436	2.74E-08	1.09E-06	1	0	0
CST1	0.815259	0.906745404	-0.09148642	-0.15167418	2.74E-08	1.09E-06	1	0	0
CCDC70	0.6981664	0.797971453	-0.09980504	-0.20180119	2.79E-08	1.10E-06	6	0	0
LEFTY2	0.5750843	0.483112315	0.091971982	0.247062664	2.80E-08	1.10E-06	3	0	0
CARD18	0.6928112	0.835501483	-0.14269031	-0.28360196	2.81E-08	1.10E-06	6	0	0
HOXD13	0.2673063	0.126582202	0.140724062	1.141854714	2.83E-08	1.11E-06	11	0	0
PI3	0.5059885	0.645728741	-0.13974026	-0.34043254	2.84E-08	1.11E-06	5	0	0
DEFB119	0.4055597	0.540890701	-0.13533104	-0.39344617	2.85E-08	1.11E-06	3	0	0
LEP	0.5499033	0.45014597	0.099757307	0.364634766	2.87E-08	1.11E-06	10	0	0
SRBD1	0.6303164	0.799264946	-0.16894851	-0.33782705	2.87E-08	1.11E-06	1	0	0
SARDH	0.6912752	0.804991106	-0.11371595	-0.21833672	2.91E-08	1.12E-06	5	0	0
SLC35E2B	0.509986	0.650422919	-0.14043692	-0.34436806	3.01E-08	1.16E-06	2	0	0
OR4A16	0.4936131	0.638563216	-0.14495008	-0.33205934	3.00E-08	1.16E-06	4	0	0
OR51A7	0.6452773	0.786754272	-0.14147693	-0.29126695	3.02E-08	1.16E-06	5	0	0
NRIP2	0.4634462	0.294398743	0.169047492	0.875540541	3.04E-08	1.16E-06	8	0	0
DNAJB8	0.6209287	0.731302864	-0.11037418	-0.25263485	3.05E-08	1.17E-06	7	0	0
NPTX2	0.4204639	0.317105248	0.103358651	0.775663244	3.06E-08	1.17E-06	5	0	0
FCN3	0.7543471	0.697198079	0.05714901	0.115618651	3.18E-08	1.21E-06	7	0	0
FETUB	0.6780719	0.856128125	-0.17805624	-0.33617212	3.24E-08	1.22E-06	2	0	0
TRIQK	0.7072447	0.832034983	-0.12479024	-0.23428091	3.23E-08	1.22E-06	5	0	0
ALX4	0.2485577	0.15181076	0.096746901	0.754842355	3.24E-08	1.22E-06	38	0	0
LILRB2	0.5678657	0.705671869	-0.13780616	-0.31186359	3.22E-08	1.22E-06	4	0	0
OR2AP1	0.7027848	0.842999987	-0.14021518	-0.26334291	3.29E-08	1.24E-06	2	0	0
KRTAP12-2	0.6155209	0.727801591	-0.11228065	-0.24090658	3.28E-08	1.24E-06	3	0	0
PAX6	0.286518	0.171752676	0.11476533	0.914452679	3.30E-08	1.24E-06	11	0	0
FAM92B	0.6842445	0.769122199	-0.08487766	-0.17625057	3.32E-08	1.24E-06	8	0	0
ZNF560	0.3245195	0.206250703	0.118268832	0.763128609	3.32E-08	1.24E-06	14	0	0
CCNB1	0.1908022	0.22747237	-0.03667016	-0.4539202	3.38E-08	1.26E-06	7	0	0
TARM1	0.5908673	0.713206116	-0.12233881	-0.29035295	3.38E-08	1.26E-06	5	0	0
CXCL5	0.6264812	0.578034337	0.048446858	0.394339062	3.39E-08	1.26E-06	5	0	0
OR56A3	0.5100901	0.63991707	-0.12982693	-0.31765544	3.42E-08	1.26E-06	3	0	0
CD300LD	0.5429885	0.661341713	-0.11835326	-0.31226611	3.44E-08	1.27E-06	6	0	0
KRTAP22-1	0.6828704	0.803177158	-0.12030674	-0.23767963	3.49E-08	1.29E-06	4	0	0
BGLAP	0.5955601	0.684116891	-0.08855677	-0.2364242	3.53E-08	1.30E-06	6	0	0
NPAP1	0.7003621	0.823992615	-0.12363049	-0.2439433	3.56E-08	1.31E-06	8	0	0
TACC1	0.4722652	0.479260477	-0.00699524	0.569569592	3.58E-08	1.31E-06	9	0	0
DEFB118	0.477524	0.633933433	-0.15640944	-0.40495991	3.59E-08	1.31E-06	6	0	0
IZUMO3	0.6632977	0.793456337	-0.13015864	-0.23142471	3.61E-08	1.31E-06	4	0	0
KRTAP6-2	0.7173576	0.831256007	-0.1138984	-0.22074979	3.61E-08	1.31E-06	7	0	0
TMC3	0.7128542	0.859713261	-0.14685903	-0.26772852	3.62E-08	1.31E-06	3	0	0
PKP3	0.6759969	0.79121083	-0.11521396	-0.23104888	3.66E-08	1.33E-06	5	0	0
FCAR	0.6010213	0.748993094	-0.14797183	-0.31435943	3.71E-08	1.34E-06	3	0	0
PLEKHB1	0.6017812	0.50636053	0.095420637	0.281755013	3.77E-08	1.36E-06	9	0	0
BPIFC	0.6450286	0.801756722	-0.15672809	-0.32021084	3.80E-08	1.37E-06	3	0	0
TBX20	0.2851166	0.150352921	0.134763725	1.005823482	3.83E-08	1.37E-06	12	0	0
IL18BP	0.6991189	0.656177103	0.042941797	0.11099183	3.87E-08	1.39E-06	10	0	0
TSHZ3	0.1405513	0.080881097	0.059670226	0.81892964	3.88E-08	1.39E-06	7	0	0
FERD3L	0.2111436	0.103643215	0.107500428	0.949319477	3.91E-08	1.40E-06	8	0	0
KANSL3	0.4537471	0.330549455	0.12319767	0.45668255	4.00E-08	1.43E-06	3	0	0
SLC2A11	0.2636935	0.386682303	-0.12298883	-0.52877327	4.01E-08	1.43E-06	4	0	0
TF	0.4731157	0.383075878	0.090039851	0.551664389	4.06E-08	1.44E-06	12	0	0
KRTAP21-2	0.5763217	0.733368398	-0.1570467	-0.340648	4.08E-08	1.45E-06	3	0	0
ROPN1	0.7416737	0.898148682	-0.15647503	-0.27282208	4.09E-08	1.45E-06	1	0	0

C18orf63	0.7445603	0.823885318	-0.07932498	-0.14682591	4.12E-08	1.45E-06	3	0	0
NDUFAF3	0.1721074	0.2414966	-0.06938915	-0.40034337	4.22E-08	1.48E-06	14	0	0
STIM1	0.1998226	0.276250936	-0.07642837	-0.3891209	4.21E-08	1.48E-06	6	0	0
CNTD2	0.2765589	0.360834463	-0.08427554	-0.40210175	4.28E-08	1.50E-06	13	0	0
ADCY4	0.4020875	0.281499636	0.120587895	1.097713314	4.33E-08	1.52E-06	10	0	0
CA1	0.6059431	0.722695925	-0.11675279	-0.2505046	4.36E-08	1.52E-06	4	0	0
CD1C	0.510271	0.677593727	-0.16732268	-0.40850532	4.44E-08	1.54E-06	5	0	0
HORMAD2	0.5384163	0.409886217	0.128530062	0.438145245	4.57E-08	1.59E-06	13	0	0
EVI2B	0.3976618	0.562514599	-0.16485283	-0.52933348	4.63E-08	1.61E-06	4	0	0
MACC1	0.2135483	0.311951229	-0.0984029	-0.55345824	4.68E-08	1.62E-06	6	0	0
TRAF1	0.2334095	0.290026005	-0.05661652	-0.31421187	4.70E-08	1.62E-06	7	0	0
OR8J1	0.5932482	0.744417826	-0.15116965	-0.32260931	4.69E-08	1.62E-06	2	0	0
ZNF135	0.4329809	0.306154356	0.126826583	0.807191828	4.69E-08	1.62E-06	13	0	0
MCFD2	0.0608727	0.095827176	-0.03495452	-0.50345729	4.74E-08	1.63E-06	10	0	0
OR56A1	0.6322987	0.787468049	-0.15516938	-0.32440642	4.75E-08	1.63E-06	2	0	0
DKK4	0.4869631	0.601628833	-0.11466575	-0.35904941	4.77E-08	1.63E-06	10	0	0
THRA	0.7669837	0.701362207	0.065621504	0.155748369	4.78E-08	1.63E-06	2	0	0
MTRNR2L1	0.5464678	0.673245936	-0.12677818	-0.29610687	4.80E-08	1.64E-06	1	0	0
KRTAP12-1	0.7566269	0.857846564	-0.1012197	-0.17859784	4.81E-08	1.64E-06	3	0	0
ADAMTS20	0.420904	0.310944649	0.109959366	0.860898367	4.86E-08	1.65E-06	7	0	0
HOXA10	0.4280662	0.335059104	0.093007065	0.436244863	4.96E-08	1.68E-06	8	0	0
B3GALT1	0.7141803	0.83515671	-0.12097638	-0.23703766	5.01E-08	1.69E-06	3	0	0
PRB4	0.7422212	0.868166076	-0.12594486	-0.23165918	5.07E-08	1.71E-06	4	0	0
FGD5	0.6929454	0.813999856	-0.12105449	-0.22939757	5.12E-08	1.72E-06	4	0	0
FOXB2	0.1764743	0.083833672	0.092640579	1.071787648	5.13E-08	1.72E-06	6	0	0
KRTAP10-8	0.6788044	0.788249832	-0.10944545	-0.20985763	5.13E-08	1.72E-06	4	0	0
PAMR1	0.3455078	0.477442192	-0.13193441	-0.51222809	5.19E-08	1.74E-06	2	0	0
GLRX5	0.3280466	0.388814427	-0.0607678	-0.41231314	5.23E-08	1.75E-06	3	0	0
OR14C36	0.6006329	0.764398775	-0.16376586	-0.35192698	5.29E-08	1.75E-06	3	0	0
BHMT2	0.5975999	0.503625473	0.093974384	0.370905682	5.26E-08	1.75E-06	10	0	0
TCF21	0.3043815	0.203534131	0.100847385	0.635692279	5.29E-08	1.75E-06	11	0	0
IL2RA	0.458336	0.578423053	-0.12008702	-0.37784676	5.29E-08	1.75E-06	7	0	0
MS4A1	0.5816438	0.721030565	-0.1393868	-0.31178549	5.30E-08	1.75E-06	3	0	0
BPIFB6	0.6135622	0.754030347	-0.14046813	-0.29577908	5.28E-08	1.75E-06	6	0	0
GPHB5	0.6864729	0.788089045	-0.10161614	-0.20928207	5.33E-08	1.76E-06	5	0	0
PEAR1	0.2537649	0.13638921	0.117375698	1.064655715	5.36E-08	1.77E-06	9	0	0
SMPD4	0.1222255	0.177142945	-0.05491747	-0.37684738	5.42E-08	1.78E-06	4	0	0
IL17A	0.6740197	0.824281692	-0.15026198	-0.29775018	5.41E-08	1.78E-06	5	0	0
ME3	0.4136041	0.316948239	0.09665589	1.102564924	5.45E-08	1.79E-06	19	0	0
ADORA3	0.8318731	0.901434404	-0.06956135	-0.11716024	5.57E-08	1.82E-06	3	0	0
KRTAP4-11	0.6434491	0.724596237	-0.08114715	-0.1721233	5.62E-08	1.84E-06	2	0	0
CLVS1	0.7369525	0.672602601	0.064349915	0.199583384	5.63E-08	1.84E-06	2	0	0
BPIFA2	0.6590512	0.853794278	-0.19474304	-0.36857106	5.64E-08	1.84E-06	1	0	0
TRH	0.5629053	0.460892952	0.102012305	0.879010512	5.76E-08	1.86E-06	13	0	0
NLRP5	0.6247312	0.742465747	-0.11773459	-0.24882762	5.75E-08	1.86E-06	2	0	0
CBLN4	0.3386094	0.276961138	0.061648304	0.588302289	5.74E-08	1.86E-06	11	0	0
COX7B2	0.7642872	0.887906245	-0.12361903	-0.22125732	5.77E-08	1.87E-06	8	0	0
ZNF671	0.3712543	0.245663298	0.125590957	1.525481443	5.79E-08	1.87E-06	13	0	0
OR5M1	0.6210096	0.780625228	-0.15961559	-0.33446269	5.93E-08	1.91E-06	4	0	0
ZNF729	0.5042088	0.570176525	-0.06596776	0.013353134	5.96E-08	1.91E-06	6	0	0
RNASE10	0.7109094	0.604406974	0.106502434	0.239985366	5.99E-08	1.92E-06	2	0	0
CDO1	0.391594	0.280566922	0.111027103	1.229769501	6.03E-08	1.93E-06	14	0	0
ANGEL1	0.7572873	0.860316105	-0.10302885	-0.18908092	6.08E-08	1.95E-06	5	0	0
OMD	0.7167669	0.664803771	0.05196317	0.116203401	6.18E-08	1.97E-06	3	0	0
CEACAM4	0.3726916	0.490899953	-0.11820832	-0.38906758	6.18E-08	1.97E-06	4	0	0
TMED6	0.8201805	0.75087601	0.069304491	0.136574058	6.20E-08	1.97E-06	6	0	0
OR52J3	0.6696526	0.813451994	-0.14379935	-0.27957053	6.34E-08	2.01E-06	3	0	0
FAM166B	0.6113198	0.508052424	0.103267418	0.351805519	6.42E-08	2.04E-06	8	0	0
NMUR2	0.5459134	0.690974336	-0.1450609	-0.34578575	6.51E-08	2.06E-06	5	0	0
OPCML	0.2962193	0.278422789	0.017796536	0.328991449	6.60E-08	2.08E-06	13	0	0
BBC3	0.3547421	0.426006417	-0.07126436	-0.41498656	6.61E-08	2.08E-06	5	0	0
CABP5	0.5759405	0.699760755	-0.12382022	-0.2792677	6.62E-08	2.08E-06	6	0	0
MS4A3	0.5326404	0.663624072	-0.13098362	-0.32146035	6.65E-08	2.09E-06	9	0	0

HTR4	0.644625	0.774000379	-0.12937533	-0.2838745	6.71E-08	2.10E-06	3	0	0
TMEM176A	0.1665133	0.093332965	0.073180379	0.95438609	6.71E-08	2.10E-06	11	0	0
SIM2	0.1850832	0.102849557	0.082233615	0.814446651	6.72E-08	2.10E-06	18	0	0
CSGALNACT	0.3322018	0.432941991	-0.10074021	-0.29906578	6.76E-08	2.10E-06	5	0	0
IL11RA	0.7634918	0.705638894	0.057852902	0.153213677	6.76E-08	2.10E-06	2	0	0
FHL5	0.5917873	0.694331199	-0.10254385	-0.14467281	6.78E-08	2.11E-06	5	0	0
OR10K1	0.4073663	0.619168207	-0.21180192	-0.5921317	6.85E-08	2.12E-06	1	0	0
TRIM49D1	0.6868772	0.856497514	-0.16962034	-0.31429122	6.96E-08	2.15E-06	1	0	0
NPS	0.6553674	0.801849722	-0.14648234	-0.29778596	7.01E-08	2.17E-06	4	0	0
ZNF217	0.4301832	0.543916968	-0.11373377	-0.33757653	7.11E-08	2.19E-06	7	0	0
SLC25A2	0.7549158	0.691199186	0.063716614	0.152722131	7.18E-08	2.21E-06	12	0	0
MAP3K9	0.1336008	0.171483569	-0.03788277	-0.28996825	7.17E-08	2.21E-06	11	0	0
MUC1	0.1067876	0.173936557	-0.06714899	-0.35146167	7.20E-08	2.21E-06	9	0	0
OR5AP2	0.712081	0.818884399	-0.10680336	-0.19850427	7.31E-08	2.24E-06	5	0	0
OR5AU1	0.4875058	0.63297939	-0.1454736	-0.37700541	7.31E-08	2.24E-06	6	0	0
FAM163B	0.5087657	0.597531314	-0.08876559	-0.25178044	7.34E-08	2.24E-06	3	0	0
DCK	0.4613844	0.596965289	-0.13558085	-0.3628823	7.39E-08	2.25E-06	2	0	0
PEX10	0.3945904	0.496291637	-0.10170126	-0.30933528	7.45E-08	2.27E-06	12	0	0
HCLS1	0.5801273	0.708111412	-0.12798412	-0.30435651	7.47E-08	2.27E-06	9	0	0
LGALS13	0.7413453	0.850807315	-0.10946205	-0.19978977	7.47E-08	2.27E-06	3	0	0
IL36A	0.7598823	0.844660658	-0.08477836	-0.16005822	7.49E-08	2.27E-06	6	0	0
NIPSNAP1	0.2681858	0.345012022	-0.07682623	-0.32532669	7.61E-08	2.30E-06	9	0	0
HYPK	0.790318	0.729062389	0.061255594	0.130542871	7.63E-08	2.30E-06	4	0	0
ABCA12	0.6849801	0.814233384	-0.12925329	-0.26585135	7.65E-08	2.30E-06	4	0	0
ZKSCAN2	0.086574	0.119527177	-0.03295315	-0.12286433	7.81E-08	2.34E-06	3	0	0
CCL11	0.6253063	0.758840812	-0.13353451	-0.27791568	7.81E-08	2.34E-06	6	0	0
CCDC129	0.5163792	0.667838169	-0.15145901	-0.36729357	7.88E-08	2.36E-06	4	0	0
CRP	0.5832154	0.74731085	-0.16409542	-0.35861949	7.90E-08	2.36E-06	4	0	0
HOXD11	0.376194	0.20788191	0.168312102	0.863718947	7.92E-08	2.36E-06	3	0	0
TPSAB1	0.5625548	0.664340433	-0.10178562	-0.23606329	7.94E-08	2.36E-06	1	0	0
RTL1	0.6836079	0.797758713	-0.11415077	-0.22236731	7.95E-08	2.36E-06	8	0	0
PCDH20	0.7565612	0.864481196	-0.10792004	-0.19705481	7.98E-08	2.37E-06	3	0	0
FAM213A	0.2817483	0.340485805	-0.05873748	-0.31619053	8.00E-08	2.37E-06	7	0	0
LAMC2	0.3786415	0.459834194	-0.08119267	-0.36885414	8.03E-08	2.37E-06	12	0	0
FAM118A	0.5748217	0.667974551	-0.09315281	-0.31361552	8.05E-08	2.38E-06	11	0	0
USP30	0.6998042	0.852318721	-0.15251454	-0.28963507	8.08E-08	2.38E-06	3	0	0
MRGPRG	0.5949569	0.679936313	-0.08497938	-0.19598879	8.15E-08	2.40E-06	7	0	0
DGKI	0.3228827	0.227384937	0.095497767	0.830978687	8.20E-08	2.41E-06	8	0	0
SPPL2C	0.5878404	0.707979144	-0.12013874	-0.26829001	8.32E-08	2.44E-06	11	0	0
DEFB121	0.520137	0.649822601	-0.12968556	-0.36275411	8.44E-08	2.47E-06	6	0	0
CYP11A1	0.6661801	0.750523934	-0.08434387	-0.17969642	8.47E-08	2.48E-06	12	0	0
OR2M3	0.5257562	0.684395861	-0.15863964	-0.37122858	8.63E-08	2.52E-06	4	0	0
PTP4A2	0.4389534	0.308179804	0.130773554	1.488310259	8.77E-08	2.55E-06	3	0	0
XAF1	0.5385064	0.458489588	0.080016781	0.237019028	8.76E-08	2.55E-06	5	0	0
OR2A25	0.6469547	0.797352551	-0.15039783	-0.29740486	8.79E-08	2.55E-06	1	0	0
CCDC179	0.8051376	0.883887703	-0.07875008	-0.13682431	8.87E-08	2.57E-06	7	0	0
EHBP1	0.7803904	0.690679213	0.08971116	0.193729225	8.94E-08	2.59E-06	6	0	0
OR8J3	0.5840061	0.722257736	-0.1382516	-0.31988024	8.96E-08	2.59E-06	4	0	0
ZFAND1	0.2512851	0.345522211	-0.0942371	-0.43250159	9.06E-08	2.61E-06	3	0	0
SET	0.305368	0.354583727	-0.04921571	-0.35347758	9.17E-08	2.64E-06	3	0	0
PP2D1	0.5895992	0.662475375	-0.07287618	-0.15003986	9.22E-08	2.65E-06	2	0	0
ADAMTS16	0.3015359	0.183919563	0.117616311	1.035818771	9.26E-08	2.66E-06	11	0	0
SUB1	0.4650597	0.644317428	-0.1792577	-0.46319425	9.40E-08	2.69E-06	4	0	0
SPATA19	0.6395554	0.776687271	-0.13713188	-0.27735469	9.41E-08	2.69E-06	7	0	0
RLN1	0.442641	0.575879096	-0.13323809	-0.37223568	9.55E-08	2.73E-06	1	0	0
IL37	0.6922417	0.815617581	-0.12337587	-0.24089288	9.65E-08	2.75E-06	4	0	0
SYT12	0.7146356	0.829524904	-0.11488933	-0.22856457	9.65E-08	2.75E-06	6	0	0
TRIM58	0.4501846	0.356287648	0.093896924	1.029903882	9.68E-08	2.75E-06	10	0	0
EVPL	0.5695701	0.647471817	-0.07790176	-0.19341688	9.70E-08	2.75E-06	5	0	0
NLRP4	0.6461327	0.771990644	-0.12585797	-0.27059996	9.67E-08	2.75E-06	9	0	0
ACCSL	0.6209393	0.748461044	-0.12752179	-0.26751655	9.93E-08	2.81E-06	4	0	0
ZBTB8A	0.548989	0.625712501	-0.07672352	-0.15089837	1.00E-07	2.83E-06	5	0	0
GDF2	0.6729394	0.621964878	0.050974523	0.13915156	1.01E-07	2.84E-06	13	0	0

FAM83G	0.3254821	0.360670589	-0.03518845	-0.27122593	1.01E-07	2.85E-06	12	0	0
CD1E	0.5400786	0.670982365	-0.13090377	-0.35518184	1.02E-07	2.87E-06	5	0	0
L1TD1	0.4917738	0.389847775	0.101926018	0.599733607	1.03E-07	2.88E-06	11	0	0
SORBS2	0.7593291	0.838630466	-0.07930141	-0.14693038	1.03E-07	2.88E-06	5	0	0
UNC93A	0.7702359	0.92551719	-0.15528129	-0.26185379	1.03E-07	2.88E-06	1	0	0
TBC1D21	0.5286019	0.648140336	-0.11953845	-0.30970506	1.03E-07	2.88E-06	8	0	0
BATF	0.4044205	0.541018578	-0.13659804	-0.4290572	1.04E-07	2.89E-06	11	0	0
SOX11	0.2495491	0.144126501	0.105422611	0.775470713	1.04E-07	2.90E-06	10	0	0
SLC24A2	0.4689885	0.484725721	-0.01573726	0.316432587	1.05E-07	2.91E-06	4	0	0
OR8D1	0.7941535	0.904730977	-0.11057746	-0.18609945	1.06E-07	2.94E-06	2	0	0
PACRGL	0.8701481	0.901112864	-0.03096472	-0.0498834	1.07E-07	2.96E-06	1	0	0
OR56B1	0.600124	0.775437301	-0.17531331	-0.36439365	1.07E-07	2.96E-06	1	0	0
HOXB2	0.5264519	0.434970384	0.091481551	0.266601012	1.07E-07	2.96E-06	5	0	0
HSBP1L1	0.3988	0.45181251	-0.05301247	-0.4445218	1.09E-07	3.01E-06	11	0	0
XIRP2	0.6661432	0.778365175	-0.11222195	-0.24226347	1.10E-07	3.04E-06	5	0	0
KRTAP22-2	0.6345771	0.728027552	-0.09345048	-0.2024139	1.11E-07	3.05E-06	4	0	0
CMTM2	0.4381229	0.30531037	0.132812563	0.709254983	1.12E-07	3.07E-06	12	0	0
KLHL33	0.6971046	0.588858448	0.108246155	0.277922952	1.13E-07	3.10E-06	10	0	0
CXCR1	0.5281953	0.622222198	-0.09402689	-0.35470011	1.13E-07	3.10E-06	5	0	0
MYT1L	0.5996462	0.7336517	-0.13400551	-0.30293747	1.13E-07	3.10E-06	6	0	0
TNF	0.4204115	0.527991827	-0.10758033	-0.3597667	1.15E-07	3.13E-06	17	0	0
TEK	0.5571774	0.455542894	0.101634552	0.263176502	1.17E-07	3.16E-06	6	0	0
INS-IGF2	0.6318666	0.729399949	-0.09753332	-0.2058977	1.17E-07	3.16E-06	13	0	0
INS	0.6318666	0.729399949	-0.09753332	-0.2058977	1.17E-07	3.16E-06	13	0	0
OR5T2	0.4247868	0.60333853	-0.1785517	-0.49637535	1.16E-07	3.16E-06	1	0	0
OR9G4	0.7080741	0.835865151	-0.12779101	-0.24563028	1.16E-07	3.16E-06	4	0	0
DAOA	0.7141597	0.824881378	-0.11072166	-0.21141229	1.17E-07	3.16E-06	3	0	0
PTGIR	0.5355665	0.469241741	0.066324714	0.155203176	1.17E-07	3.16E-06	4	0	0
SIGLEC6	0.47236	0.589247225	-0.1168872	-0.36232607	1.16E-07	3.16E-06	5	0	0
DNTT	0.6556419	0.76198763	-0.10634574	-0.23215704	1.18E-07	3.17E-06	6	0	0
BDKRB1	0.6065674	0.733936085	-0.12736865	-0.29050888	1.19E-07	3.19E-06	11	0	0
MTNR1B	0.3839771	0.303161887	0.080815186	0.323200897	1.21E-07	3.25E-06	9	0	0
OR8U1	0.6577948	0.776715273	-0.11892043	-0.25243754	1.22E-07	3.26E-06	5	0	0
GREM1	0.2177652	0.09307728	0.124687928	1.280681577	1.23E-07	3.30E-06	8	0	0
NLRP3	0.4185766	0.536342523	-0.1177659	-0.39082577	1.24E-07	3.30E-06	8	0	0
SLC2A9	0.6696482	0.790968881	-0.12132067	-0.24505416	1.24E-07	3.30E-06	4	0	0
SPHKAP	0.3001064	0.200182921	0.099923465	0.781456374	1.24E-07	3.31E-06	11	0	0
ELN	0.2464019	0.195575166	0.050826733	0.31570215	1.24E-07	3.31E-06	8	0	0
FAM107B	0.7204605	0.843723518	-0.12326306	-0.23392183	1.24E-07	3.31E-06	10	0	0
SYT9	0.6169506	0.78754683	-0.17059626	-0.35701301	1.25E-07	3.32E-06	3	0	0
DNAH5	0.6393873	0.779626591	-0.14023931	-0.30911546	1.26E-07	3.32E-06	9	0	0
NOX3	0.6390918	0.765672478	-0.12658065	-0.28736771	1.25E-07	3.32E-06	7	0	0
OR52H1	0.5925228	0.719080514	-0.12655773	-0.2680418	1.26E-07	3.33E-06	3	0	0
SMAD5	0.2050626	0.258364799	-0.05330215	-0.21684911	1.28E-07	3.37E-06	10	0	0
CALML3	0.6568284	0.76744506	-0.11061663	-0.23132263	1.30E-07	3.42E-06	8	0	0
LHX8	0.2750116	0.164800634	0.110211008	0.758170535	1.31E-07	3.45E-06	10	0	0
TERT	0.3681702	0.261599593	0.106570626	0.962550423	1.32E-07	3.46E-06	8	0	0
POU5F1B	0.6520116	0.621898283	0.030113354	0.021929351	1.33E-07	3.49E-06	2	0	0
OR2T6	0.584859	0.721356906	-0.13649786	-0.3068845	1.34E-07	3.51E-06	4	0	0
OC90	0.4975628	0.674425665	-0.17686289	-0.43987277	1.34E-07	3.51E-06	4	0	0
GRIA4	0.2345926	0.188740906	0.045851734	0.329172299	1.34E-07	3.51E-06	12	0	0
TECTB	0.5690593	0.681993026	-0.11293375	-0.2446385	1.35E-07	3.51E-06	6	0	0
OMP	0.7518149	0.820045606	-0.06823073	-0.13231681	1.40E-07	3.65E-06	7	0	0
OR2T33	0.4475327	0.581997627	-0.13446491	-0.37972019	1.41E-07	3.66E-06	3	0	0
PRMT8	0.527491	0.466177581	0.061313414	0.358207124	1.41E-07	3.67E-06	5	0	0
CLCA4	0.203056	0.284704869	-0.0816489	-0.4680384	1.42E-07	3.68E-06	1	0	0
EXOC3L1	0.5755274	0.486936874	0.088590557	0.259176333	1.42E-07	3.68E-06	7	0	0
ZNF454	0.393804	0.313380215	0.080423796	0.535371171	1.43E-07	3.70E-06	9	0	0
OR2AG2	0.8118382	0.905396836	-0.09355859	-0.15676877	1.45E-07	3.73E-06	5	0	0
PILRA	0.7040731	0.80383506	-0.09976197	-0.20699795	1.47E-07	3.79E-06	3	0	0
OR2AT4	0.681492	0.808352043	-0.12686009	-0.25224752	1.47E-07	3.79E-06	5	0	0
PCDH8	0.2372925	0.135024507	0.102268037	0.944187061	1.48E-07	3.81E-06	12	0	0
PCDHGB6	0.3433093	0.203050147	0.140259169	1.214528899	1.49E-07	3.82E-06	6	0	0

C2CD4A	0.2741833	0.328528804	-0.05434546	-0.3752628	1.49E-07	3.83E-06	11	0	0
IL3	0.7159245	0.780980853	-0.06505639	-0.1300808	1.50E-07	3.85E-06	10	0	0
CORO7-PAM	0.6100618	0.701201054	-0.09113925	-0.23436089	1.52E-07	3.87E-06	2	0	0
CAPNS2	0.735447	0.840521805	-0.10507476	-0.20709815	1.52E-07	3.88E-06	5	0	0
09-Sep	0.3482299	0.4569365	-0.10870664	-0.41865904	1.52E-07	3.88E-06	4	0	0
OR5F1	0.6391902	0.75552926	-0.11633901	-0.24092941	1.55E-07	3.94E-06	4	0	0
VNN1	0.5562012	0.676151065	-0.11994983	-0.28205552	1.55E-07	3.95E-06	8	0	0
NMS	0.6741955	0.822684738	-0.14848919	-0.29218474	1.56E-07	3.96E-06	2	0	0
OR51B4	0.6818961	0.80547172	-0.12357565	-0.24225055	1.56E-07	3.96E-06	6	0	0
RFFL	0.2968196	0.414164743	-0.1173451	-0.49138849	1.58E-07	3.99E-06	5	0	0
COL11A1	0.3259461	0.226407406	0.099538735	0.762007195	1.58E-07	4.00E-06	13	0	0
OR10G4	0.7240261	0.863720328	-0.13969425	-0.27008385	1.61E-07	4.04E-06	2	0	0
CCL18	0.5161509	0.647682733	-0.13153182	-0.31814033	1.60E-07	4.04E-06	3	0	0
ZNF418	0.441293	0.330980351	0.110312658	0.80937275	1.61E-07	4.04E-06	14	0	0
DEFB115	0.4310612	0.567298307	-0.13623708	-0.38019318	1.60E-07	4.04E-06	6	0	0
OR10G8	0.7411518	0.860644154	-0.11949231	-0.2255622	1.61E-07	4.05E-06	4	0	0
NAV1	0.7844348	0.862974456	-0.07853964	-0.13434007	1.63E-07	4.09E-06	5	0	0
CDRT1	0.7965282	0.702564543	0.09396368	0.179473734	1.63E-07	4.09E-06	2	0	0
FXR1	0.4243585	0.552172206	-0.12781367	-0.37212573	1.65E-07	4.11E-06	1	0	0
GJA5	0.65137	0.657211851	-0.00584184	0.048777116	1.65E-07	4.12E-06	6	0	0
TPM1	0.3586855	0.275834357	0.082851121	0.582748071	1.67E-07	4.17E-06	10	0	0
NLRP8	0.7095779	0.834897346	-0.12531946	-0.23450717	1.69E-07	4.21E-06	6	0	0
KBTBD12	0.3955389	0.254658938	0.140879928	0.706188429	1.70E-07	4.22E-06	10	0	0
KRTAP2-4	0.8515429	0.910822595	-0.05927967	-0.09926546	1.72E-07	4.26E-06	2	0	0
LZTFL1	0.5756156	0.589022905	-0.01340727	-0.06788615	1.73E-07	4.27E-06	6	0	0
NKX2-1	0.2075567	0.108195504	0.099361212	0.82908666	1.73E-07	4.27E-06	8	0	0
LMX1A	0.3081004	0.209485197	0.098615249	0.90023686	1.74E-07	4.27E-06	13	0	0
OR4A15	0.3707485	0.50475968	-0.13401115	-0.44188421	1.73E-07	4.27E-06	4	0	0
PRAC2	0.2721962	0.123859245	0.148336998	1.146929996	1.73E-07	4.27E-06	17	0	0
KCNK16	0.5923713	0.711329325	-0.11895804	-0.26682879	1.75E-07	4.30E-06	6	0	0
ZNF273	0.7986229	0.891698325	-0.09307538	-0.16676934	1.75E-07	4.30E-06	3	0	0
STAR	0.4816383	0.397336296	0.084301976	0.849486203	1.76E-07	4.31E-06	4	0	0
ZPBP2	0.5160631	0.441840963	0.074222166	0.364923532	1.76E-07	4.31E-06	6	0	0
SFXN5	0.6709403	0.76416719	-0.09322688	-0.19505581	1.77E-07	4.33E-06	2	0	0
LHX5	0.1837397	0.079483432	0.104256263	1.320169946	1.79E-07	4.37E-06	13	0	0
KRT5	0.5357504	0.672894487	-0.13714406	-0.32283566	1.81E-07	4.41E-06	9	0	0
PTF1A	0.260117	0.154991437	0.105125595	0.835032247	1.82E-07	4.42E-06	9	0	0
ARRDC4	0.5857605	0.716138002	-0.13037746	-0.305891	1.83E-07	4.44E-06	2	0	0
TP73	0.2522523	0.149991906	0.102260375	0.590071004	1.84E-07	4.46E-06	20	0	0
CD79A	0.406824	0.486798178	-0.07997416	-0.26788919	1.84E-07	4.46E-06	7	0	0
N4BP1	0.7120163	0.614412641	0.09760368	0.22099365	1.84E-07	4.46E-06	4	0	0
PHYHD1	0.6543375	0.578599611	0.075737847	0.253108164	1.85E-07	4.46E-06	8	0	0
TACR3	0.3413879	0.228397585	0.112990349	0.758316704	1.85E-07	4.47E-06	11	0	0
PANX3	0.5797203	0.676571396	-0.09685106	-0.22840629	1.86E-07	4.47E-06	6	0	0
OSBPL5	0.7140162	0.797417016	-0.08340077	-0.16131841	1.89E-07	4.54E-06	14	0	0
TSKU	0.4908752	0.555685971	-0.06481073	-0.21795207	1.88E-07	4.54E-06	6	0	0
OR5AS1	0.7156478	0.835433933	-0.11978617	-0.23429026	1.91E-07	4.58E-06	4	0	0
SIGLEC10	0.4177929	0.529911205	-0.11211827	-0.33127893	1.93E-07	4.63E-06	5	0	0
CPEB1	0.4676965	0.373196184	0.094500357	0.339093556	1.95E-07	4.68E-06	6	0	0
TAAR8	0.7467076	0.836098374	-0.0893908	-0.16061944	1.97E-07	4.69E-06	3	0	0
LRIT2	0.5462236	0.669726634	-0.12350299	-0.31852814	1.97E-07	4.69E-06	9	0	0
SLC32A1	0.2103127	0.087107456	0.123205243	1.237611823	1.97E-07	4.69E-06	9	0	0
EDN3	0.2475308	0.174893871	0.072636891	0.765888843	1.98E-07	4.72E-06	13	0	0
HDAC2	0.761467	0.836317531	-0.07485056	-0.13359466	2.00E-07	4.75E-06	1	0	0
OR2A2	0.6350578	0.77541258	-0.14035474	-0.29720144	2.00E-07	4.75E-06	4	0	0
C12orf56	0.4079793	0.310373098	0.09760617	1.123519412	2.00E-07	4.75E-06	8	0	0
MORC1	0.790408	0.897269112	-0.10686113	-0.19226799	2.04E-07	4.82E-06	13	0	0
ELFN1	0.6797097	0.802776822	-0.12306715	-0.24177078	2.04E-07	4.82E-06	2	0	0
DEFA4	0.7952122	0.860389253	-0.06517709	-0.11698838	2.04E-07	4.82E-06	3	0	0
HOMEZ	0.4279803	0.546548384	-0.11856808	-0.34355927	2.03E-07	4.82E-06	4	0	0
TMPRSS15	0.6521268	0.769022599	-0.11689583	-0.23871445	2.07E-07	4.88E-06	3	0	0
OR5A2	0.6035635	0.716806424	-0.11324297	-0.2516456	2.08E-07	4.90E-06	5	0	0
GNLY	0.5782772	0.724192692	-0.14591547	-0.31966258	2.08E-07	4.90E-06	1	0	0

MRGPRF	0.3278009	0.209552269	0.118248587	0.758125344	2.09E-07	4.92E-06	14	0	0
LRRC61	0.2323612	0.307529719	-0.07516856	-0.37931831	2.11E-07	4.94E-06	6	0	0
CCR5	0.5053136	0.631209389	-0.12589576	-0.35713201	2.12E-07	4.96E-06	7	0	0
ZNF382	0.2906943	0.14752784	0.143166431	1.26396458	2.14E-07	5.00E-06	8	0	0
OR51G2	0.4715533	0.595814454	-0.12426113	-0.32772063	2.16E-07	5.04E-06	2	0	0
RBP7	0.2085539	0.262019615	-0.05346576	0.113926816	2.18E-07	5.08E-06	11	0	0
C3orf18	0.6263402	0.577435391	0.048904783	0.124161318	2.18E-07	5.08E-06	6	0	0
NXF1	0.3954613	0.485068873	-0.08960757	-0.29927779	2.18E-07	5.08E-06	6	0	0
SEC14L6	0.6936865	0.78729964	-0.09361314	-0.18290639	2.19E-07	5.10E-06	4	0	0
TG	0.7001986	0.830972219	-0.13077366	-0.25314961	2.22E-07	5.14E-06	5	0	0
FES	0.4019083	0.268585449	0.133322856	0.575813027	2.23E-07	5.17E-06	3	0	0
OR14I1	0.5571647	0.722928771	-0.16576407	-0.36902623	2.24E-07	5.18E-06	2	0	0
LILRB1	0.2833145	0.385248446	-0.10193396	-0.42992518	2.26E-07	5.23E-06	3	0	0
GRM5	0.2518397	0.125167884	0.126671835	0.953931554	2.27E-07	5.23E-06	1	0	0
NLRX1	0.5840085	0.732527944	-0.14851939	-0.32146084	2.27E-07	5.23E-06	2	0	0
KCNIP2	0.2829488	0.352288818	-0.06934003	-0.4037964	2.30E-07	5.30E-06	14	0	0
KRTAP19-6	0.6177303	0.747557616	-0.12982733	-0.28854704	2.31E-07	5.31E-06	3	0	0
OR1M1	0.7020201	0.803737886	-0.10171775	-0.20004505	2.31E-07	5.31E-06	5	0	0
PEX5L	0.2377027	0.142249357	0.095453368	1.340475266	2.32E-07	5.33E-06	9	0	0
OR4C6	0.531309	0.680414615	-0.14910566	-0.35030898	2.33E-07	5.33E-06	2	0	0
PROKR2	0.1360025	0.037322066	0.098680459	1.604851808	2.33E-07	5.33E-06	2	0	0
RFPL2	0.5347727	0.673381825	-0.13860915	-0.32330229	2.34E-07	5.34E-06	5	0	0
G6PC	0.6098459	0.736140437	-0.1262945	-0.31073186	2.35E-07	5.35E-06	7	0	0
ZNF479	0.4633662	0.605664684	-0.14229844	-0.38832342	2.37E-07	5.41E-06	2	0	0
MEST	0.4012526	0.499517413	-0.09826478	-0.37560095	2.41E-07	5.48E-06	9	0	0
PLEKHS1	0.7042986	0.833195462	-0.12889686	-0.24241931	2.42E-07	5.51E-06	4	0	0
ERVV-1	0.7282691	0.838680304	-0.11041119	-0.20351076	2.44E-07	5.55E-06	6	0	0
CD28	0.5404617	0.682146989	-0.14168525	-0.36704068	2.45E-07	5.55E-06	6	0	0
IL23A	0.371517	0.445740497	-0.07422348	-0.41649427	2.46E-07	5.57E-06	7	0	0
PAX1	0.2020939	0.095289488	0.10680439	1.167340308	2.48E-07	5.60E-06	17	0	0
DEFB126	0.3858776	0.548981425	-0.16310384	-0.49774595	2.49E-07	5.62E-06	1	0	0
CATSPERB	0.5713553	0.468991656	0.102363661	0.313779046	2.50E-07	5.63E-06	2	0	0
SCGB1D1	0.5757153	0.700094741	-0.12437949	-0.28000063	2.50E-07	5.63E-06	6	0	0
KRTAP6-1	0.5138963	0.626582914	-0.11268663	-0.32614207	2.51E-07	5.64E-06	3	0	0
OR52N1	0.6104513	0.72787789	-0.11742659	-0.24304833	2.52E-07	5.66E-06	4	0	0
OR4C46	0.4201132	0.596468408	-0.17635518	-0.49571598	2.54E-07	5.69E-06	1	0	0
PTK6	0.3189529	0.40572985	-0.08677696	-0.32051723	2.54E-07	5.69E-06	13	0	0
EVX2	0.3107264	0.182511638	0.128214769	0.79274066	2.56E-07	5.72E-06	11	0	0
VSTM1	0.5196466	0.643152434	-0.12350583	-0.3231247	2.57E-07	5.73E-06	11	0	0
PGM1	0.1861816	0.236353947	-0.05017234	-0.35442566	2.59E-07	5.78E-06	17	0	0
MBL2	0.6250994	0.739428319	-0.11432897	-0.24044682	2.59E-07	5.78E-06	5	0	0
OASL	0.3191619	0.394184841	-0.07502291	-0.33543264	2.62E-07	5.84E-06	8	0	0
DEFB116	0.4544707	0.592643544	-0.13817281	-0.37321307	2.63E-07	5.84E-06	6	0	0
PRSS55	0.6303122	0.738751362	-0.10843914	-0.23047187	2.64E-07	5.86E-06	6	0	0
SLAMF7	0.3796819	0.536538565	-0.15685666	-0.49894417	2.65E-07	5.88E-06	3	0	0
FAM135B	0.3591107	0.268233377	0.090877348	0.511536127	2.67E-07	5.90E-06	7	0	0
SKOR2	0.3127133	0.19145839	0.121254938	0.695956791	2.67E-07	5.92E-06	8	0	0
GLT6D1	0.6953451	0.825666995	-0.13032188	-0.24760286	2.69E-07	5.95E-06	6	0	0
GPR37L1	0.6492392	0.614157558	0.035081662	0.145452505	2.71E-07	5.97E-06	9	0	0
OR5T3	0.4318946	0.612071456	-0.18017689	-0.49337811	2.71E-07	5.97E-06	1	0	0
OR13C2	0.5399848	0.663453647	-0.12346887	-0.30914211	2.71E-07	5.97E-06	3	0	0
LRP5	0.6789543	0.669056252	0.009898052	-0.0244668	2.76E-07	6.06E-06	3	0	0
ZNF492	0.4794823	0.429460427	0.0500219	0.630154413	2.76E-07	6.06E-06	8	0	0
GJB5	0.5473331	0.66260529	-0.11527221	-0.2734221	2.82E-07	6.17E-06	6	0	0
PDE4B	0.3219069	0.238596291	0.08331058	0.658472034	2.83E-07	6.17E-06	8	0	0
OOSP2	0.6871494	0.817225919	-0.13007656	-0.24873082	2.83E-07	6.17E-06	9	0	0
OR4E2	0.7726065	0.861676768	-0.08907022	-0.16515944	2.81E-07	6.17E-06	6	0	0
CERS3	0.7658072	0.683560763	0.082246414	0.179334397	2.82E-07	6.17E-06	8	0	0
IGLL1	0.4013731	0.487784103	-0.08641102	-0.27449794	2.83E-07	6.17E-06	5	0	0
GRP	0.1992727	0.14014184	0.059130857	0.842866856	2.84E-07	6.19E-06	13	0	0
C2orf78	0.6206235	0.758234606	-0.13761109	-0.28614503	2.90E-07	6.29E-06	2	0	0
12-Sep	0.6677024	0.56883295	0.098869428	0.252220871	2.90E-07	6.29E-06	7	0	0
RPA3	0.5441893	0.690231488	-0.14604223	-0.37871662	2.91E-07	6.32E-06	2	0	0

OR8K5	0.6625794	0.784950229	-0.12237082	-0.2526377	2.96E-07	6.41E-06	3	0	0
TMEM30B	0.4684294	0.57320967	-0.10478022	-0.29993294	2.98E-07	6.44E-06	5	0	0
NAA11	0.7122842	0.813650038	-0.10136584	-0.19632473	3.00E-07	6.49E-06	9	0	0
AARS2	0.2904022	0.337102083	-0.04669993	-0.29859753	3.04E-07	6.57E-06	13	0	0
HCN1	0.2855819	0.189719174	0.095862734	0.796247988	3.06E-07	6.58E-06	8	0	0
MKX	0.236158	0.115362914	0.120795109	1.044371881	3.05E-07	6.58E-06	17	0	0
TNRC6C	0.7794135	0.86351788	-0.08410436	-0.15025675	3.06E-07	6.58E-06	5	0	0
SP9	0.2975434	0.159657966	0.137885415	0.924430948	3.08E-07	6.62E-06	11	0	0
CAPSL	0.4589676	0.607736996	-0.14876936	-0.38117531	3.12E-07	6.70E-06	6	0	0
MMP7	0.6014451	0.65837761	-0.05693251	-0.28960512	3.19E-07	6.83E-06	6	0	0
IMPG1	0.5623521	0.677563703	-0.11521158	-0.28464973	3.20E-07	6.86E-06	3	0	0
TMEM202	0.5653064	0.655367433	-0.09006108	-0.25503347	3.22E-07	6.89E-06	2	0	0
FGF6	0.6713317	0.767820192	-0.09648854	-0.20269631	3.28E-07	7.00E-06	11	0	0
GALR1	0.2123771	0.106376516	0.106000547	1.101288619	3.29E-07	7.01E-06	21	0	0
RYR2	0.3860851	0.262642893	0.12344223	0.900578871	3.30E-07	7.02E-06	6	0	0
TMEM173	0.3495431	0.314392772	0.035150357	0.583429875	3.30E-07	7.02E-06	6	0	0
CYP1A2	0.7336974	0.806878763	-0.07318133	-0.1404224	3.33E-07	7.07E-06	7	0	0
OVGP1	0.6267394	0.721026063	-0.09428661	-0.20522988	3.45E-07	7.32E-06	5	0	0
IFNA21	0.7237388	0.850660677	-0.12692192	-0.23017807	3.46E-07	7.33E-06	1	0	0
CEL	0.4707258	0.585036744	-0.11431095	-0.32851721	3.48E-07	7.38E-06	2	0	0
ASPRV1	0.8136663	0.866204874	-0.0525386	-0.08986443	3.51E-07	7.42E-06	5	0	0
ACSM1	0.722061	0.860504129	-0.13844315	-0.25013525	3.52E-07	7.43E-06	3	0	0
OR2G6	0.6719443	0.80186844	-0.12992416	-0.26355358	3.55E-07	7.50E-06	4	0	0
CITED4	0.1934499	0.225875248	-0.03242537	0.056004681	3.58E-07	7.55E-06	9	0	0
PHOX2B	0.2493631	0.15985838	0.089504753	0.658448242	3.61E-07	7.59E-06	12	0	0
OR6B1	0.6406834	0.762240026	-0.12155664	-0.27293972	3.63E-07	7.63E-06	8	0	0
HLA-DMB	0.2228621	0.269438141	-0.04657605	-0.35676067	3.66E-07	7.69E-06	14	0	0
OR2V1	0.6496722	0.745332046	-0.09565986	-0.19151523	3.71E-07	7.79E-06	4	0	0
IRX2	0.2536448	0.113089356	0.140555487	1.1472884	3.72E-07	7.80E-06	14	0	0
CCL7	0.6454327	0.761205257	-0.11577251	-0.23447762	3.74E-07	7.83E-06	4	0	0
PRAME	0.5016884	0.613807526	-0.11211908	-0.3256738	3.79E-07	7.93E-06	12	0	0
STARD13	0.342048	0.25618815	0.085859831	0.58157524	3.80E-07	7.93E-06	6	0	0
CDSN	0.562787	0.654809937	-0.09202297	-0.22316134	3.81E-07	7.94E-06	5	0	0
OR10A5	0.7055598	0.817258876	-0.11169908	-0.21398673	3.82E-07	7.96E-06	2	0	0
C19orf18	0.7651591	0.860826833	-0.09566773	-0.17312606	3.83E-07	7.97E-06	3	0	0
DAB2	0.7050426	0.849909094	-0.1448665	-0.26518256	3.86E-07	8.02E-06	2	0	0
ACSBG2	0.6884488	0.826830313	-0.13838151	-0.27361304	3.93E-07	8.14E-06	7	0	0
CYP4F3	0.4920894	0.573499058	-0.08140968	-0.31157622	3.93E-07	8.14E-06	4	0	0
SPP2	0.7865223	0.877197691	-0.09067534	-0.15654624	3.95E-07	8.17E-06	3	0	0
ERP27	0.8219243	0.91196424	-0.09003993	-0.15244695	3.95E-07	8.17E-06	3	0	0
OR8G5	0.6154803	0.738552726	-0.12307246	-0.26626827	3.99E-07	8.24E-06	3	0	0
FILIP1	0.5951696	0.455826317	0.139343235	0.424942297	4.00E-07	8.25E-06	8	0	0
LAMB4	0.7985725	0.877980255	-0.07940772	-0.14026993	4.03E-07	8.30E-06	7	0	0
RNF150	0.8139122	0.861293389	-0.04738124	-0.08145062	4.04E-07	8.33E-06	3	0	0
RAB37	0.4678167	0.552677142	-0.08486042	-0.16662851	4.05E-07	8.34E-06	9	0	0
MEI4	0.6498417	0.761136573	-0.1112949	-0.23216769	4.07E-07	8.37E-06	3	0	0
C6	0.7056502	0.82622415	-0.12057391	-0.22334353	4.08E-07	8.38E-06	4	0	0
CBR3	0.1801583	0.243752922	-0.06359462	-0.27180011	4.15E-07	8.51E-06	7	0	0
APOC1	0.4758656	0.463542177	0.012323428	0.321999599	4.16E-07	8.52E-06	2	0	0
SPAG6	0.3089625	0.181579104	0.127383369	0.866751936	4.19E-07	8.56E-06	15	0	0
USP6	0.4471299	0.322961768	0.124168142	0.568117993	4.20E-07	8.58E-06	8	0	0
SLCO1B1	0.4773296	0.64272391	-0.1653943	-0.4374301	4.23E-07	8.63E-06	2	0	0
RINT1	0.1781592	0.269422533	-0.09126335	-0.37981159	4.24E-07	8.64E-06	4	0	0
CCDC105	0.51821	0.42302345	0.095186518	0.377458021	4.27E-07	8.69E-06	10	0	0
GFRAL	0.555914	0.69019348	-0.13427946	-0.31348473	4.27E-07	8.69E-06	6	0	0
CYP2C9	0.613625	0.731608368	-0.11798334	-0.25139163	4.31E-07	8.76E-06	3	0	0
CPE	0.7260774	0.842562448	-0.11648506	-0.21194805	4.42E-07	8.96E-06	1	0	0
PLCL2	0.4564467	0.568456392	-0.11200968	-0.32698504	4.45E-07	9.01E-06	3	0	0
TCTEX1D4	0.4160305	0.32891883	0.087111652	0.54782791	4.48E-07	9.07E-06	8	0	0
TGFBR3	0.7722335	0.900556228	-0.12832277	-0.2199607	4.51E-07	9.12E-06	5	0	0
S100A4	0.6807777	0.691203915	-0.01042618	-0.02479526	4.52E-07	9.14E-06	4	0	0
MTMR2	0.7090409	0.839777473	-0.13073659	-0.24980842	4.54E-07	9.16E-06	3	0	0
POU4F2	0.2193262	0.101276069	0.118050168	1.094306109	4.57E-07	9.22E-06	6	0	0

KLF11	0.3476198	0.258185029	0.089434808	0.893311349	4.61E-07	9.28E-06	7	0	0
GPNMB	0.6506924	0.835275984	-0.18458354	-0.35544361	4.61E-07	9.28E-06	1	0	0
OR3A3	0.6516383	0.774889353	-0.12325106	-0.24677387	4.67E-07	9.38E-06	3	0	0
DNAH14	0.4845563	0.613435906	-0.12887964	-0.33410658	4.71E-07	9.44E-06	1	0	0
OR52D1	0.7316225	0.848987608	-0.11736512	-0.21195232	4.71E-07	9.44E-06	1	0	0
OR9G1	0.611795	0.712807036	-0.10101201	-0.23077519	4.72E-07	9.44E-06	4	0	0
PRAC1	0.2544753	0.116854285	0.137620991	1.125239202	4.72E-07	9.44E-06	21	0	0
SPEF2	0.2242865	0.303925939	-0.07963947	-0.57771704	4.78E-07	9.54E-06	10	0	0
NFKBIE	0.6344332	0.744037881	-0.10960464	-0.22589292	4.78E-07	9.54E-06	2	0	0
IRX3	0.2112661	0.146438508	0.064827627	0.627298905	4.78E-07	9.54E-06	7	0	0
OR5W2	0.6476003	0.790837047	-0.14323674	-0.29773841	4.79E-07	9.54E-06	2	0	0
C1QTNF3	0.614794	0.546602182	0.068191788	0.290589242	4.80E-07	9.55E-06	7	0	0
TAS2R39	0.6934519	0.809387367	-0.1159355	-0.22313798	4.84E-07	9.61E-06	5	0	0
ENPP5	0.1364812	0.165420359	-0.02893917	-0.3283826	4.85E-07	9.63E-06	12	0	0
BLK	0.648616	0.743697387	-0.09508138	-0.19460574	4.88E-07	9.67E-06	11	0	0
FCGR3B	0.6876569	0.825679322	-0.13802242	-0.26043172	4.92E-07	9.74E-06	1	0	0
DCD	0.6545427	0.778854809	-0.12431206	-0.26930492	4.92E-07	9.74E-06	5	0	0
LRRC55	0.2852193	0.38028881	-0.09506956	-0.49983359	4.94E-07	9.76E-06	5	0	0
NLRP12	0.6575499	0.767726511	-0.11017665	-0.24143808	4.96E-07	9.79E-06	3	0	0
ZNF80	0.7214341	0.810767714	-0.08933359	-0.16281513	4.98E-07	9.81E-06	4	0	0
OR4S1	0.5905925	0.710271528	-0.119679	-0.26551085	4.98E-07	9.81E-06	5	0	0
OR7D4	0.5110332	0.646865843	-0.1358326	-0.34167184	4.99E-07	9.81E-06	2	0	0
LEKR1	0.5931242	0.668246412	-0.0751222	-0.26771466	5.06E-07	9.94E-06	2	0	0
OR51D1	0.681416	0.765217627	-0.08380166	-0.18791074	5.14E-07	1.01E-05	3	0	0
ANKRD2	0.5205049	0.599780719	-0.07927584	-0.25798317	5.17E-07	1.01E-05	9	0	0
UNC13C	0.6013708	0.732929498	-0.13155869	-0.28924361	5.18E-07	1.01E-05	5	0	0
ACVRL1	0.37781	0.277647834	0.100162194	0.555728276	5.22E-07	1.02E-05	7	0	0
PRDM13	0.3045865	0.200297954	0.104288524	0.649433956	5.24E-07	1.02E-05	15	0	0
DNAJA4	0.5459483	0.629089505	-0.08314123	-0.04766688	5.24E-07	1.02E-05	10	0	0
MZB1	0.5212345	0.606141925	-0.08490741	-0.23179536	5.27E-07	1.03E-05	9	0	0
TLX3	0.3012878	0.187358361	0.113929424	0.775030291	5.28E-07	1.03E-05	14	0	0
OR4C12	0.5896716	0.768838494	-0.17916694	-0.37715165	5.28E-07	1.03E-05	1	0	0
FOXR1	0.7092972	0.670187342	0.039109888	0.117734413	5.28E-07	1.03E-05	8	0	0
PNLIPRP3	0.6235886	0.750700841	-0.12711226	-0.2744493	5.35E-07	1.04E-05	3	0	0
GSTP1	0.2504845	0.336005427	-0.08552094	-0.31096816	5.36E-07	1.04E-05	6	0	0
DLEU7	0.6485842	0.593410359	0.055173797	0.240622202	5.42E-07	1.05E-05	9	0	0
ASIC5	0.752741	0.840769293	-0.0880283	-0.15828525	5.48E-07	1.06E-05	3	0	0
FITM1	0.7554333	0.703682938	0.051750369	0.130598875	5.47E-07	1.06E-05	6	0	0
CCL8	0.6133123	0.702263508	-0.08895121	-0.19937177	5.47E-07	1.06E-05	7	0	0
OR2B3	0.5624503	0.699528087	-0.13707781	-0.31495964	5.49E-07	1.06E-05	5	0	0
CASP1	0.7329663	0.826801568	-0.09383529	-0.18466992	5.51E-07	1.06E-05	2	0	0
CARD16	0.7329663	0.826801568	-0.09383529	-0.18466992	5.51E-07	1.06E-05	2	0	0
CARD17	0.7329663	0.826801568	-0.09383529	-0.18466992	5.51E-07	1.06E-05	2	0	0
GSDMC	0.4931029	0.630664133	-0.13756119	-0.33575415	5.53E-07	1.06E-05	6	0	0
FAM71F2	0.718046	0.687568822	0.030477163	0.080921919	5.54E-07	1.06E-05	5	0	0
ACOX2	0.5118192	0.475902296	0.035916943	0.130212867	5.55E-07	1.06E-05	5	0	0
CNTNAP5	0.2832374	0.16917244	0.114064911	0.720785793	5.55E-07	1.06E-05	9	0	0
C10orf99	0.671413	0.77033452	-0.0989215	-0.19351976	5.58E-07	1.06E-05	6	0	0
SLC4A1	0.6542548	0.751935112	-0.09768027	-0.20913109	5.62E-07	1.07E-05	9	0	0
DEFB128	0.6224351	0.735616687	-0.11318162	-0.25066407	5.66E-07	1.08E-05	4	0	0
OTOL1	0.6040575	0.758869901	-0.15481244	-0.33157702	5.67E-07	1.08E-05	4	0	0
RACGAP1	0.4707976	0.31353786	0.157259728	0.597002777	5.71E-07	1.09E-05	3	0	0
CX3CR1	0.6660529	0.722121435	-0.05606855	-0.13921242	5.73E-07	1.09E-05	8	0	0
GPR75	0.6967761	0.618075156	0.078700961	0.182961697	5.77E-07	1.09E-05	14	0	0
KRTAP19-5	0.702107	0.812244163	-0.11013715	-0.22809406	5.79E-07	1.10E-05	6	0	0
CP	0.5812031	0.667856154	-0.08665301	-0.23083751	5.80E-07	1.10E-05	4	0	0
ESRRG	0.2850825	0.173185754	0.111896709	0.708887071	5.83E-07	1.10E-05	8	0	0
FPR3	0.5297485	0.669842722	-0.14009427	-0.28499748	5.83E-07	1.10E-05	3	0	0
FOXD2	0.5005852	0.392593899	0.107991309	0.442567257	5.83E-07	1.10E-05	7	0	0
PAPOLB	0.607054	0.58158787	0.025466087	0.141154038	5.86E-07	1.10E-05	10	0	0
ZNF732	0.375	0.239204892	0.135795144	0.673265482	5.89E-07	1.11E-05	4	0	0
LRRC30	0.699338	0.786127828	-0.08678987	-0.17386704	5.89E-07	1.11E-05	6	0	0
OR2D2	0.7193186	0.834766917	-0.1154483	-0.21387937	5.92E-07	1.11E-05	3	0	0

OR5D18	0.6154075	0.723755751	-0.10834827	-0.2381525	5.95E-07	1.12E-05	7	0	0
CCR2	0.5543302	0.64809968	-0.09376948	-0.26746812	5.96E-07	1.12E-05	6	0	0
ZNF750	0.660504	0.82670881	-0.16620485	-0.32232188	5.96E-07	1.12E-05	13	0	0
ZNF728	0.5661175	0.521009193	0.045108282	0.324458951	5.99E-07	1.12E-05	8	0	0
LCP2	0.4931349	0.563846813	-0.07071191	-0.23083496	6.05E-07	1.13E-05	6	0	0
SH3BP5	0.7158322	0.702738688	0.013093531	0.045072734	6.12E-07	1.14E-05	7	0	0
TAS2R1	0.6270614	0.75074826	-0.12368688	-0.26040897	6.14E-07	1.14E-05	3	0	0
PROB1	0.7091322	0.697524364	0.011607813	0.046498955	6.12E-07	1.14E-05	7	0	0
KRTAP13-1	0.6463246	0.769937211	-0.12361259	-0.25861901	6.13E-07	1.14E-05	7	0	0
ASCL4	0.5197535	0.645095009	-0.12534151	-0.33620265	6.15E-07	1.14E-05	8	0	0
EFCAB9	0.725336	0.797057737	-0.07172177	-0.13585193	6.18E-07	1.15E-05	2	0	0
TM4SF19	0.5762595	0.7286921	-0.15243256	-0.34149846	6.21E-07	1.15E-05	12	0	0
PKD1L1	0.7085524	0.649946742	0.058605636	0.144020373	6.21E-07	1.15E-05	6	0	0
OR52E8	0.6797354	0.778932947	-0.09919754	-0.19164323	6.21E-07	1.15E-05	6	0	0
PSMB11	0.6866298	0.772297396	-0.08566757	-0.17174693	6.19E-07	1.15E-05	7	0	0
ZNF710	0.4606414	0.343394075	0.117247291	0.371086532	6.20E-07	1.15E-05	6	0	0
XCR1	0.8264431	0.897879271	-0.07143621	-0.1209315	6.23E-07	1.15E-05	2	0	0
LIPN	0.6139347	0.733072041	-0.11913736	-0.27896249	6.22E-07	1.15E-05	2	0	0
C3orf62	0.7238246	0.686804055	0.037020495	0.123353459	6.26E-07	1.15E-05	7	0	0
MCC	0.2514183	0.319257355	-0.06783904	-0.35811645	6.27E-07	1.15E-05	11	0	0
C14orf93	0.1140151	0.152796482	-0.03878137	-0.23453977	6.28E-07	1.16E-05	8	0	0
GPR132	0.4223463	0.502525989	-0.08017969	-0.28934578	6.31E-07	1.16E-05	7	0	0
SLC22A18AS	0.6916249	0.788091873	-0.09646697	-0.19062941	6.33E-07	1.16E-05	13	0	0
OR51F2	0.6736374	0.799389139	-0.12575175	-0.26098659	6.34E-07	1.16E-05	3	0	0
CNTN3	0.7387947	0.830139813	-0.09134512	-0.17888179	6.37E-07	1.17E-05	3	0	0
MRGPRX4	0.6281581	0.759977606	-0.13181955	-0.28079651	6.43E-07	1.18E-05	3	0	0
IL20RA	0.6117326	0.757265956	-0.14553337	-0.31185801	6.45E-07	1.18E-05	10	0	0
ADCY8	0.3890267	0.34859483	0.040431899	0.601852413	6.49E-07	1.19E-05	6	0	0
TTLL2	0.702028	0.811037451	-0.10900949	-0.20468373	6.52E-07	1.19E-05	4	0	0
C1QC	0.3056814	0.36982501	-0.06414362	-0.27557202	6.56E-07	1.19E-05	6	0	0
AGXT	0.4870451	0.568586436	-0.08154133	-0.2394902	6.56E-07	1.19E-05	5	0	0
UGT3A1	0.7106768	0.860347916	-0.14967107	-0.27228573	6.67E-07	1.21E-05	2	0	0
OR14A16	0.6821152	0.801555019	-0.11943978	-0.22470216	6.69E-07	1.22E-05	6	0	0
OR1L4	0.673989	0.787515597	-0.1135266	-0.22388711	6.70E-07	1.22E-05	2	0	0
SCN10A	0.5914548	0.716388183	-0.12493336	-0.28906744	6.72E-07	1.22E-05	2	0	0
KIAA1143	0.0708044	0.097516765	-0.02671236	-0.37144238	6.74E-07	1.22E-05	12	0	0
SYNE3	0.736402	0.826719023	-0.09031701	-0.17703616	6.74E-07	1.22E-05	6	0	0
HINT1	0.6669483	0.695371767	-0.0284235	-0.1467079	6.76E-07	1.22E-05	4	0	0
VN1R2	0.6438415	0.767093176	-0.12325171	-0.26172794	6.80E-07	1.23E-05	3	0	0
GRAP2	0.4526944	0.605632018	-0.15293766	-0.42138486	6.83E-07	1.23E-05	4	0	0
PIK3R6	0.3729683	0.437448585	-0.06448028	-0.27389315	6.89E-07	1.24E-05	8	0	0
FCRL5	0.6292182	0.783968106	-0.15474988	-0.31513676	7.03E-07	1.26E-05	2	0	0
RNF133	0.779247	0.85608646	-0.07683942	-0.14248155	7.02E-07	1.26E-05	5	0	0
SULF1	0.2248632	0.285303872	-0.06044069	-0.40752231	7.02E-07	1.26E-05	8	0	0
TRPM6	0.3520293	0.437840672	-0.08581134	-0.25280405	7.03E-07	1.26E-05	9	0	0
CEACAM8	0.7403577	0.809830176	-0.06947244	-0.14813196	7.02E-07	1.26E-05	4	0	0
CARD8	0.6510658	0.777670487	-0.12660467	-0.25198682	7.04E-07	1.26E-05	3	0	0
SIRPD	0.7132416	0.87127358	-0.15803196	-0.28487282	7.05E-07	1.26E-05	2	0	0
TAAR9	0.7218422	0.819739547	-0.09789731	-0.18253493	7.12E-07	1.27E-05	5	0	0
MRGPRX1	0.7363069	0.843683879	-0.10737695	-0.19393286	7.15E-07	1.28E-05	1	0	0
S100A7A	0.5538208	0.65858408	-0.10476328	-0.25629371	7.21E-07	1.29E-05	4	0	0
UTF1	0.2665442	0.163062503	0.103481689	1.101308117	7.30E-07	1.30E-05	7	0	0
NLRP9	0.6939453	0.809704469	-0.11575916	-0.22757279	7.29E-07	1.30E-05	5	0	0
OR52B2	0.5447186	0.657440153	-0.11272157	-0.23751758	7.31E-07	1.30E-05	5	0	0
VAV1	0.3474552	0.447908457	-0.10045326	-0.35522746	7.37E-07	1.31E-05	7	0	0
ZDHHC21	0.4010407	0.426319936	-0.02527926	-0.3509138	7.39E-07	1.31E-05	10	0	0
C9orf129	0.4443862	0.295880954	0.148505228	0.979632234	7.41E-07	1.31E-05	6	0	0
IL32	0.5452904	0.467115378	0.078175005	0.258707891	7.41E-07	1.31E-05	9	0	0
ESPNL	0.6686783	0.752336878	-0.08365856	-0.18543809	7.57E-07	1.34E-05	9	0	0
NOS1	0.6538583	0.743673476	-0.08981515	-0.17331991	7.63E-07	1.35E-05	7	0	0
WISP3	0.6720114	0.767940924	-0.09592953	-0.20499215	7.66E-07	1.35E-05	10	0	0
NLRP11	0.5781694	0.682287518	-0.10411814	-0.25718759	7.68E-07	1.36E-05	8	0	0
DIO2	0.7020705	0.795608995	-0.09353845	-0.17757649	7.72E-07	1.36E-05	2	0	0

FCRLA	0.55886	0.695259003	-0.13639897	-0.35171204	7.73E-07	1.36E-05	4	0	0
DARS	0.2817332	0.324868034	-0.0431348	-0.33289249	7.83E-07	1.38E-05	7	0	0
PIP	0.5681003	0.677845574	-0.10974528	-0.26399732	7.87E-07	1.38E-05	4	0	0
UBE2D2	0.6617267	0.760497981	-0.0987713	-0.21456569	7.92E-07	1.39E-05	3	0	0
LMOD2	0.6732168	0.780255664	-0.10703889	-0.21347725	7.94E-07	1.39E-05	5	0	0
ABHD5	0.0579015	0.073878316	-0.01597678	-0.20773425	8.04E-07	1.41E-05	4	0	0
A1CF	0.6941439	0.791096591	-0.09695266	-0.19043228	8.07E-07	1.41E-05	4	0	0
GRIA1	0.5618611	0.70219055	-0.14032943	-0.32581576	8.09E-07	1.42E-05	4	0	0
SLC13A1	0.760866	0.843169211	-0.08230319	-0.14868066	8.12E-07	1.42E-05	3	0	0
RARRES2	0.621526	0.558520978	0.063005043	0.395764668	8.14E-07	1.42E-05	8	0	0
SH2D3A	0.1128523	0.150293273	-0.03744096	-0.24980309	8.15E-07	1.42E-05	2	0	0
ARL14EPL	0.8259769	0.889565016	-0.06358811	-0.10581841	8.18E-07	1.43E-05	2	0	0
PDHA2	0.836922	0.903588832	-0.06666686	-0.11105528	8.21E-07	1.43E-05	10	0	0
TCL1B	0.583998	0.703077227	-0.11907924	-0.28706081	8.22E-07	1.43E-05	8	0	0
ZNF285	0.227599	0.108952052	0.118646976	1.196800209	8.24E-07	1.43E-05	5	0	0
OR4F15	0.7612785	0.871687851	-0.11040932	-0.19451657	8.28E-07	1.44E-05	5	0	0
C1orf198	0.756441	0.709017432	0.04742354	0.118343677	8.31E-07	1.44E-05	5	0	0
FAM184A	0.6721517	0.76498483	-0.0928331	-0.20858331	8.31E-07	1.44E-05	7	0	0
OPRM1	0.4820579	0.62431613	-0.14225823	-0.3804825	8.33E-07	1.44E-05	4	0	0
RLN2	0.7464761	0.824205915	-0.07772981	-0.1508682	8.39E-07	1.45E-05	2	0	0
AICDA	0.7501631	0.846355462	-0.09619237	-0.17334031	8.39E-07	1.45E-05	3	0	0
ALDH3B2	0.6561019	0.747752814	-0.09165089	-0.18877385	8.41E-07	1.45E-05	2	0	0
SDR9C7	0.754225	0.846178776	-0.09195378	-0.16372576	8.42E-07	1.45E-05	5	0	0
HOXB4	0.4676308	0.331295766	0.136334999	0.523038094	8.45E-07	1.46E-05	8	0	0
MB	0.6705414	0.558501646	0.112039731	0.270539907	8.49E-07	1.46E-05	3	0	0
TCTEX1D1	0.2255444	0.091860586	0.133683789	1.268407359	8.52E-07	1.46E-05	10	0	0
H1FOO	0.7521354	0.813885041	-0.06174967	-0.11702162	8.52E-07	1.46E-05	7	0	0
ADH7	0.6797552	0.814800666	-0.13504545	-0.25962458	8.56E-07	1.47E-05	4	0	0
BLID	0.6965413	0.835688915	-0.13914764	-0.26372172	8.60E-07	1.47E-05	8	0	0
OR5H2	0.6252901	0.733327757	-0.10803763	-0.25695467	8.64E-07	1.48E-05	3	0	0
KRTAP19-7	0.7160592	0.793914139	-0.07785494	-0.15652885	8.76E-07	1.50E-05	2	0	0
C1QTNF5	0.5906441	0.686529418	-0.09588534	-0.22633295	8.82E-07	1.50E-05	6	0	0
MFRP	0.5906441	0.686529418	-0.09588534	-0.22633295	8.82E-07	1.50E-05	6	0	0
ZNF880	0.2423273	0.126778154	0.11554913	1.11784936	8.82E-07	1.50E-05	12	0	0
MYH7B	0.6560865	0.771514854	-0.1154284	-0.24463709	8.81E-07	1.50E-05	3	0	0
OR5D14	0.5144427	0.650182	-0.13573932	-0.33238621	8.84E-07	1.50E-05	3	0	0
FAM71C	0.6266698	0.742557728	-0.11588795	-0.23523005	8.92E-07	1.52E-05	7	0	0
FHL2	0.6161107	0.539045415	0.077065307	0.224387615	8.98E-07	1.53E-05	8	0	0
L3MBTL3	0.8209668	0.906175104	-0.08520833	-0.14186137	9.04E-07	1.53E-05	2	0	0
RPS2	0.1299378	0.158321947	-0.0283842	-0.20867079	9.09E-07	1.54E-05	4	0	0
SELP	0.7292491	0.745217708	-0.01596866	-0.02089578	9.18E-07	1.55E-05	7	0	0
CA10	0.3358078	0.209375989	0.126431764	0.684669965	9.19E-07	1.55E-05	15	0	0
OR2AE1	0.7782994	0.884127852	-0.10582842	-0.19049862	9.23E-07	1.56E-05	6	0	0
IFNA13	0.7493861	0.823947845	-0.0745617	-0.1453354	9.21E-07	1.56E-05	3	0	0
TGFB1I1	0.3467231	0.28128946	0.065433627	0.23661038	9.23E-07	1.56E-05	11	0	0
OR2Z1	0.7249012	0.833657641	-0.10875641	-0.20848249	9.22E-07	1.56E-05	6	0	0
LRRK1	0.130313	0.171496076	-0.04118312	-0.35271891	9.24E-07	1.56E-05	7	0	0
CDH18	0.6251375	0.774089468	-0.14895196	-0.30461822	9.47E-07	1.59E-05	4	0	0
FAM19A5	0.3271572	0.289539991	0.037617217	0.321618041	9.49E-07	1.59E-05	7	0	0
ELMSAN1	0.7525384	0.666386991	0.086151373	0.211397853	9.54E-07	1.60E-05	4	0	0
CBFA2T3	0.3103212	0.357034436	-0.04671323	-0.10222214	9.68E-07	1.62E-05	14	0	0
OR4D5	0.7212536	0.834389789	-0.11313617	-0.21145529	9.74E-07	1.63E-05	2	0	0
FCER2	0.5126892	0.59834942	-0.08566023	-0.22889626	9.90E-07	1.66E-05	9	0	0
ZNF98	0.3772864	0.27605598	0.101230422	0.485339088	1.00E-06	1.68E-05	4	0	0
PCDHGA12	0.3637276	0.222120314	0.141607287	0.798272359	1.01E-06	1.69E-05	10	0	0
NPY2R	0.163491	0.088390542	0.075100457	0.821915133	1.01E-06	1.69E-05	11	0	0
MUC7	0.7987607	0.86616179	-0.06740104	-0.11604921	1.02E-06	1.70E-05	3	0	0
STMN2	0.5027467	0.502189459	0.000557274	0.15940588	1.02E-06	1.70E-05	11	0	0
TRIM64B	0.7651199	0.876568245	-0.11144834	-0.1938122	1.02E-06	1.70E-05	1	0	0
USP33	0.4659097	0.510684548	-0.04477488	-0.0969778	1.02E-06	1.70E-05	4	0	0
KRTAP10-11	0.6441149	0.732528258	-0.0884134	-0.18819452	1.03E-06	1.70E-05	4	0	0
C1orf226	0.7332069	0.838335478	-0.10512859	-0.19432321	1.03E-06	1.71E-05	8	0	0
TRIM61	0.5092937	0.414568027	0.094725718	0.504651257	1.03E-06	1.71E-05	10	0	0

SNTG1	0.2786926	0.20854891	0.070143651	0.858775475	1.03E-06	1.71E-05	11	0	0
DNAJC9	0.7118088	0.826418507	-0.11460973	-0.21538078	1.03E-06	1.71E-05	3	0	0
IFITM3	0.1919026	0.079045842	0.112856789	1.153825053	1.03E-06	1.71E-05	2	0	0
OR5B2	0.4581622	0.567114716	-0.10895251	-0.30683966	1.04E-06	1.71E-05	4	0	0
ZNF626	0.7603102	0.877792934	-0.11748272	-0.20568421	1.04E-06	1.71E-05	3	0	0
SCML4	0.3842883	0.480965917	-0.09667761	-0.39994405	1.04E-06	1.72E-05	8	0	0
GDF10	0.336471	0.265087152	0.071383886	0.369783437	1.04E-06	1.72E-05	5	0	0
LY86	0.6816736	0.768600247	-0.08692668	-0.17801165	1.05E-06	1.73E-05	5	0	0
NEUROD6	0.5569655	0.700342296	-0.14337683	-0.32016222	1.06E-06	1.75E-05	5	0	0
MS4A13	0.7447454	0.831273814	-0.08652845	-0.16354464	1.07E-06	1.75E-05	14	0	0
LYPD6B	0.4922743	0.543070822	-0.05079648	-0.27969354	1.07E-06	1.76E-05	7	0	0
ACY3	0.3093582	0.395954649	-0.08659644	-0.34694549	1.08E-06	1.77E-05	4	0	0
PLD4	0.6781187	0.752051874	-0.07393322	-0.15631079	1.08E-06	1.77E-05	6	0	0
OR2G3	0.6112376	0.716439563	-0.105202	-0.2175972	1.09E-06	1.78E-05	4	0	0
CTNNA2	0.3488039	0.502379986	-0.15357609	-0.51401855	1.09E-06	1.78E-05	1	0	0
RAX	0.3022243	0.197635946	0.104588338	0.724325121	1.09E-06	1.78E-05	6	0	0
SMLR1	0.7229051	0.820798236	-0.09789313	-0.17994681	1.10E-06	1.79E-05	3	0	0
ZNF574	0.5393766	0.652496865	-0.11312026	-0.26429676	1.10E-06	1.79E-05	4	0	0
KALRN	0.3231684	0.438014896	-0.11484646	-0.38652435	1.10E-06	1.79E-05	2	0	0
RNASE13	0.7347542	0.798692134	-0.06393796	-0.1252491	1.11E-06	1.80E-05	5	0	0
RASGRF2	0.2974945	0.206492961	0.091001518	1.217681576	1.12E-06	1.81E-05	8	0	0
ADAMDEC1	0.6837588	0.768056303	-0.08429752	-0.17641114	1.12E-06	1.81E-05	6	0	0
FRMD1	0.832291	0.905551247	-0.07326027	-0.12472531	1.12E-06	1.82E-05	6	0	0
TOR3A	0.331191	0.396310405	-0.06511936	-0.26241223	1.12E-06	1.82E-05	10	0	0
OR51I2	0.7862403	0.876943584	-0.09070331	-0.1635615	1.13E-06	1.83E-05	3	0	0
TGM1	0.5456758	0.622210466	-0.07653466	-0.18504267	1.13E-06	1.83E-05	4	0	0
COMMD10	0.3376544	0.400774162	-0.0631198	-0.25479324	1.14E-06	1.84E-05	8	0	0
PRRX2	0.2479332	0.145075116	0.102858107	0.729758004	1.15E-06	1.85E-05	4	0	0
C3AR1	0.5021179	0.633531483	-0.1314136	-0.42197013	1.15E-06	1.85E-05	6	0	0
RAB34	0.164597	0.229443479	-0.06484652	-0.34987999	1.15E-06	1.85E-05	19	0	0
RPL23A	0.164597	0.229443479	-0.06484652	-0.34987999	1.15E-06	1.85E-05	19	0	0
GAL3ST3	0.4470376	0.337777131	0.109260432	0.580298012	1.16E-06	1.87E-05	7	0	0
PSMA6	0.7609201	0.696544644	0.064375448	0.146325081	1.16E-06	1.87E-05	3	0	0
PRB1	0.5478991	0.740585292	-0.1926862	-0.42801181	1.16E-06	1.87E-05	1	0	0
TAS2R20	0.8661977	0.927307342	-0.06110962	-0.09739994	1.17E-06	1.87E-05	3	0	0
KRTAP26-1	0.7477901	0.856005859	-0.10821573	-0.19461184	1.17E-06	1.88E-05	6	0	0
OR5AK2	0.6324025	0.77624394	-0.14384146	-0.30064684	1.17E-06	1.88E-05	2	0	0
HOXB9	0.2072595	0.161328013	0.045931475	0.133876774	1.17E-06	1.88E-05	9	0	0
SLAMF9	0.6235087	0.713136155	-0.08962745	-0.16704005	1.18E-06	1.89E-05	4	0	0
CASD1	0.2316116	0.289845728	-0.05823409	-0.26293136	1.18E-06	1.89E-05	4	0	0
GP1BB	0.571018	0.500328499	0.070689524	0.173052076	1.18E-06	1.89E-05	10	0	0
DEFB127	0.7736402	0.872000975	-0.0983608	-0.17820543	1.19E-06	1.90E-05	4	0	0
CALCB	0.5976846	0.551130421	0.046554149	0.408419889	1.20E-06	1.91E-05	5	0	0
OR52W1	0.7701804	0.853774294	-0.08359388	-0.16105979	1.21E-06	1.92E-05	6	0	0
BTF3	0.1333087	0.179067732	-0.04575907	-0.32347428	1.21E-06	1.93E-05	9	0	0
ANKRD45	0.5047707	0.407817227	0.096953446	0.714689728	1.23E-06	1.95E-05	10	0	0
SCO2	0.3248735	0.396387213	-0.07151373	-0.2024666	1.23E-06	1.95E-05	8	0	0
IL17F	0.7183998	0.816770039	-0.09837019	-0.18283567	1.23E-06	1.95E-05	4	0	0
FER1L5	0.6126116	0.549365934	0.063245639	0.256070828	1.23E-06	1.95E-05	5	0	0
TEKT5	0.7314695	0.800765948	-0.06929642	-0.14441443	1.23E-06	1.95E-05	6	0	0
OR5T1	0.4733152	0.61182621	-0.13851105	-0.35426501	1.24E-06	1.96E-05	2	0	0
OR6X1	0.7003788	0.804561338	-0.10418257	-0.21056736	1.25E-06	1.97E-05	6	0	0
VWA8	0.2385244	0.261335955	-0.02281156	-0.28612313	1.25E-06	1.97E-05	5	0	0
GJA10	0.7892503	0.886301578	-0.09705128	-0.16847613	1.25E-06	1.97E-05	5	0	0
DCAF4L2	0.8391798	0.927003626	-0.08782379	-0.14774248	1.25E-06	1.98E-05	11	0	0
AIM2	0.5540686	0.701922498	-0.1478539	-0.33584958	1.26E-06	1.99E-05	1	0	0
DSG1	0.6835948	0.698168386	-0.01457362	0.035611426	1.27E-06	1.99E-05	8	0	0
RFPL4A	0.4258903	0.568271601	-0.14238134	-0.43740007	1.28E-06	2.01E-05	2	0	0
OR6B2	0.501788	0.592082096	-0.09029405	-0.23728271	1.28E-06	2.01E-05	5	0	0
NXPE1	0.7470405	0.834049425	-0.0870089	-0.17223658	1.28E-06	2.01E-05	5	0	0
MRAS	0.6086361	0.719084867	-0.11044873	-0.22878057	1.28E-06	2.01E-05	5	0	0
MPL	0.7589187	0.846302485	-0.08738374	-0.18187926	1.29E-06	2.02E-05	2	0	0
KRBOX1	0.7248722	0.794526132	-0.06965398	-0.13641964	1.29E-06	2.02E-05	6	0	0

OR10V1	0.6998457	0.815375349	-0.11552964	-0.22405718	1.29E-06	2.02E-05	4	0	0
HAO1	0.6981342	0.801398043	-0.10326385	-0.20232209	1.29E-06	2.02E-05	5	0	0
ERICH1	0.1855458	0.101362383	0.084183375	1.134683719	1.31E-06	2.04E-05	7	0	0
CD5	0.5152741	0.618771854	-0.10349777	-0.27847883	1.31E-06	2.05E-05	6	0	0
HBG1	0.7718734	0.863243819	-0.09137042	-0.15900676	1.32E-06	2.05E-05	2	0	0
DEFB103A	0.4058702	0.570202085	-0.16433188	-0.48042216	1.32E-06	2.06E-05	1	0	0
PGR	0.7052646	0.830919164	-0.12565452	-0.23826857	1.33E-06	2.07E-05	5	0	0
KRT17	0.4353214	0.510479646	-0.07515824	-0.24922834	1.34E-06	2.08E-05	5	0	0
OIT3	0.6136081	0.523568581	0.090039474	0.22962585	1.34E-06	2.08E-05	5	0	0
FOLR1	0.7283961	0.672319275	0.056076822	0.127404136	1.35E-06	2.09E-05	4	0	0
CLCA2	0.7723228	0.84778249	-0.07545969	-0.13828167	1.36E-06	2.11E-05	4	0	0
LGALS12	0.5320091	0.584320323	-0.05231117	-0.1425638	1.37E-06	2.12E-05	9	0	0
GPR78	0.7625806	0.838511698	-0.07593114	-0.13516606	1.37E-06	2.12E-05	2	0	0
CALN1	0.6315288	0.725246698	-0.09371788	-0.18696019	1.37E-06	2.12E-05	5	0	0
CLEC6A	0.5925792	0.720251105	-0.12767192	-0.29420166	1.37E-06	2.12E-05	2	0	0
CLEC7A	0.6273117	0.731637121	-0.10432545	-0.23626427	1.37E-06	2.12E-05	5	0	0
ACPP	0.3633389	0.452309039	-0.08897012	-0.31874303	1.37E-06	2.12E-05	5	0	0
PRTN3	0.3855257	0.464837421	-0.07931169	-0.2607227	1.38E-06	2.13E-05	6	0	0
SLC34A2	0.2407515	0.129016893	0.111734599	1.203837843	1.38E-06	2.13E-05	8	0	0
FTMT	0.8682732	0.926247511	-0.05797432	-0.09446532	1.39E-06	2.13E-05	9	0	0
BOLL	0.3959843	0.292566224	0.103418116	0.953251259	1.40E-06	2.16E-05	14	0	0
OR10G7	0.6209285	0.720045754	-0.0991173	-0.23274662	1.41E-06	2.17E-05	3	0	0
MYO6	0.3074572	0.405628357	-0.09817117	-0.32068935	1.42E-06	2.18E-05	3	0	0
OR7D2	0.6668221	0.766430101	-0.09960799	-0.20489647	1.43E-06	2.18E-05	2	0	0
DEFB125	0.5343117	0.687216875	-0.15290515	-0.35701829	1.43E-06	2.18E-05	2	0	0
C1orf158	0.5704782	0.675803751	-0.10532557	-0.25238095	1.43E-06	2.18E-05	6	0	0
ONECUT1	0.2605485	0.162931842	0.097616623	0.920317918	1.43E-06	2.18E-05	8	0	0
NLRP13	0.609456	0.759610401	-0.15015435	-0.31265916	1.43E-06	2.18E-05	3	0	0
ASCC2	0.0574218	0.094627022	-0.0372052	-0.38316915	1.43E-06	2.19E-05	6	0	0
OR7G2	0.5701686	0.698018753	-0.12785012	-0.28523266	1.44E-06	2.19E-05	4	0	0
IL10	0.6417319	0.752036773	-0.11030492	-0.23847194	1.45E-06	2.20E-05	7	0	0
ZNF582	0.2782582	0.15876532	0.119492836	1.298292588	1.45E-06	2.20E-05	14	0	0
ERVFRD-1	0.6366017	0.569721775	0.066879934	0.199029092	1.45E-06	2.20E-05	4	0	0
TMEM95	0.7216145	0.815935489	-0.09432097	-0.17846241	1.45E-06	2.21E-05	4	0	0
OR2Y1	0.6582687	0.75821722	-0.09994851	-0.20642083	1.47E-06	2.23E-05	5	0	0
SERPINB3	0.5930061	0.711976715	-0.11897062	-0.32642237	1.48E-06	2.24E-05	2	0	0
BCKDHA	0.7586637	0.809270489	-0.05060682	-0.10611866	1.48E-06	2.25E-05	2	0	0
NAGK	0.5747174	0.67579417	-0.1010768	-0.25671742	1.49E-06	2.26E-05	3	0	0
MTIF2	0.2481003	0.314741349	-0.0666411	-0.28535888	1.51E-06	2.29E-05	8	0	0
TMPRSS11F	0.6599134	0.765027409	-0.10511404	-0.21270125	1.54E-06	2.32E-05	4	0	0
PITPNM2	0.410155	0.339332761	0.070822256	0.162369016	1.54E-06	2.32E-05	5	0	0
KCNJ15	0.6854363	0.809459022	-0.12402268	-0.23671341	1.54E-06	2.33E-05	2	0	0
OR13D1	0.6668708	0.783510683	-0.11663987	-0.22957544	1.55E-06	2.34E-05	3	0	0
BRINP1	0.2862069	0.179150693	0.107056251	0.719685394	1.55E-06	2.34E-05	5	0	0
C19orf81	0.3846058	0.511711215	-0.12710539	-0.36998662	1.56E-06	2.35E-05	5	0	0
KCNIP4	0.2597348	0.155589681	0.104145149	0.856167806	1.58E-06	2.38E-05	10	0	0
KRTAP20-1	0.6267988	0.720978275	-0.0941795	-0.22023288	1.58E-06	2.38E-05	4	0	0
ALDH3B1	0.6112187	0.671690251	-0.06047157	-0.15637128	1.59E-06	2.38E-05	10	0	0
FAM216B	0.6120475	0.632777115	-0.02072962	-0.09380156	1.59E-06	2.39E-05	6	0	0
MARCO	0.5416688	0.632278915	-0.09061015	-0.27355238	1.60E-06	2.39E-05	8	0	0
TMPRSS4	0.5299539	0.656924212	-0.12697029	-0.30153801	1.60E-06	2.39E-05	4	0	0
ADAM7	0.6846751	0.786251035	-0.10157593	-0.20256322	1.61E-06	2.40E-05	7	0	0
RNASE12	0.7143336	0.831323724	-0.11699015	-0.2157271	1.61E-06	2.40E-05	8	0	0
XKR3	0.7049933	0.85077443	-0.14578114	-0.26770551	1.61E-06	2.40E-05	1	0	0
OR1L8	0.6868793	0.777737852	-0.09085851	-0.17930886	1.61E-06	2.41E-05	4	0	0
FAM71B	0.6345986	0.750469805	-0.1158712	-0.26306576	1.61E-06	2.41E-05	8	0	0
CEACAM21	0.3735383	0.433865994	-0.06032773	0.101982902	1.64E-06	2.44E-05	5	0	0
IL1RL1	0.7458713	0.847308367	-0.10143702	-0.18705723	1.65E-06	2.45E-05	3	0	0
IL18R1	0.7458713	0.847308367	-0.10143702	-0.18705723	1.65E-06	2.45E-05	3	0	0
ABCG2	0.5917805	0.504578307	0.087202188	0.261908973	1.65E-06	2.45E-05	8	0	0
MMP1	0.7223653	0.786794844	-0.06442959	-0.13657418	1.66E-06	2.47E-05	3	0	0
PMFBP1	0.7570166	0.869101775	-0.11208522	-0.2002068	1.66E-06	2.47E-05	2	0	0
IFNA14	0.7025853	0.810684426	-0.10809913	-0.21976484	1.67E-06	2.47E-05	4	0	0

WDFY4	0.4960523	0.581103853	-0.08505157	-0.24966868	1.68E-06	2.48E-05	12	0	0
EMP3	0.2685271	0.322110576	-0.05358345	-0.34395215	1.68E-06	2.48E-05	10	0	0
SORCS1	0.3581159	0.29070141	0.067414491	0.767067594	1.68E-06	2.49E-05	10	0	0
GPX2	0.5453444	0.668202577	-0.12285821	-0.30149743	1.69E-06	2.50E-05	7	0	0
APOA1	0.7553244	0.805644305	-0.05031986	-0.0950855	1.70E-06	2.50E-05	9	0	0
BANF2	0.6783971	0.788559145	-0.11016209	-0.25766451	1.72E-06	2.54E-05	7	0	0
WDR35	0.0828184	0.097177533	-0.01435913	-0.27046535	1.73E-06	2.55E-05	14	0	0
DEFB114	0.5761905	0.749649023	-0.17345854	-0.37396346	1.74E-06	2.56E-05	1	0	0
CCDC36	0.7535871	0.821919603	-0.06833247	-0.13114753	1.75E-06	2.57E-05	7	0	0
IFNA1	0.8433163	0.884527725	-0.04121147	-0.06804566	1.75E-06	2.57E-05	1	0	0
PPBP	0.6972646	0.73529257	-0.038028	-0.07180381	1.75E-06	2.57E-05	6	0	0
ACTBL2	0.6792672	0.769524915	-0.09025773	-0.19237308	1.76E-06	2.59E-05	7	0	0
AP1S1	0.3237672	0.365228236	-0.04146103	-0.21383041	1.77E-06	2.59E-05	10	0	0
C9orf92	0.7238743	0.811015264	-0.08714093	-0.15893727	1.78E-06	2.60E-05	5	0	0
CCL16	0.6601056	0.742791349	-0.0826858	-0.17209608	1.78E-06	2.60E-05	9	0	0
OR13A1	0.7562875	0.857591769	-0.10130428	-0.1815441	1.78E-06	2.60E-05	4	0	0
ACMSD	0.5910177	0.729840286	-0.13882262	-0.30682324	1.79E-06	2.62E-05	3	0	0
ZSCAN23	0.2961715	0.234996435	0.06117507	1.005061112	1.80E-06	2.62E-05	9	0	0
HRH4	0.7088579	0.796117719	-0.08725981	-0.17068284	1.80E-06	2.62E-05	5	0	0
FPR2	0.5423327	0.685238281	-0.14290554	-0.33252361	1.80E-06	2.63E-05	2	0	0
FBLIM1	0.3954181	0.459836089	-0.06441794	-0.29025129	1.81E-06	2.64E-05	4	0	0
SLC22A8	0.4865964	0.474468542	0.012127822	0.108988707	1.82E-06	2.65E-05	7	0	0
RXFP3	0.1589692	0.072443604	0.086525578	1.079003519	1.85E-06	2.68E-05	12	0	0
COL9A1	0.5946389	0.694735029	-0.10009617	-0.25386355	1.85E-06	2.68E-05	7	0	0
TUSC1	0.5790094	0.636144433	-0.057135	-0.09502059	1.85E-06	2.69E-05	4	0	0
EMILIN1	0.5770719	0.512128977	0.064942924	0.196764592	1.86E-06	2.69E-05	10	0	0
FAM181A	0.7541149	0.840385435	-0.08627053	-0.15777408	1.86E-06	2.70E-05	5	0	0
EBI3	0.1776913	0.237657967	-0.05996671	-0.23707348	1.88E-06	2.72E-05	3	0	0
GALNT15	0.4645861	0.42204375	0.042542322	0.216051135	1.88E-06	2.72E-05	10	0	0
OR8K1	0.6268688	0.746158041	-0.11928924	-0.26200834	1.89E-06	2.73E-05	4	0	0
PAPLN	0.3138806	0.373168647	-0.059288	-0.25277132	1.89E-06	2.73E-05	9	0	0
CC2D1A	0.2143333	0.144536335	0.069796962	0.381565292	1.89E-06	2.73E-05	6	0	0
HIST1H4F	0.5665481	0.491621224	0.074926839	0.782802513	1.90E-06	2.75E-05	8	0	0
OVCH2	0.3100834	0.419894056	-0.10981066	-0.44294363	1.91E-06	2.75E-05	3	0	0
CBFB	0.084622	0.126974979	-0.04235297	-0.19911124	1.92E-06	2.77E-05	11	0	0
ADAM2	0.7981973	0.861931656	-0.06373436	-0.11009809	1.94E-06	2.78E-05	7	0	0
OR51V1	0.5811875	0.695948614	-0.11476116	-0.2475467	1.94E-06	2.78E-05	3	0	0
COX6A2	0.6325427	0.623679034	0.008863673	0.003171678	1.94E-06	2.78E-05	5	0	0
TRAPPC12	0.2991403	0.343545832	-0.04440556	-0.27983361	1.95E-06	2.79E-05	8	0	0
PPP2R2B	0.6783857	0.817406681	-0.13902097	-0.26537945	1.95E-06	2.79E-05	1	0	0
OR10S1	0.5869472	0.728841259	-0.14189401	-0.31115747	1.95E-06	2.79E-05	2	0	0
RNASE2	0.4448432	0.58667762	-0.14183441	-0.39158236	1.95E-06	2.80E-05	1	0	0
GPR65	0.3852424	0.492254861	-0.10701245	-0.2845629	1.97E-06	2.81E-05	2	0	0
HDGFL1	0.820681	0.836390764	-0.01570978	-0.02571831	1.97E-06	2.82E-05	7	0	0
FCGR3A	0.5818663	0.707759843	-0.12589359	-0.27823001	1.97E-06	2.82E-05	1	0	0
CUBN	0.7928529	0.890352737	-0.09749988	-0.17616747	1.98E-06	2.83E-05	3	0	0
ALX1	0.2376284	0.113501272	0.124127136	1.121352979	1.99E-06	2.83E-05	13	0	0
NCKAP1L	0.5081234	0.637578413	-0.12945502	-0.32669997	1.99E-06	2.84E-05	5	0	0
ZNF90	0.2834494	0.116162921	0.167286465	1.217823722	2.00E-06	2.84E-05	1	0	0
DES	0.542643	0.459368193	0.083274817	0.359367402	2.01E-06	2.85E-05	9	0	0
OLIG3	0.2651244	0.181839012	0.08328537	0.53923853	2.01E-06	2.85E-05	9	0	0
OR9Q1	0.7096289	0.747858829	-0.03822992	-0.05334979	2.01E-06	2.85E-05	7	0	0
OR1C1	0.5333064	0.659108761	-0.12580241	-0.28713886	2.01E-06	2.86E-05	6	0	0
MAGI2	0.1853251	0.103439549	0.081885504	0.779207145	2.02E-06	2.86E-05	15	0	0
CETP	0.6439633	0.563254601	0.080708658	0.190352846	2.02E-06	2.86E-05	7	0	0
WFDC9	0.7205094	0.829555329	-0.10904588	-0.20121118	2.02E-06	2.86E-05	5	0	0
DNALI1	0.4577172	0.360889962	0.096827255	0.507794338	2.02E-06	2.86E-05	12	0	0
S100A10	0.2374453	0.30282879	-0.0653835	-0.31010658	2.02E-06	2.86E-05	10	0	0
ADCY2	0.2337927	0.164434112	0.069358559	0.527533907	2.05E-06	2.89E-05	6	0	0
PLA2G2F	0.7265811	0.785661839	-0.05908072	-0.12326482	2.05E-06	2.90E-05	7	0	0
GALR2	0.3311331	0.226826026	0.104307065	0.910235882	2.06E-06	2.90E-05	7	0	0
SSH3	0.2067317	0.152249366	0.054482305	0.576909022	2.06E-06	2.91E-05	13	0	0
TNFRSF10A	0.1662682	0.230388723	-0.0641205	-0.26069691	2.07E-06	2.91E-05	12	0	0

CLC	0.516806	0.641429384	-0.12462341	-0.33105307	2.07E-06	2.91E-05	4	0	0
GKN1	0.7934996	0.879043931	-0.08554435	-0.1497362	2.07E-06	2.91E-05	2	0	0
OR10A4	0.620288	0.725556184	-0.10526817	-0.22019465	2.08E-06	2.91E-05	3	0	0
UNC13D	0.1948402	0.223390867	-0.0285507	-0.29786939	2.09E-06	2.93E-05	10	0	0
XBP1	0.3146731	0.4184286	-0.10375546	-0.3406404	2.09E-06	2.94E-05	8	0	0
HOXC6	0.208576	0.095809396	0.112766561	1.061083037	2.10E-06	2.94E-05	8	0	0
FCRL6	0.5838743	0.684468263	-0.10059398	-0.23284098	2.12E-06	2.97E-05	6	0	0
SECISBP2L	0.2244669	0.261628966	-0.0371621	-0.32630102	2.13E-06	2.98E-05	13	0	0
PRSS56	0.629492	0.569821452	0.05967055	0.147110513	2.14E-06	2.98E-05	5	0	0
HRH1	0.6073143	0.706517737	-0.0992034	-0.22176595	2.14E-06	2.98E-05	10	0	0
TCERG1L	0.2307606	0.185472087	0.045288495	0.744648644	2.14E-06	2.98E-05	5	0	0
KRT28	0.5808752	0.668799183	-0.08792394	-0.20169349	2.15E-06	3.00E-05	4	0	0
PPM1N	0.7019307	0.80270515	-0.10077442	-0.19343438	2.15E-06	3.00E-05	4	0	0
PROX1	0.2259326	0.126522967	0.099409593	0.862345079	2.17E-06	3.02E-05	4	0	0
C15orf40	0.2717469	0.343244423	-0.07149749	-0.27730539	2.17E-06	3.02E-05	9	0	0
RIT2	0.6337891	0.759671216	-0.12588213	-0.26024013	2.17E-06	3.02E-05	4	0	0
SFT2D3	0.486783	0.576031579	-0.08924859	-0.2250664	2.18E-06	3.03E-05	12	0	0
CD4	0.7869611	0.737669042	0.049292076	0.1004726	2.18E-06	3.03E-05	3	0	0
LPAR2	0.3209499	0.382659875	-0.06171001	-0.22692316	2.20E-06	3.05E-05	8	0	0
SLC7A14	0.3903598	0.375195327	0.015164476	0.244246329	2.21E-06	3.06E-05	8	0	0
F11	0.7603599	0.825680656	-0.06532078	-0.12214384	2.21E-06	3.06E-05	8	0	0
EDDM3B	0.6004131	0.716949205	-0.11653612	-0.25206866	2.21E-06	3.06E-05	1	0	0
RFX8	0.231832	0.310799777	-0.07896776	-0.27253285	2.22E-06	3.06E-05	12	0	0
FGF3	0.196315	0.110758859	0.085556163	0.907606943	2.24E-06	3.09E-05	14	0	0
FAM155A	0.1471376	0.069626984	0.077510583	1.060065668	2.23E-06	3.09E-05	12	0	0
MIER3	0.7601676	0.848049725	-0.08788216	-0.15684628	2.24E-06	3.09E-05	2	0	0
MYH4	0.693424	0.804869694	-0.1114457	-0.22944576	2.24E-06	3.09E-05	6	0	0
PLD1	0.3577094	0.45343835	-0.09572894	-0.22341691	2.25E-06	3.09E-05	6	0	0
ACAN	0.3183889	0.293616027	0.024772873	0.577144289	2.25E-06	3.09E-05	9	0	0
PECR	0.6141742	0.725762137	-0.11158797	-0.25062951	2.26E-06	3.10E-05	4	0	0
DCLK3	0.7910497	0.856355643	-0.06530597	-0.11588983	2.28E-06	3.13E-05	3	0	0
IRX1	0.2885062	0.192355945	0.096150249	0.539118349	2.28E-06	3.13E-05	5	0	0
CACNA2D4	0.615686	0.711949884	-0.09626391	-0.2158813	2.28E-06	3.13E-05	7	0	0
TTC22	0.0960879	0.126865782	-0.03077793	-0.22008515	2.29E-06	3.14E-05	9	0	0
TMEM215	0.1541127	0.090620838	0.063491829	0.752680264	2.30E-06	3.16E-05	7	0	0
DNAJB13	0.7314621	0.805599058	-0.07413696	-0.18808385	2.31E-06	3.16E-05	3	0	0
PAX4	0.5098164	0.608129761	-0.09831333	-0.214428	2.32E-06	3.17E-05	9	0	0
TRIM77	0.6940174	0.801031657	-0.1070143	-0.20415034	2.32E-06	3.17E-05	2	0	0
ABHD12B	0.4633855	0.449449082	0.013936395	0.211904347	2.32E-06	3.17E-05	7	0	0
MKRN3	0.6771109	0.820574485	-0.14346361	-0.27843482	2.33E-06	3.17E-05	10	0	0
ADSSL1	0.2528424	0.297181056	-0.0443387	-0.042296	2.33E-06	3.18E-05	7	0	0
TGM5	0.5633472	0.666229207	-0.10288196	-0.23586752	2.33E-06	3.18E-05	6	0	0
FTH1	0.0682312	0.103543312	-0.03531211	-0.2707798	2.34E-06	3.18E-05	8	0	0
RHNO1	0.4271583	0.524091722	-0.09693346	-0.26733787	2.34E-06	3.18E-05	4	0	0
ARHGAP32	0.0892704	0.147456202	-0.0581858	-0.31846104	2.35E-06	3.19E-05	6	0	0
SCNN1D	0.7954519	0.870424659	-0.07497271	-0.12794424	2.35E-06	3.19E-05	8	0	0
TNS4	0.6037827	0.682700228	-0.07891752	-0.19252505	2.35E-06	3.19E-05	10	0	0
ABCG1	0.7591761	0.838835219	-0.07965914	-0.14760413	2.37E-06	3.22E-05	3	0	0
FAM69B	0.1087966	0.158202563	-0.04940601	-0.13978513	2.39E-06	3.24E-05	5	0	0
TRIM34	0.7796187	0.853273657	-0.07365492	-0.13716631	2.40E-06	3.24E-05	4	0	0
ENTPD2	0.4314834	0.488711346	-0.05722799	-0.27497056	2.40E-06	3.25E-05	5	0	0
KRTAP21-3	0.744168	0.84100801	-0.09684005	-0.18854509	2.40E-06	3.25E-05	4	0	0
ZIC5	0.2817256	0.189577038	0.092148524	0.672210096	2.43E-06	3.28E-05	10	0	0
PODN	0.1633598	0.09431396	0.069045814	0.809088763	2.44E-06	3.29E-05	9	0	0
UBXN2B	0.2986212	0.315828652	-0.01720741	-0.29266327	2.44E-06	3.29E-05	7	0	0
IFIT1B	0.8167619	0.871863011	-0.05510108	-0.09546307	2.44E-06	3.29E-05	4	0	0
NPSR1	0.6051219	0.710745314	-0.10562343	-0.23234515	2.45E-06	3.29E-05	7	0	0
OR52N2	0.6376486	0.746465919	-0.10881734	-0.23414211	2.45E-06	3.30E-05	5	0	0
DLGAP2	0.677598	0.771274475	-0.09367643	-0.18412177	2.46E-06	3.31E-05	10	0	0
RGL3	0.1933775	0.088798833	0.10457863	1.124772439	2.47E-06	3.31E-05	9	0	0
OR51L1	0.5786424	0.690607045	-0.11196461	-0.26661332	2.47E-06	3.32E-05	7	0	0
NKX2-4	0.2758075	0.171112251	0.104695241	0.718675363	2.50E-06	3.35E-05	11	0	0
TNNI1	0.6953442	0.784181008	-0.08883678	-0.17114035	2.50E-06	3.35E-05	1	0	0

HLA-DRA	0.3582349	0.417445365	-0.05921051	-0.38078253	2.51E-06	3.36E-05	3	0	0
ATP13A5	0.6870759	0.810741293	-0.12366537	-0.26385512	2.51E-06	3.36E-05	8	0	0
OR4B1	0.6968541	0.805270436	-0.10841633	-0.21011828	2.52E-06	3.37E-05	3	0	0
PTPN22	0.4870884	0.62985688	-0.1427685	-0.40239998	2.57E-06	3.43E-05	6	0	0
NID2	0.2701493	0.256819065	0.013330193	0.526348848	2.57E-06	3.43E-05	13	0	0
CLUL1	0.2231924	0.278913346	-0.05572095	-0.2893388	2.57E-06	3.44E-05	7	0	0
ATP2C1	0.1415995	0.196508766	-0.05490926	-0.36805538	2.59E-06	3.45E-05	9	0	0
SIGLEC8	0.4933529	0.59772889	-0.10437601	-0.28518435	2.59E-06	3.46E-05	3	0	0
CCDC74A	0.7327064	0.844628129	-0.11192176	-0.19755204	2.62E-06	3.48E-05	2	0	0
MEF2C	0.6699868	0.794678147	-0.12469131	-0.24290503	2.62E-06	3.49E-05	1	0	0
TNFRSF13B	0.6948335	0.762200819	-0.06736736	-0.13335273	2.62E-06	3.49E-05	4	0	0
C20orf202	0.6193654	0.663317924	-0.04395254	-0.01019835	2.64E-06	3.50E-05	5	0	0
CCDC34	0.4286647	0.514826936	-0.08616227	-0.27074312	2.64E-06	3.50E-05	7	0	0
RPS5	0.3702256	0.429474188	-0.05924857	-0.04183713	2.64E-06	3.50E-05	3	0	0
BHMT	0.5908382	0.532273146	0.058565048	0.27742294	2.65E-06	3.51E-05	12	0	0
PARP16	0.6765031	0.796275564	-0.11977242	-0.2319897	2.65E-06	3.51E-05	2	0	0
EPPIN-WFD(	0.6066396	0.736746595	-0.13010699	-0.27660435	2.66E-06	3.52E-05	5	0	0
EPPIN	0.6066396	0.736746595	-0.13010699	-0.27660435	2.66E-06	3.52E-05	5	0	0
SEMG2	0.797626	0.8895134	-0.09188737	-0.1566111	2.67E-06	3.52E-05	5	0	0
CCDC68	0.3349098	0.255363508	0.079546331	0.81977101	2.68E-06	3.54E-05	9	0	0
SLURP1	0.7229706	0.809281095	-0.0863105	-0.17822461	2.69E-06	3.54E-05	6	0	0
SFTPD	0.4705276	0.408347324	0.06218029	0.199918132	2.68E-06	3.54E-05	1	0	0
F2RL3	0.2842254	0.179174242	0.105051204	1.08820831	2.69E-06	3.55E-05	10	0	0
PRSS37	0.8231095	0.877023845	-0.05391434	-0.09047951	2.70E-06	3.55E-05	3	0	0
KRT27	0.7481172	0.836723842	-0.08860667	-0.16523395	2.71E-06	3.57E-05	8	0	0
PPP1R17	0.6222158	0.711871606	-0.08965577	-0.18014869	2.71E-06	3.57E-05	9	0	0
SOGA3	0.1889941	0.118045553	0.070948507	0.686057709	2.72E-06	3.57E-05	14	0	0
DSG3	0.6110271	0.736271417	-0.12524428	-0.29361586	2.72E-06	3.57E-05	8	0	0
ZNF667	0.2995571	0.189852031	0.109705109	0.931429191	2.73E-06	3.58E-05	5	0	0
OR51A2	0.6143737	0.748983962	-0.13461022	-0.28262628	2.74E-06	3.59E-05	3	0	0
OR4D2	0.5272231	0.650630609	-0.12340748	-0.28666582	2.75E-06	3.60E-05	5	0	0
TMEM240	0.5928003	0.507268025	0.085532269	0.862246013	2.75E-06	3.60E-05	12	0	0
RXFP4	0.5083396	0.421600172	0.08673946	0.247534647	2.76E-06	3.60E-05	6	0	0
CD8A	0.1874606	0.158732715	0.028727891	0.089090927	2.78E-06	3.62E-05	10	0	0
SPON2	0.771567	0.731836517	0.039730458	0.087955109	2.78E-06	3.62E-05	20	0	0
AHR	0.294046	0.314086065	-0.02004011	-0.12122593	2.77E-06	3.62E-05	8	0	0
SUN3	0.7328233	0.826131887	-0.09330864	-0.1820884	2.79E-06	3.64E-05	9	0	0
SEMG1	0.5007652	0.650741303	-0.14997611	-0.39525956	2.80E-06	3.64E-05	2	0	0
OR51Q1	0.5739097	0.696595724	-0.12268606	-0.2679263	2.83E-06	3.68E-05	4	0	0
RRH	0.8238508	0.803370512	0.020480332	0.03244447	2.84E-06	3.70E-05	5	0	0
FEZF2	0.1685027	0.101281622	0.067221048	0.680410092	2.86E-06	3.71E-05	11	0	0
CNGB3	0.4635448	0.583404801	-0.11986001	-0.30933281	2.88E-06	3.73E-05	5	0	0
SLC39A12	0.6181558	0.738338623	-0.12018286	-0.21923991	2.88E-06	3.73E-05	6	0	0
IGDCC3	0.389298	0.309340343	0.079957691	0.504151677	2.88E-06	3.73E-05	4	0	0
IFNA6	0.6115322	0.709289679	-0.09775744	-0.21074368	2.89E-06	3.74E-05	1	0	0
NNMT	0.5745947	0.59758623	-0.0229915	-0.02390647	2.89E-06	3.74E-05	5	0	0
PHLPP2	0.6895489	0.729751439	-0.04020257	-0.15121329	2.89E-06	3.74E-05	7	0	0
OR2W1	0.4725081	0.616512882	-0.14400475	-0.37649817	2.90E-06	3.76E-05	4	0	0
HES3	0.2559635	0.182409314	0.0735542	0.54326802	2.92E-06	3.77E-05	11	0	0
SLC6A3	0.2877719	0.189905264	0.097866629	0.881572382	2.95E-06	3.81E-05	7	0	0
ATP12A	0.1526257	0.191673872	-0.03904814	-0.16094319	2.96E-06	3.81E-05	10	0	0
SCNM1	0.3847067	0.450393098	-0.06568643	-0.26183025	2.96E-06	3.82E-05	5	0	0
LGALS2	0.6517916	0.742547677	-0.09075607	-0.18540599	2.96E-06	3.82E-05	1	0	0
CALML5	0.6996428	0.763358945	-0.06371619	-0.13294151	2.97E-06	3.83E-05	7	0	0
RNASE1	0.641662	0.567900619	0.073761427	0.208814345	2.99E-06	3.84E-05	10	0	0
FA2H	0.1625077	0.201518971	-0.03901124	-0.1511553	2.99E-06	3.84E-05	6	0	0
CACNG8	0.2664913	0.165434793	0.10105655	0.843202583	2.99E-06	3.84E-05	9	0	0
CC2D2A	0.2685677	0.215612303	0.052955368	0.667735864	2.99E-06	3.84E-05	12	0	0
THRB	0.1751885	0.219194948	-0.04400643	-0.32599406	3.01E-06	3.86E-05	19	0	0
AOC1	0.7548125	0.843098151	-0.08828566	-0.15810736	3.02E-06	3.87E-05	2	0	0
IL31RA	0.7485609	0.825868464	-0.07730756	-0.14211458	3.04E-06	3.89E-05	5	0	0
HMHB1	0.6655514	0.745971386	-0.08041998	-0.16072747	3.04E-06	3.89E-05	5	0	0
KRT26	0.6544832	0.744176794	-0.08969359	-0.19907446	3.04E-06	3.89E-05	7	0	0

TRIML2	0.6274124	0.746756439	-0.11934406	-0.24760201	3.05E-06	3.90E-05	1	0	0
GPR87	0.6359651	0.757967853	-0.12200279	-0.27598549	3.07E-06	3.91E-05	5	0	0
MSH6	0.802197	0.886595754	-0.08439873	-0.14262793	3.07E-06	3.92E-05	1	0	0
KIF15	0.1250358	0.146908772	-0.02187297	-0.32715594	3.10E-06	3.95E-05	13	0	0
KCNH5	0.7364953	0.81972426	-0.08322893	-0.15611864	3.10E-06	3.95E-05	6	0	0
ASB10	0.5571297	0.631919286	-0.07478964	-0.18978344	3.10E-06	3.95E-05	14	0	0
OSGIN1	0.8204915	0.878619876	-0.0581284	-0.10515821	3.11E-06	3.95E-05	4	0	0
SOX14	0.2143491	0.129026004	0.08532305	0.76461536	3.12E-06	3.97E-05	16	0	0
OR10A7	0.6960461	0.797459142	-0.10141301	-0.19333062	3.15E-06	4.00E-05	4	0	0
RHO	0.7875652	0.842827308	-0.05526215	-0.10028228	3.17E-06	4.02E-05	7	0	0
TNFSF8	0.4404695	0.555068649	-0.11459919	-0.34456277	3.17E-06	4.03E-05	8	0	0
CLEC5A	0.5020377	0.598316047	-0.09627839	-0.25690441	3.18E-06	4.03E-05	5	0	0
CDH26	0.7103765	0.832263447	-0.12188697	-0.22616022	3.20E-06	4.06E-05	4	0	0
OR51E1	0.578682	0.674764783	-0.09608283	-0.19761077	3.22E-06	4.07E-05	3	0	0
HBD	0.8351184	0.885986727	-0.0508683	-0.0848551	3.22E-06	4.07E-05	3	0	0
OR5AN1	0.5960644	0.757427271	-0.16136288	-0.3376414	3.22E-06	4.07E-05	2	0	0
MYOG	0.77327	0.815017562	-0.04174754	-0.07555482	3.24E-06	4.10E-05	10	0	0
SLC38A4	0.4987517	0.376883381	0.121868309	0.471287274	3.26E-06	4.12E-05	6	0	0
APOC3	0.6155031	0.651642579	-0.03613945	-0.06496827	3.29E-06	4.15E-05	6	0	0
LCE1A	0.7067481	0.815596523	-0.10884839	-0.20302895	3.31E-06	4.18E-05	3	0	0
LSMEM1	0.8115209	0.761505242	0.050015626	0.10114108	3.32E-06	4.18E-05	12	0	0
OR4F6	0.6340543	0.761037854	-0.12698359	-0.26665216	3.34E-06	4.21E-05	4	0	0
DGKB	0.7887187	0.874606213	-0.08588753	-0.14734791	3.35E-06	4.21E-05	1	0	0
CPNE6	0.5709085	0.651152841	-0.08024434	-0.18376715	3.37E-06	4.23E-05	13	0	0
CXCR6	0.3827443	0.431237775	-0.04849348	-0.22877969	3.41E-06	4.27E-05	2	0	0
MOS	0.4530262	0.391052045	0.061974155	0.465245099	3.40E-06	4.27E-05	9	0	0
MMP13	0.796525	0.878386573	-0.08186154	-0.14828905	3.41E-06	4.28E-05	9	0	0
NR2F2	0.228504	0.314486222	-0.08598224	-0.45606425	3.41E-06	4.28E-05	8	0	0
PDCD1LG2	0.3181282	0.371359054	-0.05323083	-0.33609326	3.42E-06	4.28E-05	3	0	0
CCR10	0.7226017	0.642243677	0.080358028	0.235863131	3.42E-06	4.28E-05	5	0	0
HPD	0.8373588	0.8968548	-0.05949597	-0.09754086	3.44E-06	4.31E-05	2	0	0
OTOA	0.6182565	0.737586723	-0.11933023	-0.25040474	3.45E-06	4.31E-05	2	0	0
OR56A4	0.5426535	0.643822131	-0.10116862	-0.26042992	3.46E-06	4.33E-05	5	0	0
IL18RAP	0.6035089	0.699392866	-0.09588399	-0.28284858	3.47E-06	4.33E-05	6	0	0
RIOK3	0.2611746	0.291146277	-0.02997164	-0.03204877	3.47E-06	4.33E-05	9	0	0
CD300LB	0.5352467	0.631869285	-0.09662256	-0.27070702	3.49E-06	4.35E-05	3	0	0
SLC15A2	0.7189008	0.641344091	0.077556758	0.210674538	3.50E-06	4.36E-05	6	0	0
KRTAP5-8	0.863119	0.928907415	-0.06578844	-0.10638063	3.50E-06	4.36E-05	2	0	0
DLX5	0.304161	0.195461631	0.108699408	0.611779838	3.51E-06	4.37E-05	15	0	0
NT5C1B-RD	0.832251	0.897740065	-0.06548911	-0.1124523	3.55E-06	4.40E-05	2	0	0
NT5C1B	0.832251	0.897740065	-0.06548911	-0.1124523	3.55E-06	4.40E-05	2	0	0
OR4K5	0.6283102	0.738705612	-0.11039537	-0.22774372	3.56E-06	4.41E-05	4	0	0
HLA-DPA1	0.6428258	0.553254225	0.089571598	0.243491081	3.56E-06	4.41E-05	10	0	0
YPEL4	0.4152037	0.383790327	0.031413403	0.147792079	3.56E-06	4.41E-05	4	0	0
KRT18	0.5353938	0.579657324	-0.04426356	-0.24591481	3.57E-06	4.42E-05	7	0	0
GSTM5	0.5225683	0.376094755	0.146473551	0.47278117	3.58E-06	4.43E-05	4	0	0
OR1A2	0.5792103	0.686105869	-0.10689555	-0.24136956	3.59E-06	4.44E-05	5	0	0
HOXB8	0.2671179	0.155869809	0.111248068	0.858151469	3.60E-06	4.44E-05	14	0	0
CALB1	0.6840812	0.790868326	-0.10678716	-0.21249675	3.62E-06	4.47E-05	2	0	0
HSPA12B	0.4069921	0.36351572	0.043476425	0.5444248	3.62E-06	4.47E-05	6	0	0
ANO4	0.2292465	0.155610901	0.073635637	0.579241054	3.65E-06	4.50E-05	6	0	0
GJD2	0.1812064	0.077694036	0.103512352	1.156119786	3.65E-06	4.50E-05	8	0	0
IRF8	0.1861809	0.19910902	-0.01292814	0.280895041	3.65E-06	4.50E-05	4	0	0
NEK6	0.502363	0.570899946	-0.06853694	-0.15659656	3.68E-06	4.52E-05	9	0	0
PRSS38	0.816353	0.876557132	-0.0602041	-0.10156801	3.69E-06	4.54E-05	5	0	0
LILRB4	0.3238837	0.413253191	-0.08936953	-0.34217544	3.71E-06	4.56E-05	1	0	0
ANKRD9	0.0915857	0.126956108	-0.03537038	-0.15605346	3.72E-06	4.56E-05	9	0	0
CLEC4E	0.4628755	0.58453985	-0.12166433	-0.31169332	3.75E-06	4.60E-05	3	0	0
LGALS7B	0.6382899	0.746656707	-0.10836676	-0.21786098	3.75E-06	4.60E-05	5	0	0
C6orf58	0.2240701	0.160080746	0.063989318	0.639499113	3.77E-06	4.62E-05	16	0	0
LCNL1	0.7221038	0.679253221	0.042850579	0.109337347	3.81E-06	4.66E-05	4	0	0
OR4C15	0.4243637	0.52135656	-0.09699285	-0.29811006	3.82E-06	4.67E-05	3	0	0
C20orf141	0.705485	0.776030805	-0.07054583	-0.15579065	3.82E-06	4.68E-05	5	0	0

OR7C2	0.7374772	0.84726629	-0.10978905	-0.19965521	3.85E-06	4.71E-05	3	0	0
CHST11	0.8202225	0.896214152	-0.07599164	-0.12788931	3.86E-06	4.71E-05	2	0	0
PAIP1	0.2813058	0.326133927	-0.04482811	-0.13541996	3.90E-06	4.76E-05	12	0	0
ZEB1	0.0895488	0.124199436	-0.03465059	-0.23816124	3.91E-06	4.76E-05	10	0	0
FXYD2	0.463989	0.534510407	-0.07052139	-0.25816336	3.93E-06	4.79E-05	8	0	0
NEFH	0.3048536	0.207718615	0.097134991	0.735594778	3.93E-06	4.79E-05	8	0	0
CYP21A2	0.6622587	0.790651321	-0.12839263	-0.25215759	3.94E-06	4.79E-05	1	0	0
RNASE7	0.667234	0.768677499	-0.10144354	-0.21068405	3.95E-06	4.80E-05	7	0	0
INHA	0.8094379	0.859572781	-0.05013487	-0.0864238	3.96E-06	4.81E-05	4	0	0
FRZB	0.2175826	0.155091688	0.062490896	0.839785142	3.96E-06	4.81E-05	10	0	0
KRT14	0.7050902	0.780305148	-0.07521498	-0.14527702	3.97E-06	4.82E-05	3	0	0
ERICH2	0.8163146	0.865626441	-0.04931189	-0.08370019	4.00E-06	4.85E-05	3	0	0
MUC5AC	0.510081	0.592423015	-0.08234197	-0.21263059	4.01E-06	4.86E-05	6	0	0
CHMP4C	0.2561185	0.314958159	-0.05883962	-0.29166848	4.03E-06	4.88E-05	11	0	0
SMIM17	0.4268822	0.345932711	0.080949452	0.391994213	4.03E-06	4.88E-05	6	0	0
NME5	0.3305421	0.254942436	0.075599684	0.707427442	4.04E-06	4.88E-05	11	0	0
EPGN	0.5827452	0.676131002	-0.09338576	-0.22578796	4.04E-06	4.89E-05	6	0	0
RAG2	0.7459386	0.823439115	-0.07750049	-0.14386658	4.07E-06	4.91E-05	4	0	0
C4orf45	0.7782941	0.857960197	-0.07966612	-0.13974088	4.07E-06	4.91E-05	2	0	0
OR6K6	0.6793104	0.805546268	-0.12623591	-0.24815863	4.08E-06	4.93E-05	4	0	0
CHRNE	0.3489917	0.381603376	-0.03261169	-0.2476108	4.09E-06	4.93E-05	11	0	0
FOXC2	0.1715632	0.091564554	0.07999864	0.915206587	4.10E-06	4.93E-05	7	0	0
SLC22A6	0.5925935	0.693973198	-0.10137972	-0.21965454	4.12E-06	4.95E-05	7	0	0
LRFN5	0.14722	0.078908803	0.068311193	0.831411365	4.14E-06	4.98E-05	11	0	0
ADAM18	0.7741977	0.852579908	-0.07838218	-0.14026815	4.16E-06	5.00E-05	9	0	0
ANXA1	0.7694642	0.845945326	-0.07648108	-0.13104068	4.20E-06	5.04E-05	2	0	0
FAM187B	0.8148736	0.89446421	-0.07959061	-0.13289036	4.20E-06	5.04E-05	1	0	0
SLC9C2	0.5117474	0.399073031	0.112674329	0.402302937	4.21E-06	5.04E-05	4	0	0
MR1	0.4265228	0.458565366	-0.03204253	-0.24061933	4.21E-06	5.04E-05	6	0	0
OR10A3	0.7002594	0.794656005	-0.09439664	-0.18089426	4.22E-06	5.06E-05	4	0	0
SLC26A3	0.6049369	0.735221519	-0.1302846	-0.2772309	4.23E-06	5.06E-05	1	0	0
CCDC181	0.3855712	0.268809912	0.116761305	0.727289234	4.27E-06	5.11E-05	7	0	0
KRT6A	0.5550873	0.66744067	-0.11235333	-0.23369874	4.28E-06	5.11E-05	6	0	0
ENPP2	0.6585491	0.609929363	0.04861973	0.140664642	4.33E-06	5.16E-05	6	0	0
HBG2	0.6138348	0.705007764	-0.09117295	-0.20335278	4.32E-06	5.16E-05	3	0	0
PZP	0.7236428	0.817882569	-0.09423977	-0.16967135	4.33E-06	5.16E-05	3	0	0
CD1A	0.6641566	0.781535764	-0.1173792	-0.23730525	4.34E-06	5.17E-05	4	0	0
SPTLC3	0.4684148	0.570038747	-0.10162399	-0.15459351	4.34E-06	5.17E-05	6	0	0
AMTN	0.6745029	0.777802559	-0.10329967	-0.21639713	4.37E-06	5.20E-05	3	0	0
AVPR1A	0.219496	0.118579515	0.100916448	0.878743989	4.37E-06	5.20E-05	11	0	0
TMEM155	0.3151484	0.256987601	0.058160815	1.114463002	4.38E-06	5.21E-05	9	0	0
KRTAP10-1(	0.6850032	0.781287732	-0.0962845	-0.19407567	4.39E-06	5.22E-05	2	0	0
SPACA7	0.6199291	0.725170619	-0.10524155	-0.22890289	4.40E-06	5.22E-05	5	0	0
DMRT3	0.1929015	0.110785309	0.082116182	0.755856232	4.41E-06	5.24E-05	7	0	0
SMIM21	0.7325571	0.823307226	-0.0907501	-0.17784559	4.42E-06	5.25E-05	8	0	0
TAS2R40	0.7090311	0.811717032	-0.10268591	-0.19978746	4.44E-06	5.26E-05	5	0	0
EPHX2	0.306265	0.264469511	0.041795514	0.416204525	4.44E-06	5.26E-05	7	0	0
MRGPRX3	0.6486293	0.735670593	-0.08704133	-0.17943708	4.45E-06	5.26E-05	2	0	0
NR2E1	0.2451372	0.139143496	0.105993726	0.90324979	4.45E-06	5.27E-05	16	0	0
SLC5A9	0.6978578	0.772000647	-0.07414289	-0.14552313	4.48E-06	5.29E-05	5	0	0
KAT2B	0.4113721	0.28836404	0.123008074	0.592052676	4.48E-06	5.29E-05	3	0	0
CALCA	0.2552792	0.178202623	0.077076594	0.595455169	4.48E-06	5.29E-05	33	0	0
TPRX1	0.8108573	0.86160668	-0.05074935	-0.08654576	4.50E-06	5.30E-05	1	0	0
CRX	0.8108573	0.86160668	-0.05074935	-0.08654576	4.50E-06	5.30E-05	1	0	0
DGAT2	0.5767549	0.673201487	-0.09644659	-0.21955315	4.51E-06	5.31E-05	1	0	0
RHOBTB3	0.7879618	0.832247909	-0.04428615	-0.0786096	4.52E-06	5.32E-05	3	0	0
C10orf120	0.6123603	0.702881313	-0.09052104	-0.18405848	4.52E-06	5.32E-05	6	0	0
BPIFA3	0.515678	0.651071771	-0.13539381	-0.32963799	4.52E-06	5.32E-05	4	0	0
GABRA2	0.6746592	0.778912143	-0.10425296	-0.22540536	4.54E-06	5.34E-05	4	0	0
LPCAT1	0.3268563	0.387966404	-0.06111008	-0.16127842	4.57E-06	5.37E-05	5	0	0
NKX2-3	0.2286394	0.138382342	0.090257107	0.957468647	4.59E-06	5.39E-05	9	0	0
RAD23A	0.2485525	0.316254757	-0.0677023	-0.1983116	4.60E-06	5.40E-05	11	0	0
GRAMD4	0.6920395	0.686159151	0.005880325	-0.00389444	4.61E-06	5.40E-05	2	0	0

COL6A6	0.6729104	0.761025574	-0.08811521	-0.16802866	4.63E-06	5.42E-05	3	0	0
PCDHB15	0.372112	0.293650236	0.078461735	0.612616703	4.64E-06	5.43E-05	13	0	0
NPEPL1	0.6774992	0.63193473	0.045564489	0.131641993	4.68E-06	5.47E-05	6	0	0
OSBPL9	0.6520145	0.628434782	0.023579674	0.121855753	4.69E-06	5.48E-05	2	0	0
MEDAG	0.5182262	0.480027611	0.038198634	0.359196575	4.69E-06	5.48E-05	9	0	0
SERPINB5	0.759834	0.854444686	-0.09461073	-0.19057946	4.69E-06	5.48E-05	6	0	0
MMRN2	0.3951567	0.317377493	0.077779209	0.325675558	4.71E-06	5.50E-05	10	0	0
GORASP2	0.6677024	0.774634394	-0.10693201	-0.24151494	4.72E-06	5.50E-05	10	0	0
WFDC10B	0.7934095	0.839115382	-0.04570592	-0.07517402	4.72E-06	5.50E-05	3	0	0
TXNDC15	0.5906787	0.619254938	-0.02857619	-0.08147964	4.72E-06	5.50E-05	9	0	0
VWA3A	0.6439559	0.598922075	0.045033861	0.166961077	4.74E-06	5.52E-05	8	0	0
WFDC13	0.5683934	0.655865461	-0.08747204	-0.22523221	4.78E-06	5.56E-05	6	0	0
COL26A1	0.2792089	0.190974188	0.088234722	0.841817839	4.79E-06	5.57E-05	13	0	0
ADCYAP1	0.2778795	0.167356001	0.11052351	0.941101305	4.80E-06	5.58E-05	18	0	0
FAM53A	0.3668417	0.429964116	-0.06312237	-0.12284894	4.82E-06	5.60E-05	6	0	0
VGF	0.1399184	0.12876508	0.01115336	0.069558996	4.84E-06	5.62E-05	9	0	0
CYTH4	0.4277027	0.488901139	-0.06119848	-0.25279473	4.86E-06	5.64E-05	8	0	0
IFNA8	0.5833347	0.718889031	-0.13555437	-0.29685312	4.89E-06	5.67E-05	1	0	0
SST	0.3597581	0.352067748	0.007690318	0.245875648	4.91E-06	5.68E-05	14	0	0
PYHIN1	0.6013683	0.74755661	-0.14618832	-0.31493087	4.93E-06	5.70E-05	4	0	0
CC2D2B	0.753479	0.852731221	-0.09925224	-0.18326145	4.98E-06	5.76E-05	3	0	0
AMPD1	0.6810118	0.788876643	-0.10786485	-0.20975845	5.02E-06	5.80E-05	3	0	0
DSC1	0.5844109	0.6849521	-0.10054122	-0.22651638	5.03E-06	5.80E-05	5	0	0
PAEP	0.519985	0.599050759	-0.0790658	-0.21022999	5.04E-06	5.81E-05	3	0	0
OR52K2	0.4900681	0.591734869	-0.1016668	-0.27944981	5.04E-06	5.81E-05	3	0	0
ACTA1	0.2371275	0.144269817	0.092857698	0.738051894	5.10E-06	5.88E-05	9	0	0
TDRD7	0.4969814	0.572412041	-0.07543061	-0.22005465	5.12E-06	5.90E-05	5	0	0
RBMS2	0.2283692	0.15052219	0.077847009	0.36681355	5.13E-06	5.90E-05	7	0	0
PGK2	0.6090513	0.705476648	-0.09642532	-0.21582492	5.14E-06	5.91E-05	9	0	0
HNF1B	0.2222261	0.101379171	0.120846911	1.09509124	5.14E-06	5.91E-05	10	0	0
SCN2B	0.6907514	0.682013626	0.008737786	0.074363546	5.15E-06	5.91E-05	6	0	0
ZSCAN5A	0.2662128	0.147618254	0.118594537	1.377592966	5.15E-06	5.91E-05	13	0	0
RPL10L	0.6560924	0.708923822	-0.05283147	-0.13150871	5.21E-06	5.98E-05	2	0	0
PSMF1	0.7687631	0.836859206	-0.06809611	-0.12255492	5.21E-06	5.98E-05	5	0	0
TMPRSS7	0.8222371	0.890409882	-0.06817277	-0.11719516	5.22E-06	5.98E-05	5	0	0
CHST3	0.4070529	0.349668359	0.05738459	0.185435988	5.24E-06	6.00E-05	4	0	0
MOGAT2	0.679829	0.746823682	-0.0669947	-0.13863674	5.24E-06	6.00E-05	8	0	0
OSTN	0.5140152	0.682919325	-0.16890408	-0.4030786	5.31E-06	6.07E-05	1	0	0
USP10	0.3277309	0.380842603	-0.0531117	-0.22275577	5.31E-06	6.07E-05	8	0	0
CGB5	0.6074518	0.726409657	-0.11895783	-0.25418196	5.34E-06	6.10E-05	1	0	0
BTLA	0.569078	0.693397441	-0.12431942	-0.33804024	5.37E-06	6.13E-05	5	0	0
LMO3	0.3673132	0.280935925	0.086377293	0.516158603	5.38E-06	6.14E-05	5	0	0
GABRB1	0.1726196	0.101335109	0.071284532	0.755277256	5.41E-06	6.17E-05	11	0	0
STK38L	0.4188781	0.484824333	-0.0659462	-0.22799088	5.42E-06	6.17E-05	11	0	0
OR2A5	0.6538452	0.738718473	-0.08487325	-0.1841907	5.44E-06	6.19E-05	4	0	0
HTR3B	0.6408326	0.633684361	0.007148194	0.057876151	5.44E-06	6.19E-05	3	0	0
EPO	0.167708	0.080950785	0.086757189	1.314449608	5.44E-06	6.19E-05	10	0	0
IL17D	0.7145883	0.768333948	-0.05374564	-0.15352845	5.45E-06	6.20E-05	5	0	0
CYP17A1	0.8406944	0.886666473	-0.04597209	-0.07593053	5.58E-06	6.34E-05	1	0	0
NUPR1	0.4683521	0.378836385	0.089515681	0.31048639	5.61E-06	6.37E-05	8	0	0
TUBB6	0.2515198	0.111541512	0.139978297	1.199526447	5.62E-06	6.37E-05	4	0	0
PBX4	0.1979698	0.211208242	-0.01323841	-0.193785	5.62E-06	6.38E-05	16	0	0
PTH	0.6876638	0.782600224	-0.09493647	-0.18465228	5.64E-06	6.39E-05	5	0	0
HK1	0.7203231	0.791699287	-0.07137615	-0.13406135	5.67E-06	6.42E-05	3	0	0
CNBD2	0.4453803	0.564560704	-0.11918039	-0.33538739	5.69E-06	6.43E-05	1	0	0
HOXA4	0.4390955	0.326620637	0.112474863	0.477471275	5.69E-06	6.44E-05	19	0	0
OR52B6	0.8000828	0.879744266	-0.07966148	-0.13803315	5.70E-06	6.44E-05	6	0	0
SOX15	0.7051436	0.797460935	-0.09231734	-0.17443947	5.79E-06	6.54E-05	12	0	0
SCAMP4	0.1723213	0.219874589	-0.0475533	-0.27522522	5.80E-06	6.55E-05	8	0	0
GRIK3	0.2286481	0.153982286	0.074665809	0.688612261	5.82E-06	6.56E-05	10	0	0
RALYL	0.2552588	0.146140554	0.1091182	0.969549448	5.82E-06	6.56E-05	9	0	0
TNFSF18	0.7815137	0.694113138	0.087400537	0.177631899	5.84E-06	6.57E-05	2	0	0
CDK10	0.6743415	0.765675853	-0.09133433	-0.18073741	5.84E-06	6.57E-05	1	0	0

04-Sep	0.7076197	0.794215413	-0.08659571	-0.16557332	5.84E-06	6.57E-05	6	0	0
CETN1	0.8616231	0.940563641	-0.07894059	-0.12522281	5.83E-06	6.57E-05	3	0	0
01-Dec	0.7488966	0.829163005	-0.08026638	-0.1524088	5.86E-06	6.58E-05	4	0	0
KRTAP5-1	0.5720606	0.692951321	-0.12089074	-0.27225547	5.87E-06	6.60E-05	1	0	0
OR10Q1	0.7711573	0.864468354	-0.09331105	-0.17008561	5.92E-06	6.64E-05	6	0	0
DEGS1	0.682807	0.536694729	0.146112252	0.341718001	5.95E-06	6.68E-05	1	0	0
PTH2R	0.7672487	0.886934707	-0.11968596	-0.2083529	5.99E-06	6.71E-05	4	0	0
UNC5CL	0.6302961	0.681832997	-0.05153691	-0.10628196	5.99E-06	6.71E-05	7	0	0
DLC1	0.4957403	0.619265492	-0.12352516	-0.32761314	5.99E-06	6.71E-05	4	0	0
ADAM30	0.7279918	0.700842062	0.0271497	0.083184361	6.01E-06	6.73E-05	11	0	0
PRSS57	0.3980451	0.485105123	-0.08706004	-0.28253516	6.03E-06	6.74E-05	7	0	0
PDPK1	0.3673952	0.465195437	-0.09780028	-0.25000635	6.05E-06	6.76E-05	2	0	0
GRK7	0.7952367	0.864506772	-0.06927009	-0.12762455	6.07E-06	6.78E-05	13	0	0
CD84	0.3543132	0.471989126	-0.11767596	-0.40434064	6.08E-06	6.79E-05	4	0	0
SLC26A9	0.422429	0.505665794	-0.08323683	-0.2970822	6.08E-06	6.79E-05	4	0	0
ATIC	0.1553832	0.199510697	-0.04412751	-0.33953212	6.09E-06	6.79E-05	13	0	0
OR12D3	0.6947337	0.777471172	-0.08273747	-0.17422099	6.12E-06	6.82E-05	5	0	0
CRNN	0.8351045	0.897008859	-0.06190437	-0.10434793	6.14E-06	6.82E-05	3	0	0
MBNL1	0.6321989	0.774256051	-0.14205719	-0.28830465	6.13E-06	6.82E-05	1	0	0
PDILT	0.6808221	0.75367766	-0.07285552	-0.17225625	6.14E-06	6.82E-05	6	0	0
DDC	0.5039313	0.601373304	-0.09744202	-0.22862961	6.16E-06	6.84E-05	8	0	0
CYP2C8	0.8146568	0.886044143	-0.07138736	-0.11998623	6.18E-06	6.86E-05	2	0	0
ZNF471	0.3026283	0.204177367	0.098450914	0.968108913	6.20E-06	6.88E-05	10	0	0
INMT	0.5206147	0.558041967	-0.03742729	-0.09265364	6.21E-06	6.89E-05	5	0	0
RNF216	0.0382342	0.052794434	-0.01456022	-0.20185677	6.22E-06	6.90E-05	7	0	0
PCDHGA8	0.4351011	0.336656206	0.098444864	0.419596998	6.23E-06	6.91E-05	6	0	0
CSNK1A1L	0.7990399	0.865484063	-0.06644418	-0.11876872	6.24E-06	6.91E-05	9	0	0
FAM205A	0.7716443	0.835028754	-0.06338442	-0.11358468	6.26E-06	6.92E-05	2	0	0
TRPM5	0.696717	0.768058074	-0.07134103	-0.13764679	6.28E-06	6.94E-05	18	0	0
ZCRB1	0.060062	0.078223916	-0.01816189	-0.24677969	6.37E-06	7.04E-05	6	0	0
CLDN12	0.816861	0.888327481	-0.07146645	-0.11959659	6.38E-06	7.05E-05	1	0	0
TMEM211	0.5181439	0.582084026	-0.06394011	-0.17341736	6.45E-06	7.12E-05	3	0	0
IQCF3	0.7900902	0.870486034	-0.08039587	-0.14647241	6.46E-06	7.13E-05	5	0	0
PAK1	0.0645073	0.075738199	-0.01123094	-0.22990525	6.46E-06	7.13E-05	5	0	0
VILL	0.5921788	0.657735264	-0.06555642	-0.17234824	6.48E-06	7.13E-05	5	0	0
HLX	0.3983057	0.308027092	0.090278589	0.442931472	6.52E-06	7.18E-05	9	0	0
TMEM233	0.2740588	0.330903801	-0.05684497	0.049967573	6.58E-06	7.24E-05	9	0	0
TIAM2	0.8681448	0.847265687	0.020879125	0.034967412	6.59E-06	7.24E-05	5	0	0
PANK4	0.1464666	0.179283796	-0.03281722	-0.23416777	6.59E-06	7.24E-05	17	0	0
MS4A5	0.7485494	0.837412587	-0.08886317	-0.16917388	6.60E-06	7.24E-05	6	0	0
ATG9B	0.6497047	0.720814273	-0.07110953	-0.16756527	6.62E-06	7.26E-05	4	0	0
SMTNL1	0.7681005	0.853899407	-0.08579887	-0.15585266	6.64E-06	7.29E-05	7	0	0
HIPK3	0.3972129	0.494781745	-0.09756888	-0.21557796	6.67E-06	7.32E-05	12	0	0
TAS1R2	0.695983	0.75818054	-0.06219756	-0.12115873	6.68E-06	7.32E-05	3	0	0
OR56B4	0.5218703	0.643678834	-0.12180852	-0.29750747	6.70E-06	7.34E-05	1	0	0
BHLHE23	0.2004991	0.121638725	0.078860374	0.933486012	6.73E-06	7.37E-05	4	0	0
LIFR	0.5114878	0.618540648	-0.10705285	-0.27086465	6.74E-06	7.38E-05	2	0	0
ZIM3	0.676569	0.77142938	-0.09486035	-0.21499429	6.76E-06	7.39E-05	3	0	0
FABP2	0.7557139	0.822708989	-0.06699513	-0.12272934	6.78E-06	7.41E-05	5	0	0
PLXNA1	0.6792572	0.720044601	-0.04078735	-0.06689158	6.79E-06	7.41E-05	8	0	0
LRRC56	0.1154766	0.148160765	-0.0326842	-0.20164601	6.79E-06	7.41E-05	7	0	0
CTRB2	0.6998717	0.76287416	-0.06300249	-0.1379554	6.83E-06	7.45E-05	9	0	0
GALNT3	0.1140302	0.137387075	-0.02335686	-0.36660797	6.85E-06	7.46E-05	13	0	0
CADPS	0.2156807	0.120833166	0.094847549	1.005634917	6.85E-06	7.46E-05	11	0	0
EXOC6	0.4842897	0.4161164	0.068173301	0.203644105	6.88E-06	7.48E-05	6	0	0
OR52I2	0.6867388	0.77052117	-0.08378237	-0.17547748	6.89E-06	7.49E-05	4	0	0
RTN4R	0.5906985	0.665602068	-0.0749036	-0.16953276	6.90E-06	7.50E-05	1	0	0
MCHR2	0.3740647	0.279568592	0.094496152	0.780205775	6.91E-06	7.51E-05	14	0	0
HS3ST2	0.2341831	0.136306075	0.09787705	0.858588231	6.92E-06	7.51E-05	9	0	0
SCARF2	0.1889092	0.136505119	0.052404054	0.571528236	6.94E-06	7.53E-05	13	0	0
CIB4	0.5909557	0.688131919	-0.09717618	-0.22157569	6.95E-06	7.53E-05	6	0	0
CRYBB2	0.750056	0.81522515	-0.06516913	-0.12664265	6.95E-06	7.53E-05	13	0	0
PEX11A	0.1291612	0.151215538	-0.02205432	-0.2137782	6.97E-06	7.54E-05	16	0	0

MYF6	0.6681084	0.745885631	-0.0777772	-0.17742497	6.98E-06	7.55E-05	7	0	0
DOK6	0.1643325	0.077569912	0.086762579	1.096238588	6.98E-06	7.55E-05	5	0	0
CLDN8	0.6136736	0.717907069	-0.10423345	-0.22636067	7.00E-06	7.57E-05	5	0	0
PCDHGB4	0.5385258	0.458682778	0.079843027	0.40899625	7.01E-06	7.57E-05	3	0	0
FAM136A	0.5113605	0.594848105	-0.08348762	-0.15690708	7.03E-06	7.59E-05	4	0	0
CCL21	0.6415443	0.633377728	0.00816661	0.06183808	7.03E-06	7.59E-05	3	0	0
GPX6	0.7636057	0.840913754	-0.07730809	-0.14590898	7.09E-06	7.63E-05	4	0	0
TREML1	0.5299746	0.582615123	-0.05264049	-0.11143113	7.09E-06	7.63E-05	8	0	0
OR1L6	0.6130794	0.707594919	-0.09451549	-0.20940001	7.09E-06	7.63E-05	3	0	0
ECI1	0.6265433	0.687545357	-0.06100207	-0.1606166	7.09E-06	7.63E-05	5	0	0
KPRP	0.6326833	0.725407857	-0.09272457	-0.19687611	7.13E-06	7.64E-05	5	0	0
UROC1	0.6716855	0.759072649	-0.08738716	-0.19386917	7.13E-06	7.64E-05	5	0	0
HAND2	0.220549	0.133261908	0.087287049	0.773197999	7.12E-06	7.64E-05	19	0	0
GRK6	0.3068314	0.262458648	0.044372714	0.139448414	7.12E-06	7.64E-05	12	0	0
AMBP	0.5655264	0.591488235	-0.02596179	-0.09198758	7.13E-06	7.64E-05	5	0	0
OR4K13	0.6248143	0.713726093	-0.08891177	-0.19122463	7.12E-06	7.64E-05	3	0	0
BHLHA9	0.5011681	0.452137099	0.049030996	0.29653063	7.13E-06	7.64E-05	9	0	0
SIAE	0.8647585	0.916336391	-0.05157788	-0.08263062	7.16E-06	7.67E-05	2	0	0
SFMBT1	0.4430443	0.48268834	-0.03964408	-0.19974765	7.17E-06	7.68E-05	4	0	0
11-Mar	0.4407785	0.459960735	-0.01918219	0.132253107	7.19E-06	7.69E-05	6	0	0
AQP8	0.6640843	0.741046252	-0.07696198	-0.15911371	7.23E-06	7.73E-05	3	0	0
PDHX	0.2651835	0.342729979	-0.07754644	-0.37026463	7.25E-06	7.74E-05	2	0	0
PCK1	0.5284737	0.617535836	-0.08906209	-0.21248312	7.26E-06	7.75E-05	10	0	0
OCIAD1	0.7640099	0.68126123	0.082748705	0.163121072	7.27E-06	7.75E-05	1	0	0
CUEDC1	0.1264894	0.193831458	-0.06734206	-0.23985611	7.27E-06	7.75E-05	5	0	0
TTN	0.665577	0.79045804	-0.12488106	-0.25096697	7.28E-06	7.76E-05	2	0	0
BCL11A	0.0573139	0.072382484	-0.01506856	-0.23148297	7.38E-06	7.86E-05	15	0	0
MYOM2	0.270571	0.333523317	-0.06295231	-0.25617643	7.40E-06	7.88E-05	14	0	0
LGALS8	0.5393079	0.671446935	-0.13213902	-0.29609202	7.42E-06	7.89E-05	2	0	0
CRCP	0.4178505	0.484056838	-0.06620635	-0.09356884	7.42E-06	7.89E-05	7	0	0
FAM167B	0.6561809	0.587124977	0.069055913	0.159844692	7.44E-06	7.90E-05	5	0	0
ZFP28	0.2098482	0.113634935	0.096213265	1.239761459	7.44E-06	7.91E-05	8	0	0
SOHLH1	0.6983916	0.807083536	-0.10869191	-0.19600557	7.45E-06	7.91E-05	5	0	0
ODF3	0.5956228	0.676050539	-0.08042772	-0.1877799	7.50E-06	7.95E-05	6	0	0
SOST	0.6301758	0.737701008	-0.10752516	-0.20278945	7.50E-06	7.95E-05	5	0	0
HOOK2	0.6127612	0.483112233	0.129648998	0.347564495	7.50E-06	7.95E-05	2	0	0
PCDHGA3	0.3929283	0.314277414	0.078650877	0.469043517	7.54E-06	7.98E-05	10	0	0
PLEKHN1	0.5020562	0.556493931	-0.05443777	-0.24003784	7.55E-06	7.99E-05	9	0	0
RPS3A	0.1384976	0.177705763	-0.0392082	-0.15560686	7.56E-06	7.99E-05	8	0	0
VSTM2A	0.1858568	0.111033886	0.074822909	0.699972468	7.57E-06	8.00E-05	11	0	0
SESN3	0.367149	0.371479888	-0.00433093	0.515316776	7.58E-06	8.00E-05	12	0	0
GUCA1C	0.7631615	0.850412449	-0.08725099	-0.15901861	7.66E-06	8.08E-05	4	0	0
ARHGEF15	0.5685152	0.482959917	0.085555241	0.258313673	7.67E-06	8.09E-05	6	0	0
DPRX	0.7712663	0.856921391	-0.08565507	-0.15696658	7.67E-06	8.09E-05	8	0	0
SWSAP1	0.1062388	0.149802945	-0.04356419	-0.22653247	7.68E-06	8.10E-05	15	0	0
SUN1	0.0813274	0.135844603	-0.05451716	-0.35643362	7.70E-06	8.10E-05	7	0	0
C18orf32	0.1545478	0.195552654	-0.04100485	-0.20811354	7.70E-06	8.10E-05	8	0	0
TMEM144	0.7842488	0.824030984	-0.03978218	-0.07341208	7.75E-06	8.15E-05	2	0	0
MALRD1	0.6368892	0.743741798	-0.10685255	-0.22172421	7.79E-06	8.19E-05	3	0	0
EGLN1	0.7569023	0.686661152	0.070241115	0.201720942	7.80E-06	8.19E-05	4	0	0
UGT2B4	0.8562059	0.892414583	-0.03620864	-0.0590803	7.83E-06	8.22E-05	1	0	0
SPINK14	0.8682486	0.890073558	-0.02182494	-0.03541351	7.86E-06	8.24E-05	1	0	0
SMIM2	0.749526	0.794627629	-0.04510162	-0.07515624	7.86E-06	8.24E-05	6	0	0
ZMYM1	0.6703224	0.760208198	-0.08988583	-0.17546977	7.93E-06	8.31E-05	7	0	0
KIR3DL1	0.6358646	0.741026523	-0.10516191	-0.22385681	7.95E-06	8.31E-05	2	0	0
KIR3DL3	0.6358646	0.741026523	-0.10516191	-0.22385681	7.95E-06	8.31E-05	2	0	0
KIR2DL4	0.6358646	0.741026523	-0.10516191	-0.22385681	7.95E-06	8.31E-05	2	0	0
GJA8	0.7152749	0.785660022	-0.07038508	-0.13496228	7.96E-06	8.32E-05	6	0	0
ZNF71	0.407265	0.425783476	-0.01851844	0.583876196	7.99E-06	8.35E-05	6	0	0
SATB2	0.3132672	0.235583224	0.077684	0.749272506	8.01E-06	8.37E-05	9	0	0
ODF1	0.8351689	0.902518273	-0.06734941	-0.11495718	8.06E-06	8.40E-05	8	0	0
GPR26	0.3324508	0.244373422	0.088077328	0.512046896	8.06E-06	8.40E-05	9	0	0
OR52A1	0.5280434	0.631832218	-0.10378877	-0.25447354	8.05E-06	8.40E-05	1	0	0

FOXF2	0.1891966	0.120259889	0.068936676	0.760305736	8.06E-06	8.40E-05	6	0	0
SCGB3A1	0.2230069	0.147090707	0.075916228	1.026027328	8.08E-06	8.41E-05	10	0	0
FAM177A1	0.6874738	0.739701514	-0.05222768	-0.12587752	8.10E-06	8.43E-05	3	0	0
GPR32	0.7160565	0.850634499	-0.13457796	-0.24531878	8.10E-06	8.43E-05	1	0	0
DDR1	0.5964511	0.737474608	-0.14102348	-0.30240957	8.11E-06	8.43E-05	3	0	0
S100A12	0.6151852	0.716581674	-0.10139645	-0.22471877	8.18E-06	8.50E-05	5	0	0
SPERT	0.7067853	0.790340549	-0.08355526	-0.16741407	8.19E-06	8.51E-05	4	0	0
WFIKKN1	0.7565609	0.72312218	0.033438677	0.070106836	8.21E-06	8.52E-05	6	0	0
ZMYND8	0.1768464	0.21992708	-0.04308067	-0.29006252	8.24E-06	8.55E-05	15	0	0
NLRP1	0.7461019	0.859013668	-0.11291173	-0.20079812	8.26E-06	8.56E-05	1	0	0
AFAP1L2	0.2585936	0.280822262	-0.02222864	0.095971182	8.27E-06	8.57E-05	12	0	0
METTL9	0.8227507	0.8541361	-0.03138544	-0.0537562	8.29E-06	8.59E-05	7	0	0
FSCN3	0.7219598	0.78536403	-0.06340419	-0.12932246	8.30E-06	8.59E-05	2	0	0
MATN4	0.5979561	0.562714343	0.035241742	0.118419701	8.31E-06	8.59E-05	13	0	0
TNFRSF13C	0.493621	0.549682824	-0.05606185	-0.14735221	8.31E-06	8.59E-05	5	0	0
OR51E2	0.6259145	0.719490624	-0.09357608	-0.20704421	8.34E-06	8.62E-05	5	0	0
TMEM26	0.3299328	0.255133071	0.074799779	0.717834347	8.35E-06	8.62E-05	12	0	0
C3orf22	0.6004592	0.699663916	-0.09920469	-0.20646027	8.39E-06	8.66E-05	8	0	0
CD33	0.3713469	0.446832725	-0.07548586	-0.24696421	8.40E-06	8.66E-05	3	0	0
THEM5	0.6422147	0.733089842	-0.09087517	-0.2085295	8.43E-06	8.69E-05	7	0	0
YIPF1	0.768327	0.831419732	-0.06309273	-0.11632131	8.47E-06	8.72E-05	5	0	0
FIGLA	0.4117639	0.324582936	0.087180934	0.575999451	8.47E-06	8.72E-05	10	0	0
IFNA16	0.5756808	0.726667855	-0.15098707	-0.33089973	8.48E-06	8.72E-05	1	0	0
GPR84	0.5871087	0.701428915	-0.1143202	-0.2703516	8.48E-06	8.72E-05	6	0	0
GCSAML	0.5311075	0.651255824	-0.12014836	-0.29494234	8.49E-06	8.72E-05	3	0	0
TRPM8	0.7072863	0.812084527	-0.10479826	-0.1971451	8.49E-06	8.72E-05	2	0	0
TMEM40	0.754247	0.803497216	-0.04925019	-0.09181363	8.50E-06	8.73E-05	2	0	0
PAPPA2	0.5972016	0.707149879	-0.10994824	-0.23731144	8.52E-06	8.75E-05	4	0	0
SLC25A45	0.6725132	0.645872747	0.026640481	0.086256075	8.54E-06	8.75E-05	4	0	0
THEMIS	0.7004117	0.792569297	-0.09215764	-0.18381224	8.55E-06	8.76E-05	6	0	0
FDCSP	0.7155591	0.773782036	-0.05822295	-0.11666012	8.56E-06	8.76E-05	4	0	0
SCN1A	0.5664379	0.709513338	-0.14307547	-0.31985632	8.60E-06	8.81E-05	1	0	0
TMEM156	0.49311	0.575034893	-0.08192486	-0.23016551	8.62E-06	8.82E-05	5	0	0
GJB7	0.7586489	0.878570253	-0.11992137	-0.23139659	8.63E-06	8.83E-05	4	0	0
N4BP3	0.3131594	0.36636229	-0.05320285	-0.02959629	8.64E-06	8.83E-05	6	0	0
DPYS	0.5248129	0.459853276	0.064959593	0.427861194	8.64E-06	8.83E-05	11	0	0
TSPYL5	0.4729032	0.47899753	-0.0060943	0.461615834	8.66E-06	8.84E-05	7	0	0
KDM4B	0.3465696	0.411264037	-0.06469444	-0.22728282	8.67E-06	8.84E-05	4	0	0
SVOP	0.4825382	0.574661211	-0.09212305	-0.2430384	8.69E-06	8.86E-05	11	0	0
SSTR4	0.4882169	0.463709522	0.024507397	0.202459656	8.69E-06	8.86E-05	10	0	0
OR5M11	0.7079925	0.795967964	-0.08797551	-0.17424164	8.70E-06	8.86E-05	5	0	0
THBS2	0.3599028	0.299527375	0.060375402	0.706600203	8.71E-06	8.87E-05	13	0	0
PPP3R2	0.614567	0.739424146	-0.12485718	-0.26615043	8.75E-06	8.90E-05	4	0	0
FCRL3	0.5617337	0.696851245	-0.13511754	-0.31777453	8.77E-06	8.91E-05	8	0	0
TRIML1	0.6404761	0.65876085	-0.01828477	-0.00095117	8.77E-06	8.91E-05	7	0	0
EPB41L4B	0.2971024	0.252260276	0.044842151	0.092567634	8.78E-06	8.92E-05	3	0	0
TMTC1	0.6043719	0.66869932	-0.06432743	-0.19875044	8.82E-06	8.95E-05	8	0	0
GAD2	0.1618598	0.092015793	0.069844032	0.80470168	8.86E-06	8.97E-05	14	0	0
HRASLS5	0.255238	0.201329487	0.053908473	0.719771023	8.85E-06	8.97E-05	13	0	0
CNTNAP4	0.44704	0.536563757	-0.08952377	-0.18619713	8.85E-06	8.97E-05	5	0	0
BIRC8	0.6902729	0.779221756	-0.08894891	-0.19346603	8.86E-06	8.97E-05	7	0	0
GNAI1	0.4786105	0.586221764	-0.10761129	-0.25098008	8.90E-06	9.01E-05	4	0	0
AGO1	0.6941857	0.836060535	-0.14187485	-0.26820039	8.96E-06	9.06E-05	6	0	0
TNFRSF17	0.7776203	0.850748901	-0.07312864	-0.14317226	8.97E-06	9.07E-05	4	0	0
DEFB129	0.7415962	0.850289346	-0.10869314	-0.19474957	8.97E-06	9.07E-05	2	0	0
KRT80	0.4788379	0.553280146	-0.07444223	-0.18830752	8.99E-06	9.08E-05	8	0	0
ASTN1	0.1498695	0.088628922	0.061240562	0.731541016	9.05E-06	9.13E-05	14	0	0
HEMGN	0.716984	0.821397998	-0.10441396	-0.19316298	9.05E-06	9.13E-05	3	0	0
SLC18A3	0.1796736	0.106147682	0.073525935	0.721911219	9.04E-06	9.13E-05	10	0	0
EFCAB1	0.2822238	0.332063825	-0.04984002	-0.17062734	9.06E-06	9.13E-05	11	0	0
ANKRD13B	0.1717402	0.217575928	-0.04583576	-0.21162001	9.06E-06	9.13E-05	6	0	0
NRSN1	0.5809764	0.632716373	-0.05173996	0.037245065	9.08E-06	9.14E-05	8	0	0
ZNF124	0.4949832	0.552279346	-0.05729611	-0.27825398	9.10E-06	9.15E-05	3	0	0

CLEC4A	0.6131099	0.702896845	-0.08978695	-0.20082199	9.13E-06	9.17E-05	7	0	0
WDR93	0.1289686	0.149419978	-0.02045138	-0.21047478	9.12E-06	9.17E-05	16	0	0
PRDM2	0.2280081	0.28825372	-0.06024558	-0.23774262	9.17E-06	9.21E-05	10	0	0
FAM24A	0.6574263	0.744269491	-0.08684318	-0.18052083	9.19E-06	9.22E-05	8	0	0
IGSF9	0.238895	0.305691463	-0.06679651	-0.26600163	9.24E-06	9.27E-05	7	0	0
TNFAIP8L2	0.3761093	0.420461224	-0.04435194	-0.16999674	9.25E-06	9.27E-05	6	0	0
HIST1H2BB	0.1953387	0.074289475	0.121049248	1.370639457	9.34E-06	9.36E-05	5	0	0
IQCK	0.743987	0.839584497	-0.09559745	-0.17977307	9.35E-06	9.36E-05	5	0	0
C2orf54	0.4122591	0.481087261	-0.06882817	-0.2177445	9.36E-06	9.37E-05	4	0	0
OR2H1	0.5268568	0.67284444	-0.1459876	-0.34705513	9.39E-06	9.39E-05	2	0	0
SCN4A	0.7089674	0.786048357	-0.07708092	-0.16598116	9.38E-06	9.39E-05	11	0	0
IL1RL2	0.6502886	0.728300515	-0.0780119	-0.18189866	9.47E-06	9.46E-05	10	0	0
ZNF534	0.6945774	0.786371648	-0.09179421	-0.18232836	9.48E-06	9.46E-05	2	0	0
UBALD1	0.3415178	0.407585199	-0.06606745	-0.14165127	9.51E-06	9.49E-05	8	0	0
SPATA31A3	0.8224062	0.880957983	-0.05855181	-0.09806973	9.56E-06	9.53E-05	1	0	0
DPPA2	0.6998917	0.828695257	-0.1288036	-0.24115195	9.60E-06	9.57E-05	8	0	0
LY75	0.1940502	0.232623604	-0.03857339	-0.28828074	9.63E-06	9.59E-05	11	0	0
OR2C3	0.6125373	0.729538184	-0.11700093	-0.25685126	9.65E-06	9.61E-05	4	0	0
ASB18	0.5595844	0.637648287	-0.07806391	-0.20623361	9.74E-06	9.69E-05	12	0	0
IGF1	0.5440755	0.428976565	0.11509895	0.549287094	9.78E-06	9.72E-05	5	0	0
CXCL13	0.693692	0.699735625	-0.00604366	0.018799419	9.79E-06	9.73E-05	5	0	0
BRCA2	0.20389	0.262114348	-0.05822433	-0.28330903	9.84E-06	9.77E-05	8	0	0
MAML1	0.1175949	0.145819051	-0.0282242	-0.17417771	9.85E-06	9.77E-05	6	0	0
CHRDL2	0.2405934	0.161132517	0.079460872	0.857640625	9.85E-06	9.77E-05	4	0	0
CACNA2D2	0.4559552	0.508065565	-0.05211033	-0.06982221	9.87E-06	9.79E-05	7	0	0
ENDOU	0.5751611	0.642277748	-0.0671166	-0.20860686	9.93E-06	9.84E-05	4	0	0
ASRGL1	0.3593811	0.416033925	-0.05665288	-0.02411636	9.97E-06	9.87E-05	9	0	0
EGFLAM	0.1722121	0.115459789	0.056752317	0.710676447	9.98E-06	9.88E-05	13	0	0
TMEM74B	0.2820099	0.314578901	-0.03256903	0.092672887	1.01E-05	9.96E-05	3	0	0
EBLN2	0.7964488	0.851651299	-0.05520252	-0.10255352	1.01E-05	1.00E-04	3	0	0
LYST	0.2681172	0.162197428	0.105919788	0.73144491	1.01E-05	0.000100085	3	0	0
AMY2A	0.7038061	0.77512793	-0.07132181	-0.14910695	1.02E-05	0.000100378	3	0	0
CD248	0.3368811	0.28160123	0.055279853	0.261863297	1.02E-05	0.000100378	10	0	0
KLRK1	0.5653535	0.677607776	-0.11225428	-0.31456586	1.02E-05	0.000100378	3	0	0
ZNF829	0.1905267	0.112351375	0.078175325	1.125516315	1.02E-05	0.000100408	10	0	0
MS4A6A	0.5951834	0.687658812	-0.09247545	-0.21197611	1.02E-05	0.000100676	7	0	0
ACSM4	0.699754	0.794180308	-0.09442632	-0.17937555	1.02E-05	0.000100676	4	0	0
IL1R2	0.6024174	0.690406004	-0.08798862	-0.21047424	1.02E-05	0.000100827	8	0	0
AQP10	0.4207117	0.498352888	-0.07764114	-0.27201214	1.03E-05	0.000100937	3	0	0
LCN2	0.5399788	0.622538741	-0.0825599	-0.22425654	1.03E-05	0.000100937	4	0	0
GPR25	0.6769333	0.620275568	0.056657722	0.161151706	1.03E-05	0.000101217	7	0	0
HTN1	0.7114174	0.794311048	-0.0828937	-0.15691942	1.03E-05	0.000101369	1	0	0
LYN	0.1361149	0.181173986	-0.04505909	-0.26715231	1.03E-05	0.000101429	10	0	0
ITGA2B	0.5519659	0.642880872	-0.09091493	-0.22927387	1.03E-05	0.000101429	6	0	0
WDR88	0.8371074	0.820740828	0.016366549	0.037382895	1.03E-05	0.000101429	10	0	0
NCAM2	0.2282797	0.146122776	0.082156928	0.608799312	1.03E-05	0.000101429	11	0	0
FREM3	0.2089926	0.221834693	-0.01284213	0.171775688	1.04E-05	0.000101493	11	0	0
FHDC1	0.1097795	0.148741502	-0.03896196	-0.09498794	1.04E-05	0.000101493	6	0	0
AZGP1	0.6127881	0.735523998	-0.12273592	-0.25769162	1.04E-05	0.000101493	5	0	0
CLEC2A	0.8358671	0.893255077	-0.05738798	-0.09577503	1.04E-05	0.00010178	5	0	0
DEFB110	0.6142878	0.70966773	-0.09537992	-0.20511967	1.05E-05	0.000102264	1	0	0
COL21A1	0.8517933	0.885916698	-0.03412339	-0.05719328	1.05E-05	0.000102557	2	0	0
TMEM154	0.2310279	0.267106086	-0.03607823	-0.03292131	1.06E-05	0.000103801	8	0	0
KIFAP3	0.6961187	0.80236086	-0.10624214	-0.21438634	1.07E-05	0.000104046	4	0	0
DAG1	0.7776504	0.758825432	0.018824991	0.070989864	1.07E-05	0.000104529	6	0	0
OR8D4	0.5787336	0.695418299	-0.11668471	-0.26734568	1.07E-05	0.000104529	5	0	0
LRRIQ4	0.7022758	0.831006167	-0.12873035	-0.24455591	1.07E-05	0.000104579	3	0	0
F13B	0.6082769	0.700924767	-0.09264791	-0.19582824	1.08E-05	0.000104769	2	0	0
FAM71F1	0.8086176	0.873016937	-0.06439933	-0.10997593	1.08E-05	0.000104829	4	0	0
RFPL1	0.6754408	0.763212448	-0.08777166	-0.17383281	1.09E-05	0.000105766	1	0	0
PLA2G2A	0.6245214	0.666741892	-0.04222047	-0.08007901	1.09E-05	0.000105781	6	0	0
OR6C6	0.4261078	0.538663193	-0.11255543	-0.33123614	1.09E-05	0.00010587	1	0	0
ATP4A	0.5865247	0.678637058	-0.0921124	-0.23422734	1.10E-05	0.000106691	8	0	0

SKOR1	0.4545543	0.45243508	0.002119179	0.136760225	1.10E-05	0.000106873	8	0	0
OR2A14	0.6872229	0.7764027	-0.08917978	-0.18280901	1.10E-05	0.00010691	2	0	0
H3F3B	0.4641294	0.608075161	-0.14394574	-0.38250137	1.11E-05	0.000107714	1	0	0
BTC	0.4232107	0.481904764	-0.05869411	-0.18902547	1.11E-05	0.000107726	5	0	0
GPR45	0.7413752	0.823895356	-0.08252014	-0.15230415	1.11E-05	0.000107888	13	0	0
CACNG3	0.5066133	0.601283099	-0.09466977	-0.15808469	1.12E-05	0.000107931	4	0	0
PGAP1	0.3080167	0.367406652	-0.05938992	-0.34497278	1.12E-05	0.000108046	6	0	0
ASB15	0.7756807	0.845877073	-0.07019635	-0.13312648	1.12E-05	0.000108053	4	0	0
TEKT3	0.2567599	0.182992569	0.073767361	1.157691146	1.12E-05	0.000108298	10	0	0
SALL2	0.477847	0.437232996	0.040614006	0.507790716	1.12E-05	0.000108308	5	0	0
KRTAP9-3	0.7760455	0.845109799	-0.0690643	-0.12236528	1.13E-05	0.000108656	4	0	0
REM2	0.3446405	0.266335426	0.078305067	0.372821024	1.13E-05	0.000109017	7	0	0
ZBED6CL	0.7453199	0.689155971	0.056163897	0.122593925	1.13E-05	0.000109305	4	0	0
OLFM4	0.5822268	0.651216009	-0.06898922	-0.18099342	1.14E-05	0.000109415	6	0	0
MAS1	0.7985196	0.878165845	-0.07964628	-0.13714228	1.14E-05	0.000109541	4	0	0
KRTAP15-1	0.5935393	0.697343115	-0.10380383	-0.23540102	1.14E-05	0.000109704	6	0	0
PLET1	0.7947238	0.850879963	-0.05615619	-0.09981775	1.14E-05	0.000109759	5	0	0
RNF175	0.414142	0.415623272	-0.00148129	0.358462417	1.14E-05	0.000110001	8	0	0
PLXNA4	0.7694212	0.849992911	-0.08057175	-0.14458377	1.15E-05	0.000110064	9	0	0
TRIM59	0.6265841	0.772442207	-0.14585815	-0.29876569	1.15E-05	0.000110462	2	0	0
SNED1	0.2071253	0.137066233	0.070059034	0.35755058	1.15E-05	0.000110527	8	0	0
AGMO	0.5836943	0.653182759	-0.0694885	-0.12516746	1.15E-05	0.000110744	4	0	0
HIST1H2BE	0.1892198	0.078690843	0.110528984	1.195695323	1.16E-05	0.000111086	9	0	0
STK33	0.1475601	0.164770636	-0.0172105	-0.23160901	1.16E-05	0.000111152	17	0	0
PRG3	0.6320249	0.77131164	-0.13928671	-0.28327907	1.16E-05	0.0001116	2	0	0
MTHFD2	0.1428699	0.15929424	-0.01642433	-0.26321224	1.17E-05	0.000111817	8	0	0
ALPK3	0.6871638	0.684604602	0.002559195	0.034125674	1.17E-05	0.000112419	7	0	0
OR2T3	0.540973	0.689852003	-0.14887902	-0.3450683	1.18E-05	0.000112578	1	0	0
OR2AG1	0.5867205	0.683466957	-0.09674642	-0.23890431	1.18E-05	0.000112578	4	0	0
PRSS23	0.7884146	0.86256664	-0.074152	-0.12812715	1.18E-05	0.000112578	1	0	0
PIWIL4	0.3109183	0.347057624	-0.03613936	-0.17293287	1.18E-05	0.000112578	10	0	0
FUT4	0.3109183	0.347057624	-0.03613936	-0.17293287	1.18E-05	0.000112578	10	0	0
DIO3	0.2357	0.149940701	0.08575926	0.834700244	1.18E-05	0.000112578	24	0	0
ZNF548	0.6004442	0.659863199	-0.05941899	-0.06702999	1.18E-05	0.000112578	4	0	0
ZNF831	0.7709413	0.833699298	-0.06275798	-0.1130675	1.18E-05	0.000112578	10	0	0
ERG	0.3649919	0.413241052	-0.04824916	-0.06168089	1.19E-05	0.000112908	5	0	0
ACER1	0.6154206	0.707222686	-0.09180207	-0.20133932	1.19E-05	0.000113145	7	0	0
TM4SF1	0.3297922	0.397000061	-0.06720789	-0.26618955	1.19E-05	0.000113291	10	0	0
MAPK12	0.1268041	0.181126831	-0.05432272	-0.05689101	1.19E-05	0.000113291	8	0	0
LYG1	0.7460875	0.806678934	-0.06059143	-0.11483406	1.19E-05	0.000113303	2	0	0
TBC1D26	0.8282677	0.877817537	-0.04954988	-0.08285223	1.19E-05	0.000113377	1	0	0
MUC15	0.5740119	0.691731576	-0.11771971	-0.28090149	1.20E-05	0.000114105	8	0	0
GCM1	0.8191552	0.879060351	-0.05990512	-0.10409868	1.20E-05	0.000114161	7	0	0
POU2F3	0.413491	0.485631001	-0.07214	-0.26934131	1.21E-05	0.000114365	5	0	0
FGF10	0.1828074	0.099121736	0.083685649	0.942112006	1.21E-05	0.000114644	4	0	0
CCL4	0.5131842	0.622851504	-0.1096673	-0.26355887	1.22E-05	0.000115101	2	0	0
FAM83C	0.6586517	0.703123294	-0.04447164	-0.08051464	1.22E-05	0.000115101	7	0	0
GRAMD1A	0.6403974	0.576618435	0.063778999	0.149755309	1.23E-05	0.000116284	3	0	0
MTRNR2L7	0.6965284	0.760110132	-0.0635817	-0.12711381	1.23E-05	0.00011633	3	0	0
SULT1A1	0.288719	0.355681252	-0.06696222	-0.02884095	1.23E-05	0.000116497	3	0	0
C2orf16	0.7621233	0.845422776	-0.08329947	-0.14785831	1.24E-05	0.00011745	6	0	0
SLC26A7	0.6820937	0.7752487	-0.09315497	-0.1889714	1.25E-05	0.000117629	5	0	0
CPSF4L	0.5753977	0.679863423	-0.10446573	-0.22951522	1.25E-05	0.000117629	10	0	0
SH2D1B	0.5572206	0.657825938	-0.10060533	-0.31671461	1.25E-05	0.000117671	5	0	0
CACNA1E	0.9500981	0.981816276	-0.03171813	-0.04689101	1.26E-05	0.000118408	1	0	0
SELE	0.7031982	0.803238583	-0.10004036	-0.22574841	1.26E-05	0.000118615	2	0	0
NTM	0.6032577	0.680584875	-0.07732713	-0.14279704	1.27E-05	0.000119502	8	0	0
IZUMO2	0.4220597	0.368591217	0.053468438	0.402718838	1.27E-05	0.000119635	8	0	0
CYP27A1	0.2341389	0.147491238	0.086647672	0.867785103	1.27E-05	0.000119637	9	0	0
RETNLB	0.6452571	0.746852207	-0.1015951	-0.22438473	1.27E-05	0.000119637	7	0	0
COX8C	0.7746379	0.844625514	-0.06998766	-0.12416542	1.27E-05	0.000119727	7	0	0
TNFRSF9	0.444223	0.52071478	-0.07649179	-0.13510239	1.28E-05	0.00012004	5	0	0
RFTN2	0.6767816	0.641075037	0.035706525	0.107354663	1.28E-05	0.00012005	8	0	0

EMG1	0.434797	0.54879017	-0.11399313	-0.35814514	1.28E-05	0.00012005	5	0	0
ELSPBP1	0.4726491	0.589465481	-0.1168164	-0.3076793	1.28E-05	0.000120062	3	0	0
SLC16A3	0.398731	0.452573789	-0.05384282	-0.16935893	1.28E-05	0.000120376	11	0	0
CWH43	0.3024176	0.224973015	0.077444608	0.623170793	1.29E-05	0.000120386	15	0	0
NEFM	0.4021391	0.322628493	0.079510633	0.684700783	1.29E-05	0.000121182	6	0	0
MGMT	0.4345756	0.472820862	-0.03824529	0.050909535	1.30E-05	0.000121182	19	0	0
NELL1	0.1859299	0.094390402	0.091539499	1.064859853	1.30E-05	0.000121253	13	0	0
SLC5A2	0.7533705	0.803645466	-0.05027496	-0.09561906	1.30E-05	0.00012156	8	0	0
HBB	0.7379548	0.826793332	-0.0888385	-0.1619202	1.30E-05	0.000121826	1	0	0
KRTAP24-1	0.4945174	0.596991831	-0.10247447	-0.2735471	1.30E-05	0.000121826	6	0	0
RXFP2	0.7834779	0.848001205	-0.06452335	-0.11168312	1.31E-05	0.000121842	3	0	0
DPP9	0.3727559	0.466399841	-0.0936439	-0.09798996	1.31E-05	0.000121865	2	0	0
ISLR	0.6912019	0.629254856	0.061947079	0.137252931	1.31E-05	0.000122023	6	0	0
PADI2	0.1502614	0.187701944	-0.03744054	-0.19472342	1.31E-05	0.000122152	13	0	0
VWCE	0.3553239	0.27801774	0.077306148	0.881507852	1.31E-05	0.000122152	12	0	0
SYCP1	0.565725	0.531230164	0.03449485	0.271476063	1.31E-05	0.000122189	11	0	0
OR5L2	0.4736845	0.558682061	-0.08499758	-0.20246726	1.31E-05	0.000122233	3	0	0
OR5P2	0.6126577	0.730913464	-0.11825576	-0.25086577	1.32E-05	0.00012252	1	0	0
SLC10A2	0.6528679	0.747973175	-0.09510529	-0.19318356	1.32E-05	0.000122644	5	0	0
VWA7	0.3674742	0.404897229	-0.03742307	-0.25058735	1.32E-05	0.000122779	11	0	0
MS4A6E	0.6368581	0.725732432	-0.08887432	-0.19220678	1.32E-05	0.000122779	3	0	0
SLC16A1	0.2393517	0.344697762	-0.10534611	-0.52012163	1.33E-05	0.000123503	2	0	0
ADAMTSL1	0.3650428	0.361210386	0.003832457	0.354667495	1.33E-05	0.000123533	8	0	0
GPR142	0.5772185	0.673668568	-0.09645008	-0.22360004	1.34E-05	0.000123834	7	0	0
ZNF568	0.2237363	0.16303028	0.060705971	0.998580128	1.34E-05	0.000124261	11	0	0
PTPRN	0.3910509	0.364305414	0.02674553	0.557073833	1.34E-05	0.000124327	11	0	0
LINGO4	0.4912167	0.57757799	-0.08636125	-0.24733351	1.34E-05	0.000124436	5	0	0
TKTL2	0.7722575	0.858603845	-0.0863463	-0.15502777	1.35E-05	0.000124436	13	0	0
KRTAP9-2	0.7892964	0.868060501	-0.07876409	-0.13859151	1.35E-05	0.000124979	3	0	0
PCDHA13	0.546409	0.451195198	0.09521384	0.427266436	1.37E-05	0.000126064	9	0	0
SGCD	0.6319798	0.75073645	-0.11875664	-0.25245864	1.37E-05	0.000126064	3	0	0
OR10C1	0.5429802	0.672942255	-0.12996207	-0.30453582	1.36E-05	0.000126064	1	0	0
KRTAP25-1	0.6287639	0.722922436	-0.09415849	-0.20536828	1.37E-05	0.000126064	5	0	0
M1AP	0.5707287	0.506527328	0.064201387	0.223425437	1.37E-05	0.000126088	12	0	0
HAPLN4	0.4435054	0.432771882	0.010733476	0.180480915	1.37E-05	0.00012621	9	0	0
SMR3B	0.5809338	0.705096358	-0.12416255	-0.27514112	1.38E-05	0.000126759	1	0	0
DNAJB6	0.6476262	0.723554337	-0.07592811	-0.15763599	1.38E-05	0.000126785	1	0	0
RERG	0.6301727	0.753723579	-0.12355088	-0.2552645	1.38E-05	0.000127069	4	0	0
TCHHL1	0.6311824	0.738144106	-0.10696169	-0.24386135	1.38E-05	0.000127242	5	0	0
HEPHL1	0.6233014	0.693685176	-0.07038376	-0.18343114	1.39E-05	0.000127456	8	0	0
MAT1A	0.4852506	0.542245096	-0.05699445	-0.23896036	1.39E-05	0.00012782	9	0	0
MYPN	0.6576624	0.725185393	-0.06752299	-0.14531316	1.39E-05	0.000127845	6	0	0
IL18	0.4371572	0.513520848	-0.07636366	-0.30541877	1.39E-05	0.000127845	8	0	0
GLCE	0.1153537	0.158575269	-0.0432216	-0.24079384	1.39E-05	0.000127845	9	0	0
TTPAL	0.1637343	0.199016597	-0.03528227	-0.22457034	1.39E-05	0.000127845	7	0	0
CLN8	0.3160853	0.381376345	-0.06529107	-0.37306646	1.40E-05	0.000128205	3	0	0
CDR2L	0.3679089	0.425831866	-0.05792297	-0.16710154	1.40E-05	0.000128205	8	0	0
LSMEM2	0.6897111	0.717339094	-0.02762798	-0.05370108	1.41E-05	0.000128699	8	0	0
MPZL3	0.162218	0.19149886	-0.02928089	-0.29692905	1.41E-05	0.000129079	16	0	0
OR6C74	0.5280034	0.64826262	-0.12025919	-0.28566506	1.41E-05	0.000129079	3	0	0
FOXG1	0.2951467	0.1838918	0.111254889	0.683625851	1.42E-05	0.000129704	14	0	0
IPCEF1	0.6055194	0.691169944	-0.08565054	-0.19156249	1.42E-05	0.000130161	5	0	0
CD8B	0.3819349	0.313555641	0.068379235	0.380957657	1.43E-05	0.000130503	3	0	0
OR5C1	0.8592794	0.914193258	-0.05491385	-0.09033578	1.44E-05	0.000131397	4	0	0
CD1B	0.4660941	0.578753197	-0.11265905	-0.23793311	1.44E-05	0.000131543	3	0	0
ABTB1	0.3167172	0.350510214	-0.03379298	-0.14637622	1.44E-05	0.000131611	7	0	0
RIN2	0.4307568	0.363279178	0.067477635	0.277440482	1.45E-05	0.000131879	10	0	0
JUND	0.2680764	0.228740524	0.039335861	0.193608879	1.45E-05	0.000132078	3	0	0
ZNF730	0.3941265	0.428597493	-0.03447103	0.348480041	1.45E-05	0.000132282	2	0	0
SLC22A5	0.1010003	0.132423182	-0.03142284	-0.29194656	1.46E-05	0.00013257	7	0	0
DENND3	0.3964162	0.478423312	-0.08200713	-0.21170689	1.46E-05	0.000132637	2	0	0
CERKL	0.1678712	0.085323336	0.082547894	0.995363152	1.46E-05	0.000132666	14	0	0
NEUROD1	0.1678712	0.085323336	0.082547894	0.995363152	1.46E-05	0.000132666	14	0	0

GLP2R	0.6739402	0.744136248	-0.07019603	-0.14569816	1.48E-05	0.000134454	2	0	0
OR5M9	0.6393388	0.722626742	-0.08328792	-0.18943909	1.48E-05	0.000134506	2	0	0
OR2T1	0.7573718	0.858445776	-0.10107402	-0.18726058	1.49E-05	0.000134743	6	0	0
NEUROG1	0.2256562	0.158357932	0.067298287	0.584189444	1.49E-05	0.000134743	22	0	0
ZNF229	0.3521204	0.315082723	0.037037663	0.708577757	1.49E-05	0.000134792	16	0	0
TPSD1	0.4526694	0.515930071	-0.06326065	-0.18488925	1.49E-05	0.000135184	1	0	0
CXCL6	0.1860798	0.114372746	0.071707009	0.766959592	1.50E-05	0.000135732	5	0	0
FAM81B	0.7183088	0.751367807	-0.03305902	-0.05706242	1.50E-05	0.000135732	4	0	0
LRRC3B	0.2071009	0.130368183	0.076732675	0.647547672	1.50E-05	0.000136	11	0	0
RAB26	0.7606515	0.658996737	0.101654802	0.204079499	1.51E-05	0.00013619	1	0	0
AKR1E2	0.8438193	0.901853743	-0.05803444	-0.09487166	1.51E-05	0.000136314	1	0	0
OR2A12	0.7411557	0.816896755	-0.07574101	-0.13815859	1.51E-05	0.000136673	3	0	0
PCDHGB3	0.3499164	0.233974189	0.115942257	0.713686185	1.52E-05	0.00013748	11	0	0
C8B	0.619341	0.707890598	-0.0885496	-0.20436005	1.53E-05	0.000137642	6	0	0
TRIM49B	0.7048811	0.826327955	-0.1214469	-0.22636556	1.53E-05	0.000137647	1	0	0
PRAMEF12	0.7316873	0.813494278	-0.08180701	-0.15048597	1.53E-05	0.00013811	4	0	0
OSTM1	0.2404191	0.131406907	0.109012158	0.882615862	1.53E-05	0.000138131	12	0	0
ERI1	0.5579646	0.619284532	-0.06131994	-0.18786199	1.53E-05	0.000138131	5	0	0
ARL14	0.6133659	0.723577077	-0.11021119	-0.22278216	1.54E-05	0.000138454	4	0	0
COX7A1	0.7430795	0.663197149	0.079882359	0.2269743	1.54E-05	0.000138454	8	0	0
OR6V1	0.6382992	0.725899341	-0.08760014	-0.17543517	1.54E-05	0.000138718	6	0	0
OR2T8	0.6411115	0.761455731	-0.12034428	-0.25426917	1.55E-05	0.000139011	4	0	0
PRR23C	0.6919361	0.626237706	0.0656984	0.177827834	1.55E-05	0.000139123	10	0	0
TRAPPC2L	0.5253097	0.583948239	-0.0586385	-0.16651266	1.55E-05	0.000139187	5	0	0
OR1K1	0.7172083	0.783021038	-0.0658127	-0.12808753	1.55E-05	0.000139364	4	0	0
PTPRR	0.2962062	0.300572254	-0.0043661	0.305282738	1.55E-05	0.00013937	13	0	0
VKORC1L1	0.038806	0.03393601	0.00486998	0.146828796	1.56E-05	0.0001396	2	0	0
ZNF556	0.6710535	0.771136828	-0.10008337	-0.20912472	1.56E-05	0.0001396	9	0	0
CPA3	0.640656	0.768135359	-0.12747938	-0.26921844	1.56E-05	0.000139777	4	0	0
SEMA4B	0.5954907	0.709596247	-0.11410552	-0.25428641	1.56E-05	0.000139777	3	0	0
ZSCAN10	0.4226356	0.375079365	0.047556245	0.295746277	1.57E-05	0.00014011	9	0	0
PRG2	0.6041462	0.724627251	-0.12048101	-0.27286667	1.57E-05	0.000140137	6	0	0
SLC23A3	0.5626309	0.56726165	-0.00463072	-0.06554826	1.57E-05	0.000140164	4	0	0
KRTAP10-4	0.7081116	0.78238513	-0.07427349	-0.14233786	1.57E-05	0.000140342	4	0	0
DCPS	0.2557987	0.329988455	-0.07418971	-0.29037335	1.57E-05	0.000140493	12	0	0
GLYATL2	0.3762274	0.327641193	0.048586201	0.678498588	1.58E-05	0.000140705	3	0	0
GLYATL1	0.3762274	0.327641193	0.048586201	0.678498588	1.58E-05	0.000140705	3	0	0
CCDC166	0.6523549	0.606525212	0.04582964	0.137219194	1.58E-05	0.000140735	12	0	0
DRGX	0.1855257	0.106416401	0.079109249	0.756542358	1.58E-05	0.00014084	9	0	0
RAB3IL1	0.6510525	0.737907002	-0.08685453	-0.18107477	1.59E-05	0.000141612	6	0	0
PCDHGA11	0.4038059	0.300646117	0.103159776	0.565404426	1.59E-05	0.000141792	10	0	0
KCNA7	0.3077221	0.387404619	-0.07968254	-0.15236673	1.59E-05	0.000141792	6	0	0
MAB21L2	0.9033489	0.879375384	0.023973506	0.039698453	1.60E-05	0.000141851	11	0	0
HABP2	0.7205541	0.82446432	-0.10391024	-0.19302886	1.60E-05	0.000141851	2	0	0
KRTAP5-3	0.5479638	0.625208671	-0.07724486	-0.234624	1.60E-05	0.000142142	3	0	0
MYH8	0.6544076	0.752781501	-0.09837393	-0.21994952	1.60E-05	0.000142345	4	0	0
AATK	0.2805503	0.334631081	-0.05408081	-0.22286417	1.61E-05	0.000142504	7	0	0
APOA4	0.648904	0.709848378	-0.06094435	-0.12649853	1.62E-05	0.000143467	5	0	0
KRT34	0.745827	0.813292754	-0.0674658	-0.1292539	1.62E-05	0.000143925	3	0	0
REEP3	0.166063	0.205662897	-0.03959993	-0.24070982	1.63E-05	0.000144137	10	0	0
KLRC1	0.5998696	0.680404732	-0.08053515	-0.17732631	1.63E-05	0.000144603	5	0	0
UBR7	0.075579	0.10878327	-0.03320423	-0.17805026	1.63E-05	0.000144603	10	0	0
PCDHGA9	0.4841961	0.385318675	0.098877438	0.463822829	1.64E-05	0.000145097	6	0	0
VN1R4	0.5080237	0.622026447	-0.11400272	-0.2852037	1.64E-05	0.000145097	3	0	0
FOXI1	0.5319937	0.631535973	-0.09954227	-0.24323157	1.65E-05	0.000145565	12	0	0
HIST1H1A	0.5766241	0.47712985	0.099494259	0.446663442	1.65E-05	0.000145782	10	0	0
TAAR5	0.5375316	0.662831273	-0.12529969	-0.30880899	1.66E-05	0.000146602	3	0	0
SNTB1	0.7733682	0.856330666	-0.08296246	-0.14725893	1.66E-05	0.000146794	5	0	0
MT4	0.705842	0.772434112	-0.06659211	-0.13631852	1.67E-05	0.000146963	7	0	0
ZNF486	0.267015	0.168585448	0.098429509	1.13796948	1.67E-05	0.000146963	6	0	0
CSTA	0.4960898	0.600849715	-0.10475996	-0.29002947	1.67E-05	0.000147487	8	0	0
ITSN2	0.3257097	0.370868588	-0.04515885	-0.13720311	1.68E-05	0.000147965	7	0	0
CDKN1A	0.8203103	0.875046692	-0.05473643	-0.0906539	1.69E-05	0.000148455	3	0	0

SMOC2	0.2367367	0.172951543	0.063785117	0.464730348	1.70E-05	0.000149844	8	0	0
TMEM168	0.2045681	0.254722782	-0.05015465	-0.25312161	1.71E-05	0.000150073	9	0	0
RGS14	0.4178988	0.485562927	-0.06766413	-0.26228435	1.71E-05	0.000150074	12	0	0
CLDN5	0.7316023	0.691486437	0.040115824	0.090650875	1.71E-05	0.000150074	4	0	0
KLHL28	0.7718902	0.824528858	-0.05263861	-0.09587522	1.71E-05	0.000150565	2	0	0
ANKFN1	0.5160547	0.642602407	-0.12654768	-0.31099141	1.73E-05	0.000151585	1	0	0
OR52K1	0.7347069	0.826772005	-0.09206509	-0.17063198	1.73E-05	0.000151696	4	0	0
CCR8	0.7095906	0.77852944	-0.06893883	-0.14299405	1.73E-05	0.000151722	2	0	0
USP50	0.6395692	0.724597764	-0.08502855	-0.18858302	1.73E-05	0.000151737	8	0	0
SLC25A24	0.1275602	0.15114468	-0.02358448	-0.24431125	1.74E-05	0.000152302	11	0	0
MTHFD1L	0.3479171	0.376885386	-0.02896832	-0.08355884	1.74E-05	0.000152302	3	0	0
PKD1L3	0.6969793	0.792558023	-0.09557876	-0.17970206	1.74E-05	0.000152302	4	0	0
NUMBL	0.1359111	0.164155468	-0.0282444	-0.38011224	1.74E-05	0.000152302	8	0	0
HMGN2	0.1374064	0.179526499	-0.04212013	-0.1852853	1.75E-05	0.000153069	13	0	0
CXCR5	0.6500813	0.713774713	-0.06369337	-0.11313328	1.75E-05	0.000153069	8	0	0
KIF19	0.2701445	0.183258232	0.086886246	1.094154083	1.75E-05	0.000153069	7	0	0
AKR1B15	0.4468799	0.512526596	-0.06564667	-0.22126234	1.75E-05	0.000153079	8	0	0
GAST	0.5979702	0.674648698	-0.07667851	-0.22766489	1.75E-05	0.000153079	5	0	0
ROPN1B	0.7532046	0.856261891	-0.10305729	-0.18273339	1.75E-05	0.000153086	1	0	0
HTR1D	0.6724207	0.757271003	-0.08485028	-0.18318462	1.76E-05	0.00015387	8	0	0
SLC6A16	0.8116521	0.871554602	-0.05990253	-0.10353494	1.78E-05	0.000154736	12	0	0
GPR139	0.184003	0.119393985	0.06460899	0.787839441	1.79E-05	0.000155986	9	0	0
TREX1	0.709033	0.658641233	0.050391752	0.155602168	1.80E-05	0.000156724	9	0	0
RIPK3	0.2903423	0.34632787	-0.05598553	-0.32406233	1.80E-05	0.000156781	7	0	0
AP1S3	0.3289286	0.360225848	-0.03129727	-0.04331984	1.80E-05	0.000156817	5	0	0
TUFT1	0.0770027	0.097395508	-0.02039278	-0.24732429	1.80E-05	0.000156818	6	0	0
G6PC2	0.644745	0.738182801	-0.09343776	-0.20823691	1.81E-05	0.000156861	4	0	0
AVP	0.6361329	0.697736742	-0.06160387	-0.12949994	1.81E-05	0.000156861	10	0	0
MAP1LC3C	0.6388638	0.670626861	-0.03176309	0.080189659	1.81E-05	0.000157424	6	0	0
RPGRIP1	0.8331516	0.829073456	0.004078125	0.010825561	1.82E-05	0.000157638	7	0	0
MPG	0.333037	0.274777022	0.058259966	0.299676462	1.82E-05	0.000158185	9	0	0
HOXD10	0.2310625	0.150688285	0.080374171	0.674519005	1.83E-05	0.000158802	11	0	0
TPM3	0.5145611	0.588792028	-0.07423095	-0.19466743	1.85E-05	0.000159803	3	0	0
NKX2-5	0.2589755	0.19797589	0.060999602	0.395390159	1.84E-05	0.000159803	12	0	0
OR10H4	0.7148818	0.79184895	-0.07696716	-0.15002138	1.85E-05	0.000159914	2	0	0
SNTN	0.7996642	0.853956777	-0.05429254	-0.09745452	1.85E-05	0.000160268	2	0	0
CAPN13	0.7528774	0.842656564	-0.08977916	-0.16581358	1.85E-05	0.00016038	4	0	0
GSC	0.3285407	0.242881295	0.085659377	0.515681616	1.86E-05	0.000160467	7	0	0
PSMA8	0.754962	0.704607357	0.05035467	0.111909732	1.86E-05	0.000160715	10	0	0
NAA40	0.035843	0.050207122	-0.01436409	-0.33403713	1.86E-05	0.000160716	7	0	0
RCAN3	0.0903577	0.110862092	-0.02050436	-0.19823001	1.86E-05	0.00016097	14	0	0
IFNL3	0.8619275	0.94109604	-0.07916858	-0.12538291	1.87E-05	0.000160988	1	0	0
KRTAP19-2	0.7998761	0.868753609	-0.06887755	-0.1175036	1.87E-05	0.000160988	2	0	0
SLC22A3	0.4875461	0.468787388	0.018758706	0.396160201	1.87E-05	0.000161168	5	0	0
ENPP7	0.7127197	0.774563615	-0.06184395	-0.12054679	1.87E-05	0.000161511	3	0	0
ZNF595	0.4612022	0.502399844	-0.04119768	-0.13497698	1.88E-05	0.00016173	9	0	0
PKIG	0.4539938	0.50879032	-0.05479648	-0.1726549	1.88E-05	0.000161753	10	0	0
KRTAP10-5	0.6107109	0.70146404	-0.09075318	-0.19447799	1.89E-05	0.000162414	4	0	0
NADK	0.3865433	0.455738303	-0.06919499	-0.3300085	1.90E-05	0.000163653	10	0	0
KRTAP5-7	0.6418936	0.747339826	-0.10544623	-0.21630428	1.90E-05	0.000163653	1	0	0
C17orf67	0.7891208	0.764186308	0.024934487	0.048990713	1.91E-05	0.000163692	4	0	0
ANKRD60	0.3244084	0.306792748	0.017615642	0.649402643	1.91E-05	0.000163692	8	0	0
PCDHA10	0.3816745	0.317502762	0.064171767	0.362692212	1.91E-05	0.000163796	13	0	0
ZIC4	0.3398138	0.23087063	0.108943183	0.742659291	1.92E-05	0.00016467	19	0	0
TCF24	0.2450531	0.139936272	0.105116863	1.253993494	1.93E-05	0.000165232	11	0	0
SFI1	0.517126	0.601004507	-0.0838785	-0.20534352	1.93E-05	0.000165232	5	0	0
C9orf24	0.7533288	0.801959835	-0.04863105	-0.09056185	1.93E-05	0.000165257	3	0	0
THOC3	0.7349941	0.783999379	-0.04900531	-0.09190894	1.94E-05	0.000166026	1	0	0
CARTPT	0.2512209	0.194737828	0.056483057	0.519350663	1.95E-05	0.000166282	10	0	0
SCGB1C1	0.4436042	0.502382673	-0.05877842	-0.0538158	1.95E-05	0.000166282	9	0	0
OR11G2	0.7020371	0.789544399	-0.08750727	-0.1657978	1.94E-05	0.000166282	7	0	0
TMPRSS3	0.8763449	0.908533379	-0.03218844	-0.05493523	1.94E-05	0.000166282	5	0	0
CTNNAL1	0.3221379	0.260599334	0.061538551	0.194124162	1.95E-05	0.000166319	10	0	0

SGIP1	0.1574917	0.090146369	0.067345376	0.877313219	1.95E-05	0.000166478	6	0	0
SPZ1	0.8199896	0.874777356	-0.0547878	-0.09222129	1.96E-05	0.000167037	1	0	0
IGSF23	0.7615105	0.843559243	-0.08204872	-0.15342809	1.96E-05	0.000167179	9	0	0
AGT	0.6994823	0.642911818	0.056570483	0.16488174	1.96E-05	0.000167464	7	0	0
KRTAP5-2	0.6046163	0.686513514	-0.08189721	-0.18057547	1.97E-05	0.000168204	4	0	0
IKBKE	0.2093974	0.265526908	-0.05612948	-0.2018396	1.98E-05	0.000168944	10	0	0
TMPRSS11E	0.5805391	0.672543693	-0.0920046	-0.22760636	1.99E-05	0.000169134	4	0	0
CSTL1	0.6636847	0.734186236	-0.0705015	-0.15383307	1.99E-05	0.000169134	4	0	0
ROBO3	0.1938969	0.117523213	0.076373736	0.828175871	1.99E-05	0.000169207	14	0	0
TREM2	0.5953898	0.670272586	-0.07488279	-0.17749785	2.00E-05	0.000170007	8	0	0
HAPLN2	0.4750353	0.516980934	-0.04194565	-0.21304923	2.00E-05	0.000170079	8	0	0
NAV2	0.7212585	0.786885789	-0.0656273	-0.12767094	2.00E-05	0.000170178	8	0	0
AKTIP	0.2052213	0.156747934	0.0484734	0.365592919	2.01E-05	0.000170287	2	0	0
PRKAR1A	0.2056655	0.256490228	-0.05082476	-0.22042291	2.01E-05	0.000170287	10	0	0
DIO1	0.541766	0.606864996	-0.06509896	-0.13030716	2.03E-05	0.000171843	4	0	0
TRIM42	0.6142639	0.71476392	-0.10050002	-0.21349301	2.03E-05	0.000171914	6	0	0
HSPA8	0.1860988	0.183152594	0.002946202	-0.11807208	2.04E-05	0.000173234	4	0	0
SLC6A18	0.6133879	0.71583462	-0.10244668	-0.23262451	2.05E-05	0.000173534	10	0	0
PRCC	0.0888549	0.119688367	-0.03083344	-0.24179812	2.06E-05	0.000174441	6	0	0
PLXND1	0.2958564	0.255352029	0.040504407	0.103850479	2.06E-05	0.000174441	7	0	0
ARHGDIG	0.622554	0.744364766	-0.12181079	-0.23957943	2.06E-05	0.000174552	3	0	0
CLCF1	0.2476196	0.295098671	-0.04747904	-0.24992266	2.08E-05	0.000175501	10	0	0
OR5V1	0.6226045	0.695170669	-0.0725662	-0.1681352	2.09E-05	0.000176119	4	0	0
CYP4F11	0.3235126	0.378908261	-0.05539565	-0.22601614	2.09E-05	0.000176119	2	0	0
EDNRA	0.4257304	0.363765887	0.061964533	0.333230879	2.09E-05	0.000176317	11	0	0
SLC6A5	0.2320545	0.148348289	0.083706248	0.672172431	2.09E-05	0.000176317	11	0	0
ZSCAN4	0.8242824	0.893313716	-0.06903136	-0.11502898	2.09E-05	0.000176318	4	0	0
ZNF846	0.2390271	0.155489435	0.083537692	1.18102336	2.12E-05	0.000178353	3	0	0
KRTAP10-3	0.7454642	0.80677148	-0.06130733	-0.11513303	2.12E-05	0.000178353	5	0	0
ACTL8	0.4555357	0.502510163	-0.04697443	-0.17963302	2.12E-05	0.00017868	6	0	0
SAG	0.775653	0.740473414	0.035179568	0.081047866	2.12E-05	0.00017868	12	0	0
SHCBP1L	0.7671011	0.802961149	-0.03586008	-0.06850403	2.13E-05	0.000179295	11	0	0
LMTK3	0.1503421	0.181964354	-0.03162221	-0.24970813	2.13E-05	0.000179295	10	0	0
LRCH1	0.2792215	0.336115428	-0.05689388	-0.18377625	2.14E-05	0.000179609	6	0	0
OR2B6	0.6065039	0.714485347	-0.10798146	-0.23346544	2.15E-05	0.000180426	2	0	0
CYP3A4	0.7657865	0.834873516	-0.06908703	-0.12744524	2.15E-05	0.000180631	5	0	0
SPEG	0.4069123	0.318061728	0.088850597	0.645913442	2.16E-05	0.000181333	13	0	0
TNNT2	0.6235388	0.683962063	-0.06042325	-0.11725224	2.16E-05	0.000181441	11	0	0
ADRB3	0.4495927	0.375490854	0.074101876	0.44763841	2.18E-05	0.000182685	12	0	0
ADAM21	0.8145227	0.866529963	-0.05200724	-0.09076497	2.18E-05	0.000183087	4	0	0
SPINK8	0.6535328	0.713166544	-0.05963377	-0.13601865	2.19E-05	0.000183159	3	0	0
TFAP2A	0.2317341	0.148629001	0.083105095	0.680585948	2.19E-05	0.000183159	16	0	0
CLEC12B	0.4775517	0.557419025	-0.07986733	-0.24850644	2.20E-05	0.000183989	4	0	0
PTGDR	0.3990095	0.368063587	0.030945919	0.442525915	2.20E-05	0.000183989	10	0	0
SLITRK1	0.2452526	0.169299912	0.075952649	0.627509787	2.21E-05	0.000184732	13	0	0
SKAP1	0.3840649	0.474767857	-0.09070295	-0.37226719	2.21E-05	0.000184732	9	0	0
OR2M2	0.3698524	0.481117272	-0.11126485	-0.37220686	2.22E-05	0.000184798	2	0	0
ZNF518A	0.3842603	0.432929962	-0.04866967	-0.19443367	2.21E-05	0.000184798	14	0	0
HNRNPA1L2	0.2856918	0.318540896	-0.03284908	-0.22656799	2.21E-05	0.000184798	6	0	0
MT1E	0.3522479	0.270433362	0.081814529	1.230150541	2.21E-05	0.000184798	8	0	0
MUC22	0.6994415	0.766629531	-0.06718808	-0.13446151	2.22E-05	0.000185471	4	0	0
SCGB2B2	0.8375409	0.904397543	-0.06685668	-0.10977206	2.23E-05	0.000186101	2	0	0
ADAT3	0.1572914	0.19953528	-0.04224392	-0.24385538	2.24E-05	0.000186227	9	0	0
PDE1C	0.4405369	0.521106664	-0.08056979	-0.14561171	2.26E-05	0.000188007	10	0	0
INSL5	0.8013773	0.863574777	-0.06219752	-0.11299157	2.26E-05	0.000188075	5	0	0
RBM44	0.869737	0.859606707	0.010130307	0.01611354	2.26E-05	0.000188115	10	0	0
KIF21B	0.2003847	0.232031893	-0.03164723	-0.06997377	2.27E-05	0.000188356	16	0	0
ESM1	0.677386	0.744231824	-0.06684579	-0.14360691	2.27E-05	0.000188473	3	0	0
GZMK	0.677386	0.744231824	-0.06684579	-0.14360691	2.27E-05	0.000188473	3	0	0
GCM2	0.2625578	0.170259291	0.092298542	0.726445434	2.27E-05	0.00018874	12	0	0
HIST1H3E	0.5233962	0.422129227	0.101267021	0.345927287	2.28E-05	0.000189544	9	0	0
ZNF334	0.3846856	0.363569055	0.021116592	0.537533502	2.29E-05	0.000189847	9	0	0
S100P	0.7213217	0.800910569	-0.07958887	-0.14832436	2.30E-05	0.000190373	4	0	0

GPIHBP1	0.6296772	0.64687492	-0.01719773	-0.03423079	2.30E-05	0.000190547	6	0	0
HPSE2	0.2389703	0.169101568	0.06986878	0.572172667	2.30E-05	0.000190547	12	0	0
AOC2	0.617618	0.554407295	0.063210743	0.199924307	2.31E-05	0.000191149	7	0	0
GTPBP10	0.6498841	0.724642881	-0.07475879	-0.15483046	2.32E-05	0.000192395	1	0	0
ZNF511	0.1642278	0.199008631	-0.0347808	-0.19363537	2.33E-05	0.000192754	8	0	0
DEFB124	0.6740654	0.775683961	-0.10161854	-0.19424483	2.33E-05	0.000192754	5	0	0
FAM19A1	0.5565316	0.634655493	-0.07812393	-0.16834745	2.33E-05	0.000192918	6	0	0
RP1L1	0.7081992	0.625176445	0.083022763	0.177226649	2.35E-05	0.000193837	1	0	0
SYT6	0.1597356	0.101731226	0.05800439	0.789961954	2.35E-05	0.000193928	14	0	0
ODF4	0.6025936	0.611638417	-0.00904485	-0.08464179	2.35E-05	0.000194148	5	0	0
S100A3	0.6301976	0.613977936	0.016219654	0.052795734	2.36E-05	0.000194631	8	0	0
TMEM239	0.6795263	0.751555849	-0.0720295	-0.16736156	2.36E-05	0.000194655	4	0	0
TRAF3	0.4858408	0.497745715	-0.01190494	0.009716885	2.37E-05	0.000195303	6	0	0
MCF2L	0.3247559	0.255680091	0.069075833	0.329136144	2.37E-05	0.000195328	4	0	0
MYBPC1	0.7832012	0.887892617	-0.10469145	-0.17885612	2.39E-05	0.000196528	1	0	0
IL23R	0.6482695	0.621147117	0.027122336	0.169406588	2.39E-05	0.000196661	5	0	0
FAM83E	0.5159543	0.607633193	-0.09167885	-0.24941709	2.39E-05	0.000196791	3	0	0
ELAVL2	0.1319142	0.081863955	0.05005021	0.59657494	2.40E-05	0.000196915	6	0	0
CACNA1S	0.6678693	0.728473067	-0.06060377	-0.18275602	2.40E-05	0.000196952	6	0	0
OTX1	0.1299524	0.146476386	-0.01652398	-0.20688957	2.41E-05	0.000197688	31	0	0
GABRA5	0.3148972	0.245610851	0.069286307	0.351950024	2.41E-05	0.00019782	16	0	0
GJA4	0.3795329	0.348069439	0.03146343	0.812508074	2.42E-05	0.000198243	7	0	0
IL21	0.6739917	0.770644276	-0.09665257	-0.20907583	2.42E-05	0.000198694	9	0	0
FCRLB	0.6936873	0.796472311	-0.10278504	-0.20874296	2.42E-05	0.0001987	5	0	0
CSRNP1	0.603033	0.545486039	0.057546958	0.155087243	2.43E-05	0.000199282	4	0	0
STATH	0.68944	0.757448166	-0.06800814	-0.13653001	2.43E-05	0.000199308	4	0	0
LY75-CD302	0.1981772	0.232662899	-0.03448568	-0.25918339	2.44E-05	0.000199982	12	0	0
MYADM	0.2358768	0.209433272	0.02644353	0.796880192	2.44E-05	0.000200057	15	0	0
WNT8B	0.8037995	0.863579725	-0.05978024	-0.10295114	2.45E-05	0.000200251	4	0	0
DCUN1D1	0.8488362	0.895375389	-0.04653918	-0.07717352	2.45E-05	0.000200464	5	0	0
RTP2	0.6882285	0.721699087	-0.03347059	-0.05977907	2.46E-05	0.000200979	8	0	0
BTN1A1	0.7851423	0.77703406	0.008108223	0.102751275	2.46E-05	0.000200979	5	0	0
CCL14	0.732022	0.798117472	-0.06609545	-0.13553411	2.46E-05	0.000201034	4	0	0
ADM	0.0820515	0.107581037	-0.02552951	-0.12714249	2.47E-05	0.000201202	20	0	0
OLIG2	0.179714	0.110395253	0.069318708	0.768660935	2.47E-05	0.000201332	14	0	0
SPATA16	0.7333511	0.869559602	-0.13620847	-0.24350574	2.47E-05	0.00020172	7	0	0
CCDC116	0.654517	0.714481419	-0.05996437	-0.15027859	2.48E-05	0.000201747	10	0	0
KRTAP4-5	0.7822092	0.836217546	-0.05400838	-0.09510156	2.48E-05	0.000201777	4	0	0
WEE2	0.8966137	0.917444082	-0.0208304	-0.03277236	2.49E-05	0.000203033	1	0	0
HIST1H4K	0.4406967	0.476774281	-0.03607762	-0.34652333	2.51E-05	0.000204414	2	0	0
HEXDC	0.2594005	0.301899853	-0.0424993	-0.20494709	2.52E-05	0.000204854	7	0	0
CEACAM7	0.3613618	0.394819667	-0.03345787	-0.1358846	2.52E-05	0.000204854	7	0	0
MYBL1	0.2820835	0.35566676	-0.07358328	-0.24378683	2.52E-05	0.000205014	4	0	0
SCGB2A2	0.4655914	0.5527174	-0.08712598	-0.25211792	2.53E-05	0.00020581	5	0	0
PRR23A	0.8190666	0.774067761	0.044998803	0.088669898	2.54E-05	0.000206166	9	0	0
APOL5	0.6824684	0.785147623	-0.10267921	-0.19917274	2.55E-05	0.000206561	2	0	0
VIP	0.3642081	0.441328622	-0.07712049	-0.27212361	2.55E-05	0.000206725	6	0	0
CRY2	0.2864954	0.353642309	-0.06714695	-0.24365499	2.56E-05	0.000207637	11	0	0
CLVS2	0.3067457	0.25306371	0.053681977	0.571157713	2.56E-05	0.00020765	17	0	0
SEC16B	0.6435235	0.744636087	-0.10111262	-0.21294918	2.58E-05	0.000208783	2	0	0
SP110	0.5582672	0.648602281	-0.09033507	-0.23179596	2.58E-05	0.000209093	7	0	0
KRT24	0.6769933	0.76426061	-0.08726736	-0.18570717	2.59E-05	0.000209495	7	0	0
HLA-G	0.5794523	0.686227268	-0.10677493	-0.24494542	2.59E-05	0.000209657	2	0	0
AGMAT	0.5535966	0.585729512	-0.03213288	-0.01251557	2.60E-05	0.000209849	7	0	0
CCL15	0.6964864	0.734378603	-0.03789221	-0.06732884	2.60E-05	0.000209893	5	0	0
OR7G3	0.4724245	0.59488634	-0.12246184	-0.35454705	2.60E-05	0.000209893	2	0	0
C10orf82	0.4691011	0.434615926	0.034485176	0.186451145	2.61E-05	0.00021077	9	0	0
CNOT11	0.0876853	0.123131641	-0.03544632	-0.19827934	2.61E-05	0.000210844	6	0	0
HOXB3	0.6891107	0.638865831	0.050244863	0.09383904	2.62E-05	0.000210888	6	0	0
HOXB6	0.6891107	0.638865831	0.050244863	0.09383904	2.62E-05	0.000210888	6	0	0
PCDHGA2	0.3234775	0.253527743	0.069949714	0.505953435	2.65E-05	0.000213747	12	0	0
PATE4	0.537317	0.612854899	-0.07553788	-0.18517847	2.66E-05	0.000214348	5	0	0
FAM170A	0.7856372	0.852509691	-0.0668725	-0.12029738	2.66E-05	0.000214349	6	0	0

FGF12	0.4659528	0.600303479	-0.13435067	-0.35870828	2.67E-05	0.000215193	1	0	0
NKX6-2	0.1799358	0.09540851	0.084527321	0.945843684	2.68E-05	0.000215232	24	0	0
SH3BP4	0.1653067	0.204502806	-0.0391961	0.08045993	2.68E-05	0.000215343	9	0	0
LIPH	0.517341	0.607695757	-0.09035473	-0.26247429	2.69E-05	0.000216073	9	0	0
CBX8	0.2154133	0.142894426	0.072518892	0.694134629	2.69E-05	0.000216392	9	0	0
PARP12	0.8153882	0.872451158	-0.05706301	-0.09648959	2.71E-05	0.000217772	3	0	0
LILRA5	0.8157451	0.898119666	-0.0823746	-0.13745871	2.71E-05	0.000217772	3	0	0
EN1	0.2253666	0.188674551	0.036692094	0.387328136	2.73E-05	0.000218611	14	0	0
MAEL	0.8116877	0.897060648	-0.08537296	-0.15262261	2.73E-05	0.00021876	12	0	0
OR9K2	0.3600553	0.46576114	-0.10570585	-0.33646716	2.73E-05	0.00021876	2	0	0
SMIM22	0.6876135	0.620388668	0.067224881	0.176807754	2.74E-05	0.000219822	8	0	0
OR1J2	0.6538909	0.735327318	-0.08143644	-0.16902623	2.75E-05	0.000219914	3	0	0
SPIRE2	0.7817559	0.906073392	-0.12431751	-0.21086505	2.76E-05	0.000221146	6	0	0
HIST3H3	0.8499652	0.907931915	-0.05796672	-0.10076937	2.77E-05	0.000221361	10	0	0
FABP5	0.2359161	0.270199088	-0.03428297	-0.17997673	2.77E-05	0.000221461	12	0	0
ZNF500	0.0473215	0.06160314	-0.01428164	-0.2330044	2.77E-05	0.000221691	9	0	0
UTP14C	0.8617155	0.853849913	0.007865572	0.011716789	2.78E-05	0.000222255	5	0	0
HELZ	0.1735999	0.210303265	-0.03670335	-0.13835917	2.78E-05	0.000222339	8	0	0
ZNF415	0.3415099	0.3204298	0.021080104	0.747707948	2.79E-05	0.000222533	8	0	0
KRTAP12-4	0.581	0.657668697	-0.07666874	-0.20829301	2.80E-05	0.000223033	3	0	0
IKZF1	0.3822547	0.360355531	0.021899162	0.593266853	2.80E-05	0.000223129	7	0	0
TES	0.3556717	0.385637053	-0.02996536	-0.18645736	2.80E-05	0.000223129	9	0	0
SV2C	0.1293814	0.070569368	0.058812079	0.848023316	2.83E-05	0.000225435	8	0	0
TPH1	0.7396173	0.822613642	-0.08299637	-0.151493	2.84E-05	0.000226108	1	0	0
CTAGE1	0.7753623	0.840230297	-0.06486798	-0.11882823	2.84E-05	0.000226108	11	0	0
F11R	0.2530704	0.325915699	-0.07284534	-0.2091563	2.85E-05	0.000226508	12	0	0
KIAA1024L	0.6897238	0.760212569	-0.07048881	-0.13714213	2.85E-05	0.000226508	8	0	0
OR52E4	0.8377757	0.909589871	-0.07181415	-0.11730792	2.85E-05	0.000226737	1	0	0
MTHFD2L	0.7806386	0.85028281	-0.06964425	-0.1217927	2.86E-05	0.000227238	1	0	0
ZP1	0.7007334	0.745487747	-0.04475438	-0.09052428	2.86E-05	0.00022734	5	0	0
IFITM10	0.4252838	0.43276042	-0.00747662	0.058293998	2.90E-05	0.00023001	11	0	0
OR51S1	0.5387599	0.643514057	-0.10475419	-0.25850167	2.90E-05	0.00023001	4	0	0
CEP164	0.7500039	0.827648886	-0.07764495	-0.14171519	2.90E-05	0.00023001	2	0	0
WFDC12	0.7029237	0.785407748	-0.08248407	-0.15998956	2.91E-05	0.000230955	6	0	0
C4orf3	0.8653007	0.888578149	-0.02327744	-0.03837348	2.91E-05	0.000231004	3	0	0
RBFOX1	0.3016631	0.239365302	0.062297828	0.624802773	2.94E-05	0.000232562	13	0	0
GZMA	0.3356369	0.414769474	-0.07913254	-0.34551876	2.94E-05	0.000233127	6	0	0
CD79B	0.6295165	0.695962559	-0.06644605	-0.16759044	2.95E-05	0.00023339	11	0	0
TREH	0.5767908	0.645469649	-0.06867887	-0.15750757	2.95E-05	0.00023341	7	0	0
CAPN1	0.2380325	0.274310579	-0.03627811	-0.11802036	2.96E-05	0.000234158	5	0	0
TMEM179	0.7362756	0.83338495	-0.09710935	-0.18005043	2.96E-05	0.000234158	3	0	0
MLANA	0.8521715	0.89559975	-0.04342821	-0.0722606	2.96E-05	0.000234228	4	0	0
IFNL1	0.6478998	0.748633922	-0.10073414	-0.21579934	2.97E-05	0.000234235	3	0	0
NRG3	0.1602432	0.098702791	0.061540417	0.628376421	2.98E-05	0.000234886	6	0	0
RDH12	0.7714303	0.825311585	-0.05388133	-0.10081171	2.98E-05	0.000234886	3	0	0
OR2AK2	0.7036653	0.81049898	-0.10683372	-0.2020941	2.98E-05	0.000235216	2	0	0
GM2A	0.6324921	0.736951507	-0.10445939	-0.21733582	2.99E-05	0.000235374	1	0	0
TSPYL6	0.8512559	0.888903392	-0.03764753	-0.06176633	2.99E-05	0.000235617	5	0	0
PLA2G5	0.765236	0.808885398	-0.04364936	-0.07902593	2.99E-05	0.000235873	1	0	0
FLRT1	0.791228	0.826971227	-0.03574319	-0.06144769	3.01E-05	0.000236885	6	0	0
AQP9	0.7009301	0.775178125	-0.07424803	-0.15898712	3.01E-05	0.000236885	8	0	0
BPIFB2	0.7091534	0.781727068	-0.0725737	-0.1360927	3.01E-05	0.000236885	7	0	0
IL22	0.7549916	0.838669034	-0.08367743	-0.17391829	3.02E-05	0.000237126	7	0	0
OR6K3	0.5853886	0.707156615	-0.12176798	-0.26907813	3.03E-05	0.0002382	4	0	0
SLC17A1	0.6252366	0.727463466	-0.10222686	-0.23109573	3.03E-05	0.000238426	5	0	0
NSUN4	0.45304	0.531156772	-0.0781168	-0.13114215	3.04E-05	0.000238524	6	0	0
BPIFB1	0.7561136	0.84460147	-0.08848788	-0.15848795	3.04E-05	0.000238524	2	0	0
ZNF549	0.2091517	0.173957673	0.035194049	1.068708698	3.06E-05	0.00023973	6	0	0
HMX3	0.1502741	0.080233075	0.07004101	1.011161664	3.06E-05	0.000240024	15	0	0
OR6C75	0.7880615	0.860054524	-0.07199305	-0.1247602	3.07E-05	0.000240391	2	0	0
DAB2IP	0.6593934	0.620474422	0.038918941	0.094368905	3.07E-05	0.000240433	3	0	0
RBPJ	0.8430806	0.792963235	0.050117367	0.087348126	3.07E-05	0.000240651	1	0	0
KCNA3	0.3516042	0.255871673	0.095732497	1.28706257	3.08E-05	0.000241115	12	0	0

TARBP1	0.4238012	0.502240801	-0.07843957	-0.11791237	3.09E-05	0.000241378	3	0	0
MAL	0.281717	0.227815501	0.053901496	0.616521187	3.09E-05	0.000241378	10	0	0
KPNA7	0.7899492	0.854964046	-0.0650148	-0.11231261	3.10E-05	0.000242424	2	0	0
HSPB8	0.3152203	0.290971568	0.024248756	0.432499374	3.11E-05	0.000242916	9	0	0
SMIM3	0.678537	0.691822902	-0.01328594	0.012001075	3.11E-05	0.000242977	3	0	0
PRKD1	0.634019	0.723353368	-0.08933436	-0.18740526	3.12E-05	0.000243335	1	0	0
POLG2	0.1973113	0.230877993	-0.03356664	-0.26769418	3.12E-05	0.000243335	7	0	0
LOXHD1	0.6425348	0.720992021	-0.07845727	-0.15429635	3.12E-05	0.000243335	10	0	0
NTSR1	0.2209116	0.145676381	0.075235214	0.647028651	3.13E-05	0.000243779	5	0	0
LARP4B	0.414768	0.497223306	-0.08245536	-0.11880138	3.13E-05	0.000243835	9	0	0
ASCL3	0.7997022	0.8462697	-0.04656745	-0.0809679	3.13E-05	0.000243835	5	0	0
TMEM123	0.7607565	0.820375245	-0.05961877	-0.10748833	3.13E-05	0.000243835	1	0	0
OR6C65	0.7931314	0.873985803	-0.08085439	-0.13836542	3.13E-05	0.000243835	2	0	0
SNRNP25	0.6121788	0.654493049	-0.04231422	-0.16233305	3.14E-05	0.00024403	6	0	0
WFDC8	0.672912	0.75831773	-0.08540575	-0.1764885	3.14E-05	0.000244362	3	0	0
CCL19	0.6683552	0.725429692	-0.05707452	-0.13602082	3.15E-05	0.000244461	5	0	0
KLF5	0.7134672	0.809841432	-0.09637428	-0.17853656	3.14E-05	0.000244461	4	0	0
ZNF280B	0.500297	0.52163674	-0.02133977	0.274861901	3.17E-05	0.000246228	7	0	0
MSX1	0.3445923	0.277748787	0.066843507	0.394410119	3.18E-05	0.000246681	25	0	0
CCDC159	0.3815203	0.454020352	-0.07250005	-0.07610745	3.19E-05	0.000247318	2	0	0
ST8SIA1	0.5363601	0.643315999	-0.10695587	-0.24979445	3.19E-05	0.000247644	3	0	0
MLNR	0.3362578	0.26041532	0.075842485	0.367584256	3.22E-05	0.000249582	8	0	0
ALDH7A1	0.1827707	0.090112039	0.092658629	1.137278371	3.24E-05	0.000250776	8	0	0
GOLGA1	0.6058901	0.710474683	-0.10458456	-0.22627475	3.24E-05	0.000251023	1	0	0
TRIM43	0.7041261	0.800606042	-0.09647992	-0.18895967	3.24E-05	0.000251158	3	0	0
AHSP	0.6345527	0.711755691	-0.07720295	-0.17335137	3.25E-05	0.000251346	4	0	0
ARNTL2	0.1913121	0.25443835	-0.06312622	-0.23967627	3.26E-05	0.000251897	7	0	0
ASB14	0.8640433	0.895134725	-0.03109144	-0.05053146	3.26E-05	0.000252051	4	0	0
TBCA	0.8403536	0.898004274	-0.05765067	-0.0946362	3.27E-05	0.00025288	1	0	0
ZNF573	0.2740069	0.179216883	0.094790027	0.637794521	3.28E-05	0.000253284	4	0	0
SLC36A1	0.6203191	0.704784784	-0.08446566	-0.18471219	3.28E-05	0.000253321	3	0	0
TEPP	0.7523812	0.791304106	-0.03892295	-0.06268449	3.29E-05	0.000253899	6	0	0
OR10P1	0.6167374	0.699571073	-0.08283372	-0.18167812	3.29E-05	0.000254014	4	0	0
MYH13	0.5888597	0.689271727	-0.10041202	-0.22543348	3.30E-05	0.000254014	4	0	0
TMEM71	0.5166831	0.576876277	-0.06019316	-0.26921767	3.31E-05	0.000255391	6	0	0
TLDC2	0.613224	0.698408936	-0.08518489	-0.18454408	3.32E-05	0.000255989	8	0	0
ZNF782	0.1098196	0.140430953	-0.03061138	-0.25253211	3.33E-05	0.000256006	5	0	0
ADAMTS8	0.1835976	0.147636287	0.035961282	0.359405971	3.33E-05	0.000256006	11	0	0
CLEC9A	0.8062343	0.879512466	-0.07327817	-0.12595502	3.33E-05	0.000256006	4	0	0
PLEKHG4B	0.7744679	0.838561006	-0.06409307	-0.1261709	3.35E-05	0.000257462	9	0	0
GSG1	0.7580972	0.822149088	-0.06405187	-0.12136672	3.35E-05	0.000257781	8	0	0
OR2J2	0.5632647	0.685723296	-0.12245862	-0.27931224	3.38E-05	0.000259488	1	0	0
PRR20A	0.8308449	0.884834064	-0.0539892	-0.08978053	3.39E-05	0.000259992	1	0	0
PTDSS2	0.5922563	0.633016383	-0.04076004	-0.02574174	3.39E-05	0.00026022	3	0	0
TAS2R41	0.7533024	0.831271939	-0.07796952	-0.14277304	3.41E-05	0.000261456	2	0	0
IFNW1	0.8471952	0.907721137	-0.06052598	-0.10458885	3.44E-05	0.000263683	3	0	0
STYK1	0.1417726	0.192118258	-0.05034562	-0.26990158	3.44E-05	0.000263765	14	0	0
LCE3D	0.5533357	0.707387527	-0.1540518	-0.34875769	3.45E-05	0.000264544	1	0	0
LIX1	0.5753096	0.604656949	-0.02934731	-0.17373474	3.46E-05	0.000264544	5	0	0
UBE2U	0.8066979	0.873331279	-0.06663338	-0.12238632	3.46E-05	0.000264703	12	0	0
ABHD16B	0.576448	0.641323666	-0.06487571	-0.16741043	3.47E-05	0.000265509	7	0	0
TBATA	0.6783794	0.736998017	-0.0586186	-0.12058187	3.47E-05	0.000265624	6	0	0
NPY5R	0.2604224	0.182949578	0.07747278	0.836915638	3.50E-05	0.000267454	6	0	0
MUC12	0.1111987	0.145055319	-0.03385666	-0.33899106	3.50E-05	0.000267454	2	0	0
LRRC10	0.7730973	0.829864245	-0.05676692	-0.10304483	3.53E-05	0.000269351	5	0	0
DEFB123	0.6560141	0.748454735	-0.09244066	-0.18570856	3.53E-05	0.000269549	10	0	0
C7	0.6546642	0.570707225	0.083956992	0.382373857	3.53E-05	0.00026955	6	0	0
NR2E3	0.6410975	0.728018858	-0.08692132	-0.17703722	3.53E-05	0.00026955	2	0	0
ZNF470	0.2460585	0.195663602	0.050394861	1.113479141	3.54E-05	0.000270103	10	0	0
OCIAD2	0.2521574	0.293089092	-0.04093171	-0.19968746	3.57E-05	0.000271768	13	0	0
PRDM9	0.4928161	0.584792176	-0.09197611	-0.24452583	3.57E-05	0.000272165	6	0	0
PTPRC	0.5269828	0.629188202	-0.10220536	-0.29484798	3.58E-05	0.00027252	4	0	0
UBA7	0.3147096	0.231440934	0.083268699	0.649044792	3.58E-05	0.00027252	5	0	0

CALML6	0.5792607	0.626652174	-0.04739145	-0.11245821	3.58E-05	0.000272669	9	0	0
CLPS	0.7757251	0.75897752	0.016747533	0.032975446	3.60E-05	0.000273541	6	0	0
FFAR1	0.7092909	0.799578413	-0.09028748	-0.17232513	3.60E-05	0.000273751	7	0	0
ABCC2	0.798235	0.835518963	-0.03728393	-0.06272497	3.61E-05	0.000274014	7	0	0
CEP85	0.0936775	0.104848563	-0.01117105	-0.12529848	3.61E-05	0.000274319	12	0	0
ASCC1	0.4687909	0.569728434	-0.10093756	-0.21505702	3.62E-05	0.00027485	8	0	0
ROBO2	0.3424923	0.353603655	-0.01111136	0.195657633	3.63E-05	0.000275746	3	0	0
OR1N2	0.4894434	0.583437239	-0.09399383	-0.20087444	3.65E-05	0.000276591	2	0	0
GK2	0.8384758	0.886910539	-0.04843476	-0.08107853	3.66E-05	0.000277699	10	0	0
LRRC73	0.4169236	0.472606779	-0.05568315	-0.11265487	3.67E-05	0.000277699	11	0	0
REM1	0.2538803	0.234917774	0.018962508	0.526068871	3.67E-05	0.000277699	13	0	0
SIGLEC9	0.4949462	0.559965352	-0.06501911	-0.17442067	3.67E-05	0.000277947	4	0	0
TIFA	0.1481881	0.175989186	-0.02780104	-0.24544098	3.70E-05	0.000279799	8	0	0
CSNK2A3	0.7889064	0.853779459	-0.0648731	-0.11694385	3.70E-05	0.000280105	8	0	0
SLC13A4	0.6631846	0.735845454	-0.07266087	-0.16033982	3.71E-05	0.000280602	4	0	0
SERTAD4	0.2673908	0.2906178	-0.02322696	0.027705597	3.72E-05	0.000280786	5	0	0
PDX1	0.2370934	0.165592456	0.071500917	0.45580736	3.72E-05	0.000280786	9	0	0
ABCA10	0.5637095	0.663138969	-0.09942945	-0.29756994	3.72E-05	0.000280786	2	0	0
AP1G2	0.1859076	0.209666777	-0.02375916	-0.15347954	3.76E-05	0.000284046	14	0	0
CFHR3	0.4735961	0.57489009	-0.101294	-0.24512005	3.77E-05	0.000284594	2	0	0
DOC2B	0.3007918	0.296203532	0.004588229	0.390324834	3.78E-05	0.0002848	6	0	0
ANKRD66	0.6000542	0.710820048	-0.11076584	-0.23935298	3.78E-05	0.000285308	4	0	0
OR2L2	0.5532057	0.664420247	-0.11121457	-0.25505967	3.79E-05	0.000285541	2	0	0
RESP18	0.6001684	0.65057131	-0.05040287	0.042156243	3.79E-05	0.000285593	7	0	0
CHRM4	0.576363	0.61137593	-0.03501294	-0.07264574	3.83E-05	0.000288052	7	0	0
FAM198B	0.4993867	0.468407677	0.030978979	0.159078601	3.83E-05	0.000288551	7	0	0
NAA15	0.0485662	0.075089223	-0.02652298	-0.23758252	3.86E-05	0.000290168	8	0	0
FCN2	0.5566198	0.648074314	-0.09145456	-0.19735117	3.86E-05	0.000290168	5	0	0
OR6T1	0.7016827	0.786681397	-0.08499873	-0.15769153	3.87E-05	0.000291126	3	0	0
RALB	0.5317569	0.486696221	0.045060629	0.19487776	3.88E-05	0.000291608	5	0	0
ANGPTL6	0.6600382	0.698817408	-0.03877922	-0.07810311	3.88E-05	0.000291608	8	0	0
DACT2	0.3132416	0.278341541	0.034900065	0.632942547	3.90E-05	0.000292432	7	0	0
SLC29A3	0.454147	0.49514912	-0.04100214	-0.08776022	3.90E-05	0.000292662	8	0	0
STAP1	0.6395252	0.712972853	-0.07344761	-0.15337478	3.92E-05	0.000293728	4	0	0
TINAG	0.5668106	0.675908571	-0.10909792	-0.28171409	3.93E-05	0.000294652	3	0	0
AQP2	0.7052584	0.758003675	-0.05274532	-0.10915936	3.94E-05	0.000295039	12	0	0
SPATA31D1	0.7636439	0.831520879	-0.06787702	-0.12098904	3.94E-05	0.00029522	2	0	0
HIPK4	0.7283486	0.759560135	-0.03121153	-0.067942	3.94E-05	0.00029522	10	0	0
ZNF883	0.306382	0.226784836	0.079597131	0.81933666	3.97E-05	0.000297125	8	0	0
MYH15	0.6900104	0.792530124	-0.10251973	-0.21379028	3.97E-05	0.000297419	4	0	0
PCDHGA4	0.4779937	0.411137726	0.066855963	0.354868453	3.98E-05	0.0002978	10	0	0
KRTAP19-3	0.7171048	0.777406459	-0.06030168	-0.1120322	3.99E-05	0.000298248	4	0	0
SLC18A2	0.3411812	0.269539181	0.071642005	0.653147854	4.00E-05	0.000298875	8	0	0
ZNF85	0.3780331	0.368957312	0.009075832	0.462945228	4.02E-05	0.000300652	5	0	0
NRAP	0.7988708	0.838989369	-0.04011855	-0.07798787	4.03E-05	0.000301044	6	0	0
ANO9	0.327469	0.328040629	-0.00057159	-0.10701593	4.04E-05	0.000301167	7	0	0
FLI1	0.3086844	0.380154076	-0.07146964	-0.27129722	4.04E-05	0.000301167	10	0	0
SLC6A2	0.3629226	0.298437472	0.064485125	0.432624148	4.05E-05	0.00030199	10	0	0
NMBR	0.2931671	0.241888703	0.051278383	0.485724345	4.05E-05	0.000302074	8	0	0
JOSD1	0.1153861	0.162452593	-0.04706648	-0.2939449	4.05E-05	0.000302124	5	0	0
NPAS4	0.2046947	0.140553002	0.064141726	0.632717024	4.06E-05	0.000302295	8	0	0
ICOSLG	0.0436487	0.051304385	-0.00765565	-0.11866284	4.07E-05	0.000302925	5	0	0
KCTD18	0.8220434	0.880323094	-0.05827967	-0.09767016	4.09E-05	0.000304023	1	0	0
OR5B3	0.7256992	0.81241393	-0.08671474	-0.16138188	4.09E-05	0.000304259	2	0	0
ESCO1	0.2992534	0.312490263	-0.01323689	-0.15949418	4.13E-05	0.000306894	7	0	0
NFAM1	0.5244665	0.516417563	0.008048967	0.141239395	4.13E-05	0.000307221	12	0	0
TRPC6	0.7543315	0.842185214	-0.08785376	-0.15901519	4.14E-05	0.000307326	4	0	0
CCDC27	0.7749605	0.853374005	-0.0784135	-0.14086401	4.14E-05	0.000307511	6	0	0
OSM	0.4447371	0.523340359	-0.07860326	-0.23752634	4.17E-05	0.000309775	10	0	0
ANKS4B	0.7948792	0.8744221	-0.07954291	-0.13731642	4.18E-05	0.000309945	7	0	0
OR7G1	0.8060639	0.881594989	-0.07553109	-0.12770639	4.19E-05	0.000310491	1	0	0
KCNC2	0.3218733	0.320129655	0.001743662	0.187710109	4.19E-05	0.000310666	9	0	0
FBXW10	0.7545774	0.68936601	0.065211407	0.127620582	4.19E-05	0.000310868	2	0	0

IL17C	0.3568209	0.416283949	-0.05946306	-0.24886475	4.20E-05	0.000310921	6	0	0
RWDD3	0.2876056	0.22717886	0.06042674	0.246730125	4.20E-05	0.000311214	8	0	0
GABRB3	0.5931006	0.685319286	-0.09221865	-0.20554401	4.21E-05	0.000311733	5	0	0
LHCGR	0.4085481	0.361780746	0.046767341	0.525801302	4.22E-05	0.000312035	6	0	0
CCDC8	0.379721	0.284881027	0.094839975	0.840724655	4.22E-05	0.000312097	10	0	0
CD38	0.4862497	0.506670965	-0.02042123	0.241455269	4.23E-05	0.000312453	10	0	0
ZNF648	0.5135848	0.594833139	-0.08124838	-0.18297707	4.23E-05	0.000312577	5	0	0
BLNK	0.4432374	0.522929001	-0.07969159	-0.20622382	4.23E-05	0.000312577	7	0	0
C16orf89	0.5386173	0.554190192	-0.01557293	-0.02380878	4.23E-05	0.000312577	9	0	0
RAB3IP	0.4341524	0.439894889	-0.00574248	-0.14083558	4.24E-05	0.000312734	9	0	0
CHAT	0.2314044	0.182585495	0.048818897	0.463950009	4.24E-05	0.000312978	6	0	0
DCDC2B	0.7119318	0.776313995	-0.0643822	-0.13665683	4.24E-05	0.000313026	6	0	0
FAM131C	0.2358551	0.296498177	-0.06064307	-0.14535704	4.25E-05	0.000313134	6	0	0
CAV3	0.7177493	0.709327994	0.008421262	0.03168321	4.25E-05	0.000313134	6	0	0
MFAP3L	0.7908681	0.842636899	-0.05176879	-0.09348612	4.25E-05	0.00031329	5	0	0
KRTAP23-1	0.6673108	0.750976534	-0.0836657	-0.16770177	4.25E-05	0.00031329	5	0	0
ENAM	0.72596	0.801239985	-0.07527998	-0.14076173	4.26E-05	0.0003138	4	0	0
WWTR1	0.6110178	0.711164539	-0.10014672	-0.31544843	4.27E-05	0.000313925	2	0	0
TIPARP	0.7636431	0.801760775	-0.03811771	-0.07822776	4.28E-05	0.000314771	5	0	0
HTRA1	0.1658456	0.1466805	0.019165121	0.535988629	4.29E-05	0.000315132	13	0	0
C1D	0.6662948	0.766565114	-0.10027031	-0.20681211	4.30E-05	0.000315548	2	0	0
TBX18	0.2066383	0.121119939	0.085518368	0.833229387	4.30E-05	0.000315548	13	0	0
SLC28A2	0.7772843	0.840058793	-0.06277445	-0.11489266	4.30E-05	0.00031557	6	0	0
SFRP4	0.6737144	0.781229944	-0.10751558	-0.21070329	4.30E-05	0.000315901	1	0	0
MYOD1	0.1853916	0.126222551	0.059169058	0.654114418	4.32E-05	0.000316532	6	0	0
MGRN1	0.2804719	0.332767403	-0.05229547	-0.09532007	4.32E-05	0.000316532	10	0	0
SLC30A10	0.8182714	0.896297586	-0.0780262	-0.13193507	4.32E-05	0.000316721	5	0	0
SLC15A3	0.2414825	0.153100805	0.088381681	0.638869031	4.34E-05	0.000317971	5	0	0
TMIE	0.3968763	0.393494396	0.003381856	-0.08501172	4.35E-05	0.000318459	10	0	0
NOS2	0.6325043	0.720569573	-0.08806528	-0.17940852	4.35E-05	0.000318645	4	0	0
NOL9	0.4057403	0.394028849	0.01171142	0.068997946	4.37E-05	0.000319525	13	0	0
TAS1R1	0.4057403	0.394028849	0.01171142	0.068997946	4.37E-05	0.000319525	13	0	0
CNGA3	0.3927974	0.451741497	-0.05894408	0.043753758	4.37E-05	0.000319753	7	0	0
RTP1	0.6746936	0.688931505	-0.0142379	0.009827537	4.38E-05	0.000320145	9	0	0
LRRC3	0.4027939	0.401021458	0.001772393	0.114675297	4.39E-05	0.000320556	6	0	0
HLA-DQB2	0.6361248	0.746748037	-0.11062328	-0.22687879	4.40E-05	0.000321179	4	0	0
TIE1	0.6788155	0.631692682	0.047122807	0.104029425	4.40E-05	0.000321209	7	0	0
SLC45A3	0.0951013	0.103567356	-0.00846608	-0.20353117	4.41E-05	0.000321999	9	0	0
TMEM249	0.5939167	0.665997413	-0.07208067	-0.17023556	4.43E-05	0.000322975	11	0	0
AP1B1	0.8930069	0.925010251	-0.03200333	-0.05042388	4.44E-05	0.000323761	2	0	0
KRT6C	0.5950187	0.655735485	-0.06071674	-0.18023014	4.45E-05	0.000324188	3	0	0
IQUB	0.5187222	0.578987817	-0.06026565	-0.19380751	4.46E-05	0.000324898	2	0	0
CBY3	0.8432099	0.882464139	-0.03925421	-0.06691649	4.47E-05	0.000325572	7	0	0
MYCBP	0.7156687	0.784512376	-0.0688437	-0.13075869	4.49E-05	0.000325656	1	0	0
GJA9	0.7156687	0.784512376	-0.0688437	-0.13075869	4.49E-05	0.000325656	1	0	0
ROBO1	0.2168372	0.15326169	0.063575512	0.591150577	4.48E-05	0.000325656	12	0	0
DEFA5	0.6165242	0.693847608	-0.07732336	-0.18324907	4.48E-05	0.000325656	4	0	0
LRIT1	0.5658283	0.651240317	-0.08541205	-0.20030103	4.48E-05	0.000325656	6	0	0
OR52I1	0.6590977	0.725874357	-0.06677662	-0.13720646	4.49E-05	0.000325656	3	0	0
GJD3	0.5666953	0.506658143	0.060037162	0.243660195	4.49E-05	0.000325656	14	0	0
OR10H5	0.6275051	0.741113897	-0.11360883	-0.24217399	4.49E-05	0.000325656	5	0	0
MROH9	0.6798397	0.774475129	-0.09463543	-0.18967277	4.50E-05	0.000326069	3	0	0
CDH20	0.2905591	0.216439522	0.074119578	0.50809725	4.51E-05	0.000326846	5	0	0
CCNT2	0.2322304	0.297797949	-0.06556759	-0.16355758	4.51E-05	0.000326871	5	0	0
PRR9	0.5822258	0.66054372	-0.07831791	-0.21160005	4.52E-05	0.000327706	5	0	0
PDZRN3	0.2343592	0.158763354	0.075595843	0.548601262	4.55E-05	0.000329163	10	0	0
AQP12B	0.6356929	0.716644179	-0.08095129	-0.17858326	4.55E-05	0.000329672	2	0	0
TMIGD1	0.7854318	0.849754017	-0.06432218	-0.11996143	4.57E-05	0.000330889	7	0	0
CTNND2	0.2063317	0.132861039	0.073470674	0.876837519	4.58E-05	0.000330912	12	0	0
CIB3	0.5883001	0.616046183	-0.02774612	-0.07250305	4.58E-05	0.000330912	3	0	0
OR1Q1	0.6736819	0.74947727	-0.07579533	-0.15189578	4.58E-05	0.000331201	2	0	0
LYG2	0.8270757	0.874707816	-0.04763209	-0.08295008	4.59E-05	0.000331581	11	0	0
RETN	0.6022387	0.674310112	-0.07207146	-0.16602763	4.59E-05	0.00033171	7	0	0

CUL1	0.3857162	0.318328764	0.067387401	0.456557967	4.60E-05	0.000332325	6	0	0
AKNA	0.6723825	0.636430539	0.035951939	0.123576487	4.62E-05	0.000333038	6	0	0
STOX2	0.5543181	0.642988666	-0.08867061	-0.23755484	4.65E-05	0.000335294	4	0	0
NXPH2	0.342154	0.232063447	0.110090512	0.734011463	4.65E-05	0.000335557	10	0	0
FABP12	0.6139176	0.69098543	-0.07706785	-0.21828525	4.66E-05	0.000335766	3	0	0
TAL2	0.7427075	0.811504966	-0.06879748	-0.14823014	4.67E-05	0.000336259	4	0	0
TUBB1	0.8006181	0.839052881	-0.0384348	-0.0748892	4.67E-05	0.000336259	9	0	0
LEFTY1	0.5269782	0.446798049	0.080180109	0.438358409	4.68E-05	0.000336585	3	0	0
LDLRAD3	0.5116034	0.522902713	-0.0112993	0.062463366	4.68E-05	0.000336901	4	0	0
SLC25A26	0.7165716	0.777009583	-0.06043796	-0.11433248	4.69E-05	0.000336969	2	0	0
STAT1	0.4708503	0.51871934	-0.04786905	-0.1917681	4.71E-05	0.000338631	5	0	0
OR5D16	0.3972147	0.498508355	-0.10129366	-0.29618785	4.72E-05	0.000338788	4	0	0
PABPC3	0.4738105	0.369787547	0.10402293	0.351220204	4.74E-05	0.000340082	4	0	0
ADAMTS18	0.2823124	0.23949565	0.042816766	0.512257314	4.74E-05	0.000340082	11	0	0
DIRC1	0.7974992	0.860448373	-0.06294915	-0.10825952	4.76E-05	0.000341191	2	0	0
MYH1	0.6965041	0.784630941	-0.08812681	-0.17578791	4.77E-05	0.00034185	8	0	0
ZBED2	0.5955937	0.68636572	-0.09077198	-0.23922877	4.77E-05	0.000342049	6	0	0
RRM2	0.1504721	0.194333937	-0.04386184	-0.07234111	4.78E-05	0.000342702	12	0	0
P2RX3	0.6853393	0.648545669	0.036793672	0.084804669	4.82E-05	0.000345089	6	0	0
TOR4A	0.4063902	0.300653649	0.105736591	0.647240543	4.82E-05	0.000345426	10	0	0
SLC6A1	0.1650197	0.108874907	0.056144798	0.61136681	4.83E-05	0.000345559	7	0	0
TYR	0.7312444	0.811202908	-0.07995851	-0.15249264	4.83E-05	0.00034571	4	0	0
KMT2C	0.0902697	0.035674194	0.054595546	0.732116995	4.85E-05	0.000346935	3	0	0
MFSD11	0.0786326	0.099584949	-0.02095232	-0.13120745	4.85E-05	0.000346935	5	0	0
LMO2	0.5614121	0.635884825	-0.07447276	-0.17502732	4.87E-05	0.00034809	4	0	0
CD209	0.4032851	0.489076924	-0.08579181	-0.26006198	4.88E-05	0.000348593	4	0	0
PDYN	0.3704837	0.437232912	-0.06674919	-0.07798851	4.88E-05	0.000348704	9	0	0
KCNN4	0.4190578	0.48838704	-0.06932919	-0.14888824	4.91E-05	0.000350769	9	0	0
HTN3	0.8138002	0.853201057	-0.03940089	-0.06710507	4.93E-05	0.000351429	3	0	0
DKK3	0.344623	0.368442062	-0.02381909	0.096775584	4.93E-05	0.00035155	10	0	0
PC	0.1610829	0.181266324	-0.02018341	0.170886798	4.93E-05	0.00035155	9	0	0
RHBDF1	0.3025032	0.249602188	0.052901051	0.288791382	4.93E-05	0.000351598	10	0	0
PROM2	0.6213057	0.695034864	-0.07372913	-0.18047975	4.94E-05	0.000352195	9	0	0
BMP10	0.7875078	0.866245668	-0.07873783	-0.13977642	4.95E-05	0.000352297	4	0	0
CCNY	0.7716982	0.822031282	-0.05033308	-0.093365	4.96E-05	0.000353048	6	0	0
C2orf88	0.8455278	0.810696371	0.034831398	0.075342319	4.96E-05	0.000353224	6	0	0
GIMD1	0.818525	0.867822563	-0.04929758	-0.08492068	4.99E-05	0.000354653	3	0	0
EOMES	0.1463332	0.080935701	0.065397461	0.714489905	5.00E-05	0.000355173	13	0	0
NOSTRIN	0.7003727	0.760178489	-0.05980583	-0.13095411	5.00E-05	0.000355186	4	0	0
WIF1	0.3499437	0.330766984	0.019176669	0.514281436	5.02E-05	0.000356324	13	0	0
ATMIN	0.3771338	0.422739446	-0.04560562	-0.15721967	5.02E-05	0.000356324	6	0	0
TSPAN33	0.0467802	0.05745256	-0.01067236	-0.19971505	5.04E-05	0.000357748	10	0	0
HLA-DOA	0.530949	0.588857877	-0.05790887	0.001380601	5.08E-05	0.000360073	15	0	0
STPG1	0.3827557	0.436136191	-0.0533805	-0.27169388	5.08E-05	0.0003603	2	0	0
AK4	0.2520844	0.272460035	-0.02037562	-0.16576486	5.09E-05	0.000360656	13	0	0
OR6C4	0.6021644	0.67524997	-0.07308554	-0.16992205	5.09E-05	0.000360656	3	0	0
CCL1	0.4913535	0.570480728	-0.07912719	-0.20318302	5.13E-05	0.000363491	7	0	0
NGEF	0.6755799	0.752217142	-0.0766372	-0.1653706	5.14E-05	0.000364273	10	0	0
CHST4	0.6443961	0.739503421	-0.09510734	-0.2004431	5.16E-05	0.000364974	6	0	0
S100Z	0.4072418	0.476822212	-0.0695804	-0.26459671	5.16E-05	0.000365162	6	0	0
CYP4B1	0.3061619	0.38495394	-0.07879203	-0.30718226	5.19E-05	0.000367303	2	0	0
OR6C2	0.7043735	0.798833944	-0.09446047	-0.18316788	5.21E-05	0.000368238	6	0	0
SLC13A2	0.7528627	0.745402625	0.007460083	0.031749906	5.22E-05	0.000369194	6	0	0
FABP4	0.7610908	0.781089009	-0.0199982	-0.02382539	5.24E-05	0.000370192	5	0	0
FBP1	0.4877476	0.448085059	0.03966256	0.69967248	5.24E-05	0.000370192	7	0	0
KDM4E	0.711448	0.77091733	-0.05946937	-0.12277124	5.27E-05	0.000371869	3	0	0
NR5A1	0.7327925	0.777915925	-0.04512347	-0.09422903	5.28E-05	0.000372545	5	0	0
ICAM4	0.3537593	0.292454586	0.061304753	0.666451937	5.29E-05	0.000372884	9	0	0
HLA-DQA2	0.7247348	0.824655896	-0.09992109	-0.1959736	5.29E-05	0.000373168	2	0	0
ESR1	0.8066289	0.865956528	-0.05932758	-0.10117856	5.30E-05	0.000373317	1	0	0
OTOP3	0.3412553	0.273444649	0.0678107	0.451023762	5.30E-05	0.000373358	10	0	0
ZNF43	0.4360593	0.448416255	-0.01235695	0.42203587	5.32E-05	0.000374149	7	0	0
PHGR1	0.6587555	0.718243781	-0.05948827	-0.14332838	5.32E-05	0.000374469	6	0	0

PTPN5	0.463468	0.417632916	0.04583511	0.284921994	5.36E-05	0.000376707	7	0	0
FAM209B	0.7621345	0.831242799	-0.06910832	-0.12555292	5.37E-05	0.000377227	6	0	0
OR2D3	0.8063181	0.86484617	-0.05852809	-0.09989795	5.40E-05	0.000379539	1	0	0
ACR	0.6429701	0.728632421	-0.0856623	-0.18064922	5.40E-05	0.000379539	8	0	0
FAM174B	0.0891595	0.127215362	-0.03805589	-0.37054438	5.41E-05	0.000380109	4	0	0
GGH	0.0732148	0.115473659	-0.04225886	-0.16002623	5.42E-05	0.000380374	7	0	0
SOX10	0.8285932	0.854698363	-0.02610514	-0.04592864	5.47E-05	0.000383519	3	0	0
GSTM3	0.7333024	0.779630311	-0.04632791	-0.08681896	5.48E-05	0.000384622	5	0	0
TASP1	0.0851708	0.126962503	-0.04179173	-0.26535122	5.49E-05	0.000385057	9	0	0
METTL7A	0.7019433	0.687037197	0.014906114	0.052800506	5.50E-05	0.000385379	3	0	0
PTPRM	0.1821719	0.133918416	0.048253529	0.456531455	5.51E-05	0.000385745	5	0	0
GALNTL6	0.5174376	0.581546447	-0.06410885	-0.05510008	5.53E-05	0.000386766	6	0	0
PITRM1	0.3701234	0.450299251	-0.08017582	-0.17925849	5.52E-05	0.000386766	6	0	0
B3GAT1	0.1341401	0.068335433	0.065804658	0.853127644	5.53E-05	0.000386766	7	0	0
AKR1C3	0.6722829	0.77675091	-0.10446802	-0.20770519	5.53E-05	0.000386849	2	0	0
RAB19	0.2582747	0.310925709	-0.05265098	-0.24730254	5.56E-05	0.000388574	7	0	0
PRSS1	0.4821416	0.560750249	-0.07860864	-0.21516814	5.56E-05	0.000388574	2	0	0
GABRG2	0.2739655	0.225055512	0.048909969	0.481300995	5.57E-05	0.000388842	15	0	0
AXDND1	0.3666002	0.398484598	-0.03188439	-0.23380054	5.59E-05	0.000390253	3	0	0
PCDHGA7	0.4185156	0.317633372	0.100882201	0.573388667	5.59E-05	0.000390276	9	0	0
STMN1	0.3253091	0.374691156	-0.04938209	-0.25174023	5.60E-05	0.000390803	16	0	0
S100A2	0.4682689	0.539818044	-0.07154919	-0.16901635	5.60E-05	0.000390803	5	0	0
FAM71A	0.7872853	0.819059218	-0.03177395	-0.05443675	5.61E-05	0.000390902	6	0	0
FBXO6	0.4000976	0.432513166	-0.03241557	-0.17885316	5.62E-05	0.000391422	7	0	0
MRGPRD	0.7124965	0.787966188	-0.07546965	-0.15117714	5.62E-05	0.000391422	8	0	0
DUXA	0.6752322	0.775487801	-0.10025563	-0.19824788	5.63E-05	0.000392422	7	0	0
GNA14	0.2726442	0.193490796	0.079153396	0.550583794	5.65E-05	0.000393621	5	0	0
HOXC10	0.1474209	0.103742648	0.043678224	0.432775802	5.67E-05	0.00039464	15	0	0
RNF145	0.2233408	0.287332937	-0.06399218	-0.20575568	5.67E-05	0.000394708	12	0	0
SEMA3C	0.6525167	0.754474508	-0.10195784	-0.21452352	5.69E-05	0.000395709	6	0	0
PPP2CB	0.7423501	0.805628254	-0.06327811	-0.1362075	5.73E-05	0.000398175	4	0	0
HTR1B	0.196818	0.128932514	0.067885533	0.646016547	5.77E-05	0.000400643	9	0	0
HOXC11	0.3817499	0.279459523	0.102290344	0.511881426	5.77E-05	0.000400996	12	0	0
OR8G1	0.5576593	0.669261162	-0.11160185	-0.26046216	5.79E-05	0.000402266	3	0	0
CCT8L2	0.6231913	0.718812254	-0.09562099	-0.19306045	5.80E-05	0.000402266	2	0	0
TRAT1	0.5339995	0.607744244	-0.07374469	-0.24182473	5.81E-05	0.000402697	6	0	0
NCR3LG1	0.3086127	0.334432607	-0.0258199	-0.13995661	5.81E-05	0.000402697	9	0	0
DGUOK	0.2708098	0.352500493	-0.08169068	-0.14742987	5.83E-05	0.000404172	13	0	0
ANXA10	0.6685154	0.75729495	-0.0887796	-0.1773998	5.85E-05	0.000405164	1	0	0
PAM	0.607792	0.65666308	-0.04887111	-0.13402155	5.85E-05	0.000405308	4	0	0
RXRG	0.3165812	0.283354669	0.033226501	0.494756262	5.85E-05	0.000405315	14	0	0
MRPS36	0.1494635	0.181177894	-0.03171442	0.007496621	5.89E-05	0.000407829	7	0	0
KIAA1755	0.3496162	0.313628999	0.035987242	0.462821525	5.90E-05	0.000408346	8	0	0
AADACL3	0.7238733	0.799857204	-0.07598393	-0.14257138	5.91E-05	0.000409077	3	0	0
HRAS	0.2271355	0.253697307	-0.02656177	-0.11861525	5.92E-05	0.000409623	6	0	0
HEATR4	0.7747308	0.823475183	-0.04874443	-0.09187277	5.93E-05	0.000409916	3	0	0
MUC20	0.6196063	0.744165874	-0.12455961	-0.2673959	5.94E-05	0.000410207	4	0	0
TSPAN16	0.8143939	0.86140872	-0.04701481	-0.0829528	5.94E-05	0.000410207	5	0	0
MACROD1	0.1142615	0.132782785	-0.01852124	-0.16082077	5.95E-05	0.000410772	11	0	0
CHRM2	0.226827	0.164529958	0.062297011	0.682183227	5.95E-05	0.000410948	13	0	0
WFDC1	0.3132905	0.307839747	0.005450779	0.359333581	5.96E-05	0.0004111	10	0	0
KCNK17	0.4315902	0.481472757	-0.04988254	-0.05960415	6.00E-05	0.000413911	7	0	0
NPW	0.0546389	0.06742821	-0.01278934	-0.16594792	6.01E-05	0.000414108	11	0	0
GGT5	0.5185009	0.483780587	0.034720326	0.100809571	6.03E-05	0.000415695	2	0	0
MT1M	0.375474	0.30665468	0.068819316	0.747820844	6.04E-05	0.000415889	6	0	0
CCL26	0.7092759	0.675964048	0.03331186	0.085486055	6.05E-05	0.000416341	6	0	0
CYSLTR2	0.8235719	0.867414928	-0.04384303	-0.07422208	6.06E-05	0.000416798	4	0	0
TAF4B	0.3036617	0.38290844	-0.07924678	-0.20598366	6.07E-05	0.000417318	7	0	0
RBP2	0.7508539	0.809713673	-0.05885977	-0.10316833	6.07E-05	0.0004175	2	0	0
ST6GALNAC	0.5958471	0.661276173	-0.06542908	-0.17266765	6.07E-05	0.0004175	2	0	0
ENO1	0.1526182	0.205693141	-0.05307494	-0.20456558	6.10E-05	0.000419528	8	0	0
CACNA1B	0.5457405	0.578954487	-0.03321401	-0.03554367	6.12E-05	0.000420216	5	0	0
MC3R	0.2942474	0.345263247	-0.05101584	-0.2264476	6.14E-05	0.000421748	7	0	0

CXCL16	0.3856082	0.418522007	-0.03291376	-0.11475807	6.15E-05	0.000422327	12	0	0
CHIT1	0.2775578	0.325035822	-0.04747801	-0.13532121	6.16E-05	0.000422896	6	0	0
SPATS2	0.1636628	0.228535718	-0.06487289	-0.12079792	6.19E-05	0.000424104	7	0	0
ZNF532	0.7223703	0.787642671	-0.06527239	-0.13413498	6.19E-05	0.000424104	12	0	0
CLRN3	0.7818755	0.837722577	-0.05584705	-0.09904349	6.19E-05	0.000424168	3	0	0
NR0B2	0.6657125	0.612153896	0.0535586	0.108992123	6.21E-05	0.00042525	8	0	0
SAFB2	0.1953136	0.241039956	-0.04572639	-0.26503564	6.23E-05	0.000426882	3	0	0
UNCX	0.1333173	0.06788798	0.065429329	1.005518307	6.24E-05	0.000426997	8	0	0
ASPSCR1	0.0330618	0.04001355	-0.00695175	-0.21831027	6.26E-05	0.000428282	3	0	0
MOB1A	0.1613965	0.215358834	-0.05396231	-0.13119363	6.27E-05	0.000428671	6	0	0
PAXBP1	0.162143	0.217497381	-0.05535438	-0.26170581	6.27E-05	0.000428671	5	0	0
TAL1	0.2985617	0.231672305	0.06688939	0.80089701	6.30E-05	0.000430514	22	0	0
KCNIP1	0.4234105	0.473026144	-0.04961564	-0.06141444	6.30E-05	0.000430514	7	0	0
ZNF696	0.7212351	0.82651304	-0.10527799	-0.20559952	6.31E-05	0.000430514	5	0	0
PKMYT1	0.1799035	0.14959079	0.030312748	0.057718611	6.31E-05	0.000430514	8	0	0
NRXN2	0.2517871	0.290426086	-0.03863901	-0.10162832	6.32E-05	0.000431019	11	0	0
GUCY2C	0.6704087	0.730967783	-0.06055912	-0.12804187	6.32E-05	0.000431019	2	0	0
CD207	0.5967955	0.692612394	-0.0958169	-0.19963558	6.32E-05	0.000431139	3	0	0
NPY	0.2219374	0.136562366	0.085375014	1.285538361	6.33E-05	0.00043126	12	0	0
UBE3D	0.1483102	0.182226894	-0.03391665	-0.25530209	6.33E-05	0.000431678	10	0	0
MIEF1	0.6320706	0.681524959	-0.04945432	-0.13263297	6.34E-05	0.000431901	2	0	0
OR2W3	0.7730864	0.844676512	-0.07159012	-0.12620701	6.38E-05	0.000434379	1	0	0
ABCA4	0.7810985	0.837215351	-0.05611685	-0.10058953	6.40E-05	0.000435557	7	0	0
PLEKHM3	0.2109634	0.268902927	-0.05793952	-0.17783177	6.40E-05	0.000435557	11	0	0
TMPRSS11D	0.6818201	0.771012674	-0.08919253	-0.17494895	6.41E-05	0.000435731	1	0	0
BSPH1	0.6092649	0.679148464	-0.06988358	-0.14232728	6.44E-05	0.000437521	2	0	0
CLIC5	0.7863642	0.855117637	-0.06875341	-0.12530979	6.44E-05	0.000437604	4	0	0
HYAL1	0.8253483	0.867700875	-0.04235254	-0.07129633	6.47E-05	0.000439102	4	0	0
HMX2	0.2076485	0.136241484	0.071406988	0.786789507	6.47E-05	0.000439283	13	0	0
OR1E2	0.4266339	0.508793938	-0.08216001	-0.25134171	6.47E-05	0.000439283	5	0	0
PCDHAC2	0.2151363	0.181866311	0.033270036	0.491005221	6.48E-05	0.000439759	13	0	0
TNN	0.6930185	0.765670485	-0.07265198	-0.14042281	6.49E-05	0.000440282	8	0	0
GP5	0.6851016	0.750267001	-0.06516538	-0.13514767	6.50E-05	0.000440307	5	0	0
RNF213	0.2490717	0.179780089	0.069291631	0.326261389	6.52E-05	0.000441922	5	0	0
C3orf20	0.7714635	0.854257088	-0.08279363	-0.14831804	6.54E-05	0.000442822	8	0	0
PTGDS	0.6322914	0.646105687	-0.01381425	-0.0006989	6.54E-05	0.000442822	10	0	0
IFNA7	0.5618204	0.668388532	-0.10656818	-0.24364101	6.54E-05	0.000442957	2	0	0
TMEM37	0.7036657	0.730232832	-0.02656711	-0.04501523	6.55E-05	0.000442977	4	0	0
PHF2	0.2534874	0.194897159	0.05859026	0.316364247	6.56E-05	0.000443698	4	0	0
EP400	0.2802254	0.360287492	-0.08006209	-0.23870832	6.61E-05	0.000446474	3	0	0
CREBBP	0.2692748	0.316413904	-0.04713907	-0.13226135	6.61E-05	0.000446474	5	0	0
PODXL	0.3496193	0.313147878	0.036471442	0.605231366	6.62E-05	0.000447157	7	0	0
TRIM10	0.8424347	0.872552582	-0.03011785	-0.04476412	6.63E-05	0.000447475	11	0	0
TMEM82	0.3600247	0.375814508	-0.0157898	-0.0872694	6.64E-05	0.000448124	6	0	0
C1QL2	0.2402472	0.171478054	0.068769195	0.845289113	6.67E-05	0.000449561	9	0	0
KIAA1211	0.7471247	0.831425227	-0.08430052	-0.15500381	6.67E-05	0.000449561	3	0	0
TESPA1	0.4187399	0.504403938	-0.08566405	-0.30003655	6.68E-05	0.000450007	8	0	0
ZNF366	0.5858817	0.524461433	0.061420289	0.171202707	6.69E-05	0.000450473	6	0	0
OR6C68	0.8522326	0.902842319	-0.05060974	-0.08290079	6.69E-05	0.000450817	2	0	0
SLC9B2	0.9242829	0.935668768	-0.01138588	-0.01747551	6.70E-05	0.00045106	1	0	0
FIBIN	0.3126682	0.229358347	0.083309888	0.509611012	6.70E-05	0.000451093	6	0	0
FNDC1	0.2606902	0.182502965	0.078187273	1.06428281	6.72E-05	0.000452385	6	0	0
C17orf47	0.7357032	0.802687893	-0.06698466	-0.13156877	6.76E-05	0.000454449	8	0	0
DOK3	0.6618706	0.705328899	-0.04345827	-0.09937881	6.79E-05	0.000456374	7	0	0
SAMD10	0.1324523	0.158810613	-0.02635836	-0.15658463	6.80E-05	0.000456562	10	0	0
PRPF6	0.1324523	0.158810613	-0.02635836	-0.15658463	6.80E-05	0.000456562	10	0	0
TRIM71	0.1166364	0.046758343	0.069878018	1.240649749	6.81E-05	0.000457202	11	0	0
RRNAD1	0.1252901	0.154447436	-0.02915737	-0.19266015	6.83E-05	0.000457638	16	0	0
PRKAG3	0.648127	0.654789704	-0.00666266	0.000460902	6.82E-05	0.000457638	6	0	0
PLAC8L1	0.891412	0.898473966	-0.00706201	-0.0111964	6.82E-05	0.000457638	5	0	0
CACNG1	0.7362025	0.81886612	-0.08266364	-0.15441072	6.84E-05	0.000458783	14	0	0
SETSIP	0.7145146	0.787846553	-0.07333197	-0.13909657	6.86E-05	0.000459736	1	0	0
SCN11A	0.7239801	0.793079816	-0.06909974	-0.13393329	6.86E-05	0.000459774	3	0	0

TOPAZ1	0.8699763	0.905236607	-0.03526035	-0.05808907	6.87E-05	0.00045986	13	0	0
USP17L7	0.6896028	0.77357194	-0.08396912	-0.16352963	6.87E-05	0.000459864	1	0	0
OTOS	0.5871269	0.664197115	-0.07707026	-0.21744547	6.88E-05	0.000460516	7	0	0
LST1	0.3990353	0.482700896	-0.08366555	-0.27136404	6.89E-05	0.000460643	3	0	0
BCL6B	0.2947247	0.235408002	0.059316735	0.415507894	6.91E-05	0.000461618	9	0	0
FAM13B	0.7481608	0.69098963	0.057171209	0.113110837	6.94E-05	0.000463619	1	0	0
KRT6B	0.5406389	0.629969283	-0.08933035	-0.17888369	6.94E-05	0.000463619	2	0	0
PNLIP	0.6675943	0.756646303	-0.089052	-0.17860092	6.97E-05	0.000465304	6	0	0
FEZF1	0.2298331	0.14107026	0.088762804	0.867957852	6.97E-05	0.000465484	4	0	0
MROH2A	0.7941892	0.840376516	-0.04618729	-0.0853303	7.00E-05	0.000466966	4	0	0
PMEPA1	0.6588293	0.699175687	-0.04034635	-0.07106335	7.00E-05	0.000466966	9	0	0
C6orf10	0.6838554	0.727898835	-0.04404347	-0.07456975	7.01E-05	0.000467603	3	0	0
SEMA3A	0.771026	0.854270827	-0.08324478	-0.14639726	7.02E-05	0.000467983	3	0	0
SRSF8	0.5311722	0.574085888	-0.04291364	0.012861055	7.02E-05	0.000467983	5	0	0
TRPM2	0.4897937	0.434100974	0.055692755	0.357861231	7.05E-05	0.000469431	6	0	0
DOK4	0.7567164	0.805116668	-0.04840022	-0.09499408	7.05E-05	0.000469652	4	0	0
RGS3	0.696735	0.790888944	-0.09415389	-0.1777127	7.08E-05	0.000471026	3	0	0
CHAF1B	0.1797706	0.186512184	-0.00674157	-0.047442	7.09E-05	0.000471591	6	0	0
ATCAY	0.5144936	0.617436989	-0.1029434	-0.2642998	7.09E-05	0.000471669	4	0	0
INPP4B	0.1175269	0.166518274	-0.04899139	-0.29332948	7.15E-05	0.000475103	3	0	0
GAB2	0.3414345	0.327666929	0.013767547	-0.06089678	7.17E-05	0.000476396	5	0	0
CHODL	0.7241847	0.851923868	-0.12773912	-0.23067383	7.17E-05	0.000476396	2	0	0
BRDT	0.6167772	0.575073337	0.041703821	0.195535565	7.18E-05	0.000476633	8	0	0
BCL2A1	0.4656317	0.550234055	-0.08460238	-0.28316564	7.20E-05	0.000477449	4	0	0
DDT	0.7446856	0.798778909	-0.05409327	-0.09986955	7.19E-05	0.000477449	1	0	0
FGF17	0.3687363	0.308082176	0.060654126	0.389925289	7.21E-05	0.000478103	9	0	0
ZNF333	0.1689781	0.213532843	-0.04455477	-0.07137313	7.22E-05	0.000478433	6	0	0
MPPED2	0.1132986	0.148084371	-0.03478576	-0.04340206	7.22E-05	0.000478692	9	0	0
PCDHGB7	0.4550292	0.389826577	0.065202624	0.62038916	7.25E-05	0.000480666	8	0	0
FAM183A	0.5744746	0.518901825	0.05557279	0.283677179	7.26E-05	0.000480795	5	0	0
TOMM20L	0.2646705	0.294042331	-0.02937178	-0.1236843	7.26E-05	0.000480941	14	0	0
MTRNR2L4	0.7629506	0.849441673	-0.08649108	-0.1532058	7.28E-05	0.000481706	2	0	0
KLB	0.6447576	0.586932663	0.057824902	0.152970199	7.29E-05	0.000482481	8	0	0
C11orf94	0.5474025	0.618988947	-0.07158649	-0.18252061	7.30E-05	0.000482962	10	0	0
RCVRN	0.3888095	0.441824004	-0.05301452	-0.16702474	7.31E-05	0.000483325	3	0	0
SULT6B1	0.1314691	0.183015664	-0.05154652	-0.26323769	7.33E-05	0.000484331	8	0	0
TEKT2	0.4245104	0.419727865	0.004782539	0.360223053	7.36E-05	0.000485476	5	0	0
KCNA10	0.6607671	0.717112844	-0.05634574	-0.1117075	7.35E-05	0.000485476	8	0	0
KRT32	0.7198989	0.783010219	-0.06311129	-0.12021209	7.36E-05	0.000485476	3	0	0
CLEC11A	0.3965851	0.316612124	0.079972938	0.375347038	7.36E-05	0.000485476	8	0	0
CRNKL1	0.6885172	0.747080127	-0.05856293	-0.1406485	7.36E-05	0.000485609	10	0	0
LRRC72	0.8535432	0.888600046	-0.03505689	-0.05885237	7.38E-05	0.00048655	8	0	0
NEFL	0.1495339	0.087721091	0.061812829	0.789262241	7.39E-05	0.000487029	10	0	0
CLDN19	0.6591858	0.618042658	0.041143114	0.09681078	7.44E-05	0.000489892	10	0	0
ZNF91	0.4143517	0.436317308	-0.0219656	0.183679827	7.44E-05	0.000489892	6	0	0
XRCC5	0.713966	0.763708397	-0.04974243	-0.09888977	7.46E-05	0.000491011	4	0	0
PIP4K2C	0.1452226	0.168939596	-0.02371704	-0.26874972	7.46E-05	0.000491197	10	0	0
LIPF	0.694389	0.759913614	-0.06552465	-0.14231334	7.47E-05	0.0004914	4	0	0
ANKRD65	0.5901859	0.665305918	-0.07512006	-0.1719884	7.48E-05	0.000491569	10	0	0
MFAP5	0.7344291	0.821850708	-0.08742156	-0.16570969	7.48E-05	0.000491569	8	0	0
ZNF200	0.8288083	0.911626374	-0.08281808	-0.13571547	7.48E-05	0.000491569	2	0	0
FKBP8	0.2059197	0.252665277	-0.04674556	-0.31336367	7.48E-05	0.000491569	5	0	0
TACC2	0.8152564	0.82595162	-0.01069524	-0.01917566	7.49E-05	0.000491611	3	0	0
FRMPD2	0.7187151	0.685502682	0.033212383	0.076748093	7.49E-05	0.000491657	8	0	0
NINJ1	0.1782551	0.211645572	-0.03339045	-0.11344151	7.52E-05	0.000493736	5	0	0
RD3	0.728378	0.749160261	-0.02078227	-0.0446864	7.53E-05	0.000494279	8	0	0
ABCC1	0.1602332	0.188731622	-0.02849847	-0.09766103	7.54E-05	0.000494557	9	0	0
ZNF814	0.2929749	0.185360321	0.107614572	0.809124446	7.56E-05	0.00049572	7	0	0
FAM110D	0.6401278	0.616600126	0.023527644	0.057927421	7.59E-05	0.000497587	5	0	0
EHD1	0.1792562	0.126038847	0.053217367	0.439636288	7.60E-05	0.000497614	3	0	0
TSLP	0.671036	0.709270982	-0.03823495	-0.05523682	7.61E-05	0.000498013	3	0	0
DUSP22	0.3626363	0.423097515	-0.06046124	-0.22803901	7.61E-05	0.000498354	4	0	0
C2CD4D	0.6376207	0.682337152	-0.04471645	0.029035183	7.65E-05	0.000500519	4	0	0

GALNT9	0.2467135	0.220374169	0.026339376	0.574295348	7.66E-05	0.000500892	12	0	0
SIPA1L3	0.0710826	0.098718679	-0.02763608	-0.19417751	7.69E-05	0.000502781	10	0	0
ARHGAP9	0.5400985	0.603963171	-0.06386463	-0.12189931	7.71E-05	0.000503944	4	0	0
FAM43B	0.3226967	0.279539941	0.043156715	0.512070531	7.71E-05	0.000503968	11	0	0
OR4N4	0.8219212	0.884058514	-0.06213731	-0.10392237	7.73E-05	0.000504693	1	0	0
TNP1	0.7942676	0.853190363	-0.05892272	-0.10910295	7.74E-05	0.000505437	5	0	0
CNTN4	0.1571073	0.096576235	0.060531043	0.578266989	7.75E-05	0.000505682	6	0	0
PTP4A3	0.4664746	0.537349295	-0.07087471	-0.15105135	7.77E-05	0.000507227	4	0	0
DLX6	0.2645394	0.189368725	0.075170715	0.322745476	7.78E-05	0.00050762	10	0	0
INSL3	0.7952479	0.762456888	0.032791003	0.068798462	7.83E-05	0.000510819	11	0	0
GNGT2	0.4357791	0.357019734	0.078759361	0.364331146	7.84E-05	0.000511017	12	0	0
CHRM1	0.3967124	0.447529636	-0.05081723	-0.16920115	7.87E-05	0.000512975	9	0	0
GTSF1	0.6179097	0.657154999	-0.03924526	-0.03162594	7.89E-05	0.000514021	8	0	0
HCRTR1	0.3084874	0.352968373	-0.04448097	0.0177165	7.90E-05	0.000514507	7	0	0
GPX5	0.5781427	0.596636538	-0.01849386	-0.02904806	7.91E-05	0.000514721	9	0	0
MTRNR2L5	0.79746	0.855693624	-0.05823358	-0.10261333	8.02E-05	0.000521599	3	0	0
VNN2	0.6553293	0.689318944	-0.03398968	-0.02615969	8.04E-05	0.000522825	5	0	0
SPATA32	0.2966646	0.243936962	0.05272768	1.018209268	8.04E-05	0.000522825	11	0	0
NEIL2	0.2952688	0.339761931	-0.04449316	-0.1329312	8.07E-05	0.000524597	9	0	0
MAGEL2	0.6292612	0.692059873	-0.06279871	-0.12804125	8.12E-05	0.000527309	8	0	0
OSBPL1A	0.2206356	0.277485939	-0.05685032	-0.09944445	8.17E-05	0.000530052	9	0	0
SMIM4	0.2427534	0.267475539	-0.02472215	-0.14628334	8.19E-05	0.000531071	9	0	0
RXFP1	0.6018006	0.675950028	-0.07414948	-0.17277634	8.23E-05	0.000533274	2	0	0
SAMD15	0.1845888	0.21221313	-0.02762432	-0.00309412	8.23E-05	0.000533274	5	0	0
ERGIC2	0.1757702	0.2255608	-0.04979061	-0.22353575	8.25E-05	0.000534783	11	0	0
SIAH1	0.7720388	0.832262366	-0.06022354	-0.11026218	8.26E-05	0.000534783	8	0	0
GBP5	0.8478475	0.890236075	-0.0423886	-0.069729	8.35E-05	0.000540585	3	0	0
ADCY3	0.0593555	0.076280064	-0.01692453	-0.1979324	8.36E-05	0.000540974	8	0	0
GDF3	0.3615857	0.300783453	0.06080222	0.574649947	8.39E-05	0.000542839	6	0	0
ZFR2	0.2981437	0.274608725	0.02353497	0.483975733	8.39E-05	0.000542839	12	0	0
RAC1	0.3064916	0.317755171	-0.01126359	0.087625626	8.43E-05	0.00054537	4	0	0
DSPP	0.7715209	0.837616631	-0.0660957	-0.11852005	8.45E-05	0.000545992	8	0	0
RBKS	0.1775049	0.231039897	-0.05353497	-0.15277017	8.45E-05	0.000546163	11	0	0
CD53	0.578059	0.654593699	-0.07653469	-0.20267162	8.46E-05	0.000546464	8	0	0
AOAH	0.5976506	0.663377724	-0.06572715	-0.25654324	8.48E-05	0.000547433	6	0	0
TAF1B	0.3048066	0.387710582	-0.08290398	-0.20604466	8.49E-05	0.000548025	7	0	0
FGF18	0.1403426	0.174786755	-0.03444414	-0.22520783	8.50E-05	0.000548307	5	0	0
SPHK1	0.6741709	0.591392573	0.082778339	0.187459088	8.50E-05	0.000548338	3	0	0
MOBP	0.6746973	0.723626254	-0.04892895	-0.09741084	8.56E-05	0.000551599	6	0	0
PHYHIP	0.6202938	0.557608054	0.062685737	0.435113765	8.56E-05	0.000551599	10	0	0
FAM170B	0.5891615	0.687896996	-0.09873547	-0.2126336	8.56E-05	0.000551599	4	0	0
BCR	0.4672763	0.471090705	-0.00381444	0.10252635	8.58E-05	0.000552749	7	0	0
CRHR2	0.6070661	0.687469164	-0.08040305	-0.1823889	8.64E-05	0.000556002	4	0	0
HOXC5	0.2277742	0.194047432	0.033726762	0.143586179	8.66E-05	0.000557481	12	0	0
WFIKKN2	0.7087407	0.680378593	0.028362106	0.146739283	8.67E-05	0.000558002	11	0	0
TSPO2	0.7910959	0.821400368	-0.03030447	-0.0626753	8.68E-05	0.000558407	7	0	0
EDN2	0.5090333	0.500100051	0.00893322	0.090714385	8.71E-05	0.000559754	9	0	0
PLEKHA7	0.0744379	0.091408478	-0.01697062	-0.2119744	8.71E-05	0.000560036	8	0	0
NOTCH4	0.5073694	0.496065033	0.011304387	0.076136812	8.74E-05	0.000561322	6	0	0
GABBR2	0.186495	0.122789976	0.063705024	1.042456164	8.81E-05	0.000565741	4	0	0
CLCA1	0.6751927	0.709661665	-0.03446894	-0.09644435	8.86E-05	0.000568451	6	0	0
CST9L	0.6542977	0.730529582	-0.07623191	-0.15026384	8.86E-05	0.000568451	6	0	0
MEOX2	0.1999159	0.123268343	0.076647529	0.814029582	8.88E-05	0.00056918	13	0	0
GPC6	0.277209	0.304011652	-0.02680262	0.158278053	8.87E-05	0.00056918	8	0	0
SFRP1	0.2293341	0.148920015	0.080414103	0.672709448	8.90E-05	0.000570502	17	0	0
CPT1A	0.1794675	0.228176326	-0.04870885	-0.32981231	8.92E-05	0.0005718	3	0	0
HPGDS	0.5430055	0.615210101	-0.07220461	-0.23336016	8.94E-05	0.000572529	6	0	0
SLTM	0.0548965	0.074539089	-0.01964258	-0.22064913	8.95E-05	0.000573341	2	0	0
ZNF665	0.3045256	0.24680324	0.05772235	0.917741141	8.96E-05	0.000573426	8	0	0
CRYGD	0.5594489	0.525892861	0.033556032	0.295412959	8.97E-05	0.000573718	10	0	0
RAB43	0.6150015	0.573996837	0.041004614	0.139045177	8.97E-05	0.000573718	5	0	0
NYNRIN	0.2916838	0.232080949	0.059602809	0.243175214	9.00E-05	0.000574968	8	0	0
SULT1A2	0.6206364	0.555052252	0.065584158	0.179906489	9.01E-05	0.000575529	4	0	0

CST3	0.7197394	0.775126673	-0.05538725	-0.12769025	9.01E-05	0.000575529	4	0	0
PRSS16	0.5860438	0.53861666	0.047427137	0.144738142	9.02E-05	0.000576134	6	0	0
ERMN	0.4154137	0.392402785	0.023010931	-0.00366108	9.04E-05	0.000577328	8	0	0
TMEM108	0.3716967	0.350806322	0.020890403	0.597036162	9.06E-05	0.000577805	18	0	0
SMYD5	0.2289889	0.274668865	-0.04567998	-0.19827739	9.07E-05	0.000578295	9	0	0
DYTN	0.7798549	0.82218035	-0.04232548	-0.08284505	9.15E-05	0.000583317	3	0	0
EBNA1BP2	0.0940122	0.119347352	-0.02533517	-0.28583225	9.15E-05	0.000583513	3	0	0
EDDM3A	0.8440001	0.865598555	-0.02159842	-0.03422997	9.17E-05	0.00058424	5	0	0
HLF	0.2304605	0.137046533	0.093413941	0.584862025	9.17E-05	0.000584255	10	0	0
FXYD3	0.3141758	0.387824452	-0.07364869	-0.22870813	9.18E-05	0.000584255	12	0	0
CA9	0.6651761	0.723870802	-0.05869475	-0.12793751	9.18E-05	0.0005846	6	0	0
MAS1L	0.709594	0.786291084	-0.07669705	-0.15102883	9.19E-05	0.000584719	6	0	0
RUNDC3B	0.2106539	0.222391276	-0.01173733	0.431752694	9.20E-05	0.000584844	6	0	0
UCP1	0.2402538	0.197580233	0.042673533	0.701000656	9.20E-05	0.000585102	7	0	0
TXNDC2	0.8172227	0.865077259	-0.04785452	-0.08282907	9.21E-05	0.000585102	8	0	0
CORO1A	0.7311343	0.782343941	-0.05120968	-0.12323551	9.24E-05	0.000587157	2	0	0
CHSY3	0.2632432	0.286718591	-0.02347537	0.023699074	9.26E-05	0.000587877	6	0	0
OR5P3	0.5240039	0.606360408	-0.08235654	-0.20962538	9.27E-05	0.000588382	4	0	0
CPNE3	0.8127744	0.886927668	-0.0741533	-0.13392677	9.29E-05	0.000589526	3	0	0
SLC5A12	0.5890679	0.700248985	-0.11118112	-0.24560542	9.29E-05	0.000589526	1	0	0
VHL	0.805206	0.868147091	-0.06294106	-0.10607741	9.34E-05	0.000591978	6	0	0
SFTPA1	0.4253241	0.493561821	-0.06823777	-0.21072733	9.34E-05	0.000591978	4	0	0
WWC1	0.1744081	0.164268559	0.01013958	-0.08363338	9.34E-05	0.000592139	9	0	0
AAK1	0.7416131	0.656914205	0.084698932	0.172489105	9.35E-05	0.000592177	1	0	0
SLA	0.5861617	0.655354026	-0.06919235	-0.1518535	9.36E-05	0.000592177	8	0	0
B3GNT6	0.6616248	0.70601104	-0.04438622	-0.10913476	9.35E-05	0.000592177	4	0	0
MTMR6	0.3918302	0.462440598	-0.07061038	-0.1598753	9.36E-05	0.000592177	8	0	0
ZAR1L	0.1580588	0.200701025	-0.04264219	-0.21365228	9.38E-05	0.000593384	11	0	0
PCP4	0.3910614	0.449286435	-0.05822499	-0.18969831	9.38E-05	0.000593384	8	0	0
MAVS	0.0492581	0.062808631	-0.0135505	-0.17145554	9.39E-05	0.000593625	8	0	0
DHRS7	0.8570134	0.892152614	-0.03513918	-0.05773532	9.40E-05	0.000594161	4	0	0
SLC25A23	0.6792597	0.617176207	0.06208353	0.159258444	9.41E-05	0.000594481	2	0	0
PVALB	0.5480745	0.623518891	-0.0754444	-0.17084483	9.43E-05	0.000595782	7	0	0
RSPO1	0.1869537	0.131878976	0.055074685	0.735892478	9.46E-05	0.000597438	10	0	0
SIGLEC15	0.5841018	0.649502883	-0.06540112	-0.1512379	9.47E-05	0.000597931	5	0	0
MAG	0.5340131	0.614546768	-0.08053369	-0.19924151	9.54E-05	0.000601636	9	0	0
CAMKK2	0.8698832	0.932305998	-0.06242283	-0.10422459	9.57E-05	0.000603318	2	0	0
IL12B	0.3486535	0.393794225	-0.04514077	-0.01984749	9.58E-05	0.000603781	11	0	0
OTP	0.3291842	0.25370428	0.075479938	0.501631543	9.61E-05	0.000605533	6	0	0
PTGFRN	0.2604159	0.260395728	2.01E-05	0.231293817	9.63E-05	0.000606406	8	0	0
EPS8L3	0.6666266	0.733703226	-0.06707667	-0.13821794	9.67E-05	0.000608718	6	0	0
ACTRT2	0.521454	0.580660012	-0.05920603	-0.12707704	9.68E-05	0.000608947	6	0	0
FCGR1A	0.6033757	0.697455236	-0.09407957	-0.20586794	9.68E-05	0.000608947	1	0	0
OR52M1	0.6110892	0.722525102	-0.11143587	-0.25569658	9.68E-05	0.000608947	2	0	0
FZD10	0.1719292	0.109484687	0.06244448	0.6424046	9.68E-05	0.000608947	9	0	0
AKR1B10	0.6720558	0.736867281	-0.06481152	-0.12745106	9.71E-05	0.000610595	5	0	0
DDB1	0.6880766	0.732040933	-0.04396431	-0.0861565	9.72E-05	0.000610708	6	0	0
SLC45A2	0.7087846	0.743077601	-0.03429295	-0.06849196	9.75E-05	0.000612039	10	0	0
PSMG4	0.1546379	0.103307562	0.051330291	0.560211591	9.75E-05	0.000612039	7	0	0
HLA-DPB1	0.4231759	0.495447901	-0.07227201	-0.23194682	9.74E-05	0.000612039	8	0	0
GBX2	0.1976608	0.116864664	0.08079613	0.81143579	9.75E-05	0.000612094	8	0	0
OR11A1	0.6504856	0.739816368	-0.08933074	-0.20510073	9.76E-05	0.000612546	7	0	0
FOLR2	0.7722693	0.797537437	-0.02526817	-0.04063212	9.77E-05	0.000612746	5	0	0
OR2L3	0.4870297	0.605998105	-0.11896841	-0.27954778	9.82E-05	0.00061532	4	0	0
LTB	0.6244256	0.68745987	-0.06303431	-0.17041353	9.83E-05	0.000616216	6	0	0
TMEM213	0.6103767	0.555576979	0.054799705	0.180742194	9.84E-05	0.000616701	6	0	0
ERN2	0.6064807	0.574056261	0.032424419	0.149206287	9.86E-05	0.000617255	8	0	0
NDNF	0.1356656	0.074680351	0.060985252	0.853095455	9.90E-05	0.000619771	4	0	0
CCL23	0.6574225	0.735377737	-0.07795528	-0.14201928	9.92E-05	0.000620663	6	0	0
CDC42BPA	0.1366726	0.11393278	0.022739812	0.31087941	9.95E-05	0.000622388	7	0	0
C14orf177	0.4633343	0.533325957	-0.06999165	-0.27641615	9.96E-05	0.000622604	3	0	0
SSB	0.8123438	0.842815703	-0.03047188	-0.05249229	9.97E-05	0.000622986	1	0	0
CRCT1	0.7299178	0.803507396	-0.07358958	-0.14044967	9.98E-05	0.000623538	3	0	0

PRODH2	0.6655903	0.771214552	-0.1056242	-0.20128331	9.99E-05	0.000624347	3	0	0
PFKM	0.2437725	0.307718907	-0.06394642	-0.20125494	0.000100066	0.000624926	5	0	0
EIF2AK2	0.2920347	0.334381484	-0.04234678	-0.12963538	0.000100384	0.000626692	6	0	0
PBK	0.1948359	0.235235234	-0.04039936	-0.15178328	0.000100677	0.000628303	11	0	0
TAT	0.667158	0.774688153	-0.10753019	-0.23009567	0.000100716	0.000628326	3	0	0
DDIT4L	0.2137803	0.146454723	0.067325565	0.65898604	0.000100809	0.000628686	10	0	0
ARHGEF25	0.5916333	0.534662091	0.056971233	0.159376455	0.000101486	0.000632691	10	0	0
SNX20	0.5001391	0.568209469	-0.06807039	-0.19288335	0.000101772	0.000634252	10	0	0
DLG5	0.1161787	0.084227154	0.031951516	0.304536924	0.000102279	0.000637195	12	0	0
EMX2	0.2076873	0.13748624	0.070201054	0.798516255	0.000102397	0.000637484	15	0	0
FAM178B	0.5493522	0.660415428	-0.1110632	-0.26129832	0.000102434	0.000637493	1	0	0
JAGN1	0.2782075	0.279326862	-0.00111933	0.0993663	0.000102592	0.000638255	7	0	0
PSMB2	0.2262156	0.296317044	-0.0701014	-0.23693351	0.000102828	0.000639504	7	0	0
FADS3	0.3555759	0.408970025	-0.05339408	-0.17491753	0.000102984	0.000640248	9	0	0
TIGIT	0.7686854	0.889376748	-0.12069132	-0.20788497	0.000103122	0.000640888	1	0	0
ZNF575	0.8996498	0.917418026	-0.01776825	-0.02790857	0.000103162	0.000640912	1	0	0
ITGA8	0.2853464	0.269706635	0.015639796	0.427444686	0.000103779	0.000644522	6	0	0
FAM162B	0.3420798	0.309553655	0.032526109	0.563153834	0.000103833	0.000644632	14	0	0
ADCYAP1R1	0.1924117	0.186204515	0.0062072	0.719930825	0.000104388	0.000647858	5	0	0
TDRD10	0.322181	0.249147725	0.073033226	0.500133184	0.00010465	0.00064926	10	0	0
CHRM3	0.1324403	0.076021407	0.056418879	0.765223117	0.000104696	0.000649319	8	0	0
SHISA6	0.2524806	0.185550041	0.066930565	0.475922997	0.000104812	0.000649811	7	0	0
TAS2R38	0.7378463	0.804114589	-0.0662683	-0.13154315	0.000105395	0.000653201	8	0	0
RHCG	0.271878	0.201371767	0.070506203	0.780888234	0.000105876	0.000655959	16	0	0
ASCL5	0.6466226	0.696765232	-0.05014268	-0.10796815	0.000105995	0.000656318	3	0	0
FRAT2	0.1593693	0.214266292	-0.05489702	-0.26951103	0.000106008	0.000656318	4	0	0
CSTF3	0.6090561	0.65632157	-0.04726551	-0.27778733	0.000106241	0.000657537	3	0	0
HOXC9	0.2062157	0.143864475	0.062351178	0.481385296	0.000106381	0.000658177	7	0	0
PXDNL	0.6919843	0.636354589	0.05562975	0.134224592	0.000106467	0.000658477	11	0	0
S100A5	0.6853174	0.638465462	0.046851964	0.152498852	0.000106563	0.000658617	7	0	0
SLC4A8	0.250594	0.287933684	-0.03733971	-0.19599091	0.000106527	0.000658617	11	0	0
RHOA	0.5707259	0.61161976	-0.04089387	-0.10074645	0.000106659	0.000658761	6	0	0
SFT2D1	0.0959495	0.1413334	-0.04538394	-0.25274551	0.000106641	0.000658761	7	0	0
CDK14	0.7499903	0.83731928	-0.08732901	-0.17657158	0.000106713	0.000658868	2	0	0
LYPD5	0.3782565	0.371747516	0.006508996	0.135214664	0.000107214	0.00066173	10	0	0
HHIP	0.2461867	0.184828077	0.0613586	0.402164086	0.000107328	0.000662207	10	0	0
LRFN1	0.8216829	0.863635641	-0.04195278	-0.07255842	0.000107449	0.000662723	10	0	0
COA7	0.1990478	0.217989402	-0.01894159	-0.16238678	0.000107944	0.000665318	8	0	0
CRY1	0.1386903	0.137420147	0.001270167	0.227202146	0.000107925	0.000665318	11	0	0
LILRB5	0.5938594	0.695445755	-0.10158633	-0.2194589	0.000108247	0.000666958	5	0	0
OR8B2	0.7193069	0.831008683	-0.11170175	-0.20559458	0.000108823	0.000670276	1	0	0
FREM1	0.757269	0.795830486	-0.0385615	-0.07368718	0.000108948	0.000670816	4	0	0
HSD17B1	0.4817883	0.553847449	-0.07205915	-0.22077208	0.000109069	0.000671332	5	0	0
PROZ	0.6623201	0.685078219	-0.0227581	-0.03343645	0.000109107	0.000671337	10	0	0
ZNF860	0.2376249	0.284692533	-0.04706765	-0.20113317	0.000109149	0.000671365	10	0	0
AIP	0.1623782	0.210119627	-0.04774145	-0.15675428	0.000109881	0.000675635	10	0	0
ATP6V1B1	0.5734022	0.619712498	-0.04631029	-0.1288806	0.000109929	0.000675698	4	0	0
ZFP42	0.418674	0.32294274	0.09573131	0.519695902	0.000110211	0.0006772	13	0	0
RPL3L	0.7138182	0.691044419	0.022773758	0.056093769	0.000110422	0.000678264	4	0	0
ZNF675	0.3793724	0.41560331	-0.0362309	0.197038322	0.000110714	0.000679822	2	0	0
KLHL40	0.5771034	0.530174616	0.046928751	0.176042553	0.000111577	0.000684423	11	0	0
A2M	0.5184052	0.444226647	0.074178574	0.186562308	0.00011157	0.000684423	9	0	0
NPEPPS	0.8188416	0.852889544	-0.03404795	-0.05807949	0.000111558	0.000684423	1	0	0
OR4A47	0.2798904	0.365616303	-0.08572593	-0.37375224	0.000111626	0.000684487	1	0	0
KLF17	0.7946058	0.857767826	-0.06316201	-0.11154783	0.000111901	0.000685787	8	0	0
DCDC2	0.2472479	0.181880037	0.065367852	0.636747929	0.000111914	0.000685787	10	0	0
LAMA3	0.131652	0.134227081	-0.00257509	0.105673952	0.000112153	0.000687014	9	0	0
CASQ1	0.6100637	0.635244457	-0.02518079	-0.03001416	0.00011248	0.000688781	15	0	0
LYZL4	0.6851582	0.750408374	-0.06525013	-0.14082901	0.000112704	0.000689919	7	0	0
CD163L1	0.7023035	0.804770054	-0.10246652	-0.19390081	0.000112992	0.00069121	1	0	0
HOXC4	0.1743941	0.140514154	0.033879985	0.098393152	0.000112985	0.00069121	22	0	0
TUBB2B	0.1749889	0.113442862	0.061546011	0.691846757	0.000113043	0.000691283	9	0	0
RPAP3	0.160788	0.193008135	-0.03222013	-0.21313219	0.000113146	0.000691679	8	0	0

FAM151A	0.6009583	0.535986887	0.064971446	0.194840785	0.000113672	0.000694519	5	0	0
POU3F3	0.2448711	0.152738678	0.092132416	0.803458915	0.000113688	0.000694519	10	0	0
DPEP2	0.6237889	0.58727914	0.036509756	0.114909436	0.000113914	0.000695666	6	0	0
OR2T27	0.4161114	0.54477161	-0.12866022	-0.38066334	0.00011414	0.000696807	1	0	0
SSC5D	0.5488864	0.484638707	0.06424772	0.172081134	0.000114778	0.000700461	5	0	0
PRB2	0.3531086	0.415307581	-0.06219897	-0.03286647	0.000114845	0.000700633	5	0	0
SERPINB9	0.1430567	0.060315202	0.082741501	1.158503998	0.000115534	0.000704115	3	0	0
STX3	0.7015821	0.793573508	-0.09199144	-0.18963568	0.000115505	0.000704115	5	0	0
VWF	0.6793465	0.642826779	0.036519678	0.077385614	0.000115525	0.000704115	7	0	0
INA	0.1909249	0.119332217	0.071592665	1.254573113	0.000115618	0.00070439	10	0	0
ACP6	0.1194801	0.150688105	-0.03120804	-0.1646103	0.000115864	0.000705651	8	0	0
CNN2	0.2216135	0.248291046	-0.02667752	-0.16861602	0.000116055	0.00070657	4	0	0
ANO2	0.5566716	0.642635676	-0.08596409	-0.23664516	0.000116222	0.000707349	5	0	0
NENF	0.3130143	0.315309857	-0.00229556	-0.06764199	0.000116432	0.000708386	8	0	0
COL2A1	0.2852645	0.226644077	0.058620393	0.56841183	0.000116883	0.000710891	12	0	0
MYL6B	0.1020897	0.126642969	-0.02455323	-0.13686912	0.000117144	0.000711997	13	0	0
TUBB4A	0.3115632	0.288254423	0.023308801	0.087335611	0.000117141	0.000711997	12	0	0
NUGGC	0.6063024	0.651747416	-0.045445	-0.1555979	0.000117746	0.00071541	3	0	0
FOXJ3	0.2182815	0.250569264	-0.03228774	-0.17450636	0.00011783	0.000715437	15	0	0
METTL21C	0.7770571	0.836455185	-0.0593981	-0.11167535	0.000117828	0.000715437	7	0	0
XIRP1	0.663424	0.721448336	-0.05802431	-0.12184427	0.000119159	0.000723261	8	0	0
VSIG8	0.7984955	0.845908352	-0.04741282	-0.07953982	0.000119582	0.000725335	4	0	0
PTX4	0.6120696	0.66956025	-0.05749069	-0.14361521	0.000119546	0.000725335	3	0	0
C4BPB	0.7047379	0.654813584	0.049924289	0.105382081	0.00011992	0.000726899	4	0	0
DRD1	0.215723	0.171051152	0.044671874	0.568964442	0.00011991	0.000726899	7	0	0
SCARA5	0.3448513	0.324657132	0.020194211	0.362756331	0.000120054	0.00072746	8	0	0
KAAG1	0.6468132	0.626433948	0.020379263	0.092826335	0.000120334	0.000728913	6	0	0
C15orf53	0.522106	0.588557233	-0.06645128	-0.21351989	0.000120487	0.000729591	3	0	0
HK3	0.4666755	0.461870069	0.004805465	0.037619022	0.000121054	0.00073278	7	0	0
SLC16A9	0.8338262	0.890546928	-0.05672074	-0.09393092	0.000121305	0.000734048	2	0	0
ZP2	0.6043639	0.68768078	-0.08331686	-0.17889752	0.000121435	0.000734589	4	0	0
OLFML3	0.5140481	0.471791111	0.042256946	0.096767701	0.000121944	0.000737423	10	0	0
PCDHGA1	0.4132669	0.362505175	0.050761695	0.307656075	0.000122148	0.000738406	7	0	0
SLC2A14	0.5616723	0.628957849	-0.06728551	-0.17109771	0.000122248	0.000738757	4	0	0
TENM2	0.6270691	0.69254031	-0.06547121	-0.13968134	0.0001227	0.000740995	7	0	0
TEX35	0.7807476	0.820451883	-0.0397043	-0.07598419	0.000123233	0.000743709	7	0	0
CCL13	0.6933274	0.741155541	-0.04782815	-0.09094491	0.000123227	0.000743709	6	0	0
TDGF1	0.5478886	0.636097206	-0.08820863	-0.21475347	0.000123455	0.0007448	5	0	0
POR	0.7296558	0.793040595	-0.06338481	-0.11861888	0.000123835	0.000746842	1	0	0
TNFRSF10C	0.5902461	0.583209637	0.007036439	0.036103057	0.000123943	0.000747018	3	0	0
LTA4H	0.6397158	0.724477554	-0.08476175	-0.1900216	0.000123948	0.000747018	4	0	0
LIPC	0.7856286	0.849516926	-0.06388835	-0.11141835	0.000124269	0.000748452	2	0	0
RMI2	0.8683661	0.918196536	-0.04983039	-0.08421541	0.00012423	0.000748452	4	0	0
SPNS3	0.5007843	0.57240143	-0.07161708	-0.22535011	0.000124487	0.000749515	8	0	0
NKX6-3	0.7350162	0.797040217	-0.06202398	-0.1268661	0.000124565	0.000749732	7	0	0
GPER1	0.7733181	0.702059115	0.071259007	0.141694042	0.00012494	0.000751734	8	0	0
TRHDE	0.55397	0.681439287	-0.12746926	-0.29398408	0.000125051	0.000752153	1	0	0
MAP4	0.0613268	0.054043356	0.007283396	0.160790492	0.000125132	0.000752383	8	0	0
PRDX1	0.7763153	0.811738848	-0.03542356	-0.10077965	0.000125211	0.000752608	5	0	0
SIL1	0.1091449	0.116399711	-0.00725478	0.046170988	0.000125356	0.000753228	14	0	0
SLC12A1	0.2121056	0.17667369	0.03543188	0.333818557	0.000125508	0.000753888	17	0	0
TTC39B	0.2752512	0.333588166	-0.058337	-0.21137472	0.000126088	0.00075712	5	0	0
PIWIL3	0.6800996	0.745076565	-0.06497698	-0.12753545	0.000126281	0.000758025	3	0	0
WDR41	0.6169173	0.743420639	-0.12650338	-0.26518051	0.000126499	0.000759081	1	0	0
MT2A	0.3163691	0.245344178	0.071024938	0.256069377	0.00012656	0.000759189	11	0	0
FAM131B	0.3490203	0.32133094	0.027689339	0.264156243	0.000126788	0.000760305	7	0	0
PVR	0.3860887	0.383168609	0.002920127	0.320737553	0.000127065	0.000761709	9	0	0
SF3B2	0.5997427	0.550394235	0.049348493	0.181684243	0.000127226	0.000762423	5	0	0
GAB4	0.7341296	0.786400118	-0.05227047	-0.09787963	0.000128055	0.000767132	11	0	0
DCTPP1	0.1683537	0.182640172	-0.0142865	-0.05254299	0.000128132	0.000767337	13	0	0
CYP4X1	0.4583198	0.54502375	-0.08670399	-0.2542656	0.000128459	0.00076904	2	0	0
SLCO1C1	0.5591258	0.632960677	-0.07383487	-0.15118125	0.000128559	0.000769125	9	0	0
LMAN1L	0.6523955	0.654159502	-0.00176405	-0.0049506	0.000128526	0.000769125	8	0	0

NDUFA13	0.3756633	0.377352322	-0.00168901	0.001123978	0.000128922	0.000771039	15	0	0
MAP7	0.2265839	0.260300055	-0.03371619	-0.28617953	0.000129087	0.000771767	12	0	0
C3orf84	0.8389159	0.872128968	-0.0332131	-0.05503942	0.000129155	0.000771918	4	0	0
IQCF6	0.818231	0.864534759	-0.04630372	-0.08061183	0.000130609	0.000780349	9	0	0
LRP1B	0.390967	0.484398596	-0.09343162	-0.23618507	0.000130862	0.000781601	9	0	0
CTXN3	0.6630875	0.751076199	-0.08798866	-0.19292691	0.000131132	0.000782901	6	0	0
PDPN	0.2767167	0.211366325	0.065350347	0.557598062	0.000131374	0.000783876	12	0	0
MFAP4	0.483691	0.432562697	0.051128263	0.224793157	0.000131952	0.000787062	8	0	0
ARMS2	0.7652161	0.83392472	-0.06870861	-0.12705544	0.000132007	0.000787125	4	0	0
P2RY11	0.6744664	0.726257453	-0.05179103	-0.1311463	0.000132129	0.00078759	9	0	0
HRASLS2	0.7110527	0.747266005	-0.03621335	-0.13459968	0.000132192	0.000787707	6	0	0
WT1	0.1506694	0.079422608	0.071246806	0.951140583	0.000133328	0.00079421	15	0	0
ASTE1	0.85414	0.880837369	-0.02669737	-0.04511121	0.000133465	0.000794765	7	0	0
ADHFE1	0.0688093	0.094655241	-0.02584599	-0.19585131	0.000133648	0.000795587	9	0	0
WDR83OS	0.2402357	0.166395645	0.073840063	0.50447273	0.000134337	0.000799158	1	0	0
PSG1	0.6486476	0.709180643	-0.06053302	-0.11265753	0.00013432	0.000799158	2	0	0
RSC1A1	0.8410951	0.86626243	-0.02516738	-0.04202802	0.000134967	0.000802641	2	0	0
PRLR	0.4476124	0.522333288	-0.07472084	-0.20193248	0.000135018	0.000802677	11	0	0
CREB1	0.1411038	0.1938065	-0.05270271	-0.17130926	0.000135118	0.00080276	7	0	0
SLC14A2	0.4145519	0.46979865	-0.05524674	-0.20792047	0.000135121	0.00080276	8	0	0
OR2T4	0.5221069	0.602708468	-0.0806016	-0.2231175	0.000135234	0.000803165	2	0	0
SLC27A4	0.3527987	0.411470864	-0.05867213	-0.11807076	0.000135473	0.000804053	6	0	0
IMP3	0.45087	0.398456478	0.052413562	0.211360711	0.000135459	0.000804053	11	0	0
CLCNKA	0.6226537	0.582800505	0.039853189	0.10227477	0.000135638	0.000804766	7	0	0
OBP2A	0.7702957	0.844931286	-0.0746356	-0.13178755	0.000135733	0.00080506	1	0	0
KRTCAP3	0.5654995	0.637159341	-0.0716598	-0.18441988	0.000136151	0.000807273	12	0	0
GPATCH4	0.1450386	0.196001467	-0.0509629	-0.18784151	0.000137285	0.000813462	7	0	0
TEX36	0.648491	0.710648512	-0.06215753	-0.13736727	0.000137242	0.000813462	6	0	0
IYD	0.6220766	0.709366914	-0.08729028	-0.18317834	0.000137388	0.000813801	6	0	0
MSR1	0.6213922	0.563683882	0.057708287	0.131203018	0.000137621	0.00081491	2	0	0
KHNYN	0.6852045	0.712468718	-0.02726425	-0.08007819	0.000138604	0.00082046	6	0	0
NR5A2	0.7809103	0.842608013	-0.06169771	-0.11028777	0.000139022	0.000822662	4	0	0
EVA1B	0.4302951	0.405211127	0.02508402	0.244797739	0.000139374	0.00082393	8	0	0
NAT2	0.6869767	0.699195383	-0.01221865	0.005112893	0.00013932	0.00082393	3	0	0
COX6C	0.3542767	0.408241749	-0.05396506	-0.1935696	0.000139345	0.00082393	9	0	0
ADAM20	0.6522345	0.724566979	-0.07233243	-0.15661637	0.000139499	0.000824403	2	0	0
C2orf69	0.19068	0.205000785	-0.01432076	-0.1722729	0.000139609	0.00082478	7	0	0
TMPRSS11A	0.7266128	0.778786449	-0.0521737	-0.09217572	0.00013978	0.000825519	3	0	0
CCSER2	0.2168852	0.246416069	-0.02953085	-0.03075751	0.00013994	0.000826123	10	0	0
MARS	0.5785977	0.513633147	0.064964565	0.186013256	0.000139975	0.000826123	3	0	0
TPPP2	0.4775795	0.429241742	0.048337751	0.257664172	0.000140345	0.000828035	12	0	0
AFP	0.6871431	0.717441214	-0.03029812	-0.06217251	0.00014072	0.000829975	3	0	0
TDRD6	0.8502939	0.889944058	-0.03965013	-0.07179277	0.000140808	0.000830219	16	0	0
ARID5A	0.369349	0.407126591	-0.0377776	-0.0751693	0.000141331	0.000833028	10	0	0
PHACTR1	0.5865178	0.569334251	0.017183596	0.09327767	0.00014163	0.000834246	11	0	0
CPT2	0.3639061	0.31911568	0.044790375	0.252102684	0.000142516	0.000839185	8	0	0
SNCA	0.3287764	0.395401879	-0.06662545	-0.14538984	0.0001432	0.000842937	7	0	0
ZNF329	0.2103297	0.107806152	0.102523583	1.178979849	0.000143605	0.000845046	5	0	0
DNAJC2	0.2604941	0.316620509	-0.05612643	-0.13136797	0.000143732	0.000845517	14	0	0
LCN10	0.5812507	0.640201445	-0.05895071	-0.13815275	0.000143828	0.000845801	4	0	0
FUT6	0.7294542	0.792218976	-0.0627648	-0.1146699	0.000143917	0.000846049	6	0	0
VPREB3	0.4990841	0.608358485	-0.10927436	-0.27791997	0.000144073	0.000846689	3	0	0
CST8	0.7828187	0.835943241	-0.05312455	-0.09590898	0.000144656	0.000849837	5	0	0
VSX2	0.3592375	0.318050788	0.041186683	0.36582043	0.000144817	0.000850416	6	0	0
AEBP1	0.4173555	0.406635853	0.010719627	0.448931675	0.000145117	0.000851709	7	0	0
FGF1	0.5782217	0.582841858	-0.0046202	0.042808286	0.000145374	0.000852656	5	0	0
APBB1IP	0.1514361	0.163253821	-0.01181768	0.119102638	0.000145351	0.000852656	12	0	0
TNS3	0.3922632	0.422164627	-0.02990146	-0.14391192	0.00014568	0.000853895	13	0	0
SAAL1	0.4495256	0.522769802	-0.07324416	-0.13322948	0.000145646	0.000853895	5	0	0
C8A	0.7136681	0.783947397	-0.07027928	-0.13549407	0.000145853	0.000854544	6	0	0
ZNF276	0.1978127	0.264693772	-0.06688105	-0.19227734	0.00014594	0.000854544	8	0	0
ZIM2	0.5535468	0.514574715	0.0389721	0.101913837	0.000145981	0.000854544	22	0	0
PEG3	0.5535468	0.514574715	0.0389721	0.101913837	0.000145981	0.000854544	22	0	0

POTEF	0.4502374	0.588361125	-0.13812373	-0.37863825	0.000146657	0.000858217	1	0	0
THPO	0.7026463	0.757557822	-0.05491152	-0.10560621	0.000146815	0.000858862	8	0	0
IFNK	0.8346455	0.864822777	-0.03017732	-0.05168138	0.000146926	0.000859231	3	0	0
FGB	0.6870151	0.75100036	-0.06398523	-0.13705516	0.000147055	0.000859706	4	0	0
PAQR8	0.4218521	0.457710665	-0.0358586	0.016476376	0.00014758	0.000862493	5	0	0
SLCO2A1	0.7131709	0.64650026	0.066670687	0.13957769	0.000147695	0.000862882	2	0	0
GPR55	0.7923902	0.837813866	-0.04542371	-0.08037474	0.000148535	0.000867466	4	0	0
OR8I2	0.680631	0.77021605	-0.08958504	-0.17595857	0.000148576	0.000867466	1	0	0
DRD3	0.6689145	0.754310759	-0.08539622	-0.17092936	0.000148904	0.0008691	1	0	0
OR4X1	0.3329651	0.405986799	-0.07302171	-0.28409052	0.000149565	0.000872672	4	0	0
TUSC3	0.7963216	0.84268029	-0.04635871	-0.08202252	0.000149697	0.000873161	3	0	0
PRDM16	0.1558601	0.106493426	0.049366703	0.527311667	0.00015029	0.00087605	15	0	0
FURIN	0.6910717	0.768227502	-0.07715579	-0.16103623	0.000150284	0.00087605	5	0	0
SLFN14	0.814241	0.870312512	-0.05607152	-0.09806188	0.000150649	0.000877858	3	0	0
CBLN1	0.2016045	0.104276812	0.097327645	0.947720326	0.000151104	0.000880044	15	0	0
TIAF1	0.801291	0.8571893	-0.05589829	-0.10962576	0.000151123	0.000880044	8	0	0
HIST1H4I	0.447264	0.368322033	0.078941924	0.670323474	0.000151449	0.000881657	8	0	0
TSPAN13	0.0577252	0.068489701	-0.01076455	-0.16133422	0.000151806	0.00088345	7	0	0
DUSP2	0.2576479	0.298624577	-0.04097665	0.111067718	0.000152187	0.000885381	9	0	0
LRRC71	0.4665883	0.537191166	-0.07060282	-0.13638148	0.000152533	0.000887105	8	0	0
MYO7B	0.5574458	0.625926424	-0.06848067	-0.16593039	0.000152905	0.000888691	5	0	0
FAM189A1	0.1687911	0.100596448	0.068194643	0.712198065	0.000152887	0.000888691	7	0	0
HIST1H1B	0.3563531	0.320458324	0.035894804	0.749991374	0.000153453	0.000891588	9	0	0
CTSZ	0.4643989	0.519214594	-0.05481573	-0.07884285	0.000153787	0.000893238	25	0	0
PHACTR3	0.2292651	0.172864781	0.056400281	0.786536464	0.000154106	0.000894515	5	0	0
ZCCHC10	0.1357262	0.120621294	0.015104862	0.144260878	0.00015462	0.000897206	8	0	0
KCNK9	0.1431189	0.072245993	0.070872905	0.989618827	0.00015553	0.000902195	12	0	0
ATP1A4	0.8044575	0.845366244	-0.04090878	-0.08010448	0.000155761	0.000903245	7	0	0
SLC6A19	0.5647648	0.633934472	-0.06916964	-0.16030074	0.000156039	0.000904562	10	0	0
CHID1	0.6950019	0.66847164	0.026530239	0.062641764	0.000156285	0.000905694	8	0	0
FOSL1	0.2595168	0.301241292	-0.04172446	-0.15673611	0.000156505	0.000906676	8	0	0
ARHGEF39	0.3503577	0.282613456	0.067744273	0.524104686	0.000156621	0.000907057	9	0	0
GSPT1	0.3840161	0.466876112	-0.08286003	-0.20723309	0.000156685	0.000907133	9	0	0
COL25A1	0.1596299	0.113230875	0.046398984	0.677339348	0.000157046	0.00090893	16	0	0
CACNA1A	0.2331881	0.290186699	-0.05699863	-0.32538016	0.000157374	0.000910536	3	0	0
KCNH7	0.2334517	0.155292953	0.078158788	0.584924041	0.000158485	0.000916665	9	0	0
IKZF3	0.178645	0.192742757	-0.01409774	-0.16820603	0.000159648	0.000922501	7	0	0
CSHL1	0.6874129	0.788172956	-0.10076003	-0.19468829	0.00015961	0.000922501	1	0	0
GH1	0.6874129	0.788172956	-0.10076003	-0.19468829	0.00015961	0.000922501	1	0	0
IFI30	0.3217212	0.366985089	-0.04526388	-0.21371224	0.000159763	0.000922869	8	0	0
CCDC33	0.6627422	0.725983641	-0.06324148	-0.12691888	0.000160549	0.000927112	3	0	0
VENTX	0.1658742	0.107737168	0.05813707	0.775284072	0.000160635	0.000927306	30	0	0
BNIPL	0.7547959	0.7682096	-0.01341366	-0.03340829	0.000160696	0.000927361	4	0	0
SPACA3	0.5609102	0.658107947	-0.09719773	-0.22681733	0.000160815	0.000927753	1	0	0
EHF	0.4907043	0.590807922	-0.10010361	-0.26473694	0.0001609	0.000927944	4	0	0
C12orf74	0.6816122	0.786115513	-0.10450327	-0.2231821	0.000161501	0.000931112	6	0	0
CPB2	0.7266643	0.797273921	-0.07060964	-0.13380205	0.000161789	0.000932468	4	0	0
ADORA1	0.5942009	0.649861515	-0.05566065	-0.13409277	0.000161896	0.000932789	3	0	0
SERTM1	0.5127778	0.485088695	0.027689122	0.364431155	0.000161973	0.000932929	8	0	0
DSCAML1	0.2526301	0.287027695	-0.03439758	-0.22041432	0.000162393	0.000935052	6	0	0
TDRD5	0.4595654	0.460891307	-0.00132586	0.273966613	0.00016251	0.000935351	10	0	0
SH2D4B	0.6773431	0.712166315	-0.0348232	-0.06527643	0.00016255	0.000935351	8	0	0
TGM6	0.6044421	0.683366983	-0.07892487	-0.14716988	0.000162621	0.000935462	8	0	0
ATP1A1	0.1167877	0.151269994	-0.03448231	-0.17276015	0.00016269	0.000935561	8	0	0
CLDN22	0.6590445	0.746378768	-0.08733424	-0.1790252	0.000163972	0.000942628	3	0	0
CSN3	0.7006592	0.750346583	-0.04968733	-0.08750026	0.000164515	0.00094545	2	0	0
C3orf30	0.7178623	0.758454497	-0.04059224	-0.08898869	0.000164656	0.000945954	11	0	0
VWA1	0.2359972	0.169834469	0.066162766	0.491516324	0.000165102	0.000948187	10	0	0
OR4M1	0.8105544	0.865827664	-0.05527328	-0.0946749	0.00016515	0.000948187	4	0	0
ZNF445	0.2940213	0.331440049	-0.03741875	-0.09979921	0.000165534	0.00094978	9	0	0
FAM184B	0.2418837	0.179687264	0.062196461	0.730691151	0.000165504	0.00094978	11	0	0
KCNIP3	0.3990905	0.44133604	-0.04224549	-0.00206924	0.000165869	0.000951397	9	0	0
REPIN1	0.1444206	0.150027737	-0.00560712	-0.1541662	0.000166253	0.000953299	8	0	0

SELPLG	0.4170924	0.492342038	-0.07524964	-0.23638516	0.000166736	0.000955763	6	0	0
KBTBD2	0.8696397	0.874462706	-0.00482297	-0.00928894	0.000166929	0.00095656	3	0	0
RNF222	0.6801353	0.744972147	-0.06483685	-0.13403549	0.000167333	0.000958568	4	0	0
CCAR1	0.3368167	0.364975172	-0.02815843	0.011342375	0.00016765	0.000960078	10	0	0
ZIC2	0.0868179	0.082350921	0.004466982	0.15641332	0.000168058	0.000962112	8	0	0
WDR87	0.0737615	0.098404104	-0.02464257	-0.17009077	0.000168344	0.000963441	11	0	0
OR6M1	0.5488364	0.614534843	-0.06569844	-0.15631522	0.000168457	0.000963776	7	0	0
A4GNT	0.684961	0.772891688	-0.0879307	-0.17614603	0.000168794	0.000965397	4	0	0
TNFSF15	0.3397795	0.388208646	-0.04842914	-0.29680744	0.000168973	0.000966056	5	0	0
EML2	0.0785023	0.030535401	0.047966941	1.087512347	0.000169017	0.000966056	5	0	0
MTHFS	0.2651387	0.316271636	-0.05113298	-0.14398195	0.000169206	0.000966828	5	0	0
EID3	0.2854567	0.207203192	0.078253502	0.882542222	0.000169554	0.000968511	13	0	0
SDS	0.3416353	0.417089414	-0.07545412	-0.29481849	0.000169971	0.000970582	5	0	0
C1orf116	0.590135	0.67321732	-0.0830823	-0.19102306	0.000170491	0.000973241	4	0	0
PHLDB3	0.3707584	0.353609954	0.017148481	0.141849727	0.000170599	0.000973549	12	0	0
TMEM158	0.2950836	0.261080321	0.034003231	0.200748526	0.000170713	0.000973582	8	0	0
HOXA3	0.396845	0.277824279	0.119020714	0.662264224	0.00017068	0.000973582	10	0	0
GRB14	0.5037419	0.542252183	-0.03851032	-0.15257021	0.00017126	0.000976081	6	0	0
CACNB2	0.1791767	0.122109857	0.057066858	0.816548102	0.000171249	0.000976081	11	0	0
INPP5B	0.405083	0.314681338	0.090401661	0.555527881	0.00017161	0.00097763	9	0	0
KITLG	0.2309504	0.239937429	-0.00898707	0.065977175	0.000171641	0.00097763	13	0	0
TIFAB	0.6418038	0.70787276	-0.06606893	-0.1709963	0.000171785	0.000978138	7	0	0
CEP135	0.0786876	0.105713168	-0.02702553	-0.18785459	0.000172442	0.000981258	13	0	0
SLC37A3	0.1574726	0.198527414	-0.04105483	-0.23943182	0.000172417	0.000981258	4	0	0
SLC22A16	0.4213841	0.381147965	0.040236114	0.751498518	0.000172503	0.000981294	5	0	0
MATN2	0.1520284	0.158664925	-0.0066365	0.071103255	0.0001727	0.000981957	11	0	0
RELB	0.332239	0.354786353	-0.02254734	-0.1986213	0.000172729	0.000981957	10	0	0
SPI1	0.5781852	0.643604357	-0.06541911	-0.15643541	0.000173109	0.000983801	15	0	0
ST6GALNAC	0.2333598	0.177220446	0.056139382	0.789551524	0.000173204	0.000984013	10	0	0
HIGD1C	0.7457414	0.816973643	-0.07123228	-0.1405135	0.000173256	0.000984013	5	0	0
CCDC117	0.3482748	0.414381193	-0.06610635	-0.14763282	0.000173626	0.000985802	10	0	0
NKIRAS1	0.4906398	0.453746796	0.036892966	0.104710451	0.000173831	0.000986659	4	0	0
STK19	0.6534537	0.721760471	-0.06830673	-0.15295663	0.000173918	0.000986835	33	0	0
KDELR1	0.1787358	0.202166618	-0.02343086	-0.13676307	0.000173976	0.000986855	11	0	0
VWC2	0.1508573	0.073377605	0.077479735	1.100886047	0.000174372	0.000988786	8	0	0
MEIOB	0.5596546	0.630331903	-0.0706773	-0.16519654	0.000174976	0.000991585	3	0	0
KRT10	0.7296103	0.797515999	-0.06790565	-0.11723561	0.000174935	0.000991585	2	0	0
LY6D	0.5757106	0.639881177	-0.06417059	-0.14181033	0.000175067	0.000991786	13	0	0
RARB	0.688556	0.737033323	-0.04847735	-0.1228611	0.000175219	0.000992335	5	0	0
SPC24	0.0971667	0.132995643	-0.03582895	-0.19312461	0.000175655	0.00099449	8	0	0
SCNN1B	0.7161311	0.693505701	0.022625382	0.053773872	0.00017607	0.000996526	3	0	0
OR2K2	0.8484583	0.906406122	-0.05794786	-0.09423911	0.000176185	0.000996865	1	0	0
ANKEF1	0.3518411	0.402876487	-0.05103539	-0.14508847	0.000176382	0.000997665	3	0	0
KIAA1257	0.4888169	0.501197595	-0.01238067	-0.02549934	0.000176554	0.000998008	7	0	0
AMER2	0.4185604	0.379098864	0.0394615	0.339755176	0.000176518	0.000998008	15	0	0
GUCY2D	0.2929272	0.25136517	0.041562021	0.50560003	0.0001768	0.000999084	7	0	0
SETD4	0.8272307	0.880849912	-0.05361918	-0.08983107	0.000177556	0.001003035	4	0	0
PLCB1	0.2125085	0.181614868	0.030893665	0.847126433	0.000178456	0.001007803	12	0	0
KIAA2013	0.1632529	0.188583244	-0.02533032	-0.02648559	0.000178846	0.001009686	4	0	0
TRAF3IP3	0.6735399	0.74948691	-0.07594706	-0.16892935	0.000179013	0.001009998	2	0	0
C11orf42	0.876756	0.90733376	-0.03057776	-0.04957028	0.000179	0.001009998	5	0	0
NCR2	0.6489083	0.681380801	-0.03247253	-0.06809733	0.000179348	0.001011566	7	0	0
PLOD2	0.8645581	0.908589564	-0.04403145	-0.07086613	0.000179788	0.001013732	1	0	0
UCMA	0.5937796	0.645229291	-0.05144971	-0.09935963	0.000181117	0.001020906	7	0	0
BPI	0.4304382	0.427436015	0.003002225	0.269046798	0.000181465	0.001022543	5	0	0
CH25H	0.13571	0.067301556	0.068408415	1.081308733	0.000182025	0.001025374	14	0	0
LZTR1	0.6785094	0.731391329	-0.05288192	-0.10675887	0.000182604	0.001028315	1	0	0
SEC62	0.1333933	0.171548627	-0.03815531	-0.19310834	0.000182939	0.001029877	12	0	0
SMC6	0.6105703	0.531509819	0.079060516	0.191328403	0.000183072	0.001030303	2	0	0
MYO1C	0.5282886	0.476748541	0.051540022	0.141117857	0.000183215	0.001030786	8	0	0
CSF1	0.2900958	0.257602912	0.032492903	0.105695747	0.000184005	0.001034906	9	0	0
GABRA4	0.5093082	0.564105896	-0.05479771	-0.03439777	0.000184328	0.001036073	7	0	0
PCDH10	0.1380009	0.079198977	0.058801924	0.812114122	0.000184315	0.001036073	11	0	0

GLIS1	0.730521	0.790782302	-0.06026133	-0.11721872	0.000184756	0.001038151	7	0	0
NSFL1C	0.7245144	0.804263193	-0.07974881	-0.15595857	0.000185102	0.001039771	3	0	0
GCG	0.6368566	0.712422008	-0.07556544	-0.15398891	0.000185565	0.001042044	7	0	0
QRFPR	0.3051115	0.249061846	0.056049607	0.801746867	0.0001858	0.00104304	15	0	0
CCNO	0.0967405	0.097619912	-0.00087943	-0.15627078	0.000186009	0.001043559	11	0	0
MYH6	0.5582894	0.606475316	-0.04818592	-0.1048168	0.000185968	0.001043559	7	0	0
CAMP	0.6843207	0.748699813	-0.06437908	-0.15251895	0.000186263	0.001044006	7	0	0
POLR3A	0.2069734	0.230750893	-0.02377751	-0.03351863	0.000186214	0.001044006	9	0	0
KCTD12	0.2634792	0.196684883	0.066794309	0.258100686	0.000186201	0.001044006	11	0	0
KRTAP9-1	0.8180407	0.877591248	-0.05955051	-0.10316895	0.000186566	0.001045376	8	0	0
TNFRSF12A	0.3499378	0.273667677	0.076270148	0.517143262	0.000187287	0.001049088	10	0	0
NPTX1	0.1720673	0.104712076	0.067355187	0.91684419	0.000187407	0.001049433	5	0	0
RIMS4	0.1985816	0.122310058	0.076271492	1.110409787	0.000187556	0.001049939	8	0	0
SMIM6	0.5710716	0.535122697	0.035948937	0.113823139	0.000187681	0.001050313	8	0	0
GABRB2	0.1757465	0.120286725	0.055459793	0.517040761	0.000189039	0.001057475	8	0	0
BATF2	0.499932	0.503412743	-0.00348076	0.146114662	0.000189079	0.001057475	4	0	0
WNT16	0.7315233	0.694516346	0.037006915	0.111703839	0.000189779	0.001061061	5	0	0
NOTO	0.3191031	0.272059622	0.047043437	0.547401065	0.00019007	0.001062357	7	0	0
LGALS16	0.4111626	0.50507127	-0.09390866	-0.2903947	0.000190288	0.00106324	1	0	0
ADAD1	0.7959483	0.825563842	-0.02961554	-0.05251783	0.000190358	0.001063301	16	0	0
C16orf91	0.6317009	0.670032325	-0.0383314	-0.10280531	0.000190418	0.001063303	6	0	0
UGT1A9	0.8792002	0.871596543	0.007603631	0.013699168	0.000190709	0.001064327	3	0	0
DYDC2	0.7377338	0.810159623	-0.0724258	-0.14395411	0.00019072	0.001064327	5	0	0
SH3D19	0.0649946	0.035083887	0.02991067	0.745179012	0.000190793	0.001064403	2	0	0
DNAH2	0.1056997	0.103300589	0.002399091	-0.00977939	0.000191139	0.001066002	8	0	0
SSBP3	0.6731522	0.650931289	0.022220937	0.088937392	0.000191264	0.001066368	2	0	0
SPATA2L	0.1478229	0.165496299	-0.01767336	-0.14108457	0.000191531	0.001067526	6	0	0
CD163	0.5741194	0.656004094	-0.08188472	-0.21756118	0.000192307	0.001071521	3	0	0
XCL1	0.8006299	0.848112086	-0.0474822	-0.08212274	0.000192399	0.001071698	1	0	0
MYBPH	0.6376914	0.685924282	-0.04823284	-0.09677225	0.000192733	0.001072561	3	0	0
DCHS2	0.2584978	0.205381453	0.053116343	0.300274543	0.000192636	0.001072561	6	0	0
STAU2	0.2200503	0.259853412	-0.03980308	-0.24844101	0.000192698	0.001072561	11	0	0
SNURF	0.6346476	0.677312915	-0.04266535	-0.07995309	0.000192836	0.0010728	12	0	0
C18orf54	0.2847736	0.339518132	-0.0547445	-0.14407287	0.000193157	0.001074251	5	0	0
SSTR3	0.5938835	0.573735265	0.020148273	0.069931502	0.000193377	0.001075141	7	0	0
SLC2A2	0.6347162	0.680941958	-0.04622577	-0.07217513	0.000194096	0.001078807	5	0	0
ALAS1	0.3115976	0.292682245	0.018915378	0.026103089	0.00019434	0.001079827	9	0	0
AIRE	0.5834178	0.580775128	0.002642655	0.038423294	0.000194549	0.001080656	13	0	0
CD300C	0.7025112	0.766659877	-0.0641487	-0.12910815	0.000194746	0.001081411	6	0	0
KIAA1217	0.1489577	0.087755778	0.061201942	0.739290723	0.000194899	0.001081929	10	0	0
TRAM1L1	0.3367259	0.336234927	0.000490943	0.332762271	0.000195117	0.001082805	11	0	0
CLIC6	0.4898372	0.419319784	0.070517381	0.47898374	0.000195206	0.001082963	8	0	0
OR1G1	0.5080757	0.565875774	-0.0578001	-0.14046082	0.000195523	0.001084383	5	0	0
CDC14A	0.7003219	0.651779901	0.048541985	0.118837095	0.000195789	0.001085522	3	0	0
ZNF662	0.5366757	0.506719594	0.029956137	0.443641108	0.000195959	0.001086132	9	0	0
OR2L13	0.530562	0.530871689	-0.00030971	0.132297959	0.000196262	0.001087398	11	0	0
GABRR3	0.6606395	0.740568067	-0.07992853	-0.16925981	0.000196369	0.001087398	5	0	0
ZBTB16	0.2649586	0.243343262	0.021615299	0.490642458	0.000196319	0.001087398	9	0	0
CRK	0.7137061	0.767974635	-0.05426851	-0.12824265	0.000196474	0.001087643	4	0	0
SPC25	0.5470184	0.595751268	-0.04873289	-0.11717035	0.000196718	0.001088498	6	0	0
GNAT1	0.5661974	0.619502414	-0.05330501	-0.1276661	0.00019682	0.001088498	5	0	0
GML	0.7041172	0.785319769	-0.08120259	-0.15779856	0.0001968	0.001088498	6	0	0
YEATS4	0.3604652	0.442989016	-0.08252385	-0.2641683	0.000196871	0.001088498	6	0	0
SLN	0.5681245	0.650192632	-0.08206815	-0.17626091	0.00019706	0.001089206	4	0	0
SLC46A2	0.3472931	0.28480946	0.062483643	0.562719566	0.000197318	0.001090293	10	0	0
OR2V2	0.8475991	0.894052708	-0.04645356	-0.0763118	0.000197419	0.001090519	5	0	0
OR4K15	0.5809549	0.693325026	-0.1123701	-0.25114345	0.000197494	0.001090594	1	0	0
UBASH3B	0.2134991	0.247125342	-0.03362627	-0.17337232	0.000198443	0.001095161	10	0	0
MIF	0.2127318	0.249873102	-0.03714131	-0.15524074	0.00019842	0.001095161	8	0	0
ADRA2C	0.4035323	0.37465232	0.028879973	0.438098184	0.000198696	0.001096221	5	0	0
KCNAB1	0.5544647	0.66241693	-0.10795222	-0.25247267	0.000199319	0.001098699	1	0	0
AGR2	0.8147937	0.867885097	-0.05309138	-0.09158891	0.000199302	0.001098699	3	0	0
SHMT2	0.4707211	0.490992359	-0.02027128	-0.20816276	0.000199329	0.001098699	4	0	0

GATA5	0.1637536	0.085427595	0.078325997	1.080429563	0.00019954	0.001099525	23	0	0
SIGLEC11	0.5504948	0.638086024	-0.08759117	-0.21228241	0.000199735	0.001100259	2	0	0
ZC3H12D	0.2985317	0.367460526	-0.06892881	-0.30237706	0.000199977	0.001101256	10	0	0
KCNS2	0.2501607	0.246021054	0.004139649	0.38289572	0.000200223	0.001102273	9	0	0
MT1HL1	0.6432136	0.726770452	-0.08355684	-0.186204	0.000200679	0.001104443	7	0	0
MELK	0.1704664	0.199001852	-0.02853546	-0.04721402	0.000201179	0.001106853	10	0	0
ADAM12	0.3286526	0.352775951	-0.02412331	-0.04422727	0.000201381	0.001107625	13	0	0
RGS21	0.7390245	0.805153015	-0.06612854	-0.12422038	0.000201519	0.001108047	2	0	0
CTXN2	0.1998156	0.169868045	0.029947507	0.323499447	0.000201737	0.001108907	15	0	0
SIGLEC1	0.7379189	0.763870178	-0.02595126	-0.05425188	0.000201975	0.001109873	6	0	0
TMEM196	0.2501846	0.185411003	0.064773566	0.552123524	0.000202041	0.001109897	11	0	0
TOP1MT	0.7749252	0.82067893	-0.04575375	-0.08828754	0.000202372	0.001111375	5	0	0
MCHR1	0.7197045	0.715857811	0.003846683	0.013748343	0.000202645	0.00111253	8	0	0
CA3	0.885046	0.926090476	-0.04104452	-0.06468623	0.000202932	0.001113426	1	0	0
ADAMTSL2	0.6183431	0.659730233	-0.04138718	-0.08139796	0.000202924	0.001113426	2	0	0
STX1B	0.2827484	0.222546173	0.060202271	0.580465273	0.000203021	0.001113575	8	0	0
C8orf86	0.5605037	0.514029855	0.04647386	0.186432364	0.000203375	0.001115175	8	0	0
ASPG	0.3121997	0.245532369	0.066667295	0.364229166	0.000203521	0.001115634	7	0	0
SCGB1D4	0.519558	0.598529577	-0.07897157	-0.20283231	0.000203655	0.001116026	9	0	0
CCDC60	0.4372666	0.516939254	-0.07967264	-0.20155669	0.000203853	0.001116768	11	0	0
CCDC150	0.2336568	0.285963919	-0.05230717	-0.17873639	0.000204059	0.001117323	12	0	0
MESP1	0.3254953	0.369202778	-0.04370749	-0.13780068	0.000204078	0.001117323	8	0	0
PHKG1	0.3907554	0.315597868	0.075157538	0.285656069	0.00020473	0.001120548	9	0	0
TRABD	0.3405728	0.257311903	0.083260944	0.81013731	0.000204798	0.00112058	5	0	0
PRR15	0.0584055	0.071970559	-0.0135651	-0.2122991	0.000205194	0.001122401	9	0	0
CES4A	0.6861007	0.736235949	-0.05013526	-0.10724491	0.000205375	0.00112305	10	0	0
TMEM74	0.5404507	0.60694087	-0.06649019	-0.084174	0.00020624	0.001127336	6	0	0
FOXA2	0.2669855	0.199319416	0.067666128	0.436034776	0.000206284	0.001127336	11	0	0
CNN1	0.3563698	0.32824719	0.028122594	0.30493901	0.000206779	0.001129692	7	0	0
FBXL2	0.1405237	0.167455074	-0.02693135	-0.19757923	0.000207203	0.00113161	3	0	0
PICK1	0.7449392	0.795490042	-0.05055088	-0.10348023	0.000207256	0.00113161	2	0	0
CEP97	0.3865062	0.476677795	-0.09017161	-0.22040936	0.000208298	0.001136956	5	0	0
GPR35	0.7320995	0.782584271	-0.05048475	-0.09524131	0.000208677	0.001138676	3	0	0
KRTAP19-1	0.5650271	0.638993417	-0.07396628	-0.16916861	0.00020883	0.001139166	3	0	0
HRCT1	0.3980178	0.350242267	0.047775522	0.2251009	0.000209264	0.001141183	6	0	0
MYLK2	0.6500859	0.675004797	-0.02491894	-0.04192643	0.000209451	0.001141856	11	0	0
CRYBA2	0.1534555	0.078075409	0.075380054	1.122491253	0.000209851	0.001143344	12	0	0
TAAR2	0.8169467	0.866992476	-0.05004579	-0.0884517	0.000209844	0.001143344	6	0	0
HIST1H3C	0.1562334	0.053431024	0.102802409	1.461079945	0.000209923	0.00114339	7	0	0
ZNF837	0.3755809	0.416914237	-0.04133332	-0.06939972	0.000210066	0.001143819	6	0	0
TRIL	0.2260592	0.189179593	0.036879582	0.685115625	0.000210429	0.001145449	14	0	0
KIAA1841	0.2443287	0.3069928	-0.06266413	-0.20556261	0.000210901	0.001147669	5	0	0
DRC1	0.4616798	0.436201274	0.025478551	0.29368858	0.000211931	0.001152614	11	0	0
CORO1B	0.1922359	0.216559806	-0.02432388	-0.13124112	0.000211938	0.001152614	15	0	0
ZNF233	0.8496995	0.877228845	-0.02752932	-0.04622486	0.000212022	0.001152719	3	0	0
COX5A	0.2642143	0.272074195	-0.0078599	0.124808435	0.000212358	0.001154197	11	0	0
CD164L2	0.4175585	0.470244639	-0.05268612	-0.15907004	0.000212659	0.001155132	12	0	0
RSPH6A	0.4930688	0.416425832	0.076642956	0.251917806	0.000212623	0.001155132	11	0	0
UFC1	0.7851989	0.86755454	-0.08235566	-0.14311668	0.000212911	0.001156154	2	0	0
IHH	0.1263559	0.114624055	0.011731838	0.619687987	0.000213292	0.00115787	12	0	0
TTLL10	0.5893154	0.521062144	0.068253237	0.181249401	0.000213595	0.001159162	3	0	0
CRIP3	0.306882	0.24792271	0.058959288	1.025100344	0.000213809	0.001159973	9	0	0
VIPR2	0.4041275	0.332555754	0.071571758	0.706500046	0.000214161	0.001161532	4	0	0
PEX19	0.5836672	0.692704519	-0.10903732	-0.24126987	0.000214577	0.00116344	5	0	0
EPOR	0.537577	0.494607816	0.042969221	0.209252698	0.000214913	0.001164908	6	0	0
ITIH3	0.7328878	0.706379968	0.02650788	0.057686497	0.000215274	0.001166513	8	0	0
CPNE5	0.1686204	0.114800505	0.053819868	0.486809424	0.000215833	0.00116919	7	0	0
ZDHHC22	0.4134092	0.372583191	0.040826	0.259838347	0.000216068	0.001170106	5	0	0
CHI3L1	0.3262002	0.370233137	-0.04403294	-0.20746826	0.00021671	0.001173231	4	0	0
METTL6	0.5998631	0.668017138	-0.068154	-0.21133841	0.000217675	0.001178098	5	0	0
MDGA1	0.0827276	0.101789029	-0.01906147	-0.26103251	0.000218057	0.001179672	3	0	0
C7orf26	0.4480347	0.407013189	0.041021502	0.088476256	0.000218097	0.001179672	8	0	0
SPINK13	0.6988948	0.753262298	-0.05436751	-0.10890222	0.000218319	0.001180161	4	0	0

NCF4	0.4403462	0.50716757	-0.06682139	-0.16389342	0.000218274	0.001180161	5	0	0
DACT1	0.7784124	0.809671642	-0.03125927	-0.05533707	0.000218874	0.001182804	2	0	0
SLC7A9	0.7076622	0.672368904	0.035293259	0.072559602	0.000218966	0.001182943	3	0	0
C11orf88	0.2959309	0.340021686	-0.04409082	-0.13862268	0.000220224	0.001189384	9	0	0
GALNT13	0.2611846	0.207587687	0.053596924	0.696647687	0.000220543	0.001190744	9	0	0
ANKLE1	0.395819	0.398866372	-0.00304739	0.554703978	0.000221165	0.001193746	12	0	0
ZCCHC7	0.8020465	0.845523365	-0.04347683	-0.0701992	0.000221624	0.001195504	2	0	0
CCDC183	0.5241724	0.545956133	-0.02178371	-0.0226759	0.000221617	0.001195504	4	0	0
RPL39L	0.8662285	0.912310709	-0.04608225	-0.07494909	0.000221853	0.001196262	3	0	0
SMG8	0.7631416	0.811688515	-0.04854693	-0.08947823	0.000221898	0.001196262	2	0	0
PCDHAC1	0.204706	0.142967344	0.061738639	0.740780173	0.000222074	0.00119649	13	0	0
SLC46A1	0.8350439	0.89398779	-0.05894386	-0.09727695	0.000222009	0.00119649	1	0	0
DHRS2	0.6725884	0.747847088	-0.07525866	-0.15089083	0.000222234	0.001196995	1	0	0
MYEOV	0.665437	0.660104701	0.005332291	0.014158661	0.000222565	0.001198416	5	0	0
ITPRIPL1	0.237461	0.171456396	0.066004573	0.836010181	0.000222904	0.001199163	11	0	0
SERPINA10	0.5511156	0.630903357	-0.07978772	-0.19537174	0.000222798	0.001199163	11	0	0
OR10H3	0.6763956	0.758919845	-0.08252425	-0.17390299	0.000222904	0.001199163	3	0	0
RIN1	0.2844067	0.336970635	-0.05256398	-0.15638065	0.000222985	0.001199239	11	0	0
ABCB5	0.6854181	0.75341416	-0.06799603	-0.14743649	0.00022396	0.001204123	5	0	0
AGAP1	0.291051	0.258443879	0.032607146	0.354187281	0.000224064	0.001204321	9	0	0
TMEM33	0.2545884	0.317138242	-0.06254982	-0.13305265	0.000224437	0.001205965	9	0	0
KIF12	0.4151181	0.330948471	0.084169651	0.334324934	0.000224641	0.0012067	5	0	0
ANKRD1	0.719717	0.78058345	-0.06086646	-0.12180453	0.000225177	0.001209213	5	0	0
TXN	0.1387317	0.193645712	-0.05491403	-0.16409267	0.000225759	0.001211976	3	0	0
C9orf135	0.444888	0.452667707	-0.00777972	0.0263099	0.000225843	0.001212067	11	0	0
SYT3	0.1910148	0.159884764	0.031130041	0.395125509	0.000226571	0.001215607	6	0	0
FAM124B	0.4084632	0.326329734	0.082133473	0.470098768	0.000226958	0.00121732	10	0	0
ZMYND15	0.0714689	0.101075647	-0.02960677	-0.15528116	0.000227566	0.001220214	10	0	0
ILDR1	0.4802177	0.515686076	-0.03546835	-0.10961668	0.000228105	0.001222741	7	0	0
NPHS2	0.3647298	0.305606347	0.059123446	0.529030937	0.000228576	0.001224532	6	0	0
DUS1L	0.1729468	0.216516949	-0.04357017	-0.16307745	0.00022855	0.001224532	11	0	0
ATP4B	0.7373711	0.79486606	-0.05749494	-0.10904052	0.000228655	0.001224589	12	0	0
FAM129B	0.2860384	0.227752019	0.058286422	0.34408613	0.00022932	0.001227784	6	0	0
POU4F3	0.1746252	0.12157913	0.053046024	0.525934122	0.000229555	0.00122853	10	0	0
PHYHIPL	0.1706668	0.105587038	0.06507979	0.69281174	0.000229596	0.00122853	14	0	0
OPN4	0.8592424	0.892833399	-0.03359095	-0.05364136	0.000229705	0.001228749	3	0	0
RUNX2	0.6030782	0.690289613	-0.08721137	-0.18728558	0.000229926	0.001229562	5	0	0
FABP1	0.6616185	0.74192641	-0.08030793	-0.16572537	0.000230659	0.001233114	8	0	0
SCGB1A1	0.7190387	0.774006662	-0.05496798	-0.11798605	0.00023091	0.001234087	3	0	0
DEFB134	0.6312825	0.707799512	-0.07651703	-0.16548542	0.00023107	0.001234575	6	0	0
RCC1	0.1170522	0.111445633	0.005606534	0.115440674	0.000231437	0.001235063	11	0	0
CCDC125	0.2530003	0.283270762	-0.03027044	-0.18477092	0.000231394	0.001235063	10	0	0
BNIP3	0.2059373	0.250197264	-0.04425994	-0.07182491	0.000231307	0.001235063	11	0	0
MT1G	0.4621429	0.435816614	0.02632627	0.213751453	0.000231244	0.001235063	5	0	0
FCHO1	0.2235433	0.257908538	-0.03436528	-0.17569007	0.000232123	0.001238356	10	0	0
GPR149	0.5118031	0.576691818	-0.06488876	-0.10102693	0.000233151	0.001243102	4	0	0
PLCG2	0.5115928	0.579401278	-0.06780849	-0.1601666	0.000233142	0.001243102	11	0	0
SLC25A11	0.0639537	0.079400073	-0.01544636	-0.11072571	0.000233772	0.001246044	14	0	0
JPH4	0.5589426	0.537032768	0.02190979	0.100685006	0.00023527	0.001253653	5	0	0
CDH7	0.3211523	0.310115639	0.011036681	0.321348985	0.000235515	0.001254588	10	0	0
RPL4	0.6938057	0.752600337	-0.05879465	-0.14404384	0.000235855	0.001256025	3	0	0
LACRT	0.6773356	0.737514339	-0.06017878	-0.11656064	0.000236149	0.001257216	5	0	0
CRB2	0.6131388	0.564870581	0.048268188	0.136794229	0.000236598	0.001259233	4	0	0
R3HCC1L	0.3633762	0.426246795	-0.06287064	-0.1413065	0.000236953	0.001260752	7	0	0
PARP15	0.5261801	0.448082666	0.078097401	0.514687523	0.000237252	0.001261594	12	0	0
GINS4	0.2134032	0.256168252	-0.04276506	-0.18864156	0.00023725	0.001261594	4	0	0
OR7A17	0.8542541	0.894210744	-0.03995663	-0.06572096	0.000237806	0.001264162	5	0	0
SLC7A13	0.7622144	0.833913384	-0.07169903	-0.13674833	0.000238131	0.001265181	3	0	0
FAM45A	0.2900826	0.291428677	-0.00134603	0.12093684	0.000238138	0.001265181	7	0	0
DMTN	0.328542	0.283236901	0.045305112	0.170299263	0.000238341	0.001265884	4	0	0
ZNF676	0.4031584	0.503757286	-0.10059889	-0.31439197	0.000238687	0.001267343	1	0	0
NACA2	0.8298894	0.866542611	-0.03665323	-0.06227924	0.000238937	0.001268297	11	0	0
LEMD2	0.432772	0.461044261	-0.02827224	0.01259751	0.000239569	0.001271275	7	0	0

PINLYP	0.3097821	0.365327751	-0.05554567	-0.0863694	0.000240071	0.001273563	11	0	0
ASAH2	0.7607185	0.818782564	-0.05806402	-0.11099299	0.000240721	0.0012764	6	0	0
KRTAP5-9	0.6362767	0.68510648	-0.04882973	-0.12444804	0.000240748	0.0012764	9	0	0
P2RX7	0.3457344	0.294629898	0.051104517	0.37079013	0.000241771	0.001281446	10	0	0
WFDC11	0.4802041	0.603569072	-0.12336499	-0.32384326	0.000241887	0.001281678	1	0	0
CTHRC1	0.2095904	0.240583554	-0.03099313	0.053893876	0.000242813	0.001286201	8	0	0
PRKCG	0.1158581	0.065039921	0.050818178	0.748444877	0.000242884	0.001286201	3	0	0
APOL3	0.7097751	0.748369431	-0.03859435	-0.10123407	0.000243957	0.001291503	4	0	0
IER5	0.2006639	0.253883841	-0.05321992	-0.14723281	0.000245055	0.001296848	10	0	0
ANKRD62	0.789135	0.851542095	-0.06240711	-0.13202439	0.000245111	0.001296848	12	0	0
HOXC12	0.2091576	0.139137236	0.070020406	0.585896166	0.000246373	0.001303138	11	0	0
PTX3	0.6322038	0.688636138	-0.05643234	-0.08397774	0.00024739	0.001308133	5	0	0
SPIN1	0.0301538	0.036009535	-0.00585571	-0.10783525	0.000247751	0.001309654	6	0	0
ZFP2	0.4978632	0.51317221	-0.01530903	0.136493801	0.000248083	0.001311024	5	0	0
ZNF705A	0.8717097	0.915990888	-0.04428121	-0.0706943	0.000248479	0.00131273	1	0	0
GSX1	0.2163322	0.139537352	0.076794888	0.581142663	0.000248629	0.001313138	12	0	0
STAT5A	0.3168551	0.267354763	0.049500349	0.48671805	0.000249265	0.001316108	13	0	0
KRT15	0.5051716	0.577909423	-0.07273787	-0.19182669	0.000249798	0.001318535	8	0	0
TSPAN11	0.1871035	0.130985789	0.056117683	0.98216539	0.000250271	0.001320643	12	0	0
LTBP1	0.1095093	0.138351627	-0.02884235	-0.09208242	0.0002508	0.001323042	8	0	0
ABCA3	0.1427229	0.114397502	0.02832544	0.128145579	0.000251195	0.001324736	8	0	0
C1orf54	0.5277481	0.493458065	0.034290076	0.07281214	0.000251431	0.001325593	8	0	0
C2orf74	0.3579761	0.25875228	0.099223819	1.077857484	0.000251629	0.001326244	9	0	0
C22orf15	0.4695394	0.398193985	0.071345432	0.275512454	0.000251941	0.001327504	5	0	0
GBP3	0.4555918	0.495054611	-0.03946277	-0.21962401	0.000252458	0.001329837	2	0	0
KLHL26	0.0722228	0.073891609	-0.00166879	-0.03742559	0.000252674	0.001330581	10	0	0
CTSS	0.7376094	0.782708444	-0.04509904	-0.08478254	0.000252975	0.001331385	3	0	0
MTPAP	0.6937714	0.766023364	-0.072252	-0.13917273	0.000252971	0.001331385	2	0	0
PCDH15	0.2523918	0.199109135	0.053282665	0.52522672	0.000253207	0.001332217	3	0	0
AMOTL2	0.4765975	0.46909943	0.007498098	0.028801264	0.000253824	0.001335069	7	0	0
RAX2	0.6450963	0.694528545	-0.04943228	-0.09104213	0.000254118	0.001336224	8	0	0
USP21	0.2528412	0.30448613	-0.05164494	-0.15034138	0.000254661	0.001338689	12	0	0
HHATL	0.5865806	0.634466803	-0.0478862	-0.08833063	0.000255196	0.001341107	9	0	0
TMEM182	0.1261023	0.178379851	-0.05227755	-0.22122028	0.000255372	0.001341638	10	0	0
LDLRAD2	0.6600893	0.701063051	-0.04097374	-0.07646567	0.000256102	0.001345078	6	0	0
MAP2K2	0.2882209	0.258704172	0.029516736	0.09391487	0.000256235	0.001345383	6	0	0
SLC24A3	0.2784607	0.300227691	-0.02176695	0.297269155	0.000256479	0.001346271	3	0	0
TAAR1	0.8443162	0.87393837	-0.02962212	-0.04930194	0.000256912	0.001348148	2	0	0
RSPH9	0.5991005	0.557551645	0.041548874	0.187614131	0.000257984	0.001353381	8	0	0
RFPL4B	0.8237245	0.867938509	-0.04421399	-0.07966353	0.000258425	0.001355293	9	0	0
ZNF114	0.7984063	0.760448458	0.037957875	0.071133812	0.000259012	0.001357977	7	0	0
UBE2B	0.2037326	0.220107393	-0.01637481	0.038421576	0.000259238	0.001358369	9	0	0
ATP6V0A4	0.7391125	0.8147708	-0.07565827	-0.13881081	0.0002592	0.001358369	1	0	0
RTP3	0.6436365	0.622342212	0.021294257	0.021984966	0.000259763	0.001360722	6	0	0
C8orf34	0.3071581	0.274881297	0.032276789	0.624551096	0.00025987	0.001360884	8	0	0
MBOAT4	0.5683472	0.58280811	-0.01446087	-0.05423337	0.000260596	0.001364289	6	0	0
RNF113B	0.8904402	0.910863448	-0.02042325	-0.03282985	0.00026075	0.001364698	9	0	0
THNSL2	0.4530837	0.46065103	-0.00756737	0.30789002	0.00026112	0.001366233	6	0	0
SCRN1	0.3745572	0.416945691	-0.04238845	-0.1265836	0.000261213	0.001366323	14	0	0
STX4	0.1212276	0.139771528	-0.01854396	-0.23557042	0.000261789	0.001368933	13	0	0
ATAD3C	0.7507222	0.76694159	-0.0162194	-0.02073583	0.000262226	0.00137082	6	0	0
HTR5A	0.196278	0.141392017	0.054885934	0.443005937	0.000262517	0.001371944	8	0	0
HTR1F	0.843441	0.874008985	-0.03056801	-0.05126126	0.000263148	0.001374838	4	0	0
ASTL	0.4338899	0.4781511	-0.04426121	-0.16888083	0.000263706	0.001377353	6	0	0
HIST1H2BM	0.2777306	0.260025344	0.017705228	0.514520593	0.000263794	0.001377412	5	0	0
WLS	0.2416539	0.254579416	-0.01292552	0.232812898	0.000265225	0.001384482	7	0	0
ATPAF1	0.8482655	0.882538466	-0.034273	-0.06057631	0.000266008	0.001388164	4	0	0
LAMB3	0.6264503	0.72972135	-0.10327108	-0.2152325	0.00026681	0.001391602	6	0	0
EPS8L2	0.3021874	0.355840608	-0.05365323	-0.21413882	0.000266822	0.001391602	7	0	0
EVI2A	0.6745071	0.771399365	-0.09689223	-0.21106608	0.00026697	0.00139197	5	0	0
THY1	0.7890432	0.842734127	-0.05369092	-0.09940267	0.000268867	0.001401451	5	0	0
NDUFAF1	0.2971573	0.339066258	-0.04190891	-0.09224846	0.000269503	0.00140436	11	0	0
SEMA4C	0.0730954	0.085121429	-0.01202605	-0.07732986	0.000269894	0.001405582	4	0	0

PDK4	0.1419757	0.096295884	0.045679842	0.537100492	0.000269877	0.001405582	8	0	0
PCDHB3	0.4113801	0.331054332	0.080325783	0.446269104	0.000272684	0.001419699	8	0	0
NR6A1	0.225213	0.250102784	-0.02488978	0.083541118	0.000272984	0.001420437	6	0	0
PMM2	0.8236089	0.859554879	-0.03594593	-0.06102384	0.000272943	0.001420437	4	0	0
CRMP1	0.3942002	0.343711044	0.050489124	0.303977552	0.000273178	0.00142103	4	0	0
CRHBP	0.6303262	0.660978793	-0.0306526	-0.03175207	0.000274675	0.001428399	9	0	0
HBQ1	0.4694591	0.407703467	0.061755678	0.629705866	0.000274753	0.001428399	11	0	0
PGRMC2	0.2278114	0.256865429	-0.02905405	-0.14883303	0.000274848	0.001428477	9	0	0
GZMB	0.6120704	0.641996696	-0.02992635	-0.05307068	0.000275114	0.001429447	7	0	0
OPALIN	0.6668218	0.727253555	-0.0604318	-0.12507493	0.000275571	0.001430859	7	0	0
PIGQ	0.4853596	0.417955391	0.067404183	0.419265485	0.000275626	0.001430859	6	0	0
NHLRC4	0.4853596	0.417955391	0.067404183	0.419265485	0.000275626	0.001430859	6	0	0
CMTM5	0.5872055	0.551065559	0.03613995	0.141995029	0.000275921	0.001431981	9	0	0
SRRM4	0.1512827	0.094986782	0.056295908	0.74566934	0.000276184	0.001432928	17	0	0
NR1H4	0.5957906	0.645879668	-0.05008902	-0.0942547	0.000276324	0.001433241	8	0	0
ZBBX	0.8032302	0.877943376	-0.07471322	-0.12680396	0.000276513	0.001433804	1	0	0
DDX56	0.6195099	0.703048871	-0.08353901	-0.19217284	0.000276593	0.001433806	4	0	0
TSGA13	0.5122302	0.587654804	-0.07542462	-0.19323851	0.000277331	0.001437217	2	0	0
RAPGEF1	0.2228293	0.23836807	-0.01553875	-0.11940256	0.000277729	0.001438862	4	0	0
CDH4	0.3577083	0.313128176	0.044580149	0.570408061	0.000278557	0.001442323	6	0	0
CSN1S1	0.8547022	0.882000151	-0.02729792	-0.04484055	0.000280418	0.001451121	1	0	0
ZNF23	0.3825778	0.459610207	-0.07703237	-0.21328173	0.000280382	0.001451121	4	0	0
KRTAP10-1	0.6341157	0.692658363	-0.05854262	-0.12247936	0.000280875	0.001453064	5	0	0
C1orf94	0.1533489	0.094036069	0.059312858	0.623879823	0.000280994	0.001453263	15	0	0
C17orf98	0.7583239	0.707185828	0.051138061	0.123275451	0.000281437	0.001455135	9	0	0
GPR15	0.6190544	0.682409602	-0.0633552	-0.20684513	0.000281673	0.001455931	4	0	0
CELA2B	0.668717	0.717833808	-0.04911677	-0.13779772	0.000282064	0.001457535	3	0	0
FCGR2A	0.6770373	0.744275799	-0.06723848	-0.14522987	0.000282874	0.001460456	6	0	0
TMEM177	0.2956709	0.344893434	-0.04922254	-0.25933327	0.000282714	0.001460456	7	0	0
METTL24	0.481328	0.504648921	-0.02332091	0.161650787	0.000282813	0.001460456	6	0	0
NOLC1	0.2519907	0.287197776	-0.03520703	-0.05920623	0.000282988	0.001460623	11	0	0
LCE3A	0.423541	0.392982107	0.030558911	0.173368333	0.000283204	0.001461318	8	0	0
PLEKHJ1	0.5780943	0.6929122	-0.11481789	-0.25729696	0.0002833	0.001461394	1	0	0
PPP4C	0.1965417	0.247382643	-0.05084097	-0.10167884	0.000283755	0.001463323	7	0	0
NIPAL3	0.2360752	0.287447081	-0.05137189	-0.12805817	0.000284083	0.001464168	11	0	0
CLEC3A	0.5944905	0.671135032	-0.07664452	-0.1702619	0.000284038	0.001464168	5	0	0
KLK9	0.5733297	0.646181767	-0.07285206	-0.16650448	0.00028537	0.00147038	7	0	0
PTPRT	0.204994	0.193880946	0.011113078	0.536159379	0.000286159	0.001474025	9	0	0
SLC1A7	0.7144136	0.769801355	-0.05538777	-0.11594725	0.000287541	0.001480717	7	0	0
GPR52	0.7809021	0.853555243	-0.0726531	-0.12927871	0.000287969	0.001482493	5	0	0
14-Sep	0.6459028	0.701898765	-0.05599594	-0.12137616	0.000288807	0.001486382	2	0	0
DNAJC6	0.6473844	0.548084246	0.099300193	0.236254384	0.000290046	0.001491901	1	0	0
RBPJL	0.4581128	0.394717472	0.063395355	0.353746072	0.00029052	0.001493915	12	0	0
LOR	0.7916848	0.853312003	-0.06162717	-0.10810883	0.000290705	0.001494434	6	0	0
FMO2	0.636834	0.549954781	0.086879208	0.256878783	0.000292387	0.001502651	6	0	0
MRGPRX2	0.5204437	0.607691943	-0.08724823	-0.23589857	0.00029311	0.001505936	4	0	0
LTBR	0.539525	0.636417087	-0.09689214	-0.2379943	0.000293666	0.001508362	8	0	0
ACAT2	0.8684858	0.903012996	-0.03452717	-0.05561638	0.000294217	0.001510757	1	0	0
CLEC4M	0.7228912	0.662869658	0.060021557	0.118096678	0.000294486	0.001511705	3	0	0
LRRC39	0.7817569	0.822120242	-0.04036336	-0.07516387	0.000294846	0.001513122	5	0	0
CMTM8	0.2451779	0.205343272	0.039834611	0.159264132	0.000295321	0.001515126	2	0	0
SLC35B3	0.4423463	0.515249513	-0.07290318	-0.1917258	0.00029563	0.00151624	9	0	0
SCGB1D2	0.3382556	0.403113592	-0.06485795	-0.20933932	0.000295707	0.00151624	2	0	0
BABAM1	0.1379869	0.164583627	-0.02659678	-0.1011885	0.000296192	0.001518291	12	0	0
DDI1	0.8557054	0.906875736	-0.05117037	-0.08291531	0.000296305	0.001518436	2	0	0
XPNPEP1	0.2273826	0.271101671	-0.04371902	-0.15990529	0.00029726	0.001522459	10	0	0
GAPDH	0.0825466	0.105859387	-0.02331276	-0.18543832	0.000297212	0.001522459	8	0	0
CARNS1	0.3812868	0.433891117	-0.05260427	-0.17617171	0.00029907	0.001531292	4	0	0
PLEC	0.2109193	0.25495118	-0.04403191	-0.12741523	0.00029929	0.001531982	6	0	0
EPHA1	0.1827243	0.219761591	-0.03703729	-0.18840682	0.000300124	0.001535377	11	0	0
EBPL	0.1236582	0.16382918	-0.04017098	-0.1913446	0.000300123	0.001535377	8	0	0
GPA33	0.8003865	0.843785707	-0.0433992	-0.07425486	0.000300551	0.00153712	3	0	0
AMPH	0.3379279	0.350796105	-0.01286822	0.331489378	0.000300913	0.001538535	13	0	0

DCST2	0.7556846	0.798558877	-0.04287423	-0.08036423	0.000301316	0.001540156	8	0	0
KIF25	0.8435103	0.887807703	-0.04429738	-0.07717117	0.000301609	0.001540425	6	0	0
DPY19L2	0.1001157	0.054897915	0.045217806	0.789322155	0.000301626	0.001540425	3	0	0
CKM	0.4006267	0.343448734	0.057177983	0.33316227	0.000301496	0.001540425	7	0	0
C10orf62	0.7816945	0.820350003	-0.03865548	-0.07360107	0.000302074	0.00154227	3	0	0
CCNA1	0.1612543	0.100826408	0.060427911	0.581476345	0.000303162	0.001547385	25	0	0
MESP2	0.5605927	0.553161705	0.007430982	-0.01361879	0.000303354	0.001547927	5	0	0
PRPH	0.5232233	0.486659759	0.036563511	0.111248893	0.000303609	0.001548786	9	0	0
LYNX1	0.5353018	0.575765441	-0.04046369	0.073940422	0.000304218	0.001551011	12	0	0
SCG3	0.2920351	0.31105511	-0.01902001	0.113935228	0.000304184	0.001551011	6	0	0
OR7C1	0.7745699	0.775400327	-0.00083044	0.030915489	0.000304751	0.001553288	5	0	0
NSUN5	0.2317786	0.284446526	-0.05266795	0.15551882	0.00030525	0.001555386	4	0	0
MUC21	0.6309391	0.683045946	-0.05210681	-0.10256515	0.000305562	0.001556537	9	0	0
CLNK	0.5703414	0.637996349	-0.06765494	-0.17121006	0.000306107	0.001557982	10	0	0
MFSD8	0.1879136	0.212538918	-0.02462531	-0.10650487	0.000305963	0.001557982	11	0	0
GPC5	0.0852457	0.101136645	-0.01589093	-0.15149125	0.00030604	0.001557982	11	0	0
NAT1	0.7499077	0.789919693	-0.04001199	-0.07277502	0.000306304	0.001558543	3	0	0
CRTAM	0.760381	0.852047045	-0.09166601	-0.16219442	0.000306449	0.001558651	1	0	0
MS4A2	0.7459863	0.813103583	-0.06711727	-0.12239515	0.000308733	0.001569567	4	0	0
KLHL1	0.495383	0.458005856	0.037377154	0.376297511	0.000309171	0.001571348	12	0	0
GRIA2	0.7943668	0.85098388	-0.05661713	-0.10561372	0.000309327	0.001571479	3	0	0
DTD2	0.2314736	0.261784192	-0.03031055	-0.01930906	0.000309372	0.001571479	10	0	0
SLC51B	0.6274842	0.568620563	0.058863595	0.157278314	0.000311423	0.001581003	6	0	0
CCDC144A	0.8170913	0.845209045	-0.02811771	-0.04727168	0.000311407	0.001581003	2	0	0
RTN3	0.1167156	0.146506026	-0.02979043	-0.20939965	0.000311652	0.001581716	9	0	0
ZBTB37	0.0618214	0.078660363	-0.01683899	-0.14717754	0.000312313	0.001582897	17	0	0
TDRD15	0.7911155	0.829919511	-0.03880403	-0.0639795	0.000312414	0.001582897	5	0	0
F2RL2	0.4757091	0.54531715	-0.06960805	-0.25702084	0.000312338	0.001582897	7	0	0
ABCB4	0.6696469	0.731549179	-0.06190229	-0.12529073	0.000311994	0.001582897	3	0	0
SIGLEC7	0.6002921	0.679528837	-0.07923676	-0.17217983	0.000312225	0.001582897	6	0	0
THBD	0.13151	0.07485951	0.05665052	0.817501935	0.000312226	0.001582897	10	0	0
BMPR1A	0.0701057	0.085289537	-0.01518384	-0.21473171	0.000313409	0.00158749	5	0	0
BRINP2	0.1684309	0.120793621	0.047637275	0.639776737	0.000313767	0.001588858	14	0	0
RNF19B	0.1186449	0.134195779	-0.01555085	0.070078998	0.000315051	0.001594909	7	0	0
ZNF502	0.4133755	0.37956494	0.033810514	0.412754233	0.000315413	0.001595841	12	0	0
ENOSF1	0.4557436	0.56132654	-0.10558294	-0.23071125	0.000315407	0.001595841	6	0	0
RABEPK	0.1895739	0.222152493	-0.03257864	-0.1844417	0.000315521	0.001595936	8	0	0
MAP3K2	0.371623	0.434330133	-0.06270714	-0.18285407	0.000315743	0.001596607	3	0	0
SBSN	0.5829285	0.63860717	-0.05567869	-0.1541883	0.000316457	0.001599767	6	0	0
AP3B2	0.3213579	0.325368739	-0.00401086	-0.10347132	0.000316568	0.001599877	10	0	0
IRX5	0.0994423	0.065210483	0.034231773	0.503289833	0.000316686	0.001600022	14	0	0
AKNAD1	0.1245238	0.154836556	-0.03031272	-0.098656	0.000317722	0.001603847	11	0	0
CLCC1	0.1245238	0.154836556	-0.03031272	-0.098656	0.000317722	0.001603847	11	0	0
TLX2	0.3841007	0.337980473	0.046120237	0.294308527	0.000317801	0.001603847	4	0	0
BST1	0.5216186	0.456377124	0.065241432	0.563481078	0.000317726	0.001603847	11	0	0
PURB	0.1180141	0.135689322	-0.01767518	-0.02685174	0.000318156	0.001605189	8	0	0
HSD17B3	0.7651789	0.7241925	0.040986353	0.078370837	0.000319026	0.001609125	1	0	0
SLC25A30	0.2420228	0.267192602	-0.02516977	-0.0810786	0.000319271	0.00160991	7	0	0
OLIG1	0.1783224	0.160826035	0.017496378	0.601083901	0.00031988	0.001612526	10	0	0
MYBPHL	0.6512932	0.651601197	-0.00030801	0.004269247	0.000320192	0.001613645	8	0	0
TFEB	0.6044219	0.663717145	-0.05929527	-0.11571826	0.000320448	0.001614412	9	0	0
FRRS1L	0.5564164	0.513487101	0.042929262	0.210332826	0.000320524	0.001614412	8	0	0
CD247	0.6748808	0.731732186	-0.05685143	-0.11259143	0.000321736	0.001620061	4	0	0
TNP2	0.7721389	0.826735334	-0.05459648	-0.09778073	0.000323615	0.001629065	3	0	0
PPARD	0.2392627	0.278149452	-0.03888672	-0.05397379	0.000324368	0.001632397	7	0	0
CLDN17	0.721531	0.721245777	0.000285208	0.026711574	0.000324498	0.001632594	7	0	0
ATP2B2	0.2200894	0.178437897	0.041651491	0.389281543	0.000325974	0.001639559	3	0	0
STMND1	0.2527703	0.266301941	-0.01353166	0.174494469	0.000326459	0.00164154	14	0	0
PAIP2	0.081699	0.08860402	-0.00690505	0.031474218	0.000326799	0.001642787	7	0	0
PIGV	0.0778821	0.046349996	0.031532122	0.411038006	0.000328479	0.001650215	11	0	0
TMEM64	0.2940206	0.239278658	0.05474195	0.321846109	0.000328419	0.001650215	7	0	0
LGI4	0.1779067	0.221510477	-0.04360377	-0.27070676	0.000328553	0.001650215	11	0	0
CSMD2	0.1441832	0.087767836	0.056415404	0.638543268	0.000329226	0.001651753	17	0	0

SLC22A14	0.7227881	0.756639119	-0.03385107	-0.0627824	0.000329079	0.001651753	4	0	0
PSMD6	0.6491762	0.681906856	-0.03273065	-0.0949661	0.000329124	0.001651753	8	0	0
NWD1	0.3890192	0.462812339	-0.0737931	-0.26242232	0.000329227	0.001651753	9	0	0
NCALD	0.3523077	0.345648257	0.006659448	0.069699527	0.000329525	0.001652784	10	0	0
CES5A	0.6376399	0.698235631	-0.06059574	-0.11850976	0.000329833	0.001653868	8	0	0
GLT1D1	0.5426681	0.592372006	-0.04970393	-0.12032575	0.000330486	0.001656488	3	0	0
MTG2	0.327262	0.363233438	-0.03597142	0.007200524	0.00033054	0.001656488	5	0	0
CLCN1	0.7680173	0.797229607	-0.02921232	-0.05208236	0.000330775	0.0016572	7	0	0
FAM168A	0.1533396	0.200660685	-0.0473211	-0.14720889	0.000331257	0.001659151	4	0	0
SPINK2	0.3384225	0.376740848	-0.0383183	-0.23652964	0.000332826	0.001666547	4	0	0
AARD	0.2645504	0.216034768	0.048515592	0.594008114	0.000332992	0.001666914	13	0	0
C7orf33	0.7555342	0.799221708	-0.04368755	-0.07703602	0.000333184	0.001667409	9	0	0
MILR1	0.8114435	0.869471458	-0.05802796	-0.10086373	0.000333765	0.001669849	6	0	0
CLEC4C	0.6507418	0.724513274	-0.07377151	-0.17825904	0.000334334	0.00167223	2	0	0
LMX1B	0.1496583	0.104959535	0.044698813	0.550187652	0.000335135	0.001675156	10	0	0
KCTD4	0.7683263	0.819167467	-0.05084122	-0.09407903	0.000335176	0.001675156	3	0	0
STRA6	0.7762646	0.815400043	-0.03913546	-0.07004466	0.000335199	0.001675156	2	0	0
RSPO4	0.1958077	0.135614	0.060193676	0.815445735	0.000335331	0.001675347	10	0	0
MATR3	0.1313403	0.160614029	-0.02927368	-0.06889167	0.000336015	0.001678298	10	0	0
FCRL2	0.788247	0.864122901	-0.07587588	-0.15333455	0.000336415	0.001679358	3	0	0
ACER2	0.3750647	0.343263956	0.031800783	0.351861298	0.000336414	0.001679358	6	0	0
COLGALT2	0.3839769	0.41054597	-0.02656903	-0.06761348	0.000336738	0.001680503	7	0	0
HTR3C	0.755525	0.775252425	-0.01972746	-0.0313477	0.000338027	0.001686001	3	0	0
FANCA	0.5925012	0.685588949	-0.09308772	-0.15396116	0.000337948	0.001686001	8	0	0
RREB1	0.1172787	0.160041593	-0.04276288	-0.15886124	0.000338153	0.001686156	10	0	0
SEMA5B	0.1553554	0.205370285	-0.05001488	-0.15640247	0.000338266	0.001686253	5	0	0
STK38	0.4721302	0.510728892	-0.03859871	-0.0111774	0.000338845	0.0016882	8	0	0
CEP85L	0.5816626	0.65004543	-0.0683828	-0.11106796	0.000338803	0.0016882	7	0	0
LRG1	0.3812633	0.433720899	-0.05245764	-0.23393777	0.000339345	0.001690225	7	0	0
MPDZ	0.4005477	0.408189006	-0.00764128	0.183900147	0.000339677	0.001691408	5	0	0
NPR3	0.6311255	0.704025186	-0.07289965	-0.13159741	0.000340742	0.001695768	2	0	0
BRINP3	0.1768865	0.122150942	0.054735578	0.524433109	0.000340903	0.001696099	9	0	0
TSHB	0.7805525	0.82265946	-0.04210701	-0.08149886	0.000341041	0.001696315	4	0	0
CKAP4	0.7030865	0.668597183	0.034489288	0.098335098	0.000341574	0.001698355	6	0	0
CCER2	0.4383552	0.401840658	0.036514551	0.145155888	0.000341641	0.001698355	6	0	0
HP	0.6319058	0.701992693	-0.07008689	-0.16213013	0.000342208	0.001700231	5	0	0
HPR	0.6319058	0.701992693	-0.07008689	-0.16213013	0.000342208	0.001700231	5	0	0
HIST1H2BJ	0.0650691	0.09393415	-0.02886509	-0.08678554	0.000343281	0.00170509	11	0	0
FBN1	0.3121533	0.272166779	0.039986533	0.906317279	0.000343774	0.001707066	7	0	0
SF3B4	0.4388216	0.447797065	-0.00897548	-0.13154267	0.000344072	0.001708077	11	0	0
SVOPL	0.5725198	0.618606965	-0.04608718	-0.13394964	0.000344967	0.001712046	3	0	0
TMEM132B	0.1602807	0.113806343	0.046474366	0.365530725	0.0003465	0.001719179	6	0	0
LIMD1	0.7261848	0.775498878	-0.04931406	-0.10746183	0.000346952	0.00172057	4	0	0
OR5B17	0.2254504	0.280835098	-0.05538473	-0.30477843	0.000346983	0.00172057	1	0	0
NUBP1	0.262428	0.275709752	-0.01328171	0.007070563	0.000347069	0.00172057	13	0	0
VIPR1	0.7635324	0.72605828	0.03747415	0.078154771	0.00034756	0.00172158	9	0	0
DDAH2	0.4528039	0.454765541	-0.00196161	0.174776679	0.000347475	0.00172158	17	0	0
PLCE1	0.3597432	0.398086694	-0.03834346	-0.1360669	0.000347406	0.00172158	6	0	0
MPO	0.5268489	0.596334426	-0.06948548	-0.19128975	0.000347919	0.00172288	4	0	0
KRTAP13-3	0.6043798	0.644472135	-0.04009232	-0.0935447	0.000348549	0.001725527	5	0	0
CTDSPL	0.0941741	0.071557944	0.022616148	0.34154423	0.000348861	0.001726595	7	0	0
BCL10	0.5760207	0.621311885	-0.04529122	-0.10084744	0.00034982	0.00173011	6	0	0
TFEC	0.7342292	0.801486275	-0.06725705	-0.13618311	0.000349702	0.00173011	2	0	0
TSGA10IP	0.7692009	0.738481616	0.030719306	0.068241049	0.000350144	0.001730556	8	0	0
GNB5	0.7727779	0.745207882	0.027570053	0.060393431	0.000350114	0.001730556	6	0	0
SBNO1	0.0381507	0.033413269	0.004737444	0.149421106	0.000350786	0.00173325	1	0	0
EIF4E3	0.3626617	0.327342386	0.035319329	0.414586679	0.000351474	0.001736171	13	0	0
CLEC4G	0.52926	0.615720919	-0.08646088	-0.22048013	0.000353151	0.001743975	2	0	0
RBM24	0.2115847	0.154998205	0.056586469	0.615112287	0.000354232	0.001748833	11	0	0
RGMA	0.1629522	0.113621293	0.049330896	0.554294336	0.00035448	0.001749576	12	0	0
RNF11	0.1821234	0.226259612	-0.04413626	-0.13284449	0.000354679	0.001750076	8	0	0
ST6GALNAC	0.1931368	0.170029679	0.023107146	0.556670224	0.000355	0.001750518	13	0	0
OR13C9	0.749768	0.826992574	-0.07722456	-0.14219838	0.000355043	0.001750518	2	0	0

ABCA2	0.1412465	0.16571273	-0.02446619	-0.09536163	0.000355062	0.001750518	8	0	0
LIN28A	0.1300323	0.079491568	0.050540737	0.623388734	0.000355575	0.001752175	15	0	0
FAM163A	0.1840883	0.110494842	0.073593456	0.87483597	0.000355593	0.001752175	8	0	0
PSMD9	0.0672843	0.084494206	-0.01720986	-0.15567213	0.000356245	0.001754424	13	0	0
SPATA7	0.3225755	0.348158444	-0.02558294	-0.02442388	0.000356182	0.001754424	7	0	0
MYRFL	0.7798939	0.84969824	-0.06980434	-0.12523427	0.000356365	0.00175453	4	0	0
EGFR	0.2127557	0.16726209	0.045493585	0.437666872	0.000356544	0.001754931	6	0	0
CXCL9	0.6373156	0.736288516	-0.09897294	-0.20526427	0.00035679	0.00175566	1	0	0
TMEM139	0.4248636	0.464400404	-0.03953682	-0.17596042	0.000356952	0.001755977	6	0	0
PAX8	0.8518281	0.850459752	0.001368381	0.005977074	0.000357491	0.001758144	8	0	0
ZNF385D	0.3644589	0.342536217	0.021922638	0.308177293	0.000358056	0.001760443	3	0	0
DAO	0.2023103	0.124583049	0.077727232	0.54210518	0.00035849	0.001762093	7	0	0
MC5R	0.1065758	0.140658564	-0.03408276	-0.33017693	0.000358711	0.001762697	3	0	0
CLDN9	0.5094986	0.439908544	0.069590066	0.315724167	0.000360814	0.001772546	7	0	0
HOXA9	0.2939673	0.257080755	0.036886549	0.325371056	0.000360954	0.001772746	5	0	0
SLC2A3	0.3384114	0.278880295	0.059531123	0.653916225	0.00036214	0.001778087	7	0	0
GHRL	0.6101521	0.584236935	0.02591517	0.132764837	0.000362684	0.001779779	6	0	0
SUN5	0.7221677	0.79737516	-0.07520745	-0.14561707	0.000362638	0.001779779	4	0	0
NPY1R	0.1184632	0.073921536	0.044541616	0.460832074	0.000363457	0.001783085	2	0	0
LMLN	0.1734902	0.245626445	-0.07213625	-0.17754801	0.00036356	0.001783105	10	0	0
NYAP2	0.423082	0.471290657	-0.04820865	-0.0879777	0.000363938	0.001783982	9	0	0
ARIH2OS	0.0797573	0.078081361	0.001675946	0.018945036	0.000363862	0.001783982	11	0	0
DEFB135	0.3993229	0.48905825	-0.08973539	-0.25906574	0.000364451	0.001786007	2	0	0
LPA	0.7516867	0.826454297	-0.07476762	-0.1387571	0.000365161	0.001788998	4	0	0
MROH6	0.3829158	0.423361421	-0.04044558	-0.09229288	0.000365719	0.001791246	12	0	0
TTLL5	0.8720104	0.891209183	-0.01919874	-0.03115345	0.000365833	0.001791311	2	0	0
CMPK2	0.3818405	0.41751173	-0.03567121	-0.10825157	0.000366798	0.001795546	8	0	0
ZNF469	0.7323313	0.780559027	-0.04822777	-0.09108102	0.000367078	0.001796429	7	0	0
HSPA4	0.1777546	0.18911506	-0.01136043	-0.05619478	0.000367506	0.00179803	10	0	0
KCNK18	0.6712818	0.736395368	-0.06511354	-0.13087072	0.000367645	0.001798223	11	0	0
SLC25A41	0.7248473	0.79312273	-0.06827545	-0.14684158	0.00036794	0.001799174	5	0	0
VAX2	0.2075999	0.14831609	0.059283823	0.424306427	0.000369609	0.001806844	15	0	0
AGTR1	0.2731918	0.214892391	0.058299361	0.648605941	0.000370253	0.001809082	9	0	0
DPYSL3	0.2459606	0.248553172	-0.00259256	0.281216184	0.00037037	0.001809082	16	0	0
ENGASE	0.6368188	0.691952762	-0.05513393	-0.11014134	0.000370344	0.001809082	5	0	0
NPFFR1	0.3614998	0.339348233	0.022151557	0.398497584	0.00037078	0.001810595	6	0	0
CCDC130	0.1991061	0.261267609	-0.06216151	-0.18091887	0.000371478	0.001813509	3	0	0
NRTN	0.7104351	0.770509703	-0.06007463	-0.10728212	0.000371943	0.001815282	7	0	0
CTSG	0.5678413	0.646039641	-0.07819834	-0.19424321	0.000372339	0.001816723	7	0	0
STRADB	0.5512457	0.627141219	-0.07589555	-0.18298078	0.00037272	0.001818086	1	0	0
S100A9	0.4783095	0.532478338	-0.05416884	-0.1621856	0.000373008	0.001818999	10	0	0
ZNF844	0.2670979	0.208589289	0.058508607	0.697545372	0.000375512	0.001830709	11	0	0
CBLC	0.344857	0.419910569	-0.07505358	-0.2606036	0.000375874	0.001831976	11	0	0
MS4A12	0.7370205	0.814133109	-0.07711261	-0.14172954	0.0003762	0.001833069	1	0	0
HEG1	0.2973106	0.309089566	-0.01177895	-0.00556548	0.00037735	0.00183817	7	0	0
WNT8A	0.7806075	0.802341775	-0.02173423	-0.03527468	0.000377967	0.001840675	3	0	0
LMOD3	0.7815277	0.796924795	-0.01539714	-0.03667385	0.000378931	0.001844871	6	0	0
ZBTB47	0.3650603	0.32618194	0.038878363	0.175725494	0.000379188	0.00184562	9	0	0
VEGFA	0.2993875	0.268476272	0.030911206	0.094594663	0.000379705	0.001847635	10	0	0
KRT25	0.6684388	0.733481462	-0.06504268	-0.13266691	0.00038023	0.001849689	3	0	0
MAT2B	0.3261681	0.30458331	0.021584815	0.152686501	0.000381755	0.001856603	10	0	0
STK3	0.2758415	0.195983209	0.079858334	0.515472135	0.000385354	0.0018736	11	0	0
FLG	0.6669248	0.764632944	-0.0977081	-0.19901027	0.000385982	0.001876144	4	0	0
FAM3B	0.857909	0.906981471	-0.04907245	-0.08111599	0.000386398	0.001877657	8	0	0
KLK12	0.4648385	0.472291043	-0.00745258	-0.06881384	0.000386976	0.001879955	7	0	0
KCNN2	0.1105802	0.11694638	-0.00636619	0.121319813	0.000387579	0.001881869	6	0	0
SNX24	0.8328533	0.856578469	-0.02372514	-0.0402632	0.000387496	0.001881869	4	0	0
TRIM60	0.8047453	0.854264552	-0.04951928	-0.09090964	0.000388073	0.001883756	7	0	0
SPATA21	0.7960733	0.824510715	-0.02843737	-0.04858303	0.000388515	0.001885392	5	0	0
CFH	0.4517462	0.499947438	-0.04820122	-0.2462125	0.000388988	0.001887177	6	0	0
TERF2	0.7978117	0.874095412	-0.07628367	-0.13018296	0.000389152	0.001887463	1	0	0
ANKRD29	0.1542847	0.166728284	-0.01244357	-0.02258035	0.000390011	0.001891118	6	0	0
MOK	0.2622223	0.303826068	-0.04160381	-0.0710391	0.000390417	0.001892572	9	0	0

SORCS2	0.4897697	0.52139928	-0.03162958	0.042148551	0.000390865	0.001893722	6	0	0
PRLHR	0.2743232	0.220496802	0.053826417	0.316743084	0.000390851	0.001893722	8	0	0
RDH14	0.287888	0.315917966	-0.02802998	-0.12260741	0.000391109	0.001893879	12	0	0
FBXO40	0.5849477	0.54998212	0.034965554	0.130400318	0.000391105	0.001893879	8	0	0
RGS18	0.6398264	0.739051508	-0.09922509	-0.20501053	0.0003916	0.001895748	1	0	0
KCNJ3	0.1270699	0.081547808	0.045522102	0.640018788	0.00039235	0.001898655	14	0	0
GPR183	0.6335515	0.624316244	0.009235274	-0.01504425	0.000392624	0.001898655	6	0	0
PRM2	0.8146658	0.859221277	-0.04455547	-0.07802011	0.000392496	0.001898655	11	0	0
TCFL5	0.0522626	0.062045452	-0.00978289	-0.21054252	0.000392584	0.001898655	1	0	0
HTR1A	0.1759568	0.132010361	0.043946445	0.417061576	0.000392932	0.001899632	10	0	0
PTH2	0.3046417	0.243003622	0.061638098	0.427117423	0.0003932	0.001900414	11	0	0
KDR	0.1298799	0.076205664	0.053674218	0.759114395	0.000393346	0.001900609	10	0	0
IGF2R	0.274109	0.29458364	-0.02047463	-0.15706381	0.000393822	0.001902394	17	0	0
CLDN20	0.8812632	0.86365443	0.017608779	0.029578881	0.000393949	0.001902497	6	0	0
NXPH1	0.1918	0.134030426	0.057769567	0.803424978	0.000394214	0.001903264	11	0	0
SPTBN5	0.4597842	0.499584856	-0.03980064	-0.06788613	0.00039445	0.001903378	9	0	0
DLGAP1	0.1687897	0.110334509	0.058455186	0.61657879	0.000394389	0.001903378	3	0	0
FAM220A	0.1468935	0.164326534	-0.01743302	-0.15136339	0.000394707	0.001904106	6	0	0
PDGFD	0.145006	0.166404407	-0.02139844	0.071207946	0.000395232	0.001906126	8	0	0
RD3L	0.7904363	0.820145058	-0.02970874	-0.05784494	0.000395923	0.001908944	10	0	0
CILP	0.6369025	0.680771192	-0.04386874	-0.09399374	0.000396075	0.001909165	9	0	0
DNMBP	0.2955077	0.314825912	-0.01931823	-0.028828	0.000397715	0.001916038	6	0	0
CCL25	0.6034704	0.658882041	-0.0554116	-0.15061343	0.000397649	0.001916038	9	0	0
CDC23	0.2295602	0.241041573	-0.01148132	0.028910586	0.000398472	0.001918649	13	0	0
THRSP	0.8095588	0.808830446	0.000728323	0.003666669	0.000398578	0.001918649	5	0	0
KCNJ6	0.1705205	0.142648591	0.027871958	0.419146514	0.000398442	0.001918649	10	0	0
SLC6A11	0.3169953	0.264660347	0.052334974	0.480611091	0.000399308	0.00192165	11	0	0
RBBP8NL	0.4622929	0.516397945	-0.05410502	-0.1412454	0.000399818	0.001923074	10	0	0
TMCC1	0.1431917	0.153523709	-0.01033204	-0.10047604	0.000399999	0.001923425	7	0	0
HOXB5	0.1988359	0.13886463	0.059971298	0.629341622	0.000400506	0.00192535	14	0	0
SLC10A1	0.6543502	0.649066096	0.005284147	-0.00628524	0.0004015	0.001929608	7	0	0
TMEM220	0.5728814	0.634625741	-0.06174433	-0.09602472	0.000402017	0.001931575	6	0	0
ZNF621	0.1044515	0.121429684	-0.01697823	-0.13808793	0.000402663	0.001934162	10	0	0
C22orf31	0.6246396	0.689967391	-0.0653278	-0.15210124	0.000403593	0.001937963	4	0	0
CDC42EP5	0.4712442	0.501357472	-0.03011327	0.156172584	0.000403891	0.001938505	5	0	0
NUDT17	0.2034303	0.248287407	-0.04485713	-0.19152669	0.000404148	0.001939219	8	0	0
HTR6	0.3820333	0.418002334	-0.03596905	0.057760296	0.000404342	0.001939629	10	0	0
IFFO1	0.6053508	0.540362766	0.064988058	0.292407794	0.000405366	0.001944024	11	0	0
CORT	0.846594	0.873143707	-0.02654968	-0.04445793	0.000405958	0.001946344	7	0	0
ZNF208	0.6612775	0.609802163	0.051475323	0.232970818	0.000406369	0.00194779	2	0	0
DMWD	0.264657	0.284505632	-0.01984868	0.053797006	0.000406625	0.001948496	5	0	0
TNKS2	0.2374929	0.291259006	-0.05376607	-0.10983875	0.000407208	0.001950772	6	0	0
EIF4ENIF1	0.082793	0.114429782	-0.03163676	-0.11273234	0.000410052	0.001963868	11	0	0
TMEM106A	0.1218953	0.0535742	0.068321114	1.164003384	0.000410213	0.001964117	12	0	0
AMH	0.7682274	0.80556053	-0.03733315	-0.07484346	0.000411251	0.001968559	7	0	0
IAH1	0.5771617	0.632080352	-0.0549187	-0.14924994	0.000411644	0.001969918	8	0	0
SIX3	0.1731295	0.092752818	0.080376689	0.860141047	0.000413725	0.00197882	8	0	0
MYOM1	0.6993153	0.759525725	-0.06021045	-0.12748459	0.000413622	0.00197882	6	0	0
UGT3A2	0.838158	0.895516674	-0.05735864	-0.09470748	0.000414323	0.001981153	2	0	0
CLIC4	0.1163222	0.128375377	-0.01205321	-0.0231777	0.000415391	0.001985729	9	0	0
SULT2B1	0.5418261	0.602560124	-0.06073403	-0.14680429	0.00041798	0.001997572	9	0	0
P2RX2	0.2190552	0.157614263	0.061440959	0.600119684	0.0004181	0.001997614	8	0	0
LY6H	0.1733501	0.147390823	0.025959296	0.553998657	0.000419051	0.002001625	9	0	0
XPO7	0.1272669	0.157691277	-0.03042439	-0.12421752	0.000419624	0.00200383	9	0	0
LZTS1	0.0890022	0.099038098	-0.01003591	-0.18185878	0.000419862	0.002004428	6	0	0
TXNDC11	0.0693778	0.101038244	-0.03166047	-0.13414056	0.000419974	0.002004431	9	0	0
SMARCC1	0.1054685	0.106301305	-0.00083278	0.095091994	0.000420546	0.002006629	12	0	0
APCDD1L	0.2036188	0.166541885	0.03707688	0.378822647	0.000421005	0.002008285	9	0	0
CERS4	0.7638056	0.852666676	-0.08886104	-0.16129817	0.000422269	0.002012766	2	0	0
A1BG	0.6763763	0.716702945	-0.04032669	-0.08794128	0.000422281	0.002012766	7	0	0
MAST3	0.3218446	0.355529362	-0.03368481	-0.11215061	0.000423005	0.002015679	7	0	0
MAFA	0.1846895	0.206816923	-0.02212741	-0.06670525	0.000425381	0.002025388	9	0	0
AGPAT2	0.4396773	0.424109738	0.015567602	0.052667238	0.000425182	0.002025388	4	0	0

CHST8	0.4987207	0.488290219	0.010430451	0.211142494	0.000425357	0.002025388	11	0	0
SLC16A11	0.2272031	0.253018979	-0.02581592	-0.11157301	0.000426653	0.002030905	8	0	0
RAPGEFL1	0.471108	0.537165239	-0.06605725	-0.17325645	0.000426833	0.002031223	8	0	0
KREMEN1	0.0853313	0.081658819	0.003672529	0.048596514	0.000428135	0.00203688	10	0	0
ZC3H7A	0.291319	0.32872871	-0.03740967	-0.0296214	0.000428417	0.002037677	5	0	0
ST14	0.4678332	0.504254068	-0.03642086	-0.15688115	0.00042856	0.002037698	7	0	0
EVA1C	0.0488567	0.032075631	0.016781074	0.516393188	0.000428648	0.002037698	10	0	0
SLC16A5	0.3095256	0.240199097	0.069326484	0.859506612	0.00042948	0.002041111	13	0	0
C12orf10	0.2224883	0.232734533	-0.01024625	-0.09551944	0.000429961	0.002042856	14	0	0
TBC1D10C	0.5401028	0.578925538	-0.03882273	-0.06616686	0.000430253	0.002043572	7	0	0
FGF14	0.197623	0.178536942	0.019086013	0.35342543	0.00043034	0.002043572	12	0	0
PTPRA	0.863629	0.887499467	-0.02387042	-0.04006153	0.000431174	0.002046992	12	0	0
ADAP1	0.6231184	0.691004143	-0.06788578	-0.13354156	0.000431899	0.00204984	8	0	0
TRIP6	0.2233638	0.256932301	-0.03356851	-0.13860263	0.000432002	0.00204984	12	0	0
TPRA1	0.1142855	0.140215075	-0.02592959	-0.07999	0.000433289	0.002055399	13	0	0
SCIMP	0.6310028	0.690541316	-0.05953853	-0.13130226	0.000433858	0.002057554	7	0	0
CHML	0.7771019	0.777230771	-0.00012889	0.009498361	0.0004345	0.002059858	3	0	0
SIRT4	0.3129531	0.314023431	-0.00107036	0.137217842	0.000434573	0.002059858	7	0	0
ZNF300	0.359421	0.292817872	0.066603091	0.359556931	0.00043522	0.002062379	10	0	0
SLC4A5	0.7700017	0.809799265	-0.03979752	-0.08274416	0.000435368	0.002062535	7	0	0
ST6GALNAC	0.4067174	0.348110207	0.058607195	0.207931236	0.000435795	0.002064015	2	0	0
DBF4B	0.2472011	0.275146854	-0.02794577	-0.05226791	0.000435944	0.002064175	9	0	0
CST9	0.2008696	0.247287152	-0.0464176	-0.23300696	0.000436079	0.002064269	5	0	0
SLCO6A1	0.8228362	0.860511457	-0.03767521	-0.06467135	0.000436659	0.002066468	7	0	0
LAYN	0.2601438	0.230128946	0.030014864	0.356948898	0.000437158	0.002068282	10	0	0
C3orf36	0.8326858	0.879637287	-0.04695146	-0.07835412	0.000437387	0.002068823	6	0	0
ATAD5	0.1369677	0.182370891	-0.0454032	-0.1217952	0.000438537	0.002073716	7	0	0
TREM1	0.5710637	0.605397304	-0.03433361	-0.0643317	0.000439482	0.002077637	8	0	0
TNIP1	0.2712623	0.208761047	0.062501292	0.586571475	0.000439774	0.002078468	2	0	0
ACTG2	0.7235272	0.712565567	0.010961667	0.024354936	0.000440154	0.002079473	5	0	0
SLC4A7	0.0951812	0.11018986	-0.01500861	-0.07329117	0.000440372	0.002079473	12	0	0
MEGF10	0.1877709	0.140650117	0.047120784	0.464732958	0.000440261	0.002079473	10	0	0
DNAJC12	0.584156	0.607894492	-0.02373851	-0.00796347	0.00044045	0.002079473	6	0	0
COL5A3	0.3200833	0.341378122	-0.02129486	0.173746587	0.000442155	0.00208697	5	0	0
WNT9B	0.6830777	0.636034689	0.047043037	0.103787141	0.000442358	0.002087382	3	0	0
DSC2	0.2758577	0.307325518	-0.03146784	0.003992264	0.0004428	0.002088917	9	0	0
SCNN1A	0.5154947	0.555462318	-0.03996764	-0.11705948	0.000443614	0.002092207	8	0	0
LRCOL1	0.3815108	0.406552837	-0.025042	0.153053131	0.000444474	0.002095713	10	0	0
ZNF569	0.1582591	0.110016671	0.048242421	0.944474913	0.000444632	0.002095907	14	0	0
PKLR	0.662668	0.713408447	-0.05074049	-0.11206923	0.000444818	0.002096229	8	0	0
SAMD8	0.6580262	0.720771277	-0.06274505	-0.13004609	0.000445503	0.00209891	8	0	0
BCL2L11	0.3172627	0.313662149	0.003600565	-0.00371765	0.000446097	0.002101153	7	0	0
ZNF281	0.2203489	0.241140071	-0.02079121	-0.06068991	0.000446736	0.002103095	5	0	0
OR2S2	0.6241451	0.732904968	-0.10875991	-0.22836479	0.000446743	0.002103095	1	0	0
MPV17	0.7557108	0.810442656	-0.05473188	-0.1006507	0.000447767	0.00210736	4	0	0
KCNA5	0.2441691	0.210652524	0.033516623	0.227634343	0.000448172	0.002108713	3	0	0
OR1J4	0.3904395	0.471105013	-0.08066555	-0.27400668	0.000448576	0.002110058	2	0	0
ROS1	0.558353	0.595876091	-0.03752311	-0.12099142	0.000448903	0.002111044	8	0	0
MUC16	0.6515892	0.718151137	-0.06656193	-0.11747522	0.000449673	0.002114112	3	0	0
KCNJ12	0.4641144	0.477620835	-0.01350641	0.254007607	0.000450393	0.002116939	7	0	0
APLP1	0.3775443	0.420621007	-0.04307671	-0.03446305	0.000450867	0.002118615	7	0	0
SPRR1A	0.5711633	0.606253754	-0.03509041	-0.05726771	0.000451865	0.00212275	6	0	0
METTL22	0.3463999	0.384361583	-0.03796172	-0.16731908	0.000452242	0.002123964	3	0	0
DEF6	0.2134727	0.237052588	-0.02357989	-0.18375485	0.000452806	0.002126056	14	0	0
STEAP2	0.6814197	0.616503554	0.064916184	0.142239095	0.000453355	0.002128067	1	0	0
JPH3	0.2065586	0.20876063	-0.00220205	0.388775912	0.000453709	0.002128067	10	0	0
SUZ12	0.4162666	0.450833295	-0.0345667	-0.00574817	0.00045358	0.002128067	2	0	0
ABCA5	0.0648004	0.087399056	-0.02259867	-0.16913949	0.00045361	0.002128067	9	0	0
MRPS14	0.2422097	0.280213957	-0.03800426	-0.12776291	0.000454249	0.002130041	11	0	0
GALNTL5	0.6695334	0.734917975	-0.06538453	-0.14888799	0.000454492	0.002130625	5	0	0
TNNT3	0.6326933	0.68110078	-0.04840751	-0.10986561	0.000454931	0.002132124	12	0	0
PPEF2	0.6889858	0.731405157	-0.04241935	-0.08896618	0.000455888	0.002136053	6	0	0
PROC	0.8250719	0.848754519	-0.02368263	-0.0387793	0.0004565	0.002137805	6	0	0

SCARB1	0.8406651	0.875165956	-0.0345009	-0.05712179	0.000456489	0.002137805	3	0	0
TMC7	0.2682405	0.289966176	-0.02172568	0.214842329	0.000456967	0.002139433	9	0	0
TXNRD1	0.3927678	0.384478022	0.008289762	0.587731301	0.000457545	0.002141579	12	0	0
CNKSR1	0.617426	0.672846014	-0.05541999	-0.13124255	0.000457696	0.002141728	6	0	0
FN3K	0.2338225	0.205719798	0.028102671	0.550961915	0.000458197	0.002143512	4	0	0
ANGPTL2	0.3444522	0.298943791	0.045508407	0.483868963	0.000459728	0.002150111	9	0	0
SNCG	0.4836244	0.434442696	0.049181663	0.162093121	0.000459931	0.002150502	9	0	0
SLC13A5	0.1742012	0.112941912	0.06125925	0.82665717	0.000460185	0.002151128	10	0	0
IGIP	0.8269212	0.854121127	-0.02719993	-0.0473599	0.000462397	0.002160903	5	0	0
FBXW4	0.4523864	0.484321466	-0.03193508	-0.08033786	0.000464346	0.002169449	6	0	0
CFI	0.657264	0.647884782	0.009379194	0.12513149	0.000464659	0.002170346	3	0	0
GTF2A1L	0.6770231	0.779067862	-0.10204472	-0.20106643	0.000464868	0.002170757	11	0	0
GNRH2	0.7104767	0.705013647	0.005463089	0.022643119	0.000465811	0.002174591	9	0	0
SYNPO2	0.2127114	0.176799167	0.035912211	0.703890748	0.000466451	0.002176448	13	0	0
OR6C1	0.7161016	0.797107651	-0.081006	-0.16778303	0.000466357	0.002176448	3	0	0
C1orf210	0.6117403	0.693538485	-0.08179823	-0.18148852	0.000466717	0.002177121	9	0	0
TMEM167B	0.6965606	0.817541822	-0.1209812	-0.22440089	0.000467382	0.002179659	2	0	0
KRTAP2-3	0.6410319	0.637792016	0.003239849	0.051769183	0.000467891	0.002180997	6	0	0
NCAN	0.1921887	0.162674011	0.029514738	0.549582757	0.000467912	0.002180997	11	0	0
RABAC1	0.1387649	0.144862258	-0.00609735	-0.02990061	0.000468207	0.002181804	9	0	0
SLC12A3	0.8473595	0.891717347	-0.04435785	-0.07280676	0.000468523	0.002182708	4	0	0
DYM	0.6605933	0.743185766	-0.08259248	-0.1674236	0.000469008	0.0021844	4	0	0
CALHM3	0.6386925	0.685650066	-0.04695755	-0.11567487	0.000469478	0.002186022	11	0	0
ZNF625	0.1478019	0.086268785	0.061533091	0.846644715	0.000471024	0.002192653	7	0	0
SHISA4	0.2895081	0.264965988	0.024542138	0.3470325	0.000471367	0.002193108	16	0	0
PHC3	0.3037601	0.366562764	-0.06280268	-0.20728576	0.000471341	0.002193108	8	0	0
EIF3F	0.7685854	0.808566096	-0.03998073	-0.07224369	0.000471684	0.002194015	1	0	0
SYBU	0.2249308	0.24280519	-0.01787437	0.235731148	0.000471894	0.002194164	12	0	0
HAUS4	0.207893	0.218454022	-0.010561	-0.10862826	0.000471961	0.002194164	10	0	0
ECM1	0.8173978	0.789815478	0.027582281	0.05849938	0.000472216	0.002194781	5	0	0
CPA5	0.7970882	0.822958823	-0.02587064	-0.04594317	0.000473225	0.002198903	4	0	0
OXT	0.6736304	0.686122554	-0.01249213	0.154452949	0.000475054	0.002206828	15	0	0
KNCN	0.6899373	0.737715805	-0.0477785	-0.09490622	0.000475991	0.002210036	8	0	0
CHD1	0.1907397	0.198228624	-0.00748894	0.08487647	0.000475897	0.002210036	5	0	0
BCL2L14	0.8727879	0.909568492	-0.03678063	-0.06302679	0.000476243	0.002210633	3	0	0
MRAP	0.8106042	0.842788286	-0.0321841	-0.05710152	0.000476512	0.002211312	3	0	0
SLC6A17	0.3057693	0.299766037	0.006003231	0.175693135	0.000477619	0.002215877	7	0	0
SGCZ	0.307824	0.344884442	-0.03706048	0.026338799	0.00047799	0.002216451	9	0	0
KRTAP4-3	0.7324731	0.766049032	-0.03357588	-0.06571998	0.000477914	0.002216451	3	0	0
SPEM1	0.6642125	0.662029171	0.002183295	0.017420186	0.000478227	0.002216974	13	0	0
ATP6V1G3	0.7241555	0.81787338	-0.09371791	-0.17676818	0.000478897	0.002217236	5	0	0
LIMS2	0.8276088	0.778034304	0.049574544	0.105380497	0.000478786	0.002217236	5	0	0
IGSF11	0.7161929	0.752076567	-0.0358837	-0.07824448	0.000478901	0.002217236	14	0	0
RNASE8	0.7046791	0.723310914	-0.01863181	-0.03596285	0.000478634	0.002217236	7	0	0
ST8SIA5	0.7109586	0.738627334	-0.02766876	-0.03953686	0.000478881	0.002217236	2	0	0
TMEM241	0.2667778	0.310757267	-0.04397947	-0.0771302	0.000479026	0.002217242	9	0	0
MS4A8	0.429	0.473050396	-0.04405036	-0.06268477	0.000480807	0.002224337	4	0	0
PYGL	0.0872218	0.11262919	-0.02540738	0.02459888	0.000480802	0.002224337	12	0	0
KRTAP10-7	0.7521366	0.800327725	-0.04819109	-0.10035467	0.000481326	0.002226163	4	0	0
FUT10	0.2475956	0.263188892	-0.01559324	-0.13605708	0.000481515	0.002226464	13	0	0
FSTL1	0.3660405	0.329285313	0.036755171	0.635004592	0.000482118	0.002228647	10	0	0
LRRC18	0.6169417	0.545394561	0.07154715	0.200193106	0.000482236	0.002228647	4	0	0
EIF4EBP3	0.2000873	0.158636256	0.041451059	0.367668573	0.000484469	0.002238392	10	0	0
ZNF589	0.4741943	0.498825354	-0.02463109	-0.06062515	0.000485164	0.002241023	8	0	0
FAM71D	0.6561622	0.695761968	-0.03959974	-0.10979936	0.000486691	0.0022475	8	0	0
C15orf48	0.1045599	0.127691292	-0.02313142	-0.1075868	0.000487225	0.002249385	6	0	0
PROP1	0.5571541	0.553381723	0.003772385	0.105781216	0.000488096	0.002252826	9	0	0
CERS1	0.3798348	0.399962168	-0.02012735	0.11765962	0.000488826	0.002255617	9	0	0
THSD7B	0.1526496	0.145250607	0.007399028	0.421865886	0.000489972	0.002259748	5	0	0
INTU	0.2193504	0.210241731	0.009108668	0.24187937	0.000489973	0.002259748	6	0	0
MAP1LC3A	0.815302	0.860747777	-0.0454458	-0.0840082	0.000490323	0.00226078	7	0	0
WHAMM	0.2551791	0.317610736	-0.06243166	-0.21901691	0.00049101	0.002263365	10	0	0
TMEM129	0.1360111	0.16086704	-0.02485596	-0.13883694	0.000491296	0.002264101	7	0	0

HAND1	0.1482633	0.098970658	0.049292603	0.62904862	0.000491934	0.002266458	13	0	0
SORBS3	0.8246078	0.782645228	0.041962587	0.074423141	0.000492095	0.002266618	1	0	0
PELP1	0.227139	0.229790592	-0.00265156	0.072649198	0.000492435	0.002267603	13	0	0
STK31	0.8382803	0.875776765	-0.03749646	-0.06853892	0.000493601	0.00227239	15	0	0
FXYD7	0.1826694	0.224449735	-0.04178037	-0.25835888	0.000493786	0.002272657	12	0	0
LHFPL4	0.4346751	0.47041263	-0.03573754	0.008812814	0.000493989	0.00227301	7	0	0
PCDHB5	0.3858741	0.320538444	0.065335674	0.435971856	0.000494247	0.002273031	8	0	0
CD6	0.4686399	0.539268065	-0.07062812	-0.18375929	0.000494167	0.002273031	11	0	0
LRP8	0.3160423	0.336788937	-0.02074661	-0.16661617	0.000495363	0.002277579	5	0	0
RNF224	0.3979623	0.438733135	-0.04077081	-0.08997201	0.00049801	0.002288573	5	0	0
EIF5A2	0.296272	0.322568056	-0.02629603	-0.14523208	0.000498344	0.002289523	10	0	0
FAM110C	0.1997683	0.17292601	0.026842311	0.465926233	0.000498529	0.002289784	15	0	0
MAK16	0.2696087	0.290786286	-0.02117756	0.070278356	0.000498796	0.002290424	6	0	0
SLC35F3	0.4028053	0.358310394	0.044494922	0.591642169	0.000498963	0.002290608	7	0	0
MAPK15	0.2803253	0.324835092	-0.04450982	-0.04627643	0.000499851	0.002294094	12	0	0
MED4	0.3571468	0.375588971	-0.01844222	-0.08531958	0.000500789	0.002297812	12	0	0
CYP1A1	0.1744887	0.126806348	0.047682313	0.374748579	0.000501006	0.002298221	27	0	0
MYB	0.0424697	0.052042978	-0.00957326	-0.16835588	0.000501745	0.002301021	8	0	0
DUSP28	0.4386074	0.462080992	-0.02347363	-0.10305517	0.000502271	0.002302845	8	0	0
PMS2	0.6156499	0.693527348	-0.07787746	-0.16925096	0.000503339	0.002307154	1	0	0
DMRTA2	0.1603517	0.09551754	0.064834111	0.749608259	0.000504556	0.002310959	13	0	0
C11orf21	0.4282921	0.490502809	-0.06221069	-0.19120858	0.000504437	0.002310959	5	0	0
COL6A2	0.3976736	0.419214952	-0.02154134	0.052293616	0.000504492	0.002310959	5	0	0
COX7A2	0.8584601	0.875045128	-0.01658504	-0.02709042	0.000505401	0.002314239	2	0	0
SFTPA2	0.6624671	0.709204889	-0.0467378	-0.11181481	0.000506512	0.002318735	2	0	0
MED9	0.2903407	0.314676651	-0.02433594	-0.05792806	0.000507453	0.002322449	10	0	0
02-Mar	0.0399506	0.036769943	0.003180693	0.099524193	0.000507627	0.002322654	5	0	0
NTRK1	0.467748	0.464669647	0.00307832	-0.00225212	0.000509205	0.002329278	10	0	0
KLHDC8A	0.6507407	0.717783018	-0.06704233	-0.15379597	0.000509707	0.002330387	10	0	0
KCNMB2	0.8689373	0.906130723	-0.03719346	-0.05979329	0.000509644	0.002330387	1	0	0
CYP3A43	0.7358609	0.819576896	-0.08371595	-0.15713322	0.000510006	0.002330565	4	0	0
MEOX1	0.5025135	0.563426253	-0.06091278	-0.17343645	0.000509906	0.002330565	9	0	0
DTWD2	0.180866	0.22212623	-0.04126019	-0.18814911	0.000510157	0.00233066	6	0	0
C6orf222	0.7215585	0.757173978	-0.03561548	-0.07313757	0.000510304	0.002330738	5	0	0
EMP2	0.2183525	0.176618065	0.041734429	0.272233226	0.000511117	0.002333859	9	0	0
P2RY6	0.4376622	0.463679031	-0.0260168	-0.00283864	0.000512617	0.002340111	11	0	0
CLSTN3	0.2987043	0.260735758	0.037968513	0.105184745	0.000513058	0.002341527	16	0	0
C6orf15	0.7924663	0.820705186	-0.02823887	-0.04767097	0.000515465	0.002351317	4	0	0
IFNB1	0.808263	0.850009364	-0.0417464	-0.08198743	0.000515447	0.002351317	3	0	0
CALHM1	0.6398188	0.684050987	-0.04423219	-0.10854662	0.00051628	0.002354436	11	0	0
WTIP	0.6052987	0.621453263	-0.01615454	0.081624947	0.000517392	0.00235891	3	0	0
TRPV5	0.6611145	0.730338548	-0.06922407	-0.14411886	0.000518192	0.002361954	4	0	0
DDOST	0.2489483	0.280294095	-0.03134581	-0.10298078	0.000518536	0.002362924	8	0	0
PUM1	0.1278782	0.137145702	-0.00926752	-0.10239418	0.000518839	0.002363703	12	0	0
BLOC1S2	0.0710053	0.080956031	-0.0099507	-0.08968402	0.000519192	0.00236471	13	0	0
GJC3	0.5280785	0.568519636	-0.04044115	0.012761348	0.000519344	0.002364803	6	0	0
VN1R1	0.7469048	0.79210491	-0.04520015	-0.08599788	0.000520478	0.002369363	6	0	0
CLTCL1	0.2235505	0.277686079	-0.05413557	-0.15224244	0.000520814	0.002370293	6	0	0
STK17B	0.5872592	0.587072717	0.000186523	-0.03592576	0.000521158	0.002371257	3	0	0
CABP4	0.7071661	0.682636923	0.024529134	0.059488715	0.000521932	0.002373954	8	0	0
ABCC3	0.1879038	0.192524176	-0.00462037	0.260210044	0.000522016	0.002373954	12	0	0
AURKB	0.1699617	0.148015853	0.021945878	0.027652036	0.000523658	0.002380812	17	0	0
C5AR1	0.7456035	0.830897178	-0.08529367	-0.1543	0.000523789	0.002380812	1	0	0
HAO2	0.7163891	0.737018931	-0.02062985	-0.0416355	0.000523985	0.002381096	5	0	0
GIMAP4	0.6601517	0.666408246	-0.00625657	0.003758289	0.000524334	0.002382081	7	0	0
DEFB136	0.732724	0.787817079	-0.05509308	-0.11050944	0.000524811	0.002383645	3	0	0
URI1	0.7571841	0.819844693	-0.06266055	-0.11358962	0.00052497	0.002383761	2	0	0
BICC1	0.2247918	0.190412198	0.034379648	0.532655961	0.00052536	0.00238493	8	0	0
GABRG3	0.2907135	0.247226862	0.043486648	0.521241795	0.000527103	0.002392236	15	0	0
CPAMD8	0.137024	0.112184894	0.024839108	0.45168775	0.000527836	0.002394958	9	0	0
GJB4	0.5947716	0.650634631	-0.05586304	-0.13583552	0.000528569	0.002397678	10	0	0
D2HGDH	0.2010148	0.229791992	-0.0287772	-0.09035995	0.000529337	0.002400555	5	0	0
PPM1A	0.7535007	0.789391622	-0.03589087	-0.07610778	0.000529586	0.002401075	4	0	0

HCRTR2	0.4211981	0.470929272	-0.04973116	0.033052323	0.000529936	0.00240145	9	0	0
DPYSL4	0.1379721	0.099406797	0.038565287	0.554709298	0.000529876	0.00240145	13	0	0
DLG1	0.2666474	0.268032625	-0.00138524	-0.11544774	0.00053035	0.002402112	12	0	0
C4orf51	0.4627718	0.439398466	0.023373352	0.095466986	0.000530266	0.002402112	4	0	0
DOCK1	0.2265556	0.189160481	0.037395082	0.488529182	0.000530757	0.002403347	9	0	0
DYSF	0.2104726	0.19639107	0.014081512	0.478050142	0.000531128	0.002404421	9	0	0
MYO1A	0.7528447	0.819485898	-0.06664121	-0.12959998	0.000533057	0.002412545	5	0	0
CEACAM16	0.7188906	0.75325643	-0.03436583	-0.06584417	0.000536349	0.002426829	8	0	0
NUB1	0.3103911	0.308401179	0.001989968	0.140270439	0.000537494	0.002431137	11	0	0
NTRK3	0.1252541	0.078601313	0.046652802	0.836375938	0.000537572	0.002431137	15	0	0
EVA1A	0.6682103	0.634945961	0.033264381	0.103757253	0.000539449	0.002439013	4	0	0
C16orf74	0.2589248	0.298968472	-0.04004372	-0.18911184	0.000540303	0.002442258	7	0	0
GNAT2	0.7642696	0.736824684	0.02744492	0.076094353	0.00054049	0.002442488	3	0	0
UBE2J2	0.2929043	0.326552992	-0.03364867	-0.05370511	0.000541052	0.002444412	7	0	0
PCOLCE	0.6398293	0.615229097	0.024600165	0.060736491	0.000541792	0.002447135	13	0	0
RFC4	0.1937897	0.23922573	-0.04543607	-0.13420954	0.000542265	0.002448659	10	0	0
NCF2	0.6481374	0.70191199	-0.05377457	-0.14508559	0.000542697	0.002449992	4	0	0
AFAP1	0.3784124	0.354829254	0.023583152	0.264720908	0.000543658	0.002453598	6	0	0
OR1D2	0.6208871	0.672942284	-0.05205514	-0.11250332	0.00054377	0.002453598	5	0	0
POLR1C	0.482321	0.516352156	-0.03403111	-0.0542908	0.000545062	0.002458812	5	0	0
KRT71	0.652266	0.706334574	-0.0540686	-0.11925717	0.00054529	0.002459222	6	0	0
UBFD1	0.1879549	0.213407594	-0.02545273	-0.13626103	0.000547639	0.002469195	14	0	0
GBX1	0.539174	0.534985632	0.004188327	0.054546765	0.000548544	0.002472654	7	0	0
PRSS27	0.3447306	0.396874667	-0.05214409	-0.13708704	0.000550926	0.002482765	9	0	0
RPRD2	0.6111236	0.726659515	-0.11553589	-0.26027353	0.000551599	0.002485175	5	0	0
RASL11B	0.4442678	0.389273691	0.054994078	0.205122441	0.000552184	0.00248656	6	0	0
PCDHA6	0.3606575	0.298935448	0.061722002	0.252292746	0.000552104	0.00248656	11	0	0
CD19	0.7148975	0.746969839	-0.03207234	-0.0567426	0.000553017	0.0024892	8	0	0
SLC14A1	0.6777441	0.739106554	-0.06136241	-0.13981766	0.000553048	0.0024892	5	0	0
ACTL6B	0.3480818	0.355098314	-0.00701648	0.25275916	0.000554018	0.00249294	8	0	0
GALNT16	0.4972106	0.469444215	0.02776634	0.329146855	0.000554228	0.002493263	10	0	0
CYP2B6	0.7742507	0.832958141	-0.05870745	-0.10687918	0.00055617	0.002501372	5	0	0
FAM193A	0.7777329	0.809619058	-0.0318862	-0.0568579	0.000556354	0.002501571	8	0	0
TRIM31	0.7167043	0.792192074	-0.07548778	-0.14724985	0.000556591	0.002502009	5	0	0
SLC17A4	0.6513516	0.709664012	-0.05831237	-0.11198605	0.000556939	0.002502946	3	0	0
ARTN	0.3637981	0.387858789	-0.02406064	-0.00880349	0.000557443	0.002504584	13	0	0
PRPH2	0.8106923	0.865287516	-0.05459523	-0.09837033	0.000557933	0.002505529	8	0	0
ALPK2	0.6831872	0.63206836	0.05111883	0.133371617	0.000557852	0.002505529	10	0	0
CRISPLD2	0.4057047	0.474176055	-0.06847134	-0.18479976	0.000559641	0.00251257	5	0	0
MFSD1	0.1861552	0.122259887	0.063895293	0.883550865	0.000562479	0.00252468	5	0	0
AIFM3	0.2574296	0.246868176	0.010561447	0.377603219	0.000562941	0.002526121	9	0	0
SLC23A2	0.7577636	0.817301964	-0.05953837	-0.10922743	0.000564331	0.002531725	5	0	0
PMP2	0.7276046	0.774305877	-0.04670131	-0.0854783	0.000565602	0.002535525	6	0	0
LONP2	0.2553291	0.312745569	-0.05741649	-0.10168705	0.000565542	0.002535525	11	0	0
DHRS13	0.0647535	0.073882249	-0.00912871	-0.12850136	0.000565439	0.002535525	11	0	0
HS3ST6	0.1881708	0.134882697	0.053288108	0.509812262	0.000566023	0.002536779	11	0	0
CFD	0.4716908	0.521641932	-0.04995112	-0.17311248	0.000568032	0.002545147	7	0	0
TEX37	0.7437468	0.782593019	-0.03884622	-0.07502866	0.000568368	0.002546014	3	0	0
RCN1	0.1596152	0.098992154	0.060623073	0.66514954	0.0005688	0.002547315	26	0	0
SPATS1	0.2702004	0.242922033	0.027278325	0.532421328	0.000570119	0.002552584	8	0	0
EXOC2	0.4499468	0.48346071	-0.03351394	-0.04594271	0.000571475	0.002557588	5	0	0
BMP4	0.8507395	0.878199819	-0.02746036	-0.04512304	0.000571522	0.002557588	3	0	0
ANK3	0.3202481	0.352044532	-0.03179644	-0.11980856	0.00057178	0.002558104	7	0	0
HSPA6	0.365627	0.406398657	-0.04077164	-0.15782601	0.000572547	0.0025609	3	0	0
LSAMP	0.1584963	0.128274524	0.030221794	0.731944672	0.00057322	0.002561994	7	0	0
MRPL34	0.3269009	0.368192917	-0.04129206	-0.1452436	0.000573099	0.002561994	3	0	0
RABEP1	0.1055332	0.112087471	-0.00655427	0.033366002	0.000573712	0.00256322	8	0	0
BOD1L2	0.7375914	0.795711054	-0.0581197	-0.13311198	0.00057378	0.00256322	8	0	0
ATP6V0D2	0.3375743	0.411508019	-0.07393376	-0.27799967	0.000575703	0.002571169	2	0	0
CNTF	0.7771148	0.836046537	-0.05893173	-0.11619554	0.000577701	0.002579451	6	0	0
TUBB	0.2570905	0.291760742	-0.03467021	-0.14816907	0.00057937	0.002586259	17	0	0
XPO6	0.2979119	0.325597888	-0.02768602	0.040526663	0.000580241	0.002589503	10	0	0
KLHL38	0.7422785	0.746291777	-0.00401323	0.030388976	0.000581082	0.00259261	5	0	0

PREB	0.4779678	0.507357296	-0.02938951	-0.07435334	0.000581463	0.002593543	7	0	0
CDH8	0.3182851	0.272536868	0.045748254	0.335810379	0.000581581	0.002593543	9	0	0
SNX25	0.0554253	0.05938266	-0.00395734	-0.08847209	0.000581784	0.002593805	7	0	0
TM4SF4	0.6388782	0.68991072	-0.05103255	-0.1682675	0.000582421	0.002596	5	0	0
LGALS7	0.7295307	0.76597014	-0.03643943	-0.0676965	0.000583192	0.002598791	3	0	0
SLC8A1	0.8544481	0.896931775	-0.04248363	-0.0692146	0.000583874	0.002601181	1	0	0
BCL2L15	0.7504983	0.809456542	-0.05895828	-0.12779195	0.000584147	0.002601753	8	0	0
ZNF395	0.4618386	0.470127934	-0.00828938	0.09026108	0.000585791	0.002608426	2	0	0
CCR9	0.8125634	0.847101073	-0.03453767	-0.05919773	0.000586828	0.002612399	7	0	0
C17orf58	0.2129431	0.260976973	-0.04803392	-0.1510323	0.000588241	0.002618038	5	0	0
COL6A3	0.5934741	0.581205542	0.012268539	0.072569231	0.000588593	0.002618956	6	0	0
STIL	0.0407458	0.040959074	-0.00021323	-0.00765005	0.000588904	0.002619687	10	0	0
CLEC2B	0.3453489	0.362902048	-0.01755316	-0.20576303	0.000589563	0.002620988	5	0	0
SOX5	0.4438885	0.372823042	0.071065486	0.262947766	0.000589781	0.002620988	8	0	0
CST5	0.4986831	0.492472517	0.006210546	0.069144116	0.000589481	0.002620988	5	0	0
WFDC5	0.556877	0.613075504	-0.05619851	-0.16177945	0.000589639	0.002620988	9	0	0
GPR148	0.3131664	0.349342497	-0.03617607	-0.13008293	0.000591187	0.002626225	7	0	0
CRISP1	0.534575	0.600888438	-0.06631342	-0.16854294	0.000591252	0.002626225	8	0	0
CKAP2	0.0796119	0.108421999	-0.02881011	-0.02055001	0.000591724	0.002627671	8	0	0
CRYBB3	0.7179763	0.753147164	-0.03517091	-0.06475633	0.000592896	0.002632223	9	0	0
FFAR3	0.5731842	0.611561745	-0.03837751	0.158432949	0.000593045	0.002632235	4	0	0
C1orf146	0.8313224	0.867066866	-0.03574446	-0.06335525	0.000594162	0.00263654	10	0	0
BTG4	0.298573	0.249865906	0.048707071	0.740273942	0.000594921	0.002639254	11	0	0
OR6S1	0.750721	0.761213175	-0.01049214	0.002029419	0.000595632	0.002641756	3	0	0
SCN8A	0.3599258	0.380454765	-0.02052901	-0.15063111	0.000596488	0.002644897	8	0	0
CD109	0.1453257	0.177123741	-0.03179805	-0.02046006	0.000597063	0.002646791	14	0	0
MAPK4	0.2157476	0.179210584	0.036537065	0.320358247	0.000597937	0.002649357	12	0	0
KCNC3	0.2719925	0.281684342	-0.00969188	-0.05757935	0.000597794	0.002649357	8	0	0
WNT2	0.1884443	0.131643179	0.056801079	0.524510267	0.000598209	0.00264991	13	0	0
ZNF804B	0.1220384	0.089197553	0.032840834	0.58509102	0.000598908	0.002652286	10	0	0
ZNF211	0.7382491	0.768726416	-0.03047731	-0.0569791	0.000599042	0.002652286	2	0	0
HRNR	0.782615	0.84818145	-0.06556643	-0.1158068	0.000599395	0.002653197	4	0	0
CEACAM3	0.4395619	0.493205329	-0.05364346	-0.16975685	0.000599758	0.002654146	6	0	0
COMMD7	0.3665325	0.411136127	-0.04460358	-0.22205106	0.000600218	0.002655527	7	0	0
IRF4	0.1224125	0.059915757	0.062496731	0.943200535	0.000600908	0.002657739	11	0	0
ZFP82	0.2761584	0.208569324	0.067589086	1.224987306	0.000601014	0.002657739	6	0	0
TAF13	0.237211	0.249164341	-0.01195335	-0.01184026	0.00060333	0.002667322	9	0	0
SLC3A2	0.0483428	0.053389446	-0.00504668	-0.06335897	0.000604221	0.002669943	20	0	0
LGALS3BP	0.3728282	0.423762969	-0.05093477	-0.22047906	0.000604147	0.002669943	7	0	0
HUS1	0.2977272	0.352505579	-0.05477838	-0.21136735	0.000605265	0.0026739	5	0	0
SLAMF8	0.5052666	0.519001607	-0.01373499	0.009840383	0.000606244	0.002677564	6	0	0
C6orf132	0.2316553	0.265257038	-0.03360175	-0.19301686	0.000607599	0.002682888	9	0	0
AGBL5	0.8077089	0.869196301	-0.06148738	-0.10459793	0.000608389	0.002685718	1	0	0
UCP3	0.7299813	0.784833179	-0.05485187	-0.1091496	0.000608545	0.002685746	2	0	0
CHMP7	0.4192312	0.47713655	-0.05790538	-0.17597745	0.000610503	0.002693721	3	0	0
TARDBP	0.0672705	0.074014096	-0.00674356	-0.02299758	0.000614601	0.002711138	4	0	0
CPT1C	0.2816098	0.232706531	0.048903279	0.314520071	0.000615919	0.002716284	10	0	0
SYNPO	0.5850261	0.549107996	0.03591809	0.117868739	0.000618344	0.002726309	7	0	0
PCDH12	0.5104557	0.550112324	-0.03965658	-0.09028579	0.00062239	0.002743471	3	0	0
MRPL53	0.5424683	0.576424314	-0.03395603	-0.13206267	0.000622813	0.00274466	7	0	0
FGD2	0.5461508	0.579456378	-0.03330558	-0.11926539	0.000624746	0.002752504	5	0	0
CD200	0.6627445	0.749933474	-0.08718899	-0.1784829	0.000625502	0.002755156	2	0	0
CCDC177	0.3090102	0.266173329	0.042836915	0.642024388	0.000625759	0.002755614	11	0	0
GALNT14	0.7014233	0.781620582	-0.08019729	-0.15492931	0.000626842	0.002759705	3	0	0
SIDT1	0.3671794	0.398879912	-0.03170052	-0.2011005	0.00062711	0.002760209	7	0	0
RAD50	0.7416351	0.804960582	-0.06332546	-0.11513996	0.0006275	0.002761246	2	0	0
ZNF521	0.1950053	0.260334331	-0.06532903	-0.16441147	0.00062809	0.002763166	5	0	0
ZNF197	0.210674	0.160797392	0.049876657	0.684073924	0.000628328	0.002763535	10	0	0
SYT4	0.3644022	0.421462191	-0.05706	0.001642593	0.000630713	0.002773344	9	0	0
GIT1	0.2310097	0.260171688	-0.029162	-0.07082548	0.000631093	0.002774333	4	0	0
HMGCL	0.2256358	0.265713176	-0.04007736	-0.22660938	0.000631461	0.002775271	1	0	0
VIL1	0.6899878	0.708927156	-0.01893933	-0.02436845	0.000631807	0.002776114	4	0	0
PPP3CB	0.2363995	0.229298141	0.007101352	0.032222611	0.000632859	0.002779373	14	0	0

CRYAB	0.6734567	0.623154488	0.050302188	0.139856174	0.000632817	0.002779373	3	0	0
FKBP2	0.1060957	0.129653921	-0.02355824	-0.10700889	0.000634172	0.002784456	12	0	0
PRDM5	0.151119	0.115511618	0.035607373	0.583122956	0.000634514	0.002784561	11	0	0
KEL	0.6553201	0.717822794	-0.06250265	-0.13393249	0.000634661	0.002784561	5	0	0
LILRA2	0.3491303	0.416777797	-0.06764747	-0.26986925	0.000634657	0.002784561	3	0	0
ZBTB43	0.3620409	0.385172271	-0.02313139	-0.12075326	0.000635226	0.002786355	5	0	0
GPRC5D	0.8355047	0.869647919	-0.03414324	-0.05627842	0.000636196	0.00278993	2	0	0
FAM198A	0.3255848	0.352532517	-0.02694773	0.05827148	0.000636919	0.002792417	14	0	0
CASR	0.3465073	0.357738734	-0.01123147	0.27660175	0.000637495	0.002793984	11	0	0
ELAVL3	0.2467395	0.204856058	0.041883395	0.281110083	0.000637588	0.002793984	10	0	0
SLC17A5	0.2053226	0.254038333	-0.04871578	-0.20974432	0.000638104	0.002795563	6	0	0
CPPED1	0.3968454	0.439042581	-0.04219721	0.060036519	0.000640046	0.002803388	6	0	0
MTF2	0.1594372	0.171829124	-0.01239197	-0.04585511	0.000640946	0.002806641	8	0	0
MLC1	0.4392981	0.424740398	0.014557657	0.091542288	0.000642386	0.002812261	13	0	0
POTEE	0.7798226	0.840827643	-0.06100502	-0.10721429	0.000644689	0.002821653	3	0	0
U2SURP	0.1819247	0.123690982	0.058233703	0.59546683	0.000645485	0.002823623	13	0	0
NOL7	0.056541	0.072445022	-0.01590398	-0.1581254	0.000645609	0.002823623	12	0	0
COG5	0.3436623	0.408934883	-0.06527257	-0.1464561	0.000645611	0.002823623	10	0	0
AHNAK	0.2697309	0.288296432	-0.0185655	-0.06168996	0.000645883	0.002824123	3	0	0
TPM2	0.2760019	0.259473959	0.016527984	0.244186959	0.000647024	0.002828425	3	0	0
CPO	0.7075194	0.786902307	-0.07938295	-0.14671399	0.000648104	0.002832455	2	0	0
SEC14L3	0.4444023	0.487180725	-0.04277846	-0.12481844	0.000649434	0.002837575	11	0	0
TK1	0.2008446	0.254905884	-0.05406124	-0.15016698	0.000650938	0.002843456	6	0	0
GHITM	0.3265757	0.382047328	-0.05547167	-0.07072851	0.000652476	0.002848779	10	0	0
C11orf74	0.2975402	0.305783643	-0.00824346	0.159412294	0.00065261	0.002848779	14	0	0
ODF3L2	0.5532034	0.570927638	-0.01772426	-0.03945342	0.000652633	0.002848779	6	0	0
ALOX12	0.52456	0.581754941	-0.05719496	-0.14866846	0.000654181	0.002854841	14	0	0
RGS4	0.4799896	0.509385265	-0.02939568	-0.10481809	0.000655913	0.002861007	11	0	0
CFHR5	0.6167146	0.648446185	-0.0317316	-0.02844286	0.000655793	0.002861007	6	0	0
SHD	0.3669771	0.362196968	0.004780128	0.169265541	0.000658998	0.002873764	5	0	0
CPN1	0.730336	0.722605995	0.007729965	0.012532084	0.000659184	0.002873877	7	0	0
POU5F2	0.8561692	0.88469499	-0.02852581	-0.04910566	0.000659894	0.00287619	10	0	0
SLC6A7	0.408176	0.386879888	0.0212961	0.293895444	0.000660036	0.00287619	6	0	0
ZNF431	0.6133932	0.673222529	-0.05982929	-0.08771217	0.000660394	0.002877052	6	0	0
HIST1H2AA	0.816329	0.867778514	-0.05144948	-0.09246239	0.000660715	0.002877753	10	0	0
OXER1	0.6990228	0.738709316	-0.03968654	-0.08725575	0.000662063	0.002882718	5	0	0
DOPEY2	0.058746	0.061110706	-0.00236474	0.04366729	0.000662177	0.002882718	6	0	0
BBOX1	0.4607794	0.539772794	-0.07899341	-0.19196504	0.000665129	0.002894869	2	0	0
FXR2	0.0630373	0.056900343	0.006136909	0.108083173	0.000665313	0.002894967	11	0	0
DIRAS3	0.6254998	0.582552037	0.042947731	0.114592287	0.000665551	0.002895302	6	0	0
TAF5	0.3586674	0.358635111	3.23E-05	0.06999346	0.000666601	0.002898463	6	0	0
PDE6H	0.8562109	0.89187943	-0.03566853	-0.05892444	0.000666579	0.002898463	4	0	0
CDH12	0.3581533	0.424012097	-0.06585878	-0.02236057	0.000667095	0.002899908	10	0	0
MT3	0.3873024	0.366297791	0.021004625	0.437837778	0.000667706	0.002901862	13	0	0
VCAM1	0.4931211	0.480599622	0.012521478	0.030713778	0.00066807	0.00290274	9	0	0
NPBWR1	0.6054259	0.547769078	0.057656785	0.552236367	0.000671194	0.002915604	4	0	0
VCPKMT	0.2794905	0.25159823	0.027892273	0.10999352	0.00067137	0.002915664	13	0	0
MRAP2	0.2161991	0.248903506	-0.0327044	-0.13807627	0.000671547	0.002915726	8	0	0
CSMD3	0.129334	0.08218331	0.047150732	0.459064579	0.000674663	0.002928545	10	0	0
GRIP1	0.6807729	0.619327143	0.061445781	0.142060432	0.000676529	0.002935938	4	0	0
COPS4	0.2872526	0.331601997	-0.04434939	-0.0826188	0.000677564	0.002939006	11	0	0
LRP12	0.1016794	0.075046455	0.026632972	0.427869109	0.000677507	0.002939006	6	0	0
VDAC3	0.133652	0.163971878	-0.03031989	-0.20896607	0.000677924	0.002939854	9	0	0
LRTM1	0.6764125	0.640282944	0.036129558	0.081748974	0.000678857	0.002943189	5	0	0
LCAT	0.7198721	0.686292743	0.033579337	0.069740789	0.000682276	0.0029573	14	0	0
C5orf38	0.1456462	0.098845347	0.046800821	0.625175726	0.00068444	0.002965963	14	0	0
NKX2-8	0.1905826	0.147250477	0.04333214	0.337174829	0.000684814	0.002966866	12	0	0
PRL	0.7855297	0.780809338	0.004720349	0.006279742	0.000685964	0.002971129	5	0	0
NFIA	0.8314459	0.8832567	-0.05181082	-0.08817167	0.000686598	0.002972011	4	0	0
FGF8	0.3034704	0.260406644	0.043063748	0.634440207	0.000686664	0.002972011	9	0	0
IGLON5	0.1842778	0.163128104	0.021149696	0.236456281	0.000686416	0.002972011	6	0	0
SLC30A8	0.8086234	0.870605497	-0.06198206	-0.11184017	0.000687077	0.002973078	4	0	0
ME1	0.3025476	0.355474752	-0.05292711	-0.13268033	0.000689553	0.002980917	7	0	0

FEZ1	0.3197714	0.280883994	0.038887378	0.26533922	0.000689548	0.002980917	12	0	0
MEGF11	0.2422693	0.1743264	0.067942863	0.410839276	0.0006894	0.002980917	5	0	0
AXIN1	0.3730599	0.407162913	-0.03410304	-0.02888699	0.00068949	0.002980917	6	0	0
RNF149	0.2551535	0.27357807	-0.01842459	-0.168384	0.000690003	0.002982144	7	0	0
INTS4	0.1699076	0.201477918	-0.03157034	0.019087051	0.000690182	0.002982197	5	0	0
STRIP1	0.7648631	0.799017298	-0.03415423	-0.08124489	0.000690461	0.002982686	4	0	0
KRTAP1-1	0.7224051	0.783001298	-0.06059621	-0.11447563	0.000691635	0.002987035	2	0	0
GPR27	0.0844656	0.051713718	0.032751904	0.828641569	0.000691845	0.002987226	12	0	0
GSC2	0.2301135	0.1615891	0.068524403	0.696360953	0.000694036	0.002995964	8	0	0
MOGS	0.2697193	0.314785504	-0.04506621	-0.0986974	0.000694498	0.002996518	11	0	0
DHRS7B	0.8267212	0.878739643	-0.05201847	-0.08701391	0.000694361	0.002996518	1	0	0
BNC2	0.0967369	0.08243845	0.014298443	0.32889988	0.000694963	0.002997801	8	0	0
CCDC152	0.6972347	0.707962531	-0.01072781	0.010630599	0.000695313	0.002998591	11	0	0
KIAA1549L	0.6572339	0.721846251	-0.06461237	-0.1346131	0.000695934	0.003000197	8	0	0
CLLU1OS	0.848837	0.890513874	-0.04167689	-0.06955765	0.000696064	0.003000197	4	0	0
TMEM260	0.4892068	0.553045443	-0.06383861	-0.15725298	0.000696188	0.003000197	3	0	0
AP5S1	0.1828768	0.185650111	-0.00277327	0.001924293	0.000696422	0.003000485	10	0	0
ST6GAL2	0.240691	0.196166325	0.044524694	0.528720499	0.000698492	0.00300868	10	0	0
OR10G2	0.6663321	0.727123467	-0.06079137	-0.12040024	0.000699352	0.003011662	3	0	0
STXBP1	0.3767211	0.418737233	-0.04201609	-0.07020226	0.000700204	0.003014607	6	0	0
BTRC	0.1855729	0.199356596	-0.0137837	0.018911102	0.000701031	0.003016766	10	0	0
BBS2	0.3100289	0.344669511	-0.03464064	-0.18240804	0.000701041	0.003016766	6	0	0
FBXO42	0.1576898	0.19061328	-0.03292352	-0.10236169	0.000702017	0.003018067	8	0	0
ST3GAL3	0.754933	0.786329819	-0.03139682	-0.06789267	0.000701955	0.003018067	8	0	0
KIRREL3	0.2940517	0.299812953	-0.00576124	0.245700384	0.000701684	0.003018067	6	0	0
PLA2G1B	0.6201748	0.653004881	-0.03283004	-0.0300767	0.00070164	0.003018067	5	0	0
EYS	0.7418245	0.786953664	-0.04512917	-0.08644802	0.00070247	0.003018704	2	0	0
CARD14	0.6900621	0.738740712	-0.04867858	-0.12302425	0.000702502	0.003018704	2	0	0
GRXCR2	0.8138678	0.857674374	-0.04380659	-0.07561218	0.00070288	0.003019222	7	0	0
CLEC19A	0.4562867	0.521608621	-0.06532194	-0.21120749	0.000702959	0.003019222	3	0	0
SH2D5	0.0855551	0.093900425	-0.00834535	-0.12980438	0.000704152	0.003023341	13	0	0
WNT3A	0.1331092	0.091885866	0.041223361	0.471673747	0.000704255	0.003023341	9	0	0
KIAA1958	0.171782	0.18146805	-0.00968605	-0.06591536	0.000705462	0.003027797	6	0	0
SH3BGRL2	0.1589395	0.171609014	-0.01266954	0.028609709	0.000706152	0.00302931	5	0	0
MAPRE2	0.2664865	0.234679415	0.031807081	0.08179482	0.000706089	0.00302931	9	0	0
PCDHB4	0.4567625	0.386548486	0.070214006	0.369691443	0.000706576	0.003030402	13	0	0
SLCO3A1	0.1383672	0.167936743	-0.02956953	-0.06469849	0.000707041	0.003031671	5	0	0
CDKL4	0.6534266	0.704998543	-0.05157193	-0.10415558	0.000707418	0.003032445	3	0	0
EIF1	0.0719361	0.087771692	-0.01583554	-0.11270936	0.000707559	0.003032445	3	0	0
OPTC	0.8140556	0.834269035	-0.02021342	-0.02960934	0.000707908	0.003033213	6	0	0
ZNF775	0.0408708	0.044991307	-0.00412048	-0.11707821	0.000708355	0.003034407	10	0	0
CYP2C18	0.6027323	0.660493766	-0.05776146	-0.12506837	0.000708949	0.003036226	9	0	0
PCDHA5	0.5200286	0.456300797	0.063727812	0.175321134	0.000710898	0.003043417	3	0	0
STK4	0.2162074	0.230786282	-0.01457884	-0.07370495	0.000710968	0.003043417	9	0	0
TMEM204	0.5966385	0.566345706	0.030292765	0.099612911	0.000712572	0.003049555	8	0	0
DCDC2C	0.5427162	0.520106477	0.022609699	0.180342387	0.000713639	0.003053393	9	0	0
DCTN2	0.2919671	0.326606253	-0.03463911	-0.14453113	0.00071636	0.003064308	13	0	0
CREB3L3	0.3810687	0.424120024	-0.04305134	-0.17535552	0.000717397	0.003068011	9	0	0
RHAG	0.6331272	0.708729364	-0.07560213	-0.16676713	0.000718632	0.00307203	6	0	0
RPA1	0.2692785	0.274784649	-0.00550616	0.120851598	0.000718851	0.00307203	4	0	0
TCF15	0.2499417	0.16634609	0.083595591	0.885777414	0.00071884	0.00307203	7	0	0
CTRB1	0.7869928	0.820024926	-0.03303213	-0.06235924	0.000719819	0.00307522	10	0	0
ADRM1	0.2175598	0.239119998	-0.02156018	-0.06007093	0.00071994	0.00307522	4	0	0
KCNB1	0.1238567	0.079429795	0.044426913	0.581054102	0.000721014	0.003079073	9	0	0
RBP3	0.6802101	0.744097899	-0.06388781	-0.13134549	0.000722741	0.003085713	7	0	0
SLC38A8	0.5287287	0.542026394	-0.01329768	-0.01939243	0.000723269	0.003087232	6	0	0
PBOV1	0.6921571	0.744858297	-0.05270115	-0.10092303	0.000723636	0.003088065	5	0	0
GCNT3	0.3488457	0.400224204	-0.05137849	-0.19304893	0.000724303	0.003090175	1	0	0
SLC39A14	0.2669145	0.252622555	0.014291942	0.119779724	0.000727374	0.003102539	15	0	0
TBC1D19	0.6467744	0.564066672	0.082707747	0.194179636	0.00072772	0.003103277	1	0	0
C10orf67	0.357587	0.392510371	-0.03492339	-0.12900539	0.0007304	0.003113968	6	0	0
CDPF1	0.6343769	0.703829303	-0.06945241	-0.14988002	0.000731497	0.003117901	4	0	0
SLC3A1	0.8771403	0.915814833	-0.03867451	-0.0620735	0.000732819	0.003122794	3	0	0

WISP1	0.6444137	0.673161312	-0.02874764	-0.051649	0.000734014	0.003127144	7	0	0
SLC26A10	0.4079037	0.435778944	-0.0278752	-0.12317084	0.000734405	0.003128066	20	0	0
KHDC1L	0.7456499	0.812323562	-0.06667365	-0.1212667	0.000735958	0.003133936	4	0	0
LIPT2	0.0429965	0.04973684	-0.00674035	-0.1221119	0.000736355	0.003134884	9	0	0
MXD3	0.229828	0.252714386	-0.02288641	-0.1354284	0.000736985	0.003136819	11	0	0
YWHAZ	0.2254674	0.267833414	-0.042366	-0.11507989	0.000737327	0.00313753	14	0	0
TXNDC9	0.7241685	0.759415029	-0.03524653	-0.06099148	0.000738766	0.003142911	3	0	0
GET4	0.4475386	0.511059608	-0.06352103	-0.01299185	0.000740437	0.003148525	7	0	0
TMEM235	0.60531	0.647307397	-0.04199745	-0.11412182	0.000740424	0.003148525	10	0	0
B3GNT7	0.3163706	0.359212932	-0.04284229	-0.16956187	0.000742343	0.00315588	9	0	0
GCC1	0.75734	0.804172712	-0.04683266	-0.09011669	0.000742672	0.003156533	6	0	0
SLC22A1	0.8266046	0.860357275	-0.03375267	-0.05909769	0.000742877	0.003156654	7	0	0
SH3TC2	0.5275372	0.528090345	-0.00055313	0.043025499	0.000743627	0.003159093	10	0	0
TM4SF18	0.6098718	0.638836928	-0.02896511	-0.04810208	0.000744318	0.003161279	4	0	0
WDR27	0.0317324	0.04182619	-0.01009384	-0.18922429	0.000746482	0.003169722	6	0	0
LCT	0.7823377	0.824124265	-0.04178659	-0.07942561	0.000746705	0.003169917	6	0	0
ATP10B	0.7624391	0.807930951	-0.0454919	-0.08394192	0.000748124	0.00317519	6	0	0
TBX10	0.5831657	0.54653535	0.036630395	0.12514997	0.000752039	0.003191052	6	0	0
SYCN	0.4001756	0.33798458	0.062190995	0.455168636	0.000752294	0.003191376	10	0	0
CSNK2A2	0.4553598	0.478364144	-0.02300433	-0.01608747	0.000754826	0.003201361	2	0	0
IL17RE	0.5061756	0.561259542	-0.05508397	-0.15994898	0.000755287	0.00320256	8	0	0
SLC22A25	0.6983116	0.746864927	-0.0485533	-0.07663074	0.000756214	0.003205732	5	0	0
CLEC1B	0.7738559	0.835560304	-0.06170442	-0.11722978	0.000757054	0.003207775	3	0	0
BANP	0.6305478	0.693953319	-0.06340547	-0.15159232	0.000756922	0.003207775	3	0	0
ALDH1A2	0.8067081	0.857266393	-0.05055826	-0.08665461	0.000757755	0.003209989	1	0	0
HIST1H4J	0.4255493	0.400684168	0.024865143	-0.01749132	0.000758224	0.003210459	4	0	0
ESPL1	0.1886102	0.213375078	-0.02476491	-0.18506997	0.000758153	0.003210459	5	0	0
HACE1	0.4705265	0.521414684	-0.05088816	-0.09943012	0.000761835	0.003224225	9	0	0
PARD3	0.0724164	0.04662279	0.025793655	0.495790194	0.000762238	0.003225169	8	0	0
IFITM1	0.5262939	0.575840295	-0.04954637	-0.13331412	0.000763022	0.003227726	11	0	0
ICOS	0.7145756	0.724141155	-0.00956555	-0.0020825	0.000763823	0.003230354	9	0	0
CMTM7	0.2611875	0.282587589	-0.02140011	-0.1731468	0.000765402	0.003235277	11	0	0
SHISA9	0.1212098	0.075929436	0.045280344	0.637317953	0.000765528	0.003235277	8	0	0
ME2	0.0856406	0.115627031	-0.02998644	-0.13803283	0.00076711	0.003241196	8	0	0
TGIF1	0.6401393	0.619527679	0.020611582	0.124539638	0.000768193	0.003245008	10	0	0
ACSL1	0.2883589	0.315415059	-0.02705612	-0.09921421	0.000769437	0.003249497	8	0	0
KCNJ4	0.1441961	0.140464866	0.003731267	0.22620361	0.000770236	0.003252107	9	0	0
WNK2	0.1959527	0.147416938	0.048535781	0.743211032	0.000771384	0.003256187	8	0	0
IGSF22	0.65698	0.702388636	-0.04540862	-0.09758485	0.00077185	0.003257389	5	0	0
PEX11G	0.4981599	0.559803441	-0.06164352	-0.16575448	0.000774397	0.003267368	6	0	0
KIAA0930	0.2289842	0.252211029	-0.0232268	-0.07334471	0.000775476	0.003271152	7	0	0
HHLA2	0.6811855	0.736959036	-0.05577352	-0.11963118	0.000775744	0.003271514	6	0	0
SARS	0.3907314	0.416865163	-0.0261338	-0.09351257	0.000776516	0.003273999	8	0	0
PRIM2	0.5550458	0.620877007	-0.06583118	-0.17426123	0.000778386	0.00328111	5	0	0
FXYD1	0.54569	0.49101939	0.054670646	0.161719496	0.000779387	0.003284558	9	0	0
NKX1-2	0.1639192	0.116012372	0.047906805	0.419628739	0.000780324	0.003287734	8	0	0
CD34	0.2761813	0.224745101	0.051436199	0.702933049	0.000780818	0.003289045	15	0	0
OR6A2	0.7290341	0.791842368	-0.06280827	-0.11649244	0.000783419	0.003299226	4	0	0
MANSC4	0.6521986	0.740650929	-0.08845236	-0.19207634	0.000783703	0.003299647	6	0	0
GKAP1	0.7993494	0.828915639	-0.02956623	-0.05176307	0.000785201	0.00330363	1	0	0
SLC6A12	0.3591988	0.403368624	-0.04416981	-0.12097844	0.000784929	0.00330363	10	0	0
ZNF611	0.1156488	0.073349352	0.042299426	0.660154683	0.000785143	0.00330363	3	0	0
TIMD4	0.6100676	0.681012352	-0.07094475	-0.17045819	0.000785539	0.003304277	2	0	0
RAB5A	0.188977	0.21408027	-0.02510326	-0.09477085	0.000786498	0.003307533	5	0	0
CFL1	0.7693113	0.817919153	-0.04860783	-0.09537533	0.000787345	0.003310317	5	0	0
RRP7A	0.7807157	0.843537558	-0.06282189	-0.11212284	0.000788476	0.003314298	4	0	0
CEP128	0.3391455	0.412455056	-0.07330961	-0.28629645	0.000789163	0.003316408	4	0	0
MASP2	0.7875688	0.827586998	-0.04001819	-0.07063422	0.000789806	0.003317555	7	0	0
PLEK	0.6219075	0.678746955	-0.05683941	-0.14539113	0.000789688	0.003317555	8	0	0
PDZD9	0.1995779	0.246333465	-0.04675556	-0.15639983	0.000790751	0.003320745	9	0	0
TLR4	0.2452349	0.283795229	-0.03856037	-0.17679941	0.000792299	0.00332647	6	0	0
SGSH	0.4521678	0.417409548	0.034758257	0.095691432	0.000794628	0.00333515	8	0	0
SLC19A1	0.8093387	0.843439281	-0.03410053	-0.05879375	0.000794739	0.00333515	5	0	0

PROX2	0.7637705	0.761392152	0.002378335	0.016950039	0.000795584	0.003337915	7	0	0
PPP1R35	0.1577167	0.182742985	-0.02502628	-0.12879834	0.000797999	0.003347266	12	0	0
SSH1	0.1856143	0.117133333	0.068481	0.483159265	0.000800309	0.003356172	8	0	0
CA6	0.805098	0.848280058	-0.04318203	-0.0754095	0.000801582	0.003360722	5	0	0
CXADR	0.1059778	0.142313331	-0.03633555	-0.22566839	0.000801936	0.003361423	4	0	0
APOA5	0.6812467	0.696441302	-0.01519459	-0.02385549	0.000802279	0.003362072	8	0	0
LIN7B	0.3070828	0.335278071	-0.0281953	-0.06787772	0.000802628	0.00336275	9	0	0
C17orf78	0.882943	0.895838068	-0.01289509	-0.02079072	0.000803045	0.003363713	7	0	0
B3GALT2	0.4668608	0.415510171	0.051350588	0.252194597	0.000804351	0.003368397	6	0	0
TPD52L3	0.7555209	0.803221248	-0.04770034	-0.09622749	0.000805225	0.00337127	8	0	0
SCG5	0.6005492	0.538643516	0.06190567	0.213586489	0.000806015	0.003373791	7	0	0
BCL9	0.162224	0.113928153	0.04829587	0.456437943	0.000806317	0.003374266	8	0	0
SLC7A4	0.4607906	0.438872663	0.021917906	0.401228033	0.000807024	0.003376435	11	0	0
DMRT1	0.1362329	0.084689615	0.051543286	0.628300623	0.000807424	0.003377321	8	0	0
SH3YL1	0.7295349	0.810496038	-0.08096109	-0.14611897	0.000808466	0.00338089	3	0	0
MRPL24	0.1923929	0.222869903	-0.03047705	-0.13324263	0.00081159	0.003392393	12	0	0
NAPG	0.2577323	0.287862973	-0.03013065	-0.13103079	0.000811594	0.003392393	11	0	0
NPM3	0.2643263	0.294486419	-0.03016012	-0.03950367	0.000813596	0.003399967	10	0	0
PAQR9	0.2358074	0.184521588	0.051285844	0.548767796	0.000814357	0.003401643	14	0	0
MMP3	0.8282536	0.895242185	-0.06698862	-0.11091711	0.000814376	0.003401643	1	0	0
IQCF2	0.6628094	0.734849338	-0.07203989	-0.14873846	0.000815236	0.003404441	2	0	0
PLSCR5	0.7274541	0.809690405	-0.0822363	-0.15252585	0.000817887	0.003414718	1	0	0
CABP2	0.6930231	0.747313042	-0.05428994	-0.11044107	0.00081899	0.003418528	11	0	0
CPXM1	0.1833725	0.104540313	0.078832143	1.159356743	0.000819315	0.003419086	13	0	0
SLC30A2	0.1636816	0.12352146	0.040160138	0.556175038	0.000820003	0.00342116	11	0	0
SNX32	0.0832573	0.044563378	0.038693875	0.826736048	0.000820575	0.003422752	12	0	0
HOMER2	0.0821384	0.108852931	-0.02671457	-0.14100542	0.000821983	0.003426235	10	0	0
UPF2	0.1515014	0.189750397	-0.03824895	-0.15760615	0.000822245	0.00342653	12	0	0
OR10A2	0.6547469	0.704883842	-0.05013692	-0.11289545	0.000822869	0.003428332	3	0	0
LOXL3	0.5255736	0.481265762	0.04430784	0.361484975	0.00082466	0.003434998	4	0	0
DCAF4L1	0.8186096	0.810497315	0.008112265	0.019478532	0.000828211	0.00344899	7	0	0
KCNJ9	0.3302943	0.371293556	-0.04099929	-0.1037645	0.000830986	0.003459741	7	0	0
TRAM1	0.323098	0.363303214	-0.04020523	-0.13700137	0.00083266	0.003465907	5	0	0
STMN4	0.6001035	0.66926842	-0.06916493	-0.15892441	0.000833001	0.003466523	5	0	0
CSF3	0.4117815	0.379227853	0.032553614	0.146335443	0.000834233	0.003470842	6	0	0
ADIPOR2	0.8889518	0.910773765	-0.02182196	-0.0345431	0.000835428	0.003474202	2	0	0
FOXB1	0.1668088	0.104132209	0.062676636	0.727490047	0.000835342	0.003474202	11	0	0
SLC25A47	0.7462455	0.759817981	-0.01357253	-0.01415924	0.000836295	0.003477004	7	0	0
BTNL8	0.7905849	0.851466328	-0.06088147	-0.1152011	0.00083663	0.00347759	6	0	0
TIMP4	0.6821099	0.713658833	-0.03154889	-0.07459981	0.000837512	0.003480447	6	0	0
ZNF213	0.1759953	0.184509884	-0.00851456	-0.04752048	0.00083789	0.003481213	8	0	0
CCL20	0.631847	0.692395763	-0.06054875	-0.14089621	0.000841148	0.003492443	6	0	0
DLK2	0.5041903	0.602540774	-0.09835044	-0.22118603	0.000841177	0.003492443	6	0	0
NPR2	0.5619709	0.507951677	0.054019176	0.185511033	0.000840953	0.003492443	5	0	0
TPPP3	0.292322	0.255645903	0.036676086	0.616295284	0.000842445	0.003496899	15	0	0
TRIM67	0.2263409	0.180764692	0.045576255	0.292708038	0.000843496	0.00349964	9	0	0
WWC2	0.1227085	0.090470854	0.032237667	0.410912758	0.000843453	0.00349964	7	0	0
GABRD	0.3621314	0.334158085	0.027973305	0.375162723	0.000844199	0.003500037	6	0	0
NECAB1	0.3849146	0.332436767	0.052477798	0.248950041	0.000844372	0.003500037	8	0	0
OPA3	0.188509	0.175363215	0.013145812	0.305094544	0.000844095	0.003500037	10	0	0
GPR4	0.188509	0.175363215	0.013145812	0.305094544	0.000844095	0.003500037	10	0	0
BPNT1	0.4335629	0.479362561	-0.04579967	-0.15193301	0.000844642	0.003500347	8	0	0
PTPRH	0.6269024	0.683903279	-0.05700089	-0.12255809	0.000844886	0.00350055	6	0	0
ATF4	0.1735648	0.187552314	-0.0139875	-0.14487961	0.00084669	0.003507212	8	0	0
CCDC80	0.744568	0.802420079	-0.05785204	-0.10915676	0.000847201	0.003508092	2	0	0
PCGF6	0.3375647	0.346326779	-0.00876213	0.043980469	0.000847293	0.003508092	9	0	0
DOK5	0.2407847	0.213173649	0.02761101	0.691825608	0.000849365	0.003515859	7	0	0
PDE11A	0.7248801	0.782136241	-0.05725611	-0.12142695	0.000850917	0.003521469	8	0	0
OR10H2	0.6028059	0.682724677	-0.07991874	-0.17916388	0.000851156	0.003521644	2	0	0
ZNF518B	0.195771	0.228066784	-0.03229574	-0.18978241	0.000852598	0.003526797	12	0	0
TMEM8B	0.5028415	0.466535981	0.036305476	0.12970139	0.00085358	0.003530048	6	0	0
TLR2	0.6437149	0.702964048	-0.0592491	-0.12516765	0.000854441	0.003532793	1	0	0
GIPR	0.2078076	0.226941004	-0.01913345	0.011171638	0.000854721	0.003533136	5	0	0

BCL2	0.0781963	0.099776337	-0.02158001	-0.17405868	0.000856144	0.003538203	15	0	0
EIF4E1B	0.2074132	0.155342058	0.052071135	0.463339685	0.000857548	0.003542371	12	0	0
GJB6	0.1647919	0.155354075	0.009437839	0.535684698	0.000857469	0.003542371	9	0	0
RERE	0.0447142	0.041525318	0.003188837	0.095937633	0.000859464	0.00354947	7	0	0
IL10RA	0.2195439	0.249398159	-0.02985423	-0.04004829	0.000860076	0.003551026	8	0	0
R3HDML	0.5192329	0.542442867	-0.02320995	0.008485778	0.000860237	0.003551026	6	0	0
NOL12	0.6817313	0.65907049	0.022660782	0.051645142	0.00086092	0.00355303	4	0	0
IGFBP7	0.0718792	0.037925595	0.03395358	0.771441758	0.000861323	0.003553874	10	0	0
C5orf15	0.3082263	0.322616347	-0.01439008	0.037471036	0.000862855	0.003559377	11	0	0
THG1L	0.2461198	0.246192947	-7.32E-05	0.075591035	0.000863817	0.003562527	5	0	0
SLC9A9	0.4173241	0.466522255	-0.04919818	-0.22433823	0.00086524	0.003567185	6	0	0
FBN3	0.0480542	0.019970443	0.028083804	0.953861206	0.000865345	0.003567185	1	0	0
SPON1	0.606563	0.570877244	0.035685805	0.181111864	0.000866219	0.003569967	9	0	0
LYZ	0.6598416	0.705956111	-0.0461145	-0.09605115	0.000867197	0.003573178	1	0	0
GPR20	0.2792185	0.297350794	-0.01813225	0.101334995	0.000867944	0.003575436	7	0	0
IPO5	0.6085046	0.661555083	-0.05305045	-0.13664785	0.000868605	0.003577338	5	0	0
TM6SF1	0.2389868	0.210766342	0.028220481	0.798059471	0.000869169	0.003578839	7	0	0
IL17RD	0.2787726	0.25115814	0.027614484	0.093959309	0.000874143	0.003595191	7	0	0
PPP4R2	0.5142249	0.543098628	-0.02887375	-0.02585352	0.000873985	0.003595191	6	0	0
RELN	0.112995	0.075373218	0.037621758	0.593853595	0.000873664	0.003595191	14	0	0
VSTM5	0.3721354	0.404662717	-0.03252729	0.036571295	0.000873457	0.003595191	6	0	0
CEACAM5	0.3111321	0.328998693	-0.01786661	-0.06733841	0.000874111	0.003595191	6	0	0
LY6G6F	0.8142074	0.836834877	-0.02262752	-0.03972329	0.000876785	0.003605229	6	0	0
PRX	0.6316111	0.592460216	0.039150933	0.105118544	0.000877411	0.003606978	5	0	0
FAM50B	0.5043765	0.534950422	-0.03057394	-0.08856456	0.000878206	0.00360942	26	0	0
DNM1L	0.4725667	0.498128813	-0.0255621	-0.10321283	0.00087943	0.003613621	7	0	0
TBC1D28	0.6051051	0.696968134	-0.09186303	-0.20081225	0.000880854	0.003618642	1	0	0
CRABP1	0.3912947	0.367333964	0.023960741	0.346698772	0.000881691	0.003621254	20	0	0
GPRC6A	0.7773941	0.8075468	-0.03015266	-0.05371692	0.000883442	0.003627614	3	0	0
PIWIL1	0.6865347	0.743372116	-0.05683739	-0.1210932	0.000883796	0.003628238	8	0	0
RASSF7	0.410172	0.431192704	-0.02102073	-0.05869316	0.000886353	0.003637519	8	0	0
CHRNB1	0.3570486	0.347080343	0.009968234	0.348230424	0.000886463	0.003637519	14	0	0
CNN3	0.1364346	0.098299266	0.03813529	0.393771226	0.000888661	0.003645704	12	0	0
STK32B	0.0976488	0.058021724	0.039627107	0.672581512	0.000890522	0.003652505	13	0	0
UBE2V1	0.2129982	0.230132892	-0.01713474	-0.0010557	0.000890798	0.003652802	8	0	0
DNASE2B	0.7741516	0.820563622	-0.04641198	-0.08199533	0.000894876	0.003668685	7	0	0
GAL3ST2	0.6483517	0.710960677	-0.06260896	-0.13652811	0.000897185	0.003677312	7	0	0
GLTPD2	0.5690621	0.605526483	-0.03646436	-0.07019436	0.000898173	0.00368052	9	0	0
C1orf52	0.409536	0.350242386	0.059293627	0.179860711	0.000898572	0.003681315	5	0	0
EXOC1	0.3065237	0.351824737	-0.04530108	-0.02865854	0.000902278	0.003693964	13	0	0
SLC15A4	0.1475553	0.181656871	-0.03410155	-0.16752158	0.000901928	0.003693964	9	0	0
F10	0.5337879	0.535923551	-0.00213568	0.022046568	0.000902117	0.003693964	11	0	0
PSD4	0.0988057	0.12359369	-0.02478796	-0.15061785	0.000902643	0.003694616	8	0	0
CLIP2	0.0551165	0.059699787	-0.00458333	-0.01703006	0.000903429	0.003696146	7	0	0
ASAH2B	0.3314627	0.356634469	-0.02517173	0.134232029	0.000903314	0.003696146	2	0	0
SLIT3	0.1778022	0.123114096	0.054688131	0.663625138	0.000905688	0.003702008	16	0	0
SFXN4	0.1291631	0.138089148	-0.00892608	-0.03867519	0.000905586	0.003702008	11	0	0
ANO3	0.4874179	0.517009177	-0.02959133	-0.08067077	0.000905449	0.003702008	4	0	0
ZNF430	0.2296849	0.267513526	-0.03782858	0.100158019	0.000905319	0.003702008	3	0	0
TANK	0.2970468	0.22490448	0.072142345	0.699567348	0.000906037	0.003702594	5	0	0
MIF4GD	0.1273214	0.154015839	-0.02669439	-0.07329657	0.000907275	0.003706806	8	0	0
MOB2	0.827484	0.808649325	0.01883465	0.036471208	0.000907777	0.003708012	5	0	0
HEATR5A	0.0698435	0.07184807	-0.00200462	-0.03238029	0.000909318	0.003713462	8	0	0
C17orf50	0.7744205	0.802563411	-0.02814291	-0.04275393	0.000909928	0.003715108	4	0	0
SLC35G2	0.234607	0.186337748	0.048269294	0.597384828	0.000912048	0.003722915	12	0	0
SPDYC	0.7051703	0.752845214	-0.04767487	-0.08216007	0.000912431	0.003723631	11	0	0
ABI3	0.4511801	0.409835506	0.041344627	0.227996185	0.000919106	0.003750018	14	0	0
TLL1	0.1821408	0.123720764	0.058420041	0.673262304	0.000920118	0.003753293	8	0	0
CCDC13	0.32849	0.327314273	0.001175718	0.496599624	0.000921669	0.003758364	11	0	0
MIP	0.3527019	0.401679092	-0.04897723	-0.13317887	0.00092178	0.003758364	10	0	0
ADCY10	0.8233805	0.8530649	-0.02968437	-0.06029858	0.000922228	0.003758481	5	0	0
DAPL1	0.4570906	0.507465929	-0.05037535	-0.15303952	0.000922203	0.003758481	8	0	0
MOV10L1	0.6492358	0.619792911	0.029442896	0.138919089	0.000923232	0.00376172	11	0	0

LECT2	0.8254141	0.863302457	-0.03788835	-0.06401301	0.000924556	0.003766004	2	0	0
TWIST1	0.1465103	0.08738102	0.059129322	0.638772817	0.000924704	0.003766004	22	0	0
AGAP2	0.4588735	0.506068391	-0.0471949	-0.21599598	0.000925665	0.003769066	8	0	0
SLC17A2	0.7638137	0.772159297	-0.00834562	-0.01444543	0.000926229	0.003770505	8	0	0
NAP1L4	0.1438992	0.159945672	-0.01604645	-0.07885485	0.000926974	0.003772683	22	0	0
SYT2	0.185989	0.136104879	0.049884127	0.758791733	0.000927823	0.003774423	12	0	0
SCGB2A1	0.7000398	0.778504805	-0.07846504	-0.15652979	0.000927652	0.003774423	4	0	0
RIPPLY3	0.5388313	0.489781427	0.049049888	0.241904576	0.000929264	0.003779429	9	0	0
ENPP6	0.6167826	0.653637951	-0.03685531	-0.10007367	0.000930849	0.003785016	2	0	0
SLC35D2	0.218392	0.196501808	0.021890181	0.531450888	0.000932028	0.003788091	8	0	0
SCP2D1	0.6996473	0.758045565	-0.05839829	-0.11287664	0.000931821	0.003788091	7	0	0
CRYBB1	0.6545987	0.706278529	-0.05167988	-0.14178507	0.000933438	0.003792964	6	0	0
METAP1D	0.313233	0.363951305	-0.05071834	-0.17231575	0.000934596	0.003796354	6	0	0
MED12L	0.0944834	0.109965527	-0.01548215	-0.16714358	0.000934696	0.003796354	5	0	0
ETV5	0.2525419	0.231550667	0.020991184	0.315824604	0.000934967	0.003796599	5	0	0
C1orf100	0.7759532	0.821412528	-0.04545929	-0.08747225	0.000936957	0.00380373	5	0	0
PDE8A	0.2123035	0.218779391	-0.00647592	-0.08411007	0.000937148	0.00380373	9	0	0
RALGPS2	0.3114629	0.333038673	-0.02157576	-0.12321762	0.00093822	0.003807222	8	0	0
C10orf90	0.6650515	0.715021805	-0.04997033	-0.11663564	0.000939408	0.003811177	4	0	0
ETV1	0.2505039	0.219697343	0.030806563	0.163281921	0.000941237	0.00381601	14	0	0
OR1F1	0.4520586	0.495840483	-0.04378191	-0.16757169	0.000941151	0.00381601	7	0	0
MISP	0.5860737	0.587918545	-0.00184482	-0.0158239	0.000941059	0.00381601	8	0	0
CDRT15L2	0.6884888	0.762674867	-0.0741861	-0.14562455	0.000942165	0.003818909	1	0	0
SLC22A24	0.6970224	0.773517548	-0.07649516	-0.14856544	0.000943961	0.003825323	2	0	0
YLPM1	0.2611745	0.286946864	-0.02577232	-0.02025246	0.000944754	0.003827674	11	0	0
XPA	0.337469	0.362690108	-0.02522111	-0.08967881	0.000945322	0.00382911	4	0	0
SDK1	0.1823146	0.149085029	0.033229569	0.194994447	0.000945997	0.00383098	4	0	0
SLC2A10	0.2601519	0.292195949	-0.03204404	0.251668718	0.000947259	0.003835224	10	0	0
GREM2	0.2803829	0.33228976	-0.0519069	-0.05575142	0.00094758	0.003835658	7	0	0
ANKRD55	0.6685118	0.732651213	-0.06413946	-0.12916547	0.000949044	0.003840714	9	0	0
TAS2R8	0.738517	0.804647554	-0.06613059	-0.13002852	0.0009497	0.003842504	6	0	0
DNAH12	0.319913	0.329448218	-0.00953523	0.054044422	0.000950206	0.003843684	11	0	0
TKT	0.218525	0.234667735	-0.01614273	-0.0527489	0.000950473	0.003843897	7	0	0
CYP24A1	0.1765962	0.113783164	0.062813031	0.767827649	0.000952894	0.003852818	11	0	0
RFX2	0.0437192	0.037345509	0.006373709	0.177532965	0.000953294	0.003853569	2	0	0
KIF6	0.342592	0.380394992	-0.037803	0.154706365	0.000954126	0.003856061	10	0	0
HIST1H4D	0.2565066	0.259152867	-0.00264625	0.227880982	0.000959204	0.003875711	9	0	0
LIN28B	0.2178352	0.1382507	0.079584484	0.638446051	0.000959965	0.003877913	9	0	0
UBTD2	0.0392218	0.033973765	0.005247996	0.134792985	0.000961219	0.003882105	10	0	0
KRTAP10-9	0.5894755	0.666520141	-0.07704465	-0.17828714	0.000961485	0.003882304	3	0	0
SLC44A5	0.6532638	0.730215764	-0.076952	-0.1568119	0.000962978	0.003885709	4	0	0
KMO	0.0791752	0.091722248	-0.01254703	-0.18960676	0.000962885	0.003885709	7	0	0
SYT10	0.3013592	0.258940158	0.042419014	0.356126963	0.00096281	0.003885709	7	0	0
C4orf36	0.1917288	0.212391932	-0.02066316	-0.14622652	0.000964103	0.003889371	6	0	0
PDIA6	0.520071	0.603426691	-0.08335573	-0.19806427	0.000964641	0.00389067	4	0	0
ACTL10	0.5547717	0.57419898	-0.01942731	0.073347533	0.000965132	0.003891773	5	0	0
PRH2	0.8730944	0.911364886	-0.03827053	-0.06120503	0.000965604	0.003892801	1	0	0
SLC19A3	0.2021204	0.172933876	0.029186571	0.335563436	0.000966856	0.003895221	10	0	0
ANKUB1	0.5705101	0.686146328	-0.11563619	-0.26401684	0.000966652	0.003895221	4	0	0
KCNE2	0.770973	0.817390824	-0.0464178	-0.0909099	0.000966473	0.003895221	4	0	0
KRTAP10-2	0.641265	0.670371054	-0.02910602	-0.04924014	0.000970059	0.003907247	3	0	0
FXYD6	0.4274302	0.429736417	-0.00230621	0.068907478	0.000970358	0.003907574	11	0	0
OR13F1	0.7421078	0.797998595	-0.05589077	-0.10506579	0.000970839	0.003907758	5	0	0
RYR1	0.2388428	0.214565167	0.024277625	0.474451673	0.000970684	0.003907758	6	0	0
OR13J1	0.7028709	0.754989051	-0.05211811	-0.09785891	0.000971694	0.003910322	3	0	0
TCL1A	0.4271247	0.400211112	0.026913546	0.175532298	0.000972389	0.003912241	11	0	0
SPDYE4	0.7503743	0.805191505	-0.0548172	-0.10340095	0.000974474	0.003919751	6	0	0
BZW1	0.3570798	0.370928332	-0.01384851	-0.14999053	0.000975267	0.003922062	2	0	0
LRRTM1	0.2241506	0.202432865	0.021717734	0.465531881	0.000975584	0.003922458	15	0	0
EDAR	0.447218	0.502171862	-0.0549539	-0.19789888	0.000976393	0.00392483	8	0	0
A3GALT2	0.6994355	0.742651006	-0.04321555	-0.08548206	0.000976964	0.003926244	7	0	0
ZC3HAV1	0.1475306	0.181520666	-0.03399009	-0.0649367	0.00097719	0.003926274	12	0	0
GFY	0.2256067	0.171437864	0.054168811	0.388048554	0.000977692	0.003927408	14	0	0

TMEM218	0.3087588	0.323344292	-0.01458549	-0.1037647	0.000979202	0.003932594	10	0	0
SOX8	0.1088923	0.054397273	0.054495003	0.819118055	0.000979978	0.003934832	10	0	0
SBSPON	0.6300777	0.69992549	-0.06984774	-0.1475196	0.000981161	0.003938697	3	0	0
NAT16	0.217497	0.173438053	0.04405893	0.36066434	0.000981508	0.00393921	9	0	0
LAMA2	0.374388	0.34345622	0.030931818	0.313485022	0.00098226	0.003941345	7	0	0
UPK3A	0.2126148	0.240295024	-0.02768021	-0.2070223	0.000982892	0.003942999	7	0	0
PPP1R9A	0.6612394	0.683720373	-0.02248102	-0.02169136	0.000984782	0.003949696	7	0	0
CDH10	0.2899389	0.320491205	-0.03055232	0.096795178	0.000986686	0.003956449	11	0	0
ADRA2B	0.177313	0.183614746	-0.00630173	0.082052545	0.000988454	0.003958574	10	0	0
SCAP	0.9310601	0.939171813	-0.00811174	-0.01238243	0.000988163	0.003958574	1	0	0
TDO2	0.7373478	0.76680668	-0.02945884	-0.06761809	0.00098854	0.003958574	4	0	0
PLEKHG1	0.3986385	0.438170844	-0.03953237	-0.17449039	0.000988339	0.003958574	6	0	0
LPAR6	0.8330995	0.854394076	-0.02129462	-0.03637922	0.000987887	0.003958574	5	0	0
ATP1B2	0.8058385	0.755145863	0.050692613	0.092548748	0.000988176	0.003958574	1	0	0
PLA2G4D	0.6819968	0.73181369	-0.04981688	-0.10780862	0.000992271	0.003972629	7	0	0
HHAT	0.5117365	0.528868412	-0.01713195	-0.10188081	0.000995151	0.003983269	8	0	0
FGR	0.5251387	0.456605918	0.068532804	0.25979264	0.000996034	0.003985915	9	0	0
CACNB3	0.470118	0.503616372	-0.03349835	-0.04202394	0.000999076	0.003997195	5	0	0
EPB41	0.1917557	0.215779631	-0.02402396	-0.05777872	0.001000017	0.003998285	11	0	0
B3GAT2	0.2288201	0.219504936	0.009315155	0.377070926	0.00099966	0.003998285	10	0	0
ZNF347	0.2083184	0.161703787	0.046614584	0.889270217	0.000999994	0.003998285	7	0	0
ATP1B1	0.3742765	0.40854949	-0.034273	-0.07035145	0.001000568	0.003999597	4	0	0
KRTAP4-7	0.6923054	0.771605259	-0.07929988	-0.15434168	0.00100093	0.004000152	1	0	0
RASAL3	0.1901698	0.208165851	-0.01799608	-0.02521576	0.00100163	0.004002059	8	0	0
KNDC1	0.1241552	0.107606697	0.016548543	0.348335381	0.001002177	0.004002458	9	0	0
PABPC1L	0.1289125	0.139737368	-0.01082487	-0.16751316	0.001002168	0.004002458	11	0	0
TMEM159	0.2778652	0.31506544	-0.03720024	-0.10008872	0.001002953	0.004004666	9	0	0
ADAMTS12	0.2323891	0.174294116	0.058095031	0.588184588	0.001003897	0.004007544	13	0	0
ZNF251	0.8530531	0.892498806	-0.03944569	-0.06515053	0.001004274	0.004008157	2	0	0
F2R	0.1911953	0.160171609	0.031023657	0.709606347	0.001004558	0.0040084	17	0	0
IGSF21	0.3465178	0.334391277	0.012126494	0.327610664	0.001004997	0.004008491	8	0	0
CELF2	0.6586522	0.721386555	-0.06273436	-0.11969062	0.001005028	0.004008491	9	0	0
DOK2	0.5346855	0.577771377	-0.04308592	-0.11906926	0.001005599	0.004009021	9	0	0
KCNE3	0.4242541	0.379536771	0.044717338	0.343763021	0.001005608	0.004009021	11	0	0
C16orf54	0.4985116	0.570334047	-0.07182242	-0.19736818	0.001006902	0.004012395	10	0	0
PITPNA	0.3316584	0.368932265	-0.03727384	-0.05623577	0.001006892	0.004012395	10	0	0
HSD3B2	0.6852881	0.745837789	-0.06054969	-0.13119941	0.001007632	0.004014412	7	0	0
SLC38A10	0.1200601	0.150056247	-0.02999619	-0.05893266	0.001009433	0.004019801	9	0	0
FBXO27	0.1916766	0.244700338	-0.05302371	-0.22090145	0.001009225	0.004019801	6	0	0
ASAP3	0.218436	0.222686809	-0.00425083	0.080299955	0.001010141	0.004021728	10	0	0
H1FNT	0.7818607	0.833129446	-0.05126875	-0.09571919	0.001012841	0.004030688	8	0	0
CHADL	0.8107455	0.8434387	-0.03269316	-0.05795802	0.001012719	0.004030688	6	0	0
KLHL29	0.4949145	0.510564647	-0.01565014	-0.05220495	0.0010132	0.004031225	7	0	0
TMEM41B	0.1079239	0.115461182	-0.0075373	-0.08413918	0.001015684	0.00404021	12	0	0
CLDN4	0.5386489	0.61296659	-0.07431765	-0.19710826	0.00101659	0.004042918	2	0	0
GBP4	0.1142002	0.147734072	-0.03353387	-0.29666747	0.001018133	0.004046361	9	0	0
POU2AF1	0.5438784	0.612431939	-0.06855356	-0.16834687	0.001018068	0.004046361	1	0	0
LHX2	0.0907273	0.066605945	0.024121394	0.302161359	0.001018851	0.00404832	2	0	0
ENDOG	0.4220901	0.476450134	-0.05436001	-0.08033116	0.00101928	0.004049128	6	0	0
CIT	0.2368237	0.247238265	-0.01041452	-0.10304155	0.001019624	0.004049594	10	0	0
EBLN1	0.5808251	0.645948769	-0.06512365	-0.1536222	0.001022531	0.004059346	2	0	0
RND3	0.6439728	0.657619521	-0.01364673	-0.01971343	0.001023396	0.004061878	3	0	0
ITGA6	0.1341516	0.143885831	-0.00973424	-0.09756623	0.001025346	0.004068718	12	0	0
PTPRO	0.3100504	0.30551846	0.004531967	0.382654281	0.001027494	0.004075942	11	0	0
ZNF530	0.3499043	0.367570323	-0.01766604	0.034449784	0.001027621	0.004075942	11	0	0
SH3PXD2B	0.1887115	0.162116901	0.026594643	0.193561438	0.001028591	0.004078076	11	0	0
DEFB113	0.6959461	0.760864359	-0.06491828	-0.15441745	0.001028614	0.004078076	3	0	0
MRPL38	0.613201	0.641667432	-0.02846642	-0.07935195	0.00102947	0.004080567	6	0	0
PPP1R3B	0.2139903	0.26707169	-0.05308138	-0.26413304	0.00103135	0.004086213	8	0	0
FSD1L	0.4310545	0.470355061	-0.03930059	-0.02596346	0.001031203	0.004086213	7	0	0
VPS52	0.0897616	0.109218524	-0.01945692	-0.142712	0.001031604	0.004086319	21	0	0
TNFRSF11A	0.1207606	0.13910549	-0.01834489	-0.07937298	0.00103307	0.004091221	10	0	0
ABCC8	0.2097392	0.17215516	0.037584004	0.550277718	0.001035706	0.004100754	11	0	0

UHRF2	0.1996554	0.177620223	0.022035183	0.184821934	0.001037122	0.004105455	5	0	0
TLDC1	0.6645232	0.734570702	-0.07004752	-0.15604418	0.001037835	0.004107373	3	0	0
ENKD1	0.1376514	0.066504451	0.071146922	1.434042408	0.001038748	0.004110079	9	0	0
KDM6B	0.5656866	0.624493672	-0.05880705	-0.13876341	0.001039223	0.004111051	7	0	0
PGLYRP4	0.5500522	0.600677977	-0.05062576	-0.18986628	0.001043492	0.004123391	3	0	0
CADM3	0.240533	0.247508729	-0.00697576	0.185924459	0.001043118	0.004123391	12	0	0
SPINK7	0.622574	0.692772085	-0.07019813	-0.152567	0.001043351	0.004123391	4	0	0
DEGS2	0.4589558	0.484995096	-0.02603928	0.007555982	0.00104278	0.004123391	10	0	0
LIG1	0.3937001	0.371863706	0.021836381	0.081240917	0.001043366	0.004123391	13	0	0
BIRC7	0.4659793	0.487454578	-0.02147529	-0.0460759	0.001044275	0.004125577	9	0	0
UBE2V2	0.2331984	0.282562229	-0.04936382	-0.12546991	0.001045676	0.004130202	11	0	0
OR4K1	0.6718365	0.741973824	-0.07013737	-0.1334079	0.001049684	0.004144599	3	0	0
DHDH	0.091055	0.100829683	-0.00977464	-0.07174481	0.001049783	0.004144599	11	0	0
SYNE1	0.3932145	0.428765792	-0.0355513	-0.00543293	0.001050919	0.004148168	12	0	0
TAGAP	0.3854358	0.45362245	-0.06818662	-0.27103485	0.001051387	0.004149106	6	0	0
MYLK4	0.7063406	0.767108067	-0.06076747	-0.12629505	0.001051987	0.004150559	5	0	0
CYP26C1	0.6692971	0.625830134	0.043466984	0.100702604	0.001054258	0.004158605	8	0	0
TRIM13	0.3609048	0.424421267	-0.06351651	-0.16377897	0.001055136	0.004161151	4	0	0
SELENBP1	0.6258955	0.554274554	0.071620996	0.194270574	0.001055421	0.004161361	8	0	0
KRTAP9-9	0.6851367	0.757565929	-0.07242927	-0.16232332	0.001056715	0.004165547	3	0	0
GPAM	0.6523774	0.659782974	-0.00740556	0.024851767	0.001057104	0.004165934	2	0	0
RBMXL2	0.5818683	0.654271347	-0.07240308	-0.16383409	0.001057278	0.004165934	4	0	0
DDR2	0.6395578	0.607158298	0.032399479	0.092764097	0.001057844	0.004167249	3	0	0
MATN3	0.1864043	0.198436903	-0.01203255	0.33168524	0.001060311	0.00417586	10	0	0
HARS2	0.1825011	0.198275835	-0.01577475	-0.12522059	0.001060495	0.00417586	17	0	0
MALL	0.5965036	0.563444351	0.033059212	0.078418071	0.001061908	0.004180507	7	0	0
SLC1A6	0.4807732	0.407738742	0.073034426	0.373629917	0.001064422	0.004189482	2	0	0
POLE2	0.378567	0.346785888	0.031781143	0.323191957	0.001065981	0.004194699	13	0	0
KHDRBS2	0.1970498	0.143814465	0.053235365	0.659677438	0.001066967	0.004197657	13	0	0
UGT1A8	0.7922312	0.844110061	-0.05187887	-0.09040393	0.001069534	0.004206834	1	0	0
MLST8	0.7518612	0.793137738	-0.04127655	-0.07766984	0.001071851	0.004215022	4	0	0
CHRNG	0.5171146	0.482863095	0.034251514	0.158531801	0.001072586	0.004216989	5	0	0
CA8	0.2217409	0.198290448	0.023450417	0.812645131	0.001073227	0.004218585	6	0	0
TVP23A	0.6968962	0.708499685	-0.0116035	-0.01098878	0.001074252	0.004221688	9	0	0
SAMD9	0.4586114	0.497536459	-0.0389251	-0.07681176	0.0010747	0.004222521	4	0	0
FAM229A	0.8216156	0.794477769	0.027137833	0.047821894	0.001076222	0.004227241	3	0	0
RARG	0.2776491	0.319693934	-0.04204487	-0.15303757	0.001076372	0.004227241	6	0	0
CCDC178	0.261722	0.23520678	0.026515194	0.488201511	0.001076791	0.004227961	6	0	0
NAAA	0.3178107	0.331188623	-0.0133779	-0.13519223	0.001080112	0.004239142	9	0	0
ADRA1A	0.2672349	0.301119718	-0.0338848	0.068859186	0.001079924	0.004239142	6	0	0
LCE3E	0.5377091	0.619005474	-0.08129636	-0.22304321	0.00108244	0.004246468	6	0	0
GABRR2	0.8810878	0.866895519	0.014192316	0.024256092	0.001082452	0.004246468	8	0	0
ZDHHC11	0.5306552	0.608713254	-0.07805804	-0.1703166	0.001082947	0.004247483	8	0	0
SERPINE2	0.1418001	0.165783588	-0.02398354	-0.16660993	0.001084258	0.004251693	5	0	0
SNX10	0.1130863	0.128618848	-0.01553252	-0.18283616	0.001085408	0.004255273	11	0	0
GUCA2B	0.6632304	0.689092752	-0.02586238	-0.05628187	0.001086022	0.004255821	10	0	0
FBXO46	0.2835693	0.313723229	-0.03015389	-0.04727447	0.00108595	0.004255821	9	0	0
CHGA	0.3022606	0.297833037	0.004427595	0.132051938	0.001087537	0.004260825	13	0	0
GPN1	0.331357	0.387455884	-0.05609892	-0.2507032	0.001090381	0.004271034	3	0	0
KLK10	0.2874462	0.318158569	-0.03071236	-0.09941705	0.001091746	0.004275449	12	0	0
ARL16	0.1735996	0.223612291	-0.05001272	-0.13395641	0.001094507	0.004285326	11	0	0
PLGRKT	0.2059722	0.256652572	-0.05068036	-0.15257616	0.001095519	0.004288353	8	0	0
ACTB	0.2206648	0.251370777	-0.03070597	-0.16968509	0.001096094	0.004289665	3	0	0
SLC25A12	0.2311479	0.273912146	-0.04276425	-0.17302832	0.001096644	0.004290884	5	0	0
HAVCR1	0.6795569	0.718608595	-0.03905174	-0.06469253	0.001097133	0.00429186	5	0	0
RHOV	0.2948764	0.323350847	-0.02847448	-0.02976812	0.001101269	0.004307098	10	0	0
CNOT6	0.2400902	0.255336839	-0.01524664	0.009282907	0.00110282	0.004311287	8	0	0
GRID2IP	0.8615071	0.890824388	-0.02931728	-0.05026151	0.001102672	0.004311287	4	0	0
SGPP2	0.1757746	0.20095443	-0.0251798	-0.16802633	0.001103507	0.00431303	8	0	0
TDRP	0.3429726	0.351736511	-0.00876391	0.013868059	0.001104599	0.004315516	10	0	0
RHOJ	0.3003238	0.296506567	0.003817186	0.182182468	0.001104624	0.004315516	9	0	0
DHX15	0.2534116	0.275344149	-0.02193252	-0.09871259	0.001105062	0.004316286	8	0	0
MPZL1	0.1865748	0.235608295	-0.04903348	-0.31322504	0.001106272	0.004320072	4	0	0

NID1	0.2784623	0.234218072	0.044244222	0.457988707	0.001108188	0.004326557	7	0	0
PTRH1	0.439687	0.481725748	-0.0420387	-0.11443924	0.001108415	0.004326557	4	0	0
CHD3	0.1283286	0.129597134	-0.00126853	-0.09816878	0.001115385	0.004352816	3	0	0
CD14	0.4824991	0.489300717	-0.00680162	-0.06104526	0.00111616	0.004354894	4	0	0
LAMTOR5	0.1573076	0.183644703	-0.02633714	-0.11970442	0.001116572	0.004355552	13	0	0
STRA8	0.7679283	0.817937321	-0.05000902	-0.09266516	0.001117895	0.004359767	6	0	0
ABCG5	0.7297385	0.737216451	-0.00747798	-0.00498018	0.00112066	0.004368668	15	0	0
RBP1	0.2541756	0.26027817	-0.00610255	0.222789593	0.001120665	0.004368668	10	0	0
PIK3C2G	0.7706946	0.823910293	-0.05321567	-0.10097577	0.001121443	0.004370753	6	0	0
CHMP6	0.1511863	0.185049146	-0.03386281	-0.13740598	0.001122241	0.004372912	11	0	0
SRSF3	0.0497281	0.055707644	-0.00597956	-0.06450436	0.001123639	0.00437646	12	0	0
SALL1	0.1671385	0.118990888	0.048147572	0.597001521	0.0011234	0.00437646	19	0	0
IFRD1	0.4694701	0.517391945	-0.04792181	-0.18091442	0.001124125	0.004377037	7	0	0
ART1	0.8041285	0.824964282	-0.02083579	-0.03580863	0.001124276	0.004377037	5	0	0
ATP2A2	0.2902901	0.316162249	-0.02587212	-0.04475641	0.00112458	0.004377271	12	0	0
MYL7	0.6700235	0.70494651	-0.03492296	-0.06800504	0.001126503	0.004382852	10	0	0
C18orf21	0.2078892	0.245786152	-0.03789691	-0.14428708	0.001126429	0.004382852	10	0	0
CPEB4	0.2345137	0.247075106	-0.01256139	-0.06673202	0.001127219	0.004383189	14	0	0
HIST4H4	0.1187029	0.160342642	-0.04163971	-0.14610367	0.001127357	0.004383189	11	0	0
CLN5	0.8732332	0.898397006	-0.02516385	-0.04083088	0.001127178	0.004383189	2	0	0
PCSK6	0.8406781	0.887687008	-0.04700896	-0.07759929	0.001127567	0.004383189	1	0	0
POC5	0.3528053	0.387548782	-0.03474351	-0.08243516	0.001128104	0.004384327	6	0	0
TMEM237	0.3415628	0.418510125	-0.0769473	-0.17301257	0.001129107	0.004387276	8	0	0
TNK1	0.4113065	0.395537999	0.01576853	0.199933157	0.001129512	0.004387898	11	0	0
KRTAP5-5	0.5608459	0.607184036	-0.04633814	-0.12986128	0.001130445	0.00439057	5	0	0
CLEC10A	0.5122208	0.554613699	-0.04239286	-0.11698472	0.001131678	0.004394411	7	0	0
SLC26A1	0.773056	0.806965126	-0.03390915	-0.05875789	0.001132109	0.00439513	5	0	0
PRSS33	0.5017552	0.496086582	0.00566857	0.004356657	0.001132995	0.004397618	5	0	0
DNAJC5B	0.7839869	0.845539073	-0.06155217	-0.11172545	0.001133525	0.004398723	5	0	0
ZNF330	0.4119999	0.443223823	-0.0312239	-0.06896784	0.001134529	0.004401669	10	0	0
COL4A2	0.2196839	0.141249927	0.078433974	0.595901761	0.001136183	0.004406178	3	0	0
NUP50	0.2652438	0.323227789	-0.05798394	-0.19264021	0.001135976	0.004406178	2	0	0
HMGCLL1	0.3978588	0.414686919	-0.01682811	0.158242306	0.001137297	0.004409544	10	0	0
ZBTB8OS	0.1306166	0.137716525	-0.00709996	0.04809524	0.001138163	0.004410995	11	0	0
EDNRB	0.2641751	0.233732716	0.030442406	0.452118654	0.001137992	0.004410995	24	0	0
HLTF	0.4590223	0.496006454	-0.03698413	-0.05881263	0.001140167	0.004416853	22	0	0
SLC10A6	0.8012018	0.852309237	-0.05110742	-0.09175601	0.00114003	0.004416853	4	0	0
SLC7A5	0.6307795	0.705857589	-0.07507808	-0.15984459	0.001141529	0.004421175	1	0	0
SLC4A9	0.7038455	0.736137179	-0.03229165	-0.06324934	0.001147202	0.004441342	4	0	0
CER1	0.5942587	0.620193201	-0.02593453	-0.04540352	0.001147231	0.004441342	4	0	0
CMC2	0.9040826	0.921208299	-0.01712567	-0.02677744	0.001148699	0.004446067	2	0	0
TXLNB	0.5188689	0.559963338	-0.04109444	-0.17997498	0.00115186	0.004457337	4	0	0
HOOK1	0.1660597	0.180811589	-0.01475191	-0.10387238	0.00115299	0.004460749	11	0	0
C6orf201	0.8934663	0.882032315	0.011433958	0.018932981	0.001155112	0.004467994	6	0	0
DNPEP	0.649154	0.698579245	-0.04942522	-0.1054955	0.001158815	0.004481351	5	0	0
ARPP19	0.8250005	0.837825783	-0.01282524	-0.02401302	0.001159911	0.004484623	8	0	0
KHDC3L	0.7811593	0.832278401	-0.05111908	-0.09469419	0.001162319	0.004492963	13	0	0
SCOC	0.7336822	0.750164705	-0.01648246	-0.02470195	0.001163407	0.0044962	7	0	0
AADAC	0.7495207	0.817987054	-0.06846635	-0.12492743	0.001165256	0.004502377	3	0	0
DCST1	0.7637977	0.790002815	-0.0262051	-0.04778323	0.001166036	0.004503987	7	0	0
PYDC1	0.3564145	0.345654507	0.010760033	0.217712613	0.001166175	0.004503987	12	0	0
FAM217A	0.8382442	0.875158941	-0.03691469	-0.06417492	0.001166533	0.004504399	4	0	0
MIB2	0.0749455	0.103970977	-0.02902549	-0.14156111	0.001167813	0.00450837	9	0	0
METTL7B	0.2962981	0.346347069	-0.05004894	-0.16454234	0.001169935	0.004515592	11	0	0
MRPL42	0.3395294	0.354731067	-0.01520165	0.041652224	0.00117194	0.004522357	8	0	0
CD180	0.7679817	0.783399657	-0.01541793	-0.02054606	0.001172477	0.004522485	5	0	0
SLC52A3	0.3448074	0.392654972	-0.04784757	-0.18488795	0.001172453	0.004522485	8	0	0
LHX6	0.2993827	0.303988189	-0.00460547	0.107936008	0.001173417	0.004525138	5	0	0
CELF1	0.0782968	0.088680393	-0.01038359	-0.00155863	0.001173716	0.004525317	9	0	0
DPAGT1	0.6978167	0.722494397	-0.02467773	-0.10514407	0.001174109	0.004525794	4	0	0
CPNE7	0.2067598	0.216218458	-0.00945862	0.102519874	0.001174345	0.004525794	5	0	0
FERMT2	0.3283111	0.289379936	0.038931177	0.224428866	0.001175566	0.004529527	8	0	0
STX19	0.6742392	0.745442379	-0.07120318	-0.15219832	0.001176469	0.004530731	8	0	0

KPNA1	0.3182597	0.352018452	-0.03375877	-0.12587265	0.001176636	0.004530731	9	0	0
DNAAF3	0.1039307	0.118042295	-0.01411156	-0.13863598	0.001176449	0.004530731	12	0	0
SLC22A12	0.706798	0.764203749	-0.05740578	-0.11357377	0.001177052	0.004531359	6	0	0
GH2	0.5623305	0.612612134	-0.0502816	-0.11974201	0.001186091	0.00456518	2	0	0
RARA	0.3389961	0.2825698	0.056426321	0.294607238	0.00118681	0.004566967	12	0	0
STAT4	0.5804945	0.614867457	-0.03437298	-0.13038545	0.00118758	0.00456699	11	0	0
GNB3	0.5835714	0.629513636	-0.04594223	-0.0979783	0.001187525	0.00456699	5	0	0
THOC5	0.8422385	0.825925732	0.016312797	0.032469938	0.001187379	0.00456699	3	0	0
ARSG	0.5614208	0.557321376	0.00409942	0.536158177	0.001190798	0.004578384	4	0	0
ARHGEF10	0.08527	0.05537318	0.029896839	0.527632588	0.001191605	0.004580504	8	0	0
LRRC36	0.1916572	0.190806866	0.000850319	0.408222253	0.00119302	0.004584961	10	0	0
E2F8	0.1950952	0.152845593	0.042249624	0.321015933	0.00120235	0.004619828	8	0	0
TAS2R9	0.76134	0.833244446	-0.07190449	-0.13084668	0.001203075	0.004621622	3	0	0
PSPC1	0.3027061	0.317716073	-0.01500996	-0.04354634	0.001204296	0.004625321	6	0	0
FBXO25	0.0267115	0.020985895	0.005725625	0.199216084	0.001204878	0.004626568	2	0	0
CEBPE	0.4774522	0.535682567	-0.05823038	-0.13585367	0.00120595	0.004629692	4	0	0
ADIG	0.6068973	0.630707022	-0.02380975	-0.03398688	0.001206324	0.004630135	7	0	0
PRKG2	0.1189747	0.076212167	0.0427625	0.68580771	0.001212307	0.004650115	9	0	0
SLC10A5	0.7748904	0.816279475	-0.04138907	-0.07704124	0.001212115	0.004650115	7	0	0
GGACT	0.5816123	0.631418447	-0.04980614	-0.11478173	0.001212031	0.004650115	6	0	0
TUBA1A	0.3171885	0.347771415	-0.0305829	-0.04087647	0.001213277	0.004652841	13	0	0
GPR162	0.297314	0.306998777	-0.00968475	0.21877891	0.001215873	0.0046618	5	0	0
DDB2	0.0695339	0.078328814	-0.00879486	-0.12160481	0.001216737	0.004664114	6	0	0
NR3C1	0.6077239	0.649377475	-0.04165362	-0.1054421	0.001219512	0.004672756	5	0	0
CMKLR1	0.2538216	0.213910369	0.039911196	0.730121436	0.001219414	0.004672756	10	0	0
MUTYH	0.1865795	0.22156497	-0.03498547	-0.12143612	0.001220651	0.004676119	13	0	0
DHX34	0.1702941	0.167030646	0.00326349	0.071903249	0.001223367	0.004685524	13	0	0
MAF	0.0514422	0.041836072	0.009606173	0.23600817	0.001225132	0.004691284	13	0	0
RFPL3	0.7455366	0.721893898	0.023642704	0.05550918	0.00122563	0.004692188	3	0	0
TMED8	0.1700498	0.194881186	-0.02483137	-0.03781	0.001225909	0.004692253	11	0	0
HAUS6	0.3044304	0.3442501	-0.03981971	-0.13871298	0.001227868	0.004695966	9	0	0
DBX2	0.1886228	0.133094883	0.055527881	0.613107534	0.001227926	0.004695966	12	0	0
POSTN	0.4916459	0.518208658	-0.02656271	-0.05012024	0.001227596	0.004695966	5	0	0
TIMM44	0.0386869	0.038168276	0.000518596	0.026321725	0.001227624	0.004695966	6	0	0
MMRN1	0.5703735	0.638943823	-0.0685703	-0.17648465	0.001230458	0.004704645	4	0	0
SLC4A2	0.4200139	0.389638363	0.030375503	0.175004953	0.001232141	0.004709075	6	0	0
SLC22A31	0.388434	0.364016765	0.024417247	0.506892083	0.00123198	0.004709075	3	0	0
TAB2	0.8483106	0.883605361	-0.03529479	-0.06021144	0.001232835	0.004710723	2	0	0
ZNF296	0.2614857	0.280724863	-0.01923916	-0.13525952	0.001234234	0.004715061	11	0	0
GFI1	0.3114329	0.307312158	0.004120765	0.405590556	0.001236285	0.004721894	20	0	0
RAB11FIP5	0.0953096	0.035137406	0.060172176	1.145294887	0.001236721	0.004722552	2	0	0
GSN	0.6113586	0.642083292	-0.03072471	-0.06260544	0.001237972	0.004726325	2	0	0
SLC17A3	0.6848303	0.641626041	0.043204213	0.16063331	0.001239792	0.004731375	7	0	0
C9orf152	0.6142593	0.697501512	-0.08324217	-0.18418123	0.001239823	0.004731375	5	0	0
WASL	0.1134305	0.141297685	-0.02786715	-0.08802623	0.001240408	0.0047314	13	0	0
FOXN1	0.6437343	0.708549656	-0.06481532	-0.13793912	0.001240621	0.0047314	4	0	0
PKP1	0.4390795	0.450875534	-0.01179601	0.137368003	0.001242663	0.004738182	11	0	0
TFF3	0.6738377	0.727081053	-0.05324331	-0.11327808	0.001243036	0.004738596	12	0	0
OR7A10	0.5771737	0.558020689	0.019153021	0.140200474	0.001244477	0.00474308	3	0	0
ZFP62	0.0392882	0.035633948	0.003654279	0.059705097	0.001244958	0.004743907	6	0	0
PRKCSH	0.1037563	0.11646594	-0.01270969	-0.06780191	0.001245602	0.004745352	14	0	0
MTO1	0.083539	0.105774915	-0.0222359	-0.2151053	0.001246457	0.004746591	8	0	0
HIF3A	0.1785023	0.154992515	0.023509755	0.5378172	0.001246344	0.004746591	9	0	0
PRRT3	0.2967743	0.308519923	-0.01174566	0.000734734	0.001247568	0.004749814	11	0	0
TNFRSF1A	0.3280313	0.321683703	0.006347593	0.251254907	0.001250086	0.004758391	7	0	0
CHRD	0.6037228	0.633310696	-0.02958787	0.030059402	0.001251771	0.004761773	7	0	0
TMEM200A	0.176316	0.14674129	0.029574757	0.783392452	0.001251628	0.004761773	10	0	0
NKAIN4	0.1575826	0.191555267	-0.03397267	-0.10431237	0.001251249	0.004761773	6	0	0
SCARA3	0.0702147	0.060918255	0.009296398	0.194766236	0.001252668	0.004764176	3	0	0
CTGF	0.3147007	0.263435093	0.051265592	0.300463256	0.001254732	0.004771015	10	0	0
PEX2	0.5095551	0.564687285	-0.0551322	-0.15053507	0.001257237	0.004779528	7	0	0
PCDHA1	0.4242857	0.428779381	-0.00449365	0.064867568	0.001257979	0.004781333	9	0	0
SLC1A2	0.1854145	0.134773833	0.050640687	0.784045602	0.001260368	0.004789398	12	0	0

DNASE1L2	0.7511265	0.792699988	-0.04157345	-0.08287991	0.001262342	0.004795882	9	0	0
KDM2B	0.2606746	0.22099639	0.039678188	0.247121447	0.001263281	0.004798434	18	0	0
C12orf45	0.2518665	0.310208915	-0.05834243	-0.11088006	0.001265294	0.004805064	6	0	0
UBXN10	0.2438097	0.207366628	0.036443108	0.572277288	0.00126882	0.004816414	11	0	0
LTC4S	0.4420316	0.378378589	0.063653015	0.228005948	0.001268727	0.004816414	7	0	0
ASIC4	0.1515208	0.116760569	0.034760233	0.600035074	0.001270219	0.004820703	14	0	0
CR2	0.2882436	0.328530139	-0.04028651	-0.09699948	0.001270762	0.004821744	13	0	0
XRCC1	0.4378216	0.350396542	0.08742507	0.42271369	0.001272618	0.004827767	5	0	0
C14orf180	0.4985115	0.542131896	-0.04362038	-0.11533169	0.00127384	0.004831381	7	0	0
RARRES1	0.3207586	0.288174557	0.032584052	0.672551622	0.001275169	0.004835396	6	0	0
HHIPL1	0.3873037	0.385634006	0.001669693	0.202645957	0.001276122	0.004837988	10	0	0
BST2	0.3146902	0.374758452	-0.06006827	-0.24300532	0.001276537	0.004838538	9	0	0
WNT3	0.6588491	0.681652829	-0.02280375	-0.0465965	0.001278028	0.004843166	4	0	0
ONECUT2	0.1502962	0.112998835	0.037297344	0.504453808	0.001279229	0.004846693	9	0	0
MAP6D1	0.203479	0.2453786	-0.0418996	-0.0162595	0.001280171	0.004848215	11	0	0
SLC52A2	0.5317619	0.581767183	-0.05000526	-0.14547137	0.001280054	0.004848215	7	0	0
UBAP2L	0.1506231	0.178068976	-0.02744587	-0.13094191	0.001281361	0.004849189	11	0	0
NAB1	0.7540062	0.846266874	-0.0922607	-0.16500098	0.00128151	0.004849189	2	0	0
HIST1H2BL	0.1299523	0.148247173	-0.01829489	0.002354303	0.001281343	0.004849189	8	0	0
FASN	0.1028152	0.13173057	-0.02891541	-0.08151762	0.001281283	0.004849189	5	0	0
NEK7	0.2433441	0.288337612	-0.04499349	-0.13034254	0.001283389	0.004854251	6	0	0
CLEC14A	0.4001951	0.383558018	0.016637036	0.083562307	0.00128336	0.004854251	9	0	0
RDH8	0.4125089	0.476711305	-0.0642024	-0.19553994	0.001284452	0.004857249	6	0	0
FABP6	0.8147543	0.802710737	0.012043514	0.028517679	0.001284759	0.004857386	4	0	0
MICAL2	0.8924051	0.907703327	-0.0152982	-0.02424695	0.00128524	0.004858178	2	0	0
MYOZ1	0.7036034	0.731159253	-0.02755585	-0.05379134	0.0012908	0.004878169	9	0	0
FXYD4	0.6256245	0.681504817	-0.05588032	-0.12418107	0.00129149	0.00487894	5	0	0
CAPS	0.6122633	0.739998434	-0.12773515	-0.26936245	0.001291548	0.00487894	1	0	0
ISG20	0.5473268	0.575351514	-0.02802474	-0.05624023	0.001292358	0.00488097	6	0	0
BRK1	0.1237417	0.132048364	-0.00830669	-0.11843919	0.00129604	0.004893846	9	0	0
EGR2	0.6372764	0.711027701	-0.07375133	-0.14983057	0.001296492	0.004894524	3	0	0
C17orf97	0.177413	0.195829528	-0.01841653	-0.02572415	0.001297375	0.004896824	10	0	0
ABCG8	0.7241967	0.736508254	-0.01231159	-0.01567741	0.001300601	0.004907063	17	0	0
PRSS42	0.8511416	0.892236202	-0.04109459	-0.06795216	0.001300634	0.004907063	9	0	0
POU5F1	0.6618455	0.601405339	0.060440156	0.138482542	0.001302651	0.004913636	14	0	0
CD151	0.4350233	0.473287115	-0.03826384	-0.07481384	0.001305559	0.004923572	7	0	0
PITPNM1	0.291014	0.320319474	-0.02930546	-0.21907823	0.001308825	0.004933813	3	0	0
COL4A1	0.1316977	0.089883732	0.041814007	0.467881041	0.001308632	0.004933813	6	0	0
PYGO2	0.8668976	0.853619043	0.013278517	0.024523875	0.001310519	0.004938122	3	0	0
KLK3	0.5945876	0.648728692	-0.05414105	-0.12805819	0.001310491	0.004938122	5	0	0
GIMAP8	0.3599406	0.293772539	0.066168093	0.550769009	0.001312611	0.004944968	14	0	0
ADAMTS5	0.158343	0.110820458	0.047522554	0.492106496	0.001313469	0.004947161	10	0	0
ITGA11	0.2377912	0.277823316	-0.0400321	-0.00346329	0.00131478	0.004951057	9	0	0
EIF2AK1	0.228409	0.259859956	-0.03145092	-0.07317588	0.001315897	0.004954226	12	0	0
PLA2G12B	0.7272832	0.670545083	0.056738144	0.119443505	0.001316606	0.004954816	5	0	0
DSCAM	0.4085805	0.371464588	0.037115942	0.340220711	0.001316539	0.004954816	6	0	0
CNOT10	0.0633242	0.054354651	0.008969512	0.153207394	0.00131758	0.004957329	12	0	0
MSMP	0.1928008	0.110654111	0.08214666	1.042523756	0.001317827	0.004957329	2	0	0
MRPL44	0.1999219	0.221142136	-0.02122021	-0.00785973	0.001318427	0.004958038	10	0	0
C11orf58	0.1884498	0.131348447	0.057101363	0.374754319	0.001318568	0.004958038	7	0	0
KRT37	0.8646287	0.889162957	-0.02453423	-0.04035664	0.001318992	0.004958592	5	0	0
POLR2C	0.3094882	0.298031367	0.011456865	0.164062551	0.001327295	0.004987717	11	0	0
MED1	0.3385284	0.365314017	-0.02678565	-0.12558443	0.00132711	0.004987717	7	0	0
SBK2	0.5446522	0.540769007	0.003883159	0.105228635	0.001328238	0.004990214	10	0	0
ANO10	0.2065863	0.235520428	-0.02893409	-0.11865646	0.001329625	0.004991241	3	0	0
NPFFR2	0.4844969	0.488522968	-0.00402607	0.125527109	0.001328902	0.004991241	11	0	0
HOGA1	0.6851539	0.726894573	-0.04174062	-0.09668675	0.001329506	0.004991241	7	0	0
PI4K2A	0.6851539	0.726894573	-0.04174062	-0.09668675	0.001329506	0.004991241	7	0	0
KIRREL2	0.3762418	0.325006594	0.051235225	0.2736014	0.001330954	0.004995185	2	0	0
ARHGAP24	0.0645559	0.080231868	-0.01567602	-0.20455244	0.001332429	0.004999677	12	0	0
LMTK2	0.2652992	0.304379181	-0.03907998	-0.08936786	0.001333374	0.005002175	10	0	0
KYNU	0.648368	0.711798285	-0.06343031	-0.14109347	0.001334691	0.005006068	7	0	0
ERVV-2	0.5886894	0.670835287	-0.08214586	-0.18925966	0.001337344	0.005014972	3	0	0

EVC2	0.3115887	0.32674306	-0.01515437	0.007184371	0.001339284	0.005021194	8	0	0
ENC1	0.1366248	0.124547469	0.012077376	0.618978896	0.001340549	0.005024886	9	0	0
SLC44A3	0.2797326	0.317178851	-0.03744625	-0.10728212	0.00134238	0.005030699	8	0	0
ZNF256	0.096018	0.049609027	0.046409023	0.94495546	0.001343451	0.00503366	12	0	0
LRRC52	0.5375701	0.532351791	0.005218326	0.167962433	0.001344979	0.005038334	6	0	0
FAS	0.1574372	0.115265109	0.04217205	0.250659038	0.001345863	0.005040593	6	0	0
MTFR1L	0.4160676	0.350684928	0.065382688	0.229348551	0.001349018	0.005049247	3	0	0
PTGER4	0.0508703	0.068826095	-0.01795578	-0.16416169	0.001348775	0.005049247	10	0	0
PDGFRB	0.1818386	0.135226687	0.046611901	0.508111105	0.001348558	0.005049247	4	0	0
LRRD1	0.3037564	0.233525993	0.070230382	0.628408201	0.001349479	0.005049918	8	0	0
LCE2D	0.5807883	0.6582658	-0.07747752	-0.17954892	0.001350478	0.005051549	6	0	0
SLC25A20	0.3043477	0.297891739	0.006455965	0.219828079	0.001350939	0.00505222	11	0	0
SPRR2B	0.7483929	0.806014982	-0.05762209	-0.10373326	0.001352055	0.00505534	4	0	0
TCEA1	0.2185004	0.264910771	-0.0464104	-0.07033667	0.001352668	0.005055964	10	0	0
IGF2	0.3968357	0.454151686	-0.05731602	-0.18996322	0.001353068	0.005055964	14	0	0
HNF4A	0.6664984	0.680479227	-0.01398085	-0.03627652	0.001352937	0.005055964	13	0	0
KRTAP19-4	0.4834434	0.558328463	-0.07488508	-0.20409313	0.001354379	0.005059807	2	0	0
PCSK2	0.2088155	0.166033329	0.042782136	0.586514612	0.001357995	0.00507226	8	0	0
FAM160B1	0.1375353	0.152043203	-0.01450791	-0.12252693	0.001358308	0.005072373	9	0	0
GLP1R	0.2061312	0.200605937	0.005525292	0.303707519	0.001358707	0.005072809	9	0	0
KCNA6	0.4016597	0.427990531	-0.02633078	0.051061245	0.001359138	0.005073361	12	0	0
FOXJ1	0.26677	0.234742656	0.032027375	0.206057534	0.001360918	0.005078949	10	0	0
CDC42	0.3356785	0.380006846	-0.04432831	-0.06369695	0.001366973	0.005098364	5	0	0
CELF6	0.140045	0.147872253	-0.00782727	0.019977077	0.001366836	0.005098364	15	0	0
AMBN	0.7663174	0.805623563	-0.03930613	-0.07467442	0.001368566	0.005103244	7	0	0
DDX47	0.2969867	0.3280663	-0.0310796	-0.10954395	0.001369057	0.005104012	8	0	0
KLHL12	0.7704767	0.835637018	-0.06516027	-0.11187678	0.001370583	0.005108155	5	0	0
EVC	0.1140415	0.101085334	0.012956204	0.270442809	0.001370738	0.005108155	10	0	0
CNKSR3	0.2322626	0.191960082	0.040302539	0.270967813	0.001374092	0.005119592	5	0	0
PDSS2	0.3617941	0.401670126	-0.03987605	-0.08289635	0.001375917	0.005125327	4	0	0
RAD51D	0.7650137	0.812001075	-0.04698735	-0.08600457	0.001376428	0.005126166	5	0	0
ZMYM2	0.0528566	0.060076534	-0.00721993	-0.06146078	0.001377788	0.005130166	8	0	0
VWA3B	0.1661674	0.181536972	-0.0153696	-0.12866192	0.00137844	0.005131526	13	0	0
OR1E1	0.3301967	0.379567617	-0.04937094	-0.19386275	0.001386318	0.005157644	3	0	0
MALT1	0.1208654	0.163062966	-0.04219754	-0.11370592	0.001386278	0.005157644	9	0	0
STMN3	0.2599032	0.27017096	-0.01026778	0.169458772	0.001388717	0.005165499	10	0	0
LPIN3	0.2524084	0.285044163	-0.03263577	-0.12029687	0.001390574	0.005171331	11	0	0
ATAD1	0.823287	0.802410028	0.020876936	0.035420331	0.001396964	0.005194019	2	0	0
NT5E	0.2099253	0.159074341	0.050850917	0.571293191	0.001401625	0.00521027	13	0	0
TRAPPC10	0.5463632	0.570238796	-0.02387563	-0.05371759	0.001402671	0.005213076	6	0	0
ASCL1	0.1376391	0.088091158	0.049547962	0.742046091	0.001403626	0.005215546	10	0	0
GAS2L2	0.5106329	0.484207554	0.026425391	0.11242462	0.001405864	0.005222779	8	0	0
ROBO4	0.4506708	0.413318389	0.037352415	0.139604026	0.001408366	0.00523099	9	0	0
RHOH	0.6884766	0.678310654	0.010165995	0.035368722	0.001409012	0.005232207	7	0	0
ZNF350	0.3025331	0.26393925	0.038593874	0.536453281	0.001409497	0.005232207	9	0	0
PPP1R16B	0.3001496	0.222307857	0.077841782	0.860337135	0.001409616	0.005232207	11	0	0
RTEL1	0.4308604	0.460083995	-0.02922363	-0.02822684	0.00140986	0.005232207	11	0	0
MUC13	0.8695031	0.904978375	-0.03547523	-0.05826487	0.001410388	0.005233082	3	0	0
OR1A1	0.5912058	0.644285523	-0.05307974	-0.1222	0.001412922	0.0052414	6	0	0
NFATC3	0.2800892	0.247076716	0.033012526	0.318823816	0.00141438	0.005245723	6	0	0
PHOX2A	0.377907	0.344938565	0.032968411	0.247086825	0.001422461	0.005274603	6	0	0
B3GNT8	0.6119282	0.595039504	0.016888677	0.186280804	0.001423061	0.00527574	7	0	0
LCE1B	0.788546	0.853218918	-0.06467289	-0.1115116	0.001424778	0.005281012	2	0	0
SLC17A8	0.4424426	0.431268785	0.0111738	0.110061209	0.00142695	0.005287972	11	0	0
CDH6	0.3291039	0.363782079	-0.03467819	0.126042367	0.00142891	0.005294141	9	0	0
PGAM2	0.3989792	0.424848677	-0.02586944	-0.0367055	0.001429785	0.005296288	8	0	0
OR6C70	0.7343648	0.803388144	-0.06902339	-0.14401242	0.001434967	0.005314386	3	0	0
CCDC88C	0.0580858	0.070589948	-0.01250419	-0.12391123	0.001436436	0.005318729	9	0	0
ZNF558	0.0559947	0.043300114	0.012694628	0.309600344	0.001437138	0.005320228	5	0	0
UAP1L1	0.2553579	0.22222994	0.033127955	0.257638201	0.001438053	0.005322519	8	0	0
SNX22	0.085979	0.0942182	-0.00823925	-0.1056207	0.001438977	0.005324837	12	0	0
PROM1	0.4763335	0.499502453	-0.02316891	-0.0437809	0.00144012	0.005327892	11	0	0
CLEC2L	0.1959682	0.139774324	0.056193874	0.476008432	0.001440396	0.005327892	8	0	0

LAP3	0.1188474	0.14050395	-0.02165651	-0.06700254	0.001441003	0.005329037	8	0	0
SIRT1	0.3762048	0.388383672	-0.01217886	0.026963995	0.001442042	0.005331782	9	0	0
MEF2A	0.4871255	0.548031676	-0.0609062	-0.15449308	0.001443035	0.005334352	5	0	0
CABYR	0.2026252	0.223838976	-0.02121373	-0.07681501	0.001446684	0.005346739	11	0	0
OXLD1	0.1951254	0.241858672	-0.04673327	-0.10367389	0.001447356	0.005348122	13	0	0
LY6G6D	0.687864	0.726166161	-0.03830218	-0.08890377	0.001449734	0.005355803	5	0	0
BDNF	0.1511029	0.096162474	0.054940411	0.628264808	0.001450521	0.005357609	14	0	0
KLK4	0.5759375	0.620436339	-0.04449882	-0.07912254	0.00145336	0.005366989	9	0	0
SYDE1	0.1807141	0.155260186	0.025453937	0.338242332	0.001454685	0.005370777	14	0	0
ISL1	0.1974462	0.138612462	0.058833718	0.456776874	0.001457098	0.005378579	17	0	0
GIF	0.6377685	0.700164735	-0.06239626	-0.13095734	0.001458097	0.005381158	6	0	0
ARNT2	0.3144911	0.341857572	-0.02736644	0.064115647	0.001459671	0.00538586	12	0	0
POU1F1	0.7816079	0.835452306	-0.05384443	-0.09985745	0.00146008	0.00538626	7	0	0
RRAS2	0.8754115	0.896819377	-0.02140784	-0.03499172	0.001460787	0.005387759	4	0	0
CHRM5	0.7017727	0.706475373	-0.00470271	-0.00157966	0.001461111	0.005387847	8	0	0
KANK4	0.5675632	0.577704728	-0.01014153	0.256234119	0.001462848	0.005393142	6	0	0
CA4	0.2478879	0.206559926	0.041327926	0.612062687	0.001463768	0.005395425	9	0	0
KRT3	0.6862667	0.742087647	-0.05582091	-0.12025404	0.001465642	0.005401223	4	0	0
SOCS3	0.1681137	0.167601391	0.000512294	-0.08624601	0.001466516	0.005403335	10	0	0
AURKAIP1	0.2201941	0.233766535	-0.01357242	0.032844451	0.001466865	0.005403509	12	0	0
HMGN1	0.1157167	0.131820552	-0.01610382	0.019376242	0.001467689	0.005405437	7	0	0
PRSS21	0.6715335	0.716479421	-0.04494587	-0.0952977	0.001468046	0.005405638	6	0	0
PRAM1	0.5754002	0.616031554	-0.04063138	-0.09264536	0.001472278	0.005420111	6	0	0
GPSM2	0.8895001	0.900882684	-0.01138257	-0.0184376	0.00147437	0.005426347	4	0	0
PCDHA3	0.4089391	0.377288363	0.031650692	0.25684825	0.001474873	0.005426347	9	0	0
ID4	0.2600167	0.214075376	0.045941285	0.484940991	0.001475126	0.005426347	6	0	0
SMAD2	0.2363258	0.276398657	-0.04007286	-0.15058608	0.001475182	0.005426347	9	0	0
LIPE	0.8050948	0.835662304	-0.03056754	-0.05456991	0.001477112	0.005432333	7	0	0
ELP5	0.0599115	0.067986272	-0.00807475	-0.14199265	0.001478188	0.005434062	4	0	0
CHGB	0.1784385	0.219335768	-0.04089728	-0.06772999	0.001478138	0.005434062	9	0	0
PLEKHH2	0.0557237	0.05932548	-0.00360179	-0.11909931	0.001478945	0.005435073	3	0	0
ANKRD53	0.1492568	0.167000653	-0.0177439	-0.06431412	0.00147907	0.005435073	7	0	0
TH	0.6027612	0.660809473	-0.05804824	-0.13256087	0.001480658	0.005439794	8	0	0
CFTR	0.7378408	0.779014665	-0.04117388	-0.0793894	0.001482962	0.005447145	3	0	0
COL15A1	0.7266161	0.778974388	-0.05235831	-0.10182644	0.00148449	0.005450526	4	0	0
DLK1	0.6503728	0.712528973	-0.06215614	-0.13474781	0.001484445	0.005450526	9	0	0
PKDREJ	0.7329587	0.737331419	-0.00437277	0.08554869	0.001487861	0.005461785	5	0	0
FAM193B	0.3173917	0.355851555	-0.03845989	-0.08965259	0.001488231	0.005462025	12	0	0
JAK3	0.3513513	0.301942934	0.049408403	0.54045583	0.001489821	0.005466742	13	0	0
REV1	0.2741152	0.340179512	-0.0660643	-0.14174098	0.001491092	0.005470284	4	0	0
SLITRK5	0.0997223	0.066378511	0.033343839	0.525170768	0.001493386	0.005477583	12	0	0
IMPA2	0.0798104	0.060454005	0.019356356	0.388020857	0.001497682	0.00548963	6	0	0
DTD1	0.1623399	0.194706902	-0.03236696	-0.0647756	0.001497895	0.00548963	14	0	0
TTBK1	0.1846775	0.144317981	0.040359523	0.711998291	0.001498643	0.005491247	7	0	0
PRIMA1	0.0854327	0.048250884	0.03718184	0.598521083	0.001499557	0.005493477	11	0	0
C1QTNF7	0.6112304	0.653200866	-0.04197042	-0.09044336	0.001500237	0.005494842	6	0	0
EXOSC9	0.3017731	0.312305555	-0.01053244	-0.04616571	0.001501738	0.005499218	9	0	0
BAIAP2L2	0.7418462	0.793919452	-0.05207326	-0.10219066	0.001502926	0.005502447	6	0	0
FOXL2	0.2051771	0.14847859	0.056698556	1.027694641	0.001503839	0.005504664	14	0	0
NRGN	0.3424153	0.358998631	-0.01658334	-0.02390663	0.001506148	0.005511992	6	0	0
RRAD	0.3121578	0.275946902	0.036210914	0.223083467	0.001507605	0.005516198	10	0	0
MICALCL	0.8339203	0.863772121	-0.02985181	-0.06005984	0.001510277	0.005524849	3	0	0
ZBTB17	0.2665703	0.290313529	-0.02374319	-0.08241285	0.001512685	0.005529144	10	0	0
IP6K3	0.7432621	0.728738707	0.01452338	0.053893914	0.001512078	0.005529144	3	0	0
TMEM223	0.1324965	0.112053305	0.020443204	0.13744131	0.001512669	0.005529144	10	0	0
TAS2R7	0.7596683	0.810308756	-0.05064042	-0.09047026	0.001511974	0.005529144	5	0	0
IFT88	0.3850835	0.384259779	0.000823739	0.108713305	0.001516757	0.005542901	9	0	0
DUSP26	0.2587455	0.257365101	0.001380432	0.241897648	0.001520116	0.005552912	12	0	0
FGF4	0.2628375	0.2255239	0.037313609	0.853055417	0.001520024	0.005552912	7	0	0
PPP1R36	0.4007871	0.420269439	-0.01948234	-0.08795973	0.001521223	0.005555825	7	0	0
KLHL14	0.399741	0.458343264	-0.05860226	-0.21241911	0.001521949	0.005557346	11	0	0
SLC22A11	0.6487334	0.654678376	-0.00594502	-0.01326688	0.001523981	0.005563631	9	0	0
PCGF1	0.5090025	0.509575231	-0.00057274	-0.00904588	0.001528629	0.005578331	7	0	0

EIF4E	0.9085082	0.917294976	-0.00878675	-0.01370278	0.001528403	0.005578331	2	0	0
CCDC172	0.6957949	0.743851348	-0.04805647	-0.09503949	0.001532362	0.005590813	12	0	0
PRAMEF11	0.5435347	0.622988519	-0.0794538	-0.19350552	0.001533653	0.005593342	1	0	0
TMEM219	0.7472189	0.794348078	-0.04712919	-0.09212572	0.001533678	0.005593342	4	0	0
COLCA2	0.5129939	0.518977348	-0.00598341	0.122914657	0.001536758	0.005603433	16	0	0
SYNDIG1L	0.3139872	0.30026092	0.013726235	0.196645244	0.001538276	0.005607698	7	0	0
C22orf46	0.0936681	0.107528013	-0.01385995	-0.08678817	0.001538553	0.005607698	7	0	0
HNRNPA3	0.2112299	0.276590472	-0.06536061	-0.17551933	0.001543789	0.005625638	3	0	0
PLK1	0.9074568	0.926628456	-0.01917169	-0.0298367	0.001546452	0.005634198	1	0	0
MAD2L2	0.123095	0.088935762	0.034159221	0.410504989	0.001547363	0.00563523	7	0	0
SPTBN4	0.0914175	0.119699051	-0.02828151	-0.14648979	0.001547184	0.00563523	12	0	0
COQ3	0.3887641	0.452998925	-0.06423485	-0.12470713	0.001548412	0.005637906	9	0	0
FGL2	0.4277299	0.434633055	-0.00690317	0.135700657	0.001548855	0.005638371	7	0	0
GATA4	0.4780114	0.458583493	0.019427918	0.133127559	0.00155	0.005641397	2	0	0
CTNNBIP1	0.0312234	0.028610834	0.002612578	0.090742652	0.001551875	0.005647073	14	0	0
NEU2	0.8617331	0.899225635	-0.03749253	-0.06108533	0.001556907	0.005664236	7	0	0
WDR90	0.1392444	0.168149076	-0.02890467	-0.048518	0.001559971	0.005674232	9	0	0
HID1	0.2125661	0.230842148	-0.01827609	0.071164338	0.001564897	0.005690996	12	0	0
OR8H1	0.3174628	0.389756177	-0.0722934	-0.28778941	0.001565913	0.005693511	1	0	0
DNAI2	0.551964	0.605947204	-0.05398318	-0.01207723	0.001566224	0.005693511	8	0	0
ACTL7A	0.8845748	0.876931961	0.007642851	0.014795042	0.001566692	0.00569406	6	0	0
SEPHS2	0.2911846	0.322957624	-0.03177301	-0.03525294	0.001567219	0.005694822	15	0	0
AS3MT	0.2737388	0.255872087	0.017866685	0.38630937	0.001568602	0.005698691	14	0	0
AATF	0.1173491	0.113663992	0.003685065	-0.04229093	0.001571263	0.005707203	5	0	0
CLDN16	0.2347875	0.277146034	-0.0423585	-0.15464108	0.001572779	0.005710397	6	0	0
ZNF408	0.1465811	0.157239499	-0.01065845	0.027325995	0.001572519	0.005710397	11	0	0
B4GALNT4	0.1756278	0.152831812	0.022795948	0.217144242	0.001573839	0.005713089	23	0	0
WBP2NL	0.480077	0.508317589	-0.02824055	0.003335261	0.001577266	0.00572437	10	0	0
RAPGEF3	0.7749315	0.753545815	0.021385705	0.04567213	0.001578507	0.005727717	8	0	0
GLRX2	0.6303495	0.672960418	-0.04261092	-0.09392631	0.001579798	0.005731241	8	0	0
SLC40A1	0.7591565	0.799086158	-0.03992964	-0.08223053	0.001581264	0.005735399	3	0	0
KRTAP1-4	0.6192719	0.566176745	0.053095127	0.183186873	0.001582616	0.005739144	3	0	0
WRB	0.1878056	0.194367179	-0.00656154	0.054420215	0.001584129	0.005743468	11	0	0
PLSCR2	0.8557521	0.88020694	-0.02445486	-0.04080178	0.001585875	0.005748637	5	0	0
TTK	0.2937124	0.300815021	-0.00710259	-0.12122926	0.00158702	0.005751625	9	0	0
CCDC61	0.0662941	0.057402864	0.008891263	0.160013589	0.001587948	0.005753826	11	0	0
POU4F1	0.1129852	0.062487881	0.050497307	0.936923583	0.001588713	0.005755435	11	0	0
NTNG1	0.2220707	0.19936736	0.022703365	0.308864497	0.001592429	0.005766744	12	0	0
METTL16	0.2495546	0.256666695	-0.00711209	0.051268966	0.001592478	0.005766744	13	0	0
ITCH	0.263273	0.303933843	-0.04066089	-0.07193904	0.001593609	0.005769675	12	0	0
SLC16A4	0.6932241	0.664426894	0.028797203	0.07637626	0.001594824	0.005772907	7	0	0
HPS1	0.3803471	0.402960928	-0.0226138	-0.01466907	0.001595447	0.005773999	9	0	0
MICALL1	0.5469911	0.58425316	-0.03726208	-0.05948002	0.001595813	0.00577416	6	0	0
ZNF565	0.6521555	0.642014453	0.010141004	0.177581191	0.001599462	0.005786195	4	0	0
SLC7A2	0.1954948	0.144206692	0.051288153	0.440447056	0.001602766	0.005796415	6	0	0
AMPD3	0.0529376	0.065341098	-0.01240347	-0.14039573	0.001602934	0.005796415	3	0	0
NEK3	0.3625343	0.338087627	0.024446709	0.122838727	0.001604758	0.005801843	11	0	0
PEA15	0.3669981	0.401214107	-0.03421602	-0.06556416	0.001607133	0.005808088	8	0	0
LAMA1	0.2881437	0.275462063	0.012681681	0.35716503	0.001607038	0.005808088	7	0	0
MSANTD1	0.8170655	0.865043413	-0.04797788	-0.0822493	0.001608763	0.005812808	3	0	0
IFIT3	0.2091648	0.212916088	-0.00375127	0.154788082	0.001609477	0.005814217	4	0	0
TGFBRAP1	0.1771708	0.189223903	-0.01205311	-0.01676011	0.0016138	0.00582866	8	0	0
EEF1D	0.6715215	0.720805723	-0.04928418	-0.08740411	0.001615246	0.005832708	3	0	0
PTH1R	0.6872443	0.712446281	-0.02520196	-0.04998722	0.001621621	0.005852191	6	0	0
OLFM1	0.2481476	0.230820659	0.017326893	0.331284454	0.001621105	0.005852191	13	0	0
ADAM33	0.3649654	0.28797571	0.076989665	0.356142596	0.001621428	0.005852191	7	0	0
KRTAP1-3	0.8311182	0.860983685	-0.02986545	-0.05185747	0.001622318	0.00585353	3	0	0
OSBPL7	0.3031328	0.272100823	0.031032016	0.346621221	0.001623082	0.005855107	6	0	0
KIAA1024	0.1247421	0.075618683	0.049123403	0.728879731	0.001626301	0.005865541	10	0	0
ACAP1	0.3327302	0.378805188	-0.04607496	-0.19299714	0.001628039	0.005870629	8	0	0
CAMSAP3	0.0663099	0.074754141	-0.00844428	-0.11559609	0.00162936	0.00587421	7	0	0
VIM	0.1944162	0.204645708	-0.01022946	0.228094238	0.001633113	0.00588656	10	0	0
EPHA4	0.2844917	0.311301846	-0.02681019	0.016868734	0.001634524	0.005889279	7	0	0

SYNGR2	0.5047416	0.586522638	-0.08178101	-0.21177521	0.001634222	0.005889279	4	0	0
MYCT1	0.5024145	0.569509144	-0.06709462	-0.21070718	0.001635106	0.005890192	4	0	0
RPUSD3	0.2216905	0.251180279	-0.02948976	0.036384981	0.001647879	0.005933822	4	0	0
ADORA2B	0.3306978	0.33645009	-0.00575231	0.204894105	0.001650272	0.005941245	11	0	0
SERP2	0.3444219	0.335286342	0.00913551	0.464339011	0.001650654	0.005941429	8	0	0
CSF3R	0.6644507	0.682208124	-0.0177574	-0.01556248	0.001651392	0.005942874	8	0	0
10-Sep	0.1227958	0.113331694	0.009464086	0.179535575	0.001651719	0.005942874	15	0	0
ANK1	0.131236	0.088310972	0.04292499	0.562839455	0.001656063	0.005957309	6	0	0
TAS2R3	0.8881867	0.911177316	-0.02299063	-0.03688299	0.001657776	0.005962277	5	0	0
CTNNB1	0.8251235	0.85020013	-0.02507666	-0.04297136	0.001661803	0.005974364	7	0	0
AP2A1	0.0745348	0.093004117	-0.01846934	-0.09769308	0.001661787	0.005974364	12	0	0
PPP1R13B	0.2138472	0.23977178	-0.02592457	-0.15584888	0.001664301	0.005981536	10	0	0
TTC19	0.1543911	0.158670833	-0.00427977	0.028683231	0.001664465	0.005981536	9	0	0
BMP2	0.3630846	0.387159324	-0.02407474	0.119624868	0.001665904	0.005984343	10	0	0
TMEM210	0.4991433	0.546218184	-0.04707486	-0.13878049	0.001666957	0.005986894	8	0	0
TBX19	0.8678987	0.88189526	-0.01399656	-0.02147925	0.00166798	0.005988086	7	0	0
CRIP2	0.5413527	0.485735176	0.055617493	0.148463928	0.001668291	0.005988086	5	0	0
AARS	0.1367519	0.135900452	0.000851412	0.106591403	0.00166791	0.005988086	13	0	0
FYCO1	0.2111021	0.237834316	-0.02673226	-0.10253881	0.001670076	0.005993294	10	0	0
SLAMF1	0.5384358	0.595460102	-0.05702429	-0.13138106	0.001671576	0.005995077	6	0	0
PIGR	0.6737316	0.640960137	0.032771476	0.084509804	0.001671494	0.005995077	5	0	0
PYROXD2	0.3196493	0.293648419	0.026000853	0.335133971	0.001671441	0.005995077	4	0	0
PHYKPL	0.3905645	0.420297724	-0.02973321	-0.07038454	0.001674179	0.006003215	9	0	0
UPK1A	0.6979789	0.762275526	-0.06429659	-0.13310181	0.001676477	0.006010251	9	0	0
E2F6	0.2509831	0.302149673	-0.05116654	-0.21176261	0.001679214	0.006017564	11	0	0
TRIM54	0.668088	0.651602139	0.01648583	0.043390268	0.001679708	0.006017564	11	0	0
RMDN1	0.1651168	0.199367978	-0.03425119	-0.10943072	0.001678931	0.006017564	5	0	0
ZNF484	0.0986047	0.11809911	-0.01949437	-0.14079788	0.001680194	0.006017564	3	0	0
LRSAM1	0.0362513	0.053827878	-0.01757662	-0.15077301	0.001679862	0.006017564	9	0	0
PPP1R1B	0.2506582	0.202066585	0.048591652	0.676603894	0.001683655	0.006028756	9	0	0
PUF60	0.4950093	0.548579268	-0.05356999	-0.09835528	0.001684922	0.006032089	8	0	0
UBE4B	0.0498337	0.051099769	-0.00126611	0.001115299	0.001687964	0.006041772	10	0	0
APOL4	0.78924	0.840951666	-0.05171166	-0.10931963	0.001688644	0.006043001	4	0	0
HTR2A	0.5818575	0.605649621	-0.02379211	0.016257433	0.001691575	0.006052283	20	0	0
MED10	0.1529679	0.179290009	-0.02632207	-0.11096082	0.001692082	0.006052888	8	0	0
GALM	0.3361325	0.338607087	-0.00247461	0.239746653	0.001693787	0.00605778	9	0	0
ZNF439	0.3194409	0.30230308	0.017137833	0.457347965	0.001695251	0.006061806	7	0	0
KLHL20	0.3094146	0.338153052	-0.02873841	-0.07910467	0.001695728	0.006062304	14	0	0
CRYAA	0.7044384	0.747980745	-0.04354236	-0.08823429	0.001696319	0.006063209	14	0	0
DAXX	0.8253437	0.859001017	-0.03365728	-0.05698789	0.001696771	0.006063617	1	0	0
MAP6	0.364553	0.36244655	0.002106497	0.455189507	0.001697926	0.006064118	11	0	0
SENP8	0.8111144	0.782116705	0.028997721	0.076003957	0.001697517	0.006064118	5	0	0
SALL4	0.654409	0.608441682	0.045967322	0.127239418	0.001697905	0.006064118	8	0	0
KIF27	0.0320464	0.038203274	-0.00615691	-0.02233779	0.001699975	0.006070226	6	0	0
RHBDL2	0.6153453	0.615065567	0.000279707	0.037948927	0.00170039	0.006070358	6	0	0
UMPS	0.4996574	0.55300556	-0.05334816	-0.12758352	0.001701365	0.006070358	5	0	0
CKMT2	0.4464599	0.388596061	0.057863794	0.248056527	0.001701253	0.006070358	9	0	0
GDF1	0.2641394	0.272990892	-0.00885154	0.167629517	0.00170126	0.006070358	11	0	0
GPR1	0.7091273	0.69425088	0.014876467	0.233372481	0.001704558	0.006080538	4	0	0
GPR22	0.8049827	0.859174906	-0.05419217	-0.09248922	0.001706227	0.006085282	4	0	0
DYDC1	0.2543646	0.245476168	0.008888451	0.359686829	0.001707023	0.006086911	13	0	0
ACTRT3	0.0955708	0.101758652	-0.00618786	0.262126259	0.00170782	0.006088545	2	0	0
SNRPN	0.5921353	0.60131433	-0.00917908	-0.01798746	0.001709627	0.006093774	8	0	0
FRMD4B	0.1005278	0.068283488	0.032244354	0.530850038	0.001712864	0.006104098	5	0	0
MSH4	0.7292935	0.792397402	-0.06310386	-0.12852064	0.001721347	0.006133109	12	0	0
FBXL22	0.6195893	0.577749107	0.04184022	0.112703328	0.001726281	0.00614947	10	0	0
SYT11	0.2142541	0.252554158	-0.03830002	-0.13575564	0.001729148	0.006157238	16	0	0
KRTAP16-1	0.6436618	0.678724125	-0.03506236	-0.09809951	0.001728961	0.006157238	6	0	0
DCLK1	0.3088953	0.277365983	0.031529325	0.312376269	0.001729816	0.006158393	9	0	0
HMGB2	0.1471305	0.176417638	-0.02928712	-0.05637594	0.001730511	0.006159645	6	0	0
ZNF773	0.2816758	0.243657206	0.038018613	1.013027078	0.001731478	0.006161864	8	0	0
CHRND	0.4486408	0.408277849	0.040362982	0.26271409	0.00173495	0.006169321	10	0	0
DLEC1	0.2319657	0.194356788	0.037608947	0.863104994	0.00173455	0.006169321	8	0	0

SRI	0.659171	0.701548782	-0.04237783	-0.09130061	0.00173473	0.006169321	3	0	0
GCOM1	0.2930813	0.318501169	-0.02541987	-0.00948216	0.00173474	0.006169321	11	0	0
TMEM119	0.5980719	0.575929753	0.022142122	0.073834953	0.001736422	0.006173331	5	0	0
UBE2J1	0.2074871	0.24053243	-0.03304536	-0.05003192	0.001740168	0.006184201	11	0	0
ANKRD34C	0.1962797	0.158738954	0.037540791	0.371736559	0.001739865	0.006184201	9	0	0
GRHL1	0.8318095	0.878473419	-0.04666396	-0.08018901	0.001742094	0.006189818	3	0	0
CYP27B1	0.8438522	0.867494299	-0.02364212	-0.03902336	0.001752133	0.006222976	3	0	0
HCFC1R1	0.1731994	0.181811778	-0.0086124	-0.06609424	0.001752467	0.006222976	13	0	0
STXBP2	0.2597089	0.267312133	-0.00760319	-0.07386014	0.001751923	0.006222976	7	0	0
ISOC1	0.317894	0.323914223	-0.00602023	0.003826834	0.001756112	0.006234685	6	0	0
CNP	0.1024331	0.121237826	-0.01880476	-0.12421556	0.001758656	0.006241911	9	0	0
ZNF493	0.3609694	0.363791133	-0.00282169	0.336227331	0.001758843	0.006241911	11	0	0
SPATA13	0.2075217	0.202015069	0.005506627	0.272990732	0.00175937	0.006242546	11	0	0
KLRF2	0.8574977	0.900046756	-0.04254905	-0.07038513	0.001760243	0.006244405	5	0	0
LNX1	0.5027274	0.487983096	0.014744336	0.370852741	0.001761243	0.006246719	2	0	0
ARHGAP27	0.7573662	0.79854504	-0.0411788	-0.07580664	0.001762144	0.006248679	3	0	0
EPHA10	0.140393	0.099151625	0.041241355	0.489687887	0.00176383	0.00625342	11	0	0
PPP1R14A	0.3233076	0.291681123	0.031626454	0.621946776	0.001766081	0.006260163	11	0	0
SYNJ2	0.2440065	0.268744849	-0.02473834	0.079864419	0.001766559	0.006260622	7	0	0
STAC	0.2654728	0.279512925	-0.01404016	0.023713184	0.001768145	0.006265005	7	0	0
ATP6V1G2	0.7014542	0.795983668	-0.09452948	-0.18220597	0.001771237	0.006274719	3	0	0
PLEKHG3	0.1183852	0.129750786	-0.01136557	0.044431416	0.001773461	0.006281359	14	0	0
CTDSP2	0.1016766	0.132434617	-0.03075806	-0.1232171	0.001776363	0.006290393	12	0	0
CD302	0.2437875	0.174217681	0.069569841	0.409672482	0.001784943	0.006318282	8	0	0
CYP2F1	0.5856266	0.656182003	-0.07055544	-0.16635837	0.001784818	0.006318282	6	0	0
SNAPC1	0.1769293	0.194847291	-0.01791802	-0.04757296	0.001787206	0.006325046	7	0	0
KLK13	0.2306702	0.181622015	0.049048185	0.412758424	0.001788091	0.00632693	9	0	0
C1orf56	0.1800439	0.221222613	-0.04117867	-0.1108858	0.001790317	0.006329811	7	0	0
MAN2A1	0.1365474	0.161880713	-0.02533336	-0.10377552	0.001789404	0.006329811	8	0	0
SNX11	0.7095681	0.762943526	-0.05337543	-0.12726094	0.001790079	0.006329811	5	0	0
ZNF100	0.5724785	0.603251954	-0.03077345	-0.01846841	0.001789916	0.006329811	4	0	0
TRIM68	0.3622112	0.394927125	-0.03271595	-0.0711235	0.001791495	0.00633148	11	0	0
GPR135	0.1753786	0.138151794	0.037226817	0.860549502	0.00179141	0.00633148	11	0	0
MSX2	0.1607823	0.121707563	0.039074719	0.549491292	0.001795051	0.006342795	13	0	0
KIAA1522	0.5430466	0.545488088	-0.00244147	0.01703684	0.0017974	0.006349847	7	0	0
KDM4A	0.109137	0.136770695	-0.0276337	-0.06955719	0.00179863	0.006352938	15	0	0
PDE6B	0.6280363	0.673211702	-0.04517537	-0.10811765	0.001801277	0.006360576	14	0	0
PHAX	0.5455063	0.576402266	-0.03089594	-0.00320805	0.001801501	0.006360576	12	0	0
RAB7A	0.2634596	0.28167007	-0.01821046	-0.07672832	0.001803713	0.006364628	11	0	0
CCDC6	0.107969	0.127518083	-0.01954908	-0.04335659	0.001803668	0.006364628	8	0	0
CPXM2	0.7143868	0.767198794	-0.05281197	-0.10380657	0.00180318	0.006364628	3	0	0
ZNF175	0.241126	0.225765945	0.015360061	0.630307131	0.00180457	0.006366397	4	0	0
MYL5	0.4038631	0.419241309	-0.01537824	-0.08126684	0.001805804	0.006368247	10	0	0
LDLR	0.2558204	0.235392996	0.020427362	0.230140493	0.001805653	0.006368247	12	0	0
COLQ	0.5778429	0.611198624	-0.03335573	-0.08259211	0.001810075	0.006382053	4	0	0
ADH6	0.7825241	0.742393899	0.040130191	0.077912463	0.001814466	0.006396278	4	0	0
RGL4	0.8572093	0.890897566	-0.03368829	-0.05661809	0.001816029	0.006400531	4	0	0
PRLH	0.717198	0.760083743	-0.0428857	-0.07992369	0.001816388	0.006400537	4	0	0
CHRNB3	0.7492737	0.790790128	-0.04151645	-0.07279894	0.001816932	0.006401197	10	0	0
MMP12	0.7065714	0.769785497	-0.06321415	-0.12226274	0.00181922	0.006407998	8	0	0
B4GALT2	0.3357654	0.369924952	-0.03415959	-0.09214349	0.001820183	0.006410133	9	0	0
TP53AIP1	0.737027	0.796284885	-0.05925787	-0.11662513	0.001821373	0.006411804	17	0	0
CDIP1	0.2627814	0.25733354	0.005447828	0.432257168	0.001821284	0.006411804	15	0	0
ITIH2	0.657157	0.723778561	-0.0666216	-0.13844183	0.001824767	0.006421232	6	0	0
PGLYRP1	0.5508258	0.500898461	0.049927311	0.238631143	0.001824604	0.006421232	13	0	0
PRF1	0.5729986	0.610926759	-0.0379282	-0.09970667	0.00182669	0.006426737	10	0	0
PXDN	0.2150977	0.167425701	0.047671967	0.566551699	0.001827156	0.006427117	7	0	0
MFAP1	0.2779695	0.299463633	-0.02149417	-0.03528088	0.001827721	0.006427844	14	0	0
SLC5A7	0.3626556	0.314892851	0.047762738	0.442817018	0.001833128	0.006443579	10	0	0
JMJD8	0.1536493	0.176547271	-0.02289802	0.215648639	0.001833151	0.006443579	10	0	0
RAP1GAP2	0.258983	0.22432269	0.034660328	0.459183857	0.001833273	0.006443579	8	0	0
ULBP1	0.0792264	0.067196657	0.012029761	0.422199092	0.001836084	0.006452193	7	0	0
ATP9A	0.345857	0.346818897	-0.00096192	0.134746988	0.001837956	0.006457507	6	0	0

TANGO6	0.2304044	0.250087882	-0.01968343	-0.08133174	0.001840566	0.006465412	12	0	0
CAPN2	0.6708882	0.732727	-0.06183877	-0.13347976	0.001840989	0.00646563	4	0	0
UBD	0.6069373	0.677354212	-0.07041696	-0.15180434	0.001841934	0.006467682	5	0	0
COL20A1	0.4856858	0.525359863	-0.0396741	-0.0692477	0.001845563	0.006479158	7	0	0
CLEC18C	0.6266341	0.674094653	-0.04746059	-0.10373161	0.001850236	0.006494291	1	0	0
PLLP	0.267889	0.253730401	0.014158588	0.25486121	0.001851928	0.00649896	4	0	0
CATSPER3	0.8009348	0.832573529	-0.03163869	-0.05727894	0.001854102	0.006504045	3	0	0
FBXO39	0.2956848	0.231239462	0.06444533	0.542866682	0.001854002	0.006504045	8	0	0
KCNK4	0.4254854	0.39971964	0.025765794	0.376689215	0.00185771	0.006515424	11	0	0
PDP2	0.5506184	0.596093049	-0.04547469	-0.19180856	0.001858214	0.006515919	3	0	0
C9orf40	0.0990867	0.101557228	-0.00247054	-0.02680777	0.001860974	0.006524323	11	0	0
PREX2	0.2489795	0.199412592	0.049566893	0.551233613	0.00186164	0.006525382	13	0	0
C1QTNF9	0.7803523	0.800204524	-0.01985219	-0.03919504	0.001862378	0.006526693	5	0	0
MAFB	0.1474319	0.114124556	0.033307305	0.269972545	0.001866504	0.006539874	7	0	0
SERINC2	0.701134	0.739584919	-0.03845093	-0.07735224	0.001867042	0.006540482	6	0	0
NUDT1	0.0569846	0.074499332	-0.01751469	-0.10781465	0.001870672	0.006550819	7	0	0
DDX19B	0.066591	0.064536104	0.002054919	0.118211137	0.001870724	0.006550819	14	0	0
SNRNP48	0.340483	0.385822233	-0.04533923	-0.07318308	0.001872109	0.006554392	8	0	0
GOLGA4	0.1854481	0.207800733	-0.02235261	-0.07594399	0.001873215	0.006556984	11	0	0
DMC1	0.133944	0.149630584	-0.01568661	0.040254059	0.001874584	0.006560496	7	0	0
AADACL4	0.7035322	0.752096189	-0.04856397	-0.1054088	0.001876126	0.006564611	7	0	0
CLIP4	0.7873823	0.830506936	-0.04312465	-0.08265265	0.001877004	0.006565122	9	0	0
ERAP2	0.728675	0.736377493	-0.00770245	0.113421809	0.001876955	0.006565122	5	0	0
SAA4	0.6239866	0.692178559	-0.06819193	-0.18493987	0.001881435	0.006579336	2	0	0
PFN2	0.763154	0.784341505	-0.02118755	-0.0801972	0.001882616	0.006582185	6	0	0
PRG4	0.6030616	0.67900081	-0.07593922	-0.16759474	0.001883109	0.006582622	4	0	0
FANCE	0.2260732	0.200970129	0.025103057	0.216588665	0.001885325	0.006588058	7	0	0
AQP5	0.2623111	0.273121149	-0.01081008	0.215887722	0.001885398	0.006588058	7	0	0
ZNF331	0.2482059	0.206976047	0.041229827	0.467734505	0.001886187	0.006589529	9	0	0
KIF7	0.3927232	0.407745607	-0.01502244	0.084765881	0.001887514	0.006592881	9	0	0
OBP2B	0.5004722	0.564547293	-0.06407509	-0.17113575	0.001894362	0.006615515	4	0	0
GPR61	0.6727469	0.709213198	-0.03646627	-0.06956104	0.001897861	0.006625154	10	0	0
MYOT	0.8117914	0.841689781	-0.02989834	-0.05135045	0.001897823	0.006625154	2	0	0
GDF7	0.1328451	0.05531414	0.077530936	1.297463997	0.001902941	0.006637719	13	0	0
TMEM178A	0.3015976	0.276663346	0.024934265	0.169336352	0.001902903	0.006637719	3	0	0
PRH1	0.8725157	0.914929595	-0.0424139	-0.06772162	0.001901991	0.006637719	1	0	0
IGDCC4	0.3601665	0.389488057	-0.02932158	0.114245888	0.001902324	0.006637719	5	0	0
BSCL2	0.1665918	0.10655387	0.060037935	0.889300049	0.001903334	0.006637798	8	0	0
ARL4A	0.1465573	0.15705311	-0.01049582	0.290541241	0.001904668	0.006639868	14	0	0
ADCY1	0.350398	0.350848394	-0.00045042	0.159402228	0.001904482	0.006639868	10	0	0
LRMP	0.8016353	0.848174421	-0.04653915	-0.08043934	0.001905302	0.006640787	1	0	0
TSPAN18	0.5381096	0.469823822	0.06828574	0.183079917	0.001906298	0.006642968	2	0	0
TMEM185B	0.2153805	0.243072466	-0.02769194	-0.0914793	0.001907222	0.006643609	11	0	0
ENG	0.3780613	0.340569711	0.037491551	0.396865877	0.001907119	0.006643609	4	0	0
IL24	0.7581075	0.807053794	-0.04894627	-0.08933441	0.001909765	0.006651173	6	0	0
EDIL3	0.2133585	0.196043749	0.017314784	0.415422367	0.001910443	0.006652243	12	0	0
SLBP	0.0259767	0.028464666	-0.00248795	-0.0535089	0.001912127	0.006656816	5	0	0
TMEM59	0.1125585	0.149719803	-0.03716127	-0.13484453	0.001912523	0.0066569	16	0	0
HHIPL2	0.5260772	0.573100081	-0.04702286	-0.12153987	0.001921603	0.006687209	6	0	0
ZNF318	0.1035764	0.140325434	-0.03674906	-0.11836691	0.00192461	0.006696375	4	0	0
MYOZ2	0.7732494	0.803473876	-0.03022451	-0.05207332	0.001926616	0.006702053	5	0	0
TRDN	0.5808512	0.621929475	-0.04107829	-0.07740336	0.001927204	0.006702799	8	0	0
EPAS1	0.8974502	0.914751564	-0.01730132	-0.0272473	0.001928012	0.006704308	1	0	0
ZNF354C	0.0888283	0.055181658	0.03364664	0.733514651	0.001928753	0.006705586	8	0	0
TIAL1	0.3525163	0.379826108	-0.02730979	-0.0271281	0.001931661	0.006714394	10	0	0
KRTAP4-8	0.5458218	0.637506075	-0.09168424	-0.21816174	0.001938312	0.006736207	2	0	0
MLLT1	0.3028407	0.331446925	-0.02860625	-0.01012403	0.001943924	0.006754403	3	0	0
TFAP2C	0.1216686	0.074631468	0.04703712	0.436561066	0.001944891	0.006756454	12	0	0
GCLC	0.7996648	0.856890559	-0.05722575	-0.10061592	0.001946372	0.006760289	2	0	0
ADAMTS14	0.2624711	0.241065182	0.021405914	0.455789668	0.001951299	0.00677609	9	0	0
PER2	0.3817371	0.422554481	-0.04081734	-0.1469607	0.001955213	0.006787052	4	0	0
PSG2	0.8908224	0.921527719	-0.0307053	-0.0484746	0.001955172	0.006787052	2	0	0
PTBP3	0.0435422	0.055690586	-0.01214842	-0.09089344	0.001960023	0.006802433	9	0	0

TRMT1	0.0428127	0.04833343	-0.00552068	-0.12344028	0.001960856	0.006804007	6	0	0
TBCK	0.8374063	0.810500148	0.026906201	0.046550344	0.00196288	0.006807871	1	0	0
PLEKHG7	0.8204966	0.862755193	-0.04225862	-0.07336159	0.001963108	0.006807871	3	0	0
C14orf39	0.2654909	0.221238054	0.044252818	0.256543515	0.001962906	0.006807871	7	0	0
ZFP36L1	0.3551103	0.389376651	-0.03426633	-0.0479938	0.001965201	0.006813812	8	0	0
MED22	0.1746058	0.216974396	-0.04236859	-0.10458493	0.001966063	0.006815483	10	0	0
XRCC2	0.2169501	0.198457254	0.018492825	0.099997759	0.001966592	0.006815998	9	0	0
TFAP2D	0.1190075	0.075537197	0.043470351	0.641615397	0.001972853	0.006835059	6	0	0
SAA1	0.5575656	0.639285653	-0.08172003	-0.19406612	0.001972602	0.006835059	1	0	0
TP53I3	0.6047756	0.571059619	0.033716003	0.187193836	0.001974992	0.006841147	6	0	0
PIRT	0.6840206	0.716948715	-0.03292812	-0.06823085	0.001975859	0.006842829	10	0	0
SYNJ2BP-CC	0.5183685	0.529287826	-0.01091928	0.01983078	0.001978594	0.006849655	7	0	0
SYNJ2BP	0.5183685	0.529287826	-0.01091928	0.01983078	0.001978594	0.006849655	7	0	0
TNFRSF4	0.5137309	0.514307714	-0.00057679	-0.02883928	0.001981489	0.006858352	5	0	0
LANCL2	0.443255	0.474983399	-0.03172843	-0.04604818	0.001983901	0.006865378	6	0	0
MUC17	0.5858363	0.613805288	-0.02796903	-0.0459954	0.001985255	0.006868738	8	0	0
MS4A7	0.528795	0.600415086	-0.07162006	-0.20663249	0.00198637	0.006869945	4	0	0
MS4A14	0.528795	0.600415086	-0.07162006	-0.20663249	0.00198637	0.006869945	4	0	0
PLIN3	0.164157	0.169537422	-0.00538038	0.093357181	0.001991495	0.006886341	11	0	0
MRPL3	0.2028379	0.25138714	-0.04854925	-0.15518641	0.001993239	0.006891045	14	0	0
SBF2	0.0892588	0.108358802	-0.01909998	-0.02911528	0.001995521	0.006897605	13	0	0
GOLGA7B	0.078968	0.088071906	-0.00910387	0.065764607	0.001997427	0.00690286	9	0	0
BCL2L2	0.8221185	0.833365629	-0.01124713	-0.01750055	0.002000475	0.006912062	2	0	0
NECAP2	0.2240716	0.253897759	-0.02982611	-0.05631405	0.00200367	0.006921769	14	0	0
SMIM14	0.1646399	0.188737684	-0.02409781	0.001306227	0.002004835	0.00692446	9	0	0
DIP2C	0.3695863	0.363653239	0.005933075	0.18079961	0.002005736	0.006926239	6	0	0
LCE1C	0.4961443	0.539463522	-0.04331926	-0.17479298	0.0020081	0.006933067	2	0	0
OLR1	0.7017072	0.755432105	-0.05372495	-0.10616452	0.002010812	0.006939937	8	0	0
DNAAF2	0.129585	0.141379185	-0.01179422	-0.08056156	0.002010864	0.006939937	10	0	0
LRRC3C	0.7141362	0.757400473	-0.04326429	-0.08933581	0.00201157	0.006941038	5	0	0
EPX	0.6229205	0.641761334	-0.01884082	-0.01679838	0.002013964	0.006947964	7	0	0
HERPUD2	0.081714	0.106134453	-0.02442043	-0.19062705	0.002014397	0.006948121	11	0	0
UHRF1	0.7632643	0.787156433	-0.02389217	-0.04628736	0.002016116	0.006952713	7	0	0
HOXA1	0.1026606	0.129612021	-0.02695141	0.007796904	0.002019396	0.006962685	14	0	0
B3GALT4	0.2357734	0.259315779	-0.02354237	0.200030554	0.002022041	0.006969125	16	0	0
ATG16L2	0.0987209	0.130581449	-0.03186058	-0.16825656	0.002021903	0.006969125	10	0	0
RNF183	0.738294	0.784770781	-0.04647674	-0.09384387	0.002023864	0.006974069	4	0	0
PIP5K1C	0.1350459	0.14834258	-0.01329667	0.030260548	0.002027776	0.006986207	8	0	0
RGS9	0.2369075	0.245943657	-0.00903619	-0.09360357	0.002028935	0.006988858	6	0	0
ST8SIA6	0.3161447	0.30761329	0.008531455	0.333390993	0.002033236	0.007002327	4	0	0
ADAM8	0.4349171	0.43066947	0.004247628	0.152498208	0.002035646	0.00700928	9	0	0
CD2	0.6496151	0.705029649	-0.05541453	-0.15445212	0.00203672	0.007011632	6	0	0
STON2	0.2491086	0.271771141	-0.02266256	0.024250743	0.002037193	0.007011917	5	0	0
GOLGA8A	0.3453671	0.386394141	-0.04102707	-0.13992125	0.002041892	0.007025392	4	0	0
GOLGA8B	0.3453671	0.386394141	-0.04102707	-0.13992125	0.002041892	0.007025392	4	0	0
ICAM3	0.5323743	0.572136636	-0.03976229	-0.12212054	0.002043591	0.007029889	11	0	0
PDCL2	0.7626943	0.82349122	-0.06079688	-0.10800678	0.002044043	0.007030097	4	0	0
KCNK10	0.239961	0.2505078	-0.01054683	0.324009035	0.002045079	0.007032311	10	0	0
FEM1C	0.2346182	0.249525906	-0.01490775	-0.14039761	0.002046397	0.007035493	5	0	0
GRHL3	0.1802326	0.200407556	-0.02017492	-0.16178761	0.002047732	0.007038736	18	0	0
SSMEM1	0.797493	0.841718589	-0.04422561	-0.07684055	0.002048428	0.007039779	4	0	0
ACP1	0.2010568	0.145247721	0.055809108	0.771024133	0.002050257	0.007043367	7	0	0
GLYAT	0.6648642	0.737848702	-0.07298451	-0.14149896	0.002050179	0.007043367	3	0	0
MFAP2	0.5214701	0.534775633	-0.01330556	0.043569854	0.002053504	0.00705182	6	0	0
HBP1	0.0767397	0.058105984	0.018633727	0.169328145	0.002053362	0.00705182	5	0	0
SDCBP2	0.5638148	0.603282442	-0.03946767	-0.10893851	0.002054609	0.007054261	6	0	0
NKX3-2	0.1375926	0.095666279	0.041926354	0.435379883	0.002057127	0.007061555	10	0	0
PNMA2	0.1089119	0.069556898	0.039355051	0.659136874	0.00205955	0.007068522	11	0	0
CCL17	0.7139643	0.753298802	-0.03933446	-0.07002105	0.002063105	0.007078014	7	0	0
GIMAP5	0.5155891	0.555698385	-0.04010929	-0.0969181	0.002068182	0.007092932	3	0	0
ZNF664	0.2441415	0.237582422	0.006559076	-0.07965303	0.002068244	0.007092932	8	0	0
KATNA1	0.231409	0.245052975	-0.01364393	-0.00696434	0.002070592	0.007099626	8	0	0
SPACA1	0.6291828	0.583403497	0.045779323	0.109800187	0.002073589	0.007108542	7	0	0

ZNF649	0.204007	0.158895465	0.045111563	0.59525835	0.002074052	0.00710877	11	0	0
C4orf19	0.1452728	0.133585686	0.011687127	0.262185775	0.002075607	0.007111382	7	0	0
PDXDC1	0.4073655	0.433735178	-0.02636968	-0.09564471	0.002075541	0.007111382	2	0	0
RPEL1	0.3693407	0.35454251	0.014798144	0.234620741	0.002080781	0.007127746	6	0	0
C1orf159	0.2752243	0.293868956	-0.01864468	0.034699628	0.002083081	0.007131542	6	0	0
BTNL9	0.5212359	0.479915504	0.04132038	0.140323291	0.002082613	0.007131542	8	0	0
MDFI	0.5237219	0.550438363	-0.02671644	-0.02616425	0.002082822	0.007131542	4	0	0
SP5	0.0775657	0.052256652	0.025309085	0.427668017	0.002085049	0.007136917	15	0	0
EFS	0.2180939	0.175899345	0.04219453	0.463489328	0.00208736	0.007143463	13	0	0
EBF3	0.0917493	0.067113857	0.024635458	0.411032991	0.002088728	0.00714678	9	0	0
WBP1L	0.262419	0.253726242	0.008692793	0.125001517	0.002089697	0.007147892	8	0	0
RPAP1	0.2798643	0.301270796	-0.02140648	-0.00826203	0.00208985	0.007147892	14	0	0
EIF5A	0.1485138	0.15534152	-0.00682768	0.033201574	0.002091977	0.007153805	15	0	0
TULP3	0.149415	0.194421886	-0.04500691	-0.13995056	0.002094806	0.007162112	8	0	0
NGB	0.5063945	0.481072124	0.025322396	0.198141115	0.002096659	0.00716708	10	0	0
ZNF112	0.673881	0.742152386	-0.06827143	-0.14683919	0.002098483	0.007171949	2	0	0
EMILIN2	0.2363226	0.280127606	-0.043805	0.016220212	0.002098924	0.007172091	8	0	0
VTCN1	0.5638704	0.631123049	-0.0672527	-0.15979395	0.002101873	0.007180798	9	0	0
RPL17-C18c	0.2675212	0.291826791	-0.02430557	-0.07725757	0.0021023	0.007180889	12	0	0
SSTR1	0.1961621	0.165058181	0.031103962	0.411733759	0.002102815	0.007181281	10	0	0
C17orf99	0.6560688	0.63484425	0.021224525	0.062178458	0.002107093	0.007194522	6	0	0
CABLES2	0.3211979	0.352613348	-0.03141544	-0.10203943	0.00210833	0.007197373	6	0	0
ITIH5	0.1023423	0.065986323	0.036355929	0.53190073	0.002117503	0.007227313	12	0	0
ALPI	0.7401538	0.795351655	-0.05519788	-0.10590958	0.002119068	0.00723128	6	0	0
LCN1	0.6409413	0.68854497	-0.04760364	-0.10235949	0.002122043	0.007240055	4	0	0
ANXA13	0.7948609	0.771755347	0.023105517	0.041931271	0.002122735	0.007241037	5	0	0
FAM83D	0.3936692	0.411258909	-0.01758968	-0.11972822	0.002124171	0.007244557	9	0	0
HIST1H2BA	0.8192641	0.863148292	-0.04388419	-0.08041065	0.002128923	0.007259385	9	0	0
GNG8	0.243777	0.261836388	-0.01805943	-0.16796865	0.002130901	0.007263369	9	0	0
HOXB13	0.1185636	0.073114761	0.045448838	0.654869825	0.002133123	0.007269561	10	0	0
GUCA2A	0.6334563	0.610933599	0.022522688	0.053537188	0.002133546	0.007269619	10	0	0
CENPC	0.1563692	0.184740451	-0.02837123	-0.06009396	0.002135424	0.007274638	9	0	0
TEDDM1	0.8046659	0.831489503	-0.02682361	-0.04806177	0.002138025	0.007282116	6	0	0
PPP1CC	0.3798695	0.353998967	0.025870542	0.036251569	0.002140119	0.007287864	6	0	0
PDE7B	0.3450166	0.380511932	-0.03549529	-0.08063388	0.002140682	0.007288396	11	0	0
ZNF660	0.8711398	0.89099417	-0.01985434	-0.03344544	0.002141879	0.007289706	4	0	0
UNC5C	0.1131447	0.068728496	0.044416179	0.709992663	0.002141744	0.007289706	17	0	0
CDCP2	0.4484275	0.488960963	-0.04053348	-0.1670358	0.002144034	0.007294271	4	0	0
UGT1A7	0.8531034	0.888235539	-0.03513215	-0.05932887	0.002143935	0.007294271	4	0	0
THSD4	0.4655877	0.506817834	-0.04123013	-0.23043677	0.002146703	0.007301966	3	0	0
TJP3	0.4409711	0.441726379	-0.00075527	-0.07103514	0.002150148	0.007312298	8	0	0
CD81	0.7734433	0.743445972	0.029997312	0.057059989	0.002150912	0.007313508	13	0	0
GLIS2	0.1148954	0.080749725	0.034145657	0.475753125	0.002152256	0.007316692	4	0	0
GABRA1	0.1518657	0.111178123	0.040687592	0.417825601	0.002153192	0.007318487	12	0	0
SMAP2	0.5683571	0.52230121	0.046055881	0.139879793	0.00215845	0.007334968	2	0	0
LY96	0.5227631	0.591531616	-0.0687685	-0.24162213	0.002160972	0.007342147	5	0	0
TYRP1	0.6549668	0.591013636	0.063953185	0.145884634	0.002163989	0.007351004	1	0	0
GIGYF1	0.7517392	0.789067942	-0.0373287	-0.07426548	0.002166132	0.007356891	8	0	0
IQGAP1	0.1879393	0.227298057	-0.03935876	-0.08617828	0.002167108	0.007358812	7	0	0
SLC29A4	0.7717567	0.829365089	-0.05760842	-0.10257884	0.002167957	0.007360303	1	0	0
PPP1R18	0.3863558	0.407299089	-0.02094325	-0.08684459	0.002176163	0.007386764	24	0	0
SMAD9	0.6061967	0.601835886	0.004360828	0.07209099	0.002180472	0.007399991	8	0	0
LDB2	0.4698131	0.46620877	0.003604359	0.105846361	0.002182615	0.007405862	7	0	0
ZNHIT6	0.3407461	0.388772371	-0.04802631	-0.0623619	0.002183074	0.007406017	7	0	0
GCSH	0.1173932	0.125592546	-0.00819932	0.065319455	0.002184426	0.007409202	10	0	0
HSBP1	0.536598	0.563984759	-0.02738679	-0.09559558	0.00218493	0.007409511	5	0	0
ZNF813	0.071416	0.051480747	0.0199353	0.364138506	0.002187156	0.007415657	5	0	0
HSD17B13	0.6809474	0.663703242	0.01724414	0.073758084	0.002189165	0.007421066	5	0	0
EPS15	0.1820214	0.191263027	-0.00924158	-0.10282346	0.002190892	0.007425517	8	0	0
NDST2	0.4829793	0.500547524	-0.01756826	-0.00046124	0.002194268	0.007435556	4	0	0
ZNRF4	0.8224192	0.876244326	-0.0538251	-0.0961921	0.002194769	0.007435849	9	0	0
CLEC12A	0.6565907	0.722691991	-0.06610132	-0.15248287	0.00219655	0.007440478	6	0	0
CDK3	0.6846451	0.748662046	-0.0640169	-0.12778366	0.002197003	0.007440605	9	0	0

SMIM20	0.348832	0.329569643	0.019262381	0.028749694	0.002199332	0.007447087	5	0	0
TAGLN	0.5539852	0.504707157	0.049278052	0.193527293	0.002201242	0.007452148	11	0	0
NELFB	0.2966407	0.316943369	-0.02030267	-0.03807088	0.00220208	0.007453576	3	0	0
CDH2	0.1206051	0.085892261	0.034712858	0.504635911	0.002203941	0.00745847	11	0	0
BUB1B	0.4373339	0.460049809	-0.02271589	-0.04137297	0.002205784	0.007463299	6	0	0
PDLIM2	0.4265775	0.439439462	-0.01286195	0.045398115	0.00220626	0.0074635	9	0	0
ARPP21	0.4112284	0.455325279	-0.04409684	-0.05271	0.002207493	0.007464856	10	0	0
IBSP	0.6013501	0.602391301	-0.00104122	0.040628356	0.002207445	0.007464856	7	0	0
MICAL1	0.7313023	0.692377721	0.038924532	0.081099771	0.002209365	0.007469777	7	0	0
CLDN10	0.7820993	0.816380636	-0.03428134	-0.06659165	0.002210772	0.007473125	11	0	0
OPTN	0.1712603	0.199537613	-0.02827734	-0.05191556	0.002213199	0.007479921	15	0	0
BCAR1	0.62575	0.669278031	-0.04352804	-0.1081751	0.002223263	0.007512516	7	0	0
ZFYVE27	0.2354534	0.267902278	-0.03244891	-0.07511273	0.002223784	0.007512863	5	0	0
AGBL4	0.2898291	0.252799768	0.037029375	0.420885921	0.002224663	0.007513004	17	0	0
LY6E	0.2713793	0.290111698	-0.01873241	-0.12283089	0.002224562	0.007513004	10	0	0
C5orf46	0.762362	0.794402647	-0.0320407	-0.05638008	0.002225097	0.007513052	5	0	0
NDN	0.4883344	0.511498103	-0.02316365	-0.02427374	0.00223068	0.007530488	12	0	0
SERPINA1	0.4889508	0.526998259	-0.03804747	-0.08864913	0.002231585	0.007532124	8	0	0
COL18A1	0.1784433	0.159159712	0.019283625	0.227478551	0.002233479	0.0075371	9	0	0
EXTL1	0.7446775	0.76638118	-0.0217037	-0.04040918	0.002235478	0.007542427	3	0	0
TENM3	0.1486811	0.104434286	0.044246862	0.502958927	0.002236463	0.00754433	7	0	0
COL28A1	0.6944219	0.726976086	-0.03255417	-0.04188783	0.002238697	0.007550446	4	0	0
RHD	0.7096346	0.670305703	0.03932892	0.081081415	0.002239635	0.00755219	1	0	0
SIGMAR1	0.0362436	0.039031882	-0.00278826	0.205005359	0.002241035	0.007552649	6	0	0
C16orf86	0.0994769	0.034433066	0.065043792	1.450739389	0.002240683	0.007552649	11	0	0
USP29	0.7347632	0.759011259	-0.02424802	-0.0555829	0.002241033	0.007552649	11	0	0
SLC5A10	0.7191858	0.780270737	-0.06108492	-0.10464724	0.002244005	0.00756124	3	0	0
ARL2	0.3688312	0.335606105	0.033225131	0.064010557	0.002244892	0.007562807	6	0	0
NEBL	0.1146879	0.099607113	0.015080806	0.414880184	0.002248149	0.007571081	12	0	0
TSG101	0.0904649	0.100290927	-0.00982601	-0.00613004	0.002248192	0.007571081	11	0	0
APOBEC4	0.661485	0.691481573	-0.02999657	-0.05613995	0.002251058	0.00757931	7	0	0
SH2D4A	0.2345501	0.215994262	0.018555883	0.207279032	0.002255347	0.007592324	10	0	0
CYTL1	0.5328493	0.487109433	0.045739829	0.478251299	0.002256786	0.007595743	7	0	0
RFESD	0.2749554	0.30810359	-0.03314823	-0.07284065	0.002266063	0.007622675	12	0	0
TRPV6	0.5148888	0.574472858	-0.05958407	-0.13994632	0.002265989	0.007622675	10	0	0
GUCA1A	0.5784036	0.60968394	-0.03128031	-0.07410945	0.002267844	0.007627236	5	0	0
C16orf96	0.6357029	0.682342441	-0.04663957	-0.10513035	0.00227136	0.007636198	4	0	0
PALM	0.24972	0.233770411	0.015949596	0.418905256	0.002271192	0.007636198	7	0	0
TXNRD3	0.2722644	0.305370419	-0.03310602	0.023017602	0.002272389	0.007636794	8	0	0
FAM131A	0.2756203	0.300021504	-0.02440117	-0.10305587	0.002272348	0.007636794	12	0	0
FGF11	0.4044753	0.455009295	-0.05053403	-0.15465451	0.002279707	0.007658517	11	0	0
GINS1	0.0776146	0.098865566	-0.02125096	-0.1089784	0.002279694	0.007658517	8	0	0
PDP1	0.4333179	0.443658281	-0.01034039	-0.08118978	0.002280846	0.007660909	4	0	0
SRD5A2	0.4886795	0.445493386	0.04318609	0.321415735	0.002286941	0.007678506	12	0	0
PPAT	0.0638821	0.069208988	-0.00532689	-0.07907574	0.002286593	0.007678506	12	0	0
CERS5	0.0430773	0.038872066	0.004205249	0.125595521	0.002288816	0.007683362	8	0	0
SOSTDC1	0.6674252	0.711466183	-0.04404098	-0.09916068	0.002292135	0.007693063	3	0	0
IL4	0.7615607	0.772708536	-0.01114786	-0.01150472	0.002299279	0.007713854	7	0	0
TRPM1	0.7855147	0.816263364	-0.03074865	-0.05843907	0.00229962	0.007713854	8	0	0
THOC6	0.1160931	0.129676238	-0.01358318	-0.07543217	0.002308166	0.007739629	17	0	0
TPD52	0.5355792	0.585664754	-0.05008555	-0.18110863	0.002309284	0.007741929	2	0	0
TNFRSF11B	0.1672411	0.13801755	0.02922359	0.468337721	0.002310383	0.007744164	11	0	0
NPRL3	0.140463	0.140669411	-0.00020638	0.085507251	0.002310957	0.007744642	8	0	0
SH3BGRL3	0.2372388	0.2729844	-0.03574563	-0.20899214	0.002312005	0.007746706	5	0	0
CTSK	0.7905914	0.766452381	0.024138969	0.052460779	0.002325645	0.007788408	7	0	0
CYP2E1	0.3231399	0.368687939	-0.04554805	-0.20092051	0.002326188	0.007788408	8	0	0
PDE3A	0.1169116	0.07918319	0.037728421	0.530482577	0.002325448	0.007788408	12	0	0
SOAT2	0.685247	0.719760171	-0.03451322	-0.08532968	0.002326185	0.007788408	6	0	0
SRL	0.7245749	0.702332747	0.022242182	0.050448784	0.002328637	0.007795154	7	0	0
KLK6	0.6245867	0.672956059	-0.04836941	-0.1220361	0.002332355	0.007806143	6	0	0
GJB3	0.4616722	0.491535698	-0.02986355	-0.0486815	0.002334653	0.007812376	10	0	0
GPR6	0.2094015	0.195685265	0.013716186	0.403786193	0.002336499	0.007817096	8	0	0
TNFAIP8L3	0.6670146	0.611045761	0.055968804	0.147023752	0.002340456	0.007828874	6	0	0

XPO4	0.2661261	0.275272332	-0.00914624	-0.0574664	0.002347089	0.007848134	8	0	0
TMC8	0.378651	0.38186453	-0.00321351	0.005791908	0.002348048	0.007849877	7	0	0
RAP1GAP	0.1287664	0.103226689	0.02553967	0.520073928	0.002351366	0.007858039	7	0	0
ZG16	0.7129175	0.715789007	-0.0028715	-0.01671582	0.002351199	0.007858039	2	0	0
FAM210B	0.2476029	0.280955279	-0.03335233	-0.07658777	0.002353954	0.007865223	9	0	0
08-Sep	0.6438068	0.610429283	0.033377514	0.148285801	0.002358685	0.007879564	2	0	0
DNAH3	0.1562009	0.195957092	-0.03975623	-0.24573909	0.002363649	0.007894677	4	0	0
ACTL9	0.7058229	0.761342025	-0.0555191	-0.11376329	0.002365249	0.00789855	8	0	0
PCDHA7	0.4166837	0.416516566	0.000167171	0.126457266	0.002367973	0.007906172	7	0	0
BRICD5	0.5482852	0.578511227	-0.03022604	-0.06965191	0.002371801	0.007917479	8	0	0
UGT2B11	0.4146302	0.473839214	-0.05920902	-0.18832075	0.002372689	0.007918972	1	0	0
RGS7BP	0.2215351	0.201856947	0.019678143	0.381628552	0.002373868	0.00792143	14	0	0
MRO	0.8208143	0.843220718	-0.02240646	-0.03859298	0.002375035	0.007923851	6	0	0
PKHD1	0.5597464	0.621313982	-0.06156758	-0.1457994	0.002377666	0.007931153	5	0	0
RAD54L2	0.7620373	0.793544132	-0.03150687	-0.06682049	0.002385488	0.007955765	5	0	0
TTC1	0.2750464	0.29455376	-0.01950732	-0.05347333	0.00238622	0.007956729	13	0	0
NEO1	0.068891	0.095964395	-0.02707338	-0.19144065	0.002387688	0.007960145	5	0	0
IGLL5	0.7877699	0.849892188	-0.06212233	-0.10966627	0.002388225	0.007960453	4	0	0
HTR3A	0.4551244	0.452862988	0.002261374	0.059444768	0.002391594	0.007970202	8	0	0
COBL	0.1743144	0.140161375	0.034153067	0.57104463	0.002394997	0.00798006	11	0	0
AP1M2	0.286801	0.325158173	-0.03835717	-0.15704513	0.002397981	0.007988518	9	0	0
CWF19L1	0.1982104	0.18760714	0.01060331	0.059450717	0.002406962	0.008016951	11	0	0
SNTG2	0.1241991	0.0979664	0.026232728	0.346389216	0.002410261	0.008026445	7	0	0
ESRRB	0.4502157	0.416935755	0.033279898	0.145211152	0.002414177	0.008037995	4	0	0
INPP1	0.343132	0.351113264	-0.00798124	-0.01428243	0.002416784	0.008045182	14	0	0
TBC1D14	0.12733	0.155038858	-0.02770889	-0.15122736	0.00241871	0.008050099	8	0	0
VPREB1	0.3651101	0.415374438	-0.05026433	-0.21541378	0.002427204	0.008076869	3	0	0
SLC39A3	0.1023833	0.115702445	-0.01331918	-0.02429717	0.002433123	0.008095066	11	0	0
VAMP5	0.3925007	0.311480248	0.081020485	0.381598293	0.002438402	0.008111124	10	0	0
CYR61	0.1787524	0.138731115	0.040021294	0.379444647	0.002440112	0.008115305	16	0	0
CPD	0.282716	0.243181199	0.039534763	0.275490274	0.002440796	0.008116077	6	0	0
SNX2	0.2507395	0.265357804	-0.01461826	-0.11588569	0.00244348	0.00812199	12	0	0
C10orf105	0.6247995	0.671882686	-0.04708319	-0.10608602	0.002443061	0.00812199	5	0	0
KCNK12	0.2171776	0.164914071	0.052263512	0.638028435	0.002445871	0.008128431	9	0	0
WNT4	0.5368428	0.583688775	-0.046846	-0.07233093	0.002446427	0.008128771	5	0	0
TMEM176B	0.6127724	0.654381178	-0.04160874	-0.08432884	0.002447345	0.008130051	6	0	0
LTBP4	0.8192595	0.857030948	-0.03777145	-0.06812373	0.002447718	0.008130051	6	0	0
C1QTNF8	0.5745309	0.600978803	-0.02644794	-0.05783862	0.002449799	0.008135455	5	0	0
CDH11	0.5329551	0.565137762	-0.03218264	-0.04363172	0.002451575	0.00813875	3	0	0
DBNDD1	0.4758678	0.526221577	-0.05035374	-0.08404391	0.002452152	0.00813875	8	0	0
APOBEC3H	0.6708849	0.66220329	0.008681648	-0.02808859	0.002451788	0.00813875	6	0	0
MGAT5	0.1056494	0.133044868	-0.02739542	-0.11963045	0.002454206	0.008143956	5	0	0
ACSM5	0.6886062	0.716624773	-0.0280186	-0.03805973	0.002454629	0.008143956	4	0	0
DUSP27	0.7709256	0.803275375	-0.03234974	-0.05899417	0.002459552	0.008157755	4	0	0
ABCB11	0.6714356	0.714915279	-0.04347971	-0.09129717	0.002459698	0.008157755	3	0	0
TMEM247	0.8868627	0.91082118	-0.02395846	-0.03758931	0.00246181	0.00816325	4	0	0
CRTC1	0.2075178	0.242033819	-0.03451601	-0.10107697	0.002465198	0.008172973	10	0	0
PENK	0.2781363	0.23502028	0.043116062	0.412852766	0.002466508	0.008174915	14	0	0
CAAP1	0.1128235	0.122184557	-0.00936103	-0.04312877	0.002467108	0.008174915	5	0	0
TMEM45B	0.2213963	0.178398732	0.042997538	0.270944169	0.002467151	0.008174915	2	0	0
CLTB	0.3499165	0.337015662	0.012900804	0.050478065	0.002470118	0.008181476	7	0	0
GLTP	0.2771668	0.260582224	0.016584532	0.005839446	0.002469725	0.008181476	4	0	0
PPAN-P2RY	0.1913601	0.219095881	-0.02773583	-0.05124881	0.002470955	0.008181476	10	0	0
PPAN	0.1913601	0.219095881	-0.02773583	-0.05124881	0.002470955	0.008181476	10	0	0
RASL10B	0.3831218	0.399141325	-0.01601953	0.09223664	0.002472119	0.008183817	10	0	0
RAB6B	0.1391737	0.159562639	-0.02038891	0.237848969	0.002476876	0.008198054	11	0	0
SIRPB2	0.536046	0.581467327	-0.04542135	-0.11592832	0.002477346	0.008198097	8	0	0
EPHX3	0.331161	0.239764688	0.091396279	0.514583321	0.002479228	0.00820281	6	0	0
DPPA3	0.7174062	0.770246993	-0.05284076	-0.11492426	0.002486535	0.008225468	7	0	0
MAGEF1	0.1604818	0.186986143	-0.02650436	-0.07173414	0.002487961	0.008225849	8	0	0
PSTPIP1	0.8372612	0.89061623	-0.05335506	-0.08810572	0.00248734	0.008225849	1	0	0
USE1	0.3378712	0.360653095	-0.02278189	0.138865417	0.002488026	0.008225849	7	0	0
PTPRF	0.6603338	0.597894141	0.062439663	0.172986365	0.002493515	0.008242477	2	0	0

EXD2	0.246341	0.274432071	-0.02809111	-0.07782248	0.002494469	0.008244111	16	0	0
SUMF2	0.3767911	0.404992207	-0.02820108	-0.03523842	0.002497786	0.008250515	9	0	0
C9orf131	0.8109863	0.845916675	-0.03493037	-0.06554429	0.00249771	0.008250515	6	0	0
MIER2	0.4011483	0.396583288	0.004565052	-0.00121645	0.002497562	0.008250515	9	0	0
FKBP9	0.2914929	0.321791247	-0.03029834	0.125115211	0.00249925	0.008253829	12	0	0
ZC3H4	0.1635366	0.179094828	-0.01555822	0.025892614	0.00250199	0.008261358	10	0	0
TREML4	0.7177478	0.753471009	-0.03572317	-0.05823209	0.002503218	0.00826389	4	0	0
CNGA1	0.0977187	0.102257703	-0.00453895	-0.10004792	0.002506341	0.008272677	6	0	0
HGFAC	0.6842539	0.706933947	-0.02268002	-0.04835942	0.002507009	0.00827336	11	0	0
EAPP	0.3019514	0.340539747	-0.03858834	-0.10157875	0.002509738	0.008280841	8	0	0
CYP1B1	0.6360291	0.70660419	-0.07057508	-0.16260372	0.00251064	0.008282295	4	0	0
MBD2	0.3175511	0.339662757	-0.02211165	-0.13280311	0.002511106	0.008282308	6	0	0
SLC39A13	0.3426822	0.391916398	-0.04923417	-0.20717852	0.002512614	0.00828576	2	0	0
LY6G5C	0.7686455	0.75237102	0.016274524	0.036224441	0.00251398	0.008287467	6	0	0
RAI1	0.3115563	0.3238972	-0.01234089	-0.13000357	0.002514056	0.008287467	6	0	0
IVD	0.278918	0.30828907	-0.02937112	-0.01332592	0.002516282	0.008293281	9	0	0
PNLIPRP1	0.5877043	0.649715154	-0.06201084	-0.14339962	0.002517775	0.008296677	5	0	0
RAB42	0.480451	0.496494454	-0.0160435	-0.10547041	0.002519064	0.008299399	7	0	0
LIPG	0.173802	0.107505478	0.066296567	0.88437313	0.002528016	0.008327363	3	0	0
AQR	0.3050589	0.338683882	-0.03362502	-0.07015029	0.002530343	0.008333499	12	0	0
GDAP1	0.0538045	0.056392401	-0.00258789	0.051514703	0.00253775	0.008356359	7	0	0
RNASEH2C	0.4720244	0.518015764	-0.0459914	-0.10599998	0.002538758	0.008358144	4	0	0
WISP2	0.3855303	0.354192236	0.031338018	0.123615141	0.00254151	0.008365668	10	0	0
RYR3	0.1693604	0.161702295	0.007658073	0.329388854	0.002544427	0.008373734	12	0	0
IRF5	0.352522	0.372685889	-0.0201639	-0.05940433	0.002545193	0.008374716	7	0	0
NOV	0.2489368	0.274479037	-0.02554229	0.036294541	0.002549576	0.0083876	7	0	0
LTA	0.7162129	0.786217768	-0.07000487	-0.13699213	0.00255209	0.008394331	8	0	0
MTNR1A	0.2646227	0.222174123	0.042448529	0.453161372	0.002554784	0.008401652	9	0	0
IL6	0.7268961	0.774792246	-0.0478961	-0.09361745	0.002555423	0.008402212	6	0	0
GPX8	0.199746	0.184390645	0.015355395	0.220096035	0.002558221	0.008409872	9	0	0
PLSCR4	0.2341169	0.210069832	0.024047107	0.319922814	0.002558877	0.008410484	12	0	0
FGF7	0.6369314	0.696226816	-0.05929539	-0.12347736	0.002560766	0.008415153	4	0	0
TNPO2	0.1686275	0.186641451	-0.01801392	-0.17637344	0.002565177	0.008426559	10	0	0
ISM1	0.0860317	0.041743676	0.044288011	0.820358411	0.002564835	0.008426559	23	0	0
AKR1C2	0.46894	0.54391608	-0.07497606	-0.21148749	0.002572163	0.008447962	3	0	0
VAV3	0.0986594	0.045870057	0.052789328	1.065639057	0.002574576	0.008453314	9	0	0
TRAF5	0.2327556	0.246490833	-0.01373524	0.157403336	0.002574735	0.008453314	10	0	0
USP43	0.045376	0.051803045	-0.00642701	-0.13530905	0.00257821	0.008463171	3	0	0
BTBD17	0.346511	0.315966428	0.030544611	0.15325679	0.002583441	0.008478793	8	0	0
MOCS1	0.2862735	0.267949875	0.018323663	0.388341658	0.002588	0.008492202	11	0	0
NCL	0.5511672	0.506585996	0.044581194	0.112623657	0.002591278	0.008501402	7	0	0
HRC	0.4997921	0.528554531	-0.02876239	-0.07967449	0.002592605	0.008504199	6	0	0
TBC1D1	0.3536617	0.310206529	0.04345516	0.181290385	0.002595264	0.008511366	6	0	0
AMBRA1	0.1925915	0.20709693	-0.01450546	0.054394365	0.002596951	0.008513784	13	0	0
MED24	0.4002566	0.435922623	-0.03566599	-0.04954206	0.002596626	0.008513784	10	0	0
PSD2	0.2675399	0.303271033	-0.03573111	-0.0196071	0.00259857	0.008517534	5	0	0
SHISA2	0.1742812	0.138620286	0.035660945	0.414097839	0.00260344	0.008530379	8	0	0
NRIP3	0.3374708	0.341511202	-0.00404041	-0.00120771	0.002605457	0.008535431	8	0	0
GBE1	0.3332756	0.289736867	0.043538742	0.177156155	0.002608515	0.008542326	10	0	0
C12orf43	0.2947569	0.333233622	-0.03847668	-0.16925997	0.002608279	0.008542326	6	0	0
OSBP2	0.5834362	0.608533386	-0.0250972	-0.03304645	0.00260957	0.008544222	7	0	0
LRRFIP1	0.2542988	0.255266818	-0.00096799	0.316389723	0.002613545	0.008555675	10	0	0
ZFYVE28	0.1024246	0.112904032	-0.01047946	-0.12387703	0.002615241	0.008559664	5	0	0
FBXW5	0.4535046	0.481984905	-0.02848034	-0.07836509	0.00261591	0.008559949	5	0	0
HDC	0.7736477	0.773640632	7.05E-06	0.005557642	0.002616282	0.008559949	7	0	0
IGFBP3	0.1524822	0.108004716	0.044477511	0.525379805	0.002618157	0.008564519	22	0	0
DEK	0.2136021	0.224488812	-0.01088671	-0.02513118	0.00262407	0.008582298	9	0	0
MTMR14	0.257868	0.261736818	-0.00386885	0.051853471	0.002624851	0.008583287	12	0	0
STOML3	0.7930446	0.820470618	-0.02742597	-0.05249846	0.002625964	0.00858536	4	0	0
PLA2G2D	0.7357693	0.791177364	-0.0554081	-0.10348824	0.002628943	0.008593535	4	0	0
MYBPC3	0.7499919	0.786849085	-0.03685716	-0.06856331	0.002634548	0.008610287	6	0	0
TLCD2	0.1556204	0.162068505	-0.00644806	0.030205286	0.002641413	0.008631151	11	0	0
ATF7IP	0.3311476	0.348415632	-0.017268	-0.06376608	0.002643251	0.008635583	10	0	0

MRPL48	0.4654163	0.517077844	-0.0516615	-0.15623785	0.002651989	0.008662551	6	0	0
TULP4	0.8498865	0.871841159	-0.02195464	-0.03749186	0.002654311	0.008666981	6	0	0
TMEM248	0.3547819	0.366342164	-0.01156028	0.006448383	0.002654283	0.008666981	5	0	0
VPS26A	0.2464337	0.249049702	-0.00261604	0.068888928	0.002657066	0.008674396	4	0	0
FZD5	0.1168557	0.072307377	0.044548312	0.425055517	0.002659024	0.008679211	7	0	0
NANOS2	0.6245373	0.63663012	-0.01209278	-0.03078685	0.002662222	0.008688066	8	0	0
TGFBI	0.3205656	0.29330682	0.02725877	0.295506326	0.00266596	0.008698683	16	0	0
C1orf185	0.732262	0.757779069	-0.02551711	-0.0398118	0.002674935	0.008724793	3	0	0
CRISP2	0.4883899	0.434928609	0.053461253	0.214004204	0.002674832	0.008724793	12	0	0
C11orf53	0.6913985	0.743850955	-0.05245247	-0.1025026	0.002684802	0.008753791	6	0	0
AFF4	0.0861919	0.094462478	-0.00827055	-0.016545	0.002686397	0.0087574	7	0	0
CAST	0.6881002	0.7396173	-0.05151708	-0.10237379	0.00268846	0.008762532	3	0	0
ABLIM3	0.3031903	0.285237067	0.017953206	0.487490154	0.002689773	0.00876522	14	0	0
KCNA4	0.2298442	0.173375754	0.056468442	0.473102175	0.0026912	0.008768278	10	0	0
ARHGEF28	0.2269866	0.24221809	-0.01523152	0.016122584	0.002692544	0.008771062	9	0	0
LRRC14	0.3006041	0.333943942	-0.03333987	-0.08450653	0.002700003	0.008793764	7	0	0
OGN	0.7482704	0.720323647	0.027946785	0.05823388	0.002704903	0.008808124	2	0	0
DAW1	0.5431855	0.502699069	0.040486412	0.550330647	0.002707629	0.008815401	8	0	0
FAM177B	0.7510832	0.773736515	-0.02265329	-0.05170167	0.002709173	0.008817225	6	0	0
CPA4	0.7972124	0.836362044	-0.03914968	-0.08542591	0.002708967	0.008817225	6	0	0
OLFM3	0.1931079	0.191761896	0.001346	0.228125043	0.002723423	0.008860388	10	0	0
DMRTA1	0.4200105	0.4701994	-0.05018892	-0.18043065	0.002723241	0.008860388	3	0	0
CLEC16A	0.3136845	0.332475469	-0.01879093	-0.06082498	0.00272867	0.008875848	9	0	0
MAP9	0.1966762	0.225044494	-0.02836828	-0.06493372	0.002737152	0.008901824	14	0	0
PRC1	0.5645062	0.59651065	-0.0320044	-0.10524438	0.002737997	0.008902958	6	0	0
TRIM63	0.551646	0.592247093	-0.04060108	-0.09317137	0.002740229	0.0089086	6	0	0
ARAP3	0.2921148	0.263244974	0.02886987	0.182468881	0.002742575	0.008914612	9	0	0
KRT8	0.5032518	0.556451757	-0.05319992	-0.14991927	0.002755984	0.008956572	5	0	0
SAT2	0.1162821	0.123826214	-0.00754409	-0.06603354	0.00276292	0.008977487	12	0	0
GLYCTK	0.6107401	0.65064238	-0.03990231	-0.11042494	0.002776171	0.00901891	8	0	0
GDAP1L1	0.2736786	0.295721461	-0.02204289	0.130332475	0.002778817	0.009025872	15	0	0
CYP4A22	0.7424787	0.796304299	-0.05382556	-0.09967359	0.002780669	0.00902756	1	0	0
LRRC49	0.5401546	0.511267268	0.028887331	0.099685431	0.002780847	0.00902756	7	0	0
PRPF8	0.0497371	0.051141534	-0.00140442	0.001927613	0.002780805	0.00902756	10	0	0
STK39	0.196155	0.222932362	-0.02677731	-0.17344258	0.002788436	0.00905056	11	0	0
RELL1	0.1774878	0.157269342	0.020218443	0.135472834	0.002790126	0.009054407	11	0	0
CSDE1	0.2515343	0.282391542	-0.03085726	-0.00626778	0.002792783	0.009061388	7	0	0
EMILIN3	0.2098063	0.166269032	0.043537249	0.567829228	0.002793894	0.009063353	8	0	0
PYGM	0.622399	0.611295307	0.011103672	0.034768763	0.002794754	0.009064503	13	0	0
TMEM160	0.0390876	0.045504047	-0.0064164	-0.11502442	0.00279645	0.009068364	7	0	0
RGSL1	0.7571859	0.806965783	-0.04977992	-0.09170767	0.002797386	0.009069762	4	0	0
HFM1	0.6279119	0.640157166	-0.01224528	0.117707269	0.002798665	0.009072266	7	0	0
SNX7	0.2240393	0.183940004	0.040099275	0.306871691	0.002804233	0.009088673	5	0	0
EPPK1	0.2655493	0.280837567	-0.01528827	-0.01531103	0.002807719	0.009098327	7	0	0
FRMD4A	0.8112068	0.826069217	-0.01486244	-0.02330148	0.002808761	0.009099584	2	0	0
UNC45A	0.1092155	0.136073459	-0.02685796	-0.24538731	0.002809121	0.009099584	10	0	0
RBBP6	0.7882567	0.839926136	-0.0516694	-0.08867682	0.002810378	0.009102013	2	0	0
NGF	0.2994454	0.246340612	0.053104754	0.406739224	0.00281375	0.009111288	8	0	0
PRPS1L1	0.815306	0.852175884	-0.03686987	-0.06693034	0.002815731	0.009116057	11	0	0
TMA16	0.1026969	0.128893299	-0.02619637	-0.12614425	0.002817178	0.009119096	11	0	0
CENPV	0.2161157	0.231517236	-0.01540152	-0.01534484	0.002818471	0.009121633	12	0	0
ANKRD31	0.0710659	0.080207285	-0.00914135	-0.12070619	0.002819563	0.009123523	9	0	0
LMO1	0.2419667	0.198871799	0.043094936	0.28003581	0.002826275	0.009143591	10	0	0
SLFNL1	0.8181581	0.853375025	-0.03521688	-0.05991741	0.002832506	0.009162098	6	0	0
PITX1	0.1716735	0.189571954	-0.01789842	-0.20143987	0.002835374	0.009169719	7	0	0
CSF1R	0.6859423	0.724128682	-0.03818637	-0.07918183	0.002837018	0.009173383	6	0	0
MAP2	0.1049047	0.129938562	-0.02503388	0.008587028	0.002840246	0.009182166	10	0	0
GRB10	0.3855021	0.368669005	0.016833125	0.231548844	0.00284472	0.009193314	23	0	0
KRT20	0.8003727	0.845474684	-0.04510202	-0.08221635	0.002844339	0.009193314	7	0	0
FUT3	0.5358906	0.566711433	-0.03082087	-0.06942852	0.002846858	0.009198568	9	0	0
PGBD2	0.2527622	0.268903095	-0.01614089	-0.08998728	0.002853184	0.009216466	14	0	0
FBXL17	0.0876767	0.112289342	-0.02461264	-0.14776561	0.002853602	0.009216466	5	0	0
AZU1	0.5180197	0.572907533	-0.05488786	-0.15350152	0.002853939	0.009216466	5	0	0

SLC22A2	0.7418671	0.786357276	-0.04449021	-0.08461187	0.002857699	0.009225287	2	0	0
MAPK1	0.0555767	0.066157665	-0.01058095	-0.02099606	0.002857626	0.009225287	10	0	0
RPS20	0.3048811	0.32428145	-0.01940033	-0.06801132	0.002861744	0.009236683	6	0	0
LCOR	0.2260619	0.24288679	-0.01682487	-0.08799434	0.002863588	0.009240972	6	0	0
TLR3	0.2108941	0.205481079	0.005413066	0.142697484	0.002873313	0.009269017	7	0	0
TPH2	0.2689536	0.31957801	-0.05062441	-0.16892429	0.002873001	0.009269017	6	0	0
KDM3A	0.0494932	0.055209706	-0.0057165	-0.12794031	0.002875997	0.009276009	7	0	0
ZKSCAN7	0.4190169	0.377234953	0.041781904	0.660416148	0.002877886	0.009280431	10	0	0
C10orf88	0.2016421	0.206071072	-0.00442899	-0.07684034	0.00287931	0.009283354	11	0	0
PNMA1	0.5779239	0.611809355	-0.03388549	-0.11148289	0.002880283	0.009284824	6	0	0
INHBE	0.5261946	0.588733122	-0.06253851	-0.15915637	0.002882829	0.009291362	1	0	0
SIN3B	0.333399	0.36885586	-0.03545684	-0.04922953	0.002884484	0.009295023	9	0	0
CNTNAP1	0.2083267	0.209329686	-0.00100295	0.329279021	0.002885254	0.009295834	13	0	0
PCP4L1	0.3070917	0.285617102	0.021474606	0.513795169	0.002890316	0.009309132	8	0	0
TSPAN31	0.4981464	0.444582029	0.053564338	0.164833323	0.002891062	0.00930953	9	0	0
AMER3	0.2946517	0.255419929	0.039231804	0.287697684	0.002891725	0.009309994	12	0	0
GAL	0.6027914	0.599014668	0.003776717	0.054802827	0.002901865	0.009338588	8	0	0
SAV1	0.1935251	0.200122105	-0.006597	0.118923268	0.002902168	0.009338588	5	0	0
CPOX	0.2218098	0.252888801	-0.03107896	-0.06431805	0.002906686	0.009351449	11	0	0
COPS7A	0.2724093	0.291430784	-0.01902144	-0.10569108	0.002908599	0.009355924	13	0	0
NEURL1	0.2411772	0.213399389	0.027777769	0.369512353	0.002911942	0.009364997	11	0	0
SF3B5	0.2011317	0.23357102	-0.03243936	-0.14501929	0.002912625	0.009365516	8	0	0
POLR2H	0.2914132	0.331241674	-0.03982844	-0.05480967	0.002914817	0.009370882	7	0	0
ZNF536	0.1061631	0.066471854	0.039691205	0.610507993	0.002917035	0.009376334	4	0	0
SPATA31E1	0.771757	0.810192209	-0.03843526	-0.0646431	0.00291877	0.009380229	3	0	0
C12orf54	0.7898692	0.833566743	-0.04369757	-0.08203765	0.002919746	0.009381685	5	0	0
DDX28	0.3158031	0.340983728	-0.0251806	-0.1434736	0.00292282	0.00938988	5	0	0
MIEF2	0.205985	0.229070451	-0.02308545	-0.0992535	0.002924969	0.0093951	12	0	0
RNF212	0.5474536	0.506003162	0.041450403	0.177982925	0.002927125	0.009400343	5	0	0
TANC2	0.8426511	0.86551547	-0.02286437	-0.03976669	0.002934031	0.009420834	5	0	0
INSL6	0.695254	0.73982899	-0.04457497	-0.11203686	0.002936572	0.009427306	10	0	0
RPL7	0.2480799	0.275594404	-0.02751448	-0.09034514	0.002938646	0.009432276	6	0	0
PDLIM4	0.3340836	0.363336977	-0.02925335	0.020454198	0.002939612	0.009433473	11	0	0
NFYB	0.2854438	0.297653098	-0.01220925	-0.0798633	0.002940071	0.009433473	7	0	0
SAC3D1	0.1996202	0.242285202	-0.04266499	-0.09359841	0.002943129	0.009441594	9	0	0
PPFIBP2	0.1620464	0.193039144	-0.03099274	-0.15610852	0.002948094	0.009454142	6	0	0
ZSCAN22	0.1964956	0.213980472	-0.01748488	-0.0136792	0.002949844	0.009458064	12	0	0
ANGPTL7	0.9064341	0.90037088	0.006063243	0.009337572	0.002950601	0.0094588	5	0	0
ENTPD5	0.1714324	0.141229324	0.030203118	0.185783588	0.002951533	0.009460097	6	0	0
CNDP1	0.803026	0.852507368	-0.04948133	-0.08650295	0.002956201	0.009473363	12	0	0
AP1G1	0.1189143	0.12757656	-0.00866227	0.015300751	0.002957429	0.009475607	15	0	0
DDX4	0.8819709	0.911624222	-0.02965331	-0.05092189	0.002963225	0.00949248	13	0	0
ALX3	0.3869303	0.360259438	0.026670836	0.595309349	0.00296481	0.009495864	7	0	0
EPHA8	0.2584602	0.249924427	0.008535803	0.387870673	0.002967158	0.009501686	4	0	0
CLDN24	0.7797893	0.800247582	-0.02045832	-0.03077724	0.002970579	0.009510943	8	0	0
LURAP1	0.2445589	0.180388792	0.064170129	0.872326474	0.00297197	0.009513698	10	0	0
MRPL22	0.4673027	0.466302617	0.00100008	0.051815623	0.00297359	0.009517185	10	0	0
PLA2G4C	0.4517602	0.5041554	-0.05239521	-0.11325027	0.002974522	0.009518471	8	0	0
QDPR	0.1959467	0.221788836	-0.02584217	0.002001495	0.002976799	0.009524059	9	0	0
RUNX3	0.6203725	0.681786546	-0.06141401	-0.15139075	0.002978514	0.009527845	12	0	0
CAV2	0.7690907	0.814510296	-0.04541959	-0.08917947	0.002979405	0.009528995	2	0	0
FAM19A4	0.236335	0.199726978	0.036608025	0.558621941	0.002984224	0.009542237	13	0	0
MAP3K1	0.0551198	0.066046367	-0.01092657	-0.15378726	0.002984679	0.009542237	7	0	0
SLCO4A1	0.2174845	0.249639531	-0.03215505	-0.0790753	0.002985141	0.009542237	9	0	0
FSBP	0.5443482	0.59548518	-0.05113697	-0.11100417	0.002988396	0.009549239	9	0	0
HYDIN	0.3271713	0.323169708	0.004001641	0.211085239	0.00298808	0.009549239	12	0	0
DMRTC2	0.7788689	0.845911015	-0.06704214	-0.12191594	0.002990209	0.009553328	15	0	0
ARIH2	0.0503252	0.049253916	0.001071307	-0.02451286	0.002992339	0.009558432	12	0	0
HOXC8	0.1974687	0.152040499	0.045428195	0.436343831	0.002995279	0.009566118	15	0	0
OAS1	0.0774129	0.107620526	-0.03020761	-0.23605859	0.002999764	0.009578737	3	0	0
CTRC	0.7814773	0.824738313	-0.04326103	-0.08075173	0.003003638	0.0095894	7	0	0
IFI44L	0.3485545	0.294358864	0.054195617	0.27000068	0.003008184	0.009602205	4	0	0
RILP	0.5722832	0.54411086	0.028172324	0.097918101	0.003008726	0.009602226	11	0	0

MMP24	0.2315889	0.221297736	0.010291191	0.210872046	0.003012277	0.009611848	4	0	0
ZFHX4	0.267272	0.231746443	0.03552556	0.511179161	0.003029619	0.009665465	9	0	0
UMODL1	0.3450714	0.394453448	-0.04938206	-0.18803031	0.003031745	0.009670527	7	0	0
DNAJA2	0.2573723	0.281419946	-0.02404768	-0.08525811	0.003034231	0.009676738	9	0	0
GIMAP7	0.4148007	0.439299735	-0.024499	-0.14640187	0.003038236	0.009687787	6	0	0
WNT11	0.353768	0.30652493	0.047243022	0.358658915	0.003041545	0.009696613	6	0	0
INHBA	0.7006657	0.747482855	-0.04681712	-0.08219725	0.003042515	0.009697983	7	0	0
SDHA	0.2146021	0.167840203	0.046761941	0.191167481	0.0030489	0.009716608	2	0	0
RSPO2	0.2576751	0.224224163	0.033450975	0.423781919	0.003052345	0.009725859	11	0	0
GGT6	0.6250127	0.644906631	-0.01989389	-0.04634605	0.00305805	0.009742307	5	0	0
MRPL15	0.1721052	0.213381078	-0.04127583	-0.10907061	0.00305889	0.009743202	9	0	0
SLC25A32	0.0665909	0.085788261	-0.01919738	-0.19050267	0.003059417	0.009743202	9	0	0
LRRN1	0.1159946	0.085798604	0.030195996	0.884988183	0.003060021	0.009743392	9	0	0
C11orf40	0.76193	0.81956197	-0.05763198	-0.10768999	0.003062521	0.009747894	4	0	0
TBX1	0.2096342	0.158294577	0.051339664	0.476615568	0.003062161	0.009747894	12	0	0
GHR	0.1626954	0.120565545	0.042129822	0.497928978	0.003065998	0.009754265	6	0	0
ARRB1	0.1444165	0.102347076	0.042069407	0.419530946	0.003066698	0.009754265	5	0	0
CCER1	0.8257648	0.859003583	-0.03323883	-0.05828828	0.003066312	0.009754265	11	0	0
ANGPT4	0.3794742	0.383240111	-0.0037659	0.116281537	0.003066122	0.009754265	7	0	0
RABGGTA	0.2300491	0.216592523	0.013456537	-0.02968519	0.003068345	0.009757772	8	0	0
ANO5	0.241255	0.243546604	-0.00229157	0.287397434	0.003069881	0.009760925	7	0	0
MASP1	0.495025	0.447737532	0.047287435	0.153925626	0.003077246	0.009780878	3	0	0
STEAP1B	0.6048136	0.675267732	-0.07045415	-0.15923107	0.003077025	0.009780878	2	0	0
FCRL4	0.7475586	0.81490121	-0.06734264	-0.12376984	0.00307922	0.009785417	2	0	0
CHST13	0.1575718	0.146287235	0.011284614	0.402655207	0.003082194	0.009793133	12	0	0
PTAFR	0.6247813	0.630974832	-0.00619355	-0.02069978	0.003085913	0.009802556	7	0	0
PDE9A	0.230628	0.186084814	0.044543232	0.390986123	0.003086253	0.009802556	6	0	0
PPM1B	0.2552582	0.309615198	-0.05435698	-0.18294424	0.003088522	0.009807519	3	0	0
ZNF311	0.3883935	0.366552785	0.021840741	0.466496797	0.003088909	0.009807519	18	0	0
ITK	0.0505302	0.044985594	0.005544602	0.133492994	0.003091347	0.00981005	10	0	0
REEP6	0.0562401	0.066130576	-0.00989051	-0.03080526	0.003091125	0.00981005	11	0	0
NOVA2	0.1191343	0.121422411	-0.00228814	0.220773306	0.003090299	0.00981005	11	0	0
SLC12A4	0.4579635	0.43039889	0.02756464	0.220106004	0.003093892	0.009816388	6	0	0
FCAMR	0.7188705	0.766098563	-0.04722802	-0.10212153	0.003096252	0.009822139	5	0	0
LIPI	0.7555266	0.819625852	-0.06409928	-0.11600839	0.003106183	0.009851899	1	0	0
DNTTIP2	0.4091946	0.445080624	-0.03588607	-0.07854318	0.003112516	0.009870242	6	0	0
PRAMEF2	0.6703272	0.720485017	-0.05015777	-0.10665483	0.00311374	0.009872378	2	0	0
LGR6	0.1585581	0.14484772	0.013710372	0.112466897	0.003116381	0.009879005	4	0	0
AKAP8	0.1584576	0.180061295	-0.02160372	-0.0125209	0.003119249	0.009886348	10	0	0
FKBP7	0.159372	0.175354225	-0.01598224	-0.06736855	0.003120316	0.009887849	9	0	0
IGFALS	0.6912962	0.642760231	0.04853596	0.097723024	0.003120825	0.009887849	5	0	0
RABGEF1	0.1756318	0.215880666	-0.04024883	-0.10784251	0.00312189	0.009889476	4	0	0
AP5B1	0.2173659	0.227231923	-0.00986602	-0.09307322	0.003124661	0.009896508	9	0	0
LCE1E	0.6306722	0.683550183	-0.05287797	-0.12509481	0.003125622	0.009897804	4	0	0
POLD1	0.1543489	0.163657768	-0.00930885	0.024242394	0.003127756	0.009901207	9	0	0
SULF2	0.1464713	0.163643861	-0.01717261	0.057386198	0.003127801	0.009901207	7	0	0
POLD3	0.0405878	0.046043895	-0.00545607	-0.10844199	0.003128812	0.00990266	13	0	0
RASGRP4	0.3696646	0.369913221	-0.0002486	0.112167168	0.003130788	0.009907166	10	0	0
PRMT5	0.0453621	0.044991482	0.000370635	0.038272625	0.003132155	0.009909742	10	0	0
NT5C	0.2343356	0.262649058	-0.02831342	0.158574099	0.003136041	0.009920288	8	0	0
BTG2	0.252737	0.284238621	-0.03150162	-0.09343998	0.003138934	0.009927688	10	0	0
C15orf38-AI	0.3018064	0.307154246	-0.00534788	0.277430725	0.003139923	0.009929064	14	0	0
KCNF1	0.2366372	0.23578641	0.000850759	0.272299224	0.003142119	0.009930757	11	0	0
DAGLA	0.1374	0.151771851	-0.01437184	-0.00898466	0.003141945	0.009930757	8	0	0
SLC2A6	0.1386401	0.155868652	-0.01722855	-0.07297939	0.003143387	0.009933014	6	0	0
LAMTOR4	0.1539777	0.162646139	-0.00866847	-0.00219253	0.003144503	0.009934791	8	0	0
SYN3	0.3140633	0.299021667	0.015041628	0.348517879	0.003149906	0.009950109	12	0	0
TTR	0.6310127	0.694497124	-0.06348445	-0.13594079	0.003151036	0.009951926	8	0	0
SLC16A8	0.6836801	0.709761127	-0.02608101	-0.05585136	0.003154132	0.00995995	7	0	0
ELOF1	0.0604153	0.052944006	0.007471282	0.093640828	0.003163083	0.009986455	8	0	0
PGLYRP3	0.7439997	0.79163288	-0.0476332	-0.09209961	0.003166461	0.009995363	6	0	0
DAGLB	0.3317186	0.402852693	-0.07113407	-0.1450036	0.003170773	0.010005451	9	0	0
KDELR2	0.3317186	0.402852693	-0.07113407	-0.1450036	0.003170773	0.010005451	9	0	0

GPR12	0.2035681	0.179763757	0.023804365	0.491413972	0.003172746	0.010009917	12	0	0
MSLN	0.5651802	0.546430242	0.018749923	0.052988332	0.003174451	0.010013535	14	0	0
PNPLA6	0.2207622	0.203898576	0.016863576	0.133830746	0.003177819	0.010022395	7	0	0
TOM1L1	0.7368753	0.768202008	-0.03132673	-0.05720771	0.003184229	0.010040845	5	0	0
ERGIC1	0.2901996	0.314978341	-0.02477875	-0.06335391	0.00318543	0.010041417	11	0	0
ARMC4	0.5582601	0.563409734	-0.00514964	0.145533454	0.00318553	0.010041417	9	0	0
NUDT13	0.3902368	0.379356084	0.010880675	0.345598884	0.003186834	0.010043764	5	0	0
SPATA3	0.6845767	0.728439835	-0.0438631	-0.09025218	0.003191597	0.010057007	4	0	0
ZNF517	0.2895368	0.338499768	-0.04896301	-0.07213364	0.003195052	0.010066124	4	0	0
BTN3A2	0.2120878	0.243320592	-0.03123284	-0.14094914	0.003196646	0.01006761	8	0	0
IL27RA	0.2934636	0.29565024	-0.00218664	-0.02841057	0.003196401	0.01006761	13	0	0
NALCN	0.1518039	0.108257316	0.043546623	0.495574155	0.003203708	0.010087685	10	0	0
LCMT1	0.1077369	0.133235418	-0.02549857	-0.1079119	0.003204145	0.010087685	10	0	0
IFI44	0.6884906	0.726269255	-0.03777864	-0.09759285	0.003206144	0.010092207	3	0	0
PTGS1	0.2630155	0.252587805	0.01042765	0.598126055	0.003209703	0.010101639	10	0	0
ACAP3	0.5315921	0.570430129	-0.03883805	-0.0949438	0.003211961	0.010103427	8	0	0
C19orf71	0.6788233	0.706000728	-0.02717742	-0.04456376	0.003211487	0.010103427	5	0	0
RPTOR	0.3898859	0.422989845	-0.03310392	-0.05779739	0.003214494	0.010109622	10	0	0
DNA2	0.2234617	0.227411525	-0.00394986	0.043349898	0.003222896	0.010132491	11	0	0
EIF4B	0.2035573	0.237600844	-0.03404356	-0.11959187	0.003225142	0.010137775	9	0	0
NPHP1	0.2037131	0.244321916	-0.04060886	-0.10141219	0.003238839	0.010179046	12	0	0
NIM1K	0.2315164	0.183845807	0.047670631	0.49992128	0.003240101	0.010181229	7	0	0
RUFY1	0.1111921	0.105743343	0.005448766	0.08253791	0.003243452	0.010189421	10	0	0
YBX2	0.212165	0.203836846	0.008328131	0.108312998	0.003243844	0.010189421	14	0	0
SLC45A4	0.1277173	0.135656165	-0.00793883	-0.14110392	0.00324493	0.010191046	7	0	0
DIS3L	0.2674815	0.279185359	-0.01170382	-0.09373804	0.00324849	0.010200441	14	0	0
WDFY3	0.0819978	0.06249725	0.019500538	0.314748875	0.003249991	0.010203367	9	0	0
GNG7	0.2563069	0.263253338	-0.00694648	0.058518142	0.003251207	0.010205398	7	0	0
MTRF1	0.1333921	0.127633364	0.005758761	0.126574022	0.00325289	0.010207871	12	0	0
COTL1	0.2409039	0.275409186	-0.03450527	-0.16324418	0.003253133	0.010207871	8	0	0
C12orf75	0.3604128	0.326468832	0.033943955	0.103342655	0.003253747	0.010208012	3	0	0
SVEP1	0.1814726	0.151496368	0.02997627	0.465265291	0.003261794	0.010231469	10	0	0
IL9	0.6384522	0.605422321	0.033029885	0.108455982	0.003265123	0.01024012	4	0	0
TNFRSF1B	0.3317451	0.348109918	-0.01636483	0.125722504	0.003268273	0.010245767	10	0	0
CNR1	0.1133134	0.077760555	0.03555281	0.527282763	0.003268015	0.010245767	12	0	0
MUSK	0.7521423	0.742630333	0.009512015	0.023166512	0.003268637	0.010245767	3	0	0
DEFB108B	0.8233864	0.879771785	-0.05638539	-0.09444981	0.003269922	0.010248002	1	0	0
TMPRSS11B	0.7814991	0.822347687	-0.04084863	-0.07505046	0.003275034	0.010260438	6	0	0
SCGN	0.3280401	0.333951864	-0.00591176	0.109278634	0.003274866	0.010260438	12	0	0
ATOH7	0.6043448	0.584047914	0.020296856	0.049823583	0.003278027	0.01026802	9	0	0
ZDHHC18	0.0904905	0.079102912	0.011387622	0.159270806	0.00328068	0.010274539	5	0	0
LPL	0.655874	0.748299229	-0.09242518	-0.18751795	0.003281969	0.01027678	1	0	0
WDR38	0.4456619	0.44392991	0.001732019	0.010887744	0.003290416	0.01029604	7	0	0
OTUB1	0.2572719	0.287006295	-0.0297344	-0.11410698	0.003289795	0.01029604	11	0	0
GPR68	0.4642346	0.476626766	-0.01239216	-0.05909437	0.003289279	0.01029604	11	0	0
TTC23	0.1874019	0.226414447	-0.0390125	-0.07745762	0.003290364	0.01029604	9	0	0
ASS1	0.283902	0.294110433	-0.01020839	0.099473661	0.003298501	0.01031954	9	0	0
CRIPAK	0.9029123	0.919468355	-0.0165561	-0.02592955	0.00330018	0.010322972	1	0	0
IL33	0.6510786	0.598718924	0.052359687	0.162785054	0.00330075	0.010322972	5	0	0
ECEL1	0.4679233	0.428180371	0.039742975	0.379533746	0.003304234	0.010331443	6	0	0
CSRP3	0.8355241	0.874901225	-0.03937708	-0.06752018	0.003305186	0.010331443	4	0	0
JAM3	0.2775481	0.274469125	0.003078978	0.537847432	0.003304816	0.010331443	12	0	0
GIT2	0.2086334	0.226471694	-0.0178383	0.005501361	0.003310425	0.010346015	12	0	0
PPFIA2	0.2536137	0.252224273	0.001389451	0.242058624	0.003322015	0.010380428	12	0	0
ANAPC5	0.0464302	0.060482954	-0.01405272	-0.06988991	0.003329697	0.010402619	10	0	0
SNTA1	0.1523781	0.152814499	-0.00043641	0.063859223	0.003331319	0.010405876	11	0	0
UBA5	0.8649847	0.887292346	-0.02230766	-0.03781989	0.003335746	0.010417889	5	0	0
CCBE1	0.1311927	0.088947116	0.042245559	0.448596127	0.003339066	0.010426442	6	0	0
PRTG	0.1902792	0.156234453	0.034044779	0.40788081	0.003342206	0.010434431	11	0	0
SIRT7	0.7141631	0.771258292	-0.05709522	-0.09082787	0.003344391	0.010439434	5	0	0
NRP1	0.1472886	0.103715786	0.043572827	0.553037256	0.003346078	0.010442883	7	0	0
SLC8A2	0.2692769	0.295684736	-0.02640781	0.028235579	0.003349887	0.010452952	8	0	0
QSOX1	0.2996085	0.266874429	0.032734094	0.201533822	0.003353509	0.010462436	5	0	0

HMGA2	0.1170508	0.094017358	0.023033434	0.403006408	0.003354876	0.01046488	8	0	0
SERINC4	0.4669227	0.485601271	-0.01867859	0.026707395	0.003355509	0.010465033	13	0	0
SLC39A2	0.5280828	0.5855338	-0.05745098	-0.14179049	0.003360985	0.010480288	8	0	0
FLYWCH1	0.2572781	0.278537671	-0.02125954	-0.07305925	0.003362388	0.010482841	7	0	0
RAB24	0.2231097	0.254357334	-0.03124768	-0.06711801	0.003365545	0.01049086	7	0	0
C10orf71	0.5907668	0.579308202	0.011458582	0.065275535	0.003369448	0.010501203	11	0	0
SNRPB	0.3311007	0.359574194	-0.02847349	-0.04983793	0.003373414	0.010511736	9	0	0
ETV2	0.4196368	0.407622309	0.012014539	0.329736117	0.003377232	0.010521806	8	0	0
YWHAE	0.3797136	0.407725587	-0.02801199	-0.13320185	0.003378458	0.010523796	7	0	0
TEX15	0.4515919	0.550456436	-0.09886451	-0.27998426	0.003379206	0.0105243	1	0	0
PROK2	0.1485602	0.107029431	0.041530728	0.4626837	0.003381453	0.010529468	9	0	0
ARG1	0.7665162	0.820335413	-0.05381925	-0.09665283	0.003385503	0.010540249	4	0	0
RRAGD	0.1284334	0.143487682	-0.01505432	-0.09978942	0.003393997	0.010560309	8	0	0
PROCA1	0.3099378	0.263787105	0.046150727	0.607691208	0.003395055	0.010560824	13	0	0
RARRES3	0.454526	0.468142389	-0.01361638	0.092904014	0.003400823	0.01057693	5	0	0
GALNT7	0.0449485	0.053044331	-0.00809579	-0.06653315	0.003405539	0.010589762	6	0	0
LDLRAP1	0.3738178	0.369016242	0.00480158	0.052696162	0.003406974	0.010590553	12	0	0
SNRPD1	0.2847416	0.314754068	-0.03001247	-0.112448	0.003406513	0.010590553	10	0	0
CPLX2	0.4612229	0.457465374	0.003757547	0.18598934	0.003409624	0.010595116	7	0	0
FDFT1	0.805619	0.832492366	-0.02687341	-0.04380845	0.003409054	0.010595116	2	0	0
KIF26A	0.1070178	0.075083595	0.031934199	0.523095267	0.003410711	0.010596659	9	0	0
IQCF5	0.6480182	0.698200606	-0.05018244	-0.10374695	0.003411823	0.010598278	8	0	0
NAGPA	0.2027275	0.230858358	-0.02813084	-0.05697241	0.00341429	0.010604105	10	0	0
UBB	0.2935757	0.296907407	-0.00333175	0.353403464	0.003416179	0.010608136	9	0	0
COPS3	0.0548459	0.06706849	-0.0122226	-0.09763345	0.003420184	0.010618733	13	0	0
TEAD1	0.0615156	0.049705543	0.011810032	0.26751582	0.003422515	0.010624132	6	0	0
GRIN3A	0.1699341	0.13077292	0.039161228	0.622252301	0.003426041	0.010629823	7	0	0
CD27	0.546201	0.605220724	-0.05901973	-0.14719769	0.003426127	0.010629823	5	0	0
FASTKD5	0.2383216	0.268423222	-0.03010161	-0.12350162	0.0034253	0.010629823	12	0	0
GFPT1	0.4505465	0.511314075	-0.0607676	-0.1126981	0.00343576	0.010657869	8	0	0
PCDHA12	0.4750198	0.432673069	0.042346705	0.204039895	0.003445718	0.010680911	10	0	0
IDNK	0.3100149	0.342115556	-0.0321007	-0.10197063	0.003447684	0.010680911	6	0	0
GLB1L2	0.1892268	0.132338685	0.056888136	0.595977329	0.003446726	0.010680911	8	0	0
GRIN2B	0.2282223	0.196179082	0.032043234	0.465096345	0.003447857	0.010680911	11	0	0
LALBA	0.8050115	0.848130816	-0.04311933	-0.07697296	0.003446745	0.010680911	4	0	0
OR4N5	0.5172297	0.498309495	0.018920197	0.100109749	0.003447528	0.010680911	3	0	0
KCNG4	0.3649596	0.372927039	-0.00796744	0.130139559	0.003446122	0.010680911	3	0	0
KRT31	0.7676711	0.803121664	-0.03545054	-0.06693519	0.003448036	0.010680911	4	0	0
SULT2A1	0.4722268	0.530023338	-0.05779651	-0.14128477	0.003448548	0.010680911	6	0	0
FNDC5	0.4857681	0.517645082	-0.03187696	-0.02908908	0.003451765	0.010685261	7	0	0
TXNIP	0.1237596	0.078890693	0.044868901	0.641599746	0.003451318	0.010685261	16	0	0
ATP8B3	0.0883256	0.088710551	-0.00038492	0.022618673	0.003452335	0.010685261	8	0	0
TXNRD2	0.0424727	0.060505997	-0.01803327	-0.20439259	0.003451692	0.010685261	11	0	0
10-Mar	0.4290529	0.452290727	-0.02323787	0.035538176	0.003454219	0.010689246	10	0	0
RNLS	0.8323446	0.869857933	-0.03751336	-0.06281203	0.003457092	0.010696292	6	0	0
SATB1	0.1108486	0.130920078	-0.02007149	-0.10159597	0.00345881	0.010699761	3	0	0
SERPINF1	0.6465998	0.684490964	-0.03789116	-0.09020134	0.003459802	0.010700985	7	0	0
WDR76	0.2975237	0.28881233	0.008711362	0.572446938	0.003461622	0.010704769	9	0	0
WRAP53	0.1394221	0.16680972	-0.02738763	-0.05014504	0.003462251	0.010704867	9	0	0
ZFP3	0.20573	0.166127357	0.03960264	0.904979264	0.003467303	0.01071864	8	0	0
PIEZO1	0.4375593	0.461283445	-0.02372417	-0.08279889	0.003470631	0.010727079	3	0	0
CDK12	0.2443098	0.252756398	-0.00844656	0.040960546	0.003479161	0.010751589	12	0	0
MAP10	0.7210093	0.724570911	-0.00356164	-0.04350728	0.003483598	0.010763448	10	0	0
OR2T34	0.5488655	0.679402543	-0.13053708	-0.30284559	0.003488641	0.010777172	1	0	0
CASS4	0.5935804	0.57781826	0.015762181	0.037543985	0.003496699	0.010800205	10	0	0
NANOGNB	0.7266585	0.774631254	-0.04797277	-0.09342282	0.003498099	0.010802669	3	0	0
DNAH1	0.8105567	0.833548935	-0.02299224	-0.03960602	0.003499364	0.010804717	5	0	0
FAM124A	0.2338414	0.251005628	-0.01716419	-0.05038404	0.003501172	0.010808436	8	0	0
CAPN7	0.3603084	0.371290052	-0.01098163	-0.00941607	0.003510942	0.010836732	5	0	0
DFFA	0.0977008	0.10281074	-0.00510991	-0.0087278	0.003513213	0.010841877	13	0	0
KCNK7	0.7558903	0.787715801	-0.03182547	-0.07060519	0.003516099	0.010848917	5	0	0
REEP2	0.2893966	0.274600201	0.014796366	0.076334192	0.003519999	0.010859083	11	0	0
RAC2	0.2405591	0.263349197	-0.02279012	0.212346406	0.003520953	0.010860157	10	0	0

UGT1A1	0.8312187	0.859119528	-0.02790081	-0.04994228	0.003528313	0.010880987	5	0	0
PLXNC1	0.2223602	0.245134817	-0.02277464	0.193318453	0.003532486	0.010891982	9	0	0
CLDN7	0.5654439	0.614934727	-0.04949084	-0.11473376	0.003534621	0.010896692	4	0	0
SPIDR	0.6719433	0.729118881	-0.05717563	-0.10676325	0.003535431	0.010897319	11	0	0
SPRY2	0.0769778	0.063029346	0.013948406	0.252596659	0.003541857	0.010915249	10	0	0
BBS1	0.2261454	0.2231649	0.002980507	0.026590386	0.003547364	0.010930342	9	0	0
SLC1A3	0.1383584	0.168023493	-0.02966513	-0.20918559	0.003553431	0.010943395	15	0	0
POM121L2	0.1066856	0.07083397	0.035851669	0.508516761	0.003553297	0.010943395	10	0	0
CT62	0.1820338	0.203932958	-0.02189913	0.010273566	0.003553005	0.010943395	11	0	0
ITPA	0.2354324	0.272902896	-0.03747048	-0.11695727	0.003561048	0.01096497	8	0	0
LGI2	0.1182043	0.104584443	0.013619859	0.29864053	0.003562079	0.010966264	9	0	0
MED21	0.2987363	0.310112299	-0.01137596	-0.0574358	0.003563448	0.010968595	9	0	0
ZC3HC1	0.3034586	0.324032619	-0.02057397	-0.0081101	0.003565267	0.010972312	13	0	0
IQCE	0.1660477	0.138195593	0.027852066	0.114098491	0.003569952	0.010983942	6	0	0
KLF16	0.0569427	0.075677898	-0.0187352	-0.12812884	0.003570271	0.010983942	5	0	0
RPS6KB1	0.161267	0.16129287	-2.59E-05	0.04929788	0.003573533	0.010992092	15	0	0
CGA	0.7223242	0.749141886	-0.02681765	-0.02871749	0.003575786	0.010997136	8	0	0
TGM3	0.6530735	0.708438421	-0.05536497	-0.11434115	0.00357824	0.011002797	10	0	0
SLC36A3	0.8184243	0.854884606	-0.03646033	-0.06323677	0.00357945	0.011004631	7	0	0
RASGRP2	0.1820028	0.134981564	0.047021245	0.692911682	0.003584808	0.011016424	9	0	0
NPB	0.7441878	0.77889482	-0.03470705	-0.06804748	0.00358476	0.011016424	6	0	0
PLCD3	0.442566	0.47236835	-0.02980238	-0.09237075	0.003586213	0.011017869	4	0	0
GALNT6	0.5143337	0.539710816	-0.02537712	-0.04018634	0.003586945	0.011018232	4	0	0
CTSB	0.471667	0.516789955	-0.04512296	-0.14642454	0.003588865	0.011022239	2	0	0
EXO1	0.1534901	0.168994566	-0.01550444	-0.12841863	0.003591169	0.011027427	7	0	0
KLHL3	0.4033445	0.375727909	0.027616623	0.145564365	0.00359938	0.01105075	8	0	0
STK32C	0.0592613	0.073522897	-0.01426163	-0.05866015	0.003603829	0.011062516	8	0	0
SULT1C2	0.4423057	0.396144874	0.046160802	0.235446556	0.003606251	0.011068056	3	0	0
BLOC1S4	0.4222857	0.453645528	-0.0313598	-0.04837571	0.003613829	0.011089415	9	0	0
PXK	0.1249446	0.111757833	0.0131868	0.116304377	0.003617036	0.011097358	10	0	0
MCCC2	0.186474	0.223640104	-0.03716606	-0.14531898	0.003625435	0.011121223	6	0	0
STK32A	0.1190527	0.116151073	0.002901607	0.303174499	0.003626084	0.011121313	13	0	0
MUM1	0.2163243	0.227575507	-0.01125122	0.097680182	0.003629718	0.011128653	7	0	0
EFNA2	0.2163243	0.227575507	-0.01125122	0.097680182	0.003629718	0.011128653	7	0	0
WSB1	0.2180979	0.270005605	-0.0519077	-0.08194338	0.003634422	0.011141171	11	0	0
RRN3	0.1544351	0.176896271	-0.02246113	-0.16241476	0.003639726	0.011155524	4	0	0
MCOLN3	0.054121	0.060134671	-0.00601362	0.042452585	0.003649819	0.011184546	9	0	0
TNFAIP6	0.7200948	0.770405846	-0.05031107	-0.10479707	0.003654867	0.011190456	6	0	0
CHST12	0.1034368	0.124514847	-0.02107809	-0.08700507	0.003654519	0.011190456	5	0	0
SEC31B	0.2862382	0.312670397	-0.02643217	-0.03364564	0.003653912	0.011190456	7	0	0
NDUFB8	0.2862382	0.312670397	-0.02643217	-0.03364564	0.003653912	0.011190456	7	0	0
ATP8A2	0.3257602	0.308068928	0.017691259	0.420510543	0.0036543	0.011190456	7	0	0
NOXRED1	0.8764152	0.893449628	-0.01703442	-0.02936367	0.003655909	0.011191736	5	0	0
H3F3C	0.8942741	0.935048127	-0.04077405	-0.07096023	0.003660192	0.011202936	5	0	0
CLU	0.2830954	0.277295789	0.005799571	0.17852057	0.003669189	0.011228557	16	0	0
SUPT16H	0.1283523	0.149712983	-0.02136071	-0.10876163	0.00367241	0.011236498	9	0	0
RUNX1T1	0.1665948	0.123151342	0.043443424	0.501346321	0.003673723	0.011238596	11	0	0
LONRF1	0.0359328	0.040485559	-0.00455274	-0.11018294	0.003677434	0.01124803	6	0	0
MAD2L1	0.0536306	0.065869743	-0.01223916	-0.1034314	0.003680124	0.01125242	8	0	0
MRVI1	0.6309921	0.616490272	0.014501785	0.030445871	0.00367983	0.01125242	14	0	0
SEZ6L	0.1683403	0.135208246	0.033132004	0.554491638	0.003684003	0.011262364	8	0	0
EXOSC2	0.3140632	0.287582658	0.026480508	0.086675678	0.003686975	0.011269527	11	0	0
NQO2	0.7371929	0.773465571	-0.03627264	-0.06295822	0.003689074	0.011274022	4	0	0
RNF125	0.2593961	0.282922206	-0.0235261	-0.13752803	0.003705532	0.011321037	12	0	0
SLC1A5	0.1935165	0.200603752	-0.00708729	-0.00280366	0.003705721	0.011321037	10	0	0
MBD3L1	0.1147317	0.099408943	0.015322778	0.274940214	0.003722528	0.011370447	6	0	0
GALT	0.0366853	0.036295196	0.000390085	0.279101155	0.003733336	0.011399576	7	0	0
SWAP70	0.1430669	0.160472589	-0.01740573	-0.12208357	0.003733253	0.011399576	13	0	0
SDC3	0.2562254	0.284460586	-0.02823522	0.012919818	0.003737593	0.011406749	6	0	0
TSSK2	0.7400926	0.775522102	-0.03542951	-0.06316737	0.003737444	0.011406749	8	0	0
PIM3	0.1633565	0.152450896	0.010905647	-0.01479401	0.003736929	0.011406749	10	0	0
GPR146	0.2402685	0.26518892	-0.02492042	0.034010704	0.003741056	0.011413434	7	0	0
ADIPOQ	0.7615491	0.809650014	-0.04810095	-0.08895438	0.003747286	0.011430498	7	0	0

GSTA3	0.6710934	0.730464024	-0.05937063	-0.14085371	0.003748108	0.011431061	3	0	0
DLGAP5	0.3098166	0.350291532	-0.04047494	-0.16299507	0.003749747	0.01143217	6	0	0
MC4R	0.3436954	0.361126761	-0.01743136	0.059595117	0.00374928	0.01143217	7	0	0
STC2	0.2403491	0.234164184	0.006184927	0.21681616	0.003751214	0.011434701	8	0	0
DYRK4	0.4217093	0.358264073	0.063445187	0.354807904	0.003754251	0.011442013	3	0	0
TLL2	0.0470664	0.054803658	-0.0077373	-0.07932842	0.00375849	0.011452986	9	0	0
LRRC8C	0.3002738	0.275842261	0.024431496	0.102173558	0.003765143	0.011467413	10	0	0
HAVCR2	0.1321226	0.128797163	0.003325469	0.118014768	0.00376502	0.011467413	12	0	0
TULP1	0.7988907	0.82333564	-0.02444492	-0.04804975	0.003764191	0.011467413	7	0	0
HS3ST4	0.1779505	0.149220271	0.028730248	0.366130326	0.00376918	0.011477762	7	0	0
SUSD5	0.412964	0.383966958	0.028997056	0.231194458	0.003770002	0.011478314	7	0	0
PRR19	0.3076423	0.332641516	-0.02499922	-0.06097518	0.003774701	0.011490671	12	0	0
ZNF681	0.3169866	0.313928157	0.003058485	0.438028233	0.003779132	0.011502209	6	0	0
EVL	0.2750009	0.276084179	-0.00108327	0.143872782	0.003784403	0.011516297	7	0	0
PEX14	0.1926405	0.194648688	-0.00200821	0.06666668	0.003788422	0.01152657	12	0	0
SOWAHB	0.1519173	0.175500054	-0.0235827	-0.25156817	0.003789218	0.011527039	8	0	0
FTCD	0.8026733	0.802330944	0.000342317	0.002299595	0.003791555	0.01153219	9	0	0
FAAH	0.3075993	0.28495102	0.022648252	0.427160527	0.003792983	0.011534579	11	0	0
CAPZA3	0.6692026	0.731508727	-0.0623061	-0.1269726	0.003806292	0.011573089	7	0	0
HHEX	0.8969498	0.913164806	-0.01621503	-0.02566955	0.003807047	0.011573423	5	0	0
GNG12	0.3646849	0.344434713	0.020250168	0.219433095	0.003811583	0.011585248	7	0	0
IL2	0.6252511	0.6935316	-0.06828054	-0.15507214	0.003812978	0.011587525	4	0	0
RXRA	0.5320499	0.579310892	-0.04726097	-0.1234474	0.003817463	0.01159919	3	0	0
SERPINA3	0.5745738	0.578145732	-0.0035719	0.020718443	0.003818136	0.01159927	5	0	0
LGR5	0.0776312	0.057013526	0.020617642	0.408397944	0.003819278	0.011600775	10	0	0
LYPD4	0.8141318	0.870552777	-0.056421	-0.09706148	0.003826945	0.011622095	12	0	0
PCDHGC5	0.6860137	0.726848635	-0.04083494	-0.10396686	0.003832092	0.011635756	4	0	0
LAMA5	0.0638274	0.037199053	0.026628347	0.489256512	0.003833547	0.011638201	4	0	0
ZBTB18	0.3609737	0.291388124	0.069585581	0.355858442	0.003837775	0.011647442	7	0	0
AFG3L2	0.0704613	0.062936879	0.007524431	0.118842936	0.003837889	0.011647442	10	0	0
OXTR	0.43236	0.479194672	-0.0468347	-0.08659479	0.00384775	0.011674017	8	0	0
GZF1	0.2670086	0.295635867	-0.02862726	-0.00305473	0.003847948	0.011674017	3	0	0
STRN4	0.3272015	0.343026822	-0.01582533	-0.10136602	0.003854254	0.011691173	8	0	0
TRIM3	0.2642896	0.260026389	0.004263173	0.113509623	0.003856364	0.011695593	13	0	0
UQCRC1	0.8520628	0.880542093	-0.02847933	-0.04688337	0.00386038	0.011705794	2	0	0
PAFAH1B3	0.6136109	0.574674288	0.038936599	0.113384126	0.003865562	0.011719528	3	0	0
PINK1	0.2465057	0.268763909	-0.02225825	-0.0368036	0.003871551	0.0117357	10	0	0
CNTN6	0.5339476	0.530537154	0.003410412	0.031285494	0.003873714	0.011740272	7	0	0
GOT1L1	0.7227966	0.730783126	-0.00798655	-0.00864041	0.003877181	0.011748795	9	0	0
KRT78	0.650602	0.632368308	0.018233729	0.049059371	0.003883511	0.011765988	9	0	0
CA7	0.2477874	0.242075975	0.005711443	0.357671752	0.003887375	0.011775706	14	0	0
KRTAP13-4	0.6114785	0.683162936	-0.07168448	-0.15981333	0.00388849	0.011777096	3	0	0
SOX13	0.300453	0.312132739	-0.01167974	0.07470279	0.003889291	0.011777532	6	0	0
MICU3	0.0635849	0.038837266	0.024747601	0.602238852	0.003898868	0.01180454	11	0	0
R3HDM4	0.0457944	0.040927554	0.004866872	0.126899394	0.003907519	0.011828737	4	0	0
EIF3A	0.1611772	0.1779799	-0.01680271	-0.0372975	0.003912348	0.011839359	6	0	0
NXPH4	0.0832234	0.06978336	0.013440032	0.059004557	0.003911749	0.011839359	5	0	0
NR3C2	0.0565393	0.045675409	0.010863895	0.257600011	0.003915889	0.01184681	7	0	0
FZD6	0.2129851	0.231256921	-0.01827186	-0.08967212	0.003916131	0.01184681	14	0	0
DHX36	0.2081401	0.250133527	-0.04199347	-0.10046304	0.003920676	0.011854596	13	0	0
CHRNA10	0.8270884	0.856690993	-0.02960264	-0.05285755	0.003920688	0.011854596	6	0	0
IGSF6	0.7647064	0.746966298	0.017740097	0.032971819	0.00391965	0.011854596	3	0	0
ADTRP	0.8891306	0.913001011	-0.02387045	-0.03780161	0.003922735	0.011858787	1	0	0
GCNT4	0.7309617	0.707149696	0.023811956	0.060565597	0.003930882	0.011881413	3	0	0
NR1I3	0.6395589	0.705391849	-0.0658329	-0.1485997	0.003936567	0.011896592	6	0	0
ESYT3	0.2019957	0.164969769	0.037025976	0.451334353	0.003940804	0.011907389	11	0	0
BNC1	0.243216	0.198076766	0.045139191	0.380874876	0.003948505	0.01192865	20	0	0
ITGB3	0.447989	0.447658836	0.000330187	0.117981735	0.003950095	0.011931443	6	0	0
VEPH1	0.5708594	0.627526945	-0.05666757	-0.13974464	0.00396993	0.011989337	2	0	0
IL17RC	0.2278145	0.187746631	0.04006787	0.550473601	0.003970673	0.011989561	9	0	0
YJEFN3	0.3291461	0.35232245	-0.02317636	-0.06873211	0.003971755	0.01199081	14	0	0
BTN3A3	0.2523422	0.287151836	-0.03480966	-0.052269	0.00397407	0.011995778	4	0	0
CTSF	0.4022072	0.423357393	-0.02115018	0.043699965	0.003974878	0.011996198	9	0	0

KY	0.2096349	0.18697076	0.022664106	0.421554332	0.003978368	0.012002966	7	0	0
RAB12	0.3373848	0.277133841	0.060250951	0.447221778	0.003978459	0.012002966	6	0	0
DENND5B	0.1204565	0.08184923	0.038607306	0.423893403	0.00398054	0.012007226	7	0	0
LRP11	0.2851315	0.307676524	-0.02254506	-0.20604335	0.003986025	0.012021748	7	0	0
NDEL1	0.7221299	0.778728412	-0.05659851	-0.11306156	0.003987196	0.01202326	2	0	0
VPS72	0.4173854	0.49120486	-0.07381943	-0.23473491	0.003988515	0.012023333	2	0	0
ZFYVE1	0.0766581	0.073195036	0.003463042	0.09674064	0.003988561	0.012023333	10	0	0
AKAP12	0.2978908	0.25089812	0.04699264	0.280233675	0.003990809	0.012028088	11	0	0
LBP	0.5700735	0.614029891	-0.04395636	-0.11169474	0.003991867	0.012029255	6	0	0
PLD5	0.0999788	0.072073982	0.027904811	0.512284658	0.003992588	0.012029408	9	0	0
PHC1	0.29843	0.251840697	0.046589334	0.137705228	0.004001661	0.012054718	5	0	0
USP44	0.6148634	0.604065866	0.010797522	0.027446437	0.004004268	0.012060545	6	0	0
EVI5	0.7179181	0.722041328	-0.00412325	-0.00564294	0.00400607	0.012063948	5	0	0
FAM19A3	0.6106431	0.645535379	-0.03489227	-0.03378358	0.004008755	0.012070005	7	0	0
ACTN1	0.0841273	0.109698336	-0.02557101	-0.04805629	0.004013005	0.012080776	14	0	0
ALB	0.8818722	0.896676163	-0.01480395	-0.02375034	0.00401656	0.012089447	1	0	0
CASQ2	0.7707593	0.745319892	0.025439398	0.059599782	0.004017679	0.012090786	8	0	0
FOXE3	0.7295647	0.709103459	0.020461268	0.048143414	0.004024379	0.012108918	6	0	0
TDRD9	0.7683755	0.796074011	-0.0276985	-0.04083169	0.004027829	0.012117267	5	0	0
MEX3D	0.412746	0.450936181	-0.0381902	-0.11029686	0.004029593	0.012120539	5	0	0
MGARP	0.4540094	0.450685946	0.003323448	0.255575567	0.0040428	0.012158225	13	0	0
PLCZ1	0.7159462	0.769105316	-0.0531591	-0.10667235	0.004043659	0.012158771	7	0	0
RPL22	0.4893347	0.447886757	0.041447921	0.170687811	0.00404612	0.012164131	4	0	0
LRRC2	0.7499949	0.784330775	-0.03433585	-0.06513524	0.004050639	0.012175676	6	0	0
KRT2	0.7492936	0.794831821	-0.04553821	-0.0816092	0.004051396	0.012175911	8	0	0
HSF1	0.1183071	0.148454317	-0.03014717	-0.15517668	0.004052319	0.012176646	6	0	0
TNIP2	0.1643947	0.173712686	-0.00931799	0.00706119	0.0040531	0.012176953	9	0	0
F13A1	0.3679377	0.386138923	-0.01820124	0.026822258	0.004054288	0.012178482	7	0	0
PDZK1IP1	0.7517599	0.789957587	-0.0381977	-0.07078849	0.004059285	0.01219145	4	0	0
PDZD2	0.0872367	0.056755801	0.030480907	0.391822092	0.004059975	0.012191479	3	0	0
CAPG	0.3001399	0.333263189	-0.03312325	-0.05428412	0.00406299	0.012198492	4	0	0
IFIT1	0.0979448	0.06515588	0.032788906	0.350371941	0.004065202	0.01220309	3	0	0
DHRS3	0.0544157	0.05829034	-0.00387469	-0.04840851	0.004068575	0.012209129	12	0	0
DMBT1	0.392262	0.419675958	-0.02741398	-0.02298525	0.004068493	0.012209129	4	0	0
ETS1	0.6039618	0.578030247	0.025931575	0.092783176	0.004069572	0.012210076	6	0	0
ELF2	0.0447804	0.04212905	0.002651387	0.082501355	0.004076978	0.012230251	6	0	0
TNFRSF19	0.4593502	0.505302206	-0.04595201	-0.12578568	0.004082684	0.012245323	9	0	0
ELMO1	0.2850735	0.306085086	-0.02101154	0.157168642	0.004084992	0.012250196	13	0	0
SYN2	0.2745707	0.246955984	0.027614714	0.464670375	0.004087314	0.01225511	10	0	0
HEPN1	0.7582135	0.80226247	-0.04404892	-0.07950585	0.004091015	0.012264155	5	0	0
PHF11	0.149803	0.169399772	-0.01959675	-0.12572198	0.004093474	0.012269476	8	0	0
ZNF283	0.2512216	0.19700345	0.05421817	0.282344283	0.004095018	0.012272054	7	0	0
SFRP5	0.2971125	0.260572955	0.036539502	0.522697051	0.004102393	0.012292102	11	0	0
KLK1	0.5458331	0.591559443	-0.0457263	-0.12226184	0.004105708	0.01229998	8	0	0
PTPRK	0.1012598	0.095054654	0.006205099	0.179231681	0.004107621	0.012303656	15	0	0
TNNI2	0.7482957	0.782066017	-0.03377033	-0.07071673	0.004109575	0.012307455	12	0	0
NRN1L	0.6592722	0.627710117	0.03156206	0.071421798	0.004113269	0.012316461	13	0	0
UBOX5	0.2900103	0.319773491	-0.02976317	-0.11705841	0.00411734	0.012326592	13	0	0
NHLRC1	0.3396748	0.363481855	-0.02380701	0.101413286	0.004125383	0.01234861	11	0	0
NIPAL2	0.3065901	0.333864155	-0.0272741	-0.09102101	0.004129333	0.01235722	8	0	0
ZNF483	0.3556866	0.370824432	-0.01513782	0.162773935	0.004129637	0.01235722	7	0	0
DNLZ	0.387883	0.395020817	-0.00713782	0.028727269	0.004134339	0.012369226	7	0	0
LRTM2	0.4037645	0.402410087	0.001354416	0.145472063	0.004144123	0.012396428	10	0	0
GRAMD1B	0.6294038	0.587051432	0.042352367	0.112973366	0.004146245	0.01240071	7	0	0
MIOS	0.1503081	0.172868708	-0.02256063	-0.0635537	0.004148092	0.012404163	8	0	0
SLC39A5	0.8178044	0.846459832	-0.02865546	-0.05299135	0.004157697	0.012430815	7	0	0
SLC38A11	0.540108	0.482734857	0.05737313	0.295624087	0.004159288	0.012431577	14	0	0
PRM1	0.8259793	0.857730599	-0.03175129	-0.05544126	0.004159338	0.012431577	8	0	0
IDUA	0.1663042	0.174479025	-0.00817487	0.118334423	0.004161611	0.012433929	6	0	0
PDLIM1	0.4046554	0.427077944	-0.02242253	-0.11073067	0.004161726	0.012433929	9	0	0
SLC28A1	0.6995203	0.746414213	-0.04689387	-0.09724775	0.004162205	0.012433929	5	0	0
MCCD1	0.6940103	0.715326213	-0.02131592	-0.01985009	0.004166775	0.012445509	5	0	0
C1orf122	0.451388	0.457106239	-0.00571829	-0.07835198	0.004179371	0.012481053	6	0	0

TP53I11	0.4163394	0.440831195	-0.02449181	-0.07220577	0.004181868	0.012486431	14	0	0
OTUB2	0.2682418	0.303692946	-0.03545112	-0.03900805	0.004183922	0.012490482	8	0	0
LRIT3	0.6301302	0.638437885	-0.0083077	-0.00911858	0.004186722	0.012496764	8	0	0
ANKRD30A	0.8419126	0.86808871	-0.02617616	-0.04400211	0.004192072	0.012505201	7	0	0
APOF	0.7730935	0.805836107	-0.03274263	-0.05996443	0.004192338	0.012505201	5	0	0
MYZAP	0.2374498	0.26072772	-0.0232779	-0.00277407	0.004191453	0.012505201	10	0	0
POLR2M	0.2374498	0.26072772	-0.0232779	-0.00277407	0.004191453	0.012505201	10	0	0
SLC25A21	0.2960977	0.328710319	-0.03261261	0.014068692	0.004203842	0.012537431	9	0	0
TRABD2A	0.076416	0.100856599	-0.02444059	-0.16226627	0.00420823	0.012546346	3	0	0
KRTAP27-1	0.7465558	0.743229566	0.003326242	0.015774145	0.004208102	0.012546346	5	0	0
ARRB2	0.0414855	0.037530747	0.003954775	0.078559457	0.004212815	0.012557928	9	0	0
TBL1XR1	0.0591915	0.043084048	0.016107465	0.260175023	0.004215166	0.012562849	10	0	0
NEUROG2	0.0576418	0.05775803	-0.00011624	0.180810959	0.004218517	0.012570746	7	0	0
TMEM14A	0.1155783	0.133020342	-0.01744208	-0.11385018	0.004227617	0.012595769	10	0	0
RADIL	0.1357927	0.107282388	0.02851031	0.512036531	0.004243488	0.012640955	4	0	0
GID4	0.109446	0.100584698	0.008861307	0.026806071	0.004256515	0.012676687	14	0	0
CSNK1E	0.213918	0.223626811	-0.00970885	0.05652332	0.004257085	0.012676687	6	0	0
CTBS	0.1999622	0.210850539	-0.01088839	-0.03282418	0.004259556	0.012680397	15	0	0
COX14	0.132713	0.149308185	-0.01659514	-0.06618891	0.004261126	0.012682967	11	0	0
NUDT19	0.2339231	0.283744336	-0.04982119	-0.09443945	0.004263405	0.012687644	5	0	0
TMEM50B	0.1829938	0.193905782	-0.01091199	0.001237774	0.004264783	0.012689639	11	0	0
CRYL1	0.1768082	0.144866355	0.031941856	0.119171702	0.00427053	0.012704632	7	0	0
PCDHB2	0.3577571	0.339730358	0.018026699	0.224662253	0.004271927	0.012706679	11	0	0
TALDO1	0.0440978	0.051189022	-0.00709122	-0.07584643	0.004277331	0.012720643	8	0	0
TYMP	0.0809698	0.085974757	-0.005005	-0.08693347	0.004278632	0.012722404	7	0	0
MPV17L	0.26172	0.22780673	0.033913286	0.62717196	0.00428123	0.012728019	5	0	0
LIN7A	0.2578891	0.214886482	0.043002596	0.482849836	0.004282334	0.012729191	11	0	0
FLNB	0.1096721	0.112991475	-0.00331934	-0.0879022	0.004294417	0.012762994	9	0	0
MYO1B	0.2733021	0.296263122	-0.02296098	-0.06519304	0.004298158	0.012767763	7	0	0
TRAPPC3L	0.6848158	0.704523424	-0.01970761	-0.03208669	0.004298107	0.012767763	7	0	0
ITGB7	0.4612291	0.497091366	-0.03586228	-0.12221046	0.004297647	0.012767763	6	0	0
C8orf48	0.1245811	0.1199398	0.004641294	0.238391476	0.004299191	0.01276827	8	0	0
IRGC	0.6826629	0.70621334	-0.0235504	-0.037223	0.004299752	0.01276827	8	0	0
ANKRD37	0.1332293	0.149323543	-0.01609424	-0.20721411	0.004301381	0.012770992	7	0	0
FAIM2	0.5448833	0.525354693	0.019528591	0.392379019	0.004302838	0.012773205	11	0	0
NAE1	0.13832	0.166312916	-0.02799296	-0.08667297	0.004309053	0.012789538	5	0	0
REG4	0.7858452	0.824919261	-0.03907408	-0.06844332	0.004312394	0.012797336	8	0	0
DAPK1	0.2894491	0.282607331	0.006841753	0.35803732	0.004313503	0.012798509	14	0	0
OR4K17	0.6250826	0.678597573	-0.05351495	-0.10900322	0.004323346	0.012825592	6	0	0
KIF3A	0.1677493	0.172780279	-0.00503101	-0.05002005	0.004329568	0.012837681	11	0	0
TUBB8	0.0764225	0.050613468	0.025808983	0.472773457	0.004329078	0.012837681	2	0	0
AMHR2	0.7459477	0.772264662	-0.02631698	-0.06450809	0.004328168	0.012837681	3	0	0
SLC24A5	0.620429	0.671187522	-0.05075853	-0.11497529	0.004336356	0.012855682	8	0	0
ADAM11	0.08528	0.05957947	0.025700523	0.591578018	0.004337361	0.012856536	8	0	0
GFRA1	0.1052783	0.072937103	0.032341183	0.552849587	0.00434027	0.012863035	16	0	0
HOPX	0.4572677	0.490209246	-0.03294157	-0.13187553	0.004347846	0.012883358	7	0	0
TACR2	0.7783228	0.809585008	-0.03126225	-0.056822	0.004351086	0.012890828	9	0	0
LAG3	0.5226112	0.55142272	-0.02881155	-0.07691316	0.004351853	0.012890973	12	0	0
ALDH2	0.2786102	0.311750766	-0.03314058	-0.1066813	0.004356088	0.012901385	12	0	0
MKI67	0.2199316	0.247212307	-0.02728066	-0.04313525	0.004361388	0.012914951	8	0	0
KIAA0232	0.7454159	0.773781889	-0.02836596	-0.04741798	0.004368049	0.012928272	6	0	0
FAM160A1	0.0536301	0.070574085	-0.01694395	-0.08445253	0.004367424	0.012928272	7	0	0
COL5A1	0.1934115	0.177943684	0.015467772	0.294938562	0.004366748	0.012928272	7	0	0
TNFRSF18	0.1358196	0.14117387	-0.00535427	0.235760143	0.00437176	0.012935776	8	0	0
SAMD3	0.1476636	0.109358696	0.038304939	0.79603954	0.00437405	0.012935776	13	0	0
ZFPM2	0.2254507	0.202300015	0.02315064	0.372256911	0.004374191	0.012935776	9	0	0
C12orf76	0.6778614	0.712147058	-0.03428568	-0.09370589	0.004373136	0.012935776	3	0	0
CLPP	0.4234518	0.449140317	-0.0256885	-0.05168441	0.004372601	0.012935776	6	0	0
F2RL1	0.1539262	0.169221766	-0.01529557	-0.01514939	0.004377696	0.012941874	9	0	0
SPAG5	0.0429441	0.054116636	-0.01117258	-0.15351807	0.004377137	0.012941874	13	0	0
PABPC4	0.3133808	0.315857731	-0.00247695	0.055739515	0.004383666	0.012957389	8	0	0
ALDOA	0.2919181	0.309595867	-0.01767775	-0.05364898	0.004398117	0.012997962	9	0	0
RFXAP	0.1884674	0.205841761	-0.01737435	-0.05125882	0.004403553	0.013011882	11	0	0

OR13C3	0.805519	0.839547109	-0.0340281	-0.0600137	0.004404477	0.013012469	8	0	0
RPAIN	0.0499579	0.046237669	0.003720225	0.100328128	0.004405626	0.01301372	17	0	0
FAM189B	0.2395008	0.221005457	0.018495308	0.113539062	0.004406702	0.013014755	8	0	0
NFKBIL1	0.4163082	0.43841384	-0.02210563	-0.02746011	0.004408963	0.013019287	13	0	0
TTC7B	0.4047769	0.428990392	-0.02421353	-0.03843264	0.004410985	0.013020972	7	0	0
TRIM65	0.3476502	0.318677385	0.028972803	0.282976002	0.00441032	0.013020972	12	0	0
HOMER3	0.4628694	0.48921811	-0.0263487	0.004144866	0.004414018	0.01302778	8	0	0
CLPSL1	0.7733876	0.805507451	-0.03211981	-0.05755634	0.004419259	0.013040651	6	0	0
CDC42BPG	0.2452938	0.236921564	0.008372213	-0.05017496	0.004419833	0.013040651	5	0	0
ACOT7	0.117395	0.132049493	-0.0146545	-0.07516483	0.004430421	0.013068737	10	0	0
LHX1	0.1368471	0.081037046	0.05581007	0.84733192	0.004430809	0.013068737	14	0	0
ZNF587	0.6053031	0.617258644	-0.01195551	0.049989265	0.004438008	0.013087818	6	0	0
HNRNPH3	0.5184204	0.519713648	-0.0012933	0.039948365	0.004450058	0.013121196	5	0	0
PLB1	0.814816	0.855279783	-0.04046379	-0.07296472	0.004452057	0.013121988	2	0	0
KDELC2	0.4093255	0.391859844	0.017465621	0.11319645	0.004452064	0.013121988	7	0	0
SERPINA9	0.7081896	0.770566657	-0.06237707	-0.12632532	0.004452521	0.013121988	4	0	0
KCNE4	0.5557761	0.508062426	0.047713691	0.150217521	0.004458119	0.013136328	13	0	0
MEMO1	0.0483168	0.058179125	-0.00986233	-0.10506792	0.004463749	0.013150757	6	0	0
KRT75	0.7153495	0.761424271	-0.04607476	-0.08866364	0.004466268	0.013154486	6	0	0
FBXO22	0.386065	0.429358918	-0.04329396	-0.09881213	0.004466482	0.013154486	8	0	0
NRG1	0.1357303	0.099618061	0.036112211	0.451389929	0.00447172	0.013167751	6	0	0
ARGLU1	0.1515795	0.179536376	-0.02795684	-0.09452154	0.004479857	0.013189545	7	0	0
IMMP2L	0.2139037	0.254576524	-0.04067282	-0.08377529	0.004481288	0.013191595	10	0	0
LUC7L3	0.1079675	0.144245089	-0.03627754	-0.11007237	0.004483045	0.013194601	11	0	0
OR10G3	0.6348386	0.667255961	-0.03241733	-0.06475138	0.004484101	0.013195543	6	0	0
TENM4	0.1292693	0.10639188	0.022877439	0.140329308	0.004487127	0.013202282	11	0	0
ATF3	0.7484767	0.769176667	-0.02069994	-0.04203211	0.004495757	0.013222906	6	0	0
FAM84A	0.1629348	0.142255993	0.020678825	0.382120749	0.004496208	0.013222906	9	0	0
FBXO7	0.1559379	0.186497118	-0.0305592	-0.10272897	0.004496348	0.013222906	12	0	0
SLC5A8	0.2195297	0.198575753	0.020953974	0.303512124	0.004497836	0.013225113	12	0	0
SLIT2	0.1560418	0.120654001	0.035387832	0.328705966	0.004501689	0.013234272	13	0	0
UBLCP1	0.1646317	0.196966097	-0.03233439	-0.13912189	0.004503715	0.013238058	9	0	0
ZNF879	0.1619065	0.121973722	0.0399328	0.810692303	0.004504464	0.013238089	7	0	0
FAM47E-STI	0.1114564	0.096687765	0.014768663	0.340947484	0.004506203	0.013240444	13	0	0
SLC25A25	0.1605724	0.172477323	-0.01190495	0.009900041	0.004506741	0.013240444	9	0	0
DNASE2	0.1328711	0.157528962	-0.02465787	-0.02347537	0.004516502	0.013264349	10	0	0
MVB12A	0.3331712	0.387784941	-0.05461378	-0.20361064	0.004516693	0.013264349	9	0	0
YWHAB	0.2272428	0.22661731	0.000625514	0.077217855	0.004517097	0.013264349	6	0	0
FFAR4	0.3832419	0.368935678	0.014306259	0.247906479	0.004521979	0.013276513	6	0	0
PDZD3	0.71216	0.748861299	-0.03670134	-0.0673598	0.004531018	0.013300875	6	0	0
TSSK1B	0.7908891	0.829605881	-0.03871682	-0.06866847	0.004532135	0.013301688	5	0	0
BAX	0.077681	0.094301823	-0.01662083	-0.09866969	0.004532779	0.013301688	10	0	0
ID3	0.2006521	0.160035887	0.040616209	0.265907886	0.004536576	0.013309456	13	0	0
FUOM	0.3353665	0.322345985	0.013020522	0.098723161	0.00453691	0.013309456	11	0	0
CCDC39	0.8055499	0.830523698	-0.02497379	-0.04317635	0.004544154	0.013328527	2	0	0
DRAXIN	0.0829728	0.08440895	-0.00143614	0.177647092	0.004545255	0.013329576	8	0	0
KNG1	0.7159205	0.781040574	-0.06512009	-0.12429197	0.004560541	0.013372218	4	0	0
RBM46	0.7052481	0.718314556	-0.01306646	-0.02453289	0.004569896	0.013397458	16	0	0
MSH3	0.0959607	0.114104022	-0.01814332	-0.12386091	0.004571706	0.013400575	7	0	0
TBC1D3C	0.81473	0.84923581	-0.03450581	-0.06182859	0.004577101	0.013414197	2	0	0
CPN2	0.7197403	0.745754433	-0.0260141	-0.04796303	0.004580696	0.013422541	8	0	0
ERC2	0.1670077	0.159730618	0.00727711	0.272109594	0.004585563	0.013434607	8	0	0
TMEM161B	0.1856832	0.218826552	-0.03314338	-0.11916171	0.004589742	0.013441336	9	0	0
OGDH	0.0511842	0.047262122	0.003922102	0.070730231	0.004591607	0.013441336	8	0	0
DNAI1	0.470711	0.410895493	0.059815552	0.19289145	0.004590739	0.013441336	4	0	0
SPSB3	0.6292721	0.58665207	0.042620075	0.096863965	0.004590187	0.013441336	8	0	0
JOSD2	0.4199274	0.448688095	-0.02876071	0.002226693	0.004590936	0.013441336	8	0	0
SKA3	0.0897259	0.104808285	-0.01508238	-0.12610698	0.004594397	0.013447308	10	0	0
CAPN9	0.7043107	0.690174346	0.014136323	0.037080421	0.004597533	0.013453525	6	0	0
PSTPIP2	0.223553	0.188956768	0.034596194	0.590340334	0.004598021	0.013453525	14	0	0
VAT1L	0.2549881	0.264471765	-0.00948371	0.353677479	0.004608925	0.01348323	9	0	0
POU6F2	0.7390806	0.794633612	-0.05555305	-0.10457108	0.004620138	0.013513827	6	0	0
ADH1B	0.4148001	0.356506811	0.058293255	0.246384797	0.004626798	0.013531102	3	0	0

YDJC	0.4383327	0.461286831	-0.02295412	-0.06156917	0.004634511	0.01355145	9	0	0
SNX13	0.1506569	0.192117025	-0.04146009	-0.12133163	0.004639459	0.013561495	9	0	0
RASSF9	0.3444664	0.377053737	-0.03258738	-0.14387296	0.004639386	0.013561495	9	0	0
ABHD2	0.5013405	0.524565246	-0.02322477	-0.13145234	0.004640386	0.013561995	5	0	0
OPRD1	0.4130616	0.420809201	-0.00774756	0.2857357	0.004648005	0.01358205	6	0	0
DNM1	0.3889019	0.417634916	-0.02873301	-0.0983399	0.004651636	0.013590444	8	0	0
TMPPE	0.0445943	0.047612312	-0.00301805	0.023547918	0.004653664	0.013594155	10	0	0
STX5	0.160139	0.139081825	0.021057223	0.08070034	0.004658598	0.013606353	11	0	0
ATP11B	0.0506013	0.055943826	-0.00534255	-0.05506108	0.004669152	0.013632739	8	0	0
RHBDL1	0.1813238	0.137209076	0.044114696	0.513256459	0.0046686	0.013632739	13	0	0
LDB1	0.0730958	0.057016691	0.01607915	0.271318129	0.004671295	0.013636774	14	0	0
NDUFA4	0.0960599	0.112037086	-0.0159772	-0.07928788	0.004672142	0.013637029	10	0	0
KRT86	0.573287	0.53503793	0.038249045	0.115356899	0.004676628	0.013647902	6	0	0
SF3A3	0.7822101	0.824310126	-0.04210007	-0.08087962	0.004681213	0.013659059	4	0	0
TRAPPC6B	0.4708013	0.481245011	-0.01044372	-0.01965593	0.004682962	0.013661939	6	0	0
VPS37C	0.1512973	0.170601843	-0.01930455	0.034891499	0.004686114	0.013668912	11	0	0
MXRA8	0.5415794	0.500371496	0.041207868	0.121249658	0.004687251	0.013670004	8	0	0
CDK4	0.3359431	0.367955999	-0.0320129	-0.12151036	0.004702461	0.013712136	7	0	0
RPS27A	0.2541475	0.281566633	-0.02741918	-0.11931397	0.004705941	0.013718556	10	0	0
GNAT3	0.7623827	0.816707071	-0.05432439	-0.10324247	0.004706193	0.013718556	5	0	0
PTPRE	0.3156232	0.334321638	-0.01869848	0.011036938	0.004707213	0.013719301	9	0	0
ZNF765	0.7417717	0.771010867	-0.02923913	-0.0457545	0.004715402	0.013740935	5	0	0
FOXD3	0.1710197	0.134061162	0.036958522	0.25127318	0.00471881	0.013746397	12	0	0
ARF4	0.0651079	0.056568779	0.00853909	0.187736478	0.004718138	0.013746397	4	0	0
CDH17	0.5862828	0.639728225	-0.05344546	-0.12347615	0.004725446	0.013763494	5	0	0
CACYBP	0.2003747	0.241189355	-0.04081464	-0.09573281	0.004729921	0.013772056	12	0	0
COLEC10	0.6737389	0.726700223	-0.05296132	-0.12337473	0.004729744	0.013772056	4	0	0
HIBADH	0.2333541	0.278670506	-0.0453164	-0.08935272	0.004736203	0.013788108	11	0	0
KAZN	0.2626046	0.229310207	0.033294383	0.328535268	0.00473783	0.013790608	11	0	0
GTF2IRD1	0.0877944	0.102336243	-0.0145418	0.141357846	0.004746671	0.013814098	12	0	0
PWWP2A	0.2275926	0.232978815	-0.00538619	0.037478465	0.004757324	0.013842855	9	0	0
FLG2	0.6578805	0.713061518	-0.05518104	-0.11224595	0.004758863	0.013843561	3	0	0
PPA1	0.0508542	0.058882828	-0.00802861	-0.11625792	0.00475911	0.013843561	9	0	0
NTN1	0.3681331	0.336566703	0.031566372	0.107520297	0.004761323	0.013847752	7	0	0
CCRL2	0.2547664	0.199773316	0.054993048	0.428828132	0.00476943	0.013869081	12	0	0
TRAK1	0.1503249	0.168379372	-0.01805451	0.001342785	0.004773509	0.013874564	5	0	0
TTC23L	0.1738606	0.188668166	-0.01480757	-0.11918118	0.004772469	0.013874564	8	0	0
FUT8	0.8804059	0.902599226	-0.02219334	-0.03597938	0.004773636	0.013874564	3	0	0
P2RY14	0.6552333	0.632564872	0.02266847	0.058143962	0.004776995	0.013882077	8	0	0
FAM20A	0.1856404	0.155821858	0.029818496	0.581080284	0.004778646	0.013884625	5	0	0
P2RY12	0.7634706	0.812246839	-0.04877628	-0.08794203	0.004782873	0.013894656	2	0	0
RNF144A	0.2232699	0.249983481	-0.02671356	0.106792706	0.004784451	0.013896989	9	0	0
TBC1D12	0.3151352	0.35186314	-0.03672796	-0.08679178	0.004787132	0.013902525	13	0	0
DUOX1	0.1821539	0.151843745	0.030310154	0.472679965	0.004789026	0.013905774	17	0	0
DEAF1	0.1302879	0.148394353	-0.01810649	-0.18230052	0.00479485	0.013919755	5	0	0
BRCA1	0.2390303	0.269388533	-0.03035821	0.000165074	0.004795393	0.013919755	31	0	0
SLC18B1	0.0531737	0.0609794	-0.00780567	-0.11910022	0.004808981	0.013954682	7	0	0
TPBGL	0.3314436	0.319430155	0.012013472	0.467440342	0.00481096	0.013955907	9	0	0
NR4A1	0.4021388	0.431888589	-0.0297498	-0.11087078	0.004810637	0.013955907	8	0	0
SNAPIN	0.2568631	0.302027697	-0.04516456	-0.13298681	0.00481602	0.013968326	9	0	0
ARHGAP33	0.2470498	0.294830899	-0.04778109	-0.24596121	0.004819049	0.013974853	1	0	0
OSR1	0.2480041	0.212232172	0.035771886	0.510558868	0.004822794	0.013982588	10	0	0
FAR2	0.0771464	0.061001877	0.016144535	0.409496808	0.004823276	0.013982588	11	0	0
SUMO4	0.8747881	0.869364949	0.005423128	0.009831064	0.004825415	0.013986529	8	0	0
NOVA1	0.1588602	0.119933139	0.038927096	0.421080468	0.004836489	0.014016364	11	0	0
ZNF501	0.0892274	0.06544861	0.023778804	0.615214503	0.004841526	0.014028693	8	0	0
ANKRD24	0.2363174	0.271884582	-0.03556719	-0.04598186	0.004849997	0.014050969	12	0	0
MTX2	0.3340473	0.374212083	-0.04016479	-0.09635123	0.00485707	0.014069187	9	0	0
C1orf53	0.2144196	0.225028386	-0.01060881	-0.09359192	0.004859484	0.014073906	10	0	0
RPL22L1	0.1971535	0.228899314	-0.03174582	-0.12790247	0.004860843	0.01407557	11	0	0
CD9	0.4046253	0.443525319	-0.03890003	-0.18178202	0.004863146	0.014079965	6	0	0
SH3GL3	0.2648037	0.260867972	0.003935776	0.345580971	0.004868183	0.014092274	10	0	0
LARP7	0.0678664	0.058667535	0.009198898	0.172887498	0.004872713	0.014100085	17	0	0

CACFD1	0.1968854	0.206723959	-0.00983854	0.087606283	0.00487324	0.014100085	7	0	0
GRIN2C	0.1699151	0.134660246	0.035254841	0.475009467	0.004880242	0.014118068	6	0	0
VPS13D	0.5475206	0.586596442	-0.03907583	-0.07147709	0.004887532	0.014136878	4	0	0
DOHH	0.2207826	0.248142453	-0.02735981	-0.06445701	0.004894188	0.014153845	10	0	0
ZNF345	0.1006436	0.076850141	0.023793464	0.520592066	0.00489533	0.014154867	16	0	0
C4orf17	0.7283015	0.742538051	-0.01423657	-0.0189897	0.004899588	0.014164895	4	0	0
ACTG1	0.2916582	0.306751386	-0.01509321	-0.06866177	0.004911779	0.01419785	2	0	0
CCL28	0.6804971	0.730935987	-0.0504389	-0.12438655	0.004912853	0.01419792	5	0	0
CEBPA	0.1302037	0.095576425	0.034627247	0.267513922	0.004913386	0.01419792	7	0	0
PRRG4	0.034163	0.037775849	-0.0036129	0.005045895	0.004914633	0.014199235	7	0	0
OCEL1	0.1478776	0.1750125	-0.02713489	-0.02448562	0.004922034	0.014218329	8	0	0
CNTN2	0.2121458	0.203765069	0.008380682	0.354837889	0.004925574	0.014223972	9	0	0
MEFV	0.5041954	0.549390684	-0.04519533	-0.10697689	0.004925062	0.014223972	6	0	0
PLEKHG6	0.3724583	0.395957638	-0.02349933	0.044181101	0.004930919	0.014237115	12	0	0
PIANP	0.1974114	0.203810363	-0.00639898	0.205062592	0.004932662	0.014239855	14	0	0
VPS39	0.1544317	0.181540632	-0.0271089	-0.18168954	0.004934558	0.014243036	13	0	0
PRSS3	0.5513025	0.561824476	-0.01052202	0.046230938	0.004941023	0.014259404	5	0	0
KLHL42	0.3635913	0.397709238	-0.03411799	-0.15181718	0.004943161	0.014263278	7	0	0
PCDHA4	0.4125628	0.383659011	0.028903833	0.277319325	0.004951244	0.014284304	8	0	0
SHISA5	0.2719851	0.245706177	0.026278967	0.170561244	0.004952271	0.014284967	10	0	0
CHRNA4	0.4506403	0.476520698	-0.02588037	-0.05043022	0.004954023	0.014287724	5	0	0
LIME1	0.8129588	0.83645865	-0.0234999	-0.041042	0.004960009	0.014302689	4	0	0
PRPF3	0.2327517	0.230994494	0.001757255	0.079395109	0.004964001	0.014311897	8	0	0
SEC24B	0.2903428	0.309807635	-0.0194648	-0.17141317	0.004968008	0.014321151	3	0	0
TAS1R3	0.599888	0.65057239	-0.05068443	-0.11859665	0.004968997	0.014321697	5	0	0
KIF16B	0.2355441	0.270215663	-0.03467154	-0.09547913	0.004972407	0.014329224	13	0	0
OSBPL2	0.0357913	0.041549028	-0.00575773	-0.09685895	0.00497992	0.01434857	8	0	0
TINAGL1	0.353874	0.391122283	-0.03724831	-0.06271049	0.004986668	0.014365704	14	0	0
TRIM64C	0.5022691	0.561717217	-0.0594481	-0.16958536	0.004990859	0.014375469	2	0	0
CCDC54	0.6394723	0.686716441	-0.04724414	-0.11360793	0.004992067	0.014376641	3	0	0
CLDN1	0.2851107	0.320926213	-0.0358155	-0.13957454	0.004997453	0.014389841	7	0	0
CASKIN1	0.1635907	0.142716031	0.020874656	0.230497704	0.005004212	0.01440699	6	0	0
LRRC66	0.8305166	0.853434628	-0.02291808	-0.03986806	0.005006628	0.014411633	6	0	0
CDC25A	0.2171039	0.232428182	-0.01532432	-0.07020303	0.005013419	0.014421789	9	0	0
PLCXD3	0.1531422	0.117270919	0.035871271	0.402341213	0.005015693	0.014421789	10	0	0
ENPP4	0.1652368	0.166881401	-0.00164463	0.47522932	0.005012422	0.014421789	10	0	0
RP9	0.2187564	0.245126249	-0.02636988	-0.08710228	0.00501433	0.014421789	6	0	0
TBC1D2	0.0486518	0.0598776	-0.01122584	0.022540119	0.005015785	0.014421789	8	0	0
STXBP6	0.3101081	0.262837022	0.047271123	0.355979824	0.005015526	0.014421789	4	0	0
ZNF419	0.3022107	0.302172873	3.78E-05	0.43223368	0.005011913	0.014421789	8	0	0
PHLDB2	0.792536	0.848202726	-0.05566674	-0.10189631	0.005017694	0.014422653	6	0	0
KCNJ5	0.1296028	0.114972485	0.014630291	0.293945194	0.005017149	0.014422653	10	0	0
CCR7	0.5246746	0.596533132	-0.07185857	-0.18485858	0.005023826	0.014437966	3	0	0
ELP6	0.162941	0.174707795	-0.0117668	-0.02008485	0.00502582	0.014441384	13	0	0
KHSRP	0.0806155	0.090740182	-0.01012469	-0.09459451	0.005030173	0.014451575	7	0	0
PCLO	0.1300803	0.145676697	-0.01559638	0.093205329	0.005032723	0.014454994	7	0	0
OR6C3	0.7231716	0.786699764	-0.06352814	-0.12565975	0.005032975	0.014454994	3	0	0
ARHGAP1	0.0392957	0.036666825	0.002628838	0.079174902	0.005037005	0.014464251	9	0	0
ZNF557	0.1654216	0.176276196	-0.01085462	0.010729089	0.005050951	0.014501875	13	0	0
ZNF69	0.1833718	0.1388963	0.044475545	0.819262669	0.005051724	0.014501875	9	0	0
MTMR7	0.4523516	0.454453722	-0.00210212	0.10614799	0.005053849	0.014505656	7	0	0
RAB3B	0.1850893	0.19809706	-0.01300781	0.020274	0.005056357	0.014510531	14	0	0
GPR37	0.1438143	0.160815264	-0.01700093	-0.10707953	0.005061334	0.014522491	10	0	0
ADAMTS10	0.2988103	0.284820876	0.013989398	-0.00669587	0.005062432	0.014523318	4	0	0
COL11A2	0.1929976	0.156784648	0.036212982	0.384290905	0.005066849	0.014533663	28	0	0
BARX2	0.1166633	0.076263405	0.040399942	0.661120771	0.005071767	0.014545446	13	0	0
ACOT12	0.4109152	0.380804141	0.030111032	0.417895168	0.005077822	0.014560482	3	0	0
CENPW	0.4217265	0.463279754	-0.04155326	-0.10775217	0.005080605	0.014566135	9	0	0
HIGD1A	0.2552237	0.219322094	0.035901557	0.137465752	0.005084844	0.014575957	9	0	0
SDAD1	0.2192681	0.249498123	-0.03023004	-0.06560481	0.005086852	0.014579384	12	0	0
CIDEA	0.1778966	0.121312467	0.056584098	0.558602206	0.005093966	0.014597441	12	0	0
PDGFC	0.124969	0.093570311	0.031398719	0.376426168	0.005098908	0.014609266	12	0	0
ZNF737	0.4322838	0.418922386	0.013361371	0.32655354	0.005100407	0.014611228	4	0	0

SERPINC1	0.7956485	0.798106933	-0.00245842	-0.00340156	0.005102823	0.014615814	8	0	0
SLC22A18	0.4723128	0.498198736	-0.02588592	-0.05237805	0.005108099	0.01462859	12	0	0
POLR2D	0.1316153	0.175664591	-0.04404931	-0.13226633	0.005111875	0.01463473	9	0	0
LCN12	0.3047417	0.317335617	-0.01259392	0.023965923	0.005111275	0.01463473	3	0	0
XRN2	0.1410026	0.146067843	-0.00506525	-0.0101333	0.00511506	0.014641513	12	0	0
SOBP	0.0576602	0.056271057	0.001389156	0.089342437	0.005119372	0.014651516	6	0	0
BCAM	0.119846	0.099244243	0.020601785	0.269495678	0.005126697	0.014667801	7	0	0
ADRA1D	0.3676075	0.34012462	0.027482911	0.255482767	0.005129146	0.014672466	7	0	0
IFNLR1	0.2943834	0.321408854	-0.02702549	-0.09818535	0.005131926	0.014678078	10	0	0
CCR1	0.5559693	0.593073303	-0.03710403	-0.14797517	0.005133651	0.01468067	5	0	0
RAF1	0.0451131	0.038542145	0.00657099	0.139082832	0.005147637	0.014715972	8	0	0
PDE4A	0.2376125	0.252102943	-0.0144904	0.027569978	0.005146929	0.014715972	7	0	0
CHRNA9	0.7131027	0.74453217	-0.03142944	-0.05272495	0.005154103	0.014731046	7	0	0
RLN3	0.7241947	0.70663579	0.017558892	0.035862645	0.005154552	0.014731046	9	0	0
SLPI	0.3212998	0.337176501	-0.01587672	-0.04103344	0.005158056	0.014738712	9	0	0
SH3RF3	0.1043082	0.059142331	0.045165879	0.779774808	0.00516399	0.014753317	15	0	0
STXBP5L	0.2450996	0.249700003	-0.00460043	-0.05010412	0.005167732	0.014761657	10	0	0
PHB2	0.1508989	0.178520286	-0.02762137	-0.12589391	0.005180793	0.014791897	12	0	0
SLC15A1	0.1374599	0.100530309	0.036929577	0.519262225	0.00518067	0.014791897	3	0	0
VAC14	0.0291991	0.033649723	-0.00445061	-0.12523859	0.005180267	0.014791897	6	0	0
STAB2	0.6284683	0.668884754	-0.04041641	-0.08373445	0.005187291	0.014803188	6	0	0
CCDC182	0.6986515	0.75658198	-0.05793045	-0.12058537	0.00518798	0.014803188	6	0	0
FSCN2	0.4480797	0.416675197	0.03140455	0.099626705	0.005185698	0.014803188	8	0	0
LRRC32	0.2144387	0.181619255	0.032819482	0.502260833	0.005190755	0.014808553	7	0	0
RAD1	0.8192132	0.854027635	-0.03481448	-0.06045149	0.00519437	0.014814154	3	0	0
TMEM136	0.4106849	0.425211663	-0.01452674	0.049640429	0.005193618	0.014814154	9	0	0
MYF5	0.4697021	0.514240315	-0.04453824	-0.13313347	0.005198442	0.014823412	14	0	0
TRIM40	0.4860839	0.560295964	-0.07421209	-0.18901903	0.005201017	0.014826041	2	0	0
SLC25A52	0.7967355	0.844233863	-0.04749833	-0.08256818	0.005200444	0.014826041	7	0	0
KLHL18	0.0485811	0.050063374	-0.00148224	0.019224842	0.005211793	0.014852037	14	0	0
GPR182	0.6947296	0.699529034	-0.0047994	0.000205579	0.005211201	0.014852037	8	0	0
WSB2	0.2638901	0.293793908	-0.02990376	0.07871142	0.005213271	0.014853888	5	0	0
EPB41L1	0.6776647	0.719207668	-0.04154296	-0.09371324	0.005218619	0.014866763	8	0	0
CRH	0.6559496	0.711704809	-0.05575524	-0.1099237	0.005220187	0.014868242	13	0	0
PANX2	0.1488052	0.129807784	0.018997442	0.160455213	0.005220796	0.014868242	17	0	0
KRT33A	0.8037343	0.822112835	-0.01837858	-0.02033651	0.005223767	0.014874343	6	0	0
STAT5B	0.05173	0.040123187	0.011606789	0.190119148	0.005224852	0.01487507	9	0	0
SPRR3	0.6481408	0.686143889	-0.03800313	-0.07573908	0.005238229	0.014904915	2	0	0
PARP1	0.1982755	0.227310784	-0.02903533	-0.05771877	0.005239036	0.014904915	8	0	0
RPL24	0.2338512	0.297756314	-0.06390509	-0.16183985	0.005238089	0.014904915	10	0	0
KIAA1549	0.3487687	0.379018229	-0.03024953	-0.03484286	0.00523949	0.014904915	4	0	0
HTRA4	0.5165657	0.44832094	0.068244754	0.231743954	0.005236935	0.014904915	12	0	0
CXXC5	0.5161292	0.492567543	0.023561676	0.077254849	0.005240606	0.014905726	5	0	0
PLXNB1	0.2913655	0.317640963	-0.02627547	-0.07792649	0.005247293	0.014922379	11	0	0
LBR	0.2222022	0.257579882	-0.03537769	-0.10975742	0.0052565	0.014946193	9	0	0
OAS3	0.2853218	0.312781973	-0.02746014	-0.03421436	0.005264321	0.014966059	9	0	0
ACRV1	0.8235189	0.826621172	-0.00310232	-0.004428	0.005273599	0.014990058	4	0	0
HIST2H2AA4	0.0809262	0.102661657	-0.02173541	-0.18912521	0.00527682	0.014996838	5	0	0
LUC7L2	0.0451093	0.043963391	0.001145866	-0.03000577	0.005279878	0.015001409	15	0	0
HPX	0.5695069	0.58583901	-0.01633208	-0.02233557	0.005280101	0.015001409	5	0	0
BSX	0.2636043	0.245380466	0.018223836	0.164849763	0.005283869	0.015009737	11	0	0
PCDHGA10	0.3224269	0.259415694	0.063011231	0.435714894	0.005290643	0.015026599	11	0	0
CDK5RAP1	0.2951488	0.340403598	-0.04525484	-0.11014205	0.005296363	0.015040464	12	0	0
ARHGEF38	0.4891718	0.491052938	-0.00188112	-0.04594342	0.005303753	0.015059066	9	0	0
AP3B1	0.0619849	0.068850562	-0.00686563	-0.13383128	0.005311412	0.015077385	16	0	0
CDKL3	0.0509945	0.045531598	0.005462915	0.094139862	0.005311887	0.015077385	9	0	0
G0S2	0.2991529	0.23955574	0.059597154	0.561554951	0.005313402	0.0150793	10	0	0
ISG15	0.5288822	0.561795146	-0.0329129	-0.04287964	0.005318498	0.015091375	3	0	0
GRIN2A	0.0958258	0.058922243	0.036903583	0.657491288	0.005319489	0.0150918	13	0	0
GRK1	0.6917641	0.693665927	-0.00190181	0.007830608	0.005322537	0.015098058	12	0	0
SEL1L3	0.5403794	0.562083012	-0.02170361	-0.08096054	0.005324594	0.015101506	7	0	0
KCNJ11	0.2192402	0.232610559	-0.01337035	-0.13157108	0.005335235	0.015126994	13	0	0
FRY	0.1066606	0.118679654	-0.01201909	-0.1088353	0.005335268	0.015126994	13	0	0

KRT76	0.6587771	0.70779998	-0.04902292	-0.09898046	0.005342194	0.015142928	5	0	0
INPP5J	0.4870404	0.500411875	-0.01337147	0.009714613	0.005342576	0.015142928	7	0	0
ITGA10	0.8655824	0.859877603	0.005704829	0.010859796	0.005347625	0.015154843	5	0	0
UGT2A2	0.6612546	0.705818363	-0.04456379	-0.09348406	0.005362723	0.01519523	3	0	0
CUL5	0.318586	0.334111233	-0.01552527	0.013852744	0.005365458	0.015200577	6	0	0
DEPDC5	0.1329531	0.109708555	0.023244531	0.190856301	0.005367392	0.015203654	10	0	0
NACC1	0.0426015	0.046802037	-0.00420055	-0.09213618	0.005373459	0.015218437	7	0	0
KIR2DL1	0.8377651	0.886771802	-0.04900674	-0.08526146	0.005376512	0.01522468	4	0	0
SESN2	0.0905239	0.074427124	0.016096736	0.168724076	0.005383079	0.015240869	7	0	0
FOXE1	0.1044186	0.069995554	0.034423022	0.626135391	0.005384185	0.015241595	6	0	0
SPRR4	0.7761773	0.805823119	-0.02964584	-0.05095263	0.00539012	0.015255986	6	0	0
SCARB2	0.0498463	0.061592353	-0.01174605	-0.11921939	0.005393527	0.01526322	5	0	0
KIT	0.1705184	0.168975408	0.001542963	0.251917626	0.005399072	0.015276503	7	0	0
MX2	0.4243099	0.468749802	-0.04443995	-0.06832404	0.005413349	0.015314484	10	0	0
PERP	0.1063524	0.120518396	-0.01416602	-0.15055496	0.005418566	0.015324409	5	0	0
COLEC12	0.1455962	0.101808773	0.043787414	0.499950417	0.005418225	0.015324409	5	0	0
IL19	0.6375146	0.673664573	-0.03615	-0.06191103	0.005420107	0.015326349	8	0	0
HSPG2	0.2719208	0.263448364	0.008472434	0.073662607	0.005425574	0.015339392	10	0	0
PTTG2	0.810435	0.830936506	-0.02050151	-0.03923577	0.005426855	0.015340595	8	0	0
APH1A	0.1966722	0.169524651	0.027147551	0.131437239	0.005432532	0.015351803	10	0	0
RELT	0.3733878	0.387357606	-0.01396977	-0.04016483	0.005432434	0.015351803	8	0	0
REP15	0.691321	0.746859134	-0.05553814	-0.11413826	0.00543505	0.015356499	5	0	0
S100A16	0.6372081	0.681152707	-0.04394464	-0.1067375	0.00544026	0.015368798	4	0	0
FNDC9	0.752252	0.809278197	-0.05702618	-0.10380601	0.005453151	0.015400365	7	0	0
CLUAP1	0.2710719	0.239219948	0.031851919	0.538950686	0.005452358	0.015400365	12	0	0
PAQR4	0.3177853	0.296216071	0.021569224	0.046036299	0.005458666	0.015413513	9	0	0
RRP12	0.0622208	0.071868082	-0.00964728	-0.04394469	0.005467942	0.015437274	10	0	0
GMCL1	0.2392833	0.268662546	-0.02937926	-0.03836642	0.005473656	0.015450974	12	0	0
PLN	0.6046298	0.640494749	-0.035865	-0.07208968	0.005474933	0.015452146	3	0	0
AOX1	0.1883932	0.142699405	0.045693789	0.706901119	0.00548308	0.015465276	13	0	0
PPP3CC	0.2361779	0.222457404	0.01372051	0.202873983	0.005482485	0.015465276	10	0	0
C10orf55	0.7461698	0.775979291	-0.02980949	-0.05294538	0.005480464	0.015465276	4	0	0
WNT5B	0.1732892	0.126884412	0.046404784	0.30906631	0.005483896	0.015465276	2	0	0
BID	0.4013153	0.417153424	-0.01583809	-0.0088797	0.005481402	0.015465276	12	0	0
PAX2	0.2549437	0.215857091	0.039086596	0.459669125	0.005493086	0.01548876	4	0	0
SPOCK2	0.3667873	0.388312117	-0.02152478	-0.09989914	0.005494	0.0154889	10	0	0
PARD6B	0.2129272	0.24950267	-0.03657552	-0.06047194	0.00549914	0.015500955	9	0	0
ZGPAT	0.0886176	0.099618482	-0.01100089	-0.07686078	0.005502154	0.015507012	16	0	0
DCAKD	0.1470043	0.164868097	-0.01786383	0.022767928	0.005512299	0.015533166	12	0	0
ARNT	0.2130726	0.252526279	-0.03945366	-0.08345658	0.0055158	0.015537609	10	0	0
RIIAD1	0.6626216	0.685177328	-0.02255575	-0.04113116	0.005516475	0.015537609	7	0	0
TSSK6	0.269765	0.307134628	-0.03736963	-0.09559202	0.005515854	0.015537609	13	0	0
AP2S1	0.3273858	0.310727337	0.016658462	0.052295932	0.005518887	0.015541962	13	0	0
C16orf92	0.5380723	0.584415876	-0.04634357	-0.12890332	0.005521791	0.0155477	5	0	0
LZIC	0.0439997	0.042847859	0.001151881	0.043246026	0.005529295	0.015566385	10	0	0
MYADML2	0.7680812	0.817855272	-0.04977408	-0.08840498	0.005546914	0.015613536	7	0	0
RNASE9	0.7880607	0.824179572	-0.03611891	-0.06869354	0.005549552	0.01561851	5	0	0
ST8SIA2	0.1668521	0.134776759	0.032075345	0.413651901	0.005550654	0.015619162	6	0	0
DDX43	0.7826226	0.827477192	-0.04485461	-0.08231137	0.005552638	0.015622294	12	0	0
FGFR4	0.2719046	0.282375712	-0.01047116	0.034409171	0.00556338	0.01565006	13	0	0
TNFRSF8	0.3012867	0.283155258	0.018131413	0.546481127	0.00556692	0.015657562	11	0	0
KLHL6	0.4480771	0.372755681	0.075321387	0.307565101	0.005570839	0.015666129	9	0	0
XDH	0.5368592	0.574687112	-0.03782796	-0.07793198	0.005598035	0.015740141	5	0	0
GDPD3	0.6480751	0.678033193	-0.02995808	-0.05799129	0.00560208	0.015749045	12	0	0
TMEM132E	0.0982293	0.064408797	0.033820518	0.607167868	0.00560319	0.015749698	15	0	0
CXCR2	0.5712709	0.605587082	-0.03431614	-0.08519593	0.005609288	0.015764367	6	0	0
ZNF320	0.1040501	0.045547101	0.05850302	1.116879607	0.005614107	0.015775437	3	0	0
FARP1	0.2431283	0.276797653	-0.03366937	-0.01716933	0.005620029	0.015789606	8	0	0
EML4	0.3704951	0.39774015	-0.0272451	-0.10210056	0.005624541	0.015797332	9	0	0
UBE4A	0.2955128	0.317785856	-0.0222731	-0.05619516	0.005624337	0.015797332	11	0	0
CNGB1	0.6323827	0.672418108	-0.04003543	-0.08817921	0.005628285	0.015805374	7	0	0
ANAPC7	0.0423731	0.039251303	0.003121844	0.0881316	0.005635659	0.015823603	12	0	0
ZNF682	0.1891967	0.163907685	0.025289047	0.645106269	0.005639095	0.015830774	6	0	0

STC1	0.2256764	0.189540855	0.036135593	0.425411753	0.005642391	0.015837548	7	0	0
FOXQ1	0.0892914	0.072732294	0.016559061	0.279546735	0.005660775	0.015884176	7	0	0
CAMK2B	0.7236972	0.774442819	-0.05074559	-0.09619579	0.005661679	0.015884228	2	0	0
LUC7L	0.2130431	0.251174951	-0.03813186	-0.05584364	0.005672045	0.015910821	9	0	0
SHBG	0.3494503	0.32511339	0.024336945	0.080188678	0.00567488	0.015916124	13	0	0
PNPLA5	0.2701583	0.245432606	0.024725716	0.301745781	0.00567571	0.015916124	14	0	0
ZNF474	0.2774837	0.296353534	-0.01886985	0.064436322	0.005687919	0.015947869	4	0	0
CEACAM20	0.6283044	0.677699074	-0.04939472	-0.09857859	0.005690005	0.015951223	6	0	0
RASEF	0.2485011	0.191501972	0.056999168	0.444198454	0.005693319	0.01595802	10	0	0
PDE8B	0.4323125	0.415988521	0.01632399	0.181362064	0.005697838	0.015968192	6	0	0
NAPEPLD	0.3183243	0.372436641	-0.0541123	-0.13457365	0.005699245	0.015969639	6	0	0
OR5K4	0.8264353	0.870589881	-0.0441546	-0.07447848	0.005703493	0.015979044	2	0	0
RAB4A	0.3185109	0.298316801	0.020194083	0.142272434	0.005707398	0.015985471	12	0	0
PDE1A	0.3803293	0.403754194	-0.02342491	-0.14819466	0.005707569	0.015985471	7	0	0
FNDC7	0.6242173	0.659823963	-0.03560667	-0.06405822	0.005717024	0.016006953	3	0	0
ZNF610	0.5590237	0.535723921	0.023299763	0.284624921	0.005716242	0.016006953	8	0	0
C20orf144	0.7991174	0.812390864	-0.01327345	-0.02331902	0.005724048	0.016024119	9	0	0
ABHD1	0.230333	0.239729822	-0.00939686	0.425539812	0.005733428	0.016042866	10	0	0
TMEM225	0.6393095	0.714354033	-0.07504454	-0.18087221	0.005732471	0.016042866	2	0	0
ARHGDIB	0.3922134	0.433417105	-0.04120367	-0.18301891	0.005733362	0.016042866	12	0	0
GRIN1	0.3452704	0.354696272	-0.00942588	0.054417827	0.005738105	0.016053446	6	0	0
C1orf74	0.163831	0.175761532	-0.01193057	-0.11438567	0.005745218	0.016070841	8	0	0
PKD2	0.4508451	0.429868506	0.020976631	0.05107823	0.00575546	0.01609698	4	0	0
BEST2	0.4025141	0.437026547	-0.03451245	-0.10871955	0.005764039	0.016118459	5	0	0
TRAF2	0.358195	0.376873472	-0.01867848	0.115264391	0.005770705	0.016134584	2	0	0
SAP25	0.8328369	0.861329648	-0.0284927	-0.04965257	0.005784746	0.016171321	7	0	0
HCN4	0.2114395	0.199547793	0.011891692	0.394155913	0.005789902	0.016183212	11	0	0
FAM89A	0.1330915	0.098503344	0.034588186	0.257243794	0.00579271	0.016188539	6	0	0
PRKAR2A	0.1447275	0.150550207	-0.00582271	0.002436086	0.005794022	0.016189683	11	0	0
POLG	0.216575	0.20619455	0.010380413	-0.01613293	0.005796139	0.016193076	5	0	0
FASTKD1	0.1634647	0.180634697	-0.01716997	0.011961556	0.00579742	0.016194131	6	0	0
PPP1R15A	0.3450765	0.360833252	-0.01575676	-0.03024145	0.005798849	0.016195602	5	0	0
PITX3	0.1213867	0.082571379	0.038815356	0.341460293	0.005802293	0.016202698	11	0	0
C6orf163	0.7088943	0.689796784	0.019097487	0.03771827	0.005805057	0.016207892	4	0	0
AP3M2	0.1631561	0.187200352	-0.02404421	0.160283697	0.005806141	0.016208397	5	0	0
NMI	0.0869846	0.112005661	-0.02502105	-0.08225117	0.00581391	0.016225034	8	0	0
ATF5	0.4106458	0.44291143	-0.0322656	-0.11423282	0.005813167	0.016225034	9	0	0
PLAC9	0.7615335	0.795763655	-0.03423011	-0.05437507	0.005825521	0.016251595	3	0	0
SEC14L5	0.0761128	0.077230207	-0.00111742	0.043426382	0.005827634	0.016251595	7	0	0
C17orf49	0.1372692	0.157069955	-0.01980071	-0.10958937	0.005827958	0.016251595	11	0	0
KRTAP4-4	0.6647687	0.704163375	-0.0393947	-0.0958451	0.00582795	0.016251595	3	0	0
DAND5	0.04908	0.040288989	0.008791011	0.232427271	0.005832173	0.016260821	1	0	0
FAT1	0.1967727	0.172599282	0.024173415	0.316072117	0.005837204	0.016272317	7	0	0
TMEM232	0.1726726	0.187757599	-0.01508501	-0.02778733	0.005839554	0.016276338	11	0	0
DUT	0.1205446	0.133995104	-0.01345055	-0.11944418	0.005841504	0.016279245	10	0	0
OR1L3	0.2925786	0.340631846	-0.04805325	-0.2126471	0.005851981	0.016305908	1	0	0
DGKG	0.1834658	0.181282107	0.002183673	0.354797858	0.005858432	0.016320249	11	0	0
LYPD2	0.5876666	0.632247405	-0.04458083	-0.09523362	0.005858948	0.016320249	6	0	0
CHRNA3	0.3115485	0.289853551	0.02169492	0.289669739	0.005861976	0.01632615	12	0	0
GHDC	0.1640031	0.175727423	-0.01172437	-0.03545815	0.005867499	0.016338994	11	0	0
SERPINB2	0.7141227	0.777170564	-0.06304791	-0.12044218	0.005873247	0.016352462	1	0	0
RASSF2	0.3086981	0.268162043	0.040536026	0.237549773	0.005878442	0.016361845	7	0	0
FBXW12	0.8352598	0.839368196	-0.00410836	-0.00674875	0.005884696	0.016376713	6	0	0
EIF4EBP2	0.1370225	0.13128102	0.005741439	0.14177828	0.005892076	0.016388213	10	0	0
PAM16	0.8112983	0.829334505	-0.01803621	-0.03044154	0.005890762	0.016388213	4	0	0
TRPV3	0.5540204	0.597150414	-0.04313003	-0.10914772	0.005892483	0.016388213	9	0	0
PFKL	0.0562942	0.069050275	-0.01275611	-0.06280812	0.005891845	0.016388213	8	0	0
ZNF816	0.0735061	0.051567259	0.021938793	0.468592415	0.005900519	0.016408016	8	0	0
C6orf118	0.4412417	0.443618421	-0.00237675	0.113919026	0.005906949	0.01642335	5	0	0
WNT10A	0.2067672	0.171540774	0.035226464	0.203333968	0.005919953	0.016456956	12	0	0
OR7E24	0.8216263	0.87038082	-0.04875456	-0.0821924	0.00592253	0.016461567	1	0	0
CCDC17	0.6230334	0.652563576	-0.02953013	-0.04474758	0.005927038	0.016471245	5	0	0
ITIH4	0.7117593	0.761234224	-0.04947489	-0.09744261	0.005927848	0.016471245	2	0	0

IFNAR2	0.1846464	0.203206229	-0.01855984	-0.09504324	0.00593642	0.016492506	12	0	0
FAT4	0.0812894	0.062653144	0.018636251	0.319120953	0.005950771	0.016529817	11	0	0
BEST3	0.7488903	0.800269401	-0.05137908	-0.13200876	0.005955086	0.016539242	5	0	0
RNF17	0.8357429	0.864212188	-0.02846933	-0.05148824	0.005974696	0.016588567	9	0	0
EIF4EBP1	0.1361026	0.154831996	-0.01872944	-0.04474896	0.005985237	0.016615262	10	0	0
ANGEL2	0.2510337	0.296839617	-0.04580589	-0.07928344	0.005990668	0.016627766	16	0	0
CCT6A	0.1210586	0.143086767	-0.02202821	-0.03376551	0.006002309	0.016657498	14	0	0
SLC11A1	0.5951565	0.641612366	-0.04645584	-0.11274238	0.006011551	0.016680566	10	0	0
NHEJ1	0.4031442	0.426888704	-0.02374449	-0.00992931	0.006014669	0.016686636	10	0	0
MMP26	0.427474	0.43582477	-0.00835073	0.007645976	0.006018212	0.016693883	3	0	0
PCDH7	0.1000337	0.073770807	0.026262892	0.401496634	0.006020847	0.01669861	9	0	0
IL36B	0.6875848	0.738567315	-0.0509825	-0.11578665	0.006030327	0.016719733	5	0	0
C7orf55-LU(	0.2391936	0.244368466	-0.00517488	-0.08499751	0.006029875	0.016719733	13	0	0
ZNF580	0.0553317	0.048749772	0.006581976	0.153200987	0.006037412	0.016736788	1	0	0
ARSJ	0.1823102	0.169161442	0.013148789	0.300766241	0.006047189	0.016761302	11	0	0
FAM102B	0.2624855	0.292916054	-0.03043054	-0.02206359	0.006052433	0.016773245	6	0	0
ADAM32	0.59846	0.651825149	-0.05336516	-0.12911255	0.00605345	0.01677347	7	0	0
FBXO10	0.6964911	0.753874665	-0.05738353	-0.11266449	0.006056819	0.016776718	1	0	0
TVP23C-CDI	0.0322844	0.027690352	0.004594008	0.165928873	0.006058101	0.016776718	1	0	0
TVP23C	0.0322844	0.027690352	0.004594008	0.165928873	0.006058101	0.016776718	1	0	0
CSF2RB	0.6172628	0.605055947	0.012206896	0.051166039	0.006058363	0.016776718	5	0	0
SMIM1	0.1816899	0.176949918	0.004740007	0.044453477	0.006059452	0.016777142	13	0	0
AGAP3	0.1121496	0.085849993	0.026299624	0.18819474	0.00606435	0.016788111	9	0	0
BPIFB3	0.6138834	0.66505423	-0.05117079	-0.10838271	0.006065474	0.016788632	8	0	0
KCNK5	0.1838451	0.188647302	-0.00480217	0.167999861	0.006069029	0.01679588	10	0	0
PPY	0.7514372	0.781248774	-0.02981157	-0.05426482	0.006071657	0.016800562	8	0	0
ASB16	0.4459967	0.419773591	0.026223133	0.129889515	0.006076762	0.016812093	8	0	0
MATN1	0.7595901	0.783123153	-0.02353301	-0.04176692	0.006081636	0.016822982	8	0	0
DAPK3	0.0477322	0.043922733	0.003809436	0.092406685	0.006095124	0.016857693	6	0	0
SPRYD7	0.1053722	0.113672606	-0.0083004	-0.0032328	0.006108433	0.016891898	13	0	0
RILPL1	0.0743594	0.085396949	-0.01103752	-0.02094121	0.006114542	0.016906187	4	0	0
DIS3L2	0.3093143	0.323653091	-0.01433879	-0.00885481	0.006117877	0.016910192	10	0	0
S100B	0.4999855	0.547874193	-0.04788873	-0.15823573	0.006117536	0.016910192	7	0	0
CRTAC1	0.3342196	0.300954252	0.033265344	0.399818268	0.00611904	0.016910801	8	0	0
LTBP3	0.3940484	0.353122551	0.04092582	0.221795545	0.006121926	0.01691617	5	0	0
ABHD6	0.1296944	0.116476449	0.013217998	0.060654652	0.006123996	0.016919284	8	0	0
CAPZB	0.3010337	0.313514536	-0.01248087	0.047657797	0.006125621	0.016919352	6	0	0
MBD3	0.2222517	0.212095152	0.010156594	0.088286908	0.006125907	0.016919352	9	0	0
TJP2	0.0514449	0.060748092	-0.00930319	-0.07416927	0.006129163	0.016925739	7	0	0
TAS2R50	0.7949256	0.831298655	-0.0363731	-0.06323691	0.006130335	0.01692637	6	0	0
ZNF695	0.1898541	0.194163433	-0.00430937	0.471357463	0.006132775	0.016927893	9	0	0
LHB	0.3370147	0.355604441	-0.01858974	0.012303705	0.006132011	0.016927893	5	0	0
FAM208A	0.6888388	0.726752014	-0.03791321	-0.10516214	0.006141377	0.016948407	6	0	0
ZNF426	0.1323696	0.119760021	0.012609584	0.493663836	0.006142097	0.016948407	11	0	0
CLDND2	0.7184246	0.755281169	-0.03685659	-0.08408154	0.006149286	0.016965635	7	0	0
TNFAIP3	0.0694006	0.086845614	-0.01744501	-0.10609697	0.006151132	0.016968119	13	0	0
FABP7	0.6697	0.704840132	-0.03514015	-0.06711133	0.006164897	0.017003474	8	0	0
CYP2A13	0.6026094	0.664950138	-0.0623407	-0.13648741	0.006174056	0.017026117	6	0	0
KLF1	0.6763499	0.658950898	0.017399046	0.046375721	0.006176633	0.017030607	9	0	0
NRROS	0.1190309	0.084189759	0.034841154	0.688290042	0.00618236	0.017039922	8	0	0
PIGX	0.1190309	0.084189759	0.034841154	0.688290042	0.00618236	0.017039922	8	0	0
CTTNBP2	0.3588885	0.391090378	-0.03220189	-0.07106947	0.006182862	0.017039922	5	0	0
HINFP	0.2360327	0.276075626	-0.0400429	-0.10210144	0.00619216	0.017062927	11	0	0
TAF7	0.0612261	0.069728972	-0.00850289	-0.14058054	0.006209531	0.017108165	11	0	0
HSPA1A	0.1496886	0.122664162	0.027024485	0.725557515	0.006225425	0.017149321	22	0	0
ERCC2	0.1833589	0.186490508	-0.00313165	0.049824265	0.006230704	0.017161227	6	0	0
BTNL2	0.608636	0.681867454	-0.0732315	-0.16081969	0.006234512	0.017169078	6	0	0
GDF6	0.1794962	0.150345838	0.029150399	0.635865876	0.006237017	0.01717334	7	0	0
VSTM4	0.3656354	0.366833255	-0.00119785	0.136207694	0.006239372	0.017177187	7	0	0
TRRAP	0.3565914	0.338619159	0.017972267	0.111319041	0.006242233	0.017182427	6	0	0
KRT73	0.7543028	0.797575146	-0.04327232	-0.07755926	0.006243768	0.017184015	5	0	0
LMNB1	0.063703	0.059448567	0.004254437	0.074172769	0.006247802	0.017192478	11	0	0
SV2A	0.3978923	0.392118227	0.005774053	0.353998111	0.006249033	0.01719323	5	0	0

PHACTR2	0.1893951	0.211638946	-0.02224389	0.034146962	0.006250164	0.017193703	7	0	0
OR4K2	0.5175841	0.546983579	-0.02939946	0.027058135	0.00625274	0.017195987	3	0	0
SORD	0.2076119	0.243156764	-0.0355449	-0.09990016	0.006252912	0.017195987	14	0	0
ZNF33B	0.3131231	0.342846874	-0.02972377	-0.08706667	0.006254918	0.017198868	3	0	0
PRCD	0.3794193	0.36373246	0.015686807	0.058269329	0.006258032	0.017204791	6	0	0
HIST1H4L	0.1936293	0.136299324	0.057329947	0.765083391	0.006263056	0.017214907	11	0	0
CCDC65	0.1222232	0.098975985	0.023247256	0.614142981	0.006264405	0.017214907	5	0	0
TLR1	0.153507	0.186415553	-0.03290854	-0.17160837	0.006273814	0.017227463	4	0	0
TLR6	0.153507	0.186415553	-0.03290854	-0.17160837	0.006273814	0.017227463	4	0	0
SCIN	0.091451	0.092433209	-0.00098221	0.060814307	0.006273121	0.017227463	12	0	0
KRTAP5-6	0.7143234	0.71880921	-0.00448585	0.003154608	0.006273962	0.017227463	3	0	0
SSPN	0.3206218	0.350985059	-0.0303633	0.061828213	0.006270139	0.017227463	6	0	0
RBPMS2	0.196425	0.16156399	0.034861014	0.390418151	0.006275471	0.017228967	7	0	0
MAST2	0.6752382	0.740389388	-0.06515121	-0.1478905	0.0062802	0.017234038	2	0	0
LELP1	0.6669544	0.697419285	-0.03046486	-0.04863296	0.006279452	0.017234038	5	0	0
SLC9A3	0.232448	0.196577852	0.035870178	0.461307179	0.006279177	0.017234038	9	0	0
SOX30	0.1054447	0.059549005	0.04589567	0.821478189	0.006285018	0.017244621	5	0	0
FRMD5	0.0609356	0.068843034	-0.00790745	-0.1016835	0.006291028	0.01725847	11	0	0
NIPSNAP3A	0.1631411	0.158569175	0.004571957	-0.02358033	0.006292767	0.0172606	8	0	0
SUPT20H	0.1308082	0.142518917	-0.01171077	-0.07805479	0.006296737	0.017266209	10	0	0
CHTF18	0.3689132	0.40069147	-0.03177828	-0.03169089	0.006296107	0.017266209	6	0	0
BLZF1	0.2165773	0.201168582	0.015408741	0.012276506	0.006303603	0.017281971	14	0	0
TOP3A	0.0967024	0.105153262	-0.00845083	-0.03646661	0.006304412	0.017281971	12	0	0
PTCRA	0.7349623	0.771090288	-0.03612796	-0.07159252	0.006305688	0.017282828	8	0	0
KRT33B	0.8273124	0.86589987	-0.03858749	-0.06505802	0.006310608	0.017293669	2	0	0
VIT	0.669992	0.718521972	-0.04852993	-0.11460567	0.006317738	0.017303344	5	0	0
HAT1	0.3565418	0.384927136	-0.02838531	-0.03631407	0.006318962	0.017303344	10	0	0
OXSM	0.0664271	0.059467669	0.006959387	0.0687755	0.006318936	0.017303344	5	0	0
TRDMT1	0.650055	0.624682413	0.025372605	0.100468913	0.006315412	0.017303344	8	0	0
PLEKHA5	0.2884866	0.28684409	0.001642518	0.108856024	0.006317247	0.017303344	5	0	0
TMOD3	0.3210649	0.337194809	-0.0161299	0.003663468	0.006330677	0.017332777	11	0	0
GFRA4	0.5689276	0.6019008	-0.0329732	-0.06897332	0.006333318	0.017337361	10	0	0
HENMT1	0.2930299	0.292832949	0.00019695	0.25929197	0.006344217	0.017364547	17	0	0
RASSF5	0.1493019	0.16024054	-0.0109386	0.013845989	0.006347743	0.017371546	13	0	0
DHODH	0.2138855	0.234312512	-0.02042702	0.037754189	0.006351665	0.017379629	8	0	0
MGAT4C	0.5846189	0.63962947	-0.05501056	-0.14181986	0.00635906	0.01739721	4	0	0
POLRMT	0.1018567	0.117356834	-0.01550013	-0.16692842	0.006365802	0.017412998	5	0	0
COG2	0.1944514	0.195800363	-0.00134894	-0.02699931	0.00637706	0.017441133	9	0	0
DENND4C	0.4342222	0.447648947	-0.01342678	0.029835294	0.006382349	0.017450276	4	0	0
ZDHHC13	0.1391764	0.157544968	-0.01836861	-0.13245773	0.006381881	0.017450276	13	0	0
TOX4	0.1468315	0.148936133	-0.00210462	0.083056808	0.00638751	0.017461726	14	0	0
GLB1	0.1169722	0.118567716	-0.00159549	0.02336888	0.006391966	0.01746962	11	0	0
COX19	0.2874366	0.324590111	-0.03715352	-0.1088261	0.006392346	0.01746962	7	0	0
CHST6	0.2529066	0.235840897	0.017065697	0.263361119	0.006393904	0.017471216	9	0	0
SENP5	0.1539695	0.179583785	-0.02561429	-0.06004746	0.006398035	0.017474519	8	0	0
IRX6	0.303799	0.282646334	0.021152671	0.168633412	0.00639648	0.017474519	6	0	0
DCC	0.3483028	0.330796363	0.017506389	0.282833271	0.006397103	0.017474519	13	0	0
SLC29A1	0.18907	0.125934704	0.063135256	1.039320183	0.006414448	0.017516679	12	0	0
TRPS1	0.7988486	0.840477957	-0.04162939	-0.07240423	0.006420709	0.017531106	1	0	0
NUBPL	0.8359908	0.85613747	-0.02014671	-0.03397746	0.006425692	0.017542041	2	0	0
LCE2A	0.4963499	0.559288722	-0.06293878	-0.133809	0.006428058	0.01754369	4	0	0
CHN2	0.7897408	0.830896479	-0.04115566	-0.07239568	0.006428252	0.01754369	1	0	0
ZC3H12A	0.0561469	0.062299957	-0.00615302	-0.03059932	0.006431697	0.017549057	12	0	0
MRPS33	0.1640763	0.172475562	-0.00839925	0.076868009	0.006432175	0.017549057	11	0	0
ZNF34	0.1611409	0.198663382	-0.03752252	-0.11593326	0.006438755	0.017564337	9	0	0
SLU7	0.1831466	0.20823574	-0.02508914	-0.07079399	0.00644068	0.017566915	14	0	0
PLA1A	0.6012635	0.583120019	0.018143433	0.065217194	0.00644322	0.017571171	6	0	0
C1QL3	0.6543049	0.636659705	0.017645233	0.064347435	0.006451835	0.01759199	6	0	0
SENP3	0.1484951	0.164962232	-0.01646711	-0.05993042	0.006462392	0.017618098	7	0	0
CLDN6	0.0972156	0.081911588	0.015303971	0.129065674	0.006465202	0.017623081	7	0	0
HSPA1L	0.0726591	0.02793494	0.044724204	1.15879827	0.006469658	0.017632545	12	0	0
TM6SF2	0.4137771	0.358808269	0.054968881	0.277838026	0.00649509	0.017699169	7	0	0
DNAL1	0.0817357	0.066573498	0.015162231	0.142595481	0.006498481	0.017705719	11	0	0

PACSIN1	0.2188671	0.191459816	0.027407264	0.242417762	0.006500778	0.017709289	14	0	0
PCDHA9	0.3649426	0.421577773	-0.05663519	-0.16570058	0.006502779	0.01771205	5	0	0
GLIPR1L1	0.1320742	0.137305097	-0.00523086	0.354758924	0.00650448	0.017713993	10	0	0
RBBP4	0.0801236	0.074389927	0.005733695	0.088854403	0.006506109	0.017715738	12	0	0
LIPM	0.8875173	0.916239962	-0.02872267	-0.04544626	0.006516445	0.017741188	1	0	0
ZDHHC12	0.1017931	0.112139359	-0.01034622	-0.1122397	0.006529112	0.017772978	9	0	0
MANBAL	0.355893	0.385361301	-0.02946826	-0.08345519	0.006534453	0.017784817	12	0	0
CD69	0.4791484	0.531844894	-0.05269651	-0.17592086	0.006540356	0.017795484	4	0	0
ZFP1	0.266309	0.283059699	-0.01675067	-0.01840549	0.00653971	0.017795484	7	0	0
AURKC	0.7585794	0.728584068	0.029995319	0.069308309	0.006546169	0.017808599	11	0	0
CHAC1	0.2272688	0.253083743	-0.0258149	-0.06399013	0.006547592	0.017809768	13	0	0
FAM167A	0.5014125	0.573082583	-0.07167004	-0.18921269	0.006549013	0.017810932	1	0	0
PCDHB12	0.4571482	0.425953021	0.031195217	0.30220386	0.006551638	0.017815369	8	0	0
TCAP	0.5760772	0.54359678	0.032480398	0.094931935	0.006555914	0.017824294	8	0	0
KAZALD1	0.228447	0.263469474	-0.03502247	-0.11153076	0.006567635	0.017853457	13	0	0
HMG20B	0.0748802	0.07863312	-0.0037529	-0.0480224	0.006574146	0.017868447	8	0	0
CRBN	0.3276385	0.348802248	-0.02116377	-0.0578502	0.006578789	0.017878359	9	0	0
ATAD3B	0.1630533	0.172846017	-0.00979268	0.001384594	0.006580902	0.017881391	2	0	0
SEC13	0.4124583	0.436960652	-0.02450231	-0.04979849	0.006583502	0.017885746	10	0	0
TTPA	0.1871178	0.15036384	0.036753966	0.309729974	0.006585139	0.017886769	10	0	0
NRBF2	0.1842985	0.218043827	-0.03374534	-0.0503357	0.006585873	0.017886769	13	0	0
GNL3	0.8305136	0.840618543	-0.01010495	-0.01620983	0.006588503	0.017888301	4	0	0
ZNF786	0.0385696	0.03260752	0.005962036	0.144843761	0.006589429	0.017888301	4	0	0
LYZL2	0.5271809	0.602527671	-0.07534677	-0.18936702	0.006588531	0.017888301	1	0	0
PIN1	0.1727299	0.190683906	-0.01795404	-0.02288973	0.006597999	0.017908855	12	0	0
CATSPER4	0.5754006	0.577873506	-0.00247289	0.000247527	0.006603046	0.01791442	6	0	0
PCDHA2	0.4018395	0.388659102	0.013180406	0.164748482	0.006602661	0.01791442	9	0	0
NSRP1	0.6446959	0.738039546	-0.09334367	-0.19228964	0.006602224	0.01791442	1	0	0
LARP1B	0.1853826	0.206044532	-0.02066192	-0.01382195	0.006604304	0.017915122	8	0	0
ITGB6	0.7601405	0.719409005	0.040731538	0.089495308	0.006617096	0.017947109	7	0	0
DNAH8	0.4272026	0.4519957	-0.02479315	-0.05241076	0.006627027	0.017971327	5	0	0
MGST1	0.4600253	0.521003717	-0.06097845	-0.14797706	0.006639292	0.018001865	6	0	0
ITGA1	0.058216	0.047492959	0.010723053	0.259696157	0.006642209	0.018004331	11	0	0
PELO	0.058216	0.047492959	0.010723053	0.259696157	0.006642209	0.018004331	11	0	0
HCAR1	0.5399278	0.483291585	0.056636178	0.201345633	0.006644616	0.018008134	11	0	0
ZNF292	0.3998492	0.414298212	-0.014449	0.298650775	0.006646055	0.018009313	5	0	0
GAB1	0.0775565	0.083590584	-0.00603411	0.055469178	0.006650351	0.018016563	9	0	0
TYMS	0.0441718	0.04834111	-0.00416926	-0.07652655	0.006650739	0.018016563	4	0	0
DUPD1	0.8055947	0.838466712	-0.03287204	-0.0594289	0.006651978	0.018017196	6	0	0
GFOD1	0.3357275	0.371515834	-0.03578834	-0.02519303	0.006656156	0.01802307	7	0	0
UCN3	0.6790835	0.64940894	0.029674563	0.074567331	0.006655567	0.01802307	12	0	0
YEATS2	0.2962059	0.314412791	-0.0182069	-0.01799214	0.006658581	0.018026916	9	0	0
ADRB1	0.0813445	0.062363161	0.018981294	0.331110388	0.0066598	0.018027494	7	0	0
SFMBT2	0.1243119	0.090662911	0.03364895	0.430562429	0.006661509	0.0180294	11	0	0
TC2N	0.7049438	0.753950405	-0.04900659	-0.09622504	0.00666302	0.018030768	4	0	0
KLF2	0.273588	0.278778167	-0.00519016	0.134260859	0.006669957	0.018046818	4	0	0
GPR39	0.2115037	0.212134008	-0.00063028	0.178513071	0.006677728	0.018065116	9	0	0
FAT2	0.6949765	0.731769712	-0.03679317	-0.08596104	0.006684117	0.018076948	6	0	0
KCNMA1	0.270399	0.234080618	0.036318368	0.568337713	0.006683638	0.018076948	11	0	0
TMEM170A	0.8764975	0.906973342	-0.03047581	-0.0488677	0.006687853	0.018084325	3	0	0
DNAJC8	0.1471862	0.144765137	0.002421024	0.122278261	0.006698576	0.01811059	9	0	0
RAB36	0.3603472	0.386383155	-0.02603596	-0.09581623	0.006705405	0.018126321	13	0	0
NECAB2	0.1683669	0.137631871	0.030734998	0.597422978	0.006707696	0.018129139	7	0	0
C2CD2	0.162447	0.160372764	0.002074223	0.246072638	0.006708469	0.018129139	9	0	0
TSPEAR	0.1569166	0.121046247	0.035870334	0.386979978	0.006713064	0.018138824	11	0	0
ANK2	0.2694644	0.286578577	-0.01711417	-0.10150524	0.006714594	0.018139069	10	0	0
NDUFB2	0.8221216	0.839952961	-0.01783141	-0.0317668	0.006715177	0.018139069	5	0	0
POMC	0.268355	0.284529483	-0.01617451	0.072548313	0.006720628	0.018151058	14	0	0
PRM3	0.7953363	0.809638766	-0.01430246	-0.02657904	0.006721647	0.018151079	7	0	0
PIK3R3	0.668312	0.705622484	-0.03731047	-0.08849987	0.006724141	0.018154011	3	0	0
NEURL3	0.3018863	0.330004998	-0.0281187	0.036624031	0.006724757	0.018154011	16	0	0
RGS11	0.2184888	0.190204487	0.02828434	0.398731797	0.006727111	0.018157632	9	0	0
DOT1L	0.0351624	0.030673225	0.004489173	0.106486065	0.006735917	0.018178664	6	0	0

EIF3M	0.3030958	0.308943367	-0.00584752	-0.08287683	0.006740828	0.018189181	16	0	0
C16orf46	0.2531214	0.265434183	-0.01231276	-0.02618262	0.006752625	0.018218273	7	0	0
SSRP1	0.1248325	0.137217208	-0.01238471	-0.08446284	0.006755052	0.018222081	10	0	0
RAP1A	0.77088	0.835743439	-0.06486346	-0.12147113	0.006756923	0.018224386	5	0	0
MRPS24	0.1303708	0.146624794	-0.01625398	-0.13896281	0.006762053	0.018235481	9	0	0
C1orf68	0.5921044	0.638001117	-0.04589669	-0.10068553	0.006769041	0.01825158	4	0	0
NFYC	0.1118142	0.1333548	-0.0215406	-0.10804646	0.006784701	0.018291058	14	0	0
CBLL1	0.1475274	0.165074014	-0.01754662	-0.06012402	0.006789052	0.018300035	9	0	0
TRIM4	0.1340366	0.172122741	-0.03808611	-0.16059673	0.006792245	0.018305892	13	0	0
KCNQ5	0.1374263	0.081586325	0.055840017	0.71999991	0.006802607	0.018331064	15	0	0
KLHDC8B	0.283629	0.281548879	0.002080138	0.006751778	0.006806719	0.018336634	8	0	0
YKT6	0.062498	0.06681256	-0.0043146	-0.09112472	0.006806289	0.018336634	6	0	0
CABP7	0.2995381	0.288231816	0.011306262	0.27774403	0.006812339	0.018349019	9	0	0
SOWAHC	0.0924217	0.069595984	0.022825722	0.280447944	0.006813775	0.01835013	16	0	0
NIPSNAP3B	0.279234	0.304637193	-0.02540316	-0.02192489	0.006816985	0.01835602	9	0	0
SPIC	0.7026892	0.754790475	-0.05210123	-0.10402694	0.006819803	0.018360849	3	0	0
PIP5KL1	0.1428589	0.155344004	-0.01248513	-0.12016705	0.006823562	0.018366365	6	0	0
SMCR8	0.0409026	0.043296538	-0.00239395	-0.02298633	0.006823899	0.018366365	13	0	0
TRIM22	0.5295657	0.533818791	-0.00425305	0.050641392	0.00683061	0.01838167	6	0	0
FUBP3	0.142596	0.159065188	-0.01646918	-0.07456733	0.006835899	0.018393141	7	0	0
SMURF1	0.0751336	0.100282686	-0.02514911	-0.08228824	0.006839462	0.018399967	6	0	0
PSMD2	0.3063152	0.333551775	-0.02723659	0.039934529	0.006846649	0.018416542	6	0	0
ZNF30	0.2545191	0.281693974	-0.02717491	0.055085275	0.006849825	0.018422321	8	0	0
ACSS3	0.1786482	0.135549237	0.043098924	0.476405663	0.00685601	0.018436192	14	0	0
NTF4	0.2059581	0.194034215	0.011923907	0.039315244	0.006864779	0.018457006	7	0	0
BRWD1	0.6080554	0.686798231	-0.07874286	-0.17435836	0.006866575	0.018459065	2	0	0
ZNF346	0.0540855	0.055595118	-0.00150967	-0.02732133	0.006888745	0.018515889	11	0	0
PCBP3	0.1172489	0.119413732	-0.0021648	-0.02811475	0.00689075	0.018518503	12	0	0
ANO1	0.2649518	0.244056085	0.020895676	0.387581276	0.00689432	0.018525321	8	0	0
ELAC1	0.4998187	0.530244586	-0.03042585	-0.11142265	0.006905348	0.018549397	8	0	0
SMAD4	0.4998187	0.530244586	-0.03042585	-0.11142265	0.006905348	0.018549397	8	0	0
CDC34	0.2539886	0.250542019	0.003446622	0.146236184	0.00691489	0.018572247	4	0	0
MYRF	0.1998294	0.204464982	-0.00463556	0.157215183	0.0069177	0.018577014	8	0	0
RSPO3	0.1819822	0.180055481	0.001926737	0.257229022	0.006920276	0.018581149	12	0	0
ASB6	0.288734	0.315877944	-0.02714395	-0.03350466	0.006922746	0.018584559	3	0	0
TTC36	0.7382325	0.744195875	-0.00596333	0.004581874	0.006924655	0.018584559	6	0	0
WNK4	0.3031325	0.265982203	0.037150262	0.395500835	0.006924527	0.018584559	10	0	0
WASF2	0.0733391	0.070842164	0.002496961	0.073581456	0.006930307	0.018596946	14	0	0
UBXN11	0.5049659	0.558387014	-0.05342106	-0.13978911	0.006933587	0.018602965	10	0	0
CRYBA1	0.7683512	0.792632707	-0.02428146	-0.0518053	0.006941916	0.018622525	2	0	0
RALGPS1	0.1576988	0.171217992	-0.01351914	-0.0596804	0.006943414	0.018623759	7	0	0
ALCAM	0.0552213	0.061616827	-0.00639554	-0.13224099	0.006946445	0.018629102	14	0	0
MYH7	0.6786007	0.675253685	0.003346966	0.018717966	0.006950944	0.018638382	8	0	0
NR1I2	0.7461171	0.775614536	-0.0294974	-0.06074474	0.006972247	0.018692708	5	0	0
CNTNAP2	0.1948558	0.136695533	0.058160266	0.52186688	0.006974955	0.018697174	14	0	0
DPPA4	0.4558397	0.505919724	-0.05007998	-0.14149094	0.006978701	0.018704419	6	0	0
TAS2R5	0.9035537	0.912359762	-0.00880602	-0.01380955	0.006982468	0.01871172	5	0	0
NOTUM	0.5489677	0.513009062	0.035958666	0.303808219	0.006987514	0.018722444	9	0	0
FAM69A	0.0537399	0.067299966	-0.01356002	-0.15277433	0.006995672	0.018741503	6	0	0
GGA1	0.0340483	0.035834205	-0.00178592	-0.03422842	0.006996849	0.01874186	13	0	0
KCNMB1	0.5260379	0.557910307	-0.03187239	-0.04692917	0.007001386	0.01874901	10	0	0
FRAT1	0.0851742	0.075498615	0.009675634	0.051054824	0.00700161	0.01874901	6	0	0
ALDOB	0.5884075	0.58946801	-0.0010605	0.031053498	0.007003671	0.018751731	5	0	0
LXN	0.3383787	0.28804368	0.050334975	0.638905328	0.007009875	0.018762739	11	0	0
NUCB2	0.0484913	0.047051017	0.001440295	0.022941539	0.007009665	0.018762739	6	0	0
LIMK2	0.1757994	0.151307302	0.024492065	0.079664551	0.007012974	0.018768235	13	0	0
CHERP	0.0391186	0.033281813	0.005836836	0.129047626	0.007028425	0.018806778	8	0	0
CTDSPL2	0.082429	0.08855351	-0.00612454	0.076823416	0.007032572	0.018815066	10	0	0
LATS2	0.1623495	0.166470677	-0.00412116	0.096348113	0.007048891	0.018855916	13	0	0
MZT2B	0.1509537	0.181216289	-0.03026256	-0.24855947	0.007054795	0.018868894	1	0	0
WDFY2	0.2151596	0.233370472	-0.01821089	-0.09965323	0.007059075	0.018877528	10	0	0
FSD1	0.445826	0.430807098	0.015018852	0.087381983	0.007065151	0.018890959	8	0	0
DUSP13	0.8480475	0.86600051	-0.01795298	-0.0272037	0.007075194	0.018914991	7	0	0

CDKN2AIPN	0.1358512	0.129083956	0.006767273	-0.05384909	0.007080065	0.018925192	8	0	0
SOCS7	0.1572607	0.190071459	-0.03281075	-0.09247696	0.007084218	0.01892783	9	0	0
VEZF1	0.157086	0.117483549	0.039602469	0.15031104	0.007083904	0.01892783	8	0	0
ZNF274	0.2724299	0.258536299	0.013893583	0.449670709	0.007083735	0.01892783	5	0	0
AHSG	0.7420574	0.812854502	-0.0707971	-0.13276924	0.007094911	0.018953579	7	0	0
CSPG5	0.7486003	0.778133047	-0.02953272	-0.05498371	0.007099688	0.018963515	8	0	0
DSEL	0.1823038	0.154332287	0.027971484	0.329200433	0.007101491	0.018965506	6	0	0
SACS	0.4809425	0.476922683	0.004019845	0.18663201	0.007107963	0.018979965	8	0	0
SPEN	0.0567803	0.068896529	-0.01211619	-0.0324726	0.007109956	0.018982461	9	0	0
RPL19	0.1555727	0.162773544	-0.00720086	-0.07136744	0.007112831	0.018987311	8	0	0
TICAM1	0.1908587	0.208855383	-0.01799672	-0.03131733	0.007121136	0.019006651	8	0	0
PLIN1	0.7906091	0.746819258	0.043789838	0.086575482	0.007125327	0.019015006	3	0	0
TUBD1	0.0504739	0.044993815	0.005480085	0.077526752	0.0071297	0.019023848	16	0	0
MUC6	0.6451967	0.69404659	-0.04884988	-0.09982522	0.007132906	0.01902957	12	0	0
NDUFS5	0.4530146	0.476308223	-0.02329358	-0.06402467	0.007134678	0.019031468	7	0	0
FAM47E	0.1244961	0.114861314	0.009634814	0.023087567	0.007139537	0.019041597	8	0	0
SBNO2	0.4848124	0.513040482	-0.02822813	-0.0401076	0.007140656	0.019041751	10	0	0
ACKR4	0.5443239	0.474589619	0.069734309	0.193965786	0.007166002	0.019106497	1	0	0
MYT1	0.5867236	0.627577755	-0.04085413	-0.10185553	0.007168426	0.019110119	6	0	0
AP4S1	0.1419002	0.13969489	0.002205339	0.089225827	0.007174464	0.019123375	10	0	0
RIF1	0.1945484	0.214549758	-0.02000131	-0.09250916	0.007176617	0.019126271	6	0	0
GRM1	0.2876348	0.288863182	-0.00122842	0.139849868	0.007196856	0.019177361	8	0	0
LMF1	0.0980631	0.072268537	0.025794526	0.570785808	0.007207695	0.019203389	7	0	0
RPS18	0.0914042	0.096658488	-0.00525431	-0.09548764	0.007212019	0.019209773	22	0	0
PFN1	0.0514361	0.051459655	-2.35E-05	0.059294439	0.007212233	0.019209773	11	0	0
LDHD	0.4485349	0.440219394	0.008315517	0.094993482	0.00721333	0.019209842	11	0	0
ACP5	0.3058538	0.320965432	-0.0151116	0.178351309	0.0072313	0.019254838	11	0	0
CYP20A1	0.3530065	0.394298119	-0.04129158	-0.05048429	0.007237992	0.019269797	12	0	0
LHX3	0.1408937	0.158089428	-0.0171957	-0.15353266	0.007239214	0.01927019	3	0	0
LYZL6	0.6572796	0.68745559	-0.03017596	-0.06891745	0.007245914	0.019285163	6	0	0
CALD1	0.7331763	0.774173733	-0.04099744	-0.08354005	0.00726973	0.019345679	4	0	0
UTP3	0.0695036	0.071180526	-0.0016769	-0.01900431	0.007273452	0.019352712	11	0	0
BTBD1	0.2744162	0.300977973	-0.02656177	-0.04637839	0.007288263	0.019389245	9	0	0
EFCC1	0.248492	0.244315681	0.004176328	0.297319131	0.007293841	0.01939833	7	0	0
CNPY3	0.1100588	0.139252862	-0.02919403	-0.0986057	0.007293225	0.01939833	11	0	0
DCT	0.5923193	0.627950198	-0.03563086	-0.0772	0.007295276	0.01939927	8	0	0
PAQR7	0.5580654	0.552945312	0.005120059	0.005839922	0.007298627	0.019405302	4	0	0
ZNF22	0.4158977	0.411632209	0.004265502	0.162770562	0.007302174	0.01940898	12	0	0
MSRB1	0.131232	0.141098487	-0.00986645	-0.10354284	0.00730216	0.01940898	10	0	0
FOXO1	0.0829878	0.100833668	-0.01784591	-0.08697114	0.007312605	0.019433825	6	0	0
TRAIP	0.1343912	0.144925617	-0.01053441	-0.05861924	0.00731605	0.019440099	13	0	0
HAAO	0.1918278	0.135048584	0.056779179	0.937474569	0.007320025	0.01944778	10	0	0
ATG14	0.2316324	0.275289743	-0.04365737	-0.11671823	0.007322897	0.019452529	11	0	0
SPTBN2	0.7025926	0.730489798	-0.02789716	-0.05851495	0.007336605	0.019486057	5	0	0
SHISA8	0.1522129	0.106157927	0.046054966	0.364559755	0.007338975	0.019489466	6	0	0
LBX1	0.1631595	0.126598172	0.036561278	0.500914616	0.007343782	0.019496458	14	0	0
MPPED1	0.5709068	0.549851668	0.021055126	0.089833207	0.007343235	0.019496458	7	0	0
RAD54L	0.0875072	0.086335326	0.001171862	0.061075288	0.007362073	0.019542125	6	0	0
STAT2	0.2287419	0.243551743	-0.01480987	-0.00012744	0.007363554	0.019543163	13	0	0
MPP3	0.0775109	0.088182412	-0.01067151	0.042669297	0.007376135	0.019573659	6	0	0
ZNF496	0.1934261	0.227863213	-0.03443714	-0.01097208	0.007383129	0.019589319	10	0	0
HDAC1	0.111478	0.115168827	-0.00369083	0.05914885	0.007393183	0.019613094	12	0	0
SH3GLB1	0.0543531	0.065761065	-0.01140793	-0.05184589	0.00740105	0.019628242	14	0	0
C10orf53	0.2908001	0.263920191	0.026879912	0.265367287	0.007401082	0.019628242	9	0	0
IFNGR2	0.3312893	0.363902685	-0.0326134	0.007994151	0.007416358	0.019665849	10	0	0
OR9A4	0.6239675	0.680951445	-0.05698391	-0.12733964	0.007421128	0.019675588	3	0	0
TWIST2	0.3095669	0.244717181	0.064849723	0.388286994	0.00742471	0.019679268	6	0	0
BUD13	0.0460097	0.06414959	-0.01813987	-0.12020874	0.007424581	0.019679268	13	0	0
VTA1	0.3347381	0.345368634	-0.01063058	-0.04670998	0.007430993	0.01969301	11	0	0
VPS33B	0.3495786	0.36069952	-0.01112088	-0.00054375	0.007440575	0.019715491	6	0	0
NBPF3	0.2819124	0.284812071	-0.00289965	0.126121399	0.007446071	0.019727138	5	0	0
CAPN11	0.6990946	0.742098539	-0.04300392	-0.08863083	0.007458124	0.019756154	6	0	0
KLHL30	0.558989	0.584968954	-0.02597996	-0.04397505	0.007467321	0.019777595	9	0	0

OR2C1	0.7072281	0.75869914	-0.05147102	-0.10729653	0.007478815	0.019802188	5	0	0
CCDC43	0.1024676	0.130702175	-0.02823457	-0.11386571	0.007478071	0.019802188	8	0	0
RDM1	0.2040446	0.206424602	-0.00238003	-0.03174525	0.007482009	0.019807721	9	0	0
FAM120AOS	0.0930841	0.10617254	-0.01308845	-0.06278349	0.007483172	0.019807878	8	0	0
ARGFX	0.6193881	0.661285345	-0.04189724	-0.12014358	0.007487342	0.019813067	3	0	0
CD82	0.5295596	0.594202978	-0.06464335	-0.13409051	0.00748642	0.019813067	4	0	0
BIN2	0.6835308	0.736719033	-0.05318819	-0.11988197	0.007499584	0.019842535	12	0	0
HIST1H3A	0.3117541	0.275297502	0.03645663	0.443156651	0.007501801	0.019845473	6	0	0
DCBLD1	0.8465339	0.873277654	-0.0267438	-0.04388532	0.007508585	0.019857561	2	0	0
KCNA1	0.0978733	0.062229007	0.035644304	0.599155087	0.007507787	0.019857561	12	0	0
PPP1R16A	0.3481466	0.383519322	-0.0353727	-0.04433394	0.007516707	0.01987611	6	0	0
KTI12	0.5046058	0.528573117	-0.02396733	-0.02826135	0.007521233	0.019885145	5	0	0
NME6	0.3364185	0.350622481	-0.01420401	-0.03216407	0.007525807	0.019894307	8	0	0
SMG9	0.1083462	0.127903505	-0.01955734	0.011155065	0.007533956	0.019912915	8	0	0
KRT19	0.2188931	0.228446646	-0.00955356	0.15266158	0.007536886	0.019917722	6	0	0
ROR1	0.1276442	0.11568368	0.011960516	0.321443917	0.007538866	0.019920021	10	0	0
NEDD1	0.1122257	0.139405463	-0.02717971	-0.08235799	0.007544895	0.019930323	8	0	0
ZNF541	0.8381948	0.860430353	-0.02223554	-0.03877728	0.007544987	0.019930323	8	0	0
ERH	0.0500532	0.051620314	-0.0015671	-0.02707653	0.007547461	0.019933922	17	0	0
ALPP	0.7077076	0.745206971	-0.03749941	-0.07424529	0.007583193	0.020022399	7	0	0
COL12A1	0.1331258	0.105540352	0.027585462	0.395931939	0.007582682	0.020022399	12	0	0
CTSL	0.293707	0.279786562	0.013920402	0.265243953	0.007584597	0.020023157	8	0	0
P2RX5	0.2836364	0.240912122	0.042724254	0.420964007	0.007587332	0.020027429	9	0	0
ACTC1	0.5633905	0.591551949	-0.02816144	-0.06169257	0.007591741	0.020036119	7	0	0
MS4A10	0.6572763	0.68102056	-0.02374422	-0.05097111	0.007609173	0.020079171	6	0	0
TAC4	0.6874581	0.712420914	-0.02496284	-0.05242682	0.007616172	0.020094685	6	0	0
ST6GALNAC	0.589855	0.601537138	-0.01168213	-0.03789328	0.007620092	0.02010207	9	0	0
NTS	0.4967828	0.545387579	-0.04860482	-0.10277469	0.007628979	0.020122554	4	0	0
SHCBP1	0.4356919	0.452848753	-0.0171569	-0.03356166	0.007632026	0.020127631	8	0	0
KPTN	0.321076	0.34107293	-0.01999693	-0.04404314	0.007634373	0.02013086	10	0	0
POPDC3	0.1664625	0.160734072	0.005728418	0.369609755	0.007638972	0.020140028	11	0	0
SZRD1	0.117193	0.133120986	-0.01592799	-0.04610553	0.007650883	0.020161849	8	0	0
DMBX1	0.7693635	0.795746399	-0.0263829	-0.05238833	0.007650242	0.020161849	6	0	0
SLC29A2	0.1327982	0.148370568	-0.01557237	-0.01740941	0.007651746	0.020161849	13	0	0
UTS2R	0.6593946	0.639767803	0.019626805	0.073848744	0.007650069	0.020161849	6	0	0
SLCO1B3	0.7121798	0.749501144	-0.03732133	-0.07377147	0.00766232	0.020186747	5	0	0
PEX5	0.2334078	0.247932635	-0.01452484	-0.08297717	0.007673303	0.020212712	10	0	0
NFIB	0.6328603	0.616950305	0.015909984	0.078573636	0.007679276	0.020225476	3	0	0
WDR17	0.2938935	0.292862539	0.001030994	0.340878805	0.007682232	0.020230292	10	0	0
TULP2	0.4886138	0.512783997	-0.02417023	-0.02354508	0.007694034	0.020255422	11	0	0
COX4I1	0.3048664	0.351595616	-0.0467292	-0.13860689	0.007699153	0.020265924	3	0	0
KL	0.2141413	0.19579884	0.018342471	0.461215032	0.007703312	0.020273897	10	0	0
SMTNL2	0.3857237	0.395660776	-0.00993711	0.06225694	0.007713166	0.020296853	10	0	0
TRIM6	0.4538574	0.473019972	-0.01916261	0.029309817	0.007715627	0.020300349	4	0	0
SNRNP200	0.170899	0.191903554	-0.02100451	-0.12659614	0.007719121	0.020306564	12	0	0
DCSTAMP	0.7079356	0.746088887	-0.03815324	-0.07929127	0.007731843	0.020337048	5	0	0
02-Sep	0.074012	0.093032033	-0.01902006	-0.12214328	0.007742846	0.020363005	11	0	0
SEC14L4	0.3788216	0.379138864	-0.00031723	-0.06541448	0.007744394	0.020364089	8	0	0
UCHL1	0.3587937	0.390272656	-0.031479	-0.1042997	0.007748593	0.020372144	7	0	0
FAM83F	0.3558064	0.37989617	-0.0240898	-0.01924232	0.007754172	0.020383824	7	0	0
PTPN18	0.2676738	0.268619909	-0.0009461	0.067417065	0.007758745	0.020392128	5	0	0
FAM180A	0.6365483	0.687943404	-0.05139506	-0.12382758	0.007759605	0.020392128	11	0	0
RPS6KC1	0.312852	0.347716867	-0.03486486	-0.08029828	0.007767205	0.02040612	12	0	0
SUCLG2	0.1780951	0.201080013	-0.02298495	-0.03761157	0.007766953	0.02040612	7	0	0
ADAMTSL4	0.104422	0.093866079	0.010555925	0.154620678	0.007778666	0.020430532	12	0	0
NOP58	0.2378068	0.254846636	-0.01703988	0.013178653	0.007778775	0.020430532	10	0	0
HILPDA	0.1421284	0.127454848	0.01467355	0.034306675	0.007801547	0.020487342	14	0	0
CYBRD1	0.1589517	0.137848053	0.021103611	0.391822466	0.007808063	0.02049845	10	0	0
GPS2	0.2760645	0.305900769	-0.0298363	-0.10678014	0.007807682	0.02049845	11	0	0
KRT38	0.7056638	0.75646592	-0.05080211	-0.10038996	0.00780952	0.020499276	5	0	0
LRRN2	0.3379552	0.340283896	-0.00232867	0.251784779	0.007817187	0.020515099	10	0	0
ARL13B	0.2968399	0.350180971	-0.05334111	-0.11499789	0.007822919	0.020515099	7	0	0
BOC	0.2886352	0.249465724	0.03916947	0.186817294	0.007823289	0.020515099	3	0	0

UBE2L6	0.2585748	0.287208571	-0.02863374	-0.071611	0.007824699	0.020515099	11	0	0
MTA1	0.3113735	0.309688143	0.00168531	0.112458991	0.007824347	0.020515099	8	0	0
QPRT	0.4968129	0.54878347	-0.05197053	-0.14975934	0.007821356	0.020515099	11	0	0
GSDMB	0.9044586	0.911493207	-0.00703461	-0.01125797	0.007824542	0.020515099	2	0	0
APC2	0.1293719	0.116968415	0.01240345	0.404650781	0.007823912	0.020515099	7	0	0
ARAP2	0.2182984	0.239917572	-0.02161914	-0.10409975	0.007829763	0.020522378	7	0	0
ODF2	0.877897	0.900091448	-0.02219443	-0.0358393	0.00783379	0.020524536	2	0	0
CUX2	0.1634217	0.128968275	0.034453475	0.560676919	0.007832451	0.020524536	9	0	0
PTGER2	0.2188031	0.191815387	0.026987667	0.440861466	0.007834019	0.020524536	9	0	0
SLC2A7	0.7837796	0.810556389	-0.02677682	-0.05091033	0.007837122	0.020528095	5	0	0
RNF151	0.5457222	0.56067627	-0.01495407	-0.01412138	0.007837667	0.020528095	7	0	0
ENTPD8	0.1096236	0.128085094	-0.01846151	0.041088856	0.007849995	0.020557383	7	0	0
LEAP2	0.8330134	0.855422281	-0.02240887	-0.04011747	0.007857043	0.020572835	5	0	0
ANKRD40	0.1426607	0.159088062	-0.01642733	-0.05319668	0.007864239	0.020588671	10	0	0
ARSI	0.3211479	0.350176893	-0.02902904	-0.1303147	0.00786583	0.020589831	2	0	0
PTPRS	0.1902606	0.21000833	-0.01974771	-0.06656511	0.007870005	0.020597753	7	0	0
MMP23B	0.2884037	0.218913596	0.069490151	0.482997402	0.007873179	0.020603055	10	0	0
RECK	0.4659975	0.49903919	-0.03304173	-0.03300162	0.007876219	0.020608004	4	0	0
NCMAP	0.1598574	0.134138797	0.025718637	0.206798812	0.007880287	0.02061564	11	0	0
HSD17B2	0.5296217	0.561054605	-0.03143289	-0.11700461	0.007885289	0.020625718	8	0	0
GDNF	0.1274058	0.09062446	0.036781354	0.609661538	0.007887053	0.020627322	15	0	0
INTS6	0.1744234	0.19279162	-0.01836826	-0.13466425	0.007890563	0.020633495	5	0	0
TDRD1	0.8281765	0.843424359	-0.01524782	-0.01566097	0.007893736	0.020637759	13	0	0
PHLDB1	0.6218827	0.593095157	0.028787586	0.058615389	0.007894495	0.020637759	8	0	0
ANTXR1	0.1311232	0.148769319	-0.0176461	0.004897284	0.007897176	0.020641759	7	0	0
OR5K2	0.8159753	0.853095093	-0.03711981	-0.06230286	0.007902387	0.020650982	4	0	0
ELFN2	0.2381512	0.246335131	-0.00818396	0.10670441	0.007903007	0.020650982	11	0	0
BOK	0.2860127	0.254929847	0.031082847	0.35026718	0.007905834	0.0206529	9	0	0
ABHD3	0.2068333	0.203610666	0.003222585	-0.01735546	0.007906044	0.0206529	10	0	0
ALDH1B1	0.292151	0.302426821	-0.01027586	-0.07993715	0.007909944	0.020654064	6	0	0
PPP1R1A	0.1526406	0.128058753	0.024581863	0.320317693	0.007909438	0.020654064	11	0	0
LRRN4	0.5270119	0.476492167	0.050519723	0.129220748	0.007908734	0.020654064	5	0	0
NUCB1	0.3391712	0.354529339	-0.01535818	0.01650768	0.007915522	0.02066562	11	0	0
CYP8B1	0.6544061	0.665965619	-0.01155948	-0.02723715	0.00791759	0.020667787	8	0	0
TGM7	0.733708	0.775685142	-0.04197714	-0.08090334	0.007919641	0.020667787	9	0	0
DLX4	0.1292446	0.139919702	-0.01067507	-0.08524587	0.007919809	0.020667787	11	0	0
MCIDAS	0.082309	0.085114159	-0.00280514	-0.06841866	0.007927698	0.020682355	7	0	0
ANGPTL5	0.690253	0.755240484	-0.06498747	-0.13912683	0.007927501	0.020682355	2	0	0
IGFN1	0.5407359	0.585139706	-0.04440384	-0.09505192	0.007931404	0.020689015	5	0	0
PCDHGB2	0.4059553	0.363109712	0.04284562	0.463394642	0.007933679	0.020691938	7	0	0
GPR107	0.0732346	0.084558792	-0.01132417	-0.13023912	0.007935046	0.020692495	8	0	0
SMARCD3	0.4044316	0.380794711	0.023636922	0.056377563	0.007938768	0.020696184	4	0	0
BCAT1	0.4613437	0.476682429	-0.01533872	0.205304157	0.007937632	0.020696184	15	0	0
LRFN4	0.1430138	0.160378303	-0.01736448	-0.10192135	0.007943908	0.020703564	3	0	0
FLRT2	0.1424198	0.097789631	0.04463021	0.609551935	0.007942978	0.020703564	11	0	0
MYH3	0.7519026	0.804009796	-0.05210716	-0.09570373	0.007945783	0.020705443	5	0	0
FOXS1	0.5166499	0.535391025	-0.01874109	-0.04073738	0.007952126	0.020718961	9	0	0
PRKG1	0.248612	0.216291628	0.032320422	0.399031412	0.007953842	0.020720423	11	0	0
HIST1H3H	0.0864736	0.096383578	-0.00990998	0.081617162	0.007973547	0.02076874	11	0	0
ITGA4	0.3037592	0.311242968	-0.00748376	0.428907023	0.007981523	0.020786497	11	0	0
DYNC1I1	0.3099507	0.309589024	0.000361649	0.150194799	0.007990199	0.020806069	8	0	0
TNFSF9	0.370642	0.389349147	-0.01870715	-0.04869273	0.007995583	0.020817068	6	0	0
TAS2R13	0.7852372	0.81142318	-0.02618595	-0.04353652	0.007998404	0.02082139	2	0	0
HYAL3	0.1345276	0.127664439	0.006863125	0.06532011	0.008017701	0.020865566	17	0	0
HOXA13	0.1298136	0.10609924	0.023714326	0.213297346	0.008024556	0.020880376	8	0	0
TSHZ2	0.2851523	0.254116275	0.031036035	0.070584203	0.008027031	0.020883787	5	0	0
PTBP2	0.3230243	0.360214527	-0.03719023	-0.08134322	0.008041374	0.020918068	9	0	0
PALB2	0.2052524	0.240549606	-0.0352972	-0.08588936	0.008046872	0.020929335	11	0	0
TEX10	0.3602624	0.384386526	-0.02412414	-0.03598047	0.008052828	0.020941791	7	0	0
NPAS2	0.0724243	0.048279726	0.024144542	0.475197005	0.008070013	0.020983438	11	0	0
CD58	0.2495584	0.268306883	-0.01874844	0.012388665	0.008076273	0.020996671	9	0	0
ATP6V0A1	0.2720772	0.288012884	-0.01593568	0.029979322	0.008083876	0.021013391	7	0	0
KLF4	0.2211634	0.186095476	0.035067894	0.472056381	0.008089864	0.021023507	4	0	0

STH	0.8105547	0.842807724	-0.03225299	-0.05539959	0.008090112	0.021023507	4	0	0
RNF141	0.1293687	0.136426371	-0.00705767	-0.06979172	0.008091896	0.021025098	8	0	0
PDE12	0.0667558	0.075741813	-0.00898601	-0.06015673	0.008094997	0.021027063	12	0	0
ABI3BP	0.4438927	0.491102392	-0.04720969	-0.09558995	0.008094779	0.021027063	6	0	0
LGALS4	0.8065101	0.796154066	0.010356063	0.025560667	0.008116946	0.021081024	5	0	0
LRRC4B	0.0938968	0.068386702	0.025510116	0.367772047	0.00813929	0.021135994	6	0	0
PDLIM3	0.1800331	0.171975269	0.008057784	0.180196075	0.008141674	0.021136514	12	0	0
EXD1	0.089683	0.090752242	-0.00106921	0.009559461	0.008141847	0.021136514	14	0	0
ZMAT4	0.2586326	0.255509638	0.003122965	0.232708131	0.008156356	0.021171115	11	0	0
B4GALNT3	0.1277765	0.145188925	-0.01741245	-0.10349112	0.00816025	0.021176008	7	0	0
FGF9	0.0440007	0.046152198	-0.00215154	-0.02606221	0.008160602	0.021176008	11	0	0
ABHD4	0.2746849	0.28642596	-0.01174109	-0.05902754	0.008162547	0.021177991	8	0	0
DISP1	0.4025462	0.408039689	-0.00549352	0.0574195	0.008175682	0.021205935	7	0	0
SMCO2	0.8452067	0.866577344	-0.02137068	-0.03694041	0.008174617	0.021205935	5	0	0
SLC35F2	0.1059935	0.076351633	0.029641915	0.45619112	0.008180467	0.021215276	12	0	0
ZFAT	0.1284464	0.138982502	-0.0105361	-0.05938646	0.008186485	0.021227816	11	0	0
MTHFD1	0.1093453	0.121190775	-0.01184547	0.059931761	0.008199349	0.021258099	15	0	0
THUMPD3	0.0441292	0.049418141	-0.00528897	-0.08194374	0.008203797	0.021263484	9	0	0
ZNF816-ZNI	0.0801299	0.05039892	0.029730935	0.579058088	0.008203582	0.021263484	2	0	0
RNASEL	0.3980291	0.417944583	-0.01991547	-0.04524209	0.008207421	0.021266984	9	0	0
TCTA	0.0342004	0.033989616	0.000210752	0.010063321	0.008207519	0.021266984	13	0	0
BARX1	0.2948447	0.254103402	0.040741291	0.309754954	0.008210188	0.021270827	3	0	0
MOGAT1	0.3019303	0.285409514	0.016520803	0.26454696	0.008216948	0.021285267	8	0	0
C20orf173	0.7329811	0.766072925	-0.03309183	-0.0734211	0.008218395	0.02128594	5	0	0
ADI1	0.2877489	0.303193984	-0.01544504	-0.00964721	0.008219862	0.021286666	9	0	0
BCL6	0.0812124	0.106100981	-0.02488861	-0.13928939	0.008222331	0.021289986	12	0	0
TMEM117	0.2873245	0.298858972	-0.01153447	-0.07042602	0.008232568	0.021313415	9	0	0
PLCD1	0.2040509	0.187142521	0.016908417	0.38887948	0.008234215	0.021314602	9	0	0
UBE2G1	0.0905511	0.097517293	-0.00696617	0.041258333	0.008237054	0.021318874	11	0	0
PRKD2	0.1182926	0.129064523	-0.01077196	0.095999451	0.008239402	0.021321876	7	0	0
LIPJ	0.8432364	0.875267328	-0.03203095	-0.05381989	0.008246965	0.021338367	6	0	0
TM2D1	0.4457973	0.458674351	-0.01287702	-0.00743294	0.008251269	0.021346425	8	0	0
VDAC2	0.2558047	0.275324895	-0.01952017	-0.04922583	0.008254019	0.021350459	12	0	0
IL21R	0.225563	0.264978225	-0.03941522	-0.01118936	0.008286347	0.02143099	8	0	0
LCN6	0.491381	0.516480411	-0.02509946	-0.07098178	0.008297501	0.021456745	3	0	0
KRT77	0.7792273	0.770016065	0.009211278	0.027370572	0.008306565	0.021477087	5	0	0
AXL	0.3159797	0.290964327	0.025015422	0.226463299	0.008310355	0.021483788	8	0	0
C1QTNF2	0.3428762	0.325029829	0.017846374	0.16052019	0.008313478	0.021488766	13	0	0
ABCF3	0.3085773	0.349008192	-0.04043091	-0.05519472	0.008326906	0.021520374	9	0	0
RALA	0.1669904	0.198333069	-0.03134265	-0.09004529	0.008332749	0.021529269	10	0	0
PYCARD	0.3549552	0.37661222	-0.02165703	0.019115303	0.008332713	0.021529269	10	0	0
TTF2	0.0675273	0.082950571	-0.01542325	-0.02274556	0.008341768	0.021548857	10	0	0
TM4SF20	0.8361861	0.859569895	-0.02338384	-0.03932708	0.008342733	0.021548857	1	0	0
ZNF572	0.3157762	0.34613626	-0.03036011	0.040024981	0.008347854	0.021556844	13	0	0
INPP5E	0.2651224	0.248150192	0.016972211	0.05064855	0.008348229	0.021556844	10	0	0
TXN2	0.4630104	0.497710435	-0.03470005	-0.01489646	0.008350407	0.021559364	5	0	0
HFE	0.5779762	0.586604665	-0.00862843	0.218046608	0.008354857	0.021567749	6	0	0
APC	0.6051145	0.644974057	-0.03985958	-0.07679851	0.008356624	0.021568537	5	0	0
PIK3IP1	0.1457497	0.12555624	0.020193413	0.20644111	0.008357568	0.021568537	11	0	0
MFGE8	0.230423	0.264849773	-0.03442674	-0.04996519	0.008359491	0.021570396	8	0	0
CCL5	0.609548	0.637111153	-0.02756314	-0.06587378	0.008363684	0.021578111	7	0	0
PRR5L	0.801353	0.829403542	-0.02805056	-0.05248567	0.008369412	0.021589786	5	0	0
ZNF480	0.1439037	0.156511775	-0.01260811	0.000293881	0.008379725	0.021613281	12	0	0
ZC3H8	0.4798804	0.5286092	-0.04872883	-0.08685504	0.008396882	0.021648192	7	0	0
PIK3R1	0.0613451	0.054923301	0.006421753	0.065365817	0.00839505	0.021648192	9	0	0
FTL	0.0780625	0.079611833	-0.00154932	0.167904841	0.008396183	0.021648192	10	0	0
SSBP1	0.1181054	0.145616098	-0.02751072	-0.08266099	0.008398566	0.021649422	18	0	0
DVL3	0.3373383	0.368433702	-0.03109538	-0.03988344	0.00840093	0.021652404	6	0	0
CXCL12	0.4491656	0.461972199	-0.01280659	0.062487051	0.008402265	0.021652733	8	0	0
LAMB1	0.1288768	0.117855441	0.011021349	0.264180309	0.008406707	0.021661069	11	0	0
UBALD2	0.141552	0.179960488	-0.03840852	-0.14885206	0.008408701	0.021663093	10	0	0
CSAD	0.4362192	0.459284735	-0.02306552	-0.08974787	0.008410613	0.021664909	8	0	0
TRIP4	0.0930418	0.102532165	-0.00949039	0.031293798	0.008416014	0.021673456	12	0	0

KCTD19	0.1997124	0.187867462	0.01184496	0.448869014	0.008416348	0.021673456	10	0	0
DNM2	0.0810649	0.086169823	-0.00510489	0.050790522	0.008429014	0.021702957	14	0	0
CA2	0.2244019	0.222037186	0.002364733	0.406956185	0.008432093	0.021706414	8	0	0
SAMD12	0.0501722	0.055846146	-0.00567396	-0.07990223	0.008432777	0.021706414	12	0	0
ZP3	0.2888626	0.318594614	-0.02973197	0.01639317	0.008439995	0.021721875	9	0	0
EME2	0.0665806	0.070353277	-0.00377267	-0.00657296	0.008452194	0.02175015	9	0	0
HMOX1	0.8631156	0.871657747	-0.00854211	-0.01212768	0.008456305	0.021757609	4	0	0
RPL26	0.1546653	0.171831853	-0.01716656	-0.02736922	0.008489463	0.02183979	10	0	0
PALM2-AKA	0.2775563	0.279957353	-0.00240108	0.208071726	0.008506681	0.021877807	6	0	0
AKAP2	0.2775563	0.279957353	-0.00240108	0.208071726	0.008506681	0.021877807	6	0	0
SAP18	0.10144	0.113727373	-0.01228733	0.03023529	0.008515887	0.021898343	11	0	0
DNAJC19	0.2028118	0.22918721	-0.02637537	-0.09078092	0.00851725	0.021898709	12	0	0
FAM84B	0.1240091	0.110014549	0.013994589	0.192417888	0.008524854	0.021911978	8	0	0
ICAM5	0.0925659	0.09908543	-0.00651957	-0.06783888	0.008523705	0.021911978	9	0	0
MMADHC	0.2465917	0.285061733	-0.03847008	-0.09192843	0.008531614	0.02192621	14	0	0
SIX2	0.1427364	0.109877036	0.032859327	0.402871542	0.008539558	0.021943484	13	0	0
ZNF396	0.342029	0.366548885	-0.02451987	-0.05767901	0.008548898	0.021964336	10	0	0
ZNF627	0.047048	0.043568145	0.0034799	0.056880659	0.008550932	0.021966415	7	0	0
ZNF143	0.0461476	0.052165587	-0.00601794	-0.09810825	0.008553834	0.021970724	9	0	0
LDLRAD4	0.1174857	0.143843402	-0.02635772	-0.1131541	0.008555774	0.021972559	5	0	0
CRYGB	0.7798944	0.78281173	-0.00291729	-0.00057129	0.008568439	0.022001937	7	0	0
SPOCD1	0.4208123	0.401615926	0.019196345	0.123079564	0.008586097	0.022044122	9	0	0
TTC13	0.1172959	0.137042621	-0.01974673	-0.08187668	0.008594847	0.022060272	14	0	0
ACOXL	0.4807062	0.485645526	-0.00493937	0.003862169	0.008606105	0.022082847	8	0	0
UGGT2	0.2839443	0.307866108	-0.02392186	-0.02600636	0.008605182	0.022082847	10	0	0
LY6G6C	0.7076907	0.741354143	-0.03366346	-0.07500165	0.008616036	0.022105166	7	0	0
VAPA	0.2118883	0.24168406	-0.02979579	-0.02798961	0.008629069	0.022135438	13	0	0
B4GALNT2	0.3782436	0.370821722	0.007421884	0.2917773	0.008637864	0.022154832	17	0	0
PCDHA8	0.4167094	0.412684326	0.004025069	0.1225334	0.008647474	0.022176309	13	0	0
TRIM52	0.0256291	0.023653566	0.001975503	0.07821488	0.00864916	0.022177459	4	0	0
C4BPA	0.4978697	0.528282496	-0.03041284	-0.12776112	0.008652485	0.022179645	6	0	0
APPL2	0.2196385	0.242339879	-0.02270139	-0.07696202	0.008651359	0.022179645	10	0	0
SLC35B2	0.2260488	0.240791822	-0.01474304	-0.02323773	0.008669417	0.022219872	14	0	0
FBXO43	0.2167421	0.233847363	-0.01710522	-0.05153106	0.008674193	0.022228935	7	0	0
NMNAT1	0.0533881	0.054754073	-0.00136594	0.005025004	0.008683658	0.022250012	15	0	0
PTCHD3	0.6279105	0.646680463	-0.01876993	-0.03036747	0.008686121	0.022253144	9	0	0
TMEM88B	0.532172	0.565920666	-0.03374868	-0.08240712	0.00870466	0.022297455	8	0	0
LY6G5B	0.8069463	0.797940502	0.009005792	0.018006121	0.008711028	0.022310581	12	0	0
SDSL	0.4319454	0.442530042	-0.01058468	0.044690106	0.008724645	0.022342267	8	0	0
TBC1D32	0.2051013	0.253809227	-0.04870797	-0.11531653	0.008729475	0.022345065	6	0	0
NACC2	0.0728603	0.063047082	0.009813219	0.169701613	0.008728508	0.022345065	7	0	0
TRIM72	0.656976	0.697512391	-0.04053638	-0.0815999	0.008727623	0.022345065	4	0	0
STAC3	0.7969332	0.793230639	0.003702542	0.010851363	0.008737012	0.022360225	8	0	0
MMP15	0.1297868	0.11297609	0.016810699	0.158427592	0.008737891	0.022360225	14	0	0
ZNF708	0.3064819	0.30236419	0.004117686	0.204449177	0.008764552	0.022422051	7	0	0
ACBD4	0.1761175	0.193219669	-0.0171022	-0.05848676	0.008770135	0.022433134	10	0	0
PCDHB7	0.4794595	0.465818521	0.013641029	0.129500838	0.008772482	0.02243594	9	0	0
SLC25A3	0.200627	0.236466357	-0.03583934	-0.08395333	0.008775041	0.022439284	12	0	0
TRIM27	0.2838353	0.301959575	-0.0181243	-0.0646148	0.008776665	0.022440237	24	0	0
VMO1	0.22509	0.18046355	0.044626475	0.39902298	0.008783132	0.022453572	11	0	0
MOG	0.6634414	0.632043733	0.031397651	0.085067637	0.008795562	0.022482144	7	0	0
BFAR	0.2982375	0.313275318	-0.01503785	0.011416979	0.008804934	0.022502892	14	0	0
GOLM1	0.4344398	0.466683762	-0.03224395	-0.06534076	0.008813905	0.022522611	3	0	0
RNF157	0.1906597	0.161379605	0.029280102	0.4961972	0.008821753	0.022536243	5	0	0
TMC2	0.6014161	0.579400048	0.022016045	0.058482059	0.008821166	0.022536243	10	0	0
HNMT	0.4113116	0.38354848	0.027763113	0.187086207	0.008825253	0.022541974	7	0	0
UNC45B	0.7053165	0.717146794	-0.0118303	-0.02280522	0.00883659	0.022567717	8	0	0
OR10H1	0.8027376	0.846240396	-0.04350277	-0.07696548	0.008844442	0.022584554	7	0	0
PHF21B	0.0995549	0.074564028	0.02499091	0.613333613	0.00884614	0.022585676	12	0	0
IRF6	0.2249422	0.246458473	-0.02151623	-0.13495123	0.008859593	0.022616552	9	0	0
SLC1A4	0.0948385	0.113468304	-0.01862985	0.070829165	0.008860756	0.022616552	10	0	0
PIGB	0.1690945	0.207593803	-0.03849929	-0.07936982	0.008865268	0.02262485	11	0	0
FSHR	0.4370779	0.505226185	-0.0681483	-0.18637335	0.008869676	0.022632879	5	0	0

PSAP	0.2989548	0.328010376	-0.02905555	-0.02859946	0.008872391	0.022636587	9	0	0
ANKRD27	0.3491148	0.29127807	0.057836752	0.303677229	0.008875519	0.022641347	4	0	0
KRT7	0.5645521	0.543009248	0.021542883	0.103090443	0.008886524	0.022665748	5	0	0
CMTM4	0.0884696	0.099612213	-0.01114259	-0.10426568	0.008887612	0.022665748	8	0	0
SLC30A5	0.2702107	0.273161158	-0.00295048	-0.03867893	0.008895764	0.022683312	11	0	0
PDHB	0.1358651	0.138717713	-0.00285263	-0.00152913	0.008897701	0.022685026	9	0	0
PSMB6	0.3693013	0.38056839	-0.01126707	0.020344688	0.008906051	0.022703089	10	0	0
UBN2	0.5535961	0.575409803	-0.02181375	0.021325865	0.00891325	0.022718211	3	0	0
LMNB2	0.1644559	0.171131293	-0.00667538	-0.07947491	0.008915937	0.022721832	10	0	0
PLEKHA4	0.7047405	0.744899934	-0.04015948	-0.07860093	0.008920289	0.022729691	4	0	0
HSPA5	0.1581709	0.167980726	-0.00980987	-0.02048216	0.008937128	0.022769363	11	0	0
FGF19	0.2002157	0.150444722	0.049770941	0.491429918	0.0089385	0.022769627	10	0	0
CTNNA1	0.4028047	0.403076077	-0.0002714	0.060143805	0.00894141	0.022773805	3	0	0
PES1	0.1896932	0.144550973	0.045142271	0.535082734	0.008956033	0.022807809	12	0	0
PTPRB	0.6502914	0.645212985	0.00507841	0.024003075	0.008959861	0.02281432	6	0	0
HIST3H2BB	0.1343392	0.128949806	0.005389441	0.211252001	0.008964109	0.022821897	12	0	0
GDPD1	0.0625884	0.069894701	-0.00730635	-0.0507885	0.008971173	0.022836638	8	0	0
ENTPD1	0.7033831	0.733830748	-0.03044769	-0.06138882	0.008975755	0.022845061	4	0	0
P2RX1	0.4213643	0.424555894	-0.0031916	0.069134715	0.008985486	0.022866582	9	0	0
DUOXA1	0.2269084	0.20090431	0.026004109	0.432305882	0.009016764	0.022942924	17	0	0
EHHADH	0.0981615	0.120627379	-0.02246587	-0.16408947	0.009031063	0.022976049	10	0	0
ITGB1BP1	0.2063727	0.237026912	-0.03065419	-0.08608759	0.00903237	0.022976115	10	0	0
CPNE4	0.7504977	0.703120436	0.047377311	0.13133203	0.009038499	0.022986709	2	0	0
MAN2B2	0.2079391	0.201403414	0.006535636	0.027678176	0.009039098	0.022986709	7	0	0
IP6K1	0.0676866	0.069008766	-0.00132218	-0.03438972	0.009042233	0.022991423	15	0	0
HTATIP2	0.2353393	0.2558843	-0.02054499	-0.05877539	0.009047879	0.02300111	9	0	0
SEL1L2	0.3045858	0.296929635	0.007656214	0.152432203	0.009048608	0.02300111	5	0	0
MAPRE1	0.2320585	0.278143224	-0.04608468	-0.08398592	0.009054201	0.023012065	9	0	0
PTK2	0.1478521	0.165164898	-0.01731282	-0.17095004	0.009056153	0.023013766	5	0	0
TUBAL3	0.8307125	0.846837075	-0.0161246	-0.02917209	0.009064243	0.023031061	2	0	0
RTN2	0.1619678	0.126965978	0.035001811	0.371830602	0.009069822	0.023041972	10	0	0
AGTRAP	0.2000815	0.17506999	0.025011512	0.140874162	0.009071437	0.023042811	11	0	0
TMEM72	0.6092491	0.644760365	-0.03551124	-0.08067344	0.009077106	0.023050683	6	0	0
SEL1L	0.2220075	0.224159402	-0.00215192	0.039692946	0.009076633	0.023050683	12	0	0
LDHA	0.1399929	0.143144821	-0.00315192	-0.026868	0.009083646	0.023064025	11	0	0
FAM218A	0.3517483	0.375915855	-0.0241676	-0.05331978	0.009088549	0.023073209	9	0	0
SH2D3C	0.4281357	0.433375763	-0.00524009	-0.01152134	0.009095065	0.023083124	6	0	0
GZMM	0.447043	0.46922613	-0.02218316	-0.06882299	0.009095151	0.023083124	3	0	0
GAMT	0.1117185	0.089776513	0.021942035	0.527873879	0.009096316	0.023083124	11	0	0
ISL2	0.1546584	0.13541911	0.019239319	0.303513755	0.009104826	0.023101453	10	0	0
PDE2A	0.3924936	0.414511108	-0.02201747	-0.00267491	0.009110666	0.023112999	5	0	0
CPLX4	0.7455871	0.78550421	-0.03991715	-0.08135407	0.009114824	0.023120277	4	0	0
OTOR	0.6910293	0.738936101	-0.04790678	-0.09386351	0.009123144	0.02313811	6	0	0
ADAMTS2	0.3078462	0.274098767	0.033747436	0.422076253	0.009129982	0.023152178	6	0	0
ZNF805	0.0898484	0.058543042	0.031305394	0.386583024	0.00913841	0.023170274	8	0	0
GON4L	0.0835153	0.090822071	-0.00730678	0.011556598	0.009140358	0.023171938	14	0	0
NEU3	0.6385191	0.665331176	-0.02681209	0.007892225	0.009143276	0.02317291	6	0	0
PFAS	0.0682819	0.087402777	-0.01912091	-0.11690724	0.009143325	0.02317291	4	0	0
BLVRA	0.1269911	0.147468657	-0.02047756	-0.07207759	0.009153255	0.023194799	8	0	0
PDE10A	0.1104741	0.082900346	0.027573761	0.403619866	0.009156663	0.023200156	10	0	0
KIF5C	0.2928155	0.31898991	-0.02617446	0.06928112	0.009158735	0.023200697	12	0	0
STX17	0.1280915	0.136994959	-0.00890348	-0.06039429	0.009160757	0.023200697	8	0	0
ZNF726	0.2675502	0.291553052	-0.02400286	0.00154471	0.009160499	0.023200697	4	0	0
CREB5	0.5224037	0.54401392	-0.02161025	-0.00915246	0.00916254	0.023201937	7	0	0
ADAMTS4	0.5728686	0.533567453	0.039301151	0.127487726	0.009172331	0.023223451	10	0	0
ZNF619	0.3543205	0.385988352	-0.03166782	-0.07230369	0.009175143	0.023227293	5	0	0
NKD2	0.1410244	0.150001505	-0.00897714	0.2496342	0.009181331	0.023239678	9	0	0
RPL17	0.3018477	0.325889753	-0.02404207	-0.05000927	0.009190204	0.023258853	8	0	0
CHKA	0.1251685	0.11978758	0.005380878	0.079343362	0.009192916	0.023262434	11	0	0
GPX7	0.2562362	0.246027294	0.010208868	0.394579175	0.009207384	0.023292474	9	0	0
MDM2	0.0690422	0.076836504	-0.00779426	-0.11329522	0.009206198	0.023292474	6	0	0
ST3GAL6	0.157039	0.146476856	0.010562146	0.219810001	0.009210906	0.023298096	12	0	0
PHACTR4	0.2355605	0.244042883	-0.00848241	0.020555997	0.009219449	0.023316418	12	0	0

NOX5	0.6502768	0.627206664	0.023070105	0.058429932	0.009222263	0.023317169	7	0	0
RNF138	0.498878	0.515695071	-0.01681704	-0.01331307	0.009222346	0.023317169	9	0	0
RABGGTB	0.2724361	0.30023856	-0.02780246	-0.02245923	0.009227498	0.023326907	6	0	0
WWP2	0.2448277	0.245037951	-0.00021025	0.020232533	0.009233841	0.023339651	9	0	0
COCH	0.1569568	0.154063272	0.002893526	0.460428243	0.00923619	0.023342301	11	0	0
MTSS1L	0.2731116	0.264912568	0.008199013	0.278766693	0.009237814	0.023343116	10	0	0
SLC44A1	0.1638431	0.153082706	0.010760388	0.012843673	0.009255343	0.023384115	6	0	0
ARL14EP	0.148163	0.176911003	-0.02874798	-0.12513978	0.009263775	0.023402123	9	0	0
ZFP37	0.2251662	0.210691787	0.014474407	0.420049749	0.009275821	0.023429254	9	0	0
KISS1	0.6965488	0.679725662	0.016823176	0.046429448	0.009283185	0.023444552	5	0	0
DNAJC4	0.2877023	0.27033628	0.017365981	0.120348625	0.009286337	0.02344921	13	0	0
EBF2	0.1594375	0.140249764	0.019187785	0.366136029	0.009291216	0.02345511	12	0	0
LAMP1	0.2664974	0.284014345	-0.0175169	-0.04614724	0.009291289	0.02345511	7	0	0
INHBC	0.722385	0.699302026	0.023082948	0.0580332	0.009298182	0.023469207	6	0	0
CTPS1	0.2779542	0.30207461	-0.02412041	-0.12506773	0.009305133	0.023483448	12	0	0
HNRNPAB	0.2300917	0.259035501	-0.02894375	-0.05710849	0.009310596	0.02349393	8	0	0
ERRFI1	0.2233696	0.195246749	0.028122809	0.225471349	0.009312357	0.023494292	7	0	0
VGLL2	0.1413324	0.102348367	0.038984044	0.483755051	0.00931336	0.023494292	10	0	0
OTOP2	0.1183048	0.087024804	0.031279967	0.444399949	0.009323825	0.023514253	19	0	0
TUBA8	0.2185718	0.244089377	-0.02551756	-0.06007362	0.009323894	0.023514253	12	0	0
CAV1	0.1316589	0.11361193	0.018046992	0.122930405	0.009341891	0.023556327	17	0	0
BTBD11	0.0814747	0.061586121	0.019888601	0.332793506	0.0093589	0.0235959	9	0	0
HDDC2	0.4934409	0.470424426	0.023016468	0.035439911	0.009369379	0.023618999	10	0	0
ADA	0.1528265	0.173029629	-0.0202031	-0.01940895	0.009370843	0.023619371	6	0	0
TGFBR1	0.3654139	0.316888207	0.048525664	0.175658169	0.009372386	0.023619941	2	0	0
BMP1	0.3207925	0.278955976	0.041836498	0.22396092	0.009380968	0.023638247	3	0	0
TCF7L2	0.0930653	0.085804861	0.007260423	0.142957235	0.009388815	0.023654696	13	0	0
ZNF688	0.2971677	0.317875341	-0.02070763	-0.08349469	0.00939081	0.023656399	9	0	0
CHAMP1	0.2821774	0.317344384	-0.03516702	-0.06308342	0.00939913	0.023674033	8	0	0
PSORS1C1	0.7735763	0.785242173	-0.01166588	-0.01902194	0.009408551	0.023694436	2	0	0
DCHS1	0.4257623	0.450168018	-0.02440573	0.0556812	0.00941543	0.023708431	6	0	0
DDX59	0.286029	0.304424143	-0.01839518	0.008273914	0.009417192	0.023709539	9	0	0
INO80C	0.2975987	0.308075515	-0.01047679	-0.04490875	0.00941906	0.023710914	11	0	0
PSTK	0.1629804	0.176572011	-0.01359161	-0.10022919	0.009423057	0.023717647	11	0	0
PCSK9	0.2160735	0.183065616	0.033007846	0.439417562	0.009424783	0.023718663	11	0	0
OPRK1	0.1585849	0.120242106	0.038342747	0.422507792	0.009427304	0.023721678	10	0	0
C9orf43	0.2186349	0.256990225	-0.03835534	-0.09996654	0.00943358	0.023734142	5	0	0
ITGAL	0.4753101	0.52930524	-0.05399511	-0.16604436	0.009450121	0.023772422	6	0	0
SIRT6	0.2055527	0.234516278	-0.02896362	-0.02789362	0.009458135	0.023789246	11	0	0
HPCA	0.1982971	0.201238442	-0.00294136	0.265731665	0.009461436	0.023794212	11	0	0
AKR7A3	0.2996696	0.280375907	0.019293684	0.167455828	0.00947109	0.02381515	6	0	0
RBP4	0.2628653	0.219624771	0.043240513	0.342846157	0.009473091	0.023816842	10	0	0
FGF23	0.427918	0.468023054	-0.04010502	-0.05383111	0.00947541	0.023819336	9	0	0
SYT13	0.2193205	0.216396681	0.002923846	0.307107388	0.009480146	0.023824562	9	0	0
NKX2-2	0.1791941	0.138240653	0.04095347	0.360370311	0.009479835	0.023824562	10	0	0
NEB	0.8319598	0.8438504	-0.01189056	-0.02014883	0.009488086	0.023837835	5	0	0
TBCD	0.1999451	0.151179775	0.048765343	0.462272624	0.009487993	0.023837835	5	0	0
EMC4	0.1328346	0.156163372	-0.02332878	0.076741072	0.009494625	0.023850923	8	0	0
MAP3K13	0.1666101	0.189246868	-0.02263673	-0.15282886	0.009506304	0.023876917	11	0	0
HIST1H3I	0.1362079	0.080999802	0.055208086	0.801264932	0.009510965	0.02388528	16	0	0
FLII	0.1574898	0.172925569	-0.0154358	-0.05753264	0.009513379	0.023887997	19	0	0
C9orf50	0.610185	0.637300673	-0.02711565	-0.04382419	0.009515995	0.023891221	7	0	0
TADA2B	0.7604149	0.78195668	-0.02154181	-0.02500608	0.009518433	0.023893997	7	0	0
KLK8	0.6608331	0.721371406	-0.06053827	-0.12773159	0.00952208	0.023899808	4	0	0
ADCK1	0.3766025	0.380022452	-0.00341995	-0.00787799	0.009540254	0.023942074	8	0	0
TOMM5	0.2269727	0.256028418	-0.02905574	-0.12601112	0.009545843	0.023946048	9	0	0
SLC38A7	0.2949236	0.291624934	0.003298709	-0.01155288	0.009545527	0.023946048	10	0	0
TFF2	0.6123747	0.625470039	-0.01309532	-0.02038654	0.0095432	0.023946048	7	0	0
FBXL6	0.5553169	0.583966426	-0.02864956	-0.06081217	0.009552881	0.023960351	3	0	0
ARHGAP19	0.2449993	0.246133239	-0.00113397	0.050314386	0.009571642	0.024004049	11	0	0
ANKS3	0.0962813	0.108697306	-0.01241596	-0.03851681	0.009576179	0.024012072	11	0	0
RHOT2	0.2682536	0.28062818	-0.01237461	0.040591249	0.009580245	0.024018908	17	0	0
INPP5D	0.5435771	0.544870368	-0.00129324	0.024015735	0.009583115	0.024022747	12	0	0

ORAI3	0.264213	0.238977772	0.025235248	0.060435872	0.009585829	0.024026193	15	0	0
PI15	0.5671389	0.546869852	0.02026907	0.086603349	0.009591079	0.024032486	6	0	0
RHPN1	0.2027592	0.222697778	-0.01993853	-0.00773159	0.009592967	0.024032486	9	0	0
OAT	0.3402486	0.349860278	-0.00961171	-0.02697366	0.0095937	0.024032486	7	0	0
TP53	0.0327421	0.031735185	0.001006939	0.038189813	0.009593172	0.024032486	14	0	0
PCDH18	0.1737243	0.156723663	0.017000636	0.170325669	0.00959756	0.024038799	9	0	0
MN1	0.0962968	0.073960753	0.02233609	0.276006994	0.009604708	0.024053342	6	0	0
KCTD2	0.2994777	0.330382325	-0.03090463	-0.01380841	0.009619606	0.024087289	3	0	0
CR1	0.387687	0.373114281	0.014572692	0.416701155	0.009631789	0.024104439	3	0	0
LRTOMT	0.0861994	0.092077898	-0.0058785	-0.09264179	0.009630716	0.024104439	14	0	0
LEO1	0.2477684	0.259307007	-0.01153857	0.022885325	0.009628795	0.024104439	8	0	0
MYH2	0.5933177	0.629347165	-0.03602947	-0.09585393	0.009631831	0.024104439	7	0	0
RAB25	0.5292771	0.603954842	-0.07467771	-0.17967046	0.009640179	0.024121966	7	0	0
CD160	0.6542289	0.697828826	-0.04359995	-0.0935198	0.009643023	0.024122352	6	0	0
COL13A1	0.1095218	0.115345058	-0.00582327	0.00114964	0.009645674	0.024125619	16	0	0
MSMB	0.8213158	0.853979601	-0.03266381	-0.05613661	0.009649465	0.024131735	5	0	0
LRGUK	0.168647	0.167546212	0.001100782	0.265047538	0.009657937	0.024149555	12	0	0
GBP7	0.756741	0.780320298	-0.02357932	-0.04716227	0.009671596	0.024180338	3	0	0
GATA6	0.0650402	0.054566627	0.010473546	0.233866942	0.009697825	0.024239158	7	0	0
BBS4	0.1158212	0.137067431	-0.02124621	-0.0168655	0.009710991	0.024268682	15	0	0
NBL1	0.4814468	0.513013208	-0.03156642	-0.05771882	0.009724591	0.024299022	3	0	0
CDH19	0.7081678	0.76307164	-0.0549038	-0.11219202	0.009725841	0.024299022	8	0	0
CBL	0.2004086	0.210597969	-0.01018933	0.067480626	0.009730255	0.024300581	9	0	0
DERA	0.3475275	0.369539391	-0.02201186	-0.03094348	0.009730232	0.024300581	9	0	0
NDUFB10	0.2351531	0.225203867	0.009949211	0.029746265	0.009730529	0.024300581	10	0	0
ZNF425	0.0419085	0.03873679	0.003171702	0.10161014	0.009732359	0.024301768	7	0	0
KRTAP29-1	0.7209485	0.734930955	-0.01398244	-0.04330809	0.009734358	0.024303376	5	0	0
RAB15	0.1733037	0.196092627	-0.0227889	-0.04107136	0.009748595	0.024335532	6	0	0
IL12RB2	0.262455	0.268922927	-0.00646789	0.238823591	0.009755549	0.024349503	8	0	0
ZNF778	0.0440201	0.042051684	0.001968388	0.063309206	0.009774946	0.024394523	13	0	0
CXCL11	0.7912957	0.820352063	-0.0290564	-0.05138849	0.009776328	0.024394578	1	0	0
NFATC2IP	0.0505139	0.04684037	0.003673489	0.09326501	0.009785958	0.024415209	9	0	0
ZNF19	0.0628168	0.068968392	-0.00615157	-0.10283716	0.00978765	0.024416036	2	0	0
OR2B2	0.7018499	0.741151248	-0.03930132	-0.08098584	0.009802639	0.024450025	6	0	0
UBE3C	0.239217	0.26238196	-0.02316495	-0.0270204	0.009804308	0.024450787	7	0	0
C19orf84	0.5947227	0.616862547	-0.02213981	-0.03592023	0.009817445	0.024480146	6	0	0
RPL9	0.0395725	0.047352366	-0.00777988	-0.06009239	0.009822854	0.024490228	12	0	0
C1orf87	0.3962846	0.369188831	0.02709573	0.232165364	0.009826251	0.024495294	12	0	0
TAF15	0.0683374	0.065433534	0.002903846	0.023841777	0.009829895	0.024500971	10	0	0
RNF217	0.1167154	0.079918965	0.03679643	0.435367448	0.009833948	0.024504263	8	0	0
ZNF75A	0.2503631	0.227652818	0.022710274	0.640720148	0.009832816	0.024504263	13	0	0
SLC38A3	0.2259697	0.223562564	0.002407152	0.276541329	0.009849235	0.024535446	8	0	0
ELOVL2	0.3489999	0.355687332	-0.00668741	0.405790825	0.009850566	0.024535446	7	0	0
ARMC1	0.0770468	0.077919674	-0.00087285	0.074494087	0.009850518	0.024535446	9	0	0
TMEM92	0.3747418	0.344274623	0.030467143	0.120744201	0.009853858	0.02454024	2	0	0
IL17REL	0.5765677	0.57970079	-0.00313305	0.009999962	0.009859571	0.024551056	5	0	0
C3orf67	0.1454593	0.154710402	-0.0092511	0.282489888	0.009867923	0.024565034	12	0	0
ANKDD1B	0.355641	0.367722691	-0.01208174	0.000132652	0.00986672	0.024565034	7	0	0
PHLDA3	0.1911632	0.184600499	0.006562673	0.175644346	0.009892383	0.024615675	11	0	0
PLXDC2	0.1051665	0.084889386	0.02027709	0.296749312	0.009892236	0.024615675	13	0	0
TTLL1	0.1789823	0.206324345	-0.027342	0.022304705	0.009889956	0.024615675	15	0	0
PLA2G4F	0.7261604	0.759381215	-0.03322086	-0.08263107	0.009899931	0.024631041	6	0	0
SHISA7	0.0878752	0.058489377	0.029385854	0.537923522	0.009904562	0.024639144	10	0	0
ARHGEF10L	0.2323069	0.200992462	0.031314418	0.245685342	0.009906532	0.024640627	7	0	0
TOR1AIP2	0.1110535	0.12612525	-0.01507175	-0.02856314	0.009910878	0.024644601	13	0	0
UBAC1	0.1816171	0.213693425	-0.0320763	-0.06382684	0.009910089	0.024644601	4	0	0
KCNC1	0.2829592	0.272332064	0.010627122	0.034748823	0.009928169	0.024684174	3	0	0
UTP6	0.0911708	0.092220252	-0.00104941	0.038589335	0.009944329	0.024720927	15	0	0
TMEM174	0.765659	0.803870891	-0.03821189	-0.07122296	0.009956813	0.024745102	8	0	0
ZSWIM4	0.1283352	0.126657275	0.001677907	0.058710807	0.009955967	0.024745102	7	0	0
NLRP7	0.6788858	0.721520202	-0.04263443	-0.07771834	0.009959413	0.024748135	3	0	0
ZNF254	0.3453473	0.35612959	-0.01078226	0.172902523	0.009970529	0.024772324	8	0	0
ASNA1	0.2389442	0.248612489	-0.00966831	0.232376942	0.009973242	0.024775632	14	0	0

SH3D21	0.4458388	0.468937091	-0.02309826	-0.00581125	0.009982501	0.0247952	10	0	0
PUS3	0.1043574	0.070772497	0.033584862	0.26003463	0.009985673	0.024799644	10	0	0
NHLH1	0.6547872	0.693425213	-0.03863804	-0.0855052	0.009995225	0.02481993	8	0	0
LCE6A	0.6487519	0.710609531	-0.06185766	-0.13029444	0.009997477	0.024822085	5	0	0
ANKRD13D	0.1800345	0.205343377	-0.02530884	-0.08830052	0.00999958	0.024823871	6	0	0
CLK3	0.1940408	0.170346632	0.023694141	0.598624743	0.010001292	0.024824685	6	0	0
EFHB	0.0483455	0.05362674	-0.00528123	-0.06895011	0.010008641	0.024839488	5	0	0
SMUG1	0.1346083	0.14579319	-0.01118489	-0.00312508	0.010017979	0.024859225	14	0	0
DMAP1	0.2283218	0.252126628	-0.0238048	-0.08056006	0.010021176	0.024863717	12	0	0
CHD4	0.1279893	0.126950895	0.001038435	0.071399998	0.010029375	0.024880618	7	0	0
VCL	0.2343391	0.245213885	-0.01087481	-0.00278882	0.010050163	0.02492874	8	0	0
GALK1	0.2755635	0.255080445	0.020483096	0.09628885	0.010053618	0.024933862	8	0	0
PPP1R26	0.3616356	0.337134279	0.024501271	0.070459374	0.010056521	0.024937613	5	0	0
SPACA4	0.717674	0.719936093	-0.00226211	0.002440787	0.010059559	0.024941699	10	0	0
SLC5A5	0.3416684	0.319652463	0.022015954	0.342969208	0.010067812	0.024958712	12	0	0
PLIN2	0.758493	0.796982718	-0.03848976	-0.07050574	0.010082109	0.024990701	1	0	0
TMEM143	0.145633	0.162701159	-0.01706814	-0.07397929	0.0100876	0.025000857	9	0	0
GPR150	0.3462096	0.327096946	0.019112649	0.309122231	0.010122906	0.025081426	6	0	0
PAQR5	0.2221763	0.198554932	0.023621381	0.278956857	0.010127664	0.025089748	9	0	0
GLI4	0.2795624	0.285520652	-0.00595825	0.018535189	0.010130923	0.025094356	5	0	0
JADE1	0.204759	0.204057358	0.000701686	0.032165976	0.010138194	0.025105431	11	0	0
SH3GLB2	0.1191246	0.115128832	0.003995771	0.056050976	0.010137808	0.025105431	8	0	0
EFCAB12	0.3161078	0.33070896	-0.01460115	0.032143317	0.010142538	0.025105791	7	0	0
OGDHL	0.233075	0.255092632	-0.02201768	0.003020129	0.010140532	0.025105791	16	0	0
RPL28	0.0554278	0.070586694	-0.01515888	-0.07417785	0.010141194	0.025105791	11	0	0
RASGRF1	0.1045133	0.067558837	0.036954458	0.621765106	0.010145238	0.025109008	22	0	0
IL26	0.6494145	0.685336787	-0.03592232	-0.06420408	0.01015504	0.025129802	4	0	0
TMEM171	0.2257733	0.194621196	0.031152108	0.38000593	0.010158162	0.02513406	6	0	0
RTP4	0.3769005	0.383867678	-0.00696716	0.077193912	0.010160723	0.025136928	3	0	0
FPGT	0.2111508	0.230387206	-0.0192364	-0.06911428	0.010169783	0.025142003	11	0	0
FPGT-TNNI3	0.2111508	0.230387206	-0.0192364	-0.06911428	0.010169783	0.025142003	11	0	0
LHX9	0.0839918	0.058691509	0.025300271	0.438126629	0.010168156	0.025142003	12	0	0
CTDSP1	0.0396969	0.039340022	0.000356883	0.043459591	0.010169349	0.025142003	9	0	0
MGA	0.2455203	0.26299136	-0.01747107	0.023904144	0.010169271	0.025142003	8	0	0
HS3ST3A1	0.0987232	0.072707128	0.026016033	0.459829639	0.010173036	0.025146578	5	0	0
RNF185	0.2158616	0.247456228	-0.03159464	-0.05381587	0.010175518	0.025149247	7	0	0
FGA	0.591976	0.63647176	-0.04449579	-0.10779573	0.010178636	0.025150024	4	0	0
NR2C1	0.3128829	0.335819233	-0.0229363	-0.06302827	0.010178626	0.025150024	10	0	0
ATF7	0.1068523	0.113713272	-0.00686096	0.031815128	0.010180817	0.025151946	15	0	0
PTGES2	0.3994461	0.429751165	-0.03030511	-0.08851464	0.010185082	0.025159017	5	0	0
ZNF550	0.1961737	0.175156355	0.021017321	0.255633722	0.010187648	0.025161892	12	0	0
IFITM5	0.448639	0.432472514	0.016166519	0.098268898	0.01019147	0.0251644	6	0	0
TIMM13	0.3968314	0.376391786	0.0204396	0.092428696	0.010190834	0.0251644	11	0	0
MCMDC2	0.2751518	0.296232729	-0.02108097	0.187469325	0.010197805	0.025176576	9	0	0
RAB11FIP1	0.1721572	0.155576853	0.016580316	0.002217978	0.010203699	0.025184195	11	0	0
BCL2L13	0.1367736	0.154668543	-0.01789497	0.011014914	0.010203421	0.025184195	7	0	0
HIF1AN	0.2541404	0.29088395	-0.03674351	-0.05791919	0.010208351	0.02519221	3	0	0
GRIK5	0.4981166	0.468186466	0.029930133	0.155601935	0.010212774	0.025199658	2	0	0
ARFGAP2	0.1765665	0.164632455	0.011934076	0.107900301	0.010218697	0.025210804	16	0	0
ARRDC5	0.7560306	0.779268199	-0.02323757	-0.04923502	0.010223338	0.025218784	8	0	0
UHMK1	0.5345743	0.549665907	-0.01509159	-0.03907811	0.010235048	0.0252442	10	0	0
OXCT1	0.33904	0.36409895	-0.02505891	0.042723551	0.010250556	0.025278974	12	0	0
ULK2	0.5595797	0.541531555	0.018048145	0.107973505	0.010261654	0.025302864	8	0	0
KIAA0754	0.1493116	0.11805219	0.031259457	0.097126876	0.010283674	0.025350188	15	0	0
PLEKHO1	0.4191678	0.45535269	-0.03618485	-0.0455337	0.010283094	0.025350188	9	0	0
HAMP	0.8320241	0.851088745	-0.01906469	-0.03039343	0.010297771	0.025381451	3	0	0
TOR1B	0.2657334	0.270129134	-0.00439573	0.064616393	0.010304283	0.025394012	7	0	0
IER5L	0.239109	0.254889391	-0.01578041	0.045716129	0.010309168	0.025402561	5	0	0
AGO4	0.2325812	0.234733297	-0.00215206	0.083593502	0.010313593	0.025406484	6	0	0
ST3GAL2	0.2506097	0.260177158	-0.0095675	0.078074256	0.010312262	0.025406484	6	0	0
DCAF16	0.0824948	0.099227724	-0.01673291	-0.08714061	0.010319846	0.025418395	9	0	0
SMOC1	0.6871911	0.7345012	-0.04731011	-0.10146534	0.010322931	0.025422503	6	0	0
SYT8	0.6035683	0.612103503	-0.00853522	0.001644715	0.010324773	0.025423549	9	0	0

CCNE2	0.1024841	0.107097693	-0.00461363	-0.0892894	0.010333992	0.025442755	5	0	0
ACTR8	0.3843486	0.381962946	0.002385616	0.033530617	0.010336364	0.025445104	9	0	0
SAE1	0.0473446	0.041539063	0.005805585	0.128877912	0.010340377	0.025451489	3	0	0
RNF130	0.3572756	0.351385918	0.005889717	0.145526594	0.010347041	0.025464398	3	0	0
CGB2	0.6120718	0.685307372	-0.07323555	-0.16056974	0.010352489	0.02547431	1	0	0
GRID2	0.1672634	0.147893163	0.019370253	0.316423056	0.010354659	0.025476153	13	0	0
CGREF1	0.4506475	0.437933704	0.012713842	0.325510657	0.010356937	0.025478264	10	0	0
PSMC2	0.0504836	0.053833038	-0.00334942	-0.05054118	0.010367844	0.025501598	4	0	0
PXN	0.1968513	0.19544988	0.001401427	0.062376073	0.010382556	0.025534284	10	0	0
KBTBD11	0.0631727	0.044305948	0.018866738	0.441033396	0.010390339	0.025535916	2	0	0
AMN1	0.1983109	0.214607503	-0.01629664	0.079347892	0.010386041	0.025535916	14	0	0
CCNB1IP1	0.2658867	0.282705166	-0.01681845	-0.02603257	0.010388887	0.025535916	10	0	0
TOMM34	0.2625574	0.248700773	0.013856644	0.028820958	0.010389793	0.025535916	11	0	0
TIMP3	0.3421029	0.345631281	-0.00352835	0.161101048	0.010387494	0.025535916	9	0	0
ATL2	0.2440643	0.256148552	-0.01208429	-0.02047681	0.010396988	0.025545256	4	0	0
MRFAP1L1	0.0646717	0.090868093	-0.0261964	-0.08162398	0.010395622	0.025545256	10	0	0
SRSF12	0.4187595	0.423844655	-0.00508511	0.03948693	0.010400635	0.025547219	7	0	0
ACOT4	0.307853	0.315606762	-0.00775371	0.012576169	0.010399533	0.025547219	7	0	0
LRRTM2	0.6278581	0.58641775	0.041440321	0.101412263	0.010411497	0.025570398	13	0	0
SGCE	0.3838885	0.407551401	-0.02366292	0.024797694	0.010418104	0.02558312	57	0	0
FAM186A	0.7745495	0.804480814	-0.02993136	-0.06231496	0.010422628	0.025590727	2	0	0
TRIM33	0.1133283	0.13301565	-0.01968732	-0.09336559	0.010429808	0.025604849	6	0	0
MAP1B	0.2510855	0.236717876	0.014367651	0.270542227	0.010431397	0.025605246	9	0	0
OTOF	0.4812039	0.475616426	0.005587496	0.085230891	0.010448383	0.025639923	7	0	0
SMPDL3A	0.2019445	0.23090944	-0.02896496	-0.12203869	0.010447386	0.025639923	12	0	0
RBM38	0.1662484	0.180601318	-0.0143529	-0.07416335	0.010450904	0.025642602	10	0	0
TTYH3	0.1410961	0.15831859	-0.01722251	-0.04322892	0.010461538	0.025665183	8	0	0
ANKRD39	0.2749502	0.264993286	0.009956945	0.107221529	0.010486505	0.025722914	11	0	0
RPS3	0.1863672	0.195565724	-0.00919853	-0.00119787	0.010487983	0.025723022	9	0	0
PALD1	0.2242875	0.223113783	0.001173712	0.172554378	0.010491672	0.025728553	6	0	0
OR6B3	0.6648373	0.711654764	-0.04681745	-0.10259598	0.010495432	0.025734254	3	0	0
RET	0.1928648	0.199269513	-0.00640475	0.179642275	0.010497641	0.025736153	11	0	0
HELLS	0.0929822	0.106043161	-0.01306101	-0.00363036	0.010501015	0.025737389	13	0	0
HEXIM2	0.1642029	0.160255241	0.00394766	0.058977177	0.010499757	0.025737389	8	0	0
DTX3	0.3189634	0.317232005	0.00173141	0.043220741	0.010506194	0.025746562	11	0	0
IL13	0.2203843	0.181481924	0.03890242	0.551386949	0.01051574	0.025766435	9	0	0
PSEN2	0.2932185	0.32675252	-0.033534	-0.04837348	0.010530276	0.02579853	5	0	0
TPRG1	0.4067862	0.441144964	-0.03435879	-0.06925884	0.010531771	0.025798668	12	0	0
PYCR1	0.5639892	0.539014616	0.024974629	0.081603669	0.010542448	0.025821295	5	0	0
TNFSF14	0.5310458	0.571082166	-0.04003641	-0.09455131	0.010550963	0.025838624	4	0	0
TNIK	0.0411213	0.042610103	-0.00148885	-0.02810376	0.010561179	0.025860111	6	0	0
PCGF3	0.1606164	0.15608867	0.004527768	0.089202527	0.010563435	0.025862104	10	0	0
PPP1CB	0.0669604	0.083360053	-0.01639968	-0.1182143	0.010565507	0.025863646	7	0	0
UGT2A3	0.5536292	0.4934918	0.06013743	0.16277815	0.010574606	0.025882388	1	0	0
PLD6	0.2845559	0.307471177	-0.02291529	-0.14103225	0.010581224	0.025895051	11	0	0
ASPA	0.8792108	0.899313771	-0.02010297	-0.03058866	0.010588623	0.025909095	8	0	0
SERINC3	0.0574691	0.062619849	-0.00515075	-0.05518517	0.010589851	0.025909095	8	0	0
TMPRSS12	0.6574415	0.616571727	0.040869741	0.102529598	0.010592966	0.025913181	8	0	0
IRAK2	0.1863139	0.199623784	-0.01330988	0.133217394	0.010600959	0.025925662	13	0	0
IFIT2	0.3161648	0.304249795	0.011915045	0.221765883	0.010618983	0.0259662	10	0	0
SPATA24	0.1961649	0.186943628	0.009221264	0.034546521	0.010624683	0.025973056	11	0	0
FGD3	0.2441075	0.2441077	-2.05E-07	0.159691276	0.010624321	0.025973056	5	0	0
SERPINA12	0.538225	0.5868508	-0.04862578	-0.10131963	0.010628822	0.025979634	7	0	0
TET3	0.1653483	0.180147503	-0.01479918	-0.00092694	0.010644068	0.02600981	6	0	0
NDUFA12	0.2577486	0.295810271	-0.03806166	-0.0618784	0.010642716	0.02600981	6	0	0
VGLL4	0.3771872	0.394287839	-0.01710066	0.009849419	0.010651071	0.026023377	7	0	0
RPS26	0.3217157	0.359268326	-0.03755264	-0.0580326	0.010664823	0.026042787	10	0	0
LARP6	0.0867081	0.087846591	-0.00113846	0.193086401	0.010661074	0.026042787	8	0	0
C16orf70	0.2713957	0.261585081	0.00981061	0.119034458	0.010662016	0.026042787	12	0	0
YES1	0.1054274	0.120356999	-0.01492958	-0.00892296	0.010663464	0.026042787	6	0	0
C2orf66	0.7270271	0.760693104	-0.03366602	-0.06765449	0.010669457	0.026050555	7	0	0
PPP4R4	0.7063678	0.764156518	-0.05778872	-0.11192478	0.010677289	0.026060481	1	0	0
MYO9A	0.2099741	0.250588044	-0.04061391	-0.10637457	0.010676788	0.026060481	7	0	0

TFF1	0.7149138	0.741849946	-0.0269361	-0.04949139	0.010677881	0.026060481	10	0	0
LRRC34	0.2272579	0.219805272	0.007452635	0.217922266	0.010681315	0.026063218	13	0	0
AFAP1L1	0.2478519	0.236170524	0.011681365	0.224823859	0.010681909	0.026063218	9	0	0
PRDM12	0.1260128	0.087666409	0.038346385	0.48148336	0.010687676	0.026073742	5	0	0
FASTK	0.3271257	0.354333768	-0.02720809	-0.03394328	0.010690086	0.026076074	21	0	0
USH2A	0.6340835	0.6624265	-0.02834305	-0.05239523	0.010691739	0.026076562	4	0	0
GFRA3	0.2305343	0.214913918	0.015620348	0.306558803	0.010704033	0.026099448	13	0	0
DNAH10	0.1242527	0.126153306	-0.00190056	0.061789884	0.010703626	0.026099448	12	0	0
TXNDC5	0.885708	0.89639917	-0.01069116	-0.01726899	0.010711512	0.026114133	2	0	0
LUZP6	0.8489594	0.831946562	0.01701283	0.028861259	0.010715571	0.02612048	1	0	0
TOLLIP	0.1579586	0.190099924	-0.03214132	-0.05055306	0.010719057	0.026125425	9	0	0
VKORC1	0.2576311	0.27057927	-0.0129482	-0.07226334	0.010731562	0.026152351	4	0	0
IRAK3	0.2061087	0.201567097	0.004541651	0.525221437	0.010735673	0.026158813	9	0	0
ZMIZ2	0.3036082	0.323834818	-0.02022661	-0.02757685	0.010738564	0.026159685	9	0	0
CYLC2	0.3272511	0.356767963	-0.02951686	-0.12104447	0.010738947	0.026159685	1	0	0
NDFIP2	0.1333056	0.156062409	-0.02275685	-0.09731114	0.0107538	0.026192309	11	0	0
HIST1H2AJ	0.2898771	0.237062874	0.052814247	0.513313541	0.010762157	0.026205545	6	0	0
OCM	0.418958	0.477931476	-0.05897349	-0.18584221	0.010761686	0.026205545	1	0	0
PPP3R1	0.7602155	0.777344654	-0.01712917	-0.03694148	0.010767499	0.026214995	2	0	0
POLDIP2	0.1035625	0.121753471	-0.01819093	-0.06054253	0.010770074	0.026217704	12	0	0
PRKAB1	0.0558506	0.062309244	-0.0064586	-0.10395536	0.010775385	0.026227074	8	0	0
MSTN	0.7648825	0.804693867	-0.03981141	-0.08049964	0.010782	0.026232496	6	0	0
CCHCR1	0.1106322	0.120718693	-0.01008649	-0.05963184	0.010781995	0.026232496	17	0	0
CD72	0.2076845	0.225407719	-0.01772317	-0.04310872	0.010779278	0.026232496	4	0	0
GRIK4	0.2615076	0.282037101	-0.02052951	-0.00278759	0.010783869	0.026233483	6	0	0
WAC	0.1637597	0.182625477	-0.01886576	-0.06857238	0.010796612	0.026260922	8	0	0
SMAP1	0.519132	0.507254044	0.011877977	-0.0031948	0.010804011	0.026275355	6	0	0
NUPL2	0.2162828	0.249455042	-0.03317228	-0.07914095	0.010820297	0.026311394	11	0	0
ARHGEF3	0.2257195	0.235662002	-0.00994247	-0.05702445	0.010827036	0.026324212	10	0	0
TMPRSS9	0.7405583	0.767998344	-0.02744001	-0.0558487	0.010843459	0.02636057	8	0	0
RWDD2B	0.3808023	0.41396772	-0.03316537	0.132432911	0.010857372	0.026390814	11	0	0
SELL	0.2955478	0.32460356	-0.02905573	-0.11358855	0.01087149	0.026417972	4	0	0
TRAPPC11	0.2606332	0.271145281	-0.01051212	0.008216031	0.010870726	0.026417972	6	0	0
S100A14	0.5767563	0.556885255	0.01987104	0.045691518	0.010879518	0.026433899	6	0	0
JPH2	0.4261716	0.400108802	0.026062783	0.280597668	0.010887341	0.026449322	6	0	0
ATP6V1C2	0.1714881	0.183881674	-0.01239354	0.022848905	0.01089086	0.026454288	6	0	0
CDK15	0.851038	0.865407397	-0.01436937	-0.02370129	0.010893934	0.026458173	5	0	0
CALML4	0.5765129	0.596533934	-0.02002102	-0.03383582	0.010897597	0.026463486	7	0	0
DPYSL2	0.0992828	0.070506305	0.028776526	0.617252285	0.010913558	0.026498658	10	0	0
ULK3	0.2413659	0.266052145	-0.02468621	-0.07162236	0.010920586	0.026512132	8	0	0
LRRC70	0.6455107	0.692629191	-0.04711852	-0.10647695	0.01094386	0.02656504	2	0	0
PVRIG	0.8172455	0.856919838	-0.03967437	-0.06758312	0.010947566	0.026570438	1	0	0
GABRA6	0.115542	0.08159014	0.033951896	0.496956101	0.010950259	0.026573379	11	0	0
MMP21	0.7057159	0.748525789	-0.04280986	-0.08241224	0.010952068	0.026574173	10	0	0
FNIP2	0.2216417	0.196469504	0.025172157	0.091266087	0.010966054	0.026604511	8	0	0
CD93	0.4606628	0.406762243	0.053900538	0.179555554	0.010977136	0.026627795	10	0	0
NFAT5	0.2522225	0.284950403	-0.03272786	-0.01715148	0.01099275	0.026662063	6	0	0
MACROD2	0.3470305	0.317527038	0.029503432	0.362951974	0.010998636	0.026672733	16	0	0
KLRG2	0.0493428	0.053616005	-0.0042732	-0.04379751	0.011001859	0.026676942	8	0	0
PCDHGC4	0.2636783	0.302298627	-0.03862037	-0.18564364	0.01100852	0.026689486	8	0	0
B3GNT3	0.191148	0.208769574	-0.01762156	0.024966972	0.011012681	0.026695965	7	0	0
PRDM15	0.2744412	0.29080032	-0.01635909	-0.09693164	0.011032708	0.026740898	8	0	0
PIK3C2A	0.0929504	0.086142921	0.00680744	0.063751428	0.011039544	0.026753853	6	0	0
SNAP91	0.2385149	0.207239073	0.031275845	0.374658061	0.011045134	0.026763783	12	0	0
ZNF227	0.0619602	0.031280091	0.030680078	0.781532507	0.011049932	0.026768177	2	0	0
ZNF628	0.2918373	0.328820289	-0.03698296	-0.07863683	0.011048702	0.026768177	8	0	0
PTPRQ	0.5789805	0.647681458	-0.06870091	-0.15916901	0.011055595	0.026778279	1	0	0
FRYL	0.0537874	0.049066386	0.004721034	0.111526416	0.011057641	0.026779617	10	0	0
UXS1	0.3451131	0.363563699	-0.01845056	-0.01842758	0.011069522	0.026801325	7	0	0
ZBTB11	0.2329074	0.270667287	-0.03775993	-0.08206389	0.011071065	0.026801325	9	0	0
PCMT1	0.4001704	0.454840367	-0.05466994	-0.11571723	0.011072581	0.026801325	10	0	0
SPN	0.4886952	0.534764236	-0.04606902	-0.13788446	0.011072157	0.026801325	8	0	0
HECW1	0.17551	0.145006055	0.030503946	0.401147974	0.011083839	0.026824954	11	0	0

CDH23	0.2335306	0.228007079	0.005523536	0.179307386	0.011086762	0.026828408	5	0	0
KATNB1	0.1649699	0.194023737	-0.02905387	-0.04742267	0.011089526	0.026831477	6	0	0
CHRNA2	0.5672448	0.605362702	-0.03811785	-0.09225361	0.011097119	0.026846227	1	0	0
CLASRP	0.5944248	0.597369598	-0.00294484	0.056049208	0.011100158	0.026849957	8	0	0
RNF122	0.217591	0.226469851	-0.00887882	0.029119642	0.0111045	0.026853985	7	0	0
SPECC1L	0.0635088	0.049560038	0.013948757	0.143975805	0.011104817	0.026853985	4	0	0
CNNM2	0.0353803	0.039207439	-0.00382714	-0.11468776	0.01110734	0.026856465	2	0	0
PDK1	0.4150415	0.396249486	0.018792023	0.096531375	0.011118141	0.026878955	8	0	0
CAMTA2	0.0611648	0.04275851	0.018406305	0.254464736	0.011122273	0.026885323	9	0	0
NDUFB3	0.0795472	0.07396721	0.005579996	0.081062028	0.011126177	0.026891136	15	0	0
FNDC3B	0.1068955	0.117837937	-0.01094241	-0.06493119	0.011137929	0.026899365	14	0	0
C6orf223	0.4292713	0.464159969	-0.03488862	0.020417505	0.011138741	0.026899365	2	0	0
KRT4	0.5895108	0.555990376	0.033520442	0.080178602	0.011134171	0.026899365	5	0	0
WARS	0.2509275	0.275601101	-0.02467364	-0.05385822	0.011133743	0.026899365	9	0	0
SPIRE1	0.2487148	0.271064782	-0.02234999	0.158004149	0.01114008	0.026899365	6	0	0
PSMG2	0.2487148	0.271064782	-0.02234999	0.158004149	0.01114008	0.026899365	6	0	0
ZNF93	0.1981352	0.18920284	0.008932347	0.325161325	0.011138283	0.026899365	6	0	0
CDH13	0.3314295	0.324164162	0.007265347	0.261719853	0.011155213	0.02693228	17	0	0
GPI	0.192888	0.216213388	-0.02332537	-0.07038153	0.011166355	0.02695555	6	0	0
EIF3G	0.2048561	0.245377683	-0.04052156	-0.10035036	0.011175574	0.026970547	10	0	0
BLVRB	0.0948464	0.119012857	-0.02416647	-0.10748222	0.011175364	0.026970547	12	0	0
ATXN10	0.4236436	0.401748774	0.021894834	0.118276977	0.011203553	0.02703443	7	0	0
SLC51A	0.6468043	0.621245419	0.025558885	0.06104432	0.01121267	0.027052791	4	0	0
SMYD3	0.0807053	0.101567458	-0.02086213	-0.13723623	0.011229667	0.027087806	4	0	0
USP13	0.1458453	0.163644726	-0.01779939	-0.13288074	0.011230203	0.027087806	9	0	0
GPR108	0.0532092	0.050771621	0.002437618	0.068700273	0.011232318	0.027089263	11	0	0
LIF	0.1935297	0.214026599	-0.02049695	-0.11196828	0.011243164	0.027111775	9	0	0
C1orf21	0.1391308	0.156867999	-0.01773721	-0.07936303	0.011263404	0.027153217	10	0	0
TNC	0.3277669	0.348664574	-0.02089763	-0.05792629	0.011264891	0.027153217	6	0	0
BTBD8	0.2904826	0.310250668	-0.01976811	-0.02996793	0.011271328	0.027165082	8	0	0
RNF32	0.2459011	0.250358684	-0.00445758	0.4167594	0.011273785	0.027166443	8	0	0
TSPAN8	0.7303844	0.787700336	-0.0573159	-0.11059807	0.011274922	0.027166443	5	0	0
TSPAN1	0.6311155	0.613266881	0.017848629	0.058174999	0.011285033	0.027187152	6	0	0
CNTROB	0.3973705	0.408367626	-0.01099711	0.008001945	0.011292199	0.027200762	13	0	0
SZT2	0.1017648	0.117335476	-0.01557064	-0.04826354	0.011294132	0.027201767	13	0	0
BSN	0.1287434	0.140574959	-0.01183158	-0.01105684	0.01130322	0.027219999	8	0	0
UNC119B	0.5692301	0.58934397	-0.02011384	-0.04043436	0.011319842	0.027256368	11	0	0
UCK2	0.190231	0.161657675	0.028573338	0.146373242	0.011325479	0.027262621	12	0	0
GPR152	0.7372227	0.734175132	0.00304755	0.013951377	0.011324091	0.027262621	9	0	0
PPP1R12C	0.1872115	0.157646921	0.02956461	0.103613623	0.011331776	0.027274119	6	0	0
FAM172A	0.3974379	0.387803002	0.009634936	0.09799196	0.011338476	0.027286584	9	0	0
GAL3ST4	0.4034226	0.430745679	-0.02732311	-0.04226153	0.01134517	0.027299029	9	0	0
AGBL2	0.332306	0.34626891	-0.01396287	-0.0027903	0.011351588	0.027310809	10	0	0
C11orf49	0.2610696	0.270862795	-0.00979321	-0.05212486	0.01135685	0.027319805	11	0	0
ADIPOR1	0.1459257	0.16009535	-0.01416964	0.026014759	0.011363925	0.027333158	12	0	0
SLC22A13	0.8457444	0.857115459	-0.01137111	-0.01822573	0.011371002	0.027346514	4	0	0
VWDE	0.2158131	0.238598982	-0.02278584	0.011681896	0.011387293	0.02737835	12	0	0
DLGAP3	0.1181783	0.085872569	0.032305744	0.661685811	0.011420829	0.0274553	5	0	0
TUBB2A	0.1863636	0.178955712	0.007407933	0.218974116	0.011423222	0.027457372	7	0	0
YPEL2	0.150033	0.124730907	0.025302069	0.211389177	0.01142832	0.027465945	7	0	0
QPCT	0.4291164	0.426544011	0.002572381	0.235100932	0.011435979	0.027474694	9	0	0
SPAG9	0.0844484	0.096614158	-0.01216575	-0.05165926	0.011436047	0.027474694	11	0	0
EBF4	0.2403836	0.25199987	-0.01161631	0.075564247	0.011436556	0.027474694	6	0	0
LGALS3	0.6882242	0.713323268	-0.0250991	-0.06676843	0.011452398	0.027509067	3	0	0
CACNG6	0.3825254	0.382485503	3.99E-05	0.068644273	0.011455494	0.027512821	10	0	0
HVCN1	0.0515639	0.04619636	0.00536758	0.131608872	0.011468051	0.027539291	1	0	0
ZNF554	0.2151525	0.234987205	-0.01983473	-0.04175682	0.011472296	0.027545797	7	0	0
PLEKHA3	0.1425739	0.156300226	-0.01372629	-0.08940803	0.011490033	0.027584691	11	0	0
FGL1	0.7556568	0.797416638	-0.04175984	-0.07697898	0.011502441	0.027610784	5	0	0
PIGZ	0.1285359	0.151192534	-0.02265663	-0.16802072	0.011506419	0.027616638	6	0	0
HIST1H4A	0.1187701	0.085651365	0.03311875	0.643013158	0.011526043	0.027655064	7	0	0
PANK1	0.3925158	0.365915831	0.026599936	0.1470526	0.011527055	0.027655064	9	0	0
TRPV2	0.3886705	0.384009593	0.00466088	0.099355443	0.011525398	0.027655064	5	0	0

ZDHHC4	0.3348066	0.364441434	-0.0296348	0.1075219	0.011539409	0.027681	10	0	0
TEX22	0.2172555	0.219451965	-0.00219644	-0.03986814	0.011541431	0.027682147	8	0	0
FAM20C	0.1247238	0.103342231	0.021381567	0.267754895	0.0115456	0.027684743	3	0	0
ITFG1	0.6814266	0.74322131	-0.0617947	-0.12583862	0.011545561	0.027684743	2	0	0
INTS2	0.2775423	0.298363417	-0.02082113	-0.09641333	0.011552828	0.027698372	7	0	0
DAAM2	0.2122703	0.194589237	0.017681047	0.32473284	0.01155762	0.027706158	12	0	0
TMEM205	0.1449616	0.141656763	0.003304831	0.067207209	0.01156353	0.027716621	10	0	0
RPSA	0.0342591	0.032588312	0.001670785	0.06161831	0.011570588	0.027729832	7	0	0
MMD2	0.3944987	0.398815357	-0.00431665	0.407081854	0.01157809	0.027744104	6	0	0
OR1I1	0.6703425	0.706195745	-0.03585321	-0.1045819	0.01159033	0.027769724	4	0	0
PLEKHD1	0.1557457	0.132315793	0.023429881	0.401235512	0.011594923	0.027777018	13	0	0
COX16	0.2881228	0.260657438	0.027465326	0.07558614	0.011615958	0.027823693	10	0	0
ZYG11A	0.5626282	0.590904958	-0.02827677	-0.03128865	0.011624001	0.027838605	7	0	0
NSUN7	0.2858079	0.296746994	-0.01093913	0.005815597	0.01162576	0.027838605	8	0	0
RFX1	0.2089089	0.238332813	-0.02942394	-0.13472943	0.011626839	0.027838605	2	0	0
TTC33	0.2958968	0.326599562	-0.03070277	-0.10811676	0.011628478	0.027838813	10	0	0
RNF114	0.0458098	0.052400396	-0.00659055	-0.09884447	0.011635452	0.027851791	5	0	0
INO80B	0.0808424	0.083680574	-0.00283813	-0.05680478	0.011640396	0.027853958	13	0	0
USH1G	0.1457701	0.116720039	0.029050089	0.420775988	0.011641016	0.027853958	20	0	0
ZNF134	0.1648536	0.146359884	0.018493719	0.404998479	0.011638651	0.027853958	10	0	0
RINL	0.5159431	0.518268622	-0.00232554	0.008359016	0.011643613	0.027856455	4	0	0
RAB20	0.2160262	0.25044092	-0.03441468	-0.0271512	0.011648521	0.02786448	10	0	0
EHBP1L1	0.066786	0.075456263	-0.0086703	-0.08041078	0.011650969	0.027866619	11	0	0
MVB12B	0.5811448	0.587933895	-0.00678914	0.025411869	0.011653748	0.027869549	5	0	0
XYLB	0.4599682	0.469653769	-0.00968559	0.031161148	0.011665925	0.027894952	6	0	0
GBA2	0.5214685	0.521082987	0.000385536	0.024452373	0.011667715	0.027895514	4	0	0
SEC24A	0.056446	0.055530571	0.000915383	0.044349035	0.011671614	0.027901115	10	0	0
NAP1L5	0.6127916	0.614741194	-0.00194958	0.006341277	0.011674527	0.02790436	7	0	0
STX6	0.1677449	0.200277144	-0.03253222	-0.09869427	0.011684452	0.027924362	9	0	0
RAB32	0.3455366	0.361135405	-0.01559882	-0.00566481	0.011693336	0.027941869	16	0	0
KCNK2	0.5799824	0.628420297	-0.04843786	-0.10780838	0.011697573	0.02794827	8	0	0
SLC36A2	0.5170101	0.563080208	-0.04607007	-0.10961086	0.011704897	0.02795832	8	0	0
FOPNL	0.2826783	0.309005583	-0.02632731	-0.01721617	0.011703778	0.02795832	13	0	0
CCKBR	0.0931617	0.06517034	0.027991324	0.582656647	0.01170703	0.027959692	10	0	0
NCAPH2	0.135722	0.12336138	0.012360622	0.08501591	0.01170864	0.027959815	9	0	0
MED31	0.1494116	0.162441937	-0.01303031	-0.07464489	0.011711402	0.027962687	15	0	0
TAS2R46	0.8494044	0.872086124	-0.0226817	-0.03752617	0.011713289	0.027963471	3	0	0
AQP11	0.3272319	0.375448081	-0.04821617	-0.10524287	0.011716575	0.027965349	9	0	0
NOB1	0.0843869	0.093402902	-0.00901603	-0.06077104	0.011717194	0.027965349	9	0	0
SAR1A	0.1918651	0.228110555	-0.03624542	-0.05438196	0.0117222	0.027973573	9	0	0
POP5	0.0912411	0.107063426	-0.01582231	-0.14811847	0.011727453	0.027978664	7	0	0
PTPN21	0.230657	0.246235404	-0.01557844	0.011995261	0.011726727	0.027978664	11	0	0
SF3B3	0.0431424	0.039644891	0.003497551	0.094474677	0.01174046	0.02800597	11	0	0
AIMP1	0.2615453	0.29870052	-0.03715526	-0.15262671	0.011745121	0.028008798	7	0	0
RNF146	0.3617177	0.384678437	-0.0229607	-0.02951901	0.01174633	0.028008798	12	0	0
GPN3	0.0317725	0.030477158	0.001295375	0.084347103	0.011745032	0.028008798	9	0	0
CCDC102B	0.0999087	0.123132546	-0.02322389	-0.09068804	0.011751336	0.02801701	5	0	0
RAB4B	0.1473057	0.151696975	-0.00439124	0.037412264	0.011777747	0.028076246	10	0	0
TWISTNB	0.2990166	0.328494395	-0.02947778	-0.06363949	0.01178339	0.028082233	8	0	0
CSNK2A1	0.2208478	0.213035966	0.007811848	0.11403046	0.011782235	0.028082233	11	0	0
PLEKHF1	0.2419656	0.278065197	-0.03609957	-0.04689551	0.011787588	0.028088505	6	0	0
PDAP1	0.0427566	0.054664757	-0.01190813	-0.09572365	0.011806038	0.028128733	16	0	0
VRK1	0.1683198	0.190937948	-0.0226182	-0.05046737	0.011807975	0.028129611	6	0	0
KCNT2	0.4376539	0.43264862	0.005005264	0.125228712	0.01182087	0.028156591	4	0	0
TNFSF11	0.7127317	0.74802503	-0.03529336	-0.0642664	0.011822803	0.028157455	5	0	0
ENDOD1	0.3293347	0.350745783	-0.02141108	0.071073376	0.011829283	0.028169148	7	0	0
CCDC86	0.2437058	0.249729021	-0.0060232	-0.03114871	0.011842676	0.028197296	8	0	0
UGT2B15	0.7615329	0.783950526	-0.02241759	-0.05302365	0.011861517	0.028227168	3	0	0
CD276	0.2127309	0.218379237	-0.00564829	0.068574798	0.011861168	0.028227168	10	0	0
BRD7	0.4158657	0.413269824	0.002595903	0.008423363	0.011859905	0.028227168	7	0	0
PRR14L	0.2119156	0.197997025	0.013918593	0.058761017	0.011859826	0.028227168	9	0	0
CAPN3	0.8890133	0.90430817	-0.01529487	-0.02445859	0.01186823	0.028239397	3	0	0
BRF1	0.2059496	0.2243636	-0.01841403	-0.06986527	0.011870378	0.028240762	8	0	0

KREMEN2	0.0753926	0.06524951	0.01014305	0.077916398	0.011888203	0.028279418	10	0	0
GCH1	0.2080258	0.237173857	-0.02914809	-0.11472836	0.011901833	0.028304334	7	0	0
TRIM16L	0.2087714	0.194939644	0.01383179	0.109062032	0.011900645	0.028304334	2	0	0
ESR2	0.0631048	0.07349173	-0.01038689	-0.06478732	0.011921197	0.028343285	7	0	0
PTGR1	0.5061757	0.515851685	-0.00967595	0.065618722	0.011929501	0.028358851	6	0	0
VWC2L	0.2516738	0.279907838	-0.028234	-0.08740908	0.011932798	0.028362928	7	0	0
CCK	0.4103898	0.374401191	0.035988623	0.206094101	0.011942203	0.028377762	7	0	0
ARSB	0.3082381	0.28375545	0.024482697	0.243892081	0.011941718	0.028377762	6	0	0
UBIAD1	0.1526536	0.15515387	-0.00250026	0.042701556	0.011955212	0.02840115	8	0	0
HTRA3	0.3685274	0.377900747	-0.00937337	0.128124742	0.011954973	0.02840115	11	0	0
TMC4	0.2565126	0.263709422	-0.00719685	-0.10170764	0.011962824	0.02841547	6	0	0
LRRC42	0.0332828	0.030648013	0.00263479	0.07174389	0.011973529	0.028437132	12	0	0
CUEDC2	0.0814335	0.102231378	-0.02079791	-0.05898707	0.011979791	0.028448237	9	0	0
PLEKHF2	0.3215	0.351471524	-0.02997155	-0.09093437	0.011985708	0.028454754	9	0	0
DTX4	0.4680698	0.504629536	-0.03655972	-0.02363709	0.011985185	0.028454754	5	0	0
SNX29	0.037685	0.033886328	0.003798646	0.097232687	0.011987448	0.028455119	8	0	0
PDE6C	0.6964372	0.725402703	-0.02896547	-0.04971772	0.011997342	0.028474835	9	0	0
ATRIP	0.242574	0.259494751	-0.01692078	0.001009865	0.012001548	0.028481049	10	0	0
CLNS1A	0.3649186	0.369796004	-0.00487744	-0.078175	0.012011191	0.028500162	7	0	0
NRXN1	0.1649057	0.149320282	0.015585409	0.272327594	0.012013698	0.02850234	16	0	0
PHLDA2	0.0631661	0.071997527	-0.00883139	-0.03026349	0.012016899	0.028506164	27	0	0
GPR157	0.1173597	0.114159558	0.003200142	0.044788245	0.012030152	0.028529782	14	0	0
LEMD1	0.0625386	0.041216831	0.021321733	0.496836996	0.01203048	0.028529782	11	0	0
USH1C	0.4163267	0.417888243	-0.00156151	0.155040716	0.012031627	0.028529782	11	0	0
RCN3	0.4859707	0.465263502	0.020707203	0.605750125	0.012045151	0.028558075	6	0	0
DEPDC7	0.2672368	0.302746265	-0.03550947	0.188773857	0.012047172	0.028559091	9	0	0
TNNI3K	0.2004904	0.217498462	-0.01700802	-0.05326562	0.012063493	0.028594001	12	0	0
KCNMB3	0.7974339	0.78845074	0.008983174	0.019793913	0.012067957	0.028600803	3	0	0
OGFRL1	0.1241549	0.127901086	-0.00374621	0.010915881	0.012072321	0.028607366	5	0	0
LMF2	0.0677122	0.057776553	0.009935657	0.099841351	0.012077154	0.028615036	8	0	0
RORA	0.1188393	0.095869794	0.022969555	0.346324641	0.012085113	0.028630114	11	0	0
DEFB132	0.6436879	0.701911892	-0.05822402	-0.1269682	0.012091029	0.028636563	5	0	0
LTN1	0.2777506	0.304974445	-0.02722381	-0.05337442	0.012090189	0.028636563	11	0	0
SLFN13	0.3787846	0.368597857	0.010186734	0.081102971	0.012101322	0.028657159	11	0	0
BTBD16	0.7079278	0.719174866	-0.01124707	-0.01981225	0.012110732	0.028675656	6	0	0
CCL22	0.5465959	0.58730102	-0.04070517	-0.10849739	0.012128752	0.028714532	3	0	0
COL10A1	0.7663751	0.794479679	-0.02810462	-0.0513022	0.012132059	0.028718528	1	0	0
CCIN	0.7224775	0.748359916	-0.02588247	-0.05009836	0.012133642	0.028718528	1	0	0
C2orf80	0.7767395	0.811455409	-0.03471592	-0.06484986	0.012151695	0.028754816	8	0	0
SRCAP	0.0722423	0.076452161	-0.00420983	-0.09038425	0.01215218	0.028754816	6	0	0
CALCR	0.11733	0.098391985	0.018937994	0.322458248	0.012197023	0.028854075	22	0	0
SYNGR4	0.128982	0.143941439	-0.01495945	-0.07626682	0.012197346	0.028854075	11	0	0
N4BP2	0.0381322	0.045985703	-0.00785347	-0.10726922	0.012225506	0.028916878	8	0	0
NRM	0.228825	0.230853972	-0.00202902	0.058415389	0.012230112	0.028920142	11	0	0
ZNF74	0.2306902	0.239929243	-0.00923905	0.027207202	0.012228958	0.028920142	14	0	0
GIPC1	0.1639589	0.146232078	0.017726819	0.106398881	0.012235579	0.028929256	10	0	0
CCDC42	0.3783675	0.420155764	-0.04178825	-0.0605789	0.012237589	0.028930197	8	0	0
CX3CL1	0.52926	0.488968113	0.040291885	0.124212068	0.012241805	0.028936348	10	0	0
FRMD8	0.0714428	0.081147066	-0.00970424	-0.05372503	0.012254388	0.028962274	9	0	0
VARS	0.1749566	0.191636546	-0.01667995	-0.00139436	0.012265693	0.028985173	20	0	0
MSI1	0.0603724	0.046653116	0.013719236	0.400523845	0.012272278	0.028996915	5	0	0
IGFBP1	0.4410104	0.461007015	-0.01999666	-0.05211344	0.012276014	0.029001922	8	0	0
PTTG1	0.2065431	0.217328913	-0.01078584	-0.03994197	0.012283533	0.029015863	14	0	0
DNAJC15	0.4998348	0.490647078	0.009187686	0.229772062	0.012306098	0.029065338	6	0	0
THBS1	0.1655892	0.131465666	0.034123571	0.310708552	0.012331474	0.029121437	20	0	0
CCDC175	0.2577516	0.286829391	-0.02907779	-0.10318294	0.012334706	0.029125235	7	0	0
SAPCD2	0.0611562	0.071930386	-0.01077416	-0.0933367	0.012337353	0.029127651	5	0	0
TMEM209	0.7842001	0.811441631	-0.0272415	-0.05153972	0.012346733	0.02914596	2	0	0
C9orf153	0.8750279	0.891453397	-0.01642546	-0.0269178	0.012362318	0.029178911	6	0	0
ZNF169	0.2859267	0.311343239	-0.02541652	0.109743091	0.012383677	0.029225477	10	0	0
COL6A1	0.1081351	0.116379663	-0.00824457	0.084168877	0.012388147	0.02923218	7	0	0
PRKCE	0.0223495	0.021013103	0.001336434	0.055032748	0.012395566	0.029245839	3	0	0
GULP1	0.3033508	0.291699447	0.011651367	0.276508551	0.012411073	0.029278575	9	0	0

GLS2	0.1127614	0.127506718	-0.01474532	-0.06253594	0.012413045	0.029279375	9	0	0
NQO1	0.1707949	0.193301814	-0.0225069	0.0217712	0.012424032	0.029301439	15	0	0
LPAR3	0.1752956	0.162674923	0.012620701	0.396235312	0.012425913	0.029302021	8	0	0
FSTL4	0.1921192	0.198207989	-0.00608878	0.024635688	0.012445507	0.029344369	10	0	0
MAGOHB	0.153939	0.171544721	-0.0176057	-0.07883782	0.012450058	0.02935124	7	0	0
GPALPP1	0.1672985	0.182061076	-0.01476256	-0.02654693	0.012466837	0.029386935	12	0	0
ZFAND2B	0.8214866	0.843741381	-0.02225476	-0.03810605	0.01247225	0.029395831	1	0	0
CD36	0.7756997	0.765827064	0.009872649	0.02858265	0.012482466	0.029412644	4	0	0
CCNE1	0.0240931	0.023338115	0.000754983	0.046917517	0.012482663	0.029412644	6	0	0
FGFBP1	0.5893031	0.585283593	0.004019502	0.016574083	0.012484671	0.02941351	11	0	0
ITGB3BP	0.061521	0.068957483	-0.00743647	-0.13445525	0.012489854	0.029417993	10	0	0
FASLG	0.7657442	0.786113535	-0.0203693	-0.04358249	0.012488663	0.029417993	4	0	0
CASP8	0.0924768	0.098647029	-0.00617026	0.017494615	0.012495743	0.029420274	6	0	0
AKAP9	0.123188	0.124939096	-0.00175109	-0.06199504	0.012495066	0.029420274	9	0	0
CPED1	0.0956563	0.062576557	0.033079741	0.568265239	0.012494949	0.029420274	11	0	0
SLC16A14	0.4117296	0.438244966	-0.02651538	-0.05358555	0.01252099	0.029468738	9	0	0
NT5C2	0.1861901	0.215599244	-0.02940917	-0.02815978	0.012521256	0.029468738	11	0	0
CREBZF	0.3976752	0.340958458	0.056716787	0.340441168	0.012519084	0.029468738	6	0	0
SCTR	0.6421785	0.608182749	0.033995753	0.164802565	0.012526154	0.029476397	7	0	0
MIOX	0.6140509	0.626722966	-0.01267208	-0.02541445	0.012533802	0.029490526	11	0	0
DHX30	0.0376461	0.038116617	-0.00047052	0.006601684	0.012537792	0.029496044	7	0	0
HRH3	0.1205343	0.091497732	0.029036537	0.306661203	0.012540546	0.029498653	11	0	0
TRIM6-TRIM	0.4208904	0.442521233	-0.02163086	0.030575599	0.01255163	0.029520854	6	0	0
DNAH11	0.3659445	0.363007817	0.002936654	0.231045931	0.012562854	0.029543378	7	0	0
HCK	0.2093767	0.179336967	0.030039773	0.312195184	0.012568419	0.02955259	18	0	0
UGT1A10	0.7729206	0.812399493	-0.03947887	-0.07690553	0.012582093	0.029576988	6	0	0
SPINT3	0.5552621	0.610017989	-0.05475586	-0.13262421	0.012582042	0.029576988	3	0	0
FAM173A	0.1006844	0.111484437	-0.01080006	-0.0519562	0.012584543	0.029578871	6	0	0
PDGFA	0.121738	0.136540946	-0.01480293	-0.07050174	0.012588116	0.029583393	7	0	0
VTN	0.578034	0.611462471	-0.03342852	-0.04195374	0.012593521	0.029588341	3	0	0
SEBOX	0.578034	0.611462471	-0.03342852	-0.04195374	0.012593521	0.029588341	3	0	0
SLC25A35	0.0692666	0.061654008	0.007612608	0.085006091	0.012609536	0.029622088	7	0	0
RUVBL1	0.1329974	0.146887	-0.01388962	-0.06228556	0.012613605	0.029627766	8	0	0
MCPH1	0.0332279	0.030358165	0.002869693	0.084527228	0.012617999	0.029634206	4	0	0
GLUL	0.350475	0.354597073	-0.00412207	0.044573102	0.012636424	0.029673593	6	0	0
TGM4	0.7848318	0.800262894	-0.01543111	-0.02692277	0.012657696	0.029719654	4	0	0
FOXN3	0.2618811	0.285347743	-0.02346662	-0.01183175	0.012663899	0.029726436	6	0	0
RASL12	0.6847714	0.669427144	0.015344207	0.044334364	0.012663273	0.029726436	6	0	0
KCNS3	0.2747434	0.299062654	-0.02431922	-0.02658234	0.012674591	0.02974764	11	0	0
FAM102A	0.1319008	0.136491843	-0.00459105	-0.04995285	0.012688635	0.029776706	7	0	0
C9orf116	0.6135325	0.652741125	-0.03920864	-0.10119296	0.012693264	0.029781549	4	0	0
SYNDIG1	0.3727407	0.355776714	0.01696395	0.210425956	0.01269402	0.029781549	8	0	0
ZNF490	0.2967633	0.260985818	0.035777509	0.537186715	0.01270656	0.029807071	6	0	0
MAPK8IP1	0.2822181	0.296858017	-0.01463993	-0.01335797	0.012724787	0.029842022	15	0	0
SPNS1	0.4461742	0.505717136	-0.05954292	-0.16159061	0.012723231	0.029842022	3	0	0
WNT7A	0.1982428	0.195215872	0.003026931	0.175010249	0.012743142	0.029877255	6	0	0
C2orf70	0.1567496	0.16051376	-0.00376414	0.203417895	0.012745797	0.029879573	11	0	0
RASGEF1B	0.0986549	0.081376634	0.017278226	0.341083698	0.012750794	0.02988738	4	0	0
TCIRG1	0.239003	0.214361325	0.0246417	0.695167432	0.01276389	0.029914167	10	0	0
DSC3	0.2568847	0.24117946	0.015705289	0.200215613	0.012772645	0.029922958	15	0	0
PXMP4	0.2252422	0.239946189	-0.01470396	0.30513865	0.012772183	0.029922958	13	0	0
CECR2	0.6178137	0.64889308	-0.03107934	-0.08840785	0.012769996	0.029922958	3	0	0
MRTO4	0.1917565	0.20151435	-0.00975782	-0.02370782	0.01278075	0.029938036	11	0	0
PGBD5	0.0801895	0.064515918	0.015673568	0.245937325	0.012806462	0.029994345	10	0	0
AEBP2	0.8401713	0.830952657	0.009218614	0.037785937	0.01281141	0.030002016	7	0	0
ATPAF2	0.1237053	0.110493994	0.013211277	0.079363079	0.012826958	0.030034507	14	0	0
COL24A1	0.1717043	0.146136188	0.025568149	0.449932022	0.01283083	0.03003659	7	0	0
RAB3GAP1	0.3272002	0.312643479	0.014556715	0.034445412	0.012831197	0.03003659	11	0	0
FBXO44	0.3708984	0.383428355	-0.01252995	0.069042192	0.01284574	0.03006671	11	0	0
MCU	0.1384339	0.161021996	-0.02258805	-0.04628322	0.012848482	0.030069203	11	0	0
MCM6	0.1328918	0.151628779	-0.01873697	-0.08824398	0.012855032	0.030080606	10	0	0
ZNF853	0.1868245	0.180356649	0.006467819	0.437499498	0.012858708	0.030081359	8	0	0
TGM2	0.6306169	0.600218301	0.030398572	0.060961661	0.012858221	0.030081359	3	0	0

TFAP4	0.0557693	0.054146431	0.001622849	0.040307286	0.012882806	0.030133803	12	0	0
TOPBP1	0.059417	0.069257502	-0.00984055	-0.11133849	0.012887963	0.030141936	13	0	0
KAT6B	0.1446555	0.165978457	-0.021323	-0.07124296	0.012890984	0.030142	7	0	0
STK11	0.8831295	0.896535281	-0.01340581	-0.02149385	0.012891352	0.030142	1	0	0
SPTAN1	0.1238973	0.100554817	0.02334248	0.113887638	0.012896445	0.03014998	7	0	0
VAMP2	0.2324859	0.197226835	0.035259027	0.172353495	0.012899375	0.030152899	9	0	0
BCL11B	0.061182	0.052974591	0.008207449	0.19325745	0.012904945	0.030161987	11	0	0
TRAF3IP2	0.256238	0.275795932	-0.01955789	-0.11885104	0.012937657	0.030234505	6	0	0
CCT4	0.2253831	0.272022086	-0.04663895	-0.10830784	0.012945504	0.0302489	10	0	0
TMEM259	0.038268	0.03641842	0.001849549	0.047011598	0.012953603	0.030259942	4	0	0
TOP1	0.2650541	0.285464813	-0.02041072	-0.04232957	0.012952901	0.030259942	10	0	0
PADI1	0.7323346	0.773687916	-0.04135331	-0.08138832	0.012971911	0.030298763	5	0	0
RANBP3L	0.3491406	0.314440342	0.034700215	0.250167268	0.01297609	0.030304577	5	0	0
NTMT1	0.5591807	0.548633129	0.0105476	0.063055014	0.012986356	0.030324603	7	0	0
CYP3A7	0.8159841	0.825469317	-0.00948517	-0.01302604	0.013000547	0.030347293	2	0	0
WIPF2	0.1687287	0.178524025	-0.00979531	-0.01974331	0.013000377	0.030347293	12	0	0
PLK5	0.4974956	0.512289736	-0.01479414	0.165255572	0.013001148	0.030347293	6	0	0
C11orf95	0.1633403	0.106407923	0.056932386	0.501974484	0.013022666	0.030393565	4	0	0
GPD1L	0.6626405	0.685443222	-0.02280274	-0.04770157	0.013034523	0.030416096	5	0	0
PPIA	0.2575565	0.280075396	-0.02251889	-0.05105552	0.013035712	0.030416096	7	0	0
OSMR	0.1615016	0.166455442	-0.00495387	0.170324873	0.013040823	0.030424064	5	0	0
CERS6	0.2631713	0.285453613	-0.02228236	-0.09840612	0.013046286	0.03043285	9	0	0
OTUD1	0.3299423	0.349589451	-0.01964714	-0.06261366	0.013052762	0.030443998	6	0	0
DIP2A	0.0605277	0.069160585	-0.00863289	0.006511757	0.013064391	0.03046716	11	0	0
CCDC47	0.6847713	0.637019501	0.047751848	0.102729066	0.013087736	0.030517633	1	0	0
CD83	0.2288082	0.237253986	-0.00844578	-0.0041339	0.01309609	0.030533144	10	0	0
SPATA20	0.3046083	0.306660771	-0.00205245	0.390489743	0.013099137	0.030536277	6	0	0
FAM149A	0.6282934	0.646596915	-0.01830347	0.028475589	0.013110577	0.030558974	7	0	0
POLR3G	0.8717311	0.888140677	-0.01640959	-0.02667642	0.013114854	0.030564972	2	0	0
ERCC3	0.2220777	0.241782998	-0.0197053	-0.02276838	0.01312477	0.030584108	8	0	0
PSAPL1	0.5274558	0.528574938	-0.00111915	0.00332525	0.013137863	0.030606664	5	0	0
BMP7	0.2026241	0.168393773	0.034230361	0.363768439	0.013136876	0.030606664	13	0	0
USP54	0.0532243	0.052064429	0.001159856	0.040013518	0.013152304	0.030636328	8	0	0
WDR18	0.0308156	0.028859037	0.001956597	0.071311416	0.013172359	0.030679058	4	0	0
RNF13	0.2266602	0.247600781	-0.02094053	-0.02483919	0.013176765	0.030681353	15	0	0
OTUD4	0.0631891	0.075186058	-0.01199694	-0.05088207	0.013176284	0.030681353	7	0	0
PCCB	0.5620695	0.570024613	-0.00795511	-0.05129284	0.013182693	0.03068845	5	0	0
SLC38A2	0.2464898	0.196145376	0.050344468	0.33161559	0.013183235	0.03068845	12	0	0
GPX3	0.3358546	0.308828427	0.027026216	0.259603977	0.013192027	0.030700946	11	0	0
NXNL2	0.1808304	0.192194156	-0.01136377	-0.06454237	0.013190712	0.030700946	6	0	0
SLC35F1	0.1368245	0.088749478	0.048075054	0.720499933	0.013195525	0.030705103	8	0	0
CD2AP	0.2538761	0.269963951	-0.01608789	-0.1248188	0.013202355	0.030716435	7	0	0
SLC2A8	0.2838252	0.274623286	0.009201865	0.052397994	0.013205427	0.030716435	9	0	0
PTPN9	0.1065611	0.111930631	-0.00536949	0.006655225	0.013205533	0.030716435	13	0	0
BLOC1S3	0.0557208	0.061917324	-0.00619655	-0.03237885	0.01321595	0.030736681	12	0	0
SMAD1	0.3291166	0.359670356	-0.03055377	-0.07340918	0.013220791	0.030743953	5	0	0
BIN3	0.3294396	0.347754187	-0.01831459	-0.04442944	0.013226207	0.030752561	12	0	0
RPRD1A	0.3606047	0.382040062	-0.02143534	-0.03494133	0.013231707	0.03076136	8	0	0
FMNL2	0.2251134	0.237054114	-0.01194076	-0.06883772	0.013239849	0.0307763	9	0	0
FMO4	0.0758459	0.090407206	-0.01456126	-0.11396258	0.013248637	0.030788749	4	0	0
TYRO3	0.3430162	0.35919121	-0.01617503	-0.01278997	0.013247403	0.030788749	5	0	0
THEGL	0.143673	0.150019935	-0.00634689	-0.0446601	0.013250478	0.030789038	5	0	0
RBX1	0.1683703	0.159564892	0.008805443	0.180319803	0.013256061	0.03079802	11	0	0
CACNA1C	0.8588822	0.859144524	-0.00026231	0.001141631	0.013262093	0.030804053	2	0	0
NEURL1B	0.4558427	0.468166956	-0.01232422	-0.00136077	0.01328346	0.030843684	5	0	0
ZSCAN9	0.19797	0.21448105	-0.01651109	-0.0617569	0.013284314	0.030843684	11	0	0
TESC	0.2071378	0.205762138	0.001375691	0.247478432	0.013283857	0.030843684	7	0	0
SVIL	0.0983368	0.071904054	0.026432732	0.401153237	0.013286055	0.030843734	5	0	0
HGS	0.0411297	0.047351921	-0.00622227	-0.16568152	0.0132921	0.030853772	1	0	0
CHST1	0.2109082	0.203550056	0.007358114	0.136426924	0.013298752	0.03086522	10	0	0
SHMT1	0.2624809	0.269358563	-0.00687771	-0.03769236	0.013303586	0.03086845	7	0	0
TSPAN10	0.0516251	0.050872649	0.000752488	0.036061141	0.013303036	0.03086845	8	0	0
GMEB2	0.0940692	0.109546822	-0.01547761	-0.15316646	0.013313684	0.030887884	6	0	0

WDR47	0.1070713	0.116316178	-0.00924485	0.006638914	0.013325088	0.030903255	13	0	0
ZNF783	0.1969605	0.206389836	-0.00942929	0.059682048	0.013323642	0.030903255	6	0	0
CADM1	0.1772121	0.165594832	0.011617284	0.220919873	0.013326658	0.030903255	12	0	0
KIFC3	0.2944787	0.260664872	0.033813857	0.290316748	0.013327201	0.030903255	3	0	0
TMEM89	0.809201	0.822110211	-0.01290925	-0.02258019	0.013330924	0.030907892	11	0	0
WDR33	0.1958122	0.228148535	-0.03233629	-0.18070063	0.013339245	0.030923187	6	0	0
AAED1	0.150646	0.159424012	-0.00877797	-0.0095503	0.013344471	0.030931305	7	0	0
NEIL3	0.1640596	0.184590351	-0.02053078	-0.07113343	0.013351344	0.030939239	10	0	0
OPN1SW	0.8915499	0.922199666	-0.03064976	-0.04973175	0.013350347	0.030939239	3	0	0
MAP2K4	0.3286119	0.349244073	-0.02063222	-0.03984347	0.013375022	0.030990104	8	0	0
TELO2	0.2855953	0.26590948	0.019685858	0.072143249	0.013387174	0.031014255	6	0	0
SLC5A11	0.6936357	0.703214801	-0.00957907	-0.00147131	0.013392383	0.031022315	11	0	0
MRPL1	0.1824525	0.191516238	-0.00906373	0.031436012	0.013394374	0.031022734	10	0	0
SCEL	0.6358124	0.708262338	-0.07244989	-0.15339562	0.013396023	0.031022734	1	0	0
PAPOLG	0.1913267	0.206300774	-0.0149741	-0.0793686	0.013404839	0.03103795	8	0	0
NUSAP1	0.0730063	0.079901575	-0.00689528	-0.01955733	0.013406055	0.03103795	17	0	0
TAF9	0.1403844	0.149653419	-0.00926902	-0.02227148	0.013408056	0.031038577	16	0	0
ASAP2	0.1874463	0.207380523	-0.01993422	-0.01911348	0.013414996	0.031050634	7	0	0
NLRC4	0.654522	0.652054006	0.002467984	0.025598115	0.013429627	0.031080489	7	0	0
PLEKHM2	0.200423	0.194415586	0.006007444	0.090024001	0.013436067	0.031091172	7	0	0
MRPL23	0.3206248	0.324721792	-0.00409701	0.058761588	0.01343771	0.031091172	10	0	0
GPM6A	0.2124051	0.209196334	0.003208736	0.392749779	0.013446088	0.031106545	9	0	0
NEK2	0.178672	0.202734571	-0.02406253	-0.07583102	0.013454147	0.031121174	5	0	0
PCDHB9	0.5477571	0.529841504	0.017915596	0.152200782	0.013502962	0.031226035	6	0	0
ZNF528	0.1516736	0.120617483	0.031056159	0.465016663	0.013502809	0.031226035	13	0	0
PRICKLE1	0.0506474	0.037285386	0.013362037	0.368995323	0.013517463	0.031255538	6	0	0
STEAP3	0.1648971	0.172230537	-0.00733348	-0.04424535	0.013528359	0.031276701	7	0	0
PDCD2	0.1403755	0.158320486	-0.01794501	-0.06142404	0.013539314	0.031297995	10	0	0
GATAD2B	0.079828	0.112581387	-0.03275335	-0.10625076	0.013541587	0.031299214	8	0	0
MYO1D	0.0462531	0.04820852	-0.00195539	-0.04106986	0.013544234	0.031301299	7	0	0
GIGYF2	0.1356633	0.152781667	-0.0171184	-0.0376866	0.013552444	0.031303319	8	0	0
SYNGAP1	0.446046	0.463423498	-0.0173775	0.04933967	0.013550143	0.031303319	13	0	0
NMT2	0.2435171	0.21975612	0.023760958	0.156688548	0.013548347	0.031303319	11	0	0
CTSW	0.531901	0.562578324	-0.03067732	-0.0956481	0.013552843	0.031303319	7	0	0
P2RY2	0.1373762	0.150307135	-0.01293096	0.094477669	0.013553834	0.031303319	10	0	0
ATP1B3	0.110692	0.141933738	-0.03124175	-0.11466984	0.013582199	0.031364789	5	0	0
OPN5	0.5550459	0.57255391	-0.01750798	-0.04467074	0.013586529	0.031370749	8	0	0
ACTR1B	0.2286757	0.259160372	-0.03048468	-0.09588511	0.013600545	0.03139907	11	0	0
PCYT2	0.3801112	0.391464851	-0.0113537	-0.0143489	0.013602435	0.031399392	12	0	0
ZNF257	0.3497398	0.297261841	0.052478006	0.374840134	0.013611894	0.031417183	7	0	0
ALDH8A1	0.7236819	0.741761806	-0.01807986	-0.04014109	0.013614723	0.03141967	8	0	0
SAMD9L	0.6093011	0.617516998	-0.00821592	0.199522485	0.013628732	0.031447953	3	0	0
TAAR6	0.7564152	0.776842564	-0.02042734	-0.02898142	0.013647004	0.031484968	8	0	0
KLK5	0.4894617	0.467816918	0.021644765	0.131658644	0.013648284	0.031484968	7	0	0
TMPRSS13	0.488212	0.533948238	-0.04573627	-0.11903168	0.0136539	0.031489823	5	0	0
SIRPA	0.483536	0.484469009	-0.00093299	0.070369241	0.01365223	0.031489823	4	0	0
RGS7	0.1672854	0.099493574	0.067791815	0.865600286	0.013662757	0.031502147	10	0	0
DCAF17	0.1008868	0.123379257	-0.0224925	-0.08646498	0.013662603	0.031502147	12	0	0
GNG2	0.755392	0.804748993	-0.04935697	-0.09201211	0.013666301	0.031506268	2	0	0
PDC	0.7127366	0.7611915	-0.04845488	-0.09377449	0.013672169	0.031515747	4	0	0
LGR4	0.188965	0.217104683	-0.02813964	-0.11186908	0.013674047	0.031516025	14	0	0
LRRC14B	0.3111721	0.33509787	-0.02392579	-0.10961965	0.013687224	0.031542341	13	0	0
EDEM2	0.302912	0.318665394	-0.0157534	0.239235652	0.013711527	0.03159429	3	0	0
YTHDF2	0.1868958	0.192210021	-0.00531426	0.016084249	0.013717784	0.031604646	13	0	0
IARS2	0.3197795	0.336786881	-0.01700737	-0.01653266	0.013721142	0.031608324	7	0	0
RPS14	0.1095848	0.106834635	0.00275013	0.061775132	0.013723645	0.031610028	14	0	0
SLC8A3	0.2133097	0.222782867	-0.00947313	0.244393424	0.013743757	0.031652289	10	0	0
SYPL1	0.136848	0.158007676	-0.02115966	-0.09182753	0.013756731	0.031678101	9	0	0
RBM11	0.3468196	0.372704825	-0.02588524	-0.0936331	0.01376666	0.031696895	10	0	0
KLF13	0.1157893	0.13097589	-0.01518659	-0.10782678	0.013776445	0.031715354	5	0	0
PRELID2	0.1945155	0.19650096	-0.00198545	0.126843599	0.01378525	0.031731551	13	0	0
TFAP2E	0.3913296	0.371106602	0.02022301	0.149353236	0.013797348	0.031744596	13	0	0
TMEM138	0.0904907	0.109907105	-0.01941641	-0.05638053	0.013797997	0.031744596	16	0	0

ZSWIM7	0.1038707	0.106369567	-0.00249889	-0.01004322	0.013796968	0.031744596	11	0	0
APOC4	0.6931171	0.728406526	-0.03528939	-0.06866791	0.013797213	0.031744596	9	0	0
PPP1R3E	0.2203212	0.239669197	-0.01934797	-0.06748862	0.013807928	0.03176337	11	0	0
RBM6	0.1098517	0.115052492	-0.00520081	-0.0414979	0.013822201	0.031784886	12	0	0
MLXIP	0.7043804	0.729835767	-0.02545536	-0.03473028	0.013821488	0.031784886	4	0	0
FLCN	0.067801	0.062536087	0.00526492	0.060166108	0.013822598	0.031784886	11	0	0
NPRL2	0.2143557	0.234571911	-0.02021619	-0.07646155	0.013829758	0.031792346	15	0	0
PTGES	0.4390197	0.44697043	-0.00795072	0.035292482	0.013829426	0.031792346	5	0	0
H2AFY2	0.2197607	0.214937384	0.00482336	0.257289154	0.01383116	0.031792346	13	0	0
SH2D2A	0.2499801	0.276222098	-0.02624196	-0.15768694	0.013848554	0.031822426	7	0	0
EXOC4	0.3386086	0.35388921	-0.0152806	-0.01320636	0.01384662	0.031822426	13	0	0
TMEM91	0.4671135	0.452480208	0.014633294	0.023524988	0.013849568	0.031822426	5	0	0
SH3PXD2A	0.2746477	0.271025155	0.003622511	0.140765553	0.013860567	0.031843619	6	0	0
CPZ	0.4300319	0.451315624	-0.0212837	0.117883103	0.013877876	0.031879302	5	0	0
DNAJC14	0.1718784	0.142776103	0.029102315	0.184732504	0.013899402	0.03192466	6	0	0
PITX2	0.5828772	0.575467018	0.007410179	0.038093308	0.013907229	0.031938547	7	0	0
VPS25	0.3415612	0.341036637	0.000524606	0.038882108	0.013924647	0.031974455	8	0	0
KIAA0895L	0.1992583	0.212467349	-0.013209	-0.07621416	0.013926939	0.031975625	11	0	0
PCDHGB1	0.3092956	0.289331194	0.019964356	0.29111217	0.013941985	0.032006073	13	0	0
ECHDC3	0.1295754	0.082956522	0.046618907	0.736613774	0.013958462	0.032039798	6	0	0
RAB3D	0.0642416	0.058617714	0.005623851	0.077837884	0.013966756	0.03205344	9	0	0
POP4	0.7106104	0.788061119	-0.07745072	-0.14727977	0.01396798	0.03205344	1	0	0
MAMDC4	0.663878	0.677550722	-0.01367269	-0.01503489	0.013974939	0.032063991	6	0	0
TNFAIP2	0.1498973	0.098411839	0.051485496	1.006830396	0.013976153	0.032063991	5	0	0
PRNP	0.2199331	0.236555609	-0.01662254	-0.10371906	0.013991667	0.032095478	10	0	0
UFL1	0.2215931	0.240605448	-0.01901234	-0.00857872	0.013997638	0.032105069	5	0	0
SEC61A2	0.2401094	0.235623692	0.004485743	0.093785242	0.014007818	0.032120203	8	0	0
POLR2F	0.3816222	0.423631616	-0.04200941	-0.10362124	0.014007289	0.032120203	7	0	0
APOBEC2	0.6966557	0.694446456	0.002209221	0.013151182	0.014027418	0.032158122	6	0	0
SURF2	0.1244788	0.141351943	-0.01687314	-0.08877998	0.014029082	0.032158122	8	0	0
SLC17A9	0.374537	0.360895653	0.013641298	0.161398764	0.014029734	0.032158122	10	0	0
YBEY	0.2124935	0.22885829	-0.01636477	-0.06306835	0.014032832	0.032161113	9	0	0
GCC2	0.2799482	0.298752752	-0.01880452	-0.09539835	0.014046028	0.032187245	6	0	0
BRAT1	0.3254195	0.346780204	-0.02136073	-0.00699388	0.014051342	0.032195309	8	0	0
GEMIN5	0.1323145	0.155015844	-0.02270137	-0.07988603	0.014059251	0.032209315	11	0	0
DBN1	0.8173329	0.841869193	-0.02453629	-0.04340245	0.01407229	0.032235068	5	0	0
HTR3E	0.822659	0.85362418	-0.03096518	-0.05227536	0.014075594	0.03223852	3	0	0
C6orf48	0.2472334	0.249907386	-0.00267399	0.06909237	0.014084636	0.032255111	19	0	0
PLCB4	0.0987759	0.070794263	0.027981623	0.418483033	0.014101671	0.032290001	11	0	0
NEU4	0.7617356	0.791760197	-0.03002459	-0.0537349	0.014103484	0.03229003	10	0	0
ANKRD6	0.3820355	0.384971603	-0.00293609	0.063783289	0.014107267	0.032294568	5	0	0
TMX4	0.0663131	0.086067551	-0.01975448	-0.01221823	0.014129533	0.032341413	11	0	0
JAKMIP1	0.2762231	0.30009098	-0.02386784	0.099407492	0.014131779	0.032342426	7	0	0
LACC1	0.1798091	0.183796829	-0.00398776	0.129154139	0.014134326	0.032344127	15	0	0
AQP7	0.7689861	0.748718765	0.020267351	0.038032396	0.014148173	0.032371684	1	0	0
TECTA	0.8212066	0.847747005	-0.02654036	-0.04622097	0.014162508	0.032400349	3	0	0
PMP22	0.6986986	0.705273866	-0.0065753	-0.0139385	0.014171438	0.032416645	7	0	0
HR	0.3980471	0.37560013	0.022446989	0.273324785	0.014178673	0.032416657	5	0	0
RNF169	0.2454395	0.254431659	-0.00899216	0.04287773	0.014177504	0.032416657	9	0	0
SERF2	0.1692471	0.193836728	-0.02458958	-0.07338849	0.014177813	0.032416657	12	0	0
NUDT21	0.0936493	0.106186041	-0.01253671	-0.164726	0.014174694	0.032416657	1	0	0
TPMT	0.042296	0.041448215	0.000847819	0.02698233	0.01419668	0.03245369	8	0	0
KCNV1	0.2631088	0.283964628	-0.02085585	0.043481126	0.014203251	0.032464575	10	0	0
ZNF432	0.1152298	0.078946192	0.036283581	0.601615612	0.014209589	0.032470784	6	0	0
ZNF841	0.1152298	0.078946192	0.036283581	0.601615612	0.014209589	0.032470784	6	0	0
NMUR1	0.3342392	0.326576894	0.007662286	0.426487686	0.014211648	0.032471352	10	0	0
COQ6	0.3188101	0.335128081	-0.01631794	-0.00893663	0.01422709	0.032502495	8	0	0
COL4A3BP	0.120042	0.131207621	-0.01116564	0.049150439	0.014251228	0.032553493	9	0	0
C9orf47	0.4492845	0.446631713	0.002652738	0.144005442	0.014257599	0.032563899	13	0	0
PUS10	0.1388275	0.154356939	-0.01552948	-0.01347028	0.014264326	0.032570968	10	0	0
HSPA4L	0.3621827	0.376621941	-0.01443928	-0.00434527	0.014264291	0.032570968	9	0	0
LCE4A	0.5453328	0.596316803	-0.05098399	-0.12276566	0.014271035	0.03258214	6	0	0
CREB3L1	0.2882391	0.261539244	0.026699835	0.255046278	0.014283555	0.032598275	10	0	0

CAMKK1	0.3980775	0.339860921	0.058216627	0.222061812	0.014280208	0.032598275	1	0	0
GRIK1	0.1773925	0.138866618	0.038525837	0.347881612	0.014283469	0.032598275	12	0	0
PALM3	0.347928	0.385275645	-0.03734768	-0.1756646	0.014287983	0.032604233	9	0	0
NFATC2	0.1545268	0.124376809	0.030149986	0.347099837	0.014302107	0.03263231	11	0	0
GLRB	0.369886	0.344499363	0.025386685	0.252629093	0.014304847	0.03263441	10	0	0
APOBR	0.5797211	0.611302384	-0.03158126	-0.08092167	0.014313373	0.032649709	8	0	0
KCTD15	0.1191334	0.084225602	0.034907806	0.262755465	0.014338445	0.03270274	5	0	0
MYOC	0.7905336	0.759760885	0.030772702	0.056551569	0.014340286	0.03270278	1	0	0
ORM2	0.7548971	0.711584294	0.043312774	0.084097681	0.014344425	0.032708061	1	0	0
ZNF777	0.2035844	0.231132179	-0.02754783	-0.10165668	0.014350818	0.032718479	8	0	0
MRPS5	0.908861	0.917187872	-0.00832683	-0.01301829	0.014354446	0.03272259	5	0	0
SLC22A17	0.294322	0.291027222	0.003294828	0.121937796	0.014368661	0.032747406	9	0	0
LCMT2	0.1738528	0.19529294	-0.02144009	-0.04976243	0.014368983	0.032747406	12	0	0
AP2M1	0.4450257	0.458740112	-0.01371439	-0.00986755	0.014386831	0.032779751	10	0	0
SRC	0.6610803	0.681621332	-0.02054102	-0.04669691	0.014386266	0.032779751	7	0	0
SMG5	0.1956508	0.238021088	-0.04237027	-0.06156964	0.014388844	0.032780174	12	0	0
FAM169B	0.7682368	0.761001537	0.007235221	0.019213724	0.014401222	0.03280004	7	0	0
CHMP4B	0.1660117	0.192984693	-0.02697295	-0.08475758	0.014400153	0.03280004	9	0	0
ZC3H14	0.3194752	0.346784706	-0.02730952	-0.09220811	0.014407933	0.03281116	14	0	0
MGP	0.4315444	0.391397579	0.040146787	0.185765297	0.014414308	0.032821508	6	0	0
PRPF31	0.1944551	0.216673009	-0.02221786	-0.00757847	0.014418538	0.032826973	19	0	0
NDUFV1	0.4097832	0.389231778	0.020551434	0.048605583	0.014434035	0.032858084	12	0	0
NANOS1	0.2255863	0.216276264	0.009310005	0.150273971	0.014440358	0.032868307	6	0	0
OAS2	0.2820833	0.300005966	-0.01792266	-0.09218002	0.014481122	0.032952727	10	0	0
ZFP41	0.0865593	0.065066835	0.02149248	0.191429025	0.014489744	0.032966808	11	0	0
C14orf132	0.097689	0.080315479	0.017373497	0.26829696	0.014490985	0.032966808	13	0	0
NACAD	0.229302	0.201624033	0.027677961	0.289296088	0.014497044	0.032972227	7	0	0
KRT83	0.6365445	0.620761508	0.015782961	0.050921405	0.014497038	0.032972227	5	0	0
MSS51	0.8618508	0.879345431	-0.01749462	-0.02969109	0.014506676	0.032989951	3	0	0
CHRNB2	0.3289185	0.35752788	-0.02860936	-0.02543801	0.014514661	0.033003925	7	0	0
RNF223	0.4705601	0.505349162	-0.03478907	-0.08708271	0.014524885	0.033011801	4	0	0
ZFP69B	0.2916516	0.28646225	0.005189331	0.373717077	0.014527328	0.033011801	7	0	0
SCG2	0.2949161	0.312044201	-0.01712813	-0.00823103	0.014525898	0.033011801	8	0	0
KIAA1191	0.24155	0.25792436	-0.01637437	-0.05196605	0.014522881	0.033011801	15	0	0
LAT2	0.8396875	0.87541305	-0.0357256	-0.05941838	0.014522106	0.033011801	1	0	0
SLC35E4	0.0969699	0.093169832	0.00380007	0.180942453	0.014531799	0.033017778	8	0	0
NANOG	0.4587834	0.469705832	-0.01092245	-0.01729268	0.01454123	0.033035021	3	0	0
BCL2L10	0.5263006	0.547104051	-0.02080344	-0.05521269	0.014545164	0.033039775	10	0	0
TNNC1	0.3375891	0.318517669	0.019071393	0.04261562	0.014551712	0.033042094	11	0	0
NISCH	0.3375891	0.318517669	0.019071393	0.04261562	0.014551712	0.033042094	11	0	0
OVOL2	0.2011949	0.188906664	0.012288253	0.282778836	0.014550424	0.033042094	7	0	0
TCHH	0.7288945	0.774187599	-0.04529311	-0.08903281	0.014556485	0.03304705	6	0	0
WDR34	0.2853975	0.294042897	-0.00864544	-0.02155979	0.014557579	0.03304705	6	0	0
KRT35	0.7886949	0.81391474	-0.02521988	-0.04679545	0.014575791	0.033080019	6	0	0
UNK	0.106922	0.131220088	-0.0242981	-0.11243096	0.01457544	0.033080019	14	0	0
WNT6	0.1161435	0.101848318	0.014295155	0.264825081	0.014585546	0.033091412	11	0	0
TNFAIP8L1	0.1167965	0.117053826	-0.00025732	0.216067474	0.014584584	0.033091412	15	0	0
SCAND1	0.6448207	0.692832265	-0.04801153	-0.10208041	0.014586346	0.033091412	1	0	0
HNRNPF	0.1022132	0.113934461	-0.01172123	-0.02843485	0.014589634	0.033094684	12	0	0
GAP43	0.439803	0.465720863	-0.02591786	-0.08006253	0.014597102	0.033103252	5	0	0
C17orf100	0.0703462	0.077550829	-0.00720459	-0.07416214	0.014595495	0.033103252	14	0	0
KCNRG	0.7925758	0.796379558	-0.00380377	-0.00451206	0.014600268	0.033106246	4	0	0
EIF5	0.3977041	0.444616477	-0.04691234	-0.07517977	0.014611104	0.033126628	4	0	0
NKX6-1	0.1040596	0.081950469	0.022109147	0.417709977	0.014631947	0.033169691	14	0	0
ZNF594	0.214466	0.214928103	-0.00046208	0.021524307	0.014663084	0.033231877	13	0	0
PARP3	0.0906969	0.103273686	-0.01257679	-0.07961129	0.014673441	0.033251147	12	0	0
ADH4	0.8200139	0.835941923	-0.01592798	-0.02906058	0.014687693	0.033275035	3	0	0
TMEM229A	0.2465701	0.221585744	0.024984391	0.328578982	0.014686	0.033275035	8	0	0
CTSV	0.084127	0.069362298	0.014764752	0.226697144	0.014691427	0.033279292	6	0	0
RAD18	0.3090582	0.308813205	0.000245031	0.053223489	0.014696898	0.03328498	7	0	0
PDCL	0.2552384	0.265052378	-0.00981401	0.038185766	0.014697649	0.03328498	7	0	0
LCE5A	0.6728615	0.724750187	-0.05188867	-0.10607886	0.01470371	0.033290952	7	0	0
DPP8	0.1511918	0.16727144	-0.01607961	-0.07599775	0.014705855	0.033290952	16	0	0

PTTG1IP	0.0490899	0.056859439	-0.0077695	-0.06143708	0.014705679	0.033290952	8	0	0
ADAMTS9	0.0650196	0.051308257	0.013711306	0.326504883	0.014717932	0.033314088	9	0	0
SRPRB	0.7143832	0.692160509	0.022222644	0.064520946	0.014720148	0.033314898	10	0	0
ARHGAP17	0.2164044	0.218427267	-0.00202291	-0.02779795	0.014727375	0.03332705	7	0	0
CCDC90B	0.205208	0.241868237	-0.03666027	-0.09564965	0.014735276	0.033340723	15	0	0
SKA1	0.1407051	0.149750928	-0.00904583	-0.02166878	0.014750744	0.033371512	7	0	0
UTP20	0.1692399	0.192382955	-0.02314306	-0.03669001	0.014763939	0.033397152	8	0	0
HOXA6	0.1577602	0.115455136	0.042305034	0.562015868	0.014778837	0.033426636	12	0	0
CGRRF1	0.0941464	0.110371026	-0.01622466	-0.11886229	0.014781909	0.033429368	10	0	0
LRRC27	0.142823	0.165462126	-0.02263909	-0.03471462	0.014785149	0.033432481	10	0	0
NDST1	0.1913903	0.188270171	0.00312016	0.082589475	0.014796263	0.033453394	6	0	0
KCNH6	0.3406979	0.358595575	-0.01789766	0.048534344	0.014803226	0.033464919	7	0	0
RPF1	0.3725566	0.407538928	-0.03498235	-0.05860199	0.014810016	0.033465318	9	0	0
TMEM238	0.0482094	0.062522458	-0.01431305	-0.07737131	0.01480753	0.033465318	12	0	0
LSS	0.0927662	0.097452963	-0.00468677	-0.00023006	0.014810866	0.033465318	5	0	0
GRID1	0.1225458	0.098648525	0.023897291	0.29709	0.014814375	0.03346903	10	0	0
CNOT8	0.1216282	0.104386964	0.01724121	0.017293189	0.014816506	0.033469629	6	0	0
MOGAT3	0.3691826	0.331039293	0.038143322	0.314178481	0.014824768	0.033484075	12	0	0
SEC23IP	0.3014043	0.320630321	-0.01922602	-0.04869166	0.014827552	0.033486147	9	0	0
PFKFB3	0.2618136	0.284670941	-0.02285734	-0.01267046	0.014832153	0.033488105	10	0	0
FCF1	0.219201	0.233744522	-0.01454356	0.017690044	0.014830661	0.033488105	16	0	0
IFNG	0.7345925	0.779820945	-0.04522847	-0.08744293	0.014849581	0.033523233	5	0	0
NRBP2	0.6748538	0.693052039	-0.01819821	-0.02903506	0.014861232	0.033545314	6	0	0
KRTAP10-6	0.4647969	0.477051595	-0.01225467	-0.03521245	0.014868811	0.033558196	5	0	0
MOB1B	0.0842711	0.098432142	-0.01416105	-0.12702137	0.014881503	0.033582617	4	0	0
C6orf203	0.1757849	0.183227169	-0.00744223	-0.01288152	0.014886041	0.033584559	6	0	0
PPIF	0.2486633	0.258727086	-0.01006377	-0.00895669	0.014887981	0.033584559	11	0	0
C19orf57	0.0520115	0.048011266	0.004000245	0.096425202	0.0148875	0.033584559	4	0	0
C2orf73	0.3546663	0.305086435	0.04957985	0.184603708	0.014895893	0.033597374	3	0	0
HEY2	0.1803212	0.162145414	0.01817576	0.381536222	0.014897408	0.033597374	8	0	0
PLVAP	0.4512259	0.421703162	0.029522724	0.108091062	0.014912717	0.03362767	10	0	0
DCP1B	0.2928444	0.298454627	-0.00561018	0.238971289	0.014915887	0.03363059	14	0	0
CAMSAP1	0.2634141	0.286868476	-0.02345433	-0.03657161	0.014920761	0.033637352	6	0	0
APOB	0.5430754	0.580362211	-0.03728683	-0.1014125	0.014926917	0.033647001	13	0	0
NUDT4	0.247969	0.255836843	-0.00786784	-0.09745256	0.014931273	0.03365259	7	0	0
NCR1	0.4561688	0.486062322	-0.02989349	-0.08973879	0.01494647	0.03368261	6	0	0
EFCAB2	0.1935388	0.159288391	0.034250368	0.156259077	0.014957648	0.033702522	13	0	0
SNRK	0.1977058	0.186906864	0.010798972	0.092238423	0.014959064	0.033702522	8	0	0
IRF7	0.2580801	0.239571097	0.018508979	0.655202056	0.014967868	0.033713894	13	0	0
RFX4	0.1310924	0.101375945	0.029716471	0.445749936	0.014967871	0.033713894	17	0	0
CELA3B	0.615374	0.643578099	-0.02820406	-0.06364032	0.014983892	0.033745741	1	0	0
SUSD3	0.4653135	0.461721122	0.003592345	0.28642628	0.014988678	0.033752281	4	0	0
C20orf27	0.0576823	0.067186665	-0.00950433	-0.05001199	0.015003194	0.033776488	9	0	0
IDO2	0.7356187	0.766866078	-0.03124738	-0.05523448	0.01500679	0.033780344	5	0	0
KRT84	0.8042592	0.829187033	-0.02492783	-0.04135337	0.015015207	0.033795049	5	0	0
PKHD1L1	0.4028893	0.401916302	0.000973007	0.14249866	0.01502339	0.033809225	11	0	0
PPT1	0.3597561	0.372496209	-0.01274012	0.058536399	0.015032982	0.033826567	5	0	0
NPPA	0.639504	0.632284847	0.007219117	0.032688335	0.015039324	0.033836592	10	0	0
MYBL2	0.1450808	0.155753706	-0.01067286	-0.08719716	0.01505694	0.033871977	3	0	0
GGT7	0.2228436	0.233210972	-0.01036741	0.036968827	0.015064046	0.033883714	13	0	0
LAD1	0.1816865	0.186371643	-0.00468519	0.090334062	0.015076933	0.033908447	11	0	0
CREM	0.2188432	0.238325057	-0.01948182	-0.00434658	0.015080039	0.033911182	7	0	0
SNRPB2	0.2142387	0.234272884	-0.02003417	-0.01987652	0.015084669	0.033917339	13	0	0
ASL	0.2048022	0.219392901	-0.01459068	0.022052755	0.015086772	0.033917818	6	0	0
WDR4	0.0843727	0.098177003	-0.0138043	-0.03144139	0.015091637	0.033924502	11	0	0
LACTB	0.3865655	0.370191321	0.016374221	0.061723677	0.015097049	0.033932415	6	0	0
ABCA8	0.5789614	0.524409827	0.054551615	0.139078482	0.015112297	0.033958177	3	0	0
PPP1R27	0.7750178	0.796928043	-0.02191023	-0.04478299	0.015111494	0.033958177	10	0	0
DDX46	0.0351236	0.036569631	-0.00144605	-0.01635034	0.015114733	0.033959394	10	0	0
CSNK2B	0.170273	0.145136585	0.02513641	0.118498598	0.015119452	0.033965743	34	0	0
MTRR	0.3842277	0.359634099	0.02459359	0.41061606	0.015123533	0.033970657	12	0	0
KRT1	0.6332738	0.676194047	-0.04292024	-0.0751273	0.015134703	0.033991489	6	0	0
GALP	0.5492376	0.517118355	0.032119271	0.110899788	0.01514912	0.034016204	4	0	0

MFNG	0.3280633	0.313370733	0.014692518	0.091865942	0.0151495	0.034016204	6	0	0
FZD9	0.3390567	0.365343965	-0.02628731	0.054147921	0.015178542	0.034072883	7	0	0
OR13C8	0.7632619	0.80088554	-0.03762366	-0.06831485	0.015177973	0.034072883	4	0	0
RCSD1	0.178845	0.14756901	0.031275999	0.557044356	0.015182077	0.034076554	12	0	0
NBEA	0.0666557	0.073910289	-0.00725455	-0.12210129	0.015205585	0.034125047	8	0	0
CNOT1	0.0406431	0.043433147	-0.00279003	-0.055693	0.015214935	0.034141758	5	0	0
C8orf88	0.2571357	0.231655405	0.025480248	0.553319241	0.015219802	0.034144135	9	0	0
PGAP2	0.042666	0.04229875	0.000367243	0.01922903	0.015219094	0.034144135	19	0	0
IL1R1	0.2537768	0.215193834	0.038582939	0.38218229	0.015240967	0.034187342	2	0	0
ABHD14B	0.1271055	0.135095086	-0.00798957	-0.07205272	0.015250216	0.034203811	10	0	0
CNNM1	0.4937127	0.515283054	-0.0215704	-0.06973823	0.015256348	0.034213285	10	0	0
POLDIP3	0.0581416	0.0534301	0.004711513	0.091036228	0.01527756	0.034256573	10	0	0
ZCCHC4	0.211582	0.209252472	0.002329514	-0.02776157	0.015288516	0.034276854	6	0	0
SRRM2	0.413651	0.437978133	-0.02432712	-0.09336558	0.015294407	0.034285774	17	0	0
GPATCH2L	0.2272867	0.242028005	-0.01474135	-0.00241639	0.015302606	0.034299867	13	0	0
ZNF622	0.1417232	0.157939195	-0.01621601	-0.05746135	0.015304905	0.034300734	11	0	0
SPIB	0.3870421	0.379646649	0.007395425	0.150736446	0.015312009	0.034312369	7	0	0
RAN	0.2024321	0.21688079	-0.01444869	-0.01381763	0.015331594	0.034351965	13	0	0
MEIS3	0.1955785	0.166430543	0.02914799	0.268989333	0.015342611	0.034372355	4	0	0
C1orf216	0.4262885	0.434435107	-0.0081466	0.129879639	0.015355587	0.034393639	11	0	0
CDC42EP4	0.1487516	0.159216867	-0.01046523	0.022355917	0.015355947	0.034393639	12	0	0
LRR1	0.1888846	0.204546069	-0.01566144	0.017942224	0.015369479	0.034419649	10	0	0
DDX50	0.1096445	0.110246168	-0.00060167	0.052902251	0.01537596	0.034429866	11	0	0
ASCL2	0.4341168	0.428936401	0.00518041	0.125110117	0.015394417	0.034464166	31	0	0
AQP6	0.1292253	0.142984709	-0.01375942	-0.04736428	0.015395861	0.034464166	7	0	0
STARD9	0.1102549	0.120848484	-0.01059356	0.050715936	0.015397043	0.034464166	8	0	0
IGFLR1	0.6521119	0.700078958	-0.04796701	-0.12158974	0.015399147	0.034464574	9	0	0
ZSCAN21	0.137984	0.148848707	-0.01086468	0.009741112	0.015401202	0.034464872	8	0	0
GDI2	0.5823526	0.608518651	-0.02616607	-0.06236095	0.015408237	0.034476314	1	0	0
PKD2L1	0.5957761	0.632352663	-0.03657658	-0.08243381	0.015431109	0.034523184	7	0	0
HERPUD1	0.0224446	0.024577275	-0.0021327	-0.06462826	0.015443435	0.034546451	4	0	0
ZBTB38	0.7802851	0.801080398	-0.02079527	-0.03576819	0.015449749	0.034556265	4	0	0
MOV10	0.7009339	0.708266331	-0.00733241	0.028284106	0.015453979	0.034561416	4	0	0
DND1	0.8847387	0.884254438	0.000484275	0.003610701	0.015460085	0.034570763	10	0	0
ATP6V0E1	0.2276737	0.22605995	0.001613798	0.027136568	0.015473126	0.034595611	13	0	0
SLC6A20	0.2660067	0.25553154	0.010475154	0.328181549	0.015484274	0.034608817	11	0	0
TNFSF12	0.1001849	0.076949836	0.023235039	0.4235585	0.015484821	0.034608817	9	0	0
TNFSF12-TN	0.1001849	0.076949836	0.023235039	0.4235585	0.015484821	0.034608817	9	0	0
TMC1	0.8800646	0.89242597	-0.01236141	-0.02010226	0.015496543	0.034630699	3	0	0
STEAP4	0.3318209	0.328783456	0.003037489	0.182213513	0.015501043	0.034633741	13	0	0
PHYH	0.2595324	0.287750634	-0.02821824	-0.05553894	0.015501766	0.034633741	7	0	0
RAB27A	0.087864	0.107955919	-0.0200919	-0.10902749	0.015531548	0.034695958	11	0	0
OBSCN	0.5800629	0.614023878	-0.03396096	-0.08642482	0.015537349	0.034704594	11	0	0
TTI2	0.0801565	0.057675096	0.022481445	0.355168283	0.0155496	0.034718988	9	0	0
PHF19	0.1257832	0.145482996	-0.0196998	-0.00014119	0.015549595	0.034718988	7	0	0
STIP1	0.2606069	0.279764882	-0.01915799	-0.04667039	0.015547542	0.034718988	9	0	0
SDC4	0.3069657	0.3253391	-0.01837344	-0.02812535	0.015553233	0.034722776	9	0	0
COL16A1	0.2075614	0.162510155	0.045051256	0.362010778	0.015557289	0.03472751	13	0	0
SENP6	0.2358453	0.267005912	-0.03116064	-0.08003176	0.015569434	0.034750295	8	0	0
BTAF1	0.2195485	0.221599511	-0.00205101	0.044165385	0.015574819	0.034757989	9	0	0
UAP1	0.2262481	0.22366684	0.002581211	0.064538265	0.015606631	0.034824649	10	0	0
LRP5L	0.8293256	0.853055757	-0.02373012	-0.04022311	0.015618358	0.034846482	1	0	0
ARFGEF2	0.08786	0.120063122	-0.03220308	-0.14312766	0.015648634	0.034909688	5	0	0
L3MBTL1	0.2929356	0.274276138	0.018659445	0.413673046	0.015658472	0.034927293	10	0	0
C12orf29	0.8451575	0.864533743	-0.01937624	-0.03452273	0.015671289	0.034951534	3	0	0
SOD1	0.2168683	0.230244564	-0.01337629	-0.06842884	0.015680918	0.034968662	10	0	0
DOLK	0.1108901	0.116240048	-0.00534994	0.063070561	0.015688783	0.034981852	11	0	0
LONRF2	0.2126925	0.191277463	0.021415032	0.538929824	0.015695423	0.034992307	5	0	0
TRNAU1AP	0.1996689	0.20098491	-0.00131597	0.065492575	0.015702822	0.035004451	11	0	0
SSR3	0.1054927	0.126455508	-0.02096276	-0.08561188	0.015714396	0.035025898	14	0	0
TMEM221	0.1206087	0.124646127	-0.00403743	0.266154581	0.015719405	0.035032709	10	0	0
EPC2	0.0507156	0.060723528	-0.01000792	-0.12073133	0.015741479	0.035077546	4	0	0
LCN8	0.4959837	0.502800619	-0.00681695	0.026912887	0.01574377	0.035078293	7	0	0

COL9A3	0.2413122	0.215489796	0.025822443	0.357563097	0.015765113	0.035121485	28	0	0
NEUROD2	0.6389834	0.681721447	-0.04273804	-0.09322567	0.015779263	0.035148642	2	0	0
PRSS12	0.1502838	0.143983804	0.006300042	0.05882654	0.015790791	0.035169953	9	0	0
SLC25A22	0.2779848	0.285370085	-0.00738532	-0.06744651	0.015792759	0.035169968	10	0	0
DCAF8	0.2874046	0.33655128	-0.0491467	-0.09744024	0.015807786	0.035199063	11	0	0
S1PR3	0.4094864	0.395798065	0.013688348	0.233552662	0.015812381	0.035204925	12	0	0
SHISA3	0.1490831	0.088740929	0.060342146	0.986657532	0.015826446	0.035231865	8	0	0
CD274	0.1747242	0.200497033	-0.02577287	-0.11194173	0.015829439	0.035234154	5	0	0
NR2C2	0.0395746	0.035458555	0.004116074	0.094340465	0.015833742	0.03523936	7	0	0
CDS1	0.1782778	0.185373615	-0.00709581	-0.14660378	0.015856035	0.035284597	10	0	0
NPNT	0.189708	0.200018144	-0.01031013	-0.13618466	0.01585955	0.035285033	5	0	0
SLCO4C1	0.1175669	0.121937955	-0.00437107	0.001608315	0.015860166	0.035285033	12	0	0
SLX4	0.2648019	0.295324263	-0.03052234	-0.0274626	0.015879236	0.035323079	3	0	0
HSPB11	0.0726509	0.069482145	0.003168757	0.068156468	0.015904469	0.035374821	13	0	0
LETMD1	0.1511482	0.170622459	-0.01947425	-0.04409332	0.0159083	0.035378955	11	0	0
RABGAP1L	0.2787356	0.286736582	-0.00800094	0.037580671	0.015911356	0.035381365	6	0	0
LRRC4	0.1773024	0.132365274	0.044937153	0.7563925	0.015916255	0.035387871	5	0	0
RNF180	0.22646	0.203864858	0.022595143	0.333170947	0.015919929	0.035391651	10	0	0
SNRPD2	0.0416448	0.047482003	-0.00583725	-0.04280656	0.015931064	0.035412016	15	0	0
NLRC3	0.4192246	0.425845012	-0.00662042	0.010484462	0.015941589	0.035431022	7	0	0
THSD7A	0.2583892	0.246332649	0.012056578	0.230417325	0.015999408	0.035555122	11	0	0
LSM4	0.0890957	0.077608376	0.011487295	0.021679137	0.016005427	0.03556409	9	0	0
SOS2	0.1153214	0.091685346	0.023636103	0.071645814	0.016010441	0.035570825	7	0	0
DERL1	0.2071369	0.234696311	-0.02755945	-0.0570274	0.016013343	0.035572865	10	0	0
CR1L	0.2565589	0.222799753	0.033759189	0.493209442	0.016018731	0.035580429	9	0	0
IGSF3	0.5127306	0.50928497	0.003445623	0.020570052	0.016023787	0.035583202	5	0	0
NGRN	0.0665421	0.084323073	-0.01778097	-0.11943053	0.016025931	0.035583202	8	0	0
FAM207A	0.2738505	0.308179723	-0.03432919	-0.07443071	0.016024158	0.035583202	8	0	0
SLIT1	0.2248696	0.218341147	0.006528455	0.285114912	0.016029696	0.035587154	13	0	0
USP15	0.1463725	0.16597039	-0.01959791	-0.05390869	0.016037286	0.0355996	16	0	0
SURF1	0.2287794	0.249145871	-0.02036649	-0.09991192	0.016052186	0.035628264	8	0	0
DHFR	0.2691631	0.288455486	-0.01929234	-0.03528511	0.016060286	0.035641832	6	0	0
NCKAP5	0.4176307	0.417664094	-3.34E-05	0.035008202	0.016082049	0.035685714	8	0	0
MANSC1	0.1989537	0.221578309	-0.02262456	0.110941731	0.016086866	0.035691987	8	0	0
RFC1	0.2926521	0.303204811	-0.01055268	-0.02723486	0.016099233	0.035715008	10	0	0
GLYATL3	0.7410665	0.76672336	-0.02565681	-0.04990767	0.016104441	0.035720661	3	0	0
TMEM151A	0.1654456	0.13124226	0.034203313	0.429463752	0.016105764	0.035720661	7	0	0
ZNF20	0.083099	0.086846359	-0.00374736	0.149808428	0.016108673	0.035722695	17	0	0
PIGT	0.2890872	0.316527148	-0.02743994	-0.08843102	0.016112654	0.035727107	9	0	0
ERN1	0.2914556	0.254070697	0.037384884	0.193419545	0.016116994	0.035732312	5	0	0
PSORS1C2	0.7438839	0.765617565	-0.02173369	-0.03771761	0.016122066	0.03573914	3	0	0
TEX2	0.2102873	0.234671083	-0.02438378	-0.10612839	0.016136612	0.035766963	8	0	0
LRIG3	0.2387867	0.245274638	-0.0064879	0.090995204	0.016141555	0.035773499	15	0	0
NELFA	0.0419785	0.042091054	-0.00011253	0.107389619	0.016144954	0.035776611	12	0	0
EPHX4	0.3008437	0.301544076	-0.00070033	0.23872175	0.016156466	0.035788857	13	0	0
GEMIN4	0.8346495	0.85864235	-0.02399284	-0.04083843	0.016155973	0.035788857	3	0	0
ASXL1	0.1791939	0.137264834	0.041929089	0.165936712	0.016155127	0.035788857	6	0	0
DNAJB14	0.1909911	0.215668904	-0.02467779	0.062346237	0.016160906	0.035794271	7	0	0
BVES	0.2664552	0.25817985	0.008275346	0.3603253	0.016171605	0.035809123	13	0	0
SNX30	0.1323659	0.145730276	-0.01336439	0.007529764	0.016170597	0.035809123	11	0	0
ABHD17A	0.3899557	0.390716356	-0.0007607	0.030971029	0.016179442	0.035822056	8	0	0
FRG1	0.0871138	0.073454684	0.013659132	0.188062396	0.016182594	0.035824613	2	0	0
VIPAS39	0.0460251	0.046836534	-0.00081148	-0.00976119	0.016191877	0.03584074	10	0	0
FEV	0.3459331	0.321744852	0.024188246	0.262487454	0.016209178	0.035869105	8	0	0
P2RY1	0.0700433	0.069710958	0.000332381	0.098866705	0.016209803	0.035869105	9	0	0
HBEGF	0.2315393	0.243218672	-0.01167933	-0.03571949	0.016210692	0.035869105	4	0	0
ABCD4	0.2698046	0.279451076	-0.00964643	0.022005508	0.016226772	0.035900257	10	0	0
NUP62	0.2656697	0.256892305	0.008777417	0.037286406	0.01623892	0.035922702	13	0	0
SYF2	0.0567883	0.047027392	0.009760903	0.186938995	0.016245381	0.035932563	11	0	0
TEAD2	0.087792	0.080222119	0.007569882	0.125438314	0.016248408	0.035934827	6	0	0
LCE3C	0.3985073	0.485569337	-0.08706202	-0.27872502	0.016263305	0.035963338	1	0	0
CEP41	0.8129664	0.834619441	-0.02165303	-0.03786135	0.016271719	0.03597751	3	0	0
C11orf52	0.8132417	0.838130266	-0.02488859	-0.04302127	0.016277247	0.035985295	4	0	0

CYP2D6	0.6316211	0.658003379	-0.02638229	-0.06128368	0.016283464	0.035994603	4	0	0
PFDN6	0.1837858	0.19890148	-0.01511563	-0.04903413	0.016286476	0.035996825	32	0	0
RABL6	0.2278457	0.246443294	-0.01859764	-0.02283868	0.016292422	0.036004374	7	0	0
TPST2	0.7384712	0.778977155	-0.04050598	-0.08252873	0.016293906	0.036004374	3	0	0
SHB	0.0614571	0.058916599	0.002540453	0.030861458	0.016299851	0.036013073	9	0	0
LRRC8A	0.1064319	0.12122601	-0.01479412	-0.03496374	0.016320024	0.036048763	9	0	0
SPDYA	0.8252252	0.851999296	-0.02677409	-0.04446027	0.016349746	0.036105521	3	0	0
GPD2	0.4102726	0.412358861	-0.00208626	0.038530525	0.016348563	0.036105521	10	0	0
OTOG	0.460528	0.477234123	-0.01670616	-0.03104	0.016360419	0.036124644	10	0	0
DLX1	0.2133603	0.161218794	0.052141497	0.515898853	0.016363702	0.036127444	10	0	0
GPR156	0.3269603	0.277403953	0.049556314	0.378906706	0.016372605	0.036139753	7	0	0
ASTN2	0.2097155	0.206554375	0.003161132	0.3615232	0.016374435	0.036139753	9	0	0
CATSPER1	0.7931188	0.828877952	-0.03575911	-0.07045812	0.016375322	0.036139753	5	0	0
ZNF512B	0.1549464	0.122118147	0.032828246	0.303669998	0.016383386	0.036153102	15	0	0
ZNF239	0.3164641	0.324984317	-0.00852022	0.136128238	0.016390594	0.036160816	9	0	0
RASGRP1	0.6261652	0.614882462	0.011282727	0.101890279	0.01639293	0.036160816	8	0	0
DDX19A	0.1516927	0.162327177	-0.01063445	0.00866823	0.016392795	0.036160816	9	0	0
RANBP9	0.1347573	0.127882554	0.006874753	0.145656385	0.016399979	0.036171917	8	0	0
AIF1L	0.4117069	0.436060302	-0.02435338	0.095318385	0.016409114	0.036187614	7	0	0
NSMCE2	0.1152411	0.12852926	-0.01328815	-0.06131035	0.016429326	0.036223281	11	0	0
EIF2B1	0.1719102	0.185827502	-0.01391728	-0.05647951	0.016428837	0.036223281	16	0	0
HLA-F	0.3716795	0.402402222	-0.03072276	-0.0768354	0.016442349	0.036243083	6	0	0
AGTPBP1	0.0831126	0.090558547	-0.00744596	0.069709326	0.016440507	0.036243083	9	0	0
ELOVL5	0.089054	0.098809098	-0.00975506	-0.09817112	0.016445305	0.036245145	15	0	0
EPHA3	0.3133317	0.338940389	-0.02560867	-0.02153933	0.016455357	0.036262844	13	0	0
UIMC1	0.0478587	0.045640498	0.002218165	0.008570267	0.016466128	0.036282122	10	0	0
NINL	0.0887957	0.076862523	0.011933159	0.337224423	0.016472266	0.036291188	4	0	0
FKBP4	0.257616	0.233510226	0.024105776	0.238765858	0.016476453	0.036295955	15	0	0
CACUL1	0.1350345	0.15745213	-0.02241765	-0.05037883	0.016487611	0.036316074	11	0	0
ZNF214	0.1535317	0.129595416	0.023936299	0.383303898	0.016500118	0.03633916	20	0	0
MPZ	0.7208045	0.684812698	0.035991785	0.072861364	0.016504825	0.036343545	1	0	0
GDPD4	0.7085738	0.680604794	0.027969011	0.067183728	0.016506162	0.036343545	6	0	0
GIPC3	0.1018729	0.069835152	0.032037774	0.69927552	0.016513845	0.036355999	5	0	0
POMGNT1	0.7661143	0.769334182	-0.00321989	-0.00414837	0.016536284	0.036400932	3	0	0
SNAI3	0.0989501	0.10795262	-0.00900255	-0.07726404	0.016589764	0.036509693	7	0	0
KRT23	0.5822858	0.613616147	-0.03133038	-0.07135358	0.016589577	0.036509693	5	0	0
PDIA3	0.3586701	0.344028672	0.014641468	0.036504819	0.016596072	0.036519095	6	0	0
METTL5	0.3411444	0.364799925	-0.02365551	-0.03873072	0.016608132	0.036540763	13	0	0
ZNF707	0.2487418	0.27741015	-0.02866837	-0.04728934	0.016609993	0.036540763	6	0	0
C22orf42	0.6057603	0.644336472	-0.03857619	-0.08831846	0.016616415	0.036550408	5	0	0
ABLIM2	0.1391836	0.14339828	-0.00421467	0.11754276	0.016618828	0.036551232	7	0	0
PPARGC1B	0.1439246	0.161265232	-0.01734065	-0.08326544	0.016622373	0.036554546	9	0	0
NR1H3	0.3248498	0.320315088	0.004534667	-0.00715971	0.016626238	0.036558564	11	0	0
TCF7L1	0.0994961	0.110031857	-0.01053579	0.046657728	0.016629499	0.036561253	9	0	0
STYXL1	0.0910871	0.106502609	-0.01541555	-0.08978443	0.016644305	0.036571389	16	0	0
CDNF	0.09819	0.127104776	-0.02891473	-0.05693621	0.016644041	0.036571389	6	0	0
SLC7A10	0.30612	0.269938974	0.036181003	0.180292834	0.016638893	0.036571389	9	0	0
RGS19	0.1776766	0.1758986	0.001778021	0.138435984	0.016641454	0.036571389	12	0	0
PRR5	0.1343632	0.129048069	0.005315121	0.371139322	0.016637178	0.036571389	9	0	0
C8G	0.273144	0.290419999	-0.01727599	-0.05244002	0.016654076	0.036588377	6	0	0
RPL27	0.0352848	0.036371668	-0.0010869	-0.01671388	0.01667346	0.036626475	7	0	0
CLDN11	0.2188013	0.171575918	0.047225425	0.362189171	0.016680877	0.036638282	9	0	0
CAMK1	0.190846	0.181506626	0.009339411	0.142343187	0.016707746	0.036692446	12	0	0
TRIM36	0.3473564	0.319612566	0.027743814	0.541892216	0.016709629	0.036692446	14	0	0
MECR	0.1644011	0.181275048	-0.01687398	-0.07597245	0.01671894	0.036708398	14	0	0
FGF5	0.0689869	0.044362557	0.02462431	0.588204368	0.016732948	0.036730162	11	0	0
C16orf58	0.3345963	0.308946773	0.02564954	0.168472705	0.016730938	0.036730162	2	0	0
PRKAR1B	0.441361	0.43671293	0.004648073	-0.0251733	0.016737462	0.03673471	9	0	0
JAKMIP3	0.6326705	0.665472097	-0.03280159	-0.05769883	0.016739116	0.03673471	5	0	0
ERC1	0.2142351	0.173599829	0.040635266	0.168878133	0.016745326	0.036743843	8	0	0
GSAP	0.3608554	0.385829423	-0.02497405	-0.07970869	0.01675397	0.036758314	6	0	0
TUSC2	0.1401352	0.150317137	-0.01018193	-0.065857	0.016777171	0.036804715	13	0	0
PCDHB10	0.13279	0.100963006	0.031826992	0.413042923	0.016779629	0.036805604	6	0	0

DHX35	0.2041322	0.219344457	-0.01521223	-0.02577421	0.01678179	0.036805843	10	0	0
CLK2	0.1920463	0.196275845	-0.00422959	-0.11354697	0.016802433	0.036846612	3	0	0
NEIL1	0.4660677	0.482646769	-0.01657905	-0.00800875	0.0168079	0.036854094	13	0	0
INSM1	0.1138682	0.068797889	0.045070345	0.780689412	0.016810366	0.036854995	9	0	0
CCKAR	0.6772483	0.692525814	-0.01527748	-0.02081407	0.016814407	0.036857224	6	0	0
CCDC88B	0.5448535	0.587155259	-0.04230173	-0.10531976	0.016815492	0.036857224	5	0	0
F7	0.5728427	0.596731159	-0.02388847	-0.05229311	0.016828341	0.036880879	8	0	0
TFPI2	0.2665495	0.266040813	0.000508647	0.284247817	0.016844018	0.036910321	18	0	0
CPA6	0.27539	0.294054477	-0.01866449	0.141128075	0.01684589	0.036910321	3	0	0
ADAD2	0.786143	0.80527478	-0.01913175	-0.03225744	0.016855325	0.036926482	10	0	0
L3MBTL2	0.1453113	0.162283408	-0.01697216	-0.02875363	0.016875756	0.036966728	14	0	0
SHROOM3	0.3892449	0.416168656	-0.0269238	-0.00107615	0.016886671	0.036986119	7	0	0
PPIC	0.0399442	0.032187377	0.007756799	0.258825339	0.016909787	0.037032227	9	0	0
C9orf72	0.1863505	0.193516942	-0.00716649	-0.08444153	0.016914795	0.037038672	6	0	0
ADM2	0.0835099	0.055108336	0.028401526	0.277337427	0.016917359	0.037039764	12	0	0
ALKBH3	0.254077	0.265368677	-0.01129167	0.348925244	0.01693526	0.037074433	8	0	0
SEMA4F	0.1604541	0.179825871	-0.0193718	-0.11527011	0.016956124	0.037115578	9	0	0
GPR17	0.7071703	0.722180849	-0.01501058	-0.00454123	0.01695846	0.03711616	11	0	0
C19orf53	0.0762713	0.066590334	0.009680918	0.128947501	0.016964225	0.037124248	8	0	0
MUC4	0.6176228	0.647267529	-0.02964475	-0.06595165	0.016976832	0.037147305	10	0	0
TAPBP	0.3387297	0.364192534	-0.02546279	-0.03691953	0.016998607	0.037190414	35	0	0
GBP6	0.4709682	0.501747744	-0.03077956	-0.11371476	0.017011239	0.037213511	6	0	0
NPVF	0.8613594	0.887352076	-0.02599266	-0.04249832	0.017024736	0.037233869	5	0	0
ATP6V1F	0.1470839	0.158491486	-0.01140756	-0.04674737	0.017024519	0.037233869	11	0	0
OSR2	0.1939585	0.155901372	0.038057101	0.299020642	0.017027368	0.037233869	10	0	0
ENHO	0.2127164	0.232745038	-0.02002864	0.001469918	0.017028849	0.037233869	3	0	0
TRIM56	0.2771023	0.288399011	-0.01129676	-0.0322771	0.017032697	0.037237744	11	0	0
SMARCAL1	0.2568069	0.288507829	-0.03170091	-0.05034733	0.017040373	0.037249984	11	0	0
RPL26L1	0.1510629	0.149450201	0.001612697	-0.04734882	0.017054838	0.037277063	8	0	0
NMNAT2	0.3759959	0.395292162	-0.01929625	0.063341425	0.017064636	0.037293933	10	0	0
PABPN1L	0.619776	0.645264214	-0.02548818	-0.04927622	0.017069656	0.037300358	11	0	0
IPPK	0.1429713	0.179460416	-0.0364891	-0.06597739	0.017075505	0.037308595	6	0	0
SACM1L	0.0519925	0.051138467	0.000854011	0.004595856	0.017086481	0.037326296	17	0	0
NRN1	0.1537185	0.107867503	0.045850978	0.53000475	0.017089248	0.037326296	17	0	0
DNAH9	0.2675112	0.252489986	0.015021177	0.209801889	0.01708985	0.037326296	14	0	0
CHPF2	0.2527091	0.275424953	-0.02271587	-0.09727542	0.017098731	0.037341148	5	0	0
HNRNPDL	0.1259275	0.137771759	-0.01184421	-0.06523032	0.017101122	0.037341822	6	0	0
SRGAP3	0.0563995	0.058555097	-0.00215563	-0.04005709	0.017105651	0.037347164	12	0	0
DHRS4L2	0.8209119	0.853481492	-0.03256964	-0.05546981	0.017109277	0.037350535	1	0	0
DAZAP2	0.1190636	0.132138031	-0.01307441	-0.01282853	0.017128634	0.037383692	9	0	0
MNT	0.0819242	0.078678414	0.003245744	0.08440545	0.017128451	0.037383692	16	0	0
CXCL14	0.260332	0.241339374	0.018992594	0.305683482	0.017140641	0.037405346	10	0	0
COG4	0.0447957	0.042811932	0.001983739	0.062052121	0.017143382	0.037406776	14	0	0
F2	0.8053541	0.793394697	0.011959383	0.025001462	0.017152638	0.03742242	6	0	0
GNRH1	0.8284408	0.843526343	-0.01508553	-0.02374642	0.017160089	0.037434124	3	0	0
WDR36	0.4124118	0.440095808	-0.02768402	-0.02973106	0.017169129	0.037449289	6	0	0
MDP1	0.3101275	0.316813471	-0.006686	0.01435376	0.017176381	0.037456878	9	0	0
SLC12A6	0.3356949	0.365815593	-0.03012071	-0.06191694	0.017193528	0.037488831	9	0	0
FBXL7	0.0882659	0.0582541	0.030011792	0.534839993	0.017198598	0.037495326	8	0	0
OS9	0.1694429	0.180487113	-0.01104417	0.038702767	0.017220636	0.03753881	12	0	0
ETV7	0.4073997	0.413087386	-0.00568765	0.114393195	0.01724257	0.037582057	10	0	0
KIF1A	0.0890454	0.06887632	0.020169107	0.384037414	0.017249569	0.037590205	6	0	0
MRPL52	0.2489544	0.25817286	-0.00921847	-0.026516	0.017250501	0.037590205	14	0	0
COMMD4	0.311898	0.315097892	-0.0031999	0.050221722	0.017266765	0.037618492	3	0	0
CD300LG	0.2721533	0.250568938	0.021584389	0.097653084	0.017267676	0.037618492	13	0	0
KCNAB3	0.7505617	0.748567652	0.001994012	0.005775189	0.017273125	0.037625792	12	0	0
CMIP	0.1405763	0.155372271	-0.01479596	-0.03962485	0.01728314	0.037643035	5	0	0
BBS12	0.0517147	0.047614484	0.004100215	0.097693682	0.017294544	0.037663301	9	0	0
KCNJ1	0.7369066	0.761110319	-0.02420373	-0.05058389	0.017307482	0.037682326	6	0	0
GFAP	0.7291934	0.764034225	-0.03484081	-0.0665944	0.017307037	0.037682326	4	0	0
SDR42E1	0.4641636	0.490656935	-0.02649329	-0.06508592	0.017310148	0.037683556	8	0	0
CDKN1C	0.2240609	0.199254841	0.024806089	0.135164942	0.017313142	0.0376855	24	0	0
LPAR1	0.2059443	0.19135951	0.014584802	0.347997216	0.017322914	0.037702195	6	0	0

TTC17	0.2217832	0.252655625	-0.03087238	-0.07602888	0.017334676	0.037723217	10	0	0
KCNH4	0.2255259	0.246836296	-0.0213104	0.107427572	0.017341907	0.037729797	8	0	0
C19orf12	0.126446	0.13483322	-0.00838725	-0.01969206	0.017340814	0.037729797	13	0	0
MEPE	0.8072262	0.829498678	-0.02227247	-0.04376448	0.017356259	0.037747285	4	0	0
PAN3	0.0477894	0.046622168	0.001167203	0.035820586	0.017355421	0.037747285	10	0	0
USP20	0.2939056	0.307165699	-0.0132601	-0.04299295	0.01736478	0.037761239	4	0	0
GPR89A	0.8170934	0.849159193	-0.03206579	-0.05487521	0.017369113	0.037766083	1	0	0
MRPS26	0.0954288	0.108817063	-0.01338827	-0.01956724	0.017377429	0.037779584	5	0	0
STOM	0.2672885	0.265842717	0.001445809	0.498134915	0.017386205	0.037794082	15	0	0
FBXO28	0.2208397	0.250767517	-0.02992777	-0.0545775	0.017400149	0.037819811	7	0	0
PPIE	0.0864351	0.095065395	-0.00863027	-0.0033393	0.017408373	0.037833101	13	0	0
HAPLN3	0.2160894	0.207869933	0.008219488	0.410579704	0.017442886	0.037903515	9	0	0
CHP2	0.2415146	0.24406534	-0.00255072	0.025233961	0.017460193	0.037936526	6	0	0
ABL2	0.2159511	0.207077185	0.008873903	0.03188504	0.017482072	0.037979463	14	0	0
FBXO17	0.1980612	0.157438933	0.040622259	0.458006626	0.017496716	0.038006673	13	0	0
ZFR	0.4488724	0.495381717	-0.04650932	-0.05392564	0.017509862	0.038030623	5	0	0
FAM126B	0.0740843	0.06874893	0.005335412	0.076495736	0.0175145	0.03803609	19	0	0
KRTAP12-3	0.468927	0.479025414	-0.01009837	-0.039669	0.01751772	0.038038478	8	0	0
PPP1R3C	0.1499771	0.122919281	0.027057805	0.469810913	0.017533086	0.038062628	11	0	0
MRGPRE	0.6116661	0.605632016	0.006034084	0.02092808	0.017531426	0.038062628	12	0	0
TCEA2	0.1321942	0.101411597	0.030782586	0.311348453	0.017539373	0.038071668	14	0	0
LCP1	0.067667	0.064057358	0.003609649	0.088566709	0.017548974	0.038083291	7	0	0
LRRC63	0.067667	0.064057358	0.003609649	0.088566709	0.017548974	0.038083291	7	0	0
RCE1	0.1803002	0.189673835	-0.00937364	-0.04205477	0.017561423	0.038105696	6	0	0
RPL36AL	0.0953364	0.109932741	-0.01459637	-0.08782023	0.017564945	0.038108727	8	0	0
SEMA3B	0.4518507	0.434858105	0.0169926	0.127696469	0.017569208	0.038108758	15	0	0
AFMID	0.2405656	0.259234703	-0.0186691	-0.06318041	0.017567898	0.038108758	7	0	0
LRP2BP	0.0946996	0.100680739	-0.00598109	-0.12647591	0.017591587	0.038152685	10	0	0
RBP5	0.1983223	0.173061199	0.025261113	0.08878905	0.017596367	0.038158439	15	0	0
BAZ2B	0.2601126	0.200982705	0.059129908	0.269932841	0.017600429	0.038162631	8	0	0
UNC93B1	0.6147477	0.657462438	-0.04271478	-0.09673336	0.017606309	0.038166153	3	0	0
HSPB6	0.2493603	0.281453044	-0.03209273	-0.05782574	0.017605176	0.038166153	11	0	0
L3MBTL4	0.1472223	0.149533132	-0.00231086	0.137738679	0.017616767	0.03818421	10	0	0
ELANE	0.2751804	0.261553547	0.013626814	0.167961588	0.017620773	0.038188277	4	0	0
DKK1	0.1625465	0.131607073	0.030939383	0.328946495	0.017640529	0.038226474	12	0	0
RAD51	0.0370083	0.037954556	-0.00094622	-0.02110069	0.017645094	0.038231747	11	0	0
KIF1C	0.181568	0.198474122	-0.01690617	-0.04497833	0.017671109	0.03828349	9	0	0
EDARADD	0.7731011	0.812419423	-0.03931827	-0.0709681	0.017684702	0.038296046	4	0	0
OXSR1	0.1934504	0.217606583	-0.02415622	-0.05918259	0.017684894	0.038296046	9	0	0
ZXDC	0.1625374	0.159132939	0.003404418	0.136561672	0.017685446	0.038296046	7	0	0
MC2R	0.6333199	0.676027114	-0.0427072	-0.07915756	0.017682811	0.038296046	7	0	0
GTPBP8	0.1833916	0.197477963	-0.01408632	-0.056861	0.017721243	0.038367735	10	0	0
EBAG9	0.2593024	0.268919975	-0.0096176	-0.07953324	0.01772283	0.038367735	7	0	0
CAPN15	0.0861454	0.076807365	0.009338019	0.048196507	0.017741717	0.038403986	4	0	0
MSH5	0.0810487	0.091750775	-0.01070208	-0.02646157	0.017753315	0.038424454	17	0	0
TFIP11	0.1115657	0.125424079	-0.01385837	-0.09544629	0.017759608	0.038433437	16	0	0
GPC1	0.0980853	0.092367711	0.005717608	0.142137698	0.017779293	0.038471397	2	0	0
PI16	0.6566502	0.687289147	-0.03063896	-0.06529501	0.017783085	0.03847496	5	0	0
BTF3L4	0.0458484	0.043256842	0.002591563	0.07182723	0.017815171	0.038539732	10	0	0
PTGDR2	0.5129953	0.493839509	0.01915579	0.090732908	0.017818493	0.038542268	9	0	0
KIF9	0.0784641	0.072512776	0.005951287	0.079088861	0.017829944	0.038553089	11	0	0
ARHGAP20	0.2678303	0.244097551	0.023732736	0.305939317	0.017827199	0.038553089	10	0	0
GRTP1	0.4019978	0.421854654	-0.0198569	-0.01508677	0.017829192	0.038553089	5	0	0
BCS1L	0.2331669	0.250113871	-0.01694692	-0.09461393	0.017836465	0.038557893	5	0	0
RASSF1	0.3669031	0.337423074	0.029480031	0.623319999	0.017834323	0.038557893	13	0	0
CYLD	0.150175	0.143454856	0.00672019	0.109675408	0.017842396	0.038566065	7	0	0
INPP5F	0.1071873	0.124053853	-0.01686656	-0.04621672	0.01784554	0.038568213	8	0	0
CYP7B1	0.3393286	0.344261084	-0.00493249	0.170885984	0.017865632	0.03858954	11	0	0
EGFL7	0.3724413	0.407192013	-0.0347507	-0.14502632	0.017866165	0.03858954	2	0	0
USP8	0.0348419	0.036128057	-0.00128615	0.009869943	0.017865564	0.03858954	5	0	0
UBE2Z	0.227975	0.245699846	-0.01772488	-0.0311444	0.017862771	0.03858954	8	0	0
SLA2	0.6488418	0.67765809	-0.02881633	-0.0524844	0.017861082	0.03858954	7	0	0
SHC2	0.1219845	0.093961007	0.028023515	0.360938327	0.017871408	0.03859157	4	0	0

PRMT2	0.2262575	0.238050206	-0.01179273	0.004014021	0.017869566	0.03859157	5	0	0
GCK	0.8260918	0.876319152	-0.05022733	-0.0841649	0.017879956	0.038605379	1	0	0
GLIPR1L2	0.1397724	0.153135876	-0.01336351	-0.09154622	0.017885707	0.038613149	13	0	0
GSG1L	0.1497789	0.127833314	0.021945634	0.663015813	0.017897225	0.038633364	13	0	0
PRRT4	0.088694	0.094591794	-0.0058978	-0.03071604	0.017900786	0.038636401	5	0	0
NLE1	0.3011328	0.305388635	-0.00425579	-0.0457093	0.017912863	0.038657815	10	0	0
TIMELESS	0.2286965	0.23385526	-0.00515872	0.042589604	0.017924631	0.038678558	10	0	0
ZNF16	0.2410598	0.261459941	-0.02040019	-0.0837629	0.017931064	0.038687786	9	0	0
KLRC2	0.8637695	0.889558025	-0.02578849	-0.04196354	0.017954396	0.038724153	1	0	0
TFCP2	0.1616198	0.180756992	-0.01913716	0.02546987	0.017951175	0.038724153	14	0	0
NEURL2	0.1140472	0.129554068	-0.01550686	-0.05929525	0.017954034	0.038724153	10	0	0
CDK5R2	0.1146914	0.112125954	0.002565401	0.13054533	0.017985136	0.038771803	13	0	0
B3GNTL1	0.2588353	0.264697829	-0.00586256	-0.00201915	0.017981789	0.038771803	6	0	0
RNF24	0.3887351	0.411735483	-0.02300035	-0.03727113	0.017983	0.038771803	5	0	0
VANGL1	0.3439817	0.363934439	-0.01995275	-0.06368036	0.018005008	0.03879133	7	0	0
PTCD1	0.240749	0.258429417	-0.01768046	-0.05034078	0.018003736	0.03879133	14	0	0
MED17	0.1969271	0.197057186	-0.0001301	0.069995795	0.018000107	0.03879133	12	0	0
SERPINB7	0.6588627	0.696596775	-0.03773412	-0.07340693	0.018001527	0.03879133	5	0	0
KLF7	0.444975	0.458945138	-0.01397012	0.040122612	0.018009346	0.038796016	10	0	0
RASGEF1C	0.2843698	0.29296492	-0.00859511	0.121633219	0.018030371	0.038836645	3	0	0
TGS1	0.0565116	0.068892151	-0.01238057	-0.06696464	0.018061198	0.038898372	18	0	0
CYB5RL	0.0426719	0.048441956	-0.00577003	-0.05141061	0.018111614	0.039002269	14	0	0
MYL12B	0.1315294	0.167914966	-0.03638557	-0.15882892	0.018131618	0.03904066	3	0	0
SRD5A3	0.2889107	0.295389992	-0.00647933	0.000158301	0.018163703	0.039105051	10	0	0
ZNF184	0.2285213	0.257408656	-0.02888735	-0.0763981	0.018167002	0.039107459	12	0	0
SPDYE3	0.8505094	0.854532057	-0.00402268	-0.00615491	0.01818581	0.039143247	2	0	0
TCEA3	0.2015538	0.212565287	-0.0110115	-0.10124075	0.018190755	0.039149194	13	0	0
DGKQ	0.2583123	0.280376976	-0.02206471	0.034731351	0.018196064	0.039151224	9	0	0
CWC25	0.4659598	0.503753612	-0.03779377	-0.08800763	0.018195026	0.039151224	3	0	0
TRIM21	0.2523872	0.271172772	-0.01878558	0.004945035	0.018209223	0.039170139	4	0	0
ZNF714	0.7149563	0.729983619	-0.01502733	-0.03857976	0.018208647	0.039170139	4	0	0
GJC1	0.3810231	0.378383285	0.002639847	0.143397677	0.018223723	0.03919663	8	0	0
TMEM244	0.7604814	0.80272817	-0.0422468	-0.07752879	0.018225922	0.039196659	7	0	0
KRTAP9-4	0.7612305	0.785309127	-0.02407862	-0.04856319	0.01823016	0.039201073	3	0	0
ELMO3	0.5698148	0.582032148	-0.01221734	-0.02142262	0.018258863	0.039258087	8	0	0
OCA2	0.1769626	0.170618037	0.006344596	0.261564424	0.0182822	0.039303553	8	0	0
DCAF10	0.3327768	0.353037416	-0.02026066	-0.08334852	0.018293925	0.039324045	8	0	0
PARD6G	0.1512623	0.144582303	0.006679974	0.153733739	0.018305581	0.039344387	12	0	0
TRMT11	0.1994951	0.223886867	-0.02439181	-0.07185767	0.018317382	0.039360317	11	0	0
PRRG2	0.1228454	0.127161358	-0.00431595	0.027288762	0.018316549	0.039360317	10	0	0
CEP250	0.301174	0.313541201	-0.01236718	0.030278262	0.018321928	0.039365368	12	0	0
MUL1	0.3107737	0.32321964	-0.01244599	-0.06022454	0.01832854	0.039374859	11	0	0
DENND2D	0.2570822	0.270608406	-0.01352618	0.092159399	0.018334566	0.039383088	9	0	0
NTSR2	0.3283648	0.322876524	0.005488326	0.103262191	0.018345054	0.039400896	9	0	0
FAM78A	0.1499264	0.11045189	0.039474472	0.814151942	0.018354424	0.039416302	6	0	0
RASA2	0.0898548	0.103548433	-0.0136936	-0.10790699	0.018356855	0.039416803	9	0	0
FRMD3	0.4274174	0.421510955	0.005906449	0.328158157	0.01836627	0.039432298	5	0	0
CDH24	0.0548731	0.060625659	-0.00575258	-0.0699486	0.018380185	0.039457451	5	0	0
SRGN	0.3297999	0.26634118	0.063458763	0.465953215	0.018399507	0.039494204	7	0	0
PDIA4	0.0796741	0.065476623	0.014197509	0.099080432	0.018407035	0.039500907	9	0	0
SNUPN	0.3834144	0.384090726	-0.00067635	0.10768417	0.018406087	0.039500907	11	0	0
FARP2	0.1326557	0.134126276	-0.0014706	0.063543925	0.018421724	0.039523302	7	0	0
HSD11B2	0.0986019	0.108596674	-0.00999478	-0.11467646	0.018421878	0.039523302	13	0	0
FAM3C	0.2593126	0.27380763	-0.01449501	-0.07474791	0.018441825	0.039558097	7	0	0
SNX19	0.4570912	0.478697216	-0.02160599	-0.10514825	0.018442507	0.039558097	5	0	0
TEX19	0.6981567	0.741415429	-0.04325871	-0.08856996	0.018456746	0.039583904	4	0	0
ZCCHC24	0.1286445	0.100423716	0.028220756	0.267481412	0.018478164	0.039625101	9	0	0
SCMH1	0.1053977	0.124780726	-0.019383	0.009687478	0.018547358	0.039768729	7	0	0
MFN2	0.1969278	0.211848881	-0.01492113	-0.06908537	0.018563601	0.039790897	10	0	0
IMPA1	0.2795921	0.294045885	-0.01445377	-0.02896535	0.018560967	0.039790897	6	0	0
NTN5	0.7780125	0.788816769	-0.01080422	-0.01719262	0.018564352	0.039790897	9	0	0
TNXB	0.8921608	0.882336343	0.009824495	0.01692288	0.018569583	0.039795902	7	0	0
THRAP3	0.0517856	0.048089182	0.003696383	0.070986686	0.018591836	0.039831179	9	0	0

ZSWIM5	0.7207467	0.704940875	0.015805826	0.040261407	0.018595826	0.039831179	3	0	0
RAET1G	0.1534363	0.152728916	0.000707385	0.106695331	0.01859647	0.039831179	10	0	0
FAM166A	0.7029703	0.687584019	0.015386316	0.036053952	0.018594012	0.039831179	5	0	0
RAB2B	0.0362254	0.033514812	0.002710633	0.076417056	0.018603445	0.039836004	18	0	0
CALM1	0.4235207	0.464996506	-0.04147581	-0.10760854	0.018605386	0.039836004	4	0	0
TUBG2	0.3461531	0.363118277	-0.01696517	-0.08042361	0.018604696	0.039836004	10	0	0
TUBGCP2	0.8059974	0.835858516	-0.02986116	-0.05901133	0.018615636	0.039853192	8	0	0
DOCK2	0.1440544	0.100728009	0.043326385	0.660376382	0.018638658	0.039897717	8	0	0
ZDHHC16	0.1384457	0.148667959	-0.01022223	0.041555029	0.018678771	0.039978811	10	0	0
SLC16A13	0.0374434	0.038878015	-0.00143457	0.014190316	0.018684291	0.039985854	13	0	0
AK8	0.2359917	0.223874602	0.012117128	0.079798716	0.018689987	0.039993272	11	0	0
XKR8	0.4311377	0.453462924	-0.02232524	-0.04306831	0.018724486	0.040062314	11	0	0
ITPK1	0.15629	0.160940868	-0.00465088	0.0093052	0.018740005	0.040090736	6	0	0
SLC44A2	0.2812645	0.275662404	0.005602077	0.203531706	0.018753057	0.04010909	7	0	0
CALM3	0.0402765	0.039976773	0.000299686	0.042652575	0.018752999	0.04010909	8	0	0
SPSB4	0.1151515	0.107969129	0.007182412	0.197064483	0.018802851	0.040206245	14	0	0
GTF3C6	0.1080972	0.115704474	-0.00760729	-0.05055479	0.018803935	0.040206245	13	0	0
ANAPC11	0.4370322	0.467426674	-0.03039449	-0.09491179	0.018805207	0.040206245	11	0	0
TMEM150A	0.0680315	0.087946108	-0.01991456	-0.06621895	0.018810253	0.040212241	11	0	0
PPIL3	0.0591711	0.059622249	-0.00045111	-0.00032552	0.018819338	0.040226868	12	0	0
SPATA22	0.8941558	0.914941151	-0.02078532	-0.03305462	0.018825699	0.040235669	2	0	0
ORAI2	0.082198	0.083757061	-0.0015591	0.053433601	0.018834155	0.040248947	3	0	0
YIF1B	0.4249226	0.36820718	0.056715393	0.214166264	0.018836424	0.040248999	2	0	0
RPE65	0.6712106	0.729839834	-0.05862922	-0.12152834	0.018847944	0.040268819	2	0	0
KCNQ2	0.2400043	0.225764685	0.014239595	0.741972798	0.018851353	0.040271304	3	0	0
FOXH1	0.5515182	0.575170243	-0.02365207	-0.03277961	0.018859775	0.0402797	10	0	0
PDS5A	0.0466132	0.046591684	2.16E-05	0.027546433	0.018878107	0.040314052	5	0	0
CHP1	0.1346624	0.141643378	-0.00698096	-0.02855459	0.018892174	0.040339289	13	0	0
NSA2	0.1755554	0.208307535	-0.03275214	-0.08673723	0.018904383	0.040359509	13	0	0
ARHGEF7	0.1767444	0.187384582	-0.01064014	-0.01595637	0.018906144	0.040359509	5	0	0
ANPEP	0.4445375	0.434376746	0.010160759	0.167822849	0.018912415	0.040368092	4	0	0
C9	0.1419005	0.121609405	0.020291085	0.334635726	0.018917021	0.040373118	12	0	0
TEX12	0.8444772	0.830913053	0.013564197	0.02577754	0.018921856	0.040378634	10	0	0
LPCAT2	0.4811268	0.514264032	-0.03313721	-0.1010291	0.018933378	0.040398413	9	0	0
NOTCH3	0.2897815	0.279238588	0.010542951	0.172345363	0.018945314	0.040414266	6	0	0
DIDO1	0.1201172	0.143463927	-0.02334668	-0.03177532	0.018943693	0.040414266	9	0	0
ZBED3	0.1743582	0.151252828	0.023105327	0.081845776	0.01896155	0.04044409	9	0	0
MLLT11	0.3775057	0.420218327	-0.04271262	-0.14985643	0.018976067	0.040470243	2	0	0
UBE2C	0.1726501	0.180645822	-0.00799573	-0.05524095	0.018984382	0.040483162	14	0	0
KIF11	0.0401045	0.035983762	0.004120702	0.114946758	0.018993002	0.040496729	4	0	0
ANKRD35	0.1246889	0.104376739	0.020312158	0.346879489	0.018995354	0.04049693	12	0	0
BATF3	0.1189265	0.094872898	0.024053586	0.466000321	0.019019031	0.04053777	8	0	0
SPRYD3	0.1485628	0.153648555	-0.00508573	0.0339067	0.019030522	0.040557443	9	0	0
TMEM114	0.500801	0.522544419	-0.02174339	-0.04649344	0.019047716	0.040589264	9	0	0
ABHD11	0.1532958	0.152794868	0.000500962	0.053119496	0.019052318	0.040594248	8	0	0
B3GNT4	0.2187818	0.231086418	-0.01230462	-0.02890252	0.019071352	0.040629976	10	0	0
EME1	0.147625	0.168203608	-0.02057859	-0.03806588	0.019088998	0.040662739	15	0	0
GPR85	0.3405706	0.370275201	-0.02970459	-0.08976097	0.019102861	0.040687437	5	0	0
RIPPLY2	0.1594296	0.131053343	0.028376217	0.571511448	0.019121134	0.040716688	10	0	0
RNF126	0.1369007	0.156765272	-0.01986459	-0.00408641	0.019119264	0.040716688	6	0	0
PIK3CD	0.4074216	0.410104123	-0.00268253	0.171359085	0.019125549	0.040721254	6	0	0
CLDN14	0.7430465	0.7650327	-0.02198624	-0.03711519	0.019135807	0.04073826	9	0	0
TAF3	0.0961661	0.094997596	0.001168531	-0.04219972	0.019146827	0.040756881	8	0	0
TRIM38	0.312968	0.329082689	-0.01611465	-0.02065446	0.019161865	0.040784053	8	0	0
RAB3C	0.0959251	0.067801416	0.028123729	0.451390989	0.019243748	0.040953472	7	0	0
PRSS22	0.1616599	0.175645351	-0.01398542	-0.08043401	0.019246935	0.040955395	15	0	0
TAS2R19	0.8429741	0.864190813	-0.02121675	-0.03542363	0.019266279	0.040991694	5	0	0
SDHB	0.2669066	0.287825334	-0.02091873	-0.03736751	0.019277938	0.041001909	12	0	0
NKG7	0.4113159	0.452366617	-0.04105074	-0.13348225	0.019277003	0.041001909	3	0	0
MMP9	0.3143606	0.310251944	0.004108638	0.030101053	0.019274904	0.041001909	6	0	0
BMPR1B	0.2716229	0.276724709	-0.00510185	0.103096821	0.019287646	0.041017693	4	0	0
DPH6	0.2101965	0.206610364	0.003586149	-0.01917218	0.019312446	0.041065564	7	0	0
USP4	0.0637388	0.0475318	0.016206972	0.336535413	0.019319958	0.041074614	7	0	0

COMMD8	0.2710458	0.285729812	-0.01468399	-0.00983292	0.019323572	0.041074614	7	0	0
MYO15A	0.6904682	0.690053463	0.000414746	0.007421064	0.019321393	0.041074614	8	0	0
SLC27A2	0.1796329	0.165222439	0.014410429	0.147623799	0.019326642	0.041076272	10	0	0
RFT1	0.1684902	0.177006938	-0.00851676	-0.02056924	0.019335771	0.041085936	12	0	0
TMEM190	0.4933265	0.533203558	-0.03987702	-0.12201089	0.019335575	0.041085936	10	0	0
DECR2	0.07872	0.0896911	-0.0109711	-0.01825331	0.019340045	0.041090149	9	0	0
TEP1	0.2286409	0.225585456	0.003055411	0.108323347	0.019352478	0.041111696	9	0	0
EZR	0.2694467	0.259385041	0.01006167	0.010448406	0.019379211	0.041163611	11	0	0
C1orf105	0.7851193	0.806332193	-0.02121291	-0.03781052	0.019384992	0.041171014	2	0	0
SSUH2	0.6343613	0.683793899	-0.04943264	-0.10663739	0.019409913	0.041204731	1	0	0
CAMLG	0.0649205	0.042941349	0.021979142	0.400513642	0.019410946	0.041204731	8	0	0
TRIM14	0.1633733	0.18563863	-0.02226535	0.014283914	0.019407804	0.041204731	11	0	0
CRAT	0.1121935	0.117589418	-0.00539593	0.038625322	0.019412354	0.041204731	10	0	0
ZNF613	0.0828487	0.061207508	0.021641176	0.474359073	0.019411969	0.041204731	11	0	0
TRIB2	0.1294886	0.137690379	-0.00820174	0.001552968	0.019456409	0.041293356	9	0	0
DOCK5	0.0581073	0.045792694	0.012314566	0.318895806	0.019459659	0.041295366	8	0	0
AMIGO3	0.0628308	0.072316618	-0.00948582	-0.07839121	0.01947786	0.041314439	9	0	0
GMPPB	0.0628308	0.072316618	-0.00948582	-0.07839121	0.01947786	0.041314439	9	0	0
FGG	0.6120118	0.654768069	-0.04275625	-0.10270544	0.019473133	0.041314439	5	0	0
TTC9B	0.1545572	0.13916638	0.0153908	0.349685431	0.019474192	0.041314439	9	0	0
RND2	0.30426	0.30838864	-0.00412866	0.247877702	0.019484312	0.041323238	13	0	0
MOSPD3	0.2334872	0.247475961	-0.01398875	0.003096892	0.019506914	0.041366282	15	0	0
ANKRD26	0.1481014	0.172576569	-0.02447518	-0.06227757	0.019509457	0.041366783	6	0	0
RCC2	0.1215801	0.13685041	-0.01527034	-0.05934564	0.019518562	0.041377754	13	0	0
GLG1	0.216477	0.206175404	0.010301547	0.034438966	0.019519245	0.041377754	9	0	0
ZSWIM2	0.1209273	0.123758172	-0.00283089	0.023914319	0.019565259	0.041455698	11	0	0
ITFG2	0.2100616	0.235892569	-0.02583097	-0.04851858	0.019564898	0.041455698	16	0	0
FRS2	0.2443399	0.267478357	-0.02313845	-0.038732	0.019558738	0.041455698	9	0	0
RALBP1	0.1874845	0.212766473	-0.025282	-0.07806775	0.019561078	0.041455698	9	0	0
RNF167	0.1071329	0.110972634	-0.0038397	-0.01022303	0.019580222	0.041482502	14	0	0
PYCR2	0.206826	0.244741007	-0.03791501	-0.09633451	0.01964206	0.041608596	7	0	0
CPLX3	0.3135908	0.320836501	-0.00724571	0.03783523	0.019653613	0.041628153	12	0	0
NKAIN1	0.2753019	0.250744315	0.024557608	0.181840421	0.019656988	0.041630166	7	0	0
STRBP	0.3790549	0.409714345	-0.03065948	-0.06557914	0.019659555	0.041630166	4	0	0
ZRANB1	0.8263311	0.838714901	-0.01238377	-0.02041942	0.019661527	0.041630166	4	0	0
LY9	0.6663239	0.67737593	-0.01105206	-0.03408457	0.019724535	0.041758647	7	0	0
OR11H6	0.6497795	0.679297359	-0.02951781	-0.05217959	0.019730112	0.041765524	3	0	0
DLX2	0.0493025	0.050668176	-0.00136565	0.095327605	0.019736861	0.041774881	12	0	0
IL12A	0.1928974	0.209336957	-0.01643953	0.129687146	0.019773442	0.041838509	14	0	0
PLA2G7	0.2372401	0.23116021	0.006079874	0.383232015	0.019776253	0.041838509	18	0	0
INSC	0.7005752	0.717787563	-0.01721232	-0.02783942	0.019774829	0.041838509	6	0	0
ZNF106	0.264079	0.268767024	-0.00468798	0.004622844	0.019779533	0.041840512	12	0	0
FAM153B	0.6837975	0.732519696	-0.04872218	-0.09791461	0.019794524	0.041867287	1	0	0
MAST1	0.0572309	0.062951605	-0.00572073	-0.08155167	0.019809088	0.041893151	4	0	0
DKKL1	0.126898	0.120517841	0.006380197	0.095392324	0.019828862	0.041930025	9	0	0
SFRP2	0.2747914	0.243077705	0.031713681	0.278253445	0.019840644	0.041949993	22	0	0
DDHD2	0.8467821	0.861631189	-0.01484905	-0.02478942	0.019852327	0.041964801	1	0	0
IGFL1	0.54138	0.590359543	-0.0489795	-0.124185	0.019851922	0.041964801	7	0	0
FBLN7	0.1565749	0.118929542	0.037645332	0.404250773	0.019875553	0.042008947	6	0	0
NPAS3	0.0963907	0.072906801	0.023483902	0.345646119	0.019880823	0.042015133	12	0	0
GFPT2	0.1715444	0.147610758	0.023933634	0.21691339	0.019888803	0.042027046	5	0	0
PTPDC1	0.1317315	0.141791499	-0.01005995	-0.02598552	0.019891857	0.042028549	9	0	0
UBAP1L	0.7958748	0.82488406	-0.02900928	-0.05212651	0.019902321	0.042045704	4	0	0
FAXDC2	0.0921182	0.078535179	0.013582986	0.161746614	0.019907048	0.042050737	8	0	0
ABRA	0.7272819	0.736893797	-0.0096119	-0.01381213	0.019964409	0.042166939	9	0	0
SLC39A11	0.193923	0.208196199	-0.01427323	-0.01446484	0.019982525	0.042200231	10	0	0
CBY1	0.0957904	0.092955122	0.002835249	0.027359663	0.019987935	0.042206687	13	0	0
TRIM9	0.3378054	0.332167901	0.005637525	0.090920585	0.020010112	0.042248542	12	0	0
LOXL4	0.2418627	0.243492625	-0.00162994	0.111815932	0.020027495	0.042280267	5	0	0
ELOVL3	0.2657975	0.267515761	-0.00171826	0.24642228	0.020033363	0.042281921	14	0	0
CDAN1	0.3246098	0.337470275	-0.01286043	-0.01935355	0.020030753	0.042281921	10	0	0
FARSA	0.2771083	0.308493122	-0.03138483	-0.07368123	0.020035351	0.042281921	12	0	0
CDCA7L	0.2027563	0.223191996	-0.02043573	-0.06118016	0.020079758	0.042370652	12	0	0

TBC1D3	0.6397	0.688489327	-0.04878932	-0.10229886	0.020094258	0.04239626	2	0	0
ZAN	0.6135103	0.667734872	-0.05422453	-0.12400231	0.020122833	0.042451557	4	0	0
RNF41	0.038495	0.041917079	-0.0034221	0.02710247	0.020132312	0.042458846	13	0	0
NABP2	0.038495	0.041917079	-0.0034221	0.02710247	0.020132312	0.042458846	13	0	0
ZNF404	0.3236685	0.290661189	0.033007271	0.357066344	0.02013339	0.042458846	5	0	0
PLCB2	0.3954361	0.405225837	-0.00978978	-0.03264234	0.020152176	0.042493467	8	0	0
FLT3	0.3184277	0.272947852	0.045479838	0.711522468	0.020166328	0.042513311	7	0	0
EEF1A2	0.2010021	0.179049722	0.021952334	0.424917388	0.020164727	0.042513311	6	0	0
ZP4	0.6828204	0.724752733	-0.04193237	-0.0764453	0.020173359	0.042523136	7	0	0
CUL3	0.0337315	0.032047187	0.001684273	0.044722115	0.020202293	0.042579122	6	0	0
SLC25A4	0.3546503	0.36744558	-0.01279531	-0.0326896	0.020228249	0.042628818	10	0	0
SMC1B	0.2605987	0.259820583	0.000778091	0.303104801	0.020237739	0.042643807	12	0	0
PDCD2L	0.2414201	0.263966137	-0.02254603	-0.01778837	0.02025047	0.042665622	11	0	0
MACF1	0.2547349	0.226492822	0.028242072	0.384470004	0.020267341	0.042686124	6	0	0
USP1	0.2291206	0.241299558	-0.01217897	-0.01313437	0.02026468	0.042686124	13	0	0
SYCP2	0.2824409	0.259852377	0.022588504	0.363214007	0.020265098	0.042686124	10	0	0
NUMA1	0.2051968	0.237923669	-0.03272686	-0.06388759	0.020271225	0.04268929	12	0	0
IDI2	0.8628478	0.877108875	-0.01426109	-0.02371067	0.020290054	0.042723926	7	0	0
DENND6A	0.0394685	0.041317989	-0.00184944	0.004721916	0.020293319	0.042725785	5	0	0
SIK3	0.2217031	0.195821127	0.025881931	0.200656448	0.020298734	0.04273217	7	0	0
NMRK2	0.1224317	0.095512795	0.026918909	0.334593168	0.020321487	0.042775049	9	0	0
SUMO3	0.6765782	0.698361158	-0.02178293	-0.02969964	0.020343642	0.042816658	7	0	0
SPDEF	0.587344	0.623471693	-0.03612769	-0.08734788	0.020365842	0.042858353	8	0	0
RLF	0.2221715	0.244011546	-0.02184004	-0.06681149	0.02037312	0.042863611	9	0	0
BRI3BP	0.0761439	0.068710241	0.007433662	0.137901839	0.020372777	0.042863611	8	0	0
SGCG	0.6731082	0.664976976	0.008131174	0.033425796	0.020387803	0.042889471	3	0	0
CNST	0.169858	0.205430937	-0.03557297	-0.0833197	0.020399876	0.042894746	11	0	0
SLC25A36	0.217937	0.231581276	-0.01364427	-0.06032941	0.020393318	0.042894746	8	0	0
LDHC	0.6887393	0.728499873	-0.03976058	-0.06379521	0.020398276	0.042894746	6	0	0
LRRC25	0.3698632	0.369131069	0.000732169	0.095825514	0.020395222	0.042894746	4	0	0
STAMBPL1	0.3334232	0.351688523	-0.01826536	-0.08097492	0.020434729	0.042962994	8	0	0
BARD1	0.1854286	0.179893497	0.005535119	0.1043315	0.020449247	0.042986817	10	0	0
PIH1D1	0.0363464	0.037237759	-0.00089133	0.000957852	0.020450853	0.042986817	5	0	0
APOBEC3F	0.6011499	0.658467979	-0.05731807	-0.12752844	0.020461175	0.042998434	2	0	0
APOBEC3G	0.6011499	0.658467979	-0.05731807	-0.12752844	0.020461175	0.042998434	2	0	0
DDX1	0.6587326	0.682062123	-0.02332948	-0.06010148	0.020465786	0.043003085	8	0	0
NANS	0.0670893	0.086908334	-0.019819	0.037406466	0.020469867	0.043006623	11	0	0
FAM169A	0.3374198	0.353420455	-0.01600063	-0.08026121	0.020497017	0.043053577	11	0	0
TBC1D24	0.0543109	0.060783622	-0.00647275	-0.08086294	0.020496728	0.043053577	5	0	0
TMEM183A	0.2303394	0.253405139	-0.02306578	-0.01351969	0.020511397	0.043078736	7	0	0
BACE1	0.169391	0.167650124	0.001740853	0.14279885	0.0205238	0.043099739	13	0	0
ITM2C	0.2790259	0.271697459	0.007328405	0.017258922	0.020538805	0.043126202	14	0	0
IL4I1	0.1994047	0.189400934	0.010003792	0.038428991	0.020546268	0.04313682	13	0	0
ERF	0.1331079	0.115656865	0.017451022	0.116173295	0.020550276	0.043140187	9	0	0
TOMM20	0.4162511	0.438202673	-0.02195156	-0.05614077	0.020553815	0.043142565	10	0	0
CD101	0.6717238	0.716717338	-0.04499355	-0.10298152	0.020567291	0.0431658	3	0	0
MCCC1	0.2744308	0.298532688	-0.02410188	0.026610902	0.020590677	0.043209826	3	0	0
GTSE1	0.2055355	0.222333388	-0.01679791	-0.05521118	0.020595696	0.043215302	10	0	0
FILIP1L	0.748394	0.743618959	0.004774996	0.010648378	0.020607755	0.04323049	6	0	0
PLEKHG4	0.7831028	0.807211655	-0.02410886	-0.04722263	0.020606779	0.04323049	9	0	0
SERPINB11	0.8317585	0.878305107	-0.04654666	-0.0789029	0.020611913	0.043234157	2	0	0
ARMC12	0.5674357	0.579302368	-0.01186666	0.027810465	0.020616043	0.043237763	6	0	0
GBP2	0.799367	0.824128491	-0.0247615	-0.04336505	0.020626	0.043248531	2	0	0
FAM20B	0.4821203	0.499020959	-0.01690067	-0.0202967	0.020623602	0.043248531	11	0	0
BIRC5	0.6104775	0.59566059	0.014816915	0.026622707	0.020640868	0.043274648	4	0	0
UCN	0.5936392	0.565247785	0.028391381	0.094681595	0.020643888	0.04327592	11	0	0
ZNF789	0.0479905	0.04562182	0.002368667	0.073421954	0.020659174	0.043300895	8	0	0
ZNF70	0.0759133	0.080113143	-0.00419982	-0.05784662	0.02066063	0.043300895	8	0	0
BCDIN3D	0.2982001	0.317470864	-0.01927077	-0.06769904	0.0206641	0.043301365	12	0	0
SIPA1L1	0.0439008	0.047054224	-0.00315344	-0.07246265	0.020665683	0.043301365	4	0	0
ZDHHC1	0.3112436	0.298260187	0.012983439	0.246414538	0.020673465	0.043312611	9	0	0
SYT14	0.1318537	0.108088098	0.023765652	0.142545683	0.020694993	0.04335265	9	0	0
GSTA2	0.5439229	0.611068334	-0.06714544	-0.16506685	0.02071917	0.043398229	1	0	0

MRPS22	0.7566405	0.769843147	-0.01320267	-0.02137481	0.020763362	0.043485714	3	0	0
GNPNAT1	0.447049	0.435970174	0.011078811	0.080912281	0.020787561	0.043531313	6	0	0
TNR	0.2449108	0.258913335	-0.01400254	-0.07054352	0.02079698	0.043545953	3	0	0
TMEFF2	0.0809182	0.061366625	0.019551532	0.371718042	0.02082761	0.043604998	4	0	0
SGPP1	0.1982727	0.208516026	-0.01024334	0.041481277	0.020835234	0.043615868	6	0	0
ZNF48	0.1234509	0.132216397	-0.00876551	-0.00694355	0.020848176	0.043637867	12	0	0
TRPC7	0.6692942	0.704273816	-0.03497961	-0.06814633	0.020858987	0.043650754	5	0	0
ART5	0.1530846	0.152087045	0.00099759	0.112557463	0.0208592	0.043650754	9	0	0
TMEM206	0.0685858	0.079385335	-0.01079955	-0.00170169	0.020864093	0.0436559	10	0	0
SCUBE3	0.2745488	0.251890862	0.022657954	0.362272411	0.020873552	0.043670596	8	0	0
GUCA1B	0.8567576	0.882803365	-0.02604578	-0.04388075	0.020889951	0.043693197	5	0	0
GRHPR	0.2177804	0.23324796	-0.01546759	-0.01866459	0.020889442	0.043693197	4	0	0
KBTBD13	0.7792983	0.778925866	0.000372447	0.001834807	0.020891662	0.043693197	4	0	0
COL5A2	0.411747	0.437560299	-0.02581327	-0.02913826	0.02090769	0.043721619	9	0	0
CDC16	0.1901588	0.201134208	-0.0109754	-0.17307072	0.020913338	0.043728333	7	0	0
GATA3	0.1565559	0.127372744	0.029183149	0.357544217	0.020925279	0.0437482	25	0	0
MYOF	0.0595381	0.050963552	0.008574589	0.16093002	0.020931279	0.043755644	10	0	0
RHOQ	0.3519904	0.362567957	-0.01057757	-0.07216462	0.020937032	0.043762568	8	0	0
USP28	0.1893061	0.208401319	-0.01909523	-0.0407785	0.020944314	0.043772689	7	0	0
EXOC7	0.2311797	0.231478477	-0.00029881	0.483852884	0.02095355	0.043784254	4	0	0
FAM129C	0.4188141	0.443565885	-0.02475181	-0.09945486	0.02095473	0.043784254	6	0	0
CSRP1	0.8737872	0.88854164	-0.01475445	-0.02447173	0.020959403	0.043788917	5	0	0
CCDC114	0.1390293	0.154069508	-0.01504018	-0.03069268	0.021011747	0.043893163	4	0	0
HSD3B1	0.6819429	0.702110133	-0.02016726	-0.0430141	0.021016428	0.043897829	3	0	0
CELF4	0.0849756	0.065513638	0.019461978	0.363164588	0.021020421	0.043901057	24	0	0
WDR86	0.3358956	0.315974727	0.019920874	0.261846411	0.021030523	0.043911928	9	0	0
HSD3B7	0.4510817	0.467120293	-0.01603854	-0.0032929	0.021030277	0.043911928	13	0	0
ADAMTS17	0.0448596	0.037641452	0.007218138	0.211754448	0.021052357	0.0439524	10	0	0
FAM204A	0.1459074	0.168701925	-0.0227945	-0.08616647	0.021058251	0.043959589	16	0	0
MICU2	0.3374364	0.348669785	-0.01123335	-0.02495086	0.021075722	0.04399094	8	0	0
FHL3	0.2205133	0.227912364	-0.00739906	0.00778936	0.021085758	0.044006767	6	0	0
WDR63	0.2792228	0.23857703	0.040645775	0.407875548	0.021088398	0.044007155	8	0	0
TOP2B	0.0265388	0.024676314	0.001862471	0.075937145	0.021093387	0.044012447	3	0	0
CD86	0.7142631	0.764438299	-0.05017522	-0.0990589	0.021099922	0.044014988	2	0	0
CUZD1	0.1793208	0.171691095	0.00762973	0.275934068	0.021101967	0.044014988	15	0	0
FAM24B	0.1793208	0.171691095	0.00762973	0.275934068	0.021101967	0.044014988	15	0	0
ARPC4	0.1131819	0.119877664	-0.00669574	-0.03869586	0.021116557	0.044040299	17	0	0
DMRTB1	0.8798982	0.901049772	-0.02115158	-0.03503459	0.021136837	0.044077468	14	0	0
ADCK5	0.3866312	0.431854224	-0.04522301	-0.13414821	0.021156211	0.044112741	4	0	0
METRN	0.3397864	0.362202591	-0.02241618	-0.07033667	0.02116186	0.044119391	4	0	0
GNPDA2	0.2366214	0.253535271	-0.01691386	-0.03394396	0.021185409	0.044163353	13	0	0
TADA3	0.0933513	0.100113976	-0.00676268	-0.00603242	0.021197694	0.044178693	19	0	0
TBX2	0.0822929	0.060881905	0.021411018	0.451520863	0.021195544	0.044178693	15	0	0
SHH	0.1160819	0.091026572	0.025055327	0.298163583	0.021225765	0.044232057	9	0	0
C5orf24	0.316435	0.320106038	-0.00367105	0.043940711	0.021240069	0.044256724	11	0	0
TMPRSS5	0.777562	0.790585553	-0.01302359	-0.02113602	0.021246829	0.044265667	5	0	0
ARMC9	0.1722618	0.185419154	-0.01315738	-0.0367119	0.0212554	0.044278381	10	0	0
TMEM229B	0.4529722	0.473026371	-0.02005417	-0.05864631	0.021260698	0.044284274	3	0	0
KCNG2	0.8095065	0.834408852	-0.02490238	-0.04334084	0.021263907	0.044285815	8	0	0
FANCG	0.2457125	0.272796424	-0.0270839	-0.0421985	0.021275174	0.044304135	12	0	0
MRPL37	0.8931325	0.887888031	0.005244426	0.008502373	0.02127799	0.044304856	3	0	0
GPR158	0.1665556	0.133604718	0.032950855	0.30363941	0.021307825	0.044361827	12	0	0
TMEM230	0.1024132	0.124663098	-0.02224991	-0.07813625	0.021315622	0.044372911	5	0	0
ANKRD23	0.2465647	0.238097362	0.008467368	0.093959227	0.02131914	0.044375084	13	0	0
MINOS1	0.2069426	0.212905034	-0.00596245	0.001352064	0.021324239	0.044380547	12	0	0
CAPN8	0.7222205	0.755119259	-0.0328988	-0.07227548	0.021329529	0.044386406	6	0	0
YARS	0.067698	0.077971821	-0.01027382	-0.07698151	0.02134809	0.044419877	6	0	0
PLA2G15	0.1008686	0.087520733	0.013347829	0.05699964	0.021353872	0.044426755	10	0	0
VEGFC	0.6833901	0.683532362	-0.00014231	0.013444108	0.021377088	0.044464741	2	0	0
RABEP2	0.4005094	0.371731302	0.028778111	0.328180516	0.021375395	0.044464741	7	0	0
TMEM98	0.1702866	0.14728423	0.023002411	0.146874817	0.021388321	0.044482946	10	0	0
FADS6	0.2353804	0.219288676	0.016091761	0.361894584	0.021394202	0.044490018	10	0	0
CPA2	0.7414474	0.764547407	-0.02310005	-0.04190381	0.021422435	0.044543566	3	0	0

DIRAS1	0.3860435	0.388498544	-0.00245507	0.038111059	0.021433183	0.044560747	6	0	0
AHSA1	0.0436404	0.043349296	0.000291088	0.020855587	0.021443621	0.044577282	11	0	0
HPCAL4	0.1049964	0.112512839	-0.00751647	0.036052312	0.021453532	0.044592716	12	0	0
PI4KA	0.2025695	0.233635896	-0.03106637	-0.12026703	0.021458935	0.044598778	4	0	0
CHST2	0.3836486	0.413630866	-0.02998225	0.141908396	0.021467808	0.044601876	6	0	0
NDUFA8	0.2584936	0.275986268	-0.01749263	-0.0305342	0.021467886	0.044601876	7	0	0
DUSP16	0.259675	0.235076389	0.024598569	0.099461284	0.0214676	0.044601876	8	0	0
CCDC96	0.0906666	0.080028268	0.010638369	0.086831406	0.021477386	0.044616445	10	0	0
ISM2	0.1831891	0.193626147	-0.010437	0.206784645	0.021481124	0.044619043	10	0	0
ARPC4-TTLL	0.0593323	0.062914621	-0.00358232	-0.02266674	0.02149828	0.044649508	21	0	0
FDXR	0.1162398	0.130979795	-0.01473998	-0.04480555	0.0215082	0.044664939	9	0	0
FAH	0.2221955	0.205699707	0.016495759	0.090227808	0.021510952	0.044665483	9	0	0
UBA3	0.2083733	0.221496038	-0.01312272	0.055690657	0.021522554	0.044683375	8	0	0
GP6	0.7351214	0.764155416	-0.029034	-0.05558237	0.021524552	0.044683375	8	0	0
PIGL	0.0993784	0.104458137	-0.00507977	-0.00683623	0.021554095	0.044739527	16	0	0
MTG1	0.3434229	0.345012893	-0.00158997	0.048178777	0.021589018	0.04480683	6	0	0
ZMYND12	0.1423774	0.164845601	-0.02246816	-0.03878498	0.021591865	0.044807554	12	0	0
NIPAL4	0.29265	0.298390315	-0.00574029	0.112610473	0.021617948	0.044856492	13	0	0
HRH2	0.288217	0.27615176	0.012065203	0.166720623	0.021627083	0.044870256	9	0	0
SULT1C3	0.8202403	0.854866219	-0.0346259	-0.05894799	0.021638532	0.044888816	1	0	0
KCNG1	0.3933411	0.392670821	0.000670265	0.254263377	0.021644516	0.044896038	4	0	0
HIST1H4G	0.8843329	0.901180927	-0.01684803	-0.03000339	0.021668532	0.044940655	9	0	0
ACY1	0.1140018	0.112394558	0.001607209	0.13471258	0.021671432	0.044941473	15	0	0
PTGR2	0.1128946	0.127766888	-0.01487228	-0.03149532	0.021679424	0.044949066	11	0	0
HSPB9	0.5470096	0.581669529	-0.03465994	-0.07271886	0.021680105	0.044949066	13	0	0
ETNPPL	0.2229602	0.222950096	1.01E-05	0.172775391	0.021702846	0.044991013	13	0	0
TNFRSF25	0.4755102	0.458288532	0.017221661	0.123169913	0.021718706	0.045018688	11	0	0
ZNF280A	0.6341071	0.694537961	-0.06043086	-0.11333965	0.021734148	0.045045491	4	0	0
NPFF	0.7115338	0.728585181	-0.01705142	-0.07075952	0.021739491	0.045051358	7	0	0
IVL	0.6149094	0.646842306	-0.03193289	-0.06174617	0.021746513	0.045060705	6	0	0
PSME2	0.1270601	0.146914419	-0.01985434	-0.06603674	0.021755428	0.045071878	12	0	0
SNF8	0.1542541	0.157437397	-0.00318328	0.005208081	0.021756931	0.045071878	9	0	0
ACADL	0.2652236	0.28971182	-0.02448825	0.031323608	0.021769289	0.04509227	12	0	0
GRHL2	0.1495397	0.166470996	-0.01693133	-0.11473979	0.021784399	0.045118357	13	0	0
TAGLN2	0.2678224	0.26643031	0.001392051	0.063354695	0.021792334	0.045129581	8	0	0
MRPL33	0.0981037	0.111529446	-0.0134257	-0.0762409	0.021796078	0.045132125	16	0	0
MANEA	0.1114348	0.123769841	-0.01233503	-0.11657546	0.021800636	0.045136352	7	0	0
TMEM198	0.1103704	0.127365829	-0.01699539	-0.08664622	0.021839047	0.045205442	10	0	0
CNIH1	0.2305628	0.243342489	-0.0127797	-0.07235881	0.021837372	0.045205442	11	0	0
STX16	0.105796	0.063164856	0.042631097	0.47669653	0.021844572	0.04521166	9	0	0
HMGCR	0.3673066	0.399892969	-0.03258639	-0.05878102	0.021859091	0.04523649	2	0	0
MRPS17	0.119397	0.142096804	-0.02269982	-0.04908604	0.021891019	0.045297336	8	0	0
SCN7A	0.2699444	0.240733071	0.029211326	0.158581896	0.021942969	0.045399596	2	0	0
CXCL2	0.3106586	0.30973793	0.00092062	0.168545634	0.021947252	0.04540322	6	0	0
HSPB3	0.7476002	0.772133539	-0.02453335	-0.05298591	0.021950716	0.045405149	2	0	0
INSRR	0.2184302	0.179616295	0.038813884	0.309149316	0.021958963	0.045416969	13	0	0
RHOD	0.2501322	0.255499044	-0.00536684	0.188273023	0.021969806	0.045434157	14	0	0
KIFC1	0.3973351	0.409968769	-0.01263365	0.001371204	0.021980635	0.04545131	25	0	0
NLRP14	0.1203537	0.091239115	0.029114612	0.410284104	0.021987319	0.045454652	19	0	0
YIPF5	0.228019	0.241286257	-0.01326723	-0.02529525	0.02200777	0.045491686	11	0	0
THEG	0.6847837	0.681198276	0.003585399	0.005095412	0.022016549	0.045504589	6	0	0
SLC16A6	0.0508027	0.043847486	0.006955168	0.088161732	0.022034481	0.045536405	11	0	0
NEDD8	0.0622944	0.060725686	0.001568761	0.056159761	0.02204403	0.04555089	9	0	0
IFI6	0.1749167	0.176400168	-0.00148351	0.06405948	0.022055185	0.045568691	6	0	0
TYROBP	0.4602349	0.482850411	-0.02261554	-0.0630336	0.022083253	0.045621427	8	0	0
BTG1	0.053058	0.065054924	-0.01199688	-0.08123192	0.02208589	0.045621619	13	0	0
PKD1	0.0213582	0.019953342	0.001404847	0.066125279	0.022101038	0.045647654	1	0	0
DIMT1	0.1864728	0.203042498	-0.01656967	-0.00302327	0.022132132	0.045701743	11	0	0
CCDC91	0.7784256	0.816675097	-0.03824945	-0.07942744	0.022132322	0.045701743	3	0	0
C11orf87	0.4108972	0.417859366	-0.00696213	0.111578924	0.022136647	0.04570541	9	0	0
FOXA1	0.1638451	0.126097217	0.037747865	0.475316081	0.02214161	0.045710395	10	0	0
SQSTM1	0.1171705	0.12650329	-0.00933276	-0.00793526	0.022169368	0.045762433	7	0	0
KLHDC9	0.0722074	0.046096786	0.026110568	0.509147227	0.022178171	0.045775337	10	0	0

FAM53B	0.2260315	0.228251198	-0.00221973	0.019541654	0.022186214	0.045786669	7	0	0
SLC4A3	0.3817561	0.362550701	0.019205433	0.090119906	0.022190116	0.045789453	11	0	0
ARF3	0.0645176	0.073491072	-0.00897345	-0.05404864	0.022197247	0.045798898	8	0	0
TUBA3C	0.8451072	0.864146135	-0.01903897	-0.03451228	0.022211137	0.045822287	3	0	0
CYP19A1	0.7122713	0.738895801	-0.02662451	-0.0551472	0.022223522	0.045842564	4	0	0
PTPN7	0.497815	0.525546569	-0.02773162	-0.07262467	0.022247088	0.045885898	7	0	0
TAF1D	0.066465	0.062431756	0.004033263	0.067894602	0.022252857	0.045892519	12	0	0
RBM5	0.2066537	0.229227935	-0.02257424	-0.11460759	0.022268188	0.045918858	8	0	0
USB1	0.3670899	0.376149659	-0.00905975	0.015020011	0.022282391	0.045942863	7	0	0
ADARB1	0.5321193	0.550047917	-0.01792858	-0.03717973	0.022291723	0.045956822	5	0	0
EMC3	0.1398189	0.148240279	-0.00842139	-0.0131644	0.022299363	0.045963867	10	0	0
CACNA1H	0.0544304	0.045882206	0.008548193	0.228909384	0.022300266	0.045963867	7	0	0
OVOL1	0.2285837	0.213146756	0.015436949	0.07544907	0.022304778	0.045967884	10	0	0
CAMK2A	0.5428469	0.544577781	-0.00173085	0.035990113	0.022331465	0.046017596	8	0	0
ZDHHC19	0.688706	0.667602415	0.0211036	0.049930807	0.02233622	0.046022107	5	0	0
NUDT7	0.0881741	0.097862394	-0.00968826	-0.01744552	0.022341914	0.046028551	9	0	0
MCOLN2	0.1554295	0.117275373	0.038154144	0.45319172	0.022349732	0.046039369	7	0	0
WDR19	0.1524239	0.168824836	-0.01640091	-0.02432784	0.02236032	0.04605589	10	0	0
SNAPC5	0.2393525	0.25971398	-0.02036149	-0.07524501	0.022366083	0.046062472	7	0	0
TMEM150C	0.2340581	0.238318503	-0.00426045	0.01266715	0.022393141	0.046112902	8	0	0
PRKCZ	0.1176722	0.123795417	-0.0061232	-0.00415801	0.022401257	0.04612432	9	0	0
MCM10	0.070984	0.080671819	-0.00968782	-0.03171419	0.02243949	0.046197738	10	0	0
HIST2H2AA3	0.0399389	0.043011262	-0.00307239	-0.06288793	0.022443195	0.046200062	4	0	0
MUC5B	0.5187672	0.546079265	-0.0273121	-0.06031373	0.022446065	0.046200668	9	0	0
FAM3D	0.7489047	0.758044787	-0.00914011	-0.0182393	0.022453089	0.046204521	4	0	0
ZNF225	0.2431616	0.190907019	0.052254567	0.298362863	0.022451784	0.046204521	2	0	0
CCDC173	0.1882118	0.206460008	-0.01824817	-0.06801552	0.022492024	0.046279333	11	0	0
ATG10	0.0806445	0.075296313	0.005348172	0.036858979	0.022529737	0.046349423	6	0	0
TRMT10B	0.6312845	0.632050237	-0.00076574	0.078474839	0.022531257	0.046349423	3	0	0
RCAN2	0.1875706	0.216380899	-0.02881033	-0.06259303	0.022541171	0.046364501	10	0	0
UBR3	0.3406493	0.353858944	-0.01320967	0.022066241	0.022555433	0.046388517	11	0	0
VAMP1	0.2113898	0.225427175	-0.01403741	0.038384818	0.022560209	0.046390656	8	0	0
EMID1	0.2024354	0.209186242	-0.00675089	0.010113546	0.022561647	0.046390656	13	0	0
CCDC153	0.6521493	0.683391856	-0.03124252	-0.04886599	0.022603046	0.046470453	5	0	0
CNIH4	0.3633559	0.384777036	-0.02142113	-0.03407223	0.022614225	0.046488107	11	0	0
PPP2R5C	0.0859265	0.076255719	0.009670733	0.113920589	0.022621869	0.046498492	11	0	0
FAM83B	0.2759169	0.269301262	0.006615646	0.194218462	0.022634609	0.046519348	9	0	0
SCN3A	0.3571576	0.397986497	-0.0408289	-0.1100748	0.022638501	0.046520563	5	0	0
SSNA1	0.2062289	0.229817747	-0.02358882	-0.01778688	0.022640388	0.046520563	11	0	0
PNPLA2	0.4723292	0.495883477	-0.02355427	-0.06867434	0.022653803	0.046534579	8	0	0
DIP2B	0.1980829	0.220268483	-0.02218558	-0.02444747	0.022654993	0.046534579	10	0	0
B3GNT5	0.232881	0.245067592	-0.01218664	0.040558573	0.022672371	0.046564943	14	0	0
MAP2K3	0.1292925	0.135568733	-0.00627628	-0.02176666	0.022676094	0.046567258	18	0	0
TRMT5	0.1575791	0.178883441	-0.02130436	-0.04809421	0.022686449	0.046583188	14	0	0
NME4	0.2667594	0.294624704	-0.02786527	-0.03733915	0.022711265	0.046623703	4	0	0
C19orf67	0.797421	0.808695341	-0.01127432	-0.0181736	0.022711379	0.046623703	11	0	0
SLC25A15	0.4025225	0.412992661	-0.01047013	-0.02648421	0.022720626	0.046637347	7	0	0
CCDC85A	0.3672855	0.384706851	-0.01742134	0.013114531	0.022728997	0.046643855	14	0	0
CYP2W1	0.5618748	0.595866197	-0.03399143	-0.08449616	0.022728708	0.046643855	9	0	0
SCRT1	0.1990215	0.169609511	0.029411953	0.16422396	0.022737378	0.046655716	7	0	0
MECOM	0.1470309	0.129029947	0.01800094	0.02954428	0.022743423	0.046662782	10	0	0
GTSF1L	0.7429574	0.775380214	-0.03242281	-0.07015765	0.022748647	0.046668161	7	0	0
XRCC6	0.0574697	0.060587677	-0.00311795	0.022419857	0.022761821	0.046689847	15	0	0
LRRC38	0.1915938	0.167944371	0.023649458	0.264485971	0.02278841	0.046739042	7	0	0
FBLN2	0.6736898	0.683845448	-0.01015561	-0.01740405	0.022803478	0.046764598	3	0	0
NDUFC1	0.1725848	0.162000763	0.010583995	0.169605809	0.022834609	0.046823089	11	0	0
PACRG	0.388602	0.388041841	0.000560135	0.074303358	0.022839872	0.046828526	8	0	0
GPATCH3	0.1507193	0.168154029	-0.01743477	-0.06494773	0.022860072	0.046840149	11	0	0
TRIM39-RPF	0.8270651	0.86048591	-0.03342077	-0.05859371	0.022857902	0.046840149	12	0	0
GJA1	0.5317389	0.579859209	-0.04812034	-0.11635555	0.022863822	0.046840149	3	0	0
CPVL	0.2610542	0.25880929	0.002244893	0.210118392	0.022858012	0.046840149	10	0	0
ZNF107	0.3918616	0.422982894	-0.03112127	-0.01442785	0.022854361	0.046840149	7	0	0
MNAT1	0.2092285	0.221039225	-0.0118107	0.050848147	0.022854588	0.046840149	10	0	0

CHST5	0.6986898	0.685210902	0.013478909	0.045607603	0.022862186	0.046840149	4	0	0
TMED10	0.1302796	0.126410012	0.003869622	0.088998874	0.02287186	0.046851266	10	0	0
F3	0.0618557	0.049135197	0.012720549	0.231495407	0.022896314	0.046890646	13	0	0
C11orf96	0.7097778	0.704206656	0.005571113	0.013859028	0.022895118	0.046890646	5	0	0
S1PR5	0.124382	0.111610592	0.012771418	0.230405025	0.022911933	0.046917277	12	0	0
IGFBP4	0.1185152	0.090602859	0.027912357	0.477401374	0.022917971	0.046924284	8	0	0
ARIH1	0.0352045	0.032051161	0.003153305	0.089701135	0.022922094	0.046927369	9	0	0
C9orf16	0.1147306	0.135403393	-0.02067283	-0.01867898	0.022939279	0.046957191	6	0	0
LMBR1	0.103954	0.105476225	-0.00152224	0.060851272	0.02295391	0.046981779	3	0	0
NEURL4	0.1089819	0.121556382	-0.01257448	-0.06122095	0.022979966	0.047029743	6	0	0
SYNE2	0.1717561	0.183216215	-0.01146014	-0.0596144	0.022983938	0.047030648	9	0	0
MCM5	0.1218458	0.144791692	-0.02294589	-0.06816948	0.022985652	0.047030648	8	0	0
CD177	0.6062325	0.626704849	-0.02047236	-0.04121754	0.022997841	0.04705022	7	0	0
EXOC3L4	0.7082338	0.713229038	-0.00499527	-0.0064581	0.023014835	0.047079618	10	0	0
LIPT1	0.0688179	0.08317641	-0.01435852	-0.03632485	0.023034817	0.047115119	10	0	0
SMCP	0.7131139	0.721712449	-0.00859855	0.065168147	0.023045523	0.047131642	7	0	0
MYO16	0.406418	0.448218347	-0.04180031	-0.15086107	0.023051553	0.047133226	4	0	0
GREB1L	0.1273033	0.093506064	0.033797226	0.528546345	0.023050898	0.047133226	8	0	0
KPNA6	0.2146443	0.218713759	-0.0040695	0.045442664	0.023059092	0.047143266	14	0	0
APOE	0.2970133	0.311397627	-0.01438435	0.089400746	0.023082374	0.047185487	7	0	0
ZNF12	0.0869159	0.101350743	-0.01443483	-0.09066734	0.023092528	0.047199847	9	0	0
PIK3R5	0.3137133	0.314715062	-0.00100177	0.328369154	0.023094662	0.047199847	12	0	0
ZNF33A	0.0465927	0.044121954	0.002470795	0.051298147	0.023137462	0.047281933	10	0	0
DBF4	0.0534854	0.06080777	-0.00732239	-0.02447641	0.02315164	0.047305515	9	0	0
WNT5A	0.3222423	0.300272796	0.021969461	0.255265908	0.02316431	0.047321212	11	0	0
ATP6V0C	0.181873	0.180320311	0.001552666	0.072892778	0.023164599	0.047321212	13	0	0
LRRC8D	0.1273087	0.141736324	-0.01442765	-0.02275758	0.023195575	0.047379095	7	0	0
SYPL2	0.192014	0.155793324	0.036220643	0.437331292	0.02322202	0.047427711	9	0	0
CSNK1D	0.1893582	0.19864554	-0.00928739	-0.03124704	0.023228435	0.047435411	12	0	0
RLBP1	0.7406345	0.773392052	-0.03275758	-0.06266839	0.023246328	0.047466545	9	0	0
SERTAD3	0.2001177	0.233354867	-0.03323721	-0.0555317	0.023257119	0.047483175	4	0	0
TCP11	0.619634	0.664767295	-0.04513326	-0.10851996	0.023261022	0.047485737	4	0	0
ABR	0.1779891	0.17522654	0.002762565	0.457044511	0.023268623	0.047490444	6	0	0
ZNF790	0.1877548	0.171986587	0.015768165	0.363924539	0.023268517	0.047490444	18	0	0
MTUS1	0.0551508	0.050167678	0.00498315	0.164306678	0.023294992	0.047538853	11	0	0
DMRT2	0.1284082	0.102641531	0.025766688	0.287043807	0.023306174	0.047556262	14	0	0
MAP3K19	0.7575707	0.790892593	-0.03332192	-0.05774309	0.02332695	0.04759324	2	0	0
GABRG1	0.2112451	0.177901136	0.03334401	0.226454541	0.023336917	0.047608159	8	0	0
MIS18BP1	0.2637113	0.276706238	-0.01299499	-0.03498122	0.023376732	0.047683961	10	0	0
FBXO8	0.2553206	0.26527285	-0.00995221	-0.0257359	0.023387294	0.047700081	15	0	0
CMAS	0.3741626	0.388321151	-0.01415853	-0.03851506	0.023402305	0.047725269	10	0	0
TRIM41	0.1467654	0.169508099	-0.02274273	-0.07148271	0.023443279	0.047803394	7	0	0
PNOC	0.6014309	0.619889305	-0.0184584	-0.04023621	0.023458375	0.047828738	6	0	0
FBXO33	0.1281225	0.150663969	-0.02254143	-0.06883418	0.023475943	0.047859117	6	0	0
TNIP3	0.7813751	0.809388536	-0.02801348	-0.0503617	0.023494931	0.047888966	3	0	0
ABL1	0.2070133	0.180602661	0.026410615	0.177815963	0.023498595	0.047888966	7	0	0
ZNF561	0.1392214	0.123817199	0.015404187	0.551224881	0.023496089	0.047888966	8	0	0
WRAP73	0.1638286	0.177150781	-0.01332221	-0.12553929	0.023510567	0.047896811	3	0	0
SLC18A1	0.5674636	0.596695802	-0.02923224	-0.05853249	0.023505676	0.047896811	5	0	0
SLC6A15	0.4351173	0.453706962	-0.01858963	-0.01721552	0.023511903	0.047896811	8	0	0
ASGR2	0.5977458	0.635501167	-0.03775537	-0.09819732	0.023513126	0.047896811	4	0	0
CSNK1G3	0.2162528	0.239050106	-0.0227973	-0.06418234	0.023522747	0.04790634	14	0	0
PTPN1	0.1518022	0.172395646	-0.02059343	-0.07799119	0.023523145	0.04790634	13	0	0
POC1A	0.0467746	0.048421643	-0.00164706	-0.00318708	0.023528517	0.04791184	11	0	0
SLC12A5	0.325085	0.2931963	0.031888651	0.400603517	0.0235376	0.047924894	10	0	0
NTNG2	0.1559669	0.129849469	0.026117385	0.385293445	0.023558323	0.047961643	11	0	0
CRIP1	0.5131605	0.491199826	0.021960679	0.080163428	0.023563407	0.047965618	8	0	0
ZHX3	0.2347194	0.270604529	-0.03588513	-0.05378796	0.023565623	0.047965618	6	0	0
ADAMTS15	0.0559049	0.050093368	0.005811501	0.157156678	0.023572334	0.047970864	11	0	0
DMKN	0.4785356	0.499336872	-0.02080128	-0.03812737	0.02357355	0.047970864	7	0	0
RPS6KA1	0.3471819	0.342735288	0.004446631	0.010968442	0.023581912	0.04797475	7	0	0
APBA3	0.0913597	0.095345215	-0.00398552	0.050508326	0.023583484	0.04797475	14	0	0
MRPL54	0.0913597	0.095345215	-0.00398552	0.050508326	0.023583484	0.04797475	14	0	0

ABRACL	0.0700813	0.086896484	-0.01681517	-0.10488399	0.023598658	0.048000174	11	0	0
CAMKV	0.3781757	0.374926285	0.003249445	0.157191641	0.023609991	0.048017779	11	0	0
LDHAL6A	0.7077019	0.697157167	0.010544688	0.027632827	0.023615691	0.048023927	5	0	0
KANK2	0.2158769	0.199820939	0.01605598	0.085314184	0.023621588	0.048030474	11	0	0
TSC1	0.3213394	0.335512211	-0.01417279	-0.04889072	0.0236382	0.048053355	7	0	0
GFI1B	0.3213394	0.335512211	-0.01417279	-0.04889072	0.0236382	0.048053355	7	0	0
MAP4K2	0.3789607	0.403595802	-0.02463506	-0.05554466	0.02365026	0.048066976	9	0	0
ATXN3	0.2279092	0.241539018	-0.01362977	-0.04753511	0.023650003	0.048066976	12	0	0
DNAJC18	0.5791527	0.582253999	-0.00310131	0.03524866	0.023653824	0.048068774	2	0	0
SP140L	0.1867738	0.198814092	-0.01204028	0.107200179	0.023707871	0.048169981	9	0	0
NPM2	0.2505835	0.219078637	0.031504896	0.469506951	0.023710932	0.048169981	14	0	0
TMX1	0.2344305	0.25340532	-0.01897484	-0.01094502	0.023711684	0.048169981	4	0	0
PPIL6	0.3209397	0.328686108	-0.00774638	-0.05300094	0.023734133	0.048206239	6	0	0
CADPS2	0.0725891	0.058779169	0.013809937	0.258858658	0.023737595	0.048206239	17	0	0
ZBTB42	0.2857577	0.27205275	0.013704926	0.074030607	0.023737578	0.048206239	7	0	0
ASAP1	0.1356807	0.151323879	-0.01564317	-0.05493033	0.02375735	0.04823598	10	0	0
CTTN	0.0501888	0.057609367	-0.00742052	-0.12095892	0.023757618	0.04823598	8	0	0
CCDC171	0.0348833	0.034895397	-1.21E-05	0.01455981	0.023776024	0.048267885	7	0	0
MT1B	0.620157	0.635544608	-0.01538759	-0.03197659	0.023789328	0.048279249	6	0	0
CLUH	0.3979903	0.399126232	-0.0011359	0.132828022	0.023784565	0.048279249	3	0	0
SNAI1	0.1502736	0.155017123	-0.00474354	0.330325319	0.023789697	0.048279249	12	0	0
KIF5A	0.4821059	0.488925838	-0.00681993	0.169614079	0.023797323	0.048289261	8	0	0
SGPL1	0.4390144	0.460554771	-0.02154034	-0.03710555	0.023800075	0.048289383	7	0	0
TMEM52B	0.6767535	0.713404899	-0.03665135	-0.07345625	0.023820748	0.048325861	3	0	0
COX6B2	0.1156461	0.090886681	0.024759391	0.417994548	0.023825615	0.048330268	12	0	0
TTC8	0.2958697	0.320479379	-0.02460969	-0.07015499	0.02382956	0.048332803	14	0	0
CAMK1G	0.3239616	0.331449671	-0.00748805	0.059065645	0.023834549	0.048337458	4	0	0
TAX1BP1	0.2741553	0.297591438	-0.02343611	-0.02344407	0.02383987	0.048342782	7	0	0
TSC22D2	0.0540775	0.055752973	-0.00167546	-0.01859091	0.023858022	0.048363593	6	0	0
SP8	0.2196019	0.187381973	0.032219971	0.175510409	0.023866311	0.048363593	8	0	0
CDKN2A	0.1060962	0.131544747	-0.02544851	0.098125098	0.023861613	0.048363593	8	0	0
SLC35F4	0.731516	0.756191005	-0.02467497	-0.03936234	0.023857645	0.048363593	2	0	0
TMEM161A	0.1584155	0.161787392	-0.00337187	0.01822078	0.023863089	0.048363593	12	0	0
C19orf47	0.1272987	0.150280034	-0.02298129	-0.00590702	0.023866039	0.048363593	13	0	0
ABCA7	0.171072	0.180185599	-0.00911356	-0.06767552	0.023871683	0.048369014	5	0	0
ACAP2	0.2235489	0.240320136	-0.01677123	-0.02268218	0.023877787	0.048375917	5	0	0
NCKAP1	0.2700209	0.252791446	0.017229474	0.145219038	0.023883968	0.048380088	12	0	0
RNF44	0.1336965	0.112167402	0.021529087	0.073139433	0.023885241	0.048380088	8	0	0
IQCF1	0.7726163	0.806842915	-0.03422665	-0.06318939	0.023895885	0.048396183	2	0	0
EPRS	0.3748727	0.383867537	-0.00899486	0.035893293	0.023899562	0.048398165	10	0	0
PLEKHH1	0.2208473	0.23472801	-0.01388073	0.019699857	0.02390705	0.048407862	11	0	0
IMPACT	0.3035851	0.284928528	0.018656582	0.374100929	0.023915383	0.048419269	11	0	0
INSR	0.1060529	0.099749728	0.006303134	0.173106486	0.023945118	0.048468529	9	0	0
KLHL9	0.170056	0.183028803	-0.01297275	-0.0663052	0.023972583	0.048518646	7	0	0
AMFR	0.253514	0.270900484	-0.01738648	0.006565263	0.023976608	0.048521245	7	0	0
ZNF606	0.1709281	0.141999684	0.028928368	0.437611388	0.023979277	0.048521245	7	0	0
LTBP2	0.1180872	0.100823097	0.01726414	0.246060245	0.023992008	0.048531339	9	0	0
PSKH1	0.2361484	0.251931532	-0.01578315	0.004434753	0.023992383	0.048531339	7	0	0
DTNA	0.2096227	0.223406522	-0.0137838	0.098331102	0.023987134	0.048531339	8	0	0
PPDPF	0.254271	0.266056843	-0.01178583	0.276572589	0.02399559	0.048532352	3	0	0
CNBP	0.1270879	0.148394465	-0.02130654	-0.06355444	0.024011515	0.048551722	8	0	0
ALOX12B	0.7839829	0.806968198	-0.02298531	-0.04037667	0.024013288	0.048551722	7	0	0
MAFF	0.2429905	0.232364221	0.01062624	0.065183785	0.02400996	0.048551722	11	0	0
C2CD4B	0.2616713	0.219962445	0.041708816	0.497212124	0.02402184	0.048563539	8	0	0
IQCA1	0.5874629	0.607566106	-0.02010317	-0.04347952	0.024032194	0.048577756	11	0	0
C4orf48	0.0450413	0.04445002	0.000591282	0.114590162	0.024040431	0.048577756	13	0	0
CYP26A1	0.1001314	0.075822285	0.024309089	0.323841169	0.024041669	0.048577756	15	0	0
TOX2	0.1911312	0.191682259	-0.00055104	0.192855003	0.024036477	0.048577756	13	0	0
GNAS	0.5818749	0.604786287	-0.0229114	-0.05223202	0.024042414	0.048577756	17	0	0
SAPCD1	0.7673561	0.777652432	-0.01029634	-0.01623436	0.024061139	0.048610114	6	0	0
DNAJB1	0.0530818	0.04914582	0.003935992	0.077041129	0.024087205	0.048657294	7	0	0
KCNQ4	0.1698907	0.168815635	0.001075095	0.232413909	0.024101977	0.048681651	10	0	0
TSSK4	0.8811554	0.889044341	-0.0078889	-0.01320329	0.024114522	0.048701505	10	0	0

NT5C3B	0.3755311	0.391089849	-0.01555875	0.081050813	0.02416097	0.048789818	6	0	0
CDCP1	0.4038653	0.427656992	-0.02379165	-0.11610376	0.024166117	0.048794717	4	0	0
DOPEY1	0.1470903	0.173593767	-0.02650344	-0.09663595	0.024202717	0.048863116	9	0	0
EYA2	0.3483156	0.362911747	-0.01459618	0.002217087	0.024210466	0.04887326	10	0	0
DPYSL5	0.0737486	0.056078328	0.017670244	0.403285623	0.02421657	0.048874581	8	0	0
RAB11FIP4	0.297417	0.28311934	0.014297637	0.219453356	0.024215976	0.048874581	5	0	0
TPI1	0.2005419	0.216645922	-0.01610403	0.008819058	0.024235984	0.04890826	11	0	0
WDR83	0.0737356	0.0835021	-0.00976649	-0.03301198	0.024242742	0.048916394	21	0	0
FGF20	0.2903073	0.243744942	0.046562336	0.313581786	0.024278543	0.048983122	11	0	0
PITPNM3	0.4019315	0.408075334	-0.00614386	0.060642232	0.024281518	0.048983614	5	0	0
TRIP13	0.1712488	0.194927874	-0.02367906	-0.06469152	0.02429839	0.049010662	14	0	0
SIAH3	0.3742029	0.386582704	-0.01237978	0.023661011	0.024300391	0.049010662	14	0	0
MYO3A	0.5048834	0.484628215	0.020255197	0.213243222	0.024330943	0.049066764	10	0	0
MRPS21	0.2642784	0.230573691	0.033704707	0.767867123	0.024356966	0.049109679	12	0	0
KCNE1	0.7752753	0.799004045	-0.02372873	-0.04487896	0.0243577	0.049109679	7	0	0
TJP1	0.2223191	0.211269199	0.011049857	0.061752801	0.024365783	0.049120454	2	0	0
NSD1	0.4892527	0.483384783	0.005867914	0.081335959	0.024376043	0.049130094	7	0	0
MAML2	0.1412245	0.161180122	-0.0199556	-0.0854802	0.024374594	0.049130094	7	0	0
BAIAP3	0.1706797	0.132701055	0.037978656	0.384541066	0.024382326	0.049137237	7	0	0
ZNF219	0.5474375	0.526694397	0.020743071	0.078205744	0.024395775	0.049153294	4	0	0
CGNL1	0.2893979	0.284044654	0.005353288	0.274540431	0.024394511	0.049153294	7	0	0
MEAF6	0.1060298	0.107331312	-0.0013015	0.029069603	0.024425171	0.049206994	11	0	0
CSMD1	0.3272679	0.303028141	0.024239806	0.183951346	0.024477202	0.049306277	7	0	0
MMD	0.2518581	0.278941761	-0.02708365	-0.09740886	0.024500819	0.049348308	6	0	0
HES5	0.1112388	0.053825653	0.057413103	1.145408426	0.024524896	0.049391257	11	0	0
TBC1D20	0.1204525	0.080641226	0.039811311	0.335498186	0.024553395	0.0494431	9	0	0
SEC61G	0.3858658	0.420642899	-0.03477715	-0.0894273	0.024565565	0.049462053	5	0	0
E2F4	0.2303909	0.207419117	0.022971779	0.050112567	0.024580494	0.049486557	11	0	0
ZNF324	0.120868	0.13704237	-0.01617433	-0.14299402	0.024594416	0.04950903	9	0	0
ADCY7	0.2574569	0.246262701	0.011194231	0.054491472	0.024605303	0.049525386	9	0	0
SNAP25	0.1021683	0.065406302	0.036761971	0.450868384	0.024608962	0.049527193	13	0	0
FMO1	0.7632603	0.758396925	0.004863385	0.005612453	0.024621886	0.049547644	6	0	0
CAPS2	0.0849748	0.092296958	-0.00732217	-0.09467928	0.024644874	0.049588339	11	0	0
LYSMD2	0.1506419	0.163536979	-0.01289506	-0.03448985	0.024648591	0.049590256	16	0	0
RCOR1	0.0715526	0.070107038	0.001445595	-0.01394513	0.024682272	0.049652448	11	0	0
CAMK1D	0.150181	0.134526828	0.015654141	0.262779915	0.024693705	0.049669878	8	0	0
MET	0.1676629	0.188186724	-0.02052383	-0.09421155	0.024697919	0.049672783	11	0	0
PSAT1	0.1851864	0.170062259	0.015124169	0.102401402	0.024703059	0.049677549	8	0	0
SERPINA5	0.6011118	0.646048162	-0.04493638	-0.10799038	0.024711304	0.049688558	9	0	0
JTB	0.222039	0.243835016	-0.02179599	-0.03758295	0.024722886	0.049706275	17	0	0
NUAK1	0.3606749	0.392435544	-0.03176061	-0.10710433	0.024731438	0.049712324	9	0	0
HEATR3	0.6398565	0.656512552	-0.01665608	-0.04861216	0.024728965	0.049712324	5	0	0
HS3ST3B1	0.0780891	0.067196353	0.010892704	0.272642002	0.024734592	0.049713093	11	0	0
AOC3	0.4948804	0.499196478	-0.00431604	0.008711136	0.024782316	0.04980343	8	0	0
NXPE3	0.1194027	0.134644856	-0.01524216	0.112868358	0.024820755	0.049869504	14	0	0
DAZAP1	0.1301768	0.146735665	-0.01655883	-0.09788438	0.024833862	0.04989025	8	0	0
C9orf66	0.844993	0.856056888	-0.01106388	-0.01850486	0.024852405	0.049917404	3	0	0
B4GALNT1	0.0674479	0.08010193	-0.01265404	-0.08622207	0.024852945	0.049917404	11	0	0
GRB2	0.1412202	0.156650862	-0.01543067	-0.03880458	0.02486823	0.049942512	11	0	0
PTGER1	0.1829886	0.142654878	0.040333684	0.482793423	0.024890346	0.04998133	5	0	0
EIF2AK4	0.3850981	0.411337278	-0.02623921	-0.03659489	0.024899214	0.049993541	3	0	0
LAMC1	0.1440912	0.172421411	-0.02833017	-0.01949275	0.024902263	0.049994066	7	0	0
DCLRE1B	0.0373022	0.04055904	-0.00325679	-0.00783719	0.024909819	0.049999913	10	0	0
PPP2R3A	0.2641593	0.231093117	0.033066145	0.135087039	0.024910751	0.049999913	6	0	0
SCN4B	0.1927897	0.180099667	0.012690038	0.240811591	0.024916721	0.050006301	9	0	0
EEPD1	0.1898597	0.203962171	-0.01410252	-0.01272138	0.024943789	0.050055024	5	0	0
CCDC66	0.0293435	0.029277258	6.63E-05	0.006666375	0.024957832	0.050073927	7	0	0
PNLDC1	0.8738865	0.912654438	-0.03876798	-0.06228668	0.024958793	0.050073927	8	0	0
AES	0.4735688	0.473278389	0.000290432	0.107763303	0.024978626	0.050108112	6	0	0
PDE6A	0.788655	0.812060275	-0.02340531	-0.0430751	0.025036161	0.050206684	5	0	0
ZC2HC1B	0.8222622	0.841933107	-0.01967088	-0.03632343	0.025035653	0.050206684	5	0	0
TAOK2	0.2192389	0.200249926	0.018988986	0.144202524	0.025034203	0.050206684	9	0	0
ADCY9	0.0456336	0.035548173	0.010085456	0.249564821	0.025063586	0.050256062	6	0	0

FBXO24	0.3764826	0.349832198	0.026650377	0.160186524	0.025077956	0.050279256	8	0	0
IMPG2	0.810985	0.827067147	-0.01608214	-0.03115214	0.025096545	0.050294037	6	0	0
PEG10	0.2170998	0.199675273	0.017424503	0.389050556	0.025091701	0.050294037	52	0	0
ANKRD46	0.1236363	0.135755896	-0.01211957	-0.02980113	0.025095417	0.050294037	9	0	0
GALNT12	0.4864156	0.50246671	-0.01605115	-0.00545055	0.025094065	0.050294037	7	0	0
MRPS18C	0.1022473	0.096062552	0.006184781	0.070042872	0.025101604	0.050297672	11	0	0
TNFSF13	0.3075432	0.279802555	0.027740692	0.303035493	0.025104449	0.050297672	13	0	0
S100A6	0.1168896	0.130737846	-0.01384828	-0.05992243	0.025114145	0.050306824	12	0	0
GYG1	0.1620441	0.175315307	-0.01327119	-0.05376442	0.025129474	0.050331909	14	0	0
AP4B1	0.051888	0.058872159	-0.00698413	-0.04624213	0.025141856	0.050351087	10	0	0
EAF1	0.2136648	0.214048802	-0.00038396	0.019537122	0.025168504	0.050398826	10	0	0
CPNE9	0.5817427	0.568207281	0.013535454	0.094568429	0.025195222	0.050441066	10	0	0
CARD10	0.184702	0.201447939	-0.01674592	0.059974994	0.025193123	0.050441066	10	0	0
DTHD1	0.7134528	0.749375362	-0.0359226	-0.06742078	0.025213745	0.050472515	6	0	0
DHX9	0.1469864	0.167762502	-0.02077614	-0.06896317	0.025221882	0.050483168	15	0	0
MTA3	0.1797416	0.15958432	0.020157269	0.108275874	0.025232595	0.050498975	10	0	0
RAMP3	0.3282778	0.331017074	-0.00273926	0.224407884	0.025238563	0.050505285	7	0	0
ZMYM6	0.2081129	0.22253509	-0.01442217	-0.02788294	0.025243882	0.050510292	9	0	0
BLMH	0.3341406	0.337638309	-0.00349767	-0.01720445	0.025247047	0.05051099	6	0	0
TMPRSS6	0.7773361	0.809687091	-0.032351	-0.05914741	0.025252223	0.050515713	6	0	0
SRP68	0.2548785	0.285558823	-0.0306803	-0.04032795	0.025256065	0.050517764	10	0	0
GNG10	0.27786	0.282135468	-0.00427542	0.045111289	0.025263299	0.050520964	7	0	0
PLBD1	0.3886534	0.423501349	-0.03484797	-0.04865783	0.025284436	0.050557597	12	0	0
FAM76A	0.1214699	0.137515381	-0.01604551	-0.01370079	0.025315234	0.050613537	12	0	0
PCDHGC3	0.1289883	0.121954792	0.007033543	0.167474007	0.025335344	0.050636809	10	0	0
COMMD6	0.1197633	0.118827606	0.000935686	-0.00041584	0.025334295	0.050636809	11	0	0
SULT4A1	0.2215934	0.178468319	0.043125094	0.410563914	0.025329943	0.050636809	7	0	0
MLKL	0.0512842	0.04647791	0.004806279	-0.0040847	0.025351811	0.050658432	3	0	0
KRT9	0.6816551	0.69979237	-0.01813728	-0.02796436	0.025349742	0.050658432	9	0	0
PPP1R7	0.1144668	0.123506823	-0.00904001	-0.05473401	0.025373757	0.050696637	11	0	0
CDK7	0.0371666	0.036119669	0.001046974	0.045693352	0.025395724	0.050734876	13	0	0
STAU1	0.2152557	0.210607329	0.004648353	0.093606402	0.025402633	0.050743026	9	0	0
ONECUT3	0.0852555	0.072564174	0.012691304	0.079678146	0.025420934	0.050773928	6	0	0
SSR2	0.1773362	0.179098708	-0.0017625	0.021087858	0.025428001	0.050782388	10	0	0
CARF	0.0521505	0.047074574	0.005075972	0.124339705	0.025463883	0.050843643	7	0	0
PARD3B	0.0696602	0.059772703	0.009887528	0.243465031	0.025469593	0.050843643	11	0	0
NDST4	0.5590182	0.550865165	0.008153079	0.029078665	0.025469155	0.050843643	6	0	0
GLRA1	0.2506432	0.252994515	-0.00235133	0.097862961	0.025470012	0.050843643	11	0	0
RAD21L1	0.1905575	0.196374216	-0.00581669	0.247991746	0.025476666	0.050851267	12	0	0
GRM3	0.1658787	0.151915069	0.013963638	0.256508963	0.02549172	0.050875653	11	0	0
FAM217B	0.3535959	0.34077781	0.012818089	0.314605084	0.025501521	0.05088955	10	0	0
CDC42BPB	0.1061715	0.075592714	0.030578779	0.24302348	0.025534271	0.050949236	10	0	0
SLC35F5	0.1086172	0.121937273	-0.01332008	0.013059519	0.025547254	0.050963802	11	0	0
GJD4	0.5866887	0.610017588	-0.02332891	-0.04150371	0.025546621	0.050963802	8	0	0
ELF3	0.5359516	0.564365587	-0.02841401	-0.07656598	0.025552054	0.050967709	5	0	0
ZFPL1	0.1368134	0.148240011	-0.01142658	0.008873855	0.02555507	0.050968056	11	0	0
HELT	0.0985201	0.0811737	0.017346425	0.242365557	0.025597983	0.051042293	17	0	0
FKBP11	0.5147603	0.481759346	0.033000997	0.101147499	0.025597055	0.051042293	3	0	0
PTK7	0.2460874	0.228740312	0.017347063	0.071765209	0.025632542	0.051105522	10	0	0
COLGALT1	0.097041	0.108401236	-0.01136025	-0.01792178	0.025641627	0.051117954	8	0	0
ACTL6A	0.1339489	0.157363389	-0.02341454	-0.08188162	0.025662	0.051152881	10	0	0
CDH1	0.2203983	0.231387234	-0.0109889	0.075480458	0.025679036	0.051176169	8	0	0
SERPIND1	0.8545399	0.883758184	-0.02921828	-0.04949138	0.025679389	0.051176169	6	0	0
EEF1G	0.3029212	0.290725551	0.012195668	0.102813928	0.025690454	0.051187265	7	0	0
GMFB	0.1979688	0.207694703	-0.00972588	-0.08387142	0.025690665	0.051187265	10	0	0
ZNF768	0.0557022	0.061851424	-0.00614927	-0.0369637	0.025696954	0.05119411	9	0	0
UBAC2	0.0789628	0.089549106	-0.01058628	-0.06912812	0.025712251	0.051218895	14	0	0
BAALC	0.3681592	0.353327347	0.014831819	0.318109575	0.025726133	0.051236864	7	0	0
MKS1	0.2085236	0.21760677	-0.00908315	-0.0648465	0.025726985	0.051236864	15	0	0
MRPS10	0.2635181	0.278967179	-0.01544913	0.004205874	0.025743796	0.051264653	7	0	0
DTNBP1	0.2147622	0.237114365	-0.02235212	-0.08253709	0.025767822	0.051302199	13	0	0
CYP4F8	0.5647099	0.605004759	-0.0402949	-0.09676934	0.025768372	0.051302199	6	0	0
GPHA2	0.6167289	0.643258461	-0.02652956	-0.05549588	0.025782282	0.051324197	9	0	0

HCN3	0.2580431	0.266920317	-0.00887726	-0.00593773	0.025789065	0.051331358	17	0	0
CHL1	0.2860096	0.298755834	-0.01274627	0.037760316	0.025791603	0.051331358	12	0	0
YTHDC1	0.2404981	0.274296772	-0.03379864	-0.0761066	0.025813482	0.051369201	15	0	0
CCDC73	0.8171154	0.847316934	-0.03020154	-0.05223523	0.025818061	0.051372614	6	0	0
UNC5A	0.0494708	0.032287795	0.017183033	0.493932123	0.025854932	0.051440274	4	0	0
HSPBP1	0.2816426	0.279616871	0.00202572	0.056323143	0.025859802	0.051444255	14	0	0
PTOV1	0.2090669	0.231599732	-0.0225328	-0.05000279	0.025867778	0.051454415	9	0	0
SPRN	0.1795917	0.15203985	0.02755183	0.226547	0.025878892	0.051465107	1	0	0
SYCE1	0.1795917	0.15203985	0.02755183	0.226547	0.025878892	0.051465107	1	0	0
RIMS1	0.1915072	0.195078318	-0.00357108	0.269110021	0.025882623	0.051466821	14	0	0
UBE2Q2	0.0439775	0.045301078	-0.00132358	0.096752964	0.025895644	0.051487004	3	0	0
C1QA	0.3987604	0.426723692	-0.02796327	-0.09546529	0.025916708	0.051507759	1	0	0
DOCK8	0.5559138	0.5517645	0.00414932	0.349820089	0.025915899	0.051507759	3	0	0
ROR2	0.0727504	0.05160488	0.02114557	0.504377079	0.025917641	0.051507759	9	0	0
PABPN1	0.4151999	0.429155138	-0.01395526	-0.03203643	0.025921049	0.051507759	10	0	0
PDCD7	0.0544168	0.053804366	0.000612385	0.036260252	0.025923505	0.051507759	8	0	0
UBXN6	0.1309274	0.137390058	-0.00646262	0.034968969	0.025926185	0.051507759	8	0	0
UNC13A	0.242932	0.26170527	-0.01877327	0.062729402	0.025911219	0.051507759	8	0	0
MED18	0.1165227	0.111732488	0.004790201	0.196076745	0.025939369	0.051527468	11	0	0
NSMCE1	0.286643	0.28944292	-0.00279992	0.036742519	0.025941851	0.051527468	12	0	0
FKBP1A	0.3174618	0.340799447	-0.02333761	-0.03080066	0.025977167	0.051591903	8	0	0
LIMK1	0.6969582	0.719693738	-0.02273552	-0.0470787	0.02599128	0.051614216	6	0	0
PF4V1	0.5086346	0.479094671	0.02953994	0.104320606	0.026007425	0.051640559	7	0	0
ZNF160	0.0801283	0.053362434	0.026765868	0.586469582	0.026016087	0.051652041	8	0	0
ASPDH	0.5221576	0.558737799	-0.03658018	-0.09626469	0.026019636	0.05165337	7	0	0
CMBL	0.298352	0.301986746	-0.00363475	-0.04316443	0.026025884	0.051660056	7	0	0
ZNF771	0.1218519	0.130174725	-0.00832286	-0.00013833	0.02602941	0.051661337	13	0	0
GOSR1	0.2787163	0.290872977	-0.01215663	-0.04137224	0.026041306	0.051679227	12	0	0
TM2D3	0.1370267	0.168560556	-0.03153389	-0.07844167	0.026044298	0.051679449	9	0	0
FAM160A2	0.0354252	0.037044043	-0.0016188	-0.01343709	0.02604825	0.051679911	9	0	0
CASP4	0.562313	0.530057001	0.032256018	0.088307709	0.026053176	0.051679911	2	0	0
GGA3	0.332932	0.362711461	-0.02977949	-0.09535435	0.026053164	0.051679911	6	0	0
PIBF1	0.0745515	0.095857911	-0.02130641	-0.06006762	0.026086887	0.051741061	15	0	0
GRAMD1C	0.8635857	0.847647294	0.015938455	0.030632853	0.026096983	0.051755361	3	0	0
MAPKBP1	0.1907572	0.194588479	-0.00383125	-0.07308368	0.026100864	0.051757336	11	0	0
FAM171B	0.2111453	0.229497655	-0.01835236	-0.0091018	0.026142913	0.051833916	9	0	0
SMKR1	0.3887271	0.397361514	-0.00863441	0.029747325	0.026145263	0.051833916	5	0	0
SRP54	0.1129322	0.135347659	-0.02241545	-0.04855082	0.026152349	0.051842234	6	0	0
TCTN3	0.3311329	0.341760285	-0.01062741	0.016979516	0.026162926	0.051857469	11	0	0
PTGER3	0.1933544	0.180988555	0.012365819	0.22000848	0.026175615	0.051865424	10	0	0
ATG5	0.4985127	0.533133587	-0.03462088	-0.05961155	0.026172741	0.051865424	6	0	0
RCOR2	0.1421477	0.127608545	0.014539111	0.148004647	0.026174479	0.051865424	8	0	0
CA5A	0.8289137	0.854626272	-0.02571257	-0.04678826	0.026189533	0.051887271	2	0	0
IFI27L2	0.2494505	0.264077659	-0.01462712	0.001921938	0.026202156	0.051906546	8	0	0
ZNF385B	0.1782495	0.140588082	0.037661461	0.437159211	0.0262356	0.051967059	11	0	0
PSPN	0.9303581	0.938042017	-0.00768393	-0.01174076	0.026247194	0.051984283	1	0	0
CREG2	0.1087299	0.11754915	-0.0088192	0.179371842	0.026256601	0.051991433	13	0	0
TCTN2	0.1908288	0.195975364	-0.00514656	0.042437823	0.02625453	0.051991433	10	0	0
VDR	0.698535	0.693920242	0.004614786	0.028922911	0.026265587	0.052003485	3	0	0
PRR15L	0.5635667	0.563746535	-0.00017988	-0.01423396	0.026269242	0.052004981	9	0	0
UBP1	0.0530805	0.051960023	0.001120525	0.019517262	0.026273478	0.052007627	6	0	0
VPS8	0.1983684	0.202984749	-0.00461631	-0.0213391	0.026285156	0.052025001	9	0	0
ZNF804A	0.1175547	0.076924474	0.040630272	0.575688352	0.026292129	0.052027319	12	0	0
SPAG8	0.3103536	0.299662532	0.010691108	0.105028568	0.026291109	0.052027319	9	0	0
ZBTB22	0.3059206	0.325634874	-0.01971427	-0.03172082	0.026299331	0.05203009	17	0	0
EID2B	0.2063135	0.236643897	-0.03033041	-0.04756106	0.026299115	0.05203009	11	0	0
HLCS	0.4198732	0.421168619	-0.00129539	0.056392964	0.026310509	0.052046463	9	0	0
TRPV1	0.8959514	0.908615523	-0.01266413	-0.01999412	0.026325126	0.052069637	9	0	0
VASH1	0.0878428	0.062175792	0.025667021	0.494574216	0.026344235	0.052101686	15	0	0
GLRA3	0.0981657	0.082783586	0.015382065	0.271440933	0.026352179	0.052105907	11	0	0
ADAM29	0.0981657	0.082783586	0.015382065	0.271440933	0.026352179	0.052105907	11	0	0
UCHL3	0.3370963	0.341842618	-0.00474629	-0.00584027	0.026382494	0.052160099	13	0	0
FDXACB1	0.8310191	0.849204331	-0.01818525	-0.03086273	0.026400113	0.052183429	1	0	0

DDX17	0.9219219	0.930493638	-0.00857174	-0.01349867	0.026397681	0.052183429		6	0	0
PIP5K1A	0.1418412	0.156060243	-0.014219	0.032110424	0.026417759	0.052212553		8	0	0
PNPLA1	0.5853869	0.629306995	-0.04392009	-0.1227387	0.026436451	0.052243741		7	0	0
WDTC1	0.1739365	0.191804287	-0.01786782	-0.05046852	0.026441911	0.052248774		11	0	0
ATXN2L	0.1995018	0.190988842	0.008512923	0.06676226	0.026462237	0.052277875		8	0	0
PTCH2	0.1842474	0.203873307	-0.01962595	0.023049877	0.026474812	0.052296501		9	0	0
PM20D1	0.3573685	0.310137389	0.047231079	0.315194595	0.026491785	0.052306986		12	0	0
ZDHHC5	0.0646815	0.070763328	-0.0060818	-0.06991739	0.02648577	0.052306986		9	0	0
TAC3	0.8722072	0.9016584	-0.02945119	-0.04808895	0.026487193	0.052306986		3	0	0
CLEC17A	0.640766	0.663705511	-0.02293955	-0.04226941	0.026489056	0.052306986		5	0	0
HEXIM1	0.2662395	0.265231244	0.001008241	-0.00500532	0.026503525	0.052324406		11	0	0
TCTN1	0.291984	0.295238353	-0.00325439	0.008057717	0.026516463	0.052344187		14	0	0
ADAMTS1	0.0582747	0.045592334	0.012682368	0.278682059	0.0265215	0.052348368		6	0	0
YIPF4	0.2570547	0.268199372	-0.01114464	-0.02858213	0.026530671	0.052360709		13	0	0
MST1R	0.4767009	0.460950686	0.015750189	0.225620038	0.026563756	0.052420236		14	0	0
TSNAX	0.2702952	0.29089245	-0.02059723	-0.06922176	0.026588668	0.052463625		11	0	0
A4GALT	0.2626883	0.274803576	-0.01211526	0.011966324	0.026613467	0.05250678		10	0	0
TMEM54	0.227897	0.248409171	-0.02051215	-0.10354135	0.026620674	0.052509961		6	0	0
MMP17	0.1219351	0.101239807	0.020695246	0.43627782	0.026620934	0.052509961		7	0	0
SLC2A13	0.2704216	0.288423437	-0.01800188	-0.05852688	0.026627205	0.052516555		7	0	0
DZIP1	0.1052105	0.093497805	0.01171273	0.240844211	0.02670044	0.052655204		11	0	0
KLK11	0.610747	0.632427776	-0.02168074	-0.06646615	0.026737815	0.052723114		9	0	0
GSX2	0.1044145	0.063557432	0.040857043	0.663098522	0.026747159	0.052729945		8	0	0
C5orf52	0.0965169	0.062448856	0.034068049	0.609181876	0.026745043	0.052729945		7	0	0
UBL4B	0.6799054	0.712522952	-0.03261751	-0.06534278	0.026761199	0.052746029		9	0	0
NEUROG3	0.0810247	0.06294614	0.018078593	0.299341035	0.02675834	0.052746029		20	0	0
ABCG4	0.2304168	0.248922173	-0.01850537	0.017591977	0.026764209	0.052746165		12	0	0
MTF1	0.1282452	0.130042572	-0.00179737	-0.00702382	0.026771462	0.052754662		8	0	0
SPINK4	0.8894857	0.902532309	-0.01304656	-0.02077522	0.026782644	0.052768829		1	0	0
FGFBP3	0.1679019	0.173441554	-0.00553961	0.098917764	0.026784535	0.052768829		6	0	0
IGF2BP3	0.1214588	0.137346507	-0.0158877	0.053576525	0.026787853	0.052769569		9	0	0
KCTD17	0.1866584	0.195577167	-0.00891876	-0.02053832	0.026810292	0.052807973		9	0	0
IWS1	0.2280569	0.244069387	-0.01601249	-0.0858116	0.026838542	0.052857811		9	0	0
MSI2	0.2511421	0.26899369	-0.0178516	-0.06446313	0.026878366	0.052930431		6	0	0
CACNA1I	0.6665689	0.692241098	-0.02567216	-0.04715716	0.026916575	0.052999857		5	0	0
PLAG1	0.0697894	0.061285194	0.008504207	0.12643469	0.026926522	0.053013623		8	0	0
RAB8A	0.272102	0.259232796	0.012869246	0.051753758	0.026937922	0.053030247		8	0	0
NRCAM	0.0936821	0.062658032	0.031024076	0.400696898	0.026962754	0.053050017		5	0	0
ITPRIP	0.1353996	0.107252902	0.028146663	0.168324268	0.026957347	0.053050017		10	0	0
PPP2R2D	0.8823054	0.890979527	-0.00867415	-0.01406554	0.026951578	0.053050017		13	0	0
COX10	0.1693864	0.191966431	-0.02258004	-0.09595375	0.02695762	0.053050017		10	0	0
RANBP1	0.5968224	0.629319087	-0.03249668	-0.07668213	0.026960048	0.053050017		10	0	0
TRIP12	0.0698201	0.061263422	0.008556656	0.124194088	0.026968255	0.053051718		4	0	0
UNC119	0.7332105	0.756406222	-0.02319574	-0.05627127	0.026969534	0.053051718		4	0	0
WDR82	0.0360661	0.035343028	0.000723094	0.036431095	0.026976643	0.053059882		7	0	0
WIPF1	0.0847457	0.059793772	0.024951881	0.584896686	0.026990134	0.053080597		8	0	0
ZNF878	0.584141	0.604482963	-0.02034192	-0.0195769	0.027002605	0.053099301		5	0	0
SRSF4	0.038275	0.036790656	0.001484385	0.045447191	0.027011013	0.053104191		8	0	0
PAAF1	0.1579415	0.174237219	-0.01629575	-0.03438569	0.027010451	0.053104191		11	0	0
RFK	0.0368808	0.027897057	0.008983702	0.202427273	0.027038321	0.053152052		5	0	0
NRDE2	0.2464464	0.263503498	-0.01705714	-0.06817638	0.027049542	0.053168284		14	0	0
ZNF697	0.1353504	0.126167367	0.009183063	0.252725838	0.02706315	0.053189202		10	0	0
MLLT10	0.1234448	0.123243428	0.00020139	0.059576344	0.027099823	0.053255443		13	0	0
ALPL	0.1475925	0.125114224	0.022478236	0.422714063	0.02710993	0.053263632		9	0	0
ACTN3	0.1116351	0.107309532	0.004325524	0.320587931	0.027109086	0.053263632		12	0	0
OSER1	0.0921662	0.105348474	-0.01318223	-0.03520945	0.027137541	0.053312041		8	0	0
SLC35A3	0.105838	0.117027876	-0.01118987	-0.00180359	0.027160883	0.053352053		16	0	0
ZHX1	0.519482	0.559349489	-0.03986745	-0.05381714	0.027180678	0.05338509		3	0	0
EFCAB11	0.0528451	0.053406542	-0.00056149	-0.04253529	0.02718978	0.05339712		14	0	0
ARHGAP10	0.0601868	0.070351286	-0.01016452	-0.0590691	0.02719457	0.05340068		7	0	0
LUZP2	0.1576078	0.126149677	0.031458091	0.332262049	0.027222211	0.053430575		9	0	0
C11orf86	0.6837413	0.719422441	-0.03568117	-0.07719759	0.027218408	0.053430575		6	0	0
GPHN	0.3238315	0.338817133	-0.01498564	-0.04860209	0.02722215	0.053430575		13	0	0

ZNF615	0.1195489	0.11048143	0.009067429	0.21949123	0.027224689	0.053430575	13	0	0
COASY	0.3713479	0.398107547	-0.02675962	-0.05155447	0.027257003	0.053488141	6	0	0
VWA2	0.4137178	0.410273955	0.003443811	0.099885137	0.027265688	0.053493048	8	0	0
C11orf91	0.1002334	0.085091875	0.015141492	0.109405398	0.027268451	0.053493048	11	0	0
TEX33	0.4242933	0.449390699	-0.02509741	-0.09525698	0.027263317	0.053493048	7	0	0
NOL11	0.115448	0.127324102	-0.01187608	0.01638407	0.027285996	0.053521612	10	0	0
DYRK2	0.2560938	0.283346209	-0.02725246	-0.00401447	0.027291291	0.053526145	6	0	0
IL10RB	0.078665	0.091209912	-0.0125449	-0.07925394	0.027305422	0.053548005	14	0	0
LSM2	0.2769815	0.270414353	0.006567151	0.035099022	0.027341069	0.053606189	13	0	0
MRPS12	0.0522581	0.060670765	-0.00841266	-0.10380118	0.027340989	0.053606189	11	0	0
DPF3	0.1644577	0.12648846	0.037969197	0.306244054	0.027377612	0.053671969	7	0	0
C5	0.8493401	0.865552325	-0.01621223	-0.02696419	0.027386216	0.053680207	1	0	0
OR5M10	0.655824	0.674250947	-0.01842697	-0.03107661	0.0273878	0.053680207	3	0	0
ZBTB3	0.1227665	0.123110392	-0.00034387	0.06430342	0.027407097	0.05371216	10	0	0
NHP2	0.3099604	0.307683985	0.002276417	0.020675169	0.027451127	0.053792571	10	0	0
TTC39C	0.6188945	0.605848084	0.013046447	0.048947072	0.027458711	0.053801557	6	0	0
WDR48	0.1235279	0.111987009	0.011540896	0.076709166	0.027471214	0.053820174	10	0	0
DCTN4	0.3372121	0.356059502	-0.01884737	-0.03390612	0.027479282	0.053824223	9	0	0
RIPK1	0.247319	0.249323012	-0.00200397	0.084178022	0.027477684	0.053824223	4	0	0
TMEM181	0.2564924	0.270910866	-0.01441844	0.082763661	0.02748549	0.053826973	10	0	0
SLC35B4	0.2912167	0.316646053	-0.02542938	-0.08171886	0.027487039	0.053826973	7	0	0
GRN	0.2385158	0.261263779	-0.02274801	-0.0417764	0.02748969	0.053826973	14	0	0
NHSL1	0.0796344	0.086076074	-0.00644169	-0.04876774	0.027495619	0.053832706	3	0	0
CDC6	0.2047775	0.208048789	-0.00327131	0.037663696	0.02751401	0.053862835	14	0	0
ZGLP1	0.8476668	0.857865987	-0.01019919	-0.01827196	0.027560905	0.053948749	13	0	0
RUFY2	0.0520459	0.048781695	0.003264243	0.028794103	0.027582796	0.053984494	7	0	0
TRMU	0.8607207	0.880285579	-0.01956489	-0.0342183	0.027585185	0.053984494	4	0	0
MMP19	0.591783	0.570692821	0.021090192	0.058226556	0.027588776	0.05398563	8	0	0
SIPA1	0.2957532	0.284209893	0.011543298	0.305170576	0.027607217	0.05400993	7	0	0
KLHDC2	0.2130678	0.229381025	-0.01631327	-0.00277407	0.027605911	0.05400993	9	0	0
SPTLC1	0.5429122	0.553113195	-0.01020103	0.018070927	0.027615303	0.054019858	3	0	0
PAPOLA	0.2457997	0.274433886	-0.02863417	-0.13294093	0.027620249	0.054023641	3	0	0
OLFM2	0.0890932	0.096274248	-0.00718101	0.012322095	0.027624706	0.054026467	10	0	0
TFR2	0.5293751	0.543708498	-0.01433338	-0.01632662	0.027628242	0.054027492	6	0	0
BRAF	0.3008858	0.301553919	-0.00066816	0.072390489	0.027639927	0.054044449	11	0	0
KLHL32	0.1440327	0.15222176	-0.00818906	-0.07040067	0.027664182	0.054085978	9	0	0
CCR3	0.7278861	0.772763824	-0.04487777	-0.09105189	0.027728319	0.05419365	3	0	0
THOC7	0.1200919	0.123570124	-0.00347818	0.029444466	0.027727419	0.05419365	10	0	0
SPPL2B	0.128549	0.149301872	-0.02075283	-0.07249736	0.027723161	0.05419365	11	0	0
UBE2I	0.5942722	0.600158306	-0.00588609	-0.00217186	0.027734235	0.054199308	2	0	0
DNAJC16	0.0463697	0.047704449	-0.00133472	-0.02424773	0.027764195	0.054251944	11	0	0
CAPRIN2	0.0613389	0.06661518	-0.00527627	-0.07698331	0.02779511	0.054306437	7	0	0
SEZ6	0.2655535	0.263566808	0.001986672	0.047188386	0.027800596	0.05431124	16	0	0
LRRC4C	0.1710212	0.187804493	-0.01678325	-0.15718133	0.027804745	0.05431343	9	0	0
RPL6	0.0439218	0.050872911	-0.00695112	-0.06202858	0.02781118	0.054320083	9	0	0
PLEKHA1	0.1404951	0.127120589	0.013374466	0.185845572	0.027829412	0.054348167	13	0	0
PIGH	0.3286325	0.340279345	-0.01164681	0.179810229	0.027834649	0.054348167	8	0	0
LAMB2	0.2155543	0.180235393	0.03531889	0.231565813	0.027843038	0.054352714	7	0	0
CELSR1	0.0781857	0.061624115	0.016561541	0.233567451	0.027840218	0.054352714	10	0	0
GAS2L1	0.1004746	0.103942775	-0.00346817	0.072927962	0.027846603	0.054353758	10	0	0
KCNK3	0.2068865	0.176186417	0.030700051	0.397952238	0.02785059	0.054355624	5	0	0
PEX26	0.1871199	0.208360427	-0.02124052	-0.04585366	0.027854268	0.054356888	9	0	0
AGPS	0.0825068	0.092943811	-0.01043706	-0.00596329	0.027865066	0.054372043	8	0	0
CHCHD7	0.0777699	0.070644032	0.007125842	0.104979421	0.027915348	0.054464231	8	0	0
RGS16	0.1245854	0.131205427	-0.00661999	-0.06758505	0.027940651	0.054504458	14	0	0
CHCHD2	0.1148605	0.127769031	-0.0129085	-0.0124571	0.027942044	0.054504458	8	0	0
CDHR1	0.3006068	0.285250507	0.015356275	0.302320379	0.02799021	0.054592476	9	0	0
PEX16	0.2209239	0.24006765	-0.01914375	-0.04151338	0.028010721	0.054620824	8	0	0
ZNF83	0.299397	0.265460191	0.033936842	0.4957952	0.028010835	0.054620824	5	0	0
PAK2	0.2877647	0.2908693	-0.00310461	0.01767705	0.028041739	0.054671303	9	0	0
SH3BP2	0.3863144	0.393400195	-0.0070858	0.147597685	0.028042818	0.054671303	9	0	0
TET1	0.0665465	0.056544713	0.010001795	0.139288032	0.028047562	0.054674608	14	0	0
TEX30	0.2427768	0.27113071	-0.02835394	-0.02188024	0.028075279	0.054722692	7	0	0

ADRA1B	0.2428754	0.257854358	-0.01497896	-0.02167044	0.028102803	0.054770388	10	0	0
PGPEP1	0.1329537	0.140164708	-0.00721102	0.111537283	0.028155531	0.054867189	14	0	0
C1QTNF4	0.4994421	0.524479239	-0.02503714	0.022352538	0.028159688	0.054869329	6	0	0
PIP4K2B	0.2346967	0.247113362	-0.01241664	-0.06707309	0.028163369	0.05487054	6	0	0
PLA2G2E	0.6507095	0.659932853	-0.0092234	-0.02025511	0.028174212	0.054885703	9	0	0
KRTDAP	0.2962471	0.318902699	-0.02265563	-0.1060214	0.028186958	0.054904569	4	0	0
IL12RB1	0.2857927	0.260078407	0.025714311	0.392297872	0.028190968	0.054906417	11	0	0
ZMAT3	0.1916624	0.187759098	0.003903281	0.106907006	0.028202035	0.054922008	14	0	0
LAPTM4B	0.4092814	0.420033925	-0.01075254	-0.03510587	0.028222132	0.054955179	5	0	0
LYPD3	0.553538	0.576868196	-0.0233302	-0.04995665	0.028238136	0.054980374	10	0	0
EFCAB7	0.0453396	0.047481554	-0.00214193	-0.03985066	0.028241838	0.054981614	14	0	0
NCCRP1	0.2979351	0.303850437	-0.00591535	0.074734601	0.028268215	0.055026993	5	0	0
CTSE	0.7014203	0.69592255	0.005497709	0.026710618	0.028280864	0.055045641	10	0	0
XKR5	0.0734733	0.057420484	0.016052779	0.407246126	0.028314992	0.055094012	7	0	0
ERLIN1	0.7467934	0.774519541	-0.02772615	-0.05208402	0.028314616	0.055094012	2	0	0
NAA60	0.4114352	0.372182487	0.039252678	0.146924825	0.028317175	0.055094012	12	0	0
CRYBA4	0.6966373	0.731856504	-0.03521925	-0.07803314	0.028318002	0.055094012	9	0	0
SLC9A2	0.1206731	0.093425722	0.027247347	0.434315723	0.02832467	0.055101007	6	0	0
UBE2T	0.158871	0.185083813	-0.02621286	-0.11009771	0.028327754	0.055101029	12	0	0
SLC26A6	0.1531636	0.138804226	0.014359374	0.02368537	0.028334677	0.055108519	9	0	0
DIAPH1	0.0350664	0.033445746	0.001620645	0.052772957	0.028370737	0.055166689	8	0	0
RNF26	0.1920725	0.178297137	0.013775318	0.107529512	0.02837021	0.055166689	12	0	0
PRKAG1	0.179409	0.192907119	-0.01349808	-0.05822967	0.028399808	0.055217231	10	0	0
COA6	0.1270135	0.159782714	-0.03276917	-0.09301165	0.028413616	0.055238091	11	0	0
ART3	0.8717109	0.888950876	-0.01723995	-0.02842465	0.028423943	0.055252177	9	0	0
OR1J1	0.8838169	0.902054084	-0.01823722	-0.02913873	0.028433938	0.055265617	2	0	0
ASB2	0.5017333	0.486748532	0.014984784	0.143641264	0.028440538	0.055266468	10	0	0
MYH10	0.0597113	0.058001676	0.00170961	0.026033898	0.028438954	0.055266468	10	0	0
AP3D1	0.0401201	0.038414391	0.001705692	0.053026983	0.028450502	0.05527984	4	0	0
MAPK9	0.0515802	0.055360136	-0.00377995	-0.03823638	0.028487566	0.05534586	9	0	0
RNF39	0.2491129	0.256369417	-0.00725653	0.115101736	0.028499063	0.055356205	16	0	0
IFT52	0.0837108	0.091232623	-0.00752184	0.032501046	0.028497649	0.055356205	7	0	0
SLC35D3	0.1124132	0.083099966	0.029313248	0.387651865	0.02853217	0.05541451	9	0	0
PDE6D	0.2129947	0.223798462	-0.01080374	0.010340746	0.028547089	0.055437482	16	0	0
ALOX15B	0.3505633	0.312540293	0.038022989	0.243288268	0.028551584	0.055440209	7	0	0
NLGN2	0.0747162	0.068881346	0.005834805	0.110881703	0.028556276	0.055443318	13	0	0
VMP1	0.1423197	0.153609295	-0.01128957	-0.0737129	0.028598563	0.05551941	10	0	0
ZNF845	0.1143005	0.088565222	0.025735312	0.317906114	0.028628997	0.055572478	2	0	0
EIF2B3	0.3307932	0.348422787	-0.01762956	-0.03736649	0.028633025	0.055574282	12	0	0
KATNBL1	0.2822282	0.293396064	-0.01116782	-0.02234865	0.028638785	0.055579446	10	0	0
ZYG11B	0.5180206	0.539357464	-0.02133685	-0.06736368	0.028642851	0.055581324	5	0	0
DALRD3	0.4264592	0.45170306	-0.02524385	-0.05679771	0.028673288	0.055634368	4	0	0
QPCTL	0.0378154	0.037211873	0.00060351	0.036121751	0.02870444	0.055688787	15	0	0
TROVE2	0.0751817	0.089699938	-0.01451825	-0.11924992	0.028720403	0.055713729	17	0	0
TMED1	0.1915113	0.191881706	-0.00037042	0.004003416	0.028727977	0.055722397	13	0	0
IGFBPL1	0.3574355	0.366583213	-0.00914775	0.101766255	0.028738789	0.055729622	5	0	0
ZER1	0.234091	0.242845138	-0.00875414	-0.04010042	0.02873691	0.055729622	9	0	0
CEP55	0.1428029	0.14581263	-0.00300975	-0.03138086	0.028741024	0.055729622	8	0	0
SPSB1	0.3069403	0.276467337	0.030472934	0.089370828	0.028749458	0.055739949	6	0	0
AREL1	0.1603815	0.164708565	-0.00432707	0.037357232	0.028769888	0.055773532	15	0	0
ANXA9	0.654634	0.696366705	-0.04173268	-0.0929225	0.028790419	0.055789213	6	0	0
CNIH2	0.0956076	0.098877162	-0.00326961	0.264288992	0.028784535	0.055789213	16	0	0
RASAL1	0.2554519	0.266765847	-0.01131392	0.124741768	0.028789829	0.055789213	15	0	0
C5AR2	0.4786097	0.493241807	-0.01463212	-0.01729294	0.028785816	0.055789213	11	0	0
TPO	0.6341259	0.682460991	-0.04833512	-0.11728116	0.028808884	0.055818964	3	0	0
PLK4	0.0808733	0.073497503	0.007375843	0.078744619	0.028812536	0.055820009	9	0	0
NOC4L	0.0986398	0.105654731	-0.00701494	-0.03060258	0.028867072	0.055919624	11	0	0
MND1	0.2643419	0.262236125	0.002105734	0.028237433	0.028871435	0.055922036	7	0	0
FAM186B	0.6910794	0.719364751	-0.02828531	-0.0620835	0.028902005	0.055975204	4	0	0
KCNS1	0.4553456	0.412270378	0.043075222	0.207826046	0.028927311	0.056018166	14	0	0
AMT	0.6038665	0.584839397	0.019027098	0.056983763	0.028931756	0.056020725	12	0	0
ACADM	0.2314446	0.247602538	-0.01615794	-0.01539004	0.028942423	0.056032187	9	0	0
LRFN2	0.1508519	0.154235093	-0.00338322	0.137500818	0.028943923	0.056032187	13	0	0

STK10	0.2033725	0.210776816	-0.00740434	0.028252502	0.028978059	0.056080559	11	0	0
WIPI2	0.3902492	0.414896195	-0.02464698	-0.05785349	0.028980924	0.056080559	5	0	0
NXN	0.0903205	0.087758043	0.002562465	0.080647458	0.028981417	0.056080559	11	0	0
CKB	0.1786528	0.16762919	0.011023597	0.179070682	0.029004285	0.056118755	11	0	0
GOLPH3	0.0753945	0.087704691	-0.01231014	0.019273837	0.02902509	0.056152951	9	0	0
MOB3A	0.030698	0.029414949	0.001283101	0.042108763	0.029032051	0.056160361	7	0	0
C15orf39	0.4967586	0.539566901	-0.04280831	-0.09251032	0.029043058	0.056175594	2	0	0
CLK4	0.3122985	0.290526521	0.021772017	0.028133466	0.02908344	0.056247636	3	0	0
EP300	0.1173326	0.136644472	-0.01931192	-0.03671915	0.029092885	0.056259837	5	0	0
SEMA4A	0.0959542	0.078047616	0.017906623	0.140694575	0.029124506	0.056314914	3	0	0
SDC2	0.301024	0.296336785	0.004687236	0.073446765	0.029142408	0.056343454	8	0	0
RNF34	0.1180752	0.120528487	-0.00245332	-0.0086311	0.029150542	0.056353106	9	0	0
TMEM192	0.4710094	0.464426655	0.00658276	0.023353866	0.029158619	0.056356573	10	0	0
SYNE4	0.3652325	0.394098457	-0.02886595	-0.07365655	0.029158559	0.056356573	7	0	0
MAK	0.1941323	0.223517808	-0.02938549	-0.07736818	0.029178295	0.056388524	10	0	0
IQCB1	0.1203098	0.146030387	-0.02572059	-0.05345063	0.029197024	0.056418641	10	0	0
AKIRIN1	0.0630341	0.061538383	0.001495746	0.042114498	0.029212236	0.056441954	11	0	0
NPPB	0.1703617	0.157923586	0.012438138	0.122044006	0.029248679	0.056506281	12	0	0
HMCN1	0.1772139	0.159006866	0.018206987	0.232229074	0.029263649	0.056529112	14	0	0
POGK	0.2778259	0.26975182	0.008074036	0.041076284	0.029274085	0.056537093	7	0	0
BMI1	0.1937068	0.21964072	-0.02593397	-0.06176556	0.029273709	0.056537093	11	0	0
CDKL1	0.8988458	0.91120892	-0.01236311	-0.01998054	0.029317284	0.056614429	5	0	0
TMEM256	0.0836543	0.078694923	0.004959411	0.104861575	0.029335102	0.056642738	13	0	0
ARMC2	0.1130479	0.131405331	-0.01835743	-0.0497163	0.029341017	0.05664806	7	0	0
USP42	0.2393484	0.259030915	-0.01968253	-0.0438057	0.029351557	0.056662311	5	0	0
SYT16	0.824672	0.849811943	-0.02513992	-0.04281174	0.029366946	0.056685918	1	0	0
ALKBH1	0.1178249	0.140388895	-0.02256403	-0.0503641	0.029384305	0.05671332	11	0	0
MSRB2	0.1476787	0.121804372	0.025874329	0.127094501	0.029387495	0.056713376	8	0	0
SECTM1	0.2777657	0.25752384	0.020241866	0.341540838	0.02942317	0.056776114	8	0	0
KCTD13	0.5227595	0.518693877	0.004065622	0.084364273	0.029430868	0.056784859	5	0	0
MAGOH	0.2332498	0.247022375	-0.01377259	-0.06289676	0.029448432	0.056806525	12	0	0
AHNAK2	0.0970121	0.09065124	0.006360825	0.352222757	0.029447874	0.056806525	13	0	0
PRRT1	0.2014719	0.188212698	0.013259243	0.045097859	0.029470935	0.05684382	27	0	0
TNFRSF10D	0.3909189	0.361411411	0.029507471	0.441818395	0.029521524	0.056935275	11	0	0
SHC3	0.1830453	0.157870546	0.025174788	0.418845187	0.029565243	0.05701346	8	0	0
C6orf136	0.3605698	0.377381885	-0.01681211	-0.03283667	0.029583364	0.057042273	16	0	0
LMAN2L	0.1654768	0.177060191	-0.01158336	0.051324705	0.029593787	0.057056237	9	0	0
PARL	0.1316174	0.152180636	-0.02056321	-0.05799861	0.029630708	0.057121279	8	0	0
EXOC6B	0.4095052	0.44112579	-0.0316206	-0.03483723	0.029659512	0.057164519	11	0	0
PURA	0.2488448	0.268338688	-0.01949393	-0.04363947	0.029658988	0.057164519	7	0	0
PHC2	0.0651946	0.062312503	0.002882143	0.113537369	0.029712477	0.057260449	6	0	0
CCS	0.2823576	0.295081566	-0.01272397	-0.02460784	0.029726551	0.057281415	13	0	0
TOMM22	0.2216587	0.230611716	-0.00895302	-0.04772312	0.029730314	0.057282513	9	0	0
CCNH	0.2397956	0.256273197	-0.01647758	-0.06001465	0.029738871	0.057292845	9	0	0
TRPM4	0.0691459	0.060615919	0.008530012	0.098441826	0.02975592	0.057319533	4	0	0
MEP1A	0.7541959	0.776651669	-0.02245573	-0.04195591	0.029823935	0.057438215	8	0	0
TIMM9	0.0943424	0.092225472	0.002116935	0.075924109	0.029822451	0.057438215	19	0	0
TLE6	0.1254632	0.109819155	0.015644055	0.099781478	0.029854316	0.057490554	10	0	0
PTBP1	0.1018034	0.10333457	-0.00153116	0.039647575	0.029873852	0.057521998	9	0	0
ALS2CL	0.2050068	0.204984776	2.20E-05	0.340502066	0.029894271	0.057555135	11	0	0
ALS2CR12	0.7972026	0.801513645	-0.00431102	-0.02058997	0.029901139	0.05756218	4	0	0
METTL11B	0.5516528	0.546550569	0.005102275	0.049881644	0.029913802	0.057573565	8	0	0
ZSCAN29	0.0493895	0.052621669	-0.00323217	-0.07292848	0.029918781	0.057573565	7	0	0
RRP1	0.1848471	0.188047337	-0.00320021	0.005244154	0.029912907	0.057573565	7	0	0
DNAJA1	0.2981309	0.325345738	-0.02721481	-0.05391315	0.029965012	0.0576542	5	0	0
PUS7L	0.1259908	0.147977574	-0.02198673	-0.0344104	0.029989069	0.057694299	15	0	0
RRAGC	0.1351955	0.147153451	-0.01195797	-0.00741258	0.029995909	0.05769508	11	0	0
GTF2A1	0.1971451	0.209277877	-0.01213273	0.009559894	0.029995206	0.05769508	13	0	0
RPL27A	0.2613904	0.276097958	-0.01470752	-0.06548335	0.030022193	0.057739444	9	0	0
LUZP1	0.189905	0.170951264	0.018953686	0.12109553	0.03003176	0.057749808	8	0	0
POU2F2	0.7920746	0.812621386	-0.02054681	-0.08825389	0.030034279	0.057749808	5	0	0
DNAJC28	0.0634145	0.066513676	-0.00309915	0.029947709	0.030037241	0.057749808	10	0	0
ANXA6	0.166852	0.158865398	0.007986591	0.241637162	0.030058602	0.057770818	10	0	0

KLHDC10	0.1004939	0.100864093	-0.00037017	-0.04595464	0.030059806	0.057770818	8	0	0
TSHR	0.1984821	0.200528829	-0.0020467	0.056290678	0.030061053	0.057770818	8	0	0
CLEC18B	0.8142206	0.849899654	-0.03567909	-0.06113785	0.030057167	0.057770818	1	0	0
POLR3D	0.1856411	0.210445919	-0.02480479	-0.01754025	0.030094578	0.057829049	6	0	0
ANXA11	0.1268403	0.132385575	-0.00554532	-0.04085806	0.030125647	0.05788255	8	0	0
TMEFF1	0.8850957	0.86960152	0.015494158	0.025191806	0.030148264	0.057917793	1	0	0
CBLN3	0.3766718	0.391846641	-0.0151748	0.027193693	0.030150448	0.057917793	4	0	0
SIK1	0.2972866	0.283365956	0.013920633	0.112898211	0.030176272	0.057961193	5	0	0
RNPC3	0.1184118	0.134355819	-0.01594402	-0.0256721	0.030179608	0.057961393	16	0	0
IBTK	0.1479694	0.176393967	-0.0284246	-0.10815079	0.0301875	0.057970343	11	0	0
PPP6C	0.0510733	0.049237674	0.001835578	0.044377724	0.030199672	0.057976286	4	0	0
GLE1	0.050225	0.049137735	0.001087225	0.022456061	0.030200292	0.057976286	5	0	0
MPHOSPH9	0.9052982	0.914494471	-0.00919628	-0.01442286	0.030198489	0.057976286	1	0	0
MTX1	0.2181209	0.199546173	0.018574717	0.121160674	0.030260603	0.058062864	18	0	0
CCDC149	0.2689711	0.267736342	0.001234746	0.079032777	0.030262736	0.058062864	11	0	0
HARS	0.3442199	0.353598491	-0.0093786	-0.01187044	0.030263905	0.058062864	14	0	0
AZIN1	0.6539866	0.67707627	-0.02308967	-0.04171155	0.030253041	0.058062864	2	0	0
RALGDS	0.3763916	0.368776198	0.007615392	0.341707714	0.030264815	0.058062864	9	0	0
GBGT1	0.3763916	0.368776198	0.007615392	0.341707714	0.030264815	0.058062864	9	0	0
TMOD4	0.7476584	0.77434197	-0.02668357	-0.05730424	0.030301177	0.058122368	5	0	0
FBXO16	0.0808581	0.090906299	-0.01004823	-0.14508858	0.030302312	0.058122368	10	0	0
CLSTN2	0.1352369	0.098786362	0.036450496	0.676367598	0.030316592	0.058142142	11	0	0
USP35	0.1209071	0.106535304	0.014371818	0.061434085	0.030319104	0.058142142	10	0	0
LENEP	0.6918992	0.721420042	-0.02952086	-0.07263325	0.030324749	0.058146749	7	0	0
MORC2	0.4461513	0.477865753	-0.03171447	-0.06102434	0.030336162	0.058162416	11	0	0
SLC30A3	0.8684497	0.862547538	0.00590214	0.0158102	0.030359662	0.058201249	7	0	0
TDRKH	0.2072188	0.202334363	0.004884483	0.292005362	0.030369609	0.058207877	9	0	0
CNGA4	0.1700281	0.168669222	0.001358863	0.041536817	0.030367276	0.058207877	7	0	0
METTL14	0.4362624	0.44385711	-0.00759467	0.018081149	0.030378646	0.058218977	6	0	0
RIMS3	0.1893415	0.202748174	-0.01340668	-0.05097705	0.030383824	0.058219823	6	0	0
OR3A1	0.7456957	0.789024109	-0.04332842	-0.08351202	0.03038558	0.058219823	3	0	0
ZNF584	0.2620752	0.242152431	0.019922727	0.145742838	0.030401685	0.058244458	4	0	0
HLA-E	0.3128469	0.338479769	-0.02563287	-0.04354924	0.030411077	0.05825623	13	0	0
SEMA3F	0.0506213	0.045888283	0.004733039	0.105416333	0.030428255	0.058282911	6	0	0
IFIH1	0.1322448	0.143504262	-0.01125951	-0.06044039	0.030434822	0.058289265	12	0	0
DPP4	0.2785246	0.237710715	0.040813917	0.570191741	0.030460624	0.058332452	5	0	0
SERPINB12	0.7665009	0.805867067	-0.03936617	-0.07145262	0.030474792	0.058353354	5	0	0
GALNT5	0.3921679	0.390025922	0.002142007	0.093501644	0.030550727	0.058492511	6	0	0
PRTFDC1	0.1807442	0.189689273	-0.00894511	0.244484605	0.030572995	0.058528898	7	0	0
EGF	0.6936878	0.71351031	-0.01982249	0.032515	0.030589844	0.058548654	7	0	0
MIPOL1	0.1455054	0.14747945	-0.00197407	0.091153606	0.030587471	0.058548654	13	0	0
TXLNA	0.304041	0.318521844	-0.01448084	-0.00745041	0.030604818	0.058571063	12	0	0
ENOPH1	0.0331749	0.034859503	-0.00168465	-0.01605313	0.03061772	0.058589504	5	0	0
UQCRQ	0.308572	0.322742673	-0.01417063	-0.03693329	0.030653896	0.058652473	17	0	0
CA11	0.2013154	0.2051839	-0.00386852	0.027674146	0.030675064	0.058686715	6	0	0
MAPT	0.7747609	0.814811274	-0.04005041	-0.07416759	0.030684611	0.058698718	3	0	0
MYOCD	0.2487727	0.232449142	0.016323542	0.347934938	0.030713552	0.058741551	10	0	0
INPP5K	0.199787	0.200615312	-0.00082834	0.058262693	0.030725344	0.058757839	9	0	0
DPM3	0.1569961	0.175939901	-0.01894378	-0.03127377	0.030731778	0.058763878	9	0	0
NUDT16L1	0.1145635	0.1227963	-0.00823284	-0.01401656	0.030737521	0.058768594	8	0	0
CEACAM19	0.7751802	0.767124606	0.008055631	0.014750772	0.030744957	0.058776546	2	0	0
KBTBD8	0.1961445	0.212613692	-0.01646923	-0.06762119	0.030753641	0.058783229	7	0	0
BSG	0.1925193	0.193967538	-0.00144826	0.051442445	0.030755008	0.058783229	11	0	0
KPNB1	0.032963	0.03117524	0.001787744	0.062507412	0.030805984	0.058874388	10	0	0
HEYL	0.4026765	0.401947472	0.00072905	0.135082771	0.030813552	0.058882576	9	0	0
UBL3	0.3485146	0.355059813	-0.00654523	0.011385723	0.030839319	0.058925539	11	0	0
SNRPC	0.0474337	0.049244636	-0.00181096	0.009884925	0.030847488	0.058934868	8	0	0
EIF2D	0.2978585	0.332067729	-0.03420923	-0.11276029	0.030862895	0.058958025	5	0	0
MPZL2	0.2787507	0.306294844	-0.02754413	-0.12994792	0.030867218	0.058960002	13	0	0
CLCN7	0.5309198	0.551756989	-0.02083715	-0.06954764	0.030879206	0.058971398	3	0	0
CBX1	0.3179858	0.339486431	-0.02150064	-0.03782243	0.03087976	0.058971398	12	0	0
TMEM52	0.1992	0.207680987	-0.00848096	-0.01453725	0.030916	0.059034321	17	0	0
ACAD9	0.1139718	0.118586986	-0.00461516	-0.05732183	0.030932498	0.05905325	9	0	0

TRIM5	0.6951535	0.72753562	-0.03238211	-0.07090263	0.030931548	0.05905325	3	0	0
CDC25C	0.1178854	0.12756852	-0.00968309	-0.0240773	0.030938216	0.05905788	13	0	0
C10orf113	0.7555997	0.779036367	-0.02343669	-0.04530283	0.030960344	0.059093832	5	0	0
THSD1	0.1109744	0.088509078	0.02246528	0.505953906	0.030978717	0.059116318	6	0	0
KRTAP1-5	0.7190096	0.742603581	-0.02359402	-0.0498259	0.030978633	0.059116318	7	0	0
DYNC1LI2	0.2914275	0.287158988	0.004268521	0.006689813	0.030995746	0.059142522	9	0	0
NCK2	0.1698066	0.14665102	0.023155592	0.084229299	0.031000647	0.05914558	5	0	0
CCM2L	0.6047289	0.592762528	0.011966391	0.053503808	0.031018863	0.05917404	5	0	0
FAM129A	0.3255846	0.300390234	0.025194358	0.193530608	0.031041727	0.05921136	9	0	0
WDR45B	0.2287937	0.229148281	-0.00035454	0.003894825	0.031084196	0.059286062	6	0	0
GSR	0.0303862	0.030219331	0.000166901	0.027396968	0.031092307	0.059295228	3	0	0
FERMT1	0.4038018	0.425343907	-0.02154213	-0.0424766	0.031107253	0.059317423	8	0	0
MBP	0.4335744	0.405273704	0.028300722	0.070866488	0.031138185	0.059370095	4	0	0
PLA2G4E	0.7914986	0.79423308	-0.00273445	-0.00760106	0.031144444	0.059375717	4	0	0
ADAMTSL5	0.2258487	0.219203034	0.006645654	0.26828782	0.031152855	0.059385441	5	0	0
NUP88	0.0540292	0.045119814	0.008909359	0.14268433	0.031171224	0.059414142	9	0	0
SYK	0.2276494	0.238072436	-0.01042299	-0.0224521	0.031194468	0.059441937	10	0	0
ITGAM	0.6209357	0.663466936	-0.04253121	-0.09761097	0.031195749	0.059441937	4	0	0
ZC3H3	0.3769352	0.408500579	-0.03156541	-0.03622491	0.031204254	0.059451827	6	0	0
NUP153	0.0849955	0.081067463	0.003928023	0.069185115	0.031208695	0.059453973	10	0	0
ACADVL	0.2560545	0.23552186	0.020532625	0.211900688	0.031232916	0.059493797	7	0	0
GTDC1	0.0948022	0.104750027	-0.00994787	-0.02040192	0.031237741	0.059496668	9	0	0
TMEM51	0.1841002	0.182513316	0.001586839	0.078185577	0.031272131	0.059555844	12	0	0
NAIF1	0.164324	0.183008273	-0.01868427	-0.00474285	0.031284192	0.059572488	5	0	0
SLC35D1	0.1421945	0.14634439	-0.00414986	-0.0615626	0.031300425	0.059597073	9	0	0
FAM110B	0.2958142	0.268489363	0.027324875	0.389807285	0.031304726	0.059598936	9	0	0
CCDC87	0.1829585	0.193462917	-0.01050441	-0.03858997	0.031348891	0.059676683	14	0	0
CCDC3	0.0441666	0.047068693	-0.00290211	-0.00621029	0.031367378	0.059705539	13	0	0
RPS23	0.1911352	0.20502388	-0.01388867	-0.0239585	0.031380384	0.059723957	14	0	0
POU3F2	0.0799135	0.061460452	0.018453061	0.356730172	0.03138983	0.059735595	12	0	0
ZNF597	0.4112299	0.372726772	0.038503145	0.143732156	0.031397215	0.05974331	13	0	0
TUFM	0.093752	0.100272427	-0.00652046	-0.02536487	0.031424966	0.059789774	8	0	0
SENP7	0.1460745	0.162452978	-0.01637851	-0.05018237	0.031437691	0.059801297	7	0	0
KLHL8	0.5536	0.584099596	-0.03049956	-0.05493035	0.031436378	0.059801297	3	0	0
TOM1	0.5159897	0.528373962	-0.01238426	-0.02426843	0.031444818	0.05980851	9	0	0
SRSF10	0.0405847	0.044411251	-0.0038266	-0.05614266	0.031471742	0.059853373	4	0	0
RAB13	0.1578756	0.175653737	-0.01777818	-0.13673035	0.031477816	0.059858579	10	0	0
RND1	0.1883595	0.164281449	0.02407809	0.163900464	0.031503782	0.059901605	9	0	0
MICU1	0.1458659	0.145354415	0.00051151	0.056492368	0.031535837	0.059949343	8	0	0
PGAM5	0.0336858	0.027545362	0.006140487	0.182209321	0.031533176	0.059949343	6	0	0
ZNF264	0.374422	0.365816503	0.008605517	0.05392575	0.031538916	0.059949343	20	0	0
DNAJA3	0.1521141	0.172282812	-0.02016876	-0.05284302	0.031549669	0.059963428	10	0	0
NOL6	0.104219	0.113312073	-0.00909308	-0.06174793	0.031554737	0.059966705	11	0	0
HGF	0.3491007	0.371453701	-0.02235303	-0.06453836	0.031597221	0.06004108	10	0	0
PEPD	0.1609526	0.176337374	-0.01538475	0.043756567	0.031615281	0.060069034	10	0	0
GNAQ	0.0860054	0.074990185	0.011015231	0.187945381	0.031629142	0.060089005	5	0	0
TRIM2	0.1753857	0.183727989	-0.00834227	-0.06261149	0.03167448	0.060156022	8	0	0
ZNF234	0.1727413	0.185326041	-0.01258477	0.096925661	0.031671379	0.060156022	11	0	0
CARD9	0.6169694	0.625682989	-0.00871358	-0.00775873	0.031678711	0.060157688	6	0	0
APP	0.2039754	0.20176712	0.002208241	0.005660929	0.031682927	0.060159324	13	0	0
RER1	0.0985499	0.116824668	-0.01827479	-0.06298775	0.031698755	0.060176637	7	0	0
DOC2A	0.6845533	0.736694964	-0.05214167	-0.10568295	0.031696141	0.060176637	5	0	0
MTFP1	0.0872552	0.097229974	-0.00997482	0.071029766	0.031708303	0.060188392	13	0	0
SLC4A4	0.0455311	0.027802094	0.017729031	0.604475784	0.031735008	0.060226493	8	0	0
HSPA1B	0.1155045	0.125181213	-0.00967671	0.043536956	0.031749531	0.060234954	14	0	0
PON1	0.3383228	0.331467587	0.006855245	0.177554866	0.031756341	0.060234954	21	0	0
PON3	0.3383228	0.331467587	0.006855245	0.177554866	0.031756341	0.060234954	21	0	0
ZC3HAV1L	0.0954791	0.102094843	-0.0066157	0.259240167	0.031753722	0.060234954	8	0	0
C19orf70	0.081859	0.079179771	0.00267923	0.154474641	0.031748692	0.060234954	13	0	0
ADNP2	0.5000368	0.493187286	0.006849533	0.074033932	0.03181321	0.060336443	8	0	0
FOXN2	0.1903349	0.19969811	-0.00936318	0.196650041	0.03182988	0.060361676	6	0	0
IRF2BPL	0.2339746	0.252720341	-0.01874573	-0.08259793	0.031833895	0.060362908	7	0	0
KDM1B	0.0396535	0.036745496	0.002907972	0.066987813	0.031852	0.060390854	7	0	0

SPATA4	0.1660929	0.170371287	-0.00427843	0.176292183	0.03188679	0.060431262	12	0	0
KIF18A	0.1898899	0.208275732	-0.01838587	-0.04063533	0.031881749	0.060431262	13	0	0
TEX14	0.5289104	0.551582172	-0.02267179	-0.03813591	0.031886437	0.060431262	16	0	0
RAD51C	0.5289104	0.551582172	-0.02267179	-0.03813591	0.031886437	0.060431262	16	0	0
APOBEC3D	0.0595929	0.068293102	-0.00870025	-0.10434038	0.031921081	0.060489858	3	0	0
BPTF	0.0427652	0.037643884	0.005121276	0.146792656	0.031942472	0.060518879	4	0	0
RPL38	0.1144021	0.121532574	-0.00713048	-0.02775991	0.031943144	0.060518879	11	0	0
DOCK6	0.7172221	0.726399129	-0.00917704	-0.01690002	0.031955419	0.06053574	5	0	0
BTBD9	0.1489978	0.132674753	0.016323016	0.104869829	0.03197808	0.060565875	11	0	0
ZKSCAN1	0.2873236	0.310830377	-0.02350682	-0.08039093	0.031976741	0.060565875	9	0	0
TP53INP2	0.0739612	0.084813343	-0.01085213	-0.06087661	0.031999023	0.060599143	7	0	0
BCAS2	0.2460018	0.248978547	-0.00297679	0.023534953	0.032006178	0.060606294	12	0	0
SNCB	0.2337053	0.203569833	0.030135424	0.368908638	0.032017514	0.06061496	10	0	0
EID1	0.1136666	0.130303388	-0.01663679	-0.08592339	0.032015444	0.06061496	9	0	0
PLAUR	0.1498407	0.129262285	0.020578398	0.224739577	0.032073045	0.060713681	7	0	0
STARD3	0.2203817	0.232940754	-0.01255904	-0.02989951	0.032122412	0.060800716	13	0	0
CDKL2	0.1785884	0.157108943	0.021479429	0.292532674	0.032127851	0.060804594	11	0	0
RPL5	0.3937499	0.404761297	-0.01101137	-0.04235174	0.032156448	0.060852294	11	0	0
KLHL36	0.3275157	0.336919641	-0.00940396	0.059017022	0.032164403	0.060860928	7	0	0
ZRANB2	0.2306596	0.224262856	0.006396771	0.038144677	0.032197085	0.060916342	9	0	0
WWOX	0.3810546	0.385857763	-0.00480319	-0.03334337	0.032223159	0.060959243	8	0	0
DR1	0.0340557	0.03714519	-0.00308952	-0.0766762	0.032227508	0.06096104	12	0	0
IQCG	0.0634873	0.079160476	-0.01567315	-0.10057795	0.032235811	0.060970315	11	0	0
TMEM127	0.101494	0.11417992	-0.01268593	-0.02807342	0.032259995	0.060990331	8	0	0
KCNK15	0.185567	0.180284711	0.005282305	0.272017393	0.032257768	0.060990331	12	0	0
DYNC1LI1	0.1462053	0.153742993	-0.00753774	-0.00660593	0.032271793	0.060999777	12	0	0
GRM4	0.7589629	0.772455517	-0.01349263	-0.01869509	0.03227038	0.060999777	7	0	0
PFDN4	0.0939156	0.10067252	-0.00675693	-0.02323073	0.032279555	0.061008019	11	0	0
SMYD1	0.7973183	0.803764816	-0.00644648	-0.00353881	0.032312133	0.061059455	9	0	0
FBLN1	0.8392977	0.873861031	-0.03456336	-0.05749231	0.032313579	0.061059455	4	0	0
HIC1	0.1005557	0.105510789	-0.00495511	0.043332088	0.032322095	0.061069113	30	0	0
ZNF506	0.1260124	0.093543551	0.03246884	0.597650235	0.032371723	0.061156438	5	0	0
EHD2	0.1156321	0.107889189	0.007742925	0.313398609	0.032377456	0.061160827	9	0	0
WDR66	0.7280816	0.754368079	-0.02628646	-0.05787888	0.032403304	0.061203207	8	0	0
C9orf57	0.6404806	0.605078731	0.035401876	0.080734956	0.032410167	0.061209724	1	0	0
EIF6	0.1894753	0.208754984	-0.01927972	-0.01117844	0.032433161	0.061246702	9	0	0
ATG7	0.2930543	0.303712707	-0.01065845	-0.02240519	0.032448247	0.061268739	9	0	0
NAPB	0.2306067	0.237393427	-0.00678674	-0.04757875	0.032469111	0.061301682	9	0	0
SERHL2	0.1607843	0.179611374	-0.01882711	-0.05974722	0.032499316	0.061352251	9	0	0
KIF24	0.2066578	0.238487635	-0.03182984	-0.0952644	0.032509844	0.061365666	8	0	0
PACSIN3	0.0839944	0.054704726	0.029289656	0.44345583	0.032529685	0.061396657	12	0	0
MMP2	0.636248	0.632001697	0.004246284	0.011237036	0.032536718	0.06140347	4	0	0
C9orf3	0.1535339	0.150192676	0.003341274	0.028340197	0.032555653	0.061432739	7	0	0
AHRR	0.7740905	0.810940976	-0.03685047	-0.07014946	0.032583001	0.061477877	2	0	0
PPRC1	0.0396574	0.042127693	-0.00247028	-0.02078562	0.03259208	0.061488539	11	0	0
PITHD1	0.0412351	0.039667001	0.001568108	0.022513704	0.03264803	0.061587618	8	0	0
SRP19	0.2433152	0.2538893	-0.01057413	-0.053938	0.032654045	0.061592487	10	0	0
SAA2-SAA4	0.7147588	0.741107457	-0.02634868	-0.05377404	0.03266527	0.061594226	4	0	0
SAA2	0.7147588	0.741107457	-0.02634868	-0.05377404	0.03266527	0.061594226	4	0	0
KLHL35	0.2042616	0.19099208	0.013269478	0.344898372	0.032665044	0.061594226	9	0	0
BNIP2	0.3572952	0.365246644	-0.00795148	0.023887538	0.032671664	0.061599808	5	0	0
REEP5	0.1892101	0.184982488	0.004227621	0.057192909	0.032682979	0.061614663	9	0	0
CHCHD10	0.1957614	0.197670477	-0.00190909	0.258446549	0.032692816	0.061626731	10	0	0
ITGBL1	0.6696227	0.697515722	-0.02789297	-0.05817135	0.032712197	0.061656785	9	0	0
GLRX	0.186421	0.186975032	-0.00055402	-0.01982738	0.032735071	0.061693415	8	0	0
HUS1B	0.8759728	0.861284259	0.014688547	0.035803551	0.032742954	0.061701788	12	0	0
NUP133	0.1525945	0.171913251	-0.0193188	-0.07706906	0.03286396	0.061916806	13	0	0
POLR1E	0.346001	0.351477253	-0.00547622	-0.03975491	0.032872554	0.061926492	7	0	0
FGF22	0.1521851	0.168072637	-0.01588749	-0.03351021	0.032876043	0.06192656	7	0	0
RAD51AP2	0.165407	0.186439624	-0.02103261	-0.03803034	0.032890566	0.061947411	10	0	0
TSGA10	0.0665671	0.079628507	-0.01306144	-0.03324225	0.032916893	0.061977472	11	0	0
RPL32	0.1511046	0.148015829	0.003088774	0.074934006	0.032916646	0.061977472	8	0	0
MTCH2	0.1925475	0.20516635	-0.0126189	0.013142053	0.032911861	0.061977472	10	0	0

AP3S2	0.2149602	0.234361985	-0.01940175	-0.05137297	0.032947522	0.062028629	9	0	0
RHOBTB2	0.6477304	0.672736561	-0.02500614	-0.04766842	0.032952305	0.062031122	5	0	0
TXK	0.7552831	0.738983525	0.016299575	0.039826613	0.03295768	0.06203473	7	0	0
CCNB2	0.1497346	0.169867166	-0.02013255	-0.03251089	0.032976314	0.062063292	13	0	0
GPR83	0.2061494	0.16775425	0.038395146	0.362206288	0.033002651	0.062106342	11	0	0
RPS6KL1	0.3669299	0.395559776	-0.02862987	-0.0335596	0.033048684	0.062186445	8	0	0
CDC42EP1	0.3509808	0.336604078	0.014376677	0.064917085	0.033067331	0.062215006	6	0	0
RBM34	0.0425552	0.052406434	-0.00985122	-0.07503944	0.033084484	0.062232256	6	0	0
PFDN1	0.0461708	0.045106575	0.001064242	0.042075039	0.033089184	0.062232256	12	0	0
ALDH3A1	0.4461834	0.47637142	-0.03018798	-0.08477793	0.033089	0.062232256	7	0	0
SFPQ	0.1251248	0.138738238	-0.01361349	-0.02978606	0.033111041	0.062264588	11	0	0
USP32	0.7314109	0.759439022	-0.02802814	-0.05519794	0.033186108	0.062399208	8	0	0
YWHAH	0.0755935	0.07620155	-0.00060801	0.059114789	0.033195315	0.062409977	10	0	0
CRIM1	0.2144452	0.210532418	0.003912797	0.188048178	0.033214055	0.062438664	9	0	0
INIP	0.2527304	0.26438987	-0.0116595	-0.00856476	0.03322988	0.062461227	7	0	0
SDK2	0.0834709	0.073153083	0.010317823	0.209834068	0.033233022	0.062461227	13	0	0
ARMC3	0.3101424	0.28938799	0.020754452	0.358553308	0.033241258	0.06247016	9	0	0
ZACN	0.1298829	0.148399757	-0.01851689	-0.12775033	0.033256895	0.062492998	4	0	0
CALCOCO2	0.4084073	0.42429363	-0.01588631	-0.05885574	0.033272465	0.062515706	6	0	0
NTN4	0.114048	0.095643829	0.01840415	0.207343548	0.033277069	0.062517808	9	0	0
EXOSC1	0.114807	0.108795574	0.006011409	0.081444634	0.033293466	0.062542061	9	0	0
PPP1R32	0.7702435	0.779662086	-0.0094186	-0.01342835	0.03337233	0.062677079	9	0	0
GOT2	0.2360791	0.244004218	-0.00792512	-0.05239916	0.033372153	0.062677079	11	0	0
NDUFV3	0.0414429	0.042329366	-0.00088643	0.00024225	0.03344887	0.062814254	10	0	0
ZNF324B	0.1632576	0.176411459	-0.01315386	-0.07974903	0.033488388	0.062875298	9	0	0
C1orf61	0.1684647	0.120306335	0.048158362	0.45615615	0.033510826	0.062910841	3	0	0
C1QTNF9B	0.4104463	0.379544501	0.030901772	0.323530212	0.03352042	0.062922266	6	0	0
PGAP3	0.8879348	0.899697433	-0.01176265	-0.01874792	0.033538479	0.062949578	2	0	0
TMEM116	0.0401068	0.043283738	-0.00317695	-0.03231127	0.033552338	0.062968999	11	0	0
RAET1E	0.5659841	0.527515492	0.038468598	0.099722626	0.033557829	0.062972716	1	0	0
HIST1H1C	0.1754335	0.181144696	-0.0057112	0.016996139	0.033563559	0.062976879	7	0	0
TMF1	0.1542208	0.164789439	-0.01056864	-0.07349654	0.03356716	0.062977048	8	0	0
MIXL1	0.2444265	0.244852741	-0.00042629	0.315575343	0.033571055	0.062977768	12	0	0
TACR1	0.0773946	0.06249958	0.014894983	0.35422253	0.033581345	0.062990483	17	0	0
ZFP90	0.1643387	0.191873983	-0.02753528	-0.07715228	0.033588258	0.062996862	8	0	0
VSNL1	0.2019643	0.195327012	0.006637317	0.133953547	0.03359416	0.063001344	7	0	0
KHDC1	0.0731857	0.066003953	0.007181724	0.141860434	0.033604852	0.063014806	8	0	0
TMEM175	0.0948892	0.097486783	-0.00259762	0.007877211	0.033617162	0.063031301	14	0	0
MS4A15	0.6375698	0.670958637	-0.03338879	-0.06989576	0.033628805	0.06304654	6	0	0
VAMP3	0.3305126	0.34163564	-0.011123	-0.02900934	0.033635956	0.063053356	9	0	0
C5orf49	0.5549712	0.568397939	-0.01342677	0.07926614	0.033644537	0.063056261	13	0	0
TDP1	0.1192573	0.141227081	-0.0219698	-0.12931924	0.03364346	0.063056261	11	0	0
RPS8	0.135836	0.149429935	-0.01359395	0.022495839	0.033650216	0.063058293	9	0	0
AAAS	0.0766897	0.081835296	-0.00514556	0.106159391	0.033656169	0.063058293	6	0	0
RSAD1	0.4521676	0.45957359	-0.007406	-0.03690278	0.0336547	0.063058293	7	0	0
VOPP1	0.2691612	0.268829657	0.000331575	0.098824643	0.033684429	0.063104651	7	0	0
CCDC83	0.160918	0.176808183	-0.01589023	-0.09546435	0.033706218	0.063138875	11	0	0
PDIK1L	0.1089767	0.097514455	0.011462212	0.051232497	0.03371199	0.063141949	8	0	0
NUP98	0.0930424	0.098944976	-0.00590256	-0.00304691	0.0337149	0.063141949	17	0	0
TRHR	0.6543356	0.670935688	-0.0166001	-0.02777676	0.033742558	0.06318715	6	0	0
APOC2	0.7029032	0.742626733	-0.03972357	-0.07818478	0.033748234	0.063191181	7	0	0
DENND4B	0.1431162	0.150630522	-0.00751435	-0.02393214	0.033781231	0.063239761	6	0	0
HIF1A	0.1545532	0.163509384	-0.00895617	-0.06175163	0.033779293	0.063239761	9	0	0
ATP6V0D1	0.2115111	0.21672769	-0.00521661	0.008611774	0.033798291	0.063265095	8	0	0
MTPN	0.3942893	0.41443732	-0.02014803	-0.04890512	0.033835362	0.063327877	10	0	0
LRP10	0.1804145	0.187170144	-0.00675562	0.018988398	0.033882334	0.063409175	11	0	0
SNPH	0.2326283	0.219923161	0.01270512	0.296206996	0.03391309	0.063460112	11	0	0
PPARG	0.4102871	0.438618041	-0.02833091	-0.02516663	0.033920418	0.063460584	10	0	0
SBK3	0.6241761	0.631585538	-0.00740946	-0.01402785	0.033917039	0.063460584	13	0	0
REEP4	0.0865863	0.074530793	0.012055471	0.040832185	0.033925806	0.063464044	6	0	0
MPDU1	0.371984	0.387889979	-0.01590594	0.014289931	0.033938574	0.063481307	19	0	0
MDC1	0.2380485	0.255035401	-0.01698691	-0.03298311	0.03395668	0.063508551	21	0	0
PRAP1	0.3774273	0.374902423	0.002524864	0.044006244	0.033960255	0.063508616	9	0	0

SSFA2	0.1558863	0.146260599	0.009625737	0.075950508	0.033988573	0.063554946	7	0	0
HMGA1	0.1013991	0.10371651	-0.00231741	0.03774428	0.033995366	0.063561022	12	0	0
FAM174A	0.2492965	0.25927254	-0.00997604	-0.04639267	0.034007551	0.063577177	8	0	0
RNF220	0.1932882	0.20590888	-0.01262066	-0.0068596	0.034013251	0.063581205	12	0	0
USP14	0.2109135	0.209592662	0.00132079	0.007505051	0.034028351	0.063602803	9	0	0
SLC7A7	0.1482693	0.153553693	-0.00528441	-0.0215951	0.034032853	0.063604589	12	0	0
RECQL4	0.1485595	0.164123676	-0.01556419	-0.05760055	0.034039899	0.06361113	7	0	0
SCN1B	0.0620895	0.065876455	-0.00378694	0.012745248	0.03404683	0.063617455	8	0	0
LRCH3	0.2154481	0.23675211	-0.02130403	-0.06792905	0.034052507	0.063621435	7	0	0
NDUFS4	0.2099006	0.211350073	-0.00144946	0.068149608	0.03408928	0.063683505	14	0	0
NCOA6	0.1973798	0.214377497	-0.01699768	-0.06520423	0.034110841	0.063717146	7	0	0
ADAM23	0.0977142	0.069066649	0.028647523	0.34887156	0.034131992	0.063750016	8	0	0
ATG13	0.1877284	0.198257844	-0.01052941	-0.03393803	0.034190018	0.063851745	15	0	0
IREB2	0.3385392	0.335700244	0.002838916	0.016940274	0.034195751	0.063855804	6	0	0
CSF2	0.5800237	0.594342453	-0.01431872	-0.0673196	0.03420386	0.06385588	9	0	0
ATN1	0.260224	0.271399223	-0.01117525	-0.00649805	0.034206473	0.06385588	9	0	0
SEMA7A	0.3392611	0.324178289	0.015082762	0.255113792	0.034199433	0.06385588	11	0	0
SPAG4	0.1689449	0.188626686	-0.0196818	0.010773018	0.034226998	0.063887547	4	0	0
FMO3	0.6697431	0.712105496	-0.04236239	-0.0977136	0.034260048	0.063942582	5	0	0
MMEL1	0.6213318	0.654042564	-0.03271079	-0.07426248	0.034293841	0.063998994	5	0	0
ZBTB44	0.0870135	0.102710442	-0.01569698	-0.06283655	0.03431929	0.064039824	17	0	0
H2AFZ	0.0517096	0.060128922	-0.00841932	-0.03931173	0.034359927	0.064108983	11	0	0
PNN	0.1631963	0.173378466	-0.01018214	-0.05971037	0.034366292	0.06411419	12	0	0
AKR7A2	0.1544779	0.137698835	0.01677911	0.082696065	0.034390198	0.064152116	8	0	0
MLH3	0.2023715	0.205526148	-0.00315464	-0.00079634	0.034416798	0.064195059	8	0	0
MB21D2	0.2388765	0.236143028	0.002733444	0.000921138	0.034433753	0.064220005	10	0	0
DDX58	0.1070054	0.099509608	0.00749581	0.047085756	0.0344832	0.064305539	9	0	0
ZNF638	0.1979583	0.149067654	0.048890647	0.694091802	0.034503491	0.064336689	11	0	0
IL17RA	0.3547181	0.354730529	-1.24E-05	0.058859843	0.034514757	0.064351006	10	0	0
ANGPT1	0.525654	0.566737412	-0.04108338	-0.09660908	0.034528239	0.064362762	11	0	0
QSOX2	0.1123096	0.098649732	0.013659846	0.039575596	0.034527713	0.064362762	5	0	0
LAT	0.5060807	0.541010849	-0.03493017	-0.10660353	0.034535173	0.064367933	16	0	0
CCDC103	0.1316164	0.154452112	-0.02283571	-0.01368264	0.034538191	0.064367933	7	0	0
PWWP2B	0.1806013	0.162891896	0.017709373	0.213013777	0.034545156	0.064374224	3	0	0
EFEMP1	0.3702196	0.352118924	0.018100663	0.25888103	0.034586564	0.064444692	8	0	0
NTF3	0.0931334	0.070675021	0.022458341	0.397884325	0.034606081	0.06447436	13	0	0
RIBC2	0.3680379	0.362270769	0.005767122	0.269119537	0.034620907	0.064495282	14	0	0
OLFML2A	0.2188083	0.19882092	0.019987352	0.254511693	0.034645468	0.064534336	10	0	0
MFF	0.2371484	0.261851717	-0.02470331	-0.07007984	0.034668373	0.064570296	8	0	0
TMEM56	0.198532	0.205823353	-0.00729136	-0.01520085	0.034676032	0.064577854	11	0	0
TTC30A	0.1691504	0.185477522	-0.01632709	-0.04139357	0.034681797	0.064581885	10	0	0
ITGAE	0.6697015	0.693613584	-0.02391207	-0.04750448	0.034692135	0.064594428	9	0	0
CCSAP	0.1930215	0.214691969	-0.02167046	-0.01024786	0.034718857	0.064637473	10	0	0
RHBDF2	0.2099079	0.223560892	-0.01365301	-0.05819172	0.034728241	0.064643852	8	0	0
RAB27B	0.2374447	0.268014995	-0.03057032	-0.1621798	0.034729491	0.064643852	2	0	0
KIF17	0.1741643	0.17705745	-0.00289311	0.231438271	0.034745358	0.064666674	11	0	0
UBR1	0.1632829	0.169135943	-0.005853	0.002570597	0.034752307	0.064672896	9	0	0
IL31	0.7462707	0.788399212	-0.04212848	-0.07575525	0.03475861	0.064677917	6	0	0
KIAA1324	0.0994009	0.094675323	0.004725625	0.208039757	0.034767505	0.064687757	10	0	0
S100A1	0.3583592	0.354720891	0.003638336	0.046434597	0.03481956	0.06477789	18	0	0
NUTF2	0.1569096	0.164687106	-0.00777746	0.030561293	0.034857378	0.064838592	8	0	0
DONSON	0.20224	0.207562686	-0.00532265	-0.06247356	0.034859419	0.064838592	5	0	0
C11orf45	0.294662	0.308389171	-0.01372712	-0.03132266	0.034880393	0.064870876	13	0	0
SLC43A3	0.5746108	0.604028577	-0.0294178	-0.07738614	0.034887647	0.06487764	6	0	0
RPL31	0.493208	0.497459436	-0.00425142	0.127050645	0.034925278	0.064940885	13	0	0
CDH5	0.4256835	0.397607873	0.028075633	0.092611773	0.034939566	0.064960719	6	0	0
MRFAP1	0.567401	0.578332977	-0.01093196	-0.05367188	0.03494639	0.064966673	5	0	0
ABHD14A	0.687129	0.68725447	-0.00012546	0.001246744	0.034973238	0.065009846	2	0	0
C8orf58	0.1254577	0.102849217	0.02260849	0.246265639	0.034978324	0.065012562	6	0	0
NDUFAF2	0.1259438	0.141063846	-0.01512008	-0.06623024	0.034992818	0.065026023	13	0	0
RNASEH2A	0.1135645	0.125080163	-0.01151568	-0.06740862	0.034991581	0.065026023	10	0	0
OCLN	0.2252716	0.205352475	0.019919139	0.222281128	0.035003406	0.065032224	14	0	0
PRSS8	0.5738413	0.565711569	0.008129759	0.02242492	0.034999973	0.065032224	13	0	0

SHC4	0.0834783	0.071791259	0.011687055	0.165063886	0.035027778	0.065070764	12	0	0
KNSTRN	0.3984872	0.381025832	0.017461403	0.044477316	0.035040457	0.065087575	2	0	0
NTPCR	0.2851253	0.293816844	-0.00869159	-0.02020474	0.035048133	0.065088354	10	0	0
TMEM251	0.0401382	0.041501674	-0.00136352	0.013644868	0.035046234	0.065088354	16	0	0
MGAT1	0.5737446	0.542670301	0.031074306	0.121112577	0.035064175	0.065111404	5	0	0
IPO7	0.2922661	0.292719776	-0.00045368	0.039277961	0.035072147	0.065114436	8	0	0
CDK17	0.1442237	0.135753571	0.008470176	0.101181274	0.035073069	0.065114436	13	0	0
SLC30A6	0.0696004	0.078278496	-0.00867807	-0.01196549	0.035085637	0.065131028	12	0	0
LRRC23	0.0555483	0.043321873	0.012226386	0.248211359	0.035122639	0.065192968	13	0	0
GZMH	0.7577184	0.79811909	-0.04040067	-0.07392864	0.035152985	0.065242544	2	0	0
SLC27A6	0.1438459	0.105732314	0.038113577	0.43969667	0.03516084	0.065250371	7	0	0
ABI1	0.1700837	0.158714945	0.011368749	0.084448566	0.035172445	0.065258403	8	0	0
C20orf85	0.3339946	0.360119745	-0.02612516	-0.06380943	0.035170471	0.065258403	13	0	0
ATAD2	0.0509302	0.053194274	-0.00226412	0.027900922	0.035178876	0.065263583	11	0	0
BMP6	0.087707	0.090907325	-0.00320031	0.356635287	0.035240204	0.065370596	8	0	0
TJAP1	0.2437143	0.263192704	-0.01947839	-0.03971458	0.035256394	0.065393865	6	0	0
SMIM19	0.151612	0.166925217	-0.0153132	-0.04470313	0.035260554	0.065394819	14	0	0
LRRC17	0.4407468	0.477215483	-0.03646868	-0.12197835	0.035285654	0.065432099	6	0	0
MKNK2	0.2024411	0.218199038	-0.01575797	-0.02866182	0.035287951	0.065432099	9	0	0
NICN1	0.0734111	0.074390207	-0.00097914	0.008930731	0.035295169	0.065438596	6	0	0
DERL3	0.1647982	0.149657803	0.015140363	0.519026938	0.035298752	0.065438596	9	0	0
NDUFC2-KC	0.2348414	0.242546398	-0.00770502	0.019616864	0.035307881	0.065448755	8	0	0
SCN9A	0.1944451	0.178760647	0.015684471	0.224926474	0.035312921	0.065451334	11	0	0
BRF2	0.2669258	0.279061479	-0.0121357	-0.00889701	0.03532317	0.065463564	10	0	0
KIAA1586	0.3242079	0.34622649	-0.02201857	-0.03750352	0.035330591	0.065470553	11	0	0
FANCC	0.3245619	0.32825733	-0.00369543	0.054600291	0.035359876	0.065518052	12	0	0
ZMPSTE24	0.0371048	0.036816131	0.000288718	0.02405642	0.035368906	0.065521245	14	0	0
NUDT12	0.2302994	0.247086708	-0.01678735	-0.03712955	0.035367732	0.065521245	11	0	0
SEC14L1	0.222145	0.235771006	-0.01362599	-0.08387729	0.035376957	0.065529392	6	0	0
CACNG2	0.1959731	0.217402059	-0.02142899	-0.02242283	0.035381078	0.065530257	11	0	0
G3BP1	0.1164354	0.097464826	0.018970557	0.263514487	0.035386692	0.065533888	7	0	0
CHI3L2	0.5059658	0.486731055	0.01923475	0.060552626	0.035407881	0.065559709	9	0	0
NAF1	0.3847162	0.417430961	-0.0327148	-0.06708822	0.035407945	0.065559709	10	0	0
C7orf57	0.4830896	0.513143327	-0.03005373	0.042281072	0.035462415	0.065653786	7	0	0
TUBA4A	0.1675289	0.18052631	-0.01299744	-0.0410201	0.035502523	0.065681374	10	0	0
DNAJB2	0.1675289	0.18052631	-0.01299744	-0.0410201	0.035502523	0.065681374	10	0	0
PRSS50	0.2563941	0.242839366	0.013554718	0.256798218	0.035501609	0.065681374	8	0	0
PRSS45	0.2563941	0.242839366	0.013554718	0.256798218	0.035501609	0.065681374	8	0	0
PTCD2	0.1596878	0.170304149	-0.01061638	-0.04616115	0.03550295	0.065681374	16	0	0
OCM2	0.531863	0.571238871	-0.03937584	-0.10120297	0.035497778	0.065681374	1	0	0
LMO7	0.8167182	0.83258557	-0.01586741	-0.02925721	0.035496604	0.065681374	4	0	0
IFT43	0.857084	0.867436035	-0.01035199	-0.01712212	0.035514498	0.065695961	1	0	0
PGD	0.0322918	0.028878638	0.003413199	0.097737213	0.03551922	0.06569792	7	0	0
CAMK4	0.3101164	0.315400985	-0.00528455	0.119069127	0.035566961	0.065775809	9	0	0
MRPL40	0.0338057	0.034558122	-0.00075244	-0.00892179	0.035568665	0.065775809	6	0	0
CDKAL1	0.1888553	0.186321286	0.00253397	0.07386636	0.035585478	0.065800116	16	0	0
KDM3B	0.4983309	0.506414212	-0.00808329	-0.01265762	0.035594715	0.065810411	9	0	0
YAP1	0.150954	0.157609294	-0.00665528	0.016344643	0.035600614	0.065814534	12	0	0
HOXC13	0.1579211	0.129572299	0.028348789	0.279931358	0.035651538	0.065901885	9	0	0
ZNF25	0.1258965	0.123630997	0.002265483	0.07490982	0.035657124	0.065905417	8	0	0
MON1A	0.2721451	0.278288058	-0.006143	0.023673123	0.03567733	0.065929177	12	0	0
ACTN4	0.0486097	0.043377674	0.005231994	0.119378397	0.035675332	0.065929177	10	0	0
GABPB1	0.055498	0.05717153	-0.00167357	-0.03419333	0.035706768	0.065976779	13	0	0
SCLT1	0.1784772	0.189966507	-0.0114893	-0.03945399	0.035721334	0.065996894	16	0	0
CYC1	0.0539415	0.064641391	-0.01069985	-0.03124111	0.035736218	0.066010793	7	0	0
QRFP	0.5746698	0.615838911	-0.04116911	-0.10264077	0.035733812	0.066010793	6	0	0
GALNS	0.3279678	0.341227066	-0.01325929	-0.04445541	0.035741344	0.066013464	5	0	0
NAT8L	0.1226287	0.098532896	0.024095797	0.412282451	0.035754885	0.066024876	9	0	0
GHRHR	0.5228756	0.558537492	-0.03566191	-0.09345676	0.035754577	0.066024876	1	0	0
GFM1	0.3561323	0.377791024	-0.02165873	-0.0363975	0.035774297	0.066053921	10	0	0
PHRF1	0.0715096	0.066146779	0.00536285	0.084332178	0.035778893	0.066055607	7	0	0
STRADA	0.1292704	0.143871236	-0.01460082	-0.02021214	0.035795697	0.06607983	10	0	0
LGALSL	0.2278829	0.224745553	0.003137359	-0.01386276	0.035809367	0.066098261	6	0	0

ANLN	0.2053518	0.223239935	-0.01788809	-0.09711464	0.035832278	0.066133746	12	0	0
INSIG2	0.1615032	0.173731818	-0.01222858	-0.08431757	0.035850626	0.066160802	9	0	0
PLD2	0.1272227	0.128453997	-0.00123133	0.032540061	0.035902938	0.066250523	8	0	0
MZF1	0.1599038	0.180437213	-0.02053344	-0.03398864	0.035919779	0.066274781	11	0	0
RASL10A	0.6360334	0.67907958	-0.04304617	-0.09504908	0.035963673	0.066348945	2	0	0
LPGAT1	0.2184507	0.234459065	-0.01600836	-0.04352395	0.035988027	0.066387046	14	0	0
SYT5	0.2510097	0.289902457	-0.03889274	-0.0705825	0.03599452	0.066392195	12	0	0
C11orf16	0.7410384	0.775446084	-0.03440768	-0.06640601	0.036009001	0.066412077	11	0	0
DDX20	0.2818642	0.292418552	-0.01055434	0.009415553	0.03602694	0.066438329	7	0	0
CEP350	0.0603074	0.074700834	-0.01439342	-0.05783244	0.036038801	0.06645337	9	0	0
SLFN12L	0.7335614	0.786443019	-0.05288161	-0.09911925	0.036050376	0.066467881	1	0	0
ITLN1	0.5779923	0.613892939	-0.03590068	-0.10597047	0.03605785	0.066474827	5	0	0
FGFR3	0.3463312	0.355241093	-0.00890988	-0.01644999	0.036077654	0.066504502	8	0	0
TMEM169	0.1964509	0.202781661	-0.0063308	0.046384077	0.03609236	0.066511104	11	0	0
ZNRD1	0.8299562	0.852064126	-0.02210793	-0.0374811	0.036087523	0.066511104	1	0	0
MCAT	0.255618	0.272396736	-0.01677874	-0.01786842	0.036091021	0.066511104	10	0	0
COMTD1	0.3416741	0.343152807	-0.0014787	0.006219302	0.036112506	0.066541392	10	0	0
EXOSC10	0.0523326	0.049101846	0.003230722	0.083130591	0.036116838	0.066542538	8	0	0
EPS15L1	0.1739089	0.176773473	-0.00286455	0.01258155	0.036130443	0.066560766	7	0	0
ZNF99	0.2757177	0.284383061	-0.00866537	0.10582107	0.036139258	0.066570169	3	0	0
FBXL4	0.2406635	0.269384673	-0.02872117	-0.05492195	0.03614567	0.066575143	11	0	0
MRPS34	0.0749768	0.079066945	-0.00409015	-0.0077281	0.03617363	0.0666198	8	0	0
PTCHD4	0.2493735	0.225112915	0.024260605	0.244197461	0.036216071	0.066684267	14	0	0
MAB21L3	0.46413	0.453108024	0.011021979	0.047066122	0.036255505	0.066750024	7	0	0
EYA1	0.2289912	0.239292502	-0.01030132	-0.05488145	0.036264514	0.06675337	13	0	0
GAPDHS	0.5662653	0.579915406	-0.0136501	0.032748416	0.036264766	0.06675337	14	0	0
SPRR2A	0.7421191	0.789092972	-0.04697391	-0.09120039	0.03629552	0.066800985	4	0	0
MUC3A	0.5840254	0.578041629	0.005983809	0.094652887	0.036301807	0.066800985	6	0	0
DGKH	0.1986081	0.216140138	-0.01753207	-0.06433666	0.036301159	0.066800985	10	0	0
CTSD	0.1210338	0.127705101	-0.00667128	-0.02055898	0.036308279	0.066806042	12	0	0
MOAP1	0.0847218	0.08624366	-0.00152186	0.039216481	0.036328278	0.066831352	15	0	0
KCNJ14	0.7756556	0.783372304	-0.00771668	-0.01730141	0.036332548	0.066831352	12	0	0
SGSM1	0.2571483	0.244741943	0.01240631	0.207132901	0.036333213	0.066831352	10	0	0
SUSD4	0.2198498	0.194362905	0.025486926	0.129482292	0.036338237	0.066833738	6	0	0
SNX15	0.272743	0.292490165	-0.01974719	-0.00858952	0.036343724	0.066836977	13	0	0
SGK3	0.15066	0.119463594	0.031196446	0.196644874	0.036369281	0.066877119	7	0	0
AADACL2	0.7546971	0.803173194	-0.04847614	-0.09393034	0.036400253	0.066922943	4	0	0
TMEM86A	0.780368	0.810921025	-0.03055305	-0.05419164	0.036401663	0.066922943	3	0	0
TMCC2	0.0940316	0.10989361	-0.01586199	-0.04899892	0.036411894	0.066934892	10	0	0
PSMD4	0.1856644	0.191426888	-0.00576247	0.043328252	0.036421211	0.066945156	11	0	0
QARS	0.3327793	0.32415803	0.008621309	0.025338646	0.036445174	0.066982339	16	0	0
RPP21	0.2494021	0.261151099	-0.01174898	-0.00132736	0.036476371	0.067032806	22	0	0
PRICKLE2	0.4922117	0.486750723	0.005460974	0.093300456	0.036506962	0.06708215	4	0	0
BIVM	0.1339624	0.139785164	-0.00582275	0.132148898	0.036513858	0.067087948	13	0	0
REST	0.1287638	0.148037731	-0.01927391	-0.05031924	0.036519325	0.067091121	9	0	0
TGFBR2	0.1629924	0.171609635	-0.00861725	0.030438668	0.036523719	0.067092321	15	0	0
FBXL12	0.0739636	0.069695955	0.00426762	0.058834388	0.036539378	0.067114213	13	0	0
GAN	0.2771882	0.294594726	-0.01740648	0.00420675	0.036546333	0.067120114	7	0	0
CDA	0.3889707	0.422022585	-0.03305189	-0.1180263	0.036552961	0.067121621	7	0	0
PPM1G	0.2740383	0.284835429	-0.01079713	0.015760629	0.036554638	0.067121621	11	0	0
POPDC2	0.885448	0.87138979	0.014058176	0.022829416	0.036578441	0.067125599	1	0	0
FAM185A	0.1830774	0.202065997	-0.01898861	-0.05801052	0.036579054	0.067125599	5	0	0
SFXN2	0.0471401	0.048182329	-0.00104219	-0.00971881	0.036572052	0.067125599	12	0	0
RAB39A	0.160408	0.14952306	0.01088498	0.280355223	0.03657926	0.067125599	11	0	0
LGALS9	0.9144823	0.920831629	-0.00634937	-0.01006535	0.03656796	0.067125599	2	0	0
SRRD	0.4576292	0.495425312	-0.03779616	-0.07090316	0.036568592	0.067125599	3	0	0
KLHDC1	0.0504254	0.044214308	0.006211065	0.10578394	0.036617841	0.067189524	11	0	0
ACAA2	0.2449982	0.261354186	-0.01635596	-0.01714659	0.036634575	0.067213353	12	0	0
CIRBP	0.3154722	0.348093202	-0.03262104	-0.137801	0.03665099	0.067236592	1	0	0
TRIP10	0.0506169	0.046210131	0.00440678	0.115778103	0.036658477	0.067243448	13	0	0
NMNAT3	0.2685629	0.267051011	0.001511916	0.172990958	0.03666407	0.067246832	9	0	0
PPIAL4A	0.6875111	0.770192714	-0.08268163	-0.16161438	0.03666788	0.067246942	1	0	0
PPP1R3G	0.1248249	0.121822693	0.003002246	0.274909308	0.036672515	0.067248567	9	0	0

CHCHD5	0.2405835	0.247866598	-0.00728309	0.240009116	0.036699558	0.067284398	12	0	0
PNPLA3	0.1249199	0.120362597	0.004557333	0.188395736	0.036698958	0.067284398	10	0	0
SRP72	0.1714785	0.184087293	-0.01260876	-0.09486888	0.036707358	0.067291821	12	0	0
HUNK	0.1719507	0.179218391	-0.00726767	-0.10021869	0.036727137	0.067321199	9	0	0
ALDOC	0.4338638	0.428238769	0.005625049	0.288520243	0.03674406	0.067345336	11	0	0
RPL37A	0.4296822	0.427155676	0.002526481	0.065996018	0.036790283	0.067416277	6	0	0
TPCN2	0.1850746	0.195562963	-0.01048836	-0.00350875	0.036787914	0.067416277	11	0	0
EXO5	0.1192319	0.122743468	-0.00351156	0.131798237	0.036801391	0.067429742	12	0	0
GNL1	0.1247815	0.138658806	-0.01387726	-0.03040672	0.036809068	0.067436919	20	0	0
SGTB	0.1754859	0.191599165	-0.01611323	-0.07656618	0.036823625	0.067439728	7	0	0
WDR31	0.2214336	0.239571577	-0.018138	-0.00966015	0.036823951	0.067439728	6	0	0
LINGO1	0.2033705	0.21725333	-0.01388278	0.003591555	0.036825641	0.067439728	7	0	0
KRTAP17-1	0.6050129	0.627317037	-0.02230418	-0.06186653	0.036817644	0.067439728	6	0	0
UBE2E1	0.1609738	0.172636288	-0.01166253	-0.06515409	0.036830434	0.06744162	6	0	0
AKT1S1	0.3289161	0.350662888	-0.02174677	-0.04806739	0.036845052	0.0674615	11	0	0
MIIP	0.2310016	0.239530971	-0.00852938	-0.00237862	0.036866099	0.067486257	17	0	0
CYB561	0.0936015	0.100194773	-0.00659324	0.029096209	0.036862757	0.067486257	6	0	0
PHLPP1	0.1090491	0.119341513	-0.01029239	-0.03631941	0.036873633	0.06749316	10	0	0
ERCC5	0.7748512	0.798058739	-0.02320749	-0.04809712	0.036880731	0.067499264	5	0	0
CEP63	0.1067661	0.118809	-0.01204291	-0.04049885	0.036905738	0.067524362	18	0	0
ATXN1L	0.1149738	0.116887245	-0.00191344	0.018121829	0.03690565	0.067524362	11	0	0
IST1	0.1149738	0.116887245	-0.00191344	0.018121829	0.03690565	0.067524362	11	0	0
THEMIS2	0.3314057	0.342494701	-0.01108899	0.10915517	0.036936726	0.067553495	9	0	0
ZNF727	0.2724395	0.242299198	0.03014029	0.24554188	0.036933255	0.067553495	6	0	0
PGBD3	0.1346302	0.150097083	-0.01546688	-0.04342601	0.036926221	0.067553495	14	0	0
VWA5A	0.2318298	0.239501119	-0.00767128	-0.03088105	0.036932576	0.067553495	8	0	0
TDRD12	0.8431341	0.868198654	-0.02506457	-0.05263232	0.036952188	0.067574882	10	0	0
ARHGEF40	0.0544402	0.031779037	0.022661187	0.370993756	0.03695787	0.067578382	6	0	0
KIDINS220	0.3160924	0.338277783	-0.02218539	-0.03689333	0.036973333	0.067599766	11	0	0
CASC3	0.0636707	0.063884339	-0.00021363	0.019060755	0.036981693	0.06760816	12	0	0
GPRC5A	0.2918163	0.308346313	-0.01652999	-0.04215386	0.036996184	0.067627758	10	0	0
BRD4	0.2200173	0.256732664	-0.03671536	-0.09658701	0.03701628	0.067657598	5	0	0
FLT1	0.1385157	0.10334831	0.035167406	0.427350638	0.037045041	0.067703266	5	0	0
GTF2A2	0.3051279	0.325859948	-0.02073206	-0.07394178	0.037070309	0.067742543	12	0	0
SRD5A1	0.132813	0.141693249	-0.00888027	-0.00246089	0.037080907	0.067748104	8	0	0
ZSCAN16	0.1191954	0.136580508	-0.01738513	-0.10901816	0.037077216	0.067748104	4	0	0
NPM1	0.1407135	0.142741424	-0.00202797	0.011478793	0.037101887	0.067772629	10	0	0
ZNF680	0.1576815	0.155837068	0.001844401	0.13346669	0.037101764	0.067772629	8	0	0
IL7R	0.4811351	0.516752799	-0.03561772	-0.08232362	0.037116781	0.067792932	5	0	0
PCYOX1L	0.0517094	0.04867485	0.003034567	0.027827467	0.037190306	0.067920307	13	0	0
FUS	0.068799	0.076568939	-0.00776989	-0.00927955	0.037236678	0.067998071	6	0	0
SPRY1	0.1327157	0.13078831	0.001927413	0.094158352	0.037252919	0.068020803	12	0	0
SBK1	0.236742	0.256972545	-0.02023051	-0.05517865	0.037281809	0.068066625	8	0	0
TAB1	0.2328822	0.238859003	-0.00597676	-0.00472761	0.037288458	0.068071835	4	0	0
MKRN1	0.0368628	0.036353714	0.000509048	0.030564288	0.037334549	0.068149041	7	0	0
ACE	0.1789608	0.155714641	0.023246174	0.493424377	0.037352667	0.068175175	10	0	0
CDC25B	0.3245164	0.305171082	0.019345345	0.01870853	0.037402627	0.068259413	7	0	0
RTCB	0.3054383	0.326839355	-0.02140102	-0.06863708	0.037413131	0.068271637	9	0	0
SLC25A31	0.8868794	0.89467059	-0.00779119	-0.01403779	0.037458302	0.068347111	13	0	0
ZBTB8B	0.140632	0.109459425	0.031172619	0.478032336	0.037480563	0.068366865	15	0	0
AKR1B1	0.1517973	0.168552961	-0.01675562	-0.01243159	0.037479957	0.068366865	13	0	0
APH1B	0.7901928	0.820080573	-0.02988774	-0.05275111	0.03748002	0.068366865	2	0	0
MYL9	0.3553393	0.32132186	0.034017476	0.17261498	0.03749657	0.068389107	8	0	0
CCDC113	0.8364379	0.853190442	-0.01675259	-0.02827474	0.03752679	0.068437265	1	0	0
EPSTI1	0.2139345	0.177288683	0.036645849	0.624144482	0.037535592	0.068446358	8	0	0
CORIN	0.3970285	0.41730348	-0.02027497	-0.09281968	0.037569563	0.068501341	11	0	0
SSTR5	0.2785401	0.251886052	0.026654002	0.216839122	0.037596349	0.068543212	10	0	0
FAM200B	0.0383242	0.041684941	-0.00336078	-0.06940029	0.037624025	0.068586697	9	0	0
JAK1	0.0902813	0.10316908	-0.01288774	-0.02015686	0.037685294	0.068691407	10	0	0
NAGLU	0.2209328	0.240333759	-0.01940094	-0.0278345	0.03771846	0.068744873	10	0	0
SHROOM1	0.4695263	0.461934622	0.0075917	0.047853814	0.037749724	0.068794864	8	0	0
RALGAPA1	0.1250386	0.13418277	-0.00914419	-0.01019791	0.037762335	0.068810854	7	0	0
FZD2	0.0846292	0.071672482	0.012956686	0.117310819	0.037767164	0.068812662	12	0	0

CHD5	0.1141313	0.100365199	0.013766098	0.266560865	0.037813492	0.068883078	10	0	0
SLC35F6	0.3262584	0.344019641	-0.01776123	-0.03058413	0.037844421	0.068918422	8	0	0
CENPA	0.3262584	0.344019641	-0.01776123	-0.03058413	0.037844421	0.068918422	8	0	0
TMOD1	0.1634441	0.16271934	0.000724739	0.036599114	0.037841624	0.068918422	7	0	0
TIAM1	0.3350536	0.332166868	0.002886723	0.031749729	0.037865788	0.068943333	5	0	0
ZFHX2	0.0740736	0.079318592	-0.00524496	-0.00633126	0.037886009	0.068973148	11	0	0
NEXN	0.3786703	0.349631851	0.029038419	0.272725774	0.037890923	0.068975094	9	0	0
STAC2	0.1773817	0.155734541	0.021647116	0.404758123	0.037899226	0.068983207	6	0	0
PPM1E	0.1530882	0.158902578	-0.00581436	0.036733057	0.03790356	0.068984094	15	0	0
COL14A1	0.8660891	0.892959485	-0.02687038	-0.04358364	0.037922572	0.069011693	1	0	0
UBL5	0.1280377	0.127044164	0.000993554	0.049660244	0.037993307	0.069133403	14	0	0
NELFCD	0.4098322	0.422842291	-0.01301007	-0.01901826	0.038004552	0.069146849	7	0	0
ZNF764	0.1912289	0.197513682	-0.00628481	-0.00620381	0.038010646	0.069150922	4	0	0
CAMK2G	0.1791895	0.194844194	-0.01565465	-0.03313879	0.038049215	0.069213251	6	0	0
GUCD1	0.1334387	0.151801817	-0.01836311	-0.07406692	0.038077763	0.069244932	13	0	0
TPP1	0.2835341	0.29675212	-0.01321804	-0.01611653	0.038103277	0.069284303	4	0	0
NFE2L2	0.0826841	0.091318961	-0.00863484	0.004865844	0.038107865	0.069285623	9	0	0
FBXL15	0.0828733	0.065215568	0.01765777	0.303768088	0.038137813	0.069333045	10	0	0
KRTAP2-2	0.7558572	0.80426569	-0.04840847	-0.08842417	0.038157528	0.069361856	1	0	0
AAGAB	0.3639924	0.363649641	0.000342714	-0.00456564	0.038179293	0.069394386	13	0	0
NCR3	0.7100275	0.746066619	-0.03603916	-0.078376	0.038194344	0.069409467	5	0	0
IPO4	0.3236612	0.330361364	-0.00670014	-0.02233714	0.038198446	0.069409467	12	0	0
ESRP2	0.4732024	0.485256981	-0.01205458	-0.03363339	0.0381992	0.069409467	5	0	0
PPTC7	0.1856637	0.18972608	-0.00406236	-0.02811211	0.038247701	0.069490555	8	0	0
ARID1A	0.1588352	0.1603603	-0.00152506	0.03452279	0.038276921	0.069536601	7	0	0
PAPSS2	0.2401179	0.226766781	0.013351154	0.121357741	0.038381821	0.069713047	14	0	0
GPR171	0.7109364	0.762255041	-0.05131866	-0.1031089	0.038420244	0.069775769	2	0	0
ZNF736	0.069775	0.039812426	0.029962608	0.763453753	0.03845974	0.069840427	5	0	0
OXNAD1	0.1364698	0.144937612	-0.00846777	-0.04512734	0.038511504	0.069927348	8	0	0
CRLF3	0.1343686	0.124418768	0.009949814	0.048274273	0.038541543	0.069974807	7	0	0
TAP2	0.6461054	0.665397093	-0.01929164	-0.02942875	0.038552512	0.069987638	16	0	0
C16orf45	0.4927706	0.47932266	0.013447977	0.266266192	0.038573918	0.07001941	9	0	0
LIN9	0.2634316	0.278921577	-0.01548993	0.034255164	0.038584114	0.070029058	12	0	0
TTC25	0.0469667	0.048920456	-0.00195373	-0.00684973	0.038587042	0.070029058	12	0	0
FLVCR2	0.0427535	0.038096032	0.004657437	0.136722669	0.038607355	0.070058835	6	0	0
PPP2R2C	0.190894	0.178452084	0.012441947	0.238273954	0.038634537	0.070101067	7	0	0
SCAI	0.2193551	0.232468425	-0.01311335	0.001618984	0.038700429	0.070213524	10	0	0
OTUD7B	0.2791326	0.280242575	-0.00110995	0.019376929	0.038715188	0.070233196	12	0	0
PLD3	0.1512385	0.162944556	-0.01170603	0.046770259	0.038730417	0.070253717	13	0	0
PPP1R1C	0.1718914	0.185169819	-0.01327846	-0.05908112	0.038740453	0.070264815	5	0	0
PRSS54	0.8015338	0.830128792	-0.02859496	-0.0525507	0.038798923	0.070363749	7	0	0
ZNF600	0.0255773	0.018161749	0.007415569	0.337220806	0.038807408	0.070372022	1	0	0
SKA2	0.0628398	0.057988022	0.004851809	0.050536161	0.038837855	0.070420115	15	0	0
RRP9	0.1712686	0.178005765	-0.00673712	-0.03944905	0.038843847	0.07042386	13	0	0
LINGO3	0.249956	0.241576727	0.008379232	0.378541359	0.038858032	0.070442457	8	0	0
PNPLA7	0.0366899	0.032629213	0.00406068	0.095462447	0.03887473	0.070465605	12	0	0
ELMOD2	0.1141919	0.120965589	-0.00677364	-0.00968403	0.038896231	0.070491323	11	0	0
ZNF462	0.0684567	0.05479973	0.01365699	0.255916339	0.038896779	0.070491323	9	0	0
ITGA9	0.0497135	0.029183236	0.020530228	0.608961399	0.038914295	0.070494576	5	0	0
POLD2	0.2927097	0.255284122	0.037425547	0.244631221	0.038909249	0.070494576	3	0	0
VPS35	0.0607739	0.070003284	-0.00922941	-0.10421031	0.038905835	0.070494576	13	0	0
KRT222	0.5377799	0.506637569	0.0311423	0.181959278	0.038913341	0.070494576	11	0	0
SEC11C	0.1903504	0.210897582	-0.02054722	-0.03748826	0.038959007	0.070568446	5	0	0
SKIL	0.0553627	0.046429631	0.00893311	0.125099406	0.038987623	0.070613149	7	0	0
TRPA1	0.1101525	0.083063759	0.027088779	0.497442109	0.03901896	0.070662769	8	0	0
ZNF570	0.1933588	0.161672729	0.031686113	0.578730019	0.039035018	0.070684714	12	0	0
DNAJC5G	0.8209303	0.824027693	-0.00309737	-0.00412967	0.039053158	0.070710422	11	0	0
RPL29	0.1574426	0.158265631	-0.00082304	-0.03326556	0.039059065	0.070713978	10	0	0
POLR2G	0.3814019	0.38578354	-0.00438161	-0.00263844	0.039080416	0.070745493	10	0	0
SPOP	0.1087662	0.098182294	0.010583887	0.115514715	0.039091573	0.070758548	7	0	0
NEK10	0.3288211	0.312471268	0.016349851	0.424987415	0.039125956	0.070813637	10	0	0
BCKDHB	0.1380988	0.154381411	-0.01628265	-0.03280355	0.039195438	0.070925079	7	0	0
ATP6V0E2	0.3389497	0.341261794	-0.00231205	0.088570381	0.039192208	0.070925079	12	0	0

KIF14	0.0750531	0.079183622	-0.00413049	-0.06550948	0.039223294	0.070946434	11	0	0
ZNF639	0.1667527	0.179059546	-0.01230689	0.068372908	0.039227018	0.070946434	5	0	0
YTHDC2	0.3511939	0.36137636	-0.01018249	-0.06170722	0.039225379	0.070946434	9	0	0
GPRIN1	0.1439566	0.144631938	-0.00067529	0.065097319	0.039212442	0.070946434	9	0	0
MVD	0.0928922	0.105591266	-0.01269902	-0.0162682	0.039226078	0.070946434	9	0	0
HS3ST1	0.3375969	0.352005575	-0.01440864	0.007644668	0.039241318	0.070965143	6	0	0
ARHGAP35	0.8701281	0.87861271	-0.00848458	-0.01421733	0.039249785	0.070973298	9	0	0
DHX8	0.043022	0.03822709	0.004794872	0.125034257	0.039271839	0.070998861	14	0	0
PLCXD2	0.1713006	0.181462304	-0.0101617	-0.03720238	0.039278012	0.071002865	16	0	0
ZNF862	0.2813492	0.298336573	-0.01698734	0.003015207	0.039323206	0.071077399	7	0	0
MBLAC2	0.2157774	0.231963833	-0.0161864	-0.08415906	0.039338357	0.071097618	12	0	0
ZCCHC14	0.3364938	0.300343845	0.036149989	0.174377732	0.039346031	0.071104323	7	0	0
DHDDS	0.0553472	0.059028497	-0.00368133	-0.02001856	0.039358617	0.071119903	13	0	0
TMBIM4	0.1724545	0.190994631	-0.01854016	0.106325638	0.039383972	0.071153983	10	0	0
COL1A1	0.2813264	0.28394536	-0.00261896	0.09294919	0.039385412	0.071153983	10	0	0
ZNF32	0.2071359	0.217816942	-0.01068108	0.007898632	0.039398949	0.071171271	10	0	0
MUCL1	0.6171379	0.658945321	-0.04180738	-0.09808514	0.039431877	0.071223579	2	0	0
HSPA12A	0.2240079	0.243286663	-0.01927873	-0.02805235	0.039442704	0.071235962	4	0	0
CCDC62	0.2027377	0.215366807	-0.01262907	0.044637713	0.039448859	0.071239906	12	0	0
GTF2H1	0.0933525	0.108686248	-0.01533378	-0.07277122	0.039470266	0.071249868	13	0	0
ACIN1	0.1584499	0.152974448	0.005475485	0.048058024	0.039465084	0.071249868	13	0	0
NMB	0.327705	0.33006374	-0.00235875	0.086864221	0.039460719	0.071249868	10	0	0
SMIM5	0.4625306	0.462987197	-0.0004566	0.025209483	0.039467591	0.071249868	10	0	0
CHTOP	0.3448461	0.332160555	0.012685552	0.026134017	0.039481161	0.071262364	9	0	0
ARHGAP44	0.1110766	0.121417108	-0.01034055	-0.01688886	0.039485327	0.071262712	9	0	0
WBP1	0.0518876	0.058714027	-0.00682638	-0.05610772	0.039489302	0.071262715	13	0	0
SF3A2	0.1056458	0.120976454	-0.01533066	-0.03928295	0.039493643	0.071263378	11	0	0
SPINK1	0.7655618	0.779932541	-0.01437076	-0.0293404	0.039514727	0.07129425	5	0	0
PRDX3	0.191439	0.189232245	0.00220677	0.081893912	0.039543375	0.071324413	11	0	0
INO80E	0.2356025	0.218445244	0.017157272	0.180813183	0.039538113	0.071324413	8	0	0
MAPK3	0.1579815	0.158174661	-0.00019318	0.069505934	0.03954305	0.071324413	6	0	0
DLGAP4	0.2542539	0.242802949	0.011450909	0.307886164	0.039549059	0.071327494	7	0	0
DNMT3A	0.3090842	0.31699447	-0.00791032	0.163767259	0.039581652	0.071373694	15	0	0
TRMT2A	0.104534	0.116896035	-0.01236208	-0.06385913	0.039582635	0.071373694	12	0	0
ECT2L	0.5206581	0.497314486	0.023343649	0.146454317	0.039597097	0.071392595	9	0	0
IQGAP2	0.0513343	0.040333085	0.01100123	0.319170944	0.039602999	0.071396058	6	0	0
NDUFA4L2	0.1113459	0.089347379	0.021998525	0.18039303	0.039618681	0.071409975	12	0	0
AP1M1	0.249225	0.260749767	-0.01152476	-0.08851693	0.039616592	0.071409975	3	0	0
CENPH	0.1948243	0.211237965	-0.01641364	-0.04785897	0.03963334	0.071421482	10	0	0
CDIPT	0.3372971	0.360313736	-0.02301668	-0.04583596	0.039637011	0.071421482	14	0	0
TGFB1	0.1479241	0.152601446	-0.00467737	0.054941309	0.039631499	0.071421482	7	0	0
C8orf82	0.3484126	0.32914279	0.01926982	0.126266833	0.039645705	0.071429972	10	0	0
DSG4	0.7584552	0.776723065	-0.01826785	-0.02716913	0.039655109	0.071439739	6	0	0
CLINT1	0.4217028	0.420516679	0.001186103	0.053048297	0.039670258	0.071451844	5	0	0
ZNF3	0.1481943	0.144717224	0.003477078	0.187917868	0.03967378	0.071451844	6	0	0
NUDT2	0.116078	0.143278746	-0.02720078	-0.07596743	0.03966785	0.071451844	6	0	0
EPHA6	0.1098698	0.087477526	0.022392254	0.276345007	0.039726431	0.071510765	12	0	0
DNMT1	0.0398349	0.040314717	-0.00047983	0.032814666	0.039722322	0.071510765	6	0	0
S1PR2	0.0398349	0.040314717	-0.00047983	0.032814666	0.039722322	0.071510765	6	0	0
PRODH	0.4645289	0.49487436	-0.03034547	-0.05161797	0.039713634	0.071510765	5	0	0
APOBEC3C	0.2033271	0.216217881	-0.01289075	-0.09222951	0.039724926	0.071510765	4	0	0
PPP1R12B	0.2466131	0.25595198	-0.0093389	-0.00283897	0.039749589	0.071531776	12	0	0
UBE2D1	0.3291729	0.342142245	-0.01296937	-0.04190399	0.039753746	0.071531776	11	0	0
CNPY2	0.2295018	0.231317669	-0.00181591	-0.03013071	0.039754056	0.071531776	14	0	0
CYB5B	0.3948279	0.385662037	0.00916587	0.050458302	0.039751975	0.071531776	11	0	0
OR11H4	0.7590334	0.805774273	-0.04674091	-0.0833057	0.039764475	0.071543346	3	0	0
CASP10	0.3305882	0.341102196	-0.01051397	0.004610815	0.039783794	0.071567598	5	0	0
CBX2	0.1479261	0.126690195	0.021235914	0.210072141	0.039785935	0.071567598	9	0	0
OGFOD3	0.2985085	0.316699564	-0.01819104	-0.02681501	0.039818321	0.071618673	8	0	0
DGKZ	0.1734243	0.160599548	0.012824782	0.41864452	0.03983756	0.071646093	14	0	0
IGFL3	0.5492139	0.600441486	-0.05122762	-0.12879023	0.039867845	0.07169337	2	0	0
RPF2	0.2672917	0.264415964	0.002875693	0.075826906	0.039883731	0.071714747	10	0	0
ZNF547	0.1021069	0.088533671	0.013573211	0.14004183	0.039929347	0.071789572	14	0	0

ADPRH	0.3798611	0.357220662	0.02264042	0.300241077	0.039945954	0.071812232	14	0	0
SERPINI2	0.7829305	0.799967498	-0.01703699	-0.03405965	0.039988972	0.071880536	2	0	0
CTSC	0.0413339	0.044989771	-0.00365583	-0.09035492	0.039991964	0.071880536	7	0	0
RFWD3	0.1164029	0.114304091	0.002098764	0.098037412	0.040001605	0.071890661	11	0	0
MBLAC1	0.547802	0.559250077	-0.01144808	-0.03171222	0.04005799	0.071984782	7	0	0
C5orf51	0.110365	0.131711173	-0.02134622	-0.04718989	0.040062779	0.071986175	10	0	0
MBIP	0.2389711	0.247463393	-0.00849232	0.052909854	0.040100137	0.072046083	11	0	0
CDK20	0.3082509	0.305077852	0.003173024	0.141987662	0.040123375	0.072080614	9	0	0
SPEF1	0.4560616	0.490804038	-0.0347424	-0.04647679	0.040144907	0.072112074	7	0	0
MRPL43	0.2145219	0.226340836	-0.01181897	-0.00540634	0.040161272	0.072134246	16	0	0
MROH7	0.7472864	0.768376032	-0.02108968	-0.0358864	0.04016976	0.072135125	5	0	0
NOP16	0.1099802	0.114361957	-0.00438176	-0.03735802	0.040169805	0.072135125	13	0	0
CLEC4F	0.7253371	0.736072945	-0.01073587	-0.01912572	0.040214884	0.072201622	5	0	0
FEM1A	0.0753989	0.078090549	-0.00269162	-0.06356155	0.040214886	0.072201622	10	0	0
LSM14A	0.0642394	0.074502048	-0.01026262	-0.03419127	0.04023582	0.072231976	10	0	0
PRSS48	0.7687266	0.765313267	0.003413361	0.008536315	0.04028684	0.072316329	6	0	0
RHBDD2	0.4043999	0.455986679	-0.05158681	-0.16926515	0.040324062	0.072368659	1	0	0
LHPP	0.2356656	0.253060427	-0.01739487	-0.06899451	0.040322291	0.072368659	6	0	0
01-Mar	0.4357596	0.409079016	0.026680613	0.163107013	0.040364876	0.072434658	8	0	0
PELI1	0.0517539	0.055234123	-0.00348022	-0.04863119	0.040389362	0.072465842	11	0	0
ABCA1	0.3315575	0.341277338	-0.00971979	-0.04637683	0.040390334	0.072465842	6	0	0
BUB3	0.2690959	0.286341903	-0.01724603	-0.01987978	0.04042313	0.07251743	8	0	0
TPRG1L	0.1195325	0.121801776	-0.00226925	0.049162895	0.040449284	0.072557091	9	0	0
MLF1	0.0800206	0.066882578	0.013138022	0.264660931	0.040457526	0.072564618	12	0	0
ZNF585A	0.2067252	0.183612003	0.023113188	0.629897928	0.04046886	0.07257769	15	0	0
RNF8	0.3422525	0.364295255	-0.02204272	-0.03562388	0.040505761	0.072636606	9	0	0
ABCC4	0.1528575	0.171616349	-0.01875886	-0.07297785	0.040520846	0.072656392	7	0	0
ZNF384	0.0963145	0.112161104	-0.0158466	-0.04484441	0.040566853	0.072731615	13	0	0
JSRP1	0.0298861	0.028581848	0.001304221	0.037912433	0.040598024	0.072780226	8	0	0
ABCB10	0.0934489	0.110619517	-0.01717067	-0.04999277	0.040604305	0.07278421	8	0	0
XPOT	0.2910347	0.317157285	-0.02612262	0.003696906	0.040628592	0.072820468	6	0	0
SNRNP35	0.5541238	0.581636065	-0.02751225	-0.05751592	0.04063468	0.072824102	3	0	0
SMARCB1	0.2801832	0.269061417	0.011121764	0.046921691	0.040691769	0.072919129	7	0	0
UNKL	0.283304	0.288344911	-0.00504087	0.05255892	0.040717745	0.072958389	6	0	0
PLBD2	0.2230618	0.238787409	-0.0157256	0.061307685	0.040733852	0.072972669	7	0	0
LRRC29	0.1555434	0.152682501	0.002860857	0.021837533	0.040732996	0.072972669	17	0	0
ATP6V1C1	0.1849588	0.219355352	-0.03439652	-0.09396137	0.040750659	0.072985256	17	0	0
TPM4	0.4798515	0.449353581	0.030497893	0.09493151	0.040753085	0.072985256	9	0	0
TSACC	0.5336832	0.56456863	-0.03088541	-0.08021861	0.040763069	0.072995847	6	0	0
CNNM4	0.170412	0.181570111	-0.01115808	-0.0233688	0.040797283	0.073049822	13	0	0
PRDM1	0.1106556	0.121408602	-0.01075295	-0.0737948	0.040805802	0.073057783	11	0	0
GYS1	0.0351099	0.035398177	-0.00028829	0.009819614	0.040823992	0.073075761	8	0	0
RUVBL2	0.0351099	0.035398177	-0.00028829	0.009819614	0.040823992	0.073075761	8	0	0
PFKFB2	0.6388572	0.678297848	-0.03944063	-0.09306147	0.040851496	0.073117695	5	0	0
MFSD2A	0.2540564	0.245832954	0.008223447	0.155781572	0.040858736	0.073123358	17	0	0
RXRB	0.1729259	0.197041431	-0.02411557	-0.05149494	0.040868353	0.073133272	30	0	0
PRR13	0.0506611	0.048582511	0.002078572	0.072862918	0.040877462	0.073134979	11	0	0
SLC39A9	0.0944155	0.106225632	-0.01181015	-0.07218252	0.040874622	0.073134979	10	0	0
PROSER2	0.1774163	0.182857446	-0.00544113	-0.04969923	0.040891714	0.07315318	7	0	0
GIMAP1	0.4407986	0.451901997	-0.01110336	0.087634393	0.040902535	0.073165241	5	0	0
CEP68	0.2317465	0.246620237	-0.01487376	-0.01094431	0.040908581	0.073168759	6	0	0
C1orf35	0.7504913	0.776269873	-0.02577862	-0.0490216	0.040951672	0.073238527	4	0	0
VPS33A	0.1024994	0.11159545	-0.00909608	0.06486925	0.040959545	0.073245304	3	0	0
COX6A1	0.0454621	0.04629574	-0.00083368	-0.02594563	0.040964942	0.073247653	10	0	0
SPCS1	0.3889903	0.402472458	-0.01348218	0.001303384	0.041011922	0.073317037	10	0	0
SPCS3	0.2301625	0.247544734	-0.01738223	-0.0051798	0.041010063	0.073317037	6	0	0
ING4	0.1314268	0.132176706	-0.00074989	0.074735569	0.041016624	0.073318136	10	0	0
TRIM17	0.1640202	0.127754475	0.036265757	0.584437283	0.041030425	0.07332977	9	0	0
PQLC3	0.0622256	0.062978138	-0.00075249	0.043330548	0.04103131	0.07332977	12	0	0
TCOF1	0.2779521	0.290743603	-0.01279155	-0.02507477	0.041085618	0.073419511	11	0	0
SLC6A6	0.1958885	0.205956851	-0.01006833	-0.01477026	0.041111415	0.073455881	9	0	0
CHAD	0.4131826	0.357885626	0.055296961	0.288926297	0.041114161	0.073455881	12	0	0
PIKFYVE	0.0933905	0.102897	-0.00950651	-0.02098814	0.041173952	0.073555379	11	0	0

NDUFS3	0.2954296	0.307458265	-0.01202863	0.064247234	0.041196425	0.073588197	9	0	0
ATOH8	0.197243	0.218434751	-0.02119173	-0.14045864	0.041217062	0.073617728	1	0	0
R3HDM2	0.236369	0.252309498	-0.0159405	0.022054301	0.041252163	0.073667068	4	0	0
B3GALT5	0.6509347	0.711389997	-0.06045527	-0.12627828	0.041252901	0.073667068	2	0	0
BEND4	0.443815	0.445721117	-0.00190614	0.103268498	0.041263586	0.073678813	6	0	0
GCDH	0.0943443	0.102432823	-0.00808851	-0.00499523	0.041270318	0.073683499	11	0	0
BTBD3	0.3601945	0.3443918	0.015802653	0.228367477	0.041283084	0.073698955	11	0	0
ZBTB4	0.0345304	0.033894607	0.000635771	0.028665532	0.041321778	0.073760689	12	0	0
ZNF263	0.3766191	0.38591054	-0.00929141	0.003841558	0.041326681	0.0737621	7	0	0
CLMP	0.302494	0.260479096	0.042014914	0.299334586	0.041333725	0.073767332	8	0	0
STX2	0.1854631	0.174641326	0.010821769	0.011788359	0.041348635	0.0737866	6	0	0
TBC1D7	0.3807699	0.400632292	-0.01986243	-0.05889079	0.041367141	0.073804942	7	0	0
MKLN1	0.08547	0.087221274	-0.00175123	0.138063544	0.041367143	0.073804942	12	0	0
GDF15	0.1451123	0.146397552	-0.00128525	0.109952873	0.041381867	0.073823868	2	0	0
SHE	0.2349266	0.197838283	0.037088352	0.489119014	0.041394586	0.073831872	8	0	0
ALKBH4	0.1260155	0.142110201	-0.01609467	-0.02260692	0.041393107	0.073831872	8	0	0
KCNJ13	0.850217	0.853877153	-0.00366019	-0.00527783	0.041453525	0.073929644	3	0	0
RAD51B	0.1996743	0.204057751	-0.00438345	0.020140066	0.0414809	0.07397111	9	0	0
FER	0.3367612	0.327175511	0.009585736	0.102092365	0.041492469	0.073984385	12	0	0
MBOAT7	0.038646	0.037152483	0.001493468	0.04669236	0.041518068	0.074022671	19	0	0
MMP25	0.0484027	0.036620143	0.011782526	0.322319243	0.041527708	0.074032498	8	0	0
KRBA1	0.440188	0.423365323	0.016822682	0.107190356	0.041620316	0.07419022	8	0	0
SPATA6	0.1403733	0.148629138	-0.00825587	0.058469016	0.04163305	0.074205544	12	0	0
P4HA3	0.3152279	0.332050048	-0.01682214	0.013579376	0.041638654	0.074208158	14	0	0
MNX1	0.1326818	0.106012178	0.026669639	0.332201089	0.041676855	0.074268859	6	0	0
PKDCC	0.1650553	0.145090907	0.019964421	0.203861839	0.041691245	0.074275785	6	0	0
ZNF398	0.043352	0.041472904	0.001879122	0.068961453	0.041693165	0.074275785	10	0	0
PCDHB6	0.4100797	0.388754116	0.021325625	0.214005689	0.041700714	0.074281856	9	0	0
HOXD1	0.1703008	0.138072605	0.032228183	0.310204964	0.041707087	0.074285831	15	0	0
SAMSN1	0.8522838	0.87165769	-0.01937386	-0.03237351	0.041719084	0.07429982	6	0	0
MYEF2	0.3687136	0.375530156	-0.00681652	0.067054157	0.041732145	0.074308712	13	0	0
RBL1	0.1027629	0.106806025	-0.00404317	-0.05801003	0.041732362	0.074308712	13	0	0
GRM2	0.4709142	0.465530757	0.005383433	0.032563447	0.041757574	0.074346223	7	0	0
SH3RF2	0.1536047	0.133990132	0.019614591	0.13291459	0.04176417	0.074350586	10	0	0
CCDC84	0.0434871	0.033406632	0.010080436	0.268050958	0.041771144	0.074355621	6	0	0
ZC3H18	0.1565011	0.160434366	-0.00393322	-0.00410791	0.041788088	0.074378402	8	0	0
HDAC5	0.0873992	0.098565843	-0.01116667	-0.03749966	0.041793226	0.074380165	11	0	0
EFHD1	0.14209	0.14651488	-0.00442492	-0.00854883	0.041798887	0.074382859	10	0	0
ZNF654	0.8427342	0.852221463	-0.0094873	-0.01577625	0.041876793	0.074514104	8	0	0
MGAT4B	0.1221352	0.137921852	-0.01578667	-0.0595602	0.041890005	0.074530218	10	0	0
IL5RA	0.1150619	0.123990945	-0.00892908	-0.0784869	0.041915833	0.074568774	9	0	0
CA12	0.3209303	0.329611045	-0.00868072	0.013666323	0.041946877	0.074616601	6	0	0
TSNAXIP1	0.1109447	0.114510909	-0.0035662	-0.03007223	0.041972308	0.074654434	12	0	0
SLC35C2	0.3536348	0.371435064	-0.01780024	0.035230063	0.041988194	0.074675285	15	0	0
FGF21	0.6822984	0.706529892	-0.02423151	-0.05147346	0.042003934	0.074695871	7	0	0
CACNA1D	0.0647629	0.069555547	-0.00479266	-0.06980285	0.042072264	0.074795137	6	0	0
INSM2	0.1308262	0.116017453	0.014808772	0.613471216	0.04207142	0.074795137	12	0	0
MVP	0.4171299	0.427249552	-0.01011964	-0.05674407	0.04207117	0.074795137	9	0	0
GAL3ST1	0.4624408	0.407755275	0.054685542	0.235291657	0.042086566	0.074813147	3	0	0
MRPS30	0.1728629	0.190795656	-0.01793273	-0.09430715	0.042103464	0.074835768	12	0	0
ZNF232	0.0734647	0.057651409	0.015813317	0.354313486	0.042120217	0.07484027	3	0	0
ZNF559	0.2298984	0.230513445	-0.00061502	0.213635487	0.042122688	0.07484027	10	0	0
ZNF559-ZNI	0.2298984	0.230513445	-0.00061502	0.213635487	0.042122688	0.07484027	10	0	0
AKT2	0.2561219	0.252892509	0.003229344	-0.01663094	0.04211809	0.07484027	9	0	0
KIAA0100	0.1106105	0.097426214	0.01318424	0.063685249	0.042137653	0.074859444	10	0	0
GNAI3	0.0442475	0.052826566	-0.00857902	-0.02565271	0.042149123	0.074872405	16	0	0
HES6	0.1996164	0.211363024	-0.01174664	-0.09365467	0.042178567	0.074903124	6	0	0
WIPF3	0.3100845	0.311025209	-0.00094071	0.053140159	0.042171639	0.074903124	10	0	0
PAG1	0.2689332	0.284283888	-0.01535069	-0.00489943	0.042178945	0.074903124	7	0	0
BIN1	0.0798064	0.082206081	-0.00239971	0.037166787	0.042193368	0.074903172	10	0	0
GNAI2	0.1927826	0.202204122	-0.0094215	-0.05884064	0.042201686	0.074903172	7	0	0
TMEM151B	0.1896734	0.178224692	0.011448673	0.338287997	0.042198247	0.074903172	5	0	0
TCTE1	0.2613116	0.259830074	0.001481522	0.159104497	0.042206596	0.074903172	7	0	0

KRT12	0.7644813	0.754425011	0.010056265	0.026760791	0.042201046	0.074903172	6	0	0
IGF2BP1	0.1116441	0.080364944	0.031279123	0.646141815	0.042206881	0.074903172	18	0	0
CD300A	0.3994271	0.386324226	0.01310286	0.097126596	0.042208205	0.074903172	11	0	0
CLRN1	0.597688	0.577125099	0.020562916	0.053407687	0.042225937	0.074927227	8	0	0
EEFSEC	0.3962944	0.410162181	-0.01386782	0.005163162	0.042232169	0.074930872	7	0	0
FBXO2	0.8441309	0.882908991	-0.03877805	-0.06405588	0.042252558	0.074959632	1	0	0
ZAP70	0.5332599	0.542682894	-0.00942301	-0.01223497	0.042289505	0.075012095	9	0	0
HOXA7	0.4768271	0.437748877	0.039078251	0.141757666	0.042290494	0.075012095	10	0	0
CHMP4A	0.4758127	0.474379247	0.001433497	0.140683682	0.042314	0.075046366	6	0	0
SPATA9	0.7578206	0.797330661	-0.03951008	-0.07239045	0.042318313	0.075046595	1	0	0
R3HCC1	0.493032	0.45256581	0.040466149	0.131041206	0.042337652	0.075070582	2	0	0
CENPN	0.1228357	0.146723168	-0.02388746	-0.08085189	0.04234021	0.075070582	7	0	0
ANKRD50	0.1897739	0.180938199	0.008835717	0.137646311	0.04235466	0.075088779	9	0	0
EEF1E1	0.2425681	0.27569913	-0.03313101	-0.06338233	0.042372213	0.075106862	9	0	0
GLDN	0.4543377	0.465087129	-0.01074939	0.050907962	0.042373234	0.075106862	9	0	0
CTSA	0.3977348	0.377830847	0.019903907	0.265139759	0.042379149	0.075109922	5	0	0
LIMS1	0.1392033	0.169837758	-0.03063447	-0.06386774	0.042390385	0.075122414	5	0	0
ARCN1	0.238748	0.245885696	-0.00713766	0.027096645	0.042440604	0.075203978	11	0	0
NBR1	0.0477513	0.044937788	0.002813503	0.067311306	0.042446217	0.075206495	11	0	0
SOWAHA	0.2089029	0.205079957	0.003822971	0.102248168	0.042462969	0.075224661	13	0	0
EFCAB5	0.0376497	0.040684379	-0.00303468	-0.06429137	0.042464858	0.075224661	4	0	0
NPDC1	0.1466185	0.153850048	-0.00723153	0.197114529	0.042475591	0.075228813	7	0	0
FBXL20	0.4115791	0.458218631	-0.04663951	-0.08317777	0.042474246	0.075228813	4	0	0
MAPK7	0.1451988	0.153650467	-0.00845164	0.01200487	0.04252401	0.075307133	8	0	0
AMD1	0.1481664	0.165333629	-0.01716723	0.010144701	0.042562479	0.075367817	10	0	0
PRR11	0.0438316	0.043047892	0.000783735	0.031337462	0.042591153	0.075411146	15	0	0
SLC7A6	0.2967567	0.292440426	0.004316319	0.018093807	0.04260716	0.075432043	8	0	0
POMK	0.0430543	0.042702214	0.000352048	-0.00360661	0.042621626	0.075450206	9	0	0
NT5DC2	0.5298667	0.562015055	-0.03214836	-0.09576104	0.042653543	0.075491804	9	0	0
TTL	0.081227	0.098981039	-0.01775408	-0.09030128	0.04266393	0.075502738	7	0	0
ZDHHC24	0.0947859	0.069390851	0.02539508	0.394941273	0.042687397	0.075536815	14	0	0
PSEN1	0.1347597	0.152202237	-0.01744249	-0.04010881	0.042729962	0.075604675	10	0	0
TM9SF2	0.1798145	0.202668488	-0.02285403	-0.06982203	0.04276035	0.07565098	10	0	0
HINT3	0.1698478	0.185233839	-0.01538602	-0.04783527	0.042824127	0.075756341	10	0	0
TSPAN9	0.3773905	0.383218406	-0.00582793	0.047239494	0.042839815	0.07577662	5	0	0
RBM45	0.3841258	0.396381461	-0.01225563	-0.00330299	0.042874413	0.07583034	11	0	0
SNAPC4	0.6768388	0.699519199	-0.02268042	-0.02513488	0.042914066	0.07589002	7	0	0
PRDX5	0.03681	0.045758349	-0.00894839	-0.05877493	0.042916619	0.07589002	14	0	0
ZNF101	0.1811887	0.175510064	0.005678619	0.114204038	0.042938376	0.075921008	7	0	0
SPG11	0.1828256	0.190039163	-0.00721357	-0.08724638	0.04295214	0.075937859	12	0	0
PRR18	0.5346619	0.554065627	-0.01940375	0.173941825	0.042969908	0.07595623	3	0	0
LRRC8E	0.2512087	0.262624003	-0.0114153	0.066956642	0.042971001	0.07595623	11	0	0
PTER	0.042802	0.039948973	0.002852989	0.084230992	0.042993011	0.07598016	9	0	0
MMP10	0.543602	0.560394462	-0.01679251	-0.0535516	0.04299188	0.07598016	4	0	0
TNNI3	0.5426893	0.552185447	-0.00949618	-0.00444672	0.042998368	0.075982141	7	0	0
MAPKAP1	0.0601953	0.067309816	-0.00711451	-0.07429853	0.04308866	0.076134194	7	0	0
CNFN	0.3978024	0.412444762	-0.01464232	-0.0195425	0.043119615	0.076181384	10	0	0
COQ7	0.1866975	0.189150001	-0.00245246	0.043797093	0.043154112	0.076234823	10	0	0
ZNF586	0.279386	0.249235278	0.030150684	0.549283806	0.043191246	0.076292909	7	0	0
PADI6	0.6522344	0.631969735	0.020264652	0.058033117	0.043197704	0.07629538	5	0	0
NKIRAS2	0.485759	0.515962817	-0.03020382	-0.00408666	0.043201153	0.07629538	2	0	0
ZNF267	0.3147987	0.333998171	-0.01919952	-0.03727761	0.043217703	0.076317095	12	0	0
CYFIP2	0.1981212	0.209443683	-0.01132246	-0.02208224	0.043277576	0.076415298	8	0	0
IZUMO4	0.190807	0.201227268	-0.01042028	-0.00272827	0.043295479	0.076439384	10	0	0
ETF1	0.1263034	0.135859672	-0.00955629	-0.05498668	0.04330178	0.076442985	8	0	0
GMPR2	0.104871	0.104263747	0.000607276	0.070799764	0.043335828	0.076495562	10	0	0
SDF4	0.7310598	0.726410622	0.004649191	0.016604497	0.043346251	0.076504631	5	0	0
ZNF414	0.2987619	0.31856698	-0.01980509	-0.04541834	0.043349496	0.076504631	12	0	0
DPCD	0.1000271	0.09799272	0.002034417	0.045502756	0.043365162	0.076521781	8	0	0
APOBEC3A	0.6004692	0.631767426	-0.03129819	-0.08823185	0.043367747	0.076521781	5	0	0
TRIM69	0.2846178	0.307629904	-0.02301209	-0.06552486	0.043377476	0.07653142	3	0	0
ATP1A3	0.1231981	0.100932825	0.022265305	0.338163195	0.043402985	0.076568894	3	0	0
PRKCQ	0.0571902	0.04991639	0.007273822	0.173208979	0.043483714	0.076703766	8	0	0

LDHAL6B	0.9291815	0.938238874	-0.00905742	-0.01431173	0.043499899	0.07672477	4	0	0
IL1F10	0.7960152	0.823293994	-0.02727877	-0.04870695	0.043519301	0.076743898	3	0	0
HEPACAM2	0.606823	0.653448002	-0.04662496	-0.10944645	0.043518146	0.076743898	5	0	0
PIWIL2	0.7329152	0.742473339	-0.0095581	-0.00650022	0.043539634	0.076772205	14	0	0
WDR24	0.2575142	0.288701093	-0.03118694	-0.06543393	0.043548486	0.076780266	5	0	0
POMT1	0.2732043	0.287152784	-0.01394852	0.05483077	0.043558072	0.076789619	10	0	0
BHLHE22	0.1049276	0.089014261	0.015913324	0.130279831	0.043595219	0.076840002	13	0	0
LUM	0.4792388	0.443251284	0.035987491	0.141062115	0.043595212	0.076840002	7	0	0
CYP7A1	0.6417236	0.694236236	-0.0525126	-0.11111634	0.043605835	0.076851161	2	0	0
MCAM	0.1252969	0.078944008	0.046352871	0.88064155	0.043621241	0.076870757	4	0	0
OGFOD2	0.0447072	0.049870987	-0.00516382	-0.08504958	0.043630199	0.076878991	17	0	0
RCHY1	0.1374023	0.166367226	-0.02896489	-0.07941882	0.04365563	0.076909639	12	0	0
CCDC151	0.0755578	0.080288751	-0.00473093	-0.02495202	0.043656168	0.076909639	13	0	0
TSEN34	0.0508417	0.051481221	-0.00063949	0.019996886	0.043694654	0.07696988	12	0	0
IGSF10	0.7261811	0.703799599	0.022381541	0.071667904	0.043714353	0.076997018	6	0	0
RNASET2	0.2988796	0.312746906	-0.01386735	-0.06441319	0.043724035	0.07700651	5	0	0
TGFA	0.1637338	0.171257767	-0.00752397	-0.0327088	0.043741197	0.077029171	12	0	0
DPEP3	0.7286323	0.717615957	0.011016353	0.034548391	0.043747807	0.077033248	9	0	0
RNF38	0.0244449	0.020410529	0.004034406	0.204749583	0.043753842	0.077036313	2	0	0
UVSSA	0.0406162	0.036823701	0.003792453	0.071042024	0.043783186	0.077072845	18	0	0
BRSK1	0.5621577	0.55895959	0.003198155	0.035978339	0.043779674	0.077072845	6	0	0
TSPAN3	0.2544656	0.272075573	-0.01761001	-0.01707232	0.04379373	0.07708384	7	0	0
DDX51	0.1166401	0.133782675	-0.0171426	-0.0419094	0.043820213	0.077122887	7	0	0
GALNT2	0.622406	0.655675725	-0.03326973	-0.06887254	0.043832373	0.077136719	7	0	0
FAM181B	0.2264716	0.21714669	0.009324877	0.130804484	0.043847185	0.077147964	8	0	0
ANKRD33	0.616724	0.62668826	-0.00996427	-0.00553665	0.043847365	0.077147964	7	0	0
CEBPG	0.0778133	0.097766775	-0.0199535	-0.0681007	0.043894213	0.077222816	7	0	0
GMPPA	0.089055	0.072490799	0.016564249	0.131636005	0.043903562	0.077224114	14	0	0
AKAP5	0.0665329	0.067088465	-0.00055558	0.059462719	0.043900747	0.077224114	12	0	0
ANKRD16	0.151275	0.1740103	-0.02273527	-0.04298464	0.043914966	0.077236598	10	0	0
PPP1CA	0.1795334	0.195162792	-0.01562942	0.061534255	0.043937328	0.077268351	5	0	0
ETV3L	0.7258216	0.759659566	-0.03383796	-0.06892869	0.043943283	0.077271247	4	0	0
IRS2	0.0943152	0.075608269	0.018706904	0.346493664	0.043995615	0.077355686	13	0	0
GPR179	0.6952526	0.720919811	-0.02566722	-0.05021088	0.044011658	0.077376309	9	0	0
RIMKLA	0.1353645	0.142961216	-0.00759667	0.032875773	0.044051496	0.077436995	8	0	0
CTF1	0.3722704	0.390201213	-0.0179308	-0.04100762	0.044054811	0.077436995	7	0	0
P2RY13	0.7305698	0.768180577	-0.03761076	-0.07155895	0.044069378	0.077455008	3	0	0
PKN3	0.2917373	0.292478696	-0.00074138	0.066712368	0.044096797	0.077495606	7	0	0
NUDT3	0.1943841	0.198546743	-0.00416262	0.032029882	0.044136485	0.077557753	7	0	0
NDC1	0.2385096	0.225053864	0.013455759	0.055142384	0.0441469	0.07755911	8	0	0
ZNF488	0.064503	0.068265574	-0.00376259	-0.05662716	0.044150229	0.07755911	10	0	0
IDH3A	0.0680124	0.06887411	-0.00086172	0.196486828	0.044147618	0.07755911	6	0	0
USP47	0.3816743	0.402919111	-0.02124477	-0.03965409	0.044160818	0.077570114	7	0	0
ARV1	0.0635815	0.069498854	-0.00591739	-0.0258865	0.044173909	0.077585467	12	0	0
FN1	0.0602159	0.048977951	0.011237967	0.218424356	0.04417821	0.077585467	7	0	0
KIAA1614	0.1778662	0.149162235	0.028703922	0.301667185	0.044203048	0.077621488	9	0	0
C2orf50	0.2569334	0.250786537	0.006146906	0.027430727	0.044214471	0.077633945	9	0	0
POP7	0.1440626	0.156064764	-0.01200214	-0.00174794	0.044231494	0.077651187	7	0	0
NINJ2	0.7660219	0.812455222	-0.04643329	-0.08983774	0.04423295	0.077651187	5	0	0
FHIT	0.1783189	0.186799924	-0.00848103	-0.0473227	0.04425231	0.077677573	12	0	0
HBA1	0.1443128	0.125867411	0.018445372	0.164812925	0.04426189	0.077686786	3	0	0
ZDHHC17	0.3974256	0.417633587	-0.02020796	-0.03961736	0.044269441	0.077692436	9	0	0
FBXO4	0.1872265	0.195818368	-0.00859183	0.034241222	0.044308769	0.077753849	11	0	0
C3orf33	0.2478112	0.268348981	-0.02053777	-0.04373042	0.044338838	0.077791391	7	0	0
IL27	0.8892568	0.912048414	-0.02279166	-0.03610942	0.044338099	0.077791391	1	0	0
GRPEL2	0.1388585	0.148880196	-0.01002174	-0.04764174	0.044353425	0.077809372	11	0	0
PPARGC1A	0.5984011	0.645238092	-0.04683703	-0.10699656	0.044379547	0.077847585	1	0	0
TP63	0.5616065	0.598433595	-0.03682706	-0.04882538	0.044390463	0.077853372	7	0	0
DCAF13	0.1506445	0.16665468	-0.01601016	-0.05843829	0.044391527	0.077853372	7	0	0
NPBWR2	0.5843906	0.615732209	-0.03134158	-0.04331864	0.044403579	0.077866895	11	0	0
PID1	0.3912123	0.411186448	-0.01997412	0.052368841	0.044466593	0.077962151	8	0	0
CATSPERG	0.1719735	0.191202479	-0.01922899	-0.03572173	0.044464975	0.077962151	6	0	0
SNX16	0.4976557	0.507666768	-0.01001106	-0.05976134	0.044485006	0.077986811	5	0	0

C1RL	0.1591643	0.170098719	-0.01093445	0.003777475	0.044499239	0.078004139	12	0	0
RFX5	0.1491846	0.14981474	-0.00063011	0.033911091	0.044508246	0.078012304	12	0	0
RBM27	0.0544683	0.05206819	0.002400134	0.057284	0.044541665	0.07806325	7	0	0
PIM1	0.0938183	0.080832664	0.012985628	0.088547236	0.044565591	0.078097552	8	0	0
SUPT7L	0.16373	0.189330474	-0.02560049	-0.05382302	0.044581229	0.078117324	19	0	0
HSF5	0.7647843	0.75415212	0.010632195	0.032333834	0.044607196	0.078155189	10	0	0
PARD6A	0.0867218	0.077562667	0.009159104	0.064183632	0.044613713	0.078158972	9	0	0
FAM171A2	0.0824157	0.078364996	0.004050699	-0.0009095	0.044619124	0.078160817	6	0	0
ELMOD3	0.1263529	0.15295149	-0.0265986	-0.07614394	0.044635367	0.078181636	8	0	0
PCBP2	0.0488804	0.04608222	0.002798148	0.105065832	0.044641589	0.078184898	12	0	0
TMEM140	0.0986269	0.102339703	-0.00371275	-0.02948168	0.044717582	0.078310345	11	0	0
MFSD5	0.424341	0.417538264	0.00680276	0.095530131	0.044728286	0.078313798	8	0	0
RDH5	0.749667	0.729560123	0.020106849	0.043642288	0.044726502	0.078313798	6	0	0
LRAT	0.2551681	0.235423492	0.019744606	0.268446726	0.0447592	0.078360274	6	0	0
CSTF1	0.0936048	0.108760032	-0.01515519	-0.05378018	0.04478251	0.078393431	13	0	0
ASIC3	0.6176752	0.63564933	-0.01797409	-0.02911707	0.044805097	0.078425316	11	0	0
PTGIS	0.374316	0.37063744	0.003678511	0.244816811	0.044853829	0.078502954	8	0	0
CXCL1	0.2439865	0.256788644	-0.01280213	0.09037784	0.044880333	0.078526354	5	0	0
ECD	0.0330311	0.02910947	0.003921593	0.137860498	0.044876953	0.078526354	1	0	0
TRAF4	0.197393	0.211143277	-0.0137503	-0.06289596	0.044876751	0.078526354	8	0	0
INTS12	0.119405	0.116642548	0.002762452	0.122660972	0.045104323	0.078910568	5	0	0
NME9	0.2630901	0.269724215	-0.00663416	0.083527911	0.04517822	0.079032142	12	0	0
HIST1H2BO	0.0992464	0.112070152	-0.01282375	-0.03973709	0.045197784	0.079058656	8	0	0
CCDC136	0.1012423	0.075601346	0.025640972	0.307350746	0.045237699	0.079109643	5	0	0
ANKRD11	0.0512321	0.051450921	-0.00021879	0.042436529	0.045240166	0.079109643	15	0	0
LAMP5	0.2326897	0.235245868	-0.00255615	0.204767138	0.04523564	0.079109643	11	0	0
FBL	0.3278275	0.332043292	-0.00421576	0.013794794	0.045246608	0.079113195	10	0	0
AKR1A1	0.0992852	0.103037578	-0.0037524	-0.06698499	0.045275186	0.079155447	9	0	0
CASC4	0.1300115	0.11720863	0.012802884	0.112456326	0.045283839	0.079162859	11	0	0
RHOB	0.0687044	0.059324712	0.009379727	0.156106794	0.045291099	0.079167835	8	0	0
CDCA5	0.2001815	0.206715788	-0.00653429	0.017094549	0.0453179	0.079206963	11	0	0
MEIG1	0.3300248	0.352651773	-0.02262693	-0.04942061	0.045382284	0.079311765	7	0	0
FBXL8	0.0749508	0.085659757	-0.01070897	-0.0562835	0.045388524	0.079314942	15	0	0
TSPAN12	0.2773091	0.25385627	0.023452855	0.092245536	0.045394167	0.079317076	10	0	0
VAPB	0.3057507	0.326452914	-0.02070218	-0.02515079	0.04540466	0.079327683	8	0	0
FLT4	0.1847873	0.187568677	-0.0027814	0.099483904	0.04541794	0.079335427	5	0	0
SHF	0.2590629	0.267544785	-0.00848191	0.006195521	0.045413559	0.079335427	10	0	0
EPHX1	0.5507125	0.560917746	-0.01020529	-0.01755854	0.045426704	0.07934301	5	0	0
PTPN23	0.1486504	0.155945944	-0.00729551	-0.08187406	0.045438173	0.079352189	7	0	0
FAM69C	0.4406168	0.463167442	-0.02255063	-0.00570517	0.045440808	0.079352189	4	0	0
GCA	0.0679891	0.077925111	-0.00993605	-0.06200635	0.045448337	0.07935761	11	0	0
ARL8B	0.3782865	0.37825021	3.63E-05	0.013379274	0.045461757	0.079373316	11	0	0
CYTIP	0.6866781	0.691205104	-0.00452698	0.007376731	0.045480127	0.07938045	4	0	0
ZNF148	0.1369821	0.15989725	-0.02291519	-0.0619957	0.045483546	0.07938045	11	0	0
C7orf50	0.1556053	0.171401578	-0.01579626	-0.03811578	0.045473731	0.07938045	9	0	0
EEF2	0.0761186	0.073249482	0.002869167	0.027560344	0.045474843	0.07938045	9	0	0
PSMA5	0.0532698	0.061638429	-0.00836864	-0.02004241	0.045490976	0.079385692	11	0	0
ASH1L	0.0395545	0.037434624	0.002119833	0.052878119	0.045495771	0.079386336	9	0	0
ZNF513	0.2127721	0.230191775	-0.01741967	0.015866983	0.045503361	0.079391856	11	0	0
CD22	0.4029725	0.422449394	-0.01947689	-0.136358	0.045510265	0.07939618	2	0	0
NUP35	0.675778	0.682091091	-0.00631306	-0.02184556	0.045564851	0.079475948	3	0	0
NNAT	0.7809737	0.790090044	-0.00911634	-0.00571504	0.045564316	0.079475948	40	0	0
HES4	0.0599127	0.05174559	0.008167146	0.198574139	0.045586412	0.079505823	8	0	0
ROCK2	0.5942379	0.63773452	-0.04349665	-0.09693527	0.045596556	0.079515782	5	0	0
ECHS1	0.042818	0.0409609	0.001857056	0.060377938	0.045613801	0.079538123	3	0	0
MRM1	0.1183585	0.115111312	0.003247168	0.058528607	0.045620561	0.079542178	12	0	0
NME3	0.0471528	0.052089977	-0.00493721	-0.0160369	0.045635278	0.079560104	12	0	0
MAP4K4	0.1677279	0.177766006	-0.01003814	-0.06078237	0.045652575	0.079582523	5	0	0
USP18	0.2493414	0.262958453	-0.01361707	-0.01689201	0.045659163	0.079586273	9	0	0
GPR161	0.5893467	0.578133993	0.011212707	0.02950289	0.04566882	0.079595371	7	0	0
RGPD8	0.7572597	0.793373308	-0.03611359	-0.07954275	0.045679729	0.079606648	2	0	0
ZNF776	0.3921654	0.408953422	-0.01678804	-0.03032948	0.045727292	0.079681795	11	0	0
SLC25A18	0.5410846	0.505209264	0.035875367	0.101226201	0.045733139	0.079684241	6	0	0

ZNRF2	0.1505476	0.162775903	-0.01222834	-0.05146142	0.045771305	0.079734073	8	0	0
TSNARE1	0.2305405	0.248109684	-0.01756919	-0.01576096	0.045775075	0.079734073	8	0	0
H1F0	0.2659128	0.274790637	-0.00887787	0.068500387	0.045769423	0.079734073	9	0	0
BRD8	0.0514017	0.051161051	0.000240656	0.024258917	0.045809194	0.079778009	15	0	0
PBLD	0.0760467	0.069123977	0.006922724	0.071673096	0.045808569	0.079778009	8	0	0
RNPEP	0.2683175	0.281171903	-0.01285444	-0.05347953	0.045840297	0.079816675	12	0	0
TTC21A	0.1201013	0.099277359	0.020823892	0.112197043	0.04585075	0.079827127	8	0	0
PALMD	0.3750348	0.341808451	0.033226315	0.150108196	0.045857765	0.079831591	11	0	0
DNAJC21	0.1447545	0.140840093	0.003914441	0.065048235	0.045872193	0.079841739	9	0	0
FOXL1	0.3391704	0.344773034	-0.00560263	-0.00822183	0.045872498	0.079841739	6	0	0
KEAP1	0.5824611	0.589966202	-0.00750509	0.059244249	0.045888964	0.079862648	8	0	0
ALDH3A2	0.0436857	0.035589737	0.008095934	0.235294946	0.045919956	0.079908833	7	0	0
DRG2	0.2103218	0.216990576	-0.00666876	-0.05585456	0.045944142	0.079943163	13	0	0
H2AFV	0.1074955	0.11433604	-0.00684056	-0.03344059	0.045955212	0.079954669	11	0	0
ABCC12	0.1334457	0.114839426	0.018606227	0.200430616	0.045962653	0.079959859	1	0	0
LIPK	0.5751929	0.622204193	-0.04701126	-0.11147825	0.045987755	0.079980255	1	0	0
SPATA2	0.1205795	0.142370664	-0.02179121	-0.08855473	0.045987095	0.079980255	9	0	0
MYH9	0.1391091	0.146256346	-0.00714723	0.013541627	0.045978876	0.079980255	8	0	0
CCDC174	0.3020241	0.318926799	-0.01690269	-0.05456927	0.046029593	0.080045258	9	0	0
TBC1D5	0.0704737	0.077796313	-0.00732261	0.040665583	0.04607102	0.080109532	6	0	0
PODNL1	0.5390978	0.543836955	-0.00473911	0.011423252	0.046101016	0.080153918	8	0	0
TACO1	0.2294411	0.235379453	-0.00593838	0.060803179	0.046115275	0.080170938	9	0	0
RAPSN	0.6937832	0.705550312	-0.01176716	-0.01519947	0.046156936	0.080235589	12	0	0
SPG21	0.2059316	0.206468136	-0.00053651	0.036329144	0.046168189	0.08024607	5	0	0
DDO	0.5065029	0.52244023	-0.01593732	-0.04474829	0.046218861	0.080319882	6	0	0
SMCO3	0.7474888	0.766797998	-0.01930918	-0.03666254	0.046267893	0.080389512	4	0	0
TLE2	0.2299824	0.230400955	-0.00041854	0.099547544	0.046294288	0.080427582	9	0	0
FCHSD1	0.1880742	0.191271643	-0.00319749	0.142069599	0.046311863	0.080450322	11	0	0
FAM83H	0.0213531	0.023049682	-0.00169662	-0.07554335	0.046353967	0.080515664	6	0	0
TTF1	0.3932821	0.412708691	-0.01942663	-0.0527302	0.046369461	0.080534777	8	0	0
USP53	0.1873872	0.184265035	0.003122167	-0.01285013	0.046381416	0.080542098	11	0	0
ZNF302	0.1992971	0.210943021	-0.01164595	0.023334794	0.046382656	0.080542098	10	0	0
RHOU	0.1332621	0.129306629	0.00395544	0.398740747	0.046399076	0.080562811	11	0	0
GCKR	0.3114398	0.307964436	0.003475375	0.14411488	0.046430541	0.080601049	9	0	0
COL3A1	0.6153299	0.604237711	0.011092204	0.043020537	0.046426884	0.080601049	5	0	0
ZNF655	0.0759211	0.047351154	0.028569913	0.724318081	0.046434581	0.080601049	14	0	0
UGT1A5	0.7977629	0.795270528	0.002492419	0.009657422	0.046443659	0.080609007	4	0	0
HIST1H2BK	0.2102306	0.228359194	-0.01812856	-0.09434773	0.046472478	0.080651221	6	0	0
PRPF38A	0.0445684	0.044245386	0.000323041	0.020144227	0.046478992	0.080654721	11	0	0
AHDC1	0.3389021	0.352985543	-0.01408344	-0.00212957	0.046492776	0.080664416	7	0	0
PRKCA	0.3241869	0.350884431	-0.02669749	-0.02691451	0.046493574	0.080664416	7	0	0
N4BP2L2	0.1620295	0.167679431	-0.00564998	0.061832142	0.046550149	0.080754761	14	0	0
GLIPR2	0.168058	0.178246107	-0.01018808	0.186766754	0.046557865	0.080760336	7	0	0
GRSF1	0.0368449	0.034601197	0.002243665	0.064427639	0.046562911	0.080761278	11	0	0
FBXL5	0.0406434	0.043124649	-0.00248123	-0.05105799	0.046587443	0.080788203	10	0	0
TRAF6	0.0766226	0.085862224	-0.00923964	-0.11694813	0.046587195	0.080788203	14	0	0
CCDC7	0.1983771	0.216472287	-0.01809523	-0.03160272	0.046618978	0.080835073	8	0	0
UBXN4	0.3943871	0.408244622	-0.01385747	-0.01420133	0.046633717	0.080852813	8	0	0
LENG9	0.104408	0.114316057	-0.00990805	-0.05555132	0.04664399	0.080862807	7	0	0
ACTN2	0.3675916	0.376946148	-0.00935454	0.131205149	0.046735763	0.080994027	11	0	0
ATG3	0.1971928	0.22069497	-0.02350215	-0.06932534	0.046737744	0.080994027	12	0	0
ZNF836	0.2609896	0.250955004	0.010034548	0.098541741	0.046733849	0.080994027	9	0	0
PARN	0.0691383	0.072848298	-0.00371003	0.018558152	0.046747701	0.081003456	13	0	0
TRIM7	0.0718279	0.056400387	0.015427498	0.209420037	0.046773857	0.081040948	10	0	0
PHF20L1	0.3304989	0.346354662	-0.01585572	-0.02406468	0.046785264	0.081052883	8	0	0
FAM90A1	0.509887	0.54092081	-0.03103382	-0.07830852	0.046933374	0.081301622	3	0	0
NCBP2	0.1044642	0.121393563	-0.01692933	-0.02900217	0.046971043	0.081351161	8	0	0
CBFA2T2	0.1426804	0.146349742	-0.00366936	-0.01654614	0.04696721	0.081351161	8	0	0
DRD2	0.1251181	0.098281982	0.026836092	0.331389448	0.047002287	0.081397414	6	0	0
SRSF9	0.3147621	0.343345597	-0.0285835	0.000569771	0.047019645	0.081419613	5	0	0
CLIP1	0.1535632	0.155809184	-0.00224599	-0.07261851	0.047055893	0.081474515	6	0	0
SLC44A4	0.6534138	0.640443586	0.012970176	0.029758343	0.047074291	0.081482772	12	0	0
TTBK2	0.0701021	0.071093124	-0.00099103	-8.62E-05	0.04706569	0.081482772	12	0	0

CHRNA5	0.257706	0.253630426	0.004075557	-0.02059101	0.047074181	0.081482772	6	0	0
SNX1	0.0642042	0.073247085	-0.00904286	-0.0996897	0.047127413	0.081566852	10	0	0
CCDC63	0.2763058	0.270809535	0.005496299	0.253095179	0.047144976	0.081589376	12	0	0
PDGFRA	0.1899179	0.143607937	0.046309987	0.439253606	0.047155695	0.081600052	8	0	0
CDRT4	0.8515771	0.879095052	-0.02751794	-0.04535775	0.047161324	0.081601921	1	0	0
RAB40C	0.0584072	0.067571187	-0.00916403	-0.06313289	0.047173967	0.081615923	10	0	0
DNAJC1	0.1743454	0.18246132	-0.00811595	0.03730445	0.047192947	0.081640885	12	0	0
PSKH2	0.6487193	0.682004992	-0.03328565	-0.06033741	0.04720467	0.08165329	5	0	0
SLC26A5	0.0664264	0.068897807	-0.00247136	0.05825068	0.04722811	0.081685957	5	0	0
HKDC1	0.7349384	0.706012959	0.028925402	0.05791428	0.047248901	0.081714037	6	0	0
ADAMTS13	0.7656201	0.755764255	0.009855832	0.014593745	0.047277859	0.081756236	3	0	0
SFTPB	0.6258382	0.653692011	-0.02785376	-0.0697877	0.047302144	0.081790345	5	0	0
ZNF222	0.1406971	0.156445168	-0.01574802	-0.0251454	0.047333386	0.081836477	8	0	0
ZNF891	0.0538955	0.05242187	0.001473624	0.056398322	0.047405289	0.081952893	11	0	0
UBAP2	0.1741813	0.162612094	0.011569162	0.050026625	0.047433217	0.081993271	8	0	0
NDUFC2	0.2720755	0.280250758	-0.00817522	0.014171339	0.047458003	0.08202821	9	0	0
B4GALT1	0.2210712	0.23611424	-0.01504302	-0.05038593	0.04746612	0.082029447	9	0	0
PTGES3L-AA	0.0537986	0.057620362	-0.00382172	-0.00502296	0.047472439	0.082029447	19	0	0
PTGES3L	0.0537986	0.057620362	-0.00382172	-0.00502296	0.047472439	0.082029447	19	0	0
POGZ	0.4146289	0.42438896	-0.00976002	-0.00189311	0.047481746	0.082037626	11	0	0
METTL21A	0.0676174	0.043234408	0.024382982	0.355096471	0.047490815	0.082045392	7	0	0
CRYZL1	0.4329246	0.381958356	0.050966232	0.12884443	0.047509683	0.082070083	3	0	0
NR1D2	0.1128035	0.106762614	0.006040882	0.062309226	0.047535297	0.082091563	6	0	0
RNGTT	0.1757779	0.185471515	-0.00969357	0.012888162	0.047532453	0.082091563	15	0	0
ANGPTL4	0.3583823	0.333583175	0.024799154	0.094833455	0.047535848	0.082091563	5	0	0
IMMT	0.2396916	0.260266103	-0.02057451	-0.04092831	0.047603281	0.0822001	10	0	0
PTDSS1	0.142084	0.156890604	-0.01480662	-0.00363823	0.04761693	0.082215754	9	0	0
SEMA3G	0.4486275	0.44341071	0.005216829	0.107798432	0.047648974	0.082259534	12	0	0
CASP5	0.8652127	0.89344105	-0.02822837	-0.04579682	0.047651458	0.082259534	1	0	0
EML1	0.1691896	0.134285625	0.034904004	0.368576893	0.047681092	0.082294849	7	0	0
RRBP1	0.1407835	0.146676358	-0.00589285	-0.05919606	0.047676647	0.082294849	7	0	0
TBCE	0.239632	0.249110166	-0.00947814	-0.01996283	0.047687171	0.082297421	9	0	0
C12orf50	0.4487282	0.467877349	-0.01914917	-0.04205877	0.047723603	0.082352371	11	0	0
LRIF1	0.0815147	0.0880089	-0.00649424	0.054405964	0.047750367	0.082390628	16	0	0
GNG11	0.3409909	0.31442476	0.026566136	0.147466626	0.047788654	0.082448759	10	0	0
PCSK1	0.2875994	0.275302333	0.012297033	0.169445657	0.047799178	0.082458983	4	0	0
TAGLN3	0.297329	0.316160241	-0.01883121	-0.08484259	0.047842471	0.082509861	8	0	0
PPT2	0.3517949	0.341541836	0.010253097	0.019252368	0.047842277	0.082509861	35	0	0
MIB1	0.0604864	0.071456373	-0.01096994	-0.05005342	0.047834275	0.082509861	8	0	0
PRDM8	0.1368914	0.107162339	0.02972909	0.479341918	0.047853949	0.082513789	7	0	0
SPTY2D1	0.1935305	0.207927464	-0.01439698	0.007893891	0.047849921	0.082513789	10	0	0
RFTN1	0.1640445	0.16417079	-0.00012633	0.243393473	0.047891262	0.082570189	11	0	0
SLITRK6	0.4557094	0.466737467	-0.01102804	0.025008722	0.047912361	0.082598627	5	0	0
DMXL1	0.7214263	0.690629399	0.03079688	0.062060935	0.047995609	0.08273419	1	0	0
HPN	0.3953231	0.362856568	0.032466489	0.342663988	0.048002048	0.082737338	12	0	0
ZNF793	0.0727582	0.050008166	0.022750056	0.487294602	0.048019154	0.082752064	15	0	0
APOL2	0.4587948	0.477561437	-0.01876666	-0.11725494	0.04801982	0.082752064	4	0	0
ZNF417	0.0302681	0.030103737	0.000164381	0.020590834	0.048112812	0.082904352	4	0	0
SMAGP	0.0394258	0.037444744	0.001981015	0.030424885	0.048127265	0.082921289	7	0	0
PDGFRL	0.1835682	0.203077978	-0.01950982	-0.1251938	0.048132162	0.082921762	9	0	0
MPP4	0.7226282	0.707791438	0.014836727	0.038078163	0.04820658	0.083039425	7	0	0
CD200R1	0.5546407	0.558872357	-0.0042317	0.010994952	0.048211567	0.083039425	6	0	0
ZNF117	0.055282	0.049208087	0.006073899	0.117210376	0.048218979	0.083039425	5	0	0
ERV3-1	0.055282	0.049208087	0.006073899	0.117210376	0.048218979	0.083039425	5	0	0
ESRP1	0.0682433	0.07094175	-0.00269845	-0.03939514	0.048239139	0.083066166	8	0	0
HSD17B12	0.0659195	0.051384148	0.014535376	0.261070293	0.048245009	0.083068299	2	0	0
ELP3	0.706747	0.728305005	-0.02155796	-0.04275289	0.048258464	0.083075516	1	0	0
RGCC	0.0830245	0.063879901	0.019144594	0.325095112	0.048281653	0.083099484	8	0	0
C19orf25	0.0453025	0.043825297	0.001477229	0.058310705	0.04827716	0.083099484	9	0	0
SETD9	0.2196896	0.251911253	-0.03222167	-0.0619272	0.048303201	0.083120618	8	0	0
ZNF510	0.1182985	0.11736723	0.000931242	0.355393967	0.048299146	0.083120618	10	0	0
TM4SF5	0.531215	0.553675209	-0.02246017	-0.047852	0.048314999	0.083132079	9	0	0
C3	0.6975992	0.731385243	-0.03378608	-0.06836327	0.048319131	0.083132079	5	0	0

SOCS2	0.2263516	0.238324177	-0.01197257	0.015316636	0.04832583	0.08313563	12	0	0
CENPM	0.3073065	0.323353724	-0.01604719	-0.04126936	0.048331826	0.083137971	12	0	0
LTB4R	0.3348246	0.330774994	0.004049641	0.099846081	0.048352273	0.083165168	16	0	0
PRRC2C	0.1611724	0.179862367	-0.01869	-0.02387349	0.048370087	0.08318783	9	0	0
ARHGAP29	0.6442488	0.609759745	0.034489084	0.078074679	0.048383786	0.083203411	2	0	0
CRTAP	0.2012327	0.206691262	-0.00545859	-0.00175007	0.048436308	0.08326978	9	0	0
EML3	0.2364967	0.232125377	0.00437131	0.066453503	0.048434066	0.08326978	9	0	0
ATP6AP1L	0.1716528	0.183782184	-0.01212942	-0.02025713	0.04850055	0.083372232	16	0	0
ENSA	0.1416318	0.161625694	-0.01999389	0.000378072	0.048541617	0.083418841	17	0	0
KLHL5	0.1099487	0.1262131	-0.01626437	-0.03418796	0.048537964	0.083418841	6	0	0
NPTXR	0.1520028	0.166812322	-0.01480949	-0.03726919	0.048539478	0.083418841	4	0	0
DACH1	0.1277104	0.120143625	0.007566815	0.210185019	0.04855916	0.083440993	12	0	0
TMCO2	0.7456681	0.751330832	-0.00566273	-0.00330917	0.048573968	0.083450449	2	0	0
HOMER1	0.0544407	0.06595494	-0.01151427	-0.0895661	0.048572812	0.083450449	8	0	0
HERC2	0.0921814	0.082843224	0.009338148	0.138263692	0.048654163	0.08358022	1	0	0
PLEKHB2	0.1595608	0.15884586	0.000714934	0.064425016	0.048669876	0.083599204	7	0	0
LGMN	0.311333	0.316618784	-0.00528577	0.037641343	0.048701899	0.0836462	13	0	0
KAT5	0.1374648	0.149934562	-0.01246977	0.00078081	0.048737435	0.083699219	14	0	0
ADRA2A	0.108788	0.093218249	0.015569708	0.311976241	0.048809384	0.083814754	8	0	0
LGALS1	0.1380594	0.123746632	0.01431273	0.112997716	0.048824322	0.08383238	10	0	0
MPP5	0.1137073	0.108498337	0.005208928	0.03959922	0.048862739	0.083890312	7	0	0
GRM6	0.5226914	0.527146843	-0.00445547	-0.00012905	0.048892113	0.083932709	7	0	0
DYNLT1	0.1047845	0.095487651	0.00929682	0.04689307	0.048899215	0.083936868	10	0	0
ERCC6	0.1500687	0.167431745	-0.01736301	-0.03822022	0.048974176	0.084057495	12	0	0
SLFN11	0.0878645	0.06903247	0.01883208	0.271041104	0.049008782	0.084108842	12	0	0
HPDL	0.201486	0.20183821	-0.00035217	0.14113884	0.049027125	0.084132272	11	0	0
RPTN	0.6953804	0.734865374	-0.03948496	-0.08224622	0.049047849	0.084159784	5	0	0
VRTN	0.035137	0.033371931	0.001765053	0.059341152	0.049065194	0.084181492	7	0	0
ZNF668	0.0482249	0.051459608	-0.00323471	-0.04281432	0.04913942	0.084300779	13	0	0
ANKRD34B	0.2947278	0.294771046	-4.32E-05	0.165245064	0.049159175	0.084310476	10	0	0
IL5	0.266859	0.290340446	-0.02348141	-0.04278363	0.049153003	0.084310476	13	0	0
ENDOV	0.1572751	0.169128398	-0.01185334	0.0430864	0.049158153	0.084310476	13	0	0
PDRG1	0.1051244	0.123320209	-0.01819584	-0.06460725	0.049179287	0.084336905	6	0	0
ZNF609	0.0524951	0.052599678	-0.0001046	-0.00457941	0.049210707	0.08438272	5	0	0
SMARCC2	0.0721611	0.063212097	0.008948968	0.169323685	0.04922115	0.084384492	4	0	0
TGIF2	0.1529553	0.14737867	0.005576624	0.151628888	0.04921744	0.084384492	9	0	0
FAM200A	0.0714941	0.040486286	0.031007814	0.746983642	0.049241856	0.084411921	17	0	0
ZNF248	0.243681	0.224202909	0.019478046	0.064868806	0.049247296	0.084413178	10	0	0
RBMXL1	0.1808383	0.184045259	-0.00320691	0.071760126	0.049262747	0.084431594	9	0	0
LOX	0.129177	0.131871983	-0.00269502	0.099428556	0.049305412	0.084496644	16	0	0
TPBG	0.2459879	0.239866988	0.006120912	0.053719456	0.049335631	0.084532277	12	0	0
SERPINA6	0.6691623	0.709093748	-0.03993148	-0.08269438	0.049340933	0.084533287	5	0	0
NRP2	0.2722767	0.274768458	-0.0024918	0.139438639	0.049356856	0.084544415	10	0	0
TAS2R31	0.9149684	0.922113205	-0.00714479	-0.0110881	0.049352224	0.084544415	2	0	0
TMEM56-RV	0.0665287	0.068942604	-0.00241392	-0.02215638	0.049372069	0.084562397	12	0	0
SGSM2	0.0323832	0.033789085	-0.0014059	-0.01611347	0.049412702	0.084623911	11	0	0
TUBGCP6	0.0862657	0.093612283	-0.00734658	-0.06738147	0.049426425	0.084639332	10	0	0
JUNB	0.0358531	0.036125643	-0.00027251	0.016650859	0.049451643	0.084674431	7	0	0
HYI	0.0704293	0.077753062	-0.00732381	0.006860678	0.049493097	0.084737322	16	0	0
PFDN5	0.1503426	0.154324253	-0.0039817	-0.01550664	0.049500728	0.084742297	8	0	0
CRYM	0.7679776	0.808036662	-0.04005909	-0.09554811	0.049537761	0.084797603	3	0	0
MAP2K1	0.1865925	0.182721171	0.003871364	0.052797301	0.049625333	0.0849394	3	0	0
ZNF551	0.1517155	0.165231669	-0.01351614	0.038756514	0.049632386	0.084943366	9	0	0
PLK2	0.1126478	0.116944175	-0.0042964	-0.0119818	0.049645449	0.084957615	11	0	0
SKP1	0.1103293	0.121392371	-0.01106306	-0.01778556	0.04965691	0.084961017	10	0	0
SH2B1	0.0929612	0.097709545	-0.00474837	0.003927263	0.04965546	0.084961017	7	0	0
CLDND1	0.1542901	0.156629509	-0.00233939	0.001850264	0.049664186	0.084965361	14	0	0
PACSIN2	0.0730526	0.084212506	-0.01115986	-0.04114155	0.049685663	0.084993996	11	0	0
AQP3	0.229838	0.23775714	-0.00791917	0.286499391	0.049694829	0.085001568	8	0	0
C8orf74	0.5806071	0.594167673	-0.01356057	-0.02882036	0.049746429	0.085081715	8	0	0
SIDT2	0.1656137	0.145604734	0.020008932	0.079249133	0.049765136	0.085105595	11	0	0
ORC1	0.0472638	0.046810292	0.000453522	0.027198722	0.049854399	0.085226044	10	0	0
TMEM61	0.4595197	0.439163064	0.020356613	0.268194089	0.049850259	0.085226044	8	0	0

WNT9A	0.1546204	0.153322357	0.001298044	0.223012192	0.049851528	0.085226044	7	0	0
KCTD20	0.0430368	0.038370583	0.00466626	0.083239865	0.049859326	0.085226044	15	0	0
GGT1	0.6274639	0.618708389	0.008755524	0.033376397	0.049856248	0.085226044	10	0	0
BMPER	0.1173542	0.100443131	0.016911028	0.421965409	0.049960135	0.085382085	9	0	0
ARG2	0.0469183	0.043256659	0.003661645	0.053468877	0.049968181	0.0853877	9	0	0
NUDT22	0.4148656	0.425171573	-0.01030594	-0.00612928	0.050013008	0.085456159	11	0	0
CFLAR	0.1490729	0.153327504	-0.00425465	0.002343828	0.050057402	0.085523868	13	0	0
TFPT	0.2344781	0.230520923	0.003957136	0.04833186	0.050097646	0.085584472	15	0	0
PKNOX2	0.2984078	0.302709328	-0.00430158	0.127876372	0.050147199	0.085660968	11	0	0
CLCN2	0.0432705	0.042132966	0.001137547	0.005930033	0.050177556	0.085704661	12	0	0
GRB7	0.3677959	0.395168975	-0.02737307	-0.1131785	0.050193338	0.085723454	11	0	0
HEBP2	0.2665068	0.273328567	-0.0068218	0.045617116	0.050237905	0.085783231	8	0	0
DDX39A	0.0703467	0.081875482	-0.01152881	-0.02803487	0.050233804	0.085783231	13	0	0
RPS15A	0.2121643	0.235567113	-0.02340282	0.003684879	0.050249793	0.085795363	9	0	0
NOL8	0.1381481	0.139308829	-0.00116075	0.046291956	0.050294968	0.085864319	9	0	0
SLC22A9	0.6387421	0.679774947	-0.04103285	-0.1017037	0.05030241	0.085868852	5	0	0
CASP2	0.5151934	0.503723189	0.011470239	0.104603392	0.050310853	0.08587509	13	0	0
HMCES	0.275815	0.282148801	-0.00633375	0.051940955	0.05032365	0.085880588	9	0	0
EZH1	0.2762304	0.263556806	0.01267362	0.097095616	0.050319968	0.085880588	10	0	0
BCL7C	0.4869308	0.492854779	-0.00592397	0.001469042	0.050335759	0.085893081	12	0	0
THAP3	0.3243796	0.322012613	0.002366961	0.020457627	0.050352154	0.085912884	9	0	0
C1orf162	0.6003851	0.600834857	-0.00044971	0.023217889	0.050367331	0.085930605	8	0	0
NKTR	0.0641477	0.05748327	0.006664476	0.09502357	0.050412146	0.085990705	10	0	0
SNAP23	0.0856442	0.089256315	-0.00361212	0.013312254	0.050412101	0.085990705	11	0	0
ZMYM5	0.0328747	0.033826561	-0.00095181	-0.02009866	0.050439133	0.086028556	4	0	0
KBTBD4	0.1186887	0.126386676	-0.00769798	-0.0155359	0.050475218	0.086077755	17	0	0
TNFRSF6B	0.4406581	0.461649711	-0.02099158	-0.05399227	0.050477577	0.086077755	4	0	0
CDC37	0.083043	0.061248851	0.021794129	0.461442531	0.050508233	0.086121844	7	0	0
ZNF2	0.1156935	0.11853366	-0.00284018	-0.05895643	0.050527921	0.086147224	12	0	0
SULT1E1	0.3378349	0.380680583	-0.04284566	-0.16758676	0.050556011	0.086177456	1	0	0
ARID5B	0.0588735	0.065031825	-0.00615831	0.007513839	0.050560067	0.086177456	16	0	0
CLEC4D	0.6639107	0.701245326	-0.03733463	-0.07443208	0.050556106	0.086177456	2	0	0
ADIRF	0.3811826	0.370525096	0.010657522	0.130010448	0.050616419	0.086265307	10	0	0
APOL6	0.241217	0.228057412	0.01315959	0.138787073	0.050637395	0.086292857	7	0	0
TMEM17	0.6776225	0.702501478	-0.02487893	-0.05127616	0.05064376	0.08629399	1	0	0
PIK3CA	0.06206	0.064405788	-0.00234583	-0.0010084	0.050647682	0.08629399	8	0	0
FNDC4	0.1643885	0.160692318	0.003696146	0.23458586	0.050679612	0.086339404	11	0	0
CARD11	0.4525023	0.437763191	0.014739121	0.488720325	0.050688778	0.086339404	6	0	0
MYO1E	0.1228481	0.128122647	-0.00527454	0.016653952	0.050684693	0.086339404	11	0	0
EOGT	0.1908066	0.19498691	-0.00418026	-0.04539645	0.050703796	0.086353909	12	0	0
PPP1R2	0.2115236	0.226939937	-0.01541629	-0.04175462	0.050708056	0.086353909	8	0	0
05-Sep	0.1879677	0.181598226	0.006369491	0.235787208	0.050711738	0.086353909	13	0	0
SLC9A3R1	0.1218298	0.126062604	-0.00423282	0.032252483	0.05072724	0.086372108	8	0	0
KLHL21	0.1046287	0.091187181	0.013441505	0.198806285	0.050750133	0.086402885	3	0	0
ICA1L	0.2684292	0.274838705	-0.00640949	-0.02471071	0.050759958	0.086411409	7	0	0
OSTC	0.1030318	0.106378683	-0.00334692	0.055911031	0.050766134	0.086413722	12	0	0
ZNF341	0.2823949	0.285916051	-0.00352116	0.119652981	0.050783087	0.086426175	8	0	0
BACE2	0.2795155	0.256196697	0.023318786	0.110440599	0.050778912	0.086426175	7	0	0
SLC6A9	0.4027266	0.391383238	0.011343402	0.023651217	0.050792219	0.086433515	11	0	0
BCL7B	0.267433	0.277090638	-0.00965767	0.066744504	0.050798446	0.08643591	10	0	0
CDK2AP1	0.4999121	0.501571451	-0.0016594	0.027446062	0.050811818	0.086450461	13	0	0
ZNF177	0.2462137	0.244557956	0.001655719	0.202063766	0.050819877	0.086455971	11	0	0
NET1	0.065467	0.064598769	0.000868201	-0.00109582	0.050830707	0.086466194	6	0	0
HSPB7	0.6127901	0.640504459	-0.02771438	-0.06629786	0.050844235	0.086472805	3	0	0
API5	0.0885716	0.078266352	0.010305274	0.118234692	0.05084384	0.086472805	9	0	0
FBF1	0.0562423	0.064410354	-0.00816808	-0.06256659	0.05086593	0.086501499	10	0	0
GALR3	0.5294724	0.532426139	-0.00295377	0.014682973	0.050883089	0.086522477	7	0	0
RRP15	0.1455632	0.16656305	-0.02099981	-0.0323336	0.050908538	0.086557545	12	0	0
VPS37B	0.4852411	0.467948107	0.017292952	0.089214912	0.05091358	0.086557912	14	0	0
NKD1	0.1323703	0.135048301	-0.002678	-0.0097595	0.050940488	0.086595449	9	0	0
CTBP2	0.046244	0.04086339	0.005380583	0.119717685	0.050948424	0.086600732	8	0	0
CDON	0.2974011	0.330208689	-0.03280763	-0.07447436	0.050961268	0.086614357	8	0	0
G3BP2	0.0424335	0.040354532	0.002078983	0.053547482	0.050988851	0.086653025	15	0	0

ATP9B	0.4718777	0.478910446	-0.0070327	0.001383221	0.051017651	0.086693755	3	0	0
AIPL1	0.5544421	0.576665151	-0.02222304	-0.07210811	0.051036925	0.086710078	8	0	0
PCIF1	0.2440328	0.268914992	-0.0248822	-0.03235623	0.051032767	0.086710078	8	0	0
UBE2H	0.2826022	0.284735782	-0.00213361	0.036904859	0.051050206	0.086724426	7	0	0
PPIH	0.1633321	0.170629168	-0.00729706	-0.014314	0.051061302	0.086729073	11	0	0
MGAT2	0.1239313	0.148606861	-0.02467556	-0.06457167	0.051062612	0.086729073	7	0	0
TBR1	0.1744024	0.142894539	0.03150785	0.316886131	0.051118214	0.086815291	14	0	0
TMEM167A	0.0749426	0.08525778	-0.01031522	-0.0541522	0.051155245	0.086869955	12	0	0
TRABD2B	0.110617	0.100492898	0.010124088	0.292628163	0.051170559	0.086880154	11	0	0
PUS7	0.1862965	0.199152172	-0.01285569	-0.02554755	0.051170938	0.086880154	3	0	0
FGFR1OP	0.0301697	0.033457341	-0.00328768	-0.06743975	0.051190586	0.086897062	13	0	0
ARFIP2	0.225052	0.242814512	-0.01776255	-0.11621913	0.051187254	0.086897062	8	0	0
GMNN	0.2692808	0.287952265	-0.01867147	-0.02956889	0.051209782	0.08692142	11	0	0
SERPINE1	0.1510197	0.144224906	0.006794776	0.163162131	0.051221551	0.086924944	9	0	0
JKAMP	0.0477562	0.050544324	-0.00278809	-0.06033183	0.05121949	0.086924944	11	0	0
EPHB3	0.1194046	0.116945474	0.002459115	0.112733054	0.05128926	0.087015149	12	0	0
CHURC1	0.1664578	0.166112387	0.000345396	0.006110141	0.051288683	0.087015149	15	0	0
OXA1L	0.260943	0.266596961	-0.00565396	0.015417212	0.05130002	0.087025174	15	0	0
RPS6	0.0313694	0.030458739	0.000910669	0.036011732	0.05130593	0.087026969	6	0	0
PRMT6	0.4854324	0.501643966	-0.01621153	0.031540435	0.051325454	0.087051853	6	0	0
HEXB	0.1386515	0.125371497	0.01327998	-0.00302667	0.051348991	0.087083538	8	0	0
ZNF354B	0.1136684	0.116289707	-0.0026213	0.047721415	0.051392435	0.087146826	6	0	0
TUB	0.5140034	0.512435143	0.001568218	0.082805065	0.051396026	0.087146826	2	0	0
GTF2B	0.2478729	0.276660735	-0.02878785	-0.04373666	0.051422567	0.087175345	12	0	0
LIN52	0.0452304	0.042700915	0.002529519	0.078917566	0.051417954	0.087175345	8	0	0
CCDC122	0.1360365	0.132376691	0.003659841	0.16685941	0.051438516	0.087185902	15	0	0
SS18	0.1388531	0.142828005	-0.00397491	0.139404478	0.051436741	0.087185902	9	0	0
PTAR1	0.3398238	0.339564308	0.000259482	-0.00058957	0.051466756	0.087224824	3	0	0
BAG3	0.2118468	0.212613934	-0.0007671	0.029579129	0.051471205	0.087224824	5	0	0
PRDX6	0.1105585	0.133106264	-0.02254776	-0.04055624	0.051498254	0.087262417	11	0	0
CNPY1	0.1684079	0.157315379	0.011092521	0.232827496	0.051531691	0.08730258	8	0	0
DACT3	0.0420969	0.040136569	0.0019603	0.05293818	0.051551689	0.087328211	13	0	0
GEMIN6	0.1151815	0.1194789	-0.00429737	0.019614745	0.051558699	0.087331838	13	0	0
RRAGA	0.1512385	0.154063358	-0.00282488	-0.01080269	0.051573494	0.087348649	8	0	0
STX1A	0.143832	0.146429555	-0.00259753	0.022094337	0.051584038	0.087358258	7	0	0
GUCY1A2	0.0832161	0.063295132	0.019920995	0.327790185	0.051652366	0.087465713	10	0	0
NDUFB7	0.1701727	0.170732366	-0.00055967	0.023141662	0.05172462	0.087579796	8	0	0
CDT1	0.2042013	0.230182125	-0.02598084	-0.12461907	0.051857596	0.087796662	6	0	0
TTC39A	0.3918472	0.390526357	0.001320856	-0.01353805	0.051881349	0.087828587	9	0	0
DTYMK	0.1743774	0.196349116	-0.02197172	-0.05918945	0.051912756	0.087873462	8	0	0
OIP5	0.0297996	0.029969566	-0.00016999	0.009517603	0.051920145	0.087877675	15	0	0
NDUFS2	0.7181132	0.727667104	-0.00955389	-0.01296194	0.051962287	0.087932407	6	0	0
KCNV2	0.8966993	0.907485769	-0.01078646	-0.01762753	0.051961702	0.087932407	4	0	0
CD37	0.477017	0.511189968	-0.03417294	-0.08249875	0.051974294	0.087944429	6	0	0
TRAF7	0.0680878	0.083166174	-0.01507835	-0.06757911	0.052088573	0.088129482	13	0	0
NUDT15	0.1570017	0.159698398	-0.00269673	0.019950756	0.052094521	0.088131233	11	0	0
NAA16	0.1261885	0.132733364	-0.00654487	0.022755915	0.052109341	0.088147991	9	0	0
SLC37A2	0.3250899	0.340740461	-0.0156506	0.107435172	0.052126877	0.088169339	10	0	0
TMEM252	0.5955825	0.585663845	0.009918656	0.035795643	0.052135248	0.088175183	2	0	0
GADD45GIP	0.0698175	0.064207708	0.005609753	0.09280541	0.052159958	0.088208656	9	0	0
HCRT	0.7226369	0.729937248	-0.00730039	-0.01288329	0.0522291	0.088317257	7	0	0
GLB1L3	0.2796287	0.233841034	0.04578763	0.248263465	0.052256609	0.088355443	1	0	0
SYCP3	0.8938747	0.902532908	-0.00865822	-0.01436245	0.052299354	0.088411047	8	0	0
FAM214A	0.7054827	0.730019395	-0.02453671	-0.05823538	0.052296736	0.088411047	2	0	0
TSEN2	0.0602434	0.066258288	-0.00601488	-0.09498771	0.052311965	0.088424032	11	0	0
HBZ	0.5553849	0.581291086	-0.02590616	-0.06451317	0.052319633	0.08842866	4	0	0
FDPS	0.2343885	0.228906385	0.005482097	0.044458002	0.052350594	0.088472652	13	0	0
EYA3	0.1610854	0.163043398	-0.001958	0.049382963	0.052406642	0.08855248	12	0	0
NRARP	0.1738057	0.192179188	-0.01837348	-0.06861884	0.052407704	0.08855248	10	0	0
TMEM18	0.1409539	0.144992758	-0.00403888	-0.03880442	0.052431083	0.088583639	8	0	0
ZNF516	0.0762926	0.071717883	0.004574669	0.118899243	0.052444263	0.08859756	11	0	0
MCM4	0.1169409	0.141492206	-0.02455131	-0.10793243	0.052454293	0.088606159	7	0	0
RIOK1	0.1516149	0.159521551	-0.00790666	-0.00662251	0.052464974	0.088615856	13	0	0

EPN3	0.3882901	0.398224103	-0.00993404	0.007333442	0.052508106	0.088680357	7	0	0
SLC24A4	0.3845058	0.3736236	0.010882208	0.141089131	0.052584771	0.088801474	7	0	0
ATP1A2	0.5814719	0.570034962	0.0114369	0.043098613	0.052611301	0.08880447	7	0	0
FAM135A	0.3511712	0.369585649	-0.01841446	-0.00859743	0.05260508	0.08880447	7	0	0
SP6	0.5468457	0.528907779	0.017937901	0.037159956	0.052604435	0.08880447	4	0	0
SDHAF1	0.3234372	0.318011855	0.005425354	0.025323795	0.052591924	0.08880447	9	0	0
FOXA3	0.0386915	0.036018123	0.002673346	0.083403929	0.052610938	0.08880447	7	0	0
ANKRD44	0.2198298	0.203951825	0.015878021	0.036843668	0.052620131	0.088811016	7	0	0
ZNF441	0.2736862	0.276684525	-0.00299831	0.20627177	0.052631853	0.088822442	11	0	0
BRPF1	0.1946888	0.212654802	-0.01796601	-0.02657834	0.052642096	0.08883137	8	0	0
CYP4A11	0.6446006	0.677709589	-0.03310898	-0.06654404	0.052669671	0.088869541	2	0	0
ZNF37A	0.0504476	0.044445757	0.006001848	0.134507861	0.052678066	0.088875345	3	0	0
TMUB1	0.1587208	0.15748671	0.001234041	0.01841829	0.052703691	0.088901852	13	0	0
PHF12	0.0669953	0.075216938	-0.00822165	-0.03986373	0.052702707	0.088901852	7	0	0
TMEM81	0.835617	0.847937513	-0.01232056	-0.02156143	0.052763286	0.088994009	6	0	0
SERP1	0.0540153	0.062303803	-0.00828847	-0.05121577	0.052846743	0.089124056	10	0	0
ETFA	0.2395233	0.255324528	-0.01580126	-0.07415856	0.052850327	0.089124056	8	0	0
RASD1	0.1389244	0.140340272	-0.00141591	0.059874211	0.052872962	0.089153844	8	0	0
RBM25	0.1720295	0.174833078	-0.00280356	-0.02332366	0.052885315	0.089166292	8	0	0
TOE1	0.3192579	0.343171088	-0.02391317	-0.0452187	0.052910331	0.089200085	10	0	0
CERCAM	0.6761454	0.669956828	0.006188551	0.011424703	0.052976854	0.089297355	2	0	0
PRKCB	0.2879587	0.266979076	0.020979625	0.484932022	0.052977986	0.089297355	7	0	0
HIST1H3G	0.117522	0.088603602	0.02891843	0.512769209	0.053019959	0.089359705	11	0	0
TRA2B	0.3936535	0.387669046	0.005984448	0.070008387	0.053026868	0.089362952	8	0	0
PRR23B	0.6674321	0.660462326	0.00696977	0.031117461	0.05304861	0.089391194	8	0	0
BTBD10	0.076323	0.088722646	-0.01239961	-0.06410297	0.053079033	0.089434056	9	0	0
KCNB2	0.0797806	0.088966293	-0.0091857	-0.02966213	0.053133195	0.089516906	9	0	0
CDK18	0.2014091	0.212896429	-0.01148731	-0.04620567	0.053139811	0.089519645	11	0	0
RUNDC1	0.0727408	0.076116856	-0.00337604	0.000333765	0.053149679	0.089527859	19	0	0
DNAJB12	0.4508556	0.472490516	-0.02163495	-0.04270372	0.053162783	0.089541522	10	0	0
ARL17A	0.1392691	0.079431678	0.05983746	0.739058065	0.053169581	0.089544564	1	0	0
SNRPA	0.1800733	0.19255529	-0.01248203	-0.01010961	0.053191891	0.089573725	11	0	0
LIMCH1	0.667942	0.672616186	-0.00467418	-0.0132556	0.05320572	0.089587355	10	0	0
MED26	0.1224973	0.102976957	0.019520296	0.141013924	0.053209974	0.089587355	5	0	0
HNF1A	0.7045203	0.686368685	0.01815159	0.043274351	0.053215126	0.08958762	10	0	0
ZBTB48	0.2388144	0.237309142	0.001505255	0.072329644	0.053240487	0.089619431	8	0	0
SNRNP40	0.0374146	0.037328271	8.63E-05	0.02658271	0.053249033	0.089619431	10	0	0
ZCCHC17	0.0374146	0.037328271	8.63E-05	0.02658271	0.053249033	0.089619431	10	0	0
ARHGAP30	0.60129	0.630668081	-0.02937812	-0.07090877	0.053254009	0.089619431	10	0	0
PTPRCAP	0.6116631	0.644253856	-0.03259073	-0.07414025	0.053274212	0.08964502	13	0	0
GSTM4	0.1185066	0.127956415	-0.00944981	-0.00219598	0.053284546	0.089653998	12	0	0
TTC29	0.0933949	0.106693157	-0.01329822	-0.01321828	0.053320399	0.089698083	10	0	0
N6AMT1	0.3030911	0.304360879	-0.00126976	-0.01488134	0.05332075	0.089698083	7	0	0
SLC25A51	0.3682711	0.388232577	-0.01996146	-0.00653337	0.053330326	0.089705779	9	0	0
ABCB6	0.2214266	0.214831767	0.006594837	0.264090731	0.053348499	0.089727934	11	0	0
PDE3B	0.1912243	0.19343811	-0.00221378	0.067913107	0.053353772	0.089728388	8	0	0
ST6GALNAC	0.1541092	0.160408991	-0.00629976	0.047064435	0.053373127	0.089752522	11	0	0
CXCL3	0.2394042	0.229175546	0.010228687	0.089975536	0.053405079	0.089797835	8	0	0
PRR4	0.0469749	0.050962235	-0.00398731	-0.08155177	0.053421393	0.089808426	3	0	0
TAS2R14	0.0469749	0.050962235	-0.00398731	-0.08155177	0.053421393	0.089808426	3	0	0
TAF12	0.089773	0.08833549	0.001437496	0.052034087	0.053446777	0.08984268	14	0	0
HERC6	0.2491097	0.241545715	0.007564031	0.149348191	0.053464141	0.089863446	9	0	0
SSH2	0.0479475	0.043374672	0.004572847	0.094863487	0.05352134	0.089951157	10	0	0
MYL3	0.0592402	0.046401636	0.012838586	0.34405	0.053538142	0.089970966	12	0	0
ITSN1	0.0993657	0.10173943	-0.00237368	0.036442442	0.053572285	0.090019909	16	0	0
POLR3H	0.2269032	0.228229926	-0.00132668	0.065671605	0.053589591	0.090040552	5	0	0
PCP2	0.659332	0.656167603	0.003164433	-0.00418313	0.053619056	0.090081621	7	0	0
CDC20B	0.0569874	0.059722772	-0.00273541	-0.0473927	0.053643777	0.090106273	6	0	0
TGFB3	0.3096951	0.303796454	0.005898687	0.035144057	0.0536411	0.090106273	8	0	0
TBX3	0.144492	0.137698918	0.006793095	0.17605147	0.053675873	0.090151742	10	0	0
DSTYK	0.1501286	0.168276944	-0.01814838	-0.01630532	0.053691891	0.09016271	9	0	0
SETD2	0.0443387	0.042782452	0.001556272	0.043104426	0.053692457	0.09016271	8	0	0
JAG2	0.1689608	0.181574592	-0.01261375	-0.06581808	0.053718518	0.09019219	6	0	0

SLC6A4	0.0826351	0.063782768	0.018852294	0.357983361	0.053720069	0.09019219	10	0	0
CHCHD6	0.2620039	0.28156959	-0.01956571	-0.00377994	0.053729267	0.090199188	10	0	0
NDUFB6	0.614471	0.629566669	-0.01509565	-0.024663	0.05375262	0.090221504	5	0	0
FAM98B	0.215017	0.231885326	-0.01686837	-0.03120505	0.053748395	0.090221504	10	0	0
PDK2	0.1333189	0.132570845	0.00074808	0.140607114	0.053760537	0.090226348	9	0	0
NIFK	0.2552109	0.268948008	-0.01373709	-0.01408727	0.053777487	0.090246351	4	0	0
RSAD2	0.1170062	0.10538726	0.01161889	0.100115347	0.053823287	0.09031476	13	0	0
RAB18	0.2402169	0.246560394	-0.00634351	-0.04995863	0.053939063	0.090500564	13	0	0
MMACHC	0.0714985	0.077179026	-0.00568054	-0.01630292	0.053985654	0.090553324	12	0	0
RAB6A	0.0627375	0.069243357	-0.00650586	-0.04700118	0.053977447	0.090553324	7	0	0
SGTA	0.2399074	0.25483987	-0.0149325	-0.0030636	0.053984811	0.090553324	8	0	0
SPRR2D	0.6229401	0.596689538	0.026250586	0.10111755	0.054020774	0.090603759	4	0	0
SERTAD1	0.138836	0.13689672	0.001939247	0.081901656	0.054075186	0.09068654	9	0	0
GPX1	0.2517686	0.257641096	-0.00587251	-0.0456807	0.054097146	0.090714884	11	0	0
GNMT	0.2733135	0.282431975	-0.00911852	0.129408341	0.054130709	0.090754197	17	0	0
TMCC3	0.3900242	0.402276471	-0.01225227	-0.04442471	0.054126251	0.090754197	5	0	0
LYRM7	0.0495935	0.047726087	0.001867433	0.042948957	0.054141415	0.090759094	11	0	0
KDM2A	0.0886493	0.074781316	0.013868027	0.276734638	0.054143751	0.090759094	7	0	0
PDLIM5	0.2014448	0.2036555	-0.00221066	0.038995726	0.054158922	0.090776043	11	0	0
ZFYVE26	0.1125712	0.121060128	-0.00848896	0.014161255	0.054180074	0.09080301	12	0	0
LBX2	0.2663177	0.280756429	-0.01443876	0.046953827	0.054249899	0.090911538	17	0	0
KIF20A	0.0743849	0.074669299	-0.00028435	0.002002011	0.05428617	0.090963822	16	0	0
PTCH1	0.188423	0.166713136	0.021709869	0.145982632	0.05430888	0.090993375	4	0	0
THAP1	0.1813717	0.203849777	-0.02247812	-0.06624629	0.054322943	0.091008435	13	0	0
EI24	0.1464099	0.150710587	-0.00430065	-0.00432222	0.054339787	0.091019651	7	0	0
RPL23	0.179288	0.184186259	-0.00489825	0.05430142	0.054338933	0.091019651	12	0	0
SYT7	0.1404615	0.140257256	0.000204208	0.098482898	0.054392125	0.09109881	5	0	0
MRPL13	0.1196752	0.140309011	-0.02063379	-0.05594533	0.054455392	0.091187742	14	0	0
MAP3K3	0.0378891	0.036944673	0.000944432	0.026788831	0.054453394	0.091187742	10	0	0
WDPCP	0.8022805	0.776215819	0.02606467	0.047052599	0.054466665	0.091198104	1	0	0
DYRK3	0.123245	0.104571474	0.018673544	0.261204175	0.054484246	0.091219027	11	0	0
SREBF1	0.1978102	0.19505041	0.002759801	-0.02848933	0.054539047	0.091302254	7	0	0
NOG	0.1306477	0.120561121	0.010086622	0.210233884	0.054546423	0.09130608	10	0	0
EPHB4	0.0698411	0.058848227	0.010992922	0.229127725	0.054564867	0.09132843	10	0	0
TYW1B	0.0836516	0.077541124	0.00611048	0.082750441	0.054578468	0.091334148	6	0	0
05-Mar	0.0326004	0.032227925	0.000372497	0.020040609	0.054578133	0.091334148	14	0	0
IRS1	0.0811647	0.083683751	-0.00251909	-0.00025437	0.054590771	0.091346215	5	0	0
SPICE1	0.1576754	0.166053161	-0.00837773	-0.01719635	0.054645593	0.091423342	10	0	0
MYO5B	0.2277685	0.23420017	-0.00643171	-0.04243424	0.054647059	0.091423342	4	0	0
WDR74	0.0586207	0.048519859	0.0101008	0.243932695	0.0546832	0.091475273	5	0	0
DHPS	0.3619877	0.383842342	-0.02185462	-0.03794414	0.054745303	0.09157062	10	0	0
DNMT3L	0.6102694	0.662589987	-0.05232063	-0.11086956	0.054790546	0.091637751	4	0	0
SLC47A1	0.7918805	0.833108764	-0.04122827	-0.07267688	0.054869211	0.091760762	3	0	0
NECAP1	0.0964602	0.105392794	-0.00893257	-0.09007433	0.054890272	0.091787425	13	0	0
CISD3	0.302787	0.293391967	0.009395062	0.141770526	0.054903605	0.091801162	5	0	0
GTF2I	0.0386696	0.038245387	0.000424245	0.015213042	0.054957448	0.09187406	6	0	0
TRPC4AP	0.1099433	0.120816485	-0.01087314	-0.05998591	0.054954709	0.09187406	9	0	0
AVPR1B	0.1371578	0.128407736	0.00875004	0.148683712	0.054974606	0.091885616	9	0	0
SNW1	0.0441575	0.041034327	0.003123194	0.070448814	0.054974567	0.091885616	11	0	0
SGMS2	0.2387011	0.230519564	0.008181574	0.140874283	0.054995349	0.09191172	12	0	0
GPRIN2	0.3056236	0.302395714	0.003227866	0.135434542	0.055028697	0.091958885	6	0	0
HSPH1	0.1061276	0.105234912	0.000892644	-0.02542573	0.055036803	0.091963862	17	0	0
KIF22	0.1317857	0.12598111	0.005804584	0.07921376	0.055069021	0.092009125	11	0	0
TUBGCP3	0.4435907	0.45914621	-0.01555551	-0.06344073	0.05507654	0.092013117	10	0	0
CHAF1A	0.041235	0.038664359	0.002570616	0.06551329	0.05512964	0.092093249	7	0	0
CCDC124	0.0752587	0.072584011	0.002674723	0.008578199	0.055253767	0.092284744	7	0	0
RRP1B	0.448293	0.43695656	0.011336409	0.040412005	0.055254564	0.092284744	14	0	0
PTPMT1	0.2475603	0.257732094	-0.01017176	0.043621596	0.05528024	0.092319031	11	0	0
PADI4	0.4036452	0.419703512	-0.01605832	-0.05414319	0.055306968	0.092355067	3	0	0
TMEM199	0.1569157	0.161415462	-0.00449972	-0.03269581	0.05531227	0.092355322	15	0	0
KLHDC7A	0.3243001	0.310968617	0.013331481	0.059932287	0.05533961	0.092392371	7	0	0
ADAMTS7	0.2189477	0.212970776	0.005976916	0.027890078	0.05534906	0.092399548	9	0	0
PPP2CA	0.0821479	0.08885707	-0.00670916	-0.01563041	0.055357933	0.092404017	12	0	0

NUP188	0.2309084	0.229337059	0.00157136	0.051385284	0.055362041	0.092404017	10	0	0
GNPDA1	0.1966443	0.199390223	-0.00274591	-0.01328705	0.055380621	0.092426427	6	0	0
GPN2	0.3076026	0.313724058	-0.00612146	0.016599864	0.055392655	0.092429311	9	0	0
GLI3	0.1591636	0.167985216	-0.00882157	0.043467602	0.055391094	0.092429311	9	0	0
SYTL2	0.0672712	0.055189916	0.0120813	0.261902404	0.055402574	0.092437263	9	0	0
FAM78B	0.1675321	0.18192938	-0.01439733	-0.0138869	0.055442898	0.092495937	11	0	0
CRHR1	0.3431458	0.375965839	-0.03282003	-0.12820919	0.055515554	0.092608536	1	0	0
UNC5B	0.1509483	0.144615657	0.006332686	0.138457023	0.055559254	0.092672816	8	0	0
ADAR	0.0395349	0.044611826	-0.00507691	-0.07831664	0.055614301	0.092756008	6	0	0
IQCC	0.1685594	0.170373909	-0.00181454	0.028858191	0.055624622	0.092757875	13	0	0
PCMTD2	0.2938981	0.30199105	-0.00809298	-0.01868475	0.055625764	0.092757875	10	0	0
SEC61A1	0.368176	0.384863915	-0.01668791	-0.02666776	0.055654968	0.092797947	7	0	0
NDUFS1	0.063923	0.075520703	-0.01159768	-0.042076	0.05570254	0.092868633	16	0	0
NCOA4	0.2574665	0.279815288	-0.02234882	-0.08214926	0.055720915	0.092890633	7	0	0
SLC26A4	0.3410541	0.350122488	-0.0090684	0.060540074	0.055787669	0.092984632	7	0	0
FLT3LG	0.1529292	0.147938835	0.004990374	0.169963214	0.05578639	0.092984632	12	0	0
MRPL20	0.1235164	0.127162939	-0.00364653	-0.0178247	0.055793963	0.092986482	10	0	0
RBM23	0.1093243	0.117199529	-0.00787527	-0.00172448	0.055843778	0.093060856	9	0	0
MCL1	0.0779971	0.068681813	0.009315277	0.091764712	0.055872761	0.093100506	14	0	0
LAMP3	0.7411478	0.778745954	-0.03759819	-0.07309555	0.05588432	0.093111116	5	0	0
SYTL3	0.4210961	0.416555381	0.004540719	0.116879771	0.055947056	0.093206984	7	0	0
LCK	0.5425451	0.530534091	0.012011004	0.037971002	0.056001306	0.0932887	10	0	0
LACTB2	0.1608245	0.17895488	-0.01813038	-0.07604207	0.056022081	0.093305974	17	0	0
NOC3L	0.2238789	0.240078601	-0.01619968	0.021214251	0.056019011	0.093305974	7	0	0
FANCL	0.2524418	0.254110923	-0.00166912	-0.00286232	0.05603732	0.093322691	8	0	0
KAT2A	0.3922135	0.412400391	-0.02018687	-0.04549001	0.056067373	0.09336407	15	0	0
CCNL2	0.0417804	0.043920894	-0.00214052	-0.05568314	0.056134319	0.093466872	8	0	0
HHLA3	0.0500842	0.051405823	-0.00132161	-0.03591048	0.056147776	0.0934806	17	0	0
BORA	0.0385476	0.040455526	-0.0019079	-0.04701929	0.056175874	0.0935187	16	0	0
FBXO34	0.2521862	0.253153019	-0.00096686	0.001185598	0.056211187	0.093568802	9	0	0
CALHM2	0.3503804	0.344243821	0.006136622	0.157215862	0.056251349	0.093618538	12	0	0
FUT1	0.690896	0.710917688	-0.02002174	-0.04205504	0.056251504	0.093618538	7	0	0
MRPL36	0.1831948	0.179908394	0.003286449	0.193815027	0.05626732	0.093627484	6	0	0
COL23A1	0.2055394	0.199416354	0.006123003	0.21733112	0.056264564	0.093627484	12	0	0
C19orf33	0.4592888	0.445356521	0.013932307	0.032182724	0.056281438	0.093642288	4	0	0
TRAPPC1	0.1019461	0.099802865	0.00214322	0.045658155	0.056326992	0.093709389	14	0	0
LSM6	0.3366396	0.348930942	-0.0122913	-0.02607689	0.056366529	0.093766468	8	0	0
DYNLRB2	0.2821543	0.284512387	-0.00235808	0.038930931	0.056387982	0.093793457	11	0	0
BTD	0.1738429	0.183468822	-0.00962594	0.024716083	0.056409598	0.093820711	17	0	0
EGLN2	0.0710662	0.07979402	-0.00872786	-0.05124019	0.05643001	0.093845959	7	0	0
SNRNP27	0.1982925	0.209301604	-0.01100913	-0.0100932	0.056465357	0.093878629	11	0	0
EML5	0.1439108	0.155568833	-0.01165802	-0.07077619	0.056462208	0.093878629	7	0	0
ABCA9	0.6452047	0.684242148	-0.0390374	-0.08649951	0.056463559	0.093878629	5	0	0
ATG4D	0.13367	0.137597072	-0.00392708	-0.027928	0.05648545	0.09390333	8	0	0
MICAL3	0.2432342	0.256646575	-0.01341241	-0.08494745	0.056498437	0.093916216	11	0	0
INTS5	0.0404717	0.041944197	-0.00147245	-0.02610875	0.056543137	0.093981809	9	0	0
SMC4	0.0420581	0.044192192	-0.00213407	-0.04799154	0.05655255	0.093988745	13	0	0
IL20	0.358786	0.376450182	-0.01766417	0.048199713	0.056585265	0.094025692	7	0	0
ADAMTS3	0.0841802	0.066436254	0.017743924	0.332051491	0.056581929	0.094025692	9	0	0
XPC	0.0898423	0.08798035	0.001861906	0.015740163	0.056618916	0.094072893	11	0	0
HIST1H2AL	0.1490433	0.123002894	0.026040399	0.236121439	0.05663514	0.094091133	10	0	0
NDE1	0.0594497	0.057079159	0.002370505	0.0471776	0.056650693	0.094108255	12	0	0
LPCAT3	0.1792147	0.16317478	0.016039926	0.059044724	0.05666019	0.094115315	11	0	0
ABCB9	0.5257827	0.542314388	-0.01653167	-0.05983081	0.056671317	0.094125081	5	0	0
CASZ1	0.3192278	0.311607532	0.007620268	0.151438565	0.05668667	0.094141863	8	0	0
URB2	0.064784	0.07162325	-0.00683929	-0.02224231	0.056693912	0.094145172	9	0	0
PCBP4	0.3195798	0.318286683	0.001293076	0.095246271	0.056703949	0.094153123	9	0	0
CASP3	0.0966489	0.092609667	0.004039249	0.064561152	0.056723098	0.094176201	6	0	0
JMY	0.0482476	0.051833696	-0.00358609	-0.0784449	0.056761626	0.094226651	6	0	0
TOP3B	0.2308422	0.249752117	-0.01890989	-0.01618959	0.056763992	0.094226651	12	0	0
SNX27	0.0618311	0.067518315	-0.00568719	-0.04975007	0.056815433	0.094303313	10	0	0
ZBTB40	0.4359074	0.468821725	-0.0329143	-0.0509856	0.056885968	0.094396779	10	0	0
S100A13	0.1830671	0.201991273	-0.01892413	-0.05266854	0.056888924	0.094396779	9	0	0

MXI1	0.2509908	0.269619749	-0.01862898	-0.02449181	0.056892796	0.094396779	9	0	0
KLHDC4	0.042258	0.040535144	0.001722859	0.052591726	0.056891891	0.094396779	4	0	0
BRSK2	0.1776032	0.173148533	0.004454675	0.103378053	0.056901959	0.09440325	9	0	0
SETD1A	0.2442823	0.260806875	-0.01652461	-0.08335252	0.056907907	0.094404386	10	0	0
CADM4	0.1953605	0.192401285	0.002959182	0.099352675	0.056977863	0.094511694	6	0	0
USP24	0.0825387	0.087762412	-0.00522367	0.009604727	0.05698899	0.094512763	8	0	0
UBA2	0.1739376	0.182750236	-0.00881264	0.001392364	0.056992653	0.094512763	10	0	0
ZNF599	0.2856108	0.297003673	-0.01139285	0.041134931	0.056994315	0.094512763	12	0	0
ALG3	0.0420789	0.041648873	0.000430061	0.000310686	0.057005854	0.094523158	11	0	0
CORO2A	0.4833109	0.491789646	-0.00847872	0.003458013	0.05704976	0.094587216	2	0	0
NFASC	0.265179	0.269582211	-0.00440316	0.188674209	0.05705932	0.094594321	4	0	0
TRNP1	0.1149729	0.118849295	-0.00387641	0.055453468	0.0570876	0.094632459	10	0	0
TRIM74	0.7340701	0.794689483	-0.06061939	-0.11162006	0.057096896	0.09463912	2	0	0
ZNF440	0.0652378	0.042357163	0.022880647	0.559751555	0.057105692	0.094644954	13	0	0
CMTR1	0.2508052	0.244706646	0.006098586	0.051956729	0.057119794	0.09465958	8	0	0
SLC2A1	0.0650051	0.072813875	-0.00780875	-0.0971491	0.05713416	0.094674638	12	0	0
SFTPC	0.1170815	0.09968591	0.01739559	0.224291864	0.057157258	0.094704165	9	0	0
POM121C	0.2213913	0.226875771	-0.00548448	0.038823859	0.057209126	0.094781349	4	0	0
PTPRJ	0.142695	0.126016959	0.01667808	0.142224263	0.057237572	0.094819718	7	0	0
RAB23	0.0800163	0.094413522	-0.01439723	-0.06827303	0.057263238	0.094853476	13	0	0
DYNC2LI1	0.4088924	0.419665252	-0.0107729	-0.02069737	0.057296204	0.094899317	9	0	0
KRTAP2-1	0.7109172	0.72650633	-0.01558914	-0.02778937	0.057322734	0.094934493	7	0	0
RASGEF1A	0.4761643	0.458434229	0.017730099	0.147579209	0.057329692	0.09493725	5	0	0
CCDC170	0.21467	0.186013183	0.028656768	0.410758562	0.057393543	0.095034212	10	0	0
PHF14	0.0739899	0.090860507	-0.0168706	-0.08046636	0.057423345	0.095074783	14	0	0
EPM2A	0.2718372	0.273173213	-0.00133602	-0.05232609	0.057447384	0.095105804	8	0	0
GINS3	0.791578	0.816731334	-0.02515331	-0.04624329	0.057479724	0.095150562	8	0	0
STK16	0.1856204	0.195483965	-0.00986354	-0.01621289	0.057487567	0.095154763	17	0	0
FAM114A1	0.1666275	0.152009029	0.014618491	0.065444052	0.057515023	0.095191424	13	0	0
HNRNPC	0.1251205	0.126887961	-0.00176749	0.031508929	0.057550714	0.095241706	10	0	0
WDR11	0.4187871	0.41538636	0.003400734	0.014149031	0.057584621	0.095271448	8	0	0
MBNL2	0.4887697	0.497014433	-0.0082447	0.013924942	0.057583416	0.095271448	4	0	0
SLC9A3R2	0.8641068	0.859127382	0.004979387	0.009478371	0.057579749	0.095271448	3	0	0
GDF11	0.2805213	0.279298233	0.001223059	0.037761177	0.057600309	0.095288613	8	0	0
CCND1	0.0786494	0.086500969	-0.00785152	-0.07774279	0.057612938	0.095300715	27	0	0
PNRC2	0.2310365	0.254622769	-0.02358626	-0.05711648	0.057629486	0.095313056	7	0	0
CGB7	0.4755097	0.497936085	-0.02242641	-0.07164763	0.057631026	0.095313056	2	0	0
LYPLA1	0.3077548	0.322902276	-0.0151475	-0.07509732	0.05765734	0.095347783	7	0	0
DCAF7	0.0373715	0.039586638	-0.00221513	-0.00412103	0.057672497	0.095364055	11	0	0
CCNI	0.1684682	0.179460146	-0.01099193	0.005430599	0.057757514	0.095495831	5	0	0
RAC3	0.0725285	0.045115859	0.027412617	0.583834886	0.057809749	0.095573385	4	0	0
RPL35A	0.1387801	0.16812421	-0.02934411	-0.09062415	0.057819054	0.095576304	8	0	0
FAM221A	0.2980987	0.296322026	0.001776688	0.077875283	0.057822172	0.095576304	9	0	0
C2CD2L	0.0759028	0.081930248	-0.00602745	-0.02372403	0.057856428	0.095624115	22	0	0
MYLIP	0.1999377	0.210988689	-0.01105104	-0.01331293	0.057957467	0.095782284	9	0	0
AVPI1	0.0304114	0.027243963	0.003167464	0.094533176	0.057991575	0.095829822	6	0	0
ATP10D	0.1042476	0.119284997	-0.01503737	-0.00268535	0.057997386	0.095830596	10	0	0
ABCF2	0.0819571	0.088861205	-0.00690415	-0.03371051	0.058009202	0.095841291	12	0	0
BIRC3	0.1353735	0.142133198	-0.00675969	0.018181386	0.058024441	0.095857638	11	0	0
CRABP2	0.0932215	0.075656772	0.017564771	0.177644635	0.058098728	0.095971523	12	0	0
ACSF2	0.1583423	0.154017699	0.004324605	0.109841321	0.058106537	0.095973444	11	0	0
GBP1	0.3865723	0.396846123	-0.01027384	0.081367913	0.058123944	0.095977818	2	0	0
BCAN	0.2912482	0.260888395	0.030359836	0.239831853	0.058121175	0.095977818	5	0	0
UQCRC2	0.2069669	0.215070695	-0.00810382	-0.01737861	0.058130947	0.095980546	8	0	0
PIGP	0.0429022	0.038832156	0.00407002	0.076358932	0.058149063	0.096001619	8	0	0
ARL2BP	0.1684611	0.181195145	-0.01273407	0.014179618	0.058163291	0.096016271	6	0	0
RIN3	0.2623675	0.26118061	0.001186884	0.17372327	0.058222337	0.096104819	10	0	0
ZNF304	0.1722762	0.181543288	-0.00926705	0.045868894	0.058227646	0.096104819	10	0	0
FBXW9	0.0867036	0.096726426	-0.01002284	-0.0654876	0.058299398	0.096214392	12	0	0
RGS5	0.0407061	0.042742601	-0.00203654	-0.04424007	0.058411122	0.096381038	8	0	0
CHST14	0.2677805	0.264343139	0.003437325	0.029911515	0.058407824	0.096381038	7	0	0
BOLA3	0.4368306	0.442127018	-0.00529637	0.133848803	0.058422693	0.096391263	5	0	0
MYOM3	0.4310585	0.423282597	0.00777589	0.022399934	0.058449335	0.096425293	10	0	0

CAND1	0.0456877	0.047807458	-0.00211972	-0.05429899	0.058454071	0.096425293	10	0	0
TOM1L2	0.142091	0.156781404	-0.01469045	-0.01119452	0.058486409	0.096469765	11	0	0
DPY19L1	0.2197121	0.233745676	-0.01403353	-0.0656512	0.058498396	0.096480664	13	0	0
TMEM132A	0.2263124	0.240967746	-0.0146553	-0.02758154	0.058539051	0.09652109	9	0	0
PIK3R2	0.0942229	0.099706074	-0.0054832	-0.01588768	0.058531996	0.09652109	12	0	0
XKR7	0.1433669	0.128256425	0.015110524	0.171217147	0.058536666	0.09652109	8	0	0
CYP27C1	0.1561507	0.134627309	0.021523408	0.349318708	0.058556783	0.096541452	6	0	0
PLTP	0.3254465	0.332952928	-0.0075064	0.282817589	0.058576424	0.096564958	11	0	0
OR10AD1	0.7326996	0.76643594	-0.03373633	-0.06660006	0.05859696	0.096589935	6	0	0
GPR19	0.1111328	0.108829902	0.002302895	0.168002962	0.05862184	0.096622066	12	0	0
RANBP17	0.4619174	0.437009741	0.024907653	0.125040644	0.058670498	0.09669338	12	0	0
HIST1H2AG	0.062026	0.058087326	0.003938674	0.143184954	0.058676295	0.096694048	10	0	0
SERPINH1	0.3100891	0.302761108	0.007328001	0.113544549	0.05870363	0.096721322	10	0	0
RAVER1	0.2160262	0.188730943	0.027295259	0.093034269	0.058702746	0.096721322	8	0	0
RPS19	0.0470148	0.045266624	0.001748198	0.057037869	0.058721781	0.09674234	9	0	0
KRT74	0.6887995	0.714523556	-0.02572404	-0.0520502	0.058741083	0.096765252	7	0	0
NUP205	0.0812822	0.080575607	0.000706547	0.063164934	0.05878212	0.09682396	10	0	0
MPHOSPH8	0.1117628	0.122817719	-0.01105496	-0.0273598	0.058788739	0.09682597	8	0	0
CHMP2B	0.0526078	0.04828814	0.004319652	0.073632024	0.058804186	0.096842519	11	0	0
UFSP2	0.196039	0.213368549	-0.01732958	-0.0538172	0.058838926	0.096890835	5	0	0
ADAMTSL3	0.2052727	0.183940323	0.02133235	0.349895788	0.05885117	0.096902101	6	0	0
LFNG	0.4453628	0.43696692	0.008395886	0.058682328	0.058889239	0.096946983	8	0	0
DHRS7C	0.685183	0.70531528	-0.0201323	-0.04210611	0.058894869	0.096947354	9	0	0
ROM1	0.5297698	0.536263011	-0.00649318	0.016788474	0.058918934	0.096975874	10	0	0
YPEL3	0.7880957	0.813512226	-0.02541657	-0.04793504	0.058923008	0.096975874	5	0	0
NDST3	0.0634387	0.055312679	0.008126021	0.18615124	0.058956411	0.097021946	4	0	0
G6PC3	0.1349715	0.144038391	-0.00906693	-0.00081212	0.059001868	0.097087845	15	0	0
CCL24	0.5080047	0.53219224	-0.0241875	-0.07253324	0.059025267	0.097117438	5	0	0
MCTP1	0.1196741	0.102937508	0.016736606	0.232614495	0.059078174	0.097195573	6	0	0
CD52	0.5470173	0.576098604	-0.0290813	-0.07064344	0.059134594	0.097279472	8	0	0
HIST1H2AI	0.0958366	0.087089711	0.008746856	0.191620758	0.059183583	0.097347488	9	0	0
PSENEN	0.3060344	0.320512712	-0.01447832	-0.02885705	0.059186795	0.097347488	14	0	0
PURG	0.0827279	0.086310673	-0.0035828	0.017290137	0.059255256	0.097433284	19	0	0
PIF1	0.2411364	0.261150593	-0.02001424	-0.01965991	0.059253314	0.097433284	8	0	0
RSPRY1	0.097013	0.107480081	-0.01046711	-0.00862236	0.059245419	0.097433284	11	0	0
STK17A	0.1665052	0.171251475	-0.00474628	-0.01799907	0.059267197	0.097443986	11	0	0
ATG4C	0.104858	0.099108046	0.005749949	0.084585265	0.059314891	0.097513463	12	0	0
ZNF704	0.4551509	0.454759568	0.000391314	0.066274725	0.059336165	0.097539497	11	0	0
CREB3	0.0630145	0.057418233	0.005596225	0.07309713	0.059376446	0.097595583	9	0	0
RANGRF	0.1182744	0.117512377	0.000762019	0.067788954	0.059381166	0.097595583	11	0	0
SAYSD1	0.1598051	0.159085945	0.000719198	0.04093328	0.059422854	0.09765515	10	0	0
COX4I2	0.5743379	0.548277734	0.026060192	0.066858383	0.059450305	0.097691312	8	0	0
CAB39	0.1327359	0.145471046	-0.01273517	0.028450939	0.059472132	0.097718226	6	0	0
FMNL1	0.2543637	0.263665014	-0.00930128	0.193667696	0.059495049	0.097746927	7	0	0
ECT2	0.2926116	0.291714862	0.000896727	0.018436057	0.059585157	0.097877039	7	0	0
MUS81	0.0659114	0.075614954	-0.00970354	-0.08887257	0.059584586	0.097877039	6	0	0
PER1	0.0258952	0.026008224	-0.00011299	0.004944612	0.059604339	0.097899581	5	0	0
CPQ	0.1998838	0.204821799	-0.00493803	0.240345115	0.059617981	0.097913022	11	0	0
TPRN	0.2389729	0.25478423	-0.0158113	-0.03316785	0.05962903	0.097922201	8	0	0
ANXA7	0.1714306	0.18248296	-0.01105237	-0.02551997	0.059663003	0.097969022	10	0	0
YIPF2	0.1089639	0.119789926	-0.01082608	-0.01833863	0.059698354	0.098018096	15	0	0
PLSCR1	0.264354	0.26965308	-0.00529908	-0.08028299	0.059748951	0.098092191	10	0	0
OVCA2	0.646592	0.635539307	0.011052657	0.065069299	0.059759751	0.098100943	3	0	0
TTC34	0.5581437	0.524566487	0.033577247	0.087886567	0.059780567	0.098106999	1	0	0
C6orf106	0.149611	0.149092265	0.000518784	0.044750002	0.05978532	0.098106999	10	0	0
XYLT1	0.2934311	0.298185455	-0.00475434	0.035542727	0.059776342	0.098106999	5	0	0
ARHGAP23	0.2190871	0.199234802	0.019852334	0.188089218	0.05977263	0.098106999	8	0	0
CRYGA	0.7248937	0.761532259	-0.03663859	-0.06695355	0.059840251	0.098188156	7	0	0
GTF2E2	0.1320446	0.134684689	-0.0026401	-0.02623543	0.059846849	0.098190001	10	0	0
CDC73	0.1447371	0.13505773	0.009679342	0.042167658	0.059888497	0.098249345	8	0	0
SNX5	0.0669368	0.077530165	-0.01059333	-0.02772299	0.059908811	0.098273681	14	0	0
MAP7D1	0.595413	0.596683486	-0.00127047	0.001602599	0.060060082	0.098512815	3	0	0
CBS	0.316427	0.30403759	0.012389385	0.156197507	0.060095007	0.098561087	5	0	0

FAM8A1	0.4640922	0.487250471	-0.02315827	-0.04311858	0.060117575	0.098589085	5	0	0
PRKACG	0.9280734	0.94542375	-0.01735038	-0.02686497	0.060133972	0.09860696	4	0	0
ARHGEF18	0.1716274	0.149421609	0.022205758	0.137034326	0.060145175	0.098616314	6	0	0
GK5	0.2438704	0.2481734	-0.00430296	0.033749889	0.060151706	0.098618008	8	0	0
RIT1	0.3762935	0.39379446	-0.01750092	-0.05966965	0.060187388	0.098667488	7	0	0
ANKRD20A2	0.6335633	0.676977711	-0.04341443	-0.09418128	0.060203719	0.098685241	1	0	0
ANO7	0.5823971	0.58778527	-0.00538815	0.000939147	0.06023421	0.098726198	9	0	0
ALG1	0.0719922	0.071099294	0.000892903	0.061624072	0.06032476	0.098865579	8	0	0
HNRNPM	0.1612434	0.15683725	0.004406156	0.125022654	0.06033854	0.098879127	8	0	0
STAT3	0.0399645	0.030449271	0.009515225	0.192364685	0.060359932	0.098896113	12	0	0
ARFGAP1	0.2087026	0.220204942	-0.01150232	-0.06939372	0.060357456	0.098896113	7	0	0
PRIMPOL	0.0425643	0.042613643	-4.94E-05	0.011529356	0.060398351	0.098949941	7	0	0
ZNF45	0.2719222	0.286306298	-0.01438406	-0.01657014	0.060403819	0.098949941	10	0	0
MCM2	0.1978241	0.207688361	-0.00986429	0.001103549	0.060412968	0.09895589	13	0	0
FBXL16	0.1388365	0.139515846	-0.00067937	0.138006427	0.060454434	0.099014769	6	0	0
PSMD1	0.2170928	0.223695957	-0.00660313	0.002016209	0.060481318	0.099040712	9	0	0
VDAC1	0.1728805	0.160264767	0.012615742	0.074946741	0.060480114	0.099040712	10	0	0
NMT1	0.057281	0.0570008	0.000280152	0.009950131	0.060487236	0.09904136	6	0	0
ARHGEF33	0.7749837	0.805553119	-0.03056938	-0.05633429	0.060620722	0.099232724	2	0	0
UNC80	0.1546	0.146084927	0.008515071	0.18641873	0.060615064	0.099232724	9	0	0
GTF2H4	0.1907906	0.184125916	0.00666465	0.015430997	0.060626237	0.099232724	8	0	0
VARS2	0.1907906	0.184125916	0.00666465	0.015430997	0.060626237	0.099232724	8	0	0
PTGS2	0.1140676	0.09782097	0.016246607	0.181783458	0.060664043	0.099276485	11	0	0
COQ10A	0.3390714	0.349136657	-0.01006526	-0.00175313	0.060663101	0.099276485	11	0	0
MAPK11	0.0611888	0.06668389	-0.0054951	-0.00485244	0.060673521	0.099282937	9	0	0
MEGF6	0.1119038	0.108895933	0.003007851	0.200382007	0.060739224	0.099372317	13	0	0
GDF5	0.0353018	0.033267942	0.002033877	0.07648102	0.060737712	0.099372317	9	0	0
RSBN1	0.1814898	0.175871947	0.005617851	0.072240626	0.060746902	0.099375815	10	0	0
SV2B	0.1495077	0.154118199	-0.00461054	0.027178483	0.060780307	0.099419051	16	0	0
CALR3	0.223918	0.245389878	-0.02147184	-0.03903498	0.060784418	0.099419051	9	0	0
CCDC50	0.2744061	0.286009253	-0.01160311	0.017107799	0.060792092	0.099422537	8	0	0
MGLL	0.4507853	0.464402686	-0.0136174	0.083418555	0.060822263	0.099427341	6	0	0
PHF3	0.1333552	0.138095046	-0.00473989	-0.08123021	0.060815979	0.099427341	9	0	0
ZNF10	0.1196844	0.122422559	-0.00273818	0.026179316	0.060822746	0.099427341	14	0	0
ZNF268	0.1196844	0.122422559	-0.00273818	0.026179316	0.060822746	0.099427341	14	0	0
KIF13B	0.0446872	0.035924871	0.008762361	0.207908629	0.060840157	0.099437676	11	0	0
RPLP0	0.09168	0.09833891	-0.00665895	-0.00478504	0.060835092	0.099437676	10	0	0
CMSS1	0.0472401	0.04540273	0.001837398	0.057572797	0.060857372	0.099456749	11	0	0
TRIM37	0.0433202	0.045389148	-0.00206896	-0.04355707	0.060870744	0.09946954	11	0	0
APEH	0.0892245	0.099380581	-0.0101561	-0.06583239	0.060929171	0.099555945	11	0	0
SIVA1	0.1038011	0.121716652	-0.01791559	-0.04130674	0.060947258	0.099576428	7	0	0
KCNT1	0.3369401	0.345301015	-0.00836092	0.027463811	0.06095978	0.099587814	5	0	0
CCL2	0.3180593	0.310498104	0.007561148	0.156214128	0.06100865	0.099649499	7	0	0
CCDC25	0.5303583	0.525352228	0.005006094	0.018997338	0.061047626	0.099695004	6	0	0
CRB3	0.287003	0.295148009	-0.00814496	0.04239372	0.061047379	0.099695004	14	0	0
ATF6B	0.3617843	0.377786016	-0.01600176	-0.0144049	0.061067744	0.099718778	35	0	0
ZNF286B	0.8408675	0.858628833	-0.01776131	-0.02980532	0.061091839	0.099749041	1	0	0
ACOX1	0.0826942	0.090906706	-0.00821246	-0.05662204	0.061130058	0.099802359	13	0	0
P4HTM	0.0584997	0.063070277	-0.00457054	-0.0008523	0.061192344	0.099894955	18	0	0
GPT	0.6490296	0.636888779	0.012140813	0.021793254	0.061218776	0.099929011	9	0	0
RBM20	0.1838585	0.167083669	0.016774865	0.227406257	0.061228642	0.09993602	8	0	0
MYNN	0.0492096	0.048655291	0.000554344	0.041000244	0.061242088	0.099948872	9	0	0
KIF2C	0.0324426	0.031473448	0.000969134	0.035758827	0.061257521	0.099964962	7	0	0
MRPS27	0.1082199	0.110748286	-0.00252837	-0.03168539	0.061269132	0.099974813	12	0	0
MSC	0.143635	0.113281942	0.030353035	0.382245484	0.061294875	0.099991156	12	0	0
RBM17	0.1828532	0.208344697	-0.02549153	-0.10912421	0.061293886	0.099991156	9	0	0
CPSF2	0.0581651	0.05604608	0.002118989	0.039729983	0.061295872	0.099991156	7	0	0
GTF3C1	0.0303136	0.029107837	0.001205718	0.031812683	0.061432983	0.10020571	12	0	0
TBCC	0.0511929	0.04884642	0.002346487	0.056558729	0.061447235	0.100219842	6	0	0
RARS	0.2631931	0.279274816	-0.01608171	-0.02879936	0.061466704	0.100242481	6	0	0
PRR14	0.0666802	0.057059841	0.009620318	0.101398911	0.061502558	0.100291834	14	0	0
KCNN3	0.2828765	0.287741173	-0.0048647	0.106017132	0.061549938	0.100359972	11	0	0
LRRN4CL	0.7163116	0.727324064	-0.01101248	-0.0267152	0.061565799	0.100367585	13	0	0

RGS6	0.2155588	0.231556366	-0.01599759	0.01454743	0.061563808	0.100367585	14	0	0
NEGR1	0.080405	0.078228397	0.002176594	0.019852276	0.0616144	0.100434967	12	0	0
TMTC3	0.0585021	0.064922319	-0.00642025	-0.09116901	0.061618331	0.100434967	13	0	0
CAND2	0.1191295	0.099928776	0.019200745	0.527173409	0.061624627	0.100436102	8	0	0
SSTR2	0.1154884	0.0888219	0.026666523	0.432555579	0.061649201	0.100467024	10	0	0
PTPN4	0.1686402	0.182090047	-0.01344987	-0.06062672	0.061730002	0.100589563	12	0	0
HSD17B6	0.0351503	0.033117177	0.002033172	0.062730041	0.061767485	0.100641497	13	0	0
PEMT	0.0818382	0.088878124	-0.00703995	-0.0186777	0.061777451	0.100648593	12	0	0
KLHL2	0.1425221	0.125978279	0.016543855	0.076971892	0.061811925	0.100695611	7	0	0
BMS1	0.2240698	0.241381991	-0.01731216	-0.07378512	0.061829878	0.100706564	6	0	0
PMAIP1	0.1672828	0.178568306	-0.01128551	-0.08307513	0.061827359	0.100706564	11	0	0
PXMP2	0.1042779	0.11754008	-0.01326215	-0.02686928	0.061889403	0.100794365	9	0	0
ATE1	0.1371782	0.15301603	-0.01583784	-0.14159084	0.061912387	0.100813488	7	0	0
ALOX15	0.4253392	0.403085024	0.022254179	0.06625927	0.061910597	0.100813488	8	0	0
INCA1	0.1464208	0.151739764	-0.005319	-0.0003848	0.061946337	0.100859613	8	0	0
USP22	0.210743	0.2037317	0.007011324	0.119541085	0.061959932	0.100872592	8	0	0
ACLY	0.0412183	0.041002029	0.000216289	0.018052068	0.061977864	0.100892628	11	0	0
MED28	0.0393795	0.038952543	0.000426982	0.025928901	0.062049076	0.10099022	6	0	0
FNIP1	0.0818851	0.083065163	-0.00118007	0.023779642	0.06204596	0.10099022	13	0	0
WRN	0.0849564	0.07575487	0.009201482	0.125911009	0.062066168	0.101008873	27	0	0
FNDC8	0.4157502	0.42968285	-0.01393264	-0.00990489	0.062076649	0.101016764	9	0	0
PEF1	0.1913289	0.20457783	-0.01324897	-0.04688486	0.062094755	0.101037061	13	0	0
FUZ	0.1537011	0.16265415	-0.00895302	-0.02007136	0.062117114	0.101055108	5	0	0
MED25	0.1537011	0.16265415	-0.00895302	-0.02007136	0.062117114	0.101055108	5	0	0
ANKLE2	0.309976	0.325754331	-0.0157783	-0.0422499	0.062134887	0.101074854	6	0	0
CCDC85B	0.1226641	0.125410267	-0.00274617	0.036818313	0.062145259	0.101082558	12	0	0
THUMPD2	0.319805	0.339438427	-0.01963346	-0.04026017	0.062166827	0.101093	8	0	0
GPR155	0.170559	0.172142199	-0.0015832	0.019938907	0.062170409	0.101093	14	0	0
NUDT9	0.2751174	0.285656224	-0.01053886	-0.02350073	0.062174224	0.101093	8	0	0
MIDN	0.1742199	0.162852025	0.011367884	0.197628133	0.062169755	0.101093	9	0	0
CARHSP1	0.1719447	0.180900426	-0.00895573	0.003624465	0.062197912	0.101122349	14	0	0
SLC22A15	0.1937038	0.192515886	0.001187902	0.134501419	0.062204837	0.101124441	13	0	0
GNL2	0.1491728	0.165946859	-0.01677405	-0.04096507	0.062227142	0.101151534	11	0	0
PLCL1	0.1103958	0.12164676	-0.01125094	-0.04337581	0.062256113	0.101189456	10	0	0
ZNF747	0.0688709	0.071236591	-0.00236568	0.034445191	0.062284191	0.101221856	13	0	0
OSCAR	0.2453571	0.252428244	-0.00707112	0.05324049	0.062287334	0.101221856	8	0	0
PGGT1B	0.3208157	0.329851234	-0.00903553	-0.01361954	0.062317006	0.101260901	14	0	0
MFSD10	0.0386697	0.037254103	0.001415632	0.047283505	0.062341008	0.101290726	10	0	0
ATAD3A	0.0232761	0.021612735	0.001663374	0.066462619	0.062350612	0.101292942	4	0	0
CCDC71L	0.3788086	0.399307118	-0.02049853	-0.02002745	0.062353667	0.101292942	9	0	0
TBL2	0.1135329	0.117043381	-0.00351046	0.06066614	0.062382076	0.101329914	12	0	0
ZSCAN20	0.2380828	0.250933426	-0.0128506	-0.01440685	0.062393041	0.101338548	8	0	0
ALG12	0.3782696	0.386740728	-0.00847114	-0.02337816	0.062400434	0.101341378	5	0	0
MTOR	0.0903382	0.096484357	-0.0061462	0.015911347	0.062432696	0.101384593	10	0	0
PEX6	0.0854691	0.075752302	0.009716844	0.223095992	0.062439058	0.101385746	11	0	0
OSBPL10	0.6304485	0.593360245	0.037088277	0.086062972	0.062505425	0.101477961	1	0	0
OXR1	0.2929127	0.287176981	0.005735744	0.102644413	0.062507165	0.101477961	11	0	0
SERINC5	0.1550633	0.164521017	-0.00945771	0.092445253	0.062518939	0.10148789	5	0	0
BEST1	0.6095526	0.614589483	-0.00503685	-0.00567317	0.062539004	0.101511275	9	0	0
AKR1C1	0.8868969	0.891915198	-0.00501831	-0.00804497	0.062556025	0.101520528	2	0	0
TMEM106C	0.1634728	0.1737265	-0.01025365	0.002787495	0.062555461	0.101520528	7	0	0
SLC25A16	0.1331242	0.140528406	-0.00740417	-0.05116564	0.062567588	0.101530106	12	0	0
CLPTM1	0.2546027	0.261915574	-0.00731282	-0.01038413	0.062575047	0.101533024	9	0	0
RAB3A	0.044293	0.038902268	0.005390708	0.140210838	0.06258157	0.101534422	8	0	0
TYW3	0.1130751	0.116227579	-0.0031525	-0.06494156	0.062607195	0.101566809	12	0	0
OST4	0.1110027	0.094610933	0.016391807	0.096124726	0.062661114	0.101645089	5	0	0
TSEN15	0.0455138	0.040158261	0.005355533	0.091863957	0.062708631	0.101705567	11	0	0
SNAP47	0.0460815	0.04870232	-0.00262078	-0.04394088	0.062709738	0.101705567	12	0	0
DMP1	0.8127098	0.815768684	-0.00305884	-0.00582215	0.062724029	0.101716983	5	0	0
SLC35G1	0.2670165	0.270987859	-0.00397137	-0.01002763	0.062728119	0.101716983	7	0	0
TLR5	0.2147731	0.222462429	-0.00768933	-0.0338986	0.062739293	0.101725904	7	0	0
FECH	0.3697958	0.383955656	-0.01415982	-0.01286012	0.062758962	0.101748599	9	0	0
GPBP1L1	0.0432112	0.042324832	0.000886389	-0.01586146	0.062795999	0.101799443	7	0	0

SEPHS1	0.0383811	0.038298049	8.30E-05	0.005786122	0.062808332	0.101810234	8	0	0
AFM	0.794605	0.808381326	-0.01377629	-0.02825299	0.062822744	0.101824394	4	0	0
RASA1	0.1986435	0.19399064	0.004652821	0.08195579	0.062852381	0.101863225	13	0	0
UTS2B	0.4516041	0.410637273	0.04096686	0.129335972	0.062890887	0.101916421	7	0	0
SLC27A5	0.6762722	0.676079514	0.000192659	0.003307614	0.062937877	0.101983355	6	0	0
DCP2	0.1516418	0.155490514	-0.00384869	-0.03340515	0.062944653	0.101985121	11	0	0
LMO4	0.1918455	0.172144045	0.019701495	0.106903369	0.062979621	0.102003732	10	0	0
FUCA2	0.4674841	0.460578256	0.006905811	0.087767214	0.062967979	0.102003732	13	0	0
08-Mar	0.2589365	0.269898905	-0.01096243	-0.00729984	0.062981464	0.102003732	10	0	0
FBLN5	0.1628475	0.153902025	0.008945504	0.207441228	0.062981366	0.102003732	10	0	0
ITGAX	0.1129772	0.105047083	0.007930106	0.28634604	0.062984575	0.102003732	4	0	0
LRRC31	0.7061819	0.725691802	-0.01950987	-0.03213516	0.062998936	0.102017778	6	0	0
AGFG1	0.3552952	0.360929095	-0.0056339	-0.0111632	0.063015622	0.102035587	11	0	0
TECR	0.581887	0.617712482	-0.03582551	-0.0847822	0.063040417	0.102066521	1	0	0
UNG	0.1666518	0.163942168	0.002709637	-0.00073692	0.06304712	0.10206816	11	0	0
ZYX	0.0940817	0.107604935	-0.0135232	-0.03673248	0.06314863	0.10222327	9	0	0
COL1A2	0.2686422	0.241786952	0.026855288	0.13821751	0.063156844	0.102227339	12	0	0
MARK3	0.1148135	0.12777696	-0.01296343	-0.02272157	0.063201145	0.102280587	11	0	0
IVNS1ABP	0.1212248	0.134142265	-0.0129175	-0.04554332	0.063236345	0.102328319	9	0	0
EIF3K	0.1894348	0.166980234	0.022454524	0.21106814	0.063306046	0.102431866	9	0	0
MCM8	0.1125989	0.130731186	-0.01813229	-0.03282805	0.06331562	0.102438117	15	0	0
PELI2	0.0595462	0.059597101	-5.09E-05	0.018170628	0.06333993	0.102468205	5	0	0
GNB2	0.1923351	0.204881034	-0.01254594	-0.0391499	0.06335508	0.102482507	10	0	0
NRSN2	0.2199356	0.206609659	0.013325894	-0.01075645	0.063360198	0.102482507	7	0	0
DESI1	0.0383179	0.035398031	0.002919844	0.081612727	0.063381063	0.102507011	16	0	0
ENTPD6	0.4200103	0.436317859	-0.01630751	-0.02425324	0.063404216	0.102535211	8	0	0
ARL5C	0.5217848	0.534612775	-0.01282793	-0.02595189	0.063422564	0.102555635	3	0	0
SIRT3	0.1418589	0.153487571	-0.01162864	-0.05681197	0.063452426	0.102594674	8	0	0
ZNRF1	0.1111373	0.108867347	0.002269999	0.039689099	0.063469263	0.102612646	7	0	0
NFKBIA	0.1109572	0.099298015	0.011659182	0.037808283	0.063480632	0.102621776	6	0	0
YOD1	0.1210017	0.121255199	-0.00025345	-0.06088851	0.063504826	0.10264605	13	0	0
RPL34	0.1196954	0.119791825	-9.65E-05	0.01033421	0.063524769	0.102659456	12	0	0
ELMOD1	0.0692502	0.072227776	-0.00297753	0.066953825	0.06353726	0.102659456	10	0	0
TAS2R42	0.5769837	0.608501691	-0.03151797	-0.09185198	0.063534345	0.102659456	3	0	0
AKAP6	0.7855943	0.798917751	-0.01332348	-0.0215414	0.063538281	0.102659456	7	0	0
EXOC3L2	0.5757668	0.565806645	0.009960183	0.025132041	0.063547023	0.102664332	9	0	0
RBM22	0.10004	0.122770454	-0.02273042	-0.02671844	0.063566752	0.102686956	8	0	0
TMEM63B	0.0890116	0.102967071	-0.01395545	-0.04488654	0.063610173	0.102747845	8	0	0
DNAJC11	0.4495571	0.46755427	-0.01799714	-0.0447397	0.063723222	0.102921181	8	0	0
ATOH1	0.1027858	0.08660727	0.016178553	0.189539628	0.063746116	0.102948887	11	0	0
CDKN2D	0.0243636	0.023343601	0.001020003	0.045198115	0.063770651	0.102979237	4	0	0
C1orf123	0.1216116	0.12837777	-0.00676618	-0.00712533	0.063782024	0.102988331	11	0	0
DDAH1	0.4645594	0.444611901	0.019947486	0.080857456	0.063789558	0.102991225	7	0	0
COG1	0.1149482	0.108552243	0.006396005	-0.00480899	0.063809091	0.103013489	12	0	0
INPP4A	0.1699772	0.176661009	-0.00668384	-0.02297951	0.063825194	0.103030211	12	0	0
C1S	0.6773183	0.712461798	-0.0351435	-0.07872432	0.063946636	0.103216961	5	0	0
ARHGEF16	0.1362846	0.145342651	-0.00905804	-0.06231398	0.064009669	0.103309405	10	0	0
IER3	0.2155932	0.19253791	0.023055327	0.439899724	0.064019431	0.103315865	19	0	0
MYH11	0.1355851	0.124498474	0.011086654	0.31963322	0.064050754	0.103357115	3	0	0
PMM1	0.0426449	0.03893152	0.003713391	0.08275856	0.064057393	0.103358528	10	0	0
LIG4	0.1446884	0.161348896	-0.01666048	-0.04650897	0.064106684	0.103428756	11	0	0
LCN9	0.516729	0.512959203	0.003769749	0.013911258	0.064126841	0.103451971	6	0	0
ZNF718	0.1696871	0.151393777	0.018293318	0.484714216	0.06413784	0.10346041	6	0	0
TTC12	0.2176978	0.226734856	-0.00903702	0.005495575	0.064146037	0.103464328	12	0	0
EMC1	0.0559056	0.056475453	-0.00056981	-0.02259023	0.064201713	0.103538378	12	0	0
PDZRN4	0.4039828	0.390542234	0.013440521	0.209965986	0.064222652	0.103559965	7	0	0
NME1	0.0583355	0.057039971	0.001295577	0.043619244	0.064259446	0.103582046	13	0	0
NME1-NME	0.0583355	0.057039971	0.001295577	0.043619244	0.064259446	0.103582046	13	0	0
NME2	0.0583355	0.057039971	0.001295577	0.043619244	0.064259446	0.103582046	13	0	0
FKRP	0.173451	0.164376643	0.009074356	0.090049804	0.064251302	0.103582046	11	0	0
SON	0.0510386	0.056284582	-0.00524596	-0.03891894	0.064285416	0.103614595	10	0	0
ABCA6	0.5149166	0.533088832	-0.01817221	-0.029044	0.064334528	0.103684437	6	0	0
DDI2	0.0896635	0.089703306	-3.98E-05	0.077803984	0.064356763	0.103710953	11	0	0

02-Mar	0.1009732	0.079929005	0.0210442	0.319754823	0.064418418	0.103800983	11	0	0
SND1	0.0718276	0.079942347	-0.0081148	-0.07221652	0.06445384	0.103848732	13	0	0
NDRG3	0.0945466	0.09755601	-0.00300937	0.009389285	0.064461568	0.103851853	11	0	0
PSMB7	0.2357295	0.24453687	-0.00880732	-0.08548907	0.064551232	0.103970304	9	0	0
PCNT	0.0391118	0.041567646	-0.00245583	-0.03736457	0.064552481	0.103970304	11	0	0
FBXO47	0.4420214	0.423068124	0.018953272	0.197418196	0.064562052	0.103976382	10	0	0
ANKRD13C	0.045206	0.048126833	-0.00292081	-0.0574079	0.06461523	0.104052682	15	0	0
PCED1A	0.1679287	0.15190143	0.016027287	0.134664588	0.064663961	0.104121807	14	0	0
UCK1	0.1817899	0.194668726	-0.01287886	0.009959999	0.064679039	0.104136737	5	0	0
IGF2BP2	0.1409662	0.114512004	0.026454176	0.229499155	0.064691898	0.104138744	6	0	0
SEC24D	0.6785246	0.708062014	-0.02953737	-0.05832879	0.064690586	0.104138744	2	0	0
MRPL51	0.1087685	0.123778724	-0.01501019	-0.04813198	0.064711881	0.104161563	11	0	0
NT5M	0.1404278	0.14259143	-0.00216361	-0.01557373	0.064753441	0.104219106	8	0	0
C1QB	0.57169	0.589847947	-0.01815797	-0.03975427	0.064764635	0.104221826	8	0	0
ZNF717	0.1286443	0.099497018	0.029147304	0.340496952	0.064766753	0.104221826	1	0	0
CIB2	0.0685446	0.068015939	0.000528683	0.192094906	0.064815862	0.104291495	7	0	0
ZNF317	0.0630342	0.066423467	-0.0033893	-0.0114303	0.064830214	0.104305232	11	0	0
KIAA1109	0.163695	0.137055102	0.026639869	0.158519365	0.064866538	0.104354312	2	0	0
DRAM2	0.0656063	0.072361475	-0.00675513	-0.01571853	0.064887987	0.104379456	10	0	0
ULBP3	0.224868	0.238502048	-0.01363404	0.189613059	0.064960894	0.104487364	9	0	0
GPD1	0.572268	0.573808371	-0.00154038	0.014788975	0.064998902	0.104539124	10	0	0
KIF20B	0.3382925	0.344691001	-0.00639855	-0.02110203	0.065021113	0.104565469	6	0	0
IDH3B	0.1600935	0.178992255	-0.0188988	-0.02020488	0.065028048	0.104567247	14	0	0
ANAPC4	0.1961263	0.209202777	-0.01307649	-0.0423497	0.065042682	0.104568331	6	0	0
RFC2	0.0511513	0.049092916	0.002058422	0.063930628	0.065046213	0.104568331	7	0	0
GRIN3B	0.2080554	0.191722826	0.01633262	0.412333581	0.065108643	0.104649935	3	0	0
ZNF761	0.0585264	0.044544876	0.013981546	0.361127507	0.065105374	0.104649935	3	0	0
SRSF7	0.1241211	0.129555459	-0.00543434	0.040293403	0.065154931	0.104705568	9	0	0
VSIG10L	0.5800729	0.574739676	0.005333266	0.025082573	0.065151335	0.104705568	3	0	0
TTLL6	0.161134	0.162311465	-0.00117745	0.188073173	0.065161059	0.104706034	14	0	0
VASH2	0.3288298	0.338314365	-0.00948452	0.077609544	0.065284229	0.104894556	8	0	0
SUSD1	0.2109913	0.195049174	0.015942104	0.202874174	0.065327317	0.104954385	7	0	0
TIMM10	0.0881832	0.095126222	-0.00694299	0.024565783	0.065335427	0.104958013	11	0	0
C4orf47	0.2798508	0.301628841	-0.02177805	-0.05933334	0.065350871	0.104973421	6	0	0
LONP1	0.1330675	0.147247063	-0.01417954	-0.04748478	0.065358275	0.104975914	11	0	0
RANBP10	0.0992232	0.099076972	0.000146254	0.036006098	0.06538735	0.105013209	14	0	0
UGGT1	0.0911499	0.104280589	-0.01313071	0.007770656	0.065413149	0.105035832	10	0	0
BTN3A1	0.4194776	0.432236904	-0.01275926	0.01237158	0.065409986	0.105035832	6	0	0
APLF	0.0533987	0.062168213	-0.00876947	-0.07441466	0.065482855	0.105138349	14	0	0
ALOX5	0.1489203	0.147664313	0.001256027	0.092634374	0.065509863	0.105172297	11	0	0
ITGA2	0.1375277	0.148241227	-0.01071349	0.029885221	0.065520505	0.105179968	9	0	0
GANAB	0.2825979	0.293677351	-0.01107949	0.003775773	0.065544456	0.105209	12	0	0
RASL11A	0.5665245	0.584663176	-0.01813867	-0.04354802	0.065583311	0.105261947	7	0	0
RAMP1	0.433906	0.443305613	-0.00939965	-0.0030256	0.0656008	0.105280596	7	0	0
ACHE	0.0785953	0.06154455	0.017050716	0.380091354	0.065634725	0.105316195	5	0	0
DNTTIP1	0.1939724	0.204835612	-0.01086319	-0.05419316	0.065682457	0.105383357	12	0	0
NCAPG	0.0673205	0.071866377	-0.00454587	-0.0322355	0.065723696	0.105440091	11	0	0
ZKSCAN8	0.1928247	0.195532088	-0.00270739	0.009861173	0.065747349	0.105468602	11	0	0
ARMC6	0.0753353	0.058103761	0.017231576	0.125259601	0.065773469	0.105501067	7	0	0
SARM1	0.9069348	0.922768831	-0.01583401	-0.02501922	0.065798173	0.105531256	2	0	0
NASP	0.0326407	0.032407646	0.000233057	0.019837992	0.065834761	0.105571057	7	0	0
SLC10A4	0.276939	0.256585164	0.020353885	0.165589016	0.065831763	0.105571057	9	0	0
FOLH1	0.3389339	0.374890232	-0.03595637	0.02226529	0.065852921	0.105590737	7	0	0
PRSS35	0.2350173	0.226351046	0.008666237	0.226008374	0.065860007	0.10559266	5	0	0
MCF2L2	0.0500056	0.051884215	-0.00187866	-0.03331208	0.065884312	0.10561949	8	0	0
ECE2	0.0377966	0.038133567	-0.00033701	-0.0197059	0.065888519	0.10561949	17	0	0
SRMS	0.5287322	0.547474994	-0.01874279	-0.03689363	0.065910047	0.105644558	10	0	0
MARK2	0.1197868	0.109744253	0.010042595	0.055469725	0.066000939	0.105780791	12	0	0
SLC26A8	0.5592824	0.544620194	0.014662188	0.062492686	0.066062454	0.105869923	11	0	0
OAZ3	0.6339211	0.652281977	-0.01836091	-0.03551905	0.066126515	0.105963118	4	0	0
SCHIP1	0.5901547	0.555971596	0.034183116	0.091020369	0.066137015	0.105970475	9	0	0
LRIG2	0.1142742	0.117081616	-0.0028074	-0.04910771	0.066179819	0.106029587	10	0	0
ZPLD1	0.8597656	0.893746985	-0.03398136	-0.05589897	0.066204356	0.106049954	2	0	0

ZNF138	0.427	0.44311618	-0.01611613	-0.01981656	0.066201287	0.106049954	4	0	0
SNN	0.1978777	0.206870138	-0.00899248	-0.0227215	0.066253877	0.106119801	8	0	0
ZNRF3	0.0967483	0.085614066	0.011134205	0.133053917	0.0663422	0.106251781	6	0	0
SLC25A33	0.1987866	0.205461566	-0.00667495	-0.00919437	0.066362269	0.106274434	4	0	0
FOXD1	0.145296	0.139676724	0.005619313	0.033899613	0.066407538	0.106337435	5	0	0
GPR88	0.5886305	0.606713841	-0.01808331	-0.0345838	0.066619092	0.10665778	4	0	0
SEMA6B	0.4446167	0.45474832	-0.01013157	-0.04050847	0.066619487	0.10665778	3	0	0
ANKK1	0.4823108	0.497956869	-0.01564606	-0.0086116	0.066644718	0.106684055	8	0	0
UQCR11	0.0536749	0.051574276	0.002100665	0.047787852	0.066647794	0.106684055	13	0	0
MON1B	0.0348793	0.037923567	-0.00304427	-0.04791802	0.06666653	0.106704523	6	0	0
TMEM14B	0.1673661	0.169347839	-0.00198176	-0.00533855	0.066679066	0.106705543	7	0	0
SYCP2L	0.1673661	0.169347839	-0.00198176	-0.00533855	0.066679066	0.106705543	7	0	0
SP1	0.069176	0.063997092	0.005178927	0.04487114	0.066731471	0.10677988	14	0	0
AVEN	0.1667402	0.16365274	0.003087472	-4.09E-05	0.066749662	0.106799459	5	0	0
EIF4G3	0.1546636	0.153376233	0.001287362	0.02321142	0.066760261	0.106806891	7	0	0
URAD	0.752684	0.763085909	-0.0104019	-0.0146935	0.066798626	0.106858736	8	0	0
CYB561D1	0.2487925	0.260351667	-0.01155913	-0.03529495	0.066848897	0.106929619	13	0	0
IQSEC1	0.3022625	0.274182881	0.028079627	0.268640435	0.066918828	0.107031933	14	0	0
ARL4C	0.486916	0.49604701	-0.009131	-0.04000888	0.06698246	0.107114605	11	0	0
ZNF461	0.2162913	0.204485748	0.011805526	0.41531451	0.066981938	0.107114605	11	0	0
UPK1B	0.5489014	0.53909635	0.009805059	0.058835726	0.067029746	0.107180666	7	0	0
NDFIP1	0.2966674	0.307518339	-0.01085095	-0.02375396	0.067073726	0.107241429	11	0	0
RFC3	0.143507	0.166067793	-0.02256082	-0.08344118	0.067103179	0.107278957	7	0	0
EPHA5	0.1331642	0.105474234	0.02768993	0.374058707	0.067162073	0.107363541	9	0	0
PGF	0.4113248	0.416336421	-0.00501158	-0.00795335	0.067168156	0.107363697	12	0	0
ARL10	0.0737384	0.045258966	0.028479438	0.887792997	0.06718258	0.107377184	12	0	0
PLEKHO2	0.0912886	0.076695159	0.014593453	0.089644987	0.06722505	0.107428536	9	0	0
ASGR1	0.5548431	0.578311587	-0.02346847	-0.0460966	0.067226689	0.107428536	9	0	0
MRPL19	0.2880185	0.311007413	-0.02298895	-0.04891152	0.067257121	0.107467592	13	0	0
CSK	0.0706341	0.076777169	-0.00614302	0.057163425	0.067286204	0.107504485	8	0	0
ENO4	0.1072753	0.121833377	-0.01455807	-0.02819947	0.067394616	0.107645074	15	0	0
TMEM9B	0.1875711	0.192611615	-0.00504048	-0.03252644	0.06739343	0.107645074	12	0	0
INF2	0.2149568	0.197332334	0.017624449	0.083220951	0.067398203	0.107645074	10	0	0
ZNF207	0.2065045	0.206391735	0.00011279	0.027558587	0.067380251	0.107645074	13	0	0
CD200R1L	0.7527687	0.769062257	-0.01629353	-0.02722915	0.06740959	0.107653673	6	0	0
ZNF624	0.2737543	0.279366055	-0.00561172	0.002400167	0.067418196	0.107657832	9	0	0
VPS29	0.2056993	0.214144911	-0.00844558	0.001821793	0.067437165	0.107678535	15	0	0
ZNF121	0.1325766	0.132189057	0.000387547	0.096598847	0.067515292	0.107793686	9	0	0
PPP1R8	0.1585415	0.16315773	-0.00461624	-0.02295952	0.067530529	0.107808417	10	0	0
DYRK1A	0.1997875	0.170554024	0.029233444	0.148678668	0.067547322	0.107825629	3	0	0
CYP4F12	0.4114374	0.384645815	0.026791587	0.171735489	0.067650541	0.107980787	2	0	0
RPL11	0.2192547	0.229084481	-0.00982978	-0.05218411	0.067673312	0.107997912	11	0	0
ILF2	0.0708938	0.065307584	0.005586173	0.089199651	0.067670908	0.107997912	13	0	0
MAP3K7	0.2115809	0.233847156	-0.02226623	-0.06053358	0.067759316	0.108125542	11	0	0
POLR1B	0.1590475	0.179019532	-0.01997198	-0.04315031	0.067820494	0.108194289	13	0	0
ACOT2	0.859894	0.851941985	0.007951999	0.015429302	0.067817832	0.108194289	2	0	0
SCYL1	0.1694251	0.165513975	0.003911149	0.04806259	0.067846542	0.108226216	10	0	0
SLC11A2	0.3744967	0.40257495	-0.02807828	-0.07344511	0.067886882	0.108280935	10	0	0
DHX58	0.4840741	0.476182737	0.007891344	0.118799378	0.067969488	0.108403054	8	0	0
CD7	0.6203724	0.636658282	-0.01628586	-0.03728163	0.06801596	0.108467526	8	0	0
TIGD7	0.1507897	0.123973546	0.026816143	0.602321368	0.068027606	0.108476452	14	0	0
TRPM3	0.1906871	0.189427652	0.001259423	0.132728272	0.068046205	0.108496464	7	0	0
TTLL11	0.2314304	0.251340844	-0.01991044	-0.05822433	0.068054566	0.108500151	6	0	0
NAPSA	0.5652203	0.576727087	-0.01150679	-0.02978571	0.068088949	0.10854532	4	0	0
NPR1	0.0809225	0.070071976	0.010850554	0.16611509	0.068175643	0.108673866	11	0	0
H1FX	0.0409009	0.037602366	0.003298503	0.088984804	0.068192773	0.108691512	9	0	0
TCN2	0.1179979	0.08298432	0.035013569	0.521206717	0.068200498	0.108694164	13	0	0
METTL13	0.0587189	0.056006759	0.002712155	0.034881353	0.068242455	0.10875137	6	0	0
RCCD1	0.3451389	0.351418226	-0.00627929	0.120779277	0.068271688	0.10878829	10	0	0
MPHOSPH6	0.1363206	0.139540363	-0.00321979	-0.04520077	0.06829489	0.108815595	9	0	0
ERAL1	0.1151394	0.118651758	-0.00351241	-0.02039432	0.06842177	0.109008071	13	0	0
TCF23	0.6328779	0.632251291	0.000626592	0.010622296	0.06843162	0.109014081	9	0	0
DUSP15	0.1068845	0.098749052	0.008135468	0.317967581	0.068465498	0.109058364	12	0	0

COMT	0.208933	0.2248994	-0.01596645	-0.08628104	0.068471745	0.10905863	13	0	0
CNTLN	0.1558171	0.132721625	0.023095432	0.201249592	0.068491927	0.109081087	7	0	0
DMGDH	0.0461864	0.049714428	-0.00352802	-0.08002304	0.068536768	0.109113747	9	0	0
MAPK14	0.1511649	0.14877532	0.002389547	-0.00803632	0.068534008	0.109113747	14	0	0
ZNF564	0.1569447	0.172434393	-0.01548973	-0.03023281	0.068526869	0.109113747	13	0	0
CD40	0.2133708	0.215912933	-0.00254215	0.087289582	0.068519977	0.109113747	7	0	0
ESYT2	0.0359477	0.032024712	0.003923033	0.102799577	0.068556836	0.10913601	5	0	0
SPAG16	0.1131189	0.11456311	-0.00144418	0.0584943	0.068595307	0.109160895	12	0	0
OBSL1	0.3884333	0.385835575	0.002597677	0.036626351	0.068596813	0.109160895	9	0	0
SCN5A	0.0732933	0.072537549	0.000755736	0.052678398	0.068592265	0.109160895	6	0	0
CD3E	0.6205855	0.640711511	-0.02012605	-0.06855982	0.068585259	0.109160895	8	0	0
CWF19L2	0.1411169	0.147256502	-0.00613964	0.033969675	0.068651851	0.109219407	7	0	0
ILF3	0.0427799	0.040849224	0.001930694	0.009243148	0.068649094	0.109219407	10	0	0
RILPL2	0.1216694	0.136262503	-0.01459311	-0.06031492	0.06866449	0.109229826	10	0	0
SMU1	0.0780061	0.073389085	0.004616992	0.067817125	0.068677342	0.109240583	5	0	0
LYRM2	0.2243376	0.244714762	-0.02037712	-0.0684197	0.068704518	0.109261695	11	0	0
EYA4	0.2980326	0.265283422	0.032749178	0.30836012	0.068698671	0.109261695	19	0	0
PBX3	0.0572003	0.059315369	-0.00211509	-0.00297445	0.06870889	0.109261695	8	0	0
NLN	0.1297055	0.130287964	-0.00058246	0.044343363	0.068724284	0.109266798	9	0	0
ZKSCAN5	0.2267731	0.237943435	-0.01117032	0.017243929	0.068720631	0.109266798	17	0	0
CTXN1	0.3086701	0.326545163	-0.01787504	-0.06380011	0.068739517	0.109281331	10	0	0
ACOT11	0.1791763	0.176799389	0.002376925	0.030406404	0.068756294	0.109298316	5	0	0
EPHA2	0.24383	0.245324973	-0.00149498	0.077547954	0.068771334	0.109312535	10	0	0
NSMF	0.3614582	0.373508179	-0.01205001	-0.03928038	0.06877912	0.109315223	5	0	0
NGLY1	0.1065294	0.108935327	-0.00240593	-0.03356813	0.068852654	0.1094224	13	0	0
LIG3	0.265259	0.278154418	-0.01289545	-0.01942414	0.068957079	0.109578645	12	0	0
TMCO3	0.1271428	0.113743437	0.013399343	0.087707916	0.06898943	0.109615476	9	0	0
SGK2	0.6874051	0.718719666	-0.0313146	-0.06277004	0.068992479	0.109615476	7	0	0
FUT5	0.0527808	0.057160803	-0.00438004	-0.03194824	0.06902276	0.109634451	15	0	0
NDUFA11	0.0527808	0.057160803	-0.00438004	-0.03194824	0.06902276	0.109634451	15	0	0
VMAC	0.0527808	0.057160803	-0.00438004	-0.03194824	0.06902276	0.109634451	15	0	0
RAG1	0.0714729	0.078781365	-0.00730843	-0.0888343	0.069134125	0.109801617	17	0	0
GGPS1	0.0630088	0.072347911	-0.00933913	-0.07429279	0.069154139	0.109823679	11	0	0
PSMG3	0.2801594	0.30649857	-0.02633916	-0.09287049	0.069168482	0.109832005	4	0	0
ZWILCH	0.0471236	0.04369699	0.003426578	0.04647605	0.069171628	0.109832005	11	0	0
NCKIPSD	0.341896	0.349762792	-0.00786684	-0.00262568	0.069192951	0.109856135	6	0	0
HTR1E	0.2025923	0.208533799	-0.00594147	0.095403475	0.069267131	0.109964177	9	0	0
MED16	0.5675785	0.583208855	-0.01563035	-0.03701072	0.06931017	0.110022764	6	0	0
KPNA2	0.1704063	0.177733631	-0.0073273	0.006884723	0.069328974	0.110042874	11	0	0
MYOZ3	0.6480649	0.64891673	-0.00085184	0.025124746	0.069345545	0.110049699	5	0	0
C9orf64	0.3021863	0.321190273	-0.01900398	0.190916036	0.069345264	0.110049699	9	0	0
ST3GAL1	0.0654225	0.071903531	-0.00648098	0.036144262	0.069355043	0.110055035	11	0	0
COMMD3	0.1937835	0.207741909	-0.01395843	-0.03097737	0.06936793	0.110059545	8	0	0
KRTAP10-12	0.6751892	0.698103833	-0.02291462	-0.04538958	0.069370158	0.110059545	5	0	0
ZNF443	0.4687743	0.498757083	-0.02998283	-0.08763139	0.069382659	0.110069642	1	0	0
ADAM10	0.0908824	0.082683086	0.008199352	0.091028285	0.069415029	0.110111255	10	0	0
DVL2	0.2276515	0.246070867	-0.01841932	-0.0428658	0.069448269	0.11015424	11	0	0
BEST4	0.7581282	0.761093738	-0.0029655	-0.00039641	0.069503577	0.110213319	6	0	0
THBS4	0.1027712	0.111618652	-0.00884745	-0.10204569	0.069497801	0.110213319	7	0	0
TAF6L	0.0573713	0.057451731	-8.05E-05	0.027940365	0.069505385	0.110213319	14	0	0
ADPRHL1	0.806348	0.829132232	-0.02278424	-0.04009205	0.06951624	0.110213319	16	0	0
CDKN3	0.2749982	0.275928786	-0.00093059	0.013578751	0.069516183	0.110213319	8	0	0
C1orf194	0.07855	0.066959038	0.011590927	0.351457261	0.069563439	0.110278403	7	0	0
TICRR	0.1370901	0.140366627	-0.00327655	-0.0404008	0.069610693	0.11034356	9	0	0
RPE	0.4499528	0.459172604	-0.00921983	-0.01899071	0.069637352	0.11036854	7	0	0
ACSL5	0.736682	0.772406528	-0.03572456	-0.06937196	0.069638758	0.11036854	6	0	0
MAGI1	0.0696494	0.06649706	0.003152375	0.104644349	0.069670456	0.110409022	13	0	0
VTI1B	0.3026682	0.312265595	-0.00959742	-0.02048303	0.069681052	0.110416056	8	0	0
DUSP23	0.0906012	0.087021412	0.003579825	0.065655785	0.069699425	0.110435414	8	0	0
EVI5L	0.0459419	0.04850439	-0.00256245	-0.02873186	0.069727769	0.110470565	10	0	0
CARM1	0.0818505	0.074401023	0.007449432	0.104362183	0.069758935	0.11051018	3	0	0
AP5Z1	0.1264664	0.138916886	-0.01245047	-0.03039152	0.069799139	0.110564105	10	0	0
PQLC2	0.0914446	0.080899416	0.0105452	0.064971476	0.069806502	0.110566003	6	0	0

EID2	0.1533349	0.166507007	-0.01317209	-0.02272876	0.069823494	0.110583151	9	0	0
SPA17	0.2086352	0.2063931	0.002242068	0.029978717	0.069870794	0.110648293	11	0	0
CACTIN	0.1306721	0.137643171	-0.00697111	-0.0146707	0.069894546	0.110676134	8	0	0
PDE1B	0.210028	0.187255769	0.022772215	0.341451867	0.069925029	0.110714629	10	0	0
MIEN1	0.2725559	0.283225554	-0.01066961	-0.00777788	0.069996647	0.110818241	10	0	0
F12	0.7437483	0.774901577	-0.03115328	-0.06552913	0.07002359	0.110851112	4	0	0
OAZ1	0.2888009	0.297356886	-0.00855595	-0.00067564	0.070040704	0.11086842	9	0	0
AK1	0.3197883	0.288091069	0.031697193	0.148413835	0.07007565	0.110913948	12	0	0
TGFB2	0.0931475	0.06641648	0.026730993	0.401308501	0.070104436	0.110949122	14	0	0
RHBDL3	0.1274364	0.107750633	0.01968574	0.427822349	0.070110244	0.110949122	10	0	0
SPTBN1	0.0813989	0.066193805	0.015205104	0.18757119	0.070185216	0.111055285	8	0	0
PKN1	0.2079926	0.206452573	0.001540007	0.091373494	0.070189714	0.111055285	8	0	0
FXYD6-FXYD	0.2287539	0.211986715	0.016767218	0.117325229	0.070218484	0.111091006	16	0	0
FOLR3	0.8825565	0.902655886	-0.02009939	-0.03212752	0.070246342	0.111125277	1	0	0
GABBR1	0.4425066	0.463590548	-0.02108395	-0.04147717	0.070255119	0.11112936	6	0	0
RPRD1B	0.2642666	0.281743977	-0.01747739	-0.02518194	0.070272233	0.111146628	11	0	0
NDUFA10	0.249546	0.260813522	-0.01126752	-0.05785896	0.070325295	0.111220746	4	0	0
BSND	0.6437613	0.668356637	-0.02459533	-0.047963	0.070334097	0.11122486	6	0	0
LRRC37B	0.1318293	0.141969244	-0.01013996	-0.08107344	0.070350284	0.11124065	5	0	0
ASPM	0.1187905	0.12620252	-0.00741207	-0.04244379	0.070414921	0.111333042	12	0	0
ASZ1	0.7382857	0.768688229	-0.03040252	-0.05977303	0.070424872	0.111335762	6	0	0
AMN	0.6413573	0.653633379	-0.01227608	-0.0166743	0.070434139	0.111335762	3	0	0
NAT14	0.2699928	0.28088857	-0.01089578	-0.04228754	0.070435264	0.111335762	8	0	0
TCERG1	0.1907248	0.193910787	-0.00318599	-0.04212727	0.070553485	0.111493155	12	0	0
FAM160B2	0.6733557	0.680187228	-0.00683153	-0.00155408	0.07055053	0.111493155	4	0	0
PALM2	0.0805402	0.083498297	-0.00295808	0.017935833	0.070543949	0.111493155	9	0	0
CCDC137	0.2021883	0.212722122	-0.01053387	-0.03089239	0.070616992	0.111583681	13	0	0
DDX23	0.0535244	0.056209124	-0.00268474	-0.04767252	0.070652147	0.111629397	5	0	0
CDYL	0.8140635	0.835849124	-0.02178565	-0.0372866	0.070673879	0.11164857	3	0	0
CSRNP2	0.3186599	0.330048956	-0.01138902	-7.63E-05	0.070676732	0.11164857	11	0	0
RBM43	0.2361294	0.231061493	0.005067921	0.115181203	0.070692032	0.111662904	9	0	0
UCN2	0.6656555	0.67521539	-0.00955984	-0.02214381	0.070735067	0.111718268	7	0	0
PEBP1	0.1890658	0.178993348	0.010072413	0.47712542	0.070739539	0.111718268	13	0	0
FIGNL1	0.2017924	0.220260763	-0.01846838	-0.07490532	0.07076708	0.111742907	8	0	0
MSANTD3	0.032011	0.030936276	0.001074674	0.035920569	0.0707676	0.111742907	6	0	0
UBE2K	0.0367282	0.035270458	0.001457722	0.046838287	0.070778626	0.111750479	10	0	0
ZNF92	0.3216667	0.326976744	-0.00531002	-0.00923478	0.07081409	0.11179663	6	0	0
MTX3	0.2801147	0.289527864	-0.00941315	-0.08341438	0.070857669	0.11184574	9	0	0
KCNH2	0.0701889	0.063259316	0.006929617	0.141503527	0.070915452	0.111927098	7	0	0
DLD	0.1803496	0.192414457	-0.01206485	-0.00291242	0.070922394	0.111928205	12	0	0
TEAD3	0.0916619	0.086951561	0.004710345	0.112873959	0.070959902	0.111967696	9	0	0
FUT11	0.3589076	0.342871134	0.016036418	0.057259682	0.070957201	0.111967696	10	0	0
RGS13	0.7331181	0.746695143	-0.01357707	-0.02770711	0.070973362	0.111979083	5	0	0
PCDHA11	0.2073612	0.179687397	0.027673846	0.171096066	0.071020311	0.112043302	5	0	0
UPB1	0.5333813	0.497133421	0.036247858	0.127059945	0.071040533	0.112065347	2	0	0
CBX6	0.0897013	0.070096878	0.019604424	0.340440463	0.071069622	0.112101375	5	0	0
CMTM1	0.9148707	0.922147473	-0.00727674	-0.01134337	0.071218485	0.112326305	15	0	0
IGF1R	0.0650443	0.061069476	0.003974803	0.045563007	0.071255548	0.11237488	5	0	0
ANKMY1	0.0744074	0.077246231	-0.00283884	-0.06598504	0.071279734	0.11240314	2	0	0
DPEP1	0.7034577	0.710357296	-0.00689963	-0.00210853	0.071367027	0.1125309	5	0	0
MYO18B	0.7459602	0.747686865	-0.00172663	0.004847426	0.071491358	0.112717036	12	0	0
GPR18	0.8660288	0.875012819	-0.00898405	-0.01538816	0.071503416	0.112726138	4	0	0
FANCI	0.1808607	0.197047947	-0.0161872	-0.08522846	0.071530073	0.112758253	9	0	0
SLC35A4	0.2469665	0.238973524	0.007992944	0.034809326	0.07158774	0.11283619	13	0	0
ARL3	0.1074858	0.111906138	-0.00442038	-0.0406318	0.071592097	0.11283619	11	0	0
TOB1	0.517685	0.519359348	-0.00167433	0.002292288	0.071612996	0.112859213	6	0	0
TM7SF3	0.1023897	0.110502621	-0.00811295	-0.06624944	0.071662516	0.112927331	10	0	0
ELL2	0.1391793	0.136978339	0.002200963	0.02286977	0.071681459	0.112932543	10	0	0
ZNF720	0.2915324	0.307548794	-0.01601639	-0.04978351	0.071690012	0.112932543	13	0	0
BCAT2	0.0246814	0.023585235	0.001096125	0.044048735	0.071691009	0.112932543	3	0	0
UQCC1	0.2324261	0.252984883	-0.02055877	-0.04002376	0.07169823	0.112934	12	0	0
LSM12	0.8313938	0.842469638	-0.01107583	-0.01936478	0.071734645	0.112981435	6	0	0
EQTN	0.7973592	0.826199217	-0.02884006	-0.05077175	0.071755051	0.113003651	2	0	0

ALDH6A1	0.0465887	0.044413418	0.002175266	0.068591067	0.071781955	0.113026174	9	0	0
MTFMT	0.2907008	0.30588723	-0.01518646	-0.02646619	0.071779637	0.113026174	12	0	0
PYGO1	0.4156227	0.396079473	0.019543242	0.268614399	0.071865228	0.113147361	11	0	0
RWDD4	0.1000059	0.095406454	0.004599399	0.066916688	0.071879782	0.11315041	6	0	0
RPL13A	0.2146927	0.217957909	-0.00326518	0.003079759	0.071874894	0.11315041	9	0	0
POLM	0.3082574	0.314292835	-0.00603548	0.011333478	0.071976351	0.113292481	9	0	0
HSF2	0.1627059	0.175791735	-0.01308583	-0.0456404	0.071997469	0.113315777	9	0	0
WIZ	0.2757721	0.260611677	0.015160394	0.074497865	0.07202464	0.113348596	9	0	0
RNF40	0.2254826	0.240606426	-0.01512384	-0.05336107	0.072062882	0.113398829	14	0	0
AHCTF1	0.3355688	0.366246445	-0.03067769	-0.07292094	0.072134173	0.113493797	4	0	0
ZNF503	0.0493607	0.044852189	0.00450851	0.101957126	0.072135888	0.113493797	10	0	0
ZNF180	0.1220781	0.132087322	-0.01000923	-0.00044727	0.072150409	0.113506687	10	0	0
ZMYND10	0.5765124	0.570319493	0.006192885	0.052178115	0.072158549	0.113509536	3	0	0
ALDH1L2	0.3257977	0.335442644	-0.00964498	0.12166339	0.072165626	0.113510714	13	0	0
MYLK	0.133347	0.123782531	0.009564429	0.361676342	0.072222292	0.113584393	11	0	0
GPR151	0.5044967	0.500207817	0.004288847	0.035269755	0.072225134	0.113584393	5	0	0
ASB1	0.3464655	0.348210795	-0.00174531	-0.03023835	0.072265741	0.113620252	7	0	0
TBC1D15	0.2018892	0.218623871	-0.01673462	-0.09230359	0.07226694	0.113620252	12	0	0
KIAA0586	0.0515013	0.047064806	0.004436515	0.065776501	0.072261554	0.113620252	20	0	0
ZNF766	0.3061156	0.313984355	-0.00786875	0.012166044	0.072291971	0.113649644	9	0	0
PER3	0.0957765	0.080738009	0.015038496	0.078873198	0.072331026	0.113691687	5	0	0
GPR3	0.0788721	0.075891887	0.002980244	0.097471133	0.07233773	0.113691687	11	0	0
ATP2A1	0.6084785	0.62965923	-0.02118071	-0.04322689	0.07233226	0.113691687	7	0	0
ACAD11	0.0683257	0.07961365	-0.01128799	-0.01100907	0.072363889	0.113722835	7	0	0
ACAA1	0.0476259	0.046552002	0.001073915	0.033100873	0.072401186	0.113753779	13	0	0
FAIM	0.1428089	0.148335331	-0.00552646	0.112855066	0.072394649	0.113753779	13	0	0
TFDP2	0.4733898	0.49176701	-0.0183772	-0.0320399	0.072402606	0.113753779	7	0	0
HDAC9	0.147254	0.132990337	0.014263639	0.055747358	0.072420947	0.11377263	8	0	0
BCL3	0.0892068	0.086495842	0.002710937	0.013256091	0.072430021	0.113776918	5	0	0
FH	0.1020764	0.115438125	-0.01336174	-0.05898882	0.072480049	0.113845535	12	0	0
HMOX2	0.0749818	0.078946332	-0.00396456	0.139788965	0.072486609	0.113845869	9	0	0
AMMECR1L	0.2738037	0.304517271	-0.03071358	-0.10473776	0.072538286	0.113917056	2	0	0
LARS	0.0508975	0.048686061	0.002211456	0.054312464	0.072562028	0.11393439	9	0	0
SLC12A9	0.1664135	0.143810275	0.022603228	0.132750536	0.072558518	0.11393439	4	0	0
EDEM1	0.0550913	0.054345561	0.000745784	0.00708313	0.072598822	0.113976436	8	0	0
ZDHHC23	0.1698446	0.18850072	-0.01865615	-0.02952037	0.07260524	0.113976436	7	0	0
CBR4	0.0469712	0.049381993	-0.00241082	-0.01794098	0.07260787	0.113976436	14	0	0
CDC42EP2	0.1555961	0.148882934	0.006713188	0.076854938	0.072628459	0.113998778	6	0	0
AKR1C4	0.7129172	0.738539916	-0.02562275	-0.05195129	0.072644368	0.114013772	6	0	0
GTF2F2	0.0412437	0.04084078	0.000402884	0.020784145	0.072664997	0.11403617	11	0	0
LTB4R2	0.8231131	0.837513232	-0.01440016	-0.02480389	0.072720429	0.114113177	3	0	0
SLC5A1	0.4988525	0.51093241	-0.01207995	-0.02702239	0.07275583	0.11415874	8	0	0
TSPAN2	0.0452463	0.034764657	0.010481643	0.322595811	0.072778471	0.114184277	8	0	0
SETMAR	0.2735966	0.292691254	-0.01909466	-0.06038486	0.072846351	0.114267847	14	0	0
GRK4	0.0736184	0.082777347	-0.00915892	-0.08943916	0.072838249	0.114267847	10	0	0
TMCO6	0.0444669	0.03853574	0.005931117	0.08031572	0.072850849	0.114267847	8	0	0
UBA6	0.1109171	0.122838524	-0.0119214	-0.00297334	0.072880722	0.114303302	10	0	0
NOL4	0.0691673	0.055637639	0.013529667	0.28120015	0.072886199	0.114303302	3	0	0
KIAA0355	0.3325258	0.339855253	-0.0073295	-0.0012897	0.072934284	0.114368711	4	0	0
TARS	0.1650595	0.192975011	-0.02791546	-0.0699369	0.072948233	0.1143793	11	0	0
SLC35A1	0.9009518	0.911801667	-0.01084987	-0.01708166	0.072953791	0.1143793	1	0	0
METRNL	0.1895742	0.203864589	-0.01429043	0.038868422	0.072983203	0.114415412	6	0	0
NCAPD3	0.1092631	0.108789224	0.000473846	0.02749298	0.07300614	0.114441368	6	0	0
KIAA1211L	0.0863766	0.063674721	0.022701866	0.450835266	0.07301938	0.11445212	9	0	0
ATXN2	0.0550055	0.052338635	0.002666858	0.032873639	0.073035998	0.114468163	11	0	0
LHFPL5	0.2812382	0.287185897	-0.00594766	0.027662575	0.073070266	0.114511864	11	0	0
CFDP1	0.1120557	0.120543419	-0.00848777	-0.04808344	0.07311325	0.114569216	6	0	0
SYDE2	0.0571385	0.057135575	2.96E-06	0.002031119	0.07316548	0.114641045	8	0	0
POLH	0.1243269	0.130329573	-0.00600264	-0.01909268	0.073214854	0.114708387	10	0	0
FGFR1	0.3295435	0.31768286	0.01186062	0.211600408	0.073277041	0.114795791	5	0	0
TTC4	0.2498138	0.255574112	-0.00576036	-0.04139587	0.073288326	0.114798415	4	0	0
SLC8B1	0.5848131	0.566393478	0.018419639	0.03866562	0.073291517	0.114798415	3	0	0
FAM32A	0.0401168	0.040427115	-0.00031031	0.000434686	0.073302652	0.11480583	12	0	0

TRIM62	0.2620924	0.281297367	-0.01920497	-0.02617944	0.073346877	0.114865065	11	0	0
CENPB	0.0833739	0.095341215	-0.01196733	-0.12838501	0.073399039	0.114936717	3	0	0
GAK	0.0989994	0.09692921	0.002070202	-0.00536131	0.073426637	0.114969896	11	0	0
CDH16	0.4667679	0.48071327	-0.0139454	-0.05795628	0.073435647	0.114973966	5	0	0
TRPC3	0.1427722	0.121141629	0.021630582	0.359228289	0.073472998	0.115022405	6	0	0
CALM2	0.0935667	0.103231541	-0.00966485	0.004201781	0.073518159	0.115075525	12	0	0
RAD9B	0.0757124	0.083788733	-0.00807633	-6.80E-05	0.073519761	0.115075525	13	0	0
PDIA5	0.1961141	0.201567896	-0.00545383	4.92E-05	0.073538236	0.115094399	11	0	0
SKIV2L	0.13751	0.148715336	-0.01120537	-0.04726401	0.073573904	0.115125651	19	0	0
CYTH3	0.1835571	0.200181607	-0.01662452	-0.02133528	0.073577461	0.115125651	6	0	0
PLIN4	0.6061457	0.630153263	-0.02400751	-0.04364159	0.073568973	0.115125651	10	0	0
HORMAD1	0.8786815	0.891741449	-0.01305993	-0.02242881	0.073609345	0.115128993	9	0	0
IQGAP3	0.2739349	0.28763413	-0.01369926	-0.02806828	0.073611691	0.115128993	10	0	0
ARFIP1	0.0691666	0.073761572	-0.00459496	0.017595964	0.073601717	0.115128993	8	0	0
DHCR7	0.0617478	0.062904465	-0.00115666	0.069747341	0.073598052	0.115128993	9	0	0
LOXL1	0.4683676	0.477418664	-0.00905103	-0.00753275	0.073603352	0.115128993	9	0	0
NPC1L1	0.7679036	0.756297808	0.011605802	0.023487629	0.073627352	0.115143447	10	0	0
ADCY6	0.0665916	0.064148579	0.002442984	0.086194406	0.073683556	0.115211251	10	0	0
ZNF581	0.2611628	0.265101446	-0.00393864	0.031448178	0.073692251	0.115214803	3	0	0
PROS1	0.2444187	0.260460488	-0.01604177	-0.01840575	0.07370506	0.115224785	7	0	0
MOB4	0.2297364	0.223101342	0.006635051	0.101861836	0.073754275	0.115285463	17	0	0
SUSD2	0.4939704	0.501801165	-0.00783076	-0.0300886	0.073756729	0.115285463	7	0	0
HES1	0.0587457	0.059744441	-0.00099879	-0.0247943	0.07377635	0.115306083	24	0	0
ANGPTL3	0.8081784	0.822291328	-0.01411295	-0.02537405	0.073806306	0.115332802	5	0	0
ATP6V1E1	0.1758826	0.168683878	0.007198715	0.052443377	0.073802035	0.115332802	8	0	0
XKR9	0.0957712	0.106962786	-0.0111916	-0.06929877	0.073855501	0.11538957	16	0	0
CDC37L1	0.0515796	0.049713502	0.001866065	0.03823558	0.07385077	0.11538957	11	0	0
SHARPIN	0.7176788	0.729023386	-0.01134459	-0.01923371	0.073865339	0.11539489	7	0	0
VGLL3	0.1278168	0.128844676	-0.00102789	0.101510832	0.073916165	0.115454179	11	0	0
PCTP	0.2731567	0.279170478	-0.0060138	0.11964463	0.073913835	0.115454179	11	0	0
RCBTB2	0.4272857	0.435475389	-0.00818967	0.016822837	0.073935038	0.115473602	9	0	0
YTHDF1	0.1080598	0.11416612	-0.00610633	0.017417995	0.073975361	0.11552652	9	0	0
AUNIP	0.1998679	0.20104069	-0.00117277	0.026185473	0.074027415	0.115577622	6	0	0
TRMT12	0.2643051	0.281912086	-0.01760698	0.096911449	0.074023572	0.115577622	6	0	0
TRPM7	0.0970625	0.105285168	-0.00822266	-0.06738519	0.074022876	0.115577622	8	0	0
DCAF5	0.0916176	0.072649861	0.018967751	0.185055758	0.074034717	0.115578961	9	0	0
DBT	0.0991365	0.099258901	-0.00012242	0.02030007	0.074064218	0.11560362	10	0	0
IDE	0.0654581	0.074227677	-0.00876954	-0.02229653	0.074067406	0.11560362	12	0	0
PIGU	0.1082976	0.112943117	-0.00464551	-0.01297088	0.074069849	0.11560362	7	0	0
KIAA0556	0.0782016	0.076634537	0.001567103	0.03035839	0.07407929	0.115608296	13	0	0
DRG1	0.2807146	0.29898368	-0.01826907	-0.01939002	0.074109842	0.115645913	8	0	0
OR2H2	0.6027115	0.627151476	-0.02443997	-0.05354634	0.074153127	0.115693327	4	0	0
WDR59	0.1427188	0.120178072	0.02254074	0.190236933	0.074148722	0.115693327	3	0	0
IFRD2	0.26021	0.254193172	0.006016779	0.031912843	0.074205725	0.115765321	11	0	0
ZNF215	0.2006948	0.182567803	0.018126975	0.439319409	0.074212879	0.115766411	8	0	0
ZNF629	0.0963976	0.085140159	0.011257413	0.095925689	0.074247226	0.115809918	11	0	0
EPG5	0.1285104	0.139558038	-0.01104763	-0.04223302	0.074288899	0.115864843	10	0	0
GARS	0.1806564	0.190256275	-0.00959989	-0.05717361	0.074318601	0.11590109	8	0	0
ERGIC3	0.130041	0.123236117	0.006804864	0.084390346	0.074345368	0.115932753	12	0	0
ARL6IP5	0.1741412	0.18264376	-0.00850253	-0.0302313	0.074357231	0.115941172	10	0	0
TIPRL	0.1952243	0.205701654	-0.01047737	-0.01369066	0.074377969	0.115960309	10	0	0
CALCOCO1	0.1739901	0.167174451	0.006815601	0.011423667	0.074382435	0.115960309	10	0	0
TP53INP1	0.1567442	0.171736596	-0.01499241	-0.10509715	0.074404049	0.115983924	12	0	0
PAN2	0.1263857	0.118171458	0.008214248	0.330539604	0.074436982	0.116025176	16	0	0
TRIO	0.0634432	0.054989311	0.008453921	0.083624998	0.074459584	0.116050322	6	0	0
EFNB3	0.1090432	0.105160419	0.003882776	0.067870479	0.074554814	0.116188647	9	0	0
TMEM201	0.2504755	0.258255404	-0.0077799	0.005941346	0.074591804	0.116227904	8	0	0
RAB8B	0.0586575	0.061249075	-0.00259154	0.037929155	0.074592964	0.116227904	6	0	0
SCPEP1	0.1336586	0.138030085	-0.00437145	0.013155794	0.074611253	0.116246303	8	0	0
CES3	0.4110113	0.431755352	-0.0207441	-0.03876161	0.074646784	0.116291558	6	0	0
THEM4	0.2698012	0.240150301	0.029650853	0.554845707	0.074660737	0.116293094	13	0	0
FAM53C	0.0895425	0.093747625	-0.00420511	-0.03333518	0.074654513	0.116293094	15	0	0
EFCAB13	0.251542	0.256355091	-0.00481309	0.165156128	0.074689439	0.116327698	8	0	0

PPM1H	0.0803252	0.086063359	-0.00573812	0.020873749	0.074701759	0.116336137	10	0	0
FAN1	0.0497031	0.056518928	-0.0068158	-0.04672325	0.074707829	0.116336137	12	0	0
ZNF35	0.2528037	0.2413889	0.011414823	0.159595368	0.074738225	0.116373366	11	0	0
ZNF287	0.1175391	0.126326178	-0.00878708	-0.02439423	0.07474801	0.116378498	12	0	0
CYP4F22	0.2563968	0.251541241	0.004855547	0.223459568	0.074829989	0.116496024	8	0	0
CDC42SE1	0.047084	0.042181017	0.004902979	0.141155313	0.074879831	0.116552842	13	0	0
MYO5A	0.1662177	0.165689453	0.0005282	-0.00868525	0.07488008	0.116552842	12	0	0
KRT39	0.8699673	0.886364411	-0.01639709	-0.02711317	0.074885981	0.116552842	3	0	0
CORO6	0.3100968	0.284804024	0.025292808	0.306694934	0.074925847	0.116604772	8	0	0
ALDH9A1	0.172454	0.182251568	-0.00979759	-0.00556527	0.075011274	0.11672759	11	0	0
TACSTD2	0.2472751	0.243028479	0.004246582	0.079849263	0.075054049	0.116784022	13	0	0
CENPE	0.06602	0.074924415	-0.00890437	-0.05549761	0.075159018	0.116937208	11	0	0
ZNF713	0.205777	0.20888421	-0.00310719	-0.01896007	0.075172221	0.116947606	5	0	0
PRKRA	0.1384926	0.146250171	-0.00775754	0.025728384	0.075182004	0.116952681	11	0	0
ZNF827	0.1137443	0.127433209	-0.0136889	-0.04051918	0.075269781	0.117079074	11	0	0
B4GALT7	0.0754626	0.084638309	-0.00917572	-0.05037329	0.075335291	0.117158634	7	0	0
RFX6	0.2267211	0.22037995	0.006341155	0.186609181	0.075331233	0.117158634	11	0	0
ZNF607	0.1935761	0.205529776	-0.01195366	0.034533572	0.075340526	0.117158634	10	0	0
C2CD5	0.176752	0.184795201	-0.0080432	-0.06215753	0.07535587	0.11716865	14	0	0
CTC1	0.0417111	0.038293386	0.003417714	0.055235091	0.075360032	0.11716865	15	0	0
GAD1	0.3336383	0.29018354	0.043454809	0.337246172	0.075403224	0.117225641	2	0	0
EPM2AIP1	0.0412048	0.030064256	0.011140578	0.32662504	0.075417772	0.117238097	24	0	0
MT1X	0.115807	0.121160551	-0.00535352	-0.05860028	0.075446453	0.117262355	8	0	0
UCKL1	0.1444243	0.14993286	-0.00550861	-0.03941224	0.075441832	0.117262355	4	0	0
SCAMP1	0.1602359	0.170692503	-0.01045665	-0.02653994	0.075565383	0.117437025	6	0	0
ELF5	0.665337	0.682911887	-0.01757489	-0.03268391	0.075579119	0.117445001	14	0	0
NFIC	0.0919219	0.095728465	-0.0038066	0.058260599	0.075583611	0.117445001	6	0	0
NOP10	0.1323555	0.134098801	-0.00174331	0.072048007	0.075616112	0.117475148	9	0	0
NUTM1	0.1323555	0.134098801	-0.00174331	0.072048007	0.075616112	0.117475148	9	0	0
TMEM42	0.0808968	0.08001597	0.000880861	0.104432326	0.075636454	0.117486399	7	0	0
TMEM39A	0.1737445	0.187249203	-0.01350469	-0.01219983	0.075634799	0.117486399	8	0	0
MCUR1	0.1400003	0.156386059	-0.01638573	-0.0623461	0.07565811	0.117489511	7	0	0
LATS1	0.1224599	0.136252081	-0.01379218	0.030951583	0.075657018	0.117489511	9	0	0
CMTM3	0.3546785	0.332780166	0.02189832	0.236228082	0.07565579	0.117489511	8	0	0
SCP2	0.3039124	0.304251503	-0.00033905	0.00708307	0.075680618	0.11751429	9	0	0
CLK1	0.2181543	0.221058881	-0.00290455	0.023228393	0.075809084	0.117703578	11	0	0
BCAS3	0.0405619	0.038306209	0.002255699	0.06647185	0.07590093	0.11783598	15	0	0
CD44	0.2372521	0.240683378	-0.00343128	0.065441448	0.075973292	0.117938113	12	0	0
FNBP4	0.0610589	0.051186271	0.009872585	0.154613258	0.075984985	0.117946057	11	0	0
CHRAC1	0.1861384	0.19001985	-0.00388149	0.008332413	0.076005852	0.117968238	11	0	0
LARP1	0.6583819	0.656220883	0.002161004	0.016100133	0.076040705	0.118012121	9	0	0
MRPL4	0.2878386	0.285535481	0.002303085	-0.00669457	0.076108518	0.118107146	11	0	0
RGS10	0.0861447	0.094143582	-0.00799891	0.070153105	0.076178876	0.118206101	3	0	0
DHX33	0.2466193	0.256225105	-0.0096058	-0.01274794	0.076197537	0.11822483	12	0	0
PHF13	0.0959471	0.098192119	-0.002245	0.070993456	0.076207956	0.118230767	9	0	0
FBXL18	0.2712479	0.289479361	-0.01823142	-0.03733221	0.076262952	0.118305856	5	0	0
TSPAN17	0.1962772	0.197198569	-0.0009214	0.005677219	0.076298028	0.118350032	12	0	0
ZNF44	0.3315602	0.306300094	0.025260115	0.215961754	0.076315391	0.118366728	8	0	0
ZMYM4	0.0411321	0.041961898	-0.00082979	-0.00133463	0.076366901	0.118430618	9	0	0
IFT172	0.1441786	0.153017288	-0.0088387	-0.00293637	0.076369789	0.118430618	16	0	0
GCAT	0.0508381	0.05267702	-0.00183892	-0.01597171	0.076417539	0.118494422	8	0	0
MDK	0.262307	0.275031348	-0.01272439	-0.02110051	0.076456298	0.118544274	7	0	0
BLOC1S5	0.1716973	0.173196796	-0.00149947	0.017863547	0.076519263	0.118631644	5	0	0
CILP2	0.3134569	0.311840751	0.001616112	0.126767576	0.076535409	0.118646421	10	0	0
ZNF253	0.2053192	0.213003856	-0.00768463	0.098978462	0.076562724	0.118668252	4	0	0
HSCB	0.0665771	0.067001011	-0.00042387	0.018378655	0.076560721	0.118668252	13	0	0
DOK7	0.1692609	0.169353921	-9.30E-05	0.187744653	0.076579655	0.118673984	9	0	0
PSMC3	0.1188717	0.120966101	-0.00209442	0.021351688	0.076577318	0.118673984	11	0	0
RNH1	0.2237473	0.219817522	0.003929762	0.06582986	0.076634019	0.118747971	12	0	0
NAP1L1	0.0569632	0.035173697	0.021789499	0.237045026	0.076720676	0.118871981	10	0	0
DLAT	0.2057284	0.214509087	-0.00878065	0.011279854	0.076738541	0.118889391	5	0	0
SCFD1	0.1164286	0.126584278	-0.0101557	-0.03495004	0.076753253	0.118901912	13	0	0
AK5	0.1559372	0.143347047	0.012590146	0.369924436	0.07679189	0.118951493	10	0	0

AIG1	0.0413455	0.032586971	0.008758556	0.236126827	0.076829545	0.118999544	7	0	0
ETFB	0.0381706	0.033123523	0.005047115	0.195751654	0.07686973	0.119051504	13	0	0
MINPP1	0.4210084	0.429368996	-0.00836063	0.007177171	0.07692122	0.119120964	11	0	0
NMU	0.1949794	0.173625504	0.021353886	0.272340514	0.07693197	0.119127325	16	0	0
HACL1	0.0501419	0.051008155	-0.0008663	0.028059205	0.076964026	0.119156389	16	0	0
PKIA	0.2018062	0.193129945	0.008676287	0.284278963	0.076961924	0.119156389	11	0	0
COMP	0.3240286	0.318651692	0.005376881	0.217601587	0.077005185	0.119209823	14	0	0
ZDBF2	0.1791576	0.155858916	0.023298695	0.31351437	0.077061866	0.119276979	14	0	0
WDR25	0.1127974	0.111847991	0.000949448	0.001672125	0.07706011	0.119276979	14	0	0
PPM1M	0.0926225	0.090606789	0.002015714	0.058613248	0.077139016	0.119375788	13	0	0
ASPH	0.2269905	0.241082129	-0.01409163	-0.04804729	0.077169996	0.119413428	13	0	0
LRPPRC	0.2121682	0.228435774	-0.01626759	-0.04854587	0.077264826	0.119549854	10	0	0
ERBB3	0.1719646	0.175195818	-0.0032312	-0.04091036	0.077273907	0.119553593	9	0	0
GUSB	0.315385	0.321725857	-0.00634083	-0.02259999	0.077300777	0.119584849	6	0	0
PGM2L1	0.0508019	0.046247984	0.004553957	0.104337261	0.077332522	0.11962364	10	0	0
MXD4	0.0482787	0.043770979	0.004507727	0.095037271	0.07737447	0.119678208	7	0	0
PPM1F	0.3924949	0.39918516	-0.00669027	-0.04546599	0.077383063	0.119681178	7	0	0
KSR2	0.2055156	0.222267398	-0.01675178	-0.03494658	0.077405137	0.119704995	5	0	0
RBM12B	0.0801422	0.09625062	-0.01610844	-0.03954717	0.077447404	0.119760035	12	0	0
CADM2	0.0960994	0.089363677	0.006735693	0.052691386	0.077483175	0.11980502	8	0	0
ZNF784	0.1015176	0.109831468	-0.0083139	-0.05267692	0.077531557	0.119869494	10	0	0
TMEM80	0.0374539	0.04291099	-0.00545713	-0.07993685	0.077543617	0.119877807	13	0	0
RAE1	0.4983356	0.523551704	-0.02521606	-0.0429674	0.077551394	0.119879496	8	0	0
PAQR6	0.566157	0.554997679	0.011159326	0.028330317	0.077593149	0.119933706	8	0	0
FAM151B	0.2977694	0.310393494	-0.01262409	0.021301074	0.077654202	0.120017731	12	0	0
CD47	0.3496619	0.336800322	0.012861605	0.112099051	0.077700472	0.120058206	10	0	0
ZNF212	0.2264215	0.24683606	-0.02041453	-0.05573845	0.077700108	0.120058206	11	0	0
TAS2R10	0.8225931	0.849556604	-0.02696348	-0.045981	0.077722666	0.120082156	1	0	0
BRD9	0.1090095	0.118881484	-0.009872	-0.04227653	0.077735583	0.120091767	13	0	0
CORO1C	0.3386888	0.345911146	-0.00722235	-0.00754569	0.077754917	0.12011129	8	0	0
IMPDH2	0.2913068	0.284508217	0.006798571	0.046480288	0.077776087	0.120133647	10	0	0
KCNK1	0.1111416	0.090383793	0.020757818	0.266312177	0.07778636	0.120134631	10	0	0
RPIA	0.0832428	0.096334343	-0.01309154	-0.04378689	0.077790121	0.120134631	16	0	0
PCDHB8	0.4722002	0.495903522	-0.02370336	-0.04925502	0.077803607	0.120145113	5	0	0
STON1	0.3924119	0.400525269	-0.00811337	0.029669689	0.07782122	0.120151845	9	0	0
TIMMDC1	0.2328968	0.224436652	0.008460111	0.01090797	0.077821364	0.120151845	13	0	0
PRPF38B	0.0886085	0.087352296	0.001256201	-0.01932832	0.077899917	0.120252423	12	0	0
RAI14	0.068068	0.056759167	0.011308857	0.180856201	0.077893475	0.120252423	11	0	0
TM9SF1	0.3334627	0.326655387	0.006807301	0.271709629	0.077926945	0.120283793	9	0	0
SIT1	0.6443436	0.671263671	-0.02692005	-0.05909309	0.077976389	0.120349755	6	0	0
LIMA1	0.2677565	0.283806778	-0.01605028	-0.0168403	0.078015712	0.120400087	9	0	0
OR6C76	0.7502922	0.749711641	0.000580557	-0.00362918	0.078025755	0.120405226	2	0	0
TTC5	0.2378572	0.244305604	-0.00644841	0.034775672	0.078044567	0.120423894	13	0	0
SNX8	0.057882	0.055477165	0.002404823	0.06427147	0.07814125	0.120557844	13	0	0
KIAA0895	0.0695185	0.075452724	-0.00593423	-0.041141	0.07814482	0.120557844	13	0	0
CCDC18	0.1000802	0.113044235	-0.01296405	-0.01692234	0.07815215	0.120558782	12	0	0
TANC1	0.1457901	0.163618704	-0.01782857	-0.03266827	0.078228298	0.120654443	8	0	0
TFG	0.1975297	0.201743223	-0.00421347	-0.05570051	0.078221107	0.120654443	12	0	0
AGFG2	0.0876302	0.099081731	-0.01145156	-0.02552981	0.078234342	0.120654443	8	0	0
MIPEP	0.0690864	0.079132768	-0.01004638	-0.03688473	0.078255403	0.120676546	8	0	0
TEKT1	0.2725572	0.269969314	0.002587844	0.143125484	0.078280457	0.120704804	10	0	0
SLC41A1	0.2908253	0.304726209	-0.01390089	0.06142875	0.078295633	0.120717826	3	0	0
IFFO2	0.0739479	0.085803201	-0.01185533	-0.07798285	0.078334045	0.12076667	5	0	0
TTLL7	0.3438684	0.360677248	-0.01680887	-0.00672996	0.078352433	0.120780517	5	0	0
JAZF1	0.4973137	0.496159566	0.001154138	0.0474439	0.078369963	0.120780517	9	0	0
UTP23	0.1922606	0.207728073	-0.01546746	-0.04932842	0.078367112	0.120780517	11	0	0
BAZ1A	0.1315194	0.121634098	0.009885264	0.134358928	0.078367956	0.120780517	6	0	0
ADRB2	0.0718771	0.064129435	0.007747687	0.140935072	0.078386465	0.120786168	6	0	0
ITGB2	0.1032569	0.067232516	0.036024369	0.666701708	0.078387098	0.120786168	5	0	0
BCAS4	0.1266991	0.09491243	0.031786698	0.29578303	0.078411228	0.12081297	11	0	0
ANKRD42	0.0700709	0.079498266	-0.00942741	-0.05772655	0.078439503	0.120846154	14	0	0
RICTOR	0.034514	0.033130257	0.001383697	0.012675909	0.07851463	0.120945606	10	0	0
ERMP1	0.3447123	0.344656694	5.56E-05	0.018753431	0.078517542	0.120945606	8	0	0

JPH1	0.1172305	0.119270533	-0.00203999	0.062097055	0.078527832	0.120951068	10	0	0
ACVR1B	0.1341081	0.139408583	-0.00530052	0.043096688	0.078555398	0.120983138	8	0	0
FGF2	0.2607608	0.239196315	0.021564494	0.141099043	0.078595296	0.121034192	8	0	0
ATL3	0.3053486	0.296341723	0.009006903	0.371390978	0.078618873	0.121060105	16	0	0
CD48	0.3752906	0.40360138	-0.02831078	-0.09378644	0.078657804	0.121104455	8	0	0
ACSS1	0.3690208	0.374452876	-0.0054321	0.034141201	0.078661178	0.121104455	9	0	0
STX7	0.1066741	0.116559733	-0.00988567	-0.01492699	0.078816522	0.121333202	10	0	0
TNNC2	0.143355	0.122931628	0.020423375	0.148321812	0.078828348	0.121340993	10	0	0
SDE2	0.143175	0.11702194	0.026153021	0.172048959	0.078868255	0.121362919	9	0	0
PPID	0.0372529	0.036873548	0.000379379	0.002438629	0.078858257	0.121362919	7	0	0
C14orf28	0.3482676	0.364108375	-0.01584077	-0.02558342	0.078859924	0.121362919	10	0	0
KDM8	0.2000402	0.205913455	-0.00587326	0.021518438	0.078875315	0.121362919	10	0	0
ASIP	0.688226	0.697030808	-0.00880479	-0.01707804	0.078876424	0.121362919	4	0	0
OOEP	0.7262011	0.787366396	-0.06116533	-0.11388012	0.078932227	0.121438362	5	0	0
SIX4	0.2982545	0.312354726	-0.01410026	-0.06319571	0.0790107	0.121548668	10	0	0
ABHD16A	0.0708666	0.068225164	0.002641436	0.063505666	0.07904488	0.121590821	27	0	0
PLA2G3	0.4521672	0.474149328	-0.02198217	-0.06161481	0.079080418	0.121635055	10	0	0
PLCD4	0.3789075	0.371616184	0.007291362	0.066931999	0.079121143	0.121687259	5	0	0
DDRGK1	0.3558399	0.371453906	-0.01561402	-0.01034103	0.079170914	0.121742928	10	0	0
CERK	0.2210647	0.23452526	-0.01346059	-0.05559432	0.07917004	0.121742928	5	0	0
ZNF512	0.3459226	0.337281976	0.008640612	0.196716482	0.079204134	0.12178357	18	0	0
EDC3	0.0562643	0.05301506	0.003249196	0.103852021	0.079222943	0.121802049	11	0	0
FKBP3	0.0568545	0.052826177	0.004028277	0.078340508	0.079279358	0.121878337	10	0	0
TMEM11	0.0906779	0.093058016	-0.00238013	-0.01713898	0.079291878	0.121887137	16	0	0
PRDM6	0.1465529	0.123503436	0.023049508	0.273936555	0.07930483	0.1218966	9	0	0
BDH1	0.7251977	0.74538856	-0.02019083	-0.03704225	0.079314916	0.121900502	2	0	0
EIF4H	0.0460089	0.053105372	-0.00709643	-0.08135784	0.079320961	0.121900502	9	0	0
PPP2R2A	0.3072075	0.316941811	-0.00973431	-0.0187596	0.079355764	0.121943539	6	0	0
LARS2	0.1400487	0.147014828	-0.00696614	-0.02772145	0.07939612	0.121995101	8	0	0
RNASEH1	0.225756	0.226421182	-0.00066514	0.013881432	0.079423795	0.12202717	11	0	0
IFT57	0.2401031	0.261258703	-0.02115562	-0.07578279	0.079433055	0.122030944	5	0	0
MLPH	0.8304993	0.813994993	0.016504341	0.029620204	0.079478332	0.122090044	2	0	0
PPP1R10	0.6827738	0.691679852	-0.0089061	-0.04466918	0.0796052	0.12227446	11	0	0
THAP2	0.0642866	0.067431948	-0.00314537	-0.05857355	0.079627351	0.122298011	6	0	0
ORC4	0.1882047	0.191443688	-0.00323902	0.004211338	0.079658255	0.122335	7	0	0
KBTBD7	0.1957235	0.209020554	-0.01329704	0.115149413	0.079704414	0.122395409	8	0	0
SFT2D2	0.0807523	0.09747868	-0.01672642	-0.0745445	0.079715837	0.12240247	8	0	0
ARVCF	0.0408801	0.044472303	-0.00359223	-0.02192908	0.079739339	0.122417596	7	0	0
TANGO2	0.0408801	0.044472303	-0.00359223	-0.02192908	0.079739339	0.122417596	7	0	0
SPAST	0.0664695	0.070175378	-0.00370584	0.020528369	0.07977407	0.122460434	6	0	0
C12orf66	0.1241964	0.127691966	-0.00349561	-0.06531943	0.079799963	0.1224897	10	0	0
FAM228B	0.05602	0.052774281	0.003245758	0.052828173	0.079825408	0.122507789	6	0	0
STOX1	0.1972397	0.202519976	-0.00528027	-0.04729718	0.079819302	0.122507789	10	0	0
ZNF514	0.0458544	0.047932041	-0.00207768	-0.05353261	0.079838375	0.1225092	14	0	0
RYK	0.308359	0.30605886	0.002300142	0.019314192	0.079845834	0.1225092	10	0	0
NFXL1	0.1273797	0.131734681	-0.00435493	-0.02641558	0.079851529	0.1225092	12	0	0
VPS18	0.2143489	0.222549835	-0.0082009	-0.00515051	0.079853649	0.1225092	13	0	0
PIAS4	0.0378372	0.035926141	0.001911021	0.064082064	0.079874586	0.12253084	6	0	0
SNAPC2	0.2316784	0.245520697	-0.0138423	-0.08567487	0.079918725	0.122586583	13	0	0
ZCCHC3	0.2055418	0.232828755	-0.02728691	-0.04734052	0.079924593	0.122586583	9	0	0
RSPH3	0.1519541	0.157211475	-0.00525734	-0.02288297	0.079964261	0.122636938	11	0	0
DDX21	0.0369688	0.036708184	0.00026064	0.019161496	0.080028031	0.122724244	13	0	0
POGLUT1	0.2673107	0.285661399	-0.01835073	-0.0440605	0.080040919	0.122733515	7	0	0
GNB1	0.0527119	0.050051573	0.002660312	0.086788191	0.080084649	0.122769919	6	0	0
HNRNPUL2	0.0396656	0.037842521	0.001823095	0.05249668	0.080085195	0.122769919	14	0	0
TTC9C	0.0396656	0.037842521	0.001823095	0.05249668	0.080085195	0.122769919	14	0	0
KNOP1	0.0532066	0.052155024	0.001051558	0.024155082	0.080128152	0.122825275	12	0	0
AKIP1	0.1031813	0.110663153	-0.00748189	0.006141854	0.080156465	0.122847677	14	0	0
MYO7A	0.2062936	0.179570769	0.026722877	0.27090467	0.08015258	0.122847677	10	0	0
GLO1	0.3188233	0.335005827	-0.01618256	-0.04078879	0.080184516	0.122880167	13	0	0
HAGHL	0.1983494	0.212222779	-0.01387339	-0.02777597	0.080196475	0.122887994	11	0	0
XPO1	0.0525566	0.06030339	-0.00774681	-0.07385494	0.080298438	0.123033725	9	0	0
MPST	0.1398511	0.128598523	0.011252569	0.035072352	0.080324446	0.123063062	4	0	0

FIGN	0.1861919	0.162613152	0.023578731	0.303003223	0.080360868	0.123108348	10	0	0
CLPSL2	0.3114806	0.325744619	-0.01426404	0.117388299	0.080384305	0.123133734	9	0	0
NFKB2	0.1419796	0.144686068	-0.0027065	0.023448964	0.080406899	0.123157825	17	0	0
TEKT4	0.4378292	0.405837767	0.031991443	0.123518456	0.080418239	0.123164677	4	0	0
USP39	0.0755801	0.069194625	0.006385483	0.018254778	0.080468278	0.123230792	13	0	0
MRPL35	0.209912	0.216958752	-0.00704677	0.021091693	0.080527073	0.123298729	11	0	0
SORBS1	0.2435083	0.236518202	0.006990117	0.007107094	0.080533263	0.123298729	7	0	0
CSTB	0.1529438	0.148867701	0.004076137	0.079384079	0.080520399	0.123298729	4	0	0
ZIK1	0.127419	0.114063993	0.013355033	0.418571124	0.080546275	0.123308125	9	0	0
ZNF391	0.361504	0.357555518	0.003948504	0.182854797	0.080562275	0.123322092	8	0	0
PGP	0.0405275	0.039491373	0.001036104	0.053527707	0.080578759	0.123336799	8	0	0
C2orf68	0.1391106	0.144140104	-0.00502955	-0.05468116	0.080604413	0.123365538	10	0	0
APBB1	0.0609977	0.046244896	0.014752799	0.373110671	0.080702499	0.123505121	16	0	0
BUD31	0.0624772	0.075190891	-0.01271366	-0.05475267	0.080800751	0.123644934	14	0	0
EIF2S2	0.1090314	0.125461428	-0.01643006	-0.01425563	0.080905603	0.123794821	10	0	0
CUL2	0.3007297	0.302266926	-0.00153726	0.035333518	0.080950487	0.123852933	10	0	0
ZNF552	0.1306555	0.137630367	-0.00697488	-0.05175161	0.080981499	0.123889813	10	0	0
SNX9	0.2640119	0.261138431	0.002873432	0.007248804	0.081062718	0.124003489	7	0	0
GALNT11	0.0696115	0.069405074	0.000206384	0.056785062	0.081180331	0.124172814	12	0	0
LDHB	0.6746934	0.708978778	-0.03428542	-0.07134879	0.081201367	0.124194399	6	0	0
SLC35C1	0.5572006	0.567162397	-0.00996182	0.011779599	0.081223307	0.124217364	13	0	0
HPSE	0.1966969	0.19911374	-0.00241683	0.0372287	0.081320598	0.12435555	18	0	0
NIN	0.1111782	0.108890999	0.002287204	0.058111986	0.081343081	0.124379327	11	0	0
ZNF593	0.1693433	0.16906829	0.000275023	-0.01111476	0.081360778	0.124395781	12	0	0
SAMD13	0.4910406	0.507926698	-0.01688614	0.045043375	0.081496688	0.124567642	4	0	0
RPP14	0.1737858	0.178852697	-0.00506685	0.000783889	0.081499232	0.124567642	9	0	0
BDP1	0.1275519	0.133103498	-0.00555159	0.009640387	0.081500963	0.124567642	9	0	0
PRUNE2	0.1766297	0.183724307	-0.00709465	0.084701922	0.081499098	0.124567642	11	0	0
PLA2R1	0.1426431	0.137370964	0.005272144	0.194182802	0.081579388	0.124668865	11	0	0
CHD9	0.1569894	0.162341296	-0.00535185	-0.0145898	0.081581092	0.124668865	8	0	0
CSNK1A1	0.0976554	0.102550513	-0.00489511	0.006405783	0.081593504	0.124677209	11	0	0
GNG13	0.2036862	0.20070263	0.002983559	0.06284197	0.08160745	0.124687898	10	0	0
HIST1H2BI	0.1063099	0.074345166	0.031964755	0.525877183	0.08166161	0.124760021	15	0	0
ZBTB14	0.4608388	0.471993569	-0.01115473	-0.0331736	0.081683543	0.124782899	14	0	0
FAM49A	0.315114	0.331088647	-0.01597468	-0.05006866	0.08174903	0.124837584	7	0	0
PODXL2	0.4211531	0.424370849	-0.00321771	0.088370246	0.081729025	0.124837584	10	0	0
PRRC2A	0.1179605	0.116180438	0.001780076	0.052984603	0.081750682	0.124837584	19	0	0
C7orf25	0.2256829	0.242195546	-0.01651264	-0.03638413	0.081740691	0.124837584	8	0	0
PRSS53	0.7442056	0.769927049	-0.02572149	-0.05259075	0.0817611	0.124837584	4	0	0
SRSF2	0.0819918	0.08205009	-5.83E-05	0.032088038	0.081757214	0.124837584	9	0	0
LAMTOR1	0.3662317	0.35781403	0.008417696	0.036606572	0.081818347	0.124914358	12	0	0
USP48	0.2050541	0.194215054	0.010839061	0.029489811	0.081903606	0.125033882	8	0	0
AXIN2	0.2131037	0.200962764	0.01214091	0.058022071	0.081913806	0.125038811	5	0	0
NEK4	0.1835738	0.184740232	-0.00116641	0.013504344	0.081942143	0.125071421	8	0	0
DUOX2	0.1150282	0.102723528	0.012304638	0.17826246	0.081956033	0.125081978	13	0	0
HNRNPA1	0.0431912	0.041684234	0.001506916	0.039673527	0.082040574	0.125200352	18	0	0
DAD1	0.0798569	0.08802883	-0.00817188	-0.005637	0.082083629	0.1252554	11	0	0
RHBG	0.3974744	0.394530654	0.002943753	0.09210368	0.082111433	0.1252574	12	0	0
MORN2	0.1567582	0.169438514	-0.01268029	-0.05977728	0.082095165	0.1252574	15	0	0
JADE2	0.2090641	0.190321498	0.018742597	0.080346784	0.082107708	0.1252574	6	0	0
RNF20	0.2715639	0.28552888	-0.013965	-0.05772248	0.082112874	0.1252574	7	0	0
CEMP1	0.1268044	0.130996296	-0.00419188	-0.0017943	0.082130325	0.125273365	10	0	0
DEPDC1	0.1828486	0.196157827	-0.01330927	0.116499133	0.082218233	0.125396788	10	0	0
SYMPK	0.0488185	0.045668337	0.003150117	0.082812758	0.082351016	0.125588625	8	0	0
STXBP3	0.1095627	0.102213221	0.007349482	0.024726508	0.082374825	0.125614254	9	0	0
ZNF684	0.1820871	0.183073362	-0.00098628	0.043662209	0.08244217	0.125689712	14	0	0
GABPB2	0.046703	0.042086951	0.004616079	0.135432372	0.082439342	0.125689712	12	0	0
CCDC126	0.1584885	0.161562746	-0.00307425	-0.00424601	0.082445331	0.125689712	10	0	0
CCT3	0.4698646	0.462884567	0.006980032	0.13313186	0.082482099	0.125735078	9	0	0
ABCC5	0.1218174	0.124530683	-0.00271326	0.096823254	0.082508216	0.125758477	9	0	0
KCND2	0.0727003	0.057459253	0.015241014	0.284485073	0.082522261	0.125758477	7	0	0
TRIM55	0.6884079	0.686666067	0.001741806	0.019994268	0.082529927	0.125758477	9	0	0
HECTD1	0.0413534	0.04049473	0.00085867	0.009941769	0.082513312	0.125758477	8	0	0

NXNL1	0.7172822	0.741912049	-0.0246298	-0.04436735	0.082532506	0.125758477	3	0	0
HSD17B4	0.3005641	0.31634947	-0.01578541	-0.04124669	0.082552085	0.125777625	13	0	0
ATAT1	0.2594289	0.257338645	0.002090221	0.031559132	0.082573785	0.125800001	17	0	0
ZNF226	0.1133977	0.124383984	-0.01098624	-0.06066857	0.082582905	0.125803208	12	0	0
ARHGEF19	0.4345084	0.461236714	-0.02672828	-0.08627292	0.08268996	0.125952246	5	0	0
DDX42	0.0864451	0.096212178	-0.00976707	-0.04038711	0.082694784	0.125952246	8	0	0
FADS1	0.1979277	0.177826343	0.020101316	0.273879179	0.08277584	0.126054294	6	0	0
SLC31A2	0.3431261	0.356819694	-0.01369355	-0.02771969	0.082790021	0.126065185	5	0	0
SAP30L	0.0512006	0.049251973	0.001948644	0.058306435	0.08283785	0.126127307	8	0	0
AK9	0.0384851	0.041408972	-0.00292389	-0.03955956	0.082888027	0.126192993	12	0	0
SERPINA4	0.6036576	0.640539331	-0.03688173	-0.09120271	0.082976421	0.126316846	9	0	0
GOLT1A	0.2241188	0.1933059	0.030812925	0.27864881	0.08300315	0.126331255	13	0	0
ANKRD33B	0.1498243	0.150454954	-0.00063063	-0.01239018	0.083000608	0.126331255	11	0	0
ARC	0.2013607	0.181558433	0.019802261	0.137532471	0.083011443	0.126331255	3	0	0
MIS12	0.0476002	0.046015638	0.001584595	0.040687844	0.083014059	0.126331255	7	0	0
IL6ST	0.0735905	0.068829038	0.004761428	0.068262442	0.083053611	0.126380722	12	0	0
CCDC155	0.6977372	0.731641523	-0.03390427	-0.06851713	0.083074134	0.126401227	2	0	0
TEX38	0.236245	0.255033561	-0.0187886	-0.01914784	0.083094369	0.126421292	8	0	0
TCHP	0.1491092	0.15420665	-0.00509744	-0.03234996	0.083114635	0.126435264	17	0	0
ELAC2	0.1413408	0.144778918	-0.0034381	-0.0267373	0.083117652	0.126435264	7	0	0
NDUFB1	0.054416	0.05259483	0.001821184	0.033523419	0.083179345	0.12651838	8	0	0
WDR12	0.1066042	0.094959297	0.011644932	0.070322314	0.083199974	0.126520156	10	0	0
GPSM1	0.3340489	0.316929265	0.017119632	0.128263789	0.083191402	0.126520156	5	0	0
RNF208	0.3506283	0.366085632	-0.01545732	0.045171268	0.083201675	0.126520156	10	0	0
SAMD11	0.0541104	0.046647947	0.00746242	0.161422559	0.083292936	0.126648194	7	0	0
CPLX1	0.0523906	0.037703997	0.014686593	0.415597106	0.083354071	0.126730407	4	0	0
MYL2	0.700103	0.707768517	-0.00766547	-0.01671625	0.083375004	0.126751489	13	0	0
SERPINE3	0.8316139	0.857444729	-0.0258308	-0.04431814	0.083472905	0.126889569	4	0	0
HIPK1	0.0701699	0.076308966	-0.00613906	-0.00214787	0.083488064	0.126901857	14	0	0
DCUN1D5	0.0943636	0.099786217	-0.00542264	-0.04929304	0.083517772	0.126936256	15	0	0
NCAM1	0.1804566	0.185668771	-0.00521215	0.088456168	0.083572587	0.127000087	12	0	0
GYS2	0.7065486	0.766231618	-0.05968299	-0.11542258	0.083573931	0.127000087	1	0	0
BIK	0.1146381	0.118092849	-0.00345472	0.010318758	0.083646079	0.127098956	24	0	0
CHD8	0.0321244	0.03402638	-0.00190197	-0.05782754	0.083694128	0.127161193	3	0	0
DEFA6	0.8312713	0.816708416	0.014562875	0.025192538	0.083760665	0.127251507	1	0	0
TRAPPC4	0.0504393	0.048793765	0.001645582	0.048198805	0.083783952	0.127276104	9	0	0
CACNA1G	0.09487	0.095539494	-0.00066952	0.018640038	0.08379891	0.127288046	20	0	0
MCM3	0.102901	0.11368804	-0.01078701	-0.07216687	0.083815077	0.127301823	10	0	0
SAR1B	0.0468933	0.050122397	-0.00322906	-0.07149397	0.083822997	0.127303072	9	0	0
PMCH	0.8163855	0.827543026	-0.01115756	-0.01974029	0.083839813	0.12731783	3	0	0
EXOC3	0.1664181	0.171621257	-0.00520318	-0.02209472	0.08385079	0.127319171	11	0	0
NUFIP1	0.0787563	0.077701378	0.001054876	-0.0124592	0.083854893	0.127319171	14	0	0
ACAT1	0.1033673	0.105266595	-0.00189933	0.011663324	0.083951917	0.127455695	12	0	0
BMP8A	0.1133333	0.107949361	0.005383988	0.314514087	0.08401255	0.127532123	8	0	0
PSMD11	0.1324728	0.144478205	-0.01200537	-0.08741531	0.084023287	0.127532123	10	0	0
SMURF2	0.0665992	0.070229767	-0.00363058	-0.04485093	0.084023589	0.127532123	3	0	0
NDUFB4	0.1879702	0.197682145	-0.00971193	-0.0505676	0.084056936	0.127561148	9	0	0
PARP6	0.2667145	0.296485522	-0.02977103	-0.07665663	0.084051321	0.127561148	5	0	0
PPP2R5E	0.0402753	0.039809579	0.000465693	0.027680492	0.084109424	0.127630003	10	0	0
RPS11	0.1274946	0.129647365	-0.00215281	0.035317012	0.084117223	0.127631039	11	0	0
EXOG	0.3642917	0.394039376	-0.02974766	-0.05805251	0.084145969	0.127663855	4	0	0
CGGBP1	0.0439007	0.042535422	0.00136531	0.042885836	0.084192738	0.127702406	15	0	0
INCENP	0.3746532	0.381976476	-0.00732332	-0.01950556	0.084179581	0.127702406	5	0	0
PCDH17	0.1361627	0.115257237	0.020905499	0.254583335	0.084192224	0.127702406	7	0	0
TRAP1	0.2887972	0.294992894	-0.00619568	-0.01927817	0.084332191	0.127903111	7	0	0
GLDC	0.1172132	0.064628966	0.052584246	0.92632107	0.084375795	0.127958422	5	0	0
TRAPPC6A	0.0398319	0.0377888	0.002043052	0.061122243	0.084411367	0.128001546	11	0	0
MMP16	0.1297683	0.102124298	0.027644051	0.285843882	0.084422306	0.12800731	5	0	0
CD74	0.2843102	0.264226872	0.020083338	0.323758373	0.084436701	0.128018315	9	0	0
SCFD2	0.1105326	0.114740181	-0.00420755	-0.0586634	0.084444859	0.128019862	13	0	0
COQ10B	0.1109207	0.114540231	-0.00361953	0.039501622	0.084473582	0.128052584	15	0	0
TIGD4	0.1023944	0.0925527	0.009841703	0.073574193	0.084508232	0.128094283	7	0	0
PRELP	0.5317069	0.554492405	-0.02278552	-0.02744372	0.084524351	0.128107889	2	0	0

CYP39A1	0.1378897	0.132755591	0.005134115	0.092369846	0.084534371	0.12811225	13	0	0
SLC25A27	0.1064789	0.101079183	0.005399676	0.107720921	0.084575367	0.12816355	13	0	0
SLC35E3	0.0925194	0.101728243	-0.0092088	-0.05346329	0.084675093	0.128292994	8	0	0
COQ5	0.0366235	0.033892064	0.002731407	0.078289248	0.084672488	0.128292994	8	0	0
DBNL	0.1618247	0.178536824	-0.01671209	-0.05968247	0.084944265	0.128685253	9	0	0
SCN3B	0.2447816	0.224601889	0.020179738	0.127006132	0.084948338	0.128685253	5	0	0
MSTO1	0.1048765	0.13066181	-0.02578534	-0.08043078	0.084963407	0.128686343	7	0	0
FIG4	0.0691661	0.068194885	0.000971179	-0.01384116	0.084960604	0.128686343	15	0	0
VTI1A	0.2028928	0.210309602	-0.00741684	-0.01348159	0.084989049	0.128714311	14	0	0
MDM4	0.1626934	0.179029855	-0.0163365	-0.04099081	0.085012603	0.128739113	10	0	0
SLC25A39	0.0495989	0.046539135	0.003059719	0.197039769	0.085022991	0.128743973	6	0	0
CCDC12	0.5946986	0.605414624	-0.01071599	-0.02567524	0.085091322	0.128836566	4	0	0
TTC3	0.0767492	0.071616238	0.005132933	0.067924291	0.085251933	0.129068851	9	0	0
MRPS28	0.1651868	0.178608084	-0.0134213	-0.03718966	0.085268984	0.129072874	12	0	0
CCNJ	0.1872067	0.174259074	0.012947628	0.26504192	0.085267175	0.129072874	15	0	0
DOK1	0.265043	0.265812136	-0.00076912	-0.02513816	0.085283445	0.129075652	10	0	0
PCBD2	0.0311401	0.028613197	0.002526934	0.079196809	0.085287035	0.129075652	9	0	0
BCO2	0.1567655	0.149295499	0.007469963	0.094283185	0.085292408	0.129075652	10	0	0
STRN3	0.0630741	0.062667288	0.000406807	0.028622883	0.085407582	0.129239044	6	0	0
MYD88	0.0468201	0.045748415	0.001071679	0.033081302	0.085481774	0.129340399	14	0	0
MBD1	0.3058473	0.317556982	-0.01170964	-0.01835274	0.085534099	0.129408655	11	0	0
HSD11B1	0.7585656	0.785562076	-0.02699646	-0.05205161	0.085638297	0.129555372	7	0	0
RDH10	0.0718334	0.078409527	-0.0065761	-0.0149998	0.085652335	0.129565681	13	0	0
HNRNPR	0.0383376	0.038741652	-0.00040403	0.002030739	0.085676136	0.129590756	9	0	0
CEP112	0.0729782	0.06529815	0.007680025	0.124094766	0.085711965	0.129634016	4	0	0
METTL8	0.1636027	0.189126138	-0.02552347	-0.05023075	0.085723612	0.1296407	5	0	0
PGM3	0.1455303	0.147319982	-0.00178971	0.037469879	0.085784474	0.129721805	13	0	0
04-Mar	0.0896231	0.083576993	0.006046137	0.17585025	0.085808284	0.12974687	15	0	0
SNX6	0.2754851	0.282424703	-0.00693965	-0.02963333	0.085840319	0.129784368	9	0	0
LRRIQ3	0.142314	0.15145371	-0.00913972	-0.03688267	0.085870657	0.129819293	12	0	0
CPSF1	0.0809204	0.087365115	-0.00644469	0.011672974	0.085884866	0.129829832	9	0	0
RAB5C	0.1357442	0.121152077	0.014592136	0.095925612	0.08590446	0.129848508	7	0	0
STAG3	0.2207356	0.209570957	0.011164595	0.143260129	0.085937871	0.129888064	16	0	0
CPEB3	0.0357467	0.035180571	0.000566102	0.015523023	0.085988744	0.129954003	15	0	0
GMEB1	0.1758646	0.172329026	0.003535537	0.063627569	0.086019275	0.129989192	6	0	0
RNASEH2B	0.2922681	0.301626814	-0.00935867	-0.04154901	0.086069088	0.13005351	8	0	0
PAK1IP1	0.1969599	0.212910945	-0.015951	0.014066906	0.086081814	0.130061783	9	0	0
LEPR	0.039704	0.041842014	-0.00213799	-0.02624581	0.086156749	0.130142112	12	0	0
LEPROT	0.039704	0.041842014	-0.00213799	-0.02624581	0.086156749	0.130142112	12	0	0
ATL1	0.0923757	0.092285635	9.00E-05	-0.03829289	0.08614997	0.130142112	14	0	0
UNC13B	0.0647692	0.061405162	0.00336402	0.106180631	0.086188395	0.130178952	8	0	0
TBC1D13	0.1531037	0.163462578	-0.0103589	-0.06442258	0.086218515	0.13021348	5	0	0
MT1H	0.4277607	0.428135797	-0.00037508	0.06323588	0.086293934	0.13031641	6	0	0
CSGALNACT	0.1834743	0.182049563	0.001424752	0.035057014	0.086312293	0.130333162	10	0	0
ACTR1A	0.0504587	0.046988414	0.003470271	0.047685844	0.086344865	0.130371369	9	0	0
COMMD2	0.1906402	0.208601987	-0.01796178	-0.04872507	0.086463457	0.130539442	10	0	0
GTPBP3	0.2966719	0.308921607	-0.01224969	0.003717116	0.086494213	0.130563895	11	0	0
OGFR	0.2706203	0.272292007	-0.00167173	-0.0269372	0.086489175	0.130563895	7	0	0
CARS	0.2458342	0.250938209	-0.00510397	-0.04122645	0.086552595	0.130641029	7	0	0
SEC22C	0.0325746	0.030546134	0.00202851	0.064783664	0.086623019	0.130727869	4	0	0
MYO10	0.1010967	0.109009016	-0.00791232	0.035035938	0.086638017	0.130727869	13	0	0
ZNHIT1	0.1571656	0.150766406	0.006399162	0.034704853	0.08663163	0.130727869	15	0	0
ZMIZ1	0.0532386	0.048622941	0.004615642	0.068989812	0.086639283	0.130727869	13	0	0
ENO2	0.4705391	0.490341791	-0.01980265	-0.05336143	0.086688741	0.130791492	7	0	0
GJA3	0.0695245	0.062744116	0.006780402	0.165808019	0.086767844	0.130899828	12	0	0
HK2	0.2401724	0.255099779	-0.01492741	-0.02311211	0.086800116	0.1309375	10	0	0
ZNF614	0.8997679	0.90455514	-0.0047872	-0.00757155	0.08683644	0.130981279	1	0	0
ZNF7	0.1292174	0.137217505	-0.00800006	0.013947506	0.086845974	0.130984645	8	0	0
GAA	0.1489866	0.16134784	-0.01236128	-0.02709253	0.086867806	0.131006556	7	0	0
HSD17B8	0.3329764	0.33634176	-0.00336534	0.184386216	0.086918953	0.13107267	16	0	0
GEMIN7	0.5186663	0.528355258	-0.00968891	-0.01916965	0.086970033	0.131127649	7	0	0
MARK4	0.5186663	0.528355258	-0.00968891	-0.01916965	0.086970033	0.131127649	7	0	0
AK2	0.1236965	0.128003626	-0.00430713	0.029199476	0.086980228	0.131131997	19	0	0

CEND1	0.3120802	0.313432128	-0.00135189	0.121344517	0.087009821	0.131165587	13	0	0
ITM2B	0.1666613	0.173891015	-0.00722968	0.013124999	0.08708759	0.131271789	8	0	0
RAB28	0.0385875	0.041803346	-0.00321583	-0.05399771	0.08710972	0.131285319	9	0	0
ARL9	0.292012	0.277619524	0.01439248	0.258057681	0.087111205	0.131285319	7	0	0
XPO5	0.0904988	0.104340197	-0.01384136	-0.04235573	0.087139081	0.131316296	10	0	0
ZNF644	0.2320553	0.211318586	0.020736684	0.093839742	0.087148059	0.131318792	10	0	0
OSCP1	0.1275902	0.106600201	0.020990029	0.378310858	0.087189992	0.131370943	14	0	0
ACBD5	0.0918039	0.104036186	-0.01223232	-0.01985418	0.087233932	0.131415068	7	0	0
RPL41	0.1130033	0.122169769	-0.00916649	-0.00506067	0.087230815	0.131415068	14	0	0
RERGL	0.6265135	0.650991861	-0.02447838	-0.08177775	0.087253625	0.131423688	4	0	0
NDUFA3	0.360334	0.367500515	-0.00716651	0.06233189	0.087254309	0.131423688	7	0	0
ZNF620	0.1399692	0.138269663	0.001699528	-0.01454072	0.087370833	0.131577101	15	0	0
LRBA	0.0521163	0.053911783	-0.00179551	-0.03215215	0.087368537	0.131577101	10	0	0
COPB1	0.0618332	0.062903688	-0.00107046	0.028095262	0.087381384	0.131581942	13	0	0
IMP4	0.1844228	0.195607771	-0.011185	-0.04206386	0.087389891	0.131583704	10	0	0
BTBD18	0.7064406	0.685640018	0.020800563	0.082943814	0.087429604	0.13163245	14	0	0
CLPTM1L	0.1257793	0.143095607	-0.01731629	-0.05123902	0.087462997	0.131671674	5	0	0
HES7	0.0549517	0.05604474	-0.001093	-0.03721244	0.087479945	0.131686134	11	0	0
PSMD14	0.3241153	0.339160143	-0.01504487	-0.04465897	0.087492415	0.131693853	11	0	0
TIMM22	0.0982718	0.10091268	-0.00264091	-0.01715671	0.087579078	0.131813236	12	0	0
C11orf80	0.3328345	0.366771743	-0.03393728	-0.07722971	0.087627571	0.131875157	7	0	0
ZNF507	0.2969707	0.299287437	-0.00231677	-0.01525205	0.087644825	0.131890056	7	0	0
ZNF473	0.0897846	0.095374564	-0.00558998	0.036166612	0.087782368	0.132085952	11	0	0
TMEM86B	0.878734	0.898476955	-0.01974297	-0.03257365	0.087789763	0.132085998	3	0	0
GABARAP	0.1793087	0.176877218	0.002431482	0.021630535	0.087894731	0.132232838	14	0	0
SARS2	0.2833135	0.244166215	0.039147331	0.49318603	0.087917699	0.132256299	6	0	0
CISH	0.1103997	0.09913946	0.011260204	0.098697664	0.087943935	0.13227579	13	0	0
HNRNPA0	0.0698171	0.074134144	-0.00431702	0.015099435	0.087945405	0.13227579	10	0	0
FOXP4	0.0951481	0.10330285	-0.00815472	-0.0034049	0.087964165	0.132292911	7	0	0
FPGS	0.575409	0.545132533	0.030276497	0.072472381	0.087982959	0.132310083	2	0	0
HELQ	0.0548906	0.056738923	-0.00184837	0.018410152	0.088023412	0.132353073	8	0	0
TPT1	0.0484801	0.044679837	0.003800263	0.066823056	0.088029267	0.132353073	7	0	0
PDXP	0.0527636	0.057132788	-0.00436916	-0.08499638	0.088033685	0.132353073	7	0	0
DDX24	0.1128649	0.118863388	-0.00599845	-0.02426003	0.088051754	0.132369143	13	0	0
ZNF354A	0.1737583	0.186909084	-0.01315075	-0.0280425	0.08806299	0.13237494	4	0	0
PQLC1	0.0360808	0.033081904	0.002998854	0.084866146	0.088097169	0.132415221	7	0	0
ALAD	0.2826359	0.292851255	-0.01021532	-0.02780968	0.088112861	0.132419457	9	0	0
RNF6	0.2519497	0.254251321	-0.0023016	0.034860375	0.088114753	0.132419457	9	0	0
SMCO4	0.3333177	0.336909341	-0.00359162	-0.02118191	0.088149798	0.132449927	8	0	0
KRBA2	0.1824136	0.180030529	0.002383027	0.04502461	0.088143023	0.132449927	8	0	0
TRAPPC13	0.0424071	0.039112985	0.00329409	0.056045486	0.088194049	0.132494217	14	0	0
PHLDA1	0.833085	0.843199564	-0.01011452	-0.01720506	0.088192955	0.132494217	1	0	0
C16orf72	0.1172298	0.098149518	0.019080249	0.119356078	0.088219572	0.132521461	7	0	0
VPS36	0.3535915	0.360566485	-0.00697496	-0.0251607	0.088232324	0.132529517	13	0	0
LDB3	0.6087783	0.593245441	0.015532856	0.040973887	0.088240544	0.132530765	11	0	0
C19orf24	0.3452675	0.358645186	-0.01337772	0.003601376	0.088262875	0.132553206	4	0	0
CIDEB	0.4598676	0.455101058	0.004766559	0.094341547	0.088272513	0.13255658	16	0	0
PDCD6IP	0.0580302	0.059779153	-0.00174897	-0.03098438	0.088320524	0.132617573	12	0	0
MMP28	0.1961757	0.205777128	-0.00960141	0.186707671	0.088368994	0.132679247	10	0	0
PGLS	0.253494	0.250711519	0.00278247	0.05773935	0.088390697	0.132700723	5	0	0
HEATR1	0.1027958	0.118247201	-0.01545145	-0.00365283	0.088554336	0.132935268	8	0	0
PBXIP1	0.3698033	0.349034218	0.020769122	0.052207277	0.088579969	0.132962619	12	0	0
SEMA5A	0.4326857	0.410243897	0.022441824	0.186106826	0.088672024	0.133080225	5	0	0
DUSP5	0.1782681	0.187085145	-0.00881701	-0.03477245	0.088673158	0.133080225	11	0	0
RSL24D1	0.1222246	0.128840183	-0.00661554	0.002060337	0.088684672	0.133086369	8	0	0
RCBTB1	0.1370801	0.142473613	-0.00539351	0.000363466	0.088725024	0.133135785	11	0	0
ISG20L2	0.0656684	0.074858855	-0.0091905	-0.03733817	0.088734718	0.133139191	10	0	0
KIF21A	0.1764597	0.177961718	-0.00150206	0.007466465	0.088777652	0.133192469	6	0	0
B9D1	0.0805048	0.076515094	0.003989715	0.030791431	0.088806555	0.133224688	11	0	0
STX12	0.1636294	0.182433545	-0.01880412	-0.06990742	0.088844504	0.133270471	10	0	0
SOX21	0.1293216	0.1097845	0.019537098	0.238684522	0.088879906	0.133312427	14	0	0
BPHL	0.1746352	0.198720293	-0.02408514	-0.04249023	0.088893422	0.13332155	7	0	0
SNTB2	0.0320092	0.031545317	0.000463921	0.03570536	0.088919331	0.133349258	8	0	0

NRBP1	0.5672575	0.583623603	-0.01636613	0.017041602	0.088974537	0.13339851	6	0	0
ST6GAL1	0.3706008	0.357822763	0.012778041	0.237996228	0.088967475	0.13339851	11	0	0
CYB561A3	0.051141	0.051439859	-0.00029883	0.027490303	0.088983506	0.13339851	16	0	0
RASSF8	0.1219997	0.120045221	0.00195444	0.045268276	0.088970853	0.13339851	8	0	0
MORF4L1	0.4255471	0.431492705	-0.00594556	0.0785262	0.08898936	0.13339851	7	0	0
RPN1	0.0341689	0.037040406	-0.00287147	-0.03768163	0.089021763	0.13343593	7	0	0
SLC25A46	0.5411643	0.54648656	-0.00532222	0.010434924	0.089104992	0.133549523	7	0	0
C3orf38	0.0864985	0.081112115	0.005386392	0.048698525	0.089162218	0.133624126	14	0	0
DCLK2	0.1456821	0.13277	0.01291207	0.149815537	0.089211761	0.13368442	12	0	0
CHMP1B	0.0844014	0.089024816	-0.00462341	-0.01947066	0.089217357	0.13368442	13	0	0
TIMP2	0.1728144	0.150348233	0.022466206	0.102017955	0.089250254	0.133722543	9	0	0
CEPT1	0.1213064	0.136831583	-0.01552522	-0.0389082	0.089259842	0.133725737	9	0	0
METTL17	0.3448412	0.346465344	-0.00162416	0.016137324	0.089331221	0.133821496	10	0	0
EBF1	0.0776732	0.077822354	-0.00014913	0.136077832	0.0894326	0.133962177	15	0	0
FSTL3	0.2540388	0.250873525	0.003165304	0.032518259	0.089454904	0.133984397	8	0	0
TNFAIP8	0.0807483	0.07753602	0.003212239	-0.00175162	0.089475035	0.13400336	13	0	0
CC2D1B	0.262169	0.27073694	-0.00856795	-0.02123784	0.089520839	0.134060763	8	0	0
TLK1	0.8180788	0.851619837	-0.03354104	-0.05728341	0.089553402	0.134098332	1	0	0
MDFIC	0.5253786	0.503826369	0.021552181	0.108562603	0.089591766	0.134142329	9	0	0
REXO1	0.0253743	0.024460364	0.000913923	0.041585844	0.089597742	0.134142329	2	0	0
MFSD3	0.0455175	0.047136286	-0.00161877	-0.034658	0.089621488	0.134166681	10	0	0
STAG1	0.1154831	0.129549203	-0.0140661	-0.01856891	0.089632113	0.134171389	9	0	0
ODF3L1	0.4563354	0.451226469	0.005108965	0.074325292	0.089777163	0.1343773	10	0	0
ANAPC13	0.1361679	0.144047661	-0.00787972	-0.04358749	0.089792573	0.134389151	8	0	0
CENPO	0.185379	0.204420385	-0.01904143	-0.02597097	0.089901628	0.134541143	19	0	0
NT5DC3	0.0623241	0.068418204	-0.00609406	-0.07049084	0.08996341	0.13462237	6	0	0
CCDC85C	0.0516649	0.047793568	0.003871378	0.0681118	0.089980695	0.134637002	9	0	0
07-Sep	0.0424931	0.048884533	-0.00639145	-0.04872116	0.089993221	0.134644512	14	0	0
NLRP6	0.6750576	0.663627088	0.011430561	0.028122398	0.090040861	0.134691806	10	0	0
TMEM231	0.2475574	0.246104155	0.001453248	0.035372979	0.090035742	0.134691806	6	0	0
THOP1	0.2328347	0.248722223	-0.01588749	-0.04559779	0.09004736	0.134691806	8	0	0
DDX39B	0.3419888	0.352480907	-0.01049215	-0.00795147	0.090074823	0.134710417	19	0	0
IDH2	0.145035	0.155010882	-0.00997588	0.008486259	0.090073162	0.134710417	4	0	0
PROK1	0.6147507	0.630910438	-0.01615972	-0.04131831	0.090089431	0.134721029	8	0	0
NGDN	0.2770401	0.289115319	-0.0120752	0.003758209	0.090117881	0.13475234	12	0	0
LCTL	0.3786233	0.393730604	-0.01510726	-0.0699284	0.090171337	0.13479736	8	0	0
MYCBPAP	0.1606006	0.175034173	-0.01443353	0.145328841	0.090178051	0.13479736	14	0	0
SMARCA4	0.0313132	0.032751591	-0.00143839	-0.0505437	0.090163626	0.13479736	5	0	0
SAMHD1	0.278878	0.275673778	0.003204209	0.066981329	0.090177407	0.13479736	11	0	0
PARM1	0.2618732	0.259322618	0.002550615	-0.00397956	0.09021242	0.134837497	10	0	0
CCR4	0.771674	0.781755941	-0.0100819	-0.01597479	0.090270614	0.134893779	4	0	0
SHANK2	0.1246382	0.09482678	0.029811393	0.354571934	0.090269292	0.134893779	5	0	0
C16orf71	0.055374	0.056065854	-0.00069183	0.005041782	0.090272638	0.134893779	7	0	0
CXXC1	0.4589024	0.471360635	-0.01245819	-0.0138075	0.090390125	0.135058087	9	0	0
TTLL4	0.3742169	0.385785697	-0.01156881	-0.01475513	0.090433514	0.135100408	10	0	0
PPCDC	0.2459075	0.246090095	-0.00018264	0.022922487	0.090431027	0.135100408	8	0	0
PFKFB4	0.864045	0.876613789	-0.01256882	-0.02059829	0.090465137	0.135136395	1	0	0
MRPS18B	0.0577148	0.053399481	0.004315308	0.083125531	0.090477067	0.135142961	32	0	0
ZNF284	0.1448675	0.137395618	0.007471902	0.252752528	0.090524104	0.13520196	12	0	0
PLIN5	0.2085583	0.224323895	-0.01576556	0.029875497	0.090540124	0.135214627	12	0	0
GP1BA	0.7198159	0.734489807	-0.01467393	-0.02741416	0.090555098	0.135225729	8	0	0
INO80	0.0850615	0.091526705	-0.00646517	-0.01045561	0.090568685	0.13523476	12	0	0
ULK1	0.3585433	0.373845772	-0.01530247	-0.00472999	0.09060196	0.135273184	9	0	0
UBE2N	0.1822806	0.182359499	-7.89E-05	-0.03556684	0.09064759	0.135330046	5	0	0
CCNYL1	0.0472741	0.045594777	0.001679348	0.014944607	0.090693071	0.135380473	9	0	0
BTBD2	0.4558961	0.435895749	0.020000302	0.063301655	0.090696463	0.135380473	1	0	0
CHAC2	0.1000803	0.116328917	-0.01624863	-0.04778948	0.090832301	0.135571953	9	0	0
PAK6	0.2601367	0.244615404	0.015521316	0.035472819	0.090863816	0.135596423	3	0	0
DSG2	0.3110624	0.295689533	0.015372869	0.061441784	0.090863341	0.135596423	4	0	0
SLAIN1	0.1202766	0.124466873	-0.00419028	0.174749639	0.090880224	0.135598344	15	0	0
C19orf44	0.2677939	0.283093015	-0.01529916	-0.02907508	0.090877214	0.135598344	10	0	0
SOX2	0.1418264	0.149896783	-0.00807042	-0.05034901	0.09096634	0.135715544	24	0	0
TMEM253	0.1661805	0.14596666	0.020213885	0.123036363	0.091023331	0.135777982	12	0	0

NFIX	0.369082	0.386128691	-0.01704674	-0.05499343	0.091022391	0.135777982	2	0	0
ACBD3	0.2608739	0.266651605	-0.00577771	0.011110397	0.091061096	0.13582302	13	0	0
CLEC18A	0.6988121	0.733599198	-0.03478712	-0.06912203	0.091104292	0.135876151	1	0	0
CYTH2	0.0844726	0.08909952	-0.00462696	0.002804622	0.091222066	0.13604049	10	0	0
NSUN6	0.0524879	0.061534981	-0.00904706	-0.02958559	0.091245648	0.136064345	14	0	0
RAB5B	0.0766907	0.070382601	0.006308136	0.055139364	0.091309264	0.13614789	16	0	0
DOCK3	0.1850077	0.168463193	0.01654451	0.153551681	0.0913335	0.136172706	6	0	0
SURF4	0.1480294	0.160402269	-0.0123729	-0.00931939	0.09138732	0.136237967	8	0	0
CFL2	0.2669116	0.266824428	8.71E-05	0.036017707	0.091392463	0.136237967	8	0	0
MRPS31	0.038928	0.038491391	0.000436658	0.021083821	0.091443926	0.136303354	15	0	0
VAMP8	0.1655873	0.164430671	0.001156583	0.004301936	0.091456394	0.13631061	12	0	0
DNAJB4	0.0818434	0.077614137	0.004229243	0.082037133	0.091473539	0.136313509	15	0	0
GIPC2	0.0818434	0.077614137	0.004229243	0.082037133	0.091473539	0.136313509	15	0	0
SYCE3	0.0778699	0.080566408	-0.00269648	-0.05523638	0.091520931	0.136372802	7	0	0
CD244	0.526838	0.548273776	-0.02143577	-0.06334655	0.091750445	0.136703437	6	0	0
KXD1	0.209425	0.217959417	-0.00853445	-0.03782178	0.091772	0.136724195	10	0	0
C12orf40	0.8549903	0.861732739	-0.00674246	-0.01004128	0.091787456	0.136735865	10	0	0
RPS29	0.0489656	0.04734914	0.001616429	0.040855569	0.091847705	0.136814255	11	0	0
01-Mar	0.4170136	0.408952622	0.008060982	0.202862037	0.091860843	0.136822461	7	0	0
HIST1H1E	0.0971733	0.107230445	-0.01005717	-0.00725923	0.091973946	0.136979547	12	0	0
TTC31	0.0765177	0.081870435	-0.00535277	-0.00826254	0.092021358	0.137021466	18	0	0
RNF25	0.2261414	0.225585715	0.000555675	0.014392933	0.092012513	0.137021466	12	0	0
ZNF416	0.0551295	0.039193653	0.015935883	0.546432382	0.09202501	0.137021466	13	0	0
TCP10L2	0.6589311	0.662055359	-0.00312427	-0.01792188	0.092036262	0.137026844	3	0	0
RNF181	0.1488318	0.144502722	0.004329037	-0.0170448	0.092052596	0.137039787	6	0	0
DUSP19	0.1673506	0.17582213	-0.00847157	-0.03079843	0.09210537	0.137106973	14	0	0
EML6	0.1670934	0.184930014	-0.01783662	0.00805066	0.092139327	0.137146138	6	0	0
SAMD5	0.0721783	0.062505378	0.009672934	0.186365262	0.092162724	0.13716958	10	0	0
ZNF799	0.1047677	0.099880507	0.004887154	0.282427064	0.092170766	0.137170167	9	0	0
USO1	0.0431818	0.040438148	0.002743651	0.067445431	0.092178832	0.137170789	11	0	0
NSMCE4A	0.3232394	0.330088893	-0.00684948	-0.02654586	0.092219628	0.137208731	10	0	0
CD63	0.1617696	0.162716978	-0.00094736	0.057007383	0.092214277	0.137208731	8	0	0
ARF6	0.0406436	0.039137474	0.001506106	0.047580508	0.0922555	0.137250717	9	0	0
GYPC	0.4882694	0.458270406	0.029998956	0.131490539	0.09228723	0.137286535	11	0	0
HERC1	0.0547364	0.048633962	0.006102429	0.137423691	0.092299452	0.13729333	7	0	0
RAD17	0.1229399	0.127356834	-0.00441689	0.010832925	0.092354969	0.137351749	12	0	0
ALG9	0.2899149	0.303746623	-0.01383169	-0.00024094	0.0923617	0.137351749	12	0	0
DCP1A	0.115415	0.118901043	-0.00348603	-0.03784013	0.092430501	0.137442668	12	0	0
CYP26B1	0.0937394	0.081086499	0.012652926	0.224944243	0.092447361	0.137444949	13	0	0
FITM2	0.1217225	0.131695556	-0.00997304	-0.04132132	0.092444923	0.137444949	9	0	0
FMOD	0.4107124	0.398107131	0.012605273	0.090048337	0.092485922	0.137490883	12	0	0
HEMK1	0.0463377	0.039553035	0.006784628	0.188734037	0.092560636	0.137584153	14	0	0
CTNND1	0.7198169	0.700604857	0.019212021	0.077097232	0.092564004	0.137584153	15	0	0
PTMA	0.1522197	0.156568687	-0.00434896	-0.03936992	0.092613689	0.137642021	7	0	0
UBE2G2	0.1077136	0.112387678	-0.00467407	-0.0587813	0.092618285	0.137642021	12	0	0
ITLN2	0.7630159	0.785947273	-0.02293132	-0.04311202	0.092830446	0.137934461	5	0	0
RMDN3	0.2544311	0.248525456	0.005905651	0.081480222	0.092887447	0.138007725	6	0	0
PSMA1	0.1453223	0.146904184	-0.00158185	0.044976991	0.092907637	0.138026289	12	0	0
NUP160	0.092509	0.09745928	-0.00495024	-0.03079272	0.092971171	0.138109236	12	0	0
MTMR11	0.3533304	0.31772177	0.035608616	0.152900428	0.093008375	0.138153062	11	0	0
NFKBIZ	0.0663059	0.056506429	0.009799489	0.196323306	0.093111494	0.13829478	12	0	0
TP53TG5	0.716512	0.72670151	-0.01018951	-0.01247087	0.093129441	0.138309982	7	0	0
FIBCD1	0.076633	0.064163544	0.012469452	0.170412363	0.093143473	0.138319369	11	0	0
MLLT6	0.0821835	0.076961073	0.005222454	0.114994283	0.093182463	0.138365813	11	0	0
PSPH	0.0639355	0.070408252	-0.00647271	0.004461089	0.093240051	0.138439864	13	0	0
MERTK	0.1756823	0.162934791	0.012747517	0.06028133	0.093256359	0.13844178	5	0	0
ST8SIA4	0.105857	0.084050923	0.021806084	0.183065008	0.093264596	0.13844178	10	0	0
DYNLL2	0.3732444	0.372510256	0.000734121	0.04416639	0.093272216	0.13844178	9	0	0
TTC38	0.1492359	0.14517986	0.004056012	0.123808644	0.093259598	0.13844178	12	0	0
TCF3	0.0277994	0.024315772	0.003483608	0.139544546	0.093322222	0.138504541	2	0	0
FSIP1	0.044885	0.044622517	0.000262531	-0.00570288	0.093335886	0.138513358	13	0	0
HYOU1	0.0581172	0.057015393	0.001101777	0.047649135	0.093354867	0.138530065	7	0	0
SUFU	0.049747	0.046178493	0.003568497	0.048791124	0.093367283	0.138532134	9	0	0

ST5	0.0916079	0.091400091	0.000207784	0.043414926	0.093371708	0.138532134	12	0	0
SERPINB8	0.3147741	0.333210467	-0.01843635	-0.0571539	0.093379491	0.138532221	10	0	0
TBXA2R	0.1582314	0.134953557	0.023277886	0.196212557	0.093391615	0.138538749	13	0	0
PNMT	0.1180585	0.096511807	0.021546697	0.344911246	0.093402116	0.138542867	7	0	0
RIOK2	0.0509197	0.047245717	0.003673972	0.08869442	0.093430547	0.138573579	12	0	0
ZC2HC1A	0.1092812	0.121173386	-0.01189222	-0.05536789	0.093481279	0.138637359	10	0	0
RABIF	0.3065441	0.320483403	-0.0139393	-0.04194279	0.09352485	0.138683527	11	0	0
NF1	0.1824251	0.197161504	-0.01473642	0.011502085	0.093527874	0.138683527	11	0	0
RPA2	0.1687654	0.167000195	0.001765166	0.252754198	0.093618391	0.138806271	13	0	0
NAA30	0.1182337	0.126048897	-0.00781517	-0.0029212	0.093645913	0.1388356	12	0	0
NR1D1	0.1447442	0.152225649	-0.00748147	0.018286778	0.093662107	0.138848132	9	0	0
DUOXA2	0.0934198	0.078299288	0.01512053	0.211659967	0.093712976	0.138912061	17	0	0
NIF3L1	0.0569404	0.055428797	0.001511647	0.027290535	0.093781719	0.139002472	12	0	0
RBL2	0.2697087	0.28977897	-0.02007024	-0.00806531	0.093869168	0.139120592	10	0	0
SEC11A	0.107334	0.111332465	-0.00399843	-0.02475052	0.093985966	0.139282185	10	0	0
STEAP1	0.0259925	0.023118309	0.002874238	0.128531699	0.09402201	0.139302313	2	0	0
AHCYL2	0.1622935	0.164656261	-0.00236274	-0.01772862	0.094017994	0.139302313	7	0	0
KCNQ1	0.2655741	0.265856283	-0.00028219	0.024711172	0.094030615	0.139302313	19	0	0
UACA	0.0993308	0.098640128	0.000690712	0.142164478	0.094025356	0.139302313	10	0	0
AGA	0.078652	0.080553343	-0.00190135	-0.00722246	0.094040461	0.139305393	8	0	0
KRTAP3-2	0.7836459	0.77563825	0.008007642	0.015676608	0.09405605	0.13931698	2	0	0
CDK5RAP2	0.1701318	0.165017236	0.005114607	0.043354745	0.094125107	0.139407755	6	0	0
PCYT1A	0.1449449	0.149878132	-0.00493325	-0.01426521	0.094225897	0.139525969	10	0	0
ITGAD	0.474233	0.491010022	-0.01677697	-0.0463868	0.09422826	0.139525969	3	0	0
TAOK1	0.0304025	0.029243917	0.001158606	0.040313219	0.094216128	0.139525969	8	0	0
GNRHR	0.8606552	0.877526105	-0.01687088	-0.02807357	0.09429964	0.139620137	3	0	0
EPN1	0.5557193	0.582477078	-0.02675775	-0.04431867	0.094312542	0.139627714	7	0	0
SBF1	0.2099385	0.221517343	-0.01157884	-0.0565788	0.094348183	0.139668951	6	0	0
CCDC9	0.1934792	0.170784987	0.022694217	0.146418455	0.094370517	0.139690484	7	0	0
KCTD9	0.0359032	0.035989208	-8.60E-05	0.006145594	0.094380718	0.139694055	16	0	0
ICMT	0.2530266	0.262968511	-0.00994191	-0.00983225	0.094459428	0.1397888	9	0	0
C5orf63	0.3316284	0.344364067	-0.01273565	-0.00858337	0.094463244	0.1397888	10	0	0
PSMC3IP	0.0432417	0.046370213	-0.00312854	-0.05833651	0.094468111	0.1397888	13	0	0
KRT82	0.8048513	0.820382404	-0.01553115	-0.02559078	0.094484941	0.139802169	7	0	0
NUDT18	0.246255	0.2556113	-0.00935632	-0.00585442	0.094520397	0.139843096	4	0	0
HRK	0.1139398	0.091947313	0.021992505	0.379695132	0.09453787	0.13985741	14	0	0
STUB1	0.2063393	0.195221382	0.011117881	0.228367725	0.094693824	0.140076572	7	0	0
SLC39A4	0.6632223	0.689023023	-0.02580071	-0.0587026	0.094769827	0.140177439	11	0	0
CAD	0.3276234	0.335963823	-0.00834045	0.006507939	0.094844639	0.140276528	6	0	0
PTPRN2	0.1994051	0.19013361	0.009271491	0.146247769	0.094873243	0.140307262	10	0	0
TMEM99	0.2888975	0.301069805	-0.01217228	-0.01898488	0.094896396	0.140329932	10	0	0
NDUFAB1	0.1111421	0.118966607	-0.00782452	-0.05670568	0.094917817	0.140350038	9	0	0
ZAR1	0.1887738	0.138916051	0.049857736	0.512244988	0.094925984	0.140350545	3	0	0
MRPS25	0.2714042	0.282600149	-0.01119592	0.058828768	0.094970488	0.140393198	10	0	0
PXT1	0.0341315	0.033761233	0.000370234	0.01848602	0.094962752	0.140393198	14	0	0
RAVER2	0.3391336	0.345902105	-0.00676855	0.025405904	0.095005822	0.140433857	7	0	0
FLAD1	0.2443248	0.255546354	-0.01122155	0.004055607	0.095142574	0.140624409	12	0	0
ITGB8	0.0512115	0.054589084	-0.00337755	-0.07036364	0.09520703	0.140684898	7	0	0
UBE3B	0.2082703	0.19305357	0.015216722	0.065113187	0.09519927	0.140684898	10	0	0
RALGAPB	0.1096774	0.104493567	0.005183873	0.027220987	0.095227002	0.140702818	14	0	0
LURAP1L	0.2360795	0.235209422	0.000870095	0.097584365	0.095372562	0.140894677	4	0	0
C8orf33	0.099502	0.093509459	0.005992527	0.014292028	0.095408571	0.140936265	6	0	0
MIER1	0.1529926	0.161531545	-0.00853895	0.001966257	0.095430793	0.140957481	14	0	0
RPS6KA4	0.2148168	0.22203879	-0.00722196	-0.03748947	0.09549162	0.141035713	8	0	0
ACKR2	0.0615214	0.057704531	0.003816846	0.114338728	0.095576595	0.141149593	6	0	0
NBEAL1	0.0671123	0.057342614	0.009769672	0.102994229	0.095618858	0.141188547	10	0	0
AIF1	0.5141761	0.541049721	-0.02687358	-0.06302034	0.095636287	0.141188547	5	0	0
POMP	0.0604872	0.054519107	0.005968065	0.042828212	0.09562742	0.141188547	8	0	0
TRPC4	0.2709951	0.248144108	0.022850955	0.215087436	0.095641866	0.141188547	9	0	0
ZNF205	0.3642748	0.355244527	0.009030245	0.067155636	0.095642331	0.141188547	10	0	0
ZNF460	0.0312381	0.030157529	0.001080575	0.048692821	0.095693821	0.141252931	10	0	0
CRELD2	0.2623763	0.276635207	-0.01425889	-0.06474092	0.095712866	0.141269418	3	0	0
PLEK2	0.2823747	0.286064654	-0.00368999	0.047697656	0.095748119	0.141309821	12	0	0

VRK2	0.5057671	0.508796162	-0.0030291	-0.03264509	0.095792101	0.141339843	4	0	0
IL20RB	0.8832398	0.886601503	-0.00336168	-0.0051443	0.095785659	0.141339843	3	0	0
ADAM19	0.1929595	0.183103739	0.009855743	0.051004807	0.095787209	0.141339843	5	0	0
NEUROD4	0.3079954	0.303326983	0.00466846	0.094303431	0.095824639	0.141376221	5	0	0
CD3EAP	0.0569411	0.058438811	-0.00149772	-0.00894038	0.095844005	0.141393163	18	0	0
POLE3	0.1849492	0.18524274	-0.00029356	0.03036752	0.095862344	0.141408586	12	0	0
PMF1-BGLA	0.3123629	0.327984377	-0.01562145	-0.03041525	0.095920096	0.14145883	8	0	0
PMF1	0.3123629	0.327984377	-0.01562145	-0.03041525	0.095920096	0.14145883	8	0	0
OLA1	0.3928311	0.407293893	-0.01446279	-0.00312149	0.095914022	0.14145883	7	0	0
SMO	0.172653	0.160879098	0.011773934	0.355653876	0.095927952	0.14145883	11	0	0
MCTP2	0.0344933	0.034954426	-0.00046113	-0.01897978	0.095970114	0.141509368	7	0	0
SYNJ1	0.1176203	0.126091202	-0.00847088	-0.02661748	0.095990806	0.141528245	13	0	0
EHMT1	0.0683633	0.066105813	0.002257473	0.046389031	0.096001133	0.141531836	5	0	0
HNRNPU	0.119143	0.137574995	-0.01843199	-0.04160375	0.096048478	0.141578362	7	0	0
SPRYD4	0.2875012	0.287801457	-0.0003003	0.013047313	0.096043569	0.141578362	10	0	0
TRIM47	0.2721873	0.284621014	-0.01243368	-0.02905014	0.09616934	0.141744867	10	0	0
ULK4	0.2352932	0.232872356	0.002420892	-0.0263286	0.0962826	0.141900141	8	0	0
KDM4C	0.7686128	0.795797734	-0.02718491	-0.04951164	0.096400914	0.142051166	1	0	0
AARSD1	0.1023858	0.104487034	-0.00210119	-0.00278437	0.096396098	0.142051166	13	0	0
CCM2	0.2973469	0.30637596	-0.00902906	0.010633366	0.096461146	0.142128244	6	0	0
DENND1A	0.6468899	0.654264412	-0.00737453	-0.0234658	0.096484262	0.142150627	6	0	0
NEK11	0.0467357	0.041378765	0.005356906	0.103346813	0.096558443	0.142248232	14	0	0
RAB33B	0.0422971	0.046299717	-0.00400262	-0.0652527	0.09659857	0.14229566	10	0	0
EXT2	0.1642008	0.163844222	0.000356624	0.03129817	0.096661319	0.1423764	11	0	0
GFOD2	0.0354228	0.037242826	-0.00182003	-0.03460744	0.096675117	0.14238503	13	0	0
C8orf76	0.4157646	0.420200293	-0.00443571	-0.03126066	0.096737882	0.142461423	9	0	0
TOR2A	0.1700585	0.153088598	0.016969869	0.077612033	0.096742871	0.142461423	8	0	0
PKNOX1	0.1540259	0.157797764	-0.00377184	-0.0316657	0.096763815	0.142480566	9	0	0
DSCC1	0.33916	0.336791739	0.002368215	0.075585021	0.09681076	0.14253799	6	0	0
TRIP11	0.4713335	0.484964903	-0.01363138	0.008799521	0.096822043	0.142542901	4	0	0
FAM210A	0.1307798	0.144859774	-0.01407995	-0.03811133	0.096843615	0.142551258	7	0	0
ZNF616	0.2031419	0.195014733	0.008127142	0.137016322	0.096839004	0.142551258	9	0	0
GSDMA	0.8229807	0.831416049	-0.00843539	-0.0161971	0.096869885	0.142566653	7	0	0
HMSD	0.2315393	0.245063605	-0.01352434	-0.00476256	0.096869971	0.142566653	8	0	0
RSL1D1	0.1334941	0.139919923	-0.00642586	0.037373772	0.096890989	0.142585885	11	0	0
ZSCAN31	0.6035962	0.616094577	-0.01249834	-0.02249853	0.096912517	0.142605866	2	0	0
YIPF7	0.0305362	0.032086966	-0.00155081	-0.05914327	0.09697405	0.142684705	8	0	0
ARRDC1	0.1472038	0.142019704	0.005184055	0.050330931	0.097005721	0.142719597	8	0	0
ARHGAP5	0.1413747	0.160352222	-0.01897752	-0.06652972	0.097022032	0.14272018	6	0	0
DLG4	0.07491	0.081677576	-0.0067676	-0.02046503	0.097017105	0.14272018	11	0	0
WDR1	0.0223004	0.023047521	-0.00074709	-0.00637402	0.097058675	0.142743025	4	0	0
ZNF800	0.8091357	0.822680429	-0.01354472	-0.02310095	0.097059516	0.142743025	2	0	0
TRIB3	0.6530317	0.661351162	-0.00831951	0.008147798	0.097061437	0.142743025	4	0	0
ZNF436	0.1856169	0.195585357	-0.00996841	0.009479756	0.097091359	0.142770197	16	0	0
LYPD6	0.1396137	0.149986375	-0.01037264	0.051284942	0.097100916	0.142770197	8	0	0
FAM13C	0.0915187	0.080526519	0.010992216	0.149861707	0.097111753	0.142770197	10	0	0
IGFBP6	0.3369612	0.318281411	0.018679821	0.089174405	0.097110027	0.142770197	5	0	0
AGPAT4	0.0964864	0.086356921	0.010129479	0.108221097	0.097132502	0.142788997	11	0	0
HAPLN1	0.3450017	0.336338032	0.008663644	0.06110257	0.09714492	0.142792147	9	0	0
PDS5B	0.1345025	0.132982859	0.00151964	0.070501966	0.097150567	0.142792147	10	0	0
USP16	0.1966879	0.205809571	-0.00912165	0.013936893	0.097226297	0.142891746	13	0	0
TBCEL	0.2400697	0.259531522	-0.01946185	-0.02293135	0.097303236	0.142993105	9	0	0
ANP32D	0.7612687	0.778068637	-0.01679997	-0.0289778	0.097331894	0.143023501	5	0	0
P4HA1	0.0404183	0.041595373	-0.00117706	-0.01495363	0.097394593	0.143068749	8	0	0
MRPL12	0.0434672	0.048487714	-0.00502052	-0.03489613	0.097380756	0.143068749	10	0	0
SLC25A10	0.0434672	0.048487714	-0.00502052	-0.03489613	0.097380756	0.143068749	10	0	0
DLL3	0.1208319	0.115030604	0.005801343	0.190563611	0.097393276	0.143068749	11	0	0
PLAGL1	0.3076913	0.315487881	-0.00779653	0.080184433	0.097432406	0.143107312	19	0	0
TAF4	0.1081421	0.086287656	0.021854464	0.174177968	0.097436803	0.143107312	7	0	0
DMXL2	0.093147	0.101584413	-0.00843743	-0.05470316	0.097464608	0.14313643	9	0	0
VPS9D1	0.0244409	0.025827924	-0.00138701	-0.04028388	0.097533681	0.143226143	8	0	0
B3GNT2	0.2106408	0.219489572	-0.00884881	0.05542715	0.097554014	0.143244274	9	0	0
KCNMB4	0.0962053	0.100616128	-0.00441078	-0.0326993	0.097569547	0.143255353	7	0	0

CYB5R2	0.393628	0.345746936	0.047881093	0.182175032	0.097726873	0.143474601	1	0	0
COL22A1	0.155955	0.14082921	0.015125834	0.243478687	0.097798668	0.143568252	7	0	0
SNRPG	0.1932129	0.206751683	-0.01353878	-0.03233501	0.097849991	0.143596584	11	0	0
BAK1	0.0411702	0.042799067	-0.00162885	-0.0709775	0.097837409	0.143596584	4	0	0
RRAS	0.0555077	0.058402416	-0.00289467	-0.01469077	0.097845413	0.143596584	10	0	0
ARFRP1	0.2206062	0.233534768	-0.01292853	-0.01371322	0.097844114	0.143596584	14	0	0
GPRC5B	0.2376342	0.211087346	0.026546902	0.258787603	0.097879003	0.143627408	8	0	0
CDC26	0.1649118	0.174078659	-0.00916689	0.005202749	0.097988801	0.143776761	12	0	0
NDUFA2	0.0726856	0.072463691	0.000221915	0.015159225	0.098030164	0.143825687	13	0	0
CCNI2	0.1513906	0.138356699	0.01303393	0.112933946	0.098050555	0.143843838	8	0	0
ICAM1	0.0866646	0.071444524	0.01522009	0.314865438	0.098106255	0.14391378	13	0	0
C21orf91	0.1226418	0.14277382	-0.02013206	-0.04314924	0.098114351	0.143913887	6	0	0
AMIGO1	0.1194761	0.12326347	-0.00378734	0.075274802	0.098310141	0.144189279	9	0	0
DPT	0.8044758	0.795421786	0.009054021	0.015229366	0.098326262	0.144201131	4	0	0
FMNL3	0.6129561	0.610061559	0.002894577	0.011782195	0.098345313	0.144217278	3	0	0
TMEM147	0.1045296	0.107628657	-0.00309902	0.185247121	0.098427658	0.144326232	9	0	0
SYNPR	0.6142474	0.645409642	-0.0311622	-0.07145264	0.098475881	0.144385138	2	0	0
LCE3B	0.5437345	0.603982229	-0.06024775	-0.14900255	0.098543842	0.144443071	1	0	0
ST20-MTHF:	0.2777329	0.278316722	-0.00058379	0.020677118	0.098547721	0.144443071	7	0	0
ST20	0.2777329	0.278316722	-0.00058379	0.020677118	0.098547721	0.144443071	7	0	0
ZNF18	0.3584912	0.360341907	-0.00185071	0.006522881	0.098531105	0.144443071	6	0	0
ZNF433	0.0767592	0.058273557	0.018485606	0.362957955	0.098555659	0.144443071	16	0	0
LEF1	0.2067798	0.223489615	-0.01670986	-0.05719821	0.098601591	0.144486776	9	0	0
VSIG2	0.5952809	0.572039973	0.023240899	0.058135965	0.098599512	0.144486776	5	0	0
SPRTN	0.2806355	0.313663776	-0.03302828	-0.08891997	0.098651186	0.144537388	11	0	0
GOLGA5	0.137157	0.147886919	-0.01072989	0.000873397	0.098652247	0.144537388	10	0	0
VPS37A	0.126788	0.116876162	0.009911848	0.029045495	0.098696397	0.144590261	10	0	0
CAPN14	0.089477	0.074445101	0.015031938	0.285473956	0.09876207	0.144674656	12	0	0
SMPDL3B	0.220189	0.222011577	-0.00182256	0.014540852	0.098836966	0.144772546	6	0	0
CRELD1	0.0597481	0.062100917	-0.00235285	-0.01440662	0.098924094	0.144888335	7	0	0
TRUB1	0.2441515	0.2540067	-0.00985515	-0.02039094	0.098934896	0.144892324	11	0	0
SHKBP1	0.1291452	0.133836668	-0.00469148	-0.00303658	0.09897664	0.144941624	12	0	0
LYSMD1	0.2036195	0.217476782	-0.01385724	-0.01033759	0.09900306	0.144968477	14	0	0
SUOX	0.1949418	0.199367519	-0.00442576	-0.04694292	0.099043969	0.145016541	15	0	0
BECN1	0.134606	0.143206046	-0.00860002	0.006461837	0.099072852	0.14504699	11	0	0
COPE	0.0397905	0.040565991	-0.00077549	-0.00917415	0.09921386	0.145229723	12	0	0
DDX49	0.0397905	0.040565991	-0.00077549	-0.00917415	0.09921386	0.145229723	12	0	0
RPL3	0.3918727	0.417007497	-0.02513482	0.000109033	0.099330462	0.145388539	4	0	0
MRPL2	0.1320423	0.13424296	-0.00220067	-0.00882599	0.099353131	0.145409853	17	0	0
PCGF5	0.2780141	0.283626246	-0.00561212	0.003560593	0.099388142	0.145449226	15	0	0
URGCP-MRI	0.3634251	0.363397035	2.80E-05	0.007440257	0.099407023	0.145464988	7	0	0
INTS3	0.1472679	0.149933043	-0.00266517	0.04007987	0.099417866	0.145468987	8	0	0
BOD1	0.185094	0.193690733	-0.00859673	-0.01015683	0.099436651	0.145483449	10	0	0
MAP2K6	0.1663458	0.171646936	-0.00530117	0.024218892	0.099443972	0.145483449	12	0	0
RC3H2	0.1128321	0.121329366	-0.00849724	-0.04970598	0.099470361	0.145510186	10	0	0
APBA1	0.1058732	0.099430027	0.006443157	0.216676489	0.099511221	0.145558087	12	0	0
PRIM1	0.263835	0.266929228	-0.00309425	0.028398024	0.099549326	0.145590078	15	0	0
CSPG4	0.3761826	0.352069099	0.024113524	0.195197924	0.099616456	0.145676377	10	0	0
CHMP1A	0.1210532	0.122327626	-0.00127439	0.032203702	0.099738535	0.14584301	9	0	0
HIST1H1T	0.9056225	0.911260272	-0.00563778	-0.00882296	0.099797699	0.145917627	7	0	0
TIGD5	0.0480886	0.045590537	0.002498058	0.034211118	0.099881519	0.146016376	9	0	0
TMEM126B	0.1052417	0.090804731	0.014437001	0.064021964	0.099880652	0.146016376	16	0	0
C2orf72	0.4598855	0.468154959	-0.00826949	0.059958618	0.099950937	0.146087983	4	0	0
RELL2	0.1486702	0.157846389	-0.00917623	-0.03359362	0.09994554	0.146087983	10	0	0
PNP	0.4045258	0.414983567	-0.01045781	-0.02385224	0.099954936	0.146087983	9	0	0
KLHL24	0.1623498	0.172356378	-0.01000658	-0.02183554	0.100049956	0.146196383	11	0	0
DHRS12	0.1022374	0.092568075	0.009669372	0.090642583	0.100053557	0.146196383	10	0	0
DMPK	0.4593737	0.458459203	0.000914452	0.02903765	0.100046946	0.146196383	5	0	0
CASP7	0.1973248	0.197917618	-0.0005928	0.03138121	0.100063113	0.146198435	11	0	0
SLC25A34	0.6131154	0.609407229	0.003708145	0.003672697	0.100100543	0.146217391	9	0	0
MREG	0.8577126	0.868298074	-0.01058544	-0.01719105	0.100094341	0.146217391	2	0	0
ZNF721	0.1754782	0.163262335	0.012215888	0.031055409	0.100099132	0.146217391	10	0	0
CERS2	0.2308162	0.236104459	-0.0052883	0.177982192	0.100125234	0.146241548	10	0	0

COQ4	0.121331	0.128300209	-0.00696919	-0.04073019	0.100192547	0.146327947	17	0	0
RNF103	0.4232479	0.428309882	-0.00506194	-0.00195243	0.100235471	0.146357253	9	0	0
LCORL	0.115199	0.115934526	-0.00073556	0.068771896	0.100237092	0.146357253	11	0	0
CPNE2	0.1407759	0.124148215	0.016627639	0.134022806	0.100226019	0.146357253	8	0	0
C1QL1	0.1774808	0.151846238	0.025634601	0.283823644	0.10026539	0.146386653	9	0	0
TNRC6A	0.0446979	0.04724225	-0.00254431	-0.05258097	0.100286405	0.146405417	10	0	0
GIMAP6	0.4422265	0.446955938	-0.00472944	-0.008672	0.100325202	0.146450136	4	0	0
COPRS	0.0995947	0.103699981	-0.00410529	0.000793453	0.100334834	0.146452277	7	0	0
LRRC15	0.3139312	0.325656145	-0.01172495	0.018767126	0.100400927	0.1465249	9	0	0
CCZ1B	0.4796709	0.518796539	-0.03912569	-0.07666601	0.100397049	0.1465249	3	0	0
PDZK1	0.7105513	0.695591345	0.014959974	0.039083268	0.100414751	0.146533152	4	0	0
TTC14	0.2326968	0.237870362	-0.00517359	0.190492142	0.100479815	0.14661617	9	0	0
DHH	0.2873118	0.277971333	0.009340422	0.182928302	0.100547791	0.146703424	11	0	0
ZNF808	0.0715987	0.049305969	0.02229273	0.51066923	0.100572166	0.146727051	6	0	0
HECW2	0.208638	0.196329431	0.012308576	0.325319095	0.100677856	0.146869299	5	0	0
CYP2R1	0.1209647	0.117047415	0.003917326	-0.0093681	0.100718659	0.146881036	11	0	0
NOL3	0.1710853	0.158785371	0.012299953	0.213108063	0.100710799	0.146881036	13	0	0
RFNG	0.165444	0.175848361	-0.01040436	0.002990843	0.100694577	0.146881036	9	0	0
KMT2B	0.0459584	0.035002592	0.010955831	0.368683554	0.100706946	0.146881036	5	0	0
GNS	0.094913	0.10987894	-0.01496597	-0.03727113	0.100743577	0.14690543	13	0	0
GATC	0.0992411	0.099350183	-0.00010912	0.025734372	0.100791958	0.146964031	15	0	0
JARID2	0.0544569	0.049840209	0.004616671	0.072400987	0.100866509	0.147048826	14	0	0
FTO	0.1006553	0.106747415	-0.00609215	-0.02035054	0.1008592	0.147048826	14	0	0
AKAP11	0.0507016	0.048164882	0.002536702	0.054840482	0.100885175	0.147064084	6	0	0
MSRB3	0.190125	0.18925488	0.000870161	0.267763929	0.100986405	0.147199687	11	0	0
COG8	0.10268	0.112344083	-0.00966409	-0.03001397	0.100995543	0.147201044	11	0	0
HIRIP3	0.1195794	0.12765463	-0.00807518	-0.01223706	0.101020669	0.147225702	13	0	0
C17orf64	0.0975203	0.096474849	0.001045405	0.041494546	0.101030261	0.147227719	8	0	0
HAUS1	0.1338931	0.141849876	-0.00795681	-0.02683302	0.101087535	0.147299214	8	0	0
SUPV3L1	0.1058727	0.106568318	-0.0006956	0.001018985	0.101104554	0.147312046	12	0	0
C1R	0.2374557	0.226254626	0.011201046	0.138558337	0.101167126	0.147391242	9	0	0
KLF15	0.0367259	0.02968255	0.007043377	0.119039824	0.101246887	0.147495465	6	0	0
MAST4	0.2252877	0.228360442	-0.00307273	-0.00337592	0.101293142	0.147550864	11	0	0
NAALADL1	0.6337632	0.639096249	-0.00533301	-0.01163079	0.101303484	0.147553945	7	0	0
GOLGB1	0.0485841	0.050445599	-0.00186148	-0.04563318	0.101380733	0.147654173	11	0	0
CTRL	0.8080684	0.813969101	-0.00590069	-0.01237134	0.10138876	0.147654173	10	0	0
EIF1B	0.0836591	0.089280858	-0.00562177	0.002467438	0.101407488	0.147669457	8	0	0
FERMT3	0.4633785	0.486242621	-0.02286413	-0.05772944	0.101450033	0.147719419	11	0	0
03-Sep	0.0694274	0.076051793	-0.00662444	-0.04645132	0.101496576	0.147775192	4	0	0
LMAN2	0.1981642	0.210171148	-0.01200695	-0.01890031	0.101566292	0.147855117	15	0	0
PDE4C	0.2281888	0.246476222	-0.01828738	-0.10671928	0.101567958	0.147855117	1	0	0
ATP2B1	0.0317873	0.032672879	-0.00088557	-0.00488714	0.101593777	0.1478807	4	0	0
NPPC	0.0926744	0.071891815	0.020782609	0.345750418	0.101704924	0.147974534	9	0	0
MMP20	0.7265177	0.765349973	-0.0388323	-0.0731143	0.101690676	0.147974534	3	0	0
FNTB	0.1141948	0.117464113	-0.00326931	0.005304658	0.101698557	0.147974534	15	0	0
JMJD7	0.1817608	0.186947586	-0.00518681	-0.00620202	0.101722721	0.147974534	10	0	0
PLA2G4B	0.1817608	0.186947586	-0.00518681	-0.00620202	0.101722721	0.147974534	10	0	0
JMJD7-PLA2	0.1817608	0.186947586	-0.00518681	-0.00620202	0.101722721	0.147974534	10	0	0
SYNRG	0.064737	0.069109907	-0.00437287	-0.01381724	0.101701955	0.147974534	10	0	0
HAP1	0.1832453	0.175489734	0.007755576	0.299671005	0.101724242	0.147974534	7	0	0
SMG6	0.1664779	0.172916543	-0.00643863	-0.02294658	0.101757557	0.148010992	7	0	0
ARID4A	0.0528649	0.0528711	-6.23E-06	0.015843152	0.101863049	0.14815242	16	0	0
COMMD3-B	0.1665447	0.178064367	-0.01151968	-0.03192203	0.101881247	0.148166874	10	0	0
NUDCD2	0.0377753	0.037546065	0.000229233	0.008142308	0.101900262	0.148182511	15	0	0
ERP44	0.1292745	0.13760138	-0.00832693	-0.0703023	0.101931711	0.148216228	11	0	0
ARHGEF37	0.0360611	0.035844452	0.000216611	0.006220881	0.101956	0.148239527	11	0	0
ZNF850	0.0781696	0.062824825	0.015344761	0.309281103	0.102013277	0.148310783	4	0	0
TFDP1	0.202653	0.204432894	-0.00177994	0.182381465	0.102096718	0.148396007	5	0	0
ATP2A3	0.0413755	0.035349873	0.006025586	0.244707128	0.102091798	0.148396007	7	0	0
LSR	0.0960651	0.107474746	-0.01140964	-0.02974536	0.102087943	0.148396007	10	0	0
ATOX1	0.0350632	0.034015593	0.001047614	0.030916371	0.102128068	0.14841752	6	0	0
ERLIN2	0.1328084	0.132142354	0.000666074	0.068939774	0.102121353	0.14841752	10	0	0
KCND3	0.104155	0.089351384	0.014803633	0.233505474	0.10215335	0.148442233	17	0	0

KRI1	0.2980217	0.290203858	0.007817853	0.060933552	0.102165747	0.148448221	9	0	0
LSM11	0.1937993	0.194822505	-0.00102324	0.023505669	0.10223512	0.148536988	5	0	0
DERL2	0.0448195	0.045208885	-0.00038935	-0.00024841	0.102424103	0.148799507	8	0	0
LIN37	0.2500207	0.264883816	-0.01486314	-0.01871736	0.102432451	0.148799582	13	0	0
UPF3A	0.1176118	0.13722368	-0.01961192	-0.07661243	0.102489867	0.148870931	3	0	0
OR4M2	0.5236188	0.555653003	-0.03203423	-0.0741501	0.102512297	0.148891453	2	0	0
PMEL	0.0565455	0.052859215	0.003686306	0.050935884	0.102533751	0.148910555	16	0	0
PMVK	0.0684479	0.072321238	-0.00387337	-0.02643174	0.102547986	0.148912889	11	0	0
MKRN2	0.1922088	0.205131831	-0.01292301	-0.03735411	0.102551963	0.148912889	12	0	0
ENPP1	0.0923577	0.096165997	-0.00380825	0.004737186	0.102598953	0.148969062	12	0	0
RPUSD2	0.1461813	0.145150114	0.001031201	0.086151699	0.102640277	0.149016999	12	0	0
KAT7	0.0772391	0.064239063	0.013000066	0.182392711	0.102662762	0.14903758	11	0	0
CMC1	0.1557364	0.159304904	-0.00356847	-0.03019283	0.102680695	0.149051549	11	0	0
OAZ2	0.2965036	0.290171048	0.006332512	0.03198029	0.102711431	0.1490841	15	0	0
TAS2R4	0.8807051	0.88834184	-0.00763675	-0.01256551	0.102723495	0.149089546	3	0	0
ALG8	0.0339501	0.032142961	0.001807098	0.065536929	0.102754293	0.149122179	9	0	0
USP37	0.0468493	0.048096329	-0.00124704	-0.01355941	0.10286121	0.149265265	10	0	0
TMEM200B	0.1212645	0.098010101	0.02325443	0.314249439	0.10288001	0.149280467	9	0	0
PDCD10	0.0519847	0.050393712	0.001590945	0.024207869	0.102991426	0.149409472	11	0	0
SERPINI1	0.0519847	0.050393712	0.001590945	0.024207869	0.102991426	0.149409472	11	0	0
SMR3A	0.7394808	0.747119569	-0.00763877	-0.01605449	0.102993906	0.149409472	2	0	0
ATRAID	0.0817059	0.092821343	-0.01111542	-0.03496204	0.10301891	0.149433657	14	0	0
TP53BP1	0.0769013	0.075127664	0.001773631	0.031073329	0.103028166	0.149434999	15	0	0
LRPAP1	0.2843	0.288943434	-0.00464346	0.002591959	0.103041012	0.149441545	8	0	0
ITGA5	0.1528585	0.141359096	0.011499452	0.135261904	0.103058457	0.149454761	12	0	0
CABP1	0.0738258	0.072601925	0.001223863	0.069537609	0.10307353	0.149464534	9	0	0
PCSK5	0.0478253	0.041236223	0.006589076	0.196463682	0.103112041	0.149508292	8	0	0
DDX27	0.0674806	0.067067388	0.000413245	0.046473248	0.103176109	0.149589095	11	0	0
WARS2	0.1661988	0.184615662	-0.0184169	-0.05557104	0.103193581	0.149602333	10	0	0
TRNT1	0.1182418	0.126021892	-0.00778012	-0.05308207	0.103213294	0.149618819	7	0	0
JAM2	0.1231249	0.120532224	0.002592662	0.352521534	0.103294185	0.149723978	9	0	0
DPH5	0.1207662	0.130432593	-0.00966638	-0.04561162	0.103366796	0.149814414	9	0	0
GRINA	0.2401914	0.245229564	-0.00503815	0.014342898	0.103373282	0.149814414	8	0	0
TCF20	0.0806244	0.068657869	0.011966504	0.176242844	0.103440187	0.149899265	3	0	0
RASD2	0.0676942	0.068435765	-0.00074153	0.06685835	0.103490525	0.149960095	12	0	0
YY1AP1	0.1103579	0.132588345	-0.02223047	-0.05463892	0.103520875	0.149991955	13	0	0
NFATC4	0.4082369	0.398193447	0.010043486	0.19476271	0.103670741	0.150196964	4	0	0
SERPINB4	0.7461715	0.787796969	-0.04162551	-0.07730824	0.103760452	0.150314794	1	0	0
BAAT	0.8225298	0.830265898	-0.00773609	-0.01279681	0.103777933	0.150327976	3	0	0
CHRNA7	0.0483633	0.041527102	0.006836196	0.173480632	0.103847176	0.150416131	15	0	0
ARPC2	0.199836	0.207881306	-0.00804533	0.029402054	0.103899551	0.150477029	9	0	0
TMEM120B	0.0345448	0.034775634	-0.00023081	-0.00010955	0.103905999	0.150477029	5	0	0
GADD45A	0.0721637	0.080274807	-0.00811108	-0.00373437	0.103973243	0.15055353	8	0	0
ELK3	0.2000957	0.208786815	-0.00869111	-0.04053507	0.103975612	0.15055353	10	0	0
ZNF282	0.1649724	0.178933073	-0.01396065	-0.02595189	0.104037365	0.150630786	12	0	0
ASAH1	0.251183	0.252884451	-0.00170144	0.02219904	0.104098851	0.150707643	13	0	0
MOCOS	0.1327517	0.131686116	0.001065571	0.109369549	0.104124179	0.150732145	13	0	0
CDC5L	0.1005546	0.11520059	-0.01464597	-0.06915604	0.104145786	0.150751256	8	0	0
SLC17A7	0.137071	0.145300658	-0.00822966	-0.03442234	0.104171216	0.150775898	7	0	0
CCND2	0.0621353	0.052199132	0.009936137	0.326705528	0.104225168	0.150841815	33	0	0
BEAN1	0.2068527	0.204682124	0.002170536	0.134059877	0.104264352	0.15088635	5	0	0
ARL6IP1	0.0597087	0.064504377	-0.00479564	-0.05098693	0.104335214	0.150976716	12	0	0
FBXO21	0.1965263	0.213416372	-0.01689008	-0.08445959	0.104388606	0.15104179	10	0	0
SIAH2	0.1462898	0.147031778	-0.00074199	0.013865976	0.104413865	0.151066151	8	0	0
HIST1H3J	0.0402982	0.040147426	0.000150736	-0.01303427	0.10442929	0.151076282	9	0	0
DUSP7	0.0703245	0.063788857	0.006535626	0.063717554	0.104441032	0.151081083	8	0	0
PABPC1	0.1577083	0.181568023	-0.02385971	-0.03913327	0.104509565	0.151168029	4	0	0
ARMC10	0.1406634	0.145580737	-0.00491729	-0.0010979	0.104542553	0.151203551	15	0	0
MED27	0.1526656	0.163306125	-0.01064048	-0.03390292	0.104598616	0.151272439	8	0	0
SIN3A	0.0469988	0.045375381	0.001623444	0.043156015	0.104610003	0.151276708	8	0	0
SP2	0.0646846	0.063412432	0.001272217	0.043732151	0.104728217	0.151435448	12	0	0
MTDH	0.0938437	0.105617459	-0.01177377	-0.01197869	0.104811545	0.151531507	10	0	0
ZNF579	0.1313959	0.141712793	-0.01031693	0.046189304	0.104807834	0.151531507	6	0	0

PTN	0.1660568	0.150617977	0.015438801	0.212560439	0.104859288	0.151588312	10	0	0
ZNF689	0.7606709	0.783929673	-0.02325874	-0.04289623	0.104886141	0.151614911	1	0	0
U2AF1	0.1251315	0.132050315	-0.0069188	0.008780571	0.104915128	0.151644591	9	0	0
SLC26A2	0.0706347	0.066448985	0.004185692	0.044181008	0.104929855	0.151653657	10	0	0
TMEM134	0.1785101	0.182264765	-0.00375467	0.015998364	0.104949762	0.151670206	14	0	0
LAMC3	0.264149	0.270602132	-0.00645312	0.103445166	0.104974783	0.151671963	7	0	0
ZFYVE19	0.2519776	0.259478702	-0.00750108	-0.02065188	0.104976346	0.151671963	15	0	0
NFATC1	0.0666174	0.061135589	0.005481802	0.060402217	0.104971553	0.151671963	5	0	0
VCAN	0.120855	0.115850063	0.005004985	0.141520472	0.10500302	0.151698281	13	0	0
LRRC58	0.0662674	0.070501059	-0.00423365	-0.03901488	0.105012881	0.151700309	8	0	0
EED	0.1478678	0.153268601	-0.00540078	0.01649461	0.105127359	0.151853451	13	0	0
GLI1	0.0642833	0.073603317	-0.00932007	-0.08827773	0.105196717	0.151941401	7	0	0
MPI	0.2623014	0.267269047	-0.0049676	-0.03336283	0.105208992	0.151946894	11	0	0
PIK3AP1	0.0790261	0.0783987	0.000627377	0.171808495	0.105244555	0.151986018	10	0	0
MTURN	0.0547004	0.050841545	0.003858846	0.092298502	0.105281287	0.152026822	8	0	0
CUL9	0.2375396	0.270045983	-0.03250638	-0.08005653	0.1053889	0.152169966	4	0	0
HADH	0.2373958	0.253583282	-0.01618749	-0.00764674	0.105424597	0.152209254	11	0	0
PA2G4	0.1817645	0.188883199	-0.00711875	0.012289575	0.105552425	0.15236928	10	0	0
URB1	0.1591539	0.163917557	-0.00476368	-0.05103792	0.105548955	0.15236928	10	0	0
ZNF142	0.2307124	0.242446864	-0.0117345	-0.01043249	0.105612466	0.152443682	16	0	0
MMP14	0.4160637	0.403154615	0.012909105	0.130381624	0.105660651	0.15250096	9	0	0
SEC23B	0.0866927	0.09372465	-0.00703191	-0.0121393	0.105727371	0.152584979	15	0	0
PSMB8	0.1663612	0.174980612	-0.00861945	-0.04974765	0.105738286	0.152588455	34	0	0
SLMAP	0.0287374	0.027256305	0.001481132	0.0562439	0.105816554	0.152689116	1	0	0
GIMAP2	0.0608456	0.050031497	0.010814127	0.207607456	0.105849445	0.15272429	2	0	0
ZSWIM8	0.268369	0.281069062	-0.0127001	-0.02718181	0.105858338	0.152724835	8	0	0
TMTC4	0.1046535	0.106684764	-0.00203131	0.02617723	0.105877567	0.152740292	9	0	0
HARBI1	0.1211346	0.135676577	-0.01454201	-0.01742228	0.10591651	0.152784183	7	0	0
MRPL9	0.2740494	0.295924728	-0.02187534	-0.05983224	0.106011971	0.152909586	8	0	0
IRF2	0.0445899	0.046734341	-0.00214439	-0.0371914	0.106038746	0.152935908	6	0	0
PSMB5	0.2533327	0.252819252	0.00051344	0.033843151	0.106098779	0.152995865	16	0	0
MRPL28	0.3779088	0.379545264	-0.00163642	0.032954681	0.106089004	0.152995865	12	0	0
LSM14B	0.1607594	0.183711259	-0.02295187	-0.05260222	0.106105908	0.152995865	5	0	0
BEGAIN	0.1632511	0.153151399	0.010099685	0.127589579	0.106128224	0.153015741	4	0	0
ARHGEF1	0.3168799	0.300760874	0.016118982	0.357127778	0.106183138	0.153082611	8	0	0
SEMA6D	0.3316272	0.315476995	0.016150195	0.201922472	0.106213664	0.153114312	6	0	0
TLX1	0.5656579	0.578393464	-0.01273555	-0.02862046	0.106284524	0.153204148	4	0	0
MMS19	0.0560731	0.050444798	0.00562835	0.097522541	0.106344636	0.153278478	14	0	0
PELI3	0.305544	0.310058561	-0.00451455	0.011138262	0.10651649	0.153513842	14	0	0
CNTN1	0.1802373	0.188547609	-0.00831034	0.074769114	0.106532549	0.153524651	9	0	0
NPAS1	0.064762	0.058598147	0.006163823	0.141993539	0.106602501	0.153613116	3	0	0
ABCD2	0.0497247	0.053272992	-0.00354825	-0.08353421	0.106617459	0.153622329	9	0	0
FKBP6	0.8549816	0.870997802	-0.01601625	-0.03766981	0.106651134	0.153658506	8	0	0
RPGRIP1L	0.1096335	0.108865931	0.000767593	-0.00988686	0.106676962	0.15367103	15	0	0
ARID3A	0.0867735	0.097177545	-0.01040401	-0.06311496	0.106671996	0.15367103	5	0	0
GLT8D1	0.028279	0.027691887	0.000587139	0.031953113	0.106723538	0.153725778	7	0	0
BCAS1	0.7074667	0.711126691	-0.00366002	-0.00475484	0.106752863	0.153755669	6	0	0
LPIN1	0.4094908	0.410135308	-0.00064449	0.002718877	0.106802457	0.153814748	9	0	0
PMPCB	0.239711	0.246133105	-0.00642209	-0.04180662	0.106890208	0.153928765	9	0	0
TNRC18	0.0487861	0.042348128	0.006437983	0.11574601	0.106933193	0.153965942	10	0	0
ACP2	0.0415131	0.043213216	-0.00170011	-0.04340058	0.106925077	0.153965942	17	0	0
NUP54	0.1764848	0.181126184	-0.00464134	-0.01722504	0.106999667	0.154049288	6	0	0
LSM10	0.377111	0.377039944	7.10E-05	-0.04393082	0.107086427	0.154161824	7	0	0
ITIH1	0.7439655	0.740018393	0.003947078	0.009552769	0.1071012	0.154170717	7	0	0
ALLC	0.7945358	0.807967638	-0.01343179	-0.0261715	0.107149642	0.15422807	7	0	0
ZDHHC7	0.160018	0.167193407	-0.00717537	0.004025176	0.107210631	0.154303473	7	0	0
LIN54	0.1440045	0.129034344	0.014970127	0.11190585	0.107288722	0.154403475	7	0	0
SCLY	0.1263122	0.131339468	-0.00502727	-0.01556032	0.107304838	0.154414278	10	0	0
SMARCAD1	0.1155972	0.125172516	-0.00957535	0.025634653	0.107384934	0.154517142	12	0	0
MRPS9	0.1425622	0.148001227	-0.00543908	-0.03533704	0.107423977	0.154560922	9	0	0
NSUN2	0.0578793	0.056822354	0.001056923	-0.00112506	0.107505643	0.154666016	10	0	0
EMP1	0.3160695	0.311746406	0.004323068	0.186014627	0.107526787	0.154684027	8	0	0
DGCR2	0.1748626	0.169616481	0.005246152	0.079672266	0.107611434	0.154793382	6	0	0

APCDD1	0.3217684	0.312617368	0.009151047	0.075020625	0.107660736	0.154839465	9	0	0
SLC39A1	0.0898829	0.072366179	0.017516717	0.166510437	0.107710949	0.154899261	20	0	0
FSCN1	0.1342012	0.136573439	-0.00237222	0.077058323	0.107725	0.154907047	9	0	0
SARNP	0.0700896	0.075439971	-0.00535032	-0.02613911	0.107813975	0.155022564	18	0	0
AKAP7	0.3726368	0.377411014	-0.00477423	0.012127186	0.10786818	0.15507564	9	0	0
CYCS	0.1252787	0.137309874	-0.01203114	-0.03702795	0.107865277	0.15507564	9	0	0
MST1	0.0438574	0.041467627	0.00238979	0.065479767	0.107909934	0.155119831	1	0	0
TUBGCP5	0.0811047	0.095409819	-0.01430513	-0.16618862	0.107916216	0.155119831	11	0	0
PROCR	0.2617246	0.25873441	0.002990223	0.089870578	0.107991685	0.155215872	9	0	0
IRAK1BP1	0.2014346	0.207141249	-0.00570663	0.015096326	0.108000789	0.155216518	12	0	0
CELSR3	0.0731468	0.059137084	0.014009683	0.337878429	0.108029532	0.155245387	16	0	0
TRMT61A	0.1319793	0.13605913	-0.00407979	-0.05134472	0.108065432	0.155272097	10	0	0
GADD45B	0.0390699	0.039899372	-0.00082946	-0.01274135	0.10806206	0.155272097	11	0	0
TMEM130	0.1842205	0.194460329	-0.01023986	-0.01088123	0.108135323	0.155360073	5	0	0
SRSF5	0.2843007	0.272445229	0.011855485	0.188075472	0.108402272	0.155731129	9	0	0
RANGAP1	0.050995	0.050380482	0.000614498	0.006647236	0.108442921	0.155777048	13	0	0
NCOA3	0.0452218	0.049597281	-0.00437545	-0.0172095	0.108472237	0.155806682	12	0	0
TNFRSF10B	0.0293607	0.025539053	0.003821635	0.169448685	0.108510621	0.155849335	8	0	0
PROKR1	0.0511976	0.03542751	0.015770065	0.502184971	0.108537624	0.155875636	6	0	0
ATP6V1G1	0.220262	0.235987879	-0.0157259	-0.01932376	0.108586056	0.155932707	7	0	0
PPP2R3C	0.0574919	0.058034563	-0.00054267	-0.00851617	0.108617554	0.155965452	16	0	0
PDCL3	0.3802733	0.388058201	-0.00778495	-0.03690876	0.108699795	0.156058554	7	0	0
GRIN2D	0.0716107	0.06495118	0.006659503	0.207330081	0.108718154	0.156072419	13	0	0
CWC27	0.0423664	0.039440761	0.0029256	0.069762616	0.108914125	0.156341236	11	0	0
ANP32B	0.1129736	0.123406524	-0.01043294	0.102750329	0.108952599	0.156383949	8	0	0
ZNF791	0.0841258	0.088271205	-0.00414538	-0.01466439	0.108966878	0.156391928	14	0	0
CSDC2	0.3413037	0.339152525	0.00215115	0.018559587	0.109033815	0.156475477	7	0	0
RBMS1	0.1727547	0.170491502	0.002263195	0.063564043	0.109138253	0.156612826	15	0	0
PSMD13	0.0474555	0.047850994	-0.00039551	-0.00475292	0.10918024	0.156660543	6	0	0
MYCL	0.0510823	0.054557871	-0.00347556	-0.04993371	0.109195723	0.156670225	7	0	0
FJX1	0.1268618	0.112960884	0.01390091	0.163483753	0.109390832	0.156930724	10	0	0
KIF3B	0.2084377	0.22857895	-0.02014129	-0.04325807	0.109394784	0.156930724	14	0	0
SMIM15	0.1511316	0.16331007	-0.01217843	-0.00339098	0.109486293	0.157049437	9	0	0
CCNA2	0.1663184	0.168164594	-0.00184617	0.008923057	0.10951313	0.157075369	10	0	0
FBXW2	0.1381693	0.141944353	-0.00377503	-0.01255293	0.109582375	0.15716212	10	0	0
SNX21	0.2266693	0.220916073	0.005753202	0.09998616	0.109653158	0.157251063	10	0	0
TAX1BP3	0.0786667	0.088630567	-0.00996382	-0.02081963	0.109721689	0.157336761	11	0	0
HIST1H2BD	0.0518951	0.046667711	0.005227433	0.112342635	0.10974431	0.157356618	8	0	0
CDCA2	0.0369471	0.036940993	6.14E-06	0.009343899	0.109772557	0.157384538	16	0	0
MAN2C1	0.0621011	0.067717586	-0.00561646	-0.03522819	0.109791449	0.157399044	12	0	0
KIAA0513	0.2276169	0.227442652	0.000174229	-0.01546247	0.109928487	0.157582907	9	0	0
C17orf75	0.0768869	0.074306756	0.002580141	0.036338293	0.109972466	0.157633354	11	0	0
TRIM45	0.4441429	0.439553075	0.004589856	0.090959269	0.110015348	0.157651089	9	0	0
DPM2	0.2985962	0.317966007	-0.01936977	0.010803733	0.110010033	0.157651089	6	0	0
PPP1R14B	0.2168689	0.213342111	0.003526751	0.043453487	0.11001692	0.157651089	9	0	0
TSC2	0.2054708	0.200982923	0.004487847	0.054048793	0.110019998	0.157651089	12	0	0
HP1BP3	0.0251384	0.024142207	0.000996158	0.037482564	0.110076194	0.157719014	5	0	0
LRP6	0.1592952	0.153996425	0.005298786	0.049532002	0.110104249	0.15774661	8	0	0
UPK2	0.0587188	0.052290251	0.006428505	0.119415733	0.110144119	0.157791128	6	0	0
POFUT2	0.2334657	0.209802811	0.02366287	0.077976417	0.110199394	0.157857706	7	0	0
DGKE	0.1407951	0.14531963	-0.00452451	-0.07517259	0.110218116	0.157871915	6	0	0
NUFIP2	0.0622701	0.065500718	-0.00323066	-0.06651496	0.110313491	0.157995909	10	0	0
C1orf174	0.051606	0.048664234	0.002941805	0.053219409	0.110323919	0.157998228	7	0	0
FAM216A	0.0478376	0.045580895	0.002256738	0.055309356	0.110359378	0.158036391	16	0	0
PAFAH2	0.1730217	0.181995429	-0.00897369	-0.02220124	0.110381119	0.158054905	13	0	0
ARHGAP26	0.0459478	0.045274862	0.000672952	0.023141284	0.11041281	0.158087663	8	0	0
MEPCE	0.079901	0.066328744	0.013572241	0.037816613	0.110490756	0.158186638	8	0	0
AASDH	0.0885212	0.099036953	-0.01051578	-0.07769347	0.110512684	0.158192777	16	0	0
RGR	0.4838949	0.513275354	-0.02938049	-0.08419367	0.11050398	0.158192777	4	0	0
PIGG	0.0837129	0.085790378	-0.00207749	-0.02091924	0.11052781	0.15819776	6	0	0
TRIM50	0.9029036	0.908974895	-0.00607132	-0.00890955	0.110533805	0.15819776	8	0	0
ACSL3	0.1835764	0.159462982	0.024113449	0.102453358	0.110563643	0.158227839	4	0	0
IFI27	0.9151647	0.925281697	-0.01011702	-0.01569077	0.110581714	0.158241074	1	0	0

HNRNPK	0.0492694	0.04594399	0.003325388	0.03811338	0.110655593	0.158334162	10	0	0
WDR5	0.21783	0.229889661	-0.01205966	-0.05989345	0.110734841	0.158434916	3	0	0
GPATCH8	0.200689	0.224733261	-0.02404425	-0.06971148	0.110773993	0.158478291	9	0	0
COPS8	0.3658725	0.371505855	-0.00563333	0.00133395	0.110806553	0.158512229	10	0	0
BBS5	0.1662264	0.170141554	-0.00391511	0.027926705	0.110883579	0.158609767	8	0	0
FXN	0.2384922	0.226700405	0.011791757	0.037531201	0.110976268	0.158722828	6	0	0
SC5D	0.0399484	0.043498123	-0.00354976	-0.03309401	0.110980318	0.158722828	10	0	0
ZFAND3	0.0320677	0.029243137	0.002824594	0.169485388	0.111013496	0.158751499	5	0	0
CORO7	0.0351294	0.035484723	-0.00035532	-0.00019675	0.111018067	0.158751499	9	0	0
TRAPPC3	0.2574135	0.25288138	0.004532151	0.072865159	0.11111297	0.15887454	8	0	0
KIAA1147	0.2705223	0.259466344	0.011055994	0.038958973	0.111186918	0.158967602	7	0	0
TYSND1	0.180674	0.186589473	-0.00591551	-0.06014093	0.111201167	0.158975301	10	0	0
RBM15B	0.1369145	0.147363611	-0.01044915	-0.04163848	0.111219742	0.158989184	9	0	0
NDUFS7	0.0324711	0.034610994	-0.00213992	-0.0337568	0.111237616	0.159002062	11	0	0
CAPN10	0.0946913	0.089913704	0.004777588	0.00514442	0.11129528	0.159067857	5	0	0
BAZ2A	0.0513434	0.051144364	0.000198998	0.013938675	0.111301383	0.159067857	5	0	0
MSANTD2	0.0890021	0.073960872	0.015041206	0.099042411	0.111311586	0.159069763	8	0	0
ARHGAP12	0.0633683	0.051777557	0.011590785	0.193557316	0.111341547	0.159099904	12	0	0
CCDC158	0.4409921	0.459118823	-0.01812668	-0.03255707	0.111402869	0.159162169	3	0	0
TSFM	0.206316	0.218531874	-0.01221587	-0.01092549	0.111395014	0.159162169	11	0	0
STARD7	0.1252307	0.127691463	-0.0024608	0.017145964	0.111465227	0.159238575	9	0	0
CDK9	0.1530885	0.15977978	-0.00669125	-0.00319562	0.111500061	0.159275652	10	0	0
ERCC8	0.0600513	0.061669983	-0.00161871	-0.04763315	0.111554986	0.159337001	12	0	0
DECR1	0.035601	0.037865908	-0.00226487	-0.04572788	0.111560775	0.159337001	9	0	0
SNIP1	0.2197862	0.227711976	-0.00792579	-0.01918043	0.111606449	0.159376853	14	0	0
KRTAP4-2	0.8869038	0.894009121	-0.00710529	-0.01153216	0.111600251	0.159376853	3	0	0
EIF3B	0.0918419	0.08927147	0.002570386	0.051976334	0.111640934	0.159388028	5	0	0
PTPRD	0.1106518	0.091002503	0.01964931	0.295600169	0.111627696	0.159388028	7	0	0
DYNLRB1	0.0423257	0.04532957	-0.00300386	-0.03148566	0.111637833	0.159388028	12	0	0
HNRNPUL1	0.0997223	0.101436848	-0.00171456	0.041386772	0.111663242	0.159407188	10	0	0
FAM222B	0.0439833	0.042939752	0.001043534	0.018229875	0.111687342	0.159428903	10	0	0
DIAPH3	0.0318108	0.031410908	0.000399866	0.017801604	0.111733945	0.159482734	9	0	0
B9D2	0.1174722	0.125426227	-0.00795401	0.008445546	0.111753465	0.159497903	18	0	0
PRND	0.479344	0.499258203	-0.01991423	-0.05388118	0.111770578	0.159509635	6	0	0
RNF182	0.2668921	0.26293617	0.003955907	0.034871813	0.111779823	0.159510136	12	0	0
FOXI3	0.102735	0.081267173	0.0214678	0.354784965	0.111825672	0.159550176	10	0	0
BEND7	0.0688324	0.062538316	0.006294065	0.151784508	0.111819465	0.159550176	5	0	0
TOMM7	0.4347789	0.448985272	-0.01420638	-0.02724541	0.111859072	0.159585135	6	0	0
MAP3K4	0.3406365	0.350221676	-0.00958522	0.020647122	0.111963248	0.159721054	5	0	0
ARL5A	0.1529622	0.157711581	-0.00474939	0.002707428	0.111997343	0.159756986	7	0	0
TRAK2	0.1396475	0.153985222	-0.01433772	-0.05494392	0.112098494	0.159888556	16	0	0
KLK15	0.2066269	0.221891787	-0.01526493	-0.09823964	0.112108385	0.159889948	1	0	0
CASP9	0.0288219	0.029127662	-0.00030575	-0.00109905	0.112183246	0.159978405	8	0	0
ACBD7	0.1571915	0.169173849	-0.01198232	0.072305742	0.112188246	0.159978405	11	0	0
TUBB3	0.1091901	0.111802915	-0.0026128	0.094360245	0.112219853	0.160010754	8	0	0
RTKN2	0.1915427	0.191704026	-0.00016136	-0.011327	0.112251065	0.160042536	13	0	0
RCN2	0.271915	0.273463349	-0.00154835	0.020034726	0.112316894	0.160123663	7	0	0
TFRC	0.0610168	0.063965887	-0.00294905	-0.00929977	0.112340357	0.160131658	7	0	0
CTR9	0.2922214	0.301848166	-0.0096268	-0.06715215	0.112337142	0.160131658	6	0	0
PADI3	0.520153	0.531402088	-0.01124904	-0.03568734	0.112396789	0.160186636	6	0	0
FAM98C	0.1158686	0.124614597	-0.00874603	0.075779718	0.112391398	0.160186636	10	0	0
DRAM1	0.2443612	0.243372728	0.000988497	0.013511656	0.112440264	0.160235863	5	0	0
ZNF491	0.1690696	0.152746851	0.016322707	0.128049223	0.112450478	0.160237687	3	0	0
GNPTAB	0.0479931	0.043794415	0.00419865	0.071173868	0.112461447	0.160240588	4	0	0
NEK5	0.3229501	0.322317984	0.000632128	0.013334241	0.112512869	0.160301121	12	0	0
POMGNT2	0.1188732	0.117153845	0.001719321	0.057230092	0.112551769	0.160343807	9	0	0
ATP8B2	0.0547487	0.044499362	0.010249379	0.276700515	0.1125719	0.160353213	12	0	0
STAP2	0.0415502	0.040270641	0.00127957	0.033368664	0.112585193	0.160353213	9	0	0
MPND	0.0415502	0.040270641	0.00127957	0.033368664	0.112585193	0.160353213	9	0	0
SS18L2	0.0392084	0.035658026	0.003550333	0.075487596	0.112654337	0.160426216	4	0	0
RRM2B	0.1526406	0.157597205	-0.00495656	0.028404045	0.11265345	0.160426216	8	0	0
NUP210	0.2838516	0.291518103	-0.00766655	-0.02921162	0.11267437	0.160442006	7	0	0
CYP2S1	0.1351063	0.115565077	0.019541255	0.209337975	0.112759202	0.160550056	12	0	0

CCDC38	0.1073495	0.120441134	-0.01309165	0.040300997	0.112772622	0.160556418	9	0	0
SLC2A4RG	0.2252291	0.225571369	-0.00034229	0.047673916	0.112884368	0.160702756	5	0	0
EIF2B2	0.0451411	0.048735752	-0.00359467	-0.05197679	0.112922801	0.160744711	10	0	0
AMPD2	0.5345266	0.491705615	0.042821008	0.118161502	0.112942633	0.160760183	1	0	0
TSR1	0.0417156	0.042138769	-0.00042317	0.026194121	0.112952218	0.160761069	5	0	0
CES2	0.1408676	0.147275401	-0.00640779	-0.00523137	0.112978503	0.160772964	9	0	0
VPS4B	0.1358363	0.137321718	-0.00148539	0.052609608	0.112976393	0.160772964	9	0	0
SPDL1	0.0789197	0.088010862	-0.00909121	-0.0526715	0.113085433	0.160899597	10	0	0
NSMAF	0.1055145	0.105598931	-8.45E-05	0.018910002	0.11308123	0.160899597	14	0	0
AHCYL1	0.1896627	0.180302193	0.009360491	0.042481808	0.113141583	0.160951636	11	0	0
LIN7C	0.193541	0.196970845	-0.00342981	-0.0062538	0.113131178	0.160951636	12	0	0
E4F1	0.1217729	0.124223788	-0.00245085	-0.00769092	0.113148928	0.160951636	12	0	0
HPCAL1	0.1638342	0.163594939	0.000239216	0.031858404	0.113217987	0.161037099	11	0	0
KRT79	0.7300751	0.750156389	-0.02008131	-0.03769004	0.113261088	0.161085629	5	0	0
STK24	0.5042986	0.512965183	-0.00866654	0.029557446	0.113302195	0.161131317	5	0	0
EPB41L5	0.120428	0.104899645	0.015528367	0.090117837	0.113392106	0.161246398	5	0	0
C1orf127	0.6264503	0.645215021	-0.01876476	-0.05286399	0.113510472	0.161395298	2	0	0
DBP	0.0564173	0.051744659	0.004672633	0.129558065	0.113514813	0.161395298	12	0	0
TM7SF2	0.2031918	0.213602991	-0.0104112	-0.05081904	0.113528022	0.161401284	15	0	0
SMCO1	0.7663282	0.781369648	-0.01504149	-0.02448905	0.113601569	0.161493044	5	0	0
APPBP2	0.0489417	0.046562721	0.002378996	0.056778023	0.113618593	0.161504445	8	0	0
DHRS1	0.145998	0.146308039	-0.00031001	0.037194544	0.113742553	0.161667837	10	0	0
TARS2	0.1053884	0.120749302	-0.01536086	-0.01838405	0.113802133	0.161739703	8	0	0
SSU72	0.0702913	0.073298487	-0.00300715	-1.12E-05	0.113818768	0.161750528	11	0	0
SPPL2A	0.0444073	0.042995016	0.001412307	0.051118401	0.11383823	0.161765369	9	0	0
VSIG10	0.1155806	0.088106253	0.027474309	0.614506866	0.113876527	0.161770212	15	0	0
MED6	0.1180695	0.128151669	-0.01008218	-0.07151609	0.113867021	0.161770212	5	0	0
PEAK1	0.0718359	0.071329994	0.000505888	0.01428917	0.113877715	0.161770212	13	0	0
C15orf61	0.1856898	0.198640443	-0.01295061	-0.07081495	0.113900003	0.161789059	7	0	0
RPL21	0.2406184	0.245576087	-0.00495765	0.013462576	0.113999706	0.161917859	8	0	0
LYL1	0.3818694	0.363414688	0.018454722	0.184986398	0.114029802	0.161934959	9	0	0
RBCK1	0.0945594	0.103399169	-0.00883979	-0.03696016	0.114025339	0.161934959	10	0	0
COMMD9	0.1596215	0.168496598	-0.00887511	-0.05743203	0.114078294	0.161990997	12	0	0
FAF1	0.1505473	0.16212006	-0.01157277	-0.03758611	0.114101523	0.162011155	8	0	0
TSTD3	0.232898	0.230825854	0.002072122	0.052658932	0.11414622	0.162044711	5	0	0
CNOT2	0.0766357	0.086194268	-0.0095586	-0.06097695	0.11415226	0.162044711	10	0	0
ZNF770	0.0408473	0.039273525	0.001573763	0.030336483	0.11413663	0.162044711	6	0	0
POLR3K	0.1725811	0.18799441	-0.01541327	-0.01737508	0.114250231	0.162170952	7	0	0
MDM1	0.089636	0.098259333	-0.00862338	-0.03540176	0.114337705	0.162282273	13	0	0
MAPKAPK3	0.0742773	0.063178113	0.011099225	0.126373858	0.114364716	0.162307766	10	0	0
HIST1H2AM	0.0419083	0.041493917	0.000414336	-0.0055452	0.114445176	0.162409105	10	0	0
CNDP2	0.0676881	0.077268499	-0.00958044	-0.04165682	0.114464467	0.16242363	6	0	0
RSBN1L	0.1041878	0.11074652	-0.00655877	-0.01229493	0.114642231	0.162650139	11	0	0
CDH15	0.1067702	0.10711922	-0.00034905	0.016925548	0.114641502	0.162650139	8	0	0
MTHFR	0.1970361	0.187059475	0.009976649	-0.00273201	0.114676718	0.1626862	3	0	0
CMTM6	0.5714777	0.575577501	-0.00409983	-0.00029679	0.114746584	0.162759567	8	0	0
POLL	0.1838016	0.180738235	0.003063356	0.016442259	0.114745849	0.162759567	11	0	0
SRGAP1	0.136741	0.127306192	0.009434796	0.012429869	0.114896385	0.162949921	13	0	0
TRAPPC5	0.2268444	0.236474172	-0.00962978	-0.00433658	0.114898955	0.162949921	6	0	0
VCPIP1	0.1223534	0.131254188	-0.00890079	-0.00184151	0.114988313	0.163063755	11	0	0
GLRX3	0.1483409	0.159409922	-0.01106905	-0.01947392	0.115000121	0.163067608	7	0	0
ADD2	0.2004329	0.19545345	0.004979484	0.282046407	0.115018445	0.163080699	15	0	0
AK3	0.3923942	0.413089803	-0.02069558	-0.05625977	0.11506594	0.163135143	2	0	0
OGG1	0.1576966	0.149137657	0.008558992	0.073287415	0.115096224	0.163165181	15	0	0
B4GALT4	0.0568016	0.051745518	0.005056115	0.102393377	0.115110074	0.163171918	7	0	0
BAG2	0.2654838	0.252969009	0.012514819	0.408535727	0.115162308	0.163224169	10	0	0
VAT1	0.177834	0.160644428	0.017189599	0.416543169	0.115165135	0.163224169	8	0	0
QRICH1	0.1034672	0.105081836	-0.00161466	-0.02611036	0.115212042	0.163277749	14	0	0
DNAJC3	0.0702156	0.07267334	-0.00245778	-0.00875107	0.115245167	0.163311789	11	0	0
UGDH	0.2126012	0.230655937	-0.01805472	-0.04634535	0.115290796	0.163357312	7	0	0
MTIF3	0.0707988	0.077403399	-0.00660459	-0.00044062	0.115295507	0.163357312	18	0	0
SPATC1	0.4640506	0.420583731	0.043466872	0.147218244	0.11531651	0.163361261	2	0	0
MRI1	0.0931712	0.115462224	-0.02229098	-0.01073467	0.115311535	0.163361261	8	0	0

EFEMP2	0.0676037	0.057998224	0.009605484	0.137227561	0.115343471	0.163382436	4	0	0
PIP5K1B	0.12887	0.133253447	-0.00438345	0.037917182	0.115372072	0.163397009	10	0	0
RPS24	0.2541943	0.250879317	0.003314987	0.102072349	0.115378185	0.163397009	13	0	0
TRMT13	0.1782817	0.190383058	-0.01210136	-0.02942121	0.115416633	0.163438555	11	0	0
ILDR2	0.0975248	0.100392337	-0.00286754	0.05637251	0.115547687	0.16361122	8	0	0
H2AFY	0.4586577	0.451493181	0.007164564	0.034283731	0.115568789	0.1636158	13	0	0
LRRC24	0.3170155	0.303175994	0.013839541	0.13051654	0.115569167	0.1636158	5	0	0
CROT	0.0941186	0.10736142	-0.01324281	-0.04973804	0.115581647	0.163620554	10	0	0
EIF3L	0.3970227	0.409045593	-0.01202285	-0.00950224	0.11583027	0.16395957	11	0	0
RPS10	0.146491	0.146985738	-0.00049479	0.006278617	0.115845249	0.163967834	8	0	0
ODF2L	0.2283148	0.239223512	-0.01090867	-0.02705576	0.115864796	0.163978169	10	0	0
NBPF10	0.7814013	0.796801174	-0.01539992	-0.02795286	0.115870836	0.163978169	2	0	0
MAPKAPK2	0.0492343	0.050960723	-0.00172637	-0.04404969	0.115922197	0.164004008	8	0	0
MANF	0.035556	0.036197922	-0.00064196	-0.0111517	0.115934814	0.164004008	12	0	0
POLK	0.0480817	0.04607057	0.002011166	0.05690562	0.115908241	0.164004008	14	0	0
FBXW11	0.0458949	0.046733045	-0.00083817	-0.00642047	0.11593387	0.164004008	11	0	0
BTNL3	0.6472886	0.703504754	-0.05621617	-0.11839611	0.115911968	0.164004008	1	0	0
DGKD	0.2487823	0.259716591	-0.01093429	-0.01749809	0.115969815	0.164040584	10	0	0
RBM7	0.2795166	0.302212365	-0.02269581	-0.06678639	0.116044618	0.16413345	11	0	0
NTAN1	0.1336158	0.13020913	0.003406713	0.00475759	0.11607128	0.164158215	9	0	0
ELL3	0.2025418	0.204291239	-0.00174943	-0.00503995	0.116124393	0.164220383	11	0	0
LTF	0.8340276	0.824244626	0.009782947	0.020313705	0.116280805	0.164428613	2	0	0
ARHGDIA	0.0570293	0.047841685	0.00918761	0.229433295	0.116319409	0.164470234	12	0	0
TIPIN	0.1354099	0.134304103	0.001105838	0.020903129	0.116400067	0.164570971	7	0	0
SEC14L2	0.3399072	0.349794681	-0.00988747	-0.00841026	0.116409005	0.164570971	10	0	0
RPS19BP1	0.2147807	0.227478095	-0.01269736	-0.01465844	0.116420269	0.164573924	8	0	0
RPS15	0.1580143	0.16554578	-0.00753146	-0.0160881	0.116445443	0.164596538	10	0	0
CPNE8	0.5152333	0.522821595	-0.0075883	-0.00067552	0.1164789	0.164630855	3	0	0
CAPZA2	0.4585783	0.455742923	0.002835392	0.016810174	0.116543729	0.164709504	3	0	0
CD2BP2	0.1154289	0.120686779	-0.0052579	-0.01490344	0.116642698	0.164836388	14	0	0
ANKDD1A	0.198576	0.207656405	-0.00908044	0.028401851	0.116703086	0.164908734	11	0	0
PPM1L	0.0429596	0.040365084	0.002594501	0.056298417	0.116744276	0.164953942	6	0	0
UBE2S	0.039275	0.035522283	0.003752736	0.106232836	0.116765694	0.164971208	5	0	0
ZNF774	0.1998993	0.200505425	-0.00060611	0.045949858	0.116779627	0.164977898	14	0	0
HTT	0.1327192	0.129062926	0.003656285	0.051983666	0.116862964	0.165074395	7	0	0
FMN1	0.2148556	0.194769568	0.02008605	0.407854812	0.116866339	0.165074395	10	0	0
MEGF8	0.0527821	0.048862109	0.003919943	0.100124413	0.116917046	0.165133014	10	0	0
INTS9	0.0818542	0.078216196	0.003637993	0.053054408	0.11693625	0.16513413	21	0	0
SLC43A2	0.0345531	0.033545071	0.001008051	0.036470181	0.116933497	0.16513413	11	0	0
FEM1B	0.0743113	0.070719136	0.003592207	0.025673941	0.116979545	0.165182265	7	0	0
ANKRD63	0.0966491	0.082899255	0.013749849	0.216859039	0.117044681	0.16526123	7	0	0
SLC38A1	0.2817258	0.290076191	-0.00835041	0.069844759	0.117063486	0.165261762	6	0	0
ADAT1	0.2063935	0.210171083	-0.00377754	0.007689734	0.117061388	0.165261762	11	0	0
GFM2	0.0558473	0.062972744	-0.00712545	-0.02921911	0.117125828	0.165326198	16	0	0
C14orf119	0.2191545	0.2294116	-0.01025714	-0.03278333	0.117127564	0.165326198	8	0	0
KHDRBS3	0.0997409	0.089208554	0.010532379	0.378074142	0.117238479	0.165469732	11	0	0
CALR	0.05898	0.054672967	0.004307067	0.004275855	0.117263949	0.165492658	11	0	0
CST6	0.3775649	0.370006258	0.00755868	0.149729841	0.117433811	0.165707273	12	0	0
CBX5	0.0455031	0.044720513	0.000782609	0.021856355	0.117434497	0.165707273	21	0	0
C7orf61	0.7348268	0.732886301	0.001940516	0.005352633	0.117546986	0.165852954	4	0	0
CENPT	0.1590452	0.167414975	-0.00836975	-0.03543764	0.11760258	0.165918341	7	0	0
LYPLA2	0.2719634	0.287791712	-0.01582831	-0.02683589	0.117645907	0.165941518	9	0	0
DAP	0.3091445	0.320061329	-0.01091686	0.264333602	0.117646763	0.165941518	4	0	0
YWHAG	0.1419231	0.13052568	0.011397375	0.074427065	0.117629582	0.165941518	6	0	0
THBS3	0.0458	0.043083977	0.002715979	0.084945401	0.11767443	0.16596749	12	0	0
URGCP	0.0468832	0.048542194	-0.00165904	-0.03716325	0.117742594	0.166050571	9	0	0
MOXD1	0.2584464	0.259364844	-0.00091841	0.231602989	0.117819214	0.166145563	8	0	0
VPS54	0.086742	0.083412106	0.003329926	0.025737831	0.117842095	0.166160201	8	0	0
RHOT1	0.0552934	0.061976693	-0.00668325	-0.02706468	0.117848122	0.166160201	10	0	0
ASF1B	0.0444462	0.042880246	0.001566003	0.052462033	0.117871617	0.166180264	6	0	0
AGPAT3	0.0213203	0.022150591	-0.00083026	-0.02716242	0.117882105	0.166181989	3	0	0
TERF2IP	0.0644172	0.074845681	-0.01042845	-0.02334788	0.117911423	0.166210256	7	0	0
F5	0.1703143	0.154626434	0.015687835	0.219716528	0.118073822	0.166413019	6	0	0

EXOSC5	0.0432047	0.042217446	0.000987254	0.020930289	0.118068043	0.166413019	9	0	0
PARP4	0.0390965	0.03751751	0.001578983	0.033174743	0.118097591	0.16643344	4	0	0
ATP13A2	0.2937329	0.297597948	-0.00386509	0.005850884	0.118128707	0.166464211	13	0	0
CPSF6	0.1303824	0.136598804	-0.0062164	0.041034791	0.118149216	0.166480033	8	0	0
WFDC3	0.1329371	0.142970775	-0.01003363	-0.05617036	0.118181917	0.166513029	11	0	0
CLPB	0.4384883	0.446161764	-0.00767342	-0.00480651	0.118201346	0.166527323	5	0	0
DSP	0.218113	0.216373462	0.001739541	-0.01450646	0.11830344	0.166651576	8	0	0
JUP	0.1875881	0.209308979	-0.02172092	-0.07874605	0.118308124	0.166651576	11	0	0
KPNA5	0.166918	0.185338413	-0.01842044	-0.01281929	0.118362316	0.166706187	10	0	0
DCAF15	0.0463073	0.049442821	-0.00313554	-0.04695097	0.118365482	0.166706187	8	0	0
GPR137B	0.1285024	0.112549372	0.015953009	0.124804997	0.118381724	0.166706612	12	0	0
CMYA5	0.578697	0.57269378	0.006003185	0.024934914	0.118384374	0.166706612	13	0	0
ZC3H7B	0.0622083	0.060578314	0.001629945	0.042273745	0.118439518	0.166771173	10	0	0
ZBTB49	0.036545	0.035970476	0.000574512	0.017163037	0.118503943	0.166822598	8	0	0
PRMT3	0.0615357	0.066009646	-0.00447392	-0.04087616	0.118498195	0.166822598	11	0	0
YIF1A	0.0596689	0.063637386	-0.00396852	-0.02586757	0.118496425	0.166822598	11	0	0
KRT85	0.666936	0.673452405	-0.00651645	-0.01379361	0.118534644	0.166852722	8	0	0
NFE2L1	0.0880248	0.06684012	0.021184727	0.337999221	0.118654077	0.167007732	9	0	0
QSER1	0.0575614	0.063115433	-0.00555402	-0.03050308	0.118696591	0.167054462	10	0	0
MARVELD3	0.1700475	0.179425127	-0.0093776	-0.01428471	0.118715393	0.167067815	9	0	0
RTCA	0.2100932	0.209120821	0.000972336	0.019070538	0.118757754	0.167101206	12	0	0
PPA2	0.1974585	0.199579397	-0.00212088	0.01962031	0.118756618	0.167101206	11	0	0
PDE7A	0.0471573	0.047549861	-0.0003926	-0.01174972	0.118794997	0.167140498	6	0	0
PAX5	0.2178826	0.181533224	0.036349365	0.289875704	0.11888267	0.167250731	11	0	0
MIS18A	0.0421228	0.043316261	-0.00119347	-0.03044858	0.11890465	0.167268533	10	0	0
KIAA0753	0.0659108	0.071374564	-0.00546372	-0.05446121	0.119041507	0.167447923	8	0	0
AAR2	0.2384585	0.262967057	-0.02450852	-0.06104859	0.11906875	0.16747311	13	0	0
EMX1	0.0696887	0.049331311	0.020357389	0.354789255	0.11910247	0.167507402	8	0	0
DNM3	0.1423968	0.141231658	0.001165133	0.064752549	0.119154547	0.167567505	9	0	0
KCNJ10	0.1388371	0.135640083	0.00319697	0.100161753	0.119207357	0.167628628	13	0	0
RNF168	0.356181	0.366276385	-0.01009543	-0.02556367	0.119226391	0.16762911	10	0	0
EEF2K	0.1330057	0.135849192	-0.00284354	0.028143972	0.11922102	0.16762911	10	0	0
CHD1L	0.2700165	0.281971379	-0.01195493	-0.04907622	0.119246207	0.16763069	7	0	0
PDCD11	0.0749747	0.076651235	-0.00167653	0.02244619	0.11924015	0.16763069	19	0	0
MATK	0.3380348	0.350224232	-0.0121894	0.05599526	0.119324371	0.167727423	8	0	0
DKK2	0.4068112	0.391015535	0.015795705	0.099474478	0.119336792	0.167731738	4	0	0
NUP37	0.0835359	0.093597679	-0.01006177	-0.04090539	0.119378065	0.167776601	13	0	0
PAF1	0.0866324	0.094411576	-0.00777917	0.001365356	0.119400978	0.167795655	11	0	0
TBC1D16	0.061388	0.056156968	0.005231032	0.10031323	0.119411357	0.167797094	10	0	0
FICD	0.0941315	0.109909752	-0.01577827	-0.03193288	0.119430793	0.167798112	11	0	0
FAM57B	0.0643071	0.063884734	0.000422362	-0.02724016	0.119421468	0.167798112	6	0	0
ALKBH8	0.185209	0.188421438	-0.00321244	-0.02262321	0.119444426	0.167804122	13	0	0
FBXO36	0.0395867	0.037903901	0.001682758	0.045278871	0.119524014	0.167902781	10	0	0
PAICS	0.0382057	0.039901766	-0.00169606	-0.02909546	0.11960978	0.168010104	12	0	0
SREK1IP1	0.0416487	0.03886208	0.00278661	0.066383248	0.119699617	0.168109962	10	0	0
CDC14B	0.3621705	0.385614568	-0.02344407	-0.03069166	0.119694622	0.168109962	5	0	0
GNE	0.6015364	0.592161408	0.009374985	0.034034475	0.119728299	0.168137079	8	0	0
CDR2	0.0336527	0.031954964	0.001697747	0.057229722	0.119837712	0.168264381	1	0	0
RPRML	0.0677793	0.060578793	0.007200498	0.431642739	0.11983233	0.168264381	7	0	0
TTLL9	0.1235856	0.110279582	0.013306051	0.314490581	0.119888691	0.168322784	14	0	0
EMC8	0.1309065	0.144347087	-0.01344059	-0.05856894	0.119907159	0.168335536	8	0	0
LCLAT1	0.3351784	0.335767962	-0.00058951	-0.02542621	0.119929204	0.168353306	6	0	0
CLRN2	0.7469319	0.74883689	-0.00190499	-0.0022514	0.119939737	0.168354917	8	0	0
C1QTNF6	0.2253273	0.231511815	-0.00618447	0.014888178	0.11995611	0.168364722	4	0	0
NTN3	0.2778427	0.265256102	0.012586572	0.096692641	0.12000556	0.168420949	7	0	0
UBXN1	0.1912186	0.192844664	-0.00162603	0.015780416	0.120036351	0.168437802	12	0	0
CEP76	0.0803531	0.086092014	-0.00573887	-0.02010402	0.120028103	0.168437802	13	0	0
SLC35G6	0.4990934	0.494857318	0.004236064	0.047529573	0.120074363	0.168477961	6	0	0
SOHLH2	0.0919985	0.077515282	0.014483238	0.232856652	0.120194713	0.16859407	11	0	0
CCDC169-S(	0.0919985	0.077515282	0.014483238	0.232856652	0.120194713	0.16859407	11	0	0
CCDC169	0.0919985	0.077515282	0.014483238	0.232856652	0.120194713	0.16859407	11	0	0
MFSD12	0.3362173	0.342729207	-0.00651193	0.006288546	0.120172677	0.16859407	6	0	0
GPC2	0.1972749	0.181006521	0.016268344	0.161406176	0.120260164	0.168672686	15	0	0

UTS2	0.7850531	0.795147937	-0.01009488	-0.01427856	0.120324236	0.168749355	8	0	0
CELF3	0.173924	0.165555676	0.008368306	0.087464185	0.120344265	0.168754668	12	0	0
KRCC1	0.194557	0.211347827	-0.01679079	-0.05053019	0.120346842	0.168754668	14	0	0
C3orf52	0.195769	0.203648598	-0.00787956	-0.06019773	0.120451696	0.168888495	13	0	0
ANAPC16	0.1509009	0.153039452	-0.00213858	0.01570289	0.120506753	0.168952483	9	0	0
ACTL7B	0.7095783	0.736218019	-0.02663971	-0.05158206	0.120540949	0.168974247	4	0	0
DENND5A	0.380358	0.363148224	0.017209821	0.055446436	0.120541118	0.168974247	2	0	0
ZDHHC20	0.5580187	0.562346222	-0.00432748	0.014379111	0.120605168	0.16905082	6	0	0
YBX1	0.0663975	0.075038324	-0.00864083	0.013990999	0.12062821	0.169069906	12	0	0
CREBRF	0.0369104	0.036572666	0.000337776	0.015490919	0.120703292	0.169148703	10	0	0
MNS1	0.1994281	0.202307648	-0.00287959	0.117894698	0.120717811	0.169155833	12	0	0
SOCS6	0.0793014	0.070789599	0.008511797	0.218781884	0.120739058	0.169172389	6	0	0
SIKE1	0.4234859	0.433377304	-0.00989139	-0.02060679	0.120919278	0.169389565	8	0	0
CHST10	0.2309503	0.238286826	-0.00733656	0.116283555	0.120914697	0.169389565	13	0	0
RRP36	0.2338299	0.24492994	-0.01110008	-0.05293466	0.12092239	0.169389565	8	0	0
CTBP1	0.5231609	0.492996694	0.030164238	0.102273877	0.121012974	0.169503218	9	0	0
SYCE2	0.2140403	0.216799445	-0.00275919	0.272800479	0.121043112	0.169532192	9	0	0
FAM91A1	0.17963	0.189479896	-0.00984986	0.002284293	0.121076629	0.169556278	7	0	0
SOGA1	0.1027966	0.107902791	-0.00510623	-0.04684227	0.121079215	0.169556278	10	0	0
DUSP1	0.0868645	0.086044727	0.000819813	0.025260245	0.121139827	0.169610363	15	0	0
FIBP	0.1247203	0.129852986	-0.00513273	0.020620341	0.121146206	0.169610363	7	0	0
SMYD4	0.3861733	0.396788426	-0.01061513	-0.03060416	0.121130318	0.169610363	3	0	0
RNFT2	0.1179951	0.124698824	-0.00670373	-0.02295728	0.121188379	0.169656164	12	0	0
MTAP	0.3580436	0.365500307	-0.00745674	0.034787746	0.121254089	0.169734905	10	0	0
NDUFA5	0.2124398	0.214021326	-0.00158151	0.073724852	0.121270896	0.169745184	15	0	0
FLNC	0.2603262	0.243911439	0.016414747	0.34851989	0.121307886	0.16978371	9	0	0
RPN2	0.2319695	0.235119889	-0.00315036	0.064285722	0.121353743	0.169834639	6	0	0
HTR2B	0.642795	0.661720777	-0.01892578	-0.04716183	0.121405033	0.169882344	4	0	0
HYKK	0.1921944	0.203828931	-0.01163458	-0.02374944	0.121406773	0.169882344	9	0	0
RABGAP1	0.0917347	0.101102009	-0.00936732	-0.04792661	0.121442977	0.169906493	7	0	0
RPP30	0.1094832	0.112299905	-0.00281671	0.109579645	0.121436845	0.169906493	14	0	0
TMEM163	0.2545131	0.241976631	0.012536476	0.326721305	0.121519832	0.170000757	7	0	0
COX17	0.223874	0.229181605	-0.00530765	-0.02015528	0.12157448	0.170063943	10	0	0
SPO11	0.7429994	0.758702804	-0.01570339	-0.02284167	0.121621317	0.170116192	12	0	0
APEX1	0.0522028	0.053611852	-0.00140907	-0.03634252	0.12166687	0.170166639	13	0	0
ILKAP	0.1682497	0.173543872	-0.00529418	-0.01574622	0.121709985	0.170187127	8	0	0
TARSL2	0.2850415	0.301254135	-0.01621262	0.006821343	0.12170706	0.170187127	8	0	0
TPSG1	0.6186883	0.595405862	0.023282461	0.056122203	0.12169547	0.170187127	4	0	0
MTA2	0.0870458	0.075448683	0.011597077	0.149882652	0.121750331	0.170230272	8	0	0
OXCT2	0.8583104	0.869374755	-0.01106434	-0.01826723	0.121765707	0.170238501	1	0	0
KDM4D	0.0445308	0.047653623	-0.00312281	-0.05427568	0.121814625	0.170293618	16	0	0
SH2D7	0.0812719	0.074063351	0.007208522	0.077926084	0.121888131	0.170383097	9	0	0
SPOCK3	0.2651515	0.269362577	-0.00421111	0.073451017	0.121961317	0.170472114	13	0	0
PGBD1	0.1062121	0.096120181	0.010091941	0.225131742	0.121988871	0.170495498	12	0	0
C9orf85	0.0551796	0.049020879	0.006158678	0.148670756	0.121997057	0.170495498	18	0	0
TMEM100	0.4016559	0.380760299	0.020895599	0.095477475	0.122092063	0.170614977	6	0	0
DHRS11	0.2631414	0.270933673	-0.00779223	-0.00293126	0.122255672	0.1708303	12	0	0
C12orf57	0.472841	0.488077785	-0.01523675	-0.01225885	0.122272883	0.17084104	6	0	0
FAHD2B	0.4873971	0.489594649	-0.00219756	0.022825632	0.122354769	0.170942135	2	0	0
NOX4	0.7768558	0.815829767	-0.03897398	-0.06974518	0.12238248	0.170967533	1	0	0
HDAC10	0.0827374	0.076987456	0.005749922	0.178662783	0.122425361	0.171014118	13	0	0
OSBPL6	0.2561593	0.27972707	-0.02356775	-0.06035259	0.122515123	0.171121236	6	0	0
CDHR4	0.6909893	0.708569155	-0.01757986	-0.03839034	0.122521126	0.171121236	5	0	0
SEMA6A	0.0478174	0.041880992	0.005936417	0.228580204	0.122578543	0.171188099	8	0	0
CIAO1	0.1666641	0.157501335	0.009162792	0.092447461	0.122611233	0.171193762	10	0	0
SLC25A48	0.2353491	0.237692397	-0.00234325	0.000868707	0.122593118	0.171193762	12	0	0
ARL6IP4	0.2720818	0.27808064	-0.00599882	-0.02231341	0.1226053	0.171193762	18	0	0
CCDC40	0.0540927	0.050524298	0.00356837	0.085904691	0.122667805	0.171259419	9	0	0
CREB3L4	0.0917175	0.097593517	-0.00587599	0.078607167	0.122709768	0.17130467	14	0	0
NAV3	0.2800565	0.282201188	-0.00214464	-0.04647729	0.122744033	0.171326915	3	0	0
CTIF	0.2454068	0.23230882	0.013098028	0.077926825	0.122744807	0.171326915	10	0	0
UBTD1	0.0443995	0.04240759	0.001991935	0.043119364	0.122760835	0.171335953	14	0	0
SDR39U1	0.1259053	0.13101741	-0.00511211	-0.03817298	0.122809194	0.171390111	13	0	0

ALKBH5	0.1020956	0.107047607	-0.00495206	-0.01783975	0.122926392	0.171540322	11	0	0
TBCCD1	0.0611287	0.060729494	0.000399217	0.029724713	0.123059576	0.171712817	12	0	0
HDDC3	0.2051245	0.210179019	-0.0050545	-0.02970784	0.123123716	0.171788951	13	0	0
CEP89	0.061641	0.063764092	-0.00212305	-0.00615676	0.123156514	0.171821346	11	0	0
PDCD5	0.1346452	0.146929891	-0.01228469	-0.00459201	0.123173901	0.171832239	7	0	0
METTL2A	0.6407861	0.625748966	0.015037145	0.033726252	0.123317	0.172018487	1	0	0
CACNB4	0.1047804	0.10894535	-0.004165	0.061971259	0.123339644	0.172036695	12	0	0
CDH3	0.5007281	0.459019797	0.041708265	0.122906512	0.123372867	0.172069654	1	0	0
ANKRD28	0.1395884	0.142924891	-0.00333644	0.018772897	0.123509956	0.172247459	5	0	0
MORN4	0.2570981	0.263869542	-0.00677144	-0.00647214	0.123576644	0.172327063	10	0	0
KCNAB2	0.237573	0.238208238	-0.00063526	0.112894309	0.123647123	0.172403891	9	0	0
GPATCH2	0.1520029	0.163604311	-0.01160142	-0.01587008	0.123650962	0.172403891	11	0	0
PDIA2	0.6066143	0.621230234	-0.01461591	-0.02042621	0.123661017	0.172404508	8	0	0
SLC30A1	0.1466598	0.166668975	-0.02000914	-0.01619098	0.123686771	0.172427011	8	0	0
SUMF1	0.392449	0.417959599	-0.02551064	-0.08702539	0.123784948	0.172550464	6	0	0
CCDC112	0.2490955	0.26022806	-0.01113255	-0.04054772	0.123853629	0.172632786	13	0	0
TMEM53	0.0753021	0.080006563	-0.00470447	-0.03402537	0.123872573	0.172645775	13	0	0
MRPS18A	0.1841947	0.191144231	-0.00694951	-0.04138765	0.124044922	0.17287255	14	0	0
TMEM39B	0.1373998	0.141708776	-0.00430901	0.024233473	0.124069298	0.172893087	4	0	0
TCTEX1D2	0.2660429	0.275556117	-0.00951317	-0.02535032	0.124101084	0.172916426	8	0	0
NYAP1	0.1359447	0.146199539	-0.0102548	-0.01245476	0.124105328	0.172916426	7	0	0
CDK2	0.2134891	0.208305992	0.005183116	0.030974481	0.124241355	0.173092508	19	0	0
MOB3C	0.1900201	0.18292375	0.00709639	0.094604624	0.124298005	0.173134939	11	0	0
GABARAPL2	0.067259	0.065017044	0.002241985	0.02567554	0.12430077	0.173134939	11	0	0
STYX	0.0413713	0.039308416	0.002062881	0.044562212	0.124537471	0.173451163	8	0	0
PSME1	0.1559358	0.162665174	-0.00672934	-0.01684008	0.124560149	0.173469279	12	0	0
ADH5	0.1354604	0.126726663	0.008733742	0.053901719	0.124571299	0.173471337	9	0	0
CANX	0.1551472	0.162759421	-0.00761226	-0.01983363	0.124617843	0.173522679	7	0	0
ASXL3	0.0625875	0.049281158	0.013306375	0.309652579	0.124635509	0.173533806	11	0	0
ARID1B	0.0466897	0.047138895	-0.00044919	-0.01614554	0.124732088	0.173648802	6	0	0
HMBOX1	0.0693616	0.066591108	0.002770458	0.044863237	0.124737465	0.173648802	15	0	0
JMJD6	0.0533674	0.050583196	0.00278424	0.050656094	0.124777505	0.173691062	17	0	0
EDC4	0.2176475	0.222685529	-0.00503803	0.002836527	0.124842366	0.173767862	14	0	0
AMY2B	0.8515977	0.861069633	-0.0094719	-0.01621703	0.124878264	0.173790855	2	0	0
IFI27L1	0.0477034	0.048759748	-0.00105639	-0.01344967	0.124870876	0.173790855	15	0	0
PACS1	0.2064516	0.206478906	-2.73E-05	0.051656956	0.124900889	0.173808856	7	0	0
GORASP1	0.6120109	0.61499518	-0.00298427	0.038740661	0.124996191	0.173927981	6	0	0
CNPPD1	0.2181136	0.207148741	0.010964872	0.028649331	0.125017223	0.17393026	2	0	0
ABCF1	0.0979806	0.09279774	0.005182845	0.073565818	0.125016446	0.17393026	19	0	0
COL8A2	0.3774705	0.354425624	0.023044896	0.099412911	0.125238158	0.174183592	3	0	0
PLA2G4A	0.4811697	0.499023348	-0.01785363	-0.03218186	0.125222191	0.174183592	2	0	0
SLC20A1	0.0449039	0.045485238	-0.00058138	0.016230222	0.125228576	0.174183592	9	0	0
SLC9C1	0.6116203	0.612646996	-0.00102674	0.019602271	0.125330702	0.174298788	6	0	0
RSPH1	0.141039	0.149504218	-0.0084652	-0.03016872	0.125346164	0.174306776	14	0	0
TRIM32	0.0951765	0.090239538	0.004936975	0.23260474	0.125390081	0.174354329	7	0	0
TMBIM1	0.1117544	0.113723139	-0.00196871	-0.01412657	0.125423513	0.174368809	11	0	0
DISP2	0.2485061	0.258300339	-0.00979427	0.01249971	0.125429659	0.174368809	8	0	0
EMC10	0.3183115	0.313454758	0.00485677	0.322722388	0.125428291	0.174368809	15	0	0
CDC27	0.0859196	0.088130673	-0.00221107	0.003945518	0.125578493	0.174562183	15	0	0
RASSF3	0.0518059	0.052535962	-0.0007301	-0.00774442	0.125611588	0.174594656	4	0	0
MYO18A	0.1876442	0.180144214	0.007499969	0.000994293	0.125654635	0.174640955	12	0	0
DTX1	0.2955966	0.288900589	0.006696028	0.261753284	0.125695944	0.174684833	4	0	0
IK	0.0631018	0.057818358	0.005283395	0.074858211	0.125768939	0.174772735	5	0	0
COL19A1	0.1659792	0.152054915	0.013924329	0.234633671	0.12577957	0.174773967	8	0	0
GALNT1	0.3275105	0.339938244	-0.01242777	-0.03972829	0.125863639	0.174877236	3	0	0
CXCL10	0.4839448	0.474561144	0.009383692	0.031947373	0.125875782	0.174879389	4	0	0
ATP8B4	0.3075386	0.309370827	-0.00183219	0.079135377	0.12588469	0.174879389	6	0	0
NLGN1	0.0862317	0.079379931	0.006851768	0.167894885	0.125928602	0.174926844	7	0	0
C21orf58	0.0426897	0.04293267	-0.00024297	-0.00294966	0.125965125	0.174964027	10	0	0
CLSTN1	0.1430527	0.140414572	0.002638162	0.174725663	0.125993363	0.174989698	12	0	0
ETNK1	0.1824611	0.198682513	-0.01622142	-0.01286218	0.126023754	0.175018355	6	0	0
FAHD1	0.0747382	0.082425411	-0.00768724	-0.04647033	0.126045991	0.175035684	8	0	0
HSPA14	0.2497372	0.262621535	-0.01288434	-0.02586653	0.126059389	0.175040737	12	0	0

METTL2B	0.0583972	0.063990347	-0.00559311	-0.11339912	0.126100688	0.175084528	1	0	0
ACYP1	0.1674954	0.154740019	0.012755376	0.278341171	0.126122257	0.175100922	14	0	0
CAMTA1	0.0514472	0.051014633	0.0004326	0.018888023	0.126148479	0.175121698	15	0	0
SNRPE	0.1951269	0.220575928	-0.02544899	-0.05100599	0.126169051	0.175121698	9	0	0
AK7	0.0526434	0.053593175	-0.00094973	-0.02762397	0.126160918	0.175121698	13	0	0
FOXK2	0.0408955	0.037223344	0.003672164	0.119576353	0.126176277	0.175121698	7	0	0
CPT1B	0.8060051	0.81882818	-0.01282304	-0.02392273	0.126202707	0.175144827	5	0	0
09-Mar	0.3205078	0.31297538	0.007532396	0.025873246	0.126213056	0.175145638	4	0	0
IGFBP2	0.2892052	0.297454865	-0.00824967	0.087694149	0.126300393	0.175253276	6	0	0
TNS1	0.4948331	0.520016387	-0.02518331	-0.06651634	0.126340967	0.175287837	3	0	0
ZNF394	0.1928375	0.197450467	-0.00461293	0.010574908	0.126344846	0.175287837	12	0	0
LMCD1	0.0913551	0.081534372	0.00982069	0.035364542	0.126379006	0.175294552	10	0	0
DCBLD2	0.1218478	0.1319479	-0.01010007	-0.0529462	0.126373076	0.175294552	12	0	0
SOD2	0.0935662	0.094865306	-0.0012991	0.137483077	0.126371993	0.175294552	13	0	0
ERI3	0.1936618	0.200736446	-0.00707463	-0.04237605	0.126400508	0.175297264	11	0	0
NECAB3	0.190346	0.187822653	0.002523391	0.067244396	0.126397037	0.175297264	6	0	0
PFDN2	0.2011149	0.210581961	-0.00946705	0.003388948	0.126463446	0.175370988	14	0	0
ALDH18A1	0.1183614	0.119014132	-0.00065276	-0.00315289	0.126541237	0.175465297	9	0	0
MPP6	0.2432315	0.245979902	-0.00274839	0.016887318	0.126592322	0.175522564	5	0	0
SPATA6L	0.1626192	0.163446729	-0.0008275	0.165944482	0.126619876	0.175547198	10	0	0
CIDEC	0.7269307	0.741148534	-0.01421788	-0.02935173	0.126759462	0.175727138	4	0	0
FUCA1	0.1543784	0.155742055	-0.00136367	-0.01299465	0.126875915	0.175874983	17	0	0
ALOXE3	0.0466375	0.046177752	0.000459749	-0.00336739	0.12689105	0.175881072	12	0	0
HEATR6	0.3024966	0.308307516	-0.00581096	0.108247615	0.126900538	0.175881072	8	0	0
ATP8B1	0.1790388	0.187822385	-0.00878359	0.021371783	0.126909725	0.175881072	9	0	0
SIX1	0.3540001	0.341165921	0.012834135	0.054609667	0.126923704	0.175886855	7	0	0
TMEM186	0.1007812	0.115187808	-0.01440664	-0.02091261	0.12695496	0.175916577	9	0	0
ARL1	0.0335936	0.032884751	0.000708879	0.028163894	0.127001647	0.175967674	14	0	0
SDHD	0.1103365	0.117640511	-0.00730402	-0.00779216	0.1270407	0.176008188	18	0	0
RNF2	0.226433	0.21427618	0.012156853	0.123190705	0.127092193	0.176065929	9	0	0
GTPBP4	0.0979301	0.10141205	-0.00348198	-0.01671485	0.12730493	0.176319785	12	0	0
SERPINF2	0.6541052	0.636308341	0.017796884	0.041855064	0.127296449	0.176319785	3	0	0
OPRL1	0.0494539	0.036859604	0.012594307	0.16775527	0.127303917	0.176319785	11	0	0
PAFAH1B2	0.0665086	0.07153279	-0.00502415	-0.07430751	0.127374644	0.176402719	4	0	0
FANCM	0.0484915	0.045522026	0.002969523	0.062878416	0.1273906	0.176411196	15	0	0
ZFPM1	0.1290348	0.136147917	-0.00711312	0.006588814	0.127492315	0.176538421	7	0	0
CENPP	0.244602	0.249212162	-0.00461014	0.040065838	0.127528831	0.176570287	11	0	0
OSGEP	0.0534592	0.054453957	-0.00099472	-0.02410338	0.12754667	0.176570287	12	0	0
CYBA	0.1369355	0.150759694	-0.0138242	-0.10352622	0.127554706	0.176570287	16	0	0
BLCAP	0.1624149	0.170663939	-0.00824901	-0.03122077	0.127545812	0.176570287	32	0	0
PI4K2B	0.0587646	0.060558188	-0.00179361	-0.02831138	0.127587821	0.176602498	10	0	0
GRM8	0.0425583	0.044846625	-0.00228832	-0.02667196	0.127628182	0.176644731	6	0	0
DUS4L	0.2361468	0.255345804	-0.01919902	-0.04802073	0.127650766	0.176651097	10	0	0
TMEM97	0.0761267	0.057460846	0.018665875	0.243903465	0.127652479	0.176651097	7	0	0
KHDRBS1	0.4152498	0.411470071	0.003779765	0.03666239	0.127731339	0.176746589	9	0	0
DOLPP1	0.0607893	0.052931134	0.007858145	0.138264858	0.127746848	0.176754414	10	0	0
SYNGR3	0.2511709	0.258405655	-0.00723478	-0.04495584	0.127768068	0.176770138	7	0	0
ZNF8	0.0647457	0.043847022	0.020898664	0.517369529	0.12792884	0.176978919	9	0	0
PPP1R15B	0.0453717	0.048922786	-0.00355104	-0.02594015	0.127984175	0.177028161	8	0	0
WNT7B	0.1451612	0.119840076	0.025321074	0.24475219	0.127977152	0.177028161	8	0	0
SLC27A1	0.5931602	0.5647558	0.028404403	0.074534194	0.128049953	0.177105488	4	0	0
KCTD1	0.2011273	0.182981064	0.018146279	0.110711455	0.128113864	0.177180219	8	0	0
GAS2	0.0807819	0.083964568	-0.00318264	-0.03200458	0.128124716	0.177181565	16	0	0
PPP2R5A	0.202031	0.206537929	-0.00450697	-0.04141637	0.128148739	0.177201125	9	0	0
DICER1	0.0989154	0.098183253	0.000732182	0.00825623	0.128174667	0.177223314	10	0	0
SIK2	0.1540417	0.162427997	-0.0083863	0.029336534	0.128260561	0.177314739	6	0	0
VASN	0.3665161	0.341020621	0.025495524	0.08991713	0.128253933	0.177314739	7	0	0
AGPAT5	0.1328034	0.141225201	-0.00842176	-0.03923885	0.128360822	0.177439669	11	0	0
HMGCS2	0.5174408	0.541654947	-0.02421415	-0.05031894	0.128389415	0.177451842	6	0	0
NFS1	0.1311708	0.141313901	-0.01014313	-0.01333748	0.128384919	0.177451842	13	0	0
PSMD12	0.0798594	0.080499341	-0.00063995	0.048466185	0.128427574	0.177487542	12	0	0
HMGXB4	0.177072	0.190231696	-0.01315971	-0.01348609	0.128435036	0.177487542	9	0	0
DCAF6	0.0944783	0.083055662	0.011422636	0.097138916	0.128506639	0.177545454	5	0	0

BOP1	0.03998	0.040732989	-0.00075296	-0.03020855	0.12850338	0.177545454	5	0	0
CLDN25	0.5968339	0.607697286	-0.01086334	-0.0100776	0.128498941	0.177545454	6	0	0
AUH	0.1878927	0.202964286	-0.01507156	-0.00043911	0.128544559	0.177578972	11	0	0
POLR2E	0.0272863	0.026181858	0.001104483	0.044538662	0.128550701	0.177578972	2	0	0
METTL23	0.0636304	0.063087414	0.000542982	0.033236718	0.128576753	0.177601281	19	0	0
FAM219B	0.2302624	0.239149378	-0.00888699	0.04874121	0.128680863	0.1777314	12	0	0
IFITM2	0.8900423	0.898226858	-0.00818458	-0.01330246	0.128930701	0.178062759	5	0	0
VANGL2	0.1995057	0.19077618	0.008729517	0.100305176	0.129003697	0.178149854	8	0	0
HDAC11	0.1739613	0.181838426	-0.00787712	0.04291634	0.129064669	0.178206613	22	0	0
PRDM10	0.0403241	0.04025881	6.52E-05	0.000491536	0.129063677	0.178206613	11	0	0
PTPN14	0.1561364	0.153507506	0.002628844	0.074384508	0.129141536	0.178285297	6	0	0
SESTD1	0.2438092	0.227786582	0.01602262	0.041767008	0.12913392	0.178285297	6	0	0
PWP1	0.1158164	0.122740648	-0.00692422	-0.01043079	0.129172879	0.178314843	13	0	0
ARSA	0.1128686	0.105564472	0.007304143	-0.00445625	0.129196313	0.178333467	9	0	0
AKT1	0.2771813	0.281846942	-0.00466563	-0.01712393	0.129232856	0.178370181	4	0	0
FBXW8	0.1265353	0.133540025	-0.00700471	0.019362141	0.129249175	0.178378978	8	0	0
RBMS3	0.3395209	0.323307714	0.01621323	0.100883708	0.129308511	0.178443382	5	0	0
DEXI	0.2058396	0.21394993	-0.00811037	-0.019242	0.129315738	0.178443382	5	0	0
FAXC	0.1227347	0.12571962	-0.00298491	0.057589233	0.129339253	0.1784621	4	0	0
NCAPD2	0.1591949	0.162121139	-0.00292627	-0.00573056	0.129364225	0.178482826	9	0	0
CYP4V2	0.0198475	0.019694338	0.000153143	0.003272993	0.129608943	0.178806707	3	0	0
NOS1AP	0.1518656	0.156020068	-0.0041545	0.005097983	0.129684096	0.178896627	7	0	0
SDC1	0.1058617	0.109513289	-0.00365157	-0.03960049	0.129718792	0.178930728	14	0	0
C6orf47	0.1361004	0.130083128	0.006017291	0.029788181	0.129729014	0.178931067	21	0	0
MRPL14	0.1048243	0.11502443	-0.01020013	-0.02544265	0.129744719	0.178938968	8	0	0
NDUFV2	0.0374355	0.037243299	0.000192163	0.00955292	0.129922383	0.179170218	9	0	0
NT5C1A	0.1880749	0.169194671	0.018880244	0.208086999	0.129954424	0.17918685	5	0	0
PCSK4	0.1663764	0.164701439	0.001674964	0.040822999	0.129976214	0.179203119	13	0	0
LETM1	0.1640457	0.157975805	0.006069917	-0.00322835	0.130005516	0.179204142	2	0	0
ACSL6	0.282086	0.27383213	0.008253832	0.122368282	0.13000693	0.179204142	16	0	0
UEVLD	0.2022884	0.203180497	-0.00089211	0.072164914	0.129991645	0.179204142	11	0	0
BCAP29	0.1719567	0.170917596	0.001039065	0.026326379	0.130031737	0.179224563	14	0	0
CACHD1	0.0899022	0.095584607	-0.00568245	0.023262122	0.130057329	0.179246062	10	0	0
ATM	0.0470178	0.044784738	0.002233034	0.062695212	0.130108168	0.179274802	30	0	0
ST3GAL4	0.1226245	0.129612479	-0.00698796	0.004958458	0.130107063	0.179274802	9	0	0
C12orf42	0.1613924	0.139897677	0.02149468	0.506831412	0.130227963	0.179426082	15	0	0
SAMD14	0.0998832	0.101086822	-0.00120363	0.097987719	0.130256328	0.179451379	7	0	0
CDK19	0.0493791	0.048215256	0.00116384	0.026034105	0.13037383	0.179599464	7	0	0
KCNK6	0.2007983	0.195821178	0.004977073	0.209181805	0.130481228	0.179733608	8	0	0
COA4	0.118881	0.118782963	9.80E-05	0.033748684	0.130520187	0.179773078	10	0	0
ZNF319	0.1127688	0.113973737	-0.00120497	0.002086511	0.130529928	0.179773078	11	0	0
HYLS1	0.617261	0.663134833	-0.04587384	-0.09262891	0.130582532	0.179831718	3	0	0
UHRF1BP1L	0.170505	0.177983814	-0.00747885	0.014352712	0.130618015	0.179866773	13	0	0
PRKAA2	0.0979003	0.087211327	0.010688954	0.212576969	0.130647312	0.179893305	11	0	0
ZNF230	0.1193305	0.107119211	0.012211326	0.090659897	0.13068598	0.179932735	10	0	0
HS6ST3	0.0951189	0.087292577	0.007826305	0.173522875	0.130741693	0.179982089	9	0	0
ZNF527	0.0823753	0.084334986	-0.00195965	0.226314685	0.130741895	0.179982089	12	0	0
RBM15	0.0372913	0.038467114	-0.00117585	-0.03634193	0.130779252	0.17998416	7	0	0
C2orf91	0.7390706	0.750920032	-0.01184946	-0.00558902	0.130783539	0.17998416	5	0	0
KCTD8	0.0644333	0.052234052	0.012199223	0.274717263	0.130763349	0.17998416	10	0	0
SLC25A40	0.0461761	0.045776144	0.00039992	0.014014969	0.130773839	0.17998416	10	0	0
C22orf39	0.2791171	0.290281654	-0.01116456	0.002564805	0.130896385	0.180125637	10	0	0
PIK3CB	0.1454117	0.155427008	-0.01001533	0.015936283	0.131041272	0.180311181	6	0	0
RBFA	0.1177907	0.121874499	-0.00408378	0.048296087	0.131219984	0.180543237	11	0	0
SCGB3A2	0.7962304	0.823184818	-0.02695441	-0.04751316	0.131235759	0.180551092	2	0	0
SUMO1	0.0921958	0.092082488	0.000113333	-0.00496106	0.131344548	0.180686901	9	0	0
C3orf70	0.2011947	0.21043116	-0.00923647	0.061115889	0.131386084	0.180725657	12	0	0
GPSM3	0.0696768	0.05997324	0.009703534	0.084541839	0.131392873	0.180725657	34	0	0
LIM2	0.5804718	0.569296309	0.01117554	0.025596323	0.131511036	0.180874315	5	0	0
BRMS1	0.2689721	0.27201938	-0.00304731	-0.0173826	0.131629664	0.18102359	14	0	0
CYP2J2	0.1317707	0.137714482	-0.00594374	-0.0070704	0.131665515	0.181059011	12	0	0
TRANK1	0.2472006	0.249128597	-0.00192802	0.165719789	0.13169295	0.181082854	11	0	0
HSP90AB1	0.1336381	0.138955677	-0.00531758	-0.01095803	0.131886123	0.181334573	12	0	0

ZC3H13	0.1429379	0.150090279	-0.00715241	-0.01363239	0.131919408	0.181366435	14	0	0
MYO1F	0.1435035	0.137168091	0.006335456	0.067882693	0.131955766	0.181402515	6	0	0
RAB30	0.0543994	0.055142786	-0.00074341	-0.0311846	0.132011911	0.181465791	15	0	0
EREG	0.2602007	0.242156058	0.018044632	0.250617586	0.132185166	0.181690025	12	0	0
BYSL	0.1091943	0.121021494	-0.01182722	-0.01333006	0.132303715	0.181839037	13	0	0
EIF3I	0.1378606	0.145150967	-0.00729032	0.03977883	0.132351932	0.181891368	9	0	0
CCDC14	0.2086053	0.211497156	-0.00289186	-0.0152594	0.132372732	0.181906016	3	0	0
ELOVL4	0.1374468	0.136783522	0.000663286	0.155040881	0.132390008	0.181915819	12	0	0
ZNF670	0.2001265	0.223785945	-0.0236594	-0.06289474	0.132446784	0.181979893	9	0	0
TPX2	0.0397896	0.038207572	0.001582003	0.043443629	0.132475519	0.182005431	10	0	0
KARS	0.0506252	0.048693857	0.001931307	0.054268319	0.132604774	0.182155106	7	0	0
DCN	0.6898094	0.717170258	-0.02736086	-0.05664573	0.13278707	0.182391552	4	0	0
FLVCR1	0.3781918	0.392949473	-0.01475764	-0.00766434	0.132823935	0.182400283	5	0	0
HESX1	0.0380889	0.038814113	-0.0007252	-0.00426286	0.132817926	0.182400283	10	0	0
APPL1	0.0380889	0.038814113	-0.0007252	-0.00426286	0.132817926	0.182400283	10	0	0
RMI1	0.0540456	0.052297018	0.001748629	0.021039268	0.132847829	0.182419128	13	0	0
TMPO	0.0428973	0.039470152	0.003427131	0.062719537	0.133030901	0.18265653	5	0	0
MAEA	0.0550822	0.056198649	-0.0011165	0.010811224	0.133094616	0.1826977	9	0	0
IKBIP	0.0402977	0.036854452	0.003443278	0.062369763	0.13310163	0.1826977	15	0	0
RFXANK	0.1468741	0.151387578	-0.00451344	0.008787383	0.133081794	0.1826977	13	0	0
PSMG1	0.0549739	0.048051785	0.006922163	0.095351987	0.133098199	0.1826977	6	0	0
PTPN11	0.1562691	0.160818749	-0.00454965	-0.03191144	0.133154149	0.182755802	8	0	0
PF4	0.5700384	0.563523886	0.006514521	0.031565667	0.133191731	0.182793395	9	0	0
PAWR	0.1188383	0.135615977	-0.01677764	-0.05877189	0.133214492	0.182810645	10	0	0
ERMAP	0.0590669	0.05899678	7.01E-05	0.002507266	0.133249488	0.18284468	15	0	0
ACBD6	0.2200977	0.224468271	-0.00437053	-0.00896527	0.133345516	0.182948456	8	0	0
CUL4A	0.2020074	0.208152196	-0.00614483	0.002328168	0.133336676	0.182948456	14	0	0
PLAGL2	0.214691	0.218011398	-0.00332037	-0.01444425	0.133412392	0.18302621	5	0	0
GNB4	0.114205	0.102135136	0.012069856	0.257834087	0.133431128	0.183037913	11	0	0
LTK	0.3468405	0.337460229	0.009380266	0.17215727	0.13361344	0.18325136	11	0	0
MAP1A	0.0609847	0.056147855	0.004836865	0.054444685	0.133616901	0.18325136	13	0	0
CTDNEP1	0.1480418	0.155131835	-0.00709003	-0.00051337	0.133617378	0.18325136	12	0	0
HDHD2	0.0625708	0.069137079	-0.00656633	-0.02950059	0.133660546	0.183296548	10	0	0
NOP14	0.1117892	0.104793649	0.006995505	0.057086785	0.133711856	0.183352894	11	0	0
YTHDF3	0.0945307	0.105086534	-0.01055585	-0.01441657	0.133759817	0.183404639	12	0	0
IFI16	0.39438	0.34719678	0.047183199	0.178992497	0.133908784	0.183580826	1	0	0
ADPRM	0.0398717	0.041438056	-0.00156631	-0.03762706	0.133901357	0.183580826	7	0	0
FOXRED2	0.3449044	0.345549825	-0.00064544	0.023132895	0.13392466	0.183588559	8	0	0
RTBDN	0.1395041	0.132692456	0.006811595	0.040558665	0.133996657	0.183673217	7	0	0
SYVN1	0.0527629	0.057780766	-0.00501783	-0.03866059	0.134079173	0.18377228	9	0	0
ZFP14	0.2837443	0.299234079	-0.01548982	0.0192924	0.134108081	0.183797857	10	0	0
SLC25A1	0.1055316	0.111076798	-0.00554521	0.008881257	0.134147193	0.183837412	8	0	0
PSMA3	0.0518215	0.04800569	0.003815856	0.098493084	0.134192293	0.183885168	14	0	0
TMEM87B	0.0640061	0.06196262	0.002043483	0.033108492	0.134246875	0.183931858	12	0	0
PAFAH1B1	0.0931783	0.101512217	-0.00833388	-0.06880753	0.134341211	0.184047048	5	0	0
SYT15	0.4577766	0.450575177	0.007201394	0.008460233	0.13437549	0.184051835	6	0	0
DNASE1	0.1397897	0.1279406	0.011849098	0.108067345	0.134369029	0.184051835	4	0	0
HSD11B1L	0.0406402	0.040066147	0.000574032	0.025897335	0.134363793	0.184051835	12	0	0
SEMA3D	0.1808591	0.151503601	0.029355481	0.249197917	0.134405139	0.184078388	6	0	0
AQP1	0.6824969	0.68650698	-0.00401008	-0.00547328	0.134419557	0.184084079	3	0	0
CETN3	0.0514037	0.04677748	0.004626238	0.07694782	0.134442838	0.184101904	11	0	0
CCNL1	0.1659484	0.170971581	-0.0050232	-0.01669125	0.134488156	0.184144281	10	0	0
ZBTB45	0.0416107	0.018865342	0.022745388	0.841450266	0.134494318	0.184144281	2	0	0
PIK3CG	0.4860769	0.494868262	-0.00879139	-0.00316962	0.134558781	0.184218479	9	0	0
DFFB	0.0440207	0.042276297	0.001744428	0.042587171	0.134593231	0.184251579	10	0	0
SOCS1	0.0471355	0.045106429	0.002029104	0.063230181	0.13463816	0.184299018	14	0	0
ADPRHL2	0.338889	0.345220528	-0.00633152	0.003850065	0.134704413	0.184362854	7	0	0
ATXN7	0.0407571	0.03926328	0.001493862	0.038868594	0.134705353	0.184362854	10	0	0
RAP1B	0.135976	0.129900323	0.00607568	0.08211564	0.134737128	0.184392272	6	0	0
PTPN3	0.207202	0.1843616	0.022840442	0.194487965	0.134765656	0.184417243	7	0	0
USP49	0.0395692	0.035758843	0.003810351	0.066197421	0.13482371	0.184482611	7	0	0
MAP1S	0.2301255	0.238606224	-0.00848071	0.004499571	0.134849386	0.184503668	9	0	0
CALCRL	0.6290204	0.646457344	-0.01743695	-0.04617312	0.134934441	0.184566544	3	0	0

KIF2A	0.0660417	0.064138151	0.001903524	0.067418455	0.134918218	0.184566544	8	0	0
SCAMP2	0.045209	0.046016595	-0.00080763	-0.01496898	0.134936501	0.184566544	10	0	0
TMEM109	0.1711269	0.160398997	0.010727939	0.014784329	0.134975994	0.184606484	13	0	0
DEF8	0.2961721	0.287812316	0.008359766	0.063272086	0.134991828	0.184614063	2	0	0
CDCA7	0.1108246	0.112908317	-0.00208373	0.057137154	0.135033786	0.184657364	11	0	0
DDX60	0.2122348	0.211498793	0.00073601	-0.06507307	0.135049572	0.184664872	12	0	0
MRGBP	0.0852228	0.079940593	0.005282221	0.026223386	0.135092037	0.184708856	9	0	0
ASNSD1	0.1469536	0.158109809	-0.01115624	-0.03161983	0.135121751	0.184735401	11	0	0
CPSF4	0.2465219	0.254456171	-0.00793427	-0.01036976	0.135262758	0.184899994	8	0	0
TMEM145	0.2347176	0.235840481	-0.00112292	0.088250721	0.135259127	0.184899994	7	0	0
SCRIB	0.148184	0.150227717	-0.00204369	0.018009902	0.135403232	0.185077913	13	0	0
PRPF39	0.2926623	0.304691334	-0.01202908	-0.00757418	0.135492462	0.185185766	9	0	0
AGO3	0.0324275	0.030645724	0.001781755	0.05016105	0.135517124	0.185191249	9	0	0
CCDC157	0.1590465	0.169575956	-0.01052941	-0.01737617	0.1355087	0.185191249	10	0	0
ABHD17B	0.0515457	0.046103736	0.005441929	0.143546262	0.135607665	0.18528955	15	0	0
ARL4D	0.0289845	0.02707242	0.001912102	0.059842391	0.135609719	0.18528955	10	0	0
KIF23	0.1839256	0.192593027	-0.00866739	-0.03484503	0.135745417	0.185460832	12	0	0
BRIX1	0.0843681	0.094262471	-0.0098944	-0.01264602	0.135921495	0.185616563	12	0	0
RBM14	0.202962	0.210861832	-0.00789982	-0.02377965	0.135896609	0.185616563	9	0	0
RBM14-RBN	0.202962	0.210861832	-0.00789982	-0.02377965	0.135896609	0.185616563	9	0	0
RBM4	0.202962	0.210861832	-0.00789982	-0.02377965	0.135896609	0.185616563	9	0	0
AKAP10	0.3424323	0.31657545	0.02585685	0.165263654	0.135907467	0.185616563	10	0	0
RSRC1	0.1778547	0.158608015	0.019246684	0.392062391	0.135938865	0.18562615	7	0	0
EFR3B	0.1245094	0.135473655	-0.01096422	-0.02892092	0.136224322	0.186001784	10	0	0
CTNNBL1	0.144642	0.158341362	-0.01369941	-0.04719683	0.136266392	0.186045065	12	0	0
TMEM41A	0.2640215	0.275439909	-0.01141837	-0.03655688	0.136502392	0.18635309	11	0	0
GAS8	0.1857796	0.203075483	-0.0172959	-0.04563294	0.136514967	0.186356073	4	0	0
SCAPER	0.0471038	0.040779063	0.006324772	0.082689802	0.136549849	0.186389505	3	0	0
PDE6G	0.7907576	0.816233347	-0.02547579	-0.04518378	0.1365718	0.186405282	1	0	0
SNRPF	0.210438	0.215415045	-0.00497705	0.044898169	0.136611928	0.186445863	14	0	0
ZSWIM3	0.0913438	0.087503124	0.003840686	0.087532517	0.136622478	0.186446075	11	0	0
PLEKHA2	0.1479597	0.163654071	-0.0156944	-0.04469614	0.136647499	0.186466034	9	0	0
CNEP1R1	0.5974012	0.604806958	-0.00740572	0.023714654	0.136686215	0.186504675	4	0	0
C14orf178	0.0391518	0.038683363	0.000468477	0.02996488	0.136699109	0.18650808	7	0	0
JDP2	0.1363028	0.137008962	-0.00070613	0.177693598	0.136725624	0.186530067	13	0	0
FAM71E1	0.2752271	0.269447967	0.005779088	0.312520774	0.136744825	0.186542074	16	0	0
MGST2	0.3660879	0.377608262	-0.0115204	0.00759852	0.136765374	0.186555917	8	0	0
DOCK7	0.0447305	0.043130281	0.001600213	0.052358512	0.136852475	0.18665396	5	0	0
MSANTD4	0.089193	0.092561661	-0.00336864	0.139383831	0.136858064	0.18665396	14	0	0
NT5DC1	0.0789183	0.089081747	-0.01016348	-0.04148646	0.136964872	0.186785428	12	0	0
CS	0.2210218	0.206262702	0.01475913	0.095075486	0.13698621	0.186800324	9	0	0
ADO	0.2297294	0.226708621	0.00302074	0.005172433	0.137005427	0.186812326	7	0	0
ZZEF1	0.0374087	0.037359696	4.90E-05	0.009020847	0.137151966	0.186997922	11	0	0
ZC3H15	0.1482852	0.165022136	-0.01673691	-0.04072005	0.137202513	0.187049114	10	0	0
CCDC69	0.2835362	0.281904089	0.001632113	0.150661133	0.137226044	0.187049114	13	0	0
PEX12	0.1823073	0.185509686	-0.0032024	-0.03224786	0.137231227	0.187049114	13	0	0
FN3KRP	0.3660257	0.363261606	0.002764101	0.031791022	0.137228693	0.187049114	6	0	0
GAR1	0.054679	0.056773628	-0.0020946	-0.01623398	0.137268763	0.187086059	12	0	0
MARVELD2	0.3116284	0.307384614	0.004243758	-0.02586719	0.137297834	0.187097245	12	0	0
RB1	0.1358827	0.145635961	-0.00975327	0.012954169	0.1372962	0.187097245	20	0	0
SOCS4	0.2236041	0.218516867	0.005087189	0.020950967	0.137328576	0.187124922	8	0	0
RPLP1	0.1562163	0.171845531	-0.01562927	-0.07132791	0.137345538	0.187133817	9	0	0
COPG2	0.0530047	0.053877466	-0.00087273	-0.02756686	0.137439899	0.187233939	15	0	0
SSSCA1	0.0370358	0.040135272	-0.00309949	-0.0413807	0.137431232	0.187233939	8	0	0
CDKN2B	0.053299	0.038369624	0.014929354	0.439395987	0.13749734	0.187297964	4	0	0
CDK2AP2	0.1073726	0.11217054	-0.00479791	-0.03975895	0.137548112	0.187352896	7	0	0
EPHA7	0.0954549	0.093976874	0.001478065	0.084089113	0.137645264	0.187470989	8	0	0
PNPT1	0.1338719	0.145443475	-0.01157162	-0.02605099	0.137764517	0.187619163	5	0	0
HIST1H4B	0.3212598	0.338457437	-0.0171976	-0.02033754	0.137988083	0.187909366	13	0	0
ZNF189	0.0587494	0.067139827	-0.00839041	-0.0481816	0.138024756	0.187945037	11	0	0
BNIP3L	0.2533082	0.264689113	-0.01138094	-0.02203845	0.138423211	0.188458989	11	0	0
NR2C2AP	0.1407891	0.146973328	-0.00618425	-0.04091983	0.138414407	0.188458989	11	0	0
BIRC2	0.0737483	0.058842976	0.014905349	0.091105795	0.138458951	0.188479033	7	0	0

C18orf25	0.1447348	0.153216967	-0.00848219	-0.0004815	0.138453848	0.188479033	10	0	0
PHF5A	0.0393272	0.039409079	-8.19E-05	0.010172618	0.138528695	0.188545354	11	0	0
ACO2	0.0393272	0.039409079	-8.19E-05	0.010172618	0.138528695	0.188545354	11	0	0
SAMD7	0.8287686	0.842359957	-0.01359135	-0.02221135	0.138558944	0.188572214	2	0	0
E2F5	0.1127455	0.112884902	-0.00013944	0.021272023	0.138601094	0.188600957	10	0	0
MYLK3	0.2517104	0.239795188	0.01191523	0.39914438	0.13859754	0.188600957	4	0	0
TMEM222	0.1822224	0.191273329	-0.00905094	-0.01628451	0.138669304	0.188679459	12	0	0
SH3BP5L	0.1514733	0.169724666	-0.01825133	-0.04641214	0.138700248	0.188707247	9	0	0
VPS37D	0.1235201	0.130588161	-0.0070681	0.013909231	0.13881827	0.188853496	10	0	0
NFKBID	0.3764424	0.383515429	-0.00707302	-0.01469913	0.138843671	0.188859403	7	0	0
HCST	0.3764424	0.383515429	-0.00707302	-0.01469913	0.138843671	0.188859403	7	0	0
ERCC1	0.0398933	0.037262084	0.00263118	0.071825885	0.138964549	0.188995159	5	0	0
ISCA1	0.1734851	0.171071514	0.002413537	0.116369142	0.139068813	0.189108281	7	0	0
IPO8	0.0468882	0.045986522	0.000901662	0.007631444	0.139066427	0.189108281	6	0	0
GABPA	0.0995267	0.105033785	-0.00550706	0.024641126	0.13909579	0.189130626	7	0	0
VRK3	0.0536655	0.053267391	0.000398082	0.033297513	0.139215426	0.189278948	8	0	0
ZNF136	0.1717548	0.166936284	0.004818483	0.324156786	0.139283498	0.189357146	14	0	0
C19orf38	0.1604929	0.171955251	-0.01146234	-0.01739847	0.139300092	0.189365351	13	0	0
TMEM63A	0.0580289	0.062162174	-0.00413331	0.015545764	0.139313681	0.189369472	13	0	0
PDE5A	0.3592659	0.369387362	-0.01012143	-0.04722125	0.139346445	0.18938328	7	0	0
UBE2D4	0.0730123	0.07533267	-0.00232037	-0.03714992	0.139357297	0.18938328	10	0	0
APOLD1	0.0366865	0.035716231	0.00097026	0.021631938	0.139366074	0.18938328	11	0	0
ZNF555	0.0675229	0.064228572	0.003294373	0.028583262	0.139364878	0.18938328	8	0	0
ARHGAP28	0.1121808	0.097721177	0.014459595	0.180775482	0.139416822	0.189437888	3	0	0
TMEM107	0.160127	0.158739329	0.001387715	0.057436645	0.139456677	0.189477688	11	0	0
CACNG5	0.3539788	0.357291209	-0.00331242	0.003123228	0.139472818	0.189485264	5	0	0
IL11	0.3345871	0.347944567	-0.01335745	0.050557549	0.1395247	0.189541393	6	0	0
TMTC2	0.0561389	0.057874489	-0.00173557	-0.02808304	0.139542361	0.189551029	7	0	0
AGGF1	0.875944	0.884068483	-0.00812446	-0.01320718	0.139572513	0.189577629	3	0	0
ODF3B	0.1782579	0.183530019	-0.00527209	-0.05188985	0.139676832	0.189690593	6	0	0
KLF9	0.3252138	0.322054958	0.003158841	0.05283999	0.13970248	0.18971106	6	0	0
WNT2B	0.1134534	0.124977787	-0.01152443	-0.00256788	0.139752775	0.189764992	8	0	0
GPS1	0.5339703	0.523537567	0.010432767	0.031625706	0.139852282	0.189885732	4	0	0
YIPF3	0.0808861	0.086058574	-0.00517251	0.001688312	0.139885531	0.1899165	14	0	0
PIP4K2A	0.3103043	0.330064681	-0.01976034	-0.04273953	0.140033091	0.190102446	5	0	0
KIAA0408	0.7466096	0.756905704	-0.01029614	-0.01072325	0.140183782	0.190292614	4	0	0
FBXO11	0.034913	0.036634781	-0.00172175	-0.02958494	0.140328133	0.190474148	11	0	0
GPRIN3	0.3709878	0.36887259	0.00211517	0.150894032	0.140675636	0.190916935	10	0	0
DDX25	0.1324299	0.145835783	-0.01340587	-0.01169038	0.140675268	0.190916935	8	0	0
PDE4D	0.1504356	0.158468031	-0.00803247	-0.07048093	0.140722375	0.190965917	1	0	0
CHD6	0.1926392	0.19939392	-0.00675473	0.043588484	0.14074186	0.190977909	12	0	0
UBE3A	0.1446441	0.137015544	0.007628575	0.079340881	0.140803095	0.191046548	14	0	0
HIST2H2BE	0.0349501	0.034989065	-3.90E-05	0.010012056	0.140889919	0.191149893	16	0	0
PCDHB14	0.3268969	0.315305902	0.011591016	0.177429697	0.140925345	0.191177506	9	0	0
NTRK2	0.1267745	0.100305407	0.026469078	0.289291374	0.140931589	0.191177506	7	0	0
POLR3GL	0.1242007	0.136220702	-0.01202	-0.03410138	0.140964717	0.191186896	12	0	0
GOLGA7	0.2161067	0.229531736	-0.013425	-0.04170426	0.14097049	0.191186896	7	0	0
ZNF195	0.2811442	0.284173075	-0.00302892	-0.01768408	0.140956013	0.191186896	12	0	0
PCDHB16	0.6165278	0.584896472	0.031631334	0.0758992	0.140995559	0.191206438	4	0	0
BARHL1	0.2242477	0.204646626	0.019601039	0.135291682	0.141087672	0.191302425	10	0	0
MED23	0.0634356	0.062958981	0.000476577	-0.02592977	0.141113273	0.191308213	8	0	0
ENPP3	0.0634356	0.062958981	0.000476577	-0.02592977	0.141113273	0.191308213	8	0	0
FUBP1	0.0890586	0.085469337	0.003589244	0.067358103	0.141142467	0.191310917	11	0	0
FXYD5	0.0814054	0.077910411	0.003494978	0.149953769	0.141128101	0.191310917	13	0	0
AHCY	0.1468363	0.12581004	0.021026224	0.304841754	0.141147266	0.191310917	7	0	0
CCDC110	0.0904712	0.097421039	-0.00694984	-0.0169987	0.141228461	0.191406504	7	0	0
ZNF785	0.0919662	0.078138514	0.013827639	0.275156489	0.141315977	0.191510644	10	0	0
CTH	0.0506036	0.048814937	0.00178871	0.039247641	0.141401221	0.191597212	10	0	0
TBC1D23	0.1697524	0.156535894	0.013216456	0.101792522	0.141449171	0.191647706	15	0	0
NDUFS6	0.1053753	0.071737469	0.033637816	0.456220114	0.141537522	0.191730306	8	0	0
FBXO32	0.1351828	0.138717624	-0.00353486	0.087158113	0.141529619	0.191730306	7	0	0
SLC7A1	0.4806641	0.493649173	-0.01298503	-0.04174456	0.141542204	0.191730306	6	0	0
LRRC7	0.0720201	0.059709165	0.012310951	0.260897553	0.141646222	0.191856716	16	0	0

SLC10A7	0.0385889	0.038135992	0.000452932	0.023853948	0.141685115	0.191894904	11	0	0
TMEM59L	0.0735127	0.065881702	0.007630959	0.131235355	0.141898684	0.192169646	9	0	0
ERBB4	0.0855661	0.072039258	0.013526844	0.210640467	0.141961635	0.192232299	13	0	0
LRRTM3	0.2633327	0.281706767	-0.01837407	-0.04311954	0.141966382	0.192232299	6	0	0
CCDC142	0.0591214	0.057169933	0.001951467	0.003810905	0.142005342	0.192261538	12	0	0
BBS10	0.1609798	0.1668225	-0.00584268	-0.07551291	0.142009414	0.192261538	10	0	0
MAATS1	0.1814817	0.193801117	-0.01231945	-0.05565302	0.142030604	0.192275712	5	0	0
TATDN2	0.423721	0.42818767	-0.00446668	-0.02659356	0.142178042	0.192460782	4	0	0
ZMYND19	0.3006842	0.305704081	-0.00501985	0.058562525	0.142207043	0.192485512	3	0	0
MLX	0.2732165	0.274368504	-0.00115196	0.012976842	0.142225851	0.192496443	10	0	0
VASP	0.180838	0.170257611	0.010580364	0.069084636	0.1422487	0.192512842	3	0	0
SLC25A37	0.0914056	0.088693337	0.002712312	0.024383074	0.142261954	0.192516252	9	0	0
TMEM242	0.187172	0.187476875	-0.00030483	0.049559977	0.142367892	0.192645078	8	0	0
CAP1	0.091522	0.095386949	-0.00386491	0.00805025	0.142673254	0.19303139	10	0	0
NUP214	0.0516582	0.054791257	-0.00313308	-0.0155241	0.142674908	0.19303139	8	0	0
PTPRU	0.245871	0.250902857	-0.00503183	0.023907644	0.142801077	0.193187518	6	0	0
NUP85	0.0506007	0.052912843	-0.00231211	-0.05232183	0.142862629	0.193256213	9	0	0
GSDMD	0.1351903	0.093808028	0.041382289	0.48474285	0.142919199	0.193318156	2	0	0
RPS25	0.052817	0.051081556	0.001735427	0.053973916	0.143017213	0.193436146	7	0	0
TCF19	0.1004118	0.096873343	0.003538495	0.076984925	0.143083001	0.193510534	8	0	0
CLTC	0.0399279	0.040786569	-0.00085862	-0.03315938	0.143167624	0.193610382	6	0	0
HABP4	0.1060995	0.10862613	-0.0025266	0.173462607	0.143182961	0.193616525	10	0	0
DNAH6	0.2601124	0.268540924	-0.00842854	0.110751918	0.143220154	0.193652218	12	0	0
IAPP	0.7534923	0.740856444	0.012635872	0.0220489	0.143267815	0.19370206	3	0	0
IZUMO1	0.4121692	0.384008588	0.028160651	0.157726312	0.143280478	0.19370458	7	0	0
ABCC10	0.308702	0.304979994	0.003722053	-0.01426576	0.143298719	0.193714639	10	0	0
PAOX	0.1014877	0.10280256	-0.00131488	0.016487183	0.143313153	0.19371955	17	0	0
ANXA3	0.3642278	0.371887852	-0.00766008	0.09032866	0.14333828	0.193738914	9	0	0
TMEM79	0.0779368	0.081387715	-0.00345091	-0.01007544	0.143398086	0.193805145	11	0	0
CNTN5	0.1094381	0.105296921	0.004141151	0.175231666	0.14344398	0.193852564	14	0	0
FZR1	0.12945	0.136439142	-0.00698916	-0.00229529	0.143462675	0.193863222	8	0	0
B4GALT3	0.3118946	0.314697568	-0.00280292	0.04940439	0.143529578	0.193939017	17	0	0
MPP2	0.2550745	0.267887971	-0.01281349	0.00374536	0.143631696	0.19406238	3	0	0
TMEM101	0.7838605	0.791965948	-0.00810549	-0.0110061	0.143661915	0.19408859	5	0	0
TNRC6B	0.114318	0.121780058	-0.0074621	0.000564464	0.143792334	0.194250155	11	0	0
NAA35	0.0683425	0.070381622	-0.00203913	0.057429538	0.143861221	0.194328578	8	0	0
PASK	0.0964773	0.091034616	0.005442658	0.022160353	0.14388918	0.194351708	5	0	0
ZNF77	0.1736039	0.186200268	-0.01259637	-0.06111606	0.143911505	0.194367225	7	0	0
LBH	0.1710656	0.150905553	0.020160075	0.217116336	0.143940019	0.194376463	11	0	0
RIMBP2	0.1239393	0.132100766	-0.00816145	0.060248953	0.143937363	0.194376463	9	0	0
ECE1	0.1639639	0.167949542	-0.0039856	0.000873252	0.144067713	0.194534255	4	0	0
AMIGO2	0.0603492	0.050586203	0.009762956	0.251495651	0.144178775	0.194669566	14	0	0
BOLA1	0.0350636	0.035125793	-6.22E-05	0.00925159	0.144193939	0.194675385	16	0	0
DNAJC27	0.1160322	0.121146749	-0.00511457	-0.03519612	0.144269595	0.194748209	9	0	0
RPL18A	0.0299239	0.028947761	0.000976121	0.036141771	0.144264786	0.194748209	7	0	0
FAM49B	0.3024875	0.308490017	-0.00600248	0.132820179	0.144349111	0.194840883	3	0	0
STARD4	0.1249593	0.118571066	0.006388225	0.035765735	0.144391066	0.194882847	13	0	0
RDH13	0.6246837	0.651065838	-0.02638216	-0.0763369	0.14453794	0.195051726	2	0	0
EPS8L1	0.6246837	0.651065838	-0.02638216	-0.0763369	0.14453794	0.195051726	2	0	0
PDCD1	0.3509967	0.345297075	0.005699592	0.006909451	0.144566793	0.195075985	8	0	0
WDR70	0.4506189	0.457079883	-0.00646095	-0.00074942	0.144590684	0.195093547	8	0	0
SPATA5	0.0479777	0.047036373	0.000941313	-0.02969045	0.14460716	0.1951011	17	0	0
POLR2B	0.041001	0.041657885	-0.00065687	0.005782702	0.144621308	0.195105512	8	0	0
SGCB	0.1263568	0.123540399	0.002816424	0.008196399	0.144688629	0.195168549	10	0	0
ARHGAP39	0.1576619	0.144521927	0.013139945	0.132126864	0.144690518	0.195168549	5	0	0
NCBP1	0.0461458	0.049838507	-0.00369269	-0.0386497	0.144700678	0.195168549	18	0	0
KIAA0319	0.1010004	0.115229358	-0.01422896	-0.07540514	0.14475612	0.195227443	9	0	0
TRADD	0.1273225	0.117150149	0.01017235	0.020992327	0.144766112	0.195227443	14	0	0
LRRC8B	0.0631171	0.065911608	-0.00279452	0.006734546	0.144807061	0.195253304	10	0	0
TIMM10B	0.2531808	0.270358314	-0.01717753	-0.10322148	0.144799283	0.195253304	10	0	0
ARFGAP3	0.8375811	0.853628523	-0.01604742	-0.02699926	0.144862413	0.195313256	5	0	0
NOM1	0.1950671	0.207907584	-0.01284044	-0.01327091	0.144878571	0.195320358	10	0	0
WDR43	0.4141593	0.425139494	-0.01098016	-0.03238667	0.144919041	0.195346506	10	0	0

WDR92	0.0901799	0.099237374	-0.00905744	-0.0381816	0.14493064	0.195346506	13	0	0
DUSP6	0.0512267	0.049128821	0.002097849	0.097058692	0.144925669	0.195346506	10	0	0
CDX1	0.7124148	0.731509099	-0.01909433	-0.03242069	0.145145778	0.195607084	7	0	0
MRPL10	0.1881914	0.181496181	0.006695184	0.04598729	0.145135213	0.195607084	8	0	0
TOP2A	0.084545	0.086759538	-0.00221451	0.021858273	0.145196593	0.195660863	19	0	0
DESI2	0.2284345	0.231308002	-0.00287349	0.015610186	0.145314906	0.195777995	7	0	0
SRCIN1	0.1289705	0.121937544	0.007032926	0.051349736	0.145316261	0.195777995	6	0	0
SCAF1	0.0453877	0.048315738	-0.00292808	-0.02064851	0.145298527	0.195777995	10	0	0
TMEM8A	0.8941099	0.905121087	-0.0110112	-0.01746452	0.145384256	0.19585489	1	0	0
NCEH1	0.0817683	0.08227107	-0.00050273	0.049191096	0.145469065	0.195954424	11	0	0
GXYLT2	0.0891098	0.092574749	-0.003465	-0.03161407	0.145482274	0.195956284	5	0	0
NDC80	0.0529805	0.055178653	-0.00219816	-0.04327675	0.145492297	0.195956284	11	0	0
MAPK8	0.2726307	0.286257579	-0.01362686	-0.04595029	0.145565403	0.19602704	9	0	0
MFSD6L	0.4857132	0.465332152	0.020381096	0.058446874	0.14556669	0.19602704	5	0	0
ANP32E	0.1227102	0.128040941	-0.00533077	0.012445868	0.145652023	0.196127229	18	0	0
NPY4R	0.2491207	0.261050269	-0.01192954	-0.06948384	0.145743012	0.196235017	5	0	0
RNF219	0.2826542	0.267822976	0.014831174	0.140648391	0.145828711	0.196335667	10	0	0
EGR4	0.2019242	0.184662754	0.017261466	0.454838643	0.145844877	0.196342528	6	0	0
PACS2	0.1190447	0.123122937	-0.00407824	0.013253023	0.1458557	0.196342528	13	0	0
SMARCE1	0.1185904	0.113638888	0.004951492	0.046520161	0.14591629	0.19639461	11	0	0
RAB40B	0.1515941	0.159074371	-0.00748023	-0.02402882	0.145910836	0.19639461	7	0	0
ITPKC	0.043485	0.04349978	-1.48E-05	-0.00868144	0.145936911	0.196407627	12	0	0
TBC1D8	0.0419126	0.044489905	-0.00257727	-0.01546343	0.146029923	0.19651806	7	0	0
MAP3K12	0.0429851	0.040468556	0.002516576	0.055399956	0.146209469	0.19674492	18	0	0
ESCO2	0.1419925	0.128725005	0.013267446	0.052451442	0.146242605	0.196774746	3	0	0
EHD3	0.2117731	0.218712166	-0.00693902	0.187728458	0.146368077	0.1969288	15	0	0
TRAFD1	0.0522688	0.051627579	0.00064122	0.03196642	0.146412392	0.19696117	8	0	0
TSPO	0.128125	0.126439955	0.001684998	0.034686941	0.146414099	0.19696117	14	0	0
ENKUR	0.0326207	0.033948327	-0.00132766	-0.01843724	0.146559847	0.197142449	7	0	0
LRRC43	0.7176104	0.730095279	-0.01248491	-0.02335036	0.146580978	0.197156087	6	0	0
NR4A2	0.0429161	0.042866039	5.01E-05	0.014190833	0.146609066	0.19717908	5	0	0
ADAMTS6	0.0451993	0.041150316	0.004049024	0.1058134	0.146623243	0.197183361	8	0	0
G2E3	0.1198884	0.128231315	-0.00834293	-0.02954034	0.146670983	0.197232775	15	0	0
RPUSD4	0.0547533	0.051221839	0.003531484	0.045258963	0.146779971	0.197349742	14	0	0
IRF3	0.1106456	0.118583994	-0.00793844	-0.02094816	0.146774914	0.197349742	9	0	0
THADA	0.0989721	0.113422545	-0.01445041	-0.02192237	0.146797603	0.197358655	12	0	0
RHCE	0.0325765	0.033112287	-0.00053582	-0.0140402	0.146833338	0.19737671	13	0	0
PPIG	0.3014624	0.311228881	-0.00976649	-0.0224815	0.146844046	0.19737671	8	0	0
CCNG1	0.0991312	0.093822664	0.005308546	0.35034615	0.146833566	0.19737671	14	0	0
UQCRH	0.0410329	0.038870342	0.002162541	0.062208282	0.146943059	0.197493431	14	0	0
MICB	0.1446338	0.137083964	0.007549867	0.072216023	0.146952906	0.197493431	1	0	0
CACNA2D1	0.2988886	0.293432822	0.005455798	0.092796102	0.146996667	0.197537441	11	0	0
ACTR2	0.0835336	0.094340161	-0.01080659	-0.02587467	0.147069724	0.19762081	12	0	0
SNAI2	0.065257	0.058357343	0.006899646	0.144117769	0.147092974	0.197637245	9	0	0
SLC9B1	0.1377214	0.140217565	-0.00249613	-0.00311423	0.147174964	0.197732596	11	0	0
SEC22B	0.3838172	0.389610802	-0.00579361	0.020481863	0.147442845	0.198077662	9	0	0
STAMBP	0.2786549	0.293483574	-0.01482865	-0.03081647	0.147565465	0.198227544	12	0	0
RGS17	0.2799092	0.256110576	0.023798592	0.292680015	0.147595128	0.198252544	6	0	0
OVOL3	0.8371523	0.853138261	-0.01598594	-0.02830799	0.147654516	0.198317463	4	0	0
FAF2	0.0557375	0.054998149	0.000739381	0.025100437	0.147697019	0.198359696	5	0	0
C11orf54	0.065807	0.064283787	0.001523171	0.030883127	0.147731796	0.198391547	11	0	0
HAL	0.7403372	0.750557892	-0.01022073	-0.02330053	0.147877489	0.198572332	4	0	0
CAP2	0.324159	0.307832778	0.016326207	0.113534955	0.14792838	0.198610931	9	0	0
TMEM38B	0.1235595	0.13816531	-0.01460582	-0.06034707	0.147919138	0.198610931	4	0	0
LRRC46	0.1720121	0.181601537	-0.00958947	0.005895347	0.147944276	0.198617405	7	0	0
NUP107	0.1780549	0.188296742	-0.01024185	-0.02908914	0.147962268	0.198626692	10	0	0
NCOA2	0.0648422	0.062465419	0.002376767	0.118612563	0.147974635	0.198628428	7	0	0
SMIM18	0.8659811	0.876740244	-0.01075915	-0.01706482	0.148065691	0.19873578	2	0	0
BRIP1	0.1147527	0.116489521	-0.00173684	0.011554757	0.14812082	0.198794899	11	0	0
FAM133B	0.1768922	0.176881514	1.07E-05	0.057261777	0.148369841	0.199114214	12	0	0
NCK1	0.0906022	0.102152601	-0.01155044	-0.0276799	0.148444902	0.199140445	10	0	0
TAF1L	0.6345903	0.64925488	-0.01466462	-0.03709279	0.148432353	0.199140445	3	0	0
KLHL25	0.1900876	0.184531141	0.005556485	0.034804621	0.14843031	0.199140445	12	0	0

GGN	0.1132275	0.101164458	0.012063025	0.147845099	0.14843864	0.199140445	8	0	0
SCAF4	0.0474228	0.050965046	-0.00354223	-0.07679617	0.148421632	0.199140445	5	0	0
KANK3	0.1137123	0.112801169	0.000911097	0.084287062	0.148476646	0.199168134	9	0	0
CCNDBP1	0.4054523	0.401131348	0.004320933	0.002309505	0.148488825	0.199169575	12	0	0
MYL12A	0.0801418	0.086975165	-0.00683338	-0.03423834	0.148523682	0.199186538	10	0	0
CYB5R3	0.2270902	0.241691556	-0.01460134	-0.00028066	0.148520494	0.199186538	7	0	0
FKBP1B	0.1553394	0.149776008	0.005563417	0.170215951	0.148689156	0.199391404	11	0	0
WDYHV1	0.0433231	0.040169627	0.003153443	0.080461551	0.148698674	0.199391404	10	0	0
ALK	0.0872392	0.080205811	0.007033434	0.13171376	0.148783681	0.199490476	10	0	0
C22orf23	0.0839087	0.08357033	0.00033838	0.004460748	0.148804792	0.199503869	10	0	0
ASNS	0.0745846	0.081214564	-0.00662998	-0.06847887	0.148817505	0.199506	8	0	0
PSMC5	0.2481137	0.261522478	-0.01340883	-0.0460587	0.148872968	0.199565437	8	0	0
ARHGEF26	0.305332	0.304071763	0.001260208	0.055980756	0.148893524	0.199578077	11	0	0
KRTAP5-11	0.1413243	0.149801106	-0.00847685	-0.07863412	0.148916809	0.199594373	1	0	0
KLC4	0.9177288	0.9299737	-0.0122449	-0.01891726	0.148937763	0.199607541	1	0	0
SLC25A44	0.1401266	0.148089384	-0.00796276	-0.01296248	0.149055102	0.199749655	8	0	0
TADA2A	0.0542225	0.051598994	0.002623462	0.044506954	0.149066076	0.199749655	6	0	0
SUMO2	0.2870926	0.294311703	-0.00721911	-0.03956723	0.149116665	0.199802518	4	0	0
BAG5	0.0731266	0.070277758	0.002848885	0.061848663	0.149180528	0.199873157	7	0	0
BAZ1B	0.0415339	0.042275746	-0.00074187	-0.01109179	0.149293028	0.200008945	8	0	0
PLS1	0.0639893	0.0697675	-0.00577816	-0.04873385	0.149339824	0.200056694	11	0	0
IL34	0.582958	0.607420948	-0.02446295	-0.05040051	0.149359899	0.200068645	6	0	0
ZNF772	0.2203399	0.220954027	-0.0006141	0.130415798	0.149385873	0.200088493	11	0	0
ACOX3	0.1271027	0.141628774	-0.01452603	-0.02625926	0.149443271	0.200150426	13	0	0
CLIP3	0.8090189	0.807647076	0.001371843	0.002570605	0.149527595	0.200248409	4	0	0
NARS	0.1926592	0.206062126	-0.01340294	-0.03016173	0.149587439	0.200313597	8	0	0
AMACR	0.0379862	0.036110143	0.001876026	0.045612669	0.149687229	0.200432262	9	0	0
ZNF653	0.2096154	0.215156406	-0.00554099	-0.00144928	0.149770237	0.200528439	11	0	0
CHMP2A	0.2808099	0.29118258	-0.0103727	-0.01972761	0.149811963	0.200569334	15	0	0
ZNF250	0.0491941	0.049938347	-0.00074422	-0.02751218	0.149876618	0.200640918	4	0	0
TOPORS	0.1565813	0.154167764	0.002413573	0.067176981	0.149945209	0.20071776	6	0	0
TECPR2	0.0412338	0.038178541	0.003055253	0.079488601	0.14999943	0.200775357	14	0	0
TM9SF3	0.1372458	0.143553023	-0.0063072	-0.01141145	0.150037956	0.200797777	6	0	0
GLYR1	0.030347	0.031824435	-0.00147747	-0.03572618	0.150038571	0.200797777	8	0	0
WDR77	0.1222479	0.125073859	-0.00282597	-0.01790583	0.150130799	0.200890328	11	0	0
PPM1J	0.0565961	0.057425994	-0.00082985	-0.01168232	0.150126522	0.200890328	9	0	0
MAFG	0.1254094	0.136986289	-0.01157687	0.012480068	0.150141327	0.200890328	12	0	0
PDF	0.2597805	0.265884531	-0.00610402	-0.00625822	0.150289572	0.201073681	7	0	0
TTC37	0.0795297	0.069364616	0.010165114	0.063041465	0.150441364	0.201246742	14	0	0
WDR73	0.0876139	0.087794873	-0.00018101	-0.00647972	0.150435634	0.201246742	15	0	0
TPRKB	0.0876524	0.100470278	-0.01281792	-0.04940511	0.150465523	0.201264048	7	0	0
ORMDL2	0.0509989	0.049884775	0.001114096	0.010777968	0.150479119	0.201267224	16	0	0
CHEK2	0.2631085	0.265669619	-0.00256113	0.008993009	0.150549807	0.201346755	12	0	0
ETFDH	0.0399042	0.039519776	0.000384438	0.02709091	0.150598443	0.201384481	12	0	0
WNT1	0.16629	0.156918479	0.009371529	0.272024328	0.150600471	0.201384481	15	0	0
ERI2	0.0477376	0.048400076	-0.00066245	-0.00748926	0.150656266	0.201429056	14	0	0
DCUN1D3	0.0477376	0.048400076	-0.00066245	-0.00748926	0.150656266	0.201429056	14	0	0
USP38	0.2318173	0.2429978	-0.01118054	-0.00248327	0.150700277	0.201472881	6	0	0
CLIC1	0.0661284	0.059383461	0.006744987	0.135974706	0.150873294	0.201689156	20	0	0
IL17RB	0.2337851	0.225022542	0.008762532	0.205440243	0.15094123	0.201764935	9	0	0
PIH1D2	0.0977089	0.099660504	-0.00195164	0.048847286	0.151004079	0.201833905	13	0	0
CIR1	0.1003829	0.103502187	-0.00311928	-0.01189519	0.151216211	0.202102382	13	0	0
ECSIT	0.0292952	0.029785216	-0.00049003	-0.00964484	0.151528886	0.202505186	11	0	0
PHF7	0.047859	0.04622946	0.001629505	0.094815613	0.15162247	0.202600061	11	0	0
METTL3	0.1812898	0.185184291	-0.00389451	0.031086615	0.151613655	0.202600061	12	0	0
EPHB2	0.0288128	0.029357463	-0.00054461	-0.01843701	0.151659354	0.20263425	7	0	0
HCN2	0.1445058	0.144268571	0.000237273	0.052982156	0.151684226	0.202652386	3	0	0
UHRF1BP1	0.0878389	0.086105091	0.001733846	0.037055966	0.151707884	0.202668897	12	0	0
NOA1	0.0997663	0.09801262	0.001753693	0.015016339	0.151761289	0.202710044	8	0	0
TMEM30A	0.1179497	0.127159058	-0.00920938	-0.01571522	0.151757578	0.202710044	9	0	0
CXCL17	0.5584545	0.586065671	-0.02761118	-0.06385607	0.151777405	0.202716475	6	0	0
RNFT1	0.0325561	0.032222014	0.000334098	0.0138005	0.151876746	0.202834052	10	0	0
PLEKHG5	0.5440135	0.527746301	0.016267182	0.054945035	0.151951058	0.202918187	8	0	0

APLP2	0.1715316	0.173197383	-0.00166581	0.026815436	0.152158928	0.203180653	9	0	0
FKTN	0.1484628	0.152291631	-0.00382885	-0.05730315	0.152195884	0.203199745	6	0	0
ACRBP	0.3058991	0.274643796	0.031255293	0.293909156	0.152195383	0.203199745	6	0	0
ZNF236	0.039662	0.037743614	0.001918377	0.023882449	0.152249321	0.20325596	4	0	0
GNG3	0.4507931	0.45323023	-0.00243718	0.024739753	0.15227264	0.203271962	16	0	0
PLAT	0.5150555	0.501572931	0.013482555	0.052249541	0.152447482	0.203490217	9	0	0
TMEM62	0.1116785	0.116984947	-0.00530646	-0.05676759	0.152655421	0.203752614	2	0	0
ZZZ3	0.0407166	0.040173375	0.000543188	0.025183555	0.152695804	0.20379135	7	0	0
LRRC20	0.2111376	0.205152249	0.005985351	0.106516478	0.152731097	0.203823286	5	0	0
PRKCD	0.3887883	0.416885621	-0.02809733	-0.08181428	0.152791636	0.203888906	2	0	0
CELA2A	0.9087599	0.919999412	-0.01123949	-0.01754188	0.15292093	0.204015904	1	0	0
ODC1	0.2043647	0.188007272	0.016357428	0.066465352	0.152901191	0.204015904	12	0	0
PTPRZ1	0.3342845	0.319535841	0.014748639	0.144507553	0.152938547	0.204024231	7	0	0
VCP	0.416532	0.418357146	-0.00182516	0.056736002	0.152995189	0.204084613	9	0	0
PIGS	0.2203142	0.211483353	0.008830885	0.056357791	0.153136551	0.204257989	14	0	0
RPS13	0.1142175	0.1202859	-0.00606839	-0.03239815	0.153166087	0.204282193	11	0	0
MRPL11	0.1083177	0.097394952	0.010922706	0.072548375	0.153203999	0.204317565	9	0	0
FAM104A	0.7792905	0.767586843	0.011703613	0.021552496	0.15329226	0.204420073	1	0	0
SUCLA2	0.079669	0.084175965	-0.00450692	0.01755214	0.153307986	0.204425844	7	0	0
CYB5R4	0.1406907	0.149046488	-0.00835582	-0.0053781	0.153424559	0.204566078	8	0	0
ANKRD13A	0.1086807	0.111279672	-0.00259892	-0.01610886	0.153620084	0.204804583	11	0	0
HAS3	0.2054633	0.209716816	-0.00425353	-0.00218249	0.153626275	0.204804583	13	0	0
CEP170	0.0741215	0.086243932	-0.01212241	-0.0242313	0.153813685	0.205039186	14	0	0
NCKAP5L	0.0988775	0.10494278	-0.00606524	9.79E-05	0.153946254	0.205200654	12	0	0
ADAM15	0.0450615	0.048287392	-0.00322592	-0.04181527	0.153960348	0.205204191	6	0	0
TCF25	0.0349713	0.031076878	0.003894406	0.126230591	0.154004053	0.20524719	4	0	0
BACH2	0.4434819	0.459183353	-0.01570146	-0.04150469	0.154053696	0.205298097	9	0	0
PMPCA	0.0729339	0.073556701	-0.0006228	0.092102716	0.15411169	0.205360125	9	0	0
ZNF623	0.5563141	0.559617855	-0.00330376	0.007585718	0.154157009	0.205405253	5	0	0
GBF1	0.1092285	0.112695823	-0.00346731	-0.0101354	0.154216393	0.205469115	10	0	0
MEF2D	0.0609856	0.051272554	0.009713082	0.073595558	0.154279013	0.205537278	8	0	0
BBX	0.203479	0.204189034	-0.00071007	0.009315488	0.154317413	0.205573167	10	0	0
TBC1D9B	0.4071179	0.417650621	-0.01053268	0.032179725	0.154390308	0.205654999	6	0	0
CHKB	0.468846	0.488374298	-0.01952831	-0.02187871	0.154411174	0.205667519	4	0	0
ARPC3	0.3313056	0.334007069	-0.00270147	0.025367896	0.154448621	0.205702122	11	0	0
TERF1	0.1986533	0.213056879	-0.0144036	-0.024799	0.154522155	0.205769498	10	0	0
PPP5C	0.3199975	0.322445355	-0.0024479	0.009193936	0.154540921	0.20577921	12	0	0
UBASH3A	0.638462	0.662978128	-0.02451611	-0.05140594	0.154705607	0.205983207	6	0	0
NADK2	0.2538243	0.259090338	-0.005266	0.002411687	0.154838595	0.206144971	9	0	0
SERGEF	0.0775583	0.074644062	0.00291421	-0.00185206	0.154893597	0.206202892	13	0	0
RBM39	0.0355638	0.032265615	0.003298173	0.077485647	0.154932042	0.206238764	13	0	0
SOCS5	0.2868203	0.296215903	-0.0093956	-0.02708064	0.154985716	0.206294902	9	0	0
SETBP1	0.0509198	0.047692738	0.003227028	0.069385722	0.155010019	0.20631194	13	0	0
RBM4B	0.0361959	0.035085958	0.001109905	0.037265673	0.155035806	0.206330951	12	0	0
RPS9	0.2432749	0.247630319	-0.0043554	0.028759491	0.155132744	0.206444642	10	0	0
FAM222A	0.1491084	0.161959991	-0.01285156	-0.06323365	0.155170289	0.206479287	11	0	0
C8orf37	0.0506274	0.052784572	-0.00215722	-0.05029963	0.155250901	0.206571228	3	0	0
ESYT1	0.0411393	0.041986423	-0.00084708	-0.02196586	0.155361738	0.206679412	12	0	0
DDX55	0.1086967	0.115715428	-0.00701868	-0.01746859	0.155355877	0.206679412	9	0	0
DUSP3	0.0815539	0.083989592	-0.00243567	0.048928704	0.155366777	0.206679412	13	0	0
SH3GL2	0.1380452	0.134835798	0.003209399	0.184281812	0.15540647	0.206716883	10	0	0
UQCRFS1	0.1649974	0.176061528	-0.01106408	-0.04160904	0.155573097	0.206923179	9	0	0
MAPK8IP3	0.2246763	0.230096521	-0.00542026	0.003827898	0.155649336	0.207009231	8	0	0
GATAD1	0.324704	0.327269836	-0.00256581	-0.03038998	0.1557616	0.207143179	9	0	0
WFS1	0.1131796	0.118667352	-0.0054878	0.025396748	0.155779265	0.207145602	9	0	0
SLC43A1	0.0510239	0.046369414	0.004654439	0.248406709	0.15578652	0.207145602	10	0	0
HPS3	0.0546458	0.046301748	0.008344025	0.054444256	0.15580576	0.207153668	9	0	0
STX10	0.0488886	0.044454653	0.004433963	0.085045603	0.155815685	0.207153668	14	0	0
TARBP2	0.0443858	0.041236884	0.00314893	0.069510924	0.155841911	0.207157824	16	0	0
NAGS	0.2598138	0.266961561	-0.00714775	-0.01648593	0.155834356	0.207157824	12	0	0
PANK2	0.1281142	0.121518074	0.006596113	0.064837494	0.155903964	0.207224952	11	0	0
TSPAN14	0.2385123	0.259579061	-0.02106674	-0.06181093	0.155931869	0.207246685	5	0	0
B3GNT9	0.1794572	0.170216022	0.009241207	0.11384263	0.15596917	0.207280267	10	0	0

DHX40	0.4555426	0.461330571	-0.00578796	0.021983089	0.15598025	0.207280267	12	0	0
MTFR1	0.0481337	0.044517913	0.003615757	0.086047588	0.156032472	0.207334303	10	0	0
RAD21	0.072581	0.078796574	-0.00621559	-0.04963447	0.156124883	0.207411	11	0	0
HPS5	0.0507575	0.051754929	-0.00099746	-0.00751512	0.156117511	0.207411	12	0	0
HSP90AA1	0.0833682	0.084133535	-0.00076537	-0.03177832	0.156104708	0.207411	12	0	0
MAP3K6	0.1569455	0.147085973	0.00985956	0.017410075	0.156136838	0.207411519	12	0	0
PPIL4	0.0989871	0.109604884	-0.01061774	-0.01269244	0.156178676	0.207451732	12	0	0
SHANK1	0.3866541	0.407447686	-0.0207936	0.010978622	0.156228236	0.207502195	5	0	0
AP5M1	0.0589793	0.057620182	0.001359113	0.003121414	0.156258599	0.207527156	11	0	0
CDRT15	0.8686938	0.87565767	-0.00696384	-0.0113886	0.156308498	0.207578056	1	0	0
H2AFJ	0.228512	0.22169668	0.006815276	0.230934771	0.156331059	0.207592646	14	0	0
FRA10AC1	0.0460529	0.047573393	-0.00152046	-0.01042594	0.156456254	0.207743513	11	0	0
NNT	0.0769919	0.077185054	-0.00019318	0.036555614	0.156469549	0.207745786	15	0	0
CNTD1	0.3476817	0.366548889	-0.01886723	-0.04405885	0.156501708	0.207773105	14	0	0
ZBTB34	0.1955122	0.193904752	0.001607481	-0.01404807	0.156680413	0.207994959	5	0	0
SEC24C	0.0364498	0.034379004	0.002070807	0.065140739	0.156704703	0.208011809	7	0	0
LINGO2	0.5022621	0.461798694	0.040463442	0.118710833	0.156864066	0.20820794	1	0	0
CKAP5	0.1240562	0.126468472	-0.00241226	0.006285188	0.157185373	0.208618977	11	0	0
ABAT	0.0396751	0.037712565	0.001962576	0.056066497	0.157215232	0.208643166	7	0	0
SETX	0.0261042	0.023426186	0.002678055	0.104718263	0.157236912	0.2086565	2	0	0
BICD2	0.1295359	0.143593798	-0.01405794	0.018054795	0.157285053	0.208689504	6	0	0
CENPJ	0.0416901	0.042513946	-0.00082383	-0.01914791	0.157284649	0.208689504	9	0	0
EFTUD2	0.116256	0.124233905	-0.00797786	0.005874197	0.157367487	0.208783435	9	0	0
DDX6	0.0471929	0.041401016	0.005791906	0.129889132	0.157417847	0.208834801	12	0	0
DUSP8	0.0275374	0.021934972	0.005602416	0.193757258	0.157477353	0.208898291	4	0	0
ZMYND11	0.1841952	0.190961351	-0.00676613	0.02297409	0.157542604	0.208969394	16	0	0
ETNK2	0.3035489	0.293734931	0.009814006	0.230235909	0.157642729	0.209086741	11	0	0
HINT2	0.2340553	0.236299072	-0.00224379	0.022705249	0.157775214	0.209246986	8	0	0
NDOR1	0.1557888	0.165566967	-0.0097782	-0.02257447	0.158073417	0.209626972	13	0	0
EPB42	0.6799884	0.701178198	-0.02118984	-0.04753716	0.158102529	0.209634579	5	0	0
KCTD11	0.185231	0.168271158	0.016959848	0.07787445	0.158092083	0.209634579	8	0	0
CWC15	0.1478091	0.152121679	-0.00431263	-0.01854036	0.15813347	0.209644609	16	0	0
POT1	0.2079296	0.197870651	0.010058988	0.035313478	0.158217384	0.209740354	11	0	0
IGFBP5	0.2581659	0.257325529	0.000840406	0.095099798	0.158342197	0.209890299	11	0	0
UBE2F	0.0469461	0.044589782	0.002356291	0.040540773	0.158413222	0.209968929	8	0	0
PTPN13	0.1087016	0.110345454	-0.00164389	-0.01165629	0.158440891	0.209990085	16	0	0
TESK2	0.0444271	0.042612082	0.001815067	0.044422113	0.158577558	0.210155688	8	0	0
GNPTG	0.2002893	0.210694467	-0.01040513	0.008137907	0.158589667	0.210156207	12	0	0
PIAS2	0.8512617	0.877895244	-0.0266335	-0.04393772	0.158741893	0.210342389	1	0	0
ALDH16A1	0.0734786	0.07445173	-0.00097312	0.006124838	0.158875147	0.210503408	8	0	0
VPS16	0.1249375	0.125407922	-0.00047046	0.033164787	0.158899081	0.210519568	17	0	0
CYFIP1	0.0544972	0.060027352	-0.00553011	-0.05438173	0.159002154	0.210640567	3	0	0
C11orf65	0.130841	0.135405907	-0.00456487	0.030397421	0.159072151	0.210703066	10	0	0
DCAF11	0.0449502	0.044830368	0.000119784	0.037746324	0.159073758	0.210703066	7	0	0
RAB11B	0.1414129	0.148505645	-0.00709277	-0.00688072	0.159084574	0.210703066	7	0	0
CAGE1	0.0305577	0.030181656	0.00037609	0.018637519	0.159143683	0.210750308	13	0	0
TSPAN15	0.1221218	0.122706934	-0.00058518	0.038175647	0.159143743	0.210750308	9	0	0
CXCR4	0.059053	0.043253241	0.015799731	0.405985855	0.159213625	0.210827286	17	0	0
SDHAF2	0.1905344	0.202913588	-0.01237921	-0.00286006	0.159291241	0.210914491	9	0	0
GPCPD1	0.4265772	0.434604437	-0.00802724	0.00323969	0.159353075	0.210980789	4	0	0
CHCHD3	0.190247	0.193794896	-0.00354787	-0.00641833	0.159412124	0.211043391	12	0	0
CLPX	0.0391278	0.036676316	0.00245153	0.057963577	0.159447468	0.211060721	12	0	0
CBR1	0.2456544	0.241395003	0.004259366	0.201294519	0.159448749	0.211060721	9	0	0
CCDC57	0.0744003	0.05529502	0.019105292	0.30667723	0.159639436	0.211297537	4	0	0
TBX6	0.0911008	0.094042684	-0.00294192	-0.00066229	0.15980582	0.211502153	11	0	0
ABCE1	0.0882	0.095582703	-0.00738269	-0.01794056	0.159861459	0.211514548	16	0	0
NEK1	0.0453029	0.043043529	0.002259415	0.065327627	0.159842544	0.211514548	11	0	0
CDK1	0.1425422	0.141329655	0.001212501	0.038543573	0.159854285	0.211514548	10	0	0
RAB11FIP2	0.1048433	0.118084167	-0.01324089	-0.06601266	0.159862356	0.211514548	8	0	0
OR4K14	0.2384467	0.258739081	-0.02029241	-0.11326992	0.159897544	0.211545501	1	0	0
AIFM2	0.219419	0.203644604	0.015774348	0.141483443	0.15992577	0.211564485	11	0	0
CMTR2	0.3104608	0.316421964	-0.00596113	0.002587828	0.159935485	0.211564485	10	0	0
COX20	0.0272441	0.026843837	0.000400308	0.008302668	0.159990889	0.211622167	9	0	0

HDHD3	0.0579725	0.055990155	0.001982328	0.042423847	0.160102998	0.211754839	3	0	0
CCDC30	0.2341766	0.218450093	0.015726515	0.06922539	0.160168238	0.211825506	5	0	0
CYB5D1	0.053226	0.054644092	-0.00141813	0.008907473	0.160200342	0.211852343	16	0	0
SCAMP5	0.1181892	0.124816362	-0.0066272	0.128396186	0.160331799	0.212010553	14	0	0
ASH2L	0.0397898	0.039147977	0.000641861	0.014612454	0.160348169	0.21201657	10	0	0
SERAC1	0.0489678	0.04628438	0.002683415	0.039692059	0.160402525	0.212035921	12	0	0
DDN	0.1176633	0.123901084	-0.00623776	-0.01581326	0.160410092	0.212035921	10	0	0
FAM192A	0.0506461	0.0489948	0.001651299	0.029755714	0.160399182	0.212035921	11	0	0
SLC26A11	0.2958402	0.304243027	-0.00840287	-0.05453692	0.160396697	0.212035921	4	0	0
TP53RK	0.1040762	0.11301964	-0.00894348	-0.01440596	0.160469939	0.212099397	9	0	0
BEND6	0.0702255	0.056554736	0.0136708	0.217466508	0.160662073	0.212337701	10	0	0
CYB5D2	0.1744246	0.179049964	-0.00462535	-0.00594765	0.160820476	0.212531392	11	0	0
CENPU	0.0839477	0.082519691	0.001427967	8.84E-05	0.160850422	0.212555306	3	0	0
HOOK3	0.2549831	0.243150231	0.011832851	0.028414627	0.160881995	0.212581366	5	0	0
PSMD7	0.0385745	0.03780568	0.000768868	0.026345005	0.16099249	0.212711698	11	0	0
INO80D	0.0989204	0.092445394	0.006475056	0.087102571	0.161115869	0.212844742	9	0	0
ASCC3	0.2239791	0.241053917	-0.0170748	-0.03834951	0.16111692	0.212844742	8	0	0
ZNF780B	0.0480733	0.052605073	-0.00453174	-0.06548945	0.161147799	0.212869858	14	0	0
JAG1	0.0493753	0.047991209	0.001384072	0.03559811	0.16116886	0.212882	13	0	0
RARS2	0.1427327	0.147615373	-0.00488269	-0.00209547	0.161492896	0.2132943	17	0	0
FAM89B	0.0847542	0.08839596	-0.00364171	-0.00519244	0.161649121	0.213484915	13	0	0
BRI3	0.1110291	0.102199422	0.008829721	0.034165612	0.161712026	0.213552267	7	0	0
VPS11	0.0329406	0.033277159	-0.0003366	0.009760674	0.161772502	0.213616403	9	0	0
MANBA	0.2455817	0.244211336	0.001370375	-0.01735202	0.1618041	0.213642398	9	0	0
NCAPH	0.1190889	0.131745072	-0.01265615	-0.01431852	0.161961716	0.213834769	9	0	0
COA1	0.1074252	0.108727211	-0.00130205	0.011937115	0.16213533	0.214016726	12	0	0
KLF14	0.1688222	0.149661071	0.019161156	0.175537857	0.162126761	0.214016726	13	0	0
RPS27L	0.0564629	0.05483452	0.001628373	0.030694159	0.162112133	0.214016726	7	0	0
TFAM	0.0692302	0.082864256	-0.01363403	-0.0166346	0.162178875	0.214058451	11	0	0
RPP38	0.0584374	0.0635904	-0.00515296	-0.09701336	0.162240542	0.214092582	2	0	0
LYRM1	0.2016267	0.194863436	0.006763222	0.079653108	0.162237154	0.214092582	8	0	0
FAM209A	0.8135093	0.818693368	-0.00518404	-0.00972388	0.162231303	0.214092582	3	0	0
PTHLH	0.5593128	0.545417915	0.013894884	0.046399008	0.162364449	0.214240327	3	0	0
FBRSL1	0.0752224	0.061556363	0.013666002	0.412936048	0.162697088	0.214663452	21	0	0
MED11	0.1529645	0.156227012	-0.00326248	-0.00185471	0.162715356	0.214671763	12	0	0
DDX54	0.1445576	0.148472281	-0.00391472	0.01822283	0.162820665	0.214794899	5	0	0
TACC3	0.0494288	0.051416654	-0.00198781	-0.05229882	0.16303687	0.215064301	7	0	0
HSH2D	0.9168271	0.923474788	-0.00664767	-0.01034841	0.163119448	0.215157407	4	0	0
LCA5	0.1636815	0.167986157	-0.00430469	0.02828162	0.163275269	0.215347101	11	0	0
ZNF442	0.0664617	0.0530093	0.013452443	0.293314277	0.163393154	0.215486736	4	0	0
SOX18	0.1463948	0.134179697	0.012215102	0.061563891	0.163456698	0.21555469	11	0	0
UPP1	0.1867189	0.194583256	-0.00786438	-0.03382467	0.163502262	0.215598925	13	0	0
HSPB1	0.0831717	0.073913115	0.009258575	0.165607782	0.163530769	0.215620662	8	0	0
BCL2L2-PAB	0.2445929	0.237770028	0.006822893	0.032057929	0.163544512	0.215622933	10	0	0
JMJD4	0.087149	0.096410115	-0.00926113	-0.03431314	0.163588186	0.21566466	8	0	0
PARP14	0.1086683	0.114975333	-0.00630706	-0.02212149	0.163655988	0.215722334	11	0	0
IFNAR1	0.0459305	0.043240559	0.002689916	0.069060737	0.163647031	0.215722334	6	0	0
NF2	0.1263227	0.135440442	-0.00911775	-0.02039382	0.163672853	0.215728711	11	0	0
SEC63	0.0436031	0.045565765	-0.00196262	-0.02744024	0.163719595	0.215774462	7	0	0
NCOA5	0.17202	0.179997025	-0.00797704	-0.00088477	0.163931541	0.216037922	12	0	0
MAP1LC3B	0.0478453	0.039807478	0.008037791	0.197309269	0.16398483	0.216092271	7	0	0
ZCCHC9	0.0922461	0.093123705	-0.00087764	-0.0141155	0.164003847	0.216101455	10	0	0
PYY	0.1955244	0.185323777	0.010200667	0.190130576	0.164057982	0.216141028	16	0	0
ALKBH6	0.1731956	0.186016496	-0.01282091	-0.02414598	0.164046853	0.216141028	12	0	0
FNDC3A	0.0308208	0.029526797	0.00129402	0.045280409	0.164123528	0.216211502	10	0	0
NUTM2F	0.5789074	0.598922085	-0.02001472	-0.04821693	0.164154772	0.21623678	1	0	0
DET1	0.1408056	0.146859589	-0.00605402	-0.01537412	0.164182679	0.216257658	8	0	0
USP34	0.0422057	0.042482236	-0.00027655	0.00485266	0.164264171	0.216349109	8	0	0
HDAC4	0.1394927	0.146397795	-0.00690511	0.005080426	0.164292607	0.216370673	9	0	0
DPPA5	0.798294	0.798517059	-0.00022311	0.000301948	0.164535032	0.216674033	5	0	0
ZNF410	0.0576868	0.056038628	0.001648165	0.027041521	0.164745788	0.216935647	8	0	0
RBM18	0.1642393	0.169971307	-0.00573199	0.026128033	0.164838689	0.217042044	7	0	0
SRFBP1	0.1313882	0.134219744	-0.00283153	0.002557899	0.164867455	0.217063985	10	0	0

ZFYVE16	0.1920597	0.186436018	0.00562371	0.003644757	0.164975578	0.217190397	9	0	0
CNTFR	0.131544	0.114068718	0.017475328	0.210742484	0.165025936	0.217240748	3	0	0
FKBP10	0.1014633	0.084478489	0.016984834	0.206944193	0.165160058	0.21740135	23	0	0
COPZ2	0.5407207	0.539195715	0.001524939	0.068400938	0.165301354	0.217558119	5	0	0
GPR160	0.3350238	0.345059954	-0.01003613	0.052861861	0.165376663	0.217638552	10	0	0
LGI1	0.5823891	0.575050528	0.00733859	0.031228208	0.165389947	0.217640065	10	0	0
BMP5	0.5952322	0.621850787	-0.02661859	-0.0507596	0.165537727	0.217818552	3	0	0
IMPAD1	0.1802974	0.170510313	0.009787123	0.056313334	0.165662528	0.217966777	9	0	0
PGAM1	0.0846441	0.084650428	-6.29E-06	0.028303624	0.16568721	0.217967272	10	0	0
ZNF699	0.0384937	0.028472458	0.01002129	0.375739272	0.165678658	0.217967272	5	0	0
SPINT2	0.032338	0.035089988	-0.00275194	-0.09085243	0.165939081	0.218282608	1	0	0
DBNDD2	0.1033006	0.097716796	0.005583801	0.124735634	0.165979707	0.218320037	17	0	0
GFRA2	0.225998	0.222471544	0.003526492	0.117876947	0.166005029	0.218337333	3	0	0
GP9	0.7266162	0.740153729	-0.01353748	-0.02394949	0.166091653	0.218435246	10	0	0
DNAJC22	0.0651629	0.061721986	0.003440884	0.101833055	0.166113838	0.218448406	8	0	0
RDH11	0.4901666	0.492881781	-0.00271513	-0.00797557	0.166163078	0.218497138	6	0	0
MTSS1	0.3600458	0.36546701	-0.00542121	0.070241028	0.166260427	0.218609121	12	0	0
RPS28	0.0723126	0.076445576	-0.00413293	-0.0331498	0.166343252	0.218701991	5	0	0
ANKRD34A	0.0710604	0.080427553	-0.00936715	-0.02050217	0.166494037	0.218884193	11	0	0
BAIAP2	0.0678893	0.054573946	0.013315386	0.091141363	0.166598078	0.21900492	9	0	0
GMIP	0.1623689	0.166808334	-0.00443945	0.001362551	0.166907493	0.219395588	12	0	0
MEIS2	0.0815641	0.07079495	0.010769175	0.130843078	0.167181822	0.219740083	15	0	0
PSME3	0.2379985	0.245692508	-0.00769402	0.014440815	0.167220355	0.219774624	8	0	0
MCM3AP	0.0310269	0.032192799	-0.00116588	-0.03735925	0.167250725	0.219798432	6	0	0
SRR	0.0649653	0.066463119	-0.00149782	-0.02003172	0.167300897	0.219848259	4	0	0
SETDB2	0.0352845	0.035192512	9.20E-05	0.017987856	0.167333972	0.219875614	13	0	0
PRSS36	0.6520524	0.6522344	-0.00018196	-0.00319055	0.167451195	0.220013525	5	0	0
TIMM17A	0.1535323	0.157759337	-0.00422704	0.005357903	0.167479795	0.220020181	11	0	0
EIF4A1	0.1739706	0.182206472	-0.00823585	-0.04278591	0.167480794	0.220020181	9	0	0
ECHDC2	0.2068582	0.209545728	-0.00268752	0.050396793	0.167522648	0.220059046	11	0	0
HIST3H2A	0.0996044	0.074521146	0.025083242	0.441780192	0.167746529	0.220337	13	0	0
UST	0.0991524	0.106537975	-0.00738561	-0.03414553	0.167761505	0.220340536	10	0	0
TMEM208	0.096153	0.097778245	-0.00162522	0.020495057	0.167910309	0.220519828	14	0	0
ANKFY1	0.1174965	0.120725821	-0.00322932	0.003545874	0.167996359	0.220616686	14	0	0
RNASE11	0.0370116	0.037358119	-0.00034653	-0.01298167	0.168052587	0.220674369	4	0	0
CUTA	0.1741276	0.183680465	-0.00955285	-0.02857425	0.168117633	0.220743621	37	0	0
MFHAS1	0.3426721	0.34188096	0.000791179	0.042634327	0.168156152	0.220750924	6	0	0
HGSNAT	0.119688	0.108283382	0.011404595	0.070763403	0.168159967	0.220750924	6	0	0
TROAP	0.0547844	0.059090646	-0.00430626	-0.0506463	0.168160118	0.220750924	12	0	0
AP4E1	0.0426847	0.046228253	-0.00354355	-0.04642468	0.168187994	0.22077136	8	0	0
MAN2B1	0.0536303	0.054437437	-0.00080715	0.004144222	0.168233095	0.220814402	18	0	0
AFF1	0.0644342	0.068211831	-0.00377768	0.016683521	0.168298772	0.220884441	8	0	0
AVL9	0.0390393	0.037086997	0.001952353	0.056815097	0.168377813	0.220898188	9	0	0
ATP7B	0.0488315	0.04216249	0.006669011	0.139973172	0.168354734	0.220898188	14	0	0
ALG11	0.0488315	0.04216249	0.006669011	0.139973172	0.168354734	0.220898188	14	0	0
BCKDK	0.1972073	0.162984511	0.034222826	0.634580673	0.16833021	0.220898188	4	0	0
MCOLN1	0.2193243	0.223960999	-0.0046367	0.005097317	0.168383142	0.220898188	11	0	0
PPP1R13L	0.0468647	0.046445637	0.000419029	0.014068279	0.168360753	0.220898188	15	0	0
SCUBE1	0.3297402	0.298445878	0.03129436	0.159282724	0.168425115	0.220937093	5	0	0
RAD52	0.1273249	0.111693502	0.015631364	0.084012044	0.168498906	0.221017726	11	0	0
SLC25A13	0.1734331	0.176228824	-0.00279577	0.060842395	0.168626369	0.221168742	14	0	0
KBTBD6	0.2032669	0.203338965	-7.21E-05	0.015340032	0.168657476	0.221193367	10	0	0
BBS7	0.1320905	0.13965665	-0.00756616	3.87E-05	0.168877193	0.221465331	11	0	0
CDKN2C	0.0714417	0.075323078	-0.00388139	-0.02489635	0.168903015	0.221483001	7	0	0
CREG1	0.1549726	0.134881327	0.020091259	0.130370822	0.169033768	0.221638253	9	0	0
KCTD16	0.0407862	0.040986401	-0.00020019	0.006056076	0.16909465	0.221701874	8	0	0
HECTD3	0.0342618	0.034487795	-0.000226	0.000602359	0.169135792	0.2217234	11	0	0
UROD	0.0342618	0.034487795	-0.000226	0.000602359	0.169135792	0.2217234	11	0	0
ANKS6	0.2254259	0.214597401	0.010828494	0.224270139	0.169244938	0.221850268	5	0	0
CCDC134	0.1470832	0.155611593	-0.00852843	-0.05765423	0.169357115	0.221981088	5	0	0
CLSPN	0.0338446	0.033510598	0.000334038	0.016862438	0.169427836	0.222057557	8	0	0
MICALL2	0.0973585	0.085670701	0.011687753	0.065252251	0.169528175	0.22217283	11	0	0
RBM8A	0.0651197	0.067097641	-0.00197792	-0.01470151	0.169619434	0.222276187	12	0	0

EXOSC7	0.3474592	0.356407407	-0.00894819	-0.01388063	0.169634684	0.222279931	8	0	0
GSTO2	0.0722388	0.066686023	0.005552741	0.241001658	0.169661311	0.222298581	10	0	0
DCTN3	0.1587978	0.166273668	-0.00747585	-0.02658803	0.169674394	0.222299484	10	0	0
FANCF	0.1153113	0.116105201	-0.0007939	-0.01831081	0.169717308	0.222339467	12	0	0
E2F2	0.0458969	0.04799877	-0.00210189	-0.04387179	0.169801626	0.222433681	8	0	0
FAM171A1	0.0765276	0.077090809	-0.00056324	0.045220091	0.169815773	0.222435968	7	0	0
TAF1C	0.1484971	0.15652852	-0.00803144	-0.02886929	0.169889924	0.222512759	7	0	0
ERBB2	0.0866792	0.090017205	-0.00333802	0.128492479	0.16989921	0.222512759	14	0	0
FIS1	0.0635321	0.063536105	-3.99E-06	-0.01059942	0.169926018	0.22253162	7	0	0
ZNF485	0.2298365	0.239962739	-0.01012621	-0.03907215	0.169980604	0.222581091	7	0	0
MEN1	0.3873204	0.394264684	-0.00694426	0.03363362	0.169988615	0.222581091	7	0	0
GOLGA2	0.0467091	0.046645481	6.36E-05	0.008233709	0.170011141	0.222594337	5	0	0
DNAJC17	0.100882	0.106692251	-0.00581028	-0.03841943	0.170060334	0.222642493	14	0	0
PPFIA3	0.2148106	0.220638087	-0.0058275	0.010495014	0.170115001	0.222697808	5	0	0
HSPBAP1	0.0977492	0.093220987	0.004528197	0.032750081	0.170245388	0.222852231	9	0	0
GALNT4	0.1138661	0.119195831	-0.00532972	-0.0339263	0.170276299	0.222860164	11	0	0
POC1B-GAL	0.1138661	0.119195831	-0.00532972	-0.0339263	0.170276299	0.222860164	11	0	0
CDKN2AIP	0.0419936	0.042723936	-0.00073036	-0.01232496	0.170301851	0.222861547	9	0	0
SH3GL1	0.0303177	0.02808261	0.002235052	0.0598606	0.170302206	0.222861547	6	0	0
FKBPL	0.1475372	0.162947587	-0.01541043	-0.04898078	0.170334766	0.222887894	12	0	0
ANKRD7	0.0935912	0.070440539	0.023150708	0.368492172	0.170407426	0.222966705	2	0	0
TUBGCP4	0.8873247	0.896367727	-0.00904306	-0.01520241	0.170542168	0.223126729	2	0	0
APBB2	0.1067036	0.104744716	0.001958853	0.036075786	0.170690287	0.223304229	3	0	0
VPS4A	0.1153637	0.117637168	-0.00227345	0.01209761	0.170746776	0.223361839	10	0	0
USP19	0.286876	0.290011559	-0.00313558	-0.02043579	0.170768694	0.223374219	13	0	0
FAM86C1	0.0896044	0.081965903	0.007638489	0.115110292	0.17079466	0.223391892	1	0	0
ESAM	0.0687945	0.071370045	-0.00257554	-0.00483081	0.170811614	0.223395368	8	0	0
CDADC1	0.1118664	0.113743886	-0.00187746	-0.00354345	0.170822228	0.223395368	15	0	0
DHX57	0.1166516	0.117624932	-0.00097337	-0.04232034	0.170885115	0.223461316	14	0	0
MOCS2	0.2398393	0.241845709	-0.00200645	-0.02432928	0.170968818	0.223554474	8	0	0
GRWD1	0.1350627	0.129233804	0.005828863	0.032960323	0.171009315	0.223591127	12	0	0
APAF1	0.0330588	0.033008037	5.08E-05	0.008702153	0.171115753	0.223697678	18	0	0
POFUT1	0.0559936	0.057600582	-0.001607	-0.01381101	0.171109852	0.223697678	4	0	0
CRTC3	0.4160939	0.423843503	-0.00774956	0.005465171	0.171150856	0.223727262	5	0	0
DENND4A	0.0346481	0.035282214	-0.00063414	0.006272821	0.171243829	0.22382274	5	0	0
HBM	0.4670401	0.488054599	-0.0210145	-0.06307308	0.171248855	0.22382274	3	0	0
GRPEL1	0.1665092	0.16667574	-0.0001665	-0.02680218	0.171472548	0.224098777	8	0	0
ZFYVE21	0.1152016	0.121830745	-0.00662911	-0.02556564	0.171521869	0.224146903	14	0	0
INPPL1	0.061787	0.058185497	0.003601547	0.046792364	0.171566663	0.224189106	4	0	0
MICA	0.0892854	0.075493522	0.01379188	0.235475798	0.171707533	0.224356837	12	0	0
KRT13	0.6374179	0.648543913	-0.01112602	-0.03545005	0.171791535	0.224450246	3	0	0
ZNF692	0.3999281	0.408870146	-0.00894201	-0.0259	0.171884462	0.2245553	9	0	0
CNNM3	0.0894783	0.095778663	-0.00630032	-0.02319074	0.171899941	0.224559166	9	0	0
APOL1	0.4678955	0.479384941	-0.0114894	-0.01121432	0.172117269	0.224826694	4	0	0
EMCN	0.890742	0.906010319	-0.01526828	-0.02424986	0.172131802	0.224829304	1	0	0
FAM221B	0.0679878	0.067139315	0.000848512	0.022358328	0.172314729	0.225035457	12	0	0
PLXNA2	0.0417289	0.041793929	-6.50E-05	-0.00302253	0.172353597	0.225069831	8	0	0
NPAT	0.0495935	0.048922556	0.000670972	0.032872743	0.172385715	0.225095383	20	0	0
OTUD3	0.2537848	0.254575795	-0.00079096	0.004804603	0.172403914	0.225102759	7	0	0
DST	0.041989	0.041255444	0.000733523	0.044407932	0.172431355	0.2251222	11	0	0
LSM3	0.0842299	0.084926771	-0.00069682	0.010569063	0.172551366	0.225262487	10	0	0
SUDS3	0.2412939	0.240824163	0.000469751	0.011489625	0.172579134	0.22528234	9	0	0
ABCC11	0.8649439	0.883502617	-0.01855873	-0.03029656	0.17263499	0.225333557	3	0	0
TNFRSF21	0.1720493	0.179408127	-0.00735883	-0.0160834	0.172692548	0.225381178	7	0	0
SYNC	0.3224546	0.325588279	-0.00313365	0.107813822	0.172773046	0.225469831	7	0	0
CA13	0.1600395	0.166560667	-0.00652114	0.006159101	0.172831032	0.225529093	14	0	0
ZBTB6	0.2428891	0.248757736	-0.00586864	-0.01681609	0.172907467	0.22561242	7	0	0
COIL	0.2982076	0.308026546	-0.00981899	-0.00724023	0.172924948	0.225618817	7	0	0
C19orf54	0.0471537	0.049201471	-0.00204776	-0.05235556	0.172955383	0.225642111	3	0	0
ZBTB10	0.1390931	0.137655646	0.001437461	0.057104769	0.172983847	0.225662833	10	0	0
PIK3C2B	0.0391367	0.037172404	0.001964253	0.058856584	0.173054942	0.225739159	1	0	0
ACADSB	0.045785	0.044165238	0.001619782	0.045091569	0.173119004	0.225806302	12	0	0
IPO11	0.0515221	0.050447445	0.001074672	0.032841781	0.173305851	0.226033547	9	0	0

SPTLC2	0.222994	0.224871417	-0.0018774	-0.0012287	0.173318431	0.226033547	9	0	0
BSDC1	0.0768501	0.074141427	0.002708681	0.059381561	0.173417347	0.226146106	9	0	0
C17orf51	0.4882899	0.465076058	0.023213838	0.068826818	0.173547749	0.226299704	1	0	0
ADD1	0.0593987	0.053991593	0.005407066	0.075462786	0.173573178	0.226305356	10	0	0
LASP1	0.0863908	0.097564945	-0.01117419	-0.04292367	0.173577318	0.226305356	8	0	0
GXYLT1	0.5423131	0.531972669	0.010340457	0.031014652	0.173671809	0.226412092	4	0	0
IFT80	0.038145	0.038086605	5.84E-05	-0.00852743	0.173712234	0.226448334	11	0	0
KATNAL2	0.0590684	0.061266119	-0.00219775	-0.03341878	0.173738572	0.226466209	3	0	0
HS2ST1	0.0487463	0.046953263	0.001792989	0.019318098	0.173769859	0.226490531	12	0	0
TLR9	0.1735361	0.182124259	-0.00858811	0.021459164	0.173856396	0.226570393	9	0	0
TWF2	0.1735361	0.182124259	-0.00858811	0.021459164	0.173856396	0.226570393	9	0	0
SCRN3	0.0707677	0.074245696	-0.00347798	-0.01257027	0.174001125	0.226742529	12	0	0
LPP	0.2433426	0.26246959	-0.019127	-0.0743062	0.17405545	0.226747425	9	0	0
PSD3	0.312221	0.276996444	0.035224555	0.167017657	0.174045684	0.226747425	1	0	0
RUSC2	0.1081839	0.086017892	0.02216604	0.263714785	0.174043214	0.226747425	3	0	0
ST13	0.1217034	0.128664573	-0.00696113	-0.02044783	0.174053557	0.226747425	13	0	0
DDX18	0.0975739	0.099665262	-0.0020914	0.006533937	0.174087237	0.226772364	13	0	0
BSPRY	0.3336734	0.341425802	-0.00775238	-0.06451791	0.174126174	0.226806613	5	0	0
ACER3	0.3256614	0.333088794	-0.00742743	-0.03611924	0.174270943	0.226970424	7	0	0
CLMN	0.1878472	0.190104674	-0.00225744	-0.04094002	0.174277246	0.226970424	8	0	0
NIP7	0.1656301	0.17896787	-0.01333773	-0.04090161	0.174343966	0.227040831	10	0	0
C15orf41	0.1058055	0.108233942	-0.00242849	0.095044623	0.174385256	0.227078114	11	0	0
GUF1	0.0706598	0.073345597	-0.00268584	-0.04798441	0.174674727	0.227438542	9	0	0
VAMP4	0.0657584	0.058486655	0.007271738	0.048931897	0.174803259	0.227589376	9	0	0
PRPF4	0.0566619	0.055294721	0.001367199	0.041270059	0.174914399	0.227717549	10	0	0
OPA1	0.1999424	0.200889937	-0.00094758	0.04021835	0.17495764	0.227740782	14	0	0
CST11	0.5296999	0.544712354	-0.01501246	-0.04528092	0.174953613	0.227740782	4	0	0
ACACB	0.6508394	0.670662916	-0.01982356	-0.04299438	0.175018483	0.227803448	9	0	0
EFR3A	0.1999003	0.21224735	-0.01234704	-0.0116747	0.175034564	0.227807846	6	0	0
GRASP	0.1591962	0.145467014	0.013729164	0.194398607	0.175104465	0.227882287	8	0	0
SEPSECS	0.0592629	0.060955711	-0.00169285	-0.02193966	0.175121076	0.227887369	11	0	0
GPATCH11	0.1358964	0.150999196	-0.01510278	-0.00279861	0.175182983	0.227951392	6	0	0
EGLN3	0.2224041	0.221196633	0.001207506	0.018740552	0.175420318	0.228227102	9	0	0
SPTSSA	0.2224041	0.221196633	0.001207506	0.018740552	0.175420318	0.228227102	9	0	0
LRRC6	0.1925733	0.188987932	0.003585396	0.069163717	0.175492063	0.228287325	10	0	0
SLC9A8	0.1288476	0.121505739	0.007341816	0.009066848	0.17548501	0.228287325	8	0	0
HNF4G	0.0878187	0.086457843	0.001360827	0.049237469	0.175542289	0.228336101	8	0	0
CAMK2N1	0.112232	0.108228942	0.004003024	0.06552092	0.175712537	0.228540976	7	0	0
ZNF335	0.0584166	0.059924515	-0.00150794	0.009655495	0.175753339	0.22857747	12	0	0
DCAF4	0.3558961	0.348450314	0.007445814	0.020137755	0.175776865	0.228591492	8	0	0
ZNF787	0.0589719	0.057281846	0.001690079	-0.00755049	0.175829793	0.228643745	6	0	0
POLN	0.2462781	0.243895641	0.002382468	0.138867328	0.17587134	0.228664615	8	0	0
HAUS3	0.2462781	0.243895641	0.002382468	0.138867328	0.17587134	0.228664615	8	0	0
CHUK	0.1632659	0.167527693	-0.00426174	0.00976088	0.176174059	0.2290416	8	0	0
ITPR3	0.2610197	0.23284587	0.028173796	0.067853807	0.176217808	0.229081873	2	0	0
TEC	0.0338351	0.028422613	0.005412536	0.202717412	0.176347166	0.229233423	6	0	0
NRAS	0.1183971	0.117062305	0.00133483	0.046756374	0.176507324	0.229411253	4	0	0
DUSP14	0.0370971	0.03711392	-1.68E-05	0.013248294	0.176509551	0.229411253	3	0	0
GALE	0.1584486	0.159373869	-0.00092528	0.019483109	0.176591628	0.22948612	13	0	0
PIGY	0.4542229	0.45252385	0.001699	-0.06134486	0.176592743	0.22948612	3	0	0
LENG8	0.0509513	0.049083305	0.001868009	0.05046057	0.176637198	0.229527261	10	0	0
PDE4DIP	0.4412568	0.424206159	0.017050678	0.098451393	0.176654109	0.229532606	2	0	0
SNRNP70	0.2283664	0.231187693	-0.00282125	-0.0061634	0.176682729	0.229553164	5	0	0
TMED9	0.0949472	0.101758231	-0.00681107	0.00012277	0.176815863	0.229695477	13	0	0
EN2	0.1387027	0.121226732	0.017475997	0.218061672	0.176817877	0.229695477	13	0	0
MLH1	0.0767531	0.066419414	0.0103337	0.284271252	0.176988816	0.229900884	36	0	0
DPF2	0.039675	0.038499675	0.00117535	0.038917912	0.177158221	0.230104269	5	0	0
KNTC1	0.0623088	0.057548489	0.004760355	0.093782957	0.177181237	0.230104286	13	0	0
CYP2A6	0.7471914	0.768250862	-0.02105947	-0.03957727	0.177183892	0.230104286	1	0	0
ANKHD1	0.0497921	0.05214906	-0.00235699	-0.04205295	0.177312937	0.230255201	11	0	0
SERPINB6	0.1366289	0.136988793	-0.00035988	0.051678688	0.177340891	0.230258161	10	0	0
C16orf87	0.1278471	0.130603988	-0.00275691	0.025073711	0.177330758	0.230258161	10	0	0
SLC2A4	0.0627717	0.057173652	0.00559809	0.174234574	0.177542867	0.230503718	16	0	0

STAB1	0.512088	0.491437943	0.020650023	0.059756768	0.177618409	0.230585103	9	0	0
C6orf89	0.8658842	0.87377371	-0.00788952	-0.01335662	0.177668049	0.230599473	5	0	0
LRRC57	0.0876675	0.091561332	-0.00389383	0.028889475	0.177663028	0.230599473	14	0	0
PLCG1	0.1032916	0.098819737	0.004471879	0.052569183	0.177645724	0.230599473	7	0	0
B3GALNT1	0.1884832	0.175191293	0.01329193	0.328454522	0.177704888	0.230630599	7	0	0
TFCP2L1	0.1662625	0.16866777	-0.00240531	0.025201088	0.177744712	0.230665593	9	0	0
TGOLN2	0.1062521	0.104623684	0.001628417	0.033931847	0.177810864	0.230734746	6	0	0
TRMT10C	0.1394886	0.146927291	-0.00743866	-0.02663538	0.178038168	0.23100895	10	0	0
COPS6	0.1030477	0.107683229	-0.00463554	0.002604219	0.178047933	0.23100895	12	0	0
STON1-GTF.	0.2809351	0.282236121	-0.00130106	0.054183151	0.178098985	0.231058474	10	0	0
C12orf65	0.0667901	0.062754297	0.004035803	0.071231223	0.17812445	0.231058546	14	0	0
TINF2	0.1041825	0.109411027	-0.00522853	-0.00509344	0.178124805	0.231058546	11	0	0
SH3BP1	0.396592	0.358991632	0.037600352	0.139072236	0.17815385	0.231079509	2	0	0
DXO	0.1096622	0.095971889	0.013690275	0.371709336	0.178220585	0.231138349	40	0	0
TPK1	0.2296011	0.230042225	-0.00044111	0.086128255	0.178224987	0.231138349	12	0	0
BRPF3	0.1057341	0.11044002	-0.00470592	-0.00780882	0.178350381	0.231284248	6	0	0
ADSS	0.2607822	0.268312039	-0.00752984	-0.00839043	0.178363705	0.231284804	11	0	0
RASAL2	0.1855179	0.182003694	0.003514228	0.056856319	0.1783895	0.23130153	9	0	0
GLOD4	0.1043196	0.105577447	-0.00125786	0.006241345	0.178449725	0.231362893	12	0	0
OCLM	0.8560823	0.856454477	-0.00037213	-0.00076017	0.178565292	0.231477139	2	0	0
CHPF	0.1670599	0.172297245	-0.0052373	-0.05230876	0.178559912	0.231477139	7	0	0
EIF3J	0.0833897	0.087345065	-0.00395539	-0.07092546	0.17857656	0.231477139	11	0	0
KPNA4	0.0625089	0.062263298	0.000245649	0.022034888	0.178743736	0.231677096	14	0	0
ELL	0.0259987	0.025455199	0.000543547	0.021037448	0.178759873	0.231681269	9	0	0
NUDC	0.0698529	0.072170453	-0.00231757	-0.04364575	0.178873414	0.231811673	9	0	0
SLC25A42	0.0837186	0.074445221	0.009273332	0.075806713	0.178892502	0.231819661	8	0	0
PSMB4	0.2426856	0.242179187	0.000506447	0.002459534	0.178989053	0.231928021	8	0	0
MGAT3	0.5291374	0.542828581	-0.01369123	-0.01493104	0.179016004	0.231946185	3	0	0
SP100	0.1877033	0.177234774	0.010468562	0.295361138	0.179096592	0.23203384	5	0	0
KLHL7	0.0399772	0.042289385	-0.00231217	-0.03569214	0.179131766	0.232062648	12	0	0
HIVEP3	0.0434624	0.036285782	0.007176649	0.281895222	0.179216579	0.232155753	6	0	0
NES	0.0629772	0.057363929	0.005613312	0.118498582	0.179368029	0.232305284	7	0	0
ZBTB20	0.0330713	0.034420657	-0.00134939	-0.02559444	0.179362117	0.232305284	8	0	0
RNASEK	0.0681135	0.064832363	0.003281127	0.028152702	0.179370867	0.232305284	5	0	0
CHEK1	0.5279843	0.548918728	-0.02093446	-0.03116721	0.179676966	0.232668116	3	0	0
CYB5A	0.0749604	0.057720716	0.017239639	0.426302752	0.179675772	0.232668116	13	0	0
STT3A	0.0626627	0.0661447	-0.00348203	-0.06752915	0.179725981	0.232714784	1	0	0
TBC1D9	0.0611069	0.060976597	0.000130305	-0.04914653	0.179772477	0.232728396	8	0	0
TIGD3	0.221933	0.212920995	0.009011963	0.101500616	0.179769396	0.232728396	10	0	0
EFNB2	0.0572327	0.050969151	0.006263501	0.113476272	0.17977542	0.232728396	6	0	0
MLN	0.6004923	0.624384338	-0.02389203	-0.05008631	0.179790158	0.232730679	5	0	0
RAPGEF4	0.4653409	0.469167682	-0.00382677	-0.02600673	0.179889293	0.232825596	5	0	0
MYO5C	0.2798349	0.283804903	-0.00396996	0.060727454	0.179889446	0.232825596	12	0	0
STK25	0.624871	0.630508257	-0.00563726	-0.00862163	0.179919099	0.232847173	9	0	0
WDR20	0.0355972	0.036824592	-0.0012274	-0.03163191	0.179989556	0.232921549	9	0	0
C17orf53	0.3015915	0.308774628	-0.00718316	-0.03217008	0.180017303	0.23294065	10	0	0
ITPR2	0.0977953	0.097273228	0.000522026	0.039181725	0.180054688	0.232972218	15	0	0
CCNG2	0.1081313	0.120817018	-0.01268569	-0.0160696	0.180233409	0.233186643	7	0	0
B3GAT3	0.170234	0.16991794	0.000316081	0.035352883	0.180281185	0.233231631	13	0	0
MAP3K5	0.0846935	0.094510776	-0.00981727	-0.07410687	0.180385966	0.233312159	4	0	0
NDUFB9	0.0806082	0.089213971	-0.00860575	-0.03502527	0.180395463	0.233312159	14	0	0
GPR176	0.2238315	0.227203439	-0.00337198	-0.03254383	0.180380341	0.233312159	5	0	0
USP7	0.0888861	0.091174216	-0.00228811	-0.00989438	0.180379476	0.233312159	5	0	0
PGPEP1L	0.6818076	0.717658304	-0.03585069	-0.07289025	0.180459787	0.233378523	1	0	0
METTL4	0.076231	0.077466314	-0.00123536	-0.03779427	0.180528344	0.233450351	11	0	0
DCLRE1C	0.2048193	0.210930609	-0.00611133	-0.03238287	0.180560756	0.233475432	9	0	0
FKBP15	0.1429186	0.153502284	-0.01058372	-0.02254082	0.180631089	0.233532704	8	0	0
HIP1R	0.1445259	0.149530386	-0.00500453	0.061136349	0.180624741	0.233532704	7	0	0
FUK	0.0296305	0.030046332	-0.00041582	0.006508114	0.180667357	0.233553771	9	0	0
RECQL5	0.1262724	0.127752204	-0.00147985	0.043008174	0.180686448	0.233553771	11	0	0
CINP	0.0798391	0.078860883	0.000978247	0.057903289	0.180726003	0.233588066	17	0	0
01-Sep	0.182756	0.179981163	0.00277484	0.049229299	0.180761161	0.233616672	8	0	0
NOD1	0.046976	0.044702474	0.002273544	0.060087148	0.180798486	0.233648076	7	0	0

DYRK1B	0.2336243	0.240805913	-0.0071816	0.024353625	0.18088885	0.233748013	11	0	0
EXT1	0.3279626	0.327823511	0.000139086	0.015516039	0.180954761	0.233816339	4	0	0
ZC3H10	0.0401708	0.040923288	-0.00075251	-0.01916987	0.181047804	0.233919709	13	0	0
FOSB	0.0777752	0.078217293	-0.00044206	-0.02385528	0.181075929	0.233922632	9	0	0
CCDC106	0.1554991	0.147400801	0.008098273	0.083830831	0.181076149	0.233922632	6	0	0
PIAS3	0.3186763	0.315260911	0.003415389	0.021340706	0.181135048	0.233965016	6	0	0
SLC5A6	0.0646229	0.070835898	-0.00621301	-0.03978905	0.181130273	0.233965016	6	0	0
IL1RAP	0.1484	0.150302904	-0.00190288	0.039417973	0.181159714	0.233980027	13	0	0
SPINT1	0.1310237	0.136800396	-0.0057767	-0.0470094	0.181216774	0.234036871	7	0	0
TADA1	0.2383456	0.235309061	0.003036559	0.022757469	0.181239768	0.234049714	8	0	0
ALG6	0.1166957	0.122778807	-0.00608314	-0.04959637	0.181523875	0.234332245	10	0	0
CFB	0.5918204	0.58026723	0.011553135	0.039623724	0.181509983	0.234332245	10	0	0
STOML2	0.0403937	0.044317285	-0.00392357	-0.07766106	0.181489585	0.234332245	7	0	0
TAPBPL	0.0737121	0.063169178	0.010542926	0.240662982	0.181479508	0.234332245	10	0	0
ORC6	0.0459603	0.04905734	-0.00309702	-0.0641054	0.181517688	0.234332245	11	0	0
AKAP13	0.0410837	0.040505339	0.000578325	0.000690317	0.181597405	0.234410295	9	0	0
HSD17B11	0.1408605	0.135942334	0.004918122	0.038965282	0.181638531	0.234446509	10	0	0
SERPINB1	0.2467402	0.246079029	0.000661179	0.019028275	0.181849233	0.234701579	8	0	0
FAHD2A	0.0259806	0.026868726	-0.00088813	-0.03113338	0.181954921	0.234821086	7	0	0
ZBTB21	0.0538318	0.055521869	-0.00169012	-0.03210129	0.18198243	0.234839691	8	0	0
GPT2	0.1490343	0.155969297	-0.00693504	-0.04772003	0.18206046	0.234906583	9	0	0
MAPK10	0.063017	0.062922001	9.50E-05	-4.40E-05	0.182084842	0.234921142	17	0	0
FIZ1	0.8709427	0.862697668	0.00824501	0.014975003	0.18218693	0.235035947	3	0	0
CATSPERD	0.0600636	0.066185896	-0.00612234	-0.03628238	0.182214361	0.235054428	11	0	0
SRM	0.0790186	0.071817436	0.007201192	0.062239367	0.182327779	0.235182752	6	0	0
RAD51AP1	0.1057958	0.112170403	-0.0063746	-0.04486286	0.182340062	0.235182752	14	0	0
SETD5	0.0343036	0.033632119	0.000671509	0.018003575	0.182387688	0.235227265	11	0	0
ATP13A3	0.6176591	0.66930472	-0.05164559	-0.1140776	0.182410227	0.235234677	1	0	0
HMMR	0.0353956	0.036644679	-0.0012491	-0.03474419	0.182419666	0.235234677	15	0	0
CABIN1	0.3259406	0.334630274	-0.00868967	-0.01784581	0.182449989	0.235256865	6	0	0
TCAIM	0.2850311	0.28672043	-0.00168937	0.008242999	0.182556243	0.235376816	7	0	0
SS18L1	0.0338984	0.03402116	-0.00012279	-0.00244417	0.182569261	0.235376816	4	0	0
DRAP1	0.2111794	0.211670118	-0.00049074	0.026364915	0.182599369	0.235398712	7	0	0
CAB39L	0.0352761	0.035253421	2.27E-05	0.014717019	0.182639102	0.235406085	14	0	0
LLGL1	0.140913	0.142541019	-0.00162803	0.010265703	0.182644462	0.235406085	7	0	0
AQP4	0.6368956	0.638192205	-0.00129662	0.000280427	0.182632791	0.235406085	5	0	0
TPD52L1	0.7706925	0.785302456	-0.01460992	-0.02674915	0.182705518	0.235467857	1	0	0
PGA5	0.4875705	0.475838791	0.011731723	0.049126428	0.182764514	0.235526967	2	0	0
ZNF467	0.2531387	0.242121641	0.01101704	0.038407459	0.182947723	0.235746129	12	0	0
SLC25A17	0.2877385	0.293589043	-0.00585054	0.016352527	0.183174891	0.236021899	12	0	0
PRKAG2	0.1179557	0.105517921	0.012437795	0.054430446	0.183299655	0.23614873	9	0	0
TMEM19	0.2083485	0.224190255	-0.01584175	-0.03064398	0.183294255	0.23614873	8	0	0
GTF3C3	0.3580597	0.364341374	-0.00628165	0.038416077	0.183338863	0.236182278	8	0	0
ARSK	0.0787349	0.086761626	-0.00802672	-0.03154105	0.18364328	0.236557445	12	0	0
SLC45A1	0.4096894	0.410110543	-0.00042114	0.053013695	0.183778575	0.236714723	9	0	0
CCNF	0.1037407	0.104238354	-0.00049762	0.032103196	0.183810358	0.23673866	10	0	0
ZFP36	0.1637891	0.167372322	-0.00358327	0.01746331	0.184105812	0.237102164	7	0	0
LRRC26	0.3426631	0.332121851	0.01054125	0.103710227	0.184278314	0.237307283	13	0	0
TRPV4	0.3859837	0.389670653	-0.00368693	0.017197046	0.18430943	0.237329122	6	0	0
GJB2	0.116579	0.100442462	0.016136496	0.214334316	0.184321736	0.237329122	14	0	0
DDX11	0.0387283	0.041595712	-0.00286742	-0.08249166	0.184345001	0.237342039	1	0	0
ABHD15	0.0482824	0.050553663	-0.00227125	-0.05013175	0.184362354	0.237347343	6	0	0
ITPRIPL2	0.1849755	0.163484508	0.021490951	0.140254101	0.184545941	0.23756664	6	0	0
UBE2Q1	0.1388822	0.143896133	-0.00501397	-0.00453296	0.184576106	0.237588419	6	0	0
ZC3H11A	0.3253038	0.326763187	-0.00145934	0.015613362	0.18464955	0.2376659	12	0	0
PCDHB13	0.4447593	0.448579628	-0.00382033	0.01243615	0.184703967	0.237718883	4	0	0
YPEL1	0.0297531	0.025854662	0.003898412	0.166921827	0.184731735	0.237737562	4	0	0
ZHX2	0.0412577	0.041403263	-0.00014555	0.013962777	0.184788613	0.237793698	14	0	0
NCOA1	0.1084617	0.097037349	0.011424319	0.289952517	0.184808216	0.237801862	7	0	0
TRIT1	0.3356145	0.352646701	-0.0170322	-0.03173049	0.184909943	0.237835104	11	0	0
EFNA5	0.0552332	0.055862532	-0.00062938	0.01142337	0.184862927	0.237835104	8	0	0
RIMKLB	0.4724808	0.456703913	0.01577687	0.096941599	0.184851098	0.237835104	4	0	0
TDG	0.0941015	0.093317128	0.000784362	0.054427631	0.184912574	0.237835104	7	0	0

MYO19	0.2513016	0.269441234	-0.01813964	-0.01879314	0.184913611	0.237835104	7	0	0
CYP4F2	0.6766941	0.701065279	-0.0243712	-0.04917115	0.184893531	0.237835104	2	0	0
C3orf80	0.077611	0.058451566	0.019159462	0.371600711	0.184940998	0.237853272	8	0	0
MBOAT2	0.141678	0.14012678	0.001551214	0.037582759	0.185007771	0.237922089	9	0	0
TXNL4B	0.1635342	0.17003226	-0.0064981	-0.02067545	0.185025787	0.237928199	15	0	0
ANKH	0.0777509	0.076055575	0.001695347	0.051805929	0.185087309	0.237973188	11	0	0
UGCG	0.1268512	0.129677139	-0.00282596	0.030254084	0.185083718	0.237973188	6	0	0
DYNC1H1	0.3950234	0.401385079	-0.00636166	-0.02974858	0.185158145	0.238030139	5	0	0
GPANK1	0.3039014	0.310967429	-0.00706599	-0.00569605	0.185185858	0.238048702	19	0	0
HIST1H2BH	0.0512819	0.051154513	0.00012739	0.011214524	0.185308428	0.238144084	8	0	0
SEMA3E	0.0942547	0.079011401	0.015243329	0.225405935	0.185291769	0.238144084	7	0	0
IDI1	0.0864694	0.097950049	-0.01148064	-0.04279744	0.185313168	0.238144084	8	0	0
ZNF821	0.0335783	0.033432997	0.000145283	0.00871773	0.185279777	0.238144084	11	0	0
DUSP11	0.229246	0.235311622	-0.00606559	-0.0052299	0.185346528	0.23816989	10	0	0
DENR	0.0308945	0.03030067	0.000593843	0.02222768	0.185789412	0.238721892	8	0	0
ANAPC15	0.1139399	0.117795733	-0.00385582	-0.03344447	0.185824643	0.238732956	13	0	0
GNAZ	0.2032026	0.213716608	-0.01051403	-0.02926663	0.185823044	0.238732956	5	0	0
CNRIP1	0.1053454	0.093279278	0.012066074	0.24090457	0.185943541	0.238868596	14	0	0
DEPDC1B	0.1366814	0.14318735	-0.00650597	-0.05200193	0.186072813	0.239017544	11	0	0
ARID3B	0.0508886	0.04740377	0.003484806	0.089000795	0.186172992	0.239129103	4	0	0
CENPL	0.0939622	0.102692476	-0.00873032	-0.00762583	0.186232444	0.239188336	15	0	0
RBAK	0.096223	0.092179007	0.004043959	-0.01577135	0.18628217	0.239218847	5	0	0
FAM57A	0.0285946	0.026269054	0.002325514	0.107766644	0.186282874	0.239218847	5	0	0
TMEM128	0.1365841	0.134624204	0.001959927	0.025393037	0.186298141	0.239221324	14	0	0
ECM2	0.8096712	0.797809122	0.011862103	0.021199249	0.186357983	0.239275596	3	0	0
TMC5	0.2886499	0.289389404	-0.00073946	-0.00467783	0.186367087	0.239275596	7	0	0
PHF23	0.1375809	0.149247025	-0.01166616	-0.03752543	0.186422548	0.23932967	10	0	0
ANAPC10	0.042246	0.044365955	-0.00212	-0.03159768	0.186443971	0.239340042	14	0	0
ICA1	0.3371718	0.339183067	-0.00201129	0.076477635	0.18648301	0.239355895	7	0	0
NKAIN2	0.0911956	0.080835126	0.010360523	0.217815293	0.186503901	0.239365579	10	0	0
DPP7	0.2962075	0.29397233	0.002235164	0.046919819	0.186550278	0.23940797	5	0	0
NCLN	0.1414382	0.147676868	-0.00623863	0.023252038	0.186589389	0.23944103	7	0	0
GTF2H5	0.0489211	0.046368692	0.002552407	0.038518785	0.186685821	0.239547638	13	0	0
ZFP91	0.0323773	0.033223817	-0.00084654	-0.01602075	0.186726124	0.239582213	6	0	0
NEMF	0.0521702	0.045534586	0.006635571	0.136526751	0.186754577	0.23960158	9	0	0
KLHDC3	0.0411244	0.042471483	-0.00134704	-0.01287065	0.186891055	0.239759528	13	0	0
KIF13A	0.0660565	0.061540009	0.004516452	0.032234161	0.187047251	0.239942747	11	0	0
C11orf68	0.0733864	0.071882926	0.001503456	0.02560676	0.187066157	0.239948833	7	0	0
TUBG1	0.0378628	0.038315776	-0.00045295	-0.00573682	0.187078751	0.239948833	12	0	0
GEM	0.2862882	0.283693854	0.002594323	0.017815056	0.187153374	0.24002738	9	0	0
SLC33A1	0.0661653	0.068867306	-0.00270204	-0.0027017	0.187175678	0.240038822	13	0	0
GEMIN2	0.1399513	0.1451144	-0.00516309	0.017816384	0.187237014	0.240100313	8	0	0
HIGD2A	0.0432426	0.043989258	-0.00074666	-0.01265771	0.18727465	0.240131407	9	0	0
ELOVL6	0.1452581	0.145280173	-2.21E-05	0.034146829	0.18750919	0.240414957	12	0	0
TBC1D22B	0.1488419	0.138052765	0.010789089	0.029625491	0.187615386	0.240533922	11	0	0
RAB14	0.8299013	0.820807311	0.009094036	0.01570597	0.187767433	0.240677141	1	0	0
ZSCAN2	0.0777246	0.086737575	-0.00901302	-0.01335162	0.18777317	0.240677141	12	0	0
B4GALT6	0.112837	0.118155582	-0.00531859	-0.03298032	0.187748509	0.240677141	3	0	0
ZBTB12	0.1281706	0.122658364	0.005512233	0.048790964	0.187818347	0.240708101	21	0	0
ATP8A1	0.1399484	0.135138081	0.004810317	-0.00102299	0.187913751	0.240813164	7	0	0
DHCR24	0.1295192	0.136535994	-0.00701678	0.060953012	0.187994408	0.240882105	13	0	0
ZNF24	0.7418388	0.783576739	-0.04173791	-0.07775212	0.187981584	0.240882105	2	0	0
KANSL1L	0.0490606	0.045747243	0.003313401	0.061216043	0.188132767	0.241042168	9	0	0
CYP2C19	0.6150478	0.599501272	0.015546515	0.034775938	0.188166157	0.241067728	3	0	0
TAF6	0.1500057	0.154191843	-0.00418615	-0.0036253	0.188200604	0.241094638	22	0	0
HNRNPLL	0.2925729	0.29719161	-0.00461867	-0.01982962	0.188297936	0.241202098	9	0	0
ZCWPW2	0.0532799	0.050837329	0.002442561	0.018643706	0.188314122	0.241205606	15	0	0
ZNF608	0.0963264	0.068433787	0.027892574	0.445419192	0.18839417	0.241290905	2	0	0
WDR46	0.2098988	0.210980661	-0.00108188	-0.00124077	0.188548946	0.241471896	29	0	0
PCCA	0.0788278	0.072433772	0.00639403	0.049184076	0.188565564	0.241475935	11	0	0
GALNT18	0.1155976	0.107665097	0.007932515	0.087517254	0.188665999	0.241575544	10	0	0
SYCE1L	0.9017281	0.90754112	-0.005813	-0.00957877	0.188670284	0.241575544	11	0	0
GFER	0.0623604	0.061019141	0.001341256	0.031334535	0.188687231	0.241579997	8	0	0

SPAG1	0.1415592	0.145196898	-0.00363767	0.148855335	0.188782794	0.241685096	15	0	0
BAG6	0.1052653	0.105042958	0.000222379	0.033126418	0.188806786	0.241698559	37	0	0
EPCAM	0.3028969	0.310532858	-0.00763598	0.000889069	0.188863949	0.241737229	4	0	0
CCSER1	0.1223269	0.123233577	-0.0009067	0.100108338	0.188856344	0.241737229	13	0	0
H6PD	0.2090644	0.213266271	-0.00420188	0.075865223	0.188890022	0.241753349	4	0	0
PCED1B	0.2061286	0.194802186	0.011326393	0.190355651	0.18896797	0.241835855	16	0	0
SLC25A28	0.0896547	0.095880914	-0.00622617	-0.03619644	0.189027589	0.241877637	8	0	0
TXNL4A	0.0687328	0.067773055	0.000959713	0.050615523	0.189018162	0.241877637	9	0	0
SPARCL1	0.4934733	0.467532179	0.025941122	0.090890455	0.189096964	0.241949148	4	0	0
EXOC5	0.0539125	0.053105453	0.000807063	-0.00465897	0.189274233	0.242158687	12	0	0
APOPT1	0.0552989	0.054954656	0.000344265	0.029362437	0.189552561	0.242497484	8	0	0
TAF1A	0.1649869	0.167060039	-0.00207312	-0.00366085	0.189569146	0.242501405	14	0	0
TTC27	0.0712548	0.076252328	-0.00499754	-0.02338022	0.189603701	0.242528311	11	0	0
AGRP	0.8429642	0.844853982	-0.00188977	-0.00403121	0.189638039	0.242554936	8	0	0
HIBCH	0.0414961	0.040347885	0.001148231	0.025594639	0.189715008	0.24263608	5	0	0
PNRC1	0.0691126	0.062518267	0.006594347	0.09909553	0.189828515	0.242763939	9	0	0
CCDC144NL	0.7497911	0.728884637	0.020906475	0.039575608	0.189998899	0.242964512	4	0	0
FLOT2	0.1370072	0.143672823	-0.0066656	-0.01208424	0.190214606	0.243223011	10	0	0
NDUFAF6	0.0328378	0.032137237	0.000700517	0.008745628	0.190283057	0.243293193	8	0	0
CELF5	0.4767126	0.503185642	-0.02647302	-0.046771	0.190504791	0.243559336	4	0	0
TBC1D4	0.0528897	0.050907825	0.001981915	0.04324143	0.190585442	0.243645082	7	0	0
MME	0.6943549	0.724890102	-0.03053522	-0.06102054	0.190772219	0.243866476	2	0	0
MAD1L1	0.1499865	0.151997395	-0.00201086	0.007071265	0.190933323	0.244055023	7	0	0
MSRA	0.2165697	0.222945884	-0.00637619	-0.01049138	0.190967836	0.244081743	11	0	0
SOX9	0.1700924	0.152045125	0.018047283	0.207857759	0.19107562	0.244202104	12	0	0
RPL10A	0.137872	0.136285969	0.001585994	0.057067856	0.191288865	0.24445722	10	0	0
ANKHD1-EIF	0.0483101	0.050541874	-0.00223177	-0.04096927	0.19136338	0.244535022	12	0	0
SUGCT	0.0688561	0.065096778	0.003759331	0.059247866	0.191445175	0.244622117	14	0	0
CEBPB	0.2162364	0.222629158	-0.00639276	-0.00931744	0.191530266	0.24471341	13	0	0
DAB1	0.0956714	0.086042813	0.009628615	0.091310424	0.191592312	0.244757814	9	0	0
OMA1	0.0956714	0.086042813	0.009628615	0.091310424	0.191592312	0.244757814	9	0	0
C3orf58	0.0456	0.045822626	-0.00022267	-0.004562	0.191648303	0.24481155	9	0	0
LIAS	0.0758169	0.076042128	-0.00022519	0.0128711	0.191661674	0.24481155	12	0	0
IFIT5	0.2339385	0.237325761	-0.00338731	0.000978472	0.191761108	0.244921116	12	0	0
CTCF	0.026309	0.027332404	-0.00102336	-0.01754339	0.191791126	0.244942013	5	0	0
TYK2	0.1075528	0.10785133	-0.00029852	0.010915606	0.192044215	0.245230317	10	0	0
CDS2	0.0439987	0.047778402	-0.00377966	-0.0375006	0.192034531	0.245230317	10	0	0
GBA	0.2057022	0.204907608	0.000794594	0.042563754	0.19209595	0.245278917	9	0	0
TBPL1	0.3164884	0.30933379	0.007154655	0.019410586	0.192167074	0.245352267	9	0	0
COG6	0.1996719	0.201950217	-0.00227829	0.013171481	0.192386513	0.245586389	5	0	0
SMPD3	0.1880027	0.186244751	0.001757987	0.016239005	0.192375081	0.245586389	14	0	0
ZNHIT3	0.0474446	0.04921187	-0.00176725	-0.03386623	0.192391522	0.245586389	13	0	0
TRMT1L	0.0785167	0.074060538	0.004456178	0.021419231	0.192429758	0.245617715	18	0	0
CYSTM1	0.2149037	0.215598374	-0.00069463	0.080059952	0.192465449	0.24564579	14	0	0
SCD	0.1519591	0.151466347	0.000492785	0.040680556	0.192518133	0.245695548	9	0	0
RASIP1	0.4266248	0.406647131	0.019977704	0.069895625	0.192706479	0.245918422	10	0	0
ZNF792	0.0764852	0.071880589	0.004604575	0.035921379	0.192774259	0.245987415	10	0	0
ACADS	0.2613767	0.258387308	0.002989425	0.056843428	0.192789103	0.245988856	10	0	0
ZNF14	0.0674326	0.060902098	0.006530521	0.222667875	0.192844945	0.246025104	14	0	0
PATL2	0.0376626	0.03618377	0.001478847	0.037840484	0.192992066	0.246195284	13	0	0
FREM2	0.0721555	0.065628923	0.006526545	0.117765305	0.193224606	0.2464744	9	0	0
CCND3	0.123511	0.115525748	0.007985263	0.064133865	0.193397054	0.246676828	6	0	0
BTG3	0.1540146	0.154163315	-0.00014876	-0.00974036	0.193425394	0.246695433	10	0	0
RNASE4	0.0408242	0.041822169	-0.000998	-0.02401231	0.193508831	0.246784299	10	0	0
GARNL3	0.3387018	0.337900968	0.000800833	-0.0280151	0.193545727	0.246813805	4	0	0
UROS	0.1121214	0.122012157	-0.00989078	-0.01255649	0.193593514	0.246857193	17	0	0
SFTA2	0.6210682	0.618375386	0.002692782	0.002390839	0.193621737	0.246875629	5	0	0
WEE1	0.0377044	0.033639147	0.004065284	0.134240198	0.193787382	0.247051708	10	0	0
SERPINB10	0.8807914	0.895011672	-0.01422027	-0.02284877	0.193775236	0.247051708	1	0	0
STK35	0.1881193	0.191175426	-0.00305612	0.026569102	0.193901298	0.247179366	12	0	0
PJA2	0.0344249	0.034763878	-0.000339	-0.0055782	0.193972737	0.24723529	9	0	0
ZFP69	0.110776	0.110419765	0.000356276	0.036791735	0.194013476	0.247252075	12	0	0
CDK6	0.0651894	0.055713963	0.009475486	0.189681356	0.194007884	0.247252075	14	0	0

ALDH5A1	0.3105876	0.309046751	0.001540894	0.085469817	0.194073114	0.247310506	8	0	0
WDR5B	0.0336013	0.03365032	-4.90E-05	-0.00858079	0.19420006	0.247454694	11	0	0
MAPKAPK5	0.0256758	0.024902049	0.000773795	0.031066993	0.194333035	0.247606543	12	0	0
GALNT10	0.102738	0.092664248	0.010073746	0.129417632	0.194372354	0.24763905	7	0	0
ADPGK	0.6218461	0.634032952	-0.0121868	-0.03623392	0.194488348	0.247769231	3	0	0
HELZ2	0.220841	0.237234533	-0.01639358	0.005066739	0.194587852	0.247878388	8	0	0
ILVBL	0.1326497	0.12992027	0.002729415	0.057049462	0.194632638	0.247917832	11	0	0
SPTSSB	0.1982817	0.191219612	0.007062103	0.175018457	0.194708846	0.247997292	5	0	0
ZFAND6	0.0376488	0.035017723	0.002631063	0.079104032	0.194880417	0.248198193	8	0	0
C6orf52	0.0474149	0.045294789	0.002120077	0.060692571	0.194902398	0.248208563	7	0	0
MUT	0.0658865	0.076716432	-0.01082994	-0.04020947	0.194931842	0.248228436	15	0	0
COX18	0.0694097	0.079969817	-0.01056007	-0.05093853	0.19505143	0.248345457	9	0	0
NANP	0.1427347	0.145201009	-0.00246634	0.013715957	0.195042986	0.248345457	16	0	0
ZBTB7C	0.2058201	0.214459455	-0.00863931	-0.05662531	0.195104599	0.24839552	1	0	0
ANKZF1	0.0396063	0.03986338	-0.00025708	0.009549791	0.195120783	0.248398492	18	0	0
EXOC8	0.0531101	0.054150226	-0.00104013	-0.00010799	0.195147263	0.248414571	18	0	0
PCDH9	0.0491902	0.045401381	0.003788809	0.094425115	0.195201073	0.248465435	12	0	0
FHOD1	0.057544	0.058395268	-0.00085123	0.009041981	0.195312102	0.248589119	17	0	0
ARID4B	0.0469396	0.045838942	0.001100708	0.034963518	0.195470417	0.248737666	13	0	0
LSM5	0.0390632	0.037429972	0.001633212	0.047550872	0.195466486	0.248737666	10	0	0
ATXN7L2	0.0634014	0.06987376	-0.00647233	0.023986885	0.195621092	0.248911742	7	0	0
RBM28	0.0837532	0.089130123	-0.00537693	-0.0184622	0.195927519	0.24928396	8	0	0
TTYH2	0.1383897	0.134739438	0.003650259	0.127535042	0.195964802	0.24931371	13	0	0
GHRH	0.5904333	0.621716456	-0.03128313	-0.0601021	0.196009573	0.249352982	4	0	0
PRRT2	0.1225002	0.125147309	-0.00264713	-0.00366991	0.196052038	0.24938276	11	0	0
CYS1	0.107344	0.097828546	0.009515472	0.2415227	0.196206231	0.249533709	7	0	0
KIAA0825	0.0589121	0.057821401	0.001090746	0.026649801	0.196207287	0.249533709	21	0	0
KRTCAP2	0.1037559	0.085571089	0.018184813	0.117985427	0.196318332	0.249657232	17	0	0
RPP25L	0.0452817	0.044138407	0.001143268	0.022541576	0.196433583	0.249786086	6	0	0
SNAPC3	0.2405407	0.242585037	-0.00204434	-0.01073559	0.196604376	0.249967824	11	0	0
MYO9B	0.0277658	0.026727973	0.001037838	0.03943574	0.196593781	0.249967824	6	0	0
KCNN1	0.0737427	0.074783409	-0.00104075	0.119008804	0.196703413	0.250076015	7	0	0
OSBPL3	0.1074285	0.11039352	-0.00296503	-0.03258674	0.196751985	0.250120037	5	0	0
STRAP	0.1473173	0.156728958	-0.00941165	-0.01610899	0.196888328	0.250275623	8	0	0
MT1F	0.1497123	0.146978364	0.002733964	0.092689292	0.197120344	0.250552794	9	0	0
U2AF2	0.3073215	0.306671325	0.00065017	0.009673381	0.197206325	0.250644321	9	0	0
DCUN1D2	0.0975005	0.093734012	0.003766506	0.051873134	0.197232771	0.25066017	6	0	0
TMEM125	0.76996	0.757761913	0.012198042	0.023380585	0.197292908	0.250717715	3	0	0
C3orf14	0.2842776	0.279049288	0.005228312	0.174278976	0.197306007	0.250717715	14	0	0
MGAT4A	0.0379293	0.034295847	0.003633465	0.087447939	0.197508261	0.250921389	4	0	0
SEC22A	0.2267115	0.225854238	0.000857222	-0.01832622	0.197506265	0.250921389	9	0	0
CCL27	0.3653351	0.363760033	0.001575114	0.018154983	0.197487453	0.250921389	8	0	0
PEX3	0.1360017	0.14910819	-0.01310648	-0.03960846	0.197615978	0.251040455	18	0	0
ZNF823	0.2014526	0.21209653	-0.01064397	-0.01379337	0.197754603	0.251198765	8	0	0
ZNF652	0.0875194	0.089897962	-0.00237856	-0.02291149	0.197893016	0.251356784	7	0	0
CHDH	0.2091604	0.196932842	0.012227509	0.239508415	0.197940246	0.25139897	8	0	0
ARHGEF2	0.6943267	0.717588917	-0.02326219	-0.05655638	0.197996938	0.251435364	4	0	0
ANKRD17	0.2109624	0.218363008	-0.00740058	-0.03761179	0.197993599	0.251435364	7	0	0
HAUS5	0.3132315	0.32873186	-0.01550034	-0.02160368	0.198106053	0.251556118	8	0	0
DNAJC24	0.0919844	0.085332335	0.006652106	0.04796857	0.198373565	0.251877974	13	0	0
BRAP	0.1264581	0.13300612	-0.00654797	-0.00411157	0.198406626	0.251902119	15	0	0
CCAR2	0.3892694	0.386205983	0.003063372	0.020361412	0.198435652	0.251921139	10	0	0
UPP2	0.0612973	0.060082051	0.001215224	0.031372925	0.198501363	0.251968892	8	0	0
ACSS2	0.6498311	0.643648341	0.006182788	0.017728352	0.198494087	0.251968892	2	0	0
PLAA	0.1155635	0.116823483	-0.00126002	0.01605662	0.198583896	0.252055817	10	0	0
CSNK1G1	0.1401681	0.149493093	-0.00932497	-0.01852137	0.198660557	0.252135278	11	0	0
GOT1	0.059766	0.062986831	-0.00322083	-0.05161542	0.198857354	0.25236719	11	0	0
UPF1	0.5597185	0.556289788	0.003428691	-0.0215123	0.199182436	0.252761862	3	0	0
HERC4	0.2089831	0.210006215	-0.00102316	0.011906292	0.199248309	0.252827568	10	0	0
TRUB2	0.0423411	0.042708741	-0.00036762	-0.01507288	0.199315304	0.252894687	17	0	0
MASTL	0.0360657	0.034651158	0.001414513	0.032681482	0.199682837	0.253343097	11	0	0
ARNTL	0.0940504	0.095480637	-0.00143022	0.050946203	0.199921343	0.253609817	6	0	0
CLN6	0.220236	0.220623018	-0.00038706	0.132879524	0.199909591	0.253609817	3	0	0

TONSL	0.1774966	0.186159739	-0.00866317	0.012521236	0.199950895	0.253629367	6	0	0
ZBTB25	0.062736	0.057867886	0.004868094	0.069569663	0.200123226	0.253830011	8	0	0
CDCA8	0.1132949	0.122230912	-0.00893601	-0.00280765	0.200212163	0.25389626	6	0	0
ARHGEF4	0.273493	0.272570045	0.00092294	0.047565728	0.200217925	0.25389626	7	0	0
TOX	0.1209011	0.105053298	0.015847843	0.262730818	0.200193555	0.25389626	17	0	0
PEX7	0.0434881	0.043912171	-0.00042403	-0.01646716	0.200324153	0.253977099	7	0	0
FGFR2	0.2500296	0.245126558	0.004903023	0.087399089	0.200311427	0.253977099	5	0	0
SLC9A5	0.762333	0.778728945	-0.01639593	-0.03293616	0.200323313	0.253977099	6	0	0
FZD7	0.037521	0.038677088	-0.00115608	-0.02419841	0.200463107	0.254135304	9	0	0
UBAP1	0.0661899	0.068984719	-0.00279478	-0.01365415	0.2005261	0.254197196	6	0	0
GOLPH3L	0.0873178	0.089370127	-0.00205233	0.039695768	0.200683219	0.25437839	11	0	0
BRD1	0.4396797	0.431559887	0.008119802	0.019684344	0.200714423	0.254399964	2	0	0
TNNT1	0.2629254	0.262012368	0.000913036	0.028315741	0.200826372	0.254523869	8	0	0
AAMDC	0.0428644	0.04065631	0.002208055	0.049963883	0.200860143	0.254548684	11	0	0
MORN1	0.2769725	0.295193854	-0.01822139	-0.05260139	0.200970183	0.254642525	10	0	0
UGT8	0.3404404	0.356759259	-0.01631882	0.012997127	0.200976783	0.254642525	8	0	0
ADAL	0.1211334	0.126143638	-0.00501019	0.015132162	0.200975424	0.254642525	15	0	0
ALPK1	0.0284533	0.030471907	-0.00201863	-0.05567018	0.201071843	0.254736034	4	0	0
HECTD2	0.0916634	0.098209521	-0.00654607	-0.01883109	0.20107899	0.254736034	10	0	0
RAB44	0.6491925	0.633654874	0.015537593	0.034412429	0.201225222	0.254892967	1	0	0
MRPS2	0.224444	0.230918228	-0.00647418	-0.01011007	0.20123129	0.254892967	7	0	0
ZNF566	0.0464002	0.034048698	0.01235155	0.36511751	0.20128468	0.25494259	17	0	0
ING2	0.0429462	0.041549523	0.001396723	0.043313554	0.201313127	0.254960617	6	0	0
POLR2K	0.0656679	0.068316122	-0.0026482	-0.02289385	0.201468532	0.255139419	10	0	0
C12orf49	0.0746901	0.074932415	-0.00024234	-0.00642346	0.201561533	0.255239174	9	0	0
PHF20	0.2654627	0.264154351	0.00130833	0.057684718	0.201589826	0.25525698	11	0	0
ATP6V0A2	0.4486485	0.452348121	-0.00369961	0.007733264	0.201615838	0.255271896	5	0	0
QRICH2	0.8805328	0.882877743	-0.00234494	-0.00467485	0.201689416	0.25534703	6	0	0
ZNF223	0.1108765	0.105101572	0.005774914	0.072380387	0.201720176	0.255367947	4	0	0
LRRCC1	0.0410751	0.045195704	-0.0041206	-0.0623357	0.201735032	0.255368729	14	0	0
RPP40	0.255157	0.257704654	-0.00254766	-0.02180583	0.201749803	0.255369404	8	0	0
HIST1H3F	0.048336	0.048260904	7.51E-05	0.008515627	0.201819817	0.25544	10	0	0
ARL15	0.0509292	0.0527303	-0.00180108	-0.03004388	0.20193251	0.255564599	13	0	0
SFXN3	0.2628008	0.261363819	0.00143697	-0.0217635	0.202004959	0.255638252	12	0	0
ISOC2	0.3254051	0.344113066	-0.01870801	-0.04582726	0.202028463	0.255649958	5	0	0
RING1	0.0766615	0.069664996	0.006996497	0.055943874	0.202210672	0.255862477	26	0	0
MMP11	0.0997648	0.088417343	0.01134741	0.386794692	0.202286561	0.255940446	11	0	0
WDR54	0.1781132	0.190459457	-0.01234626	-0.02389848	0.202460981	0.25614306	10	0	0
KDM1A	0.0440901	0.043962382	0.000127751	0.007261578	0.202586752	0.256284101	9	0	0
ENTHD1	0.6929478	0.719199955	-0.02625216	-0.05638274	0.202762434	0.256488258	6	0	0
RCAN1	0.0360364	0.033094802	0.002941567	0.060452283	0.202793253	0.256509153	7	0	0
FAM161B	0.0621248	0.068957509	-0.00683266	-0.04065667	0.202962193	0.256704738	7	0	0
NUAK2	0.3338654	0.328137357	0.005728029	0.019333197	0.203023091	0.256763656	9	0	0
WDR6	0.060457	0.062512267	-0.00205531	-0.02895194	0.203051209	0.25678111	9	0	0
GKN2	0.7107965	0.711672527	-0.00087606	-0.0006531	0.203128728	0.256861032	4	0	0
GANC	0.04832	0.048311528	8.43E-06	0.010019988	0.203247706	0.256993364	12	0	0
SPR	0.1162701	0.111269752	0.005000323	0.110235085	0.203370547	0.25713056	11	0	0
ANKRD52	0.0891597	0.079614855	0.009544797	0.082604747	0.203421408	0.257176737	11	0	0
SUCO	0.1729717	0.171781516	0.0011902	0.008757605	0.203436342	0.25717749	8	0	0
CCDC24	0.0986059	0.109841058	-0.0112352	-0.04407176	0.203530519	0.257278413	14	0	0
SMC5	0.0350044	0.03195913	0.003045226	0.090387575	0.203570526	0.257310849	7	0	0
SEZ6L2	0.0525806	0.05098356	0.001597059	0.035765566	0.203747848	0.257516835	8	0	0
BMPR2	0.0438315	0.043136533	0.000694958	0.012740962	0.203852411	0.257612686	12	0	0
METAP2	0.1096014	0.106200141	0.003401278	0.02128044	0.203845114	0.257612686	5	0	0
FBXO31	0.0453417	0.042588666	0.002752998	0.103229927	0.204122366	0.25793566	9	0	0
USP12	0.0221766	0.021549322	0.000627241	0.026072165	0.204255158	0.258085277	4	0	0
BMP3	0.1616963	0.150074701	0.011621591	0.282308588	0.204304657	0.258129637	10	0	0
KLF3	0.1137598	0.115000365	-0.00124055	0.015922867	0.204344873	0.258134817	11	0	0
METAP1	0.086305	0.092332928	-0.00602791	-0.02763109	0.20433499	0.258134817	7	0	0
RAD9A	0.1484763	0.155571861	-0.00709552	-0.03924787	0.204351933	0.258134817	7	0	0
MEIS1	0.1605366	0.148194202	0.012342443	0.166415057	0.204399963	0.258177305	9	0	0
SPAG17	0.2826912	0.276057067	0.006634133	0.176192897	0.204505173	0.258255635	12	0	0
IFNGR1	0.2593956	0.279206278	-0.01981064	-0.04930068	0.204479861	0.258255635	6	0	0

JMJD1C	0.1631062	0.150779823	0.012326404	0.080708944	0.20449569	0.258255635	11	0	0
PCDHB11	0.4213761	0.4196166	0.001759452	0.099224342	0.204550842	0.258295121	8	0	0
GPR153	0.2084324	0.203580738	0.004851643	0.084027983	0.204594164	0.258331638	6	0	0
TRMT61B	0.0597591	0.065294453	-0.00553537	-0.0489191	0.204680509	0.258414437	13	0	0
TSTD2	0.037115	0.038304434	-0.00118939	-0.02723227	0.204688554	0.258414437	16	0	0
VWA5B2	0.062905	0.058857564	0.004047427	0.182512197	0.204717766	0.258415835	12	0	0
ZW10	0.0942794	0.096051254	-0.00177182	0.023907068	0.204718477	0.258415835	13	0	0
KPNA3	0.0447231	0.046413274	-0.00169013	-0.04043173	0.204740336	0.258418588	12	0	0
COA3	0.2987095	0.309965168	-0.01125564	-0.02757217	0.204749474	0.258418588	11	0	0
PCMTD1	0.0406604	0.042163154	-0.00150279	-0.00112939	0.204811062	0.258478131	9	0	0
ACTR3	0.1108803	0.123757059	-0.01287673	-0.01861759	0.204871928	0.258536755	7	0	0
ELOVL7	0.3653511	0.365052004	0.000299073	0.000299154	0.204904013	0.258552427	8	0	0
HAUS2	0.1009502	0.100615932	0.000334313	0.034197028	0.204913177	0.258552427	13	0	0
CNOT7	0.067134	0.06439783	0.00273614	0.021393922	0.205034539	0.258687359	13	0	0
DGCR6	0.075266	0.075508577	-0.00024255	0.002863887	0.205057877	0.258698606	9	0	0
SLC52A1	0.6963121	0.687962175	0.008349952	0.030769212	0.205152457	0.258799723	2	0	0
PARK7	0.0796498	0.070329485	0.009320333	0.295317481	0.20552337	0.259249397	6	0	0
AJUBA	0.1241036	0.123091137	0.00101248	0.043092157	0.205618891	0.259351647	11	0	0
SLC50A1	0.1573494	0.15478911	0.002560243	0.055276085	0.206017622	0.259818033	14	0	0
CCDC121	0.1428977	0.148028523	-0.00513085	-0.04120608	0.206012912	0.259818033	15	0	0
ARMC8	0.1051628	0.115424939	-0.01026218	-0.03678647	0.206078843	0.259876969	16	0	0
RIMS2	0.1386525	0.116997158	0.021655313	0.374343639	0.206177793	0.259983471	7	0	0
ZSWIM1	0.0463571	0.044405847	0.001951217	0.050821183	0.2061974	0.259989917	6	0	0
MTBP	0.0772008	0.080442808	-0.00324204	-0.02019839	0.206377237	0.260198378	13	0	0
DHX37	0.2172141	0.210336539	0.006877558	0.279216417	0.206445211	0.260265784	8	0	0
BAIAP2L1	0.1907194	0.18375926	0.00696011	0.08660377	0.20650042	0.260317088	8	0	0
FZD4	0.0321055	0.029724016	0.002381503	0.080502368	0.206561809	0.260376176	9	0	0
REV3L	0.1252886	0.130581541	-0.00529293	0.008868161	0.206580052	0.260380872	13	0	0
OAF	0.1138411	0.105009866	0.008831189	0.092199528	0.206642019	0.260440676	11	0	0
COQ2	0.1494104	0.151949427	-0.00253906	-0.03154287	0.20669162	0.260479656	11	0	0
CLDN3	0.1096701	0.103556697	0.006113389	0.22390892	0.206701993	0.260479656	12	0	0
ZNF221	0.1259308	0.126961694	-0.00103089	0.081672053	0.206829957	0.260622602	9	0	0
SOAT1	0.0701443	0.059862893	0.010281426	0.156967052	0.206890505	0.260680583	15	0	0
KCTD3	0.2538845	0.271488853	-0.01760431	-0.06498531	0.206919687	0.260680726	5	0	0
MCMBP	0.0468433	0.046399232	0.000444055	0.003527758	0.206907259	0.260680726	7	0	0
ATP2C2	0.2606387	0.272616277	-0.01197756	-0.04852766	0.20695198	0.260703098	7	0	0
PDLIM7	0.0896177	0.085033769	0.004583914	0.016971937	0.207183417	0.260976317	7	0	0
GLMN	0.0622457	0.064031224	-0.00178557	0.00105744	0.207228403	0.261009407	16	0	0
TMEM110-M	0.1973434	0.208373421	-0.01103	-0.01222234	0.207253344	0.261009407	4	0	0
CRADD	0.2835295	0.279809193	0.003720272	0.006601125	0.207472858	0.261267512	11	0	0
CACNG4	0.2059058	0.197462277	0.008443555	0.127628944	0.20754829	0.261344155	8	0	0
NELFE	0.2491386	0.254295184	-0.00515658	-0.02685527	0.20764332	0.261437501	19	0	0
TIMM50	0.2081396	0.191842174	0.016297432	0.041068108	0.207651574	0.261437501	7	0	0
XYLT2	0.1211991	0.122342448	-0.00114334	0.035482511	0.207752708	0.26154647	8	0	0
TRIAP1	0.0396451	0.039199452	0.000445676	0.014554297	0.207824344	0.261618293	13	0	0
KCTD6	0.0512667	0.050832856	0.000433795	0.016568182	0.208239435	0.262122429	6	0	0
DEDD2	0.0835763	0.087013692	-0.00343742	-0.01524516	0.208436174	0.262351664	11	0	0
XPR1	0.164623	0.165904518	-0.00128154	0.00923158	0.208535413	0.262458156	7	0	0
SVIP	0.2369251	0.249484245	-0.01255912	-0.07021105	0.208638607	0.262569608	3	0	0
ZDHHC3	0.1944722	0.194392926	7.93E-05	-0.00037245	0.208705491	0.262635353	7	0	0
ZNF740	0.048115	0.04737867	0.000736326	0.013862672	0.208737102	0.262656704	8	0	0
POC1B	0.1022416	0.106687818	-0.00444617	-0.02811565	0.208788005	0.262702326	13	0	0
C2	0.1713826	0.171605058	-0.00022247	0.015492923	0.208822052	0.262714562	26	0	0
MRRF	0.0544807	0.051863506	0.002617223	0.060857901	0.208827024	0.262714562	6	0	0
REL	0.0323317	0.031698354	0.000633395	0.022693173	0.208969242	0.26287504	10	0	0
SHOX2	0.1960539	0.16882858	0.02722535	0.309133126	0.209238129	0.26319483	11	0	0
ESPN	0.1214462	0.115009437	0.006436785	0.178717448	0.209255401	0.263198098	9	0	0
MADD	0.3394138	0.33927784	0.000135942	0.037953146	0.209359828	0.263310979	13	0	0
PCBD1	0.3353021	0.342041579	-0.00673944	-0.02725418	0.209576685	0.263565238	6	0	0
DNAH7	0.1547321	0.142337565	0.012394489	0.466024485	0.209606488	0.263584237	8	0	0
GDAP2	0.0404439	0.045986083	-0.00554222	-0.0672597	0.209629195	0.263594311	12	0	0
PRMT7	0.0947177	0.096119747	-0.00140206	-0.00337651	0.209690355	0.26365273	16	0	0
TOMM40	0.1309376	0.140987231	-0.01004963	-0.03638868	0.209848603	0.263833208	6	0	0

ABTB2	0.0822723	0.082539469	-0.00026717	-0.0029879	0.209865427	0.263835866	6	0	0
WDR81	0.0610847	0.056202954	0.004881725	0.022829535	0.210013052	0.264002951	14	0	0
ACACA	0.0472562	0.046729883	0.000526304	0.014371383	0.21010696	0.26410249	4	0	0
AGR3	0.8218326	0.84386248	-0.0220299	-0.04124186	0.210359788	0.264383236	3	0	0
RAB2A	0.0540661	0.054539538	-0.00047347	-0.00500614	0.210346996	0.264383236	11	0	0
PCID2	0.0474591	0.045957994	0.001501073	0.036705702	0.210433495	0.26445734	15	0	0
ADK	0.0567677	0.054716535	0.002051198	0.04179592	0.21046826	0.264482499	13	0	0
STK11IP	0.0587223	0.058006645	0.000715645	0.009673868	0.210492942	0.264494984	10	0	0
RPL15	0.1525485	0.147885029	0.004663444	-0.00807011	0.210532172	0.264521405	7	0	0
SPATA33	0.2969031	0.306112607	-0.00920953	-0.02337628	0.210543465	0.264521405	10	0	0
EGR3	0.0454481	0.046975231	-0.00152711	-0.02073268	0.210795881	0.264809181	10	0	0
PRR22	0.8975206	0.90711086	-0.00959025	-0.01514477	0.210802045	0.264809181	8	0	0
DIRAS2	0.1300503	0.131939998	-0.00188969	0.110467115	0.210862722	0.264862545	9	0	0
DDA1	0.1910027	0.191077795	-7.51E-05	0.01120525	0.21087406	0.264862545	10	0	0
PRDM4	0.0385343	0.03857432	-4.00E-05	0.021699386	0.210907268	0.264869072	2	0	0
RSRC2	0.0777689	0.071616531	0.006152391	0.091162758	0.210908792	0.264869072	15	0	0
ARHGAP21	0.0280001	0.026829901	0.001170166	0.043374444	0.210935672	0.264884282	6	0	0
TIMM8B	0.0867722	0.093068321	-0.00629609	-0.01738824	0.211008129	0.264956721	17	0	0
NRL	0.0453053	0.044736222	0.000569028	0.032506381	0.211044053	0.264983279	14	0	0
SF3B1	0.0720988	0.075774175	-0.00367542	-0.01952153	0.21108034	0.265010288	9	0	0
ANG	0.0405041	0.041114487	-0.00061038	-0.01244684	0.211155779	0.265086446	11	0	0
PKIB	0.0572788	0.055915503	0.001363309	0.018012728	0.211176968	0.265094491	16	0	0
ARID3C	0.2255539	0.213903914	0.011650023	0.068666569	0.211436445	0.265401643	5	0	0
TMEM38A	0.0642547	0.066595051	-0.00234035	-0.00299992	0.211550208	0.26552586	11	0	0
PSRC1	0.0670187	0.067845116	-0.00082644	-0.01556286	0.211611816	0.265547438	9	0	0
C2orf15	0.094553	0.090607212	0.003945785	-0.0232233	0.211597431	0.265547438	12	0	0
PET100	0.1978076	0.205666295	-0.00785867	-0.02588389	0.211597287	0.265547438	5	0	0
EDEM3	0.0471834	0.050512374	-0.003329	-0.04387458	0.211643123	0.265568144	13	0	0
FBXO3	0.2213215	0.225050721	-0.00372919	0.01688702	0.211731931	0.265660994	10	0	0
FAM111B	0.218852	0.23319602	-0.01434399	0.030872747	0.211957157	0.265924983	3	0	0
WWP1	0.1028245	0.095114903	0.007709583	0.089355309	0.2120658	0.266024073	6	0	0
ARID2	0.0358196	0.035480795	0.000338762	0.001191266	0.212061387	0.266024073	10	0	0
DCDC1	0.1101791	0.109159959	0.001019126	0.035648869	0.21219386	0.266166101	14	0	0
SF3A1	0.2371403	0.242605239	-0.00546495	-0.01174504	0.212251574	0.266219877	11	0	0
PARS2	0.0732981	0.077866276	-0.00456822	0.002702834	0.212285338	0.266243607	11	0	0
TMSB10	0.0500455	0.045445377	0.004600119	0.074933139	0.212338369	0.266291497	6	0	0
AASS	0.1199259	0.123080745	-0.0031548	0.028394717	0.212367533	0.26630945	11	0	0
ICAM2	0.427497	0.392127658	0.03536934	0.195202797	0.212389477	0.266318349	5	0	0
UCP2	0.12324	0.11689004	0.006349919	0.087642151	0.212408764	0.266323914	10	0	0
PBX2	0.1913877	0.197024971	-0.00563728	0.026768375	0.212441961	0.266346918	13	0	0
PNKD	0.0752264	0.080374937	-0.00514858	-0.02934314	0.212486095	0.26638363	8	0	0
ZNF181	0.0415831	0.041165448	0.000417637	0.009805629	0.212565443	0.266464481	13	0	0
RHOC	0.1424963	0.132076918	0.010419353	0.013049358	0.212622229	0.266517038	9	0	0
NDRG2	0.1021057	0.089096505	0.01300924	0.149186121	0.212657509	0.266542633	9	0	0
RPAP2	0.0346708	0.034736048	-6.52E-05	0.006440257	0.212784338	0.266654964	15	0	0
TMEM178B	0.0778183	0.060212245	0.017606098	0.413475241	0.212764777	0.266654964	11	0	0
SRSF1	0.0437534	0.043011656	0.000741696	0.01370197	0.212791732	0.266654964	12	0	0
COPS2	0.0754385	0.072702374	0.002736134	0.033288508	0.212877204	0.266743435	9	0	0
YY1	0.033677	0.033124972	0.000552059	0.017945038	0.212951395	0.266817759	5	0	0
NPTN	0.5870743	0.607710202	-0.02063586	-0.04027428	0.212994252	0.266852815	6	0	0
ZNF567	0.1468714	0.137817827	0.009053601	0.078452308	0.213061598	0.266918546	9	0	0
CD55	0.0689701	0.05987941	0.00909071	0.182929007	0.213088929	0.266934141	14	0	0
SLC1A1	0.1238043	0.12271376	0.001090552	0.027807479	0.213204064	0.267059717	8	0	0
GDE1	0.1910697	0.203966673	-0.01289701	-0.04923872	0.21332629	0.267194157	9	0	0
GCFC2	0.2139169	0.204065069	0.009851853	0.097721156	0.213698835	0.267642086	13	0	0
DHX16	0.1180598	0.121893723	-0.00383396	-0.00117742	0.213750442	0.267669338	15	0	0
ARHGAP18	0.2081968	0.202713682	0.005483111	0.07524559	0.213744805	0.267669338	9	0	0
NEK9	0.1116729	0.091750827	0.019922114	0.523281434	0.213768759	0.267673587	12	0	0
PHF1	0.2478321	0.246554643	0.001277447	0.030914945	0.213840275	0.267744445	16	0	0
ACVR1C	0.1113377	0.115544405	-0.00420669	-0.0141006	0.213938393	0.267839882	10	0	0
BHLHE41	0.0701924	0.071391619	-0.00119926	-0.01318483	0.213946364	0.267839882	11	0	0
NGFR	0.2112409	0.197759894	0.013481001	0.067324874	0.214406379	0.268397042	9	0	0
MRPL27	0.0644738	0.065231949	-0.00075818	-0.00558874	0.214427693	0.26840499	10	0	0

NDRG4	0.43192	0.419170283	0.012749701	0.109669121	0.214468419	0.268417558	6	0	0
PNPO	0.1426377	0.146492991	-0.00385527	-0.03001106	0.21448263	0.268417558	16	0	0
GTF2F1	0.2281425	0.220101706	0.008040766	0.129933124	0.214464974	0.268417558	5	0	0
MRPL45	0.0464833	0.045786179	0.000697082	0.04327887	0.214853351	0.268857863	2	0	0
RNF7	0.3218453	0.335008293	-0.01316303	-0.03688485	0.214896101	0.268878721	5	0	0
TTC24	0.6694236	0.668376305	0.001047285	0.001570764	0.215089125	0.269101461	5	0	0
DCUN1D4	0.07921	0.074512017	0.004697938	0.068882311	0.215173918	0.26918877	13	0	0
RNPS1	0.0670619	0.071239413	-0.0041775	-0.00710548	0.215201896	0.269204994	9	0	0
E2F1	0.1264187	0.130213653	-0.00379499	-0.01710258	0.215217738	0.269206035	7	0	0
PIPOX	0.0425479	0.040186716	0.002361212	0.061676609	0.215268066	0.269250211	8	0	0
MBOAT1	0.1929255	0.199672565	-0.00674706	-0.00788153	0.215413554	0.269413395	4	0	0
TAF10	0.167404	0.170038669	-0.00263466	-0.00184719	0.215487995	0.269487706	10	0	0
C2orf49	0.0537325	0.057965638	-0.0042331	-0.03958065	0.215699342	0.269733208	10	0	0
DYNC2H1	0.0920802	0.098461645	-0.00638141	-0.01235107	0.215722595	0.269743479	13	0	0
ZKSCAN3	0.0630755	0.055020152	0.00805538	0.187978183	0.215796957	0.269817652	7	0	0
TOX3	0.3593292	0.345721247	0.013607973	0.067975567	0.215990816	0.270041216	9	0	0
SORT1	0.2197683	0.229436842	-0.00966854	-0.0233515	0.216008447	0.270044435	9	0	0
MMP27	0.7100069	0.722373893	-0.01236695	-0.0187313	0.21619452	0.270258218	2	0	0
PPIB	0.1517021	0.164597881	-0.01289573	-0.03390724	0.216655632	0.270815765	9	0	0
CDK5	0.0654874	0.06670476	-0.00121732	0.027902979	0.216800211	0.270970286	23	0	0
MX1	0.1835193	0.18560004	-0.00208071	0.00131608	0.216809466	0.270970286	10	0	0
MED15	0.0470869	0.046868121	0.000218758	0.032229297	0.216827047	0.270973378	6	0	0
MAP2K7	0.0589881	0.056975517	0.002012608	0.038826953	0.216866562	0.271003878	8	0	0
PTMS	0.287769	0.280933475	0.006835511	0.065408223	0.216906629	0.271035063	6	0	0
TMA7	0.1449774	0.145811174	-0.00083376	0.000907691	0.217079887	0.271232662	15	0	0
KCNH3	0.0823291	0.075480073	0.006849023	0.053861995	0.217154578	0.271307085	9	0	0
KCNQ3	0.196275	0.203314906	-0.00703993	0.153505398	0.217177361	0.271316651	8	0	0
SMCHD1	0.0437071	0.046932384	-0.0032253	-0.06073602	0.217290307	0.27143079	5	0	0
NPHP3	0.1032208	0.105460289	-0.00223953	-0.00217587	0.217415618	0.271557558	12	0	0
COPS7B	0.0467344	0.049656431	-0.00292203	-0.05234418	0.217657438	0.271840667	11	0	0
USF2	0.0374817	0.036692219	0.000789494	0.02801172	0.217762229	0.271952607	3	0	0
ZNF17	0.0667921	0.06739515	-0.00060304	0.006796205	0.217806342	0.271988759	6	0	0
ZNF641	0.3400752	0.34878439	-0.00870918	-0.03424216	0.217850199	0.272024587	9	0	0
ZNF146	0.0930078	0.094310625	-0.00130286	0.021210779	0.218031752	0.272232335	12	0	0
SOX12	0.0811883	0.080150345	0.001038004	0.003416746	0.218110645	0.272311884	8	0	0
TBX21	0.1827221	0.159209255	0.023512811	0.355614769	0.218131024	0.272318372	12	0	0
SQLE	0.2261536	0.227313506	-0.00115986	0.035899514	0.218229566	0.272422431	9	0	0
HIST1H2AH	0.0945983	0.09860604	-0.0040077	-0.04301298	0.218298685	0.27248975	7	0	0
HMG20A	0.0779243	0.073982722	0.003941562	0.055556166	0.218445548	0.272654095	12	0	0
HMBS	0.2566159	0.26942718	-0.01281125	-0.02730618	0.218472701	0.272669012	9	0	0
MTMR3	0.2597003	0.256263964	0.003436334	0.028882053	0.218513155	0.272681553	4	0	0
WDR7	0.2778218	0.280907802	-0.003086	-0.02551519	0.218670001	0.272858296	7	0	0
LAX1	0.5644548	0.571229327	-0.00677453	-0.00384862	0.218799989	0.273001503	6	0	0
GNAL	0.1127961	0.117958662	-0.00516259	0.112971654	0.218901105	0.273108668	10	0	0
DGAT1	0.1260213	0.1178115	0.008209753	0.119566333	0.218931187	0.273116551	11	0	0
ZNF28	0.1563446	0.125591393	0.030753245	0.3803961	0.218953105	0.273116551	4	0	0
ZNF468	0.1563446	0.125591393	0.030753245	0.3803961	0.218953105	0.273116551	4	0	0
CTNNA3	0.529742	0.530603319	-0.00086133	-0.00734767	0.219157562	0.273352576	4	0	0
ATP6V1D	0.1363213	0.145573634	-0.00925233	-0.04357626	0.219192669	0.273377353	9	0	0
DSTN	0.1048361	0.118443621	-0.01360751	-0.01960547	0.219218573	0.27339065	12	0	0
PHIP	0.0547216	0.051497997	0.0032236	0.048557009	0.219256378	0.273418785	11	0	0
ZNF141	0.0610222	0.058633231	0.002389015	0.07549038	0.219325305	0.273485725	8	0	0
CLCN3	0.0460206	0.044730323	0.001290311	0.035524469	0.219368872	0.273521035	9	0	0
GSTM1	0.6246379	0.552981609	0.071656281	0.172845862	0.219489184	0.273631585	1	0	0
VPS28	0.2923516	0.280590617	0.011760939	0.055297871	0.219503303	0.273631585	7	0	0
GSKIP	0.0664626	0.069265015	-0.00280242	-0.03390998	0.219496066	0.273631585	7	0	0
FANCD2	0.0457711	0.047694365	-0.00192323	-0.02794492	0.219538156	0.273656013	6	0	0
HIST2H2AB	0.0780606	0.078833277	-0.0007727	0.010444829	0.219595238	0.273708144	12	0	0
SPRR2F	0.7679671	0.759723574	0.008243491	0.019587598	0.219632749	0.273735875	5	0	0
ACKR3	0.4330807	0.422563037	0.010517706	0.346739441	0.21996438	0.27413015	4	0	0
ST7	0.1196346	0.112091801	0.007542756	0.057736895	0.220068573	0.274221893	11	0	0
TRIM24	0.262468	0.268761881	-0.0062939	-0.01363316	0.220061113	0.274221893	7	0	0
ZBED6	0.1320945	0.135191806	-0.00309729	0.018202526	0.2201372	0.274288352	14	0	0

POLQ	0.0535615	0.051959951	0.00160159	0.049269644	0.220463253	0.27467273	11	0	0
FAM227B	0.0807581	0.08071153	4.66E-05	0.060486103	0.220476321	0.27467273	11	0	0
FAM189A2	0.4540406	0.452695938	0.001344656	0.029744164	0.220526221	0.274715815	2	0	0
RPS6KA5	0.0795261	0.082215764	-0.00268962	0.02332991	0.220663345	0.274867543	10	0	0
HJURP	0.1176218	0.118941907	-0.0013201	0.012337493	0.220763017	0.274972603	11	0	0
EIF2S1	0.0968775	0.098253153	-0.00137565	-0.02058237	0.22085299	0.275065567	10	0	0
DNAJC5	0.2027468	0.209053998	-0.0063072	0.038950274	0.220911852	0.275119773	5	0	0
CRISPLD1	0.3203634	0.321759962	-0.00139653	0.075731109	0.221122476	0.27535441	10	0	0
EXOSC4	0.0498667	0.049951017	-8.43E-05	0.005061949	0.221130962	0.27535441	11	0	0
LSG1	0.209777	0.209430067	0.000346964	0.011090176	0.221287164	0.275529786	11	0	0
TWSG1	0.0527203	0.045173854	0.007546469	0.147215989	0.221307334	0.275535773	11	0	0
TMEM104	0.1355595	0.140529673	-0.00497016	0.005667945	0.221371511	0.275596545	9	0	0
ZFAND5	0.0600666	0.055757592	0.004309049	0.021661057	0.22146292	0.275691208	5	0	0
KCMF1	0.0531216	0.052179214	0.000942362	0.006053809	0.221550317	0.275780865	4	0	0
PIGC	0.1539742	0.156563043	-0.0025888	0.001299794	0.221620669	0.275830152	9	0	0
ZNF266	0.2285886	0.219178988	0.009409614	0.207093158	0.221610293	0.275830152	8	0	0
CYP2U1	0.095822	0.084882658	0.010939341	0.197591818	0.221660978	0.275842038	7	0	0
KIF5B	0.2228441	0.221597992	0.001246114	0.025665578	0.221655002	0.275842038	10	0	0
NAPA	0.0831867	0.080633769	0.002552907	0.004610762	0.221802756	0.275999322	12	0	0
RSF1	0.0450151	0.043965605	0.001049473	0.040289881	0.221839648	0.276026079	10	0	0
BOD1L1	0.0413525	0.043162158	-0.00180968	-0.03058186	0.222041026	0.276257481	8	0	0
ARHGAP8	0.1381596	0.144559694	-0.00640009	-0.02894439	0.222246208	0.276493583	11	0	0
WASF3	0.0680545	0.059594636	0.008459857	0.162764472	0.222359204	0.276603217	9	0	0
IQCH	0.062917	0.06464132	-0.00172428	0.009384952	0.222365175	0.276603217	10	0	0
TXNDC12	0.1222804	0.128533282	-0.00625291	-0.00290524	0.222552549	0.276817095	9	0	0
SPATA25	0.8210627	0.825635078	-0.00457236	-0.00635247	0.222606474	0.276864969	8	0	0
DCTN5	0.0357348	0.036670557	-0.00093577	-0.02191221	0.222814686	0.277104716	8	0	0
SETDB1	0.1063656	0.109894245	-0.00352863	0.017124728	0.223062647	0.27739386	11	0	0
PRR3	0.0467436	0.042390828	0.00435282	0.098102655	0.223086735	0.277404581	42	0	0
EFHD2	0.1144289	0.123021485	-0.00859262	-0.0081538	0.223208668	0.277536962	8	0	0
BNIP1	0.4212088	0.422233277	-0.00102445	0.023076831	0.223372204	0.277711378	8	0	0
U2AF1L4	0.0579497	0.056790658	0.001159092	0.019705621	0.22339028	0.277711378	11	0	0
TBC1D22A	0.0604455	0.059513235	0.000932226	-0.01032071	0.223395393	0.277711378	8	0	0
BLOC1S6	0.0360643	0.03564179	0.00042254	0.017132518	0.223556229	0.277872802	20	0	0
PPARA	0.1668328	0.15276373	0.01406903	0.140129014	0.223582302	0.277885952	5	0	0
CHST9	0.2432497	0.220042536	0.023207126	0.223495742	0.223878382	0.278215385	11	0	0
SPATS2L	0.4455971	0.442460656	0.003136449	0.076876699	0.223932263	0.278240825	10	0	0
TMEM246	0.0695754	0.072087383	-0.00251197	-0.04291327	0.223945392	0.278240825	6	0	0
ATP11A	0.1064638	0.103905401	0.00255838	0.095067951	0.223942392	0.278240825	6	0	0
PSMD3	0.1280697	0.133775567	-0.00570588	0.024319865	0.223971085	0.278253473	14	0	0
ZBTB7A	0.0461985	0.043992319	0.002206134	0.051649428	0.224369215	0.278728788	12	0	0
TOMM40L	0.2278876	0.23028245	-0.00239488	0.05357886	0.224476757	0.278823761	13	0	0
PIK3C3	0.3871162	0.390048355	-0.00293218	0.012579427	0.224461599	0.278823761	8	0	0
ZNF497	0.1589288	0.170414604	-0.01148582	0.045448096	0.22457007	0.27892035	5	0	0
PYGB	0.1835673	0.194634063	-0.0110668	-0.01760003	0.224641634	0.278989914	9	0	0
SMAD6	0.0384015	0.038254704	0.00014676	0.000603526	0.224663481	0.278997728	10	0	0
NIT2	0.127029	0.129919573	-0.00289056	0.03071015	0.224892974	0.279263388	13	0	0
PCOLCE2	0.1615138	0.160604409	0.000909387	0.074364675	0.225009479	0.279388716	17	0	0
COX15	0.1230618	0.124463233	-0.00140147	-0.00693353	0.225038012	0.279404803	17	0	0
CCNT1	0.1880948	0.200660176	-0.0125654	-0.02568664	0.225160027	0.279536945	8	0	0
MSH2	0.1241328	0.127964966	-0.00383212	-0.01951305	0.225255574	0.279636211	9	0	0
MPLKIP	0.0720883	0.068178689	0.003909567	0.058226047	0.225329057	0.279708075	13	0	0
SWI5	0.0499184	0.047700493	0.002217917	0.054415697	0.225377969	0.279749429	1	0	0
MSL2	0.1525983	0.165330831	-0.01273256	0.001645945	0.225425956	0.27978963	5	0	0
EFNA1	0.0855434	0.090804381	-0.00526103	-0.00128303	0.225508041	0.279872143	13	0	0
ZNF322	0.1296854	0.127978311	0.001707054	-0.00672356	0.225552101	0.279907457	10	0	0
GTF2H3	0.0791758	0.07788005	0.001295744	-0.02182163	0.225622575	0.279975543	14	0	0
LMOD1	0.2558998	0.260104535	-0.00420473	-0.02611218	0.225716182	0.28005295	10	0	0
FNBP1	0.1043077	0.09110098	0.013206682	0.120509986	0.225708174	0.28005295	5	0	0
RANBP2	0.154734	0.164517256	-0.0097833	-0.00743406	0.225809895	0.280130465	7	0	0
NOXA1	0.0684305	0.065808453	0.002622056	0.0356204	0.225797378	0.280130465	3	0	0
CRYGC	0.8402148	0.85072903	-0.0105142	-0.01748159	0.226033715	0.280369343	9	0	0
SLC24A1	0.0578685	0.055094027	0.00277452	0.053835847	0.226033527	0.280369343	7	0	0

NOD2	0.1426973	0.129699756	0.0129975	0.174725744	0.226086035	0.280405117	6	0	0
ZNF543	0.030016	0.021563233	0.008452754	0.355515702	0.226093823	0.280405117	6	0	0
PREP	0.0402089	0.042670225	-0.00246136	-0.05462818	0.226200969	0.280518605	3	0	0
KLHL11	0.1657457	0.168612878	-0.00286718	-0.02620991	0.226217797	0.280520078	7	0	0
CSN2	0.7156088	0.742966103	-0.02735731	-0.05339298	0.226242511	0.28053133	1	0	0
CNOT4	0.0587273	0.060040226	-0.00131296	-0.00640796	0.226300579	0.280583933	10	0	0
ZNF571	0.0687685	0.058591654	0.010176796	0.388744362	0.226409302	0.280699332	10	0	0
NLRC5	0.0972163	0.087213716	0.010002554	0.100733181	0.226473227	0.280759179	6	0	0
DCXR	0.0524714	0.054943059	-0.00247169	-0.03953468	0.226642936	0.280950149	8	0	0
NEU1	0.0470868	0.04696012	0.00012672	0.016611394	0.226666275	0.280959661	24	0	0
LYPLAL1	0.1898181	0.187445307	0.002372791	0.114723877	0.22687573	0.281180423	13	0	0
SREBF2	0.0372847	0.03471115	0.002573546	0.087353399	0.226866285	0.281180423	7	0	0
LMAN1	0.0530281	0.053382367	-0.0003543	-0.0046575	0.226897725	0.281188254	2	0	0
ERAP1	0.0699675	0.074665964	-0.00469851	-0.01871969	0.226982689	0.281271138	10	0	0
TEAD4	0.1089211	0.119930326	-0.01100927	-0.00689446	0.226995971	0.281271138	8	0	0
DUSP18	0.0557507	0.056349647	-0.00059891	0.041773483	0.227191284	0.281493705	8	0	0
PRPF40A	0.0537907	0.052550061	0.001240616	0.043150604	0.227408681	0.281743601	8	0	0
NXPH3	0.1436683	0.133587121	0.010081187	0.218173935	0.227455675	0.281782359	12	0	0
KLF10	0.0793952	0.076966942	0.002428277	0.038622658	0.227495282	0.281811961	15	0	0
GGCX	0.110653	0.117519327	-0.0068663	-0.00862993	0.22765558	0.281991055	11	0	0
EDF1	0.468027	0.471955394	-0.00392835	0.005939897	0.227742282	0.28207897	8	0	0
RHBDD3	0.0563953	0.055571153	0.000824139	0.001827457	0.227780219	0.282106477	14	0	0
STK36	0.0484455	0.047745764	0.000699709	0.019940709	0.22779627	0.282106877	14	0	0
POLE4	0.2102112	0.205342079	0.004869153	0.18281945	0.22787208	0.282180722	5	0	0
PTGES3	0.0494376	0.038320696	0.01111695	0.314548374	0.227887364	0.282180722	13	0	0
RPL7A	0.1042393	0.111882815	-0.00764355	-0.00206379	0.227906312	0.282184704	9	0	0
PSMB10	0.2820922	0.279466284	0.00262591	0.059488385	0.227922273	0.282184986	7	0	0
UBE2R2	0.1683351	0.16640357	0.001931485	-0.01901104	0.228108702	0.282376816	4	0	0
LIPA	0.0831041	0.078476891	0.004627252	0.056515569	0.228130959	0.282384877	11	0	0
HBS1L	0.5584315	0.600277842	-0.04184631	-0.10247963	0.228288707	0.282560641	1	0	0
KHK	0.1293294	0.119545189	0.009784224	0.061513049	0.228374135	0.282627578	6	0	0
MEF2B	0.1680614	0.168625757	-0.00056436	0.047503381	0.228374302	0.282627578	11	0	0
CNTRL	0.1350916	0.135412241	-0.0003206	0.133871697	0.228424221	0.282669852	7	0	0
BEND5	0.0984931	0.086627058	0.011866017	0.049415043	0.228515249	0.282762988	8	0	0
RCL1	0.070141	0.06465078	0.005490188	0.029958574	0.228554931	0.28279258	8	0	0
ACTA2	0.0515107	0.049879962	0.001630753	0.034275362	0.228634582	0.28287162	9	0	0
MC1R	0.8542334	0.864314589	-0.01008115	-0.0163746	0.228713444	0.282949671	5	0	0
APOM	0.1080024	0.106483384	0.001518992	0.040715232	0.228830305	0.283074719	27	0	0
CREBL2	0.0632803	0.06893315	-0.00565289	-0.01384474	0.228936515	0.283186574	13	0	0
ACAD10	0.0929416	0.095211377	-0.00226979	-0.00067794	0.228962928	0.283199716	13	0	0
ENTPD4	0.3109014	0.314129591	-0.00322821	-0.00280816	0.228989254	0.283212748	9	0	0
USP5	0.099015	0.10670602	-0.00769102	-0.03442322	0.229227067	0.283487325	21	0	0
GSS	0.1140973	0.107394389	0.006702896	0.033084848	0.229243122	0.283487634	12	0	0
WNT10B	0.1522517	0.142258526	0.009993205	0.060377255	0.229305497	0.283545219	13	0	0
SCRT2	0.1690464	0.156768422	0.012277976	0.242295641	0.229366252	0.283600793	5	0	0
METTL18	0.0415514	0.043888741	-0.00233733	-0.06600793	0.229530693	0.28375132	8	0	0
RAP2B	0.0352638	0.035317791	-5.40E-05	-0.00816579	0.229535454	0.28375132	6	0	0
FAU	0.0920027	0.103136248	-0.01113356	-0.03236189	0.229526592	0.28375132	11	0	0
RNF166	0.2143107	0.219006253	-0.0046956	-0.01785285	0.229613505	0.283828245	8	0	0
ZBTB7B	0.0445663	0.044170961	0.000395299	0.016776978	0.229689444	0.283902548	8	0	0
KLHL22	0.0640998	0.063823706	0.000276061	0.043335005	0.229755782	0.283964975	8	0	0
SLC38A6	0.1428418	0.152915897	-0.01007405	-0.02743674	0.229893718	0.284115878	15	0	0
TSTA3	0.043386	0.040933098	0.002452916	0.041980647	0.229928856	0.284128729	5	0	0
PDXK	0.2851132	0.291712274	-0.00659903	-0.02302729	0.229936258	0.284128729	10	0	0
SDF2L1	0.1190555	0.124177813	-0.00512236	-0.00784717	0.229951641	0.284128729	9	0	0
LRRC41	0.1165091	0.125077977	-0.0085689	0.022752149	0.229984761	0.284150077	14	0	0
EIF1AD	0.0692733	0.072451401	-0.00317813	-0.01859324	0.230043264	0.284202782	15	0	0
RBM42	0.1022315	0.086301601	0.015929917	0.069685392	0.230131195	0.284291833	12	0	0
EXOSC6	0.3407675	0.349334533	-0.00856698	0.193900557	0.230277869	0.284453434	8	0	0
TRERF1	0.0461311	0.044845447	0.001285616	0.032356362	0.230383361	0.284564146	8	0	0
RUNDC3A	0.1238111	0.114763923	0.009047151	0.060123922	0.230501694	0.284690702	10	0	0
KRTAP9-6	0.5345199	0.536627237	-0.00210738	-0.00979545	0.230517605	0.28469075	3	0	0
TMEM69	0.038957	0.039501904	-0.00054494	-0.02338359	0.230598178	0.284770651	9	0	0

ALG14	0.034925	0.033392372	0.001532661	0.031403767	0.230741725	0.284928301	11	0	0
SLC4A1AP	0.1976901	0.200557776	-0.0028677	-0.0108848	0.230869038	0.285034072	17	0	0
TMEM131	0.0694569	0.073586984	-0.0041301	-0.01105757	0.230875056	0.285034072	9	0	0
KDM5A	0.1116647	0.115549138	-0.00388444	-0.01472682	0.230853542	0.285034072	15	0	0
PLCH2	0.6828143	0.684687783	-0.00187349	0.0033066	0.230946977	0.28510324	5	0	0
MPP7	0.2246789	0.234698396	-0.01001953	-0.06031672	0.2311151	0.285291152	1	0	0
YAF2	0.2394694	0.248926677	-0.0094573	-0.0438365	0.231190823	0.285364986	8	0	0
RPP25	0.1075346	0.095037824	0.012496805	0.196273899	0.23133293	0.285520743	13	0	0
SRA1	0.8559451	0.866294256	-0.01034912	-0.01827261	0.231404501	0.285589426	7	0	0
PON2	0.226351	0.223797547	0.002553499	0.054924797	0.231502426	0.285690623	17	0	0
SRP9	0.212793	0.218773295	-0.00598025	-0.02585801	0.231625623	0.285822991	11	0	0
VSTM2L	0.1247997	0.12579337	-0.00099364	0.054171565	0.231863778	0.286097187	9	0	0
CBX4	0.121866	0.11168204	0.010183941	0.098174525	0.231946638	0.286152132	7	0	0
SAMD4B	0.0838223	0.087661475	-0.00383916	0.003597679	0.231988077	0.286152132	5	0	0
EIF3D	0.1944407	0.200597469	-0.00615681	-0.02363056	0.231975863	0.286152132	10	0	0
XPNPEP3	0.0435812	0.045040937	-0.00145978	-0.02717015	0.231956152	0.286152132	14	0	0
PPP6R2	0.046013	0.044489976	0.001522976	0.039600474	0.231987222	0.286152132	6	0	0
RAP1GDS1	0.0706997	0.074033232	-0.00333358	-0.06941717	0.232067655	0.286230605	6	0	0
C12orf73	0.0886357	0.087976534	0.000659165	0.046976528	0.232105435	0.286256824	8	0	0
HM13	0.1312463	0.141309131	-0.01006286	-0.04390465	0.232120832	0.286256824	8	0	0
BAP1	0.1081478	0.106081873	0.002065911	0.078205826	0.232203077	0.286338563	17	0	0
GOLIM4	0.058499	0.060950255	-0.00245129	-0.01944394	0.232266067	0.286396547	9	0	0
NFE2	0.1530125	0.159929554	-0.00691708	0.045287485	0.232320267	0.286443687	10	0	0
FAM208B	0.3116782	0.312695203	-0.00101698	-0.00401125	0.232431724	0.28656141	3	0	0
ZFAND2A	0.1253526	0.126148274	-0.00079566	0.065288653	0.232609429	0.286756706	3	0	0
ZNF365	0.1412442	0.131418923	0.009825324	0.209959402	0.232622105	0.286756706	15	0	0
PRR5-ARHG	0.1469608	0.153598089	-0.00663726	-0.02248602	0.232638363	0.28675704	10	0	0
PARP2	0.0467911	0.042757798	0.004033301	0.080292779	0.232739596	0.286862108	10	0	0
CHD7	0.051632	0.053480315	-0.00184833	-0.04322515	0.232758021	0.286865104	7	0	0
MEX3A	0.0669724	0.055229621	0.011742816	0.200619843	0.23285202	0.286961237	13	0	0
TRAPPC8	0.5372889	0.511606812	0.025682107	0.069339936	0.232926938	0.287033841	1	0	0
SLITRK3	0.1320307	0.111037445	0.020993253	0.317515532	0.233074887	0.287196425	9	0	0
ARAP1	0.0952577	0.097247342	-0.00198966	-0.0271635	0.23312477	0.287218428	16	0	0
STARD10	0.0952577	0.097247342	-0.00198966	-0.0271635	0.23312477	0.287218428	16	0	0
NMD3	0.1842342	0.159781584	0.024452664	0.239648115	0.233168071	0.287252045	6	0	0
VPS13B	0.1040016	0.112222717	-0.00822112	-0.01799552	0.23323763	0.287318004	13	0	0
TIRAP	0.0972911	0.087791109	0.009499969	0.173533106	0.233333004	0.287415752	7	0	0
PPP3CA	0.2400089	0.252320983	-0.01231204	0.002749475	0.233453821	0.2875066	4	0	0
TCF7	0.1617391	0.164319146	-0.00258002	-0.00286648	0.233454687	0.2875066	8	0	0
TMX2	0.0384231	0.038778533	-0.00035541	-0.00171161	0.233454846	0.2875066	11	0	0
HIST1H2AB	0.0629012	0.057749619	0.005151575	0.107903679	0.233531319	0.287581032	7	0	0
TTI1	0.1722607	0.179257151	-0.00699646	0.002338523	0.233641184	0.287696572	11	0	0
RGL1	0.0340451	0.03529373	-0.00124864	-0.02305856	0.233827845	0.287906654	14	0	0
TRAM2	0.0669094	0.067487635	-0.00057826	-0.00050254	0.234101392	0.28822368	6	0	0
TSPYL1	0.1415602	0.146040548	-0.00448035	-0.04431695	0.234123402	0.288230993	13	0	0
DTWD1	0.0559813	0.054283688	0.001697661	0.061688029	0.23421366	0.28832232	10	0	0
LDLRAD1	0.5772662	0.581853322	-0.00458708	-0.01340475	0.234251771	0.288349445	8	0	0
PSMA4	0.1889603	0.189631536	-0.00067121	-0.01364244	0.234383014	0.288491198	10	0	0
ATRNL1	0.0502337	0.043534349	0.006699345	0.239312317	0.234453611	0.28855829	11	0	0
ADAM22	0.1177783	0.116706174	0.001072132	0.093868078	0.234513868	0.288612648	16	0	0
MITF	0.0549385	0.053362675	0.001575779	0.040400091	0.234570175	0.288662138	11	0	0
FOXP1	0.0769675	0.073611793	0.003355709	0.058174706	0.234595796	0.288673862	12	0	0
IGSF9B	0.0928997	0.075527419	0.017372321	0.363544906	0.234617355	0.288680584	9	0	0
TRA2A	0.2841908	0.292172525	-0.00798171	-0.00317822	0.234639771	0.288688361	10	0	0
HIST1H2AC	0.0480679	0.047940536	0.000127317	0.007540569	0.23503889	0.289159581	12	0	0
SPRR2G	0.7243064	0.738269441	-0.01396306	-0.0281923	0.235219353	0.289322063	5	0	0
ZBTB24	0.2089066	0.204615181	0.004291423	0.032244369	0.235216471	0.289322063	4	0	0
LMNA	0.1039503	0.093890783	0.010059476	0.188234041	0.235272795	0.289348111	13	0	0
COX8A	0.0425048	0.044319368	-0.00181461	-0.04176982	0.235257906	0.289348111	10	0	0
KCNG3	0.1310389	0.128162886	0.002876023	0.036471244	0.235315429	0.289380702	9	0	0
AZI2	0.0405797	0.039172276	0.001407463	0.004566191	0.235420026	0.289489482	13	0	0
CLCN6	0.0467024	0.045525948	0.001176403	0.014892978	0.235490925	0.289556812	17	0	0
ACO1	0.1555775	0.157757843	-0.00218034	0.066846096	0.235696505	0.289789726	7	0	0

CARS2	0.0394608	0.039489218	-2.84E-05	0.009586827	0.235810693	0.289910246	13	0	0
ALKBH7	0.3373553	0.341785224	-0.00442997	-0.01726367	0.235902292	0.289995912	7	0	0
HSD17B14	0.1444186	0.108591406	0.035827197	0.49244999	0.235912709	0.289995912	7	0	0
ACVR1	0.0554793	0.05874995	-0.00327064	-0.04420361	0.236119839	0.290230635	6	0	0
SPG7	0.0313041	0.030391357	0.000912773	0.043995028	0.236301715	0.290434287	11	0	0
C1orf50	0.0479492	0.046208021	0.001741148	0.024949877	0.236363421	0.290490224	11	0	0
BAG4	0.0259829	0.025880472	0.000102383	0.007461211	0.236424897	0.290526831	7	0	0
USP31	0.1379293	0.151136457	-0.01320718	-0.03702651	0.236425604	0.290526831	4	0	0
BET1	0.0694408	0.075153945	-0.00571319	-0.00653745	0.236475078	0.290567719	11	0	0
LRRC1	0.0482002	0.05061725	-0.00241701	-0.03952407	0.236508519	0.290588902	7	0	0
APOA2	0.8154002	0.811818549	0.003581651	0.007874152	0.236883925	0.291030213	4	0	0
PDZD7	0.0443871	0.042969033	0.001418078	0.039120519	0.236935914	0.291074147	10	0	0
PIGK	0.0487718	0.04691356	0.001858206	0.019148036	0.236952915	0.291075097	8	0	0
RBBP5	0.0449004	0.043176805	0.001723557	0.045975315	0.237026847	0.291145975	7	0	0
LRP2	0.0625962	0.05667747	0.005918688	0.168064473	0.237048881	0.2911531	8	0	0
ECI2	0.0488982	0.037325509	0.011572664	0.334880912	0.237070487	0.2911597	9	0	0
WDR53	0.0477606	0.050160221	-0.00239961	-0.02056615	0.237178201	0.291272044	9	0	0
SSBP2	0.3249385	0.31641564	0.008522851	0.059736223	0.237253209	0.291344212	9	0	0
KCNH1	0.108033	0.102224184	0.005808846	0.13365171	0.237395789	0.291499341	10	0	0
EXTL2	0.0435481	0.045123347	-0.00157529	0.001753938	0.237436821	0.291529766	10	0	0
ORC3	0.1254311	0.130021188	-0.0045901	0.003297437	0.23761954	0.291734142	18	0	0
CYTH1	0.1555595	0.155106226	0.000453283	0.038526981	0.237648337	0.291749526	7	0	0
ZNF343	0.0645425	0.056357977	0.008184504	0.100141191	0.237768523	0.291877095	9	0	0
SCNN1G	0.0807163	0.087838221	-0.0071219	-0.02053945	0.2377856	0.291878082	10	0	0
SKAP2	0.7611059	0.749398048	0.011707839	0.022072692	0.237829702	0.291912239	1	0	0
NR2F6	0.1382894	0.129135113	0.009154289	0.080109244	0.238082337	0.292202327	5	0	0
PLEKHA6	0.2462212	0.248622378	-0.00240121	0.144863639	0.238113674	0.29222079	8	0	0
ATP13A4	0.0863704	0.085983168	0.000387233	0.039769335	0.238239666	0.292355408	14	0	0
RORC	0.5311992	0.531715615	-0.00051642	-0.00414496	0.238285626	0.292391803	5	0	0
WBP11	0.0831556	0.087872279	-0.00471672	-0.01345912	0.23831258	0.292404874	12	0	0
TPGS1	0.0319556	0.032642745	-0.00068718	-0.01897697	0.23834533	0.292425052	6	0	0
PARPBP	0.0509721	0.054756826	-0.00378473	-0.04453258	0.238477872	0.292567655	12	0	0
CYB5R1	0.0922436	0.076803361	0.015440244	0.236429793	0.238546143	0.292631395	7	0	0
ATP2B4	0.0608249	0.058180481	0.002644444	0.037991341	0.238612338	0.292692579	7	0	0
SIGIRR	0.3064512	0.317993114	-0.01154189	-0.03379771	0.238666145	0.292738561	9	0	0
CREB3L2	0.0466695	0.045321787	0.001347684	0.026060201	0.238773788	0.292750473	13	0	0
AKR1D1	0.0466695	0.045321787	0.001347684	0.026060201	0.238773788	0.292750473	13	0	0
CAPRIN1	0.1348536	0.138812215	-0.00395864	-0.00654437	0.238724595	0.292750473	10	0	0
CCDC15	0.065378	0.067360016	-0.00198205	-0.0067068	0.2387707	0.292750473	11	0	0
POU6F1	0.1162887	0.104898868	0.011389818	0.136442646	0.238758641	0.292750473	7	0	0
E2F7	0.0458844	0.047089166	-0.00120476	-0.02637678	0.238758465	0.292750473	10	0	0
PICALM	0.4859564	0.489977007	-0.00402059	-0.00091837	0.238817746	0.292784354	7	0	0
ASB7	0.050539	0.051283822	-0.00074484	-0.02015985	0.238892046	0.292855426	12	0	0
OGFOD1	0.141632	0.149808137	-0.00817616	0.000593739	0.239301953	0.293337878	13	0	0
LYSMD3	0.1638315	0.167885982	-0.00405451	0.001099769	0.239330036	0.293351244	8	0	0
TRAPPC9	0.4119503	0.411644841	0.000305489	0.092323582	0.239345567	0.293351244	10	0	0
TBC1D10B	0.0461706	0.047180814	-0.00101019	-0.01670708	0.239955374	0.29407855	6	0	0
ANKRD18A	0.0649994	0.050154392	0.014845004	0.320908342	0.240034576	0.294135421	3	0	0
IKZF5	0.0459634	0.044672997	0.001290373	0.036871312	0.24002952	0.294135421	10	0	0
CEP152	0.1345503	0.143613179	-0.00906291	-0.02724399	0.240097467	0.294192387	8	0	0
TBCB	0.4401818	0.460957202	-0.02077542	-0.02170086	0.240294259	0.294413404	5	0	0
INVS	0.1462436	0.153754276	-0.00751067	-0.06520487	0.240425075	0.294553561	10	0	0
CRTC2	0.1090471	0.104793637	0.004253499	0.006358909	0.240463527	0.294580549	11	0	0
C1orf115	0.403726	0.403958655	-0.00023266	0.10858634	0.240581152	0.294704517	10	0	0
TMOD2	0.09762	0.100804177	-0.00318413	0.003791635	0.240605979	0.294714801	12	0	0
ERMARD	0.1103238	0.117932586	-0.00760879	-0.03221833	0.240772207	0.294898272	10	0	0
FIP1L1	0.2521077	0.249315398	0.002792333	-0.03433407	0.240862519	0.294988743	11	0	0
LNP1	0.0635045	0.063769258	-0.00026474	0.024702634	0.24089596	0.294989413	13	0	0
DHX29	0.0381552	0.037493256	0.000661948	0.013333185	0.240888349	0.294989413	14	0	0
SLC17A6	0.1837092	0.175053712	0.008655501	0.141866675	0.240959336	0.295046876	8	0	0
KLHL10	0.0269893	0.017975251	0.009014092	0.405523632	0.241022927	0.295104595	2	0	0
MBTPS1	0.0537254	0.054051893	-0.00032648	0.014499273	0.241184684	0.295282491	8	0	0
HSD17B7	0.0492859	0.043634238	0.005651656	0.144534625	0.24125706	0.295350941	1	0	0

UBXN7	0.1146365	0.117952857	-0.00331635	0.031013989	0.24130872	0.295394022	11	0	0
GGNBP2	0.2116368	0.225786332	-0.01414957	-0.01831191	0.241424617	0.295515726	9	0	0
AKAP8L	0.0532905	0.052938056	0.000352456	0.021548331	0.241665969	0.295770783	14	0	0
SMIM7	0.0668333	0.06846338	-0.00163005	0.013084696	0.241660105	0.295770783	11	0	0
ENOX1	0.1554224	0.153106558	0.00231589	0.028680227	0.241709332	0.29580367	10	0	0
SPHAR	0.8232768	0.83127186	-0.00799509	-0.01500514	0.241779456	0.2958693	7	0	0
MARS2	0.1275232	0.137317317	-0.00979415	-0.01922648	0.241801489	0.295876075	8	0	0
SMARCD2	0.1115989	0.109325651	0.002273209	0.009051045	0.241856683	0.295923423	12	0	0
RAB31	0.3525543	0.344122892	0.008431424	0.098639282	0.241888052	0.295941617	5	0	0
ECH1	0.2005715	0.21666236	-0.01609088	-0.03737222	0.241941989	0.295967229	7	0	0
PCNA	0.0434249	0.046390061	-0.00296521	-0.02614494	0.241934548	0.295967229	12	0	0
CCPG1	0.0407045	0.039658197	0.001046282	0.022017246	0.241958552	0.295967305	9	0	0
CMPK1	0.1595593	0.17076176	-0.01120247	-0.02541003	0.242105066	0.296069131	11	0	0
EFHC1	0.1374632	0.141894749	-0.00443155	0.017727134	0.242101591	0.296069131	9	0	0
OR2F2	0.5241984	0.555109727	-0.03091137	-0.08115547	0.242107825	0.296069131	1	0	0
CD3G	0.6481309	0.64940781	-0.00127689	0.000295991	0.242088018	0.296069131	4	0	0
BEND3	0.0466986	0.047213417	-0.00051485	-0.01356297	0.242241697	0.296212645	6	0	0
BTN2A2	0.2582687	0.269270431	-0.0110017	0.053856802	0.242402999	0.296389678	6	0	0
SERINC1	0.0493838	0.049098627	0.000285219	0.009674191	0.242471017	0.296452636	15	0	0
AGBL3	0.1687313	0.167322664	0.001408588	0.035947335	0.242554239	0.296534172	8	0	0
PGS1	0.0753133	0.078702108	-0.00338883	-0.01357928	0.242725388	0.296723184	13	0	0
SLC7A6OS	0.0984121	0.098435431	-2.33E-05	0.009863774	0.242840698	0.296843914	15	0	0
MAPK1IP1L	0.1168176	0.122520525	-0.00570291	-0.05136122	0.242894158	0.296868799	9	0	0
C16orf95	0.104258	0.102627123	0.001630846	0.053680375	0.242879625	0.296868799	12	0	0
GSE1	0.0945254	0.090172584	0.004352774	0.01386569	0.24300898	0.296968663	8	0	0
ZNF576	0.0473851	0.0457638	0.001621263	0.073675119	0.243005295	0.296968663	13	0	0
ZNF592	0.0533786	0.047350659	0.006027947	0.163564511	0.243116973	0.297080394	7	0	0
CLGN	0.1848439	0.18619463	-0.00135076	0.119272939	0.243205651	0.297168509	14	0	0
RGS2	0.0826719	0.080587582	0.002084366	0.192973104	0.243250681	0.29718304	13	0	0
C20orf96	0.12455	0.113397835	0.011152144	0.097582947	0.243238235	0.29718304	10	0	0
TGDS	0.1686061	0.171718871	-0.00311279	0.002537746	0.243287173	0.297207379	7	0	0
RPRM	0.2133563	0.194397212	0.018959128	0.439932811	0.243352643	0.297267112	16	0	0
PRRC1	0.0564564	0.050875919	0.005580464	0.060013427	0.243557561	0.297476909	9	0	0
RMND5B	0.0398666	0.040330409	-0.00046376	0.007079773	0.243546549	0.297476909	5	0	0
PPWD1	0.0564802	0.058424282	-0.00194405	-0.00876711	0.243641067	0.297528357	9	0	0
TMEM120A	0.3635844	0.3522059	0.011378479	0.030993698	0.24366353	0.297528357	9	0	0
MRPS6	0.0870311	0.082480924	0.004550196	0.029879209	0.243666036	0.297528357	9	0	0
SLC5A3	0.0870311	0.082480924	0.004550196	0.029879209	0.243666036	0.297528357	9	0	0
ZNF362	0.0807748	0.078432612	0.002342209	0.037086055	0.243800239	0.297671959	6	0	0
NDUFS8	0.0770124	0.081626216	-0.00461378	0.012442311	0.243883881	0.297753814	14	0	0
HSF4	0.3661343	0.334255368	0.031878971	0.143486389	0.243939987	0.297802043	9	0	0
SLC36A4	0.1089004	0.111336431	-0.00243605	0.055906587	0.244015418	0.297873854	11	0	0
SLCO5A1	0.1406706	0.130605223	0.010065385	0.135442274	0.244036308	0.297879082	7	0	0
HIVEP1	0.2056686	0.222971136	-0.01730254	-0.06187771	0.244144209	0.297970232	3	0	0
C19orf73	0.1009918	0.100364987	0.000626852	0.017785499	0.244128921	0.297970232	5	0	0
RBPMS	0.319484	0.315510751	0.003973213	0.084656344	0.244263078	0.298095023	7	0	0
MFSD9	0.1421398	0.143718077	-0.00157824	-0.04567494	0.244451039	0.298283817	8	0	0
TMEM126A	0.0483654	0.050153162	-0.00178774	-0.00484261	0.244435692	0.298283817	8	0	0
ATF7IP2	0.778647	0.768483838	0.010163198	0.01871267	0.244486305	0.298306556	1	0	0
SPNS2	0.1221986	0.110756368	0.011442264	0.302125438	0.244583448	0.298404783	6	0	0
MINK1	0.0358377	0.036853281	-0.00101556	-0.01612621	0.244679108	0.298501189	2	0	0
TECPR1	0.2053561	0.212126326	-0.00677027	-0.0083116	0.24476163	0.298574459	8	0	0
HEBP1	0.1455933	0.137941381	0.007651906	0.196604704	0.24477246	0.298574459	7	0	0
MRPL49	0.1098366	0.113390629	-0.003554	-0.01787031	0.244946381	0.29876629	14	0	0
KDELC1	0.0449699	0.037610807	0.007359072	0.220497552	0.245048964	0.298871088	15	0	0
FGFRL1	0.0896937	0.088249341	0.001444363	0.121038017	0.245121431	0.298939143	8	0	0
KLK7	0.3341778	0.334191176	-1.33E-05	0.028945456	0.245212159	0.299029459	14	0	0
FAM149B1	0.0555786	0.052635261	0.002943346	0.05062939	0.245272631	0.299082868	14	0	0
ZNF646	0.039007	0.038391342	0.000615678	0.017028324	0.245299095	0.299094804	14	0	0
SCCPDH	0.1853871	0.19064844	-0.00526134	0.047702507	0.245532489	0.299359032	8	0	0
MPV17L2	0.0373098	0.036821826	0.000487934	0.011974358	0.245549901	0.299359912	10	0	0
SCAMP3	0.1434603	0.147138326	-0.00367806	6.59E-05	0.245856225	0.299712992	15	0	0
STK40	0.1065027	0.101792173	0.004710566	0.046244343	0.245875866	0.299716565	9	0	0

C1orf109	0.0594844	0.057417242	0.002067152	0.040200509	0.245964099	0.29978337	5	0	0
RPS7	0.1269112	0.131826503	-0.0049153	-0.05784767	0.245956001	0.29978337	12	0	0
BDKRB2	0.1199488	0.106011531	0.013937288	0.163988921	0.246002722	0.299810072	7	0	0
PML	0.1778299	0.182327607	-0.00449775	-0.01227989	0.246092335	0.299898907	14	0	0
ASPHD1	0.0595141	0.057614931	0.001899135	0.037424744	0.246124553	0.299917792	9	0	0
UVRAG	0.054502	0.055776236	-0.00127428	-0.03160638	0.246194225	0.299982311	13	0	0
ABT1	0.0725556	0.072901812	-0.00034622	0.035968046	0.246267542	0.300051262	8	0	0
USP25	0.4560602	0.456847562	-0.00078737	0.004625214	0.246443976	0.300245833	4	0	0
RAB38	0.2562981	0.25808345	-0.00178536	0.03446928	0.246521603	0.300320007	11	0	0
ALKBH2	0.1978356	0.201637015	-0.00380141	-0.03561739	0.246565548	0.300353142	10	0	0
DNAJB7	0.8832404	0.881173712	0.002066733	0.003978308	0.246730553	0.300533731	5	0	0
MAP3K7CL	0.2897217	0.256548511	0.033173184	0.171738755	0.246777762	0.300570823	2	0	0
HIST1H4C	0.1234104	0.111920513	0.011489858	0.105854172	0.247041165	0.300871212	8	0	0
GCLM	0.0517797	0.051161333	0.000618417	-0.01960671	0.2471033	0.300926452	8	0	0
NEDD9	0.6743292	0.663803152	0.010526098	0.030985154	0.247444357	0.301321338	9	0	0
SPRY4	0.0772333	0.062831623	0.014401675	0.273429209	0.247495212	0.301362805	2	0	0
TCTE3	0.0751887	0.080912746	-0.00572404	-0.04016932	0.247592677	0.301461018	11	0	0
DOCK10	0.1766967	0.166812827	0.009883832	0.289905493	0.247622048	0.301473956	7	0	0
TTC26	0.1898105	0.191692496	-0.00188195	-0.01807847	0.24763692	0.301473956	11	0	0
COL4A4	0.0565335	0.050414499	0.006119021	0.116970938	0.247804427	0.301616463	16	0	0
CYB561D2	0.2236779	0.220576347	0.003101522	0.064863621	0.247800962	0.301616463	14	0	0
NHLRC2	0.0416404	0.041725237	-8.48E-05	0.008221831	0.247774651	0.301616463	18	0	0
PAPSS1	0.1241285	0.119569835	0.004558671	0.042135278	0.247899663	0.301711906	9	0	0
COMMD5	0.1539081	0.160006996	-0.00609887	0.00418305	0.248340289	0.302227672	6	0	0
STARD6	0.2192012	0.22526775	-0.00606655	0.079956524	0.248433585	0.302320699	12	0	0
PHKG2	0.1691655	0.173520393	-0.00435489	0.083861627	0.248496167	0.30237634	10	0	0
PIAS1	0.2656798	0.258324944	0.007354812	0.06002162	0.248526931	0.302393259	10	0	0
HIVEP2	0.0416742	0.040364136	0.00131005	0.024922031	0.24858255	0.302401682	6	0	0
XKR6	0.0578271	0.046376561	0.011450552	0.339473841	0.248584434	0.302401682	15	0	0
SRRM5	0.0440439	0.042420396	0.001623547	0.077832682	0.24856675	0.302401682	12	0	0
NUDT6	0.0871585	0.088522581	-0.00136403	-0.03863258	0.248676452	0.302493106	16	0	0
SUN2	0.0468554	0.045267941	0.001587469	0.032456055	0.248930449	0.302761004	9	0	0
DNAL4	0.0468554	0.045267941	0.001587469	0.032456055	0.248930449	0.302761004	9	0	0
CEP290	0.0524366	0.055347564	-0.00291101	-0.05713057	0.249025101	0.302855588	13	0	0
RPS6KB2	0.2114658	0.213425344	-0.00195957	0.016163231	0.249218426	0.303070153	12	0	0
GPATCH1	0.1933779	0.194044432	-0.0006665	0.018261583	0.249243995	0.303080698	8	0	0
CAPZA1	0.0605113	0.058915573	0.001595708	0.024057119	0.249295593	0.30312289	15	0	0
GORAB	0.1859753	0.187880496	-0.00190523	0.018998469	0.249322002	0.303134452	8	0	0
MED20	0.0492573	0.046024399	0.003232941	0.059832698	0.249488807	0.303295504	15	0	0
SUPT6H	0.0448756	0.04629151	-0.00141586	-0.0287782	0.249473157	0.303295504	12	0	0
CBX7	0.1635877	0.167748965	-0.0041613	0.092744624	0.249505194	0.303295504	6	0	0
ITGA3	0.1171821	0.12405075	-0.0068686	0.127655292	0.249600561	0.303390869	7	0	0
HPS6	0.2304798	0.229342761	0.001136994	-0.00623871	0.249690294	0.303479374	7	0	0
P2RX4	0.3260488	0.328830879	-0.00278209	-0.01738249	0.249732718	0.303510369	9	0	0
NAT8	0.4471692	0.418981937	0.028187247	0.091811259	0.249826664	0.303603974	1	0	0
AACS	0.0389297	0.03615182	0.002777905	0.058340595	0.249849865	0.303611597	10	0	0
ZNF316	0.7500771	0.767081084	-0.017004	-0.0299069	0.249947809	0.303695845	4	0	0
ASPHD2	0.0510622	0.051219811	-0.0001576	0.024634427	0.249953059	0.303695845	6	0	0
AFTPH	0.0432681	0.042067929	0.001200212	0.025893379	0.250097016	0.303837514	8	0	0
ATRN	0.1451147	0.144442571	0.000672113	0.068054974	0.250103538	0.303837514	8	0	0
BCL2L1	0.0566096	0.05492983	0.001679738	0.036529951	0.250142571	0.303864352	3	0	0
ABHD13	0.0418405	0.042625326	-0.00078484	-0.01315216	0.250241524	0.303958783	9	0	0
WFDC2	0.3510409	0.359866817	-0.00882596	0.041025052	0.250254201	0.303958783	12	0	0
CCDC107	0.0953601	0.090943238	0.004416895	0.080091346	0.25029071	0.303982541	7	0	0
COX5B	0.0408039	0.042008635	-0.00120473	-0.04000464	0.250322169	0.303987918	11	0	0
IRGQ	0.0444715	0.045324197	-0.00085272	-0.01686189	0.250329034	0.303987918	9	0	0
IRF9	0.5207275	0.521177198	-0.00044969	-0.01074413	0.250580165	0.30427228	12	0	0
SEC61B	0.0562399	0.059856133	-0.0036162	-0.03524804	0.2506021	0.304278315	11	0	0
HEXA	0.1438472	0.142426036	0.001421176	0.029670276	0.250674115	0.304345152	6	0	0
CTSO	0.22224	0.221248121	0.000991929	0.004162568	0.250776241	0.304448535	9	0	0
GSK3A	0.0437299	0.046392733	-0.00266278	-0.04550123	0.250811082	0.304470225	11	0	0
COX6B1	0.1055708	0.112306683	-0.00673591	0.007504043	0.25088189	0.304514961	10	0	0
SAMM50	0.0876009	0.08861917	-0.00101825	0.125776197	0.250871906	0.304514961	13	0	0

RNF121	0.0333509	0.032373164	0.000977725	0.032363984	0.250959996	0.304589152	13	0	0
RPL8	0.1089057	0.108252744	0.000652961	0.054258508	0.250995004	0.304600691	12	0	0
ALG2	0.0772118	0.082333528	-0.00512168	-0.03525816	0.251003468	0.304600691	10	0	0
WDHD1	0.0535295	0.052578255	0.000951262	0.003867386	0.251172834	0.30476481	8	0	0
CCP110	0.2119945	0.224535704	-0.01254125	-0.01157584	0.251189685	0.30476481	8	0	0
ZNF337	0.1192104	0.128796435	-0.00958606	-0.01981461	0.251186517	0.30476481	9	0	0
IRF1	0.1076125	0.116679438	-0.00906691	-0.00686367	0.251225113	0.304787178	8	0	0
SCRN2	0.1645766	0.17267923	-0.00810262	-0.03258413	0.251250532	0.304797399	11	0	0
CHORDC1	0.1284466	0.13617512	-0.00772856	-0.05230407	0.251292078	0.304827182	13	0	0
OR8S1	0.4095302	0.430393381	-0.02086314	-0.07001798	0.251420493	0.30496233	1	0	0
STPG2	0.1976057	0.203818814	-0.0062131	0.079226657	0.251868173	0.305484687	9	0	0
E2F3	0.0523328	0.054173442	-0.00184063	-0.03954231	0.252020359	0.3056486	14	0	0
ACVR2B	0.1593749	0.156855345	0.002519594	0.044512462	0.252051072	0.305665181	6	0	0
RALY	0.0437943	0.042223275	0.001570992	0.037620322	0.252125658	0.305734961	5	0	0
WDR61	0.1868335	0.195359466	-0.00852593	-0.03716063	0.252314277	0.305943001	5	0	0
BRMS1L	0.0384379	0.036632466	0.001805455	0.050081917	0.252380555	0.306002679	7	0	0
BCL7A	0.9090091	0.912686561	-0.00367743	-0.00575127	0.252421679	0.306031853	3	0	0
MAPRE3	0.1243551	0.1239499	0.000405164	0.015935168	0.252843765	0.306522864	6	0	0
GATAD2A	0.1097731	0.099817577	0.009955528	0.151480011	0.252909889	0.306582304	9	0	0
FOXD4L5	0.9575386	0.959522913	-0.00198427	-0.00295848	0.253024793	0.306700865	2	0	0
FAM43A	0.1971437	0.194882708	0.00226101	0.104126515	0.253180273	0.306868589	7	0	0
TCF12	0.1769742	0.179528748	-0.00255459	0.024148216	0.253263649	0.306928163	9	0	0
SLC20A2	0.1095757	0.109870211	-0.00029451	-0.0042628	0.253283582	0.30693158	11	0	0
CALY	0.0507998	0.039173452	0.011626313	0.398198417	0.253416261	0.307071616	6	0	0
AP3M1	0.0525452	0.050001335	0.002543818	0.052375035	0.253723011	0.307422544	14	0	0
SDCBP	0.0735138	0.07484217	-0.0013284	0.098399391	0.253932772	0.307655918	15	0	0
DUSP12	0.0336104	0.033974516	-0.00036407	-0.00266647	0.254206753	0.307925463	9	0	0
MTMR9	0.1488462	0.134095656	0.014750586	0.183016541	0.254191335	0.307925463	8	0	0
PREX1	0.1887604	0.17648921	0.012271218	0.269283744	0.254201151	0.307925463	8	0	0
ZFAND4	0.1899575	0.189949366	8.09E-06	0.044057898	0.254280235	0.307993672	16	0	0
PRELID1	0.1021262	0.108749688	-0.0066235	-0.00185573	0.254330289	0.308033498	9	0	0
ATP13A1	0.1117568	0.115485914	-0.00372915	0.014714704	0.254641545	0.308368831	12	0	0
DPM1	0.2099503	0.222682532	-0.01273228	-0.00302657	0.254714752	0.308436659	13	0	0
EIF4A2	0.0511732	0.051816369	-0.00064313	-0.02665607	0.254865924	0.30859888	8	0	0
BTBD6	0.3311823	0.340008474	-0.00882619	-0.04743737	0.25495056	0.308680522	15	0	0
ANKRD36B	0.0383148	0.040807518	-0.00249276	-0.07257807	0.254998905	0.30870898	1	0	0
USP45	0.0373836	0.036056668	0.001326969	0.0409789	0.255008488	0.30870898	1	0	0
AANAT	0.2074045	0.21098033	-0.00357587	-0.01242347	0.255032215	0.308716867	11	0	0
SRSF11	0.0888373	0.085100499	0.003736825	0.029944663	0.255088593	0.308764274	18	0	0
MRS2	0.2624618	0.263922478	-0.00146072	-0.02083573	0.255227536	0.308911605	12	0	0
TFB1M	0.0743249	0.081075278	-0.00675039	0.002623774	0.255280602	0.308954986	11	0	0
PFN4	0.1146505	0.120425145	-0.0057746	-0.04420471	0.255444603	0.309111937	7	0	0
RHEB	0.095318	0.087663394	0.007654654	0.106876708	0.255444756	0.309111937	13	0	0
MORC3	0.0722334	0.08260661	-0.01037322	-0.02958477	0.255622785	0.309306501	10	0	0
RUFY3	0.0510774	0.051293632	-0.00021625	-0.00293309	0.255668472	0.309340914	5	0	0
DIABLO	0.805516	0.806351355	-0.0008354	-0.00153048	0.255724748	0.309388134	10	0	0
SYNCRIP	0.0776507	0.071689606	0.005961126	0.062775534	0.255823648	0.309486912	5	0	0
NAA25	0.084978	0.086464648	-0.00148667	0.005765544	0.255874588	0.30952766	10	0	0
MPC2	0.0400529	0.040137628	-8.47E-05	-0.00894869	0.256155452	0.30984652	11	0	0
PKD1L2	0.6729305	0.671928841	0.001001644	0.007640276	0.256233365	0.309919862	6	0	0
MYSM1	0.485861	0.494505532	-0.0086445	-0.00823544	0.256284181	0.309960423	9	0	0
PHPT1	0.0577725	0.053586437	0.004186095	0.048094833	0.256334454	0.310000322	9	0	0
KCNK13	0.0896927	0.082284929	0.007407782	0.176314808	0.256396633	0.310054613	13	0	0
LAMTOR2	0.0413311	0.039581145	0.001749992	0.041789737	0.256444213	0.310091242	11	0	0
BAG1	0.1302267	0.12896855	0.001258136	0.027718868	0.256513776	0.310154448	11	0	0
CCDC180	0.2538503	0.265175352	-0.01132506	0.025328901	0.2565919	0.310227995	6	0	0
PNISR	0.0419479	0.041196583	0.000751347	0.023484612	0.256705064	0.310343895	15	0	0
GPR21	0.5982758	0.611712018	-0.0134362	-0.02645061	0.25683899	0.310484878	12	0	0
TNPO1	0.071002	0.073225256	-0.00222328	-0.00884358	0.2568797	0.310513162	12	0	0
DEPTOR	0.157048	0.169091403	-0.01204338	-0.03358671	0.256960232	0.310554688	11	0	0
ZFHX3	0.4126721	0.431686708	-0.01901464	-0.04313033	0.256965997	0.310554688	7	0	0
EFCAB6	0.0619645	0.051746582	0.010217939	0.320410676	0.256949329	0.310554688	12	0	0
UFSP1	0.0469732	0.047069614	-9.64E-05	0.017300214	0.257177069	0.310746962	8	0	0

IFT74	0.0730768	0.074446197	-0.00136937	0.014570181	0.257162799	0.310746962	14	0	0
CCDC78	0.0763904	0.078565115	-0.00217476	-0.01330679	0.257147426	0.310746962	13	0	0
BIRC6	0.1746761	0.177762778	-0.00308663	-0.00865049	0.25730559	0.310881311	6	0	0
PBRM1	0.1551767	0.151114919	0.004061792	0.022868956	0.257354464	0.310919417	4	0	0
TMEM115	0.1755766	0.176629612	-0.00105302	0.006505525	0.257465391	0.311032481	7	0	0
YRDC	0.0935562	0.087178098	0.006378121	0.055157517	0.257735007	0.311337223	9	0	0
NDRG1	0.4151488	0.436004663	-0.02085585	-0.06910144	0.257761716	0.311348518	2	0	0
MORN5	0.0576766	0.051541301	0.006135347	0.046448359	0.257849294	0.311428586	9	0	0
FDX1	0.2594219	0.259103379	0.000318475	0.043863955	0.25786273	0.311428586	9	0	0
KCTD14	0.1642247	0.169477385	-0.00525269	0.052277047	0.258056904	0.311642111	6	0	0
FRS3	0.0903377	0.091649944	-0.00131229	-0.02772253	0.258142889	0.311703976	13	0	0
TOMM6	0.0903377	0.091649944	-0.00131229	-0.02772253	0.258142889	0.311703976	13	0	0
ADH1A	0.6717159	0.702905266	-0.03118941	-0.06640093	0.25829545	0.311867195	2	0	0
TSN	0.0994073	0.09797027	0.001437046	-0.02038922	0.258472826	0.312039349	4	0	0
TMEM87A	0.0489634	0.047974795	0.000988642	0.021136928	0.258458688	0.312039349	17	0	0
UTP15	0.0367017	0.038094271	-0.00139258	-0.04170012	0.258494693	0.312044745	13	0	0
SRPK1	0.3272802	0.348161952	-0.02088176	-0.02834966	0.258556222	0.312098015	5	0	0
SLC34A3	0.6254577	0.639371168	-0.01391344	-0.03103387	0.258708254	0.312260514	7	0	0
STRIP2	0.1588394	0.162082559	-0.00324312	0.0209309	0.258779897	0.31232597	10	0	0
SNRPA1	0.075206	0.078588852	-0.00338288	-0.05248968	0.259059157	0.312620941	8	0	0
ZNF830	0.123838	0.127924157	-0.00408618	-0.01747976	0.259057741	0.312620941	14	0	0
RNF152	0.0678228	0.067823497	-6.99E-07	0.089194381	0.259132919	0.312688915	6	0	0
ANKAR	0.3032425	0.301227672	0.002014814	0.260704324	0.259255922	0.312810182	7	0	0
KCTD21	0.0830259	0.072464343	0.01056152	0.041938679	0.259268296	0.312810182	8	0	0
ZNF76	0.0902761	0.096151916	-0.00587583	-0.04652497	0.259573378	0.313157201	2	0	0
ACOT8	0.2041329	0.201571658	0.002561269	0.091081925	0.259604244	0.313173374	12	0	0
PPP1R12A	0.0877331	0.083364286	0.004368833	0.052287371	0.259631524	0.313185219	7	0	0
GLT8D2	0.0595934	0.051948349	0.007645028	0.098640967	0.259701161	0.313248152	8	0	0
LYRM9	0.5128629	0.511354758	0.001508116	0.01085637	0.259724089	0.313254742	3	0	0
SLX4IP	0.0425377	0.038449413	0.004088276	0.120436257	0.259826158	0.313356776	11	0	0
ITGB1	0.1505845	0.132026756	0.018557793	0.246191031	0.259964871	0.313502987	6	0	0
COPS5	0.8740456	0.870865756	0.003179856	0.005441659	0.260224077	0.313794476	2	0	0
C1orf43	0.0534087	0.051042042	0.002366636	0.038253329	0.260504947	0.314090934	11	0	0
CEP57	0.0303454	0.031545714	-0.00120033	-0.04212278	0.26053839	0.314110141	8	0	0
CHMP5	0.0520778	0.050733239	0.001344605	0.025330529	0.260943903	0.31457789	11	0	0
USP46	0.1111675	0.107482856	0.003684678	0.029974363	0.261087753	0.314730153	8	0	0
THAP10	0.0912815	0.092691649	-0.00141011	0.036133085	0.261131008	0.314761139	16	0	0
KATNAL1	0.15438	0.153106301	0.001273725	0.028275314	0.261198975	0.314821908	11	0	0
DZIP3	0.0482268	0.049077568	-0.00085076	-0.02067022	0.261247663	0.314859433	17	0	0
RNF139	0.0635172	0.06894909	-0.00543191	-0.01142598	0.261538976	0.315189349	9	0	0
UBL7	0.0311136	0.031262982	-0.00014933	0.007108168	0.261918001	0.315624917	15	0	0
VPS26B	0.061113	0.063521549	-0.00240856	-0.03733843	0.261969497	0.315665764	11	0	0
RNF115	0.0753386	0.078774	-0.00343537	-0.00302237	0.262253816	0.315987132	17	0	0
CRIPT	0.05885	0.063036221	-0.00418625	-0.0405658	0.262293148	0.316013293	6	0	0
VEGFB	0.3280448	0.327140017	0.000904818	0.011909712	0.262405319	0.316127203	11	0	0
TAF5L	0.0441703	0.044052645	0.000117633	0.000381828	0.262641484	0.316390466	9	0	0
MAX	0.0418271	0.041748876	7.82E-05	0.018584442	0.262670628	0.316404323	15	0	0
GTF3C5	0.1041446	0.110085128	-0.00594052	-0.05098904	0.262901432	0.316661076	11	0	0
TEFM	0.0481703	0.044146212	0.00402406	0.126029463	0.262950255	0.316698615	5	0	0
USP36	0.3258686	0.335227624	-0.00935901	-0.01373064	0.262989741	0.316724904	6	0	0
HCFC2	0.1502154	0.145306983	0.004908456	0.001072304	0.263191156	0.316946191	6	0	0
PEX13	0.0438917	0.04298588	0.000905773	0.026895775	0.263213993	0.316952412	10	0	0
IKZF4	0.0462499	0.04379656	0.002453388	0.055950072	0.263240094	0.316962562	4	0	0
MFAP3	0.1939209	0.197372072	-0.00345116	-0.02302025	0.263312815	0.317028842	16	0	0
TMED7-TIC/	0.0394813	0.039626852	-0.0001456	-0.00273653	0.263431083	0.31712866	8	0	0
TICAM2	0.0394813	0.039626852	-0.0001456	-0.00273653	0.263431083	0.31712866	8	0	0
DNAJC13	0.4566101	0.477107328	-0.02049723	-0.05690164	0.263593462	0.317302841	3	0	0
UBQLN4	0.0388778	0.037646976	0.001230828	0.03212449	0.263657775	0.31735896	10	0	0
R3HDM1	0.0546284	0.052828195	0.001800242	0.040958606	0.263908688	0.317639661	15	0	0
ADORA2A	0.7561836	0.769380689	-0.01319704	-0.02899588	0.263979325	0.31770336	2	0	0
MED30	0.0592282	0.051861971	0.007366222	0.251706738	0.264002305	0.317709698	9	0	0
C11orf1	0.1097923	0.11235577	-0.00256347	0.019055799	0.264087405	0.317790788	15	0	0
NFU1	0.2324757	0.230828852	0.001646831	0.044267861	0.264131606	0.317822654	11	0	0

CKAP2L	0.0452607	0.046003916	-0.00074317	-0.02442477	0.264172144	0.317833471	9	0	0
PSMD8	0.2677785	0.271633015	-0.00385448	-0.01994316	0.264176037	0.317833471	11	0	0
RTN1	0.1146477	0.110833952	0.003813755	0.069881401	0.264273994	0.317929999	11	0	0
IPO9	0.1374185	0.142892177	-0.00547367	-0.01229279	0.264348338	0.317997672	10	0	0
OSBPL8	0.0398367	0.040557543	-0.00072087	-0.01883562	0.264365705	0.317997672	7	0	0
UGP2	0.0689573	0.07436016	-0.00540284	0.010993909	0.264443555	0.318069984	9	0	0
TAF2	0.2005825	0.206039437	-0.00545696	0.003975201	0.264517576	0.318137681	9	0	0
ACCS	0.2191217	0.22076083	-0.00163912	0.113611791	0.264712661	0.318350964	11	0	0
YWHAQ	0.1946886	0.203392973	-0.00870442	-0.00353515	0.264856789	0.318502941	7	0	0
ADNP	0.2056417	0.200578385	0.005063272	0.060031399	0.264971098	0.318619041	10	0	0
IFT27	0.1713263	0.172308936	-0.0009826	0.067889828	0.265035328	0.318674911	13	0	0
PKM	0.0893946	0.089062675	0.000331893	-0.00187644	0.265088194	0.318717109	5	0	0
ZNF57	0.1458169	0.148532547	-0.00271567	0.060445031	0.265140772	0.318758958	7	0	0
TMEM207	0.8068825	0.833397998	-0.02651553	-0.04553626	0.265429908	0.319085177	2	0	0
L3HYPDH	0.0433839	0.04455945	-0.00117559	-0.02224433	0.265473017	0.319115611	10	0	0
EXPH5	0.1275253	0.129354141	-0.00182887	0.022777263	0.265554988	0.319192753	9	0	0
PHKB	0.1184886	0.121758399	-0.00326983	0.004553317	0.265821168	0.319491286	10	0	0
ARPC5	0.0549418	0.054566284	0.000375554	-0.01182829	0.266018562	0.319707111	14	0	0
FER1L6	0.9214399	0.926751006	-0.00531109	-0.00818413	0.26611812	0.319805333	2	0	0
ANAPC2	0.0897288	0.091989735	-0.00226098	0.026399941	0.266288796	0.319989002	11	0	0
DLL1	0.0532264	0.056121425	-0.00289506	-0.0279403	0.266491022	0.320210556	12	0	0
PCDH1	0.048834	0.045784351	0.003049694	0.056930497	0.266527747	0.32023323	4	0	0
PRDX2	0.0863821	0.072171152	0.014210994	0.390917116	0.266701286	0.320420273	14	0	0
CHPT1	0.3293559	0.32398884	0.005367105	0.118510172	0.26685905	0.32058834	6	0	0
POU2F1	0.0401634	0.040551878	-0.00038848	-0.00440556	0.267001066	0.320737467	10	0	0
COL4A3	0.0850515	0.082448145	0.002603402	0.083125738	0.267116959	0.320812289	14	0	0
DTX3L	0.2215945	0.218252943	0.003341562	0.011117009	0.267092591	0.320812289	10	0	0
DLX3	0.1597983	0.15426235	0.005535992	0.101094882	0.267117011	0.320812289	11	0	0
CSRNP3	0.690644	0.703124509	-0.01248056	-0.02759915	0.267244712	0.320944168	6	0	0
OMG	0.8131426	0.820732681	-0.00759005	-0.01306227	0.267288818	0.320975646	6	0	0
SLC12A8	0.8403722	0.840154348	0.000217842	0.000475703	0.267434915	0.321129586	3	0	0
RBM19	0.1442633	0.138729895	0.005533374	0.063485676	0.267583529	0.32128653	7	0	0
FBXL13	0.0478169	0.046846501	0.000970433	0.022247051	0.26775337	0.321468936	14	0	0
C6orf226	0.088409	0.088065438	0.000343583	-0.00521473	0.267904697	0.321629092	11	0	0
SLC19A2	0.0683804	0.071799126	-0.00341874	-0.01178307	0.267943857	0.321654577	14	0	0
KISS1R	0.3409217	0.340306686	0.000614985	0.166503155	0.268099408	0.321819769	7	0	0
TSC22D4	0.1352807	0.137488311	-0.00220758	0.004412697	0.26826393	0.321995707	9	0	0
CASC10	0.1278955	0.129939002	-0.0020435	0.000835457	0.268391478	0.322127245	16	0	0
DCTN1	0.0486672	0.048399852	0.000267341	-0.00387053	0.268602486	0.322358928	6	0	0
COG3	0.2193581	0.227989259	-0.00863115	-0.00178791	0.268760618	0.322510615	6	0	0
HIGD1B	0.6744605	0.668155968	0.006304533	0.020030676	0.26876484	0.322510615	2	0	0
THAP6	0.0440755	0.04247887	0.001596678	0.036474716	0.26881464	0.322548794	8	0	0
TLR10	0.3672676	0.367003384	0.000264218	0.094044768	0.269099533	0.322869036	5	0	0
RAB1B	0.071472	0.057655405	0.013816608	0.298459381	0.269135368	0.322890432	40	0	0
SRGAP2	0.7911256	0.800545938	-0.00942036	-0.01704761	0.26931161	0.323080265	2	0	0
TRIM39	0.1420473	0.149826308	-0.00777905	-0.03041485	0.269473464	0.323252812	19	0	0
SOX6	0.5180888	0.54429705	-0.02620829	-0.06597491	0.269538356	0.323289142	4	0	0
LRRC45	0.0261474	0.026620995	-0.00047357	-0.02529609	0.269539799	0.323289142	10	0	0
CEBPZ	0.0431957	0.042414717	0.000780958	0.003156163	0.269570119	0.323303889	12	0	0
CEP120	0.1292704	0.13031126	-0.00104083	0.022647145	0.26963636	0.323361711	9	0	0
CCDC51	0.0301832	0.02994952	0.000233695	0.006729358	0.269697151	0.32341299	14	0	0
KIAA1328	0.0441071	0.046090898	-0.00198379	-0.04507504	0.269786464	0.323498464	10	0	0
ANAPC1	0.0295401	0.0299795	-0.00043936	-0.01469917	0.270086699	0.323833839	4	0	0
TMEM135	0.2284007	0.236193778	-0.00779312	-0.01437447	0.270120321	0.323833839	10	0	0
HPS4	0.2509268	0.268113574	-0.01718677	-0.00308635	0.270110999	0.323833839	5	0	0
ATF1	0.0814779	0.087585996	-0.00610812	-0.01651662	0.270143542	0.323840033	10	0	0
PTCD3	0.1024337	0.10956342	-0.00712967	-0.02025419	0.270192815	0.323877453	12	0	0
NEDD4L	0.0946985	0.083742162	0.010956384	0.108896538	0.270218308	0.323886365	4	0	0
GSTM2	0.2128274	0.19664482	0.01618259	0.265009665	0.270296594	0.323958551	10	0	0
EIF2A	0.0353184	0.035786839	-0.00046845	-0.01992718	0.270445422	0.324115267	11	0	0
HIST2H2AC	0.0394192	0.039436037	-1.68E-05	0.009432699	0.270544713	0.324212599	13	0	0
CHTF8	0.1264305	0.136247722	-0.00981727	-0.02290669	0.270787392	0.324481739	11	0	0
MDN1	0.1133548	0.115597253	-0.00224244	0.004530626	0.271104672	0.32481853	11	0	0

HLA-DMA	0.0405726	0.040442078	0.000130535	0.009935234	0.27118908	0.324897958	12	0	0
SMPD2	0.2390083	0.230900791	0.008107539	0.022369424	0.271224821	0.324912161	7	0	0
RTN4RL1	0.0900843	0.085065424	0.005018887	0.074564112	0.271237165	0.324912161	10	0	0
TMCO4	0.1458975	0.146349441	-0.00045194	0.03645792	0.271397247	0.32508221	8	0	0
RTTN	0.1023838	0.106212161	-0.0038284	-0.01465803	0.271576746	0.325275494	14	0	0
SEC16A	0.8127445	0.82931861	-0.0165741	-0.03012791	0.271656145	0.325348866	3	0	0
FLOT1	0.0592958	0.051158267	0.008137545	0.194265192	0.271853849	0.325563907	34	0	0
EXOSC3	0.0240093	0.023984072	2.52E-05	0.006654755	0.271889205	0.325584509	2	0	0
TTC21B	0.1553691	0.145777831	0.009591289	0.039243218	0.272031241	0.325732848	10	0	0
PAXIP1	0.0398569	0.041410603	-0.00155366	-0.04015576	0.272386092	0.326135977	6	0	0
ARHGAP22	0.0568784	0.060789639	-0.00391124	-0.04801567	0.272577461	0.326322602	3	0	0
SF1	0.0367549	0.034558448	0.002196403	0.050950355	0.272578346	0.326322602	11	0	0
ZNF423	0.0536876	0.051810923	0.001876627	0.0249963	0.272730696	0.326483198	11	0	0
MAU2	0.0607008	0.057814791	0.002886026	0.077236205	0.273211625	0.327037086	11	0	0
PRKAB2	0.0700581	0.070771185	-0.00071307	-0.00211452	0.273390714	0.327229619	10	0	0
NUS1	0.2461234	0.258732656	-0.01260926	-0.00789895	0.273497389	0.327335458	7	0	0
EEF1A1	0.0498091	0.04759927	0.002209833	0.077989786	0.273724444	0.327585349	5	0	0
C17orf80	0.0385091	0.038489295	1.98E-05	-3.88E-05	0.27379341	0.327646024	10	0	0
NR2F1	0.1091401	0.10924084	-0.00010073	0.031319204	0.27423262	0.32813199	11	0	0
FKBP5	0.2858351	0.293446266	-0.0076112	-0.01514886	0.274254385	0.32813199	6	0	0
SMARCD1	0.0422141	0.040502244	0.001711886	0.031205102	0.274236382	0.32813199	10	0	0
YBX3	0.0236254	0.023054976	0.000570376	0.024262735	0.274431656	0.328322185	4	0	0
KIF1B	0.0308446	0.03016107	0.000683488	0.033446156	0.274592323	0.328492491	6	0	0
SPOPL	0.1482384	0.152144265	-0.00390588	-0.00800382	0.27463529	0.328521979	14	0	0
HTRA2	0.1385446	0.140294645	-0.00175001	-0.01504839	0.274734559	0.32861881	9	0	0
UQCC2	0.1439352	0.145687149	-0.00175194	-0.03795703	0.274772673	0.328642482	8	0	0
EMC6	0.0645706	0.060260428	0.004310172	0.054956221	0.275005032	0.328898464	12	0	0
NOP9	0.0373201	0.036549594	0.000770468	0.023097342	0.275185999	0.32909295	7	0	0
LPXN	0.0305296	0.031102135	-0.00057258	-0.00943304	0.275207739	0.329097007	8	0	0
BCLAF1	0.0433812	0.04345558	-7.44E-05	0.008357374	0.275273009	0.329109232	10	0	0
ARF5	0.2001594	0.206015294	-0.00585589	-0.01505906	0.275261523	0.329109232	8	0	0
HSP90B1	0.0539473	0.054216036	-0.00026876	0.017428226	0.275259474	0.329109232	11	0	0
FZD3	0.0420076	0.041646824	0.00036073	0.021166892	0.275318178	0.329141294	9	0	0
PPP6R3	0.0247425	0.024697883	4.46E-05	-0.00140454	0.27537486	0.329187116	6	0	0
DNMT3B	0.251795	0.242523016	0.009272015	0.054755265	0.275412803	0.329210532	5	0	0
LEMD3	0.039538	0.036779885	0.002758073	0.086370493	0.275449254	0.329232161	8	0	0
CLP1	0.2167672	0.216780421	-1.32E-05	0.001304846	0.275505869	0.329255948	10	0	0
NIPA2	0.1002613	0.11558203	-0.01532068	-0.02891621	0.275495253	0.329255948	11	0	0
IPMK	0.055436	0.053382419	0.002053625	0.035060206	0.275784249	0.329544723	13	0	0
CISD1	0.055436	0.053382419	0.002053625	0.035060206	0.275784249	0.329544723	13	0	0
PPP1R37	0.3126246	0.330197808	-0.01757323	0.003029181	0.275877556	0.329634257	2	0	0
C9orf78	0.2288007	0.234973174	-0.00617248	-0.03008287	0.275949293	0.329698009	4	0	0
GINM1	0.0453403	0.044593114	0.000747214	0.030440317	0.275985152	0.32971889	4	0	0
MAN1C1	0.0466704	0.040818046	0.005852316	0.167604132	0.27621447	0.329970876	11	0	0
TYW1	0.1317209	0.124228289	0.007492648	0.047948551	0.276289207	0.330038176	3	0	0
CLHC1	0.0480199	0.045864041	0.002155863	0.055374216	0.276504947	0.33027389	15	0	0
ADSL	0.0374418	0.038130722	-0.00068896	-0.01059859	0.276648318	0.330423136	7	0	0
CEP170B	0.1188482	0.117173898	0.001674275	0.01109263	0.276751427	0.330524278	6	0	0
PIFO	0.1318044	0.134899826	-0.00309545	-0.01345326	0.276869874	0.330643722	13	0	0
UCHL5	0.0755339	0.074782739	0.000751131	0.005991141	0.276910481	0.330648185	12	0	0
CCDC148	0.0939187	0.08938458	0.004534089	0.035032627	0.276905717	0.330648185	9	0	0
ZBTB32	0.4771304	0.457110296	0.020020138	0.075977653	0.277025384	0.330763366	4	0	0
C12orf4	0.0663902	0.066841177	-0.00045099	-0.02849394	0.277202473	0.330952776	13	0	0
CUX1	0.0434344	0.042561348	0.000873095	0.000688506	0.277373959	0.331135472	11	0	0
HAUS8	0.045976	0.044876126	0.001099922	0.034021091	0.277569963	0.331347412	8	0	0
C5orf30	0.3820066	0.386324147	-0.00431751	-0.0144092	0.277957617	0.331788089	7	0	0
SLC16A7	0.0552707	0.050396984	0.004873746	0.160822378	0.278380388	0.332270622	11	0	0
CDH22	0.182438	0.170247947	0.012190018	0.155280176	0.27843827	0.332317594	4	0	0
SAP30BP	0.1933524	0.195347038	-0.0019946	0.022820411	0.278538932	0.332415616	10	0	0
EHMT2	0.0885451	0.089891595	-0.0013465	0.015014522	0.278685998	0.332568999	31	0	0
EIF4G2	0.0770085	0.077633508	-0.00062501	0.023741997	0.278779615	0.332658584	9	0	0
AGK	0.1593357	0.153093383	0.006242287	0.054736402	0.278828391	0.332694653	8	0	0
CDC20	0.1457411	0.149293894	-0.00355279	0.001539215	0.278953197	0.332821428	7	0	0

SAFB	0.0416977	0.042410433	-0.0007127	-0.01876393	0.278972581	0.332822416	10	0	0
KDM5B	0.3910755	0.391242535	-0.000167	-0.00334684	0.279258166	0.333140969	9	0	0
MAB21L1	0.3838837	0.381144676	0.002739037	0.000393268	0.279443629	0.333340046	15	0	0
EGR1	0.0401386	0.041236855	-0.00109829	-0.02871776	0.279476597	0.333357202	9	0	0
LYAR	0.0975987	0.097431188	0.000167491	0.003704504	0.279538978	0.333409437	9	0	0
C12orf71	0.7044385	0.683724787	0.020713756	0.050006299	0.279568895	0.333422947	3	0	0
CASC1	0.1427408	0.148884203	-0.00614342	-0.02411841	0.279770571	0.333641287	11	0	0
C2orf76	0.045247	0.047836297	-0.00258925	-0.02427088	0.280091832	0.3340022	20	0	0
DPYD	0.1316692	0.137533805	-0.00586459	0.002050638	0.280135322	0.334030758	11	0	0
COPB2	0.0388185	0.039298989	-0.00048052	-0.02010331	0.280153393	0.334030758	9	0	0
NFKBIB	0.0800121	0.074985862	0.005026198	0.103488508	0.280171651	0.334030758	19	0	0
FCER1G	0.7474294	0.768847802	-0.02141837	-0.03947986	0.280336081	0.334182371	7	0	0
RPL14	0.1200282	0.123014724	-0.0029865	-0.01968517	0.280329083	0.334182371	9	0	0
BMP2K	0.0371698	0.038938266	-0.00176844	-0.03362674	0.280759404	0.334664762	7	0	0
MKKS	0.0471378	0.042945337	0.004192457	0.116509659	0.280811066	0.334704099	12	0	0
POLE	0.029053	0.027647585	0.001405454	0.03556339	0.280859858	0.334717768	8	0	0
PGBD4	0.0552818	0.054326568	0.000955199	0.03885992	0.280846537	0.334717768	9	0	0
GOLGA3	0.1970068	0.195580346	0.001426444	-0.01237642	0.280888252	0.334729366	7	0	0
MCEE	0.0761228	0.07998239	-0.00385962	-0.01957211	0.281017273	0.334860869	12	0	0
CDC123	0.0590789	0.056486382	0.002592497	0.040026727	0.281053926	0.334882297	15	0	0
DNAJB5	0.09775	0.102471062	-0.00472107	-0.01737839	0.281132434	0.33495359	11	0	0
OSGEPL1	0.2447736	0.246468595	-0.00169501	0.017475501	0.281398249	0.335248024	12	0	0
ZNF397	0.1491736	0.144495011	0.0046786	0.041343443	0.281472624	0.335292089	14	0	0
IER3IP1	0.1340295	0.138504317	-0.00447486	-0.0240699	0.281459878	0.335292089	8	0	0
SLC39A8	0.5382259	0.545624182	-0.00739833	0.005168745	0.281618586	0.335443682	3	0	0
TPD52L2	0.0417344	0.041105926	0.000628429	0.016113443	0.281748207	0.335553508	8	0	0
CEP44	0.0600862	0.06136124	-0.00127508	-0.01764426	0.281784466	0.335574409	17	0	0
TMEM254	0.2294379	0.232553504	-0.00311557	0.00485069	0.281988298	0.335794856	11	0	0
TSPYL4	0.3572372	0.367508006	-0.01027077	-0.04359244	0.282116845	0.335925628	6	0	0
DCLRE1A	0.040624	0.04091282	-0.00028882	0.002821094	0.282187254	0.33598716	21	0	0
PSMB3	0.0348597	0.035946051	-0.0010863	-0.0254947	0.282302522	0.336102093	11	0	0
KCNJ2	0.1580822	0.158000108	8.21E-05	0.096635872	0.282361985	0.336150575	8	0	0
ENTPD3	0.316818	0.304107235	0.012710757	0.13305465	0.282604773	0.336417284	10	0	0
ECHDC1	0.1391403	0.130033842	0.009106452	0.159458081	0.282630606	0.336425706	11	0	0
MANEAL	0.2722842	0.269254501	0.003029744	0.026052929	0.282966815	0.336803557	11	0	0
CXXC4	0.073929	0.067055576	0.006873405	0.104599583	0.283035762	0.336840915	5	0	0
PRR16	0.1873513	0.18972968	-0.00237833	0.134856799	0.283032344	0.336840915	11	0	0
GDPD5	0.1368851	0.146010108	-0.00912505	0.010413343	0.283058311	0.336845399	11	0	0
ARPC1A	0.1475959	0.147646927	-5.10E-05	0.006137993	0.283273095	0.337078632	8	0	0
CPSF7	0.029945	0.029855548	8.95E-05	0.002912409	0.283316968	0.337108474	5	0	0
FOXK1	0.0961988	0.089335124	0.00686366	0.09639141	0.283491676	0.337293977	1	0	0
CHST15	0.1553311	0.151713566	0.003617541	0.027199515	0.283553938	0.337345677	11	0	0
MTCH1	0.0721077	0.079123743	-0.00701602	-0.00548689	0.283696546	0.337448576	9	0	0
KMT2A	0.0551598	0.054400502	0.000759274	0.022296818	0.283696871	0.337448576	11	0	0
SHPK	0.0434806	0.042394701	0.001085863	0.0466788	0.283670536	0.337448576	13	0	0
DPY19L3	0.0996618	0.115421581	-0.01575975	-0.03624043	0.283730366	0.337466038	6	0	0
ZNF438	0.0517337	0.053660646	-0.00192696	-0.02041984	0.283797418	0.337523406	8	0	0
CYHR1	0.1254859	0.133133426	-0.00764753	-0.05300194	0.284076456	0.337832868	10	0	0
MAD2L1BP	0.0798859	0.075038288	0.004847638	0.028587629	0.284107171	0.337846995	17	0	0
RAB3GAP2	0.0382867	0.03748272	0.000803957	0.024238647	0.284276328	0.33800333	8	0	0
TBC1D31	0.0319969	0.030739343	0.001257595	0.020068596	0.284275209	0.33800333	4	0	0
ANXA5	0.0745547	0.07685696	-0.00230225	0.004623076	0.284606582	0.33835114	5	0	0
RANBP3	0.0244355	0.025643638	-0.0012081	-0.02149821	0.284589621	0.33835114	9	0	0
COL8A1	0.0952756	0.083277577	0.011997984	0.173125563	0.284687722	0.338395672	8	0	0
DBR1	0.0311442	0.031886007	-0.00074179	-0.01952725	0.284719507	0.338395672	4	0	0
LRP4	0.2513926	0.246701206	0.004691362	0.073449464	0.284672818	0.338395672	6	0	0
TMIGD2	0.6094104	0.618029211	-0.00861881	-0.01285415	0.284703955	0.338395672	6	0	0
EMC9	0.0499264	0.049247846	0.000678577	0.010923859	0.284909977	0.338573752	12	0	0
C19orf66	0.342066	0.350693336	-0.00862733	-0.00622916	0.284916631	0.338573752	8	0	0
POLR3F	0.1363038	0.139721263	-0.00341748	-0.00283227	0.284925971	0.338573752	11	0	0
FNBP1L	0.0657007	0.06547446	0.000226245	-0.01362799	0.28500165	0.338575519	13	0	0
MDH1B	0.1304653	0.134130882	-0.00366555	-0.03548144	0.284949098	0.338575519	15	0	0
HELB	0.0354851	0.037184271	-0.00169916	-0.04986626	0.284997476	0.338575519	8	0	0

C21orf2	0.2047611	0.207449416	-0.00268829	0.019751384	0.285002965	0.338575519	5	0	0
PCSK7	0.141685	0.143123373	-0.00143841	-0.01237786	0.285067371	0.338629603	11	0	0
MMS22L	0.1520989	0.161655711	-0.00955683	-0.05376216	0.285153279	0.33870922	10	0	0
GTF3C2	0.0576423	0.056650767	0.000991506	-0.00447601	0.285259768	0.338813271	8	0	0
FMO5	0.0315573	0.03157551	-1.82E-05	0.00060615	0.285319561	0.338861849	6	0	0
TMEM179B	0.0978996	0.096020816	0.001878754	0.022763439	0.285550565	0.339091296	8	0	0
MYPOP	0.1115391	0.101103034	0.010436073	0.060174541	0.285789797	0.339352917	7	0	0
MPPE1	0.1011286	0.100339521	0.000789045	0.022070431	0.285890769	0.33945034	7	0	0
LCA5L	0.1540257	0.157494792	-0.00346909	-0.007213	0.286190806	0.339761603	10	0	0
FAM219A	0.034179	0.035924525	-0.00174555	-0.03599677	0.286237824	0.339794931	4	0	0
KLHL41	0.7072774	0.702441645	0.004835718	0.022097789	0.286453766	0.339983771	3	0	0
RAPGEF6	0.0345892	0.034594461	-5.22E-06	0.006171229	0.286433362	0.339983771	13	0	0
MYL6	0.0716837	0.074901347	-0.00321765	0.007424021	0.28644424	0.339983771	14	0	0
SAP130	0.0532839	0.05496698	-0.00168311	-0.02506234	0.286555149	0.340081596	7	0	0
TEX261	0.0466784	0.046315007	0.000363369	0.006165374	0.286584643	0.340094095	10	0	0
ETAA1	0.2294019	0.228097944	0.001303935	-0.00403722	0.286623835	0.340118102	10	0	0
TUBA1C	0.09867	0.092988659	0.005681377	0.038553562	0.286908133	0.340410417	9	0	0
NAALADL2	0.6268	0.602627358	0.024172594	0.055830432	0.28695097	0.340438722	1	0	0
FAM206A	0.0902255	0.093049804	-0.00282428	0.011461162	0.287175522	0.340682595	13	0	0
ROCK1	0.0451302	0.043449948	0.001680252	0.049923073	0.287501983	0.341047325	11	0	0
ZNF706	0.0616019	0.065308307	-0.00370643	-0.01614479	0.287766629	0.341338685	7	0	0
KLRB1	0.8003345	0.810335387	-0.01000088	-0.02045453	0.287806861	0.341363831	2	0	0
TXNL1	0.0450271	0.044523357	0.000503736	-0.00831122	0.287842663	0.34138372	5	0	0
HDAC3	0.0527652	0.050606628	0.002158603	0.049979021	0.287984204	0.341529006	13	0	0
DPY30	0.0449685	0.042377089	0.002591419	0.063144079	0.288155554	0.341694404	11	0	0
TMEM25	0.0607862	0.05620891	0.004577288	0.068125522	0.288161773	0.341694404	10	0	0
MYLPF	0.0476545	0.048492379	-0.00083788	-0.01371259	0.288227193	0.341749385	7	0	0
MAN1A1	0.0362901	0.025645552	0.010644513	0.413331161	0.288269767	0.341754679	6	0	0
HAGH	0.0981508	0.095309993	0.00284082	-0.000484	0.288255124	0.341754679	9	0	0
PRPF4B	0.1094952	0.120803871	-0.01130867	-0.02039459	0.288407587	0.341895471	6	0	0
ENTPD7	0.0911168	0.093425845	-0.002309	-0.00536718	0.288493129	0.341974276	12	0	0
B3GALNT2	0.0323212	0.033086305	-0.00076508	-0.02189837	0.288518398	0.341981628	6	0	0
PDGFB	0.196753	0.192425473	0.004327501	0.047786734	0.288678758	0.342149091	11	0	0
C12orf60	0.0352902	0.035052306	0.000237878	-0.00271257	0.288713891	0.342168121	11	0	0
ZNF703	0.0743755	0.066054645	0.008320854	0.10284069	0.28897853	0.342459128	9	0	0
HEY1	0.109835	0.115131305	-0.00529632	-0.02338622	0.289091814	0.342570743	9	0	0
RPS16	0.1517759	0.141833013	0.009942922	0.047032067	0.289163282	0.342628583	5	0	0
EWSR1	0.1219987	0.122097574	-9.89E-05	0.01119763	0.28917883	0.342628583	12	0	0
STX8	0.1327587	0.12902367	0.003734988	0.060375181	0.289203355	0.342635007	7	0	0
GDF9	0.0864648	0.087806397	-0.00134156	-0.03346899	0.289344876	0.342757394	12	0	0
RNMT	0.0717868	0.077498381	-0.00571162	-0.0315326	0.289327745	0.342757394	7	0	0
KLLN	0.0468825	0.048110007	-0.0012275	-0.01965382	0.289418639	0.342822131	15	0	0
STOML1	0.0504663	0.051247347	-0.00078109	-0.00589182	0.289444351	0.342829946	12	0	0
SEMA4G	0.0703397	0.05571586	0.014623801	0.419981048	0.28947719	0.342846201	4	0	0
TCP11L1	0.0313112	0.031607048	-0.0002958	0.001230452	0.289538379	0.342896029	10	0	0
VPS13C	0.0733669	0.078336854	-0.00496999	-0.04427765	0.289737555	0.343109256	6	0	0
CLTA	0.0606923	0.056429165	0.004263155	0.041506084	0.289794772	0.343154357	9	0	0
PSMD5	0.3200799	0.33162872	-0.01154882	0.066785589	0.290185947	0.343572192	11	0	0
TTC16	0.0410946	0.039255278	0.001839332	0.028638496	0.290169138	0.343572192	9	0	0
ZCWPW1	0.033253	0.033367959	-0.00011498	-0.00646262	0.290292795	0.343676011	10	0	0
NOP2	0.2068093	0.210028706	-0.00321937	-0.00501049	0.290628266	0.344050464	8	0	0
MED13	0.0503724	0.050639177	-0.00026677	-0.00105381	0.290726212	0.3441437	7	0	0
GMPR	0.2291567	0.22273656	0.006420165	0.144142564	0.290748786	0.344147709	7	0	0
BBS9	0.0732431	0.074220856	-0.00097771	-0.00899137	0.290999964	0.344376838	5	0	0
SRXN1	0.1586295	0.149986471	0.008642989	0.190327491	0.290984448	0.344376838	8	0	0
KRAS	0.2065145	0.208445438	-0.00193095	0.032085272	0.291221413	0.344616169	15	0	0
ROMO1	0.0999896	0.105642269	-0.00565263	0.013622607	0.291263041	0.344642691	11	0	0
MLLT3	0.0522107	0.051589281	0.000621429	0.013664243	0.291299754	0.344663393	11	0	0
PPIL1	0.325232	0.33012606	-0.00489407	0.007320964	0.291348753	0.34469863	11	0	0
ZEB2	0.416201	0.410839091	0.00536186	0.158597526	0.291406314	0.344743991	3	0	0
MRPS15	0.1477105	0.147674836	3.57E-05	0.049403859	0.291502337	0.344834845	7	0	0
SWT1	0.0623311	0.063050703	-0.00071963	-0.00529662	0.291781479	0.345119534	16	0	0
SLK	0.0459594	0.04565858	0.000300834	0.025658866	0.291769878	0.345119534	5	0	0

USF1	0.0383982	0.036636	0.001762226	0.031713928	0.292042371	0.345381874	10	0	0
ADH1C	0.4016946	0.402506035	-0.00081145	0.083664887	0.292061044	0.345381874	4	0	0
SFSWAP	0.1266679	0.122278525	0.004389363	0.042234938	0.292060257	0.345381874	14	0	0
SUGP1	0.0516149	0.0485475	0.003067441	0.077441999	0.292149847	0.345464113	12	0	0
MED7	0.7160595	0.703719915	0.012339581	0.024729745	0.292187449	0.345485799	1	0	0
TMEM203	0.0808441	0.087012478	-0.00616835	-0.03200888	0.292267181	0.345557294	12	0	0
TTC9	0.1001744	0.099495628	0.000678784	0.02513123	0.292393214	0.345683521	8	0	0
RIPK2	0.0403452	0.038773493	0.001571702	0.044307032	0.292750743	0.34606059	7	0	0
SH2B3	0.0499784	0.043495088	0.006483275	0.118086966	0.29273223	0.34606059	8	0	0
DAAM1	0.1436357	0.151184974	-0.00754932	-0.04286419	0.293112987	0.346465964	11	0	0
BUB1	0.0704278	0.071034848	-0.00060709	-0.01958086	0.293242267	0.346595934	11	0	0
NRG2	0.0512519	0.044331178	0.006920741	0.216901299	0.293269205	0.346604932	14	0	0
ABHD10	0.2147952	0.206270996	0.0085242	0.064808152	0.293296408	0.346614244	13	0	0
GLI2	0.2176195	0.212309433	0.005310069	0.092614594	0.293349476	0.34663128	3	0	0
ZWINT	0.0375072	0.039403133	-0.00189596	-0.05537584	0.29334568	0.34663128	6	0	0
IFT122	0.0512655	0.050393845	0.000871675	-0.00143482	0.293373909	0.346637315	16	0	0
WDR60	0.1666971	0.172494537	-0.00579747	-0.01243837	0.293470066	0.346728088	5	0	0
MBD4	0.0524188	0.051415924	0.00100289	0.00083891	0.293511219	0.346737625	15	0	0
TLCD1	0.0327946	0.030148275	0.002646368	0.074290259	0.293539559	0.346737625	7	0	0
KRT40	0.6019495	0.631802263	-0.02985274	-0.07182243	0.293555466	0.346737625	4	0	0
PRKACA	0.0596913	0.057329072	0.00236222	0.044919109	0.293525946	0.346737625	8	0	0
GALC	0.3650972	0.370697721	-0.0056005	0.052431456	0.293657881	0.346835754	12	0	0
ATG9A	0.0431087	0.042833548	0.000275153	0.012726861	0.293816359	0.34700008	19	0	0
MAT2A	0.033799	0.034703775	-0.00090474	-0.03647071	0.293860678	0.34702041	8	0	0
PDCD6	0.02533	0.025017667	0.000312329	0.000431115	0.293872268	0.34702041	3	0	0
PPHLN1	0.0753764	0.078588008	-0.00321159	-0.0274707	0.294491939	0.347729257	7	0	0
FGD4	0.1382257	0.140126681	-0.00190095	-0.00084008	0.294549892	0.347774792	5	0	0
SPRED1	0.0995212	0.094179102	0.005342064	0.012080714	0.29465125	0.347871566	5	0	0
PLOD1	0.0681172	0.058652469	0.009464727	0.189771166	0.294766108	0.34796536	6	0	0
PSMC4	0.0569457	0.059584919	-0.00263927	0.027130119	0.294769496	0.34796536	11	0	0
SLC25A29	0.1842786	0.182602975	0.001675639	0.01473979	0.294819634	0.348001642	9	0	0
SERBP1	0.1989784	0.207861351	-0.00888297	-0.04470557	0.294903806	0.34807809	5	0	0
ZNF446	0.7486156	0.767912553	-0.01929693	-0.03627286	0.295203941	0.348409415	2	0	0
COMMD1	0.0420153	0.042969254	-0.00095396	-0.02236161	0.29536226	0.348573332	8	0	0
WFDC10A	0.1069518	0.099562674	0.007389166	0.112127445	0.295438859	0.34864079	3	0	0
SUV39H2	0.0515759	0.051121391	0.000454476	0.010976938	0.295465154	0.348648882	4	0	0
DMTF1	0.0696698	0.069086265	0.000583491	0.011804339	0.29596405	0.349214604	16	0	0
HS6ST1	0.3729993	0.380357868	-0.00735854	-0.02432026	0.296034096	0.349230882	3	0	0
PCM1	0.0636358	0.062991349	0.000644452	0.019518675	0.296055729	0.349230882	5	0	0
NKX3-1	0.1953633	0.196588257	-0.00122498	-0.0065985	0.296042769	0.349230882	8	0	0
SLC31A1	0.0282973	0.028616753	-0.00031946	-0.01045078	0.296041876	0.349230882	8	0	0
ABHD12	0.0484095	0.046940147	0.001469349	0.023253029	0.296106935	0.349268314	6	0	0
APMAP	0.1004806	0.098311982	0.00216861	-0.09929087	0.296183603	0.349335774	5	0	0
RAPGEF5	0.0795271	0.084926626	-0.00539954	-0.05090412	0.296408278	0.34957778	10	0	0
ARHGAP31	0.0425569	0.040043194	0.002513675	0.080263136	0.296759162	0.349945584	9	0	0
TCP1	0.0659477	0.067024172	-0.0010765	-0.02450822	0.296750918	0.349945584	10	0	0
PDZD8	0.1119158	0.114032146	-0.00211638	-0.00997087	0.296831717	0.349985122	8	0	0
ATXN7L3B	0.0600299	0.062086794	-0.00205687	-0.04669331	0.296944687	0.350080621	9	0	0
ZNF865	0.0629891	0.05919626	0.003792855	0.061634183	0.296951749	0.350080621	4	0	0
ATG2A	0.0388227	0.03816957	0.000653163	0.024330819	0.296994738	0.35008528	10	0	0
PPP2R5B	0.0388227	0.03816957	0.000653163	0.024330819	0.296994738	0.35008528	10	0	0
DNER	0.0850693	0.078069	0.007000339	0.154434	0.297071906	0.350153231	5	0	0
RNF214	0.074426	0.073824179	0.000601858	0.007634577	0.297093304	0.350155441	11	0	0
CDK5R1	0.2478147	0.250195263	-0.0023806	-0.02491779	0.29715653	0.350206947	8	0	0
LCE1D	0.4480218	0.426268638	0.021753145	0.127268633	0.297206779	0.350243153	2	0	0
03-Mar	0.1896658	0.196214276	-0.00654843	-0.00136466	0.297494454	0.350559131	6	0	0
PXDC1	0.1949035	0.186499537	0.008403945	0.251056735	0.297514216	0.350559387	7	0	0
SSX2IP	0.1547812	0.161593175	-0.00681195	-0.03913813	0.297546388	0.350574264	12	0	0
STAM	0.0409982	0.041188922	-0.00019069	-0.0130307	0.297729907	0.350767446	11	0	0
RNF187	0.045415	0.046605639	-0.00119064	-0.01773696	0.297759518	0.350779292	7	0	0
SGSM3	0.3689095	0.372466439	-0.00355698	0.029408278	0.298051344	0.35110002	12	0	0
TMEM184C	0.1885786	0.189326827	-0.00074824	-0.02619767	0.29808587	0.35111763	11	0	0
RNF19A	0.3902565	0.405609568	-0.01535311	-0.05117054	0.298297565	0.351343914	9	0	0

PLOD3	0.0492492	0.048355356	0.000893834	0.019656215	0.29847737	0.35153261	5	0	0
GABARAPL1	0.057216	0.052474254	0.004741783	0.057933461	0.298606222	0.351661274	7	0	0
MED29	0.0473765	0.047055789	0.000320695	0.011349022	0.29865586	0.351696639	12	0	0
ROGDI	0.0314774	0.030186978	0.001290406	0.037642918	0.298740138	0.351772788	7	0	0
DZIP1L	0.0604498	0.049761521	0.01068827	0.254211882	0.299305232	0.352345671	8	0	0
HIST1H4E	0.1790671	0.172315279	0.006751812	0.119623841	0.29928284	0.352345671	9	0	0
ZBTB9	0.0584946	0.060122743	-0.00162816	-0.01809283	0.299298536	0.352345671	20	0	0
H2AFX	0.2239895	0.215093029	0.008896502	0.056742517	0.299273411	0.352345671	6	0	0
IL15	0.1974645	0.202394961	-0.00493042	-0.01582329	0.299331979	0.352354032	14	0	0
TEX9	0.0416164	0.041221953	0.000394438	0.013634292	0.299377544	0.35238454	9	0	0
MXD1	0.83614	0.850035842	-0.01389585	-0.02426225	0.299537284	0.352526294	3	0	0
LSM7	0.1174608	0.11956923	-0.0021084	-0.00024729	0.2995344	0.352526294	11	0	0
TLE1	0.0398161	0.034388474	0.005427628	0.177939528	0.299561809	0.352532026	5	0	0
PTPN12	0.0527895	0.050897563	0.001891939	0.053816235	0.299619651	0.352576393	12	0	0
KLC2	0.1053592	0.105971293	-0.00061205	0.03203646	0.299658482	0.352576393	14	0	0
POLR2I	0.0890131	0.090212312	-0.0011992	0.010242702	0.299644693	0.352576393	10	0	0
NARF	0.1796344	0.174524002	0.005110412	0.001188996	0.299724985	0.352631508	9	0	0
TMEM9	0.5595941	0.564838214	-0.00524409	0.024733192	0.299774905	0.352643976	7	0	0
FST	0.0577615	0.053909848	0.003851687	0.090076956	0.299773375	0.352643976	9	0	0
ANKRA2	0.0373127	0.038603311	-0.0012906	-0.03763112	0.299804185	0.352650249	15	0	0
FAM120B	0.0548064	0.057493015	-0.00268657	-0.02650787	0.299819561	0.352650249	13	0	0
C4orf46	0.0385382	0.038208793	0.000329422	0.024663368	0.299852501	0.352665866	10	0	0
PPP1R3D	0.0495222	0.048711106	0.000811045	0.034228437	0.299929671	0.352733499	5	0	0
CEP72	0.0824807	0.083438132	-0.00095746	-0.03391321	0.30005759	0.3528608	10	0	0
TMED2	0.0850097	0.086814824	-0.00180517	-0.00281812	0.300253585	0.353068138	13	0	0
TNKS	0.0485649	0.049822222	-0.00125728	-0.00538906	0.30040393	0.353221771	8	0	0
FCHSD2	0.0437373	0.042668225	0.001069091	0.022453757	0.300426942	0.353225672	8	0	0
STAM2	0.0403046	0.039418519	0.00088608	0.02709246	0.300554161	0.353328926	6	0	0
CCDC127	0.0388617	0.037356447	0.001505247	0.040963901	0.30054168	0.353328926	2	0	0
CTU2	0.1354583	0.141929232	-0.00647096	-0.01986969	0.300664884	0.353435926	7	0	0
GSTCD	0.0422576	0.0413997	0.000857918	0.023434951	0.301116954	0.353944144	15	0	0
ROPN1L	0.076951	0.076238302	0.00071265	0.145186599	0.301141793	0.353950144	10	0	0
WDR78	0.1099614	0.108499393	0.001462015	0.018459443	0.301285633	0.354049605	15	0	0
MTHFSD	0.0689592	0.06810142	0.000857739	-0.0174804	0.301271859	0.354049605	9	0	0
SLC25A19	0.0392134	0.041209729	-0.00199634	-0.04814119	0.301281056	0.354049605	9	0	0
ZNF544	0.0547333	0.04825889	0.006474392	0.260009561	0.301399075	0.354159709	12	0	0
MON2	0.0449913	0.045368975	-0.00037764	0.002081748	0.301615164	0.354390409	12	0	0
HAX1	0.0989117	0.101606402	-0.00269471	-0.01216198	0.301721852	0.354446106	10	0	0
ZNF526	0.0884122	0.092165928	-0.00375368	-0.00406012	0.301699473	0.354446106	9	0	0
NR1H2	0.0589016	0.049860518	0.009041111	0.244649053	0.301715674	0.354446106	9	0	0
SMIM12	0.1494028	0.149962806	-0.00056004	0.023587727	0.301795287	0.354509153	10	0	0
CALB2	0.0982381	0.104004879	-0.00576673	-0.02746905	0.302126139	0.354874553	10	0	0
C5orf22	0.1138276	0.119566552	-0.00573891	-0.00694026	0.302397029	0.35516192	9	0	0
SART1	0.2783539	0.280129728	-0.00177582	0.007695224	0.302410395	0.35516192	7	0	0
ZNF174	0.0326167	0.031675166	0.000941518	0.02654804	0.302481823	0.355222548	6	0	0
RASGRP3	0.4712067	0.436020667	0.035186024	0.10954614	0.302692035	0.355423887	1	0	0
TMEM262	0.839295	0.852665692	-0.01337065	-0.02350198	0.302692901	0.355423887	6	0	0
PLRG1	0.0570539	0.056290646	0.000763291	-0.00192689	0.302833607	0.355565827	8	0	0
INSIG1	0.0333734	0.030261559	0.003111849	0.134513816	0.302870146	0.355585451	5	0	0
SLIRP	0.0490313	0.048448021	0.000583317	0.018485507	0.303262307	0.356022565	12	0	0
KBTBD3	0.0458855	0.045389225	0.000496316	-0.00848555	0.303396635	0.356156951	16	0	0
UBE2D3	0.0387526	0.038852104	-9.95E-05	0.007467094	0.303464396	0.356213181	6	0	0
PAQR3	0.1252008	0.131047077	-0.00584629	-0.01049079	0.303569401	0.356289803	10	0	0
LRRC10B	0.1489585	0.139233546	0.009724912	0.235933391	0.303554773	0.356289803	7	0	0
IL22RA1	0.5698246	0.56517416	0.004650436	0.019562315	0.303731803	0.356433762	4	0	0
AMZ1	0.4836906	0.515490665	-0.03180004	-0.08779745	0.303730316	0.356433762	2	0	0
SMARCA2	0.0732989	0.076930483	-0.00363154	-0.02641357	0.303827112	0.356522282	6	0	0
DLL4	0.0465173	0.04426252	0.00225479	0.07841324	0.303893622	0.356569972	14	0	0
TEN1	0.1047704	0.108804007	-0.00403364	-0.00557353	0.303907513	0.356569972	11	0	0
EFCAB14	0.0510765	0.048415732	0.002660808	0.056016096	0.303983697	0.356621218	9	0	0
SRF	0.0489732	0.047089906	0.001883252	0.031139176	0.303990957	0.356621218	11	0	0
P4HB	0.0428698	0.043486536	-0.00061678	-0.00754045	0.304115418	0.356743894	11	0	0
DYNC1I2	0.252188	0.255160826	-0.00297284	-0.00758441	0.3042912	0.356926752	14	0	0

TPST1	0.0520042	0.050759151	0.001245028	0.040905342	0.304324548	0.356942526	7	0	0
TUBB4B	0.0533868	0.055167036	-0.00178022	-0.04725495	0.304353678	0.35695335	4	0	0
LMBRD2	0.0596572	0.061524131	-0.00186694	-0.0092398	0.304491562	0.357091713	14	0	0
MYRIP	0.1920018	0.190683147	0.001318615	0.044214167	0.304535649	0.357120066	10	0	0
OARD1	0.8487632	0.847337262	0.00142589	0.002810604	0.304571739	0.357138967	4	0	0
MZT1	0.0314445	0.031176954	0.00026754	0.004514481	0.304591591	0.357138967	11	0	0
11-Sep	0.1375389	0.139686123	-0.00214723	0.065426422	0.304795974	0.357355249	11	0	0
TBC1D17	0.0434199	0.043700294	-0.00028037	-0.00191518	0.304848266	0.357393196	16	0	0
RHOBTB1	0.2924071	0.294184552	-0.00177741	0.038629797	0.304876839	0.357403333	6	0	0
ATP5S	0.0360676	0.036405324	-0.00033768	0.003870041	0.305233072	0.357797554	11	0	0
HSPA9	0.0518172	0.052512438	-0.00069529	-0.02493706	0.305387588	0.357955286	12	0	0
PPP1R9B	0.0606556	0.063754059	-0.00309842	-0.0283733	0.305660344	0.35825158	8	0	0
RAD23B	0.0599869	0.058272133	0.001714784	0.027515064	0.305772277	0.358359355	9	0	0
INPP5A	0.1010782	0.096293333	0.004784828	0.062487338	0.30583534	0.358409843	11	0	0
FOXF1	0.043979	0.042055633	0.001923351	0.081480063	0.305982184	0.358549058	11	0	0
ZNF383	0.041483	0.032266122	0.009216856	0.327109405	0.305994114	0.358549058	5	0	0
RNF4	0.4132196	0.393942976	0.019276615	0.073461986	0.306277215	0.358857337	2	0	0
N4BP2L1	0.0592858	0.055994732	0.003291033	0.03928456	0.306411806	0.358991583	12	0	0
PSMC6	0.1321025	0.13226157	-0.00015909	0.018485735	0.30653337	0.35911055	10	0	0
BLM	0.1159704	0.120915981	-0.00494558	-0.01830502	0.30657177	0.35913208	10	0	0
DEFB1	0.5754761	0.577619374	-0.00214331	-0.01294063	0.306619888	0.359164989	5	0	0
SP3	0.1288636	0.137324982	-0.00846143	-0.00964743	0.306665334	0.359194764	8	0	0
NCSTN	0.0442061	0.04610431	-0.00189817	-0.00248036	0.306800228	0.359306139	9	0	0
YME1L1	0.030837	0.031625764	-0.00078873	-0.02642482	0.306800486	0.359306139	5	0	0
KIFC2	0.1287437	0.136864217	-0.00812047	-0.05279035	0.306820762	0.359306424	10	0	0
DPH1	0.2522541	0.251815053	0.000439014	-0.00042868	0.307338664	0.359889422	8	0	0
SLC22A4	0.0889343	0.094286726	-0.00535238	-0.01225146	0.307361126	0.359892229	14	0	0
RGS9BP	0.0552689	0.055307541	-3.86E-05	0.004288513	0.307436352	0.359956813	11	0	0
NUP155	0.0835879	0.077604153	0.00598376	0.010737249	0.307585092	0.360107454	12	0	0
FAM83A	0.8472446	0.872709363	-0.02546477	-0.0422316	0.307691537	0.360208563	1	0	0
DPH2	0.1408247	0.144289558	-0.00346483	-0.04738716	0.307832599	0.360326666	15	0	0
THNSL1	0.0297187	0.030094855	-0.00037613	0.005141476	0.307820823	0.360326666	6	0	0
ADCY5	0.3608259	0.374085353	-0.01325943	0.021550121	0.308157114	0.360682981	2	0	0
CAPNS1	0.1742171	0.174575323	-0.00035826	0.040516126	0.308233375	0.360748699	6	0	0
PKN2	0.0426258	0.043526573	-0.00090081	-0.01403634	0.308271729	0.360770047	10	0	0
MTRNR2L3	0.6727866	0.689682553	-0.01689591	-0.03526569	0.308328638	0.360813105	1	0	0
NIPBL	0.0556111	0.053964794	0.001646314	0.009938696	0.30860066	0.36110787	11	0	0
EEF1B2	0.0289832	0.028863796	0.000119433	0.009018401	0.308662033	0.361156124	13	0	0
CUL7	0.2323772	0.228133602	0.004243623	0.01418582	0.308778188	0.361268466	11	0	0
C15orf65	0.0533187	0.050250949	0.003067724	0.043965749	0.309172601	0.361706332	11	0	0
REPS1	0.0352414	0.035008373	0.000233074	-0.00699904	0.309241276	0.361763079	9	0	0
HIP1	0.0294545	0.029690631	-0.00023612	-0.00627167	0.309384838	0.361907419	7	0	0
RGS12	0.0301245	0.029366549	0.000757937	0.027118007	0.309494717	0.362012341	4	0	0
XKR4	0.1884202	0.175758391	0.012661807	0.148378525	0.309607046	0.362120116	7	0	0
HMGN4	0.0651882	0.063952498	0.001235664	0.013943407	0.309885392	0.362422039	9	0	0
HPGD	0.1809957	0.166720328	0.014275386	0.079427512	0.310120418	0.362673261	5	0	0
SKI	0.0838204	0.081925575	0.001894846	0.022334804	0.310171577	0.362709441	6	0	0
RAB22A	0.1547528	0.15453706	0.000215769	0.029391938	0.310368824	0.362916437	11	0	0
SFR1	0.0442279	0.04465791	-0.00043002	-0.02121908	0.310420192	0.36295284	8	0	0
TM9SF4	0.1797249	0.187411712	-0.0076868	-0.02394008	0.310469473	0.362963138	9	0	0
GNB1L	0.0438292	0.044311432	-0.0004822	-0.000964	0.310459716	0.362963138	5	0	0
FRK	0.531989	0.520961719	0.011027253	0.029506678	0.31058961	0.363079922	8	0	0
IPO13	0.0438016	0.04305575	0.000745812	0.000546507	0.310713467	0.363201039	16	0	0
AGRN	0.0494755	0.051634178	-0.00215871	-0.03231976	0.310811776	0.363292279	7	0	0
WDR55	0.1465819	0.145469678	0.001112228	0.034551385	0.31087732	0.363345211	8	0	0
NUDCD3	0.0465934	0.046017211	0.000576145	0.010515888	0.310899854	0.363347871	9	0	0
PEX11B	0.0576058	0.05797806	-0.00037227	0.031356118	0.310929594	0.363358952	19	0	0
ARL5B	0.0715739	0.067877075	0.003696851	0.056400582	0.311220784	0.363651855	9	0	0
FASTKD3	0.1954472	0.192420304	0.003026891	0.02124345	0.311299374	0.36371999	8	0	0
RASSF4	0.1066038	0.110493155	-0.00388932	-0.02555681	0.311338329	0.36374181	8	0	0
MYO1G	0.4638564	0.460690869	0.003165508	0.001146747	0.31142588	0.363796704	6	0	0
EIF4E2	0.0456181	0.046651354	-0.00103327	-0.016834	0.311524191	0.363887847	7	0	0
TSC22D1	0.0658784	0.065855304	2.31E-05	-0.00351202	0.311634012	0.363992422	6	0	0

L2HGDH	0.2095539	0.214398446	-0.00484453	0.004623094	0.311738466	0.364090717	12	0	0
CNPY4	0.0467464	0.046605877	0.000140554	0.012397991	0.311861395	0.364210573	18	0	0
BTN2A1	0.0512354	0.048861916	0.002373533	0.048170779	0.311883277	0.364212413	5	0	0
GLB1L	0.0555849	0.055239367	0.000345499	0.002776921	0.311911748	0.364221947	14	0	0
ZNF131	0.0430656	0.044007008	-0.00094145	-0.01267773	0.312020481	0.364325197	3	0	0
PPME1	0.0591362	0.058854285	0.000281876	0.007913656	0.312202292	0.364513755	18	0	0
MAPK8IP2	0.1511928	0.151804615	-0.00061181	0.009146388	0.312348286	0.364660474	6	0	0
TSHZ1	0.0539956	0.052128208	0.001867376	0.051182343	0.312513235	0.364829302	9	0	0
DEDD	0.0420118	0.040103471	0.001908313	0.047511971	0.312566138	0.364867312	11	0	0
C1QL4	0.3095299	0.297421801	0.01210809	0.075835022	0.312819896	0.365139767	8	0	0
PANX1	0.1447791	0.145964987	-0.00118585	-0.02505637	0.313243508	0.365610435	10	0	0
KIF26B	0.0469854	0.047324965	-0.00033955	-0.02266528	0.313285305	0.365635427	13	0	0
ZNF79	0.050558	0.053768192	-0.00321016	-0.06748783	0.313504072	0.365843138	6	0	0
ANO6	0.0828938	0.086700434	-0.00380664	-0.00350487	0.313502266	0.365843138	11	0	0
GPX4	0.1432052	0.152044774	-0.00883957	-0.00725422	0.31360251	0.365934202	7	0	0
MLF2	0.1708153	0.150580552	0.020234793	0.127467101	0.313667515	0.365986244	8	0	0
DROSHA	0.0503745	0.051796359	-0.00142188	-0.02016881	0.313971337	0.366316913	11	0	0
PNO1	0.049735	0.049976571	-0.00024162	-0.01107607	0.314033197	0.366364807	11	0	0
CTNS	0.0349533	0.035064268	-0.00011097	-0.00112432	0.31405324	0.366364807	8	0	0
EIF4G1	0.0766844	0.071329879	0.005354511	0.083196966	0.314235088	0.366549411	2	0	0
ZMAT5	0.0451769	0.046600491	-0.00142355	-0.00740765	0.314252358	0.366549411	11	0	0
PIGM	0.1840065	0.178884314	0.00512221	0.053508143	0.314304843	0.36658679	8	0	0
DIS3	0.0573531	0.058664122	-0.00131101	-0.01248435	0.314327297	0.366589141	15	0	0
C2CD3	0.0606476	0.060241692	0.000405936	0.012631802	0.314512668	0.366781484	17	0	0
SPECC1	0.0499382	0.04938875	0.000549418	0.015572469	0.314756821	0.367042347	5	0	0
NVL	0.0508174	0.049288544	0.001528868	0.040082311	0.314792266	0.367059815	8	0	0
HES2	0.1343635	0.129834259	0.004529244	0.148362859	0.314875986	0.367133568	7	0	0
PPP1R14C	0.0531761	0.048459455	0.004716654	0.090612114	0.314966555	0.367215297	12	0	0
SMPD1	0.0800788	0.084150417	-0.00407164	-0.0419975	0.315414555	0.367713712	7	0	0
POU3F1	0.0959761	0.090367785	0.005608284	0.06044142	0.315622918	0.367932709	11	0	0
ZNF519	0.0786905	0.084253647	-0.00556318	-0.05597302	0.316409167	0.368825295	2	0	0
PTEN	0.0441848	0.043679586	0.000505224	0.029911328	0.316509596	0.368918385	44	0	0
TSPAN19	0.1210063	0.124805218	-0.00379894	0.021289493	0.316592524	0.368991066	13	0	0
DDX52	0.2799614	0.296477595	-0.01651623	-0.02616647	0.316848199	0.369265061	11	0	0
RAP2A	0.0553683	0.052314709	0.003053612	0.046520758	0.316874648	0.369271891	8	0	0
TBL3	0.0494655	0.049103149	0.000362322	0.014459446	0.317043436	0.369444586	8	0	0
RPS27	0.2950185	0.293588284	0.001430213	0.008620404	0.317342585	0.369769155	14	0	0
TSSK3	0.3456359	0.339859869	0.005776005	0.022077943	0.317735885	0.370203381	5	0	0
PHTF1	0.1507201	0.154165305	-0.00344518	0.005091624	0.317788163	0.37024024	12	0	0
ARPC1B	0.2862807	0.28821997	-0.00193931	0.203740677	0.318019265	0.37048542	9	0	0
SLC30A9	0.0612254	0.060524415	0.000701033	0.036629238	0.318104098	0.37056018	10	0	0
TSKS	0.7909009	0.795135364	-0.00423443	-0.0067905	0.318295289	0.370758819	3	0	0
TRPC1	0.1100461	0.108586825	0.001459299	0.151038594	0.318348757	0.370797019	11	0	0
CHN1	0.0814653	0.082330964	-0.00086561	0.005140025	0.318397335	0.37082952	13	0	0
RSU1	0.1224641	0.123807331	-0.00134326	-0.00880425	0.318535401	0.370966233	11	0	0
BCL2L12	0.052591	0.052179061	0.000411894	0.014442234	0.318582997	0.370997574	10	0	0
TFB2M	0.0490919	0.0484846	0.000607329	0.017919879	0.318620031	0.371016042	9	0	0
HMGXB3	0.2247754	0.228709183	-0.00393381	-0.01010295	0.318640227	0.371016042	14	0	0
RRS1	0.1161375	0.11926024	-0.00312275	0.019035999	0.318679443	0.371037617	11	0	0
MYC	0.0585661	0.059257993	-0.00069187	-0.01311267	0.318974734	0.371357317	20	0	0
MTRF1L	0.0482976	0.049304723	-0.00100717	-0.00551192	0.319078404	0.371453901	12	0	0
RGS20	0.5834769	0.591404525	-0.00792767	-0.03414124	0.319813646	0.372285667	3	0	0
FAP	0.5383165	0.575492624	-0.03717613	-0.09464214	0.319966341	0.372439243	1	0	0
CCDC59	0.092398	0.091590024	0.000807945	-0.01243067	0.320023104	0.372481142	17	0	0
ERCC4	0.0869522	0.085045827	0.001906336	-0.00899566	0.320464605	0.37297081	11	0	0
MGME1	0.0355828	0.035081747	0.000501043	0.018554367	0.320815046	0.373354443	10	0	0
COL27A1	0.0426684	0.040409181	0.002259199	0.063031012	0.32085077	0.373371791	6	0	0
CCDC28B	0.2450429	0.248451192	-0.00340824	-0.02887527	0.320909927	0.373407367	11	0	0
ANKIB1	0.1019015	0.107373491	-0.00547195	-0.00720987	0.32092298	0.373407367	15	0	0
FCHO2	0.131841	0.128856503	0.002984532	0.088180337	0.320985892	0.373448786	8	0	0
TGFBR3L	0.272321	0.260255701	0.012065261	0.228611775	0.321000219	0.373448786	9	0	0
C8orf44	0.047934	0.047558434	0.000375565	0.017997234	0.321370107	0.37385486	13	0	0
RPL18	0.1209958	0.123532186	-0.00253641	0.00505604	0.321692504	0.374205639	16	0	0

MED8	0.0995534	0.097108911	0.002444512	0.028203353	0.32178848	0.374293007	12	0	0
PITPNC1	0.1377339	0.138975213	-0.00124135	0.019048953	0.321921686	0.374423667	14	0	0
VLDLR	0.0625078	0.058619668	0.003888168	0.114226858	0.322031373	0.374526955	6	0	0
CCT6B	0.0341211	0.032725747	0.001395341	0.046362642	0.322090603	0.374571553	1	0	0
CCDC115	0.0526245	0.051804132	0.000820361	0.016593517	0.322279729	0.374767195	13	0	0
ZRANB3	0.0480624	0.046072936	0.001989472	0.037698286	0.322428777	0.374894465	11	0	0
ISYNA1	0.1998997	0.20085812	-0.00095846	0.080847414	0.322430978	0.374894465	9	0	0
NPC1	0.1010097	0.10702837	-0.00601869	-0.00525456	0.322767575	0.375261504	7	0	0
ID2	0.0462998	0.043438416	0.002861365	0.054590992	0.322855292	0.37531483	10	0	0
KMT2D	0.033499	0.034126837	-0.0006278	-0.00800918	0.322847405	0.37531483	7	0	0
ZNF700	0.0884764	0.078717729	0.009758662	0.143128272	0.322963141	0.375391543	2	0	0
ZNF763	0.0884764	0.078717729	0.009758662	0.143128272	0.322963141	0.375391543	2	0	0
XRCC3	0.0540251	0.054590403	-0.0005653	0.000549466	0.322992339	0.375401153	15	0	0
AKIRIN2	0.0882523	0.086436155	0.00181614	0.023901157	0.323077837	0.375466961	8	0	0
GRK5	0.1070408	0.102392213	0.004648561	0.014658143	0.323090827	0.375466961	4	0	0
TMUB2	0.0629701	0.064626587	-0.00165651	-0.00981997	0.323148863	0.375510075	14	0	0
AKAP3	0.094765	0.098218099	-0.00345309	-0.0153261	0.323477332	0.375867416	15	0	0
NCDN	0.1317199	0.134146409	-0.00242656	-0.01104829	0.323612031	0.375999571	12	0	0
FYN	0.0758446	0.068587014	0.007257541	0.100120552	0.323637123	0.376004368	11	0	0
POLA2	0.1534342	0.156982926	-0.00354877	0.005613207	0.323965573	0.376361585	7	0	0
DIXDC1	0.3407186	0.341505741	-0.00078714	0.004330463	0.324083485	0.376474183	6	0	0
RPL12	0.0277158	0.027884903	-0.00016908	-0.00093894	0.324335025	0.376741987	9	0	0
FZD1	0.0460576	0.046634259	-0.00057666	-0.01100656	0.324540604	0.376956371	9	0	0
XXYLT1	0.1255663	0.130360962	-0.0047947	-0.02379396	0.324613553	0.376992275	6	0	0
FLYWCH2	0.0425925	0.043809635	-0.00121711	-0.01584971	0.32460477	0.376992275	6	0	0
PIGW	0.0431946	0.041716348	0.001478215	0.037843008	0.324973876	0.377386303	8	0	0
TTC30B	0.0492478	0.049196458	5.14E-05	0.015761978	0.325013783	0.37740821	8	0	0
S1PR1	0.0573697	0.051838801	0.00553088	0.142260022	0.325206305	0.377582877	15	0	0
NDUFAF7	0.0461533	0.045106168	0.001047085	0.010526066	0.325196532	0.377582877	12	0	0
HIST1H2BC	0.0501984	0.04987211	0.000326273	0.019328928	0.325514138	0.377915825	10	0	0
LAPTM5	0.5066869	0.481311827	0.025375102	0.085653823	0.325647845	0.378046586	4	0	0
COL9A2	0.3120989	0.289855369	0.022243536	0.193200552	0.325942917	0.378364647	10	0	0
SSBP4	0.1417193	0.13948623	0.002233068	0.060653488	0.325971968	0.378373881	9	0	0
POLR3E	0.05177	0.047492549	0.004277471	0.064112481	0.326100259	0.378498301	8	0	0
SDR16C5	0.5447524	0.560981159	-0.01622878	-0.0399136	0.32637996	0.378774056	8	0	0
CKS2	0.1211583	0.122626704	-0.00146838	-0.02086355	0.326380075	0.378774056	9	0	0
SMIM8	0.1326662	0.122067474	0.01059873	0.131916947	0.326478207	0.378863427	10	0	0
NBAS	0.0593131	0.056454366	0.002858706	0.053027541	0.326509634	0.378875383	12	0	0
ACTR10	0.0518469	0.049580093	0.002266807	0.036712412	0.326979391	0.379395935	9	0	0
SRRT	0.1853311	0.173254299	0.012076782	0.089381771	0.327135984	0.379553076	9	0	0
TMEM67	0.2208271	0.207348136	0.013478927	0.053844967	0.327200891	0.379603828	11	0	0
RNF31	0.0544902	0.051795439	0.00269478	0.059416709	0.327434709	0.379850523	3	0	0
TPPP	0.0268862	0.024884144	0.002002033	0.046072395	0.327542016	0.379950434	5	0	0
REXO4	0.103136	0.093888747	0.009247261	0.033051691	0.327609803	0.380004489	6	0	0
SPAG7	0.2837377	0.295979689	-0.012242	-0.00393845	0.327671205	0.380051134	11	0	0
SLC39A6	0.0615877	0.06607943	-0.00449176	-0.02895782	0.327826921	0.380207155	17	0	0
PKP2	0.3244213	0.337027571	-0.01260626	0.055275094	0.32793089	0.380303145	5	0	0
ABCD3	0.1425463	0.143481406	-0.00093511	0.005639367	0.327980368	0.380335934	9	0	0
APRT	0.0639913	0.062347847	0.001643405	0.010354657	0.328350316	0.38074032	10	0	0
METTL25	0.0867994	0.089486044	-0.00268667	-0.00903131	0.328494664	0.380883076	18	0	0
ABI2	0.1742706	0.172251501	0.002019144	0.035187933	0.328548039	0.380904497	9	0	0
TCP11L2	0.0436664	0.043535702	0.000130709	0.020937992	0.328555613	0.380904497	4	0	0
ISCU	0.0558243	0.054925343	0.000898979	0.018773402	0.328740477	0.381094182	12	0	0
FBXO38	0.1522691	0.157868889	-0.00559978	-0.02479442	0.328882738	0.38123446	14	0	0
ALS2	0.0458425	0.046215547	-0.00037302	-0.01222267	0.329237443	0.381620963	14	0	0
RNF186	0.5536582	0.561970269	-0.00831206	-0.02170421	0.329396893	0.38178111	5	0	0
ZADH2	0.0509499	0.04918912	0.001760818	0.049554947	0.329446053	0.381813415	10	0	0
LTV1	0.1122886	0.107520357	0.004768271	0.016923705	0.329632452	0.382004759	7	0	0
CISD2	0.0407232	0.041120413	-0.0003972	-0.00067188	0.32983823	0.382218535	7	0	0
SRSF6	0.0399654	0.04038632	-0.00042097	-0.00487474	0.330177587	0.382587067	11	0	0
TMEM165	0.0242093	0.024084183	0.000125099	0.011927418	0.330364649	0.382779092	8	0	0
TRAF3IP1	0.0830036	0.081200615	0.00180303	0.022225665	0.330501068	0.382912418	9	0	0
WBP4	0.0365333	0.036457155	7.61E-05	0.001291492	0.330573321	0.382971391	11	0	0

EMC7	0.2753507	0.280283467	-0.00493277	0.010157533	0.331089635	0.383544771	11	0	0
NOMO2	0.0352288	0.033258252	0.001970534	0.064265889	0.331179801	0.383585866	1	0	0
TMEM189-U	0.3406726	0.353040799	-0.01236824	-0.01692566	0.331189268	0.383585866	6	0	0
TMEM189	0.3406726	0.353040799	-0.01236824	-0.01692566	0.331189268	0.383585866	6	0	0
NABP1	0.0880722	0.09259296	-0.00452073	-0.01635865	0.331453574	0.383867198	14	0	0
TATDN1	0.0773076	0.082868577	-0.005561	-0.01863069	0.331782147	0.38422292	10	0	0
ING1	0.1139208	0.118954221	-0.00503347	0.003356982	0.331868707	0.38429835	11	0	0
IL7	0.1053621	0.100882376	0.004479768	0.025773186	0.331990549	0.384386001	9	0	0
KIAA0391	0.0660529	0.063889371	0.00216352	0.037856748	0.331978045	0.384386001	8	0	0
HSF2BP	0.0958338	0.088365371	0.007468419	0.026178573	0.332008693	0.384386001	11	0	0
ATG2B	0.0941398	0.099417749	-0.00527796	-0.03302677	0.332153042	0.38450999	9	0	0
PPP1R14D	0.7690703	0.764678492	0.004391825	0.008323811	0.332158663	0.38450999	4	0	0
RTN4RL2	0.0649484	0.05995855	0.004989851	0.127229762	0.332214032	0.384549266	8	0	0
PPM1K	0.0443385	0.044200832	0.000137618	0.000953259	0.332284199	0.384605666	10	0	0
RGS8	0.5971497	0.595703922	0.001445819	-0.00016435	0.332314995	0.38461649	2	0	0
MRPL30	0.1374602	0.140445206	-0.00298499	0.005165722	0.332459913	0.384739903	16	0	0
SPHK2	0.0354668	0.035640353	-0.00017351	0.004343934	0.332464527	0.384739903	15	0	0
FYTTD1	0.0333256	0.032424003	0.000901553	0.011435606	0.332574969	0.38484288	7	0	0
RMND1	0.0574519	0.057350095	0.000101778	0.016134329	0.333037529	0.385353275	10	0	0
SLC35E1	0.0368308	0.036976133	-0.00014529	0.008032695	0.333664615	0.386053963	10	0	0
MLYCD	0.0264151	0.026403025	1.21E-05	0.002903946	0.333764445	0.386144558	4	0	0
LMBR1L	0.1479408	0.148412289	-0.00047144	0.011902595	0.333925893	0.386306425	12	0	0
FAM117A	0.1077098	0.106348458	0.00136139	2.00E-05	0.334069742	0.386422991	9	0	0
TMEM200C	0.0617413	0.058513868	0.003227416	0.057297119	0.334060285	0.386422991	5	0	0
WSCD2	0.0709808	0.060618706	0.010362086	0.235999647	0.334241416	0.386596636	10	0	0
PRPF19	0.0440109	0.043255829	0.000755085	0.017568827	0.334390869	0.38674456	11	0	0
TK2	0.0890155	0.080769526	0.008246008	0.087325971	0.334567621	0.386924034	10	0	0
GEN1	0.0317475	0.031893893	-0.00014637	-0.00890069	0.334672534	0.387020411	7	0	0
CASP6	0.116617	0.110070549	0.00654645	0.041005431	0.33474601	0.387080422	11	0	0
LRIG1	0.0265793	0.025412247	0.001167016	0.043379876	0.334882367	0.387197683	3	0	0
TMEM245	0.2780908	0.286094713	-0.00800393	-0.01564215	0.334890592	0.387197683	6	0	0
ANP32A	0.0464795	0.049118111	-0.00263866	-0.01471421	0.334960042	0.387253017	10	0	0
MCM7	0.1977156	0.202865251	-0.00514967	-0.01331811	0.334983318	0.387254966	15	0	0
RIC3	0.2089222	0.184468909	0.024453265	0.195194208	0.335100673	0.387365666	5	0	0
ICK	0.035654	0.036939349	-0.00128534	-0.02965675	0.335278464	0.38754621	7	0	0
PKP4	0.1332916	0.126878005	0.006413644	0.036159017	0.335305392	0.38755236	8	0	0
GINS2	0.6269177	0.641261441	-0.0143437	-0.01261396	0.335466859	0.387714001	3	0	0
DCAF12	0.3937646	0.396557571	-0.00279296	-0.03324098	0.33556423	0.387801548	5	0	0
ZSCAN25	0.1699806	0.172093804	-0.00211317	7.43E-05	0.335619537	0.38782952	7	0	0
LITAF	0.6066506	0.589252627	0.017397978	0.04128896	0.33563168	0.38782952	1	0	0
ZNF587B	0.0262822	0.025435914	0.000846283	0.034049553	0.335678995	0.387859207	1	0	0
ABHD8	0.0315777	0.032643685	-0.00106593	-0.02998329	0.335735226	0.38789919	3	0	0
ZBTB26	0.0880081	0.093544148	-0.00553604	-0.01739993	0.335809554	0.387960075	8	0	0
CDK11B	0.0230794	0.022936711	0.000142728	0.009752245	0.335854469	0.387986974	5	0	0
LEPROTL1	0.0771223	0.080162174	-0.00303989	-0.00840381	0.336071731	0.388212956	8	0	0
CA14	0.4422157	0.448965219	-0.00674948	0.022566105	0.336414687	0.388584095	5	0	0
DBI	0.0513205	0.053899278	-0.00257873	-0.02465119	0.336584613	0.388755336	16	0	0
ACTR5	0.1553787	0.149280792	0.006097922	0.04067175	0.336761105	0.388934139	8	0	0
ARRDC3	0.0389407	0.03763233	0.001308357	0.024224087	0.336910514	0.389060591	7	0	0
PSMB9	0.123868	0.124662306	-0.0007943	0.034095929	0.336913977	0.389060591	33	0	0
MAP3K10	0.0296968	0.031160348	-0.00146355	-0.03429064	0.337060512	0.389204748	4	0	0
RWDD2A	0.0412975	0.040199194	0.001098299	0.03031363	0.33718865	0.389327645	11	0	0
PLCB3	0.167851	0.164790339	0.003060709	0.146448882	0.337557389	0.389728312	7	0	0
PHOSPHO1	0.1041305	0.099203734	0.004926795	0.218053242	0.337716263	0.389886643	13	0	0
CCT8	0.0611224	0.060396965	0.000725406	0.008446791	0.337738695	0.389887445	11	0	0
ITPKA	0.0452602	0.04646715	-0.001207	-0.01929506	0.33791484	0.390065682	7	0	0
SNX3	0.0617249	0.061143934	0.000580928	-0.00598414	0.338027977	0.39017117	6	0	0
GOPC	0.1124681	0.113309359	-0.00084128	0.142608129	0.338107964	0.390238381	11	0	0
PRPF18	0.036093	0.035021163	0.001071825	0.034431053	0.338186135	0.390303488	7	0	0
CRKL	0.030092	0.029869033	0.000222933	0.009065273	0.338456098	0.390589922	8	0	0
CCDC71	0.3651142	0.361678512	0.003435702	0.103131707	0.338481044	0.39059358	14	0	0
TBK1	0.0636178	0.064045354	-0.00042759	0.009827052	0.338712905	0.390835992	9	0	0
S100PBP	0.0764993	0.076460649	3.86E-05	-0.00343444	0.338934243	0.391066232	4	0	0

DDIT3	0.1654791	0.153093323	0.012385802	0.056770941	0.339024759	0.391145509	12	0	0
SDHC	0.0393839	0.039570582	-0.00018672	0.004725325	0.339074807	0.391178089	8	0	0
SLC25A38	0.1515433	0.152216393	-0.0006731	-0.00678381	0.339267917	0.391375699	8	0	0
CEP78	0.2468937	0.250153065	-0.0032594	0.039230892	0.339345227	0.391389364	13	0	0
MRPL41	0.0959886	0.095797935	0.000190697	0.004396736	0.339316161	0.391389364	6	0	0
TMEM121	0.2178442	0.220409638	-0.00256543	0.123495867	0.339339931	0.391389364	7	0	0
LRP3	0.0920375	0.093121635	-0.00108416	-0.01519783	0.339642332	0.391706846	3	0	0
ZC2HC1C	0.0441284	0.04399836	0.000130039	0.006065089	0.340011562	0.392107463	8	0	0
B2M	0.0828866	0.086357784	-0.00347122	0.003175136	0.340319113	0.392436905	11	0	0
ATP6V1E2	0.0554366	0.054691009	0.000745626	0.041216706	0.340678143	0.39280041	6	0	0
IRF2BP1	0.0870818	0.082905017	0.004176797	0.039528686	0.340677592	0.39280041	5	0	0
IRF2BP2	0.0348549	0.034909813	-5.50E-05	-0.00843778	0.340891207	0.392999701	8	0	0
TNKS1BP1	0.1469026	0.136574563	0.010328066	0.076065952	0.340894812	0.392999701	7	0	0
PRR7	0.1497183	0.14763205	0.00208623	0.140112114	0.341086151	0.393195013	8	0	0
PPP6R1	0.0384709	0.036131039	0.002339861	0.059772385	0.341464203	0.393605524	6	0	0
FAM117B	0.0425604	0.042874724	-0.00031432	-0.00343232	0.341631946	0.393769499	9	0	0
ELF1	0.0407352	0.040992483	-0.00025733	-0.01166968	0.341694273	0.393769499	8	0	0
NOXO1	0.0589651	0.061334829	-0.00236975	-0.05404738	0.341669581	0.393769499	6	0	0
CEP95	0.0292831	0.029834935	-0.00055188	-0.02198684	0.341689065	0.393769499	13	0	0
ADAM17	0.0346303	0.033889705	0.000740575	0.025739673	0.341757995	0.393817629	7	0	0
HERC5	0.0738719	0.069309211	0.004562699	0.195425841	0.341924022	0.393983634	11	0	0
PSIP1	0.1206951	0.119584428	0.001110664	0.003467371	0.34217643	0.394249145	5	0	0
CPSF3	0.0780282	0.077828563	0.000199634	0.018741851	0.342330015	0.394400768	11	0	0
B4GALT5	0.0816716	0.079974466	0.001697122	0.016732574	0.342356534	0.394405986	8	0	0
NAT9	0.1211918	0.128602419	-0.00741066	-0.01281948	0.342385111	0.394413574	7	0	0
CKS1B	0.0522461	0.051035227	0.001210873	0.029516812	0.342589435	0.394623602	12	0	0
LHX4	0.0356351	0.036914992	-0.00127991	-0.03690314	0.342626215	0.394640623	4	0	0
GPBP1	0.0460099	0.046196059	-0.00018619	-0.00275735	0.342857879	0.394831392	8	0	0
NETO2	0.127355	0.112758721	0.014596239	0.385281638	0.34281582	0.394831392	9	0	0
SNAP29	0.0443136	0.043875454	0.000438183	0.027108625	0.342846853	0.394831392	9	0	0
HIST1H2AE	0.1269088	0.11896412	0.007944696	0.097759057	0.34301583	0.394987925	9	0	0
YARS2	0.2282231	0.215248203	0.012974895	0.031659657	0.343067997	0.395013272	10	0	0
EPB41L3	0.2143609	0.208895362	0.005465529	0.137580252	0.343081889	0.395013272	7	0	0
CCDC82	0.1432534	0.142488744	0.000764673	0.011896806	0.343451591	0.395413553	13	0	0
ZFP36L2	0.0366357	0.033334411	0.003301277	0.107177227	0.343739468	0.395719583	24	0	0
SDF2	0.0796522	0.082724171	-0.00307193	-0.02860836	0.34378047	0.395741384	14	0	0
ID1	0.0342313	0.034774152	-0.00054286	-0.00802893	0.344047021	0.396022806	9	0	0
IFT46	0.0641725	0.060747819	0.003424699	0.049227161	0.344176967	0.39614696	4	0	0
HIST1H2BN	0.0615164	0.063046654	-0.00153027	-0.00312624	0.344264122	0.396221849	10	0	0
LRRFIP2	0.6092478	0.615605359	-0.00635759	-0.00439208	0.344388396	0.396339446	2	0	0
SMARCA5	0.0515503	0.049397299	0.002152952	0.014028016	0.34448197	0.3964217	12	0	0
HTR7	0.0767351	0.072068822	0.004666252	0.165771286	0.344545265	0.396469101	7	0	0
DISC1	0.1914099	0.177853609	0.013556264	0.143612786	0.344648606	0.396562573	8	0	0
ATXN1	0.0429214	0.044056556	-0.00113517	-0.02535932	0.344747576	0.396651005	8	0	0
WRNIP1	0.0492047	0.048979056	0.00022567	0.012057652	0.344895323	0.396795543	7	0	0
NPL	0.2703846	0.271504037	-0.00111941	0.005152173	0.345080793	0.396983459	11	0	0
EPB41L2	0.1391988	0.124293187	0.014905587	0.043794108	0.345663679	0.397628511	9	0	0
RC3H1	0.0644924	0.059928614	0.004563793	0.068968773	0.345991341	0.397979907	6	0	0
IFI35	0.4692579	0.471105414	-0.00184752	0.035290456	0.346027203	0.397995634	7	0	0
ACVR2A	0.095515	0.093827762	0.001687192	0.045842353	0.346161829	0.39809942	13	0	0
GNG5	0.0959124	0.099530171	-0.00361775	-0.01106761	0.346508592	0.398472662	9	0	0
PRMT1	0.5956228	0.577290482	0.018332341	0.307286862	0.346688206	0.398653652	3	0	0
SMC2	0.128302	0.134220389	-0.00591839	-0.03825943	0.346855754	0.398820746	10	0	0
NUP93	0.0563569	0.059256291	-0.00289935	-0.02061084	0.346887771	0.398831991	10	0	0
TCEANC2	0.0493982	0.048700833	0.000697347	0.005201654	0.347139297	0.399095598	16	0	0
PI4KB	0.1027701	0.102716587	5.35E-05	0.009272707	0.347247768	0.399194717	14	0	0
AUTS2	0.0681089	0.062943707	0.005165208	0.071058331	0.347348236	0.399284622	9	0	0
MRPL55	0.1515428	0.144930865	0.006611954	0.048125547	0.347583421	0.399529367	15	0	0
PLK3	0.064231	0.059958656	0.004272372	0.03566857	0.34781566	0.399745077	8	0	0
NFRKB	0.0517196	0.04693059	0.004789023	0.075490793	0.347793596	0.399745077	5	0	0
TTC7A	0.037944	0.038367904	-0.00042391	0.001825899	0.348299319	0.400274714	11	0	0
TIGD1	0.0845591	0.086173473	-0.0016144	-0.00809142	0.348321127	0.400274714	14	0	0
CAMK2N2	0.1023232	0.095325257	0.006997922	0.132593713	0.348350512	0.400282836	9	0	0

CEBPD	0.3041778	0.30191435	0.00226341	0.044078925	0.348517166	0.400448679	6	0	0
DNPH1	0.1474092	0.154526671	-0.00711743	-0.01917298	0.348616272	0.400536894	8	0	0
ESRRA	0.0351055	0.036170633	-0.00106508	-0.02636511	0.348788954	0.400709626	9	0	0
GDPGP1	0.0683098	0.068261367	4.84E-05	0.016307431	0.348835534	0.400737471	7	0	0
TAF8	0.2898281	0.291179709	-0.0013516	0.039228411	0.349207602	0.401139205	9	0	0
PYROXD1	0.1529571	0.15895931	-0.00600222	-0.03064472	0.349271141	0.4011865	8	0	0
FOXRED1	0.0804611	0.083992353	-0.00353126	-0.00709356	0.349459488	0.401377137	14	0	0
IBA57	0.1876541	0.189154	-0.00149991	0.020691373	0.349520761	0.401421807	11	0	0
MZT2A	0.0297261	0.028642572	0.00108352	0.03465459	0.349644924	0.401538696	2	0	0
BRD2	0.0575793	0.057018798	0.000560536	0.0082322	0.350277608	0.402239527	17	0	0
CENPK	0.0393083	0.038034726	0.001273581	0.013101786	0.350306842	0.402247344	8	0	0
THYN1	0.0460419	0.045587387	0.000454522	0.034069909	0.350531989	0.402454343	7	0	0
ACAD8	0.0460419	0.045587387	0.000454522	0.034069909	0.350531989	0.402454343	7	0	0
PET117	0.0683606	0.072113754	-0.00375317	-0.01555651	0.3506666	0.402583123	12	0	0
METTL1	0.2352893	0.23737433	-0.00208504	0.036098582	0.35144191	0.403447395	10	0	0
TMEM68	0.0502423	0.047821538	0.002420764	0.043457885	0.351562368	0.403552054	18	0	0
TRIM44	0.212418	0.207660532	0.004757506	0.078613137	0.351578077	0.403552054	7	0	0
GMFG	0.0440535	0.04331125	0.000742237	0.014438317	0.3516521	0.40361119	5	0	0
FASTKD2	0.0324875	0.033833221	-0.00134573	-0.05192917	0.351890248	0.403858682	10	0	0
GLS	0.0481411	0.047940047	0.000201095	0.034139917	0.351988821	0.403945966	7	0	0
RGMB	0.0429422	0.038857071	0.004085112	0.110684748	0.352348143	0.404306589	7	0	0
VPS51	0.8445951	0.857404125	-0.01280906	-0.02232778	0.352346433	0.404306589	7	0	0
BPGM	0.1253762	0.133928527	-0.00855231	0.01374324	0.352677716	0.404658874	8	0	0
PRPF40B	0.1309095	0.133135974	-0.0022265	-0.00591615	0.352746706	0.404712142	10	0	0
IGHMBP2	0.081198	0.086306992	-0.005109	-0.04453029	0.352906998	0.404870149	6	0	0
ZFYVE9	0.0556569	0.054704641	0.000952236	0.029550432	0.352930286	0.404870969	11	0	0
BZW2	0.0411032	0.041413169	-0.00031	-0.0122053	0.353039843	0.404970748	15	0	0
CCNC	0.2778474	0.279910606	-0.00206322	-0.02976311	0.353087719	0.404999765	7	0	0
RAB10	0.02757	0.02746211	0.000107918	-0.0052273	0.353241241	0.405149949	3	0	0
DPP3	0.0745498	0.069864707	0.004685061	0.065568505	0.35357405	0.405505733	8	0	0
SRP14	0.0757218	0.071489072	0.00423271	0.070737831	0.353723643	0.40565136	11	0	0
COX7C	0.2229023	0.224976834	-0.00207457	0.016549663	0.353804675	0.405718346	11	0	0
AIDA	0.0340863	0.03318324	0.000903101	0.026875586	0.354072148	0.405999108	10	0	0
CELSR2	0.1822443	0.185279259	-0.00303492	-0.01570412	0.354162027	0.406065345	10	0	0
DPH3	0.1460409	0.152626832	-0.00658588	-0.00737099	0.354175193	0.406065345	7	0	0
ATP6V1B2	0.0677067	0.064457708	0.003249012	0.034771313	0.354213195	0.406082957	7	0	0
ZNF691	0.037814	0.037280663	0.000533325	0.020462221	0.354260367	0.406085125	8	0	0
SNX17	0.0383302	0.03784009	0.000490061	0.010019872	0.354238554	0.406085125	14	0	0
ZNF224	0.0679183	0.065300965	0.002617322	0.05402104	0.354362016	0.406175685	12	0	0
SUGT1	0.0262297	0.02546752	0.000762165	0.033341456	0.354412958	0.406208116	5	0	0
UBE2O	0.2285722	0.230601463	-0.00202929	-0.01377088	0.35443992	0.406213061	8	0	0
NFKB1	0.1192365	0.123289362	-0.00405287	-0.01709497	0.354598753	0.406343166	10	0	0
CRLF1	0.21453	0.202939366	0.011590618	0.112433726	0.354737048	0.406475672	6	0	0
CDC7	0.0298685	0.026300092	0.003568423	0.153775086	0.355102026	0.406848481	17	0	0
EIF3H	0.0336459	0.034224552	-0.00057869	-0.02212667	0.355107771	0.406848481	5	0	0
RMDN2	0.7565784	0.756752703	-0.00017434	0.002775829	0.355984572	0.407826985	2	0	0
ERP29	0.0348301	0.034558925	0.000271147	0.009459598	0.356248253	0.408103	10	0	0
HRASLS	0.2058171	0.200546982	0.005270166	0.077482973	0.356436934	0.408293067	9	0	0
LRRC40	0.0341125	0.033647058	0.000465423	0.010453757	0.356514535	0.408355878	16	0	0
CD3D	0.6773929	0.677511884	-0.00011896	0.001007418	0.356572328	0.408369917	8	0	0
SLC46A3	0.1107026	0.108303981	0.002398654	0.190045915	0.356557768	0.408369917	11	0	0
KLC1	0.5226125	0.522392331	0.000220184	0.007152616	0.356682252	0.408469727	7	0	0
CRYGN	0.502283	0.498533072	0.003749964	0.011297704	0.357225026	0.40903928	7	0	0
MOB3B	0.493442	0.49838265	-0.00494067	-0.00677926	0.357225205	0.40903928	7	0	0
MAGI3	0.0528488	0.050688506	0.002160267	0.031179832	0.35737292	0.409182297	13	0	0
JAKMIP2	0.183894	0.171410015	0.012483976	0.229415433	0.357729065	0.409563929	8	0	0
LRWD1	0.1301499	0.130429378	-0.0002795	0.010409561	0.357780276	0.409570273	9	0	0
PDCD4	0.18459	0.186887467	-0.00229744	-0.01371952	0.357764884	0.409570273	15	0	0
ARMC7	0.2063364	0.196825154	0.009511243	0.107083377	0.35780341	0.409570615	5	0	0
CGN	0.3297518	0.313305359	0.016446451	0.091763615	0.358077479	0.409858179	4	0	0
CDK11A	0.7111216	0.745399046	-0.03427744	-0.0669964	0.358231889	0.410008753	1	0	0
MXRA7	0.2627944	0.256490565	0.006303853	0.08270552	0.358464407	0.410248698	5	0	0
SCAF11	0.1239523	0.121092029	0.002860278	0.041940428	0.358596045	0.410373168	8	0	0

COG7	0.1000851	0.103023718	-0.00293858	-0.00509735	0.358629111	0.410384824	14	0	0
TRIM25	0.0340757	0.034141933	-6.63E-05	-0.00726542	0.358711993	0.410453481	12	0	0
CCDC167	0.1521113	0.157258029	-0.00514676	-0.0136802	0.358954523	0.410704793	11	0	0
FBXW7	0.0468121	0.048104985	-0.00129292	-0.03782929	0.359485549	0.41128614	4	0	0
RAPH1	0.0946601	0.094668037	-7.94E-06	-0.00149061	0.359512827	0.411291113	7	0	0
MVK	0.1026633	0.100050107	0.002613178	-0.00853008	0.359575049	0.41133606	12	0	0
POLB	0.03939	0.038264657	0.001125348	0.031890002	0.36003106	0.411831447	10	0	0
MGST3	0.0445971	0.043644156	0.000952946	0.020586003	0.360155639	0.411947678	6	0	0
ASB13	0.156661	0.165051719	-0.00839072	-0.00094159	0.360287509	0.412072234	5	0	0
DGKA	0.0324199	0.032644556	-0.00022468	0.002077065	0.360418333	0.412195576	11	0	0
POLR1D	0.0612636	0.057758123	0.003505486	0.058010434	0.36044421	0.412198887	6	0	0
RTN4IP1	0.0978607	0.105933293	-0.00807255	-0.02863466	0.360469355	0.412201362	9	0	0
ORMDL3	0.2182252	0.205296005	0.012929169	0.072923864	0.360631251	0.412360201	11	0	0
DNAJB9	0.0416926	0.041640013	5.26E-05	-0.01197733	0.360810564	0.412538936	8	0	0
TAOK3	0.1476297	0.154333891	-0.00670423	-0.00039692	0.360850889	0.412558742	11	0	0
MRPL17	0.0368767	0.037055166	-0.0001785	-0.00036489	0.361068001	0.412780653	9	0	0
QTRT1	0.0506924	0.039079179	0.011613177	0.358787646	0.361130684	0.412826001	9	0	0
ZNF367	0.025121	0.022938907	0.002182071	0.117699328	0.361211811	0.412892425	9	0	0
GADL1	0.1535974	0.143839376	0.009758044	0.118960086	0.361391149	0.413071096	10	0	0
POP1	0.0309163	0.030768443	0.000147836	0.004398048	0.362003787	0.413744977	10	0	0
STIM2	0.0409389	0.040935967	2.96E-06	0.008651247	0.362157589	0.413875932	6	0	0
COQ9	0.1861553	0.190705026	-0.0045497	-0.00669903	0.362164515	0.413875932	8	0	0
SNCAIP	0.3889736	0.397779119	-0.00880548	-0.01421244	0.362212732	0.413904661	8	0	0
NUDT5	0.0519083	0.050884702	0.001023565	0.01369754	0.36228599	0.413962	15	0	0
DENND2C	0.0575978	0.056034498	0.001563288	0.038980132	0.362397861	0.41406345	15	0	0
USP2	0.0539682	0.056059425	-0.00209126	-0.0455453	0.362531446	0.414189694	8	0	0
PRRC2B	0.0684005	0.066486873	0.001913641	0.035457774	0.363039348	0.414743551	2	0	0
TAF11	0.0399096	0.039733036	0.000176582	0.006080854	0.36322637	0.41493078	19	0	0
ZG16B	0.5103834	0.522712388	-0.01232896	-0.03725053	0.363266637	0.41495035	8	0	0
CHCHD4	0.0375136	0.036383683	0.001129941	0.031223749	0.363429044	0.415109427	12	0	0
HMGN3	0.2725306	0.273282617	-0.00075203	-0.00090999	0.363679378	0.415368908	12	0	0
TNFSF4	0.4034151	0.40815098	-0.00473591	-0.00618212	0.363838054	0.415523676	9	0	0
CAMK2D	0.0495483	0.049334697	0.000213587	0.004444531	0.363999137	0.415681175	9	0	0
THAP8	0.0607126	0.063704331	-0.00299175	-0.03698872	0.364138242	0.415813555	12	0	0
DCTD	0.0708476	0.068151816	0.002695833	0.100644054	0.364253012	0.415865183	10	0	0
SLC30A4	0.037352	0.034585713	0.002766286	0.081141947	0.364213795	0.415865183	10	0	0
APOBEC3B	0.1377109	0.129613838	0.0080971	0.176003882	0.364233733	0.415865183	3	0	0
ZNF746	0.3272306	0.325551549	0.001679018	0.004298618	0.364500444	0.416121187	5	0	0
RUSC1	0.3236635	0.298075753	0.025587735	0.146040763	0.365228125	0.416925385	6	0	0
SLAIN2	0.1584575	0.154634689	0.003822845	-0.00924416	0.365255003	0.416929532	6	0	0
SPSB2	0.4843637	0.485171244	-0.00080754	0.036310752	0.366096659	0.417863668	2	0	0
TXNDC17	0.0495318	0.05036244	-0.00083069	-0.01246167	0.366318536	0.418090313	8	0	0
ESD	0.1112734	0.112988157	-0.00171474	-0.01910789	0.366356461	0.418106992	10	0	0
SLC2A12	0.3095852	0.304332497	0.005252744	0.010510897	0.366793265	0.418578864	9	0	0
TMBIM6	0.0460253	0.042398603	0.003626654	0.056320538	0.367043125	0.418837351	5	0	0
THAP5	0.0360726	0.035577374	0.000495253	0.003992672	0.367087993	0.418861902	10	0	0
ARRDC2	0.2309132	0.227679629	0.003233574	0.032417323	0.367134893	0.418888769	7	0	0
RNF207	0.1031222	0.099585343	0.003536844	0.043828218	0.367203799	0.418940738	10	0	0
EIF4A3	0.1360802	0.141132835	-0.00505259	-0.00388918	0.367321083	0.419047891	8	0	0
ZNF585B	0.1637597	0.161676441	0.002083271	0.225878276	0.367401441	0.419112907	7	0	0
LNPEP	0.0392882	0.039376266	-8.81E-05	0.007862732	0.367618275	0.419333591	10	0	0
SLC4A11	0.0803689	0.079227907	0.001140997	0.013676702	0.368488048	0.420298991	5	0	0
ZSCAN32	0.0815003	0.077151129	0.004349147	0.05131084	0.368530711	0.420320923	7	0	0
SLFN5	0.1276868	0.133206305	-0.00551954	-0.00634611	0.368651088	0.420431482	15	0	0
COA5	0.1686335	0.165440576	0.003192947	0.017048028	0.369202115	0.421008072	12	0	0
SLC22A23	0.0879101	0.088000031	-8.99E-05	0.023968291	0.369203611	0.421008072	4	0	0
SMC3	0.0476977	0.048207941	-0.0005102	-0.01994755	0.369307399	0.42109965	10	0	0
ENY2	0.0487637	0.049182927	-0.00041924	-0.01189447	0.369339935	0.421109978	14	0	0
KCNC4	0.0362307	0.034472114	0.001758616	0.030270155	0.369462501	0.421222948	14	0	0
COPA	0.267515	0.267980794	-0.00046575	0.016040525	0.369700952	0.421414444	11	0	0
C11orf71	0.0369386	0.038085927	-0.00114728	-0.0342907	0.369675929	0.421414444	13	0	0
MRPS35	0.0407933	0.04061709	0.000176218	0.012092084	0.369681566	0.421414444	7	0	0
ZFP57	0.7026247	0.667033041	0.035591687	0.075125066	0.370219341	0.421978527	8	0	0

ATP6V1A	0.038427	0.038106475	0.000320519	0.005485545	0.370294209	0.422037042	12	0	0
MFN1	0.0604039	0.061059388	-0.00065545	-0.00048682	0.370774023	0.422557051	7	0	0
UBE2W	0.0590854	0.053592958	0.005492459	0.061417626	0.371402582	0.423246503	10	0	0
GTPBP1	0.0355437	0.033944602	0.001599067	0.060537031	0.371619805	0.423467143	8	0	0
ZNF672	0.1461271	0.147730065	-0.00160299	-0.01224596	0.37200097	0.423874557	10	0	0
CIC	0.1205865	0.116729544	0.003856986	0.015477576	0.372065865	0.42392157	6	0	0
ARFGEF1	0.0489963	0.049497017	-0.00050068	-0.01108515	0.372287571	0.424147232	9	0	0
KDM7A	0.0942049	0.093924658	0.000280227	0.010416872	0.372821259	0.424728284	6	0	0
DNAJC25	0.0539935	0.056154779	-0.00216129	-0.04117877	0.372922703	0.424816869	7	0	0
TEF	0.2277137	0.221251752	0.006461967	0.078485787	0.372977309	0.42485209	12	0	0
HIST1H3B	0.0469588	0.044440648	0.002518149	0.071080574	0.373371263	0.425273828	7	0	0
UBA52	0.0593148	0.059783244	-0.00046847	0.006617295	0.373401922	0.425281742	6	0	0
RRM1	0.0599471	0.059141528	0.000805523	0.046660781	0.373475123	0.425338104	11	0	0
KCNH8	0.065688	0.058230006	0.007457953	0.13700294	0.373574381	0.425417039	9	0	0
ENO3	0.1048031	0.105388023	-0.00058488	-0.0002541	0.37359187	0.425417039	6	0	0
STRN	0.0415475	0.039128387	0.002419075	0.060358814	0.373787004	0.42561222	5	0	0
EPS8	0.0458129	0.046891414	-0.00107849	-0.00938279	0.373844367	0.425650515	8	0	0
DDX10	0.0458197	0.044856329	0.000963367	0.007554777	0.374038458	0.42584447	11	0	0
IGSF8	0.0525003	0.050348194	0.002152128	0.031853008	0.374096757	0.425869607	11	0	0
PM20D2	0.1005252	0.108919636	-0.00839445	-0.02088342	0.374203	0.425869607	9	0	0
PSMB1	0.0543419	0.053257652	0.001084237	0.006701765	0.374191494	0.425869607	14	0	0
TBP	0.0543419	0.053257652	0.001084237	0.006701765	0.374191494	0.425869607	14	0	0
CPNE1	0.0726301	0.075215623	-0.00258555	-0.03105925	0.374154191	0.425869607	7	0	0
RBM12	0.0726301	0.075215623	-0.00258555	-0.03105925	0.374154191	0.425869607	7	0	0
SYT1	0.0748097	0.07457845	0.000231201	0.044918739	0.374441678	0.426114201	8	0	0
CSE1L	0.1518793	0.153475778	-0.00159648	-0.02303538	0.374801804	0.426496964	8	0	0
DARS2	0.04665	0.046549021	0.000100961	0.007669239	0.37485748	0.426533258	16	0	0
TUBA1B	0.0345362	0.034653719	-0.00011755	1.67E-05	0.375147862	0.426809518	14	0	0
WDSUB1	0.0351087	0.032710768	0.002397952	0.050879753	0.375546633	0.427236104	5	0	0
RBM26	0.0305884	0.030959917	-0.00037149	-0.01324857	0.37584168	0.427544642	7	0	0
CCT2	0.2298742	0.233145128	-0.00327092	0.016910611	0.375980483	0.427675415	11	0	0
NUDCD1	0.0500921	0.050424058	-0.00033193	-0.00872847	0.376106088	0.42779116	13	0	0
UBE2E2	0.1258321	0.12382177	0.002010366	0.044179605	0.376254024	0.427932287	15	0	0
RPH3AL	0.0481114	0.044125116	0.003986272	0.076179586	0.376345681	0.428009393	2	0	0
HAS2	0.0891513	0.085045844	0.004105471	0.098922486	0.376410799	0.428056308	10	0	0
DOCK4	0.0253203	0.024556359	0.000763963	0.027541968	0.376690741	0.4283475	10	0	0
RPUSD1	0.2079585	0.210726486	-0.00276798	0.020779827	0.376765233	0.428405048	6	0	0
PIGN	0.0361409	0.035235232	0.00090571	0.036515965	0.376837341	0.428459876	10	0	0
WBP2	0.0398159	0.039485085	0.000330767	0.005747066	0.376963147	0.428575748	2	0	0
PRKD3	0.0458322	0.044296265	0.001535937	0.043391299	0.377005886	0.428597172	6	0	0
AMZ2	0.0430845	0.042161032	0.000923448	0.007932265	0.377507216	0.429139905	6	0	0
TPGS2	0.1103997	0.113707219	-0.00330752	-0.0209318	0.37769494	0.429326095	9	0	0
MEX3B	0.0509721	0.050355647	0.00061647	0.016452814	0.377784299	0.429400455	11	0	0
MRPL16	0.278487	0.280860262	-0.00237328	-0.01917338	0.377900674	0.429505512	7	0	0
PCGF2	0.0398642	0.035785344	0.004078906	0.142035585	0.378106524	0.429712243	4	0	0
ATR	0.0977117	0.100475164	-0.00276345	0.005668361	0.378245072	0.429842464	13	0	0
MDH2	0.0301992	0.030089831	0.000109376	0.006343466	0.378447691	0.430045476	15	0	0
RGL2	0.0527085	0.048464614	0.004243874	0.103529836	0.378730817	0.430339939	38	0	0
KERA	0.6396755	0.669748867	-0.03007341	-0.06155544	0.379029204	0.430651704	2	0	0
BANF1	0.0378238	0.038441281	-0.00061751	-0.00595786	0.379095531	0.430699781	13	0	0
TDRD3	0.0550487	0.053617405	0.001431277	0.021085865	0.379146389	0.430730278	9	0	0
CRYZ	0.109178	0.11001268	-0.00083466	-0.00315426	0.379242853	0.430812579	10	0	0
BDH2	0.1255396	0.122978323	0.002561279	-0.00477192	0.379468745	0.431041888	3	0	0
MSMO1	0.1219754	0.125217456	-0.00324203	0.000802836	0.379590778	0.431142994	10	0	0
GPR63	0.0985544	0.095361887	0.003192493	0.122780096	0.37960583	0.431142994	9	0	0
GPR180	0.188876	0.181840534	0.007035511	0.027556245	0.379805881	0.431315581	9	0	0
NUBP2	0.0878166	0.090254265	-0.00243766	-0.01207756	0.379789521	0.431315581	14	0	0
GCHFR	0.0407992	0.037493007	0.003306163	0.109660228	0.379956529	0.431459342	7	0	0
DNAAF1	0.0817434	0.075896467	0.005846944	0.069548972	0.38013967	0.431639979	12	0	0
SLC7A8	0.1217438	0.10268133	0.019062488	0.332541968	0.380542705	0.432070264	8	0	0
FBXO41	0.0662645	0.056034681	0.010229817	0.256205375	0.380597706	0.432105358	5	0	0
GOSR2	0.0303299	0.030257476	7.24E-05	0.003735221	0.380739754	0.43223927	7	0	0
TTC32	0.0422048	0.043151155	-0.0009464	-0.00905608	0.380824215	0.432280433	13	0	0

STX11	0.0324765	0.031495125	0.000981414	0.025403768	0.380817703	0.432280433	5	0	0
NTHL1	0.112152	0.112495489	-0.00034347	-0.00469515	0.381150441	0.432593134	12	0	0
PTRH2	0.0711645	0.069020986	0.002143552	0.015002129	0.38117205	0.432593134	12	0	0
NXT1	0.1226275	0.114863601	0.007763879	0.092325798	0.381158235	0.432593134	7	0	0
THAP4	0.0417953	0.042684124	-0.00088884	-0.02843928	0.381494355	0.432931525	7	0	0
GADD45G	0.0530539	0.049424822	0.003629063	0.098385173	0.381692116	0.433128546	6	0	0
TMEM43	0.0407054	0.039949371	0.000755994	0.026084357	0.382264299	0.433722955	11	0	0
C6orf141	0.2642904	0.268381422	-0.004091	0.043334041	0.382259548	0.433722955	8	0	0
HBA2	0.4964084	0.519902197	-0.02349378	-0.06542472	0.382651697	0.43413504	1	0	0
ZNF277	0.0265735	0.025994767	0.000578703	0.021679107	0.382723528	0.43418907	11	0	0
ADAP2	0.0533985	0.048845632	0.004552849	0.138910655	0.38313667	0.434630278	10	0	0
SAP30	0.0554722	0.054632726	0.000839464	0.014398009	0.383325981	0.434801726	8	0	0
PPP2R1A	0.0728108	0.06852901	0.004281769	0.022241037	0.38333629	0.434801726	10	0	0
NT5C3A	0.1393368	0.141477723	-0.00214088	-0.00205506	0.383509678	0.434970886	9	0	0
CCDC146	0.0295149	0.027579272	0.001935664	0.072460951	0.383543394	0.43498162	1	0	0
USPL1	0.046852	0.047223678	-0.00037167	-0.0111482	0.383679785	0.43510879	7	0	0
BHLHA15	0.2811875	0.283455175	-0.00226766	-0.00590122	0.383814734	0.435224713	4	0	0
HNRNPL	0.0529068	0.052673995	0.000232763	-0.00920612	0.383830537	0.435224713	4	0	0
GTF3A	0.1767834	0.173733141	0.003050224	0.036200833	0.384348151	0.435784085	8	0	0
CLOCK	0.6563582	0.661855915	-0.00549773	-0.01174992	0.384397672	0.435807061	4	0	0
UBE2QL1	0.3078214	0.300399576	0.007421872	0.238523509	0.384417011	0.435807061	6	0	0
CES1	0.3812214	0.356811428	0.02441001	0.115671085	0.384460893	0.435829261	2	0	0
HSDL2	0.189764	0.207049702	-0.01728566	-0.03301125	0.384514063	0.435861988	3	0	0
NCS1	0.171432	0.173692637	-0.00226067	0.011848316	0.384761212	0.436114579	9	0	0
CEP70	0.039382	0.038137487	0.001244558	0.030991141	0.384874625	0.436215562	13	0	0
SLC9A4	0.0583832	0.060283062	-0.00189985	-0.0395349	0.38524707	0.4366101	1	0	0
GIN1	0.0516657	0.050341835	0.001323829	0.028176225	0.385430948	0.436790894	14	0	0
CD46	0.1201522	0.123216855	-0.00306464	0.002789636	0.385574499	0.436925968	11	0	0
TUBE1	0.0691403	0.068665437	0.000474826	-0.01985129	0.385845134	0.437205024	15	0	0
NIPAL1	0.0606408	0.062875604	-0.00223483	-0.01605762	0.385903506	0.437243543	7	0	0
SKIDA1	0.0547653	0.055830561	-0.00106523	0.022048874	0.38598658	0.437310044	13	0	0
TUT1	0.1657333	0.16744434	-0.00171101	0.020624489	0.386029102	0.437330595	9	0	0
CSTF2T	0.0446568	0.042771178	0.00188564	0.047727134	0.386233207	0.437506556	10	0	0
SPATA17	0.1415377	0.147918961	-0.00638124	-0.00782776	0.386766836	0.438055694	11	0	0
TMEM243	0.1702923	0.180140377	-0.00984804	-0.00460495	0.386751651	0.438055694	8	0	0
OR4Q3	0.3852084	0.407169274	-0.02196089	-0.07801935	0.387002956	0.438295448	1	0	0
ASB4	0.425257	0.423016411	0.002240543	0.019611896	0.387037358	0.438306734	7	0	0
HIST1H1D	0.0596247	0.056095364	0.00352932	0.08518116	0.387178596	0.438438999	5	0	0
LSM1	0.0315148	0.030898368	0.000616455	0.024003471	0.387250166	0.438492359	10	0	0
RNF43	0.3049874	0.301263083	0.003724353	0.031964692	0.387449307	0.438690157	9	0	0
KANSL2	0.1040464	0.106481013	-0.00243459	-0.00892407	0.387556008	0.438783269	11	0	0
IL15RA	0.3473228	0.335329155	0.011993613	0.045485267	0.387649533	0.438861455	7	0	0
ATAD2B	0.0413929	0.041637932	-0.00024504	0.015184702	0.388142271	0.439354006	5	0	0
UBXN2A	0.0413929	0.041637932	-0.00024504	0.015184702	0.388142271	0.439354006	5	0	0
IDO1	0.793854	0.804335044	-0.01048102	-0.01868894	0.388158094	0.439354006	1	0	0
MPC1	0.09638	0.093687276	0.002692679	0.021005647	0.388200787	0.439374601	25	0	0
CNIH3	0.0394967	0.040582275	-0.0010856	-0.01477839	0.388235572	0.439386245	12	0	0
WDR89	0.0368828	0.036049292	0.000833472	0.027903824	0.388328441	0.43946362	6	0	0
FBXL14	0.0331257	0.034016799	-0.00089107	-0.02775584	0.388552189	0.439670418	6	0	0
MBD6	0.042625	0.042153026	0.00047196	0.006274374	0.388560203	0.439670418	8	0	0
FAM214B	0.0565501	0.055996264	0.000553831	0.011339176	0.388814789	0.439930737	5	0	0
ISCA2	0.0489397	0.048568428	0.000371294	0.007401088	0.388973161	0.440082169	13	0	0
SBDS	0.0565961	0.057401767	-0.00080563	-0.0051631	0.389186996	0.440296327	6	0	0
PRKCH	0.7210578	0.734351476	-0.01329371	-0.0259986	0.389481928	0.440602199	1	0	0
IL4R	0.232652	0.234380217	-0.00172818	0.01642196	0.389613806	0.440723589	8	0	0
EIF2AK3	0.0508175	0.051941093	-0.00112359	-0.02015619	0.389854543	0.440968095	11	0	0
BAMBI	0.2112589	0.206677015	0.004581909	0.127829784	0.390303638	0.441448231	5	0	0
RDX	0.1913114	0.195525653	-0.00421424	-1.19E-05	0.390333582	0.441454261	7	0	0
RPL13	0.0409096	0.040385139	0.000524448	0.006092297	0.390419475	0.441523563	9	0	0
MAP2K5	0.0453026	0.043865071	0.001437486	0.031465357	0.390495136	0.441581285	10	0	0
TP53BP2	0.0808033	0.084607603	-0.00380426	-0.022174	0.390689919	0.441764992	7	0	0
FKBP14	0.0423969	0.042318629	7.83E-05	-0.00124421	0.390706851	0.441764992	14	0	0
CALU	0.0374641	0.036943419	0.000520661	0.015586247	0.39138784	0.442507079	10	0	0

NAA50	0.0328386	0.031961022	0.000877616	0.019916299	0.391455095	0.442555221	12	0	0
RALGAPA2	0.041555	0.041965612	-0.00041061	-0.01178259	0.391601131	0.442692417	6	0	0
MMAB	0.0334101	0.03397351	-0.00056345	-0.01508305	0.391733617	0.442814279	11	0	0
HSPA13	0.0375344	0.037939449	-0.00040506	-0.01329471	0.391887943	0.442960811	14	0	0
MTFR2	0.0527078	0.052026806	0.000680987	0.011195054	0.39224744	0.443339221	12	0	0
TRIB1	0.0669248	0.064222523	0.002702321	0.021620062	0.392413632	0.443499111	10	0	0
FAM229B	0.0534614	0.054562938	-0.00110151	-0.03029846	0.392439993	0.443500959	15	0	0
GATM	0.0467035	0.04713856	-0.00043511	-0.00341771	0.392496199	0.443536531	8	0	0
ALG5	0.0390416	0.038087057	0.000954553	0.020581089	0.393032259	0.44411432	11	0	0
RHOG	0.0428952	0.04324909	-0.00035387	-0.00631821	0.393065952	0.444124411	7	0	0
MARCKSL1	0.18222	0.18498482	-0.00276483	-0.02899273	0.393304979	0.444358563	8	0	0
PNPLA8	0.0377341	0.037511645	0.000222458	-0.00138001	0.393322733	0.444358563	11	0	0
IRAK4	0.0478375	0.04863037	-0.0007929	-0.00538146	0.394100051	0.445208699	15	0	0
STARD5	0.09958	0.100858245	-0.00127826	0.006445081	0.394545307	0.445683627	9	0	0
NFIL3	0.0364987	0.031586744	0.004911955	0.203636901	0.394608292	0.445726704	9	0	0
PRKCI	0.0589234	0.056999292	0.001924129	0.030090889	0.394728211	0.445834081	4	0	0
M6PR	0.054021	0.055972102	-0.00195111	-0.02589185	0.395083281	0.446178928	11	0	0
SLC39A7	0.122158	0.1245134	-0.00235538	-0.01559837	0.395301789	0.446397588	29	0	0
PRKRIP1	0.0495121	0.047030518	0.00248157	0.061448603	0.395358694	0.446433741	1	0	0
SENP1	0.1569652	0.161066298	-0.00410113	-0.01012068	0.395600383	0.446678532	9	0	0
SLC6A13	0.4191472	0.407553829	0.0115934	0.042097451	0.395723859	0.446789823	10	0	0
IMPDH1	0.0363694	0.035831465	0.000537945	0.023457101	0.395892573	0.446952174	10	0	0
NUDT16	0.2045813	0.2017662	0.002815149	0.05721727	0.396080446	0.447136133	6	0	0
HIST1H4H	0.043532	0.042961444	0.000570555	0.017897778	0.396306982	0.447363713	8	0	0
REN	0.5863697	0.601301332	-0.01493166	-0.03292247	0.396375869	0.447396461	6	0	0
RAB6C	0.5581856	0.575227199	-0.01704156	-0.04263446	0.396389888	0.447396461	1	0	0
PINX1	0.3076804	0.316452073	-0.00877163	-0.02586224	0.396410825	0.447396461	6	0	0
PPP1R11	0.0988814	0.098015155	0.000866253	0.025131834	0.397563663	0.448669342	17	0	0
PIGO	0.174643	0.176208733	-0.00156572	-0.01485373	0.397598188	0.448680074	8	0	0
NAGA	0.1096295	0.111870577	-0.0022411	-0.0102261	0.397644819	0.448704465	7	0	0
RAB1A	0.1003685	0.100302861	6.57E-05	0.018466949	0.397721237	0.448760516	14	0	0
RGP1	0.1061866	0.102276628	0.003909968	-0.00718608	0.397744532	0.448760516	4	0	0
ITPKB	0.1552361	0.153360944	0.001875182	0.058571594	0.397943195	0.448956419	11	0	0
NPHP4	0.0492429	0.049335506	-9.26E-05	0.011764209	0.398073554	0.449075241	12	0	0
CTTNBP2NL	0.0281311	0.02754239	0.000588692	0.025369494	0.398148232	0.449102993	7	0	0
ZNF202	0.132477	0.130688329	0.001788691	0.016654255	0.398133495	0.449102993	9	0	0
AUP1	0.0836074	0.084597316	-0.00098993	-0.01431728	0.398223566	0.44915972	10	0	0
PFKP	0.3484688	0.339011738	0.009457032	0.055685123	0.398394283	0.449295766	2	0	0
PUS1	0.0324546	0.030126255	0.002328355	0.094876274	0.398373538	0.449295766	5	0	0
IL36RN	0.7121669	0.701939379	0.010227516	0.020617414	0.398577532	0.449474167	2	0	0
SUCLG1	0.2725987	0.277987423	-0.00538873	-0.03241965	0.398907275	0.449817734	7	0	0
MRPS16	0.10246	0.102560231	-0.00010022	-0.00774621	0.399863572	0.450867733	12	0	0
ARL8A	0.0553574	0.056173027	-0.00081559	-0.00996702	0.40031293	0.451346034	9	0	0
FARS2	0.0312071	0.030569102	0.000638025	0.00720531	0.400562819	0.451571007	5	0	0
DZANK1	0.0554825	0.056465418	-0.00098288	-0.02495679	0.400555315	0.451571007	11	0	0
C11orf24	0.062342	0.062440689	-9.87E-05	-0.00507199	0.400600996	0.451585662	7	0	0
TMEM65	0.1712834	0.174947753	-0.00366439	0.041269611	0.400684813	0.451623378	6	0	0
MAP4K1	0.0609783	0.058927244	0.002051056	0.080544414	0.400666255	0.451623378	5	0	0
GLCCI1	0.0471091	0.047971036	-0.00086189	0.007397817	0.400935643	0.451877699	10	0	0
DDX41	0.0472266	0.047911939	-0.00068531	0.011693466	0.401476089	0.45245838	9	0	0
ING3	0.0692253	0.073780177	-0.00455489	0.002034118	0.401518042	0.45247723	11	0	0
METTL15	0.0761385	0.077483157	-0.00134465	-0.01547063	0.401769104	0.452731712	13	0	0
EIF5B	0.0407427	0.039253472	0.001489234	0.030792914	0.401988006	0.452949923	5	0	0
RWDD1	0.1137599	0.116335414	-0.00257548	-0.0397261	0.402196234	0.453156081	14	0	0
IPP	0.2780906	0.276306439	0.001784208	0.086604694	0.402232862	0.453168882	11	0	0
ARL6	0.1674374	0.16904482	-0.00160739	-0.02319537	0.402321189	0.453228141	15	0	0
C4orf33	0.0375899	0.038382794	-0.00079292	-0.01662251	0.402335998	0.453228141	11	0	0
ANXA4	0.0432125	0.041185101	0.002027359	0.058155053	0.402438372	0.453314993	12	0	0
FLRT3	0.1700959	0.165387579	0.004708287	0.137967753	0.402573262	0.45343846	7	0	0
PAK4	0.1909714	0.196260177	-0.00528878	0.011689408	0.402651718	0.45349835	10	0	0
CDK13	0.0579878	0.060166106	-0.00217829	-0.02949021	0.402945849	0.453801127	8	0	0
COL7A1	0.2221697	0.221521115	0.00064859	0.057327995	0.403060939	0.453902242	11	0	0
TMEM141	0.0402	0.039769783	0.000430187	0.011963632	0.40311637	0.453936164	5	0	0

IER2	0.0816561	0.080318352	0.001337763	0.045858805	0.403466182	0.454301554	13	0	0
ELP2	0.0468118	0.047629727	-0.00081796	-0.00517519	0.40366485	0.45449672	16	0	0
DNAJC30	0.0327989	0.032934662	-0.00013578	0.004729764	0.403764308	0.454580166	7	0	0
CKLF	0.2105195	0.20111917	0.009400351	0.034303914	0.404503717	0.455355468	15	0	0
CKLF-CMTM	0.2105195	0.20111917	0.009400351	0.034303914	0.404503717	0.455355468	15	0	0
IMMP1L	0.0471335	0.046360951	0.000772555	0.01933481	0.404658705	0.455472767	15	0	0
ELP4	0.0471335	0.046360951	0.000772555	0.01933481	0.404658705	0.455472767	15	0	0
TMEM217	0.0897569	0.090849886	-0.00109302	-0.02364607	0.405156994	0.45600501	12	0	0
TIGD6	0.0341048	0.03270001	0.001404836	0.044692649	0.405288748	0.456124677	10	0	0
CDKN1B	0.1999505	0.173045348	0.02690515	0.330359431	0.405433681	0.456259161	6	0	0
DUS3L	0.3688918	0.36638819	0.002503626	0.093256998	0.405494697	0.456299196	5	0	0
RBBP9	0.0463623	0.044831083	0.001531239	0.035658587	0.405631954	0.456425014	6	0	0
RAB21	0.033338	0.032085914	0.00125207	0.038517821	0.405884931	0.456681019	7	0	0
ARL6IP6	0.0616102	0.061603166	7.00E-06	0.010110789	0.406352782	0.457178742	6	0	0
LRRC47	0.0824207	0.086978283	-0.00455763	-0.02390482	0.406441723	0.457250125	8	0	0
SYNGR1	0.0823827	0.081993533	0.000389174	0.068260475	0.40694467	0.457787231	4	0	0
GPR137	0.0549006	0.053256892	0.001643739	0.045319207	0.407161216	0.458002105	6	0	0
BMP8B	0.1356713	0.13502283	0.000648461	0.125310733	0.407540115	0.458399567	8	0	0
FBXL3	0.04259	0.043716023	-0.00112603	-0.02267854	0.407952627	0.458834785	7	0	0
BET1L	0.0394882	0.040624654	-0.00113647	-0.03422566	0.407989133	0.458847072	15	0	0
EMC2	0.0686165	0.066081373	0.002535082	0.068069171	0.408048176	0.4588847	8	0	0
TPR	0.0478592	0.047484698	0.000374488	0.008461337	0.408178135	0.458989518	8	0	0
DUSP4	0.051391	0.042319563	0.009071456	0.303171428	0.408192562	0.458989518	11	0	0
OTUD6B	0.0338396	0.034900733	-0.00106112	-0.03288312	0.408299195	0.459080639	10	0	0
XRCC4	0.0383871	0.03861674	-0.00022964	-0.00793908	0.408371214	0.459132833	13	0	0
SNX18	0.1796721	0.175154062	0.004518052	0.026036698	0.408543704	0.459297974	10	0	0
POMT2	0.0505772	0.050162235	0.000414982	-0.0047375	0.408707175	0.459452954	17	0	0
C8orf59	0.0480477	0.046961282	0.001086427	0.021607937	0.40874097	0.459462148	12	0	0
POLR3B	0.1067193	0.105184523	0.00153477	0.008196872	0.408805622	0.459506025	10	0	0
ARHGEF12	0.0612804	0.060909713	0.000370731	0.008618113	0.409008134	0.459704843	11	0	0
TATDN3	0.0262459	0.027502244	-0.0012563	-0.04031054	0.409130328	0.459784559	5	0	0
ZNF451	0.6932796	0.705778332	-0.01249874	-0.02541452	0.409129429	0.459784559	1	0	0
TMEM102	0.2695234	0.269812727	-0.00028936	0.004356477	0.409256149	0.459897143	9	0	0
GART	0.0397828	0.039124375	0.000658449	0.027139385	0.409316606	0.459936264	6	0	0
ANKS1A	0.0391532	0.039290899	-0.00013765	-0.00161893	0.409465976	0.460046464	17	0	0
ZNFX1	0.0495078	0.047795906	0.001711901	0.033954333	0.409459166	0.460046464	11	0	0
C2CD4C	0.0673334	0.059562684	0.007770759	0.136916454	0.409907619	0.460513815	9	0	0
TECRL	0.6463326	0.66457137	-0.01823877	-0.04017918	0.410112245	0.460714846	2	0	0
BHLHE40	0.0216825	0.021355178	0.000327327	0.014509128	0.410200638	0.460785285	9	0	0
INTS7	0.0582931	0.058280102	1.30E-05	-0.00417051	0.410366937	0.460943222	17	0	0
SCD5	0.1469628	0.138566101	0.008396662	0.079375742	0.410514913	0.461080561	12	0	0
UBQLN1	0.0952034	0.098889612	-0.00368626	-0.02585414	0.410978135	0.461571937	10	0	0
SPTB	0.0747977	0.077968164	-0.0031705	0.001364528	0.411132318	0.461716189	7	0	0
PPOX	0.1021516	0.105072858	-0.00292123	-0.00752368	0.411184486	0.461745864	12	0	0
TM2D2	0.0348507	0.034546072	0.000304651	0.018041504	0.411261374	0.461803294	7	0	0
CAT	0.0621858	0.067940322	-0.00575457	-0.11987544	0.411379341	0.461877925	12	0	0
PRPSAP1	0.0808601	0.079632777	0.001227287	0.042301192	0.411365602	0.461877925	11	0	0
KLF6	0.141486	0.147458757	-0.00597274	0.025184827	0.411429581	0.461905418	7	0	0
TMEM106B	0.2472321	0.245653378	0.001578759	0.029685697	0.411940233	0.462420829	8	0	0
DLST	0.1380934	0.138114187	-2.08E-05	0.013953853	0.411970256	0.46242559	10	0	0
RIC8B	0.0886401	0.085758627	0.002881449	0.04795607	0.412133135	0.462579469	11	0	0
HGD	0.7529071	0.770604922	-0.01769782	-0.03452054	0.412255839	0.462688238	4	0	0
PPM1D	0.0343654	0.032427669	0.001937761	0.061055302	0.412366968	0.462784004	2	0	0
PHOSPHO2	0.1194557	0.117316523	0.002139202	0.006626077	0.412630046	0.463021303	9	0	0
KLHL23	0.1194557	0.117316523	0.002139202	0.006626077	0.412630046	0.463021303	9	0	0
EEA1	0.0468479	0.046027564	0.000820299	0.004912438	0.412692412	0.463062315	8	0	0
ZNF280D	0.0496685	0.046469307	0.00319919	0.092928665	0.412932257	0.46330245	6	0	0
BMF	0.1036018	0.107341019	-0.00373922	-0.02586449	0.412975273	0.46332173	12	0	0
IL17B	0.642258	0.663092845	-0.02083489	-0.04630918	0.413147678	0.463486162	4	0	0
AP3S1	0.1160778	0.118804974	-0.00272722	-0.01980182	0.413499713	0.463852077	12	0	0
FRAS1	0.17049	0.17658988	-0.00609984	-0.03736744	0.413955727	0.464305542	13	0	0
CLEC2D	0.9305654	0.932105193	-0.00153978	-0.00235987	0.413949662	0.464305542	1	0	0
TPP2	0.0398957	0.039226803	0.000668871	0.016336567	0.414181057	0.46452923	8	0	0

LGI3	0.0695017	0.063197892	0.006303857	0.176931756	0.41437364	0.464687108	7	0	0
UBTF	0.0827156	0.074851665	0.007863954	0.087111205	0.414366523	0.464687108	6	0	0
GNA11	0.3428076	0.339443088	0.003364465	0.071541677	0.414441999	0.464734711	3	0	0
UBR2	0.031243	0.030627651	0.000615359	0.01866779	0.414633582	0.464920476	12	0	0
PYURF	0.2318609	0.2305744	0.001286487	0.010833364	0.415039668	0.465346721	11	0	0
GSK3B	0.0473699	0.046726136	0.000643796	0.012331269	0.415123164	0.465411244	8	0	0
EDRF1	0.0772455	0.077214211	3.13E-05	0.020230276	0.415244249	0.465517899	15	0	0
FSTL5	0.1162084	0.106809418	0.009398961	0.145711797	0.415339775	0.465595887	10	0	0
RPL37	0.0903723	0.079154886	0.011217378	0.273609316	0.415891928	0.466156582	10	0	0
CD59	0.1546371	0.144026049	0.010611019	0.060298533	0.415891257	0.466156582	11	0	0
CIPC	0.0460478	0.04655972	-0.00051191	0.009600465	0.416542252	0.466856328	10	0	0
PSCA	0.0620599	0.063976264	-0.00191634	-0.00596728	0.416649463	0.466947311	7	0	0
DNAJC7	0.9053533	0.907710922	-0.00235758	-0.00371103	0.417001829	0.467313016	1	0	0
RANBP6	0.0372239	0.041621299	-0.00439738	-0.11445188	0.417033318	0.467319105	4	0	0
TMEM234	0.0547127	0.052610688	0.002102041	0.054480991	0.417263415	0.467534153	13	0	0
CHCHD1	0.0716886	0.07091368	0.000774901	0.03145479	0.41727736	0.467534153	7	0	0
RUNX1	0.795952	0.817254125	-0.02130217	-0.03765659	0.417771583	0.468058662	3	0	0
FGD6	0.0390557	0.03864413	0.000411565	0.017583521	0.417861492	0.468130152	13	0	0
TNPO3	0.0938368	0.093653755	0.000183037	0.010951725	0.417988418	0.4682431	12	0	0
ZC3H12C	0.1165853	0.121110337	-0.00452501	-0.01022299	0.418084568	0.468321559	8	0	0
ADD3	0.3966029	0.407541492	-0.01093858	-0.03829904	0.418234819	0.468460608	1	0	0
ZNF326	0.1570111	0.158763346	-0.00175227	-0.00914108	0.418504594	0.468707324	7	0	0
TBRG1	0.1382544	0.137413064	0.000841333	0.026834374	0.418507348	0.468707324	8	0	0
SLFN12	0.2130438	0.187289195	0.02575462	0.247319871	0.418840108	0.46905071	10	0	0
ACYP2	0.0803353	0.081861925	-0.00152661	-0.02825338	0.419314594	0.46955276	5	0	0
ZBTB1	0.0630175	0.064793859	-0.00177633	-0.01472753	0.419461734	0.469688205	9	0	0
UQCR10	0.0368157	0.036925903	-0.00011023	0.006240936	0.419706015	0.469932398	10	0	0
KCTD7	0.0435154	0.042828548	0.000686838	0.019056377	0.419806035	0.470015046	9	0	0
RIC8A	0.08346	0.085699414	-0.00223938	-0.02420443	0.419877498	0.470065714	11	0	0
MRC2	0.1262773	0.124291467	0.001985835	0.033207569	0.419962787	0.470131853	11	0	0
MTR	0.1925576	0.201724498	-0.00916686	-0.03901056	0.420818336	0.471060206	8	0	0
PARP9	0.2171817	0.219740108	-0.0025584	0.006105613	0.421252376	0.471516639	13	0	0
UBR4	0.0259735	0.026236928	-0.00026338	-0.00756457	0.421310845	0.471552657	6	0	0
ZNF260	0.0772794	0.077373597	-9.42E-05	0.041282808	0.421385268	0.471606525	10	0	0
GPLD1	0.2490451	0.250124757	-0.00107966	-0.00276334	0.421430439	0.471627651	5	0	0
TSSC4	0.0595184	0.060637292	-0.00111892	-0.01443458	0.421469828	0.471642304	11	0	0
HIPK2	0.0331768	0.032259421	0.000917387	0.02823591	0.421517229	0.471644514	9	0	0
PWP2	0.148498	0.146810499	0.001687486	0.063023137	0.421524395	0.471644514	8	0	0
CCDC88A	0.1011123	0.103760184	-0.00264786	0.031327842	0.421601129	0.471700945	8	0	0
CCDC92	0.0627859	0.061980045	0.000805895	0.023386573	0.421725067	0.471803349	5	0	0
CACNB1	0.3611249	0.363879211	-0.00275436	0.001281986	0.421745266	0.471803349	9	0	0
DLG2	0.0763314	0.0741054	0.002225954	0.018640174	0.421776696	0.471809082	14	0	0
TBKBP1	0.0558513	0.053642866	0.002208419	0.046025016	0.42203214	0.472065385	2	0	0
GUK1	0.0592271	0.060703498	-0.00147644	0.00801593	0.4221446	0.472148166	7	0	0
ADAM9	0.0398994	0.038956071	0.000943341	0.028075591	0.422185119	0.472148166	7	0	0
DGCR6L	0.0295096	0.028645967	0.00086362	0.022233875	0.422172048	0.472148166	7	0	0
CDV3	0.0532731	0.051219674	0.002053468	0.032378141	0.422938707	0.472961447	8	0	0
TWF1	0.0714844	0.073434288	-0.00194988	-0.0145804	0.423111189	0.47312483	12	0	0
ZNF749	0.0789973	0.077119283	0.00187799	0.075701825	0.423141664	0.473129412	11	0	0
PAGR1	0.0632828	0.062167354	0.001115492	0.020095461	0.423365405	0.473320569	8	0	0
ZDHHC8	0.6238984	0.653423268	-0.02952482	-0.06567788	0.423343714	0.473320569	1	0	0
MRPL39	0.0386539	0.037951573	0.000702327	0.017172417	0.423631935	0.47358903	6	0	0
TRIM46	0.0531259	0.052220477	0.000905403	0.031556999	0.423760828	0.473703597	15	0	0
RIPK4	0.0614231	0.05440647	0.007016637	0.159885991	0.423864529	0.473789991	6	0	0
FAR1	0.025893	0.025222378	0.000670648	0.018673754	0.423896128	0.473795784	13	0	0
CTDP1	0.0355215	0.036818875	-0.00129737	-0.02280631	0.424020349	0.473905096	3	0	0
ADCK2	0.1371419	0.140394932	-0.00325301	0.011013302	0.424666705	0.47459792	9	0	0
TPCN1	0.0620888	0.060632397	0.001456441	0.015015408	0.424778253	0.474693006	11	0	0
PGLYRP2	0.5605075	0.579342128	-0.01883464	-0.04685956	0.424923694	0.474825953	1	0	0
ATG4B	0.0451918	0.046420866	-0.00122904	-0.03230219	0.425200801	0.475106003	5	0	0
AGO2	0.0410657	0.039505148	0.001560557	0.038691766	0.425682051	0.475614108	4	0	0
TAP1	0.1064864	0.107812807	-0.00132639	0.015796273	0.425817996	0.475736363	20	0	0
PTS	0.0417378	0.041781097	-4.33E-05	-0.00408358	0.426052775	0.475939373	11	0	0

GPR33	0.7034893	0.711172866	-0.00768357	-0.01545333	0.426047762	0.475939373	1	0	0
TBRG4	0.0505782	0.049169654	0.001408505	0.025268527	0.426133816	0.475977182	11	0	0
SMDT1	0.2378656	0.239998398	-0.00213276	-0.00920779	0.426139696	0.475977182	11	0	0
SHPRH	0.1356257	0.137547231	-0.00192149	-0.02131208	0.426284263	0.476079361	9	0	0
WIPI1	0.0883124	0.086106571	0.002205871	0.041666727	0.426276603	0.476079361	9	0	0
SEC31A	0.0265437	0.026905181	-0.0003615	0.000636489	0.426611198	0.476414821	3	0	0
GLUD1	0.0430684	0.041279758	0.001788667	0.057587293	0.426757317	0.476548328	4	0	0
AGL	0.0887918	0.092890083	-0.00409832	-0.00695384	0.427091782	0.476892125	15	0	0
HMGB1	0.0500262	0.050291539	-0.00026531	-0.00749518	0.427445126	0.477256958	6	0	0
CITED2	0.0380995	0.037077275	0.001022179	0.022821794	0.427641579	0.477446584	21	0	0
STT3B	0.0475694	0.046046143	0.001523282	0.032957233	0.427873706	0.47767601	5	0	0
DTNB	0.130553	0.132022803	-0.00146981	0.016347047	0.428092063	0.477890038	8	0	0
ATP6V1H	0.1824227	0.184727811	-0.0023051	-0.00323322	0.428263977	0.478052197	16	0	0
ARHGEF11	0.061002	0.058249222	0.002752771	0.048726558	0.428656764	0.478460869	11	0	0
COPZ1	0.1233234	0.120954912	0.002368538	0.080919726	0.42908462	0.478908634	9	0	0
IFT140	0.0500913	0.049331184	0.000760088	0.012273924	0.429617425	0.47947347	13	0	0
WDR75	0.0410657	0.040025777	0.001039879	0.026188137	0.430275735	0.480148421	13	0	0
FBXL19	0.0488025	0.047513177	0.001289335	0.035443718	0.430261983	0.480148421	14	0	0
RBFOX2	0.0380211	0.037377291	0.00064381	0.028838741	0.430473939	0.480339715	7	0	0
CENPF	0.0300244	0.03083546	-0.00081106	-0.02424228	0.430515642	0.480356364	9	0	0
TIA1	0.044506	0.045807449	-0.00130148	-0.02888853	0.430676316	0.480505748	2	0	0
CELA1	0.7008401	0.718875918	-0.0180358	-0.03659952	0.430743826	0.480521288	3	0	0
ZNF235	0.0404029	0.039479612	0.000923246	0.021083609	0.430717862	0.480521288	9	0	0
CHSY1	0.0368314	0.035409192	0.001422181	0.04126807	0.43092673	0.48069543	6	0	0
PPP2R1B	0.1491917	0.153243269	-0.00405156	0.013745789	0.430989849	0.480735941	9	0	0
MARCKS	0.064846	0.056879801	0.007966201	0.125218953	0.431302469	0.481047523	10	0	0
CCDC97	0.1660519	0.170551711	-0.00449986	-0.00811994	0.431322829	0.481047523	11	0	0
HIST1H2AK	0.0571557	0.058263629	-0.0011079	0.012456281	0.43135766	0.481056457	9	0	0
FBXO5	0.0487535	0.048955464	-0.00020195	-0.01073872	0.431501652	0.481187119	10	0	0
TOR1A	0.7769478	0.80034048	-0.02339271	-0.04360488	0.431575565	0.481239623	2	0	0
CCNK	0.0342026	0.032943735	0.001258824	0.020277583	0.431929033	0.481603826	14	0	0
RNF215	0.6133087	0.623266287	-0.00995754	-0.01653772	0.432133212	0.481801535	2	0	0
TESK1	0.0438823	0.042641884	0.001240399	0.032710226	0.432266767	0.481920484	7	0	0
ST3GAL5	0.0292112	0.028469386	0.000741836	0.024832061	0.432696509	0.482369606	7	0	0
CBLB	0.0532719	0.053950686	-0.00067878	-0.02107506	0.432785961	0.482439341	9	0	0
FOS	0.0340988	0.034422397	-0.00032363	-0.0106844	0.432836155	0.482465309	8	0	0
GNAO1	0.0604576	0.052239832	0.008217785	0.272359244	0.433227677	0.482871713	11	0	0
DHTKD1	0.0590003	0.057070543	0.001929728	0.032964763	0.433288986	0.482910039	8	0	0
ISY1-RAB43	0.0349833	0.034978011	5.28E-06	0.00230528	0.433378527	0.482949814	10	0	0
ISY1	0.0349833	0.034978011	5.28E-06	0.00230528	0.433378527	0.482949814	10	0	0
SART3	0.0537033	0.052419994	0.001283277	0.027339409	0.433528679	0.483087127	12	0	0
CENPQ	0.0385395	0.039218232	-0.00067876	-0.0134307	0.433954005	0.48350626	14	0	0
TBPL2	0.7922901	0.809169551	-0.01687941	-0.03003803	0.43395873	0.48350626	1	0	0
FOXN4	0.0642025	0.060210427	0.003992115	0.104836271	0.434005182	0.48352798	10	0	0
MRPL32	0.0535762	0.053803316	-0.00022716	-0.00633095	0.434292663	0.483817214	8	0	0
BCL9L	0.0397101	0.038623862	0.001086279	0.028363894	0.434318742	0.483817214	5	0	0
KIF3C	0.1771431	0.178233581	-0.00109048	0.175051212	0.434392632	0.483869473	8	0	0
XAB2	0.0539578	0.055635915	-0.0016781	-0.01765473	0.434635176	0.484109577	4	0	0
CAPN5	0.1176569	0.119605969	-0.00194909	0.114282677	0.434782487	0.484243585	6	0	0
ITPR1	0.0535245	0.053793809	-0.00026935	-0.00808953	0.434940483	0.484389476	11	0	0
ALDH4A1	0.060408	0.059954836	0.000453188	0.007377239	0.434968414	0.484390506	13	0	0
VEZT	0.035274	0.034880753	0.000393242	0.016365314	0.435176547	0.484592199	11	0	0
INHBB	0.0660894	0.04976779	0.016321636	0.361683534	0.435448749	0.484756864	6	0	0
PTPRG	0.0820218	0.080680259	0.001341558	0.028492166	0.435422579	0.484756864	15	0	0
EIF2B5	0.0387673	0.037134843	0.001632453	0.025108606	0.435459556	0.484756864	12	0	0
CLDN23	0.0425132	0.039505802	0.003007427	0.1149068	0.435432042	0.484756864	6	0	0
ATXN7L1	0.18714	0.188690638	-0.00155061	0.025055859	0.435544288	0.484821098	6	0	0
THTPA	0.0475528	0.045792005	0.001760799	0.039225188	0.435777318	0.485050389	12	0	0
FADD	0.2643272	0.277586147	-0.01325893	-0.03548685	0.43586965	0.485123056	12	0	0
CCT5	0.042906	0.043672112	-0.00076611	-0.02532986	0.435926762	0.485156515	10	0	0
FOXO3	0.0377783	0.037078343	0.000699916	0.016120589	0.435978181	0.485183636	7	0	0
SNRPD3	0.0599114	0.057983027	0.001928325	0.024428718	0.436072025	0.485257962	19	0	0
THUMPD1	0.3073733	0.311211411	-0.00383808	-0.00574492	0.43619719	0.485367132	5	0	0

SKP2	0.041971	0.041992606	-2.16E-05	-0.00738886	0.436583815	0.485762632	15	0	0
TMED3	0.0626297	0.054724599	0.00790512	0.219106365	0.436606791	0.485762632	6	0	0
PCBP1	0.0717322	0.069183544	0.00254864	0.054683247	0.4367388	0.485879365	12	0	0
KRR1	0.1765155	0.175331275	0.001184266	0.020277855	0.436987658	0.486126071	16	0	0
PSD	0.0811327	0.075107643	0.006025026	0.065080581	0.437054446	0.486170215	7	0	0
UGT1A4	0.8041809	0.817620552	-0.01343968	-0.02397321	0.437088831	0.486178312	2	0	0
SPATA5L1	0.0499889	0.05133526	-0.00134638	-0.02066204	0.437205051	0.486277428	7	0	0
TMCO1	0.046989	0.047583415	-0.00059442	-0.02038282	0.437719901	0.486819876	10	0	0
MRPL18	0.0820758	0.083495469	-0.00141968	-0.00095165	0.437767872	0.48684304	9	0	0
SEMA4D	0.0204541	0.015073947	0.005380125	0.280446019	0.437897308	0.486956793	1	0	0
UNC79	0.0668848	0.068312719	-0.00142794	0.003184419	0.43793796	0.486971807	11	0	0
AAMP	0.0441502	0.044961944	-0.00081174	-0.01186229	0.438065132	0.487083021	8	0	0
TTLL3	0.8426261	0.848886077	-0.00626002	-0.01055364	0.438241633	0.487249066	1	0	0
PHF21A	0.040843	0.039452316	0.001390636	0.033435295	0.438512986	0.487520543	9	0	0
GSTA4	0.0468999	0.048159711	-0.0012598	-0.01992182	0.438963906	0.487991609	8	0	0
SURF6	0.3616893	0.355624575	0.006064699	0.079230595	0.439083586	0.488064155	3	0	0
ANKRD54	0.0807628	0.0784753	0.002287509	0.007446904	0.439079973	0.488064155	9	0	0
RTF1	0.7513091	0.768842099	-0.01753298	-0.03558172	0.439231977	0.488198844	2	0	0
EPHB6	0.0326272	0.03094255	0.00168465	0.060739086	0.439442933	0.488403052	5	0	0
DCTN6	0.1331473	0.129136149	0.004011131	0.035328435	0.439726775	0.488688236	10	0	0
RBM48	0.038688	0.039553696	-0.00086573	-0.02317585	0.439817682	0.4887287	13	0	0
SNX33	0.0709836	0.065609898	0.005373701	0.098896259	0.439805867	0.4887287	6	0	0
ZNF669	0.1666413	0.168450281	-0.00180903	0.024281266	0.439966571	0.488863859	7	0	0
TRIM28	0.0227696	0.023240862	-0.00047126	-0.01842852	0.440722324	0.48967327	5	0	0
EAF2	0.040607	0.04013961	0.000467377	0.018450771	0.440777101	0.489703796	9	0	0
COPG1	0.1167769	0.117039226	-0.00026237	0.01065429	0.44118614	0.490127879	6	0	0
UBE2M	0.0456853	0.045722885	-3.76E-05	0.007197907	0.44129046	0.490213408	6	0	0
RHOF	0.0304198	0.030302114	0.000117686	0.002574911	0.441938421	0.490859477	16	0	0
PCK2	0.1394689	0.141031862	-0.00156295	-0.0199062	0.441954154	0.490859477	9	0	0
RRP8	0.0423295	0.041523013	0.000806483	0.019109016	0.442152644	0.491049525	12	0	0
SMG7	0.0362465	0.036447073	-0.00020058	-0.00526686	0.442902639	0.491852005	12	0	0
PIK3R4	0.0402037	0.039781372	0.000422325	0.00911867	0.442975438	0.491902393	8	0	0
MDH1	0.0532687	0.053352699	-8.40E-05	-0.00781531	0.443053918	0.491959084	23	0	0
ETV3	0.1117556	0.113904852	-0.00214923	-0.01318644	0.443163563	0.49205037	10	0	0
STXBP5	0.0427324	0.038055935	0.004676423	0.125210575	0.443406409	0.492289529	8	0	0
PCDHB1	0.2610699	0.249347191	0.011722672	0.075344972	0.443806778	0.492703538	10	0	0
AASDHPPT	0.8202816	0.835448879	-0.01516732	-0.02611687	0.444670101	0.493631425	1	0	0
SLC35A5	0.0400262	0.039747989	0.000278236	0.002192292	0.444767198	0.493708656	10	0	0
PGM2	0.055251	0.053821675	0.001429281	0.008558185	0.444906836	0.493833097	7	0	0
MBTD1	0.0335813	0.033843956	-0.00026261	-0.00950952	0.445083249	0.493998339	10	0	0
TRMT112	0.0268699	0.026704531	0.00016538	0.007104372	0.445307087	0.494185616	12	0	0
SIX5	0.0322762	0.033186831	-0.00091059	-0.02562986	0.44529239	0.494185616	6	0	0
C5orf47	0.1172691	0.109587673	0.0076814	0.130455137	0.44537259	0.494227728	6	0	0
HECA	0.1840402	0.178192112	0.005848046	0.020044674	0.445408308	0.494236786	9	0	0
HS1BP3	0.1846337	0.186376076	-0.0017424	-0.00749062	0.445451201	0.494253803	6	0	0
BROX	0.0456445	0.045688173	-4.36E-05	0.012797237	0.445482887	0.494258386	13	0	0
SEMA6C	0.1305304	0.131546253	-0.00101589	0.013293831	0.446198368	0.495021583	9	0	0
UQCRB	0.2521234	0.255529686	-0.00340625	-0.00824915	0.446283263	0.495085144	12	0	0
MYBPC2	0.0989978	0.094487039	0.004510718	0.077149816	0.446517192	0.495314018	9	0	0
SERPINA11	0.6195631	0.640625041	-0.02106194	-0.04747537	0.446722302	0.495510898	1	0	0
TRIM26	0.0612224	0.060432557	0.000789872	0.037053434	0.446801967	0.495568615	33	0	0
TAMM41	0.1369439	0.139239109	-0.00229524	0.004781411	0.447113972	0.495884009	9	0	0
FAM173B	0.0470979	0.047736411	-0.00063851	-0.02097597	0.447487553	0.496267653	16	0	0
OLFML2B	0.0344473	0.027202671	0.007244621	0.265516536	0.447753485	0.496470481	10	0	0
CAMSAP2	0.0422712	0.042163808	0.000107437	0.009224884	0.447748243	0.496470481	6	0	0
CAMKMT	0.0777522	0.080263534	-0.00251137	0.005329521	0.447723962	0.496470481	14	0	0
DTL	0.0999181	0.101422875	-0.00150479	0.005170672	0.447962955	0.496672039	16	0	0
SIRT5	0.062921	0.059087171	0.003833844	0.042178659	0.448126582	0.496822745	9	0	0
EZH2	0.0385266	0.038870824	-0.00034422	-0.00621627	0.448367431	0.497059042	8	0	0
MAP3K8	0.0350775	0.034601398	0.000476104	0.017867336	0.448526355	0.497204494	12	0	0
GSTZ1	0.1056017	0.104916213	0.000685502	0.002686774	0.449190276	0.497909696	14	0	0
KMT2E	0.0426618	0.042404868	0.000256898	-0.00489307	0.449287366	0.497936523	17	0	0
NEDD4	0.0801791	0.077852319	0.002326762	0.044998993	0.449297764	0.497936523	6	0	0

NDUFAF5	0.1533953	0.151991077	0.001404179	0.01017947	0.44926358	0.497936523	14	0	0
CCDC138	0.117901	0.121423399	-0.00352245	-0.02198499	0.449802206	0.498433978	11	0	0
NRIP1	0.0791555	0.076247024	0.00290845	0.046629658	0.449790821	0.498433978	9	0	0
ASIC1	0.0616877	0.058450858	0.003236861	0.042999017	0.450145172	0.498783209	8	0	0
SETD6	0.0478295	0.043388143	0.004441386	0.176876986	0.450560544	0.499212622	5	0	0
AKT3	0.0244207	0.024753315	-0.00033265	-0.01029333	0.450794402	0.49944088	2	0	0
AP4M1	0.0363717	0.036541142	-0.00016945	-0.00461574	0.451134363	0.499786655	22	0	0
THOC1	0.1168323	0.114352695	0.002479622	0.021817229	0.451301382	0.499940806	10	0	0
FAM161A	0.0466457	0.045591288	0.001054377	0.021446866	0.451694332	0.500306001	11	0	0
GTPBP2	0.0474071	0.048234679	-0.00082762	-0.01676942	0.451742623	0.500306001	13	0	0
MEGF9	0.1891396	0.191054359	-0.00191471	-0.00799298	0.45172183	0.500306001	8	0	0
ZBED4	0.0291416	0.027130116	0.002011506	0.058330526	0.45168173	0.500306001	4	0	0
LAMTOR3	0.2641977	0.265833757	-0.00163607	-0.0195975	0.451823396	0.500364561	8	0	0
FAM81A	0.0452852	0.0462686	-0.00098339	-0.00902826	0.451898689	0.500417046	11	0	0
ORC5	0.0449818	0.045208349	-0.00022655	-0.01305078	0.45220296	0.500723071	6	0	0
DEPDC4	0.0355061	0.03463947	0.00086666	0.023699878	0.45230034	0.500799982	15	0	0
NLK	0.0361262	0.034863404	0.001262783	0.034702438	0.452584681	0.501083878	8	0	0
SCUBE2	0.5375406	0.553841787	-0.01630122	-0.04155711	0.452661954	0.501138498	3	0	0
CPS1	0.1366376	0.134759004	0.001878547	0.030961476	0.452749247	0.50117327	9	0	0
WDR37	0.0245663	0.024443516	0.000122832	-0.00550409	0.452741612	0.50117327	9	0	0
JUN	0.0400219	0.039580947	0.000440952	0.010104429	0.452892195	0.501300568	13	0	0
NME7	0.0420589	0.042596653	-0.00053771	-0.01606206	0.453119181	0.501520866	12	0	0
SHC1	0.0584847	0.057337959	0.001146787	0.019998105	0.453642778	0.502007457	11	0	0
PRKAR2B	0.2691736	0.265378551	0.003795042	-0.00137503	0.453605189	0.502007457	6	0	0
SCYL2	0.0295372	0.029610683	-7.35E-05	0.006838294	0.45362914	0.502007457	9	0	0
TBXAS1	0.0391668	0.037238993	0.001927831	0.068548266	0.454048788	0.502425755	3	0	0
LMBRD1	0.2085093	0.205236306	0.003272954	0.027731391	0.454084475	0.502434247	6	0	0
SNX14	0.0396468	0.039027543	0.000619297	0.01112993	0.454238933	0.502574147	9	0	0
CCT7	0.063374	0.062862607	0.000511361	0.036188346	0.45434337	0.50265869	8	0	0
TSPAN5	0.2223075	0.224439442	-0.00213194	0.171168932	0.455091075	0.503454852	5	0	0
ANKRD12	0.0484874	0.048182736	0.000304642	0.001025207	0.455471204	0.503844302	9	0	0
KIAA1671	0.0707343	0.072741458	-0.0020072	-0.03542935	0.456197898	0.504617052	1	0	0
XRN1	0.3487007	0.346972775	0.001727953	-0.00055896	0.456330716	0.50473284	8	0	0
DSN1	0.130854	0.134240228	-0.00338618	0.00377241	0.45641512	0.504795066	11	0	0
MRPL50	0.0436012	0.04403119	-0.00043003	-0.00641351	0.456965468	0.505372589	12	0	0
JRKL	0.0568743	0.056908706	-3.44E-05	0.007046382	0.45722464	0.505628038	14	0	0
CEP104	0.2536248	0.251749763	0.001874988	0.019071401	0.45733263	0.505716279	15	0	0
SOS1	0.0381894	0.037886597	0.000302815	0.007613253	0.457951176	0.506369046	6	0	0
RITA1	0.0362102	0.037377273	-0.00116703	-0.03344534	0.457988946	0.506379592	6	0	0
HIST1H3D	0.0592347	0.056077014	0.003157718	0.078135797	0.45820105	0.506520434	7	0	0
HIST1H2AD	0.0592347	0.056077014	0.003157718	0.078135797	0.45820105	0.506520434	7	0	0
HIST1H2BF	0.0592347	0.056077014	0.003157718	0.078135797	0.45820105	0.506520434	7	0	0
NR4A3	0.0379502	0.038600883	-0.00065066	-0.01311718	0.458816218	0.507169215	8	0	0
TLE3	0.0597634	0.05922062	0.00054279	0.057281631	0.459617431	0.507992251	10	0	0
URM1	0.0269654	0.027395346	-0.00042998	-0.00911361	0.459845118	0.508201266	7	0	0
IQCD	0.0700571	0.068470177	0.001586926	0.015950443	0.45986321	0.508201266	12	0	0
HDGF	0.0680252	0.067783103	0.000242077	-0.00187872	0.459980395	0.508268135	14	0	0
TST	0.0643787	0.062433075	0.00194561	0.021270665	0.459958114	0.508268135	6	0	0
ADAT2	0.0811331	0.084679868	-0.00354673	-0.01373572	0.460255778	0.508488429	14	0	0
FBXO15	0.0402403	0.040224407	1.59E-05	-0.00293421	0.460264811	0.508488429	10	0	0
TIMM21	0.0402403	0.040224407	1.59E-05	-0.00293421	0.460264811	0.508488429	10	0	0
FGFR1OP2	0.0493444	0.049599262	-0.00025486	-0.00567744	0.460335206	0.508534877	10	0	0
MRPL21	0.0422288	0.042778039	-0.00054922	-0.01627487	0.460469519	0.508651925	9	0	0
TLE4	0.0411964	0.038334253	0.002862163	0.081024888	0.46131033	0.509549334	8	0	0
MAP3K14	0.0370412	0.036472764	0.000568407	0.014867333	0.46141802	0.509636899	3	0	0
CRISP3	0.7885111	0.800608633	-0.01209753	-0.02169307	0.461677426	0.509892014	1	0	0
ZNF133	0.1121987	0.114848939	-0.00265023	-0.00330402	0.46178746	0.509982137	13	0	0
STARD3NL	0.0464588	0.044364798	0.002093952	0.080946887	0.462211583	0.510419096	9	0	0
WSCD1	0.1896813	0.17521499	0.014466265	0.10849804	0.462244177	0.510423663	1	0	0
CHIC2	0.0374586	0.03701753	0.000441105	0.011973696	0.462378036	0.510540042	7	0	0
KDSR	0.0650929	0.064632371	0.000460549	0.019784143	0.462423924	0.51055928	10	0	0
PRKAA1	0.0361022	0.037050596	-0.0009484	-0.02006699	0.46249057	0.510601432	10	0	0
CD320	0.0326311	0.031939482	0.000691575	0.036317142	0.462595427	0.510685763	6	0	0

OTUD7A	0.056248	0.054428621	0.001819353	0.038101425	0.463365527	0.511504437	7	0	0
ZDHHC6	0.0316754	0.031436067	0.000239314	0.006921077	0.463414781	0.511527326	12	0	0
ZBTB46	0.2840506	0.27224426	0.011806364	0.052650083	0.463678551	0.511786984	3	0	0
CDC45	0.0419869	0.040997929	0.00098898	0.015489133	0.463799123	0.511888565	15	0	0
LLGL2	0.306608	0.313864923	-0.00725697	-0.02484732	0.464125031	0.512216746	7	0	0
HIRA	0.0733794	0.069220015	0.004159338	0.033564635	0.46455994	0.512665174	10	0	0
FAM114A2	0.0597912	0.059258122	0.000533084	-0.01115599	0.46465398	0.512737405	14	0	0
UBR5	0.507934	0.511020099	-0.00308608	0.005633846	0.464847826	0.512919755	5	0	0
KLRG1	0.0424754	0.04320517	-0.00072977	-0.01479651	0.465211772	0.51328976	9	0	0
TRMT6	0.0420712	0.042163205	-9.20E-05	0.011939849	0.465546454	0.513627435	14	0	0
CORO2B	0.084255	0.080465242	0.003789745	0.123626527	0.465611412	0.513667504	10	0	0
KLHL17	0.0886971	0.091161723	-0.00246465	-0.00641693	0.466018393	0.514084869	5	0	0
IARS	0.0458992	0.045766384	0.000132806	0.001443445	0.466309354	0.514374205	7	0	0
NPHS1	0.07432	0.07164642	0.002673614	0.050033971	0.466398788	0.514441219	6	0	0
ILK	0.041139	0.040216275	0.000922725	0.022417999	0.466705822	0.514748224	10	0	0
MRPL47	0.1618114	0.159879185	0.001932231	0.022360561	0.466737805	0.514751846	7	0	0
DPY19L4	0.0543524	0.053354024	0.000998362	0.007532944	0.466948103	0.514952113	6	0	0
RASA3	0.0315372	0.031145256	0.000391951	0.012337306	0.467485559	0.515513125	8	0	0
CDCA4	0.03217	0.032210575	-4.05E-05	0.003604278	0.467576591	0.515581811	5	0	0
NFYA	0.033613	0.034185984	-0.00057294	-0.01022315	0.467956352	0.51596884	15	0	0
BCCIP	0.0986625	0.098659561	2.96E-06	0.013688416	0.46799264	0.515977133	15	0	0
FZD8	0.0640284	0.061410994	0.002617439	0.056207453	0.468403305	0.516398161	10	0	0
SGMS1	0.0344818	0.035483912	-0.00100208	-0.02233754	0.469301584	0.517356681	4	0	0
BICD1	0.1408275	0.137961779	0.002865718	0.032197763	0.469691201	0.517754372	8	0	0
C2orf42	0.0512289	0.052726188	-0.00149728	-0.03127184	0.469862982	0.517911901	3	0	0
ITGAV	0.0764504	0.073232304	0.003218116	0.026893952	0.470526226	0.518611098	8	0	0
DHX38	0.0733651	0.071762616	0.001602481	0.002633481	0.470568454	0.51862577	18	0	0
NUDT14	0.3238721	0.338192934	-0.01432088	-0.02834116	0.470975584	0.519042585	5	0	0
ANKRD10	0.052406	0.053565028	-0.00115898	0.000421968	0.471053962	0.519097067	13	0	0
MOCS3	0.0665557	0.068253893	-0.00169815	0.010790181	0.471197767	0.519223638	12	0	0
POLI	0.0257004	0.025487305	0.000213137	-0.0037014	0.471375882	0.519387997	5	0	0
PHTF2	0.0585518	0.057895146	0.000656677	0.010190653	0.471809941	0.519834332	18	0	0
C20orf194	0.0259041	0.025085454	0.000818652	0.026655016	0.471945494	0.519951744	12	0	0
EDN1	0.0490006	0.048256049	0.000744516	0.015812019	0.472013741	0.519994991	10	0	0
GAS1	0.0657584	0.064182644	0.00157572	0.026531124	0.472128304	0.520089255	6	0	0
MAP4K3	0.0567642	0.058832131	-0.00206789	-0.00289641	0.472237148	0.520177209	5	0	0
IDH1	0.0537342	0.053050678	0.000683549	0.037466326	0.472594649	0.520539034	10	0	0
UNC50	0.0991025	0.099679345	-0.0005768	0.010431223	0.473045988	0.521004166	11	0	0
TNFAIP1	0.05258	0.05051889	0.002061154	0.027784726	0.473151785	0.521088691	15	0	0
TMEM60	0.0451588	0.044843768	0.000315047	0.010938555	0.473496882	0.521436733	25	0	0
POTED	0.9042475	0.896605764	0.007641772	0.0121095	0.474369947	0.52236612	1	0	0
RAET1L	0.0999113	0.085758664	0.014152651	0.253552033	0.474852005	0.522864851	9	0	0
ZDHHC14	0.0779441	0.078512461	-0.00056833	0.003150332	0.474895048	0.522880146	10	0	0
ALG10	0.038386	0.038857322	-0.00047135	-0.00037051	0.475122424	0.523098385	3	0	0
KRIT1	0.067117	0.0664752	0.000641812	0.019011916	0.475253256	0.523210311	16	0	0
FSD2	0.753705	0.762293952	-0.00858895	-0.01605383	0.47532029	0.523251994	4	0	0
MEA1	0.0540785	0.052714323	0.001364186	0.016530931	0.47535061	0.523253255	13	0	0
ATP6V0B	0.1380563	0.138688267	-0.00063197	-0.00611632	0.4756765	0.523579853	8	0	0
REXO2	0.1085054	0.111235608	-0.00273019	-0.01642365	0.475868614	0.523727033	8	0	0
PARP11	0.1187031	0.125189887	-0.00648683	-0.00371185	0.475849754	0.523727033	9	0	0
TOR1AIP1	0.1128761	0.11449237	-0.00161628	0.023010964	0.476399017	0.524278608	10	0	0
LGALS14	0.3888889	0.395088124	-0.00619925	-0.00548328	0.477084071	0.5250003	3	0	0
SPATC1L	0.1734158	0.177262122	-0.0038463	-0.0062501	0.477638465	0.525578129	7	0	0
PATL1	0.0789998	0.075281575	0.003718255	0.029958408	0.477745921	0.525664122	7	0	0
NIT1	0.057611	0.057560408	5.06E-05	0.004451967	0.477821295	0.525714805	11	0	0
SASS6	0.0392061	0.040069492	-0.00086342	-0.01211926	0.477985427	0.525863131	9	0	0
HSPE1	0.0479428	0.049045547	-0.00110275	-0.01463485	0.478027497	0.525877158	18	0	0
ZNF562	0.054813	0.044681075	0.010131895	0.276164365	0.478199351	0.526033951	8	0	0
PLA2G12A	0.0609466	0.06199761	-0.00105103	-0.02022875	0.478439169	0.526233209	7	0	0
BBIP1	0.0348153	0.035088376	-0.0002731	-0.00277483	0.478418173	0.526233209	11	0	0
07-Mar	0.1111122	0.116724297	-0.00561206	-0.02410087	0.478730239	0.526521067	11	0	0
SLC12A2	0.0382293	0.03694805	0.001281221	0.031368551	0.478861102	0.526632701	9	0	0
PSMA2	0.0584697	0.057917103	0.000552638	0.004821015	0.479246559	0.527024297	9	0	0

CDHR3	0.1804616	0.182652385	-0.00219076	0.023714998	0.4797324	0.52752623	6	0	0
TUBA3E	0.9222272	0.924134703	-0.00190754	-0.00310595	0.47988983	0.527666993	3	0	0
TXNDC16	0.05159	0.051422116	0.000167908	0.000317212	0.480672216	0.528462478	9	0	0
GPR137C	0.05159	0.051422116	0.000167908	0.000317212	0.480672216	0.528462478	9	0	0
MRPS23	0.1023506	0.100100093	0.002250538	0.018833181	0.480871917	0.52864963	10	0	0
THAP11	0.177869	0.180873324	-0.00300434	0.007991047	0.481402645	0.529200652	11	0	0
ETV6	0.0270648	0.026195561	0.000869262	0.023060511	0.481478282	0.529251362	9	0	0
ARHGEF17	0.0362571	0.033116517	0.003140612	0.130565962	0.481709643	0.52947323	7	0	0
BCAR3	0.0335267	0.033696959	-0.00017024	-0.01049912	0.482145137	0.52991943	13	0	0
DUSP10	0.0438556	0.043412983	0.000442621	0.008626828	0.482948508	0.53076988	11	0	0
MITD1	0.0348731	0.034364187	0.000508911	0.012304692	0.483378095	0.531209455	14	0	0
KDELR3	0.2185046	0.215017516	0.003487057	0.169554661	0.483431945	0.531236084	7	0	0
LRRC28	0.1420909	0.146157323	-0.00406647	-0.01317072	0.48422841	0.532046114	8	0	0
RETSAT	0.1438938	0.143683485	0.00021028	0.013240763	0.484265663	0.532054453	12	0	0
MALSU1	0.4558513	0.463402696	-0.00755142	-0.00011663	0.484306812	0.53206707	8	0	0
RPLP2	0.0265276	0.026969784	-0.00044215	-0.00753932	0.48440275	0.532139874	8	0	0
PPIP5K2	0.0492905	0.048520431	0.000770033	0.020492561	0.48452836	0.532245263	12	0	0
RORB	0.1181608	0.112742707	0.005418078	0.020816034	0.48457599	0.532264986	6	0	0
MYCN	0.0590496	0.056168847	0.002880774	0.039827494	0.484988128	0.532652444	9	0	0
CCDC28A	0.0666254	0.064806076	0.001819306	0.016053814	0.484985936	0.532652444	11	0	0
PCNP	0.0362632	0.035278532	0.000984676	0.028112778	0.485089146	0.532730769	9	0	0
ZCCHC2	0.0400689	0.03939804	0.000670902	0.023254708	0.485190222	0.532793287	11	0	0
CTU1	0.1262349	0.129496666	-0.00326181	-0.02241141	0.485205483	0.532793287	10	0	0
ITGB4	0.3056563	0.31314547	-0.00748914	-0.03156386	0.485270837	0.532832429	4	0	0
PREPL	0.0354097	0.034928234	0.000481481	0.011373215	0.485405596	0.53294777	13	0	0
SUGP2	0.039343	0.039707399	-0.00036443	-0.00322626	0.485493972	0.533012173	6	0	0
EARS2	0.0492313	0.048835755	0.000395526	0.007367062	0.485525674	0.533014352	15	0	0
ATG12	0.0840385	0.087618032	-0.00357956	-0.02754106	0.485986566	0.533487671	12	0	0
DDX31	0.1157666	0.119927112	-0.0041605	-0.0126371	0.48609699	0.533576231	11	0	0
TRIM8	0.0476008	0.047776596	-0.00017577	-0.0042536	0.486717401	0.534224546	9	0	0
TMED5	0.0491513	0.047706825	0.001444515	0.025835264	0.4868131	0.534296889	14	0	0
GID8	0.0579468	0.056465629	0.001481143	0.02909728	0.48706033	0.534535524	5	0	0
RAB11FIP3	0.1720372	0.171444048	0.000593186	-0.00192931	0.487343647	0.534813733	10	0	0
MTMR4	0.0588008	0.060036887	-0.00123607	0.003248101	0.487478491	0.534928982	12	0	0
PLEKHA8	0.0443735	0.043760103	0.000613381	0.013919054	0.488120282	0.535600476	12	0	0
LRRIQ1	0.0622855	0.060799844	0.001485671	0.032244961	0.488274899	0.535737358	11	0	0
OSBPL11	0.0492424	0.048822527	0.000419918	0.008181737	0.488610511	0.536072799	7	0	0
GNA13	0.1369951	0.131997592	0.004997498	0.053476806	0.488778198	0.536223975	7	0	0
MAP3K11	0.0393988	0.039128229	0.000270618	0.007604668	0.489301383	0.536765114	6	0	0
PAPPA	0.3458481	0.340887451	0.004960617	0.011526752	0.489544725	0.536999217	4	0	0
MTMR12	0.0342536	0.033534536	0.000719108	0.022968214	0.489761774	0.5371716	10	0	0
PPFIA1	0.038389	0.036897871	0.001491098	0.060287588	0.489757705	0.5371716	5	0	0
ZNF618	0.0462922	0.04531903	0.000973132	0.01944801	0.489893202	0.537282896	3	0	0
ZBTB41	0.0441189	0.043053999	0.001064918	0.032798059	0.490063926	0.537404413	9	0	0
CRB1	0.0441189	0.043053999	0.001064918	0.032798059	0.490063926	0.537404413	9	0	0
FBRS	0.2759128	0.27832575	-0.00241292	-0.01293468	0.490103671	0.53741514	4	0	0
KIAA1324L	0.1474786	0.150286882	-0.00280831	0.09601664	0.490284075	0.537547232	7	0	0
USP3	0.0615381	0.060037761	0.001500295	0.040617852	0.490254296	0.537547232	9	0	0
UTP18	0.0346835	0.034423238	0.00026031	0.005822428	0.490790557	0.538069647	9	0	0
NDUFA9	0.0944289	0.095209676	-0.00078078	-0.0110577	0.490827308	0.538077048	15	0	0
LPCAT4	0.0372801	0.037735387	-0.00045526	-0.01549939	0.490870968	0.538092023	8	0	0
IFNA5	0.8291137	0.825706247	0.003407467	0.005870413	0.492285468	0.539609616	1	0	0
EPC1	0.0864578	0.081842851	0.004614972	0.048143395	0.492366431	0.539665381	6	0	0
CSNK1G2	0.0512025	0.051846733	-0.00064418	-0.01208856	0.492642812	0.539935318	7	0	0
PUSL1	0.035252	0.033002951	0.002249017	0.04003058	0.492780409	0.540034213	9	0	0
SPRR1B	0.8399034	0.856176246	-0.01627283	-0.02736171	0.492793263	0.540034213	1	0	0
AKAP1	0.0371923	0.03744818	-0.00025583	-0.01287931	0.493014972	0.540244168	8	0	0
SLC37A1	0.1769451	0.177564186	-0.00061911	0.008898824	0.493157104	0.540366902	13	0	0
SLC30A7	0.0341006	0.033565532	0.000535075	0.015744128	0.493421331	0.540592016	16	0	0
PLAC8	0.8133068	0.808880578	0.004426186	0.007777009	0.493422831	0.540592016	1	0	0
RHBDD1	0.1465401	0.134452682	0.012087376	0.129292482	0.493667712	0.540827271	9	0	0
PATZ1	0.0352225	0.035988246	-0.0007657	-0.02452523	0.494103792	0.541271948	8	0	0
EPB41L4A	0.064186	0.062637659	0.001548349	0.023860765	0.494145256	0.541284311	5	0	0

SMAD7	0.0532067	0.052803369	0.000403368	0.004450527	0.494476101	0.541613639	8	0	0
LRRC59	0.3805462	0.375231121	0.005315098	0.017070144	0.494641293	0.541761494	6	0	0
BAD	0.0512018	0.050147284	0.001054475	0.02497303	0.494912276	0.542025193	21	0	0
FGGY	0.0458567	0.045061566	0.00079513	0.015347722	0.495584087	0.542727817	11	0	0
POLR3C	0.0300214	0.030115001	-9.36E-05	0.002907572	0.495743477	0.542869223	16	0	0
GMPS	0.0316772	0.030476191	0.001201035	0.032876566	0.496092809	0.543218597	10	0	0
CCR6	0.7209347	0.717724142	0.003210603	0.006350942	0.49624343	0.543345629	1	0	0
SIRT2	0.069233	0.067167769	0.00206526	0.050670284	0.496269407	0.543345629	18	0	0
ARPC5L	0.0695583	0.06998375	-0.00042547	-0.0076669	0.496356174	0.543407455	8	0	0
06-Mar	0.0588405	0.058887042	-4.65E-05	0.003656174	0.496817532	0.543879349	8	0	0
GMDS	0.043805	0.043825626	-2.06E-05	-0.00031823	0.497056067	0.54410727	6	0	0
PPCS	0.1320985	0.131631117	0.000467375	0.015488705	0.497530843	0.54459375	11	0	0
RSPH4A	0.0509992	0.047657776	0.003341427	0.063729319	0.497724809	0.544772819	10	0	0
SMIM13	0.0432632	0.042580901	0.000682249	0.015587423	0.497846933	0.544873236	10	0	0
PROSER1	0.0729105	0.073321769	-0.00041126	0.003946304	0.49811431	0.545132605	14	0	0
C6orf62	0.0923235	0.089768166	0.002555377	0.004359352	0.498433625	0.545448779	7	0	0
PMS1	0.0471767	0.045240449	0.001936272	0.031819205	0.498972214	0.546004858	11	0	0
MLXIPL	0.3269018	0.318500953	0.008400884	0.125897162	0.499722842	0.54679288	11	0	0
FBXO45	0.0397264	0.039970356	-0.00024398	0.017743849	0.499882761	0.546934497	8	0	0
HIST1H2BG	0.1258632	0.116227388	0.009635845	0.11847501	0.499937354	0.546960862	9	0	0
FHOD3	0.0385351	0.037685669	0.000849437	0.066135539	0.500048116	0.547048674	4	0	0
LRP1	0.0410205	0.041168713	-0.00014821	0.00362231	0.500354261	0.547316827	13	0	0
SETD3	0.0331918	0.033090433	0.000101352	0.001994587	0.500330126	0.547316827	14	0	0
RHEBL1	0.1336484	0.132526124	0.001122287	0.028653645	0.501270991	0.548252728	8	0	0
RELA	0.0467837	0.046082555	0.000701096	0.010988597	0.501701378	0.548689995	8	0	0
PKD2L2	0.8032782	0.822576818	-0.01929857	-0.03383437	0.502139717	0.549135905	1	0	0
NUP43	0.1169951	0.116311767	0.000683322	-0.00877772	0.50289137	0.54989085	7	0	0
KSR1	0.1621912	0.160341235	0.001849971	0.04236719	0.502865405	0.54989085	4	0	0
SOX4	0.118115	0.121273248	-0.00315828	-0.00609151	0.502958667	0.54993091	12	0	0
FARSB	0.0905889	0.088500495	0.002088405	0.024585354	0.503203903	0.550154414	8	0	0
KIN	0.0440956	0.04411736	-2.17E-05	0.004248403	0.503224427	0.550154414	15	0	0
DPP10	0.1413902	0.139136779	0.00225345	0.035584494	0.503677896	0.550616611	3	0	0
RHPN2	0.1701147	0.1583286	0.011786116	0.169498602	0.504037294	0.550975921	4	0	0
TMEM44	0.0368097	0.035255131	0.001554616	0.040119087	0.504533561	0.551484792	9	0	0
ACTR6	0.6342582	0.651553064	-0.01729487	-0.03673917	0.504723571	0.551658865	2	0	0
TMEM214	0.159118	0.158437389	0.000680581	-0.00254259	0.504814991	0.551725164	8	0	0
SUPT3H	0.0429089	0.041250953	0.001657941	0.050255551	0.504852354	0.55173238	12	0	0
PNKP	0.1897093	0.204262268	-0.01455297	-0.03135523	0.505684179	0.552607776	4	0	0
PRKACB	0.0436759	0.043911885	-0.00023596	-0.01412193	0.505808664	0.552710137	6	0	0
ALYREF	0.0390759	0.039179701	-0.00010379	0.002471455	0.505913329	0.55279083	7	0	0
RAPGEF2	0.0856949	0.085014982	0.000679948	0.00210221	0.505985797	0.552836334	5	0	0
PHGDH	0.8849193	0.883269376	0.00164995	0.002662331	0.506239837	0.553080204	1	0	0
DPF1	0.1417698	0.140909834	0.000859984	0.018098492	0.50660182	0.553441969	6	0	0
TMEM184B	0.1662949	0.15871111	0.007583777	0.052859781	0.507888301	0.554813604	9	0	0
LETM2	0.0755068	0.072533825	0.002973	0.055382394	0.509036946	0.55603451	10	0	0
ULBP2	0.0923754	0.08572719	0.006648193	0.163075072	0.509224353	0.556205347	7	0	0
NSL1	0.0856876	0.086092426	-0.00040482	-0.02159923	0.50961202	0.556594885	8	0	0
ARF1	0.1173792	0.118779751	-0.00140052	0.013490931	0.510421487	0.557411093	10	0	0
XRRA1	0.0638322	0.060059525	0.003772712	0.029713661	0.510391746	0.557411093	6	0	0
SHOC2	0.0473509	0.047319892	3.10E-05	-0.00044653	0.510679473	0.557624926	12	0	0
MLEC	0.0997718	0.100759272	-0.00098751	-0.00776498	0.510677888	0.557624926	10	0	0
AHI1	0.0490192	0.047596232	0.001423012	0.031377489	0.511182815	0.558140559	10	0	0
ZC3H6	0.0397521	0.038914781	0.000837277	0.020952173	0.511435514	0.558382479	10	0	0
ZNHIT2	0.3248878	0.326831582	-0.00194381	0.00517977	0.512548239	0.559529228	8	0	0
CCDC89	0.5834602	0.588302622	-0.00484242	-0.00050411	0.512529242	0.559529228	6	0	0
FOXJ2	0.1221307	0.118696872	0.003433859	0.017078732	0.513844678	0.560910361	9	0	0
DPH7	0.0717844	0.068959311	0.00282508	0.054080785	0.515106617	0.562253669	5	0	0
C16orf90	0.8233418	0.829564082	-0.00622227	-0.01084392	0.51521357	0.562336189	3	0	0
APOD	0.6810892	0.683343316	-0.00225412	0.00136957	0.515315885	0.562413637	7	0	0
EIF2B4	0.0438432	0.042952242	0.000890932	0.025608951	0.516125674	0.563229289	14	0	0
MFSD6	0.2141809	0.202827935	0.01135301	0.133929115	0.516126037	0.563229289	4	0	0
SPRED3	0.110538	0.101564693	0.008973312	0.050423589	0.516718172	0.56384116	6	0	0
ACOT13	0.0968276	0.101125413	-0.00429785	0.006522718	0.517132244	0.564258664	7	0	0

MAN1A2	0.0335466	0.034708781	-0.00116218	-0.02427636	0.517851362	0.564974576	5	0	0
CCDC77	0.0470946	0.046221762	0.000872827	0.013464239	0.517840643	0.564974576	15	0	0
RNF5	0.0568558	0.056605193	0.00025056	0.010064351	0.517957481	0.565055981	29	0	0
CDCA3	0.0306766	0.030597591	7.90E-05	-0.00403308	0.518533943	0.565650456	13	0	0
NUCKS1	0.1494327	0.151092718	-0.00165998	0.00467904	0.51903897	0.566166938	7	0	0
PAIP2B	0.0390347	0.039111468	-7.68E-05	0.002687873	0.519335611	0.56645214	5	0	0
CDK8	0.0410689	0.041608157	-0.00053923	-0.01223519	0.519363595	0.56645214	9	0	0
ACOT1	0.4046333	0.415632186	-0.01099891	-0.03112328	0.519491979	0.566557713	2	0	0
UTRN	0.0453189	0.046420946	-0.00110201	-0.02720982	0.519617385	0.566660024	6	0	0
JAK2	0.0719078	0.072453101	-0.0005453	0.004646758	0.519749036	0.566769133	4	0	0
PIGF	0.1137284	0.114843372	-0.00111498	0.000881185	0.520142113	0.567163289	11	0	0
TMED4	0.038069	0.037115173	0.000953822	0.025191985	0.520837613	0.567887138	6	0	0
SASH1	0.556074	0.564304078	-0.00823004	-0.03077814	0.520884376	0.567903603	5	0	0
SAMD4A	0.037755	0.037219991	0.000534998	0.012989292	0.520940247	0.567929994	10	0	0
TBC1D2B	0.0317668	0.031250431	0.000516349	0.015610281	0.521118267	0.568089542	7	0	0
KIAA1551	0.3044368	0.304818571	-0.00038176	0.019088622	0.521424374	0.568388694	6	0	0
PSME4	0.0874671	0.087202625	0.000264482	-0.00782818	0.522304266	0.569313237	3	0	0
MPHOSPH1	0.0471654	0.048411092	-0.00124574	-0.00201316	0.522535427	0.569530592	11	0	0
TRIM35	0.0379522	0.037529751	0.000422465	0.01036148	0.522936467	0.569933066	5	0	0
FAM98A	0.0273775	0.026958706	0.000418816	0.013566269	0.523024389	0.569994255	13	0	0
MGAM	0.5247541	0.541391901	-0.01663777	-0.04420234	0.523118123	0.570061769	1	0	0
WDR62	0.0398423	0.040093212	-0.00025092	-0.01163573	0.523414316	0.570349889	11	0	0
FAM168B	0.0469837	0.045696043	0.00128767	0.028631039	0.523631065	0.570551412	8	0	0
PCF11	0.0435934	0.04304889	0.000544491	0.012383921	0.523706771	0.570599238	10	0	0
ALMS1	0.0487313	0.044763629	0.003967671	0.055071849	0.524025466	0.570911788	7	0	0
CWC22	0.0395471	0.039918651	-0.00037156	-0.00089992	0.524373346	0.571256094	10	0	0
SETD1B	0.0311758	0.031358914	-0.00018311	-0.00801733	0.524948922	0.571848398	12	0	0
RMND5A	0.0219453	0.02067096	0.001274374	0.064585986	0.525037859	0.571875814	2	0	0
CCDC93	0.0391159	0.034964206	0.004151702	0.110245354	0.525006551	0.571875814	5	0	0
PLCH1	0.1088793	0.104743236	0.004136066	0.069122656	0.525704397	0.572567043	9	0	0
PHB	0.0772085	0.077109627	9.89E-05	-0.00816564	0.52584196	0.572682093	9	0	0
AMDHD1	0.0536868	0.048307256	0.005379518	0.114552351	0.526830444	0.573723791	10	0	0
TMEM45A	0.1342838	0.131928903	0.002354865	0.010253342	0.527155187	0.574042586	8	0	0
SNX4	0.0343899	0.032903915	0.001486009	0.031465539	0.527813499	0.574724558	8	0	0
NDUFAF4	0.0526181	0.053586247	-0.00096817	-0.01773954	0.528268934	0.575185552	10	0	0
NCOR2	0.0703486	0.067055346	0.003293268	0.081901936	0.528676977	0.575594892	6	0	0
PRADC1	0.0341929	0.03357398	0.000618897	0.024048384	0.528776458	0.575668258	15	0	0
NOL10	0.0548094	0.054391128	0.000418321	0.037502747	0.529067872	0.57588065	6	0	0
C7orf43	0.4629013	0.464257394	-0.00135613	0.007791333	0.529023436	0.57588065	6	0	0
ITGA7	0.0566251	0.056436622	0.000188507	-0.00064643	0.529060386	0.57588065	12	0	0
LYSMD4	0.0352558	0.035903275	-0.00064744	-0.01694212	0.529818506	0.576662706	6	0	0
ERCC6L2	0.0924377	0.097457081	-0.00501935	-0.00214695	0.530573166	0.577449047	11	0	0
GALK2	0.0626968	0.062430945	0.000265851	0.010334936	0.530734324	0.577589396	9	0	0
ELOVL1	0.0611593	0.059958056	0.001201257	0.005636851	0.530797552	0.577623158	14	0	0
SDCCAG8	0.0430384	0.043075649	-3.72E-05	-0.00059864	0.531213717	0.578040965	14	0	0
TSR3	0.0566306	0.053626443	0.003004131	0.06597077	0.531481904	0.578297708	9	0	0
NHLRC3	0.0710904	0.071760825	-0.00067038	-0.00063219	0.531696838	0.57849648	14	0	0
CNOT3	0.0366875	0.03558336	0.001104141	0.033509564	0.531921388	0.578705691	8	0	0
TRIM11	0.1347221	0.132654348	0.00206775	0.014083755	0.531982226	0.578736774	10	0	0
C5orf34	0.0659949	0.064469077	0.001525815	0.008301738	0.532380325	0.579134735	12	0	0
CIB1	0.0617031	0.060943092	0.000759985	0.027739624	0.532761011	0.579513705	9	0	0
QKI	0.0479575	0.047298014	0.000659445	0.013846853	0.533139104	0.57988981	6	0	0
CLYBL	0.1395891	0.136591898	0.002997216	0.094485035	0.533373397	0.580109468	3	0	0
HRG	0.684838	0.703503602	-0.01866563	-0.03824411	0.534111019	0.5808765	1	0	0
TRPT1	0.0485974	0.047499448	0.001097974	0.013714543	0.534219286	0.580959019	10	0	0
FOSL2	0.0398189	0.040571902	-0.00075299	-0.02153337	0.534646782	0.581388668	12	0	0
GTF3C4	0.0431311	0.04312449	6.61E-06	-0.00120184	0.534989333	0.581725898	9	0	0
SGK1	0.1910871	0.18088293	0.010204146	0.129198844	0.535457656	0.582164547	7	0	0
C6orf120	0.0528087	0.053837382	-0.00102868	-0.01543937	0.535989071	0.582706993	8	0	0
ENAH	0.0635078	0.064084072	-0.00057627	0.000192898	0.536182301	0.582881735	9	0	0
MAZ	0.0536517	0.052137153	0.001514527	0.023850334	0.536702519	0.583411902	6	0	0
ANKMY2	0.0452539	0.044526226	0.000727665	0.018759436	0.536763586	0.583442924	12	0	0
AEN	0.0981023	0.09856965	-0.00046734	0.0183408	0.536913784	0.583570818	10	0	0

HSDL1	0.0582242	0.055669057	0.002555159	0.038239831	0.537652004	0.584302371	9	0	0
TOB2	0.0464793	0.04731587	-0.00083657	-0.02240478	0.537636822	0.584302371	9	0	0
WDR3	0.0387713	0.040175641	-0.00140439	-0.02732432	0.537771529	0.584396858	13	0	0
MED13L	0.210194	0.205024182	0.005169847	0.010364701	0.537940365	0.584544916	6	0	0
OXGR1	0.139593	0.133575565	0.006017408	0.08699003	0.539457593	0.586158077	8	0	0
TYW5	0.0484048	0.048682076	-0.00027724	-0.00595093	0.539677255	0.586361233	11	0	0
OR13C4	0.5773643	0.565446029	0.011918296	0.029575018	0.541270381	0.588056548	1	0	0
LAPTM4A	0.1186084	0.112327008	0.006281353	0.032943452	0.541438588	0.588203665	10	0	0
LPIN2	0.0163122	0.01610584	0.000206381	0.011280686	0.541905439	0.588675183	3	0	0
HADHA	0.0482012	0.047431859	0.000769296	0.017720617	0.542297083	0.589064951	12	0	0
RAD54B	0.3178552	0.328614948	-0.01075977	-0.04680871	0.543626151	0.590472878	3	0	0
CEP192	0.1124679	0.116051659	-0.00358377	-0.00451961	0.54510723	0.592045736	7	0	0
FAM118B	0.1077001	0.107708045	-7.92E-06	0.012810317	0.54547907	0.592413721	13	0	0
ABCB8	0.1131372	0.11344397	-0.00030679	0.02425826	0.546043519	0.592990831	9	0	0
PPP4R1	0.3313626	0.337726997	-0.00636438	-0.01323064	0.546231726	0.593159305	5	0	0
UGT1A3	0.820371	0.82631877	-0.00594776	-0.01069862	0.546349928	0.593251744	3	0	0
GGCT	0.1579127	0.148103702	0.009809029	0.086840473	0.546489125	0.593366968	1	0	0
YPEL5	0.0429258	0.042062104	0.000863666	0.009255316	0.547073257	0.593965251	12	0	0
POLD4	0.0939808	0.088340589	0.005640213	0.080458288	0.547236968	0.594107031	1	0	0
FOXC1	0.0515803	0.053411577	-0.00183131	-0.03031248	0.547506262	0.594363414	6	0	0
CRLS1	0.04798	0.048135231	-0.00015523	-0.01088294	0.548421481	0.595320927	4	0	0
BCHE	0.4240365	0.425756045	-0.00171953	-0.01349049	0.549544872	0.596468189	5	0	0
NAT10	0.0803948	0.082100682	-0.00170587	0.015086541	0.549913382	0.596832048	12	0	0
LYRM4	0.0336176	0.032819742	0.000797891	0.014333547	0.550165313	0.596960984	5	0	0
PDSS1	0.2621483	0.270485842	-0.00833756	-0.00972749	0.550144196	0.596960984	3	0	0
MCRS1	0.0509979	0.051252763	-0.00025481	0.001720956	0.550144911	0.596960984	10	0	0
LENG1	0.0815328	0.081808731	-0.00027589	-0.01646511	0.550117841	0.596960984	11	0	0
SPCS2	0.043993	0.044249572	-0.00025655	-0.00727118	0.550433432	0.597215778	5	0	0
PILRB	0.0374904	0.036490584	0.000999818	0.028170051	0.551114215	0.597882088	3	0	0
RFX3	0.0388775	0.039790027	-0.00091253	-0.01947977	0.551108803	0.597882088	7	0	0
CYP3A5	0.3443367	0.349106939	-0.00477029	-0.01929282	0.551613832	0.59838791	1	0	0
WDR91	0.026003	0.025471262	0.000531735	0.015601243	0.552326301	0.599124556	5	0	0
ZNF843	0.057378	0.054504848	0.002873104	0.05499768	0.552544281	0.59932476	5	0	0
FAM120A	0.0424855	0.042443621	4.19E-05	0.017355727	0.552928394	0.599697432	7	0	0
RPS17	0.0215729	0.021295943	0.000276922	0.012709523	0.552954735	0.599697432	1	0	0
TMEM258	0.0595894	0.061895896	-0.00230648	-0.00721335	0.553290351	0.600025137	16	0	0
IKZF2	0.054552	0.052701941	0.001850035	0.019623676	0.553578851	0.60030171	3	0	0
WNK1	0.0358395	0.035486605	0.000352926	0.00930614	0.553973208	0.600693033	8	0	0
RPL35	0.0549477	0.054518374	0.00042932	-0.00162295	0.554425023	0.601146609	7	0	0
CYP46A1	0.0433478	0.04176954	0.001578235	0.041347551	0.554500335	0.601191924	16	0	0
RAB35	0.1229759	0.121710337	0.001265581	0.016392244	0.555215038	0.601930423	6	0	0
PTK2B	0.0410535	0.039955509	0.001098009	0.01996154	0.555925244	0.602663957	7	0	0
ETHE1	0.1051187	0.100091809	0.005026879	0.025483457	0.55602269	0.602733166	7	0	0
THAP9	0.0504298	0.051088578	-0.00065883	-0.01016387	0.557405235	0.60419534	11	0	0
TBC1D10A	0.1255724	0.122036023	0.003536423	0.033697858	0.557697669	0.604475791	7	0	0
WDR26	0.7890509	0.785604036	0.003446883	0.006236846	0.558212255	0.604996979	1	0	0
RECQL	0.0434497	0.043016671	0.000432988	0.016589519	0.558808189	0.605580903	11	0	0
MGAT5B	0.3606746	0.343999402	0.016675223	0.067595672	0.558818551	0.605580903	2	0	0
DYNLL1	0.0656086	0.061183194	0.004425412	0.093361578	0.558898965	0.605631454	8	0	0
UFM1	0.0501913	0.04857336	0.001617921	0.023507718	0.559030098	0.605736956	13	0	0
NSUN3	0.2053644	0.207452041	-0.0020876	-0.0145546	0.55920516	0.605853443	15	0	0
CDYL2	0.1732342	0.171691372	0.001542815	0.020347016	0.559175258	0.605853443	9	0	0
NCAPG2	0.0831589	0.076584117	0.006574791	0.052743486	0.559795753	0.606420041	4	0	0
ZBTB5	0.031057	0.030882538	0.000174423	0.006827315	0.559766587	0.606420041	3	0	0
SH3BGR	0.0734565	0.075176393	-0.0017199	-0.00458774	0.559964344	0.606566039	9	0	0
IFT20	0.0369537	0.036392855	0.00056089	0.020677794	0.560053482	0.606625959	15	0	0
DNAJB11	0.0363577	0.035379969	0.000977742	0.027526086	0.560647696	0.607232915	12	0	0
SCYL3	0.1512965	0.152099064	-0.00080259	-0.00294709	0.561384701	0.607994445	8	0	0
AURKA	0.0407051	0.039898129	0.000807004	0.027485843	0.562537974	0.609206684	11	0	0
OSBP	0.1549604	0.153882991	0.001077435	0.052174038	0.563239905	0.60993002	8	0	0
DNAJC10	0.0983228	0.095284816	0.00303798	0.015659564	0.563786876	0.610485475	9	0	0
ING5	0.0343957	0.034419316	-2.36E-05	0.003737175	0.564253672	0.610917171	3	0	0
FOXM1	0.0445977	0.04541867	-0.00082099	-0.02179142	0.564231133	0.610917171	6	0	0

CCDC102A	0.0284981	0.028777902	-0.00027976	-0.00879175	0.564522857	0.611171725	5	0	0
HMGCS1	0.0302993	0.030072632	0.000226657	0.005041272	0.56532621	0.612004521	7	0	0
C7orf31	0.0418839	0.041901744	-1.78E-05	0.008011226	0.56580266	0.612483343	9	0	0
HSPE1-MOE	0.0506592	0.051621714	-0.0009625	-0.00907479	0.567231908	0.613993453	22	0	0
KIF18B	0.0776591	0.0726871	0.004972009	0.031490763	0.567420511	0.614160538	8	0	0
EXOSC8	0.2194257	0.219522999	-9.73E-05	0.030223468	0.567581057	0.614297239	8	0	0
FBXO30	0.0721688	0.071010481	0.001158369	0.021631732	0.56779908	0.614496126	10	0	0
ERLEC1	0.0589884	0.05729599	0.001692382	0.014293049	0.568630548	0.615358846	14	0	0
LNX2	0.050701	0.049915617	0.000785359	0.005951014	0.568764154	0.615466297	7	0	0
CIAPIN1	0.0363123	0.036279741	3.26E-05	0.000673475	0.568866729	0.615540157	6	0	0
SUPT4H1	0.0388977	0.031517792	0.007379881	0.236036093	0.569517348	0.616206983	1	0	0
TDP2	0.0373105	0.03617265	0.001137804	0.033092456	0.56992846	0.616614601	6	0	0
MAFK	0.3865993	0.395053391	-0.00845406	-0.01507443	0.570046769	0.616705401	7	0	0
SEH1L	0.0752209	0.074693012	0.000527854	0.017966279	0.570341423	0.616986958	2	0	0
ESF1	0.0785421	0.077335174	0.00120688	0.010605584	0.570446973	0.617063923	15	0	0
ZNF407	0.0336223	0.033381699	0.000240637	0.005869323	0.570633958	0.617228963	6	0	0
GAS2L3	0.0971033	0.091776513	0.005326807	0.165564884	0.57197344	0.618640513	10	0	0
NAB2	0.0295958	0.029537271	5.86E-05	0.004051173	0.572486007	0.619157563	10	0	0
AGPAT1	0.0469839	0.046498053	0.000485816	0.010315511	0.572879249	0.619545505	25	0	0
MIA3	0.2274649	0.23076959	-0.00330473	0.042910746	0.573419396	0.620092263	8	0	0
EHD4	0.0855794	0.086218801	-0.00063936	-0.01257353	0.57579372	0.622622304	4	0	0
KCTD10	0.0610102	0.061171781	-0.00016153	0.001253199	0.576743894	0.623612159	8	0	0
PEX1	0.0373161	0.037532663	-0.00021661	-0.01228519	0.576822902	0.623642816	8	0	0
RPL30	0.1873055	0.187411716	-0.00010617	0.02712837	0.576841788	0.623642816	6	0	0
REC8	0.4389211	0.437291093	0.001629973	0.009337583	0.577116234	0.623901921	8	0	0
ZBTB39	0.2218382	0.222953739	-0.00111552	-0.01252325	0.577246598	0.624005243	8	0	0
MYBBP1A	0.0278411	0.027063534	0.000777544	0.01526374	0.579296544	0.6261835	4	0	0
KLF12	0.04258	0.043467327	-0.00088733	-0.02170873	0.580624469	0.627581082	9	0	0
ZSCAN30	0.0388998	0.038907636	-7.88E-06	0.004516543	0.580881413	0.627820971	14	0	0
CEP19	0.0269101	0.026687098	0.000223022	0.005696782	0.581329029	0.6282669	7	0	0
BLOC1S1	0.216824	0.218442047	-0.00161807	-0.00321725	0.582279379	0.629256067	13	0	0
KCTD5	0.0543511	0.054180874	0.000170188	0.004501156	0.582928486	0.629881639	10	0	0
VPS45	0.0417243	0.041048454	0.000675894	0.012104953	0.583063538	0.629989615	10	0	0
TMEM70	0.0408377	0.040296055	0.000541611	0.013370725	0.583301278	0.630208525	11	0	0
USP6NL	0.0609812	0.055014148	0.005967026	0.092320866	0.583377582	0.630253	6	0	0
HSPA2	0.9051796	0.907236287	-0.00205673	-0.00323861	0.584328292	0.631242079	1	0	0
MTMR10	0.0446599	0.043079209	0.001580647	0.045701538	0.585613205	0.632592053	5	0	0
STXBP4	0.0488129	0.047445414	0.00136745	0.031853757	0.586103033	0.633083048	11	0	0
GPAA1	0.0597378	0.057899278	0.001838499	0.032383798	0.587015525	0.634030499	8	0	0
KAT6A	0.0832656	0.082235747	0.001029816	0.017891463	0.587483303	0.634497533	3	0	0
KLRC4	0.7270106	0.73892495	-0.01191437	-0.02500306	0.587671994	0.634663107	2	0	0
ORC2	0.3040185	0.315072304	-0.01105377	-0.01990143	0.589018146	0.6360786	12	0	0
TRMT10A	0.0404061	0.040535595	-0.00012953	0.006498424	0.589456632	0.636475478	10	0	0
HNRNPA2B1	0.0463191	0.046783529	-0.00046444	-0.00301556	0.589426797	0.636475478	12	0	0
MSL1	0.0592227	0.057721878	0.001500866	0.021655287	0.590072536	0.637102155	6	0	0
TMEM14C	0.0279275	0.026967153	0.000960337	0.040930755	0.591404729	0.63850209	3	0	0
PEBP4	0.0342068	0.033555682	0.000651151	0.017733931	0.592393264	0.639530853	2	0	0
MORN3	0.0350609	0.032080592	0.002980287	0.098163124	0.592848965	0.639984293	2	0	0
DAP3	0.0506186	0.049986521	0.000632094	0.015122532	0.592887504	0.639987378	1	0	0
GTF2E1	0.0509521	0.049526414	0.001425644	0.029904968	0.593710999	0.640837725	12	0	0
CCDC58	0.0400108	0.039228418	0.000782386	0.015559493	0.595914495	0.643138719	7	0	0
FAM162A	0.0400108	0.039228418	0.000782386	0.015559493	0.595914495	0.643138719	7	0	0
FAM126A	0.1849424	0.186265205	-0.0013228	0.002485549	0.596253845	0.643466243	10	0	0
HS3ST5	0.0801236	0.074251451	0.005872136	0.105518063	0.596752902	0.64396607	3	0	0
RPS12	0.030008	0.029895671	0.000112291	0.004098913	0.597979531	0.645250924	8	0	0
COX11	0.0665963	0.063620548	0.002975705	0.046732676	0.598509124	0.645783532	10	0	0
LIX1L	0.036087	0.03604601	4.10E-05	0.007585894	0.598616673	0.645860722	7	0	0
NBEAL2	0.0297939	0.027197332	0.002596545	0.082939916	0.598848912	0.646072426	6	0	0
MTTP	0.0308752	0.030662701	0.000212514	0.007012557	0.599420852	0.646650571	11	0	0
LRRC19	0.4421741	0.450101341	-0.00792723	-0.02507326	0.601122608	0.648447411	1	0	0
PRICKLE4	0.0559765	0.055617898	0.000358583	0.00890046	0.601675838	0.648966134	7	0	0
PHF10	0.04461	0.041653381	0.002956605	0.054643138	0.601672681	0.648966134	5	0	0
MARK1	0.0795903	0.078870612	0.000719737	0.00718741	0.603548246	0.650946563	8	0	0

IGFL4	0.0688575	0.064470336	0.004387116	0.079948426	0.60368543	0.651055371	3	0	0
GNA12	0.0380169	0.036184146	0.001832787	0.059733372	0.604029726	0.651387516	8	0	0
ASB8	0.9117429	0.918291317	-0.00654839	-0.01021319	0.60414833	0.651476249	1	0	0
RB1CC1	0.8427007	0.845770397	-0.00306974	-0.00518441	0.605165205	0.652533553	1	0	0
SCAF8	0.3652047	0.36141931	0.003785419	0.059338007	0.606880023	0.654343258	3	0	0
KIAA0319L	0.1594507	0.157860252	0.001590421	0.001204494	0.607765903	0.655180625	15	0	0
BTBD7	0.1180944	0.118021741	7.27E-05	-0.00229039	0.607760446	0.655180625	10	0	0
USHBP1	0.1379313	0.126306014	0.011625265	0.118077917	0.607766238	0.655180625	1	0	0
NOC2L	0.0343986	0.034097056	0.000301497	0.010027434	0.608736557	0.656147769	6	0	0
B3GALT6	0.0482802	0.048075238	0.000204924	0.011157709	0.60872683	0.656147769	2	0	0
ANO8	0.1575714	0.157104174	0.000467242	0.028193855	0.609224869	0.656634653	7	0	0
TMEM150B	0.5705573	0.584252848	-0.01369555	-0.03363845	0.609409073	0.656793722	1	0	0
CSPP1	0.0589051	0.058491926	0.000413151	0.003705997	0.610520522	0.657952057	13	0	0
C1orf131	0.0398998	0.039429265	0.000470571	0.013396198	0.611580823	0.659015538	8	0	0
GNPAT	0.0398998	0.039429265	0.000470571	0.013396198	0.611580823	0.659015538	8	0	0
DOCK9	0.0314956	0.032332303	-0.0008367	-0.01252362	0.611941265	0.659364324	5	0	0
NRG4	0.0311007	0.02986882	0.001231857	0.039456538	0.612670007	0.660109884	7	0	0
NUMB	0.3580023	0.324993329	0.033008953	0.135582347	0.613353311	0.660806403	1	0	0
ZNF165	0.1122724	0.115819321	-0.00354694	-0.0121706	0.614515881	0.662019151	7	0	0
PSMC1	0.1772823	0.175237636	0.002044641	0.051826548	0.614714985	0.662193873	6	0	0
PSG11	0.7868488	0.7855761	0.001272736	0.002495421	0.616083916	0.663628678	2	0	0
NADSYN1	0.0530597	0.046665125	0.006394562	0.143705194	0.617043668	0.664622582	8	0	0
ZNF428	0.1871714	0.190206789	-0.00303534	-0.0006271	0.617713321	0.665303917	9	0	0
CSRP2	0.0428376	0.042826969	1.06E-05	0.000777536	0.618169328	0.665755078	7	0	0
SP4	0.0866546	0.085538556	0.001116084	-0.00133324	0.619763436	0.667431821	4	0	0
FAM111A	0.0399154	0.039201321	0.000714076	0.011962153	0.620033784	0.667682874	16	0	0
MRPS7	0.0500499	0.049271487	0.000778384	0.011063073	0.620256794	0.667882924	7	0	0
CIZ1	0.459851	0.452199991	0.007651043	0.143073493	0.620847524	0.668424496	3	0	0
ZNF780A	0.0433595	0.04242964	0.000929894	0.017791548	0.620871549	0.668424496	13	0	0
GOLT1B	0.1110406	0.109137889	0.001902662	0.019138421	0.621539389	0.669103324	11	0	0
HSPD1	0.0266658	0.027071236	-0.00040542	-0.01285393	0.62169438	0.669230008	2	0	0
NCOA7	0.0406082	0.040217775	0.00039045	0.010486657	0.622000946	0.669519833	8	0	0
SPRED2	0.0817768	0.081860553	-8.38E-05	0.015150831	0.622730558	0.67026496	9	0	0
DVL1	0.0467541	0.046046374	0.000707774	0.016932814	0.622959875	0.670471546	2	0	0
NAA20	0.1294865	0.131859017	-0.00237252	-0.02454091	0.623139433	0.670624557	11	0	0
PPFIBP1	0.1090135	0.105724795	0.003288752	0.030381013	0.623708164	0.671196354	6	0	0
MAF1	0.060001	0.060278255	-0.00027722	-0.00549727	0.623825518	0.671282367	12	0	0
DDIT4	0.0367692	0.035546238	0.00122292	0.045831679	0.624588803	0.672063397	13	0	0
PRCP	0.8827934	0.877422443	0.005370991	0.008705377	0.625767998	0.67329183	1	0	0
NUDT8	0.1353879	0.133071933	0.002316015	0.00660966	0.626557807	0.674101183	7	0	0
POLR2J	0.0894156	0.079839932	0.009575701	0.146115898	0.627424906	0.674993588	1	0	0
PLEKHH3	0.027904	0.027625225	0.000278788	0.010957949	0.628083689	0.675661791	12	0	0
ACTR3B	0.0343408	0.033681949	0.000658816	0.018185287	0.628230651	0.675779354	4	0	0
IL16	0.815731	0.817441012	-0.00171004	-0.00298465	0.628982813	0.67654787	1	0	0
ATF6	0.0686536	0.066544012	0.002109622	0.022343777	0.630733899	0.678390694	9	0	0
SESN1	0.0472244	0.046710889	0.00051348	0.008054675	0.631740425	0.679432527	9	0	0
FAM76B	0.0256663	0.025725996	-5.97E-05	-0.00461501	0.632140961	0.679822537	7	0	0
ZNF687	0.0360315	0.034541246	0.001490267	0.049291648	0.633065809	0.680776326	6	0	0
FADS2	0.0391417	0.038795088	0.000346573	0.010012163	0.633947524	0.681683621	18	0	0
RBM33	0.1373581	0.137749195	-0.00039112	0.005882502	0.635760546	0.683551202	9	0	0
SLC38A9	0.7796989	0.785113656	-0.00541471	-0.01025086	0.636036183	0.683806569	2	0	0
PTRHD1	0.0361318	0.035798784	0.000332997	0.011752227	0.636238397	0.683982971	15	0	0
CNR2	0.0634292	0.064089104	-0.00065986	-0.0088752	0.63741677	0.685208701	5	0	0
DIRC2	0.0767623	0.076865019	-0.00010267	0.013352544	0.63838189	0.686205057	9	0	0
FEN1	0.0394202	0.039177798	0.000242377	0.006453956	0.639303142	0.687154141	17	0	0
MBD5	0.045291	0.044355223	0.000935781	0.027953904	0.640526117	0.688427399	7	0	0
HNRNPD	0.0350322	0.034629664	0.000402519	0.011591802	0.64079981	0.688680292	7	0	0
DSE	0.0415333	0.041893075	-0.00035974	-0.00214131	0.641541574	0.68943617	4	0	0
NDUFB5	0.0458488	0.04518214	0.000666634	0.023432669	0.642948612	0.690906854	6	0	0
IL25	0.6061656	0.617301933	-0.01113635	-0.026045	0.64319705	0.691132416	4	0	0
FAM110A	0.0471626	0.046267879	0.000894763	0.023405758	0.643467006	0.691381072	4	0	0
CASKIN2	0.0417321	0.040936703	0.000795446	0.035493406	0.644593682	0.692550155	9	0	0
ATF2	0.0295302	0.028735718	0.000794527	0.020893284	0.645054175	0.693003395	9	0	0

INTS10	0.0418891	0.040958874	0.000930228	0.023493056	0.645899501	0.693869996	5	0	0
TBC1D30	0.1757933	0.175495813	0.000297529	0.109640749	0.647076636	0.695092923	7	0	0
VPS13A	0.2388502	0.23741102	0.001439206	0.06179338	0.647604166	0.695617938	4	0	0
ADARB2	0.0759953	0.072579627	0.003415704	0.064240031	0.647895557	0.695889257	13	0	0
MPRIP	0.0519121	0.050017009	0.001895051	0.040028516	0.650110298	0.698226245	5	0	0
EXTL3	0.6060395	0.602127099	0.003912356	0.009191516	0.650802856	0.698928209	1	0	0
ORMDL1	0.0345983	0.034196542	0.000401728	0.013159476	0.65124779	0.699364169	6	0	0
SCN2A	0.2127031	0.217636853	-0.00493375	-0.02259546	0.651905107	0.700028139	5	0	0
NFX1	0.0332422	0.033770251	-0.00052803	-0.01305357	0.652066531	0.700159561	6	0	0
PPIL2	0.0274204	0.027368154	5.22E-05	0.001321876	0.65259208	0.700681924	2	0	0
IFT81	0.2354013	0.235514161	-0.00011287	0.008989436	0.652909445	0.700980714	11	0	0
OPLAH	0.8326526	0.843718397	-0.01106581	-0.01765014	0.653315222	0.701374383	2	0	0
TLN1	0.0556385	0.056086714	-0.00044822	-0.0036174	0.65369145	0.701736285	7	0	0
PRKDC	0.0381266	0.038822264	-0.00069568	-0.00913399	0.654521314	0.702585091	9	0	0
PSMA7	0.0864237	0.088185061	-0.00176139	-0.00723505	0.656276333	0.704426829	6	0	0
CLCNKB	0.584926	0.580745257	0.004180783	0.00898414	0.657780023	0.705998593	2	0	0
NAA38	0.0447614	0.045258515	-0.00049716	-0.01242928	0.659390912	0.70768522	4	0	0
POLR2L	0.0287814	0.029132816	-0.00035147	-0.0130159	0.659927488	0.708218718	1	0	0
QRSL1	0.0638172	0.063802031	1.52E-05	-0.00063088	0.661067187	0.70939937	8	0	0
NARS2	0.0438216	0.044426464	-0.00060487	-0.01228282	0.661296	0.709602457	8	0	0
NOTCH1	0.0860342	0.084711748	0.001322471	0.016216252	0.663111053	0.711464966	8	0	0
ANKS1B	0.2093946	0.210144531	-0.00074996	0.014928152	0.663087747	0.711464966	10	0	0
RGPD4	0.8886629	0.892393982	-0.00373113	-0.00623634	0.663708036	0.712062887	3	0	0
IKBKB	0.1507833	0.149028411	0.001754894	0.012671141	0.664670234	0.713052534	8	0	0
ZNF524	0.0544794	0.054615871	-0.0001365	-0.01142209	0.665887837	0.714316043	5	0	0
SP7	0.0346998	0.032043912	0.002655866	0.090052648	0.66630813	0.714724156	4	0	0
MAN2A2	0.0221604	0.021770981	0.000389419	0.013689908	0.66673769	0.715142161	3	0	0
SYT17	0.0545236	0.053967351	0.000556205	0.011889832	0.667571139	0.715993299	7	0	0
POLR1A	0.0403813	0.040203755	0.000177495	0.001599774	0.669190429	0.717687128	10	0	0
CPEB2	0.0470299	0.04659411	0.000435783	0.007822736	0.669285764	0.717746455	5	0	0
WASF1	0.0389713	0.038128585	0.000842724	0.02165786	0.670576259	0.719044406	8	0	0
CDC40	0.0389713	0.038128585	0.000842724	0.02165786	0.670576259	0.719044406	8	0	0
RCOR3	0.0646259	0.066094056	-0.00146815	-0.02282724	0.670989801	0.719444827	4	0	0
TMEM170B	0.0970491	0.098891683	-0.00184261	-0.01273419	0.671193977	0.719620729	4	0	0
ELK4	0.3247204	0.325771251	-0.00105081	0.010110125	0.672504608	0.720982824	4	0	0
ANKRD49	0.0325326	0.03265872	-0.00012608	-0.00560018	0.675110569	0.723733379	7	0	0
ZNF563	0.1683589	0.159416142	0.008942716	0.218764641	0.675465651	0.724070757	6	0	0
GYPB	0.6737686	0.688089221	-0.01432065	-0.02990333	0.675807161	0.724350259	1	0	0
GYPA	0.6737686	0.688089221	-0.01432065	-0.02990333	0.675807161	0.724350259	1	0	0
FTSJ3	0.5999153	0.607453281	-0.007538	-0.01772113	0.678790551	0.727504471	1	0	0
CHD2	0.3091511	0.316470239	-0.00731911	-0.00444769	0.67921434	0.727915177	5	0	0
PLEKHG2	0.0354141	0.035153653	0.000260432	0.005684564	0.681466361	0.730285036	7	0	0
NMRK1	0.0571778	0.05152945	0.005648337	0.104925373	0.682552466	0.731405247	7	0	0
NDUFA7	0.1602968	0.156183514	0.004113296	0.035413679	0.683279759	0.732140854	5	0	0
ZBTB2	0.0293722	0.02912469	0.000247469	0.008425903	0.683794551	0.73264869	4	0	0
SMAD3	0.6270459	0.6647731	-0.03772715	-0.08300503	0.684326808	0.733175177	1	0	0
MAN1B1	0.0706056	0.066204891	0.004400711	0.069540278	0.685308293	0.734182866	3	0	0
PANK3	0.2628658	0.264935502	-0.00206973	0.000138473	0.685526343	0.734372602	8	0	0
UGT2B17	0.342225	0.344822239	-0.00259725	-0.00071637	0.685934013	0.734765436	2	0	0
THEM6	0.13392	0.134277264	-0.00035725	0.058109093	0.686351649	0.735168897	9	0	0
PPP2R5D	0.0505317	0.049706574	0.000825138	0.005823958	0.688598655	0.737531677	11	0	0
ZNF596	0.0797871	0.080301206	-0.00051415	-0.00823785	0.688783826	0.737685956	1	0	0
VAV2	0.0451048	0.044331977	0.000772796	0.007625605	0.690552898	0.739536468	5	0	0
SERTAD2	0.0367756	0.03706269	-0.00028714	-0.00684497	0.692881762	0.741986225	4	0	0
SMYD2	0.0575543	0.057305252	0.000249	0.000824517	0.696242419	0.745540539	4	0	0
SMNDC1	0.0338652	0.033812958	5.22E-05	0.003142472	0.697311457	0.746640695	10	0	0
STX18	0.065447	0.063448194	0.001998785	0.032272389	0.698491789	0.747859881	8	0	0
UBN1	0.2449773	0.239976764	0.005000576	0.004079646	0.699357063	0.748741615	2	0	0
TSEN54	0.0440563	0.043767827	0.000288434	0.011384398	0.699970275	0.749327429	8	0	0
TMX3	0.2405117	0.240044068	0.000467659	0.001172963	0.699987794	0.749327429	4	0	0
DENND1C	0.3787729	0.361239566	0.017533306	0.110675134	0.700617524	0.749956786	3	0	0
BFSP1	0.0641804	0.063712998	0.000467449	0.014932507	0.700874219	0.750186787	12	0	0
APIP	0.1797933	0.172600371	0.007192916	0.050729898	0.705495716	0.755088385	3	0	0

AP1AR	0.0502741	0.049975309	0.000298751	0.012945657	0.707042003	0.756653065	8	0	0
MCM9	0.0577212	0.05720605	0.0005151	0.012248622	0.707019178	0.756653065	2	0	0
RGS1	0.6688756	0.690614402	-0.02173878	-0.04547336	0.708454879	0.758119844	1	0	0
AMDHD2	0.3316589	0.335856023	-0.00419709	-0.00850551	0.71183599	0.761692538	5	0	0
MIA2	0.913505	0.913033105	0.000471867	0.000737337	0.715813429	0.765902861	1	0	0
HERC3	0.6819857	0.693855721	-0.01186999	-0.02453744	0.71616068	0.766228702	1	0	0
HDLBP	0.1345896	0.131302959	0.003286598	0.024273503	0.717593245	0.767715624	2	0	0
LRCH4	0.0413731	0.041296648	7.64E-05	0.005090474	0.718953411	0.769124919	9	0	0
SOX7	0.2171386	0.217576306	-0.00043774	-0.0127354	0.719898002	0.770089495	5	0	0
CANT1	0.0571072	0.056750449	0.000356712	0.020299015	0.720261278	0.77043215	6	0	0
PSG8	0.7524345	0.752233413	0.000201108	0.000380592	0.721258401	0.771452721	1	0	0
H3F3A	0.1265033	0.123572504	0.002930753	0.025551725	0.721969449	0.772167205	7	0	0
FRMPD1	0.2518635	0.248349351	0.003514147	0.112406426	0.724707596	0.775049516	4	0	0
TSPAN4	0.3462609	0.350978719	-0.00471786	-0.01628835	0.726265876	0.776669733	3	0	0
TIGD2	0.323424	0.32680859	-0.00338462	-0.01749009	0.727934132	0.778407356	5	0	0
RPS21	0.0343118	0.034630652	-0.00031885	-0.0048828	0.728624369	0.779099004	6	0	0
RNF10	0.9180781	0.919020554	-0.00094248	-0.00147404	0.730154533	0.780688626	4	0	0
CDK5RAP3	0.5066717	0.505352233	0.001319505	0.001780181	0.73102166	0.781569175	2	0	0
IRGM	0.5035606	0.50711571	-0.00355512	-0.00995263	0.732758741	0.783379673	1	0	0
CBX3	0.0328287	0.03273	9.87E-05	0.00422734	0.734695148	0.785403038	16	0	0
C21orf62	0.4783838	0.477129893	0.001253918	0.003502052	0.736067055	0.786822737	4	0	0
OSTF1	0.0464784	0.044140933	0.002337448	0.066370917	0.737525716	0.788334997	6	0	0
TRIM23	0.4845685	0.496863964	-0.01229545	-0.02406991	0.740978497	0.791978448	2	0	0
SUPT5H	0.0324552	0.031347256	0.001107905	0.032282505	0.741901985	0.792918246	4	0	0
CEP57L1	0.0501911	0.049203659	0.000987425	0.024933652	0.741949532	0.792921814	11	0	0
PPP1R21	0.0247573	0.024498868	0.000258431	0.008923055	0.742571415	0.793539138	4	0	0
ASB5	0.122252	0.121325514	0.000926448	0.010141896	0.747429558	0.798683143	1	0	0
HEATR5B	0.0456498	0.044042106	0.00160771	0.041992093	0.748326988	0.799594475	6	0	0
SH3RF1	0.3640047	0.370392123	-0.00638739	-0.01037924	0.749664116	0.800975492	3	0	0
PLXDC1	0.0192172	0.01928283	-6.56E-05	-0.00323621	0.749727553	0.800995555	1	0	0
RABL3	0.0594935	0.057409975	0.002083481	0.043634556	0.750603007	0.80188311	8	0	0
ZMAT2	0.8535152	0.856480732	-0.00296556	-0.00494614	0.75169965	0.803006845	1	0	0
MMAA	0.6607225	0.670571456	-0.00984895	-0.02105773	0.753827688	0.805232173	4	0	0
SLC39A10	0.1127511	0.111298977	0.001452102	0.017168321	0.755774065	0.807263199	1	0	0
SYS1	0.6588041	0.661628868	-0.00282477	-0.00194283	0.756042482	0.807501814	2	0	0
CRYGS	0.8946099	0.89867297	-0.00406306	-0.00671448	0.756129216	0.807546363	2	0	0
SPPL3	0.1319027	0.127118729	0.00478393	0.164291509	0.75741071	0.808866835	7	0	0
MRPL46	0.0412187	0.0409698	0.000248906	0.006428832	0.760998124	0.8126012	12	0	0
MRPS11	0.0412187	0.0409698	0.000248906	0.006428832	0.760998124	0.8126012	12	0	0
HADHB	0.1433312	0.132128916	0.011202332	0.109451636	0.763825931	0.815572205	1	0	0
ALG10B	0.087351	0.083958948	0.003392071	0.035414746	0.767209792	0.819136545	2	0	0
FAM13A	0.2740061	0.276855583	-0.00284947	-0.01570418	0.774746007	0.827035137	6	0	0
CARD6	0.1545841	0.145104075	0.009480011	0.104595662	0.775849336	0.828163643	5	0	0
THAP7	0.0228652	0.022885274	-2.01E-05	-0.00165567	0.776033781	0.828311229	8	0	0
GIP	0.4858675	0.489632526	-0.003765	-0.01091264	0.77682157	0.829102748	1	0	0
NRF1	0.0336846	0.033471511	0.000213087	0.007959752	0.780470344	0.832947524	6	0	0
FBXO48	0.0384527	0.038370434	8.23E-05	0.000534719	0.780770422	0.8332182	12	0	0
LLPH	0.0342433	0.034008744	0.000234567	0.005873556	0.783491934	0.836072782	8	0	0
TRMT44	0.1615848	0.157015516	0.004569245	0.038939291	0.785584352	0.838255752	1	0	0
TRIOBP	0.1832244	0.171789238	0.011435135	0.093255732	0.786045924	0.838698376	9	0	0
P2RX6	0.7032392	0.732948808	-0.02970958	-0.05887676	0.788364146	0.841121844	1	0	0
TTLL12	0.0270287	0.026814563	0.00021409	0.00819112	0.790038442	0.842858047	5	0	0
NOSIP	0.0344811	0.034460812	2.03E-05	-0.0046947	0.797285194	0.85048812	5	0	0
PRR12	0.0344811	0.034460812	2.03E-05	-0.0046947	0.797285194	0.85048812	5	0	0
MADCAM1	0.0656086	0.061832753	0.003775856	0.106036385	0.798727271	0.851975756	3	0	0
SPINK6	0.5331992	0.52999913	0.003200026	0.008524155	0.799539139	0.852791032	1	0	0
CD164	0.0463916	0.045807869	0.000583697	0.012567016	0.80173403	0.855081261	11	0	0
SLCO1B7	0.5735904	0.569367302	0.004223114	0.010477926	0.804372706	0.857844503	1	0	0
NOP56	0.0339395	0.033689622	0.000249861	0.011405899	0.80459172	0.858027058	3	0	0
CACNA2D3	0.1061298	0.104125675	0.002004108	0.046428031	0.807113486	0.86066513	6	0	0
PLAU	0.114349	0.095332377	0.019016602	0.2394457	0.81378298	0.867725553	1	0	0
RNF170	0.0388757	0.038959207	-8.35E-05	-0.00038737	0.826374472	0.881099306	5	0	0
FOXP2	0.058667	0.056443977	0.002223007	0.070618617	0.828319572	0.883120718	6	0	0

RABL2B	0.4517925	0.451861556	-6.91E-05	0.011368514	0.82907487	0.883873447	2	0	0
BRD3	0.0311975	0.030794271	0.000403201	0.014189257	0.829444583	0.884215039	1	0	0
INTS8	0.0622816	0.062218962	6.27E-05	0.001251555	0.832616459	0.887543612	1	0	0
CLN3	0.8718446	0.874988862	-0.00314425	-0.00513484	0.833729725	0.888677503	1	0	0
FCGRT	0.3234608	0.318631789	0.004828996	0.021045071	0.841552191	0.896962211	1	0	0
LANCL1	0.7230085	0.727463254	-0.00445478	-0.00874129	0.847320474	0.903002972	1	0	0
DGCR8	0.0379306	0.037830296	0.000100302	0.005180963	0.847299987	0.903002972	3	0	0
ZNF286A	0.0294599	0.029715314	-0.00025543	-0.00930855	0.859917658	0.916373544	1	0	0
PITPNB	0.1487676	0.145965877	0.002801767	0.072490174	0.862609057	0.919187028	4	0	0
ZSWIM6	0.0366684	0.036575981	9.24E-05	0.001184825	0.862932375	0.919476925	5	0	0
AVIL	0.8247546	0.840200234	-0.01544568	-0.02645059	0.867135195	0.923845374	1	0	0
MAPK6	0.9335078	0.934941182	-0.00143339	-0.00219009	0.867088699	0.923845374	1	0	0
OR4A5	0.1380515	0.138660767	-0.00060924	-0.00592455	0.878529396	0.935929155	1	0	0
HIC2	0.079781	0.080235961	-0.00045492	-0.00729173	0.881823911	0.939383124	1	0	0
TMEM50A	0.0294128	0.029094976	0.000317864	0.011682484	0.883686459	0.941311339	1	0	0
APTX	0.0528696	0.052753006	0.000116622	0.001266796	0.88610445	0.943830953	5	0	0
SPESP1	0.1100926	0.109685539	0.000407094	0.005662908	0.888275938	0.946087721	3	0	0
NPC2	0.0341777	0.033725306	0.000452438	0.010227123	0.897941553	0.956325616	3	0	0
CEACAM1	0.6888684	0.7051282	-0.01625976	-0.033181	0.900794499	0.959307097	1	0	0
SMG1	0.073071	0.072800247	0.00027074	0.004709618	0.90160005	0.960107967	1	0	0
GAS7	0.160031	0.151707876	0.008323122	0.072407834	0.907197778	0.966011598	1	0	0
FABP3	0.6585568	0.662377292	-0.00382045	-0.00822078	0.907632894	0.966417548	1	0	0
AIMP2	0.0765007	0.071388605	0.005112126	0.087885498	0.912275689	0.971303381	1	0	0
POMZP3	0.1839473	0.180970576	0.00297676	0.022314587	0.916806399	0.976069304	1	0	0
SUCNR1	0.8294918	0.833990299	-0.00449848	-0.00771014	0.925348624	0.98510523	1	0	0
SLC48A1	0.5278048	0.533194613	-0.00538979	-0.01438648	0.9275977	0.987382335	1	0	0
HIST2H2BF	0.1703408	0.16657819	0.003762561	0.030418274	0.92814844	0.987851319	1	0	0
HIST2H3D	0.1703408	0.16657819	0.003762561	0.030418274	0.92814844	0.987851319	1	0	0
DHRS9	0.8581731	0.859077328	-0.00090427	-0.0015019	0.939289165	0.999649348	1	0	0
OR4F5	NA	NA	NA	NA	NA	1	1	NA	NA
SLC2A5	NA	NA	NA	NA	NA	1	1	NA	NA
PRAMEF1	NA	NA	NA	NA	NA	1	1	NA	NA
HNRNPCL1	NA	NA	NA	NA	NA	1	1	NA	NA
PRAMEF4	NA	NA	NA	NA	NA	1	1	NA	NA
PRAMEF6	NA	NA	NA	NA	NA	1	1	NA	NA
PRAMEF20	NA	NA	NA	NA	NA	1	1	NA	NA
CELA3A	NA	NA	NA	NA	NA	1	1	NA	NA
ZNF683	NA	NA	NA	NA	NA	1	1	NA	NA
CYP4Z1	NA	NA	NA	NA	NA	1	1	NA	NA
ASB17	NA	NA	NA	NA	NA	1	1	NA	NA
AMY1B	NA	NA	NA	NA	NA	1	1	NA	NA
CHIA	NA	NA	NA	NA	NA	1	1	NA	NA
NOTCH2	NA	NA	NA	NA	NA	1	1	NA	NA
FAM72B	NA	NA	NA	NA	NA	1	1	NA	NA
PPIAL4G	NA	NA	NA	NA	NA	1	1	NA	NA
FAM72D	NA	NA	NA	NA	NA	1	1	NA	NA
SRGAP2B	NA	NA	NA	NA	NA	1	1	NA	NA
NBPF9	NA	NA	NA	NA	NA	1	1	NA	NA
NBPF11	NA	NA	NA	NA	NA	1	1	NA	NA
GPR89B	NA	NA	NA	NA	NA	1	1	NA	NA
PPIAL4D	NA	NA	NA	NA	NA	1	1	NA	NA
NBPF15	NA	NA	NA	NA	NA	1	1	NA	NA
LCE2C	NA	NA	NA	NA	NA	1	1	NA	NA
LCE2B	NA	NA	NA	NA	NA	1	1	NA	NA
SPRR2E	NA	NA	NA	NA	NA	1	1	NA	NA
S100A7	NA	NA	NA	NA	NA	1	1	NA	NA
XCL2	NA	NA	NA	NA	NA	1	1	NA	NA
C1orf112	NA	NA	NA	NA	NA	1	1	NA	NA
CFHR1	NA	NA	NA	NA	NA	1	1	NA	NA
CFHR2	NA	NA	NA	NA	NA	1	1	NA	NA
FAM72A	NA	NA	NA	NA	NA	1	1	NA	NA
SIPA1L2	NA	NA	NA	NA	NA	1	1	NA	NA
OPN3	NA	NA	NA	NA	NA	1	1	NA	NA

OR2L5	NA	NA	NA	NA	NA	1	1	NA	NA
OR2T12	NA	NA	NA	NA	NA	1	1	NA	NA
OR2T5	NA	NA	NA	NA	NA	1	1	NA	NA
OR2T29	NA	NA	NA	NA	NA	1	1	NA	NA
OR2T10	NA	NA	NA	NA	NA	1	1	NA	NA
FEZ2	NA	NA	NA	NA	NA	1	1	NA	NA
CDC42EP3	NA	NA	NA	NA	NA	1	1	NA	NA
ARHGAP25	NA	NA	NA	NA	NA	1	1	NA	NA
LRRTM4	NA	NA	NA	NA	NA	1	1	NA	NA
CHMP3	NA	NA	NA	NA	NA	1	1	NA	NA
RNF103-CH	NA	NA	NA	NA	NA	1	1	NA	NA
RGPD3	NA	NA	NA	NA	NA	1	1	NA	NA
CBWD2	0.3269312	0.318913794	0.008017453	0.034744716	0.986238469	1	1	0	0
FOXD4L1	NA	NA	NA	NA	NA	1	1	NA	NA
RABL2A	NA	NA	NA	NA	NA	1	1	NA	NA
CCDC74B	NA	NA	NA	NA	NA	1	1	NA	NA
POTEI	NA	NA	NA	NA	NA	1	1	NA	NA
CFC1B	NA	NA	NA	NA	NA	1	1	NA	NA
CFC1	NA	NA	NA	NA	NA	1	1	NA	NA
POTEJ	NA	NA	NA	NA	NA	1	1	NA	NA
TUBA3D	NA	NA	NA	NA	NA	1	1	NA	NA
COBLL1	NA	NA	NA	NA	NA	1	1	NA	NA
MYO3B	NA	NA	NA	NA	NA	1	1	NA	NA
GRM7	NA	NA	NA	NA	NA	1	1	NA	NA
DAZL	NA	NA	NA	NA	NA	1	1	NA	NA
CLEC3B	NA	NA	NA	NA	NA	1	1	NA	NA
RNF123	NA	NA	NA	NA	NA	1	1	NA	NA
TEX264	NA	NA	NA	NA	NA	1	1	NA	NA
SHQ1	NA	NA	NA	NA	NA	1	1	NA	NA
FRG2C	NA	NA	NA	NA	NA	1	1	NA	NA
OR5H1	NA	NA	NA	NA	NA	1	1	NA	NA
OR5H14	NA	NA	NA	NA	NA	1	1	NA	NA
OR5H15	NA	NA	NA	NA	NA	1	1	NA	NA
OR5K3	NA	NA	NA	NA	NA	1	1	NA	NA
OTOP1	NA	NA	NA	NA	NA	1	1	NA	NA
USP17L26	NA	NA	NA	NA	NA	1	1	NA	NA
USP17L29	NA	NA	NA	NA	NA	1	1	NA	NA
FGFBP2	NA	NA	NA	NA	NA	1	1	NA	NA
TAPT1	NA	NA	NA	NA	NA	1	1	NA	NA
UGT2B10	NA	NA	NA	NA	NA	1	1	NA	NA
UGT2B28	NA	NA	NA	NA	NA	1	1	NA	NA
ODAM	NA	NA	NA	NA	NA	1	1	NA	NA
GC	NA	NA	NA	NA	NA	1	1	NA	NA
AREG	NA	NA	NA	NA	NA	1	1	NA	NA
ANTXR2	NA	NA	NA	NA	NA	1	1	NA	NA
BANK1	NA	NA	NA	NA	NA	1	1	NA	NA
ENPEP	0.6923434	0.697920015	-0.00557664	-0.01140984	0.949137806	1	1	0	0
SETD7	NA	NA	NA	NA	NA	1	1	NA	NA
GYPE	NA	NA	NA	NA	NA	1	1	NA	NA
PALLD	NA	NA	NA	NA	NA	1	1	NA	NA
AADAT	NA	NA	NA	NA	NA	1	1	NA	NA
KLKB1	NA	NA	NA	NA	NA	1	1	NA	NA
FRG2	NA	NA	NA	NA	NA	1	1	NA	NA
CDH9	NA	NA	NA	NA	NA	1	1	NA	NA
EMB	NA	NA	NA	NA	NA	1	1	NA	NA
GTF2H2C	NA	NA	NA	NA	NA	1	1	NA	NA
SMN2	NA	NA	NA	NA	NA	1	1	NA	NA
SMN1	NA	NA	NA	NA	NA	1	1	NA	NA
NAIP	NA	NA	NA	NA	NA	1	1	NA	NA
LHFPL2	NA	NA	NA	NA	NA	1	1	NA	NA
FBN2	NA	NA	NA	NA	NA	1	1	NA	NA
RNF14	NA	NA	NA	NA	NA	1	1	NA	NA
SPINK5	NA	NA	NA	NA	NA	1	1	NA	NA

SPINK9	NA	NA	NA	NA	NA	1	1	NA	NA
CCNJL	NA	NA	NA	NA	NA	1	1	NA	NA
SIMC1	NA	NA	NA	NA	NA	1	1	NA	NA
FAM153A	NA	NA	NA	NA	NA	1	1	NA	NA
HNRNPH1	NA	NA	NA	NA	NA	1	1	NA	NA
SSR1	NA	NA	NA	NA	NA	1	1	NA	NA
GCNT2	NA	NA	NA	NA	NA	1	1	NA	NA
RNF144B	NA	NA	NA	NA	NA	1	1	NA	NA
OR2J3	NA	NA	NA	NA	NA	1	1	NA	NA
HLA-A	NA	NA	NA	NA	NA	1	1	NA	NA
HLA-C	NA	NA	NA	NA	NA	1	1	NA	NA
HLA-B	NA	NA	NA	NA	NA	1	1	NA	NA
C4A	NA	NA	NA	NA	NA	1	1	NA	NA
HLA-DRB5	NA	NA	NA	NA	NA	1	1	NA	NA
HLA-DRB1	NA	NA	NA	NA	NA	1	1	NA	NA
HLA-DQA1	NA	NA	NA	NA	NA	1	1	NA	NA
HLA-DQB1	NA	NA	NA	NA	NA	1	1	NA	NA
MAPK13	NA	NA	NA	NA	NA	1	1	NA	NA
DEFB112	NA	NA	NA	NA	NA	1	1	NA	NA
GSTA1	NA	NA	NA	NA	NA	1	1	NA	NA
GSTA5	NA	NA	NA	NA	NA	1	1	NA	NA
OR2A4	NA	NA	NA	NA	NA	1	1	NA	NA
CTAGE9	NA	NA	NA	NA	NA	1	1	NA	NA
WTAP	NA	NA	NA	NA	NA	1	1	NA	NA
PLG	NA	NA	NA	NA	NA	1	1	NA	NA
RPS6KA2	NA	NA	NA	NA	NA	1	1	NA	NA
TCP10	NA	NA	NA	NA	NA	1	1	NA	NA
RBAK-RBAK	NA	NA	NA	NA	NA	1	1	NA	NA
CCZ1	NA	NA	NA	NA	NA	1	1	NA	NA
RSPH10B	NA	NA	NA	NA	NA	1	1	NA	NA
ANKRD61	NA	NA	NA	NA	NA	1	1	NA	NA
RSPH10B2	NA	NA	NA	NA	NA	1	1	NA	NA
C1GALT1	NA	NA	NA	NA	NA	1	1	NA	NA
VPS41	NA	NA	NA	NA	NA	1	1	NA	NA
SPDYE1	NA	NA	NA	NA	NA	1	1	NA	NA
ZNF679	NA	NA	NA	NA	NA	1	1	NA	NA
POM121	NA	NA	NA	NA	NA	1	1	NA	NA
GTF2IRD2	NA	NA	NA	NA	NA	1	1	NA	NA
GTF2IRD2B	NA	NA	NA	NA	NA	1	1	NA	NA
TRIM73	NA	NA	NA	NA	NA	1	1	NA	NA
ABCB1	NA	NA	NA	NA	NA	1	1	NA	NA
CYP51A1	NA	NA	NA	NA	NA	1	1	NA	NA
ERVW-1	NA	NA	NA	NA	NA	1	1	NA	NA
GNGT1	NA	NA	NA	NA	NA	1	1	NA	NA
RASA4B	NA	NA	NA	NA	NA	1	1	NA	NA
POLR2J3	NA	NA	NA	NA	NA	1	1	NA	NA
POLR2J2	NA	NA	NA	NA	NA	1	1	NA	NA
SRPK2	NA	NA	NA	NA	NA	1	1	NA	NA
NAMPT	NA	NA	NA	NA	NA	1	1	NA	NA
PPP1R3A	NA	NA	NA	NA	NA	1	1	NA	NA
MTRNR2L6	NA	NA	NA	NA	NA	1	1	NA	NA
GSTK1	NA	NA	NA	NA	NA	1	1	NA	NA
ARHGEF35	NA	NA	NA	NA	NA	1	1	NA	NA
OR2A1	NA	NA	NA	NA	NA	1	1	NA	NA
ARHGEF5	NA	NA	NA	NA	NA	1	1	NA	NA
DEFA1	NA	NA	NA	NA	NA	1	1	NA	NA
DEFA1B	NA	NA	NA	NA	NA	1	1	NA	NA
DEFA3	NA	NA	NA	NA	NA	1	1	NA	NA
DEFB4B	NA	NA	NA	NA	NA	1	1	NA	NA
DEFB104B	NA	NA	NA	NA	NA	1	1	NA	NA
DEFB106B	NA	NA	NA	NA	NA	1	1	NA	NA
DEFB105B	NA	NA	NA	NA	NA	1	1	NA	NA
DEFB107B	NA	NA	NA	NA	NA	1	1	NA	NA

PRR23D1	NA	NA	NA	NA	NA	1	1	NA	NA
PRR23D2	NA	NA	NA	NA	NA	1	1	NA	NA
DEFB105A	NA	NA	NA	NA	NA	1	1	NA	NA
DEFB106A	NA	NA	NA	NA	NA	1	1	NA	NA
DEFB104A	NA	NA	NA	NA	NA	1	1	NA	NA
DEFB4A	NA	NA	NA	NA	NA	1	1	NA	NA
ZNF705B	NA	NA	NA	NA	NA	1	1	NA	NA
USP17L8	NA	NA	NA	NA	NA	1	1	NA	NA
USP17L3	NA	NA	NA	NA	NA	1	1	NA	NA
SLC35G5	NA	NA	NA	NA	NA	1	1	NA	NA
USP17L2	NA	NA	NA	NA	NA	1	1	NA	NA
FAM86B1	NA	NA	NA	NA	NA	1	1	NA	NA
FAM86B2	NA	NA	NA	NA	NA	1	1	NA	NA
ZDHHC2	0.0646753	0.064724886	-4.95E-05	-0.0009567	0.944189099	1	1	0	0
LOXL2	NA	NA	NA	NA	NA	1	1	NA	NA
UBXN8	NA	NA	NA	NA	NA	1	1	NA	NA
FNTA	NA	NA	NA	NA	NA	1	1	NA	NA
SNX31	NA	NA	NA	NA	NA	1	1	NA	NA
CYP11B1	NA	NA	NA	NA	NA	1	1	NA	NA
PARP10	NA	NA	NA	NA	NA	1	1	NA	NA
MROH1	NA	NA	NA	NA	NA	1	1	NA	NA
FOXD4	NA	NA	NA	NA	NA	1	1	NA	NA
KANK1	0.0444107	0.041705692	0.002704991	0.073749273	0.953937161	1	2	0	0
IFNA4	NA	NA	NA	NA	NA	1	1	NA	NA
IFNA10	NA	NA	NA	NA	NA	1	1	NA	NA
IFNE	NA	NA	NA	NA	NA	1	1	NA	NA
ANKRD20A3	NA	NA	NA	NA	NA	1	1	NA	NA
SPATA31A6	NA	NA	NA	NA	NA	1	1	NA	NA
CNTNAP3B	NA	NA	NA	NA	NA	1	1	NA	NA
ANKRD20A1	NA	NA	NA	NA	NA	1	1	NA	NA
ANKRD20A4	NA	NA	NA	NA	NA	1	1	NA	NA
CBWD3	NA	NA	NA	NA	NA	1	1	NA	NA
GDA	NA	NA	NA	NA	NA	1	1	NA	NA
GCNT1	NA	NA	NA	NA	NA	1	1	NA	NA
NUTM2G	NA	NA	NA	NA	NA	1	1	NA	NA
TXNDC8	NA	NA	NA	NA	NA	1	1	NA	NA
ORM1	NA	NA	NA	NA	NA	1	1	NA	NA
LCN15	NA	NA	NA	NA	NA	1	1	NA	NA
LYZL1	NA	NA	NA	NA	NA	1	1	NA	NA
TIMM23B	NA	NA	NA	NA	NA	1	1	NA	NA
TIMM23	NA	NA	NA	NA	NA	1	1	NA	NA
AGAP6	NA	NA	NA	NA	NA	1	1	NA	NA
SYNPO2L	NA	NA	NA	NA	NA	1	1	NA	NA
AGAP5	NA	NA	NA	NA	NA	1	1	NA	NA
EIF5AL1	NA	NA	NA	NA	NA	1	1	NA	NA
FAM25A	NA	NA	NA	NA	NA	1	1	NA	NA
NUTM2A	NA	NA	NA	NA	NA	1	1	NA	NA
DHX32	NA	NA	NA	NA	NA	1	1	NA	NA
FANK1	NA	NA	NA	NA	NA	1	1	NA	NA
FRG2B	NA	NA	NA	NA	NA	1	1	NA	NA
AP2A2	NA	NA	NA	NA	NA	1	1	NA	NA
OR51A4	NA	NA	NA	NA	NA	1	1	NA	NA
OR52N4	NA	NA	NA	NA	NA	1	1	NA	NA
OR52N5	NA	NA	NA	NA	NA	1	1	NA	NA
OR52L1	NA	NA	NA	NA	NA	1	1	NA	NA
MTRNR2L8	NA	NA	NA	NA	NA	1	1	NA	NA
TRIM48	NA	NA	NA	NA	NA	1	1	NA	NA
OR4P4	NA	NA	NA	NA	NA	1	1	NA	NA
OR4S2	NA	NA	NA	NA	NA	1	1	NA	NA
OR8H2	NA	NA	NA	NA	NA	1	1	NA	NA
OR8H3	NA	NA	NA	NA	NA	1	1	NA	NA
PGA3	NA	NA	NA	NA	NA	1	1	NA	NA
KRTAP5-10	NA	NA	NA	NA	NA	1	1	NA	NA

TRIM49	NA	NA	NA	NA	NA	1	1	NA	NA
UBTFL1	NA	NA	NA	NA	NA	1	1	NA	NA
NAALAD2	NA	NA	NA	NA	NA	1	1	NA	NA
MMP8	NA	NA	NA	NA	NA	1	1	NA	NA
TRIM29	NA	NA	NA	NA	NA	1	1	NA	NA
OR8B3	NA	NA	NA	NA	NA	1	1	NA	NA
OR8A1	NA	NA	NA	NA	NA	1	1	NA	NA
IQSEC3	NA	NA	NA	NA	NA	1	1	NA	NA
CLECL1	NA	NA	NA	NA	NA	1	1	NA	NA
CLEC1A	0.9548879	0.956412212	-0.0015243	-0.00227733	0.964576301	1	1	0	0
TAS2R43	NA	NA	NA	NA	NA	1	1	NA	NA
TAS2R30	NA	NA	NA	NA	NA	1	1	NA	NA
SLCO1A2	NA	NA	NA	NA	NA	1	1	NA	NA
FAM19A2	NA	NA	NA	NA	NA	1	1	NA	NA
CPM	NA	NA	NA	NA	NA	1	1	NA	NA
EPYC	NA	NA	NA	NA	NA	1	1	NA	NA
SLC41A2	0.0270249	0.027032158	-7.28E-06	-0.00028363	0.973561326	1	1	0	0
RFC5	NA	NA	NA	NA	NA	1	1	NA	NA
HCAR2	NA	NA	NA	NA	NA	1	1	NA	NA
UBC	NA	NA	NA	NA	NA	1	1	NA	NA
CHFR	NA	NA	NA	NA	NA	1	1	NA	NA
ZNF605	NA	NA	NA	NA	NA	1	1	NA	NA
ZNF26	NA	NA	NA	NA	NA	1	1	NA	NA
ZNF84	NA	NA	NA	NA	NA	1	1	NA	NA
ZNF140	NA	NA	NA	NA	NA	1	1	NA	NA
TPTE2	NA	NA	NA	NA	NA	1	1	NA	NA
PRR20B	NA	NA	NA	NA	NA	1	1	NA	NA
TEX29	NA	NA	NA	NA	NA	1	1	NA	NA
OR11H12	NA	NA	NA	NA	NA	1	1	NA	NA
POTEG	NA	NA	NA	NA	NA	1	1	NA	NA
POTEM	NA	NA	NA	NA	NA	1	1	NA	NA
OR11H2	NA	NA	NA	NA	NA	1	1	NA	NA
OR4L1	NA	NA	NA	NA	NA	1	1	NA	NA
DHRS4	0.0303407	0.029593783	0.000746955	0.031084584	0.939872121	1	2	0	0
KTN1	NA	NA	NA	NA	NA	1	1	NA	NA
TMEM63C	NA	NA	NA	NA	NA	1	1	NA	NA
GOLGA6L6	NA	NA	NA	NA	NA	1	1	NA	NA
NIPA1	NA	NA	NA	NA	NA	1	1	NA	NA
GOLGA6L2	NA	NA	NA	NA	NA	1	1	NA	NA
GOLGA8M	NA	NA	NA	NA	NA	1	1	NA	NA
GOLGA8H	NA	NA	NA	NA	NA	1	1	NA	NA
ARHGAP11E	NA	NA	NA	NA	NA	1	1	NA	NA
GOLGA8K	NA	NA	NA	NA	NA	1	1	NA	NA
ARHGAP11/	NA	NA	NA	NA	NA	1	1	NA	NA
TMCO5A	NA	NA	NA	NA	NA	1	1	NA	NA
PPIP5K1	NA	NA	NA	NA	NA	1	1	NA	NA
CKMT1B	NA	NA	NA	NA	NA	1	1	NA	NA
STRC	NA	NA	NA	NA	NA	1	1	NA	NA
CKMT1A	NA	NA	NA	NA	NA	1	1	NA	NA
ANXA2	NA	NA	NA	NA	NA	1	1	NA	NA
TLN2	NA	NA	NA	NA	NA	1	1	NA	NA
DAPK2	NA	NA	NA	NA	NA	1	1	NA	NA
ISLR2	NA	NA	NA	NA	NA	1	1	NA	NA
GOLGA6C	NA	NA	NA	NA	NA	1	1	NA	NA
GOLGA6D	NA	NA	NA	NA	NA	1	1	NA	NA
ACSBG1	NA	NA	NA	NA	NA	1	1	NA	NA
CHRNB4	NA	NA	NA	NA	NA	1	1	NA	NA
SYNM	NA	NA	NA	NA	NA	1	1	NA	NA
OR4F4	NA	NA	NA	NA	NA	1	1	NA	NA
NOMO1	NA	NA	NA	NA	NA	1	1	NA	NA
NPIPA5	NA	NA	NA	NA	NA	1	1	NA	NA
NOMO3	NA	NA	NA	NA	NA	1	1	NA	NA
NPIPA8	NA	NA	NA	NA	NA	1	1	NA	NA

ACSM2B	NA	NA	NA	NA	NA	1	1	NA	NA	
GGA2	NA	NA	NA	NA	NA	1	1	NA	NA	
NPIPB8	NA	NA	NA	NA	NA	1	1	NA	NA	
BOLA2	NA	NA	NA	NA	NA	1	1	NA	NA	
SULT1A4	NA	NA	NA	NA	NA	1	1	NA	NA	
BOLA2B	NA	NA	NA	NA	NA	1	1	NA	NA	
SULT1A3	NA	NA	NA	NA	NA	1	1	NA	NA	
KAT8	NA	NA	NA	NA	NA	1	1	NA	NA	
TP53TG3D	NA	NA	NA	NA	NA	1	1	NA	NA	
TP53TG3	NA	NA	NA	NA	NA	1	1	NA	NA	
PDPR	NA	NA	NA	NA	NA	1	1	NA	NA	
NPIPB15	NA	NA	NA	NA	NA	1	1	NA	NA	
ACSF3	0.4291004	0.425998126	0.003102262	0.010228875	0.970169031	1	1	0		0
PRDM7	NA	NA	NA	NA	NA	1	1	NA	NA	
OR1D5	NA	NA	NA	NA	NA	1	1	NA	NA	
C1QBP	0.8967071	0.900265795	-0.00355872	-0.00565133	0.975786929	1	1	0		0
TRIM16	NA	NA	NA	NA	NA	1	1	NA	NA	
LGALS9C	NA	NA	NA	NA	NA	1	1	NA	NA	
TVP23B	NA	NA	NA	NA	NA	1	1	NA	NA	
GRAP	NA	NA	NA	NA	NA	1	1	NA	NA	
GRAPL	NA	NA	NA	NA	NA	1	1	NA	NA	
AP2B1	0.8269622	0.833157541	-0.00619535	-0.01063977	0.973128076	1	1	0		0
CCL3	NA	NA	NA	NA	NA	1	1	NA	NA	
TBC1D3B	NA	NA	NA	NA	NA	1	1	NA	NA	
TBC1D3H	NA	NA	NA	NA	NA	1	1	NA	NA	
CCL4L2	NA	NA	NA	NA	NA	1	1	NA	NA	
TBC1D3G	NA	NA	NA	NA	NA	1	1	NA	NA	
KRTAP3-3	NA	NA	NA	NA	NA	1	1	NA	NA	
KRTAP4-9	NA	NA	NA	NA	NA	1	1	NA	NA	
KRTAP9-8	NA	NA	NA	NA	NA	1	1	NA	NA	
KRTAP9-7	NA	NA	NA	NA	NA	1	1	NA	NA	
ATXN7L3	0.0958534	0.093626979	0.002226434	0.022841765	0.94859419	1	2	0		0
PLEKHM1	NA	NA	NA	NA	NA	1	1	NA	NA	
KANSL1	NA	NA	NA	NA	NA	1	1	NA	NA	
ARL17B	NA	NA	NA	NA	NA	1	1	NA	NA	
LRRC37A	NA	NA	NA	NA	NA	1	1	NA	NA	
TLK2	NA	NA	NA	NA	NA	1	1	NA	NA	
CSH2	NA	NA	NA	NA	NA	1	1	NA	NA	
CSH1	NA	NA	NA	NA	NA	1	1	NA	NA	
LRRC37A3	NA	NA	NA	NA	NA	1	1	NA	NA	
KCNJ16	0.7773014	0.780362955	-0.00306158	-0.00559934	0.977757014	1	1	0		0
CYGB	NA	NA	NA	NA	NA	1	1	NA	NA	
POTEC	NA	NA	NA	NA	NA	1	1	NA	NA	
TCF4	NA	NA	NA	NA	NA	1	1	NA	NA	
ELAVL1	0.0213796	0.020381949	0.000997609	0.04661062	0.951507181	1	1	0		0
NANOS3	NA	NA	NA	NA	NA	1	1	NA	NA	
ZNF429	NA	NA	NA	NA	NA	1	1	NA	NA	
LRFN3	NA	NA	NA	NA	NA	1	1	NA	NA	
HKR1	NA	NA	NA	NA	NA	1	1	NA	NA	
CAPN12	NA	NA	NA	NA	NA	1	1	NA	NA	
IFNL2	NA	NA	NA	NA	NA	1	1	NA	NA	
MIA	NA	NA	NA	NA	NA	1	1	NA	NA	
CYP2A7	NA	NA	NA	NA	NA	1	1	NA	NA	
CEACAM6	NA	NA	NA	NA	NA	1	1	NA	NA	
PSG3	NA	NA	NA	NA	NA	1	1	NA	NA	
PSG6	NA	NA	NA	NA	NA	1	1	NA	NA	
PSG7	NA	NA	NA	NA	NA	1	1	NA	NA	
PSG5	NA	NA	NA	NA	NA	1	1	NA	NA	
PSG4	NA	NA	NA	NA	NA	1	1	NA	NA	
PSG9	NA	NA	NA	NA	NA	1	1	NA	NA	
CGB1	NA	NA	NA	NA	NA	1	1	NA	NA	
MYH14	NA	NA	NA	NA	NA	1	1	NA	NA	
ZNF701	NA	NA	NA	NA	NA	1	1	NA	NA	

LILRB3	NA	NA	NA	NA	NA	1	1	NA	NA
LILRA6	NA	NA	NA	NA	NA	1	1	NA	NA
KIR2DL3	NA	NA	NA	NA	NA	1	1	NA	NA
KIR3DL2	NA	NA	NA	NA	NA	1	1	NA	NA
RFPL4AL1	NA	NA	NA	NA	NA	1	1	NA	NA
ZSCAN5B	NA	NA	NA	NA	NA	1	1	NA	NA
ZNF583	NA	NA	NA	NA	NA	1	1	NA	NA
CST4	NA	NA	NA	NA	NA	1	1	NA	NA
CST2	NA	NA	NA	NA	NA	1	1	NA	NA
GGTLC1	NA	NA	NA	NA	NA	1	1	NA	NA
ZFP64	NA	NA	NA	NA	NA	1	1	NA	NA
TPTE	NA	NA	NA	NA	NA	1	1	NA	NA
BACH1	NA	NA	NA	NA	NA	1	1	NA	NA
IGSF5	NA	NA	NA	NA	NA	1	1	NA	NA
POTEH	NA	NA	NA	NA	NA	1	1	NA	NA
RIMBP3	NA	NA	NA	NA	NA	1	1	NA	NA
TMEM191C	NA	NA	NA	NA	NA	1	1	NA	NA
RIMBP3C	NA	NA	NA	NA	NA	1	1	NA	NA
GGTLC2	NA	NA	NA	NA	NA	1	1	NA	NA
TTC28	NA	NA	NA	NA	NA	1	1	NA	NA
SMTN	NA	NA	NA	NA	NA	1	1	NA	NA
ISX	NA	NA	NA	NA	NA	1	1	NA	NA
IL2RB	NA	NA	NA	NA	NA	1	1	NA	NA
GYG2	NA	NA	NA	NA	NA	1	1	NA	NA
ARSD	NA	NA	NA	NA	NA	1	1	NA	NA
ARSE	NA	NA	NA	NA	NA	1	1	NA	NA
ARSH	NA	NA	NA	NA	NA	1	1	NA	NA
ARSF	NA	NA	NA	NA	NA	1	1	NA	NA
MXRA5	NA	NA	NA	NA	NA	1	1	NA	NA
PRKX	NA	NA	NA	NA	NA	1	1	NA	NA
NLGN4X	NA	NA	NA	NA	NA	1	1	NA	NA
VCX3A	NA	NA	NA	NA	NA	1	1	NA	NA
STS	NA	NA	NA	NA	NA	1	1	NA	NA
VCX	NA	NA	NA	NA	NA	1	1	NA	NA
PNPLA4	NA	NA	NA	NA	NA	1	1	NA	NA
VCX2	NA	NA	NA	NA	NA	1	1	NA	NA
VCX3B	NA	NA	NA	NA	NA	1	1	NA	NA
FAM9A	NA	NA	NA	NA	NA	1	1	NA	NA
TBL1X	NA	NA	NA	NA	NA	1	1	NA	NA
GPR143	NA	NA	NA	NA	NA	1	1	NA	NA
SHROOM2	NA	NA	NA	NA	NA	1	1	NA	NA
WWC3	NA	NA	NA	NA	NA	1	1	NA	NA
CLCN4	NA	NA	NA	NA	NA	1	1	NA	NA
MID1	NA	NA	NA	NA	NA	1	1	NA	NA
HCCS	NA	NA	NA	NA	NA	1	1	NA	NA
ARHGAP6	NA	NA	NA	NA	NA	1	1	NA	NA
AMELX	NA	NA	NA	NA	NA	1	1	NA	NA
MSL3	NA	NA	NA	NA	NA	1	1	NA	NA
FRMPD4	NA	NA	NA	NA	NA	1	1	NA	NA
PRPS2	NA	NA	NA	NA	NA	1	1	NA	NA
TLR7	NA	NA	NA	NA	NA	1	1	NA	NA
TLR8	NA	NA	NA	NA	NA	1	1	NA	NA
TMSB4X	NA	NA	NA	NA	NA	1	1	NA	NA
FAM9C	NA	NA	NA	NA	NA	1	1	NA	NA
ATXN3L	NA	NA	NA	NA	NA	1	1	NA	NA
EGFL6	NA	NA	NA	NA	NA	1	1	NA	NA
TCEANC	NA	NA	NA	NA	NA	1	1	NA	NA
RAB9A	NA	NA	NA	NA	NA	1	1	NA	NA
TRAPPC2	NA	NA	NA	NA	NA	1	1	NA	NA
OFD1	NA	NA	NA	NA	NA	1	1	NA	NA
GPM6B	NA	NA	NA	NA	NA	1	1	NA	NA
GEMIN8	NA	NA	NA	NA	NA	1	1	NA	NA
GLRA2	NA	NA	NA	NA	NA	1	1	NA	NA

FANCB	NA	NA	NA	NA	NA	1	1	NA	NA
MOSPD2	NA	NA	NA	NA	NA	1	1	NA	NA
ASB9	NA	NA	NA	NA	NA	1	1	NA	NA
ASB11	NA	NA	NA	NA	NA	1	1	NA	NA
PIGA	NA	NA	NA	NA	NA	1	1	NA	NA
PIR	NA	NA	NA	NA	NA	1	1	NA	NA
BMX	NA	NA	NA	NA	NA	1	1	NA	NA
ACE2	NA	NA	NA	NA	NA	1	1	NA	NA
CA5B	NA	NA	NA	NA	NA	1	1	NA	NA
ZRSR2	NA	NA	NA	NA	NA	1	1	NA	NA
AP1S2	NA	NA	NA	NA	NA	1	1	NA	NA
GRPR	NA	NA	NA	NA	NA	1	1	NA	NA
MAGEB17	NA	NA	NA	NA	NA	1	1	NA	NA
CTPS2	NA	NA	NA	NA	NA	1	1	NA	NA
S100G	NA	NA	NA	NA	NA	1	1	NA	NA
SYAP1	NA	NA	NA	NA	NA	1	1	NA	NA
TXLNG	NA	NA	NA	NA	NA	1	1	NA	NA
RBBP7	NA	NA	NA	NA	NA	1	1	NA	NA
REPS2	NA	NA	NA	NA	NA	1	1	NA	NA
NHS	NA	NA	NA	NA	NA	1	1	NA	NA
SCML1	NA	NA	NA	NA	NA	1	1	NA	NA
RAI2	NA	NA	NA	NA	NA	1	1	NA	NA
BEND2	NA	NA	NA	NA	NA	1	1	NA	NA
SCML2	NA	NA	NA	NA	NA	1	1	NA	NA
CDKL5	NA	NA	NA	NA	NA	1	1	NA	NA
RS1	NA	NA	NA	NA	NA	1	1	NA	NA
PPEF1	NA	NA	NA	NA	NA	1	1	NA	NA
PHKA2	NA	NA	NA	NA	NA	1	1	NA	NA
PDHA1	NA	NA	NA	NA	NA	1	1	NA	NA
MAP3K15	NA	NA	NA	NA	NA	1	1	NA	NA
SH3KBP1	NA	NA	NA	NA	NA	1	1	NA	NA
MAP7D2	NA	NA	NA	NA	NA	1	1	NA	NA
EIF1AX	NA	NA	NA	NA	NA	1	1	NA	NA
RPS6KA3	NA	NA	NA	NA	NA	1	1	NA	NA
CNKSR2	NA	NA	NA	NA	NA	1	1	NA	NA
KLHL34	NA	NA	NA	NA	NA	1	1	NA	NA
SMPX	NA	NA	NA	NA	NA	1	1	NA	NA
MBTPS2	NA	NA	NA	NA	NA	1	1	NA	NA
YY2	NA	NA	NA	NA	NA	1	1	NA	NA
SMS	NA	NA	NA	NA	NA	1	1	NA	NA
PHEX	NA	NA	NA	NA	NA	1	1	NA	NA
DDX53	NA	NA	NA	NA	NA	1	1	NA	NA
PTCHD1	NA	NA	NA	NA	NA	1	1	NA	NA
ACOT9	NA	NA	NA	NA	NA	1	1	NA	NA
SAT1	NA	NA	NA	NA	NA	1	1	NA	NA
APOO	NA	NA	NA	NA	NA	1	1	NA	NA
CXorf58	NA	NA	NA	NA	NA	1	1	NA	NA
KLHL15	NA	NA	NA	NA	NA	1	1	NA	NA
EIF2S3	NA	NA	NA	NA	NA	1	1	NA	NA
ZFX	NA	NA	NA	NA	NA	1	1	NA	NA
PDK3	NA	NA	NA	NA	NA	1	1	NA	NA
PCYT1B	NA	NA	NA	NA	NA	1	1	NA	NA
POLA1	NA	NA	NA	NA	NA	1	1	NA	NA
ARX	NA	NA	NA	NA	NA	1	1	NA	NA
MAGEB18	NA	NA	NA	NA	NA	1	1	NA	NA
MAGEB6	NA	NA	NA	NA	NA	1	1	NA	NA
MAGEB5	NA	NA	NA	NA	NA	1	1	NA	NA
MAGEB10	NA	NA	NA	NA	NA	1	1	NA	NA
DCAF8L1	NA	NA	NA	NA	NA	1	1	NA	NA
IL1RAPL1	NA	NA	NA	NA	NA	1	1	NA	NA
MAGEB2	NA	NA	NA	NA	NA	1	1	NA	NA
MAGEB3	NA	NA	NA	NA	NA	1	1	NA	NA
MAGEB4	NA	NA	NA	NA	NA	1	1	NA	NA

MAGEB1	NA	NA	NA	NA	NA	1	1	NA	NA
NR0B1	NA	NA	NA	NA	NA	1	1	NA	NA
CXorf21	NA	NA	NA	NA	NA	1	1	NA	NA
GK	NA	NA	NA	NA	NA	1	1	NA	NA
TAB3	NA	NA	NA	NA	NA	1	1	NA	NA
FTHL17	NA	NA	NA	NA	NA	1	1	NA	NA
DMD	NA	NA	NA	NA	NA	1	1	NA	NA
FAM47A	NA	NA	NA	NA	NA	1	1	NA	NA
TMEM47	NA	NA	NA	NA	NA	1	1	NA	NA
FAM47B	NA	NA	NA	NA	NA	1	1	NA	NA
MAGEB16	NA	NA	NA	NA	NA	1	1	NA	NA
FAM47C	NA	NA	NA	NA	NA	1	1	NA	NA
PRRG1	NA	NA	NA	NA	NA	1	1	NA	NA
LANCL3	NA	NA	NA	NA	NA	1	1	NA	NA
XK	NA	NA	NA	NA	NA	1	1	NA	NA
DYNLT3	NA	NA	NA	NA	NA	1	1	NA	NA
SYTL5	NA	NA	NA	NA	NA	1	1	NA	NA
SRPX	NA	NA	NA	NA	NA	1	1	NA	NA
RPGR	NA	NA	NA	NA	NA	1	1	NA	NA
OTC	NA	NA	NA	NA	NA	1	1	NA	NA
TSPAN7	NA	NA	NA	NA	NA	1	1	NA	NA
MID1IP1	NA	NA	NA	NA	NA	1	1	NA	NA
BCOR	NA	NA	NA	NA	NA	1	1	NA	NA
ATP6AP2	NA	NA	NA	NA	NA	1	1	NA	NA
MPC1L	NA	NA	NA	NA	NA	1	1	NA	NA
CXorf38	NA	NA	NA	NA	NA	1	1	NA	NA
MED14	NA	NA	NA	NA	NA	1	1	NA	NA
USP9X	NA	NA	NA	NA	NA	1	1	NA	NA
DDX3X	NA	NA	NA	NA	NA	1	1	NA	NA
NYX	NA	NA	NA	NA	NA	1	1	NA	NA
CASK	NA	NA	NA	NA	NA	1	1	NA	NA
GPR34	NA	NA	NA	NA	NA	1	1	NA	NA
GPR82	NA	NA	NA	NA	NA	1	1	NA	NA
MAOA	NA	NA	NA	NA	NA	1	1	NA	NA
MAOB	NA	NA	NA	NA	NA	1	1	NA	NA
NDP	NA	NA	NA	NA	NA	1	1	NA	NA
EFHC2	NA	NA	NA	NA	NA	1	1	NA	NA
FUNDC1	NA	NA	NA	NA	NA	1	1	NA	NA
DUSP21	NA	NA	NA	NA	NA	1	1	NA	NA
KDM6A	NA	NA	NA	NA	NA	1	1	NA	NA
CXorf36	NA	NA	NA	NA	NA	1	1	NA	NA
KRBOX4	NA	NA	NA	NA	NA	1	1	NA	NA
ZNF674	NA	NA	NA	NA	NA	1	1	NA	NA
CHST7	NA	NA	NA	NA	NA	1	1	NA	NA
SLC9A7	NA	NA	NA	NA	NA	1	1	NA	NA
RP2	NA	NA	NA	NA	NA	1	1	NA	NA
JADE3	NA	NA	NA	NA	NA	1	1	NA	NA
RGN	NA	NA	NA	NA	NA	1	1	NA	NA
NDUFB11	NA	NA	NA	NA	NA	1	1	NA	NA
RBM10	NA	NA	NA	NA	NA	1	1	NA	NA
UBA1	NA	NA	NA	NA	NA	1	1	NA	NA
CDK16	NA	NA	NA	NA	NA	1	1	NA	NA
USP11	NA	NA	NA	NA	NA	1	1	NA	NA
ZNF157	NA	NA	NA	NA	NA	1	1	NA	NA
ZNF41	NA	NA	NA	NA	NA	1	1	NA	NA
ARAF	NA	NA	NA	NA	NA	1	1	NA	NA
SYN1	NA	NA	NA	NA	NA	1	1	NA	NA
TIMP1	NA	NA	NA	NA	NA	1	1	NA	NA
CFP	NA	NA	NA	NA	NA	1	1	NA	NA
ELK1	NA	NA	NA	NA	NA	1	1	NA	NA
UXT	NA	NA	NA	NA	NA	1	1	NA	NA
ZNF81	NA	NA	NA	NA	NA	1	1	NA	NA
ZNF182	NA	NA	NA	NA	NA	1	1	NA	NA

ZNF630	NA	NA	NA	NA	NA	1	1	NA	NA
SPACA5	NA	NA	NA	NA	NA	1	1	NA	NA
SPACA5B	NA	NA	NA	NA	NA	1	1	NA	NA
SSX5	NA	NA	NA	NA	NA	1	1	NA	NA
SSX1	NA	NA	NA	NA	NA	1	1	NA	NA
SSX3	NA	NA	NA	NA	NA	1	1	NA	NA
SSX4	NA	NA	NA	NA	NA	1	1	NA	NA
SLC38A5	NA	NA	NA	NA	NA	1	1	NA	NA
FTSJ1	NA	NA	NA	NA	NA	1	1	NA	NA
PORCN	NA	NA	NA	NA	NA	1	1	NA	NA
EBP	NA	NA	NA	NA	NA	1	1	NA	NA
TBC1D25	NA	NA	NA	NA	NA	1	1	NA	NA
RBM3	NA	NA	NA	NA	NA	1	1	NA	NA
WAS	NA	NA	NA	NA	NA	1	1	NA	NA
SUV39H1	NA	NA	NA	NA	NA	1	1	NA	NA
GLOD5	NA	NA	NA	NA	NA	1	1	NA	NA
GATA1	NA	NA	NA	NA	NA	1	1	NA	NA
HDAC6	NA	NA	NA	NA	NA	1	1	NA	NA
ERAS	NA	NA	NA	NA	NA	1	1	NA	NA
PCSK1N	NA	NA	NA	NA	NA	1	1	NA	NA
TIMM17B	NA	NA	NA	NA	NA	1	1	NA	NA
PQBP1	NA	NA	NA	NA	NA	1	1	NA	NA
SLC35A2	NA	NA	NA	NA	NA	1	1	NA	NA
PIM2	NA	NA	NA	NA	NA	1	1	NA	NA
OTUD5	NA	NA	NA	NA	NA	1	1	NA	NA
KCND1	NA	NA	NA	NA	NA	1	1	NA	NA
GRIPAP1	NA	NA	NA	NA	NA	1	1	NA	NA
TFE3	NA	NA	NA	NA	NA	1	1	NA	NA
CCDC120	NA	NA	NA	NA	NA	1	1	NA	NA
PRAF2	NA	NA	NA	NA	NA	1	1	NA	NA
WDR45	NA	NA	NA	NA	NA	1	1	NA	NA
GPKOW	NA	NA	NA	NA	NA	1	1	NA	NA
MAGIX	NA	NA	NA	NA	NA	1	1	NA	NA
PLP2	NA	NA	NA	NA	NA	1	1	NA	NA
PRICKLE3	NA	NA	NA	NA	NA	1	1	NA	NA
SYP	NA	NA	NA	NA	NA	1	1	NA	NA
CACNA1F	NA	NA	NA	NA	NA	1	1	NA	NA
CCDC22	NA	NA	NA	NA	NA	1	1	NA	NA
FOXP3	NA	NA	NA	NA	NA	1	1	NA	NA
PPP1R3F	NA	NA	NA	NA	NA	1	1	NA	NA
GAGE10	NA	NA	NA	NA	NA	1	1	NA	NA
GAGE12J	NA	NA	NA	NA	NA	1	1	NA	NA
GAGE12C	NA	NA	NA	NA	NA	1	1	NA	NA
GAGE12G	NA	NA	NA	NA	NA	1	1	NA	NA
PAGE1	NA	NA	NA	NA	NA	1	1	NA	NA
PAGE4	NA	NA	NA	NA	NA	1	1	NA	NA
USP27X	NA	NA	NA	NA	NA	1	1	NA	NA
CLCN5	NA	NA	NA	NA	NA	1	1	NA	NA
AKAP4	NA	NA	NA	NA	NA	1	1	NA	NA
CCNB3	NA	NA	NA	NA	NA	1	1	NA	NA
DGKK	NA	NA	NA	NA	NA	1	1	NA	NA
SHROOM4	NA	NA	NA	NA	NA	1	1	NA	NA
BMP15	NA	NA	NA	NA	NA	1	1	NA	NA
NUDT10	NA	NA	NA	NA	NA	1	1	NA	NA
CXorf67	NA	NA	NA	NA	NA	1	1	NA	NA
NUDT11	NA	NA	NA	NA	NA	1	1	NA	NA
GSPT2	NA	NA	NA	NA	NA	1	1	NA	NA
MAGED1	NA	NA	NA	NA	NA	1	1	NA	NA
SSX7	NA	NA	NA	NA	NA	1	1	NA	NA
SPANXN5	NA	NA	NA	NA	NA	1	1	NA	NA
XAGE5	NA	NA	NA	NA	NA	1	1	NA	NA
XAGE3	NA	NA	NA	NA	NA	1	1	NA	NA
FAM156A	NA	NA	NA	NA	NA	1	1	NA	NA

GPR173	NA	NA	NA	NA	NA	1	1	NA	NA
TSPYL2	NA	NA	NA	NA	NA	1	1	NA	NA
KDM5C	NA	NA	NA	NA	NA	1	1	NA	NA
IQSEC2	NA	NA	NA	NA	NA	1	1	NA	NA
SMC1A	NA	NA	NA	NA	NA	1	1	NA	NA
RIBC1	NA	NA	NA	NA	NA	1	1	NA	NA
HSD17B10	NA	NA	NA	NA	NA	1	1	NA	NA
HUWE1	NA	NA	NA	NA	NA	1	1	NA	NA
PHF8	NA	NA	NA	NA	NA	1	1	NA	NA
FAM120C	NA	NA	NA	NA	NA	1	1	NA	NA
WNK3	NA	NA	NA	NA	NA	1	1	NA	NA
TSR2	NA	NA	NA	NA	NA	1	1	NA	NA
FGD1	NA	NA	NA	NA	NA	1	1	NA	NA
GNL3L	NA	NA	NA	NA	NA	1	1	NA	NA
ITIH6	NA	NA	NA	NA	NA	1	1	NA	NA
MAGED2	NA	NA	NA	NA	NA	1	1	NA	NA
TRO	NA	NA	NA	NA	NA	1	1	NA	NA
PFKFB1	NA	NA	NA	NA	NA	1	1	NA	NA
APEX2	NA	NA	NA	NA	NA	1	1	NA	NA
ALAS2	NA	NA	NA	NA	NA	1	1	NA	NA
PAGE2B	NA	NA	NA	NA	NA	1	1	NA	NA
PAGE2	NA	NA	NA	NA	NA	1	1	NA	NA
FAM104B	NA	NA	NA	NA	NA	1	1	NA	NA
MTRNR2L1(	NA	NA	NA	NA	NA	1	1	NA	NA
PAGE5	NA	NA	NA	NA	NA	1	1	NA	NA
PAGE3	NA	NA	NA	NA	NA	1	1	NA	NA
MAGEH1	NA	NA	NA	NA	NA	1	1	NA	NA
USP51	NA	NA	NA	NA	NA	1	1	NA	NA
FOXR2	NA	NA	NA	NA	NA	1	1	NA	NA
RRAGB	NA	NA	NA	NA	NA	1	1	NA	NA
KLF8	NA	NA	NA	NA	NA	1	1	NA	NA
UBQLN2	NA	NA	NA	NA	NA	1	1	NA	NA
SPIN3	NA	NA	NA	NA	NA	1	1	NA	NA
SPIN2B	NA	NA	NA	NA	NA	1	1	NA	NA
SPIN2A	NA	NA	NA	NA	NA	1	1	NA	NA
FAAH2	NA	NA	NA	NA	NA	1	1	NA	NA
ZXDB	NA	NA	NA	NA	NA	1	1	NA	NA
ZXDA	NA	NA	NA	NA	NA	1	1	NA	NA
SPIN4	NA	NA	NA	NA	NA	1	1	NA	NA
ARHGEF9	NA	NA	NA	NA	NA	1	1	NA	NA
AMER1	NA	NA	NA	NA	NA	1	1	NA	NA
MTMR8	NA	NA	NA	NA	NA	1	1	NA	NA
ZC4H2	NA	NA	NA	NA	NA	1	1	NA	NA
ZC3H12B	NA	NA	NA	NA	NA	1	1	NA	NA
LAS1L	NA	NA	NA	NA	NA	1	1	NA	NA
MSN	NA	NA	NA	NA	NA	1	1	NA	NA
VSIG4	NA	NA	NA	NA	NA	1	1	NA	NA
HEPH	NA	NA	NA	NA	NA	1	1	NA	NA
EDA2R	NA	NA	NA	NA	NA	1	1	NA	NA
AR	NA	NA	NA	NA	NA	1	1	NA	NA
OPHN1	NA	NA	NA	NA	NA	1	1	NA	NA
YIPF6	NA	NA	NA	NA	NA	1	1	NA	NA
STARD8	NA	NA	NA	NA	NA	1	1	NA	NA
EFNB1	NA	NA	NA	NA	NA	1	1	NA	NA
PJA1	NA	NA	NA	NA	NA	1	1	NA	NA
FAM155B	NA	NA	NA	NA	NA	1	1	NA	NA
EDA	NA	NA	NA	NA	NA	1	1	NA	NA
AWAT2	NA	NA	NA	NA	NA	1	1	NA	NA
OTUD6A	NA	NA	NA	NA	NA	1	1	NA	NA
IGBP1	NA	NA	NA	NA	NA	1	1	NA	NA
DGAT2L6	NA	NA	NA	NA	NA	1	1	NA	NA
AWAT1	NA	NA	NA	NA	NA	1	1	NA	NA
P2RY4	NA	NA	NA	NA	NA	1	1	NA	NA

ARR3	NA	NA	NA	NA	NA	1	1	NA	NA
RAB41	NA	NA	NA	NA	NA	1	1	NA	NA
PDZD11	NA	NA	NA	NA	NA	1	1	NA	NA
KIF4A	NA	NA	NA	NA	NA	1	1	NA	NA
GDPD2	NA	NA	NA	NA	NA	1	1	NA	NA
DLG3	NA	NA	NA	NA	NA	1	1	NA	NA
TEX11	NA	NA	NA	NA	NA	1	1	NA	NA
SLC7A3	NA	NA	NA	NA	NA	1	1	NA	NA
SNX12	NA	NA	NA	NA	NA	1	1	NA	NA
FOXO4	NA	NA	NA	NA	NA	1	1	NA	NA
CXorf65	NA	NA	NA	NA	NA	1	1	NA	NA
IL2RG	NA	NA	NA	NA	NA	1	1	NA	NA
MED12	NA	NA	NA	NA	NA	1	1	NA	NA
NLGN3	NA	NA	NA	NA	NA	1	1	NA	NA
GJB1	NA	NA	NA	NA	NA	1	1	NA	NA
ZMYM3	NA	NA	NA	NA	NA	1	1	NA	NA
NONO	NA	NA	NA	NA	NA	1	1	NA	NA
ITGB1BP2	NA	NA	NA	NA	NA	1	1	NA	NA
TAF1	NA	NA	NA	NA	NA	1	1	NA	NA
OGT	NA	NA	NA	NA	NA	1	1	NA	NA
CXCR3	NA	NA	NA	NA	NA	1	1	NA	NA
PIN4	NA	NA	NA	NA	NA	1	1	NA	NA
ERCC6L	NA	NA	NA	NA	NA	1	1	NA	NA
RPS4X	NA	NA	NA	NA	NA	1	1	NA	NA
CITED1	NA	NA	NA	NA	NA	1	1	NA	NA
HDAC8	NA	NA	NA	NA	NA	1	1	NA	NA
PHKA1	NA	NA	NA	NA	NA	1	1	NA	NA
PABPC1L2B	NA	NA	NA	NA	NA	1	1	NA	NA
PABPC1L2A	NA	NA	NA	NA	NA	1	1	NA	NA
NAP1L2	NA	NA	NA	NA	NA	1	1	NA	NA
CDX4	NA	NA	NA	NA	NA	1	1	NA	NA
CHIC1	NA	NA	NA	NA	NA	1	1	NA	NA
ZCCHC13	NA	NA	NA	NA	NA	1	1	NA	NA
SLC16A2	NA	NA	NA	NA	NA	1	1	NA	NA
RLIM	NA	NA	NA	NA	NA	1	1	NA	NA
ABCB7	NA	NA	NA	NA	NA	1	1	NA	NA
UPRT	NA	NA	NA	NA	NA	1	1	NA	NA
ZDHHC15	NA	NA	NA	NA	NA	1	1	NA	NA
MAGEE2	NA	NA	NA	NA	NA	1	1	NA	NA
PBDC1	NA	NA	NA	NA	NA	1	1	NA	NA
MAGEE1	NA	NA	NA	NA	NA	1	1	NA	NA
FGF16	NA	NA	NA	NA	NA	1	1	NA	NA
ATRX	NA	NA	NA	NA	NA	1	1	NA	NA
MAGT1	NA	NA	NA	NA	NA	1	1	NA	NA
COX7B	NA	NA	NA	NA	NA	1	1	NA	NA
ATP7A	NA	NA	NA	NA	NA	1	1	NA	NA
PGAM4	NA	NA	NA	NA	NA	1	1	NA	NA
PGK1	NA	NA	NA	NA	NA	1	1	NA	NA
TAF9B	NA	NA	NA	NA	NA	1	1	NA	NA
CYSLTR1	NA	NA	NA	NA	NA	1	1	NA	NA
LPAR4	NA	NA	NA	NA	NA	1	1	NA	NA
P2RY10	NA	NA	NA	NA	NA	1	1	NA	NA
GPR174	NA	NA	NA	NA	NA	1	1	NA	NA
ITM2A	NA	NA	NA	NA	NA	1	1	NA	NA
TBX22	NA	NA	NA	NA	NA	1	1	NA	NA
BRWD3	NA	NA	NA	NA	NA	1	1	NA	NA
HMGN5	NA	NA	NA	NA	NA	1	1	NA	NA
SH3BGRL	NA	NA	NA	NA	NA	1	1	NA	NA
POU3F4	NA	NA	NA	NA	NA	1	1	NA	NA
CYLC1	NA	NA	NA	NA	NA	1	1	NA	NA
RPS6KA6	NA	NA	NA	NA	NA	1	1	NA	NA
HDX	NA	NA	NA	NA	NA	1	1	NA	NA
APOOL	NA	NA	NA	NA	NA	1	1	NA	NA

SATL1	NA	NA	NA	NA	NA	1	1	NA	NA
ZNF711	NA	NA	NA	NA	NA	1	1	NA	NA
POF1B	NA	NA	NA	NA	NA	1	1	NA	NA
CHM	NA	NA	NA	NA	NA	1	1	NA	NA
DACH2	NA	NA	NA	NA	NA	1	1	NA	NA
KLHL4	NA	NA	NA	NA	NA	1	1	NA	NA
CPXCR1	NA	NA	NA	NA	NA	1	1	NA	NA
TGIF2LX	NA	NA	NA	NA	NA	1	1	NA	NA
PABPC5	NA	NA	NA	NA	NA	1	1	NA	NA
PCDH11X	NA	NA	NA	NA	NA	1	1	NA	NA
NAP1L3	NA	NA	NA	NA	NA	1	1	NA	NA
FAM133A	NA	NA	NA	NA	NA	1	1	NA	NA
DIAPH2	NA	NA	NA	NA	NA	1	1	NA	NA
RPA4	NA	NA	NA	NA	NA	1	1	NA	NA
PCDH19	NA	NA	NA	NA	NA	1	1	NA	NA
TNMD	NA	NA	NA	NA	NA	1	1	NA	NA
SRPX2	NA	NA	NA	NA	NA	1	1	NA	NA
SYTL4	NA	NA	NA	NA	NA	1	1	NA	NA
CSTF2	NA	NA	NA	NA	NA	1	1	NA	NA
NOX1	NA	NA	NA	NA	NA	1	1	NA	NA
XKRX	NA	NA	NA	NA	NA	1	1	NA	NA
ARL13A	NA	NA	NA	NA	NA	1	1	NA	NA
TRMT2B	NA	NA	NA	NA	NA	1	1	NA	NA
CENPI	NA	NA	NA	NA	NA	1	1	NA	NA
DRP2	NA	NA	NA	NA	NA	1	1	NA	NA
TAF7L	NA	NA	NA	NA	NA	1	1	NA	NA
TIMM8A	NA	NA	NA	NA	NA	1	1	NA	NA
BTK	NA	NA	NA	NA	NA	1	1	NA	NA
RPL36A	NA	NA	NA	NA	NA	1	1	NA	NA
GLA	NA	NA	NA	NA	NA	1	1	NA	NA
HNRNPH2	NA	NA	NA	NA	NA	1	1	NA	NA
ARMCX4	NA	NA	NA	NA	NA	1	1	NA	NA
ARMCX1	NA	NA	NA	NA	NA	1	1	NA	NA
ARMCX6	NA	NA	NA	NA	NA	1	1	NA	NA
ARMCX3	NA	NA	NA	NA	NA	1	1	NA	NA
ARMCX2	NA	NA	NA	NA	NA	1	1	NA	NA
NXF5	NA	NA	NA	NA	NA	1	1	NA	NA
ZMAT1	NA	NA	NA	NA	NA	1	1	NA	NA
TCEAL2	NA	NA	NA	NA	NA	1	1	NA	NA
TCEAL6	NA	NA	NA	NA	NA	1	1	NA	NA
BEX5	NA	NA	NA	NA	NA	1	1	NA	NA
TMSB15A	NA	NA	NA	NA	NA	1	1	NA	NA
ARMCX5	NA	NA	NA	NA	NA	1	1	NA	NA
GPRASP1	NA	NA	NA	NA	NA	1	1	NA	NA
GPRASP2	NA	NA	NA	NA	NA	1	1	NA	NA
BHLHB9	NA	NA	NA	NA	NA	1	1	NA	NA
RAB40AL	NA	NA	NA	NA	NA	1	1	NA	NA
BEX1	NA	NA	NA	NA	NA	1	1	NA	NA
NXF3	NA	NA	NA	NA	NA	1	1	NA	NA
BEX4	NA	NA	NA	NA	NA	1	1	NA	NA
TCEAL8	NA	NA	NA	NA	NA	1	1	NA	NA
TCEAL5	NA	NA	NA	NA	NA	1	1	NA	NA
BEX2	NA	NA	NA	NA	NA	1	1	NA	NA
TCEAL7	NA	NA	NA	NA	NA	1	1	NA	NA
RAB40A	NA	NA	NA	NA	NA	1	1	NA	NA
TCEAL4	NA	NA	NA	NA	NA	1	1	NA	NA
TCEAL3	NA	NA	NA	NA	NA	1	1	NA	NA
TCEAL1	NA	NA	NA	NA	NA	1	1	NA	NA
MORF4L2	NA	NA	NA	NA	NA	1	1	NA	NA
GLRA4	NA	NA	NA	NA	NA	1	1	NA	NA
TMEM31	NA	NA	NA	NA	NA	1	1	NA	NA
PLP1	NA	NA	NA	NA	NA	1	1	NA	NA
RAB9B	NA	NA	NA	NA	NA	1	1	NA	NA

TMSB15B	NA	NA	NA	NA	NA	1	1	NA	NA
H2BFWT	NA	NA	NA	NA	NA	1	1	NA	NA
H2BFM	NA	NA	NA	NA	NA	1	1	NA	NA
SLC25A53	NA	NA	NA	NA	NA	1	1	NA	NA
ZCCHC18	NA	NA	NA	NA	NA	1	1	NA	NA
FAM199X	NA	NA	NA	NA	NA	1	1	NA	NA
ESX1	NA	NA	NA	NA	NA	1	1	NA	NA
IL1RAPL2	NA	NA	NA	NA	NA	1	1	NA	NA
TEX13A	NA	NA	NA	NA	NA	1	1	NA	NA
NRK	NA	NA	NA	NA	NA	1	1	NA	NA
SERPINA7	NA	NA	NA	NA	NA	1	1	NA	NA
MUM1L1	NA	NA	NA	NA	NA	1	1	NA	NA
CXorf57	NA	NA	NA	NA	NA	1	1	NA	NA
RNF128	NA	NA	NA	NA	NA	1	1	NA	NA
TBC1D8B	NA	NA	NA	NA	NA	1	1	NA	NA
MORC4	NA	NA	NA	NA	NA	1	1	NA	NA
RIPPLY1	NA	NA	NA	NA	NA	1	1	NA	NA
CLDN2	NA	NA	NA	NA	NA	1	1	NA	NA
RBM41	NA	NA	NA	NA	NA	1	1	NA	NA
NUP62CL	NA	NA	NA	NA	NA	1	1	NA	NA
PIH1D3	NA	NA	NA	NA	NA	1	1	NA	NA
FRMPD3	NA	NA	NA	NA	NA	1	1	NA	NA
PRPS1	NA	NA	NA	NA	NA	1	1	NA	NA
TSC22D3	NA	NA	NA	NA	NA	1	1	NA	NA
MID2	NA	NA	NA	NA	NA	1	1	NA	NA
TEX13B	NA	NA	NA	NA	NA	1	1	NA	NA
VSIG1	NA	NA	NA	NA	NA	1	1	NA	NA
PSMD10	NA	NA	NA	NA	NA	1	1	NA	NA
ATG4A	NA	NA	NA	NA	NA	1	1	NA	NA
COL4A6	NA	NA	NA	NA	NA	1	1	NA	NA
COL4A5	NA	NA	NA	NA	NA	1	1	NA	NA
IRS4	NA	NA	NA	NA	NA	1	1	NA	NA
GUCY2F	NA	NA	NA	NA	NA	1	1	NA	NA
NXT2	NA	NA	NA	NA	NA	1	1	NA	NA
ACSL4	NA	NA	NA	NA	NA	1	1	NA	NA
TMEM164	NA	NA	NA	NA	NA	1	1	NA	NA
AMMECR1	NA	NA	NA	NA	NA	1	1	NA	NA
CHRDL1	NA	NA	NA	NA	NA	1	1	NA	NA
PAK3	NA	NA	NA	NA	NA	1	1	NA	NA
CAPN6	NA	NA	NA	NA	NA	1	1	NA	NA
DCX	NA	NA	NA	NA	NA	1	1	NA	NA
TRPC5	NA	NA	NA	NA	NA	1	1	NA	NA
TRPC5OS	NA	NA	NA	NA	NA	1	1	NA	NA
LHFPL1	NA	NA	NA	NA	NA	1	1	NA	NA
AMOT	NA	NA	NA	NA	NA	1	1	NA	NA
HTR2C	NA	NA	NA	NA	NA	1	1	NA	NA
IL13RA2	NA	NA	NA	NA	NA	1	1	NA	NA
LRCH2	NA	NA	NA	NA	NA	1	1	NA	NA
RBMXL3	NA	NA	NA	NA	NA	1	1	NA	NA
LUZP4	NA	NA	NA	NA	NA	1	1	NA	NA
PLS3	NA	NA	NA	NA	NA	1	1	NA	NA
AGTR2	NA	NA	NA	NA	NA	1	1	NA	NA
SLC6A14	NA	NA	NA	NA	NA	1	1	NA	NA
CT83	NA	NA	NA	NA	NA	1	1	NA	NA
KLHL13	NA	NA	NA	NA	NA	1	1	NA	NA
WDR44	NA	NA	NA	NA	NA	1	1	NA	NA
DOCK11	NA	NA	NA	NA	NA	1	1	NA	NA
IL13RA1	NA	NA	NA	NA	NA	1	1	NA	NA
ZCCHC12	NA	NA	NA	NA	NA	1	1	NA	NA
LONRF3	NA	NA	NA	NA	NA	1	1	NA	NA
KIAA1210	NA	NA	NA	NA	NA	1	1	NA	NA
PGRMC1	NA	NA	NA	NA	NA	1	1	NA	NA
SLC25A43	NA	NA	NA	NA	NA	1	1	NA	NA

SLC25A5	NA	NA	NA	NA	NA	1	1	NA	NA
CXorf56	NA	NA	NA	NA	NA	1	1	NA	NA
UBE2A	NA	NA	NA	NA	NA	1	1	NA	NA
NKRF	NA	NA	NA	NA	NA	1	1	NA	NA
06-Sep	NA	NA	NA	NA	NA	1	1	NA	NA
SOWAHD	NA	NA	NA	NA	NA	1	1	NA	NA
RPL39	NA	NA	NA	NA	NA	1	1	NA	NA
UPF3B	NA	NA	NA	NA	NA	1	1	NA	NA
RNF113A	NA	NA	NA	NA	NA	1	1	NA	NA
NDUFA1	NA	NA	NA	NA	NA	1	1	NA	NA
AKAP14	NA	NA	NA	NA	NA	1	1	NA	NA
NKAP	NA	NA	NA	NA	NA	1	1	NA	NA
RHOXF1	NA	NA	NA	NA	NA	1	1	NA	NA
ZBTB33	NA	NA	NA	NA	NA	1	1	NA	NA
TMEM255A	NA	NA	NA	NA	NA	1	1	NA	NA
ATP1B4	NA	NA	NA	NA	NA	1	1	NA	NA
LAMP2	NA	NA	NA	NA	NA	1	1	NA	NA
CUL4B	NA	NA	NA	NA	NA	1	1	NA	NA
MCTS1	NA	NA	NA	NA	NA	1	1	NA	NA
C1GALT1C1	NA	NA	NA	NA	NA	1	1	NA	NA
CT47A1	NA	NA	NA	NA	NA	1	1	NA	NA
GLUD2	NA	NA	NA	NA	NA	1	1	NA	NA
GRIA3	NA	NA	NA	NA	NA	1	1	NA	NA
THOC2	NA	NA	NA	NA	NA	1	1	NA	NA
XIAP	NA	NA	NA	NA	NA	1	1	NA	NA
STAG2	NA	NA	NA	NA	NA	1	1	NA	NA
SH2D1A	NA	NA	NA	NA	NA	1	1	NA	NA
TENM1	NA	NA	NA	NA	NA	1	1	NA	NA
DCAF12L2	NA	NA	NA	NA	NA	1	1	NA	NA
DCAF12L1	NA	NA	NA	NA	NA	1	1	NA	NA
ACTRT1	NA	NA	NA	NA	NA	1	1	NA	NA
SMARCA1	NA	NA	NA	NA	NA	1	1	NA	NA
OCRL	NA	NA	NA	NA	NA	1	1	NA	NA
APLN	NA	NA	NA	NA	NA	1	1	NA	NA
XPNPEP2	NA	NA	NA	NA	NA	1	1	NA	NA
SASH3	NA	NA	NA	NA	NA	1	1	NA	NA
ZDHHC9	NA	NA	NA	NA	NA	1	1	NA	NA
UTP14A	NA	NA	NA	NA	NA	1	1	NA	NA
BCORL1	NA	NA	NA	NA	NA	1	1	NA	NA
ELF4	NA	NA	NA	NA	NA	1	1	NA	NA
AIFM1	NA	NA	NA	NA	NA	1	1	NA	NA
RAB33A	NA	NA	NA	NA	NA	1	1	NA	NA
ZNF280C	NA	NA	NA	NA	NA	1	1	NA	NA
SLC25A14	NA	NA	NA	NA	NA	1	1	NA	NA
GPR119	NA	NA	NA	NA	NA	1	1	NA	NA
RBMX2	NA	NA	NA	NA	NA	1	1	NA	NA
ENOX2	NA	NA	NA	NA	NA	1	1	NA	NA
ARHGAP36	NA	NA	NA	NA	NA	1	1	NA	NA
IGSF1	NA	NA	NA	NA	NA	1	1	NA	NA
OR13H1	NA	NA	NA	NA	NA	1	1	NA	NA
FRMD7	NA	NA	NA	NA	NA	1	1	NA	NA
RAP2C	NA	NA	NA	NA	NA	1	1	NA	NA
MBNL3	NA	NA	NA	NA	NA	1	1	NA	NA
HS6ST2	NA	NA	NA	NA	NA	1	1	NA	NA
USP26	NA	NA	NA	NA	NA	1	1	NA	NA
TFDP3	NA	NA	NA	NA	NA	1	1	NA	NA
GPC4	NA	NA	NA	NA	NA	1	1	NA	NA
GPC3	NA	NA	NA	NA	NA	1	1	NA	NA
CCDC160	NA	NA	NA	NA	NA	1	1	NA	NA
PHF6	NA	NA	NA	NA	NA	1	1	NA	NA
HPRT1	NA	NA	NA	NA	NA	1	1	NA	NA
PLAC1	NA	NA	NA	NA	NA	1	1	NA	NA
FAM122B	NA	NA	NA	NA	NA	1	1	NA	NA

FAM122C	NA	NA	NA	NA	NA	1	1	NA	NA
MOSPD1	NA	NA	NA	NA	NA	1	1	NA	NA
SMIM10	NA	NA	NA	NA	NA	1	1	NA	NA
CT55	NA	NA	NA	NA	NA	1	1	NA	NA
ZNF75D	NA	NA	NA	NA	NA	1	1	NA	NA
ZNF449	NA	NA	NA	NA	NA	1	1	NA	NA
CT45A1	NA	NA	NA	NA	NA	1	1	NA	NA
CT45A6	NA	NA	NA	NA	NA	1	1	NA	NA
SAGE1	NA	NA	NA	NA	NA	1	1	NA	NA
MMGT1	NA	NA	NA	NA	NA	1	1	NA	NA
SLC9A6	NA	NA	NA	NA	NA	1	1	NA	NA
FHL1	NA	NA	NA	NA	NA	1	1	NA	NA
MAP7D3	NA	NA	NA	NA	NA	1	1	NA	NA
BRS3	NA	NA	NA	NA	NA	1	1	NA	NA
HTATSF1	NA	NA	NA	NA	NA	1	1	NA	NA
VGLL1	NA	NA	NA	NA	NA	1	1	NA	NA
CD40LG	NA	NA	NA	NA	NA	1	1	NA	NA
ARHGEF6	NA	NA	NA	NA	NA	1	1	NA	NA
RBMX	NA	NA	NA	NA	NA	1	1	NA	NA
GPR101	NA	NA	NA	NA	NA	1	1	NA	NA
ZIC3	NA	NA	NA	NA	NA	1	1	NA	NA
FGF13	NA	NA	NA	NA	NA	1	1	NA	NA
F9	NA	NA	NA	NA	NA	1	1	NA	NA
MCF2	NA	NA	NA	NA	NA	1	1	NA	NA
SOX3	NA	NA	NA	NA	NA	1	1	NA	NA
CDR1	NA	NA	NA	NA	NA	1	1	NA	NA
LDOC1	NA	NA	NA	NA	NA	1	1	NA	NA
SPANXC	NA	NA	NA	NA	NA	1	1	NA	NA
SPANXA1	NA	NA	NA	NA	NA	1	1	NA	NA
SPANXD	NA	NA	NA	NA	NA	1	1	NA	NA
MAGEC3	NA	NA	NA	NA	NA	1	1	NA	NA
MAGEC1	NA	NA	NA	NA	NA	1	1	NA	NA
MAGEC2	NA	NA	NA	NA	NA	1	1	NA	NA
SPANXN4	NA	NA	NA	NA	NA	1	1	NA	NA
SLITRK4	NA	NA	NA	NA	NA	1	1	NA	NA
SPANXN1	NA	NA	NA	NA	NA	1	1	NA	NA
SLITRK2	NA	NA	NA	NA	NA	1	1	NA	NA
FMR1	NA	NA	NA	NA	NA	1	1	NA	NA
FMR1NB	NA	NA	NA	NA	NA	1	1	NA	NA
AFF2	NA	NA	NA	NA	NA	1	1	NA	NA
CXorf40A	NA	NA	NA	NA	NA	1	1	NA	NA
TMEM185A	NA	NA	NA	NA	NA	1	1	NA	NA
HSFX1	NA	NA	NA	NA	NA	1	1	NA	NA
MAGEA8	NA	NA	NA	NA	NA	1	1	NA	NA
CXorf40B	NA	NA	NA	NA	NA	1	1	NA	NA
MAMLD1	NA	NA	NA	NA	NA	1	1	NA	NA
MTM1	NA	NA	NA	NA	NA	1	1	NA	NA
MTMR1	NA	NA	NA	NA	NA	1	1	NA	NA
CD99L2	NA	NA	NA	NA	NA	1	1	NA	NA
HMGB3	NA	NA	NA	NA	NA	1	1	NA	NA
GPR50	NA	NA	NA	NA	NA	1	1	NA	NA
VMA21	NA	NA	NA	NA	NA	1	1	NA	NA
PASD1	NA	NA	NA	NA	NA	1	1	NA	NA
PRRG3	NA	NA	NA	NA	NA	1	1	NA	NA
FATE1	NA	NA	NA	NA	NA	1	1	NA	NA
CNGA2	NA	NA	NA	NA	NA	1	1	NA	NA
MAGEA4	NA	NA	NA	NA	NA	1	1	NA	NA
GABRE	NA	NA	NA	NA	NA	1	1	NA	NA
MAGEA10	NA	NA	NA	NA	NA	1	1	NA	NA
GABRA3	NA	NA	NA	NA	NA	1	1	NA	NA
GABRQ	NA	NA	NA	NA	NA	1	1	NA	NA
MAGEA2B	NA	NA	NA	NA	NA	1	1	NA	NA
MAGEA12	NA	NA	NA	NA	NA	1	1	NA	NA

CSAG1	NA	NA	NA	NA	NA	1	1	NA	NA
MAGEA3	NA	NA	NA	NA	NA	1	1	NA	NA
CETN2	NA	NA	NA	NA	NA	1	1	NA	NA
NSDHL	NA	NA	NA	NA	NA	1	1	NA	NA
ZNF185	NA	NA	NA	NA	NA	1	1	NA	NA
PNMA5	NA	NA	NA	NA	NA	1	1	NA	NA
PNMA3	NA	NA	NA	NA	NA	1	1	NA	NA
MAGEA1	NA	NA	NA	NA	NA	1	1	NA	NA
ZNF275	NA	NA	NA	NA	NA	1	1	NA	NA
ZFP92	NA	NA	NA	NA	NA	1	1	NA	NA
TREX2	NA	NA	NA	NA	NA	1	1	NA	NA
HAUS7	NA	NA	NA	NA	NA	1	1	NA	NA
BGN	NA	NA	NA	NA	NA	1	1	NA	NA
ATP2B3	NA	NA	NA	NA	NA	1	1	NA	NA
DUSP9	NA	NA	NA	NA	NA	1	1	NA	NA
PNCK	NA	NA	NA	NA	NA	1	1	NA	NA
SLC6A8	NA	NA	NA	NA	NA	1	1	NA	NA
BCAP31	NA	NA	NA	NA	NA	1	1	NA	NA
ABCD1	NA	NA	NA	NA	NA	1	1	NA	NA
PLXNB3	NA	NA	NA	NA	NA	1	1	NA	NA
SRPK3	NA	NA	NA	NA	NA	1	1	NA	NA
IDH3G	NA	NA	NA	NA	NA	1	1	NA	NA
SSR4	NA	NA	NA	NA	NA	1	1	NA	NA
PDZD4	NA	NA	NA	NA	NA	1	1	NA	NA
L1CAM	NA	NA	NA	NA	NA	1	1	NA	NA
AVPR2	NA	NA	NA	NA	NA	1	1	NA	NA
ARHGAP4	NA	NA	NA	NA	NA	1	1	NA	NA
NAA10	NA	NA	NA	NA	NA	1	1	NA	NA
RENBP	NA	NA	NA	NA	NA	1	1	NA	NA
HCFC1	NA	NA	NA	NA	NA	1	1	NA	NA
TMEM187	NA	NA	NA	NA	NA	1	1	NA	NA
IRAK1	NA	NA	NA	NA	NA	1	1	NA	NA
MECP2	NA	NA	NA	NA	NA	1	1	NA	NA
OPN1LW	NA	NA	NA	NA	NA	1	1	NA	NA
OPN1MW2	NA	NA	NA	NA	NA	1	1	NA	NA
TEX28	NA	NA	NA	NA	NA	1	1	NA	NA
TKTL1	NA	NA	NA	NA	NA	1	1	NA	NA
FLNA	NA	NA	NA	NA	NA	1	1	NA	NA
EMD	NA	NA	NA	NA	NA	1	1	NA	NA
DNASE1L1	NA	NA	NA	NA	NA	1	1	NA	NA
TAZ	NA	NA	NA	NA	NA	1	1	NA	NA
ATP6AP1	NA	NA	NA	NA	NA	1	1	NA	NA
GDI1	NA	NA	NA	NA	NA	1	1	NA	NA
FAM50A	NA	NA	NA	NA	NA	1	1	NA	NA
PLXNA3	NA	NA	NA	NA	NA	1	1	NA	NA
LAGE3	NA	NA	NA	NA	NA	1	1	NA	NA
UBL4A	NA	NA	NA	NA	NA	1	1	NA	NA
SLC10A3	NA	NA	NA	NA	NA	1	1	NA	NA
FAM3A	NA	NA	NA	NA	NA	1	1	NA	NA
G6PD	NA	NA	NA	NA	NA	1	1	NA	NA
IKBKG	NA	NA	NA	NA	NA	1	1	NA	NA
CTAG2	NA	NA	NA	NA	NA	1	1	NA	NA
GAB3	NA	NA	NA	NA	NA	1	1	NA	NA
DKC1	NA	NA	NA	NA	NA	1	1	NA	NA
SMIM9	NA	NA	NA	NA	NA	1	1	NA	NA
F8	NA	NA	NA	NA	NA	1	1	NA	NA
H2AFB1	NA	NA	NA	NA	NA	1	1	NA	NA
F8A1	NA	NA	NA	NA	NA	1	1	NA	NA
FUNDC2	NA	NA	NA	NA	NA	1	1	NA	NA
CMC4	NA	NA	NA	NA	NA	1	1	NA	NA
MTCP1	NA	NA	NA	NA	NA	1	1	NA	NA
BRCC3	NA	NA	NA	NA	NA	1	1	NA	NA
VBP1	NA	NA	NA	NA	NA	1	1	NA	NA

RAB39B	NA	NA	NA	NA	NA	1	1	NA	NA
CLIC2	NA	NA	NA	NA	NA	1	1	NA	NA
TMLHE	NA	NA	NA	NA	NA	1	1	NA	NA
SRY	NA	NA	NA	NA	NA	1	1	NA	NA
RPS4Y1	NA	NA	NA	NA	NA	1	1	NA	NA
ZFY	NA	NA	NA	NA	NA	1	1	NA	NA
TGIF2LY	NA	NA	NA	NA	NA	1	1	NA	NA
PCDH11Y	NA	NA	NA	NA	NA	1	1	NA	NA
TSPY2	NA	NA	NA	NA	NA	1	1	NA	NA
AMELY	NA	NA	NA	NA	NA	1	1	NA	NA
TBL1Y	NA	NA	NA	NA	NA	1	1	NA	NA
TSPY8	NA	NA	NA	NA	NA	1	1	NA	NA
TSPY10	NA	NA	NA	NA	NA	1	1	NA	NA
USP9Y	NA	NA	NA	NA	NA	1	1	NA	NA
DDX3Y	NA	NA	NA	NA	NA	1	1	NA	NA
UTY	NA	NA	NA	NA	NA	1	1	NA	NA
TMSB4Y	NA	NA	NA	NA	NA	1	1	NA	NA
NLGN4Y	NA	NA	NA	NA	NA	1	1	NA	NA
KDM5D	NA	NA	NA	NA	NA	1	1	NA	NA
EIF1AY	NA	NA	NA	NA	NA	1	1	NA	NA
RPS4Y2	NA	NA	NA	NA	NA	1	1	NA	NA
RBMY1J	NA	NA	NA	NA	NA	1	1	NA	NA

Supplementary Table 2c. Enhancer results from RnBeads (DMR) analysis

Enhancer	mean.mea	mean.mea	mean.mea	mean.mea	comb.p.val	comb.p.ad	num.sites	mean.num	mean.num	target_gn
chr1:1550	0.057858	0.193331	-0.13547	-1.57301	2.39E-21	3.95E-17	2	0	0	CLK2
chr11:6393	0.066052	0.241551	-0.1755	-1.7258	2.83E-21	3.95E-17	1	0	0	MACROD1;GPR137;SLC22A11
chr17:3338	0.072549	0.201257	-0.12871	-1.35525	9.69E-21	8.62E-17	3	0	0	UNC45B;SLFN12
chr17:3339	0.070544	0.207345	-0.1368	-1.43206	1.24E-20	8.62E-17	2	0	0	SLFN11;SLFN13;SLFN12L
chr6:2948	0.197808	0.523789	-0.32598	-1.36102	1.62E-19	8.85E-16	1	0	0	HLA-F;HLA-A
chr20:3060	0.121426	0.33655	-0.21512	-1.45401	1.90E-19	8.85E-16	2	0	0	ASXL1
chr3:50265	0.052478	0.121857	-0.06938	-1.07755	2.26E-19	9.03E-16	1	0	0	MST1R
chr8:30580	0.141666	0.330228	-0.18856	-1.16758	2.74E-19	9.55E-16	2	0	0	GSR
chr16:7308	0.141065	0.395546	-0.25448	-1.42469	6.13E-19	1.90E-15	1	0	0	ZFHX3
chr15:9896	0.043896	0.127227	-0.08333	-1.34832	1.37E-18	3.82E-15	1	0	0	IGF1R
chr13:4159	0.195554	0.460025	-0.26447	-1.189	4.09E-18	9.22E-15	4	0	0	MRPS31;ELF1
chr14:9573	0.113486	0.294823	-0.18134	-1.30362	4.12E-18	9.22E-15	1	0	0	DICER1;CLMN
chr22:2423	0.09584	0.263162	-0.16732	-1.36787	4.29E-18	9.22E-15	1	0	0	MIF
chr8:2511C	0.78888	0.560307	0.228573	0.486371	4.73E-18	9.43E-15	2	0	0	DOCK5
chr16:6706	0.141857	0.276781	-0.13492	-0.91793	5.62E-18	1.05E-14	2	0	0	DYNC1LI2;NAE1;FBXL8;TRADD
chr7:27161	0.681195	0.426849	0.254346	0.66733	1.74E-17	3.03E-14	10	0	0	HOXA4;HOXA5;HOXA3
chr1:21854	0.090041	0.309015	-0.21897	-1.67303	2.86E-17	4.69E-14	1	0	0	TGFB2
chr17:3851	0.151756	0.264646	-0.11289	-0.76375	3.68E-17	5.71E-14	1	0	0	ORMDL3;MED24;RARA
chr6:43979	0.128294	0.262259	-0.13396	-0.97724	1.43E-16	2.10E-13	1	0	0	MAD2L1BP;NFKBIE
chr12:8112	0.109811	0.304776	-0.19496	-1.39356	2.16E-16	2.88E-13	1	0	0	NANOGNB;FOXJ2
chr12:8112	0.109811	0.304776	-0.19496	-1.39356	2.16E-16	2.88E-13	1	0	0	DPPA3;CLEC4C;NANOG;SLC2A14;SLC2A3;FOXJ2;NECAP1;CLEC4A;ZNF705A
chr11:6173	0.183649	0.311611	-0.12796	-0.73044	2.56E-16	3.25E-13	2	0	0	RAB3IL1
chr8:29413	0.320983	0.709942	-0.38896	-1.12113	2.86E-16	3.48E-13	1	0	0	DUSP4
chr11:1227	0.152839	0.429964	-0.27713	-1.43394	3.00E-16	3.49E-13	1	0	0	CRTAM
chr10:6008	0.111587	0.265503	-0.15392	-1.18008	3.24E-16	3.62E-13	1	0	0	IPMK;UBE2D1
chr15:7252	0.066165	0.141411	-0.07525	-0.99126	4.63E-16	4.97E-13	1	0	0	SENP8;MYO9A;HEXA
chr3:15285	0.138918	0.346049	-0.20713	-1.25756	6.23E-16	6.44E-13	1	0	0	MBNL1
chr4:1857C	0.060476	0.112273	-0.0518	-0.7949	7.61E-16	7.59E-13	1	0	0	FAM53A
chr4:99851	0.124963	0.289491	-0.16453	-1.13688	8.24E-16	7.93E-13	2	0	0	EIF4E;METAP1
chr12:5344	0.050602	0.125184	-0.07458	-1.10429	8.94E-16	8.32E-13	2	0	0	CSAD
chr16:302C	0.054002	0.101727	-0.04773	-0.80379	9.44E-16	8.50E-13	1	0	0	IL32;ZNF174
chr17:4011	0.114352	0.236556	-0.1222	-0.98748	1.15E-15	1.00E-12	1	0	0	KRT19;JUP;HSPB9;STAT5B;STAT3
chr17:435C	0.10978	0.229755	-0.11997	-0.90377	1.45E-15	1.22E-12	3	0	0	ARHGAP27
chr5:17081	0.243248	0.497493	-0.25424	-1.00283	1.71E-15	1.36E-12	1	0	0	RANBP17;NPM1
chr11:6398	0.322483	0.55981	-0.23733	-0.7772	1.68E-15	1.36E-12	1	0	0	ESRRA
chr21:4022	0.431589	0.646497	-0.21491	-0.57209	1.80E-15	1.39E-12	1	0	0	ETS2
chr3:12478	0.594568	0.428722	0.165845	0.462596	1.89E-15	1.43E-12	1	0	0	ITGB5;HEG1
chr1:28471	0.11647	0.277423	-0.16095	-1.18438	2.29E-15	1.60E-12	1	0	0	RPA2;PTAFR;RCC1
chr3:11111	0.276811	0.600628	-0.32382	-1.09019	2.29E-15	1.60E-12	1	0	0	CD96;PLCXD2
chr9:13014	0.14579	0.392344	-0.24655	-1.36883	2.20E-15	1.60E-12	1	0	0	SLC2A8;ZNF79;FAM129B
chr4:66898	0.10273	0.237647	-0.13492	-1.16075	2.60E-15	1.77E-12	3	0	0	S100P
chr20:4228	0.058036	0.125231	-0.0672	-0.87042	2.97E-15	1.98E-12	3	0	0	SGK2;IFT52;MYBL2
chr19:4638	0.235994	0.429538	-0.19354	-0.83736	3.14E-15	2.04E-12	1	0	0	DMWD;SYMPK;PPP5C
chr7:47641	0.330325	0.607492	-0.27717	-0.85951	3.63E-15	2.27E-12	1	0	0	TNS3;HUS1
chr10:7299	0.463998	0.744651	-0.28065	-0.67093	3.66E-15	2.27E-12	1	0	0	ITIH5
chr1:43432	0.16697	0.360922	-0.19395	-1.06761	3.96E-15	2.30E-12	1	0	0	ZNF691;SLC2A1;KDM4A
chr1:43432	0.16697	0.360922	-0.19395	-1.06761	3.96E-15	2.30E-12	1	0	0	CCDC30;SLC2A1;FAM183A
chr19:4614	0.083775	0.170163	-0.08639	-0.94203	3.92E-15	2.30E-12	1	0	0	ERCC2;PPM1N;SYMPK
chr3:16016	0.156131	0.386913	-0.23078	-1.2565	4.17E-15	2.37E-12	1	0	0	SMC4;IFT80;TRIM59
chr3:24537	0.199373	0.339608	-0.14024	-0.81844	4.73E-15	2.64E-12	3	0	0	THRB
chr13:5292	0.203326	0.44006	-0.23673	-1.07706	4.82E-15	2.64E-12	1	0	0	UTP14C
chr11:8809	0.179567	0.422615	-0.24305	-1.19038	5.12E-15	2.75E-12	1	0	0	CTSC
chr11:667C	0.352357	0.638747	-0.28639	-0.84025	5.67E-15	2.99E-12	1	0	0	SYT12;KDM2A;SSH3
chr4:84468	0.321588	0.635775	-0.31419	-1.00691	6.06E-15	3.13E-12	2	0	0	LIN54
chr4:84469	0.232752	0.566064	-0.33331	-1.24675	6.80E-15	3.45E-12	1	0	0	PLAC8;COQ2;HPSE;MRPS18C;HELQ
chr12:5221	0.196724	0.338538	-0.14181	-0.78135	6.97E-15	3.47E-12	5	0	0	KRT85
chr2:13158	0.058407	0.12056	-0.06215	-0.93249	7.96E-15	3.90E-12	1	0	0	IMP4;PTPN18;GPR148;PLEKHB2
chr9:13138	0.917128	0.827639	0.08949	0.146441	9.07E-15	4.36E-12	1	0	0	PKN3
chr8:30580	0.155704	0.304194	-0.14849	-0.92305	9.74E-15	4.53E-12	1	0	0	GSR;PPP2CB
chr9:1245C	0.695817	0.436062	0.259755	0.66205	9.73E-15	4.53E-12	1	0	0	DAB2IP;TTLL11;OR1J1
chr17:2653	0.052357	0.100578	-0.04822	-0.82644	1.03E-14	4.71E-12	1	0	0	SRR
chr7:10805	0.177725	0.41868	-0.24096	-1.19292	1.09E-14	4.83E-12	2	0	0	BCAP29;NRCAM;PNPLA8
chr7:10805	0.177725	0.41868	-0.24096	-1.19292	1.09E-14	4.83E-12	2	0	0	DNAJB9;PNPLA8
chr22:507C	0.352041	0.613415	-0.26137	-0.78404	1.11E-14	4.83E-12	1	0	0	TUBGCP6
chr6:1127C	0.66041	0.382708	0.277701	0.771585	1.14E-14	4.89E-12	1	0	0	MAK;NEDD9;TMEM170B
chr11:4801	0.318222	0.470107	-0.15188	-0.54393	1.29E-14	5.46E-12	2	0	0	AGBL2;PTPRJ;OR4B1
chr15:7252	0.29786	0.575577	-0.27772	-0.92758	1.35E-14	5.60E-12	1	0	0	SENP8;MYO9A;PARP6;CELF6;ARIH1
chr11:127C	0.175798	0.370629	-0.19483	-1.03465	1.45E-14	5.97E-12	1	0	0	TEAD1
chr17:7626	0.137896	0.252899	-0.115	-0.82992	1.50E-14	6.08E-12	1	0	0	TNRC6C;TMC8;TMC6;SOCS3;PGS1
chr1:45186	0.18494	0.337329	-0.15239	-0.82601	1.57E-14	6.26E-12	4	0	0	KIF2C;UROD
chr17:3703	0.646933	0.461726	0.185207	0.478123	1.60E-14	6.30E-12	2	0	0	RPL23;LASP1;PLXDC1
chr21:4697	0.067883	0.140415	-0.07253	-0.94957	1.70E-14	6.59E-12	1	0	0	POFUT2;SLC19A1
chr7:29325	0.234937	0.576835	-0.3419	-1.26055	1.74E-14	6.65E-12	1	0	0	CHN2
chr10:6438	0.419751	0.72738	-0.30763	-0.77891	1.77E-14	6.66E-12	1	0	0	IL15RA;PFKFB3
chr6:28831	0.072364	0.136097	-0.06373	-0.82676	2.00E-14	7.43E-12	2	0	0	ZSCAN23
chr12:4705	0.134646	0.325586	-0.19094	-1.21416	2.12E-14	7.77E-12	1	0	0	SLC38A2
chr3:12234	0.4838	0.728118	-0.24432	-0.57993	2.34E-14	8.38E-12	1	0	0	TAMM41;MKRN2
chr3:12236	0.4838	0.728118	-0.24432	-0.57993	2.34E-14	8.38E-12	1	0	0	TSEN2;RAF1
chr20:3913	0.205036	0.37729	-0.17225	-0.86008	2.70E-14	9.55E-12	2	0	0	MAFB
chr17:386C	0.766755	0.628012	0.138743	0.283876	2.91E-14	1.02E-11	1	0	0	RAPGEFL1;RARA;IGFBP4
chr19:4097	0.112167	0.235088	-0.12292	-1.00445	3.00E-14	1.03E-11	1	0	0	AKT2;PLD3;C19orf54
chr20:5046	0.160776	0.361167	-0.20039	-1.11996	3.19E-14	1.09E-11	1	0	0	PRNP;RASSF2;SLC23A2
chr12:4766	0.197021	0.468194	-0.27117	-1.20781	3.32E-14	1.12E-11	1	0	0	SLC38A4;PCED1B
chr11:1144	0.274948	0.413795	-0.13885	-0.57155	3.37E-14	1.12E-11	2	0	0	MUC5AC;AP2A2
chr10:2478	0.582819	0.804647	-0.22183	-0.45858	3.56E-14	1.17E-11	1	0	0	KIAA1217
chr14:6233	0.401471	0.716294	-0.31482	-0.81976	3.63E-14	1.18E-11	1	0	0	SNAPC1
chr19:162C	0.862072	0.765186	0.096887	0.169906	3.67E-14	1.18E-11	1	0	0	OR10H4
chr1:18658	0.092294	0.211152	-0.11886	-1.11232	4.16E-14	1.30E-11	1	0	0	PLA2G4A
chr1:18658	0.092294	0.211152	-0.11886	-1.11232	4.16E-14	1.30E-11	1	0	0	TPR;PTGS2;PLA2G4A
chr15:9941	0.311056	0.546606	-0.23555	-0.79383	4.25E-14	1.32E-11	1	0	0	LRRC28;TTC23
chr8:12521	0.180353	0.381153	-0.2008	-1.03905	4.35E-14	1.33E-11	1	0	0	TMEM65;RNF139
chr10:121C	0.319256	0.611976	-0.29272	-0.91765	5.03E-14	1.53E-11	1	0	0	FAM45A;GRK5;TIAL1
chr1:19252	0.203949	0.525308	-0.32136	-1.3231	5.30E-14	1.54E-11	1	0	0	RGS1;RGS13;RGS2
chr10:1208	0.205056	0.488518	-0.28346	-1.21294	5.23E-14	1.54E-11	1	0	0	EIF3A;RGS10
chr17:3982	0.083356	0.157666	-0.07431	-0.84478	5.28E-14	1.54E-11	1	0	0	EIF1;DHX58
chr22:297C	0.699637	0.507874	0.191763	0.45448	5.19E-14	1.54E-11	1	0	0	EMID1;GAS2L1
chr22:3964	0.075331	0.14908	-0.07375	-0.89861	5.48E-14	1.58E-11	1	0	0	SUN2;APOBEC3A;PDGFB;RPL3
chr12:6605	0.201367	0.512954	-0.31159	-1.30693	5.83E-14	1.66E-11	1	0	0	LEMD3;HMGA2
chr10:7594	0.195278	0.399949	-0.20467	-0.99786	5.93E-14	1.67E-11	1	0	0	PLAU;ADK
chr17:4332	0.181569	0.378177	-0.19661	-1.01885	6.32E-14	1.76E-11	1	0	0	CCDC103;FAM187A;ARHGAP27
chr4:18518	0.258117	0.497075	-0.23896	-0.92964	6.49E-14	1.78E-11	3	0	0	RWDD4
chr12:1209	0.092479	0.192104	-0.09962	-0.97977	6.44E-14	1.78E-11	1	0	0	RNF10
chr3:19459	0.26946	0.455147	-0.18569	-0.73504	6.64E-14	1.80E-11	1	0	0	TMEM44;FAM43A
chr15:9334	0.091506	0.212251	-0.12075	-1.13063	6.74E-14	1.81E-11	1	0	0	CHD2
chr2:88315	0.085728	0.222863	-0.13714	-1.28247	7.22E-14	1.92E-11	1	0	0	RGPD2;SMYD1;THNSL2
chr2:17167	0.25747	0.022058	0.235412	3.060623	7.94E-14	2.07E-11	1	0	0	GAD1
chr4:2632C	0.061212	0.122107	-0.06089	-0.89151	7.97E-14	2.07E-11	1	0	0	RBPJ;TBC1D19
chr12:1252	0.338203	0.533997	-0.19579	-0.64367	8.11E-14	2.08E-11	1	0	0	NCOR2;UBC
chr4:95428	0.749342	0.577036	0.172307	0.371303	9.44E-14	2.40E-11	1	0	0	PDLIM5

chr16:3019	0.239771	0.307934	-0.06816	-0.51763	3.37E-13	6.58E-11	6	0	0	FLYWCH2;OR1F1
chr19:4728	0.14571	0.232175	-0.08646	-0.78765	3.29E-13	6.58E-11	7	0	0	PPP5D1;STRN4
chr19:4822	0.153798	0.298601	-0.1448	-0.91382	3.44E-13	6.67E-11	1	0	0	KPTN;EHD2;KDELR1
chr10:1060	0.759889	0.596297	0.163592	0.344625	3.47E-13	6.68E-11	1	0	0	COL17A1
chr2:17698	0.349465	0.138977	0.210488	1.34947	3.57E-13	6.82E-11	3	0	0	HOXD10;HOXD9;HOXD8;HOXD1
chr7:10600	0.207853	0.383784	-0.17593	-0.85405	3.91E-13	7.37E-11	1	0	0	NAMPT
chr12:9353	0.454275	0.708048	-0.25377	-0.6291	3.99E-13	7.47E-11	1	0	0	EEA1
chr14:6212	0.128219	0.313695	-0.18548	-1.22768	4.19E-13	7.79E-11	1	0	0	HIF1A;SNAPC1
chr20:3390	0.380524	0.700191	-0.31967	-0.86279	4.44E-13	8.21E-11	1	0	0	ACSS2;GSS;EIF6;ERGIC3
chr11:6442	0.589221	0.747725	-0.1585	-0.33858	4.58E-13	8.39E-11	1	0	0	NRXN2;EHD1;BATF2
chr12:3309	0.038402	0.109255	-0.07085	-1.30092	4.60E-13	8.39E-11	1	0	0	TSPAN9;PRMT8
chr3:11363	0.197312	0.346593	-0.14928	-0.78247	4.78E-13	8.60E-11	1	0	0	SPICE1
chr17:3907	0.182481	0.356108	-0.17363	-0.92755	4.77E-13	8.60E-11	1	0	0	KRTAP4-6
chr6:12611	0.22249	0.352617	-0.13013	-0.64128	4.85E-13	8.67E-11	1	0	0	NCOA7
chr7:22894	0.133337	0.280337	-0.147	-0.91698	4.99E-13	8.70E-11	2	0	0	STEAP1B;TOMM7
chr12:6457	0.801427	0.638264	0.163163	0.323879	4.93E-13	8.70E-11	1	0	0	C12orf66
chr12:6457	0.801427	0.638264	0.163163	0.323879	4.93E-13	8.70E-11	1	0	0	SRGAP1
chr22:3205	0.215416	0.353175	-0.13776	-0.75711	4.97E-13	8.70E-11	7	0	0	PISD;DEPDC5
chr4:18518	0.358521	0.611094	-0.25257	-0.75306	5.06E-13	8.77E-11	1	0	0	ENPP6;IRF2;CASP3
chr20:5659	0.558376	0.780441	-0.22206	-0.47581	5.12E-13	8.81E-11	1	0	0	PMEPA1
chr12:1322	0.431196	0.76646	-0.33526	-0.81549	5.23E-13	8.91E-11	1	0	0	MMP17
chr20:1550	0.484272	0.774354	-0.29008	-0.67199	5.24E-13	8.91E-11	2	0	0	FKBP1A
chr3:53551	0.664339	0.443218	0.221122	0.573271	5.49E-13	9.17E-11	1	0	0	TKT;CACNA1D
chr7:54360	0.085959	0.16674	-0.08078	-0.88113	5.59E-13	9.17E-11	1	0	0	WIPI2
chr7:54369	0.085959	0.16674	-0.08078	-0.88113	5.59E-13	9.17E-11	1	0	0	RBAK;TNRC18;ACTB;RNF216
chr8:27138	0.407379	0.612455	-0.20508	-0.57661	5.50E-13	9.17E-11	1	0	0	PTK2B
chr15:5964	0.167116	0.293907	-0.12679	-0.77894	5.52E-13	9.17E-11	1	0	0	RNF111;BNIP2
chr20:5030	0.404403	0.67451	-0.27011	-0.72404	5.52E-13	9.17E-11	1	0	0	KCNG1;NFATC2
chr21:3445	0.179728	0.297171	-0.11744	-0.69511	5.62E-13	9.17E-11	1	0	0	SYNJ1;OLIG2;OLIG1;IFNAR2;IL10RB;IFNAR1;IFNGR2;SON;GART
chr1:42327	0.424169	0.645245	-0.22108	-0.67254	5.82E-13	9.44E-11	2	0	0	HIVEP3;FOXJ3
chr10:7401	0.483855	0.332189	0.151665	0.683809	5.86E-13	9.46E-11	2	0	0	ASCC1;MICU1
chr19:4182	0.549675	0.373838	0.175837	0.625639	5.95E-13	9.55E-11	2	0	0	HNRNPUL1;TGFB1;B9D2
chr8:16932	0.431306	0.68628	-0.25497	-0.65789	6.04E-13	9.63E-11	1	0	0	CLN8
chr6:12623	0.200366	0.396011	-0.19565	-0.94862	6.14E-13	9.74E-11	1	0	0	TRMT11
chr13:4562	0.162249	0.312659	-0.15041	-0.90552	6.20E-13	9.78E-11	1	0	0	KCTD4
chr3:58456	0.263008	0.414399	-0.15139	-0.63744	6.73E-13	1.06E-10	2	0	0	KCTD6
chr17:2791	0.271675	0.398587	-0.12691	-0.56319	6.95E-13	1.08E-10	2	0	0	EFCAB5
chr10:9918	0.376547	0.69839	-0.32184	-0.8739	7.06E-13	1.09E-10	1	0	0	ARHGAP19
chr7:24861	0.271932	0.600409	-0.32848	-1.11443	7.57E-13	1.17E-10	1	0	0	CYCS;C7orf31
chr12:2585	0.651292	0.832718	-0.18143	-0.34976	7.93E-13	1.22E-10	1	0	0	RASSF8
chr12:1228	0.322535	0.573491	-0.25096	-0.8112	8.06E-13	1.23E-10	1	0	0	LRRC43;DIABLO;VPS33A;ZCCHC8;KNTC1;RSRC2;DENR;CCDC62;VPS37B
chr11:7481	0.086161	0.188776	-0.10262	-1.04762	8.43E-13	1.28E-10	1	0	0	SLCO2B1
chr4:40721	0.089624	0.163164	-0.07354	-0.79757	8.79E-13	1.33E-10	1	0	0	RBM47;APBB2
chr9:12310	0.533122	0.756514	-0.22339	-0.49703	9.04E-13	1.36E-10	1	0	0	CDK5RAP2
chr9:35076	0.355207	0.598892	-0.24368	-0.73747	9.46E-13	1.40E-10	1	0	0	RFX3
chr9:35087	0.355207	0.598892	-0.24368	-0.73747	9.46E-13	1.40E-10	1	0	0	RFX3
chr9:11813	0.50832	0.763877	-0.25556	-0.58054	9.43E-13	1.40E-10	2	0	0	TNFSF8;TNC;DEC1
chr14:1051	0.2591	0.415215	-0.15612	-0.66005	9.50E-13	1.40E-10	1	0	0	PLD4
chr15:8527	0.212588	0.325692	-0.1131	-0.59276	9.92E-13	1.45E-10	1	0	0	SLC28A1;PDE8A
chr18:4645	0.551773	0.386153	0.16562	0.503929	1.01E-12	1.46E-10	1	0	0	SMAD7
chr19:1447	0.147024	0.278849	-0.13183	-0.87933	1.15E-12	1.67E-10	1	0	0	ASF1B;PKN1
chr12:6648	0.472489	0.652844	-0.18036	-0.45817	1.24E-12	1.77E-10	1	0	0	CD9
chr16:2077	0.127495	0.24384	-0.11635	-0.88085	1.24E-12	1.77E-10	2	0	0	ACSM3
chr10:1150	0.120459	0.298663	-0.1782	-1.24243	1.25E-12	1.78E-10	1	0	0	TCF7L2;CASP7
chr19:4170	0.329973	0.504689	-0.17472	-0.59828	1.28E-12	1.81E-10	1	0	0	ITPKC;CCDC97;TGFB1
chr7:54898	0.407473	0.643029	-0.23556	-0.64546	1.32E-12	1.85E-10	1	0	0	EGFR
chr14:7503	0.201835	0.345123	-0.14329	-0.87237	1.34E-12	1.86E-10	2	0	0	MLH3;ZC2HC1C
chr17:1965	0.353822	0.491441	-0.13762	-0.4629	1.37E-12	1.90E-10	2	0	0	B9D1;ALDH3A1;ULK2
chr11:6777	0.39142	0.539226	-0.14781	-0.45229	1.38E-12	1.91E-10	1	0	0	UNC93B1;NDUFS8
chr1:24180	0.507478	0.650963	-0.14349	-0.35307	1.46E-12	2.01E-10	1	0	0	KMO;CHML
chr2:10607	0.142117	0.251734	-0.10962	-0.78292	1.60E-12	2.16E-10	1	0	0	FHL2
chr7:87193	0.120061	0.217141	-0.09708	-0.8044	1.60E-12	2.16E-10	1	0	0	ABCB4;ABCB1;SLC25A40
chr11:1968	0.134992	0.25823	-0.12324	-0.8875	1.58E-12	2.16E-10	1	0	0	NAV2
chr15:6453	0.425172	0.671877	-0.24671	-0.64793	1.60E-12	2.16E-10	1	0	0	USP3
chr11:6665	0.312642	0.507045	-0.1944	-0.86553	1.66E-12	2.22E-10	3	0	0	RBM14;SYT12;KDM2A
chr6:11770	0.676021	0.822088	-0.14607	-0.29098	1.69E-12	2.25E-10	4	0	0	ADTRP
chr11:6665	0.176954	0.350162	-0.17321	-1.05629	1.72E-12	2.28E-10	2	0	0	RBM4;PC;KDM2A;CLCF1
chr10:9104	0.685302	0.450695	0.234607	0.593826	1.75E-12	2.31E-10	1	0	0	CH25H
chr1:17768	0.227262	0.423408	-0.19615	-0.86925	1.89E-12	2.48E-10	1	0	0	MFAP2
chr19:2014	0.079982	0.158531	-0.07855	-0.9053	1.92E-12	2.50E-10	1	0	0	MKNK2;IZUMO4
chr3:41308	0.633065	0.853727	-0.22066	-0.42561	1.97E-12	2.54E-10	1	0	0	CTNNB1
chr3:41309	0.633065	0.853727	-0.22066	-0.42561	1.97E-12	2.54E-10	1	0	0	CTNNB1
chr7:73129	0.556511	0.744458	-0.18795	-0.41334	1.99E-12	2.56E-10	1	0	0	STX1A;ABHD11
chr13:2548	0.401993	0.662589	-0.2606	-0.7071	2.11E-12	2.71E-10	1	0	0	PARP4;PABPC3;MTMR6
chr9:21681	0.458058	0.668155	-0.2101	-0.53493	2.16E-12	2.76E-10	1	0	0	IFNA8;IFNA1
chr2:19810	0.612473	0.804603	-0.19213	-0.38809	2.19E-12	2.78E-10	1	0	0	TTC32;PUM2
chr1:45187	0.185117	0.36673	-0.18161	-0.94919	2.27E-12	2.83E-10	1	0	0	KIF2C;PLK3
chr10:3502	0.372557	0.566079	-0.19352	-0.59059	2.26E-12	2.83E-10	1	0	0	CUL2
chr10:6419	0.125785	0.306364	-0.18058	-1.22026	2.25E-12	2.83E-10	1	0	0	EGR2
chr6:27797	0.101885	0.197758	-0.09587	-0.85889	2.30E-12	2.87E-10	2	0	0	HIST1H3H;HIST1H4J;HIST1H1B
chr3:12455	0.083349	0.190883	-0.10753	-1.10564	2.36E-12	2.92E-10	1	0	0	ITGB5;HEG1
chr17:2964	0.35005	0.595586	-0.24554	-0.75014	2.37E-12	2.92E-10	1	0	0	NF1;EVI2B;EVI2A
chr6:33240	0.056277	0.093851	-0.03757	-0.67681	2.39E-12	2.92E-10	3	0	0	BRD2;SLC39A7;PFDN6
chr11:9347	0.336897	0.580115	-0.24322	-0.76649	2.40E-12	2.93E-10	1	0	0	TAF1D
chr1:94126	0.16591	0.34225	-0.17634	-1.00176	2.50E-12	3.03E-10	1	0	0	BCAR3
chr1:94127	0.16591	0.34225	-0.17634	-1.00176	2.50E-12	3.03E-10	1	0	0	TMED5;DNTTIP2;GCLM
chr1:16955	0.169139	0.376504	-0.20737	-1.10941	2.54E-12	3.06E-10	1	0	0	BLZF1;SLC19A2;F5;SELP;SCYL3
chr20:8218	0.23711	0.356579	-0.11947	-0.56869	2.59E-12	3.10E-10	2	0	0	TRIB3
chr11:1267	0.474454	0.750639	-0.27618	-0.65085	2.61E-12	3.11E-10	1	0	0	FAM118B
chr2:24019	0.49993	0.681	-0.18107	-0.44686	2.64E-12	3.14E-10	2	0	0	HDAC4
chr20:3106	0.615991	0.475083	0.140907	0.367908	2.89E-12	3.41E-10	1	0	0	KIF3B
chr18:7772	0.085383	0.139938	-0.05455	-0.71908	2.93E-12	3.45E-10	6	0	0	PQLC1
chr14:7121	0.865869	0.779557	0.086311	0.149619	2.99E-12	3.47E-10	2	0	0	TTC9
chr20:3028	0.827597	0.670099	0.157498	0.300512	2.96E-12	3.47E-10	1	0	0	BCL2L1
chr22:4333	0.166855	0.33813	-0.17128	-0.97706	2.98E-12	3.47E-10	1	0	0	POLDIP3;ARFGAP3;PACSIN2
chr1:94169	0.630768	0.793264	-0.1625	-0.32704	3.05E-12	3.53E-10	2	0	0	DNTTIP2;GCLM
chr2:20148	0.547047	0.745724	-0.19868	-0.44006	3.10E-12	3.58E-10	1	0	0	AOX1
chr2:11245	0.085622	0.216048	-0.13043	-1.24122	3.16E-12	3.62E-10	1	0	0	ANAPC1
chr10:2992	0.662857	0.885763	-0.22291	-0.41282	3.18E-12	3.64E-10	1	0	0	PITRM1
chr10:9944	0.402705	0.710897	-0.30819	-0.80468	3.23E-12	3.68E-10	1	0	0	RRP12;PI4K2A
chr6:13468	0.61418	0.844013	-0.22983	-0.4523	3.27E-12	3.71E-10	1	0	0	SGK1
chr3:18567	0.275757	0.455899	-0.18014	-0.68569	3.47E-12	3.92E-10	2	0	0	IGF2BP2
chr2:15606	0.646591	0.785121	-0.13853	-0.27618	3.59E-12	4.04E-10	1	0	0	PXDN
chr10:7309	0.476419	0.626133	-0.14971	-0.38713	3.62E-12	4.06E-10	1	0	0	SLC29A3
chr22:3793	0.272493	0.433589	-0.1611	-0.65101	4.04E-12	4.51E-10	1	0	0	IL2RB;LGALS2;GGA1;SH3BP1
chr8:67042	0.222166	0.436641	-0.21447	-0.94396	4.06E-12	4.52E-10	1	0	0	MTFR1
chr3:15470	0.080759	0.151322	-0.07056	-0.77723	4.14E-12	4.57E-10	2	0	0	ANKRD28
chr15:8545	0.523519	0.650574	-0.12706	-0.49062	4.13E-12	4.57E-10	2	0	0	PDE8A
chr17:7790	0.10139	0.140156	-0.03877	-0.43085	4.23E-12	4.63E-10	1	0	0	WRAP53;KDM6B;CHD3;PER1;CTC1
chr17:8016	0.12016	0.245947	-0.12579	-0.97556	4.34E-12	4.73E-10	1	0	0	SIRT7;MAFG;MYADML2;CCDC57;SLC16A3;CSNK1D;SECTM1;NARF
chr20:5236	0.605031	0.893786	-0.28875	-0.55532	4.39E-12	4.76E-10	1	0	0	ZNF217

Supplementary Table 2d. pDMRs by RnBeads.

gene	mean.meai	mean.meai	mean.meai	mean.meai	comb.p.val	comb.p.ad	num.sites	mean.num	mean.num.na.g2
SYTL1	0.267155	0.432841	-0.16569	-0.76933	1.54E-16	1.38E-12	10	0	0
ZNF385A	0.317379	0.447082	-0.1297	-0.94785	8.62E-17	1.38E-12	11	0	0
PTPN6	0.325487	0.496326	-0.17084	-0.69072	4.24E-16	1.90E-12	12	0	0
TBX4	0.36327	0.099768	0.263502	2.001752	3.84E-16	1.90E-12	6	0	0
MAP1LC3B	0.355048	0.565143	-0.2101	-0.71987	1.20E-15	4.30E-12	10	0	0
KLRD1	0.753831	0.853369	-0.09954	-0.17672	2.89E-15	8.63E-12	1	0	0
TSTD1	0.387094	0.548592	-0.1615	-0.81671	1.11E-14	2.85E-11	13	0	0
RNASE3	0.379134	0.637105	-0.25797	-0.73373	1.69E-14	3.80E-11	1	0	0
CST7	0.355344	0.575926	-0.22058	-0.80531	2.31E-14	4.60E-11	2	0	0
HOXD12	0.383191	0.189724	0.193467	1.090421	4.54E-14	8.15E-11	12	0	0
HOXD4	0.432059	0.213338	0.218721	1.050172	5.19E-14	8.46E-11	17	0	0
HOXA5	0.572521	0.356266	0.216255	0.678082	6.41E-14	9.58E-11	35	0	0
LGALS9B	0.224959	0.362169	-0.13721	-0.66355	7.25E-14	1.00E-10	1	0	0
CDHR2	0.293039	0.467574	-0.17454	-0.60825	1.05E-13	1.11E-10	10	0	0
SIM1	0.366479	0.161861	0.204618	1.228848	1.01E-13	1.11E-10	6	0	0
HOXB1	0.770457	0.59891	0.171547	0.375116	9.33E-14	1.11E-10	13	0	0
PARVG	0.438204	0.603503	-0.1653	-0.46686	1.04E-13	1.11E-10	10	0	0
NUP210L	0.601068	0.409366	0.191702	0.683163	1.25E-13	1.25E-10	8	0	0
ATG16L1	0.677485	0.854749	-0.17726	-0.33095	1.78E-13	1.68E-10	1	0	0
PFN3	0.681597	0.538663	0.142934	0.449153	2.37E-13	2.13E-10	9	0	0
TCN1	0.504394	0.720644	-0.21625	-0.5823	2.68E-13	2.29E-10	5	0	0
PISD	0.212119	0.347858	-0.13574	-0.76859	2.91E-13	2.37E-10	6	0	0
OR6Q1	0.367029	0.58988	-0.22285	-0.67	3.25E-13	2.54E-10	1	0	0
CABLES1	0.30283	0.465999	-0.16317	-0.67845	4.71E-13	3.52E-10	8	0	0
SFTA3	0.278954	0.125756	0.153197	1.177703	5.42E-13	3.89E-10	15	0	0
ANTXRL	0.505011	0.658595	-0.15358	-0.37653	6.89E-13	4.46E-10	1	0	0
C17orf107	0.202093	0.345202	-0.14311	-0.90996	6.82E-13	4.46E-10	9	0	0
HOXB7	0.463088	0.261899	0.20119	0.976592	6.96E-13	4.46E-10	5	0	0
IL1A	0.442188	0.588691	-0.1465	-0.44602	8.13E-13	4.86E-10	6	0	0
EVPLL	0.356833	0.521951	-0.16512	-0.53617	7.90E-13	4.86E-10	1	0	0
SLCO2B1	0.086161	0.188776	-0.10262	-1.04762	8.43E-13	4.88E-10	1	0	0
AJAP1	0.277719	0.093741	0.183978	1.540651	1.30E-12	7.26E-10	4	0	0
IL1B	0.431561	0.609701	-0.17814	-0.7011	1.48E-12	8.07E-10	6	0	0
SCT	0.645222	0.488653	0.15657	0.823851	1.60E-12	8.47E-10	7	0	0
ZPBP	0.394896	0.639122	-0.24423	-0.68094	1.77E-12	9.07E-10	1	0	0
CD80	0.137355	0.266641	-0.12929	-0.85174	2.38E-12	1.19E-09	4	0	0
SPTA1	0.307847	0.570359	-0.26251	-0.87411	2.59E-12	1.20E-09	2	0	0
HOXD9	0.396509	0.203997	0.192513	1.05543	2.62E-12	1.20E-09	10	0	0
SERPING1	0.572198	0.413406	0.158793	0.472248	2.54E-12	1.20E-09	8	0	0
OR1S2	0.643684	0.850417	-0.20673	-0.39644	2.95E-12	1.32E-09	1	0	0
GRIK2	0.332377	0.185816	0.146561	0.939194	3.26E-12	1.39E-09	15	0	0
RNF135	0.400649	0.69066	-0.29001	-0.77081	3.22E-12	1.39E-09	1	0	0
HLA-DOB	0.687548	0.849597	-0.16205	-0.30105	3.40E-12	1.42E-09	2	0	0
CD5L	0.46191	0.550919	-0.08901	-0.24928	4.11E-12	1.68E-09	1	0	0
SPATA12	0.318427	0.490526	-0.1721	-0.62224	5.48E-12	2.18E-09	3	0	0
REG1B	0.674774	0.897074	-0.2223	-0.40559	5.74E-12	2.24E-09	1	0	0
PLA2G6	0.574867	0.448592	0.126275	0.350898	6.07E-12	2.32E-09	1	0	0
GPBAR1	0.761932	0.608793	0.153139	0.392341	7.04E-12	2.58E-09	3	0	0
KIF2B	0.670239	0.826737	-0.1565	-0.30668	7.02E-12	2.58E-09	8	0	0
ART4	0.762676	0.654614	0.108062	0.231818	7.81E-12	2.75E-09	13	0	0
ZNF358	0.673365	0.504151	0.169214	0.460073	7.82E-12	2.75E-09	4	0	0
OR51I1	0.603874	0.771622	-0.16775	-0.35511	9.42E-12	3.25E-09	2	0	0
OTX2	0.431921	0.229406	0.202514	1.019839	9.80E-12	3.32E-09	9	0	0

AGER	0.734201	0.630236	0.103965	0.234451	1.06E-11	3.52E-09	5	0	0
CIITA	0.300093	0.456651	-0.15656	-0.64462	1.13E-11	3.70E-09	10	0	0
IFNA2	0.438393	0.628691	-0.1903	-0.55332	1.24E-11	3.95E-09	2	0	0
FCN1	0.584445	0.758435	-0.17399	-0.37039	1.25E-11	3.95E-09	2	0	0
ADAM28	0.216574	0.364614	-0.14804	-0.67512	1.34E-11	4.12E-09	5	0	0
PRDM11	0.514444	0.661949	-0.1475	-0.39614	1.38E-11	4.12E-09	7	0	0
SCO1	0.186577	0.319428	-0.13285	-0.53193	1.37E-11	4.12E-09	3	0	0
GNA15	0.525779	0.645649	-0.11987	-0.45175	1.66E-11	4.88E-09	8	0	0
C1QTNF1	0.670038	0.542924	0.127114	0.34671	1.70E-11	4.92E-09	10	0	0
FAM107A	0.560819	0.416805	0.144014	0.421178	1.88E-11	5.36E-09	4	0	0
GJC2	0.675608	0.572683	0.102924	0.23801	1.94E-11	5.43E-09	9	0	0
ZNF154	0.421742	0.210547	0.211195	1.347678	2.09E-11	5.76E-09	12	0	0
CCDC81	0.479288	0.315343	0.163944	0.703263	2.24E-11	6.08E-09	9	0	0
PAX3	0.289508	0.139626	0.149882	1.126867	2.34E-11	6.16E-09	15	0	0
DAPP1	0.385831	0.568175	-0.18234	-0.58054	2.31E-11	6.16E-09	7	0	0
GAPT	0.541525	0.726966	-0.18544	-0.45219	2.54E-11	6.61E-09	4	0	0
SPP1	0.424554	0.570278	-0.14572	-0.58301	2.72E-11	6.96E-09	7	0	0
RBM47	0.395669	0.536347	-0.14068	-0.45704	2.86E-11	7.18E-09	10	0	0
NEK8	0.204546	0.290751	-0.0862	-0.65388	2.88E-11	7.18E-09	4	0	0
ZKSCAN4	0.418589	0.255784	0.162805	1.00125	2.97E-11	7.29E-09	9	0	0
SPOCK1	0.401671	0.635996	-0.23433	-0.65003	3.62E-11	8.77E-09	1	0	0
DPP6	0.37807	0.203097	0.174973	0.981465	3.82E-11	9.15E-09	13	0	0
OR10W1	0.588121	0.774848	-0.18673	-0.39879	4.19E-11	9.90E-09	3	0	0
SENP2	0.795024	0.703383	0.091641	0.195951	5.09E-11	1.19E-08	4	0	0
SLC7A11	0.345979	0.452102	-0.10612	-0.47693	5.43E-11	1.25E-08	7	0	0
OR5A1	0.548421	0.764479	-0.21606	-0.47599	5.56E-11	1.26E-08	2	0	0
OR10K2	0.606456	0.828435	-0.22198	-0.44855	5.74E-11	1.27E-08	3	0	0
LYVE1	0.5702	0.756153	-0.18595	-0.40708	5.70E-11	1.27E-08	3	0	0
RPH3A	0.462806	0.640927	-0.17812	-0.46125	6.21E-11	1.36E-08	1	0	0
S100A8	0.431399	0.582954	-0.15155	-0.42352	6.41E-11	1.37E-08	7	0	0
CD300E	0.460907	0.624813	-0.16391	-0.49452	6.37E-11	1.37E-08	7	0	0
DRD5	0.475442	0.278695	0.196748	0.821725	6.70E-11	1.40E-08	5	0	0
HEPACAM	0.625482	0.775035	-0.14955	-0.3143	6.65E-11	1.40E-08	7	0	0
HTR3D	0.428422	0.56827	-0.13985	-0.42254	7.04E-11	1.45E-08	3	0	0
DRD4	0.396596	0.22207	0.174526	1.380457	7.85E-11	1.60E-08	9	0	0
BFSP2	0.686443	0.801749	-0.11531	-0.22566	7.96E-11	1.60E-08	5	0	0
VSX1	0.313672	0.166229	0.147443	1.071431	8.21E-11	1.64E-08	14	0	0
TBX5	0.29319	0.132514	0.160676	1.248131	8.37E-11	1.65E-08	19	0	0
OR10J3	0.526277	0.723128	-0.19685	-0.45109	8.47E-11	1.65E-08	1	0	0
PCDHGA6	0.415782	0.266638	0.149144	0.776375	9.14E-11	1.76E-08	10	0	0
HYAL2	0.56535	0.438713	0.126637	0.340712	9.79E-11	1.85E-08	11	0	0
SIGLECL1	0.279059	0.455912	-0.17685	-0.68869	9.81E-11	1.85E-08	1	0	0
SLC4A10	0.361888	0.227768	0.13412	0.849554	1.00E-10	1.85E-08	8	0	0
RNASE6	0.693808	0.786498	-0.09269	-0.23934	9.93E-11	1.85E-08	4	0	0
P4HA2	0.33134	0.443308	-0.11197	-0.48149	1.02E-10	1.86E-08	9	0	0
BAHD1	0.226178	0.304662	-0.07848	-0.41589	1.13E-10	2.05E-08	6	0	0
FPR1	0.380051	0.545302	-0.16525	-0.53933	1.19E-10	2.14E-08	2	0	0
LPAR5	0.403426	0.505863	-0.10244	-0.36226	1.26E-10	2.23E-08	13	0	0
INTS1	0.355434	0.458271	-0.10284	-0.35088	1.35E-10	2.34E-08	5	0	0
OR9I1	0.712124	0.8516	-0.13948	-0.24987	1.34E-10	2.34E-08	2	0	0
TAS2R16	0.643995	0.787921	-0.14393	-0.28864	1.36E-10	2.35E-08	6	0	0
TMC6	0.446101	0.558131	-0.11203	-0.46181	1.38E-10	2.36E-08	13	0	0
MLIP	0.58169	0.782021	-0.20033	-0.40262	1.41E-10	2.38E-08	5	0	0
SLC34A1	0.771193	0.767961	0.003232	0.003123	1.43E-10	2.40E-08	2	0	0
LIMD2	0.342726	0.178064	0.164662	1.18645	1.44E-10	2.40E-08	4	0	0

ECSCR	0.770932	0.683343	0.087588	0.179041	1.47E-10	2.42E-08	6	0	0
CNBD1	0.58958	0.771185	-0.18161	-0.37523	1.50E-10	2.44E-08	4	0	0
WDR72	0.682144	0.861129	-0.17898	-0.33194	1.58E-10	2.56E-08	2	0	0
LAIR1	0.372119	0.528529	-0.15641	-0.50117	1.68E-10	2.69E-08	7	0	0
ZNF578	0.356834	0.243989	0.112845	0.85718	1.74E-10	2.76E-08	10	0	0
OR14J1	0.646489	0.804962	-0.15847	-0.32108	1.78E-10	2.80E-08	7	0	0
S100A11	0.05051	0.090131	-0.03962	-0.51558	1.83E-10	2.86E-08	5	0	0
SPATA18	0.236598	0.308795	-0.0722	-0.44963	1.92E-10	2.97E-08	12	0	0
CD68	0.267333	0.34346	-0.07613	-0.49756	2.04E-10	3.13E-08	7	0	0
ZNF835	0.306828	0.137065	0.169763	1.322544	2.15E-10	3.27E-08	8	0	0
HCAR3	0.399164	0.599137	-0.19997	-0.57409	2.21E-10	3.32E-08	1	0	0
EXD3	0.357004	0.471077	-0.11407	-0.41049	2.28E-10	3.41E-08	3	0	0
ETS2	0.188137	0.281249	-0.09311	-0.61869	2.37E-10	3.51E-08	7	0	0
KCNJ8	0.467643	0.23639	0.231253	1.055029	2.44E-10	3.58E-08	2	0	0
PUM2	0.399004	0.515494	-0.11649	-0.46381	2.48E-10	3.62E-08	5	0	0
ABCC9	0.252646	0.108464	0.144183	1.351059	2.54E-10	3.67E-08	15	0	0
OR10Z1	0.552691	0.773042	-0.22035	-0.47911	2.66E-10	3.78E-08	4	0	0
DENND1B	0.168725	0.238458	-0.06973	-0.40283	2.70E-10	3.78E-08	15	0	0
ZSCAN18	0.533344	0.460374	0.07297	0.339739	2.69E-10	3.78E-08	8	0	0
SMOX	0.28083	0.411164	-0.13033	-0.53421	2.68E-10	3.78E-08	1	0	0
CLIC3	0.293264	0.397902	-0.10464	-0.5208	2.86E-10	3.94E-08	11	0	0
KRTAP11-1	0.693767	0.832054	-0.13829	-0.26703	2.85E-10	3.94E-08	5	0	0
ZNF540	0.493669	0.427892	0.065777	0.484599	2.99E-10	4.09E-08	11	0	0
LYPD8	0.59247	0.727879	-0.13541	-0.31998	3.18E-10	4.25E-08	8	0	0
FOXI2	0.316937	0.156228	0.160709	1.193507	3.14E-10	4.25E-08	12	0	0
ZBP1	0.482695	0.583458	-0.10076	-0.40499	3.17E-10	4.25E-08	7	0	0
GPR141	0.533361	0.63636	-0.103	-0.31745	3.28E-10	4.33E-08	2	0	0
EPDR1	0.533361	0.63636	-0.103	-0.31745	3.28E-10	4.33E-08	2	0	0
PARVB	0.871819	0.812111	0.059708	0.105387	3.43E-10	4.48E-08	8	0	0
OSGIN2	0.312793	0.425137	-0.11234	-0.49566	3.98E-10	5.18E-08	12	0	0
RAB17	0.782358	0.874384	-0.09203	-0.15868	4.10E-10	5.29E-08	2	0	0
CPB1	0.07143	0.135542	-0.06411	-0.83781	4.22E-10	5.40E-08	1	0	0
OR6P1	0.500816	0.672679	-0.17186	-0.46926	4.33E-10	5.47E-08	6	0	0
ZSCAN1	0.368131	0.169854	0.198277	1.141317	4.36E-10	5.47E-08	7	0	0
PLXNB2	0.593306	0.482408	0.110898	0.292441	4.36E-10	5.47E-08	2	0	0
SOX17	0.325138	0.17617	0.148968	1.266955	4.55E-10	5.67E-08	10	0	0
LSP1	0.497966	0.637538	-0.13957	-0.39194	4.66E-10	5.76E-08	5	0	0
C15orf62	0.367076	0.512682	-0.14561	-0.50333	4.73E-10	5.81E-08	11	0	0
SLC22A10	0.7173	0.865374	-0.14807	-0.26735	4.84E-10	5.88E-08	1	0	0
OCSTAMP	0.558598	0.680647	-0.12205	-0.30671	4.85E-10	5.88E-08	8	0	0
HOXD3	0.306725	0.10174	0.204985	1.578133	4.97E-10	5.92E-08	7	0	0
CCDC140	0.347686	0.183355	0.164332	0.972004	4.96E-10	5.92E-08	9	0	0
ZNF132	0.321092	0.192467	0.128625	1.413577	4.98E-10	5.92E-08	9	0	0
HAS1	0.245966	0.163547	0.082419	0.565902	5.12E-10	6.00E-08	2	0	0
TNFSF10	0.287969	0.355271	-0.0673	-0.66305	5.47E-10	6.37E-08	4	0	0
GHSR	0.291105	0.138688	0.152417	1.280209	5.60E-10	6.48E-08	17	0	0
ARMC5	0.327038	0.395577	-0.06854	-0.42592	5.70E-10	6.56E-08	12	0	0
AFF3	0.429579	0.595489	-0.16591	-0.48279	5.74E-10	6.56E-08	4	0	0
OR5M3	0.695837	0.868469	-0.17263	-0.31566	5.81E-10	6.59E-08	1	0	0
OR3A2	0.637891	0.795456	-0.15757	-0.32732	5.90E-10	6.63E-08	5	0	0
OR7A5	0.665531	0.648518	0.017013	0.003692	5.91E-10	6.63E-08	3	0	0
ZNF529	0.266065	0.080423	0.185642	1.7837	6.15E-10	6.85E-08	13	0	0
HOXD8	0.286631	0.111159	0.175472	1.41806	6.34E-10	6.94E-08	16	0	0
GCSAM	0.417973	0.545512	-0.12754	-0.63837	6.29E-10	6.94E-08	5	0	0
ALG1L	0.53015	0.808882	-0.27873	-0.60029	6.35E-10	6.94E-08	1	0	0

SCRG1	0.751704	0.840266	-0.08856	-0.15877	6.39E-10	6.94E-08	2	0	0
KRTAP7-1	0.700751	0.845019	-0.14427	-0.27352	6.45E-10	6.97E-08	6	0	0
INSL4	0.725986	0.896219	-0.17023	-0.30018	6.64E-10	7.13E-08	1	0	0
PAX7	0.264386	0.10121	0.163177	1.299564	6.81E-10	7.15E-08	6	0	0
ANGPTL1	0.635967	0.446878	0.189089	0.508622	6.84E-10	7.15E-08	4	0	0
ANGPT2	0.658346	0.554797	0.103548	0.270511	6.78E-10	7.15E-08	7	0	0
OR51B6	0.515656	0.703415	-0.18776	-0.44062	6.86E-10	7.15E-08	1	0	0
FUT2	0.571065	0.686091	-0.11503	-0.29836	6.76E-10	7.15E-08	7	0	0
MUSTN1	0.720075	0.643871	0.076203	0.16646	7.09E-10	7.31E-08	9	0	0
NOS3	0.719869	0.794784	-0.07492	-0.15171	7.06E-10	7.31E-08	4	0	0
UBQLNL	0.622087	0.773697	-0.15161	-0.32271	7.47E-10	7.65E-08	5	0	0
NREP	0.668821	0.798141	-0.12932	-0.27874	7.68E-10	7.83E-08	4	0	0
MPEG1	0.612814	0.790592	-0.17778	-0.37567	7.73E-10	7.83E-08	5	0	0
NKAPL	0.512189	0.409976	0.102214	0.715624	7.86E-10	7.88E-08	12	0	0
OR4C3	0.506975	0.687022	-0.18005	-0.43111	7.83E-10	7.88E-08	1	0	0
UNC5D	0.452659	0.369765	0.082894	0.571473	8.02E-10	7.95E-08	12	0	0
A2ML1	0.674547	0.866308	-0.19176	-0.36045	8.01E-10	7.95E-08	4	0	0
ABLIM1	0.551765	0.438998	0.112767	0.362968	8.59E-10	8.47E-08	8	0	0
LILRA4	0.458503	0.581011	-0.12251	-0.36987	8.74E-10	8.56E-08	7	0	0
RASSF6	0.329334	0.401048	-0.07171	-0.46748	8.93E-10	8.71E-08	13	0	0
SH2B2	0.523596	0.634922	-0.11133	-0.26009	9.60E-10	9.30E-08	6	0	0
SPAM1	0.723795	0.853798	-0.13	-0.23995	1.00E-09	9.66E-08	4	0	0
C2orf40	0.647242	0.804294	-0.15705	-0.31588	1.02E-09	9.80E-08	3	0	0
OR8B4	0.653897	0.795537	-0.14164	-0.27937	1.03E-09	9.80E-08	4	0	0
UBQLN3	0.69634	0.829538	-0.1332	-0.25216	1.11E-09	1.05E-07	3	0	0
PRRX1	0.265716	0.128268	0.137448	1.169396	1.15E-09	1.07E-07	7	0	0
SH3TC1	0.612526	0.504697	0.107829	0.27441	1.14E-09	1.07E-07	1	0	0
KLHL31	0.70219	0.601141	0.101049	0.263704	1.15E-09	1.07E-07	10	0	0
LAIR2	0.33504	0.518929	-0.18389	-0.61824	1.14E-09	1.07E-07	2	0	0
KRT81	0.758628	0.699004	0.059625	0.138034	1.17E-09	1.08E-07	5	0	0
EVX1	0.342464	0.19898	0.143484	0.861568	1.21E-09	1.11E-07	18	0	0
TAC1	0.306287	0.12148	0.184807	1.24161	1.22E-09	1.12E-07	8	0	0
PATE2	0.627183	0.798145	-0.17096	-0.3535	1.26E-09	1.15E-07	3	0	0
SERPINB13	0.642644	0.811614	-0.16897	-0.33437	1.28E-09	1.16E-07	8	0	0
FANCD2OS	0.821272	0.724279	0.096993	0.188446	1.37E-09	1.23E-07	11	0	0
SPINT4	0.687572	0.891985	-0.20441	-0.37076	1.39E-09	1.24E-07	1	0	0
ABHD17C	0.466533	0.433964	0.032568	0.032571	1.40E-09	1.25E-07	3	0	0
SOX1	0.300721	0.193345	0.107376	0.69025	1.43E-09	1.27E-07	13	0	0
OR10J1	0.53291	0.738304	-0.20539	-0.45062	1.45E-09	1.28E-07	5	0	0
IQCJ-SCHII	0.472088	0.670751	-0.19866	-0.49678	1.46E-09	1.28E-07	4	0	0
IQCJ	0.472088	0.670751	-0.19866	-0.49678	1.46E-09	1.28E-07	4	0	0
TRIM15	0.531131	0.693011	-0.16188	-0.43254	1.51E-09	1.31E-07	14	0	0
OR2B11	0.604349	0.746718	-0.14237	-0.35107	1.53E-09	1.33E-07	7	0	0
OR4D9	0.642671	0.793448	-0.15078	-0.30367	1.55E-09	1.34E-07	3	0	0
AGXT2	0.649801	0.806426	-0.15662	-0.31869	1.58E-09	1.35E-07	8	0	0
GPR31	0.595142	0.729223	-0.13408	-0.31829	1.58E-09	1.35E-07	5	0	0
ATP10A	0.724085	0.829883	-0.1058	-0.20145	1.64E-09	1.39E-07	3	0	0
BPIFA1	0.557199	0.734931	-0.17773	-0.39613	1.65E-09	1.39E-07	6	0	0
UMOD	0.58465	0.778897	-0.19425	-0.41165	1.68E-09	1.41E-07	3	0	0
OR6Y1	0.748466	0.883824	-0.13536	-0.23691	1.77E-09	1.47E-07	1	0	0
WDFY1	0.461325	0.388988	0.072337	0.182368	1.76E-09	1.47E-07	7	0	0
GALNT8	0.532788	0.661638	-0.12885	-0.30255	1.79E-09	1.49E-07	7	0	0
FCER1A	0.62585	0.799031	-0.17318	-0.35131	1.82E-09	1.51E-07	7	0	0
NETO1	0.287045	0.141226	0.145819	0.997275	1.87E-09	1.54E-07	5	0	0
GPR62	0.545881	0.653767	-0.10789	-0.26097	1.90E-09	1.55E-07	6	0	0

OR52E2	0.604042	0.770337	-0.16629	-0.3454	1.89E-09	1.55E-07	2	0	0
NPLOC4	0.628392	0.502318	0.126073	0.366543	1.92E-09	1.56E-07	4	0	0
OR5H6	0.6047	0.775362	-0.17066	-0.36681	1.97E-09	1.59E-07	2	0	0
OR2M5	0.720061	0.883059	-0.163	-0.29612	2.01E-09	1.61E-07	5	0	0
SALL3	0.23038	0.098332	0.132048	1.220673	2.07E-09	1.65E-07	14	0	0
KRTAP20-2	0.657409	0.815046	-0.15764	-0.31582	2.07E-09	1.65E-07	5	0	0
TP53I13	0.179638	0.273799	-0.09416	-0.55516	2.10E-09	1.66E-07	3	0	0
SORCS3	0.19437	0.063984	0.130386	1.525946	2.13E-09	1.68E-07	10	0	0
ST7L	0.343882	0.47646	-0.13258	-0.5147	2.15E-09	1.68E-07	3	0	0
PATE1	0.598138	0.745255	-0.14712	-0.32122	2.15E-09	1.68E-07	6	0	0
RTN4	0.599214	0.43989	0.159323	0.434905	2.17E-09	1.69E-07	2	0	0
GNG4	0.369185	0.213823	0.155361	0.916616	2.18E-09	1.69E-07	11	0	0
REG3A	0.569308	0.739306	-0.17	-0.37182	2.20E-09	1.70E-07	5	0	0
OR9Q2	0.578712	0.715855	-0.13714	-0.30632	2.23E-09	1.71E-07	10	0	0
NRXN3	0.570317	0.710473	-0.14016	-0.30981	2.22E-09	1.71E-07	4	0	0
TMEM132D	0.346211	0.210589	0.135621	0.919313	2.26E-09	1.73E-07	6	0	0
PRDM14	0.2968	0.137883	0.158917	1.194078	2.30E-09	1.75E-07	8	0	0
TTYH1	0.247115	0.112915	0.1342	1.822111	2.35E-09	1.78E-07	10	0	0
SLC41A3	0.705408	0.628827	0.076581	0.167536	2.38E-09	1.79E-07	7	0	0
GRXCR1	0.579733	0.750368	-0.17064	-0.38049	2.40E-09	1.80E-07	6	0	0
TNK2	0.507893	0.694375	-0.18648	-0.44707	2.43E-09	1.81E-07	4	0	0
OR51B5	0.617382	0.773337	-0.15596	-0.33318	2.43E-09	1.81E-07	5	0	0
RP1	0.651263	0.797361	-0.1461	-0.3104	2.56E-09	1.90E-07	4	0	0
DBH	0.684498	0.78898	-0.10448	-0.20763	2.58E-09	1.90E-07	8	0	0
MSGN1	0.630507	0.796922	-0.16642	-0.34548	2.62E-09	1.91E-07	6	0	0
PRSS58	0.672874	0.861259	-0.18839	-0.35148	2.61E-09	1.91E-07	1	0	0
SCARF1	0.648099	0.551288	0.096811	0.259096	2.60E-09	1.91E-07	8	0	0
VAX1	0.239362	0.111002	0.128361	1.227255	2.72E-09	1.98E-07	11	0	0
KCNA2	0.374944	0.489146	-0.1142	-0.41535	2.75E-09	1.98E-07	5	0	0
PATE3	0.41864	0.573616	-0.15498	-0.43543	2.75E-09	1.98E-07	4	0	0
SIGLEC5	0.529481	0.653306	-0.12383	-0.33498	2.82E-09	2.01E-07	6	0	0
SIGLEC14	0.529481	0.653306	-0.12383	-0.33498	2.82E-09	2.01E-07	6	0	0
ANXA2R	0.445338	0.368116	0.077222	0.084454	2.93E-09	2.09E-07	7	0	0
RNF112	0.632383	0.632998	-0.00061	0.062651	2.94E-09	2.09E-07	8	0	0
OR6K2	0.722868	0.879535	-0.15667	-0.28488	2.97E-09	2.09E-07	6	0	0
OR4D10	0.670043	0.80541	-0.13537	-0.27751	2.99E-09	2.10E-07	5	0	0
NELL2	0.509771	0.691905	-0.18213	-0.4334	3.08E-09	2.15E-07	1	0	0
VSTM2B	0.228305	0.076016	0.152289	1.669838	3.07E-09	2.15E-07	3	0	0
HMGB4	0.625633	0.800545	-0.17491	-0.35694	3.14E-09	2.18E-07	4	0	0
CMA1	0.701152	0.837321	-0.13617	-0.25518	3.17E-09	2.19E-07	3	0	0
ZNF716	0.606888	0.770961	-0.16407	-0.34254	3.20E-09	2.21E-07	3	0	0
CDHR5	0.584924	0.449545	0.135378	0.634405	3.22E-09	2.21E-07	11	0	0
OR5J2	0.568008	0.745726	-0.17772	-0.38878	3.21E-09	2.21E-07	2	0	0
TFAP2B	0.32926	0.15466	0.1746	1.163142	3.27E-09	2.22E-07	5	0	0
DENND2A	0.392093	0.472083	-0.07999	-0.30578	3.26E-09	2.22E-07	4	0	0
PIEZO2	0.272297	0.143263	0.129034	0.99315	3.27E-09	2.22E-07	12	0	0
SFN	0.481119	0.611563	-0.13044	-0.34536	3.31E-09	2.22E-07	10	0	0
HOXA11	0.317231	0.19499	0.122242	0.727986	3.30E-09	2.22E-07	30	0	0
MNDA	0.532351	0.676948	-0.1446	-0.37403	3.37E-09	2.25E-07	7	0	0
KRTAP8-1	0.636432	0.800555	-0.16412	-0.3296	3.49E-09	2.32E-07	5	0	0
APCS	0.48548	0.682203	-0.19672	-0.48424	3.53E-09	2.34E-07	3	0	0
MS4A4A	0.55283	0.672186	-0.11936	-0.31945	3.63E-09	2.40E-07	8	0	0
SLC5A4	0.626649	0.75642	-0.12977	-0.27648	3.65E-09	2.41E-07	6	0	0
OR10G9	0.625283	0.800274	-0.17499	-0.35259	3.67E-09	2.41E-07	3	0	0
JRK	0.614413	0.705816	-0.0914	-0.20498	3.73E-09	2.44E-07	3	0	0

IL22RA2	0.792024	0.909969	-0.11794	-0.19844	3.81E-09	2.47E-07	3	0	0
CLDN15	0.752905	0.670853	0.082052	0.168382	3.80E-09	2.47E-07	10	0	0
C16orf78	0.637339	0.781684	-0.14435	-0.30156	3.82E-09	2.47E-07	7	0	0
BARHL2	0.316054	0.160697	0.155357	1.140742	3.90E-09	2.51E-07	11	0	0
OR5B12	0.684728	0.820882	-0.13615	-0.27026	3.89E-09	2.51E-07	6	0	0
KLK2	0.57827	0.719785	-0.14151	-0.31099	3.95E-09	2.53E-07	1	0	0
CASP14	0.643009	0.820689	-0.17768	-0.34701	3.96E-09	2.53E-07	5	0	0
COL17A1	0.671141	0.804991	-0.13385	-0.28776	4.09E-09	2.60E-07	5	0	0
ZNF709	0.568405	0.477403	0.091002	0.37942	4.10E-09	2.60E-07	9	0	0
EFCAB3	0.70893	0.889817	-0.18089	-0.32378	4.21E-09	2.66E-07	1	0	0
GREB1	0.574361	0.725087	-0.15073	-0.37464	4.34E-09	2.72E-07	7	0	0
SIRPB1	0.449611	0.582099	-0.13249	-0.39966	4.32E-09	2.72E-07	3	0	0
SLAMF6	0.311057	0.436469	-0.12541	-0.53472	4.37E-09	2.73E-07	3	0	0
ADM5	0.680644	0.591764	0.08888	0.189638	4.38E-09	2.73E-07	9	0	0
OR6N1	0.659124	0.820085	-0.16096	-0.31984	4.41E-09	2.73E-07	6	0	0
OR4D1	0.697024	0.840965	-0.14394	-0.27116	4.45E-09	2.75E-07	8	0	0
ZIC1	0.322681	0.167136	0.155545	0.923999	4.48E-09	2.76E-07	12	0	0
SLC28A3	0.67429	0.844171	-0.16988	-0.32819	4.52E-09	2.77E-07	4	0	0
HBE1	0.701428	0.835874	-0.13445	-0.25453	4.60E-09	2.81E-07	4	0	0
CD1D	0.556609	0.676945	-0.12034	-0.28548	4.67E-09	2.84E-07	8	0	0
CD96	0.750205	0.863938	-0.11373	-0.20002	4.74E-09	2.87E-07	2	0	0
CLDN18	0.713327	0.858876	-0.14555	-0.26906	4.76E-09	2.87E-07	11	0	0
TMEM132(	0.248092	0.107542	0.14055	1.289803	4.76E-09	2.87E-07	5	0	0
VPS53	0.715531	0.902941	-0.18741	-0.33148	4.93E-09	2.96E-07	1	0	0
PARP8	0.835972	0.747003	0.088969	0.171341	5.09E-09	3.04E-07	2	0	0
OR4D11	0.652756	0.839004	-0.18625	-0.35914	5.14E-09	3.06E-07	2	0	0
GLIPR1	0.410241	0.505161	-0.09492	-0.56403	5.19E-09	3.08E-07	8	0	0
MYL10	0.449544	0.575226	-0.12568	-0.4425	5.37E-09	3.18E-07	3	0	0
NFE2L3	0.278228	0.346402	-0.06817	-0.37011	5.44E-09	3.21E-07	5	0	0
FBP2	0.650436	0.681801	-0.03137	-0.10437	5.46E-09	3.21E-07	3	0	0
CTLA4	0.556885	0.741798	-0.18491	-0.43987	5.53E-09	3.24E-07	3	0	0
DDHD1	0.122627	0.175315	-0.05269	-0.38811	5.55E-09	3.24E-07	7	0	0
RNF148	0.691976	0.780551	-0.08857	-0.18447	5.68E-09	3.31E-07	4	0	0
RNF111	0.580371	0.431762	0.148609	0.477673	5.71E-09	3.31E-07	6	0	0
SEC23A	0.695879	0.857889	-0.16201	-0.29843	5.79E-09	3.35E-07	2	0	0
FCRL1	0.498058	0.657865	-0.15981	-0.46825	5.85E-09	3.37E-07	7	0	0
CD300LF	0.426557	0.538719	-0.11216	-0.36235	5.97E-09	3.43E-07	5	0	0
NXPE2	0.595128	0.741	-0.14587	-0.34658	6.00E-09	3.44E-07	7	0	0
LRRN3	0.706414	0.594885	0.111529	0.245699	6.38E-09	3.64E-07	4	0	0
SIRPG	0.579953	0.747823	-0.16787	-0.39376	6.39E-09	3.64E-07	6	0	0
REEP1	0.311344	0.375892	-0.06455	-0.31191	6.63E-09	3.77E-07	10	0	0
GAPVD1	0.081803	0.134068	-0.05226	-0.41517	6.68E-09	3.78E-07	5	0	0
OR5M8	0.65215	0.799169	-0.14702	-0.30126	6.75E-09	3.81E-07	4	0	0
FSIP2	0.42989	0.340947	0.088943	0.501394	6.85E-09	3.85E-07	10	0	0
KRTAP19-8	0.723232	0.856634	-0.1334	-0.25285	6.88E-09	3.85E-07	4	0	0
OR52A5	0.672629	0.791352	-0.11872	-0.24612	6.97E-09	3.88E-07	3	0	0
ALDH1A3	0.61911	0.767866	-0.14876	-0.30621	6.97E-09	3.88E-07	1	0	0
DQX1	0.729026	0.858959	-0.12993	-0.25571	7.13E-09	3.96E-07	10	0	0
NXPE4	0.673545	0.803951	-0.13041	-0.27932	7.19E-09	3.98E-07	5	0	0
MEI1	0.448938	0.313418	0.135519	1.000076	7.23E-09	3.99E-07	9	0	0
IP6K2	0.503472	0.363263	0.140208	0.487083	7.27E-09	4.00E-07	2	0	0
OR5I1	0.620819	0.79299	-0.17217	-0.35376	7.36E-09	4.04E-07	5	0	0
FUT7	0.42478	0.52339	-0.09861	-0.29571	7.43E-09	4.06E-07	3	0	0
IL6R	0.212639	0.285714	-0.07307	-0.45485	7.53E-09	4.10E-07	10	0	0
OR8B12	0.571698	0.737863	-0.16616	-0.36149	7.56E-09	4.11E-07	4	0	0

CABS1	0.760504	0.854839	-0.09434	-0.1675	7.66E-09	4.15E-07	3	0	0
PAX9	0.317478	0.155582	0.161897	1.131782	7.68E-09	4.15E-07	10	0	0
LAMA4	0.629401	0.51545	0.113951	0.310316	7.72E-09	4.16E-07	8	0	0
KRTAP20-3	0.721226	0.842739	-0.12151	-0.22268	7.91E-09	4.25E-07	5	0	0
MROH2B	0.68674	0.842423	-0.15568	-0.3004	7.99E-09	4.27E-07	6	0	0
HOXA2	0.363935	0.222815	0.14112	0.86678	8.00E-09	4.27E-07	20	0	0
ARL11	0.348947	0.417546	-0.0686	-0.45093	8.09E-09	4.30E-07	7	0	0
REG3G	0.667202	0.815191	-0.14799	-0.28064	8.28E-09	4.39E-07	6	0	0
ANKRD22	0.430208	0.543617	-0.11341	-0.39021	8.30E-09	4.39E-07	10	0	0
ACTR3C	0.227404	0.294103	-0.0667	-0.55871	8.41E-09	4.44E-07	8	0	0
GMNC	0.620252	0.780385	-0.16013	-0.36911	8.53E-09	4.49E-07	4	0	0
TBX15	0.424689	0.281886	0.142803	0.639801	8.58E-09	4.50E-07	12	0	0
OR10J5	0.603812	0.75645	-0.15264	-0.33351	8.95E-09	4.68E-07	4	0	0
TMEM88	0.781328	0.691457	0.08987	0.210121	9.00E-09	4.69E-07	19	0	0
GP2	0.641966	0.786027	-0.14406	-0.29606	9.12E-09	4.74E-07	9	0	0
OR8B8	0.666691	0.80112	-0.13443	-0.25858	9.19E-09	4.76E-07	8	0	0
OR1N1	0.632815	0.76017	-0.12736	-0.27986	9.43E-09	4.86E-07	5	0	0
OR51M1	0.565163	0.713569	-0.14841	-0.32681	9.42E-09	4.86E-07	2	0	0
NME8	0.77432	0.880663	-0.10634	-0.19164	9.60E-09	4.93E-07	5	0	0
SECISBP2	0.628318	0.766038	-0.13772	-0.28185	9.78E-09	5.01E-07	1	0	0
OR52E6	0.63567	0.770931	-0.13526	-0.27627	1.00E-08	5.11E-07	3	0	0
OR13G1	0.558212	0.747534	-0.18932	-0.42266	1.01E-08	5.17E-07	3	0	0
CACNG7	0.197167	0.055599	0.141568	1.671101	1.02E-08	5.18E-07	5	0	0
KRT72	0.557143	0.476144	0.080999	0.307841	1.04E-08	5.27E-07	7	0	0
FRMD6	0.631067	0.802619	-0.17155	-0.34146	1.04E-08	5.27E-07	6	0	0
CDX2	0.393656	0.257511	0.136144	0.680473	1.05E-08	5.30E-07	8	0	0
OR6N2	0.653478	0.814752	-0.16127	-0.3219	1.06E-08	5.33E-07	4	0	0
TAS2R60	0.763401	0.89134	-0.12794	-0.22547	1.06E-08	5.33E-07	4	0	0
OR10T2	0.546347	0.751181	-0.20483	-0.45229	1.08E-08	5.38E-07	3	0	0
SGCA	0.436851	0.520026	-0.08318	-0.38082	1.08E-08	5.38E-07	5	0	0
CCDC168	0.741128	0.897445	-0.15632	-0.27275	1.09E-08	5.42E-07	1	0	0
FUT9	0.26434	0.131585	0.132755	1.001804	1.10E-08	5.43E-07	9	0	0
ZNF839	0.077954	0.104619	-0.02666	-0.33467	1.11E-08	5.47E-07	5	0	0
OR1L1	0.617356	0.755443	-0.13809	-0.32257	1.14E-08	5.61E-07	3	0	0
PCYOX1	0.331685	0.446582	-0.1149	-0.45876	1.19E-08	5.81E-07	4	0	0
LYPD1	0.409727	0.528039	-0.11831	-0.55053	1.19E-08	5.81E-07	8	0	0
IRX4	0.274454	0.137606	0.136848	1.147906	1.18E-08	5.81E-07	16	0	0
SLC23A1	0.679887	0.811379	-0.13149	-0.25417	1.19E-08	5.81E-07	2	0	0
OR10AG1	0.554496	0.718399	-0.1639	-0.36856	1.22E-08	5.94E-07	4	0	0
SULT1C4	0.459318	0.333685	0.125632	0.612955	1.23E-08	5.95E-07	9	0	0
LY6K	0.426614	0.554605	-0.12799	-0.4438	1.25E-08	6.03E-07	13	0	0
OR2G2	0.74112	0.886011	-0.14489	-0.26144	1.25E-08	6.03E-07	3	0	0
DNASE1L3	0.499992	0.601671	-0.10168	-0.35247	1.26E-08	6.07E-07	5	0	0
C9orf84	0.683762	0.871155	-0.18739	-0.34652	1.27E-08	6.09E-07	7	0	0
SLC12A7	0.247333	0.331479	-0.08415	-0.20585	1.29E-08	6.14E-07	11	0	0
TEX26	0.612348	0.501991	0.110357	0.318875	1.29E-08	6.14E-07	11	0	0
MYL1	0.509611	0.657133	-0.14752	-0.38761	1.32E-08	6.24E-07	4	0	0
ALOX5AP	0.549911	0.667491	-0.11758	-0.29088	1.34E-08	6.34E-07	7	0	0
OLFML1	0.657944	0.560986	0.096958	0.247457	1.38E-08	6.47E-07	6	0	0
LILRA1	0.663049	0.814368	-0.15132	-0.29593	1.37E-08	6.47E-07	2	0	0
OR9A2	0.672758	0.815847	-0.14309	-0.27621	1.39E-08	6.51E-07	5	0	0
EGFL8	0.834026	0.776154	0.057872	0.110374	1.40E-08	6.56E-07	4	0	0
KIF4B	0.516961	0.64874	-0.13178	-0.31611	1.41E-08	6.57E-07	5	0	0
LCE1F	0.432821	0.544169	-0.11135	-0.30687	1.43E-08	6.68E-07	3	0	0
LGSN	0.71736	0.870741	-0.15338	-0.28306	1.45E-08	6.74E-07	4	0	0

POM121L1	0.717437	0.802957	-0.08552	-0.16035	1.48E-08	6.84E-07	5	0	0
OR6F1	0.692399	0.852841	-0.16044	-0.30471	1.50E-08	6.95E-07	4	0	0
C1orf189	0.80791	0.733233	0.074677	0.141945	1.52E-08	7.03E-07	4	0	0
OR4D6	0.627204	0.77802	-0.15082	-0.32491	1.56E-08	7.16E-07	4	0	0
PTGFR	0.588239	0.722717	-0.13448	-0.35747	1.57E-08	7.19E-07	3	0	0
OR11L1	0.705328	0.818726	-0.1134	-0.21803	1.57E-08	7.19E-07	7	0	0
CHRNA1	0.73871	0.84404	-0.10533	-0.19711	1.58E-08	7.19E-07	7	0	0
SLC47A2	0.673863	0.803913	-0.13005	-0.2516	1.63E-08	7.43E-07	4	0	0
ELAVL4	0.421935	0.317942	0.103993	0.83528	1.66E-08	7.55E-07	11	0	0
GATA2	0.339976	0.207329	0.132647	1.055218	1.67E-08	7.55E-07	8	0	0
MT1A	0.483645	0.450748	0.032897	0.531998	1.68E-08	7.57E-07	8	0	0
KRT36	0.378395	0.330243	0.048152	0.426209	1.68E-08	7.57E-07	4	0	0
EFNA4	0.103425	0.150638	-0.04721	-0.37793	1.71E-08	7.65E-07	16	0	0
EFNA3	0.103425	0.150638	-0.04721	-0.37793	1.71E-08	7.65E-07	16	0	0
TREML2	0.455948	0.575057	-0.11911	-0.32552	1.72E-08	7.68E-07	6	0	0
APLNR	0.504757	0.583888	-0.07913	-0.1773	1.72E-08	7.68E-07	4	0	0
WFDC6	0.518883	0.674784	-0.1559	-0.34852	1.77E-08	7.86E-07	7	0	0
OR5AC2	0.667349	0.815691	-0.14834	-0.28524	1.79E-08	7.91E-07	3	0	0
OR2M4	0.709619	0.86219	-0.15257	-0.28292	1.82E-08	8.03E-07	3	0	0
TMEM184/	0.663402	0.640254	0.023147	0.08505	1.86E-08	8.19E-07	2	0	0
RTKN	0.445821	0.563348	-0.11753	-0.38994	1.88E-08	8.21E-07	8	0	0
SORL1	0.19616	0.25316	-0.057	-0.24587	1.87E-08	8.21E-07	7	0	0
KRTAP4-6	0.47696	0.641347	-0.16439	-0.41962	1.88E-08	8.21E-07	1	0	0
FAM228A	0.734163	0.691145	0.043018	0.231428	1.89E-08	8.22E-07	7	0	0
ZNF677	0.222584	0.101386	0.121197	1.169407	1.89E-08	8.22E-07	8	0	0
DNHD1	0.755246	0.672589	0.082658	0.182172	1.93E-08	8.37E-07	10	0	0
KRTAP13-2	0.625963	0.767761	-0.1418	-0.29152	1.93E-08	8.38E-07	3	0	0
TET2	0.238735	0.328745	-0.09001	-0.41248	1.95E-08	8.42E-07	7	0	0
FSHB	0.50288	0.647048	-0.14417	-0.35546	1.96E-08	8.44E-07	5	0	0
CCDC141	0.471794	0.413333	0.058461	0.647647	1.98E-08	8.50E-07	7	0	0
SP140	0.630994	0.765108	-0.13411	-0.28949	1.98E-08	8.50E-07	4	0	0
OR51T1	0.576029	0.705258	-0.12923	-0.29007	1.98E-08	8.50E-07	6	0	0
MDGA2	0.255734	0.133171	0.122563	0.933329	2.00E-08	8.53E-07	10	0	0
MTUS2	0.651013	0.793196	-0.14218	-0.28698	2.01E-08	8.56E-07	7	0	0
KRTAP6-3	0.717105	0.806158	-0.08905	-0.17302	2.03E-08	8.62E-07	8	0	0
OR2M7	0.622069	0.843228	-0.22116	-0.43285	2.12E-08	8.92E-07	1	0	0
PCDHGA5	0.390439	0.271736	0.118703	0.584892	2.12E-08	8.92E-07	12	0	0
FSCB	0.642786	0.780694	-0.13791	-0.27591	2.11E-08	8.92E-07	4	0	0
KRTAP21-1	0.652903	0.803894	-0.15099	-0.30776	2.11E-08	8.92E-07	4	0	0
PAH	0.214332	0.079904	0.134427	1.514411	2.13E-08	8.94E-07	10	0	0
SIGLEC12	0.446094	0.56957	-0.12348	-0.36844	2.14E-08	8.96E-07	11	0	0
ELMO2	0.583554	0.745655	-0.1621	-0.3505	2.16E-08	9.02E-07	3	0	0
MAMSTR	0.535415	0.606405	-0.07099	-0.30773	2.19E-08	9.12E-07	9	0	0
SPARC	0.447679	0.403874	0.043805	0.627122	2.26E-08	9.41E-07	9	0	0
NMRAL1	0.736411	0.853367	-0.11696	-0.21174	2.27E-08	9.41E-07	3	0	0
SIX6	0.245773	0.116081	0.129692	1.122399	2.29E-08	9.51E-07	11	0	0
ZFP30	0.258693	0.138059	0.120634	1.536037	2.32E-08	9.56E-07	7	0	0
ZNF781	0.258693	0.138059	0.120634	1.536037	2.32E-08	9.56E-07	7	0	0
MYL4	0.526228	0.712254	-0.18603	-0.42966	2.33E-08	9.57E-07	1	0	0
REG1A	0.7109	0.886142	-0.17524	-0.31393	2.36E-08	9.69E-07	1	0	0
CPA1	0.677118	0.593132	0.083986	0.212879	2.37E-08	9.72E-07	6	0	0
KCNU1	0.726757	0.833891	-0.10713	-0.20702	2.39E-08	9.73E-07	7	0	0
LZTS2	0.196108	0.259785	-0.06368	-0.31409	2.38E-08	9.73E-07	9	0	0
SLC16A10	0.206294	0.209879	-0.00359	-0.23596	2.41E-08	9.80E-07	9	0	0
COLEC11	0.70633	0.637938	0.068392	0.205812	2.45E-08	9.90E-07	15	0	0

TMEM212	0.793146	0.719107	0.074039	0.172866	2.45E-08	9.90E-07	8	0	0
STAT6	0.563219	0.481441	0.081778	0.247039	2.45E-08	9.90E-07	2	0	0
UGT1A6	0.559608	0.694203	-0.13459	-0.31869	2.58E-08	1.04E-06	5	0	0
OR5B21	0.761874	0.869113	-0.10724	-0.19594	2.62E-08	1.05E-06	3	0	0
CYP11B2	0.612115	0.758534	-0.14642	-0.31155	2.63E-08	1.06E-06	6	0	0
BPIFB4	0.620417	0.771335	-0.15092	-0.30964	2.64E-08	1.06E-06	1	0	0
MAMDC2	0.543268	0.43671	0.106558	0.423049	2.69E-08	1.08E-06	7	0	0
TSPAN32	0.401785	0.506351	-0.10457	-0.34874	2.70E-08	1.08E-06	27	0	0
COX7A2L	0.771411	0.739225	0.032186	0.069807	2.72E-08	1.08E-06	5	0	0
TMEM216	0.650552	0.783099	-0.13255	-0.35616	2.75E-08	1.09E-06	9	0	0
APOBEC1	0.595419	0.756867	-0.16145	-0.32986	2.75E-08	1.09E-06	5	0	0
CGB8	0.652885	0.820019	-0.16713	-0.32438	2.74E-08	1.09E-06	1	0	0
CST1	0.815259	0.906745	-0.09149	-0.15167	2.74E-08	1.09E-06	1	0	0
CCDC70	0.698166	0.797971	-0.09981	-0.2018	2.79E-08	1.10E-06	6	0	0
LEFTY2	0.575084	0.483112	0.091972	0.247063	2.80E-08	1.10E-06	3	0	0
CARD18	0.692811	0.835501	-0.14269	-0.2836	2.81E-08	1.10E-06	6	0	0
HOXD13	0.267306	0.126582	0.140724	1.141855	2.83E-08	1.11E-06	11	0	0
PI3	0.505988	0.645729	-0.13974	-0.34043	2.84E-08	1.11E-06	5	0	0
DEFB119	0.40556	0.540891	-0.13533	-0.39345	2.85E-08	1.11E-06	3	0	0
LEP	0.549903	0.450146	0.099757	0.364635	2.87E-08	1.11E-06	10	0	0
SRBD1	0.630316	0.799265	-0.16895	-0.33783	2.87E-08	1.11E-06	1	0	0
SARDH	0.691275	0.804991	-0.11372	-0.21834	2.91E-08	1.12E-06	5	0	0
SLC35E2B	0.509986	0.650423	-0.14044	-0.34437	3.01E-08	1.16E-06	2	0	0
OR4A16	0.493613	0.638563	-0.14495	-0.33206	3.00E-08	1.16E-06	4	0	0
OR51A7	0.645277	0.786754	-0.14148	-0.29127	3.02E-08	1.16E-06	5	0	0
NRIP2	0.463446	0.294399	0.169047	0.875541	3.04E-08	1.16E-06	8	0	0
DNAJB8	0.620929	0.731303	-0.11037	-0.25263	3.05E-08	1.17E-06	7	0	0
NPTX2	0.420464	0.317105	0.103359	0.775663	3.06E-08	1.17E-06	5	0	0
FCN3	0.754347	0.697198	0.057149	0.115619	3.18E-08	1.21E-06	7	0	0
FETUB	0.678072	0.856128	-0.17806	-0.33617	3.24E-08	1.22E-06	2	0	0
TRIQK	0.707245	0.832035	-0.12479	-0.23428	3.23E-08	1.22E-06	5	0	0
ALX4	0.248558	0.151811	0.096747	0.754842	3.24E-08	1.22E-06	38	0	0
LILRB2	0.567866	0.705672	-0.13781	-0.31186	3.22E-08	1.22E-06	4	0	0
OR2AP1	0.702785	0.843	-0.14022	-0.26334	3.29E-08	1.24E-06	2	0	0
KRTAP12-2	0.615521	0.727802	-0.11228	-0.24091	3.28E-08	1.24E-06	3	0	0
PAX6	0.286518	0.171753	0.114765	0.914453	3.30E-08	1.24E-06	11	0	0
FAM92B	0.684245	0.769122	-0.08488	-0.17625	3.32E-08	1.24E-06	8	0	0
ZNF560	0.32452	0.206251	0.118269	0.763129	3.32E-08	1.24E-06	14	0	0
CCNB1	0.190802	0.227472	-0.03667	-0.45392	3.38E-08	1.26E-06	7	0	0
TARM1	0.590867	0.713206	-0.12234	-0.29035	3.38E-08	1.26E-06	5	0	0
CXCL5	0.626481	0.578034	0.048447	0.394339	3.39E-08	1.26E-06	5	0	0
OR56A3	0.51009	0.639917	-0.12983	-0.31766	3.42E-08	1.26E-06	3	0	0
CD300LD	0.542988	0.661342	-0.11835	-0.31227	3.44E-08	1.27E-06	6	0	0
KRTAP22-1	0.68287	0.803177	-0.12031	-0.23768	3.49E-08	1.29E-06	4	0	0
BGLAP	0.59556	0.684117	-0.08856	-0.23642	3.53E-08	1.30E-06	6	0	0
NPAP1	0.700362	0.823993	-0.12363	-0.24394	3.56E-08	1.31E-06	8	0	0
TACC1	0.472265	0.47926	-0.007	0.56957	3.58E-08	1.31E-06	9	0	0
DEFB118	0.477524	0.633933	-0.15641	-0.40496	3.59E-08	1.31E-06	6	0	0
IZUMO3	0.663298	0.793456	-0.13016	-0.23142	3.61E-08	1.31E-06	4	0	0
KRTAP6-2	0.717358	0.831256	-0.1139	-0.22075	3.61E-08	1.31E-06	7	0	0
TMC3	0.712854	0.859713	-0.14686	-0.26773	3.62E-08	1.31E-06	3	0	0
PKP3	0.675997	0.791211	-0.11521	-0.23105	3.66E-08	1.33E-06	5	0	0
FCAR	0.601021	0.748993	-0.14797	-0.31436	3.71E-08	1.34E-06	3	0	0
PLEKHB1	0.601781	0.506361	0.095421	0.281755	3.77E-08	1.36E-06	9	0	0
BPIFC	0.645029	0.801757	-0.15673	-0.32021	3.80E-08	1.37E-06	3	0	0

TBX20	0.285117	0.150353	0.134764	1.005823	3.83E-08	1.37E-06	12	0	0
IL18BP	0.699119	0.656177	0.042942	0.110992	3.87E-08	1.39E-06	10	0	0
TSHZ3	0.140551	0.080881	0.05967	0.81893	3.88E-08	1.39E-06	7	0	0
FERD3L	0.211144	0.103643	0.1075	0.949319	3.91E-08	1.40E-06	8	0	0
KANSL3	0.453747	0.330549	0.123198	0.456683	4.00E-08	1.43E-06	3	0	0
SLC2A11	0.263693	0.386682	-0.12299	-0.52877	4.01E-08	1.43E-06	4	0	0
TF	0.473116	0.383076	0.09004	0.551664	4.06E-08	1.44E-06	12	0	0
KRTAP21-2	0.576322	0.733368	-0.15705	-0.34065	4.08E-08	1.45E-06	3	0	0
ROPN1	0.741674	0.898149	-0.15648	-0.27282	4.09E-08	1.45E-06	1	0	0
C18orf63	0.74456	0.823885	-0.07932	-0.14683	4.12E-08	1.45E-06	3	0	0
NDUFAF3	0.172107	0.241497	-0.06939	-0.40034	4.22E-08	1.48E-06	14	0	0
STIM1	0.199823	0.276251	-0.07643	-0.38912	4.21E-08	1.48E-06	6	0	0
CNTD2	0.276559	0.360834	-0.08428	-0.4021	4.28E-08	1.50E-06	13	0	0
ADCY4	0.402088	0.2815	0.120588	1.097713	4.33E-08	1.52E-06	10	0	0
CA1	0.605943	0.722696	-0.11675	-0.2505	4.36E-08	1.52E-06	4	0	0
CD1C	0.510271	0.677594	-0.16732	-0.40851	4.44E-08	1.54E-06	5	0	0
HORMAD2	0.538416	0.409886	0.12853	0.438145	4.57E-08	1.59E-06	13	0	0
EVI2B	0.397662	0.562515	-0.16485	-0.52933	4.63E-08	1.61E-06	4	0	0
MACC1	0.213548	0.311951	-0.0984	-0.55346	4.68E-08	1.62E-06	6	0	0
TRAF1	0.233409	0.290026	-0.05662	-0.31421	4.70E-08	1.62E-06	7	0	0
OR8J1	0.593248	0.744418	-0.15117	-0.32261	4.69E-08	1.62E-06	2	0	0
ZNF135	0.432981	0.306154	0.126827	0.807192	4.69E-08	1.62E-06	13	0	0
MCFD2	0.060873	0.095827	-0.03495	-0.50346	4.74E-08	1.63E-06	10	0	0
OR56A1	0.632299	0.787468	-0.15517	-0.32441	4.75E-08	1.63E-06	2	0	0
DKK4	0.486963	0.601629	-0.11467	-0.35905	4.77E-08	1.63E-06	10	0	0
THRA	0.766984	0.701362	0.065622	0.155748	4.78E-08	1.63E-06	2	0	0
MTRNR2L1	0.546468	0.673246	-0.12678	-0.29611	4.80E-08	1.64E-06	1	0	0
KRTAP12-1	0.756627	0.857847	-0.10122	-0.1786	4.81E-08	1.64E-06	3	0	0
ADAMTS2(	0.420904	0.310945	0.109959	0.860898	4.86E-08	1.65E-06	7	0	0
HOXA10	0.428066	0.335059	0.093007	0.436245	4.96E-08	1.68E-06	8	0	0
B3GALT1	0.71418	0.835157	-0.12098	-0.23704	5.01E-08	1.69E-06	3	0	0
PRB4	0.742221	0.868166	-0.12594	-0.23166	5.07E-08	1.71E-06	4	0	0
FGD5	0.692945	0.814	-0.12105	-0.2294	5.12E-08	1.72E-06	4	0	0
FOXB2	0.176474	0.083834	0.092641	1.071788	5.13E-08	1.72E-06	6	0	0
KRTAP10-8	0.678804	0.78825	-0.10945	-0.20986	5.13E-08	1.72E-06	4	0	0
PAMR1	0.345508	0.477442	-0.13193	-0.51223	5.19E-08	1.74E-06	2	0	0
GLRX5	0.328047	0.388814	-0.06077	-0.41231	5.23E-08	1.75E-06	3	0	0
OR14C36	0.600633	0.764399	-0.16377	-0.35193	5.29E-08	1.75E-06	3	0	0
BHMT2	0.5976	0.503625	0.093974	0.370906	5.26E-08	1.75E-06	10	0	0
TCF21	0.304382	0.203534	0.100847	0.635692	5.29E-08	1.75E-06	11	0	0
IL2RA	0.458336	0.578423	-0.12009	-0.37785	5.29E-08	1.75E-06	7	0	0
MS4A1	0.581644	0.721031	-0.13939	-0.31179	5.30E-08	1.75E-06	3	0	0
BPIFB6	0.613562	0.75403	-0.14047	-0.29578	5.28E-08	1.75E-06	6	0	0
GPHB5	0.686473	0.788089	-0.10162	-0.20928	5.33E-08	1.76E-06	5	0	0
PEAR1	0.253765	0.136389	0.117376	1.064656	5.36E-08	1.77E-06	9	0	0
SMPD4	0.122225	0.177143	-0.05492	-0.37685	5.42E-08	1.78E-06	4	0	0
IL17A	0.67402	0.824282	-0.15026	-0.29775	5.41E-08	1.78E-06	5	0	0
ME3	0.413604	0.316948	0.096656	1.102565	5.45E-08	1.79E-06	19	0	0
ADORA3	0.831873	0.901434	-0.06956	-0.11716	5.57E-08	1.82E-06	3	0	0
KRTAP4-11	0.643449	0.724596	-0.08115	-0.17212	5.62E-08	1.84E-06	2	0	0
CLVS1	0.736953	0.672603	0.06435	0.199583	5.63E-08	1.84E-06	2	0	0
BPIFA2	0.659051	0.853794	-0.19474	-0.36857	5.64E-08	1.84E-06	1	0	0
TRH	0.562905	0.460893	0.102012	0.879011	5.76E-08	1.86E-06	13	0	0
NLRP5	0.624731	0.742466	-0.11773	-0.24883	5.75E-08	1.86E-06	2	0	0
CBLN4	0.338609	0.276961	0.061648	0.588302	5.74E-08	1.86E-06	11	0	0

COX7B2	0.764287	0.887906	-0.12362	-0.22126	5.77E-08	1.87E-06	8	0	0
ZNF671	0.371254	0.245663	0.125591	1.525481	5.79E-08	1.87E-06	13	0	0
OR5M1	0.62101	0.780625	-0.15962	-0.33446	5.93E-08	1.91E-06	4	0	0
ZNF729	0.504209	0.570177	-0.06597	0.013353	5.96E-08	1.91E-06	6	0	0
RNASE10	0.710909	0.604407	0.106502	0.239985	5.99E-08	1.92E-06	2	0	0
CDO1	0.391594	0.280567	0.111027	1.22977	6.03E-08	1.93E-06	14	0	0
ANGEL1	0.757287	0.860316	-0.10303	-0.18908	6.08E-08	1.95E-06	5	0	0
OMD	0.716767	0.664804	0.051963	0.116203	6.18E-08	1.97E-06	3	0	0
CEACAM4	0.372692	0.4909	-0.11821	-0.38907	6.18E-08	1.97E-06	4	0	0
TMED6	0.820181	0.750876	0.069304	0.136574	6.20E-08	1.97E-06	6	0	0
OR52J3	0.669653	0.813452	-0.1438	-0.27957	6.34E-08	2.01E-06	3	0	0
FAM166B	0.61132	0.508052	0.103267	0.351806	6.42E-08	2.04E-06	8	0	0
NMUR2	0.545913	0.690974	-0.14506	-0.34579	6.51E-08	2.06E-06	5	0	0
OPCML	0.296219	0.278423	0.017797	0.328991	6.60E-08	2.08E-06	13	0	0
BBC3	0.354742	0.426006	-0.07126	-0.41499	6.61E-08	2.08E-06	5	0	0
CABP5	0.575941	0.699761	-0.12382	-0.27927	6.62E-08	2.08E-06	6	0	0
MS4A3	0.53264	0.663624	-0.13098	-0.32146	6.65E-08	2.09E-06	9	0	0
HTR4	0.644625	0.774	-0.12938	-0.28387	6.71E-08	2.10E-06	3	0	0
TMEM176A	0.166513	0.093333	0.07318	0.954386	6.71E-08	2.10E-06	11	0	0
SIM2	0.185083	0.10285	0.082234	0.814447	6.72E-08	2.10E-06	18	0	0
CSGALNAC	0.332202	0.432942	-0.10074	-0.29907	6.76E-08	2.10E-06	5	0	0
IL11RA	0.763492	0.705639	0.057853	0.153214	6.76E-08	2.10E-06	2	0	0
FHL5	0.591787	0.694331	-0.10254	-0.14467	6.78E-08	2.11E-06	5	0	0
OR10K1	0.407366	0.619168	-0.2118	-0.59213	6.85E-08	2.12E-06	1	0	0
TRIM49D1	0.686877	0.856498	-0.16962	-0.31429	6.96E-08	2.15E-06	1	0	0
NPS	0.655367	0.80185	-0.14648	-0.29779	7.01E-08	2.17E-06	4	0	0
ZNF217	0.430183	0.543917	-0.11373	-0.33758	7.11E-08	2.19E-06	7	0	0
SLC25A2	0.754916	0.691199	0.063717	0.152722	7.18E-08	2.21E-06	12	0	0
MAP3K9	0.133601	0.171484	-0.03788	-0.28997	7.17E-08	2.21E-06	11	0	0
MUC1	0.106788	0.173937	-0.06715	-0.35146	7.20E-08	2.21E-06	9	0	0
OR5AP2	0.712081	0.818884	-0.1068	-0.1985	7.31E-08	2.24E-06	5	0	0
OR5AU1	0.487506	0.632979	-0.14547	-0.37701	7.31E-08	2.24E-06	6	0	0
FAM163B	0.508766	0.597531	-0.08877	-0.25178	7.34E-08	2.24E-06	3	0	0
DCK	0.461384	0.596965	-0.13558	-0.36288	7.39E-08	2.25E-06	2	0	0
PEX10	0.39459	0.496292	-0.1017	-0.30934	7.45E-08	2.27E-06	12	0	0
HCLS1	0.580127	0.708111	-0.12798	-0.30436	7.47E-08	2.27E-06	9	0	0
LGALS13	0.741345	0.850807	-0.10946	-0.19979	7.47E-08	2.27E-06	3	0	0
IL36A	0.759882	0.844661	-0.08478	-0.16006	7.49E-08	2.27E-06	6	0	0
NIPSNAP1	0.268186	0.345012	-0.07683	-0.32533	7.61E-08	2.30E-06	9	0	0
HYPK	0.790318	0.729062	0.061256	0.130543	7.63E-08	2.30E-06	4	0	0
ABCA12	0.68498	0.814233	-0.12925	-0.26585	7.65E-08	2.30E-06	4	0	0
ZKSCAN2	0.086574	0.119527	-0.03295	-0.12286	7.81E-08	2.34E-06	3	0	0
CCL11	0.625306	0.758841	-0.13353	-0.27792	7.81E-08	2.34E-06	6	0	0
CCDC129	0.516379	0.667838	-0.15146	-0.36729	7.88E-08	2.36E-06	4	0	0
CRP	0.583215	0.747311	-0.1641	-0.35862	7.90E-08	2.36E-06	4	0	0
HOXD11	0.376194	0.207882	0.168312	0.863719	7.92E-08	2.36E-06	3	0	0
TPSAB1	0.562555	0.66434	-0.10179	-0.23606	7.94E-08	2.36E-06	1	0	0
RTL1	0.683608	0.797759	-0.11415	-0.22237	7.95E-08	2.36E-06	8	0	0
PCDH20	0.756561	0.864481	-0.10792	-0.19705	7.98E-08	2.37E-06	3	0	0
FAM213A	0.281748	0.340486	-0.05874	-0.31619	8.00E-08	2.37E-06	7	0	0
LAMC2	0.378642	0.459834	-0.08119	-0.36885	8.03E-08	2.37E-06	12	0	0
FAM118A	0.574822	0.667975	-0.09315	-0.31362	8.05E-08	2.38E-06	11	0	0
USP30	0.699804	0.852319	-0.15251	-0.28964	8.08E-08	2.38E-06	3	0	0
MRGPRG	0.594957	0.679936	-0.08498	-0.19599	8.15E-08	2.40E-06	7	0	0
DGKI	0.322883	0.227385	0.095498	0.830979	8.20E-08	2.41E-06	8	0	0

SPPL2C	0.58784	0.707979	-0.12014	-0.26829	8.32E-08	2.44E-06	11	0	0
DEFB121	0.520137	0.649823	-0.12969	-0.36275	8.44E-08	2.47E-06	6	0	0
CYP11A1	0.66618	0.750524	-0.08434	-0.1797	8.47E-08	2.48E-06	12	0	0
OR2M3	0.525756	0.684396	-0.15864	-0.37123	8.63E-08	2.52E-06	4	0	0
PTP4A2	0.438953	0.30818	0.130774	1.48831	8.77E-08	2.55E-06	3	0	0
XAF1	0.538506	0.45849	0.080017	0.237019	8.76E-08	2.55E-06	5	0	0
OR2A25	0.646955	0.797353	-0.1504	-0.2974	8.79E-08	2.55E-06	1	0	0
CCDC179	0.805138	0.883888	-0.07875	-0.13682	8.87E-08	2.57E-06	7	0	0
EHBP1	0.78039	0.690679	0.089711	0.193729	8.94E-08	2.59E-06	6	0	0
OR8J3	0.584006	0.722258	-0.13825	-0.31988	8.96E-08	2.59E-06	4	0	0
ZFAND1	0.251285	0.345522	-0.09424	-0.4325	9.06E-08	2.61E-06	3	0	0
SET	0.305368	0.354584	-0.04922	-0.35348	9.17E-08	2.64E-06	3	0	0
PP2D1	0.589599	0.662475	-0.07288	-0.15004	9.22E-08	2.65E-06	2	0	0
ADAMTS16	0.301536	0.18392	0.117616	1.035819	9.26E-08	2.66E-06	11	0	0
SUB1	0.46506	0.644317	-0.17926	-0.46319	9.40E-08	2.69E-06	4	0	0
SPATA19	0.639555	0.776687	-0.13713	-0.27735	9.41E-08	2.69E-06	7	0	0
RLN1	0.442641	0.575879	-0.13324	-0.37224	9.55E-08	2.73E-06	1	0	0
IL37	0.692242	0.815618	-0.12338	-0.24089	9.65E-08	2.75E-06	4	0	0
SYT12	0.714636	0.829525	-0.11489	-0.22856	9.65E-08	2.75E-06	6	0	0
TRIM58	0.450185	0.356288	0.093897	1.029904	9.68E-08	2.75E-06	10	0	0
EVPL	0.56957	0.647472	-0.0779	-0.19342	9.70E-08	2.75E-06	5	0	0
NLRP4	0.646133	0.771991	-0.12586	-0.2706	9.67E-08	2.75E-06	9	0	0
ACCSL	0.620939	0.748461	-0.12752	-0.26752	9.93E-08	2.81E-06	4	0	0
ZBTB8A	0.548989	0.625713	-0.07672	-0.1509	1.00E-07	2.83E-06	5	0	0
GDF2	0.672939	0.621965	0.050975	0.139152	1.01E-07	2.84E-06	13	0	0
FAM83G	0.325482	0.360671	-0.03519	-0.27123	1.01E-07	2.85E-06	12	0	0
CD1E	0.540079	0.670982	-0.1309	-0.35518	1.02E-07	2.87E-06	5	0	0
L1TD1	0.491774	0.389848	0.101926	0.599734	1.03E-07	2.88E-06	11	0	0
SORBS2	0.759329	0.83863	-0.0793	-0.14693	1.03E-07	2.88E-06	5	0	0
UNC93A	0.770236	0.925517	-0.15528	-0.26185	1.03E-07	2.88E-06	1	0	0
TBC1D21	0.528602	0.64814	-0.11954	-0.30971	1.03E-07	2.88E-06	8	0	0
BATF	0.404421	0.541019	-0.1366	-0.42906	1.04E-07	2.89E-06	11	0	0
SOX11	0.249549	0.144127	0.105423	0.775471	1.04E-07	2.90E-06	10	0	0
SLC24A2	0.468988	0.484726	-0.01574	0.316433	1.05E-07	2.91E-06	4	0	0
OR8D1	0.794154	0.904731	-0.11058	-0.1861	1.06E-07	2.94E-06	2	0	0
PACRGL	0.870148	0.901113	-0.03096	-0.04988	1.07E-07	2.96E-06	1	0	0
OR56B1	0.600124	0.775437	-0.17531	-0.36439	1.07E-07	2.96E-06	1	0	0
HOXB2	0.526452	0.43497	0.091482	0.266601	1.07E-07	2.96E-06	5	0	0
HSBP1L1	0.3988	0.451813	-0.05301	-0.44452	1.09E-07	3.01E-06	11	0	0
XIRP2	0.666143	0.778365	-0.11222	-0.24226	1.10E-07	3.04E-06	5	0	0
KRTAP22-2	0.634577	0.728028	-0.09345	-0.20241	1.11E-07	3.05E-06	4	0	0
CMTM2	0.438123	0.30531	0.132813	0.709255	1.12E-07	3.07E-06	12	0	0
KLHL33	0.697105	0.588858	0.108246	0.277923	1.13E-07	3.10E-06	10	0	0
CXCR1	0.528195	0.622222	-0.09403	-0.3547	1.13E-07	3.10E-06	5	0	0
MYT1L	0.599646	0.733652	-0.13401	-0.30294	1.13E-07	3.10E-06	6	0	0
TNF	0.420411	0.527992	-0.10758	-0.35977	1.15E-07	3.13E-06	17	0	0
TEK	0.557177	0.455543	0.101635	0.263177	1.17E-07	3.16E-06	6	0	0
INS-IGF2	0.631867	0.7294	-0.09753	-0.2059	1.17E-07	3.16E-06	13	0	0
INS	0.631867	0.7294	-0.09753	-0.2059	1.17E-07	3.16E-06	13	0	0
OR5T2	0.424787	0.603339	-0.17855	-0.49638	1.16E-07	3.16E-06	1	0	0
OR9G4	0.708074	0.835865	-0.12779	-0.24563	1.16E-07	3.16E-06	4	0	0
DAOA	0.71416	0.824881	-0.11072	-0.21141	1.17E-07	3.16E-06	3	0	0
PTGIR	0.535566	0.469242	0.066325	0.155203	1.17E-07	3.16E-06	4	0	0
SIGLEC6	0.47236	0.589247	-0.11689	-0.36233	1.16E-07	3.16E-06	5	0	0
DNTT	0.655642	0.761988	-0.10635	-0.23216	1.18E-07	3.17E-06	6	0	0

BDKRB1	0.606567	0.733936	-0.12737	-0.29051	1.19E-07	3.19E-06	11	0	0
MTNR1B	0.383977	0.303162	0.080815	0.323201	1.21E-07	3.25E-06	9	0	0
OR8U1	0.657795	0.776715	-0.11892	-0.25244	1.22E-07	3.26E-06	5	0	0
GREM1	0.217765	0.093077	0.124688	1.280682	1.23E-07	3.30E-06	8	0	0
NLRP3	0.418577	0.536343	-0.11777	-0.39083	1.24E-07	3.30E-06	8	0	0
SLC2A9	0.669648	0.790969	-0.12132	-0.24505	1.24E-07	3.30E-06	4	0	0
SPHKAP	0.300106	0.200183	0.099923	0.781456	1.24E-07	3.31E-06	11	0	0
ELN	0.246402	0.195575	0.050827	0.315702	1.24E-07	3.31E-06	8	0	0
FAM107B	0.72046	0.843724	-0.12326	-0.23392	1.24E-07	3.31E-06	10	0	0
SYT9	0.616951	0.787547	-0.1706	-0.35701	1.25E-07	3.32E-06	3	0	0
DNAH5	0.639387	0.779627	-0.14024	-0.30912	1.26E-07	3.32E-06	9	0	0
NOX3	0.639092	0.765672	-0.12658	-0.28737	1.25E-07	3.32E-06	7	0	0
OR52H1	0.592523	0.719081	-0.12656	-0.26804	1.26E-07	3.33E-06	3	0	0
SMAD5	0.205063	0.258365	-0.0533	-0.21685	1.28E-07	3.37E-06	10	0	0
CALML3	0.656828	0.767445	-0.11062	-0.23132	1.30E-07	3.42E-06	8	0	0
LHX8	0.275012	0.164801	0.110211	0.758171	1.31E-07	3.45E-06	10	0	0
TERT	0.36817	0.2616	0.106571	0.96255	1.32E-07	3.46E-06	8	0	0
POU5F1B	0.652012	0.621898	0.030113	0.021929	1.33E-07	3.49E-06	2	0	0
OR2T6	0.584859	0.721357	-0.1365	-0.30688	1.34E-07	3.51E-06	4	0	0
OC90	0.497563	0.674426	-0.17686	-0.43987	1.34E-07	3.51E-06	4	0	0
GRIA4	0.234593	0.188741	0.045852	0.329172	1.34E-07	3.51E-06	12	0	0
TECTB	0.569059	0.681993	-0.11293	-0.24464	1.35E-07	3.51E-06	6	0	0
OMP	0.751815	0.820046	-0.06823	-0.13232	1.40E-07	3.65E-06	7	0	0
OR2T33	0.447533	0.581998	-0.13446	-0.37972	1.41E-07	3.66E-06	3	0	0
PRMT8	0.527491	0.466178	0.061313	0.358207	1.41E-07	3.67E-06	5	0	0
CLCA4	0.203056	0.284705	-0.08165	-0.46804	1.42E-07	3.68E-06	1	0	0
EXOC3L1	0.575527	0.486937	0.088591	0.259176	1.42E-07	3.68E-06	7	0	0
ZNF454	0.393804	0.31338	0.080424	0.535371	1.43E-07	3.70E-06	9	0	0
OR2AG2	0.811838	0.905397	-0.09356	-0.15677	1.45E-07	3.73E-06	5	0	0
PILRA	0.704073	0.803835	-0.09976	-0.207	1.47E-07	3.79E-06	3	0	0
OR2AT4	0.681492	0.808352	-0.12686	-0.25225	1.47E-07	3.79E-06	5	0	0
PCDH8	0.237293	0.135025	0.102268	0.944187	1.48E-07	3.81E-06	12	0	0
PCDHGB6	0.343309	0.20305	0.140259	1.214529	1.49E-07	3.82E-06	6	0	0
C2CD4A	0.274183	0.328529	-0.05435	-0.37526	1.49E-07	3.83E-06	11	0	0
IL3	0.715924	0.780981	-0.06506	-0.13008	1.50E-07	3.85E-06	10	0	0
CORO7-PA	0.610062	0.701201	-0.09114	-0.23436	1.52E-07	3.87E-06	2	0	0
CAPNS2	0.735447	0.840522	-0.10507	-0.2071	1.52E-07	3.88E-06	5	0	0
09-Sep	0.34823	0.456936	-0.10871	-0.41866	1.52E-07	3.88E-06	4	0	0
OR5F1	0.63919	0.755529	-0.11634	-0.24093	1.55E-07	3.94E-06	4	0	0
VNN1	0.556201	0.676151	-0.11995	-0.28206	1.55E-07	3.95E-06	8	0	0
NMS	0.674196	0.822685	-0.14849	-0.29218	1.56E-07	3.96E-06	2	0	0
OR51B4	0.681896	0.805472	-0.12358	-0.24225	1.56E-07	3.96E-06	6	0	0
RFFL	0.29682	0.414165	-0.11735	-0.49139	1.58E-07	3.99E-06	5	0	0
COL11A1	0.325946	0.226407	0.099539	0.762007	1.58E-07	4.00E-06	13	0	0
OR10G4	0.724026	0.86372	-0.13969	-0.27008	1.61E-07	4.04E-06	2	0	0
CCL18	0.516151	0.647683	-0.13153	-0.31814	1.60E-07	4.04E-06	3	0	0
ZNF418	0.441293	0.33098	0.110313	0.809373	1.61E-07	4.04E-06	14	0	0
DEFB115	0.431061	0.567298	-0.13624	-0.38019	1.60E-07	4.04E-06	6	0	0
OR10G8	0.741152	0.860644	-0.11949	-0.22556	1.61E-07	4.05E-06	4	0	0
NAV1	0.784435	0.862974	-0.07854	-0.13434	1.63E-07	4.09E-06	5	0	0
CDRT1	0.796528	0.702565	0.093964	0.179474	1.63E-07	4.09E-06	2	0	0
FXR1	0.424359	0.552172	-0.12781	-0.37213	1.65E-07	4.11E-06	1	0	0
GJA5	0.65137	0.657212	-0.00584	0.048777	1.65E-07	4.12E-06	6	0	0
TPM1	0.358685	0.275834	0.082851	0.582748	1.67E-07	4.17E-06	10	0	0
NLRP8	0.709578	0.834897	-0.12532	-0.23451	1.69E-07	4.21E-06	6	0	0

KBTBD12	0.395539	0.254659	0.14088	0.706188	1.70E-07	4.22E-06	10	0	0
KRTAP2-4	0.851543	0.910823	-0.05928	-0.09927	1.72E-07	4.26E-06	2	0	0
LZTFL1	0.575616	0.589023	-0.01341	-0.06789	1.73E-07	4.27E-06	6	0	0
NKX2-1	0.207557	0.108196	0.099361	0.829087	1.73E-07	4.27E-06	8	0	0
LMX1A	0.3081	0.209485	0.098615	0.900237	1.74E-07	4.27E-06	13	0	0
OR4A15	0.370749	0.50476	-0.13401	-0.44188	1.73E-07	4.27E-06	4	0	0
PRAC2	0.272196	0.123859	0.148337	1.14693	1.73E-07	4.27E-06	17	0	0
KCNK16	0.592371	0.711329	-0.11896	-0.26683	1.75E-07	4.30E-06	6	0	0
ZNF273	0.798623	0.891698	-0.09308	-0.16677	1.75E-07	4.30E-06	3	0	0
STAR	0.481638	0.397336	0.084302	0.849486	1.76E-07	4.31E-06	4	0	0
ZPBP2	0.516063	0.441841	0.074222	0.364924	1.76E-07	4.31E-06	6	0	0
SFXN5	0.67094	0.764167	-0.09323	-0.19506	1.77E-07	4.33E-06	2	0	0
LHX5	0.18374	0.079483	0.104256	1.32017	1.79E-07	4.37E-06	13	0	0
KRT5	0.53575	0.672894	-0.13714	-0.32284	1.81E-07	4.41E-06	9	0	0
PTF1A	0.260117	0.154991	0.105126	0.835032	1.82E-07	4.42E-06	9	0	0
ARRDC4	0.585761	0.716138	-0.13038	-0.30589	1.83E-07	4.44E-06	2	0	0
TP73	0.252252	0.149992	0.10226	0.590071	1.84E-07	4.46E-06	20	0	0
CD79A	0.406824	0.486798	-0.07997	-0.26789	1.84E-07	4.46E-06	7	0	0
N4BP1	0.712016	0.614413	0.097604	0.220994	1.84E-07	4.46E-06	4	0	0
PHYHD1	0.654337	0.5786	0.075738	0.253108	1.85E-07	4.46E-06	8	0	0
TACR3	0.341388	0.228398	0.11299	0.758317	1.85E-07	4.47E-06	11	0	0
PANX3	0.57972	0.676571	-0.09685	-0.22841	1.86E-07	4.47E-06	6	0	0
OSBPL5	0.714016	0.797417	-0.0834	-0.16132	1.89E-07	4.54E-06	14	0	0
TSKU	0.490875	0.555686	-0.06481	-0.21795	1.88E-07	4.54E-06	6	0	0
OR5AS1	0.715648	0.835434	-0.11979	-0.23429	1.91E-07	4.58E-06	4	0	0
SIGLEC10	0.417793	0.529911	-0.11212	-0.33128	1.93E-07	4.63E-06	5	0	0
CPEB1	0.467697	0.373196	0.0945	0.339094	1.95E-07	4.68E-06	6	0	0
TAAR8	0.746708	0.836098	-0.08939	-0.16062	1.97E-07	4.69E-06	3	0	0
LRIT2	0.546224	0.669727	-0.1235	-0.31853	1.97E-07	4.69E-06	9	0	0
SLC32A1	0.210313	0.087107	0.123205	1.237612	1.97E-07	4.69E-06	9	0	0
EDN3	0.247531	0.174894	0.072637	0.765889	1.98E-07	4.72E-06	13	0	0
HDAC2	0.761467	0.836318	-0.07485	-0.13359	2.00E-07	4.75E-06	1	0	0
OR2A2	0.635058	0.775413	-0.14035	-0.2972	2.00E-07	4.75E-06	4	0	0
C12orf56	0.407979	0.310373	0.097606	1.123519	2.00E-07	4.75E-06	8	0	0
MORC1	0.790408	0.897269	-0.10686	-0.19227	2.04E-07	4.82E-06	13	0	0
ELFN1	0.67971	0.802777	-0.12307	-0.24177	2.04E-07	4.82E-06	2	0	0
DEFA4	0.795212	0.860389	-0.06518	-0.11699	2.04E-07	4.82E-06	3	0	0
HOMEZ	0.42798	0.546548	-0.11857	-0.34356	2.03E-07	4.82E-06	4	0	0
TMPRSS15	0.652127	0.769023	-0.1169	-0.23871	2.07E-07	4.88E-06	3	0	0
OR5A2	0.603563	0.716806	-0.11324	-0.25165	2.08E-07	4.90E-06	5	0	0
GNLY	0.578277	0.724193	-0.14592	-0.31966	2.08E-07	4.90E-06	1	0	0
MRGPRF	0.327801	0.209552	0.118249	0.758125	2.09E-07	4.92E-06	14	0	0
LRRC61	0.232361	0.30753	-0.07517	-0.37932	2.11E-07	4.94E-06	6	0	0
CCR5	0.505314	0.631209	-0.1259	-0.35713	2.12E-07	4.96E-06	7	0	0
ZNF382	0.290694	0.147528	0.143166	1.263965	2.14E-07	5.00E-06	8	0	0
OR51G2	0.471553	0.595814	-0.12426	-0.32772	2.16E-07	5.04E-06	2	0	0
RBP7	0.208554	0.26202	-0.05347	0.113927	2.18E-07	5.08E-06	11	0	0
C3orf18	0.62634	0.577435	0.048905	0.124161	2.18E-07	5.08E-06	6	0	0
NXF1	0.395461	0.485069	-0.08961	-0.29928	2.18E-07	5.08E-06	6	0	0
SEC14L6	0.693686	0.7873	-0.09361	-0.18291	2.19E-07	5.10E-06	4	0	0
TG	0.700199	0.830972	-0.13077	-0.25315	2.22E-07	5.14E-06	5	0	0
FES	0.401908	0.268585	0.133323	0.575813	2.23E-07	5.17E-06	3	0	0
OR14I1	0.557165	0.722929	-0.16576	-0.36903	2.24E-07	5.18E-06	2	0	0
LILRB1	0.283314	0.385248	-0.10193	-0.42993	2.26E-07	5.23E-06	3	0	0
GRM5	0.25184	0.125168	0.126672	0.953932	2.27E-07	5.23E-06	1	0	0

NLRX1	0.584009	0.732528	-0.14852	-0.32146	2.27E-07	5.23E-06	2	0	0
KCNIP2	0.282949	0.352289	-0.06934	-0.4038	2.30E-07	5.30E-06	14	0	0
KRTAP19-6	0.61773	0.747558	-0.12983	-0.28855	2.31E-07	5.31E-06	3	0	0
OR1M1	0.70202	0.803738	-0.10172	-0.20005	2.31E-07	5.31E-06	5	0	0
PEX5L	0.237703	0.142249	0.095453	1.340475	2.32E-07	5.33E-06	9	0	0
OR4C6	0.531309	0.680415	-0.14911	-0.35031	2.33E-07	5.33E-06	2	0	0
PROKR2	0.136003	0.037322	0.09868	1.604852	2.33E-07	5.33E-06	2	0	0
RFPL2	0.534773	0.673382	-0.13861	-0.3233	2.34E-07	5.34E-06	5	0	0
G6PC	0.609846	0.73614	-0.12629	-0.31073	2.35E-07	5.35E-06	7	0	0
ZNF479	0.463366	0.605665	-0.1423	-0.38832	2.37E-07	5.41E-06	2	0	0
MEST	0.401253	0.499517	-0.09826	-0.3756	2.41E-07	5.48E-06	9	0	0
PLEKHS1	0.704299	0.833195	-0.1289	-0.24242	2.42E-07	5.51E-06	4	0	0
ERVV-1	0.728269	0.83868	-0.11041	-0.20351	2.44E-07	5.55E-06	6	0	0
CD28	0.540462	0.682147	-0.14169	-0.36704	2.45E-07	5.55E-06	6	0	0
IL23A	0.371517	0.44574	-0.07422	-0.41649	2.46E-07	5.57E-06	7	0	0
PAX1	0.202094	0.095289	0.106804	1.16734	2.48E-07	5.60E-06	17	0	0
DEFB126	0.385878	0.548981	-0.1631	-0.49775	2.49E-07	5.62E-06	1	0	0
CATSPERB	0.571355	0.468992	0.102364	0.313779	2.50E-07	5.63E-06	2	0	0
SCGB1D1	0.575715	0.700095	-0.12438	-0.28	2.50E-07	5.63E-06	6	0	0
KRTAP6-1	0.513896	0.626583	-0.11269	-0.32614	2.51E-07	5.64E-06	3	0	0
OR52N1	0.610451	0.727878	-0.11743	-0.24305	2.52E-07	5.66E-06	4	0	0
OR4C46	0.420113	0.596468	-0.17636	-0.49572	2.54E-07	5.69E-06	1	0	0
PTK6	0.318953	0.40573	-0.08678	-0.32052	2.54E-07	5.69E-06	13	0	0
EVX2	0.310726	0.182512	0.128215	0.792741	2.56E-07	5.72E-06	11	0	0
VSTM1	0.519647	0.643152	-0.12351	-0.32312	2.57E-07	5.73E-06	11	0	0
PGM1	0.186182	0.236354	-0.05017	-0.35443	2.59E-07	5.78E-06	17	0	0
MBL2	0.625099	0.739428	-0.11433	-0.24045	2.59E-07	5.78E-06	5	0	0
OASL	0.319162	0.394185	-0.07502	-0.33543	2.62E-07	5.84E-06	8	0	0
DEFB116	0.454471	0.592644	-0.13817	-0.37321	2.63E-07	5.84E-06	6	0	0
PRSS55	0.630312	0.738751	-0.10844	-0.23047	2.64E-07	5.86E-06	6	0	0
SLAMF7	0.379682	0.536539	-0.15686	-0.49894	2.65E-07	5.88E-06	3	0	0
FAM135B	0.359111	0.268233	0.090877	0.511536	2.67E-07	5.90E-06	7	0	0
SKOR2	0.312713	0.191458	0.121255	0.695957	2.67E-07	5.92E-06	8	0	0
GLT6D1	0.695345	0.825667	-0.13032	-0.2476	2.69E-07	5.95E-06	6	0	0
GPR37L1	0.649239	0.614158	0.035082	0.145453	2.71E-07	5.97E-06	9	0	0
OR5T3	0.431895	0.612071	-0.18018	-0.49338	2.71E-07	5.97E-06	1	0	0
OR13C2	0.539985	0.663454	-0.12347	-0.30914	2.71E-07	5.97E-06	3	0	0
LRP5	0.678954	0.669056	0.009898	-0.02447	2.76E-07	6.06E-06	3	0	0
ZNF492	0.479482	0.42946	0.050022	0.630154	2.76E-07	6.06E-06	8	0	0
GJB5	0.547333	0.662605	-0.11527	-0.27342	2.82E-07	6.17E-06	6	0	0
PDE4B	0.321907	0.238596	0.083311	0.658472	2.83E-07	6.17E-06	8	0	0
OOSP2	0.687149	0.817226	-0.13008	-0.24873	2.83E-07	6.17E-06	9	0	0
OR4E2	0.772607	0.861677	-0.08907	-0.16516	2.81E-07	6.17E-06	6	0	0
CERS3	0.765807	0.683561	0.082246	0.179334	2.82E-07	6.17E-06	8	0	0
IGLL1	0.401373	0.487784	-0.08641	-0.2745	2.83E-07	6.17E-06	5	0	0
GRP	0.199273	0.140142	0.059131	0.842867	2.84E-07	6.19E-06	13	0	0
C2orf78	0.620624	0.758235	-0.13761	-0.28615	2.90E-07	6.29E-06	2	0	0
12-Sep	0.667702	0.568833	0.098869	0.252221	2.90E-07	6.29E-06	7	0	0
RPA3	0.544189	0.690231	-0.14604	-0.37872	2.91E-07	6.32E-06	2	0	0
OR8K5	0.662579	0.78495	-0.12237	-0.25264	2.96E-07	6.41E-06	3	0	0
TMEM30B	0.468429	0.57321	-0.10478	-0.29993	2.98E-07	6.44E-06	5	0	0
NAA11	0.712284	0.81365	-0.10137	-0.19632	3.00E-07	6.49E-06	9	0	0
AARS2	0.290402	0.337102	-0.0467	-0.2986	3.04E-07	6.57E-06	13	0	0
HCN1	0.285582	0.189719	0.095863	0.796248	3.06E-07	6.58E-06	8	0	0
MKX	0.236158	0.115363	0.120795	1.044372	3.05E-07	6.58E-06	17	0	0

TNRC6C	0.779414	0.863518	-0.0841	-0.15026	3.06E-07	6.58E-06	5	0	0
SP9	0.297543	0.159658	0.137885	0.924431	3.08E-07	6.62E-06	11	0	0
CAPSL	0.458968	0.607737	-0.14877	-0.38118	3.12E-07	6.70E-06	6	0	0
MMP7	0.601445	0.658378	-0.05693	-0.28961	3.19E-07	6.83E-06	6	0	0
IMPG1	0.562352	0.677564	-0.11521	-0.28465	3.20E-07	6.86E-06	3	0	0
TMEM202	0.565306	0.655367	-0.09006	-0.25503	3.22E-07	6.89E-06	2	0	0
FGF6	0.671332	0.76782	-0.09649	-0.2027	3.28E-07	7.00E-06	11	0	0
GALR1	0.212377	0.106377	0.106001	1.101289	3.29E-07	7.01E-06	21	0	0
RYR2	0.386085	0.262643	0.123442	0.900579	3.30E-07	7.02E-06	6	0	0
TMEM173	0.349543	0.314393	0.03515	0.58343	3.30E-07	7.02E-06	6	0	0
CYP1A2	0.733697	0.806879	-0.07318	-0.14042	3.33E-07	7.07E-06	7	0	0
OVGP1	0.626739	0.721026	-0.09429	-0.20523	3.45E-07	7.32E-06	5	0	0
IFNA21	0.723739	0.850661	-0.12692	-0.23018	3.46E-07	7.33E-06	1	0	0
CEL	0.470726	0.585037	-0.11431	-0.32852	3.48E-07	7.38E-06	2	0	0
ASPRV1	0.813666	0.866205	-0.05254	-0.08986	3.51E-07	7.42E-06	5	0	0
ACSM1	0.722061	0.860504	-0.13844	-0.25014	3.52E-07	7.43E-06	3	0	0
OR2G6	0.671944	0.801868	-0.12992	-0.26355	3.55E-07	7.50E-06	4	0	0
CITED4	0.19345	0.225875	-0.03243	0.056005	3.58E-07	7.55E-06	9	0	0
PHOX2B	0.249363	0.159858	0.089505	0.658448	3.61E-07	7.59E-06	12	0	0
OR6B1	0.640683	0.76224	-0.12156	-0.27294	3.63E-07	7.63E-06	8	0	0
HLA-DMB	0.222862	0.269438	-0.04658	-0.35676	3.66E-07	7.69E-06	14	0	0
OR2V1	0.649672	0.745332	-0.09566	-0.19152	3.71E-07	7.79E-06	4	0	0
IRX2	0.253645	0.113089	0.140555	1.147288	3.72E-07	7.80E-06	14	0	0
CCL7	0.645433	0.761205	-0.11577	-0.23448	3.74E-07	7.83E-06	4	0	0
PRAME	0.501688	0.613808	-0.11212	-0.32567	3.79E-07	7.93E-06	12	0	0
STARD13	0.342048	0.256188	0.08586	0.581575	3.80E-07	7.93E-06	6	0	0
CDSN	0.562787	0.65481	-0.09202	-0.22316	3.81E-07	7.94E-06	5	0	0
OR10A5	0.70556	0.817259	-0.1117	-0.21399	3.82E-07	7.96E-06	2	0	0
C19orf18	0.765159	0.860827	-0.09567	-0.17313	3.83E-07	7.97E-06	3	0	0
DAB2	0.705043	0.849909	-0.14487	-0.26518	3.86E-07	8.02E-06	2	0	0
ACSBG2	0.688449	0.82683	-0.13838	-0.27361	3.93E-07	8.14E-06	7	0	0
CYP4F3	0.492089	0.573499	-0.08141	-0.31158	3.93E-07	8.14E-06	4	0	0
SPP2	0.786522	0.877198	-0.09068	-0.15655	3.95E-07	8.17E-06	3	0	0
ERP27	0.821924	0.911964	-0.09004	-0.15245	3.95E-07	8.17E-06	3	0	0
OR8G5	0.61548	0.738553	-0.12307	-0.26627	3.99E-07	8.24E-06	3	0	0
FILIP1	0.59517	0.455826	0.139343	0.424942	4.00E-07	8.25E-06	8	0	0
LAMB4	0.798573	0.87798	-0.07941	-0.14027	4.03E-07	8.30E-06	7	0	0
RNF150	0.813912	0.861293	-0.04738	-0.08145	4.04E-07	8.33E-06	3	0	0
RAB37	0.467817	0.552677	-0.08486	-0.16663	4.05E-07	8.34E-06	9	0	0
MEI4	0.649842	0.761137	-0.11129	-0.23217	4.07E-07	8.37E-06	3	0	0
C6	0.70565	0.826224	-0.12057	-0.22334	4.08E-07	8.38E-06	4	0	0
CBR3	0.180158	0.243753	-0.06359	-0.2718	4.15E-07	8.51E-06	7	0	0
APOC1	0.475866	0.463542	0.012323	0.322	4.16E-07	8.52E-06	2	0	0
SPAG6	0.308962	0.181579	0.127383	0.866752	4.19E-07	8.56E-06	15	0	0
USP6	0.44713	0.322962	0.124168	0.568118	4.20E-07	8.58E-06	8	0	0
SLCO1B1	0.47733	0.642724	-0.16539	-0.43743	4.23E-07	8.63E-06	2	0	0
RINT1	0.178159	0.269423	-0.09126	-0.37981	4.24E-07	8.64E-06	4	0	0
CCDC105	0.51821	0.423023	0.095187	0.377458	4.27E-07	8.69E-06	10	0	0
GFRAL	0.555914	0.690193	-0.13428	-0.31348	4.27E-07	8.69E-06	6	0	0
CYP2C9	0.613625	0.731608	-0.11798	-0.25139	4.31E-07	8.76E-06	3	0	0
CPE	0.726077	0.842562	-0.11649	-0.21195	4.42E-07	8.96E-06	1	0	0
PLCL2	0.456447	0.568456	-0.11201	-0.32699	4.45E-07	9.01E-06	3	0	0
TCTEX1D4	0.41603	0.328919	0.087112	0.547828	4.48E-07	9.07E-06	8	0	0
TGFBR3	0.772233	0.900556	-0.12832	-0.21996	4.51E-07	9.12E-06	5	0	0
S100A4	0.680778	0.691204	-0.01043	-0.0248	4.52E-07	9.14E-06	4	0	0

MTMR2	0.709041	0.839777	-0.13074	-0.24981	4.54E-07	9.16E-06	3	0	0
POU4F2	0.219326	0.101276	0.11805	1.094306	4.57E-07	9.22E-06	6	0	0
KLF11	0.34762	0.258185	0.089435	0.893311	4.61E-07	9.28E-06	7	0	0
GPNMB	0.650692	0.835276	-0.18458	-0.35544	4.61E-07	9.28E-06	1	0	0
OR3A3	0.651638	0.774889	-0.12325	-0.24677	4.67E-07	9.38E-06	3	0	0
DNAH14	0.484556	0.613436	-0.12888	-0.33411	4.71E-07	9.44E-06	1	0	0
OR52D1	0.731622	0.848988	-0.11737	-0.21195	4.71E-07	9.44E-06	1	0	0
OR9G1	0.611795	0.712807	-0.10101	-0.23078	4.72E-07	9.44E-06	4	0	0
PRAC1	0.254475	0.116854	0.137621	1.125239	4.72E-07	9.44E-06	21	0	0
SPEF2	0.224286	0.303926	-0.07964	-0.57772	4.78E-07	9.54E-06	10	0	0
NFKBIE	0.634433	0.744038	-0.1096	-0.22589	4.78E-07	9.54E-06	2	0	0
IRX3	0.211266	0.146439	0.064828	0.627299	4.78E-07	9.54E-06	7	0	0
OR5W2	0.6476	0.790837	-0.14324	-0.29774	4.79E-07	9.54E-06	2	0	0
C1QTNF3	0.614794	0.546602	0.068192	0.290589	4.80E-07	9.55E-06	7	0	0
TAS2R39	0.693452	0.809387	-0.11594	-0.22314	4.84E-07	9.61E-06	5	0	0
ENPP5	0.136481	0.16542	-0.02894	-0.32838	4.85E-07	9.63E-06	12	0	0
BLK	0.648616	0.743697	-0.09508	-0.19461	4.88E-07	9.67E-06	11	0	0
FCGR3B	0.687657	0.825679	-0.13802	-0.26043	4.92E-07	9.74E-06	1	0	0
DCD	0.654543	0.778855	-0.12431	-0.2693	4.92E-07	9.74E-06	5	0	0
LRRC55	0.285219	0.380289	-0.09507	-0.49983	4.94E-07	9.76E-06	5	0	0
NLRP12	0.65755	0.767727	-0.11018	-0.24144	4.96E-07	9.79E-06	3	0	0
ZNF80	0.721434	0.810768	-0.08933	-0.16282	4.98E-07	9.81E-06	4	0	0
OR4S1	0.590593	0.710272	-0.11968	-0.26551	4.98E-07	9.81E-06	5	0	0
OR7D4	0.511033	0.646866	-0.13583	-0.34167	4.99E-07	9.81E-06	2	0	0
LEKR1	0.593124	0.668246	-0.07512	-0.26771	5.06E-07	9.94E-06	2	0	0
OR51D1	0.681416	0.765218	-0.0838	-0.18791	5.14E-07	1.01E-05	3	0	0
ANKRD2	0.520505	0.599781	-0.07928	-0.25798	5.17E-07	1.01E-05	9	0	0
UNC13C	0.601371	0.732929	-0.13156	-0.28924	5.18E-07	1.01E-05	5	0	0
ACVRL1	0.37781	0.277648	0.100162	0.555728	5.22E-07	1.02E-05	7	0	0
PRDM13	0.304586	0.200298	0.104289	0.649434	5.24E-07	1.02E-05	15	0	0
DNAJA4	0.545948	0.62909	-0.08314	-0.04767	5.24E-07	1.02E-05	10	0	0
MZB1	0.521235	0.606142	-0.08491	-0.2318	5.27E-07	1.03E-05	9	0	0
TLX3	0.301288	0.187358	0.113929	0.77503	5.28E-07	1.03E-05	14	0	0
OR4C12	0.589672	0.768838	-0.17917	-0.37715	5.28E-07	1.03E-05	1	0	0
FOXR1	0.709297	0.670187	0.03911	0.117734	5.28E-07	1.03E-05	8	0	0
PNLIPRP3	0.623589	0.750701	-0.12711	-0.27445	5.35E-07	1.04E-05	3	0	0
GSTP1	0.250484	0.336005	-0.08552	-0.31097	5.36E-07	1.04E-05	6	0	0
DLEU7	0.648584	0.59341	0.055174	0.240622	5.42E-07	1.05E-05	9	0	0
ASIC5	0.752741	0.840769	-0.08803	-0.15829	5.48E-07	1.06E-05	3	0	0
FITM1	0.755433	0.703683	0.05175	0.130599	5.47E-07	1.06E-05	6	0	0
CCL8	0.613312	0.702264	-0.08895	-0.19937	5.47E-07	1.06E-05	7	0	0
OR2B3	0.56245	0.699528	-0.13708	-0.31496	5.49E-07	1.06E-05	5	0	0
CASP1	0.732966	0.826802	-0.09384	-0.18467	5.51E-07	1.06E-05	2	0	0
CARD16	0.732966	0.826802	-0.09384	-0.18467	5.51E-07	1.06E-05	2	0	0
CARD17	0.732966	0.826802	-0.09384	-0.18467	5.51E-07	1.06E-05	2	0	0
GSDMC	0.493103	0.630664	-0.13756	-0.33575	5.53E-07	1.06E-05	6	0	0
FAM71F2	0.718046	0.687569	0.030477	0.080922	5.54E-07	1.06E-05	5	0	0
ACOX2	0.511819	0.475902	0.035917	0.130213	5.55E-07	1.06E-05	5	0	0
CNTNAP5	0.283237	0.169172	0.114065	0.720786	5.55E-07	1.06E-05	9	0	0
C10orf99	0.671413	0.770335	-0.09892	-0.19352	5.58E-07	1.06E-05	6	0	0
SLC4A1	0.654255	0.751935	-0.09768	-0.20913	5.62E-07	1.07E-05	9	0	0
DEFB128	0.622435	0.735617	-0.11318	-0.25066	5.66E-07	1.08E-05	4	0	0
OTOL1	0.604057	0.75887	-0.15481	-0.33158	5.67E-07	1.08E-05	4	0	0
RACGAP1	0.470798	0.313538	0.15726	0.597003	5.71E-07	1.09E-05	3	0	0
CX3CR1	0.666053	0.722121	-0.05607	-0.13921	5.73E-07	1.09E-05	8	0	0

GPR75	0.696776	0.618075	0.078701	0.182962	5.77E-07	1.09E-05	14	0	0
KRTAP19-5	0.702107	0.812244	-0.11014	-0.22809	5.79E-07	1.10E-05	6	0	0
CP	0.581203	0.667856	-0.08665	-0.23084	5.80E-07	1.10E-05	4	0	0
ESRRG	0.285082	0.173186	0.111897	0.708887	5.83E-07	1.10E-05	8	0	0
FPR3	0.529748	0.669843	-0.14009	-0.285	5.83E-07	1.10E-05	3	0	0
FOXD2	0.500585	0.392594	0.107991	0.442567	5.83E-07	1.10E-05	7	0	0
PAPOLB	0.607054	0.581588	0.025466	0.141154	5.86E-07	1.10E-05	10	0	0
ZNF732	0.375	0.239205	0.135795	0.673265	5.89E-07	1.11E-05	4	0	0
LRRC30	0.699338	0.786128	-0.08679	-0.17387	5.89E-07	1.11E-05	6	0	0
OR2D2	0.719319	0.834767	-0.11545	-0.21388	5.92E-07	1.11E-05	3	0	0
OR5D18	0.615407	0.723756	-0.10835	-0.23815	5.95E-07	1.12E-05	7	0	0
CCR2	0.55433	0.6481	-0.09377	-0.26747	5.96E-07	1.12E-05	6	0	0
ZNF750	0.660504	0.826709	-0.1662	-0.32232	5.96E-07	1.12E-05	13	0	0
ZNF728	0.566117	0.521009	0.045108	0.324459	5.99E-07	1.12E-05	8	0	0
LCP2	0.493135	0.563847	-0.07071	-0.23083	6.05E-07	1.13E-05	6	0	0
SH3BP5	0.715832	0.702739	0.013094	0.045073	6.12E-07	1.14E-05	7	0	0
TAS2R1	0.627061	0.750748	-0.12369	-0.26041	6.14E-07	1.14E-05	3	0	0
PROB1	0.709132	0.697524	0.011608	0.046499	6.12E-07	1.14E-05	7	0	0
KRTAP13-1	0.646325	0.769937	-0.12361	-0.25862	6.13E-07	1.14E-05	7	0	0
ASCL4	0.519753	0.645095	-0.12534	-0.3362	6.15E-07	1.14E-05	8	0	0
EFCAB9	0.725336	0.797058	-0.07172	-0.13585	6.18E-07	1.15E-05	2	0	0
TM4SF19	0.57626	0.728692	-0.15243	-0.3415	6.21E-07	1.15E-05	12	0	0
PKD1L1	0.708552	0.649947	0.058606	0.14402	6.21E-07	1.15E-05	6	0	0
OR52E8	0.679735	0.778933	-0.0992	-0.19164	6.21E-07	1.15E-05	6	0	0
PSMB11	0.68663	0.772297	-0.08567	-0.17175	6.19E-07	1.15E-05	7	0	0
ZNF710	0.460641	0.343394	0.117247	0.371087	6.20E-07	1.15E-05	6	0	0
XCR1	0.826443	0.897879	-0.07144	-0.12093	6.23E-07	1.15E-05	2	0	0
LIPN	0.613935	0.733072	-0.11914	-0.27896	6.22E-07	1.15E-05	2	0	0
C3orf62	0.723825	0.686804	0.03702	0.123353	6.26E-07	1.15E-05	7	0	0
MCC	0.251418	0.319257	-0.06784	-0.35812	6.27E-07	1.15E-05	11	0	0
C14orf93	0.114015	0.152796	-0.03878	-0.23454	6.28E-07	1.16E-05	8	0	0
GPR132	0.422346	0.502526	-0.08018	-0.28935	6.31E-07	1.16E-05	7	0	0
SLC22A18A	0.691625	0.788092	-0.09647	-0.19063	6.33E-07	1.16E-05	13	0	0
OR51F2	0.673637	0.799389	-0.12575	-0.26099	6.34E-07	1.16E-05	3	0	0
CNTN3	0.738795	0.83014	-0.09135	-0.17888	6.37E-07	1.17E-05	3	0	0
MRGPRX4	0.628158	0.759978	-0.13182	-0.2808	6.43E-07	1.18E-05	3	0	0
IL20RA	0.611733	0.757266	-0.14553	-0.31186	6.45E-07	1.18E-05	10	0	0
ADCY8	0.389027	0.348595	0.040432	0.601852	6.49E-07	1.19E-05	6	0	0
TTLL2	0.702028	0.811037	-0.10901	-0.20468	6.52E-07	1.19E-05	4	0	0
C1QC	0.305681	0.369825	-0.06414	-0.27557	6.56E-07	1.19E-05	6	0	0
AGXT	0.487045	0.568586	-0.08154	-0.23949	6.56E-07	1.19E-05	5	0	0
UGT3A1	0.710677	0.860348	-0.14967	-0.27229	6.67E-07	1.21E-05	2	0	0
OR14A16	0.682115	0.801555	-0.11944	-0.2247	6.69E-07	1.22E-05	6	0	0
OR1L4	0.673989	0.787516	-0.11353	-0.22389	6.70E-07	1.22E-05	2	0	0
SCN10A	0.591455	0.716388	-0.12493	-0.28907	6.72E-07	1.22E-05	2	0	0
KIAA1143	0.070804	0.097517	-0.02671	-0.37144	6.74E-07	1.22E-05	12	0	0
SYNE3	0.736402	0.826719	-0.09032	-0.17704	6.74E-07	1.22E-05	6	0	0
HINT1	0.666948	0.695372	-0.02842	-0.14671	6.76E-07	1.22E-05	4	0	0
VN1R2	0.643841	0.767093	-0.12325	-0.26173	6.80E-07	1.23E-05	3	0	0
GRAP2	0.452694	0.605632	-0.15294	-0.42138	6.83E-07	1.23E-05	4	0	0
PIK3R6	0.372968	0.437449	-0.06448	-0.27389	6.89E-07	1.24E-05	8	0	0
FCRL5	0.629218	0.783968	-0.15475	-0.31514	7.03E-07	1.26E-05	2	0	0
RNF133	0.779247	0.856086	-0.07684	-0.14248	7.02E-07	1.26E-05	5	0	0
SULF1	0.224863	0.285304	-0.06044	-0.40752	7.02E-07	1.26E-05	8	0	0
TRPM6	0.352029	0.437841	-0.08581	-0.2528	7.03E-07	1.26E-05	9	0	0

CEACAM8	0.740358	0.80983	-0.06947	-0.14813	7.02E-07	1.26E-05	4	0	0
CARD8	0.651066	0.77767	-0.1266	-0.25199	7.04E-07	1.26E-05	3	0	0
SIRPD	0.713242	0.871274	-0.15803	-0.28487	7.05E-07	1.26E-05	2	0	0
TAAR9	0.721842	0.81974	-0.0979	-0.18253	7.12E-07	1.27E-05	5	0	0
MRGPRX1	0.736307	0.843684	-0.10738	-0.19393	7.15E-07	1.28E-05	1	0	0
S100A7A	0.553821	0.658584	-0.10476	-0.25629	7.21E-07	1.29E-05	4	0	0
UTF1	0.266544	0.163063	0.103482	1.101308	7.30E-07	1.30E-05	7	0	0
NLRP9	0.693945	0.809704	-0.11576	-0.22757	7.29E-07	1.30E-05	5	0	0
OR52B2	0.544719	0.65744	-0.11272	-0.23752	7.31E-07	1.30E-05	5	0	0
VAV1	0.347455	0.447908	-0.10045	-0.35523	7.37E-07	1.31E-05	7	0	0
ZDHHC21	0.401041	0.42632	-0.02528	-0.35091	7.39E-07	1.31E-05	10	0	0
C9orf129	0.444386	0.295881	0.148505	0.979632	7.41E-07	1.31E-05	6	0	0
IL32	0.54529	0.467115	0.078175	0.258708	7.41E-07	1.31E-05	9	0	0
ESPNL	0.668678	0.752337	-0.08366	-0.18544	7.57E-07	1.34E-05	9	0	0
NOS1	0.653858	0.743673	-0.08982	-0.17332	7.63E-07	1.35E-05	7	0	0
WISP3	0.672011	0.767941	-0.09593	-0.20499	7.66E-07	1.35E-05	10	0	0
NLRP11	0.578169	0.682288	-0.10412	-0.25719	7.68E-07	1.36E-05	8	0	0
DIO2	0.702071	0.795609	-0.09354	-0.17758	7.72E-07	1.36E-05	2	0	0
FCRLA	0.55886	0.695259	-0.1364	-0.35171	7.73E-07	1.36E-05	4	0	0
DARS	0.281733	0.324868	-0.04313	-0.33289	7.83E-07	1.38E-05	7	0	0
PIP	0.5681	0.677846	-0.10975	-0.264	7.87E-07	1.38E-05	4	0	0
UBE2D2	0.661727	0.760498	-0.09877	-0.21457	7.92E-07	1.39E-05	3	0	0
LMOD2	0.673217	0.780256	-0.10704	-0.21348	7.94E-07	1.39E-05	5	0	0
ABHD5	0.057902	0.073878	-0.01598	-0.20773	8.04E-07	1.41E-05	4	0	0
A1CF	0.694144	0.791097	-0.09695	-0.19043	8.07E-07	1.41E-05	4	0	0
GRIA1	0.561861	0.702191	-0.14033	-0.32582	8.09E-07	1.42E-05	4	0	0
SLC13A1	0.760866	0.843169	-0.0823	-0.14868	8.12E-07	1.42E-05	3	0	0
RARRES2	0.621526	0.558521	0.063005	0.395765	8.14E-07	1.42E-05	8	0	0
SH2D3A	0.112852	0.150293	-0.03744	-0.2498	8.15E-07	1.42E-05	2	0	0
ARL14EPL	0.825977	0.889565	-0.06359	-0.10582	8.18E-07	1.43E-05	2	0	0
PDHA2	0.836922	0.903589	-0.06667	-0.11106	8.21E-07	1.43E-05	10	0	0
TCL1B	0.583998	0.703077	-0.11908	-0.28706	8.22E-07	1.43E-05	8	0	0
ZNF285	0.227599	0.108952	0.118647	1.1968	8.24E-07	1.43E-05	5	0	0
OR4F15	0.761279	0.871688	-0.11041	-0.19452	8.28E-07	1.44E-05	5	0	0
C1orf198	0.756441	0.709017	0.047424	0.118344	8.31E-07	1.44E-05	5	0	0
FAM184A	0.672152	0.764985	-0.09283	-0.20858	8.31E-07	1.44E-05	7	0	0
OPRM1	0.482058	0.624316	-0.14226	-0.38048	8.33E-07	1.44E-05	4	0	0
RLN2	0.746476	0.824206	-0.07773	-0.15087	8.39E-07	1.45E-05	2	0	0
AICDA	0.750163	0.846355	-0.09619	-0.17334	8.39E-07	1.45E-05	3	0	0
ALDH3B2	0.656102	0.747753	-0.09165	-0.18877	8.41E-07	1.45E-05	2	0	0
SDR9C7	0.754225	0.846179	-0.09195	-0.16373	8.42E-07	1.45E-05	5	0	0
HOXB4	0.467631	0.331296	0.136335	0.523038	8.45E-07	1.46E-05	8	0	0
MB	0.670541	0.558502	0.11204	0.27054	8.49E-07	1.46E-05	3	0	0
TCTEX1D1	0.225544	0.091861	0.133684	1.268407	8.52E-07	1.46E-05	10	0	0
H1FOO	0.752135	0.813885	-0.06175	-0.11702	8.52E-07	1.46E-05	7	0	0
ADH7	0.679755	0.814801	-0.13505	-0.25962	8.56E-07	1.47E-05	4	0	0
BLID	0.696541	0.835689	-0.13915	-0.26372	8.60E-07	1.47E-05	8	0	0
OR5H2	0.62529	0.733328	-0.10804	-0.25695	8.64E-07	1.48E-05	3	0	0
KRTAP19-7	0.716059	0.793914	-0.07785	-0.15653	8.76E-07	1.50E-05	2	0	0
C1QTNF5	0.590644	0.686529	-0.09589	-0.22633	8.82E-07	1.50E-05	6	0	0
MFRP	0.590644	0.686529	-0.09589	-0.22633	8.82E-07	1.50E-05	6	0	0
ZNF880	0.242327	0.126778	0.115549	1.117849	8.82E-07	1.50E-05	12	0	0
MYH7B	0.656086	0.771515	-0.11543	-0.24464	8.81E-07	1.50E-05	3	0	0
OR5D14	0.514443	0.650182	-0.13574	-0.33239	8.84E-07	1.50E-05	3	0	0
FAM71C	0.62667	0.742558	-0.11589	-0.23523	8.92E-07	1.52E-05	7	0	0

FHL2	0.616111	0.539045	0.077065	0.224388	8.98E-07	1.53E-05	8	0	0
L3MBTL3	0.820967	0.906175	-0.08521	-0.14186	9.04E-07	1.53E-05	2	0	0
RPS2	0.129938	0.158322	-0.02838	-0.20867	9.09E-07	1.54E-05	4	0	0
SELP	0.729249	0.745218	-0.01597	-0.0209	9.18E-07	1.55E-05	7	0	0
CA10	0.335808	0.209376	0.126432	0.68467	9.19E-07	1.55E-05	15	0	0
OR2AE1	0.778299	0.884128	-0.10583	-0.1905	9.23E-07	1.56E-05	6	0	0
IFNA13	0.749386	0.823948	-0.07456	-0.14534	9.21E-07	1.56E-05	3	0	0
TGFB1I1	0.346723	0.281289	0.065434	0.23661	9.23E-07	1.56E-05	11	0	0
OR2Z1	0.724901	0.833658	-0.10876	-0.20848	9.22E-07	1.56E-05	6	0	0
LRRK1	0.130313	0.171496	-0.04118	-0.35272	9.24E-07	1.56E-05	7	0	0
CDH18	0.625138	0.774089	-0.14895	-0.30462	9.47E-07	1.59E-05	4	0	0
FAM19A5	0.327157	0.28954	0.037617	0.321618	9.49E-07	1.59E-05	7	0	0
ELMSAN1	0.752538	0.666387	0.086151	0.211398	9.54E-07	1.60E-05	4	0	0
CBFA2T3	0.310321	0.357034	-0.04671	-0.10222	9.68E-07	1.62E-05	14	0	0
OR4D5	0.721254	0.83439	-0.11314	-0.21146	9.74E-07	1.63E-05	2	0	0
FCER2	0.512689	0.598349	-0.08566	-0.2289	9.90E-07	1.66E-05	9	0	0
ZNF98	0.377286	0.276056	0.10123	0.485339	1.00E-06	1.68E-05	4	0	0
PCDHGA12	0.363728	0.22212	0.141607	0.798272	1.01E-06	1.69E-05	10	0	0
NPY2R	0.163491	0.088391	0.0751	0.821915	1.01E-06	1.69E-05	11	0	0
MUC7	0.798761	0.866162	-0.0674	-0.11605	1.02E-06	1.70E-05	3	0	0
STMN2	0.502747	0.502189	0.000557	0.159406	1.02E-06	1.70E-05	11	0	0
TRIM64B	0.76512	0.876568	-0.11145	-0.19381	1.02E-06	1.70E-05	1	0	0
USP33	0.46591	0.510685	-0.04477	-0.09698	1.02E-06	1.70E-05	4	0	0
KRTAP10-1	0.644115	0.732528	-0.08841	-0.18819	1.03E-06	1.70E-05	4	0	0
C1orf226	0.733207	0.838335	-0.10513	-0.19432	1.03E-06	1.71E-05	8	0	0
TRIM61	0.509294	0.414568	0.094726	0.504651	1.03E-06	1.71E-05	10	0	0
SNTG1	0.278693	0.208549	0.070144	0.858775	1.03E-06	1.71E-05	11	0	0
DNAJC9	0.711809	0.826419	-0.11461	-0.21538	1.03E-06	1.71E-05	3	0	0
IFITM3	0.191903	0.079046	0.112857	1.153825	1.03E-06	1.71E-05	2	0	0
OR5B2	0.458162	0.567115	-0.10895	-0.30684	1.04E-06	1.71E-05	4	0	0
ZNF626	0.76031	0.877793	-0.11748	-0.20568	1.04E-06	1.71E-05	3	0	0
SCML4	0.384288	0.480966	-0.09668	-0.39994	1.04E-06	1.72E-05	8	0	0
GDF10	0.336471	0.265087	0.071384	0.369783	1.04E-06	1.72E-05	5	0	0
LY86	0.681674	0.7686	-0.08693	-0.17801	1.05E-06	1.73E-05	5	0	0
NEUROD6	0.556965	0.700342	-0.14338	-0.32016	1.06E-06	1.75E-05	5	0	0
MS4A13	0.744745	0.831274	-0.08653	-0.16354	1.07E-06	1.75E-05	14	0	0
LYPD6B	0.492274	0.543071	-0.0508	-0.27969	1.07E-06	1.76E-05	7	0	0
ACY3	0.309358	0.395955	-0.0866	-0.34695	1.08E-06	1.77E-05	4	0	0
PLD4	0.678119	0.752052	-0.07393	-0.15631	1.08E-06	1.77E-05	6	0	0
OR2G3	0.611238	0.71644	-0.1052	-0.2176	1.09E-06	1.78E-05	4	0	0
CTNNA2	0.348804	0.50238	-0.15358	-0.51402	1.09E-06	1.78E-05	1	0	0
RAX	0.302224	0.197636	0.104588	0.724325	1.09E-06	1.78E-05	6	0	0
SMLR1	0.722905	0.820798	-0.09789	-0.17995	1.10E-06	1.79E-05	3	0	0
ZNF574	0.539377	0.652497	-0.11312	-0.2643	1.10E-06	1.79E-05	4	0	0
KALRN	0.323168	0.438015	-0.11485	-0.38652	1.10E-06	1.79E-05	2	0	0
RNASE13	0.734754	0.798692	-0.06394	-0.12525	1.11E-06	1.80E-05	5	0	0
RASGRF2	0.297494	0.206493	0.091002	1.217682	1.12E-06	1.81E-05	8	0	0
ADAMDEC	0.683759	0.768056	-0.0843	-0.17641	1.12E-06	1.81E-05	6	0	0
FRMD1	0.832291	0.905551	-0.07326	-0.12473	1.12E-06	1.82E-05	6	0	0
TOR3A	0.331191	0.39631	-0.06512	-0.26241	1.12E-06	1.82E-05	10	0	0
OR51I2	0.78624	0.876944	-0.0907	-0.16356	1.13E-06	1.83E-05	3	0	0
TGM1	0.545676	0.62221	-0.07653	-0.18504	1.13E-06	1.83E-05	4	0	0
COMMD10	0.337654	0.400774	-0.06312	-0.25479	1.14E-06	1.84E-05	8	0	0
PRRX2	0.247933	0.145075	0.102858	0.729758	1.15E-06	1.85E-05	4	0	0
C3AR1	0.502118	0.633531	-0.13141	-0.42197	1.15E-06	1.85E-05	6	0	0

RAB34	0.164597	0.229443	-0.06485	-0.34988	1.15E-06	1.85E-05	19	0	0
RPL23A	0.164597	0.229443	-0.06485	-0.34988	1.15E-06	1.85E-05	19	0	0
GAL3ST3	0.447038	0.337777	0.10926	0.580298	1.16E-06	1.87E-05	7	0	0
PSMA6	0.76092	0.696545	0.064375	0.146325	1.16E-06	1.87E-05	3	0	0
PRB1	0.547899	0.740585	-0.19269	-0.42801	1.16E-06	1.87E-05	1	0	0
TAS2R20	0.866198	0.927307	-0.06111	-0.0974	1.17E-06	1.87E-05	3	0	0
KRTAP26-1	0.74779	0.856006	-0.10822	-0.19461	1.17E-06	1.88E-05	6	0	0
OR5AK2	0.632402	0.776244	-0.14384	-0.30065	1.17E-06	1.88E-05	2	0	0
HOXB9	0.207259	0.161328	0.045931	0.133877	1.17E-06	1.88E-05	9	0	0
SLAMF9	0.623509	0.713136	-0.08963	-0.16704	1.18E-06	1.89E-05	4	0	0
CASD1	0.231612	0.289846	-0.05823	-0.26293	1.18E-06	1.89E-05	4	0	0
GP1BB	0.571018	0.500328	0.07069	0.173052	1.18E-06	1.89E-05	10	0	0
DEFB127	0.77364	0.872001	-0.09836	-0.17821	1.19E-06	1.90E-05	4	0	0
CALCB	0.597685	0.55113	0.046554	0.40842	1.20E-06	1.91E-05	5	0	0
OR52W1	0.77018	0.853774	-0.08359	-0.16106	1.21E-06	1.92E-05	6	0	0
BTF3	0.133309	0.179068	-0.04576	-0.32347	1.21E-06	1.93E-05	9	0	0
ANKRD45	0.504771	0.407817	0.096953	0.71469	1.23E-06	1.95E-05	10	0	0
SCO2	0.324873	0.396387	-0.07151	-0.20247	1.23E-06	1.95E-05	8	0	0
IL17F	0.7184	0.81677	-0.09837	-0.18284	1.23E-06	1.95E-05	4	0	0
FER1L5	0.612612	0.549366	0.063246	0.256071	1.23E-06	1.95E-05	5	0	0
TEKT5	0.73147	0.800766	-0.0693	-0.14441	1.23E-06	1.95E-05	6	0	0
OR5T1	0.473315	0.611826	-0.13851	-0.35427	1.24E-06	1.96E-05	2	0	0
OR6X1	0.700379	0.804561	-0.10418	-0.21057	1.25E-06	1.97E-05	6	0	0
VWA8	0.238524	0.261336	-0.02281	-0.28612	1.25E-06	1.97E-05	5	0	0
GJA10	0.78925	0.886302	-0.09705	-0.16848	1.25E-06	1.97E-05	5	0	0
DCAF4L2	0.83918	0.927004	-0.08782	-0.14774	1.25E-06	1.98E-05	11	0	0
AIM2	0.554069	0.701922	-0.14785	-0.33585	1.26E-06	1.99E-05	1	0	0
DSG1	0.683595	0.698168	-0.01457	0.035611	1.27E-06	1.99E-05	8	0	0
RFPL4A	0.42589	0.568272	-0.14238	-0.4374	1.28E-06	2.01E-05	2	0	0
OR6B2	0.501788	0.592082	-0.09029	-0.23728	1.28E-06	2.01E-05	5	0	0
NXPE1	0.747041	0.834049	-0.08701	-0.17224	1.28E-06	2.01E-05	5	0	0
MRAS	0.608636	0.719085	-0.11045	-0.22878	1.28E-06	2.01E-05	5	0	0
MPL	0.758919	0.846302	-0.08738	-0.18188	1.29E-06	2.02E-05	2	0	0
KRBOX1	0.724872	0.794526	-0.06965	-0.13642	1.29E-06	2.02E-05	6	0	0
OR10V1	0.699846	0.815375	-0.11553	-0.22406	1.29E-06	2.02E-05	4	0	0
HAO1	0.698134	0.801398	-0.10326	-0.20232	1.29E-06	2.02E-05	5	0	0
ERICH1	0.185546	0.101362	0.084183	1.134684	1.31E-06	2.04E-05	7	0	0
CD5	0.515274	0.618772	-0.1035	-0.27848	1.31E-06	2.05E-05	6	0	0
HBG1	0.771873	0.863244	-0.09137	-0.15901	1.32E-06	2.05E-05	2	0	0
DEFB103A	0.40587	0.570202	-0.16433	-0.48042	1.32E-06	2.06E-05	1	0	0
PGR	0.705265	0.830919	-0.12565	-0.23827	1.33E-06	2.07E-05	5	0	0
KRT17	0.435321	0.51048	-0.07516	-0.24923	1.34E-06	2.08E-05	5	0	0
OIT3	0.613608	0.523569	0.090039	0.229626	1.34E-06	2.08E-05	5	0	0
FOLR1	0.728396	0.672319	0.056077	0.127404	1.35E-06	2.09E-05	4	0	0
CLCA2	0.772323	0.847782	-0.07546	-0.13828	1.36E-06	2.11E-05	4	0	0
LGALS12	0.532009	0.58432	-0.05231	-0.14256	1.37E-06	2.12E-05	9	0	0
GPR78	0.762581	0.838512	-0.07593	-0.13517	1.37E-06	2.12E-05	2	0	0
CALN1	0.631529	0.725247	-0.09372	-0.18696	1.37E-06	2.12E-05	5	0	0
CLEC6A	0.592579	0.720251	-0.12767	-0.2942	1.37E-06	2.12E-05	2	0	0
CLEC7A	0.627312	0.731637	-0.10433	-0.23626	1.37E-06	2.12E-05	5	0	0
ACPP	0.363339	0.452309	-0.08897	-0.31874	1.37E-06	2.12E-05	5	0	0
PRTN3	0.385526	0.464837	-0.07931	-0.26072	1.38E-06	2.13E-05	6	0	0
SLC34A2	0.240751	0.129017	0.111735	1.203838	1.38E-06	2.13E-05	8	0	0
FTMT	0.868273	0.926248	-0.05797	-0.09447	1.39E-06	2.13E-05	9	0	0
BOLL	0.395984	0.292566	0.103418	0.953251	1.40E-06	2.16E-05	14	0	0

OR10G7	0.620928	0.720046	-0.09912	-0.23275	1.41E-06	2.17E-05	3	0	0
MYO6	0.307457	0.405628	-0.09817	-0.32069	1.42E-06	2.18E-05	3	0	0
OR7D2	0.666822	0.76643	-0.09961	-0.2049	1.43E-06	2.18E-05	2	0	0
DEFB125	0.534312	0.687217	-0.15291	-0.35702	1.43E-06	2.18E-05	2	0	0
C1orf158	0.570478	0.675804	-0.10533	-0.25238	1.43E-06	2.18E-05	6	0	0
ONECUT1	0.260548	0.162932	0.097617	0.920318	1.43E-06	2.18E-05	8	0	0
NLRP13	0.609456	0.75961	-0.15015	-0.31266	1.43E-06	2.18E-05	3	0	0
ASCC2	0.057422	0.094627	-0.03721	-0.38317	1.43E-06	2.19E-05	6	0	0
OR7G2	0.570169	0.698019	-0.12785	-0.28523	1.44E-06	2.19E-05	4	0	0
IL10	0.641732	0.752037	-0.1103	-0.23847	1.45E-06	2.20E-05	7	0	0
ZNF582	0.278258	0.158765	0.119493	1.298293	1.45E-06	2.20E-05	14	0	0
ERVFRD-1	0.636602	0.569722	0.06688	0.199029	1.45E-06	2.20E-05	4	0	0
TMEM95	0.721615	0.815935	-0.09432	-0.17846	1.45E-06	2.21E-05	4	0	0
OR2Y1	0.658269	0.758217	-0.09995	-0.20642	1.47E-06	2.23E-05	5	0	0
SERPINB3	0.593006	0.711977	-0.11897	-0.32642	1.48E-06	2.24E-05	2	0	0
BCKDHA	0.758664	0.80927	-0.05061	-0.10612	1.48E-06	2.25E-05	2	0	0
NAGK	0.574717	0.675794	-0.10108	-0.25672	1.49E-06	2.26E-05	3	0	0
MTIF2	0.2481	0.314741	-0.06664	-0.28536	1.51E-06	2.29E-05	8	0	0
TMPRSS11	0.659913	0.765027	-0.10511	-0.2127	1.54E-06	2.32E-05	4	0	0
PITPNM2	0.410155	0.339333	0.070822	0.162369	1.54E-06	2.32E-05	5	0	0
KCNJ15	0.685436	0.809459	-0.12402	-0.23671	1.54E-06	2.33E-05	2	0	0
OR13D1	0.666871	0.783511	-0.11664	-0.22958	1.55E-06	2.34E-05	3	0	0
BRINP1	0.286207	0.179151	0.107056	0.719685	1.55E-06	2.34E-05	5	0	0
C19orf81	0.384606	0.511711	-0.12711	-0.36999	1.56E-06	2.35E-05	5	0	0
KCNIP4	0.259735	0.15559	0.104145	0.856168	1.58E-06	2.38E-05	10	0	0
KRTAP20-1	0.626799	0.720978	-0.09418	-0.22023	1.58E-06	2.38E-05	4	0	0
ALDH3B1	0.611219	0.67169	-0.06047	-0.15637	1.59E-06	2.38E-05	10	0	0
FAM216B	0.612047	0.632777	-0.02073	-0.0938	1.59E-06	2.39E-05	6	0	0
MARCO	0.541669	0.632279	-0.09061	-0.27355	1.60E-06	2.39E-05	8	0	0
TMPRSS4	0.529954	0.656924	-0.12697	-0.30154	1.60E-06	2.39E-05	4	0	0
ADAM7	0.684675	0.786251	-0.10158	-0.20256	1.61E-06	2.40E-05	7	0	0
RNASE12	0.714334	0.831324	-0.11699	-0.21573	1.61E-06	2.40E-05	8	0	0
XKR3	0.704993	0.850774	-0.14578	-0.26771	1.61E-06	2.40E-05	1	0	0
OR1L8	0.686879	0.777738	-0.09086	-0.17931	1.61E-06	2.41E-05	4	0	0
FAM71B	0.634599	0.75047	-0.11587	-0.26307	1.61E-06	2.41E-05	8	0	0
CEACAM2'	0.373538	0.433866	-0.06033	0.101983	1.64E-06	2.44E-05	5	0	0
IL1RL1	0.745871	0.847308	-0.10144	-0.18706	1.65E-06	2.45E-05	3	0	0
IL18R1	0.745871	0.847308	-0.10144	-0.18706	1.65E-06	2.45E-05	3	0	0
ABCG2	0.59178	0.504578	0.087202	0.261909	1.65E-06	2.45E-05	8	0	0
MMP1	0.722365	0.786795	-0.06443	-0.13657	1.66E-06	2.47E-05	3	0	0
PMFBP1	0.757017	0.869102	-0.11209	-0.20021	1.66E-06	2.47E-05	2	0	0
IFNA14	0.702585	0.810684	-0.1081	-0.21976	1.67E-06	2.47E-05	4	0	0
WDFY4	0.496052	0.581104	-0.08505	-0.24967	1.68E-06	2.48E-05	12	0	0
EMP3	0.268527	0.322111	-0.05358	-0.34395	1.68E-06	2.48E-05	10	0	0
SORCS1	0.358116	0.290701	0.067414	0.767068	1.68E-06	2.49E-05	10	0	0
GPX2	0.545344	0.668203	-0.12286	-0.3015	1.69E-06	2.50E-05	7	0	0
APOA1	0.755324	0.805644	-0.05032	-0.09509	1.70E-06	2.50E-05	9	0	0
BANF2	0.678397	0.788559	-0.11016	-0.25766	1.72E-06	2.54E-05	7	0	0
WDR35	0.082818	0.097178	-0.01436	-0.27047	1.73E-06	2.55E-05	14	0	0
DEFB114	0.57619	0.749649	-0.17346	-0.37396	1.74E-06	2.56E-05	1	0	0
CCDC36	0.753587	0.82192	-0.06833	-0.13115	1.75E-06	2.57E-05	7	0	0
IFNA1	0.843316	0.884528	-0.04121	-0.06805	1.75E-06	2.57E-05	1	0	0
PPBP	0.697265	0.735293	-0.03803	-0.0718	1.75E-06	2.57E-05	6	0	0
ACTBL2	0.679267	0.769525	-0.09026	-0.19237	1.76E-06	2.59E-05	7	0	0
AP1S1	0.323767	0.365228	-0.04146	-0.21383	1.77E-06	2.59E-05	10	0	0

C9orf92	0.723874	0.811015	-0.08714	-0.15894	1.78E-06	2.60E-05	5	0	0
CCL16	0.660106	0.742791	-0.08269	-0.1721	1.78E-06	2.60E-05	9	0	0
OR13A1	0.756287	0.857592	-0.1013	-0.18154	1.78E-06	2.60E-05	4	0	0
ACMSD	0.591018	0.72984	-0.13882	-0.30682	1.79E-06	2.62E-05	3	0	0
ZSCAN23	0.296172	0.234996	0.061175	1.005061	1.80E-06	2.62E-05	9	0	0
HRH4	0.708858	0.796118	-0.08726	-0.17068	1.80E-06	2.62E-05	5	0	0
FPR2	0.542333	0.685238	-0.14291	-0.33252	1.80E-06	2.63E-05	2	0	0
FBLIM1	0.395418	0.459836	-0.06442	-0.29025	1.81E-06	2.64E-05	4	0	0
SLC22A8	0.486596	0.474469	0.012128	0.108989	1.82E-06	2.65E-05	7	0	0
RXFP3	0.158969	0.072444	0.086526	1.079004	1.85E-06	2.68E-05	12	0	0
COL9A1	0.594639	0.694735	-0.1001	-0.25386	1.85E-06	2.68E-05	7	0	0
TUSC1	0.579009	0.636144	-0.05714	-0.09502	1.85E-06	2.69E-05	4	0	0
EMILIN1	0.577072	0.512129	0.064943	0.196765	1.86E-06	2.69E-05	10	0	0
FAM181A	0.754115	0.840385	-0.08627	-0.15777	1.86E-06	2.70E-05	5	0	0
EBI3	0.177691	0.237658	-0.05997	-0.23707	1.88E-06	2.72E-05	3	0	0
GALNT15	0.464586	0.422044	0.042542	0.216051	1.88E-06	2.72E-05	10	0	0
OR8K1	0.626869	0.746158	-0.11929	-0.26201	1.89E-06	2.73E-05	4	0	0
PAPLN	0.313881	0.373169	-0.05929	-0.25277	1.89E-06	2.73E-05	9	0	0
CC2D1A	0.214333	0.144536	0.069797	0.381565	1.89E-06	2.73E-05	6	0	0
HIST1H4F	0.566548	0.491621	0.074927	0.782803	1.90E-06	2.75E-05	8	0	0
OVCH2	0.310083	0.419894	-0.10981	-0.44294	1.91E-06	2.75E-05	3	0	0
CBFB	0.084622	0.126975	-0.04235	-0.19911	1.92E-06	2.77E-05	11	0	0
ADAM2	0.798197	0.861932	-0.06373	-0.1101	1.94E-06	2.78E-05	7	0	0
OR51V1	0.581187	0.695949	-0.11476	-0.24755	1.94E-06	2.78E-05	3	0	0
COX6A2	0.632543	0.623679	0.008864	0.003172	1.94E-06	2.78E-05	5	0	0
TRAPPC12	0.29914	0.343546	-0.04441	-0.27983	1.95E-06	2.79E-05	8	0	0
PPP2R2B	0.678386	0.817407	-0.13902	-0.26538	1.95E-06	2.79E-05	1	0	0
OR10S1	0.586947	0.728841	-0.14189	-0.31116	1.95E-06	2.79E-05	2	0	0
RNASE2	0.444843	0.586678	-0.14183	-0.39158	1.95E-06	2.80E-05	1	0	0
GPR65	0.385242	0.492255	-0.10701	-0.28456	1.97E-06	2.81E-05	2	0	0
HDGFL1	0.820681	0.836391	-0.01571	-0.02572	1.97E-06	2.82E-05	7	0	0
FCGR3A	0.581866	0.70776	-0.12589	-0.27823	1.97E-06	2.82E-05	1	0	0
CUBN	0.792853	0.890353	-0.0975	-0.17617	1.98E-06	2.83E-05	3	0	0
ALX1	0.237628	0.113501	0.124127	1.121353	1.99E-06	2.83E-05	13	0	0
NCKAP1L	0.508123	0.637578	-0.12946	-0.3267	1.99E-06	2.84E-05	5	0	0
ZNF90	0.283449	0.116163	0.167286	1.217824	2.00E-06	2.84E-05	1	0	0
DES	0.542643	0.459368	0.083275	0.359367	2.01E-06	2.85E-05	9	0	0
OLIG3	0.265124	0.181839	0.083285	0.539239	2.01E-06	2.85E-05	9	0	0
OR9Q1	0.709629	0.747859	-0.03823	-0.05335	2.01E-06	2.85E-05	7	0	0
OR1C1	0.533306	0.659109	-0.1258	-0.28714	2.01E-06	2.86E-05	6	0	0
MAGI2	0.185325	0.10344	0.081886	0.779207	2.02E-06	2.86E-05	15	0	0
CETP	0.643963	0.563255	0.080709	0.190353	2.02E-06	2.86E-05	7	0	0
WFDC9	0.720509	0.829555	-0.10905	-0.20121	2.02E-06	2.86E-05	5	0	0
DNALI1	0.457717	0.36089	0.096827	0.507794	2.02E-06	2.86E-05	12	0	0
S100A10	0.237445	0.302829	-0.06538	-0.31011	2.02E-06	2.86E-05	10	0	0
ADCY2	0.233793	0.164434	0.069359	0.527534	2.05E-06	2.89E-05	6	0	0
PLA2G2F	0.726581	0.785662	-0.05908	-0.12326	2.05E-06	2.90E-05	7	0	0
GALR2	0.331133	0.226826	0.104307	0.910236	2.06E-06	2.90E-05	7	0	0
SSH3	0.206732	0.152249	0.054482	0.576909	2.06E-06	2.91E-05	13	0	0
TNFRSF10/	0.166268	0.230389	-0.06412	-0.2607	2.07E-06	2.91E-05	12	0	0
CLC	0.516806	0.641429	-0.12462	-0.33105	2.07E-06	2.91E-05	4	0	0
GKN1	0.7935	0.879044	-0.08554	-0.14974	2.07E-06	2.91E-05	2	0	0
OR10A4	0.620288	0.725556	-0.10527	-0.22019	2.08E-06	2.91E-05	3	0	0
UNC13D	0.19484	0.223391	-0.02855	-0.29787	2.09E-06	2.93E-05	10	0	0
XBP1	0.314673	0.418429	-0.10376	-0.34064	2.09E-06	2.94E-05	8	0	0

HOXC6	0.208576	0.095809	0.112767	1.061083	2.10E-06	2.94E-05	8	0	0
FCRL6	0.583874	0.684468	-0.10059	-0.23284	2.12E-06	2.97E-05	6	0	0
SECISBP2L	0.224467	0.261629	-0.03716	-0.3263	2.13E-06	2.98E-05	13	0	0
PRSS56	0.629492	0.569821	0.059671	0.147111	2.14E-06	2.98E-05	5	0	0
HRH1	0.607314	0.706518	-0.0992	-0.22177	2.14E-06	2.98E-05	10	0	0
TCERG1L	0.230761	0.185472	0.045288	0.744649	2.14E-06	2.98E-05	5	0	0
KRT28	0.580875	0.668799	-0.08792	-0.20169	2.15E-06	3.00E-05	4	0	0
PPM1N	0.701931	0.802705	-0.10077	-0.19343	2.15E-06	3.00E-05	4	0	0
PROX1	0.225933	0.126523	0.09941	0.862345	2.17E-06	3.02E-05	4	0	0
C15orf40	0.271747	0.343244	-0.0715	-0.27731	2.17E-06	3.02E-05	9	0	0
RIT2	0.633789	0.759671	-0.12588	-0.26024	2.17E-06	3.02E-05	4	0	0
SFT2D3	0.486783	0.576032	-0.08925	-0.22507	2.18E-06	3.03E-05	12	0	0
CD4	0.786961	0.737669	0.049292	0.100473	2.18E-06	3.03E-05	3	0	0
LPAR2	0.32095	0.38266	-0.06171	-0.22692	2.20E-06	3.05E-05	8	0	0
SLC7A14	0.39036	0.375195	0.015164	0.244246	2.21E-06	3.06E-05	8	0	0
F11	0.76036	0.825681	-0.06532	-0.12214	2.21E-06	3.06E-05	8	0	0
EDDM3B	0.600413	0.716949	-0.11654	-0.25207	2.21E-06	3.06E-05	1	0	0
RFX8	0.231832	0.3108	-0.07897	-0.27253	2.22E-06	3.06E-05	12	0	0
FGF3	0.196315	0.110759	0.085556	0.907607	2.24E-06	3.09E-05	14	0	0
FAM155A	0.147138	0.069627	0.077511	1.060066	2.23E-06	3.09E-05	12	0	0
MIER3	0.760168	0.84805	-0.08788	-0.15685	2.24E-06	3.09E-05	2	0	0
MYH4	0.693424	0.80487	-0.11145	-0.22945	2.24E-06	3.09E-05	6	0	0
PLD1	0.357709	0.453438	-0.09573	-0.22342	2.25E-06	3.09E-05	6	0	0
ACAN	0.318389	0.293616	0.024773	0.577144	2.25E-06	3.09E-05	9	0	0
PECR	0.614174	0.725762	-0.11159	-0.25063	2.26E-06	3.10E-05	4	0	0
DCLK3	0.79105	0.856356	-0.06531	-0.11589	2.28E-06	3.13E-05	3	0	0
IRX1	0.288506	0.192356	0.09615	0.539118	2.28E-06	3.13E-05	5	0	0
CACNA2D4	0.615686	0.71195	-0.09626	-0.21588	2.28E-06	3.13E-05	7	0	0
TTC22	0.096088	0.126866	-0.03078	-0.22009	2.29E-06	3.14E-05	9	0	0
TMEM215	0.154113	0.090621	0.063492	0.75268	2.30E-06	3.16E-05	7	0	0
DNAJB13	0.731462	0.805599	-0.07414	-0.18808	2.31E-06	3.16E-05	3	0	0
PAX4	0.509816	0.60813	-0.09831	-0.21443	2.32E-06	3.17E-05	9	0	0
TRIM77	0.694017	0.801032	-0.10701	-0.20415	2.32E-06	3.17E-05	2	0	0
ABHD12B	0.463385	0.449449	0.013936	0.211904	2.32E-06	3.17E-05	7	0	0
MKRN3	0.677111	0.820574	-0.14346	-0.27843	2.33E-06	3.17E-05	10	0	0
ADSSL1	0.252842	0.297181	-0.04434	-0.0423	2.33E-06	3.18E-05	7	0	0
TGM5	0.563347	0.666229	-0.10288	-0.23587	2.33E-06	3.18E-05	6	0	0
FTH1	0.068231	0.103543	-0.03531	-0.27078	2.34E-06	3.18E-05	8	0	0
RHNO1	0.427158	0.524092	-0.09693	-0.26734	2.34E-06	3.18E-05	4	0	0
ARHGAP32	0.08927	0.147456	-0.05819	-0.31846	2.35E-06	3.19E-05	6	0	0
SCNN1D	0.795452	0.870425	-0.07497	-0.12794	2.35E-06	3.19E-05	8	0	0
TNS4	0.603783	0.6827	-0.07892	-0.19253	2.35E-06	3.19E-05	10	0	0
ABCG1	0.759176	0.838835	-0.07966	-0.1476	2.37E-06	3.22E-05	3	0	0
FAM69B	0.108797	0.158203	-0.04941	-0.13979	2.39E-06	3.24E-05	5	0	0
TRIM34	0.779619	0.853274	-0.07365	-0.13717	2.40E-06	3.24E-05	4	0	0
ENTPD2	0.431483	0.488711	-0.05723	-0.27497	2.40E-06	3.25E-05	5	0	0
KRTAP21-3	0.744168	0.841008	-0.09684	-0.18855	2.40E-06	3.25E-05	4	0	0
ZIC5	0.281726	0.189577	0.092149	0.67221	2.43E-06	3.28E-05	10	0	0
PODN	0.16336	0.094314	0.069046	0.809089	2.44E-06	3.29E-05	9	0	0
UBXN2B	0.298621	0.315829	-0.01721	-0.29266	2.44E-06	3.29E-05	7	0	0
IFIT1B	0.816762	0.871863	-0.0551	-0.09546	2.44E-06	3.29E-05	4	0	0
NPSR1	0.605122	0.710745	-0.10562	-0.23235	2.45E-06	3.29E-05	7	0	0
OR52N2	0.637649	0.746466	-0.10882	-0.23414	2.45E-06	3.30E-05	5	0	0
DLGAP2	0.677598	0.771274	-0.09368	-0.18412	2.46E-06	3.31E-05	10	0	0
RGL3	0.193377	0.088799	0.104579	1.124772	2.47E-06	3.31E-05	9	0	0

OR51L1	0.578642	0.690607	-0.11196	-0.26661	2.47E-06	3.32E-05	7	0	0
NKX2-4	0.275807	0.171112	0.104695	0.718675	2.50E-06	3.35E-05	11	0	0
TNNI1	0.695344	0.784181	-0.08884	-0.17114	2.50E-06	3.35E-05	1	0	0
HLA-DRA	0.358235	0.417445	-0.05921	-0.38078	2.51E-06	3.36E-05	3	0	0
ATP13A5	0.687076	0.810741	-0.12367	-0.26386	2.51E-06	3.36E-05	8	0	0
OR4B1	0.696854	0.80527	-0.10842	-0.21012	2.52E-06	3.37E-05	3	0	0
PTPN22	0.487088	0.629857	-0.14277	-0.4024	2.57E-06	3.43E-05	6	0	0
NID2	0.270149	0.256819	0.01333	0.526349	2.57E-06	3.43E-05	13	0	0
CLUL1	0.223192	0.278913	-0.05572	-0.28934	2.57E-06	3.44E-05	7	0	0
ATP2C1	0.1416	0.196509	-0.05491	-0.36806	2.59E-06	3.45E-05	9	0	0
SIGLEC8	0.493353	0.597729	-0.10438	-0.28518	2.59E-06	3.46E-05	3	0	0
CCDC74A	0.732706	0.844628	-0.11192	-0.19755	2.62E-06	3.48E-05	2	0	0
MEF2C	0.669987	0.794678	-0.12469	-0.24291	2.62E-06	3.49E-05	1	0	0
TNFRSF13I	0.694833	0.762201	-0.06737	-0.13335	2.62E-06	3.49E-05	4	0	0
C20orf202	0.619365	0.663318	-0.04395	-0.0102	2.64E-06	3.50E-05	5	0	0
CCDC34	0.428665	0.514827	-0.08616	-0.27074	2.64E-06	3.50E-05	7	0	0
RPS5	0.370226	0.429474	-0.05925	-0.04184	2.64E-06	3.50E-05	3	0	0
BHMT	0.590838	0.532273	0.058565	0.277423	2.65E-06	3.51E-05	12	0	0
PARP16	0.676503	0.796276	-0.11977	-0.23199	2.65E-06	3.51E-05	2	0	0
EPPIN-WFI	0.60664	0.736747	-0.13011	-0.2766	2.66E-06	3.52E-05	5	0	0
EPPIN	0.60664	0.736747	-0.13011	-0.2766	2.66E-06	3.52E-05	5	0	0
SEMG2	0.797626	0.889513	-0.09189	-0.15661	2.67E-06	3.52E-05	5	0	0
CCDC68	0.33491	0.255364	0.079546	0.819771	2.68E-06	3.54E-05	9	0	0
SLURP1	0.722971	0.809281	-0.08631	-0.17822	2.69E-06	3.54E-05	6	0	0
SFTPD	0.470528	0.408347	0.06218	0.199918	2.68E-06	3.54E-05	1	0	0
F2RL3	0.284225	0.179174	0.105051	1.088208	2.69E-06	3.55E-05	10	0	0
PRSS37	0.82311	0.877024	-0.05391	-0.09048	2.70E-06	3.55E-05	3	0	0
KRT27	0.748117	0.836724	-0.08861	-0.16523	2.71E-06	3.57E-05	8	0	0
PPP1R17	0.622216	0.711872	-0.08966	-0.18015	2.71E-06	3.57E-05	9	0	0
SOGA3	0.188994	0.118046	0.070949	0.686058	2.72E-06	3.57E-05	14	0	0
DSG3	0.611027	0.736271	-0.12524	-0.29362	2.72E-06	3.57E-05	8	0	0
ZNF667	0.299557	0.189852	0.109705	0.931429	2.73E-06	3.58E-05	5	0	0
OR51A2	0.614374	0.748984	-0.13461	-0.28263	2.74E-06	3.59E-05	3	0	0
OR4D2	0.527223	0.650631	-0.12341	-0.28667	2.75E-06	3.60E-05	5	0	0
TMEM240	0.5928	0.507268	0.085532	0.862246	2.75E-06	3.60E-05	12	0	0
RXFP4	0.50834	0.4216	0.086739	0.247535	2.76E-06	3.60E-05	6	0	0
CD8A	0.187461	0.158733	0.028728	0.089091	2.78E-06	3.62E-05	10	0	0
SPON2	0.771567	0.731837	0.03973	0.087955	2.78E-06	3.62E-05	20	0	0
AHR	0.294046	0.314086	-0.02004	-0.12123	2.77E-06	3.62E-05	8	0	0
SUN3	0.732823	0.826132	-0.09331	-0.18209	2.79E-06	3.64E-05	9	0	0
SEMG1	0.500765	0.650741	-0.14998	-0.39526	2.80E-06	3.64E-05	2	0	0
OR51Q1	0.57391	0.696596	-0.12269	-0.26793	2.83E-06	3.68E-05	4	0	0
RRH	0.823851	0.803371	0.02048	0.032444	2.84E-06	3.70E-05	5	0	0
FEZF2	0.168503	0.101282	0.067221	0.68041	2.86E-06	3.71E-05	11	0	0
CNGB3	0.463545	0.583405	-0.11986	-0.30933	2.88E-06	3.73E-05	5	0	0
SLC39A12	0.618156	0.738339	-0.12018	-0.21924	2.88E-06	3.73E-05	6	0	0
IGDCC3	0.389298	0.30934	0.079958	0.504152	2.88E-06	3.73E-05	4	0	0
IFNA6	0.611532	0.70929	-0.09776	-0.21074	2.89E-06	3.74E-05	1	0	0
NNMT	0.574595	0.597586	-0.02299	-0.02391	2.89E-06	3.74E-05	5	0	0
PHLPP2	0.689549	0.729751	-0.0402	-0.15121	2.89E-06	3.74E-05	7	0	0
OR2W1	0.472508	0.616513	-0.144	-0.3765	2.90E-06	3.76E-05	4	0	0
HES3	0.255964	0.182409	0.073554	0.543268	2.92E-06	3.77E-05	11	0	0
SLC6A3	0.287772	0.189905	0.097867	0.881572	2.95E-06	3.81E-05	7	0	0
ATP12A	0.152626	0.191674	-0.03905	-0.16094	2.96E-06	3.81E-05	10	0	0
SCNM1	0.384707	0.450393	-0.06569	-0.26183	2.96E-06	3.82E-05	5	0	0

LGALS2	0.651792	0.742548	-0.09076	-0.18541	2.96E-06	3.82E-05	1	0	0
CALML5	0.699643	0.763359	-0.06372	-0.13294	2.97E-06	3.83E-05	7	0	0
RNASE1	0.641662	0.567901	0.073761	0.208814	2.99E-06	3.84E-05	10	0	0
FA2H	0.162508	0.201519	-0.03901	-0.15116	2.99E-06	3.84E-05	6	0	0
CACNG8	0.266491	0.165435	0.101057	0.843203	2.99E-06	3.84E-05	9	0	0
CC2D2A	0.268568	0.215612	0.052955	0.667736	2.99E-06	3.84E-05	12	0	0
THRB	0.175189	0.219195	-0.04401	-0.32599	3.01E-06	3.86E-05	19	0	0
AOC1	0.754812	0.843098	-0.08829	-0.15811	3.02E-06	3.87E-05	2	0	0
IL31RA	0.748561	0.825868	-0.07731	-0.14211	3.04E-06	3.89E-05	5	0	0
HMHB1	0.665551	0.745971	-0.08042	-0.16073	3.04E-06	3.89E-05	5	0	0
KRT26	0.654483	0.744177	-0.08969	-0.19907	3.04E-06	3.89E-05	7	0	0
TRIML2	0.627412	0.746756	-0.11934	-0.2476	3.05E-06	3.90E-05	1	0	0
GPR87	0.635965	0.757968	-0.122	-0.27599	3.07E-06	3.91E-05	5	0	0
MSH6	0.802197	0.886596	-0.0844	-0.14263	3.07E-06	3.92E-05	1	0	0
KIF15	0.125036	0.146909	-0.02187	-0.32716	3.10E-06	3.95E-05	13	0	0
KCNH5	0.736495	0.819724	-0.08323	-0.15612	3.10E-06	3.95E-05	6	0	0
ASB10	0.55713	0.631919	-0.07479	-0.18978	3.10E-06	3.95E-05	14	0	0
OSGIN1	0.820491	0.87862	-0.05813	-0.10516	3.11E-06	3.95E-05	4	0	0
SOX14	0.214349	0.129026	0.085323	0.764615	3.12E-06	3.97E-05	16	0	0
OR10A7	0.696046	0.797459	-0.10141	-0.19333	3.15E-06	4.00E-05	4	0	0
RHO	0.787565	0.842827	-0.05526	-0.10028	3.17E-06	4.02E-05	7	0	0
TNFSF8	0.440469	0.555069	-0.1146	-0.34456	3.17E-06	4.03E-05	8	0	0
CLEC5A	0.502038	0.598316	-0.09628	-0.2569	3.18E-06	4.03E-05	5	0	0
CDH26	0.710376	0.832263	-0.12189	-0.22616	3.20E-06	4.06E-05	4	0	0
OR51E1	0.578682	0.674765	-0.09608	-0.19761	3.22E-06	4.07E-05	3	0	0
HBD	0.835118	0.885987	-0.05087	-0.08486	3.22E-06	4.07E-05	3	0	0
OR5AN1	0.596064	0.757427	-0.16136	-0.33764	3.22E-06	4.07E-05	2	0	0
MYOG	0.77327	0.815018	-0.04175	-0.07555	3.24E-06	4.10E-05	10	0	0
SLC38A4	0.498752	0.376883	0.121868	0.471287	3.26E-06	4.12E-05	6	0	0
APOC3	0.615503	0.651643	-0.03614	-0.06497	3.29E-06	4.15E-05	6	0	0
LCE1A	0.706748	0.815597	-0.10885	-0.20303	3.31E-06	4.18E-05	3	0	0
LSMEM1	0.811521	0.761505	0.050016	0.101141	3.32E-06	4.18E-05	12	0	0
OR4F6	0.634054	0.761038	-0.12698	-0.26665	3.34E-06	4.21E-05	4	0	0
DGKB	0.788719	0.874606	-0.08589	-0.14735	3.35E-06	4.21E-05	1	0	0
CPNE6	0.570908	0.651153	-0.08024	-0.18377	3.37E-06	4.23E-05	13	0	0
CXCR6	0.382744	0.431238	-0.04849	-0.22878	3.41E-06	4.27E-05	2	0	0
MOS	0.453026	0.391052	0.061974	0.465245	3.40E-06	4.27E-05	9	0	0
MMP13	0.796525	0.878387	-0.08186	-0.14829	3.41E-06	4.28E-05	9	0	0
NR2F2	0.228504	0.314486	-0.08598	-0.45606	3.41E-06	4.28E-05	8	0	0
PDCD1LG2	0.318128	0.371359	-0.05323	-0.33609	3.42E-06	4.28E-05	3	0	0
CCR10	0.722602	0.642244	0.080358	0.235863	3.42E-06	4.28E-05	5	0	0
HPD	0.837359	0.896855	-0.0595	-0.09754	3.44E-06	4.31E-05	2	0	0
OTOA	0.618256	0.737587	-0.11933	-0.2504	3.45E-06	4.31E-05	2	0	0
OR56A4	0.542654	0.643822	-0.10117	-0.26043	3.46E-06	4.33E-05	5	0	0
IL18RAP	0.603509	0.699393	-0.09588	-0.28285	3.47E-06	4.33E-05	6	0	0
RIOK3	0.261175	0.291146	-0.02997	-0.03205	3.47E-06	4.33E-05	9	0	0
CD300LB	0.535247	0.631869	-0.09662	-0.27071	3.49E-06	4.35E-05	3	0	0
SLC15A2	0.718901	0.641344	0.077557	0.210675	3.50E-06	4.36E-05	6	0	0
KRTAP5-8	0.863119	0.928907	-0.06579	-0.10638	3.50E-06	4.36E-05	2	0	0
DLX5	0.304161	0.195462	0.108699	0.61178	3.51E-06	4.37E-05	15	0	0
NT5C1B-RI	0.832251	0.89774	-0.06549	-0.11245	3.55E-06	4.40E-05	2	0	0
NT5C1B	0.832251	0.89774	-0.06549	-0.11245	3.55E-06	4.40E-05	2	0	0
OR4K5	0.62831	0.738706	-0.1104	-0.22774	3.56E-06	4.41E-05	4	0	0
HLA-DPA1	0.642826	0.553254	0.089572	0.243491	3.56E-06	4.41E-05	10	0	0
YPEL4	0.415204	0.38379	0.031413	0.147792	3.56E-06	4.41E-05	4	0	0

KRT18	0.535394	0.579657	-0.04426	-0.24591	3.57E-06	4.42E-05	7	0	0
GSTM5	0.522568	0.376095	0.146474	0.472781	3.58E-06	4.43E-05	4	0	0
OR1A2	0.57921	0.686106	-0.1069	-0.24137	3.59E-06	4.44E-05	5	0	0
HOXB8	0.267118	0.15587	0.111248	0.858151	3.60E-06	4.44E-05	14	0	0
CALB1	0.684081	0.790868	-0.10679	-0.2125	3.62E-06	4.47E-05	2	0	0
HSPA12B	0.406992	0.363516	0.043476	0.544425	3.62E-06	4.47E-05	6	0	0
ANO4	0.229247	0.155611	0.073636	0.579241	3.65E-06	4.50E-05	6	0	0
GJD2	0.181206	0.077694	0.103512	1.15612	3.65E-06	4.50E-05	8	0	0
IRF8	0.186181	0.199109	-0.01293	0.280895	3.65E-06	4.50E-05	4	0	0
NEK6	0.502363	0.5709	-0.06854	-0.1566	3.68E-06	4.52E-05	9	0	0
PRSS38	0.816353	0.876557	-0.0602	-0.10157	3.69E-06	4.54E-05	5	0	0
LILRB4	0.323884	0.413253	-0.08937	-0.34218	3.71E-06	4.56E-05	1	0	0
ANKRD9	0.091586	0.126956	-0.03537	-0.15605	3.72E-06	4.56E-05	9	0	0
CLEC4E	0.462876	0.58454	-0.12166	-0.31169	3.75E-06	4.60E-05	3	0	0
LGALS7B	0.63829	0.746657	-0.10837	-0.21786	3.75E-06	4.60E-05	5	0	0
C6orf58	0.22407	0.160081	0.063989	0.639499	3.77E-06	4.62E-05	16	0	0
LCNL1	0.722104	0.679253	0.042851	0.109337	3.81E-06	4.66E-05	4	0	0
OR4C15	0.424364	0.521357	-0.09699	-0.29811	3.82E-06	4.67E-05	3	0	0
C20orf141	0.705485	0.776031	-0.07055	-0.15579	3.82E-06	4.68E-05	5	0	0
OR7C2	0.737477	0.847266	-0.10979	-0.19966	3.85E-06	4.71E-05	3	0	0
CHST11	0.820223	0.896214	-0.07599	-0.12789	3.86E-06	4.71E-05	2	0	0
PAIP1	0.281306	0.326134	-0.04483	-0.13542	3.90E-06	4.76E-05	12	0	0
ZEB1	0.089549	0.124199	-0.03465	-0.23816	3.91E-06	4.76E-05	10	0	0
FXYD2	0.463989	0.53451	-0.07052	-0.25816	3.93E-06	4.79E-05	8	0	0
NEFH	0.304854	0.207719	0.097135	0.735595	3.93E-06	4.79E-05	8	0	0
CYP21A2	0.662259	0.790651	-0.12839	-0.25216	3.94E-06	4.79E-05	1	0	0
RNASE7	0.667234	0.768677	-0.10144	-0.21068	3.95E-06	4.80E-05	7	0	0
INHA	0.809438	0.859573	-0.05013	-0.08642	3.96E-06	4.81E-05	4	0	0
FRZB	0.217583	0.155092	0.062491	0.839785	3.96E-06	4.81E-05	10	0	0
KRT14	0.70509	0.780305	-0.07521	-0.14528	3.97E-06	4.82E-05	3	0	0
ERICH2	0.816315	0.865626	-0.04931	-0.0837	4.00E-06	4.85E-05	3	0	0
MUC5AC	0.510081	0.592423	-0.08234	-0.21263	4.01E-06	4.86E-05	6	0	0
CHMP4C	0.256119	0.314958	-0.05884	-0.29167	4.03E-06	4.88E-05	11	0	0
SMIM17	0.426882	0.345933	0.080949	0.391994	4.03E-06	4.88E-05	6	0	0
NME5	0.330542	0.254942	0.0756	0.707427	4.04E-06	4.88E-05	11	0	0
EPGN	0.582745	0.676131	-0.09339	-0.22579	4.04E-06	4.89E-05	6	0	0
RAG2	0.745939	0.823439	-0.0775	-0.14387	4.07E-06	4.91E-05	4	0	0
C4orf45	0.778294	0.85796	-0.07967	-0.13974	4.07E-06	4.91E-05	2	0	0
OR6K6	0.67931	0.805546	-0.12624	-0.24816	4.08E-06	4.93E-05	4	0	0
CHRNE	0.348992	0.381603	-0.03261	-0.24761	4.09E-06	4.93E-05	11	0	0
FOXC2	0.171563	0.091565	0.079999	0.915207	4.10E-06	4.93E-05	7	0	0
SLC22A6	0.592593	0.693973	-0.10138	-0.21965	4.12E-06	4.95E-05	7	0	0
LRFN5	0.14722	0.078909	0.068311	0.831411	4.14E-06	4.98E-05	11	0	0
ADAM18	0.774198	0.85258	-0.07838	-0.14027	4.16E-06	5.00E-05	9	0	0
ANXA1	0.769464	0.845945	-0.07648	-0.13104	4.20E-06	5.04E-05	2	0	0
FAM187B	0.814874	0.894464	-0.07959	-0.13289	4.20E-06	5.04E-05	1	0	0
SLC9C2	0.511747	0.399073	0.112674	0.402303	4.21E-06	5.04E-05	4	0	0
MR1	0.426523	0.458565	-0.03204	-0.24062	4.21E-06	5.04E-05	6	0	0
OR10A3	0.700259	0.794656	-0.0944	-0.18089	4.22E-06	5.06E-05	4	0	0
SLC26A3	0.604937	0.735222	-0.13028	-0.27723	4.23E-06	5.06E-05	1	0	0
CCDC181	0.385571	0.26881	0.116761	0.727289	4.27E-06	5.11E-05	7	0	0
KRT6A	0.555087	0.667441	-0.11235	-0.2337	4.28E-06	5.11E-05	6	0	0
ENPP2	0.658549	0.609929	0.04862	0.140665	4.33E-06	5.16E-05	6	0	0
HBG2	0.613835	0.705008	-0.09117	-0.20335	4.32E-06	5.16E-05	3	0	0
PZP	0.723643	0.817883	-0.09424	-0.16967	4.33E-06	5.16E-05	3	0	0

CD1A	0.664157	0.781536	-0.11738	-0.23731	4.34E-06	5.17E-05	4	0	0
SPTLC3	0.468415	0.570039	-0.10162	-0.15459	4.34E-06	5.17E-05	6	0	0
AMTN	0.674503	0.777803	-0.1033	-0.2164	4.37E-06	5.20E-05	3	0	0
AVPR1A	0.219496	0.11858	0.100916	0.878744	4.37E-06	5.20E-05	11	0	0
TMEM155	0.315148	0.256988	0.058161	1.114463	4.38E-06	5.21E-05	9	0	0
KRTAP10-1	0.685003	0.781288	-0.09628	-0.19408	4.39E-06	5.22E-05	2	0	0
SPACA7	0.619929	0.725171	-0.10524	-0.2289	4.40E-06	5.22E-05	5	0	0
DMRT3	0.192901	0.110785	0.082116	0.755856	4.41E-06	5.24E-05	7	0	0
SMIM21	0.732557	0.823307	-0.09075	-0.17785	4.42E-06	5.25E-05	8	0	0
TAS2R40	0.709031	0.811717	-0.10269	-0.19979	4.44E-06	5.26E-05	5	0	0
EPHX2	0.306265	0.26447	0.041796	0.416205	4.44E-06	5.26E-05	7	0	0
MRGPRX3	0.648629	0.735671	-0.08704	-0.17944	4.45E-06	5.26E-05	2	0	0
NR2E1	0.245137	0.139143	0.105994	0.90325	4.45E-06	5.27E-05	16	0	0
SLC5A9	0.697858	0.772001	-0.07414	-0.14552	4.48E-06	5.29E-05	5	0	0
KAT2B	0.411372	0.288364	0.123008	0.592053	4.48E-06	5.29E-05	3	0	0
CALCA	0.255279	0.178203	0.077077	0.595455	4.48E-06	5.29E-05	33	0	0
TPRX1	0.810857	0.861607	-0.05075	-0.08655	4.50E-06	5.30E-05	1	0	0
CRX	0.810857	0.861607	-0.05075	-0.08655	4.50E-06	5.30E-05	1	0	0
DGAT2	0.576755	0.673201	-0.09645	-0.21955	4.51E-06	5.31E-05	1	0	0
RHOBTB3	0.787962	0.832248	-0.04429	-0.07861	4.52E-06	5.32E-05	3	0	0
C10orf120	0.61236	0.702881	-0.09052	-0.18406	4.52E-06	5.32E-05	6	0	0
BPIFA3	0.515678	0.651072	-0.13539	-0.32964	4.52E-06	5.32E-05	4	0	0
GABRA2	0.674659	0.778912	-0.10425	-0.22541	4.54E-06	5.34E-05	4	0	0
LPCAT1	0.326856	0.387966	-0.06111	-0.16128	4.57E-06	5.37E-05	5	0	0
NKX2-3	0.228639	0.138382	0.090257	0.957469	4.59E-06	5.39E-05	9	0	0
RAD23A	0.248552	0.316255	-0.0677	-0.19831	4.60E-06	5.40E-05	11	0	0
GRAMD4	0.692039	0.686159	0.00588	-0.00389	4.61E-06	5.40E-05	2	0	0
COL6A6	0.67291	0.761026	-0.08812	-0.16803	4.63E-06	5.42E-05	3	0	0
PCDHB15	0.372112	0.29365	0.078462	0.612617	4.64E-06	5.43E-05	13	0	0
NPEPL1	0.677499	0.631935	0.045564	0.131642	4.68E-06	5.47E-05	6	0	0
OSBPL9	0.652014	0.628435	0.02358	0.121856	4.69E-06	5.48E-05	2	0	0
MEDAG	0.518226	0.480028	0.038199	0.359197	4.69E-06	5.48E-05	9	0	0
SERPINB5	0.759834	0.854445	-0.09461	-0.19058	4.69E-06	5.48E-05	6	0	0
MMRN2	0.395157	0.317377	0.077779	0.325676	4.71E-06	5.50E-05	10	0	0
GORASP2	0.667702	0.774634	-0.10693	-0.24151	4.72E-06	5.50E-05	10	0	0
WFDC10B	0.793409	0.839115	-0.04571	-0.07517	4.72E-06	5.50E-05	3	0	0
TXNDC15	0.590679	0.619255	-0.02858	-0.08148	4.72E-06	5.50E-05	9	0	0
VWA3A	0.643956	0.598922	0.045034	0.166961	4.74E-06	5.52E-05	8	0	0
WFDC13	0.568393	0.655865	-0.08747	-0.22523	4.78E-06	5.56E-05	6	0	0
COL26A1	0.279209	0.190974	0.088235	0.841818	4.79E-06	5.57E-05	13	0	0
ADCYAP1	0.27788	0.167356	0.110524	0.941101	4.80E-06	5.58E-05	18	0	0
FAM53A	0.366842	0.429964	-0.06312	-0.12285	4.82E-06	5.60E-05	6	0	0
VGF	0.139918	0.128765	0.011153	0.069559	4.84E-06	5.62E-05	9	0	0
CYTH4	0.427703	0.488901	-0.0612	-0.25279	4.86E-06	5.64E-05	8	0	0
IFNA8	0.583335	0.718889	-0.13555	-0.29685	4.89E-06	5.67E-05	1	0	0
SST	0.359758	0.352068	0.00769	0.245876	4.91E-06	5.68E-05	14	0	0
PYHIN1	0.601368	0.747557	-0.14619	-0.31493	4.93E-06	5.70E-05	4	0	0
CC2D2B	0.753479	0.852731	-0.09925	-0.18326	4.98E-06	5.76E-05	3	0	0
AMPD1	0.681012	0.788877	-0.10786	-0.20976	5.02E-06	5.80E-05	3	0	0
DSC1	0.584411	0.684952	-0.10054	-0.22652	5.03E-06	5.80E-05	5	0	0
PAEP	0.519985	0.599051	-0.07907	-0.21023	5.04E-06	5.81E-05	3	0	0
OR52K2	0.490068	0.591735	-0.10167	-0.27945	5.04E-06	5.81E-05	3	0	0
ACTA1	0.237128	0.14427	0.092858	0.738052	5.10E-06	5.88E-05	9	0	0
TDRD7	0.496981	0.572412	-0.07543	-0.22005	5.12E-06	5.90E-05	5	0	0
RBMS2	0.228369	0.150522	0.077847	0.366814	5.13E-06	5.90E-05	7	0	0

PGK2	0.609051	0.705477	-0.09643	-0.21582	5.14E-06	5.91E-05	9	0	0
HNF1B	0.222226	0.101379	0.120847	1.095091	5.14E-06	5.91E-05	10	0	0
SCN2B	0.690751	0.682014	0.008738	0.074364	5.15E-06	5.91E-05	6	0	0
ZSCAN5A	0.266213	0.147618	0.118595	1.377593	5.15E-06	5.91E-05	13	0	0
RPL10L	0.656092	0.708924	-0.05283	-0.13151	5.21E-06	5.98E-05	2	0	0
PSMF1	0.768763	0.836859	-0.0681	-0.12255	5.21E-06	5.98E-05	5	0	0
TMPRSS7	0.822237	0.89041	-0.06817	-0.1172	5.22E-06	5.98E-05	5	0	0
CHST3	0.407053	0.349668	0.057385	0.185436	5.24E-06	6.00E-05	4	0	0
MOGAT2	0.679829	0.746824	-0.06699	-0.13864	5.24E-06	6.00E-05	8	0	0
OSTN	0.514015	0.682919	-0.1689	-0.40308	5.31E-06	6.07E-05	1	0	0
USP10	0.327731	0.380843	-0.05311	-0.22276	5.31E-06	6.07E-05	8	0	0
CGB5	0.607452	0.72641	-0.11896	-0.25418	5.34E-06	6.10E-05	1	0	0
BTLA	0.569078	0.693397	-0.12432	-0.33804	5.37E-06	6.13E-05	5	0	0
LMO3	0.367313	0.280936	0.086377	0.516159	5.38E-06	6.14E-05	5	0	0
GABRB1	0.17262	0.101335	0.071285	0.755277	5.41E-06	6.17E-05	11	0	0
STK38L	0.418878	0.484824	-0.06595	-0.22799	5.42E-06	6.17E-05	11	0	0
OR2A5	0.653845	0.738718	-0.08487	-0.18419	5.44E-06	6.19E-05	4	0	0
HTR3B	0.640833	0.633684	0.007148	0.057876	5.44E-06	6.19E-05	3	0	0
EPO	0.167708	0.080951	0.086757	1.31445	5.44E-06	6.19E-05	10	0	0
IL17D	0.714588	0.768334	-0.05375	-0.15353	5.45E-06	6.20E-05	5	0	0
CYP17A1	0.840694	0.886666	-0.04597	-0.07593	5.58E-06	6.34E-05	1	0	0
NUPR1	0.468352	0.378836	0.089516	0.310486	5.61E-06	6.37E-05	8	0	0
TUBB6	0.25152	0.111542	0.139978	1.199526	5.62E-06	6.37E-05	4	0	0
PBX4	0.19797	0.211208	-0.01324	-0.19379	5.62E-06	6.38E-05	16	0	0
PTH	0.687664	0.7826	-0.09494	-0.18465	5.64E-06	6.39E-05	5	0	0
HK1	0.720323	0.791699	-0.07138	-0.13406	5.67E-06	6.42E-05	3	0	0
CNBD2	0.44538	0.564561	-0.11918	-0.33539	5.69E-06	6.43E-05	1	0	0
HOXA4	0.439096	0.326621	0.112475	0.477471	5.69E-06	6.44E-05	19	0	0
OR52B6	0.800083	0.879744	-0.07966	-0.13803	5.70E-06	6.44E-05	6	0	0
SOX15	0.705144	0.797461	-0.09232	-0.17444	5.79E-06	6.54E-05	12	0	0
SCAMP4	0.172321	0.219875	-0.04755	-0.27523	5.80E-06	6.55E-05	8	0	0
GRIK3	0.228648	0.153982	0.074666	0.688612	5.82E-06	6.56E-05	10	0	0
RALYL	0.255259	0.146141	0.109118	0.969549	5.82E-06	6.56E-05	9	0	0
TNFSF18	0.781514	0.694113	0.087401	0.177632	5.84E-06	6.57E-05	2	0	0
CDK10	0.674342	0.765676	-0.09133	-0.18074	5.84E-06	6.57E-05	1	0	0
04-Sep	0.70762	0.794215	-0.0866	-0.16557	5.84E-06	6.57E-05	6	0	0
CETN1	0.861623	0.940564	-0.07894	-0.12522	5.83E-06	6.57E-05	3	0	0
01-Dec	0.748897	0.829163	-0.08027	-0.15241	5.86E-06	6.58E-05	4	0	0
KRTAP5-1	0.572061	0.692951	-0.12089	-0.27226	5.87E-06	6.60E-05	1	0	0
OR10Q1	0.771157	0.864468	-0.09331	-0.17009	5.92E-06	6.64E-05	6	0	0
DEGS1	0.682807	0.536695	0.146112	0.341718	5.95E-06	6.68E-05	1	0	0
PTH2R	0.767249	0.886935	-0.11969	-0.20835	5.99E-06	6.71E-05	4	0	0
UNC5CL	0.630296	0.681833	-0.05154	-0.10628	5.99E-06	6.71E-05	7	0	0
DLC1	0.49574	0.619265	-0.12353	-0.32761	5.99E-06	6.71E-05	4	0	0
ADAM30	0.727992	0.700842	0.02715	0.083184	6.01E-06	6.73E-05	11	0	0
PRSS57	0.398045	0.485105	-0.08706	-0.28254	6.03E-06	6.74E-05	7	0	0
PDPK1	0.367395	0.465195	-0.0978	-0.25001	6.05E-06	6.76E-05	2	0	0
GRK7	0.795237	0.864507	-0.06927	-0.12762	6.07E-06	6.78E-05	13	0	0
CD84	0.354313	0.471989	-0.11768	-0.40434	6.08E-06	6.79E-05	4	0	0
SLC26A9	0.422429	0.505666	-0.08324	-0.29708	6.08E-06	6.79E-05	4	0	0
ATIC	0.155383	0.199511	-0.04413	-0.33953	6.09E-06	6.79E-05	13	0	0
OR12D3	0.694734	0.777471	-0.08274	-0.17422	6.12E-06	6.82E-05	5	0	0
CRNN	0.835104	0.897009	-0.0619	-0.10435	6.14E-06	6.82E-05	3	0	0
MBNL1	0.632199	0.774256	-0.14206	-0.2883	6.13E-06	6.82E-05	1	0	0
PDILT	0.680822	0.753678	-0.07286	-0.17226	6.14E-06	6.82E-05	6	0	0

DDC	0.503931	0.601373	-0.09744	-0.22863	6.16E-06	6.84E-05	8	0	0
CYP2C8	0.814657	0.886044	-0.07139	-0.11999	6.18E-06	6.86E-05	2	0	0
ZNF471	0.302628	0.204177	0.098451	0.968109	6.20E-06	6.88E-05	10	0	0
INMT	0.520615	0.558042	-0.03743	-0.09265	6.21E-06	6.89E-05	5	0	0
RNF216	0.038234	0.052794	-0.01456	-0.20186	6.22E-06	6.90E-05	7	0	0
PCDHGA8	0.435101	0.336656	0.098445	0.419597	6.23E-06	6.91E-05	6	0	0
CSNK1A1L	0.79904	0.865484	-0.06644	-0.11877	6.24E-06	6.91E-05	9	0	0
FAM205A	0.771644	0.835029	-0.06338	-0.11358	6.26E-06	6.92E-05	2	0	0
TRPM5	0.696717	0.768058	-0.07134	-0.13765	6.28E-06	6.94E-05	18	0	0
ZCRB1	0.060062	0.078224	-0.01816	-0.24678	6.37E-06	7.04E-05	6	0	0
CLDN12	0.816861	0.888327	-0.07147	-0.1196	6.38E-06	7.05E-05	1	0	0
TMEM211	0.518144	0.582084	-0.06394	-0.17342	6.45E-06	7.12E-05	3	0	0
IQCF3	0.79009	0.870486	-0.0804	-0.14647	6.46E-06	7.13E-05	5	0	0
PAK1	0.064507	0.075738	-0.01123	-0.22991	6.46E-06	7.13E-05	5	0	0
VILL	0.592179	0.657735	-0.06556	-0.17235	6.48E-06	7.13E-05	5	0	0
HLX	0.398306	0.308027	0.090279	0.442931	6.52E-06	7.18E-05	9	0	0
TMEM233	0.274059	0.330904	-0.05684	0.049968	6.58E-06	7.24E-05	9	0	0
TIAM2	0.868145	0.847266	0.020879	0.034967	6.59E-06	7.24E-05	5	0	0
PANK4	0.146467	0.179284	-0.03282	-0.23417	6.59E-06	7.24E-05	17	0	0
MS4A5	0.748549	0.837413	-0.08886	-0.16917	6.60E-06	7.24E-05	6	0	0
ATG9B	0.649705	0.720814	-0.07111	-0.16757	6.62E-06	7.26E-05	4	0	0
SMTNL1	0.768101	0.853899	-0.0858	-0.15585	6.64E-06	7.29E-05	7	0	0
HIPK3	0.397213	0.494782	-0.09757	-0.21558	6.67E-06	7.32E-05	12	0	0
TAS1R2	0.695983	0.758181	-0.0622	-0.12116	6.68E-06	7.32E-05	3	0	0
OR56B4	0.52187	0.643679	-0.12181	-0.29751	6.70E-06	7.34E-05	1	0	0
BHLHE23	0.200499	0.121639	0.07886	0.933486	6.73E-06	7.37E-05	4	0	0
LIFR	0.511488	0.618541	-0.10705	-0.27086	6.74E-06	7.38E-05	2	0	0
ZIM3	0.676569	0.771429	-0.09486	-0.21499	6.76E-06	7.39E-05	3	0	0
FABP2	0.755714	0.822709	-0.067	-0.12273	6.78E-06	7.41E-05	5	0	0
PLXNA1	0.679257	0.720045	-0.04079	-0.06689	6.79E-06	7.41E-05	8	0	0
LRRC56	0.115477	0.148161	-0.03268	-0.20165	6.79E-06	7.41E-05	7	0	0
CTRB2	0.699872	0.762874	-0.063	-0.13796	6.83E-06	7.45E-05	9	0	0
GALNT3	0.11403	0.137387	-0.02336	-0.36661	6.85E-06	7.46E-05	13	0	0
CADPS	0.215681	0.120833	0.094848	1.005635	6.85E-06	7.46E-05	11	0	0
EXOC6	0.48429	0.416116	0.068173	0.203644	6.88E-06	7.48E-05	6	0	0
OR52I2	0.686739	0.770521	-0.08378	-0.17548	6.89E-06	7.49E-05	4	0	0
RTN4R	0.590698	0.665602	-0.0749	-0.16953	6.90E-06	7.50E-05	1	0	0
MCHR2	0.374065	0.279569	0.094496	0.780206	6.91E-06	7.51E-05	14	0	0
HS3ST2	0.234183	0.136306	0.097877	0.858588	6.92E-06	7.51E-05	9	0	0
SCARF2	0.188909	0.136505	0.052404	0.571528	6.94E-06	7.53E-05	13	0	0
CIB4	0.590956	0.688132	-0.09718	-0.22158	6.95E-06	7.53E-05	6	0	0
CRYBB2	0.750056	0.815225	-0.06517	-0.12664	6.95E-06	7.53E-05	13	0	0
PEX11A	0.129161	0.151216	-0.02205	-0.21378	6.97E-06	7.54E-05	16	0	0
MYF6	0.668108	0.745886	-0.07778	-0.17742	6.98E-06	7.55E-05	7	0	0
DOK6	0.164332	0.07757	0.086763	1.096239	6.98E-06	7.55E-05	5	0	0
CLDN8	0.613674	0.717907	-0.10423	-0.22636	7.00E-06	7.57E-05	5	0	0
PCDHGB4	0.538526	0.458683	0.079843	0.408996	7.01E-06	7.57E-05	3	0	0
FAM136A	0.51136	0.594848	-0.08349	-0.15691	7.03E-06	7.59E-05	4	0	0
CCL21	0.641544	0.633378	0.008167	0.061838	7.03E-06	7.59E-05	3	0	0
GPX6	0.763606	0.840914	-0.07731	-0.14591	7.09E-06	7.63E-05	4	0	0
TREML1	0.529975	0.582615	-0.05264	-0.11143	7.09E-06	7.63E-05	8	0	0
OR1L6	0.613079	0.707595	-0.09452	-0.2094	7.09E-06	7.63E-05	3	0	0
ECI1	0.626543	0.687545	-0.061	-0.16062	7.09E-06	7.63E-05	5	0	0
KPRP	0.632683	0.725408	-0.09272	-0.19688	7.13E-06	7.64E-05	5	0	0
UROC1	0.671685	0.759073	-0.08739	-0.19387	7.13E-06	7.64E-05	5	0	0

HAND2	0.220549	0.133262	0.087287	0.773198	7.12E-06	7.64E-05	19	0	0
GRK6	0.306831	0.262459	0.044373	0.139448	7.12E-06	7.64E-05	12	0	0
AMBP	0.565526	0.591488	-0.02596	-0.09199	7.13E-06	7.64E-05	5	0	0
OR4K13	0.624814	0.713726	-0.08891	-0.19122	7.12E-06	7.64E-05	3	0	0
BHLHA9	0.501168	0.452137	0.049031	0.296531	7.13E-06	7.64E-05	9	0	0
SIAE	0.864759	0.916336	-0.05158	-0.08263	7.16E-06	7.67E-05	2	0	0
SFMBT1	0.443044	0.482688	-0.03964	-0.19975	7.17E-06	7.68E-05	4	0	0
11-Mar	0.440779	0.459961	-0.01918	0.132253	7.19E-06	7.69E-05	6	0	0
AQP8	0.664084	0.741046	-0.07696	-0.15911	7.23E-06	7.73E-05	3	0	0
PDHX	0.265184	0.34273	-0.07755	-0.37026	7.25E-06	7.74E-05	2	0	0
PCK1	0.528474	0.617536	-0.08906	-0.21248	7.26E-06	7.75E-05	10	0	0
OCIAD1	0.76401	0.681261	0.082749	0.163121	7.27E-06	7.75E-05	1	0	0
CUEDC1	0.126489	0.193831	-0.06734	-0.23986	7.27E-06	7.75E-05	5	0	0
TTN	0.665577	0.790458	-0.12488	-0.25097	7.28E-06	7.76E-05	2	0	0
BCL11A	0.057314	0.072382	-0.01507	-0.23148	7.38E-06	7.86E-05	15	0	0
MYOM2	0.270571	0.333523	-0.06295	-0.25618	7.40E-06	7.88E-05	14	0	0
LGALS8	0.539308	0.671447	-0.13214	-0.29609	7.42E-06	7.89E-05	2	0	0
CRCP	0.41785	0.484057	-0.06621	-0.09357	7.42E-06	7.89E-05	7	0	0
FAM167B	0.656181	0.587125	0.069056	0.159845	7.44E-06	7.90E-05	5	0	0
ZFP28	0.209848	0.113635	0.096213	1.239761	7.44E-06	7.91E-05	8	0	0
SOHLH1	0.698392	0.807084	-0.10869	-0.19601	7.45E-06	7.91E-05	5	0	0
ODF3	0.595623	0.676051	-0.08043	-0.18778	7.50E-06	7.95E-05	6	0	0
SOST	0.630176	0.737701	-0.10753	-0.20279	7.50E-06	7.95E-05	5	0	0
HOOK2	0.612761	0.483112	0.129649	0.347564	7.50E-06	7.95E-05	2	0	0
PCDHGA3	0.392928	0.314277	0.078651	0.469044	7.54E-06	7.98E-05	10	0	0
PLEKHN1	0.502056	0.556494	-0.05444	-0.24004	7.55E-06	7.99E-05	9	0	0
RPS3A	0.138498	0.177706	-0.03921	-0.15561	7.56E-06	7.99E-05	8	0	0
VSTM2A	0.185857	0.111034	0.074823	0.699972	7.57E-06	8.00E-05	11	0	0
SESN3	0.367149	0.37148	-0.00433	0.515317	7.58E-06	8.00E-05	12	0	0
GUCA1C	0.763161	0.850412	-0.08725	-0.15902	7.66E-06	8.08E-05	4	0	0
ARHGEF15	0.568515	0.48296	0.085555	0.258314	7.67E-06	8.09E-05	6	0	0
DPRX	0.771266	0.856921	-0.08566	-0.15697	7.67E-06	8.09E-05	8	0	0
SWSAP1	0.106239	0.149803	-0.04356	-0.22653	7.68E-06	8.10E-05	15	0	0
SUN1	0.081327	0.135845	-0.05452	-0.35643	7.70E-06	8.10E-05	7	0	0
C18orf32	0.154548	0.195553	-0.041	-0.20811	7.70E-06	8.10E-05	8	0	0
TMEM144	0.784249	0.824031	-0.03978	-0.07341	7.75E-06	8.15E-05	2	0	0
MALRD1	0.636889	0.743742	-0.10685	-0.22172	7.79E-06	8.19E-05	3	0	0
EGLN1	0.756902	0.686661	0.070241	0.201721	7.80E-06	8.19E-05	4	0	0
UGT2B4	0.856206	0.892415	-0.03621	-0.05908	7.83E-06	8.22E-05	1	0	0
SPINK14	0.868249	0.890074	-0.02182	-0.03541	7.86E-06	8.24E-05	1	0	0
SMIM2	0.749526	0.794628	-0.0451	-0.07516	7.86E-06	8.24E-05	6	0	0
ZMYM1	0.670322	0.760208	-0.08989	-0.17547	7.93E-06	8.31E-05	7	0	0
KIR3DL1	0.635865	0.741027	-0.10516	-0.22386	7.95E-06	8.31E-05	2	0	0
KIR3DL3	0.635865	0.741027	-0.10516	-0.22386	7.95E-06	8.31E-05	2	0	0
KIR2DL4	0.635865	0.741027	-0.10516	-0.22386	7.95E-06	8.31E-05	2	0	0
GJA8	0.715275	0.78566	-0.07039	-0.13496	7.96E-06	8.32E-05	6	0	0
ZNF71	0.407265	0.425783	-0.01852	0.583876	7.99E-06	8.35E-05	6	0	0
SATB2	0.313267	0.235583	0.077684	0.749273	8.01E-06	8.37E-05	9	0	0
ODF1	0.835169	0.902518	-0.06735	-0.11496	8.06E-06	8.40E-05	8	0	0
GPR26	0.332451	0.244373	0.088077	0.512047	8.06E-06	8.40E-05	9	0	0
OR52A1	0.528043	0.631832	-0.10379	-0.25447	8.05E-06	8.40E-05	1	0	0
FOXF2	0.189197	0.12026	0.068937	0.760306	8.06E-06	8.40E-05	6	0	0
SCGB3A1	0.223007	0.147091	0.075916	1.026027	8.08E-06	8.41E-05	10	0	0
FAM177A1	0.687474	0.739702	-0.05223	-0.12588	8.10E-06	8.43E-05	3	0	0
GPR32	0.716057	0.850634	-0.13458	-0.24532	8.10E-06	8.43E-05	1	0	0

DDR1	0.596451	0.737475	-0.14102	-0.30241	8.11E-06	8.43E-05	3	0	0
S100A12	0.615185	0.716582	-0.1014	-0.22472	8.18E-06	8.50E-05	5	0	0
SPERT	0.706785	0.790341	-0.08356	-0.16741	8.19E-06	8.51E-05	4	0	0
WFIKKN1	0.756561	0.723122	0.033439	0.070107	8.21E-06	8.52E-05	6	0	0
ZMYND8	0.176846	0.219927	-0.04308	-0.29006	8.24E-06	8.55E-05	15	0	0
NLRP1	0.746102	0.859014	-0.11291	-0.2008	8.26E-06	8.56E-05	1	0	0
AFAP1L2	0.258594	0.280822	-0.02223	0.095971	8.27E-06	8.57E-05	12	0	0
METTL9	0.822751	0.854136	-0.03139	-0.05376	8.29E-06	8.59E-05	7	0	0
FSCN3	0.72196	0.785364	-0.0634	-0.12932	8.30E-06	8.59E-05	2	0	0
MATN4	0.597956	0.562714	0.035242	0.11842	8.31E-06	8.59E-05	13	0	0
TNFRSF13(	0.493621	0.549683	-0.05606	-0.14735	8.31E-06	8.59E-05	5	0	0
OR51E2	0.625915	0.719491	-0.09358	-0.20704	8.34E-06	8.62E-05	5	0	0
TMEM26	0.329933	0.255133	0.0748	0.717834	8.35E-06	8.62E-05	12	0	0
C3orf22	0.600459	0.699664	-0.0992	-0.20646	8.39E-06	8.66E-05	8	0	0
CD33	0.371347	0.446833	-0.07549	-0.24696	8.40E-06	8.66E-05	3	0	0
THEM5	0.642215	0.73309	-0.09088	-0.20853	8.43E-06	8.69E-05	7	0	0
YIPF1	0.768327	0.83142	-0.06309	-0.11632	8.47E-06	8.72E-05	5	0	0
FIGLA	0.411764	0.324583	0.087181	0.575999	8.47E-06	8.72E-05	10	0	0
IFNA16	0.575681	0.726668	-0.15099	-0.3309	8.48E-06	8.72E-05	1	0	0
GPR84	0.587109	0.701429	-0.11432	-0.27035	8.48E-06	8.72E-05	6	0	0
GCSAML	0.531107	0.651256	-0.12015	-0.29494	8.49E-06	8.72E-05	3	0	0
TRPM8	0.707286	0.812085	-0.1048	-0.19715	8.49E-06	8.72E-05	2	0	0
TMEM40	0.754247	0.803497	-0.04925	-0.09181	8.50E-06	8.73E-05	2	0	0
PAPPA2	0.597202	0.70715	-0.10995	-0.23731	8.52E-06	8.75E-05	4	0	0
SLC25A45	0.672513	0.645873	0.02664	0.086256	8.54E-06	8.75E-05	4	0	0
THEMIS	0.700412	0.792569	-0.09216	-0.18381	8.55E-06	8.76E-05	6	0	0
FDCSP	0.715559	0.773782	-0.05822	-0.11666	8.56E-06	8.76E-05	4	0	0
SCN1A	0.566438	0.709513	-0.14308	-0.31986	8.60E-06	8.81E-05	1	0	0
TMEM156	0.49311	0.575035	-0.08192	-0.23017	8.62E-06	8.82E-05	5	0	0
GJB7	0.758649	0.87857	-0.11992	-0.2314	8.63E-06	8.83E-05	4	0	0
N4BP3	0.313159	0.366362	-0.0532	-0.0296	8.64E-06	8.83E-05	6	0	0
DPYS	0.524813	0.459853	0.06496	0.427861	8.64E-06	8.83E-05	11	0	0
TSPYL5	0.472903	0.478998	-0.00609	0.461616	8.66E-06	8.84E-05	7	0	0
KDM4B	0.34657	0.411264	-0.06469	-0.22728	8.67E-06	8.84E-05	4	0	0
SVOP	0.482538	0.574661	-0.09212	-0.24304	8.69E-06	8.86E-05	11	0	0
SSTR4	0.488217	0.46371	0.024507	0.20246	8.69E-06	8.86E-05	10	0	0
OR5M11	0.707992	0.795968	-0.08798	-0.17424	8.70E-06	8.86E-05	5	0	0
THBS2	0.359903	0.299527	0.060375	0.7066	8.71E-06	8.87E-05	13	0	0
PPP3R2	0.614567	0.739424	-0.12486	-0.26615	8.75E-06	8.90E-05	4	0	0
FCRL3	0.561734	0.696851	-0.13512	-0.31777	8.77E-06	8.91E-05	8	0	0
TRIML1	0.640476	0.658761	-0.01828	-0.00095	8.77E-06	8.91E-05	7	0	0
EPB41L4B	0.297102	0.25226	0.044842	0.092568	8.78E-06	8.92E-05	3	0	0
TMTC1	0.604372	0.668699	-0.06433	-0.19875	8.82E-06	8.95E-05	8	0	0
GAD2	0.16186	0.092016	0.069844	0.804702	8.86E-06	8.97E-05	14	0	0
HRASLS5	0.255238	0.201329	0.053908	0.719771	8.85E-06	8.97E-05	13	0	0
CNTNAP4	0.44704	0.536564	-0.08952	-0.1862	8.85E-06	8.97E-05	5	0	0
BIRC8	0.690273	0.779222	-0.08895	-0.19347	8.86E-06	8.97E-05	7	0	0
GNAI1	0.47861	0.586222	-0.10761	-0.25098	8.90E-06	9.01E-05	4	0	0
AGO1	0.694186	0.836061	-0.14187	-0.2682	8.96E-06	9.06E-05	6	0	0
TNFRSF17	0.77762	0.850749	-0.07313	-0.14317	8.97E-06	9.07E-05	4	0	0
DEFB129	0.741596	0.850289	-0.10869	-0.19475	8.97E-06	9.07E-05	2	0	0
KRT80	0.478838	0.55328	-0.07444	-0.18831	8.99E-06	9.08E-05	8	0	0
ASTN1	0.149869	0.088629	0.061241	0.731541	9.05E-06	9.13E-05	14	0	0
HEMGN	0.716984	0.821398	-0.10441	-0.19316	9.05E-06	9.13E-05	3	0	0
SLC18A3	0.179674	0.106148	0.073526	0.721911	9.04E-06	9.13E-05	10	0	0

EFCAB1	0.282224	0.332064	-0.04984	-0.17063	9.06E-06	9.13E-05	11	0	0
ANKRD13E	0.17174	0.217576	-0.04584	-0.21162	9.06E-06	9.13E-05	6	0	0
NRSN1	0.580976	0.632716	-0.05174	0.037245	9.08E-06	9.14E-05	8	0	0
ZNF124	0.494983	0.552279	-0.0573	-0.27825	9.10E-06	9.15E-05	3	0	0
CLEC4A	0.61311	0.702897	-0.08979	-0.20082	9.13E-06	9.17E-05	7	0	0
WDR93	0.128969	0.14942	-0.02045	-0.21047	9.12E-06	9.17E-05	16	0	0
PRDM2	0.228008	0.288254	-0.06025	-0.23774	9.17E-06	9.21E-05	10	0	0
FAM24A	0.657426	0.744269	-0.08684	-0.18052	9.19E-06	9.22E-05	8	0	0
IGSF9	0.238895	0.305691	-0.0668	-0.266	9.24E-06	9.27E-05	7	0	0
TNFAIP8L2	0.376109	0.420461	-0.04435	-0.17	9.25E-06	9.27E-05	6	0	0
HIST1H2BE	0.195339	0.074289	0.121049	1.370639	9.34E-06	9.36E-05	5	0	0
IQCK	0.743987	0.839584	-0.0956	-0.17977	9.35E-06	9.36E-05	5	0	0
C2orf54	0.412259	0.481087	-0.06883	-0.21774	9.36E-06	9.37E-05	4	0	0
OR2H1	0.526857	0.672844	-0.14599	-0.34706	9.39E-06	9.39E-05	2	0	0
SCN4A	0.708967	0.786048	-0.07708	-0.16598	9.38E-06	9.39E-05	11	0	0
IL1RL2	0.650289	0.728301	-0.07801	-0.1819	9.47E-06	9.46E-05	10	0	0
ZNF534	0.694577	0.786372	-0.09179	-0.18233	9.48E-06	9.46E-05	2	0	0
UBALD1	0.341518	0.407585	-0.06607	-0.14165	9.51E-06	9.49E-05	8	0	0
SPATA31A	0.822406	0.880958	-0.05855	-0.09807	9.56E-06	9.53E-05	1	0	0
DPPA2	0.699892	0.828695	-0.1288	-0.24115	9.60E-06	9.57E-05	8	0	0
LY75	0.19405	0.232624	-0.03857	-0.28828	9.63E-06	9.59E-05	11	0	0
OR2C3	0.612537	0.729538	-0.117	-0.25685	9.65E-06	9.61E-05	4	0	0
ASB18	0.559584	0.637648	-0.07806	-0.20623	9.74E-06	9.69E-05	12	0	0
IGF1	0.544076	0.428977	0.115099	0.549287	9.78E-06	9.72E-05	5	0	0
CXCL13	0.693692	0.699736	-0.00604	0.018799	9.79E-06	9.73E-05	5	0	0
BRCA2	0.20389	0.262114	-0.05822	-0.28331	9.84E-06	9.77E-05	8	0	0
MAML1	0.117595	0.145819	-0.02822	-0.17418	9.85E-06	9.77E-05	6	0	0
CHRDL2	0.240593	0.161133	0.079461	0.857641	9.85E-06	9.77E-05	4	0	0
CACNA2D2	0.455955	0.508066	-0.05211	-0.06982	9.87E-06	9.79E-05	7	0	0
ENDOU	0.575161	0.642278	-0.06712	-0.20861	9.93E-06	9.84E-05	4	0	0
ASRGL1	0.359381	0.416034	-0.05665	-0.02412	9.97E-06	9.87E-05	9	0	0
EGFLAM	0.172212	0.11546	0.056752	0.710676	9.98E-06	9.88E-05	13	0	0
TMEM74B	0.28201	0.314579	-0.03257	0.092673	1.01E-05	9.96E-05	3	0	0
EBLN2	0.796449	0.851651	-0.0552	-0.10255	1.01E-05	1.00E-04	3	0	0
LYST	0.268117	0.162197	0.10592	0.731445	1.01E-05	0.0001	3	0	0
AMY2A	0.703806	0.775128	-0.07132	-0.14911	1.02E-05	0.0001	3	0	0
CD248	0.336881	0.281601	0.05528	0.261863	1.02E-05	0.0001	10	0	0
KLRK1	0.565353	0.677608	-0.11225	-0.31457	1.02E-05	0.0001	3	0	0
ZNF829	0.190527	0.112351	0.078175	1.125516	1.02E-05	0.0001	10	0	0
MS4A6A	0.595183	0.687659	-0.09248	-0.21198	1.02E-05	0.000101	7	0	0
ACSM4	0.699754	0.79418	-0.09443	-0.17938	1.02E-05	0.000101	4	0	0
IL1R2	0.602417	0.690406	-0.08799	-0.21047	1.02E-05	0.000101	8	0	0
AQP10	0.420712	0.498353	-0.07764	-0.27201	1.03E-05	0.000101	3	0	0
LCN2	0.539979	0.622539	-0.08256	-0.22426	1.03E-05	0.000101	4	0	0
GPR25	0.676933	0.620276	0.056658	0.161152	1.03E-05	0.000101	7	0	0
HTN1	0.711417	0.794311	-0.08289	-0.15692	1.03E-05	0.000101	1	0	0
LYN	0.136115	0.181174	-0.04506	-0.26715	1.03E-05	0.000101	10	0	0
ITGA2B	0.551966	0.642881	-0.09091	-0.22927	1.03E-05	0.000101	6	0	0
WDR88	0.837107	0.820741	0.016367	0.037383	1.03E-05	0.000101	10	0	0
NCAM2	0.22828	0.146123	0.082157	0.608799	1.03E-05	0.000101	11	0	0
FREM3	0.208993	0.221835	-0.01284	0.171776	1.04E-05	0.000101	11	0	0
FHDC1	0.10978	0.148742	-0.03896	-0.09499	1.04E-05	0.000101	6	0	0
AZGP1	0.612788	0.735524	-0.12274	-0.25769	1.04E-05	0.000101	5	0	0
CLEC2A	0.835867	0.893255	-0.05739	-0.09578	1.04E-05	0.000102	5	0	0
DEFB110	0.614288	0.709668	-0.09538	-0.20512	1.05E-05	0.000102	1	0	0

COL21A1	0.851793	0.885917	-0.03412	-0.05719	1.05E-05	0.000103	2	0	0
TMEM154	0.231028	0.267106	-0.03608	-0.03292	1.06E-05	0.000104	8	0	0
KIFAP3	0.696119	0.802361	-0.10624	-0.21439	1.07E-05	0.000104	4	0	0
DAG1	0.77765	0.758825	0.018825	0.07099	1.07E-05	0.000105	6	0	0
OR8D4	0.578734	0.695418	-0.11668	-0.26735	1.07E-05	0.000105	5	0	0
LRRIQ4	0.702276	0.831006	-0.12873	-0.24456	1.07E-05	0.000105	3	0	0
F13B	0.608277	0.700925	-0.09265	-0.19583	1.08E-05	0.000105	2	0	0
FAM71F1	0.808618	0.873017	-0.0644	-0.10998	1.08E-05	0.000105	4	0	0
RFPL1	0.675441	0.763212	-0.08777	-0.17383	1.09E-05	0.000106	1	0	0
PLA2G2A	0.624521	0.666742	-0.04222	-0.08008	1.09E-05	0.000106	6	0	0
OR6C6	0.426108	0.538663	-0.11256	-0.33124	1.09E-05	0.000106	1	0	0
ATP4A	0.586525	0.678637	-0.09211	-0.23423	1.10E-05	0.000107	8	0	0
SKOR1	0.454554	0.452435	0.002119	0.13676	1.10E-05	0.000107	8	0	0
OR2A14	0.687223	0.776403	-0.08918	-0.18281	1.10E-05	0.000107	2	0	0
H3F3B	0.464129	0.608075	-0.14395	-0.3825	1.11E-05	0.000108	1	0	0
BTC	0.423211	0.481905	-0.05869	-0.18903	1.11E-05	0.000108	5	0	0
GPR45	0.741375	0.823895	-0.08252	-0.1523	1.11E-05	0.000108	13	0	0
CACNG3	0.506613	0.601283	-0.09467	-0.15808	1.12E-05	0.000108	4	0	0
PGAP1	0.308017	0.367407	-0.05939	-0.34497	1.12E-05	0.000108	6	0	0
ASB15	0.775681	0.845877	-0.0702	-0.13313	1.12E-05	0.000108	4	0	0
TEKT3	0.25676	0.182993	0.073767	1.157691	1.12E-05	0.000108	10	0	0
SALL2	0.477847	0.437233	0.040614	0.507791	1.12E-05	0.000108	5	0	0
KRTAP9-3	0.776045	0.84511	-0.06906	-0.12237	1.13E-05	0.000109	4	0	0
REM2	0.34464	0.266335	0.078305	0.372821	1.13E-05	0.000109	7	0	0
ZBED6CL	0.74532	0.689156	0.056164	0.122594	1.13E-05	0.000109	4	0	0
OLFM4	0.582227	0.651216	-0.06899	-0.18099	1.14E-05	0.000109	6	0	0
MAS1	0.79852	0.878166	-0.07965	-0.13714	1.14E-05	0.00011	4	0	0
KRTAP15-1	0.593539	0.697343	-0.1038	-0.2354	1.14E-05	0.00011	6	0	0
PLET1	0.794724	0.85088	-0.05616	-0.09982	1.14E-05	0.00011	5	0	0
RNF175	0.414142	0.415623	-0.00148	0.358462	1.14E-05	0.00011	8	0	0
PLXNA4	0.769421	0.849993	-0.08057	-0.14458	1.15E-05	0.00011	9	0	0
TRIM59	0.626584	0.772442	-0.14586	-0.29877	1.15E-05	0.00011	2	0	0
SNED1	0.207125	0.137066	0.070059	0.357551	1.15E-05	0.000111	8	0	0
AGMO	0.583694	0.653183	-0.06949	-0.12517	1.15E-05	0.000111	4	0	0
HIST1H2BE	0.18922	0.078691	0.110529	1.195695	1.16E-05	0.000111	9	0	0
STK33	0.14756	0.164771	-0.01721	-0.23161	1.16E-05	0.000111	17	0	0
PRG3	0.632025	0.771312	-0.13929	-0.28328	1.16E-05	0.000112	2	0	0
MTHFD2	0.14287	0.159294	-0.01642	-0.26321	1.17E-05	0.000112	8	0	0
ALPK3	0.687164	0.684605	0.002559	0.034126	1.17E-05	0.000112	7	0	0
OR2T3	0.540973	0.689852	-0.14888	-0.34507	1.18E-05	0.000113	1	0	0
OR2AG1	0.586721	0.683467	-0.09675	-0.2389	1.18E-05	0.000113	4	0	0
PRSS23	0.788415	0.862567	-0.07415	-0.12813	1.18E-05	0.000113	1	0	0
PIWIL4	0.310918	0.347058	-0.03614	-0.17293	1.18E-05	0.000113	10	0	0
FUT4	0.310918	0.347058	-0.03614	-0.17293	1.18E-05	0.000113	10	0	0
DIO3	0.2357	0.149941	0.085759	0.8347	1.18E-05	0.000113	24	0	0
ZNF548	0.600444	0.659863	-0.05942	-0.06703	1.18E-05	0.000113	4	0	0
ZNF831	0.770941	0.833699	-0.06276	-0.11307	1.18E-05	0.000113	10	0	0
ERG	0.364992	0.413241	-0.04825	-0.06168	1.19E-05	0.000113	5	0	0
ACER1	0.615421	0.707223	-0.0918	-0.20134	1.19E-05	0.000113	7	0	0
TM4SF1	0.329792	0.397	-0.06721	-0.26619	1.19E-05	0.000113	10	0	0
MAPK12	0.126804	0.181127	-0.05432	-0.05689	1.19E-05	0.000113	8	0	0
LYG1	0.746088	0.806679	-0.06059	-0.11483	1.19E-05	0.000113	2	0	0
TBC1D26	0.828268	0.877818	-0.04955	-0.08285	1.19E-05	0.000113	1	0	0
MUC15	0.574012	0.691732	-0.11772	-0.2809	1.20E-05	0.000114	8	0	0
GCM1	0.819155	0.87906	-0.05991	-0.1041	1.20E-05	0.000114	7	0	0

POU2F3	0.413491	0.485631	-0.07214	-0.26934	1.21E-05	0.000114	5	0	0
FGF10	0.182807	0.099122	0.083686	0.942112	1.21E-05	0.000115	4	0	0
CCL4	0.513184	0.622852	-0.10967	-0.26356	1.22E-05	0.000115	2	0	0
FAM83C	0.658652	0.703123	-0.04447	-0.08051	1.22E-05	0.000115	7	0	0
GRAMD1A	0.640397	0.576618	0.063779	0.149755	1.23E-05	0.000116	3	0	0
MTRNR2L7	0.696528	0.76011	-0.06358	-0.12711	1.23E-05	0.000116	3	0	0
SULT1A1	0.288719	0.355681	-0.06696	-0.02884	1.23E-05	0.000116	3	0	0
C2orf16	0.762123	0.845423	-0.0833	-0.14786	1.24E-05	0.000117	6	0	0
SLC26A7	0.682094	0.775249	-0.09315	-0.18897	1.25E-05	0.000118	5	0	0
CPSF4L	0.575398	0.679863	-0.10447	-0.22952	1.25E-05	0.000118	10	0	0
SH2D1B	0.557221	0.657826	-0.10061	-0.31671	1.25E-05	0.000118	5	0	0
CACNA1E	0.950098	0.981816	-0.03172	-0.04689	1.26E-05	0.000118	1	0	0
SELE	0.703198	0.803239	-0.10004	-0.22575	1.26E-05	0.000119	2	0	0
NTM	0.603258	0.680585	-0.07733	-0.1428	1.27E-05	0.00012	8	0	0
IZUMO2	0.42206	0.368591	0.053468	0.402719	1.27E-05	0.00012	8	0	0
CYP27A1	0.234139	0.147491	0.086648	0.867785	1.27E-05	0.00012	9	0	0
RETNLB	0.645257	0.746852	-0.1016	-0.22438	1.27E-05	0.00012	7	0	0
COX8C	0.774638	0.844626	-0.06999	-0.12417	1.27E-05	0.00012	7	0	0
TNFRSF9	0.444223	0.520715	-0.07649	-0.1351	1.28E-05	0.00012	5	0	0
RFTN2	0.676782	0.641075	0.035707	0.107355	1.28E-05	0.00012	8	0	0
EMG1	0.434797	0.54879	-0.11399	-0.35815	1.28E-05	0.00012	5	0	0
ELSPBP1	0.472649	0.589465	-0.11682	-0.30768	1.28E-05	0.00012	3	0	0
SLC16A3	0.398731	0.452574	-0.05384	-0.16936	1.28E-05	0.00012	11	0	0
CWH43	0.302418	0.224973	0.077445	0.623171	1.29E-05	0.00012	15	0	0
NEFM	0.402139	0.322628	0.079511	0.684701	1.29E-05	0.000121	6	0	0
MGMT	0.434576	0.472821	-0.03825	0.05091	1.30E-05	0.000121	19	0	0
NELL1	0.18593	0.09439	0.091539	1.06486	1.30E-05	0.000121	13	0	0
SLC5A2	0.753371	0.803645	-0.05027	-0.09562	1.30E-05	0.000122	8	0	0
HBB	0.737955	0.826793	-0.08884	-0.16192	1.30E-05	0.000122	1	0	0
KRTAP24-1	0.494517	0.596992	-0.10247	-0.27355	1.30E-05	0.000122	6	0	0
RXFP2	0.783478	0.848001	-0.06452	-0.11168	1.31E-05	0.000122	3	0	0
DPP9	0.372756	0.4664	-0.09364	-0.09799	1.31E-05	0.000122	2	0	0
ISLR	0.691202	0.629255	0.061947	0.137253	1.31E-05	0.000122	6	0	0
PADI2	0.150261	0.187702	-0.03744	-0.19472	1.31E-05	0.000122	13	0	0
VWCE	0.355324	0.278018	0.077306	0.881508	1.31E-05	0.000122	12	0	0
SYCP1	0.565725	0.53123	0.034495	0.271476	1.31E-05	0.000122	11	0	0
OR5L2	0.473684	0.558682	-0.085	-0.20247	1.31E-05	0.000122	3	0	0
OR5P2	0.612658	0.730913	-0.11826	-0.25087	1.32E-05	0.000123	1	0	0
SLC10A2	0.652868	0.747973	-0.09511	-0.19318	1.32E-05	0.000123	5	0	0
VWA7	0.367474	0.404897	-0.03742	-0.25059	1.32E-05	0.000123	11	0	0
MS4A6E	0.636858	0.725732	-0.08887	-0.19221	1.32E-05	0.000123	3	0	0
SLC16A1	0.239352	0.344698	-0.10535	-0.52012	1.33E-05	0.000124	2	0	0
ADAMTSL1	0.365043	0.36121	0.003832	0.354667	1.33E-05	0.000124	8	0	0
GPR142	0.577218	0.673669	-0.09645	-0.2236	1.34E-05	0.000124	7	0	0
ZNF568	0.223736	0.16303	0.060706	0.99858	1.34E-05	0.000124	11	0	0
PTPRN	0.391051	0.364305	0.026746	0.557074	1.34E-05	0.000124	11	0	0
LINGO4	0.491217	0.577578	-0.08636	-0.24733	1.34E-05	0.000124	5	0	0
TKTL2	0.772258	0.858604	-0.08635	-0.15503	1.35E-05	0.000124	13	0	0
KRTAP9-2	0.789296	0.868061	-0.07876	-0.13859	1.35E-05	0.000125	3	0	0
PCDHA13	0.546409	0.451195	0.095214	0.427266	1.37E-05	0.000126	9	0	0
SGCD	0.63198	0.750736	-0.11876	-0.25246	1.37E-05	0.000126	3	0	0
OR10C1	0.54298	0.672942	-0.12996	-0.30454	1.36E-05	0.000126	1	0	0
KRTAP25-1	0.628764	0.722922	-0.09416	-0.20537	1.37E-05	0.000126	5	0	0
M1AP	0.570729	0.506527	0.064201	0.223425	1.37E-05	0.000126	12	0	0
HAPLN4	0.443505	0.432772	0.010733	0.180481	1.37E-05	0.000126	9	0	0

SMR3B	0.580934	0.705096	-0.12416	-0.27514	1.38E-05	0.000127	1	0	0
DNAJB6	0.647626	0.723554	-0.07593	-0.15764	1.38E-05	0.000127	1	0	0
RERG	0.630173	0.753724	-0.12355	-0.25526	1.38E-05	0.000127	4	0	0
TCHHL1	0.631182	0.738144	-0.10696	-0.24386	1.38E-05	0.000127	5	0	0
HEPHL1	0.623301	0.693685	-0.07038	-0.18343	1.39E-05	0.000127	8	0	0
MAT1A	0.485251	0.542245	-0.05699	-0.23896	1.39E-05	0.000128	9	0	0
MYPN	0.657662	0.725185	-0.06752	-0.14531	1.39E-05	0.000128	6	0	0
IL18	0.437157	0.513521	-0.07636	-0.30542	1.39E-05	0.000128	8	0	0
GLCE	0.115354	0.158575	-0.04322	-0.24079	1.39E-05	0.000128	9	0	0
TTPAL	0.163734	0.199017	-0.03528	-0.22457	1.39E-05	0.000128	7	0	0
CLN8	0.316085	0.381376	-0.06529	-0.37307	1.40E-05	0.000128	3	0	0
CDR2L	0.367909	0.425832	-0.05792	-0.1671	1.40E-05	0.000128	8	0	0
LSMEM2	0.689711	0.717339	-0.02763	-0.0537	1.41E-05	0.000129	8	0	0
MPZL3	0.162218	0.191499	-0.02928	-0.29693	1.41E-05	0.000129	16	0	0
OR6C74	0.528003	0.648263	-0.12026	-0.28567	1.41E-05	0.000129	3	0	0
FOXG1	0.295147	0.183892	0.111255	0.683626	1.42E-05	0.00013	14	0	0
IPCEF1	0.605519	0.69117	-0.08565	-0.19156	1.42E-05	0.00013	5	0	0
CD8B	0.381935	0.313556	0.068379	0.380958	1.43E-05	0.000131	3	0	0
OR5C1	0.859279	0.914193	-0.05491	-0.09034	1.44E-05	0.000131	4	0	0
CD1B	0.466094	0.578753	-0.11266	-0.23793	1.44E-05	0.000132	3	0	0
ABTB1	0.316717	0.35051	-0.03379	-0.14638	1.44E-05	0.000132	7	0	0
RIN2	0.430757	0.363279	0.067478	0.27744	1.45E-05	0.000132	10	0	0
JUND	0.268076	0.228741	0.039336	0.193609	1.45E-05	0.000132	3	0	0
ZNF730	0.394126	0.428597	-0.03447	0.34848	1.45E-05	0.000132	2	0	0
SLC22A5	0.101	0.132423	-0.03142	-0.29195	1.46E-05	0.000133	7	0	0
DENND3	0.396416	0.478423	-0.08201	-0.21171	1.46E-05	0.000133	2	0	0
CERKL	0.167871	0.085323	0.082548	0.995363	1.46E-05	0.000133	14	0	0
NEUROD1	0.167871	0.085323	0.082548	0.995363	1.46E-05	0.000133	14	0	0
GLP2R	0.67394	0.744136	-0.0702	-0.1457	1.48E-05	0.000134	2	0	0
OR5M9	0.639339	0.722627	-0.08329	-0.18944	1.48E-05	0.000135	2	0	0
OR2T1	0.757372	0.858446	-0.10107	-0.18726	1.49E-05	0.000135	6	0	0
NEUROG1	0.225656	0.158358	0.067298	0.584189	1.49E-05	0.000135	22	0	0
ZNF229	0.35212	0.315083	0.037038	0.708578	1.49E-05	0.000135	16	0	0
TPSD1	0.452669	0.51593	-0.06326	-0.18489	1.49E-05	0.000135	1	0	0
CXCL6	0.18608	0.114373	0.071707	0.76696	1.50E-05	0.000136	5	0	0
FAM81B	0.718309	0.751368	-0.03306	-0.05706	1.50E-05	0.000136	4	0	0
LRRC3B	0.207101	0.130368	0.076733	0.647548	1.50E-05	0.000136	11	0	0
RAB26	0.760652	0.658997	0.101655	0.204079	1.51E-05	0.000136	1	0	0
AKR1E2	0.843819	0.901854	-0.05803	-0.09487	1.51E-05	0.000136	1	0	0
OR2A12	0.741156	0.816897	-0.07574	-0.13816	1.51E-05	0.000137	3	0	0
PCDHGB3	0.349916	0.233974	0.115942	0.713686	1.52E-05	0.000137	11	0	0
C8B	0.619341	0.707891	-0.08855	-0.20436	1.53E-05	0.000138	6	0	0
TRIM49B	0.704881	0.826328	-0.12145	-0.22637	1.53E-05	0.000138	1	0	0
PRAMEF12	0.731687	0.813494	-0.08181	-0.15049	1.53E-05	0.000138	4	0	0
OSTM1	0.240419	0.131407	0.109012	0.882616	1.53E-05	0.000138	12	0	0
ERI1	0.557965	0.619285	-0.06132	-0.18786	1.53E-05	0.000138	5	0	0
ARL14	0.613366	0.723577	-0.11021	-0.22278	1.54E-05	0.000138	4	0	0
COX7A1	0.74308	0.663197	0.079882	0.226974	1.54E-05	0.000138	8	0	0
OR6V1	0.638299	0.725899	-0.0876	-0.17544	1.54E-05	0.000139	6	0	0
OR2T8	0.641111	0.761456	-0.12034	-0.25427	1.55E-05	0.000139	4	0	0
PRR23C	0.691936	0.626238	0.065698	0.177828	1.55E-05	0.000139	10	0	0
TRAPPC2L	0.52531	0.583948	-0.05864	-0.16651	1.55E-05	0.000139	5	0	0
OR1K1	0.717208	0.783021	-0.06581	-0.12809	1.55E-05	0.000139	4	0	0
PTPRR	0.296206	0.300572	-0.00437	0.305283	1.55E-05	0.000139	13	0	0
VKORC1L1	0.038806	0.033936	0.00487	0.146829	1.56E-05	0.00014	2	0	0

ZNF556	0.671053	0.771137	-0.10008	-0.20912	1.56E-05	0.00014	9	0	0
CPA3	0.640656	0.768135	-0.12748	-0.26922	1.56E-05	0.00014	4	0	0
SEMA4B	0.595491	0.709596	-0.11411	-0.25429	1.56E-05	0.00014	3	0	0
ZSCAN10	0.422636	0.375079	0.047556	0.295746	1.57E-05	0.00014	9	0	0
PRG2	0.604146	0.724627	-0.12048	-0.27287	1.57E-05	0.00014	6	0	0
SLC23A3	0.562631	0.567262	-0.00463	-0.06555	1.57E-05	0.00014	4	0	0
KRTAP10-4	0.708112	0.782385	-0.07427	-0.14234	1.57E-05	0.00014	4	0	0
DCPS	0.255799	0.329988	-0.07419	-0.29037	1.57E-05	0.00014	12	0	0
GLYATL2	0.376227	0.327641	0.048586	0.678499	1.58E-05	0.000141	3	0	0
GLYATL1	0.376227	0.327641	0.048586	0.678499	1.58E-05	0.000141	3	0	0
CCDC166	0.652355	0.606525	0.04583	0.137219	1.58E-05	0.000141	12	0	0
DRGX	0.185526	0.106416	0.079109	0.756542	1.58E-05	0.000141	9	0	0
RAB3IL1	0.651052	0.737907	-0.08685	-0.18107	1.59E-05	0.000142	6	0	0
PCDHGA1	0.403806	0.300646	0.10316	0.565404	1.59E-05	0.000142	10	0	0
KCNA7	0.307722	0.387405	-0.07968	-0.15237	1.59E-05	0.000142	6	0	0
MAB21L2	0.903349	0.879375	0.023974	0.039698	1.60E-05	0.000142	11	0	0
HABP2	0.720554	0.824464	-0.10391	-0.19303	1.60E-05	0.000142	2	0	0
KRTAP5-3	0.547964	0.625209	-0.07724	-0.23462	1.60E-05	0.000142	3	0	0
MYH8	0.654408	0.752782	-0.09837	-0.21995	1.60E-05	0.000142	4	0	0
AATK	0.28055	0.334631	-0.05408	-0.22286	1.61E-05	0.000143	7	0	0
APOA4	0.648904	0.709848	-0.06094	-0.1265	1.62E-05	0.000143	5	0	0
KRT34	0.745827	0.813293	-0.06747	-0.12925	1.62E-05	0.000144	3	0	0
REEP3	0.166063	0.205663	-0.0396	-0.24071	1.63E-05	0.000144	10	0	0
KLRC1	0.59987	0.680405	-0.08054	-0.17733	1.63E-05	0.000145	5	0	0
UBR7	0.075579	0.108783	-0.0332	-0.17805	1.63E-05	0.000145	10	0	0
PCDHGA9	0.484196	0.385319	0.098877	0.463823	1.64E-05	0.000145	6	0	0
VN1R4	0.508024	0.622026	-0.114	-0.2852	1.64E-05	0.000145	3	0	0
FOXI1	0.531994	0.631536	-0.09954	-0.24323	1.65E-05	0.000146	12	0	0
HIST1H1A	0.576624	0.47713	0.099494	0.446663	1.65E-05	0.000146	10	0	0
TAAR5	0.537532	0.662831	-0.1253	-0.30881	1.66E-05	0.000147	3	0	0
SNTB1	0.773368	0.856331	-0.08296	-0.14726	1.66E-05	0.000147	5	0	0
MT4	0.705842	0.772434	-0.06659	-0.13632	1.67E-05	0.000147	7	0	0
ZNF486	0.267015	0.168585	0.09843	1.137969	1.67E-05	0.000147	6	0	0
CSTA	0.49609	0.60085	-0.10476	-0.29003	1.67E-05	0.000147	8	0	0
ITSN2	0.32571	0.370869	-0.04516	-0.1372	1.68E-05	0.000148	7	0	0
CDKN1A	0.82031	0.875047	-0.05474	-0.09065	1.69E-05	0.000148	3	0	0
SMOC2	0.236737	0.172952	0.063785	0.46473	1.70E-05	0.00015	8	0	0
TMEM168	0.204568	0.254723	-0.05015	-0.25312	1.71E-05	0.00015	9	0	0
RGS14	0.417899	0.485563	-0.06766	-0.26228	1.71E-05	0.00015	12	0	0
CLDN5	0.731602	0.691486	0.040116	0.090651	1.71E-05	0.00015	4	0	0
KLHL28	0.77189	0.824529	-0.05264	-0.09588	1.71E-05	0.000151	2	0	0
ANKFN1	0.516055	0.642602	-0.12655	-0.31099	1.73E-05	0.000152	1	0	0
OR52K1	0.734707	0.826772	-0.09207	-0.17063	1.73E-05	0.000152	4	0	0
CCR8	0.709591	0.778529	-0.06894	-0.14299	1.73E-05	0.000152	2	0	0
USP50	0.639569	0.724598	-0.08503	-0.18858	1.73E-05	0.000152	8	0	0
SLC25A24	0.12756	0.151145	-0.02358	-0.24431	1.74E-05	0.000152	11	0	0
MTHFD1L	0.347917	0.376885	-0.02897	-0.08356	1.74E-05	0.000152	3	0	0
PKD1L3	0.696979	0.792558	-0.09558	-0.1797	1.74E-05	0.000152	4	0	0
NUMBL	0.135911	0.164155	-0.02824	-0.38011	1.74E-05	0.000152	8	0	0
HMGN2	0.137406	0.179526	-0.04212	-0.18529	1.75E-05	0.000153	13	0	0
CXCR5	0.650081	0.713775	-0.06369	-0.11313	1.75E-05	0.000153	8	0	0
KIF19	0.270144	0.183258	0.086886	1.094154	1.75E-05	0.000153	7	0	0
AKR1B15	0.44688	0.512527	-0.06565	-0.22126	1.75E-05	0.000153	8	0	0
GAST	0.59797	0.674649	-0.07668	-0.22766	1.75E-05	0.000153	5	0	0
ROPN1B	0.753205	0.856262	-0.10306	-0.18273	1.75E-05	0.000153	1	0	0

| | | | | | | | | | |
[truncated: 361,585 more chars]
